# Multiscale network analysis reveals molecular mechanisms and key regulators of the tumor microenvironment in gastric cancer

**DOI:** 10.1002/ijc.32643

**Published:** 2019-10-11

**Authors:** Won‐Min Song, Xiandong Lin, Xuehong Liao, Dan Hu, Jieqiong Lin, Umut Sarpel, Yunbin Ye, Yael Feferman, Daniel M. Labow, Martin J. Walsh, Xiongwei Zheng, Bin Zhang

**Affiliations:** ^1^ Department of Genetics and Genomic Sciences, Icahn Institute of Genomics and Multiscale Biology Icahn School of Medicine at Mount Sinai New York, NY; ^2^ Mount Sinai Center for Transformative Disease Modeling Icahn School of Medicine at Mount Sinai New York, NY; ^3^ Laboratory of Radiation Oncology and Radiobiolog Fujian Cancer Hospital & Fujian Medical University Cancer Hospital Fuzhou Fujian China; ^4^ Fujian Provincial Key Laboratory of Translational Cancer Medicine Fuzhou Fujian China; ^5^ Department of Pathology Zhongshan Hospital of Xiamen University Xiamen Fujian China; ^6^ Department of Pathology Fujian Cancer Hospital & Fujian Medical University Cancer Hospital Fuzhou Fujian China; ^7^ Department of Surgery Icahn School of Medicine at Mount Sinai New York NY; ^8^ Laboratory of Immuno‐Oncology Fujian Cancer Hospital & Fujian Medical University Cancer Hospital Fuzhou Fujian China; ^9^ Department of Pharmacological Sciences Icahn School of Medicine at Mount Sinai New York NY; ^10^ The Mount Sinai Center for RNA Biology and Medicine Icahn School of Medicine at Mount Sinai New York NY

**Keywords:** gastric cancer, tumor microenvironment, gene coexpression network, network module, key driver, DNA methylation, somatic mutations

## Abstract

Gastric cancer (GC) is the third leading cause of cancer deaths and the fourth most prevalent malignancy worldwide. The high incidence and mortality rates of gastric cancer result from multiple factors such as ineffective screening, diagnosis, and limited treatment options. In our study, we sought to systematically identify predictive molecular networks and key regulators to elucidate complex interacting signaling pathways in GC. We performed an integrative network analysis of the transcriptomic data in The Cancer Genome Atlas (TCGA) gastric cancer cohort and then comprehensively characterized the predictive subnetworks and key regulators by the matched genetic and epigenetic data. We identified 221 gene subnetworks (modules) in GC. The most prognostic subnetworks captured multiple aspects of the tumor microenvironment in GC involving interactions among stromal, epithelial and immune cells. We revealed the genetic and epigenetic underpinnings of those subnetworks and their key transcriptional regulators. We computationally predicted and experimentally validated specific mechanisms of anticancer effects of *GKN2* in gastric cancer proliferation and invasion *in vitro*. The network models and the key regulators of the tumor microenvironment in GC identified here pave a way for developing novel therapeutic strategies for GC.

## Abstract 2

**What's new?**

Gene signatures have been identified for diagnosis and classification of gastric cancer (GC) as well as prediction of therapeutic response. However, key molecular mechanisms underlying prognosis remain to be revealed. Our study systematically identifies and characterizes predictive molecular networks and key regulators. The most prognostic subnetworks capture multiple aspects of the tumor microenvironment in GC involving interactions among stromal, epithelial, and immune cells. The authors computationally predicted and experimentally validated specific mechanisms of anti‐cancer effects of GKN2 in GC proliferation and invasion *in vitro*. These network models and key regulators pave the way for developing novel therapeutic strategies for GC.

**Abbreviations**
DMR: differentially methylated region
EBV: Epstein–Barr virus
EBV‐HEMG: EBV‐specific hyper‐methylation gene signature
EBV‐HOMG: EBV‐specific hypo‐methylation gene signature
eMSGs: expression associated methylation site genes
EMT: epithelial‐mesenchymal transition
FC: expression fold change
FE: fold enrichment
FET: Fisher's Exact Test
GC: gastric cancer
GEO: gene expression omnibus
GIST: gastrointestinal stromal tumor
GMB: gastric mucosal barrier
IMAC: intermicrovillar adhesion complex
IMB: intestinal mucosal barrier
SMGs: somatic mutation associated genes
TCGA: The Cancer Genome Atlas
TCGA‐GCC: TCGA gastric cancer discovery cohort
vTCGA‐GCC: TCGA gastric cancer validation cohort

## Introduction

Gastric cancer (GC) is the third leading cause of cancer death and the fourth most prevalent malignancy worldwide, accounting for 8% of cancer incidence and 10% of cancer deaths, and approximately 21,320 cases of GC (13,020 men and 8,300 women) were diagnosed and 10,540 patients died from GC in 2012 in the United States.^1^ The molecular mechanisms driving tumorigenesis of GC include a number of biological and cellular processes activated in tumor pathogenesis such as proliferation, angiogenesis, the bypass of senescence and cell death pathways, evasion of tumor suppressing mechanisms, immortality, invasion and so on.^2^ leading inconsistent treatment responses and marginal improvements.^3^

High‐throughput molecular profiling data makes it possible to dissect the heterogeneity of GC in a comprehensive and unbiased manner.^3^ A number of gene signatures have been identified for diagnosis and classification of GC as well as prediction of therapeutic response.^3^ However, the reproducibility of such gene signature‐based models are usually poor due to multiple factors including limited cross validation of predictive gene lists per tumor type and outcome.^4^ Recently, The Cancer Genome Atlas (TCGA), a comprehensive multi‐Omics cohort for studying multiple cancers was developed. TCGA includes genomic, transcriptomic and epigenomic molecular data of primary gastric adenocarcinomas. Analysis of the TCGA gastric cancer data stratifies five distinct molecular subtypes GC, specifically, Epstein–Barr virus positive (EBV), microsatellite instability (MSI), genomically stable (GS) and chromosomal instability (CIN) and these subtypes complement histopathological classifications by their distinctive patterns of DNA methylation, somatic genomic alterations and gene/protein expressions.^5^

Network biology has been successfully established to systematically model molecular interactions underlying complex human diseases.^6^ Here, we employed an integrative multiscale gene network analysis framework to the gastric cancer data in The Cancer Genome Atlas (TCGA‐GCC)^5^ to reveal key molecular mechanisms underlying GC prognosis in a data‐driven manner. We postulate that these key pathways emerge as coherent modules and associate to respective key genetic and/or epigenetic alterations. Combination of these key molecular events will further generate hypothesis on multiple molecular “niche” exploited in GC etiology. Note that all the *p*‐values reported in the manuscript were corrected for multiple testing unless stated otherwise.

## Materials and Methods

### Bioinformatics analysis

#### *Gene expression data processing*

Ilumina HiSeq RNA Sequencing data, processed by Reads per kilo base per million (RPKM) method from TCGA, have been downloaded and comprehensive data quality control (QC) has been performed: log2(RPKM +1) transform, quantile‐normalization, correction for batch effects by center, platform and tissue source site (TSS) ids from TCGA sample barcodes, and correction for confounding factors including ethnic group, age and gender, resulting to 218 annotated primary tumor tissue samples across 26,539 genes.

#### *Integrative network analyses of TCGA GC cohort*

In order to handle multifaceted, large‐scale –omics data for TCGA GC cohort (TCGA‐GCC; see Supporting Information Table S1 for cohort description), we developed an integrative network analysis framework to prioritize altered pathways in GC etiology (illustrated in Supporting Information Fig. S1). The backbone of the framework work lies in constructing multiscale coexpression network (Supporting Information Fig. S1C) by Multiscale Embedded Gene Co‐expression Network Analysis (MEGENA).^7^ Briefly, MEGENA first selects gene pairs with significant correlations (FDR < 0.05) and then embeds them onto a three‐dimensional topological sphere, leading to planar filtered network (PFN). Multiscale clustering is performed on PFN to unveil gene modules varying compactness, leading to a hierarchy of parent and child modules. The gene modules are annotated by enrichments of known pathway and functions from MSigDB^8^ (Supporting Information Fig. S1d).

Then, we generated a compendium of gene signatures reflecting genomic and epigenomic alterations (Supporting Information Fig. S1b; see Supporting Materials and Methods in Supporting Material 1). These gene signatures were intersected with gene modules to test enrichments. For instance, mutation signature is identified as differentially expressed genes in somatic mutants of a gene through limma^9^ with FDR < 0.05 and fold change >1.2. Gene modules enriched for the mutation signatures are then associated to this somatic gene mutation.

The gene modules were prioritized by associations to overall and recurrence‐free survival across the whole TCGA‐GCC as well as within clinical subtypes (Supporting Information Fig. S2). For each group of patients, the association between a module and an outcome was tested by two approaches. First, the module eigen‐gene^10^ (i.e., first principal component of module gene expressions) was modeled by univariate Cox proportional hazard model.^11^ Second, median expression of the module eigen‐gene was used to stratify patients into low‐ and high‐expression groups, and these were tested for significant difference in outcomes by logrank test (see Supporting Materials and Methods in Supporting Material 1). Overall, the importance of each gene module is determined by a score *MS*_*m*_ that summarizes the module's correlations to GC survival by

$${\mathrm{MS}}_{m}=\sum_{t=1}^{T}-\log_{10}\left(p_{t}\right),$$

where *m* = m^th^ module, *T* = number of tests, *p*_*t*_ = Cox or logrank *p*‐values.

To validate the network interactions of the nominated drivers, differentially expressed genes by respective gene perturbation from *in vivo*/*in vitro* experiments were projected to the network neighbor genes to test for enrichments (Supporting Information Fig. S1f; see Supporting Materials 1).

### Experimental procedures

#### *Patients and specimens*

One hundred and four patients who underwent gastric carcinoma resection between July 2012 and April 2015 were recruited in our study. The study protocol was approved by the Research Ethics Committee of the Fujian Provincial Cancer Hospital, and informed consent was obtained from all participants (namely, Fujian cohort; Approval number: SQ2015‐068‐01). There were 82 men and 22 women with a median age of 58.2 years (interquartile range, 22.0–82.0 years) (see Supporting Information Table S2). Histologic type was determined according to Lauren.^12^ None of the patients had received any chemotherapy prior to surgery. Fresh tumor tissues and adjacent nontumorous stomach tissues were obtained immediately after tumor resection. One part of the tissues was immediately snap‐frozen in liquid nitrogen and stored at −80°C, and the other part was fixed in 10% buffered formalin and embedded in paraffin.

#### *Cell lines*

Human gastric cancer cell line (AGS ‐ RRID:CVCL_0139) was purchased from the Cell Banks, the Chinese Academy of Sciences (Shanghai, China). AGS cell line was authenticated by DNA finger printing analysis. AGS was grown in RPMI1640 (Gibco; Thermo Fisher Scientific, Inc., Waltham, MA), supplemented with 10% fetal bovine serum (FBS) (Gibco; Thermo Fisher Scientific, Inc.). AGS was cultured in a 5% CO_2_ incubator at 37°C. When the cells reached the logarithmic growth phase, succeeding experiments were performed. All experiments were performed with mycoplasma‐free cells.

### Ethics approval and consent to participate

The study protocol for Fujian cohort was approved by the Research Ethics Committee of the Fujian Provincial Cancer Hospital, and informed consent was obtained from all participants (approval number: SQ2015‐068‐01).

### Data availability

The original gene expression data, analyzed as “TCGA‐GCC” and “vTCGA‐GCC,” that support findings of our study are available from TCGA data portal (https://portal.gdc.cancer.gov/). Respective patient barcodes for TCGA‐GCC and vTCGA‐GCC are provided in Supporting Material 2C within this manuscript. Another independent validation data, GSE84437, that support findings our study, are available from Gene Expression Omnibus (GEO) with the accession code “GSE84437.”

The primary software MEGENA (version 1.3.7) for network analysis is publicly available as R package in The Comprehensive R Archive Network (CRAN) (https://cran.r-project.org/web/packages/MEGENA/index.html). The development version of MEGENA is available from GitHub repository (https://github.com/songw01/MEGENA).

## Results

### Multiscale gene coexpression network of gastric cancer

**Figure 1:**
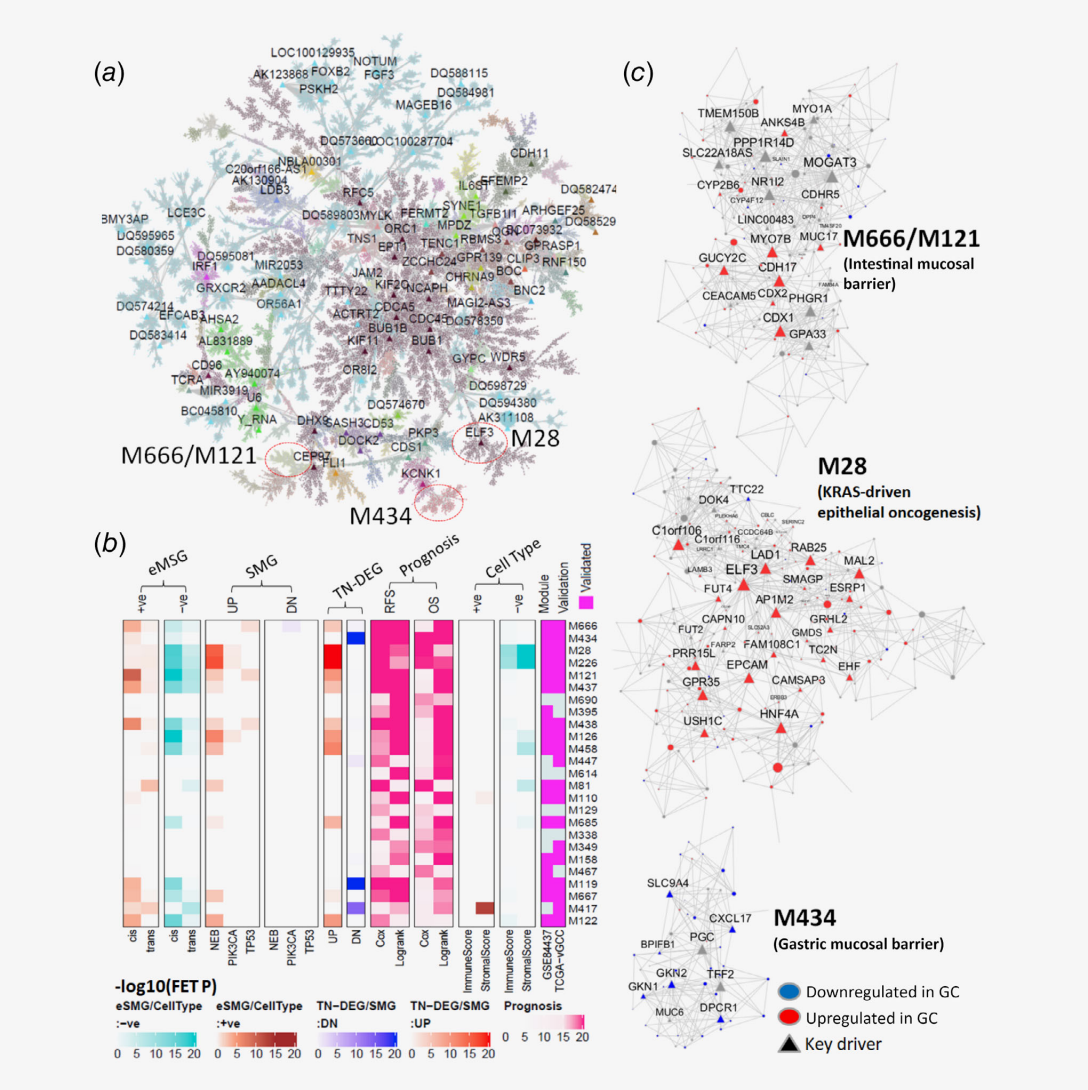
Multiscale coexpression network of TCGA‐GCC illustrates GC microenvironment. (*a*) TCGA‐GCC coexpression network: A global Planar Filtered Network (PFN) of the primary tumor gene expression data from TCGA gastric cancer. Nodes are colored by MEGENA modules identified at the default compactness scale α = 1. Global hub genes are labeled. (*b*) Heatmap for enrichments (i.e., ‐log10(FET p‐value)) of key molecular features of the top 25 gene modules most predictive of survival. The color bars are shown below. The columns are organized into several different categories including (right to left): (i) Module conservation (columns 1,2) in GSE84437 and vTCGA‐GCC. (ii) Cell type specificity (tracks 3–6): modules enriched for genes correlated to immune and/or stromal scores inferred by ESTIMATE. Negative (−ve) and positive (+ve) correlation signatures are in columns 3–4 and 5–6. (iii) Prognosis power of a module defined as ∑ − log10(Cox/logrank *p*‐value) based on overall survival in the whole cohort and in subtypes (column 7–8) (track 8), and based on recurrence‐free survival in the whole cohort and subtypes (columns 9–10); (iv) Enrichments of tumor vs normal DEGs, down‐/up‐regulated in tumor (column 11/12) (vi) Enrichments of key gene SMGs: module enrichment for the SMGs down‐/up‐regulated (DN: columns 13‐15/UP: columns 16–18) by the mutations in *NEB, PIK3CA* or *TP53*; (vii) eMSG: module enrichments of negatively (columns 19–20) or positively (columns 21–22) correlated cis‐eMSGs and trans‐eMSGs. (*c*) Subnetworks of top modules enriched in down‐/up‐regulated (blue/red) DEGs in tumor, compared to normal tissue. Network key drivers are labeled as triangles. Module names are respective locations in the global networks are labeled. [Color figure can be viewed at http://wileyonlinelibrary.com]

We developed an integrative network analysis framework to analyze multi‐Omics data in the TCGA gastric cancer cohort (denoted TCGA‐GCC; see Materials and Methods), using Multiscale Embedded Gene co‐Expression Network Analysis (MEGENA)^7^ (Fig. 1 *a*). Two hundred and twenty‐one modules pertaining a parent–child hierarchy were identified and were associated with known pathways (e.g., cell cycles, extra‐cellular matrix, focal adhesions, immune system process, DNA metabolic process, proteasome, biological oxidations) or reflected some unknown biological processes (see Supporting Material 2A). These modules were prioritized by their prognostic power of survival in the whole TCGA‐GCC population as well as various subtypes via Cox proportional hazard model (Fig. 1 *b*; Supporting Information Fig. S2; see Materials and Methods). Among 221 modules, top five modules were further interrogated (M666, M434, M28, M226 and M121). Taking account of module overlaps by the hierarchy, the biology of top five modules was effectively captured within M121/M666 (associated with intestinal brush border), M434 (associated with digestion) and M28 (associated with epithelial cell differentiation) and respective module hub genes were nominated as key drivers (Fig. 1 *c*).

Reproducibility of the TCGA‐GCC modules was assessed using two additional independent cohorts. As TCGA‐GCC has expanded with more samples during our study, we collected the RNA‐seq data from an additional 135 primary gastric tumor samples (termed *vTCGA‐GCC*) to verify the findings from the original TCGA‐GCC study. Also, we collected a microarray based gene expression study of 433 gastric cancer patients (Illumina HumanHT‐12 V3.0 expression beadchip) from GEO with an accession number GSE84437 (denoted as *GSE84437*). The module preservation analysis^13^ showed that 76.0% (168) and 94.6% (209) of the 221 TCGA‐GCC modules were significantly preserved in GSE84437 and vTCGA‐GCC, respectively (Fig. 1 *b* and Supporting Material 2B).

### The most prognostic modules capture multiple facets of GC tumor microenvironment

To characterize the GC network modules, we identified genetic, epigenetic and cell type gene signatures associated with GC. We systematically identified gene signatures as potential functional manifestation of nonsynonymous somatic mutations as differentially expressed genes between the mutants and wildtypes (Somatic Mutation associated Genes (SMGs); see the section *Identification of Somatic Mutation Associated Gene Signatures* in Supporting Material 1). Also, gene signatures correlated to *cis*−/*trans*‐methylation sites were identified (expression associated methylation site gene (eMSGs); see *Extraction of cis−/trans‐methylation significantly correlated gene signatures* in Supporting Material 1). These signatures were then used to characterize genetic and/or epigenetic alterations in the previously identified gene modules and key regulators using Fisher's Exact Test (FET) (Fig. 1 *b*; SMGs: Supporting Material 2B, eMSGs: Supporting Material 7). We further inferred abundances of stromal and immune cell components in the primary tumors by using ESTIMATE^14^ and CIBERSORT,^15^ respectively. Gene signatures significantly correlated with cell type compositions were identified and then projected onto gene modules to determine their cell type specificity (Fig. 1 *b*; see Supporting Material 4).

Interestingly, the top modules of TCGA‐GCC network suggest strong cross‐talks among different cell types in GC tumor microenvironment (Fig. 5). As summarized in Figure 5*a*, our data reveal these modules are regulated by somatic mutations in *NEB* and *PIK3CA*, epigenetic alterations (methylation changes by EBV infection) and loss of *GKN1/2* and *TFF1/2* expressions. The complex signaling network across stromal, epithelial and immune cells demonstrates activated or suppressed key oncogenic/tumor suppressive pathways in GC such as epithelial‐mesenchymal (EMT), gastrointestinal mucosal barrier and cytotoxic CD8+ T‐cell/Natural Killer (NK) cell activation (Fig. 5*a*, Table 1). Furthermore, eigen‐genes of these modules (i.e., first principal components of gene modules)^10^ are significantly correlated with each other, suggesting cross‐talks (Fig. 5*b*).

**Table 1:** Summaries of top modules of TCGA‐GCC and their functions in GC microenvironment

Key module	Associated pathways and functions
M28: GC carcinogenesis, intestinal fibrosis	GC epithelial cell specific carcinogenic pathways: KRAS dependency Epithelial specific splicing factor, ESRP1/2 Epithelial carcinogenic genes: HNF4A, EHF, EPCAM Intestinal microvilliar niche: VIL1 and USH1C Interactions with stromal tumor via NEB: Upregulated genes by NEB mutation coincides with M28 NEB mutation correlates to low stromal score NEB mutation signature coincides with GIST tumorigenic pathways (i.e. KIT/PDGFRA mutations)
M666/M121: intestinal mucosa homeostasis	Silenced by EBV via hypermethylation Enterocyte development niche: intestinal intermicrovillar adhesion complex (IMAC), core bundle adhesion Intestinal mucosal barrier maintenance: CDX1/2 regulated MUC2, TFF3
M102: NK‐/T‐cell cytotoxic pathway	Upregulated by EBV via hypomethylation Further up‐regulated by PIK3CA mutation CD8+ T‐cell markers: CD8A, NKG7 TIGIT/CD96 checkpoint signaling RANTES signaling: CCR5, CCL5
M434: gastric mucosal protection, GKN1/2‐TFF1/2 axis	EGJ‐specific loss of GKN2 expression GKN2 activation recovers expression of heterodimeric interaction partner TFF1 GKN2 activation inhibits GC invasion and proliferation *in vitro*

In the rest of the article, we will focus on the modules shown in Figure 1 *c* and comprehensively investigate their network structures and biological implications as well as their interactions in the context of GC tumor microenvironment.

### Deactivation of gastric mucosal barrier drives GC proliferation and invasion

The second ranked module, M434, represents downregulation of tumor suppressors such as *TFF2*, *GKN1* and *GKN2* in gastric mucosal barrier (GMB) homeostasis in GC (Fig. 1 *c*). M434 was highly enriched for the genes downregulated in tumor compared to adjacent normal tissue (Bonferroni corrected FET *p* = 1.04E‐25, 9.25 fold enrichment (FE)) and highly reproduced in vTCGA‐GCC and GSE84437 with high overlaps (% overlap with vTCGA‐GCC = 43%, GSE84437 = 48%).

**Figure 2:**
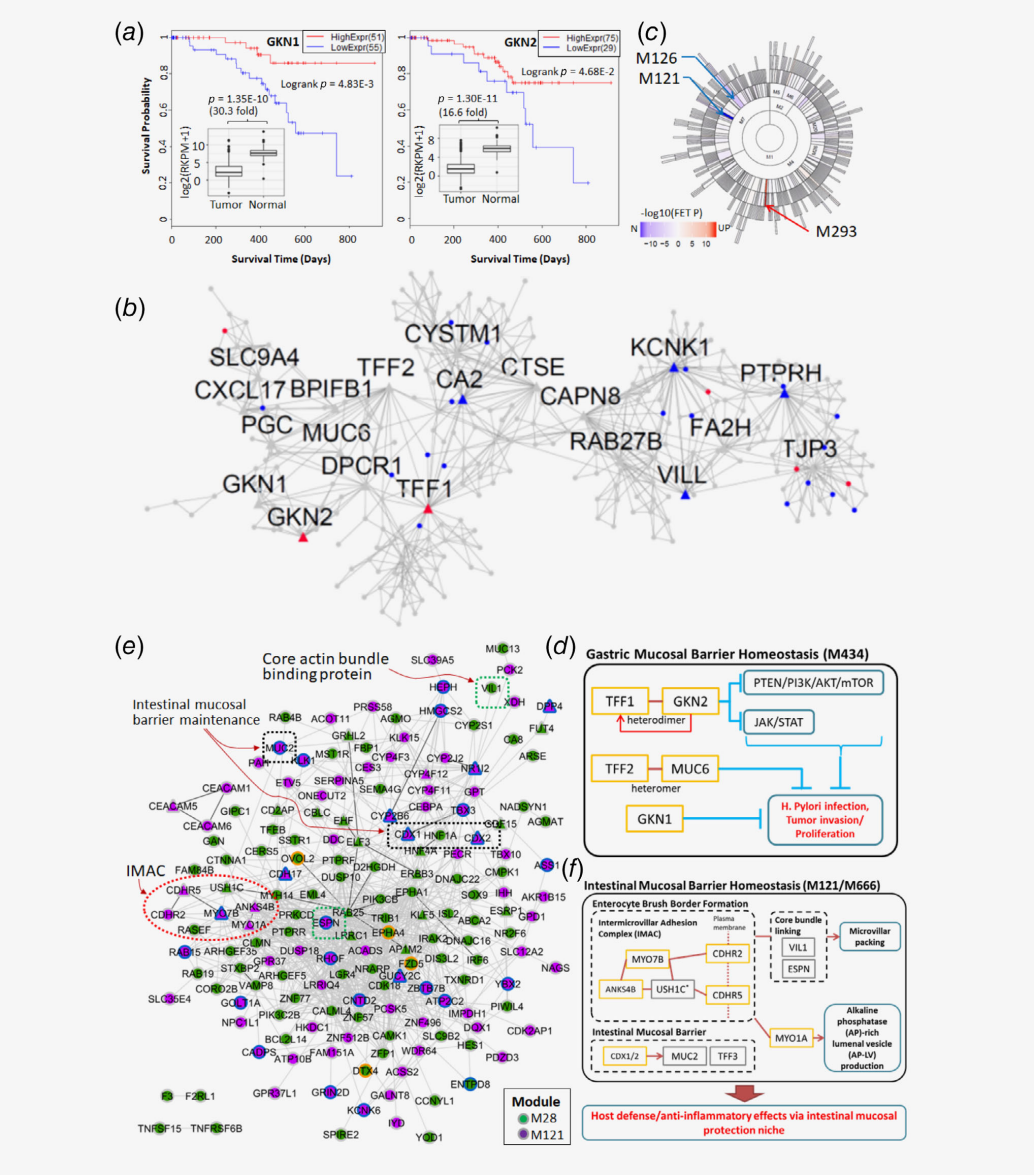
(*a*‐*c*) Gastric Mucosal Barrier (GMB) module, M434, is suppressed in primary GC. (*a*) Prognostic significance of *M434* key drivers, *GKN1/2*, in TCGA‐GCC. Kaplan–Meier plots of node positive patients in TCGA‐GCC by GKN1/2 expressions. Significant suppressions of *GKN1/2* in primary compared to adjacent normal tissues are shown as embedded mini‐plot with relevant *t*‐test statistics on the top. (*b*) 5‐layer neighborhood network of GKN2 in TCGA‐GCC MEGENA. Up‐/down‐regulated genes in *GKN2* overexpressed AGS cells shown in red/blue. (*c*) Significant enrichment of up‐/down‐regulated genes in GKN2 overexpression in TCGA‐GCC MEGENA modules. Hierarchical organization of MEGENA modules are illustrated in the sunburst plot, fill colors representing –log10(FET *p*‐value) of up−/down‐regulated signatures in blue/red, respectively. Most significantly enriched modules are labeled by arrows, namely M121, M294 and M126. (*d*) GMB signaling pathways, captured by key drivers of M434. (*e*) F: Intestinal Mucosal Barrier (IMB) modules, M121/M666, are suppressed through hyper‐methylation by EBV infection. Experimentally validated protein–protein interactions from the String database^16^ are intersected with M28 and M121. Node colors show module membership (M28: green, M121: purple), border colors show hyper‐/hypo‐methylation (hyper: blue, hypo: orange), and dotted box shows specific pathways/complexes in IMB niche. Network structure of *M666*, a daughter module of M121. The red and blue nodes are the genes up‐ and down‐regulated in EBV subtype compared to other molecular subtypes, respectively. (*f*) IMB pathways captured by M121/M666. Some components of IMB are from GC epithelial module, M28 (*USH1C* and *VIL1*). [Color figure can be viewed at http://wileyonlinelibrary.com]

Suppression of the genes in M434 is associated with poor overall survival in GC and increased tumor sizes. For instance, lower expression levels of the key drivers *GKN1* and *GKN2* are associated with poor survival in node positive cases in TCGA‐GCC (Fig. 2 *a*). Within node positive patients, tumor weights in the group with median‐low *GKN1* or *GKN2* expressions were significantly higher than those in the group with median‐high *GKN1* expression (Supporting Information Fig. S3*b*), but are independent of pathological T stage (Supporting Information Fig. S3*c*). We confirmed prognostic significance of *GKN1/2* in an independent km‐plotter cohort^17^ (Supporting Information Fig. S3*e*).

#### *Novel downstream functions of overexpressing GKN2 in GC*

M434 captures a tumor suppressive axis *TFF1‐TFF2‐GKN1‐GKN2* (Fig. 1 *c*), where *TFF2*, *GKN1/2* are found in M434, and *TFF1* is found at a neighboring module directly connected to M434 (Supporting Information Fig. S3*f*). Within the *TFF1‐TFF2‐GKN1‐GKN2* axis, we investigated functional impacts of overexpressing *GKN2* in GC, as detailed downstream mechanisms of *GKN2* are largely unknown, compared to other genes in the axis. We first comprehensively validated clinic‐pathological features of *GKN2* expression in an independent cohort of 104 patients who underwent gastric carcinoma resection in the Fujian Provincial Cancer Hospital (Fujian cohort; see *Patients and Specimens* in Materials and Methods, Supporting Material 1). The correlations between *GKN2* gene expression in cancer tissues and pathological features are shown in Supporting Information Table S2. *GKN2* mRNA expression was significantly correlated with tumor size and location with *p* = 0.023 and 0.006, respectively. The average mRNA level of *GKN2* in the group with large tumors was 5.2‐fold lower than the group smaller tumors while the average expression in the patients with non‐EGJ (esophagogastric junction) was 3.8‐fold higher than EGJ.

Next, we transfected human gastric cancer cell line AGS, with lenti‐virus containing *GKN2* (GKN2‐LV), and performed RNA‐sequencing of GKN2‐LV cells with the control (ctrl‐LV) (see Supporting Materials and Methods, Supporting Material 1; Supporting Information Fig. S6). We confirmed *GKN2* over‐expression confers antitumoral effect and inhibiting invasion in AGS by inhibiting PTEN/PI3K/AKT/mTOR and JAK/STAT axes (see Supporting Results, Supporting Material 1; Supporting Information Figs. S7, S8, S9 and S10). Using *edgeR* 18 (see Supporting Materials and Methods, Supporting Material 1), we identified 253 upregulated and 401 downregulated genes (FDR < 0.05 and fold change >1.2 or < 0.833) that represent the down‐stream pathways of *GKN2* over‐expression in GC (Supporting Information Fig. S3*a*; Supporting Material 4; Fig. S7).

The altered genes by GKN2‐LV were captured in *GKN2*'s neighborhood in the TCGA‐GCC network. The downregulated genes were significantly enriched in *GKN2*'s network neighborhood (Fig. 2 *b*). Interestingly, *TFF1* was upregulated by *GKN2* overexpression (Fig. 2 *a*,*b*), known synergistic heterodimeric interaction partner of *GKN2* conferring antiproliferative and proapoptotic effects on gastric cancer cells.^19^ This suggests *GKN2* alone is capable of conferring antitumor effect.

We systematically identified *de novo* downstream functions of *GKN2* as enriched gene modules by the *GKN2* overexpression signatures (Fig. 2 *c*). M121 and M126 were most significantly downregulated whereas M293 was most significantly upregulated. M121 represents intestinal niche/fibrosis in GC as discussed in later sections, and M293 is enriched for cholesterol biosynthesis pathway.

### GC epithelium interacts with stromal cells via an actin‐binding protein, Nebulin

The third ranked module, M28 (Fig. 1 *c*) contains several GC carcinogenic pathways activated in GC epithelium. M28 is upregulated in tumor compared to adjacent normal tissue (Boferroni FET *p* = 4.43E‐29, 4.72 FE), and its gene interactions conserved in independent cohorts, vTCGA‐GCC and GSE84437 (Fig. 1 *b*; Supporting Material 2B, C).

**Figure 3:**
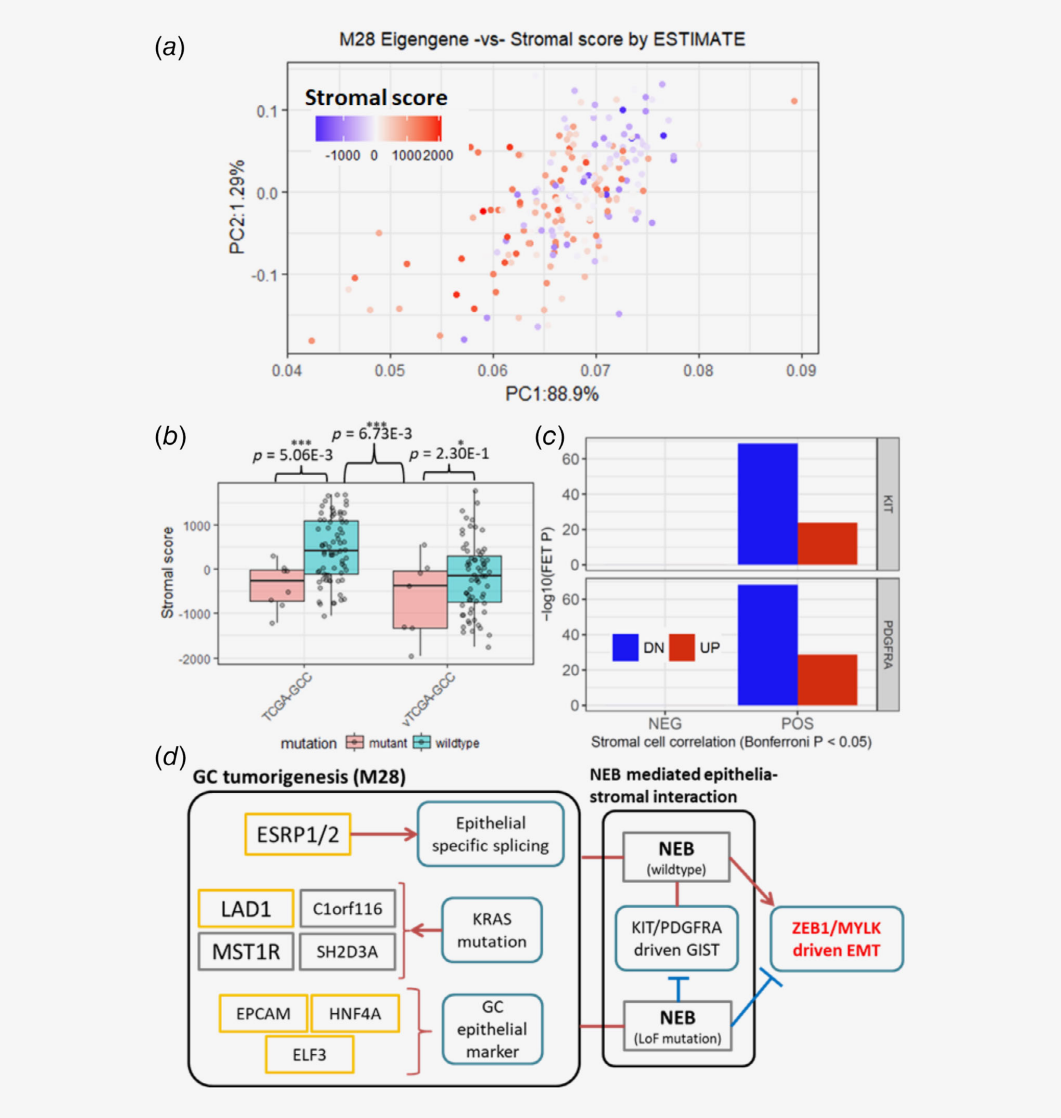
GC epithelial/tumorigenic module, M28, is upregulated in primary GC. (*a*) Correlation between M28 eigen‐gene and TCGA‐GCC stromal score inferred by ESTIMATE algorithm. The x‐ and y‐ axes are the first and second PC of M28, respectively. (*b*) ESTIMATE inferred stromal score distributions by NEB mutation status, in TCGA‐GCC (left) and vTCGA‐GCC (right). (*c*) Enrichment of oncogenic *KIT/PDGFRA* mutation signatures from gastrointestinal stromal tumors (GISTs) in stromal score correlated gene signature in TCGA‐GCC. (*d*) *A GC tumorigenesis pathway captured by M28*, interacts with GC stromal cells via *NEB* to promote epithelial‐mesenchymal transition (EMT) in GC. [Color figure can be viewed at http://wileyonlinelibrary.com]

M28 contains several oncogenic pathways. For instance, M28 significantly intersects with cell viability signatures from KRAS mutant epithelial cells^20^ (Bonferroni corrected FET *p*‐value = 5.421E‐2, 21.8 FE). Comparing by *KRAS* mutation status, *KRAS* mutant viability signatures genes in M28, *MST1R* and *SH2D3A*, were consistently upregulated in nonsynonymous *KRAS* mutants in TCGA‐GCC and vTCGA‐GCC. Furthermore, M28 is epithelial tumor specific. Several key drivers of M28 such as *EPCAM* 14 and *ESRP1* 21 are epithelial tumor cell specific markers (Fig. 1 *c*). Expression of the genes in M28 is negatively correlated with ESTIMATE inferred stromal score (Fig. 3 *a*).

To some extent, the association between M28 and stromal score can be explained by nonsynonymous somatic mutations in an actin‐binding protein, *Nebulin* (*NEB*). *NEB* somatic nonsynonymous mutations were present in 26 (~11.6%) of the 224 samples, and associated to key stromal phenotypes such as lower stromal scores (Fig. 3 *b*) and less occurrence of N3 patients (Supporting Information Fig. S4*c*) in *NEB* mutants. On the contrary, the downregulated *NEB* SMGs are enriched in an epithelial‐mesenchymal transition (EMT) module, M109 (Supporting Information Fig. S4*a*,*B*), a key pathway mediating epithelial‐stromal interaction within tumor microenvironment.^22^

The epithelial‐stromal interaction may involve key tumorigenic pathways in gastrointestinal stromal tumor (GIST). *KIT/PDGFRA* mutations serve as characteristic oncogenic mutations, present in ~98% of GISTs.^23^ The genes downregulated in active *KIT/PDGFRA* mutations from GISTs, curated from the CREED database (initial source data: GSE17743),^24^ are significantly enriched with the stromal score correlated gene signature (Fig. 3 *c*).

Taken together, these results suggest *NEB* may serve as a gateway for epithelial‐stromal interactions in advanced GC microenvironment (Fig. 3 *d*), mediating the cross‐talk between tumorigenic pathways of epithelial and stromal tumor cells, and activating (*NEB* wildtype) or suppressing (*NEB* mutant) EMT, a critical pathway for cancerous cell dissemination.

### GC epithelium leverages intestinal mucosal barrier niche against EBV infection

M28 strongly interacts with the intestinal epithelium specific/goblet cell‐like modules, M666 and its parent module, M121 (Fig. 1 *c*). Eigen‐gene of M666 strongly correlates to M28 eigen‐gene (Fig. 5*b*), and M121/M666 contains genes in several key intestinal mucosal barrier (IMB) functions including the members of the intermicrovillar adhesion complex (IMAC) such as *MYO7B*, *ANKS4B*, *CDHR2* and *CDHR5*, which are critical for enterocyte brush border formation^25^ *ESPN* involved in actin bundling^26^ mucins (*TFF3* and *MUC2*) secreted by intestinal goblet cells^27^ (Fig. 2 *e*,*f*). M121/M666 are also conserved in vTCGA‐GCC and GSE84437 (Fig. 1 *b*; Supporting Material 2B, C).

M121/M666 is specifically silenced in the EBV subtype via hypermethylation. By comparing the EBV subtype and the rest of the samples, we identified the genes downregulated in the EBV subtype (using the threshold of FDR < 0.05 and fold change >1.2) and the cis‐hyper‐methylated genes in the EBV subtype (FDR < 0.05) and then intersected them to derive an EBV‐specific hypermethylation gene signature (EBV‐HEMG; see Supporting Material 6A, B). These EBV‐HEMGs are significantly enriched in M121 (Bonferroni corrected FET *p* < 2.63E‐22, 6.49 FE). Projection of M121 and M28 onto the protein–protein interaction network from the String database^16^ (Fig. 2 *e*) revealed that many key features of IMB maintenance involve interactions with GC epithelium specific pathways (M28), and are suppressed in the EBV subtype. Furthermore, suppression of key genes in M121/M666 including *MYO7B* and *PPP1R14D*, were associated with poor recurrence‐free survival (see Supporting Information Fig. S5*a*,*b*).

### *PIK3CA* mutations and *EBV* infection aberrantly upregulate T‐cell receptors in CD8+ T‐cell cytotoxic pathways

**Figure 4:**
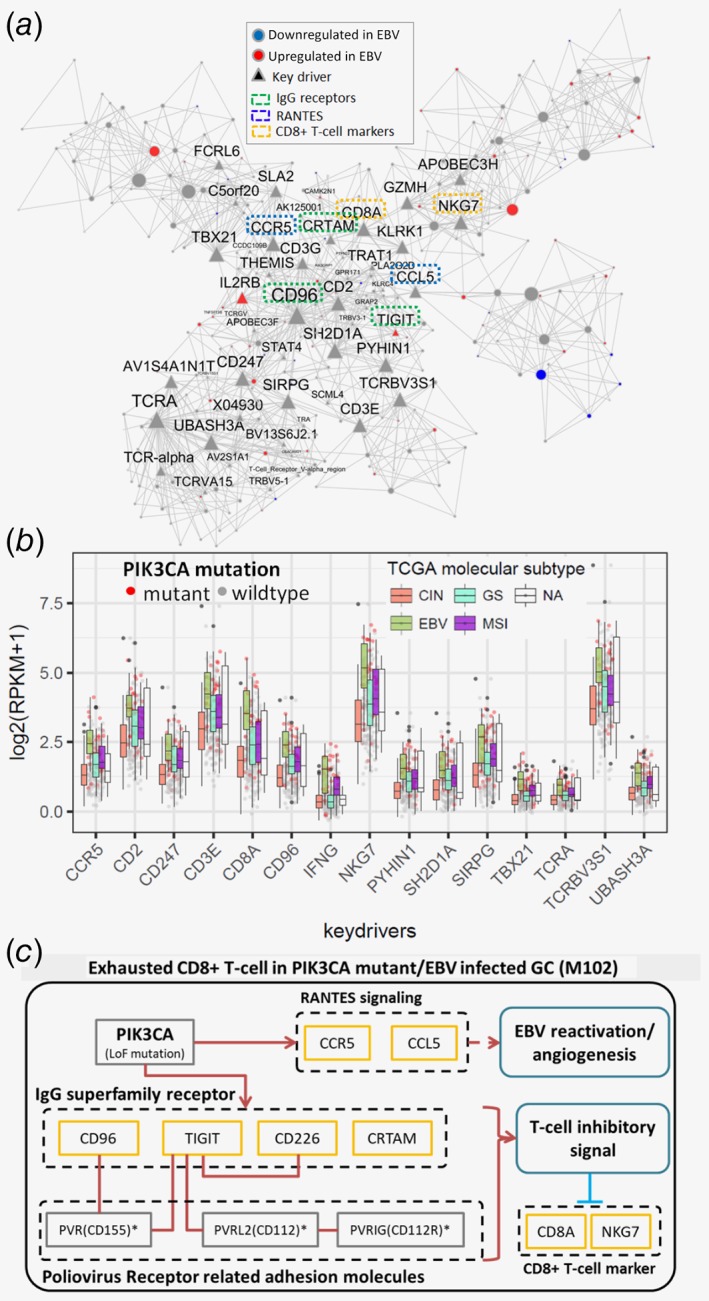
The NK‐/T‐cell cytotoxicity related module, M102, is synergistically upregulated by EBV and PIK3CA mutations. (*a*) Network structure of M102. The red and blue nodes are the genes up‐ and down‐regulated in the EBV subtype compared to the other subtypes. Triangle nodes are the inferred key drivers of M102. The key driver genes involved in specific signaling pathways are labeled by dotted boxes with green (Ig superfamily receptors), blue (RANTES signaling) and yellow (CD8+ T‐cell markers). (*b*) EBV subtype specific upregulation of M102 key drivers. (*c*) Exhausted CD8+ T‐cell pathway captured by M102. Cosignaling T‐cell receptors are upregulated by EBV infection/*PIK3CA* mutation, which may lead to CD8+ T‐cell exhaustion. [Color figure can be viewed at http://wileyonlinelibrary.com]

In contrast to the suppression of the IMB complex in GC, a group of immunoglobulin(Ig)‐superfamily receptors involved in NK−/T‐cell mediated cytotoxicity are activated in the EBV subtype via hypo‐methylation (Fig. 4 *a*,*b*). Similar to the identification of EBV‐HEMGs, we also derived a set of EBV‐specific hypo‐methylation genes (EBV‐HOMGs) that were upregulated and hypomethylated in the EBV subtype in comparison with all the other subtypes (Supporting Material 6A, C). M102, a module associated with immune response, is highly enriched for the EBV‐HOMGs (Bonferroni corrected FET *p* = 1.14E‐69, 8.15 FE), and is conserved in vTCGA‐GCC and GSE84437 (Fig. 1 *b*; Supporting Material 2B, C). Also, cosignaling Ig receptors for T‐cell activation genes including *CD96*, *TIGIT*, *CRTAM* and *CD226*,^28^ were EBV‐HOMGs present in M102 (Fig. 4 *a*).

Using the cell type specific markers from CIBERSORT,^15^ we found that the key driver genes of M102 were specifically expressed in several immune cell types including CD8+ T‐cells, macrophage‐M1, activated CD4 memory T‐cells and activated NK cells (Supporting Material 5E). For instance, expression of *CD96* was highly correlated with the markers in CD8+ T‐cells (Spearman ρ = 0.572, *p* = 2.39E‐20), activated CD4 memory T‐cells (Spearman ρ = 0.511, *p* = 5.04E‐12), macrophage‐M1 (Spearman ρ = 0.452, *p* = 1.63E‐8) and activated NK (Spearman ρ = 0.180, *p* = 7.56E‐3) cell populations.

Enrichment of *PIK3CA* mutations in the EBV subtype has been well established within TCGA‐GCC.^5^ Not surprisingly, the upregulated *PIK3CA* SMGs in the TCGA‐GCC tumors are highly enriched in M102 (Bonferroni FET *p* = 1.89E‐110, 14.3 FE), and M102 key drivers including *TIGIT* (co‐inhibitory Ig receptor) show higher expressions in EBV subtype, compared to other subtypes (Fig. 4 *b*). These results indicate *PIK3CA* mutations and EBV infection synergistically upregulate NK−/T‐cell cytotoxic pathways including cosignaling T‐cell activation receptors (Fig. 4 *c*).

## Discussion

**Figure 5:**
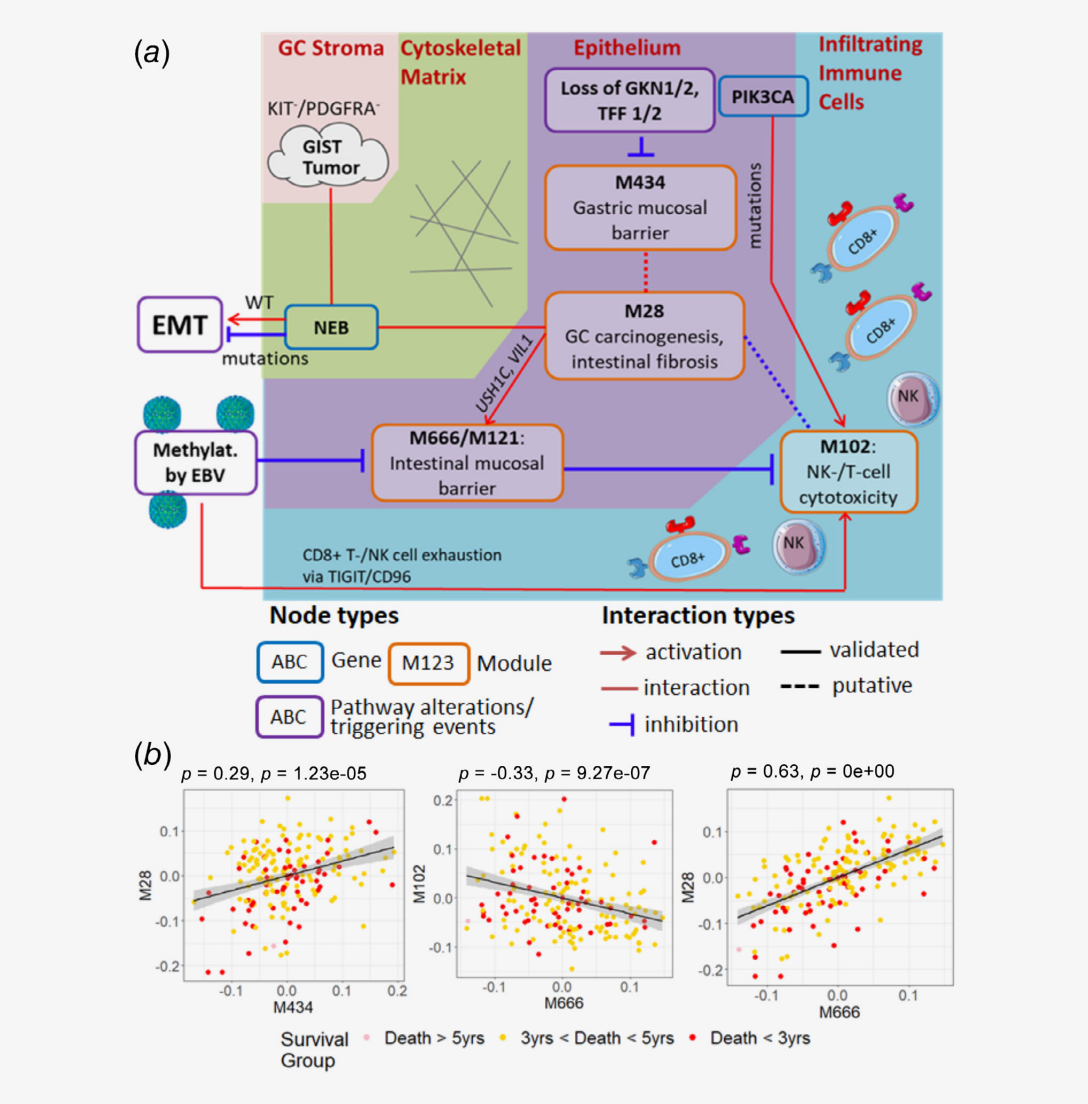
Molecular interactions among stromal, epithelial and immune cells in GC microenvironment. (*a*) Key prognostic pathways in GC: The most prognostic network modules (M666/M121, M434 and M28) and their interacting partner M102 capture complex signaling circuits among stromal, epithelial and immune cells in GC microenvironment, involving genetic (somatic mutations in *NEB* and *PIK3CA)*, epigenetic (altered methylation by EBV infection) and transcriptomic alterations (loss of *GKN1*, *GKN2, TFF1* and *TFF2*) as triggering events. Respective cellular domains/cell types are bordered by different background colors. Activation, interaction and inhibition relationships are shown. Interactions between genes with different mutation status and pathways are further labeled with *WT* (wildtype) and *mutations* (mutated). Putative interactions identified by the TCGA‐GCC network, but without literature support or independent validations are marked in short‐dashed lines. (*b*) Cross‐talks across key prognostic pathways. Spearman's correlations between eigen‐genes of key prognostic modules were computed to evaluate interactions/cross‐talks between modules. Coefficients and significance *p*‐values are shown on the top. Colors represent overall survival of respective patients. [Color figure can be viewed at http://wileyonlinelibrary.com]

Our integrative network approach not only revealed robust network structures and key regulators of GC, but also presented detailed signaling circuits underlying survival‐associated GC microenvironments involving cross‐talks among epithelial, stromal and immune cells (Table 1; Fig. 5 *a*,*b*). We validated *GKN2*, an emerging driver of gastric mucosa, through *in silico* and *in vitro* perturbation experiments as well as pathological examination of a large number of human gastric cancer specimens.

### Gastrointestinal mucosal barrier: different modes of protections for different contexts of GC progression

Several modules involved gastric‐specific mucosa (M434) and intestinal‐specific mucosa (M121 and M666) emerged as key tumor suppressive pathways in GC. Our analysis reveals different modes of operations for these mucosal protective functions. The gastric mucosal barrier (GMB) module is specifically associated with GC proliferation and invasion while the intestinal mucosal barrier (IMB) niche is specifically associated with the EBV infection.

#### *GKN2 is an independent regulator of GC proliferation and invasion in GMB niche*

The GMB module (M434), ranked as the second module most predictive of survival time, reveals the antitumor effect of the *TFF1‐TFF2‐GKN1‐GKN2* axis. These genes are robustly downregulated in GC compared to normal gastric tissue^29^ and their downregulation in tumor is associated with increased tumor size and poor overall survival in GC. These findings are confirmed in the independent Fujian cohort.

The *TFF1‐TFF2‐GKN1‐GKN2* axis exerts pressures on GC tumorigenesis via gastric mucosal defense^30^ silencing oncogenic *PTEN/PI3K/AKT/mTOR* 31 *JAK/STAT* pathway^32^ *ERK1/2* axes^33^ and eventually inhibits GC proliferation and invasion.^19^, ^32^ *GKN1* is known to play an important role in the progression of gastric cancers via inhibition of EMT and cancer cell migration^34^ and *TFF2* forms heteromer with *MUC6*, which assembles and stabilizes the laminated structure of gastric mucus and has antibiotic activity against Helicobacter pylori.^30^ Heterodimeric interaction between *GKN2* and *TFF1* has synergistic antiproliferative and proapoptotic effects in GC.^19^

In our study, *in vitro* overexpression of *GKN2* in GC cells silences *PTEN/PI3K/AKT/mTOR* and *JAK/STAT* pathway, and inhibits GC proliferation and invasion as previously reported. Interestingly, overexpression of *GKN2* alone recovered expression of its heterodimeric interaction partner, *TFF1* to confer antitumor effects.

On the contrary, *GKN2* overexpression upregulated cholesterol synthesis module, M293, includes *HMGCR*, which promotes GC growth and migration.^35^ Although we observed a clear antitumor effect of *GKN2* overexpression in our clinical and *in vitro* studies, the unexpected activation of a GC oncogenic pathway requires further in‐depth investigation to clarify its roles conditioned on recovered *GKN2* functions *in vivo*.

#### *EBV infection silences the IMB niche*

Two top ranked modules, M121 and M666, include many of intestinal epithelia specific genes in the IMB niche and are specifically downregulated by hypermethylation in the EBV subtype.

The IMB niche in GC acquires several intestinal features such as brush border formation and intestinal cell specific goblet cells. For instance, IMAC is a critical adhesion complex for brush border formation, and consists of *USH1C*, *MYO7B*, *ANKS4B* and intestinal specific protocadherins *CDHR2/5* as essential components for the complex formation.^25^ *USH1C* belongs to GC epithelial specific module M28 while *MYO7B*, *ANKS4B* and *CDHR2/5* fall into M121. These data suggest that IMAC formation requires contributions from both actively expressed intestinal components and GC specific epithelium to become fully functional.

On the other hand, the IMB niche involving M121 and M666 is not limited to the intestinal subtype in TCGA‐GCC. For instance, an intestinal brush border specific gene, *ESPN*, which falls into both M121 and M666, is not differentially expressed between intestinal and diffuse subtypes in TCGA‐GCC (*t*‐test *p* = 0.93). Many other genes in the modules show similar results. Although intestinal phenotypes such as gland formation and fibrosis are observed in early stages of GC and atropic gastritis^36^ and constitute a major subtype of GC as intestinal subtype by Lauren classification,^12^ the IMB niche defined by M121 and M666 is generally applicable to a broader spectrum of GC beyond the intestinal subtype.

In summary, the IMB niche represents an emergent protective mechanism in advanced GC. With the known protective roles of IMB in host defense in intestine against gut microbiome^37^ the observed suppression of gastric IMB may indicate a prerequisite for EBV infection to function in GC development and progression.

### Nebulin mutations: blocking stromal–epithelial cell interactions in GC

Stromal cells constitute large portions of GC tumors, and cancer‐stromal cell interactions promote tumor growth and metastasis.^38^ Coexistence of often benign gastro‐stromal intestinal tumors (GISTs) with gastric adenocarcinoma has been frequently observed.^23^ In our TCGA‐GCC analysis, a KRAS dependent GC tumorigenic module (M28) associates to varying degree of “stromal‐ness.” This stromal‐ness corresponded to GIST specific oncogenic mutations, *KIT* and *PDGFRA*,^23^ pertaining stromal–epithelial interactions in GC.

In our study, somatic nonsynonymous mutations in an actin‐binding protein, Nebulin (*NEB*) is associated to this stromal–epithelial interactions. Nebulin is a giant 600‐ to 900‐kDa filamentous protein constituting cytoskeletal matrix that coexists with the thick and thin filaments within the sarcomeres of skeletal muscle^39^ and is an overexpressed protein in gastric cancer tissues compared to normal.^40^ However, its roles in GC progression have remained largely unknown.

We present *NEB*‐centered molecular mechanisms of GC invasion by leveraging differentially expressed genes in *NEB* somatic nonsynonymous mutation. Specifically, upregulated genes in NEB mutants coincided with GC epithelial specific M28, and the respective downregulated genes were associated to EMT module (M109), which bears *ZEB1* as a top key driver, a key transcription factor in EMT and metastasis^41^ (Supporting Information Fig. S4*a*,*b*). Another interesting key driver of M109 is *MYLK* (myosin light chain kinase), which phosphorylates myosin regulatory light chains to facilitate myosin interaction with actin filaments to produce contractile activity,^42^ and involved in cell motility and morphology.

Together with *NEB* functions in maintaining cytoskeletal matrix, the downregulation of M109 in *NEB* mutants strongly suggests disruption of dynamic changes in actin cytoskeleton via loss‐of‐function mutation in *NEB*, a necessary process of EMT for cancerous cells to acquire cell motility and morphological changes.^22^ This also explains the underrepresentation of N3 patients in *NEB* mutant samples in TCGA‐GCC (Supporting Information Fig. S4*c*).

Overall, *NEB*‐centered GC mechanisms illustrate the complex cross‐talks between GC stroma and epithelium via gastric actin cytoskeleton, and propose *NEB* mutation as a protective mechanism by disrupting EMT.

### PIK3CA and EBV driven methylation: recipes for GC immune evasion

M102 is NK−/T‐cell cytotoxic pathway module, including key receptors for T‐cell and NK‐cell activation for immune surveillance of GC tumor. Interestingly, M102 is subject to combination of genetic alteration (*PIK3CA* mutation) and epigenetic alteration (hypomethylation in EBV subtype), which synergistically upregulated M102 compared to other GC subtypes in TCGA‐GCC.

Among the key driver genes of M102, *TIGIT* is a poliovirus receptor (PVR)–like protein, an immunoreceptor expressed in T‐cells that acts as inhibitory checkpoint on both of T‐cells and NK‐cells.^43^ Recently, *TIGIT* emerged as a promising immune checkpoint blockade target in GC. TIGIT+ CD8+ T‐cell population increases in GC, and these cells show functional exhaustions impairing antitumoral activities.^44^ Blocking TIGIT showed synergistic effect in recovering antitumoral CD8+ T‐cell functions with anti PD‐L1 treatment.^44^ As M102 also includes CD8+ T‐cell markers such as *CD8A* and *NKG7* as key drivers, these strongly suggest M102 depicts exhausted CD8+ T‐cells by exploiting coinhibitory *TIGIT* signaling.

The top key driver of M102, *CD96* (also known as T Cell‐Activated Increased Late Expression Protein), is expressed in T‐ and NK‐cells with adhesive functions to modulate their interactions and enhance cytotoxicity.^45^ In HIV‐1 infected adults, *CD96* is associated with different cell effector functions of CD8+ T cells.^45^ It has been shown that targeting NK cell activity via *CD96* has promising anticancer potential, complementary to the existing *PD‐1* and *PD‐L1* targeted therapeutics^46^ and suppresses metastasis in melanoma lung metastasis mouse model (B16F10).^47^ To our knowledge, the effectiveness of *CD96* blockade, thereby recovering NK‐cell antitumoral activity in GC has not been explored.

These results suggest targeted immunotherapy for *PIK3CA*‐mutant and/or EBV‐infected GC patients, via targeting adaptive and innate immune response systems. Especially, *CD96* and *TIGIT* are attractive blockade targets in such GC patients though future investigation is needed to confirm the finding.

In summary, the most prognostic coexpression network modules reveal a series of pathways representing complex cross‐talks among epithelial, stromal and immune cells in GC (Fig. 5 *a*), which are potentially driven by environmental factor (i.e., EBV infection), genetic alterations (i.e., *NEB* and *PIK3CA* mutations) and epigenetic alterations (i.e. hypo‐/hyper‐methylations). We further systematically investigate key regulators and proposed interactions of the top modules. In particular, we validate antitumor effects of one key network driver *GKN2* overexpression and identified downstream pathways of *GKN2* in GC. These findings enable generation of novel hypotheses regarding complex interplay among multifaceted axes (e.g., different pathways and different cell types) in GC progression. For instance, we propose the IMB niche in M121 and M666 as a suppressor of EBV driven immune evasion in M102, where host defense function of the IMB niche may be capable of inhibiting EBV activation.

Our integrative network analysis of TCGA‐GCC reveals some fundamental patterns of molecular interactions and specific mechanisms in GC progression. The network models and the key regulators identified here pave a way for defining novel therapeutic strategies. Future work will include validation of prioritized key drivers and subnetworks in GC and develop network‐based biomarkers for stratifying GC patients.

## Supporting information

Supporting Material 1. Supporting Materials and Methods Descriptions, Supporting Results and Supporting Figures 1–10.
Supporting Material 2. A. Ranked table of gene modules identified by MEGENA. B. Module preservation statistics of TCGA‐GCC modules in GSE84437 and vTCGA‐GCC cohorts. C. Patient barcodes of primary tumor samples used in TCGA‐GCC and vTCGA‐GCC.
Supporting Material 3. A. Somatic mutation oncomatrix generated using maftools R package. B. Significant enrichment of somatic mutation gene signatures in MEGENA modules with BH FDR corrected FET p‐value <0.05.
Supporting Material 4. Differentially expressed genes in *GKN2* over‐expressed AGS cells compared to the mock control.
Supporting Material 5. Computationally inferred cell types in TCGA‐GCC primary tumor samples. A. ESTIMATE inferred stromal/immune cell scores. B. CIBERSORT inferred immune cell type compositions. C. ESTIMATE inferred stromal cell correlated gene signature in TCGA‐GCC. D. ESTIMATE inferred stromal cell correlated gene signature in vTCGA‐GCC. E. Correlation between hub genes of M102 and CIBERSORT inferred cell type compositions.
Supporting Material 6. Differentially expressed genes (DEGs) and differentially methylated CpG sites (DMR) in EBV subtype, compared to other primary GC in TCGA‐GCC. A. Differential expression between EBV subtype and the rest of primary GC in TCGA‐GCC. B. List of genes in EBV‐specific hyper‐methylation gene signature (EBV‐HEMG) by intersecting significantly down‐regulated genes and hyper‐methylated genes in *cis* in EBV subtype. C. List of genes in EBV‐specific hypo‐methylation gene signature (EBV‐HOMG) by intersecting significantly up‐regulated genes and hypo‐methylated genes in *cis* in EBV subtype.
Supporting Material 7. Supporting results for correlation analysis between methylation and gene expression profiles. A. cis−/trans‐eMSG signatures identified by Bonferroni corrected p‐value <0.05 by Spearman correlation analysis. B. Table of all pairwise tests between eMSGs and MSigDB signatures. C. Table of all pairwise tests between eMSGs and gene modules of TCGA‐GCC.
