# Supplementary material for: Multiscale network analysis reveals molecular mechanisms and key regulators of the tumor microenvironment in gastric cancer
Source: Int J Cancer. 2019 Oct 11;146(5):1268–80. doi: 10.1002/ijc.32643 (PMC7004118; doi:10.1002/ijc.32643)
Supplement: Supplementary file 1 — Supporting Material 1. Supporting Materials and Methods Descriptions, Supporting Results and Supporting Figures 1–10. Supporting Material 2. A. Ranked table of gene modules identified by MEGENA. B. Module preservation statistics of TCGA‐GCC modules in GSE84437 and vTCGA‐GCC cohorts. C. Patient barcodes of primary tumor samples used in TCGA‐GCC and vTCGA‐GCC. Supporting Material 3. A. Somatic mutation oncomatrix generated using maftools R package. B. Significant enrichment of somatic mutation gene signatures in MEGENA modules with BH FDR corrected FET p‐value <0.05. Supporting Material 4. Differentially expressed genes in GKN2 over‐expressed AGS cells compared to the mock control. Supporting Material 5. Computationally inferred cell types in TCGA‐GCC primary tumor samples. A. ESTIMATE inferred stromal/immune cell scores. B. CIBERSORT inferred immune cell type compositions. C. ESTIMATE inferred stromal cell correlated gene signature in TCGA‐GCC. D. ESTIMATE inferred stromal cell correlated gene signature in vTCGA‐GCC. E. Correlation between hub genes of M102 and CIBERSORT inferred cell type compositions. Supporting Material 6. Differentially expressed genes (DEGs) and differentially methylated CpG sites (DMR) in EBV subtype, compared to other primary GC in TCGA‐GCC. A. Differential expression between EBV subtype and the rest of primary GC in TCGA‐GCC. B. List of genes in EBV‐specific hyper‐methylation gene signature (EBV‐HEMG) by intersecting significantly down‐regulated genes and hyper‐methylated genes in cis in EBV subtype. C. List of genes in EBV‐specific hypo‐methylation gene signature (EBV‐HOMG) by intersecting significantly up‐regulated genes and hypo‐methylated genes in cis in EBV subtype. Supporting Material 7. Supporting results for correlation analysis between methylation and gene expression profiles. A. cis−/trans‐eMSG signatures identified by Bonferroni corrected p‐value <0.05 by Spearman correlation analysis. B. Table of all pairwise tests between eMSGs a [file IJC-146-1268-s001.pdf]

Supporting Materials for *Multiscale Network Analysis Reveals Molecular Mechanisms and Key Regulators of the Tumor Microenvironment in Gastric Cancer*

Won-Min Song, Xiandong Lin, Xuehong Liao, Dan Hu, Jieqiong Lin, Umut Sarpel, Yunbin Ye, Yael Feferman, Daniel M. Labow, Martin J. Walsh, Xiongwei Zheng and Bin Zhang

Table of Contents

Supporting Material 1

Supporting Materials and Methods 3

Bioinformatics Analysis 3

Co-expression network analysis 3

Extraction of Somatic Mutation Gene Signatures (SMGs) 3

Extraction of cis-/trans-methylation significantly correlated gene signatures (cis-/trans-eMSGs) 4

Extraction of EBV subtype specific differentially expressed genes (DEGs) and differentially methylated CpG sites (DMRs) 4

Acquiring prognostic significance of individual gene expressions from KM-plotter 4

Extraction of gene perturbation signatures: 4

In-silico validation of gene-gene interactions 5

Eigen-gene analysis to clinical traits 5

Code Availability 5

Experimental Procedure 6

Tissue microarray and Immunohistochemical Staining for GKN2 6

Statistical analysis of pathological features 6

Real-time RT-PCR assay 6

Lentiviral Transduction and Establishment of Stable Cell Lines 7

Cellular fractionation and western blotting (WB) 7

Cell viability assay using the xCELLigence Real Time Cell Analyzer (RTCA) 7

Measurement of colony formation 8

Cell migration and invasion assay 8

RNA preparation for mRNA-Seq 8

Library construction and sequencing 8
Supporting Results 9
NEB mutations as an up-stream regulator of Epithelial-Mesenchymal Transition (EMT) pathways 9
GKN2 over-expression inhibits proliferation and invasion of gastric cancer cells........ 9
GKN2 suppresses the oncogenic PTEN/PI3K/AKT/mTOR and JAK/STAT pathways 10
Global epigenetic alterations functionally impact key pathways in gastric cancer progression 10
Supporting Tables 12
Supporting Figures 14
References........ 25
Supporting Material 2A 27
Supporting Material 2B 28
Supporting Material 2C 30
Supporting Material 3A 31
Supporting Material 3B 32
Supporting Material 4 34
Supporting Material 5A 40
Supporting Material 5B 45
Supporting Material 5C 48
Supporting Material 5D 69
Supporting Material 5E 75
Supporting Material 6A 76
Supporting Material 6B 87
Supporting Material 6C 88
Supporting Material 7A 89
Supporting Material 7B 90
Supporting Material 7B 100

Supporting Materials and Methods

Bioinformatics Analysis

Co-expression network analysis

Gene co-expression networks were identified by using Multiscale Embedded Gene Co-expression Network Analysis (MEGENA)[1]. Briefly, MEGENA first selects gene pairs with significant correlations (FDR < 0.05) and then embeds them onto a 3-dimensional topological sphere. The resulting co-expression network belongs to a class of geometrical networks called "planar filtered networks (PFNs)" which can be drawn on the surface of the sphere without any link intersections. PFNs then go through unsupervised clustering for identification of network clusters (*i.e.* gene modules) at various resolutions[1]. The resulting gene modules are organized in a hierarchy. The hierarchy represents multi-scale organization of gene modules with different degrees of compactness. It captures a series of relationships of higher-order (i.e. parent) modules possessing children modules residing within these parent modules. MEGENA recognizes more compact children modules within the parent modules[1]. Furthermore, candidate key drivers of gene modules are further identified by means of statistically significant hubs[1]. Gene modules are then annotated by the enriched MSigDB signatures and associated with outcomes through the enrichment test of the previously identified gene. For MSigDB signatures, we used publicly curated functions and pathways signatures from v5.0: c2.cp.biocarta (BIOCARTA selection), c2.cp.kegg (KEGG), c2.cp.reactome (REACTOME), c5.bp (Gene ontology – biological processes), c5.cc (Gene ontology – cellular components), c5.mf (Gene ontology – molecular functions), c6.all (oncogenic signatures) and h.all (hallmark signatures). MEGENA yielded total of 221 gene modules with sizes greater than 50 genes.

Extraction of Somatic Mutation Gene Signatures (SMGs)

We first downloaded publicly available somatic mutation calls by Varscan pipeline for gastric cancer from The Cancer Genome Atlas (TCGA) database (https://portal.gdc.cancer.gov/, chosen fields: Primary site-Stomach, Data Type-Masked Somatic Mutation, Workflow Type-VarScan2 Variant Aggregation and Masking). We processed the downloaded .maf file using R package *maftools* (version 1.1.60)[2]. Using maftools software, we further excluded mutations that are: i) silent (flags: 3'UTR, 5'UTR, 3'Flank, Targeted_Region, Silent, Intron, RNA, IGR, Splice_Region, 5'Flank, lincRNA), ii) any of the following genes marked as blacklist in *maftools* package : TTN, MUC16, SYNE1, FLG, iii) and genes whose non-silent mutations were less than 5% of all samples (i.e. mutations less than 4 samples were discarded for primary tumor analysis). Per gene with somatic mutations after these filters, differentially expressed genes in mutant samples compared to the wildtypes were identified by R package *limma* version 3.28.17 based on Benjamini-Hochberg False Discovery Rate (BH FDR) < 0.05 and expression fold change (FC) > 1.2.

Extraction of cis-/trans-methylation significantly correlated gene signatures (cis-/trans-eMSGs)

We leveraged publicly available Illumina Infinium 450k methylation BeadChip array data for TCGA-GCC cohort data analysis. We leveraged beta-values, and applied the same data quality control procedure as gene expression data: quantile normalization, followed by correction for batch effects by center, platform and tissue source site (TSS) ids from TCGA sample barcodes, and correction for confounding factors including race, age and gender. This led to 355 primary tumor samples for methylation alone, and 211 samples after intersecting for common samples with gene expression data. In order to define cis-regulatory CpG sites, we leveraged the annotations for CpG probes mapped to respective gene symbols for Illumina 450k methylation array. Within cis pairs and trans pairs, we applied Spearman correlation test to evaluate statistical significance with Bonferroni corrected p-value < 0.05 as the significance threshold. This resulted to: negative cis-eMSGs – 4,004 genes, positive cis-eMSGs – 1,529 genes, negative trans- eMSGs – 8,266 genes, and positive trans- eMSGs – 7,352 genes.

Extraction of EBV subtype specific differentially expressed genes (DEGs) and differentially methylated CpG sites (DMRs)

To obtain the EBV specific DEGs, we applied limma (3.36.1) to the normalized TCGA-GCC gene expressions by contrasting EBV subtype (as compiled by previous TCGA publication[3]) against the non-EBV subtypes within primary GC tumors in TCGA-GCC. Significant DEGs were defined by FDR < 0.05 with fold change > 1.2 for up-regulations, and fold change <1/1.2 for down-regulations. To obtain EBV specific DMRs, we similarly applied limma to the normalized beta-values by contrasting EBV subtype against non-EBV subtype samples with FDR < 0.05. The full results of EBV specific DEGs and DMRs are reported in Supplemental Material 5.

Acquiring prognostic significance of individual gene expressions from KM-plotter

We utilized publicly available microarray gene expression data from km-plotter website for gastric cancer (http://kmplot.com/analysis/index.php?p=service&cancer=gastric). We specifically required data sets used for analysis to exclude QC-failing arrays (Array Quality Control = "exclude biased arrays") and further exclude GSE62254, which is reported to have markedly different survival characteristics by the authors. We chose to use "auto select best cutoff = yes", and ran analysis on overall survival (i.e. OS in Survival dropdown menu).

Extraction of gene perturbation signatures:

- ESRP1/2 knock down signature: We extracted ESRP1/2 double knock-down gene expression signatures by short interfering RNA (siRNA) knockdown in human PNT2 cells as described by Warzecha et al. 2009.6 The microarray data were publicly available from Gene Expression Omnibus (GEO) under accession number, GSE17468. The downloaded data were further quantile normalized, then t-test was performed between 4 replicates of GFP controls

and 4 replicates of ESRP1/2 double knock-down samples. FDR adjusted p-value < 0.05 with fold-change > 1.2. Gene symbols from significantly differentially expressed probes were aggregated. This resulted in 1,025 up-regulated, and 736 down-regulated genes.

- GKN2 over-expression signature: 2 replicates of lenti-viral transduced AGS cells over-expressing GKN2 were subject to RNA-sequencing, and Fragments Per Kilobase of transcript per Million mapped reads (FPKM) values were calculated. R package software "edgeR"[4] was utilized to calculate differentially expressed genes with FDR corrected p-value < 0.05, fold change > 1.2. This resulted in 253 up-regulated, and 401 down-regulated genes.

In-silico validation of gene-gene interactions

We validated the network structures around ESRP1/2 and GKN2 using the gene perturbation signatures derived independent experiments (see *Extraction of gene perturbation signatures*). The gene perturbation signatures were projected onto the neighborhoods of the corresponding genes in a given PFN for enrichment test. Specifically, l-layer neighborhood of a gene, g, is defined as the set of genes whose shortest paths to g consists of l edges at most. The network structure around a gene is successfully validated if the BH FDR corrected FET p-value < 0.05 within 5-layer neighborhood.

Eigen-gene analysis to clinical traits

To relate each cluster with clinical outcomes, principal component analysis (PCA) is first performed for each cluster and then the correlation between the first (or multiple) principal component(s) and each trait is computed as cluster relevance to the trait.

For patient survival data, the association is examined by multivariate Cox proportional hazards regression model that regresses patient survival onto the first (or multiple) principal component(s) of a given module, and Cox p-value is calculated to evaluate the significance. To further investigate prognostic power of each cluster, logrank p-value is calculated to characterize the difference between the survival curves of two molecular subtypes defined by the median expression of the first PC of each cluster.

Code Availability

MEGENA software (version 1.3.7) is publicly available as R package in The Comprehensive R Archive Network (CRAN) (https://cran.r-project.org/web/packages/MEGENA/index.html). The development version of MEGENA is available from GitHub repository (https://github.com/songw01/MEGENA). Other codes for performing Somatic Mutation Gene (SMG) signatures, methylation correlated signatures (cis-/trans-meSCGs) and *in-silico* gene-gene interaction validation are available from the corresponding author upon reasonable request.

Experimental Procedure

Tissue microarray and Immunohistochemical Staining for GKN2

Tissue microarray recipient blocks containing 104 gastric cancer tissues and normal tissues were constructed from formalin-fixed paraffin embedded specimens. We located the appropriate tissue of the normal tissue and tumor under the microscope. Then three tissue cores (1.5 mm in diameter) were transferred to a new recipient paraffin block using a microarray instrument, according to established methods. One cylinder of normal gastric mucosa adjacent to each tumor was also transferred to the recipient block. Sections (3 mm thick) were cut the day before use and stained according to standard protocol.

Immunostaining was done on 5-um paraffin embedded tissue sections using antigen retrieval with EDTA buffer and the EliVision plus horseradish peroxidase. The sections were incubated overnight at 4˚C with GKN2 antibodies (1/200; Abcam, Cambridge, UK). The results were reviewed independently by two pathologists. Immunostaining was assessed semiquantiatively by measuring both the intensity of the staining (0,1,2,or3) and extent of staining(0,0%;1,0-10%;2,10-50%;3,50-100%).The scores for the intensity and extent of staining were multiplied to give a weighted score for each case (maximum possible,9). For the statistical analysis, the weighted scores were grouped in two categories where scores of 0-3 were considered negative and 4-9,positive.

Statistical analysis of pathological features

All data were expressed as means ± standard error of the mean (SEM) obtained from at least three independent experiments. Statistical comparisons between experimental and control groups were assessed by using the Student's t-test. $P < 0.05$ was considered statistically significant.

Real-time RT-PCR assay

Quantitative RT-PCR was used to validate the network results. Total RNA from 104 paired gastric cancer and adjacent tissues was treated with DNaseI (Sigma, St Louis, MO, USA) to eliminate any genomic DNA contamination. Reverse transcription for mRNAs was performed using M-MLV Reverse Transcriptase (Takara, Japan). The cDNA template was amplified by real-time RT-PCR using the SYBR Green Master Mix (Roche, USA). Primers were designed using Primer Premier 5.0 software. Real-time RT-PCR reactions were performed in triplicate on the ABI7500 system (Applied Biosystems, CA, USA).

Using the comparative Ct method $2^{-\Delta\Delta Ct}$, and gastric carcinoma sample No. 99 as a calibrator, the relative expression levels in all gastric carcinoma samples and adjacent non-tumorous tissues were quantified. Expression levels of mRNA were normalized to β-actin mRNA expression.

Lentiviral Transduction and Establishment of Stable Cell Lines

The lentiviruses containing GKN2(GKN2-LV) for overexpressing and control (ctrl-LV) were purchased from Genechem Co., Ltd. (Shanghai, China). The targeting sequences are GKN2-LV (5′-ATGAAAATACTTGTGGCATTTCTGGTGGTGCTGACCATCTTTGGGATACAATCTCATGGATACGAGGTTTTTAACATCATCAGCCCAAGCAACAATGGTGGCAATGTTCAGGAGACAGTGACAATTGATAATGAAAAAAATACCGCCATCATTAACATCCATGCAGGATCATGCTCTTCTACCACAATTTTTGACTATAAACATGGCTACATTGCATCCAGGGTGCTCTCCCGAAGAGCCTGCTTTATCCTGAAGATGGACCATCAGAACATCCCTCCTCTGAACAATCTCCAATGGTACATCTATGAGAAACAGGCTCTGGACAACATGTTCTCCAGCAAATACACCTGGGTCAAGTACAACCCTCTGGAGTCTCTGATCAAAGACGTGGATTGGTTCCTGCTTGGGTCACCCATTGAGAAACTCTGCAAACATATCCCTTTGTATAAGGGGGAAGTGGTTGAAAACACACATAATGTCGGTGCTGGAGGCTGTGCAAAGGCTGGGCTCCTGGGCATCTTGGGAATTTCAATCTGTGCAGACATTCATGTT-3′). All the transfection experiments were performed according to the manufacturer’s instructions. GKN2-LV was transduced into AGS cells at a multiplicity of infection (MOI) of 100 and 10 respectively. Polybrene (Genechem, Shanghai, China) was added in each well at a final concentration of 10 μg/mL to enhance infection solution. The effects of gene interference on GKN2 overexpression were validated using RT-qPCR and Western blotting.

Cellular fractionation and western blotting (WB)

Protein extracts (35 μg) from the different groups of gastric cancer cells were loaded onto 8–12% SDS-PAGE gel (Beyotime), then subjected to electrophoresis for 90 min at 100 V and transferred to NC membranes. The membranes were blocked and incubated with primary antibodies overnight at 4°C, followed by washing and incubation with horseradish peroxidase (HRP)-conjugated secondary antibodies. Immunoreactive bands were visualized by enhanced chemiluminescence (ECL, Thermo) and exposed to x-ray film. Relative protein expression was determined by image analysis using ChemiDocTM XRS gel imaging system(Bio-Rad,USA).

The primary antibodies used were anti-GKN2,anti-GKN1,anti-PCNA,anti-Survivin, anti-BCL-2,anti-MMP7,anti-MMP9,anti-Timp2,anti-MMP2,Anti-PI3K,anti-p-PI3K,anti-AKT,anti-p-AKT,anti-PTEN,anti-mTOR,anti-p-JAK2,anti-p-STAT3,anti-p-STAT5,anti-ERK,anti-p-erk,anti-MEK,anti-p-mek,anti-GAPDH,anti-β-actin(Cell Signaling Technology, USA 1:1000).The secondary antibodies were anti-rabbit and anti-mouse HRP-conjugated IgG (Cell Signaling Technology, USA 1:3000).

Cell viability assay using the xCELLigence Real Time Cell Analyzer (RTCA)

Optimal seeding concentration for proliferation of AGS was determined. AGS cells (5000 cell in 100 μL medium/well) were seeded in 16 well plates (E-plate 16 ACEA Biosciences Inc, San Diego USA) following the xCELLigence Real Time Cell Analyzer (RTCA) DP instrument manual as provided by the manufacturer. Baseline cell index were calculated for at least two

measurements from three replicate experiments. Cell proliferation was monitored for another 72 h.

Measurement of colony formation

To measure the proliferative ability of a single cell in vitro, Plate colony formation assay was performed. Log phase cells were plated into 6-well plates at a density of 500 cells/well and cultured in 90%F12+10%FBS for 2 weeks to allow colony formation. Colonies were fixed in 1% formaldehyde and stained with 0.5% crystal violet solution. Colony number was calculated by Image J software and imaged.

Cell migration and invasion assay

Invasion assays were performed using transwell chamber with a pore size of 8 μm (Corning, New York, NY, USA). Plates were coated with Matrigel prior to cell seeding. The upper chamber was filled with 5 × 105 AGS cells in F12 containing 0.1 % fetal bovine serum. And, F12 containing 10 % fetal bovine serum was added to the lower chamber. The membrane was photographed, and numbers of invading to lower chamber through the Matrigel were quantified from five randomly selected microscopic fields per membrane. The migration assay was carried using a similar system without adding Matrigel.

RNA preparation for mRNA-Seq

Total RNA was extracted using RNAiso Plus Total RNA extraction reagent (Cat#9109, TAKARA) following the manufacturer's instructions and checked for a RIN number to inspect RNA integrity by an Agilent Bioanalyzer 2100 (Agilent technologies, Santa Clara, CA, US).Qualified total RNA was further purified by RNAClean XP Kit (Cat A63987, Beckman Coulter,Inc.Kraemer Boulevard Brea, CA,USA)and RNase-Free DNase Set (Cat#79254, QIAGEN, GmBH, Germany).

Library construction and sequencing

The rRNA-depleted and RNA-fragmented libraries were constructed with sequential first- and second-strand cDNA synthesis, followed by end repair, adenylation of the 3' ends, ligation of adapters, and enrichment of the cDNA templates, according to experimental instructions. The quality and quantity of each libraries were determined with a Qubit® 2.0 Fluorometer (Life Technologies, USA) and Agilent Bioanalyzer 2100(Agilent Technologies, USA), respectively. The constructed library was sequenced using the Illumina HiSeq™ 2500 System (Illumina, USA) by Shanghai Biotechnology Corporation (Shanghai, China)

Supporting Results

NEB mutations as an up-stream regulator of Epithelial-Mesenchymal Transition (EMT) pathways

Notably, the up-regulated SMGs of *NEB* were highly enriched in the top ranked modules including M28, M226 (a daughter module of M28), M121 (the parent module of M437 and the top module M666) and M475 (**Supporting Material 2B**). M28 was involved in phospholipid metabolism and upregulated in tumor, as previously described. M121 and M475 were associated with biological oxidations (FET p=4.18E-7, 9.69 FE) and apoptotic cleavage of cellular proteins (FET p=2.65E-6, 22.7 FE), respectively.

Particularly, we identified key regulators of epithelial specific splicing in these modules, *ESRP1* in M28 and *ESRP2* (a top driver of M475). *ESRP1* and *ESRP2* orchestrate coordinated changes in epithelial alternative splicing, and are known to be abrogated during the epithelial-mesenchymal transition (EMT)[5]. In TCGA-GCC, *ESRP1* and *ESRP2* were significantly up-regulated in the *NEB* mutants by 1.23-fold (t-test p =1.13E-2,) and 2.15-fold (t-test p =9.95E-5), respectively. The *ESRP1- ESRP2* centered network was validated by the significant enrichment of the signature of the double knock-down of *ESRP1* and *ESRP1* from PNT cells[5] (the details of the analysis can be found in the subsection of *in-silico validation of gene-gene interactions* and *Extraction of gene perturbation signatures* in Materials and Methods). For example, the genes up-regulated by the double knock-down of *ESRP1* and *ESRP2* were enriched in the 5-layer neighborhood of *ESRP1* (FET p =5.54E-6, 1.74 FE) and in the 3-layer neighborhood of *ESRP2* (FET p =2.45E-7, 3.15 FE). The *ESRP1* and *ESRP2* centered networks were also associated with differential isoform expressions. By segregating primary tumor samples into high- and low-*ESRP1/2* expression groups by medians, we performed differential expression analysis on isoforms and then projected these isoform signatures onto the respective genes in the *ESRP1* and *ESRP2* centered networks. The up-regulated isoform signatures were highly enriched in the 5-layer neighborhood of *ESRP1* (FET p =4.01E-39, 4.38 FE) and the 3-layer neighborhood of *ESRP2* (FET p=1.33E-65, 5.20 FE). Such striking overlap strongly validated *ESRP1/2*-driven epithelial splicing pathways in the GC co-expression network.

On the other hand, the SMGs down-regulated by *NEB* are enriched in an EMT associated module M109 (FET p-value=5.39E-126, 4.38 FE; **Supporting Figure 4B**). M109 is associated with key EMT pathways such as focal adhesion (FET p =9.01E-9, 3.85 FE), cytoskeletal protein binding (FET p =9.56E-7, 3.71 FE) and integrin (FET p =2.55E-5, 6.45 FE). *ZEB1*, a hub node in M109 (**Supporting Figure 4B**), is a transcription factor that promotes tumor invasion and metastasis by inducing epithelial-mesenchymal transition (EMT) in carcinoma cells[6].

Overall, these results strongly suggest that *NEB* somatic mutations play protective roles by targeting key dys-regulated pathways contributing to GC invasiveness such as phospholipid metabolism, biological oxidations, apoptosis and EMT.

GKN2 over-expression inhibits proliferation and invasion of gastric cancer cells

The effect of *GKN2* over-expression on gastric cancer cell viability was investigated by RTCA and colony formation assays (see *Cell viability assay using the xCELLigence Real Time Cell Analyzer* in **Supporting Materials and Methods**). As depicted in **Supporting Figure 8A,C**, the

viability of AGS_GKN2 was reduced and was significantly lower than AGS_CON ($P < 0.05$) by RTCA assays. Between 24 and 96 h after the transfection, AGS_GKN2 did not grow at all, while AGS_CON cells were proliferative (**Supporting Figure 8B,D**). Thus, the colony formation ability of AGS_GKN2 cells was dramatically increased compared with that of AGS_CON ($P<0.05$). These results indicated that GKN2 had an anti-oncogenic role in the GC cells. To further determine impact of GKN2 on cell growth, a series of genes related to proliferation were analyzed. Western blot analyses of AGS_GKN2 showed that PCNA, Survivin and Bcl-2, proliferation marker genes, were significantly down-regulated and it suggests GKN2 controls cell growth through regulating activation of these genes (**Supporting Figure 8E**).

Changes in cell migration and invasion after GKN2 transfection were measured using transwell assays (see *Cell migration and invasion assay* in **Supporting Materials and Methods**). Both migration and invasion assays indicated that overexpression of GKN2 significantly reduced the number of AGS cells in the lower chamber (**Supporting Figure 9A-D**). In view of the important role of the matrix metalloproteinase (MMP) family in the invasion and metastasis of cancer, we detected the protein levels of MMP2/7/9 and Timp2 in gastric cancer cell. Compared with the control, both the expression level of MMP2, MMP7 and MMP9 were downregulated significantly in GKN2-transfected cancer cells and Timp2 were dramatically upregulated inversely (**Supporting Figure 9E**).

GKN2 suppresses the oncogenic PTEN/PI3K/AKT/mTOR and JAK/STAT pathways

We identified that over-expression of GKN2 significantly downregulated the protein expressions of p-PI3K, AKT, p-AKT and mTOR, and upregulated negative regulatory factor PTEN in GKN2-transfected AGS by western blot analyses (**Supporting Figure 10A**). Furthermore, we observed common inhibition of p-JAK2, and cell line specific inhibition of p-STAT3 and p-STAT5. Specifically, p-STAT3 inhibition was observed only in AGS cells (**Supporting Figure 10B**).

Global epigenetic alterations functionally impact key pathways in gastric cancer progression

We systematically evaluated epigenetic impact on gastric cancer transcriptome by analyzing correlations between gene expression and DNA methylation changes at CpG sites in *cis* and *trans* (**Supporting Materials and Methods**). This analysis yielded a large number of cis-/trans-methylation genes (cis-/trans-eMSGs) including 4,004 negative cis-eMSGs, 1,529 positive cis-eMSGs, 8,266 negative trans-eMSGs, and 7,352 positive trans-eMSGs. These eMSG signatures were tested for enrichment in the MEGENA derived modules so as to identify epigenetic regulation of these gene modules (**Supporting Material 6A** for gene signature lists, **Supporting Material 6C** for module enrichments).

The enriched MSigDB signatures in the eMSGs suggested that methylations indeed impacted diverse known pathways and functions driving tumorigenesis in gastric cancer. Negative cis-eMSGs were strongly associated with signal transduction (FET p=2.09E-43, 1.86 FE), hallmark allograft rejection (FET p= 1.48E-34, 3.48 FE) and anatomical structure development (FET

p=9.65E-24, 1.80 FE). Positive cis-meSCGs were enriched for similar pathways including membrane (FET p= 6.04E-27, 2.06 FE), anatomical structure development (FET p=2.5E-24, 2.48 FE) and signal transduction (FET p=1.20E-13, 1.79 FE). Negatively correlated trans-meSCGs was significantly enriched for cell cycle associated functions such as hallmark E2F targets (FET p= 8.53E-36, 2.38 FE) and hallmark G2M checkpoint (FET p= 7.64E-21, 2.03 FE), epithelial-mesenchymal transition associated pathways such as hallmark epithelial-mesenchymal transition (FET p= 6.78E-28, 2.21 FE), and immune response pathways such as immune system process (FET p= 1.92E-25, 1.90 FE). Lastly, positive trans-meSCGs were also enriched for hallmark E2F targets (FET p=1.44E-35, 2.53 FE), hallmark epithelial-mesenchymal transition (FET p= 2.00E-17, 2.03 FE) and immune system process (FET p= 8.66E-25, 1.98 FE) (see **Supporting Material 6B** for details).

Supporting Tables

**Survival**			
overall followup (days)	*overall survival*	*recurrence-free followup*(days)	*recurrence*
Min. : 0.0	alive: 238	Min. : 0.00	no event: 170
1st Qu.: 6.0	death: 57	1st Qu.: 16.75	recurred: 31
Median : 242.0		Median : 347.50	
Mean : 293.8		Mean : 310.66	
3rd Qu.: 445.0		3rd Qu.: 462.25	
Max. :3196.0		Max. :2405.00	
**Histological Subtype**			
Lauren Classification	*Signet Ring*	*WHO Classification*	*Neoplasm histologic grade*
Diffuse : 69	NO : 47	Mixed : 19	G1 : 4
Intestinal:196	YES : 22	Mucinous : 18	G2 : 72
Mixed : 19		Papillary : 22	G3 :146
		Poorly_Cohesive: 69	
		Tubular :140	
**Pathologic Classification**			
Pathologic T	*Pathologic N*	*Pathologic M*	*TNM Stage*
T3 :155	N0 :97	M0 :273	Stage_IIB :60
T4a : 60	N1 :64	M1 : 20	Stage_IIIB:57
T2 : 44	N2 :58		Stage_IIA :56
T4b : 14	N3 :65		Stage_IIIA:40
T1b : 10			Stage_IB :24
(Other): 2			Stage_IV :20
			(Other) :38
**Other classifications**			
Anatomic Region	*TCGA: Molecular Subtype*	*TCGA: MSI status*	
ANTRUM :114	CIN:147	MSI-H: 64	
FUNDUS_BODY:116	EBV: 26	MSI-L: 44	
GEJ_CARDIA : 57	GS : 58	MSS :187	
	MSI: 64		

Supporting Table 1. Summary table of clinical features used in the analysis from TCGA gastric cancer data.

Pathological Features	Mean (log10RQ)	Range	P value
Sex			
<60	0.3218	-2.990~3.228	0.434
>60	0.571	-2.712~3.116	
Lauren's type			
Intestinal	0.525	-2.296~3.116	0.665
Diffuse	0.3901	-2.990~3.228	
Tumor size			
<5cm	0.746	-2.990~3.228	**0.023**
>5cm	0.026	-2.712~3.080	
Location			
EGJ(oesophagogastric junction)	-0.3206	-2.520~2.361	**0.006**
Non-EGJ	0.6733	-2.990~3.227	
Depth of invasion			
<T2	0.947	-1.862~3.228	0.201
>T2	0.354	2.990~3.080	
Differentiation			
moderate	0.556	-2.295~3.116	0.594
poor	0.376	-2.990~3.228	
TNM			
<III stage	0.854	-1.862~3.228	**0.076**
>III stage	0.248	-2.990~3.080	
Lymph node metastasis			
absent	0.63	-2.296~3.228	0.479
present	0.37	2.990~3.116	

[a] RQ=$2^{-\Delta\Delta Ct}$

Supporting Table 2. Correlation between tumor GKN2 mRNA expression and pathological features of gastric carcinoma of Fujian cohort.

Supporting Figures

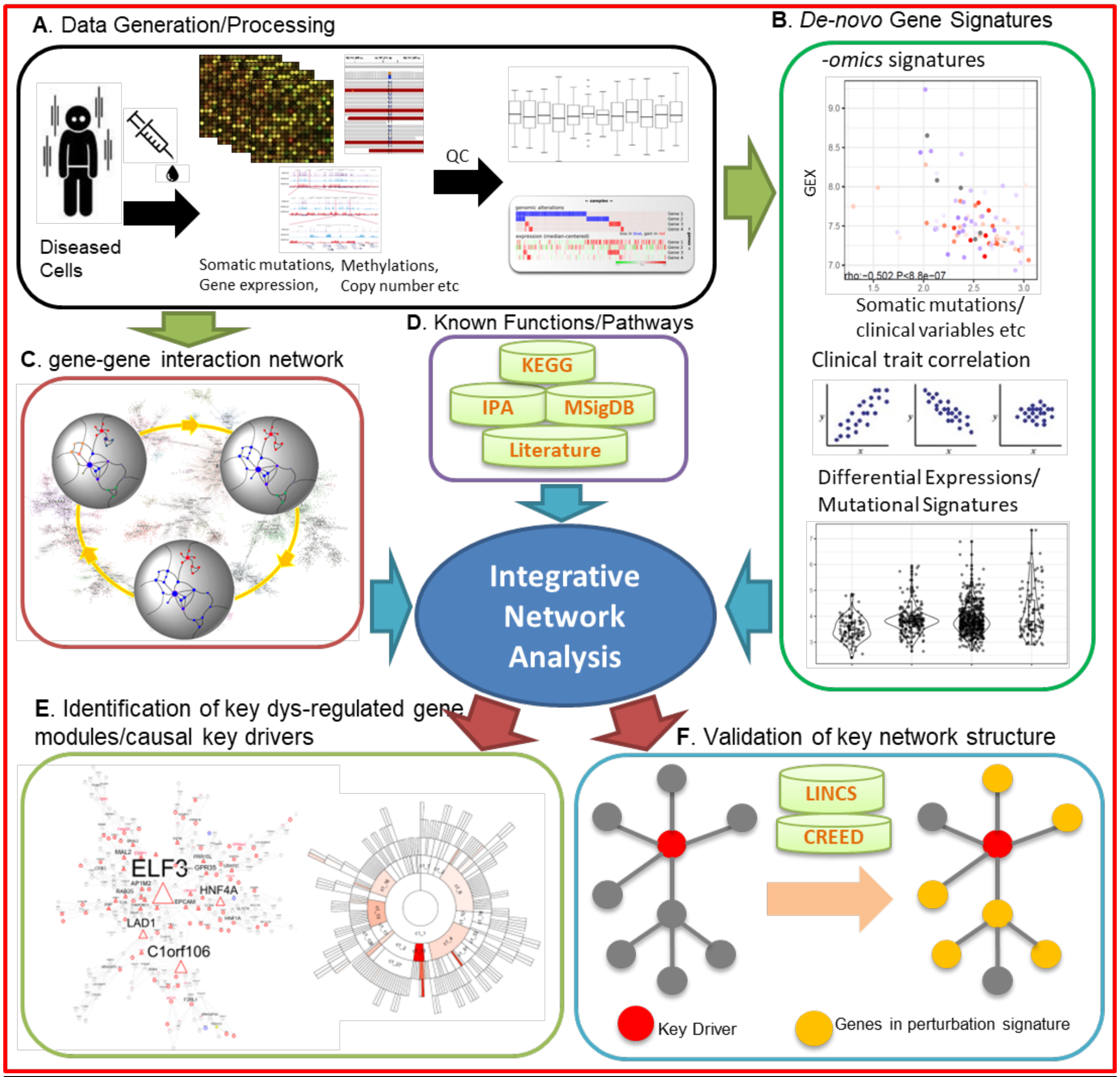

Supporting Figure 1. Overview of integrative network analysis of TCGA gastric cancer cohort. **A**. Data Generation/Processing: Illustration of multiple –omics data generations to capture molecular features of diseased cells. Quality Controls (QC) are performed to remove outliers and confounding effects. **B.** Gene Signatures: various gene signatures are extracted by associating gene expressions to clinical traits and somatic mutation status. **C.** Co-expression: MEGENA is applied to identify co-expression network from the primary tumor gene expression data. **D.** Known pathways: established gene signatures from public domain (for instance, MSigDB) are projected to co-expression modules to associate to enriched pathways. **E.** Identification of key dys-regulated gene modules: Enrichments of gene signatures are gathered to pin-point key dys-regulated gene modules. **F.** Validation of key network structure: Key hub genes from key gene modules are functionally validated by projecting gene perturbations signatures onto the neighborhood network structure.

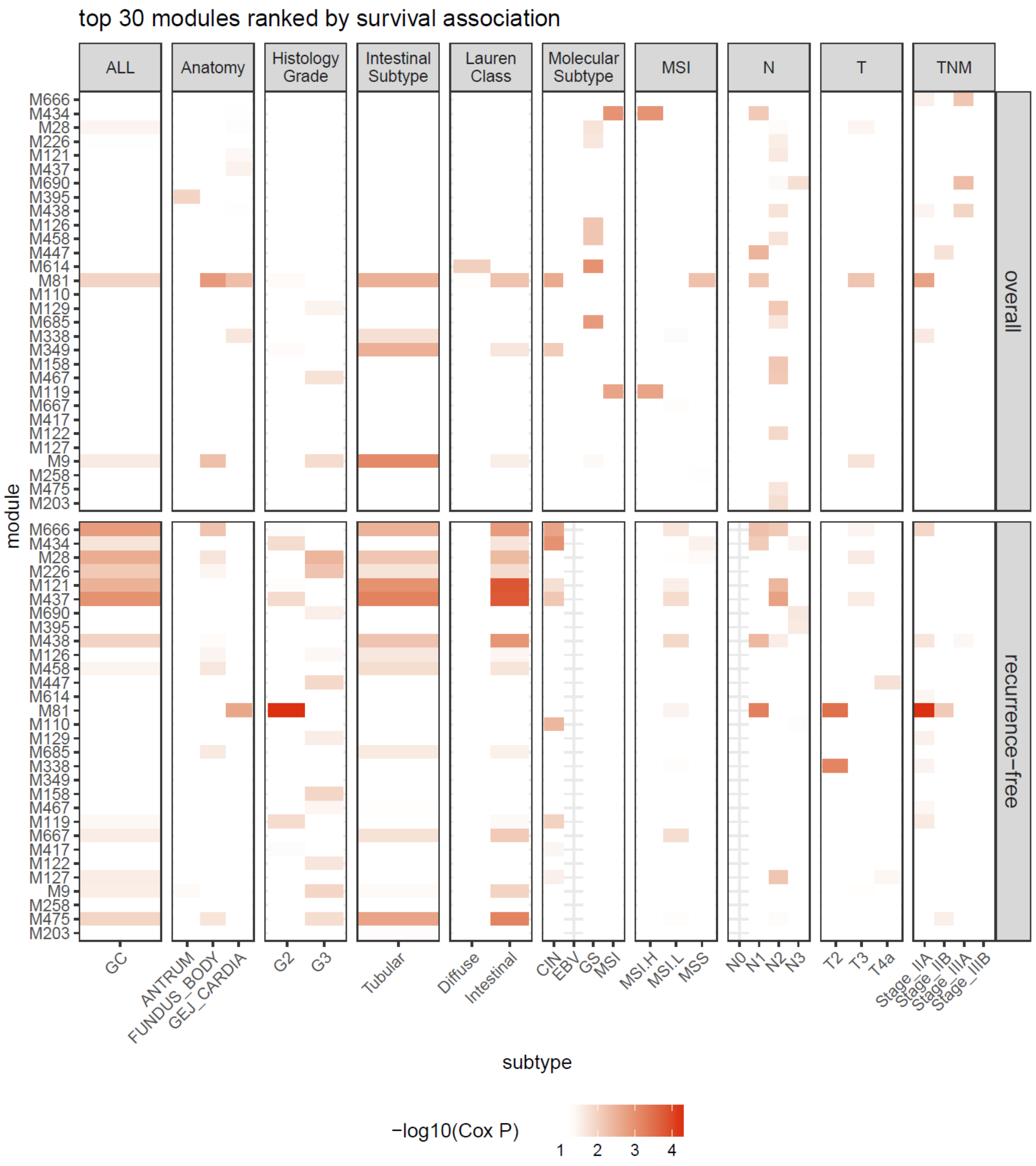

Supporting Figure 2. Heatmap of Cox p-values for overall (top) and recurrence-free (bottom) survival within various subtypes of gastric cancer for top 30 ranked modules.

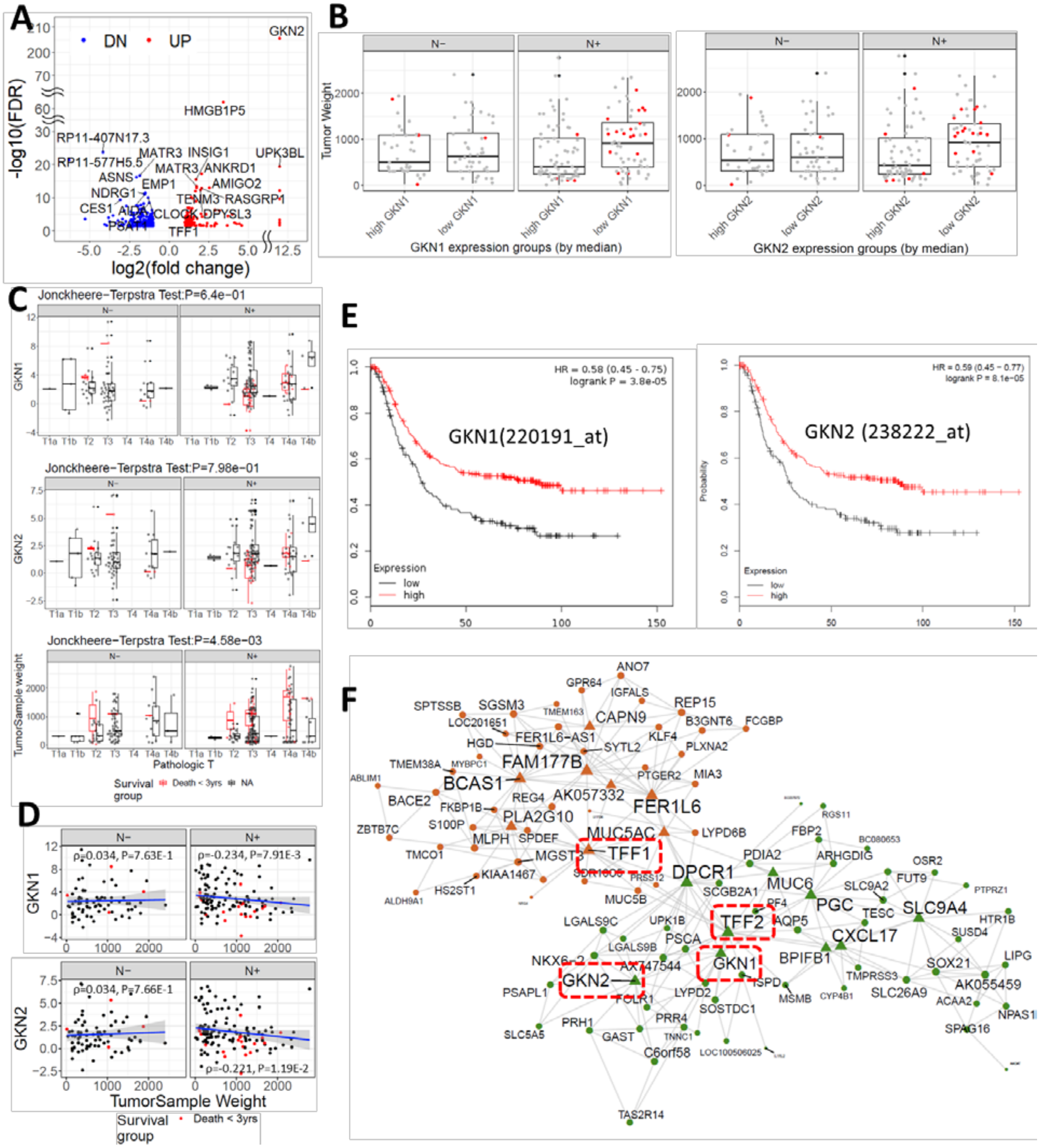

Supporting Figure 3. Prognostic significance of *GKN1/2* in *TFF1-TFF2-GKN1-GKN2* axis. **A.** Significant differential expressions by fold change > 1.2 or < 0.83 and FDR < 0.05 are highlighted. Top 10 most up-/down-regulated genes by GKN2 over-expression in AGS cells (colored red/blue) are labeled. **B.** *GKN1/2* expression associates to tumor weights measured at resection (y-axis, in grams) within node-positive group. High-/low-expression groups were defined by the median expressions, and patients whose deaths were observed within 3-years of follow-up were marked by red dots as the poor prognosis group. Node-positive/-negative status is specified on the top of each subplot. **C**. Association between pathological tumor (T) stages and

GKN1/*GKN2*/tumor weight. The significance p-values were evaluated by non-parametric Jonckheere-Terpstra test. Patients with death within 3 years of follow-up are marked red. D. Correlation between *GKN1*/*GKN2* expressions and tumor sample weights. Significance p-values by Spearman's correlations are shown. **E**. Validation of prognostic significance of *GKN1/2* from independent microarray data sets from GEO[7]. **F**. Module M436 is directly connected to M434, and together constitutes *TFF1*-*TFF2*-*GKN1*-*GKN2* axis (highlighted in red boxes).

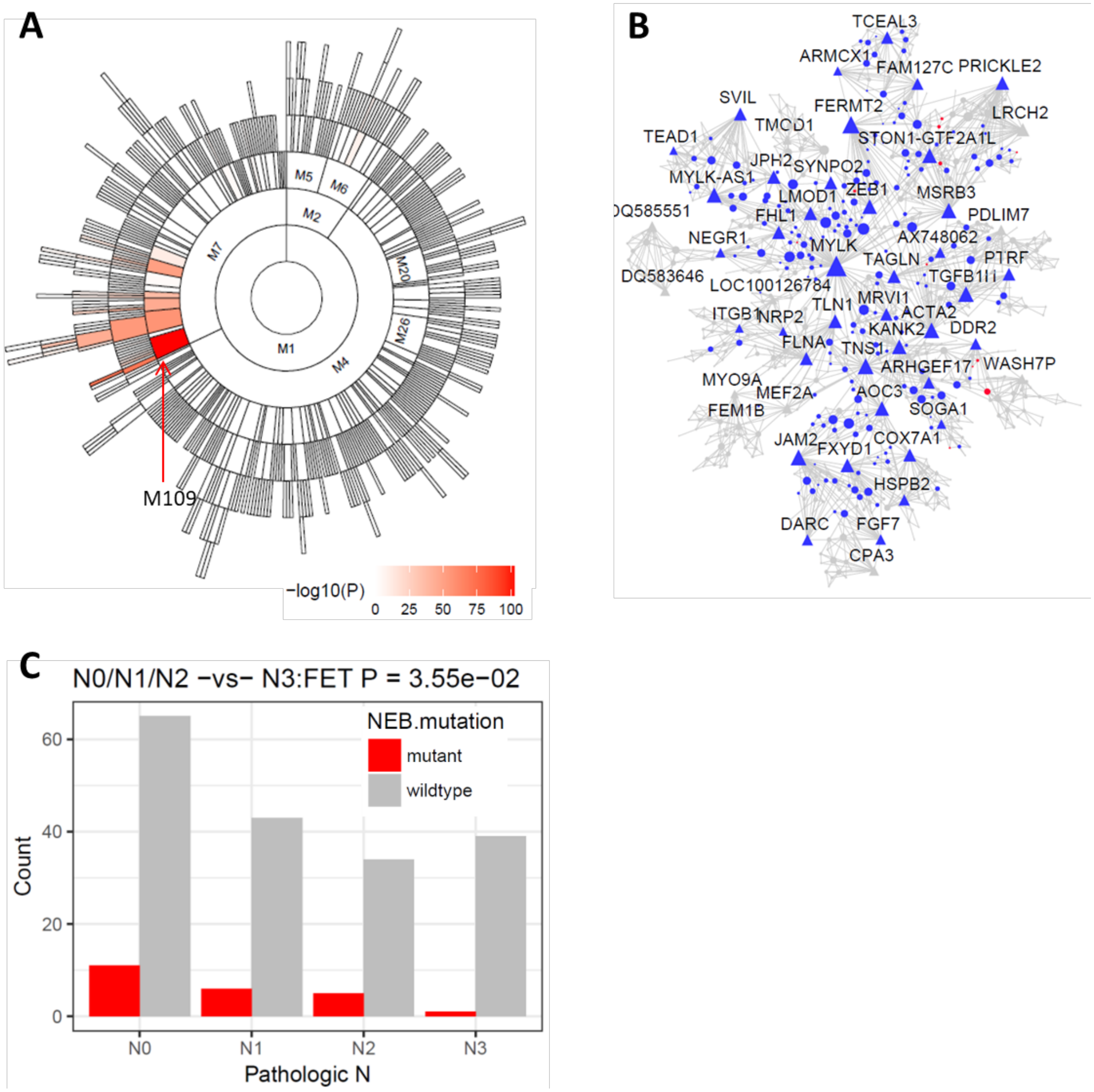

Supporting Figure 4. Suppression of Epithelial-Mesenchymal Transition (EMT) in *NEB* mutant samples. A. Sunburst plot illustrates enrichment of down-regulated SMGs by *NEB* mutation in hierarchically organized MEGENA modules. Red heatmap colors are proportional to -log10(FET p-value) of the SMGs, as illustrated by the colorbar on the bottom. **B.** Visualization of the top enriched gene module, M109, by down-regulated SMGs by NEB mutation. Significant hub genes identified by MEGENA are labeled with respective gene symbols. **C**. Distribution of pathological node status in TCGA-GCC across *NEB* mutant and wildtype samples. The statistical significance of under-representation of N3 against the rest in *NEB* mutants is shown on the top.

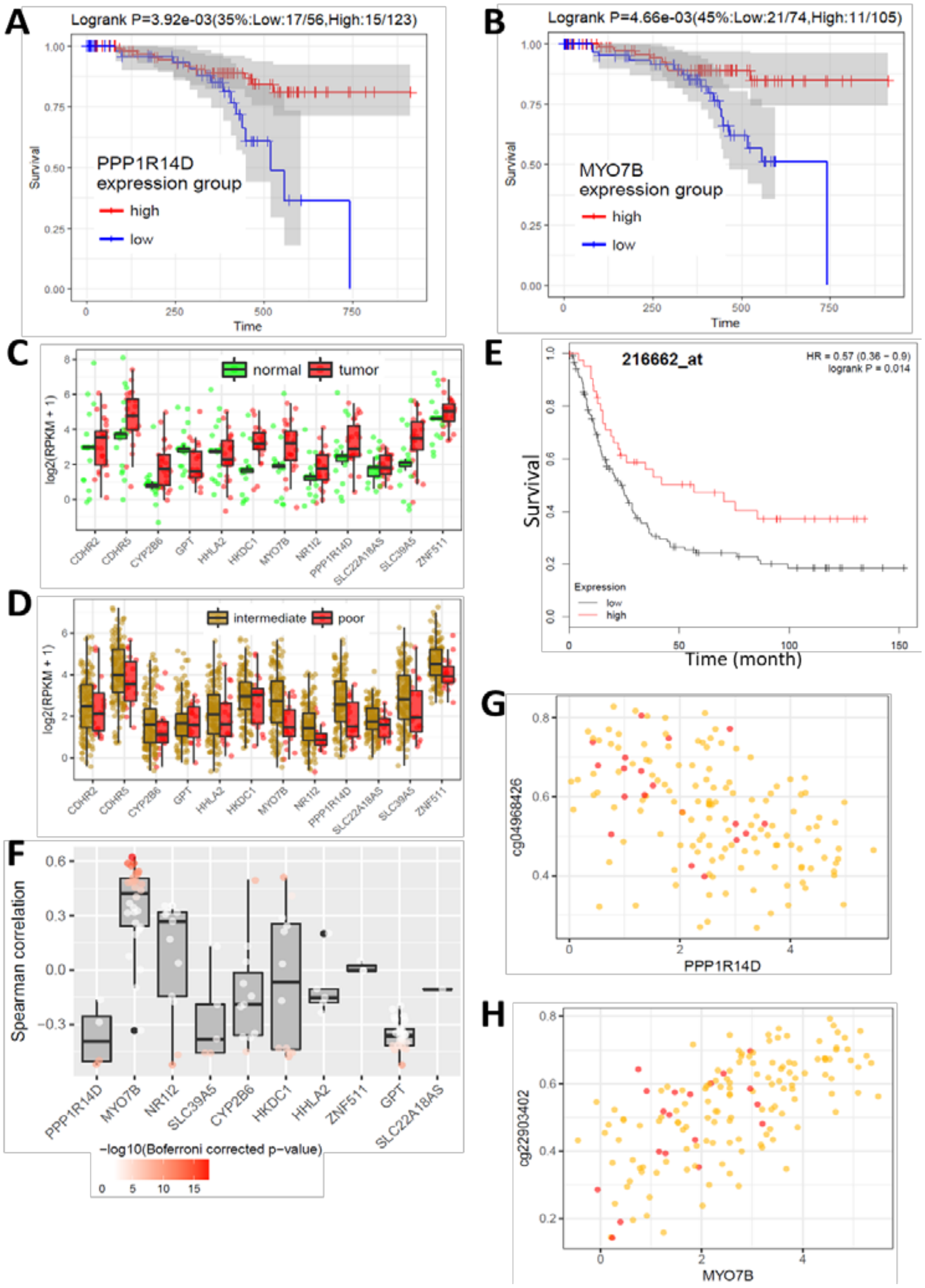

Supporting Figure 5. Epigenetic regulations of the top ranked module M666 associates with poor prognosis. A/B. Kaplan-Meier plots for overall survival of TCGA-GCC patients when segregated by *PPP1R14D/MYO7B* expressions in primary tumors. Quantile thresholds to define high and low expression groups, and respective logrank p-values and number of deceased/living patients in high and low expression groups (formatted as low: #. deceased/#. living, high: #. deceased/#. living) are shown at the top. **C.** Comparisons of gene expressions between adjacent normal and primary tumor samples of TCGA-GCC for key regualtors of M666. **D**. Comparisons

of gene expressions between patients with poor recurrence-free outcomes (i.e. recurrence within 3 years of follow up) and those with intermediate outcomes (i.e. recurrence-free survival observed between 3 and 5 years of follow up) tumor samples of TCGA-GCC for key driver genes of M666. **E**. Kaplan-Meier plot of node positive gastric cancer patients segregated by *MYO7B* expression from *km-plotter*. **F**. Correlations between gene expression and methylation at CpG sites of the promoter regions of the respective genes. Each point represents the Spearman correlation coefficient between the gene and a CpG site. Color intensity represents the Bonferroni corrected p-value of correlation. The boxplots summarize the correlation coefficient distributions. **G**. Scatter plot of *PPP1R14D* gene expression against methylation at the CpG site, cg04968426. Red and golden colors represent poor and intermediate recurrence-free outcome groups, respectively. Significance of survival difference between the group with high methylation and low expression and the group with low methylation and high expression is shown at the top with. **H**. Scatter plot of *MYO7B* gene expression against the methylation at the CpG site, cg22903402. . Red and golden colors represent poor and intermediate recurrence-free outcome groups, respectively. Similar to G, the significance of survival difference is shown at the top.

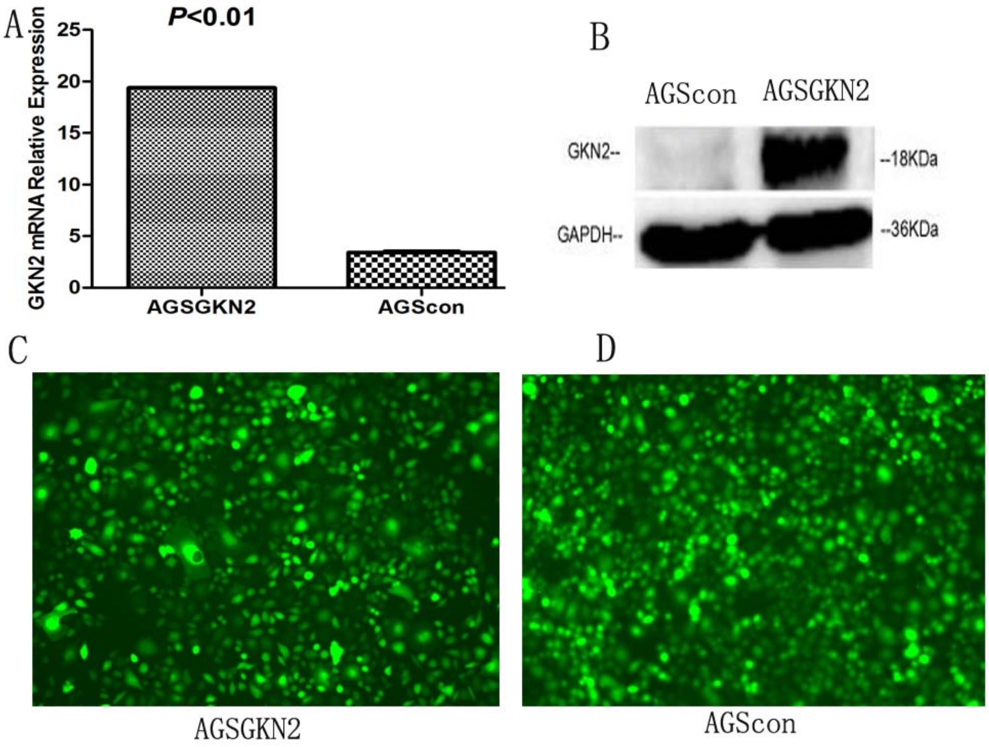

Supporting Figure 6. Expression of *GKN2* in AGS after transfection with GKN2-LV and ctrl-LV at protein and mRNA levels. **A**: mRNA expression of *GKN2* in AGS_CON and AGS_GKN2; **B**: Western blot of *GKN2* in AGS and AGS_GKN2; **C**: AGS_GKN2 cells; **D**:AGS_CON cells ; **denotes $P < 0.05$

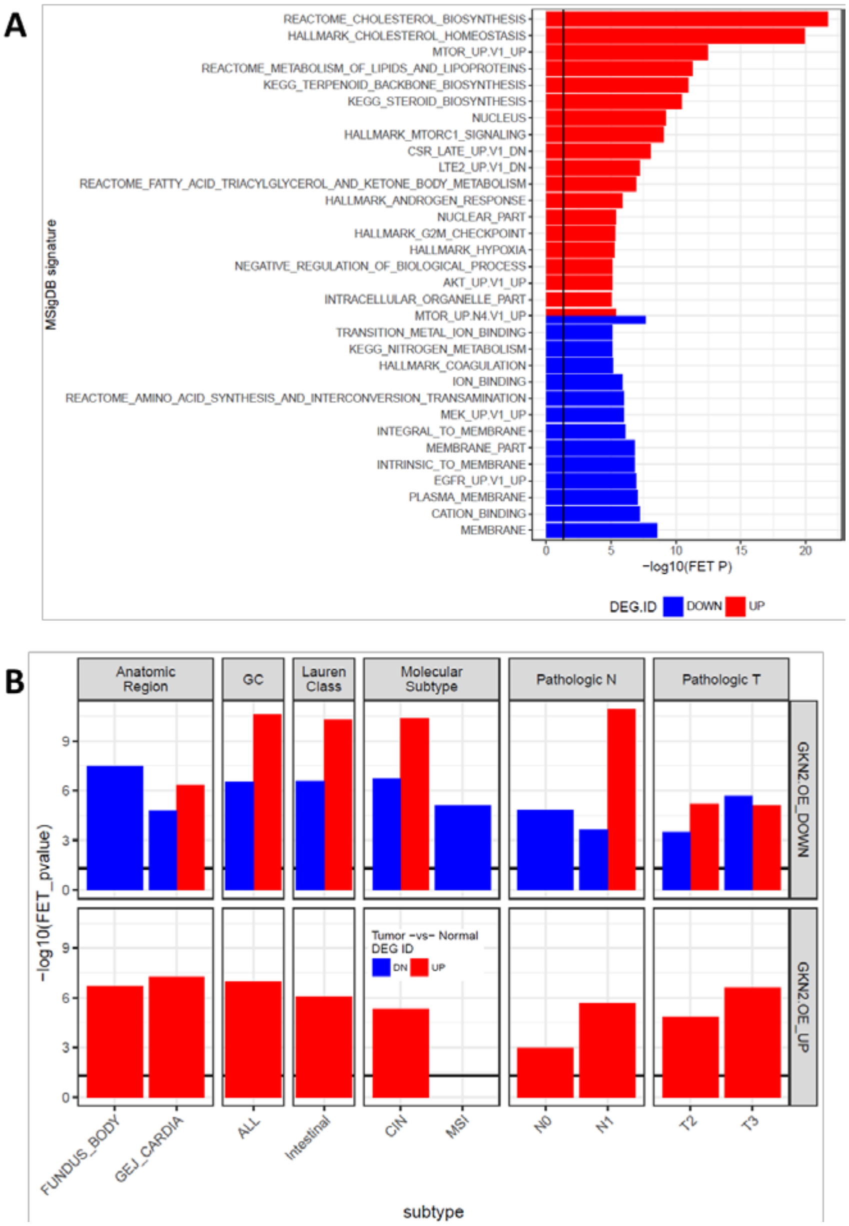

Supporting Figure 7. Enrichments of *GKN2* over expression signatures from AGS cells. A. Enrichments of MSigDB signatures in differentially expressed genes by *GKN2* over-expression in AGS gastric cancer cells. The signatures were identified by Bonferroni corrected FET p-value < 0.05. Signatures enriched in up-regulated genes by *GKN2* over-expression (denoted as UP, colored red) are shown at the top, and those enriched in the down-regulated genes (denoted as DOWN, colored blue) are shown at the bottom. **B.** Enrichments of primary tumor -vs- adjacent normal tissue DEG signatures from various GC subtypes, with differentially expressed genes by GKN2 over-expression in AGS gastric cancer cells. Up-/down-regulated signatures by *GKN2* over expressions are specified on the right strips in the subtype against –log10(FET p-value) bar plots, and categories of the subtypes are specified on the top strips.

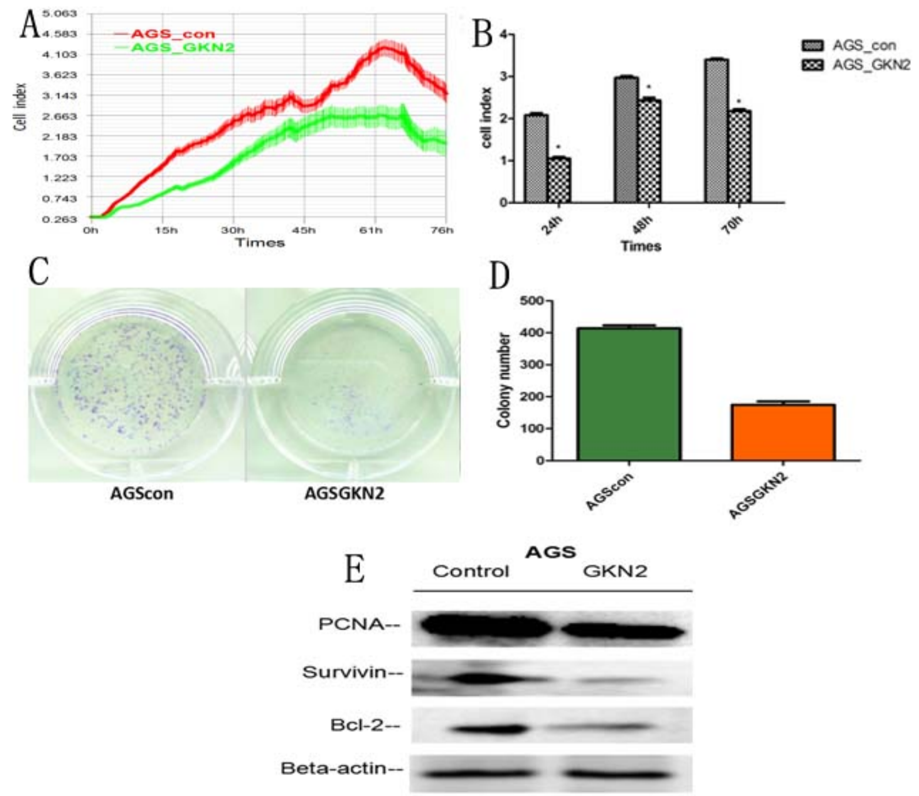

Supporting Figure 8. Overexpression of *GKN2* inhibits proliferation of AGSs. **A**: Cell viability was detected by xCELLigence real time cell proliferation system (RTCA) for 70h. **B**: *GKN2* significantly the proliferation of AGS than compared with that of AGS_GKN2. **C**: *GKN2* overexpression group displayed a significant decrease in term of colony formation numbers. Thus, the colony formation ability of AGS_GKN2 was significantly weaker than compared with that of control cells(D). *: $P<0.05$. **E.** Effects of *GKN2* on the expression of proliferation -related genes PCNA, Survivin and Bcl-2 were examined by western blotting. Beta-actin acted as the loading control.

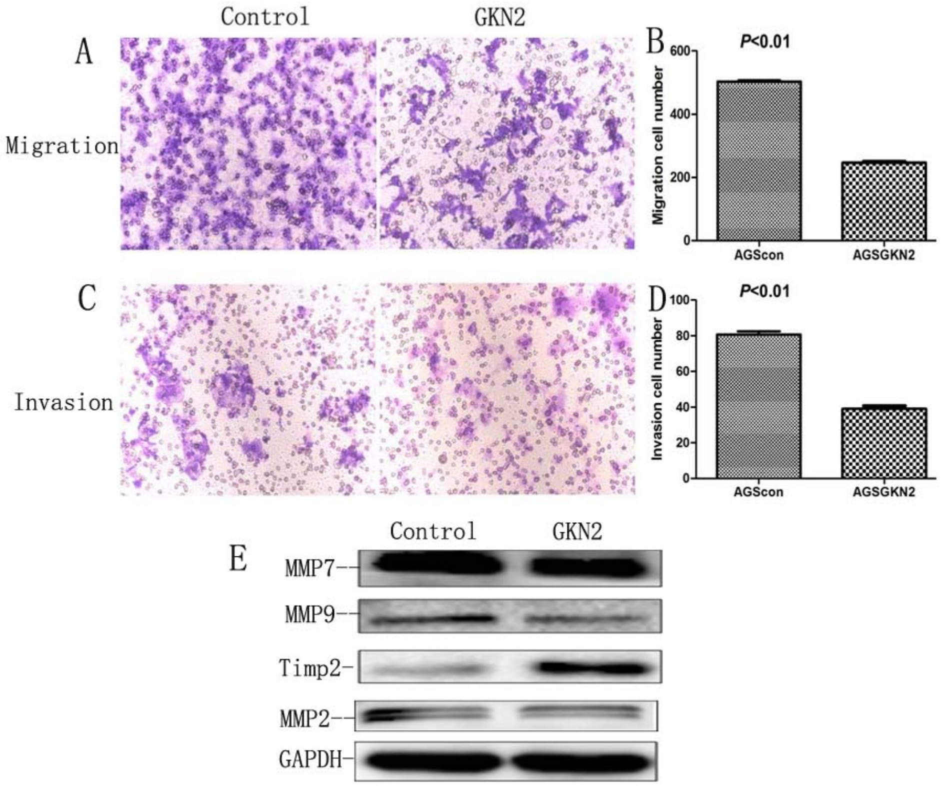

Supporting Figure 9. Effects of *GKN2* on migration/invasion in AGS. A, B: *GKN2* reduced cell migration compared with the control. **C, D**: *GKN2* reduced cell invasion compared with the control. **E**: Effects of *GKN2* on the expression of *MMP2/7/9* and *Timp2* in AGS. *MMP2/7/9* and *Timp2* were examined by western blotting. *GAPDH* acted as the loading control.

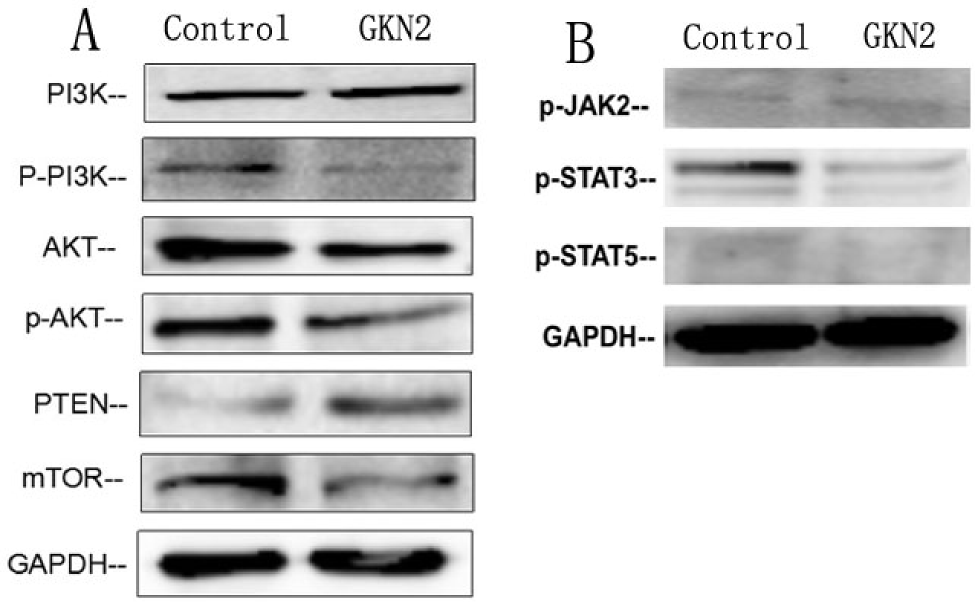

Supporting Figure 10. Western blot analyses on *GKN2*-transfected AGS cell lines reveal impacts on oncogenic pathways of gastric cancer. GAPDH acted as the loading control. **A**. Effects of *GKN2* on protein markers of the *PTEN/PI3K/AKT/mTOR* pathway in AGS . Protein indicators related to the *PTEN/PI3K/AKT/mTOR* pathway, *PI3K, p- PI3K, AKT, p-AKT, mTOR* and *PTEN* were examined by western blotting. . **B**. Effects of *GKN2* on protein markers of the *JAK/STAT* pathway in AGS. Protein indicators related to the *JAK/STAT* pathway, *p-JAK2, p-STAT3* and *p- STAT5* were examined by western blotting. *GAPDH* acted as the loading control.

References

1 Song, W. M. & Zhang, B. Multiscale Embedded Gene Co-expression Network Analysis. *PLoS Comput Biol* **11**, e1004574, doi:10.1371/journal.pcbi.1004574 (2015).

2 Mayakonda, A., Koeffler, P. H. Maftools: Efficient analysis, visualization and summarization of MAF files from large-scale cohort based cancer studies. *BioRxiv*, doi:http://dx.doi.org/10.1101/052662 (2016).

3 Cancer Genome Atlas Research, N. Comprehensive molecular characterization of gastric adenocarcinoma. *Nature* **513**, 202-209, doi:10.1038/nature13480 (2014).

4 McCarthy, D. J., Chen, Y. & Smyth, G. K. Differential expression analysis of multifactor RNA-Seq experiments with respect to biological variation. *Nucleic acids research* **40**, 4288-4297, doi:10.1093/nar/gks042 (2012).

5 Warzecha, C. C., Shen, S., Xing, Y. & Carstens, R. P. The epithelial splicing factors ESRP1 and ESRP2 positively and negatively regulate diverse types of alternative splicing events. *RNA biology* **6**, 546-562 (2009).

6 Zhang, P., Sun, Y. & Ma, L. ZEB1: at the crossroads of epithelial-mesenchymal transition, metastasis and therapy resistance. *Cell cycle* **14**, 481-487, doi:10.1080/15384101.2015.1006048 (2015).
7 Szasz, A. M. *et al.* Cross-validation of survival associated biomarkers in gastric cancer using transcriptomic data of 1,065 patients. *Oncotarget* **7**, 49322-49333, doi:10.18632/oncotarget.10337 (2016).

Supporting Material 2A: Module Summary Table

	module	module.parent	module.size	top10_keydrivers	top10_hubs(connectivity)	GO_KEGG	Subtype.Signature	Tumor_vs_Normal.Signature
1	M666	M438	52	PPP1R14D,NR1I2,CDHR2,MYO7B,SLC22A18AS,CDHR5,HHLA2,SLC39A5,GPT,ZNF511	PPP1R14D(24)	KEGG_ARACHIDONIC_ACID_METABOLISM	DEG−subtype.Molecular.Subtype__CIN_vs_EBV__UP	DEG−TumorNormal.Molecular.Subtype__CIN__UP
2	M434	M119	56	TFF2,PGC,DPCR1,CXCL17,GKN1,BPIFB1,MUC6,GKN2,SLC9A4	()	DIGESTION	DEG−subtype.Molecular.Subtype__CIN_vs_EBV__UP	DEG−TumorNormal.neoplasm_histologic_grade__G3__DN
3	M28	M4	244	ELF3,EPCAM,LAD1,HNF4A,AP1M2,GPR35,C1orf106,PRR15L,CAMSAP3,MAL2	ELF3(44),C1orf106(32),HNF4A(27),LAD1(27),GPR35(20),MAL2(19),AP1M2(19),EPCAM(18),RAB25(17)	KEGG_TIGHT_JUNCTION	DEG−subtype.Lauren.Class__Diffuse_vs_Mixed__DN	DEG−TumorNormal.neoplasm_histologic_grade__G2__UP
4	M226	M28	223	ELF3,EPCAM,LAD1,AP1M2,GPR35,HNF4A,PRR15L,C1orf106,MAL2,RAB25	ELF3(44),C1orf106(32),HNF4A(27),LAD1(27),GPR35(20),MAL2(19),AP1M2(19),EPCAM(18),RAB25(17)	KEGG_TIGHT_JUNCTION	DEG−subtype.Lauren.Class__Diffuse_vs_Mixed__DN	DEG−TumorNormal.neoplasm_histologic_grade__G2__UP
5	M121	M7	194	MYO7B,CDH17,MOGAT3,NR1I2,PPP1R14D,TMEM150B,CDX1,CYP2B6,GUCY2C,PHGR1	MOGAT3(37),CDH17(30),PPP1R14D(28),CDX1(27),MYO7B(23),NR1I2(22),TMEM150B(21),PHGR1(20)	MONOOXYGENASE_ACTIVITY	DEG−subtype.Molecular.Subtype__EBV_vs_MSI__DN	DEG−TumorNormal.neoplasm_histologic_grade__G2__UP
6	M437	M121	76	CDH17,CDX1,GUCY2C,PHGR1,GPA33,FAM84A,NOS2,CLDN3,AF086125,CDX2	CDX1(26),CDH17(23),PHGR1(19)	REPRODUCTION	DEG−subtype.Molecular.Subtype__EBV_vs_MSI__DN	DEG−TumorNormal.neoplasm_histologic_grade__G2__UP
7	M690	M467	85	CHRNA9,GPR139,MIR369,MIR889,AX746877,LINC00277,SPINK6,ALX4,CACNG7,HPD	GPR139(59),CHRNA9(44)	HEMATOPOIETIN_INTERFERON_CLASSD200_DOMAIN_CYTOKINE_RECEPTOR_BINDING	NA	NA
8	M395	M110	127	DQ585295,DQ582474,DGKK,DQ594374,MYL7,LCE2C,LOC643648,PLA2G2E,CCL16,OC90	DQ585295(56),DQ582474(44),DGKK(19),MYL7(16)	MYOSIN_COMPLEX	NA	NA
9	M438	M121	112	MOGAT3,PPP1R14D,TMEM150B,NR1I2,MYO7B,LINC00483,CYP2B6,MUC17,SLC22A18AS,ANKS4B	MOGAT3(36),PPP1R14D(28),TMEM150B(21),NR1I2(21)	KEGG_ARACHIDONIC_ACID_METABOLISM	DEG−subtype.Molecular.Subtype__CIN_vs_EBV__UP	DEG−TumorNormal.neoplasm_histologic_grade__G2__UP
10	M126	M7	246	PTPRH,FA2H,VILL,KCNK1,TJP3,CAPN8,BC034929,B3GNT3,RAB27B,CORO2A	KCNK1(41),PTPRH(33),TJP3(30),CAPN8(23),RAB27B(21),FA2H(20),UNC5CL(19),VILL(18)	KEGG_GLYCEROPHOSPHOLIPID_METABOLISM	DEG−subtype.Lauren.Class__Diffuse_vs_Mixed__DN	DEG−TumorNormal.Molecular.Subtype__CIN__UP
11	M458	M126	101	PTPRH,CORO2A,TJP3,B3GNT3,BCL2L15,TMC5,ANXA4,KCNK5,PLS1,PRR15	TJP3(27),PTPRH(26)	KEGG_NITROGEN_METABOLISM	DEG−subtype.Lauren.Class__Diffuse_vs_Mixed__DN	DEG−TumorNormal.Molecular.Subtype__CIN__UP
12	M447	M123	63	EXOC5,NAA30,EIF2S1,KIAA0586,CNIH,TRMT5,PPM1A,KIAA1737,PPP2R5E,EXD2	EXOC5(24)	PROTEIN_FOLDING	NA	NA
13	M614	M338	64	Y_RNA,5S_rRNA,RN7SK,BC062350,TRNA_Pseudo,Mir_324,OCLM,Mir_633,TRNA,TRNA_Gln	Y_RNA(48),5S_rRNA(22)	NA	NA	NA
14	M81	M4	54	DHX15,NFXL1,PDS5A,N4BP2,ANKRD17,NOP14,RLF,ZMYM1,GUF1,LRBA	DHX15(23),PDS5A(21)	KEGG_PENTOSE_PHOSPHATE_PATHWAY	NA	NA
15	M110	M7	195	DQ585295,DQ582474,CCL16,PLA2G2E,DQ594374,DGKK,MIMT1,RELN,MYL7,LCE2C	DQ585295(56),DQ582474(44),PEG3−AS1(20),DGKK(19),F2(17),MYL7(16)	HORMONE_BINDING	NA	NA
16	M129	M7	105	CHRNA9,GPR139,MIR369,MIR889,AX746877,SPINK6,CACNG7,ZNF474,LINC00277,ALX4	GPR139(59),CHRNA9(44)	AROMATIC_COMPOUND_METABOLIC_PROCESS	NA	NA
17	M685	M458	54	CORO2A,TJP3,PRSS8,BCL2L15,PLEKHG6,TMC5,IL22RA1,CDC42EP5,PDZK1IP1,TLCD2	TJP3(25)	KEGG_NITROGEN_METABOLISM	DEG−subtype.WHO.Class__Papillary_vs_Poorly_Cohesive__UP	DEG−TumorNormal.neoplasm_histologic_grade__G2__UP
18	M338	M86	155	U6,Y_RNA,SPDYE1,LOC100505716,AX746867,Metazoa_SRP,5S_rRNA,MRS2P2,TRNA_Pseudo,RN7SK	Y_RNA(58),U6(56),5S_rRNA(23),SPDYE1(20),LOC100505716(20),RN7SK(16)	NA	NA	NA
19	M349	M93	55	AHSA2,PILRB,RPL32P3,INE1,LOC100272228,MIR3916,MIR631,GIGYF1,AK128346,LOC100289230	AHSA2(31),PILRB(20)	SINGLE_STRANDED_RNA_BINDING	NA	NA
20	M158	M11	120	YJEFN3,WDR83,NDUFA11,NDUFB7,UBA52,GADD45GIP1,C19orf53,C19orf70,C19orf60,DHPS	WDR83(26),NDUFA11(20),NDUFB7(19),YJEFN3(18),C19orf53(18),GADD45GIP1(16)	KEGG_RIBOSOME	NA	NA
21	M467	M129	96	CHRNA9,GPR139,MIR369,MIR889,AX746877,SPINK6,LINC00277,ALX4,CACNG7,HPD	GPR139(59),CHRNA9(44)	AROMATIC_COMPOUND_METABOLIC_PROCESS	NA	NA
22	M119	M7	181	TFF1,VSIG1,CA2,DPCR1,TFF2,CTSE,ANXA10,MUC5AC,AKR1B10,FER1L6	TFF1(29),CA2(24),CYSTM1(24),TFF2(21),CTSE(21),DPCR1(18),BCAS1(18),FER1L6(17),AKR1B10(17),FAM177B(17)	DIGESTION	DEG−subtype.Molecular.Subtype__CIN_vs_EBV__UP	DEG−TumorNormal.Anatomic.Region__FUNDUS_BODY__DN
23	M667	M438	60	MOGAT3,TMEM150B,ANKS4B,MYO1A,ACOT11,DDC,MUC17,C14orf176,LINC00483,SLAIN1	MOGAT3(32)	AMINO_ACID_TRANSPORT	DEG−subtype.Lauren.Class__Diffuse_vs_Mixed__DN	DEG−TumorNormal.neoplasm_histologic_grade__G2__UP
24	M417	M115	86	FILIP1,MYOCD,DMD,JA202350,LOC401093,MIR143HG,DNAJB5,PDE1C,SLMAP,VCL	FILIP1(27),SLMAP(16)	DYSTROPHIN_ASSOCIATED_GLYCOPROTEIN_COMPLEX	DEG−subtype.WHO.Class__Papillary_vs_Poorly_Cohesive__DN	DEG−TumorNormal.Anatomic.Region__GEJ_CARDIA__DN
25	M122	M7	134	GALNT3,ERN2,SLC44A4,AKR7A3,AKR7L,FAM83E,FUT3,AGR2,SLC50A1,GFPT1	GALNT3(24),SLC44A4(22),ERN2(20),AKR7A3(17),GFPT1(17),AKR7L(16)	KEGG_O_GLYCAN_BIOSYNTHESIS	DEG−subtype.Molecular.Subtype__EBV_vs_MSI__DN	DEG−TumorNormal.Molecular.Subtype__CIN__UP
26	M127	M7	186	FABP1,MEP1A,FABP2,BTNL3,PLA2G12B,SI,TM6SF2,C10orf112,HAPLN4,OSTBETA	FABP2(33),TM6SF2(28),BTNL3(27),PLA2G12B(21),MEP1A(20),FABP1(20),SI(16)	LIPID_METABOLIC_PROCESS	DEG−subtype.Molecular.Subtype__CIN_vs_EBV__UP	DEG−TumorNormal.Anatomic.Region__FUNDUS_BODY__DN
27	M9	M4	91	MAK16,TTI2,DOCK5,FUT10,XPO7,GTF2E2,KCTD9,TNFRSF10A,INTS10,LOC389641	DOCK5(21),XPO7(17),TTI2(17)	OXIDOREDUCTASE_ACTIVITY_ACTING_ON_SULFUR_GROUP_OF_DONORS	NA	NA
28	M258	M41	60	NOP56,MCM8,TRMT6,STK35,SNRPB,FASTKD5,CDC25B,C20orf72,MRPS26,PCNA	SNRPB(19),MCM8(17)	RNA_PROCESSING	NA	DEG−TumorNormal.Molecular.Subtype__CIN__UP
29	M475	M137	154	PKP3,ESRP2,SPINT1,EPS8L2,CDS1,C6orf132,CDC42BPG,MYO5B,OR7E14P,LOC100652770	PKP3(39),ESRP2(25),CDS1(24),EPS8L2(16)	INTRACELLULAR_SIGNALING_CASCADE	DEG−subtype.Lauren.Class__Diffuse_vs_Mixed__DN	DEG−TumorNormal.neoplasm_histologic_grade__G2__UP
30	M203	M25	66	MRPS2,BC015688,C9orf116,METTL11A,RPL35,RPL7A,MRRF,NPM3,UBAC1,FXN	BC015688(26),MRPS2(22)	KEGG_RIBOSOME	NA	NA

Supporting Material 2B: Module Preservation Analysis

	Module	medianRank.pres	medianRank.qual	Zconnectivity.pres	log.p.Bonfconnectivity.pres	Zsummary.pres	Zsummary.qual	log.p.Bonfsummary.pres	is.preserved	comparison
1	M110	442.0	530.5	4.50	−2.7	4.1	7.4	−1.90	TRUE	TCGA_GCC.vs.GSE84437
2	M119	311.0	578.0	15.00	−51.0	14.0	7.8	−40.00	TRUE	TCGA_GCC.vs.GSE84437
3	M121	371.0	566.0	11.00	−27.0	8.5	8.7	−16.00	TRUE	TCGA_GCC.vs.GSE84437
4	M122	510.0	581.5	13.00	−35.0	7.8	6.1	−18.00	TRUE	TCGA_GCC.vs.GSE84437
5	M126	471.0	595.0	19.00	−74.0	15.0	7.2	−53.00	TRUE	TCGA_GCC.vs.GSE84437
6	M127	364.0	570.5	17.00	−63.0	11.0	7.1	−34.00	TRUE	TCGA_GCC.vs.GSE84437
7	M129	458.5	544.0	3.30	−0.5	2.9	5.5	−0.25	FALSE	TCGA_GCC.vs.GSE84437
8	M158	555.0	517.0	7.60	−11.0	5.4	6.8	−6.20	TRUE	TCGA_GCC.vs.GSE84437
9	M203	505.0	530.0	6.80	−8.4	4.5	9.2	−4.20	TRUE	TCGA_GCC.vs.GSE84437
10	M226	450.0	551.0	12.00	−29.0	9.3	9.7	−19.00	TRUE	TCGA_GCC.vs.GSE84437
11	M258	399.0	390.5	8.70	−15.0	6.7	7.1	−9.10	TRUE	TCGA_GCC.vs.GSE84437
12	M28	501.0	583.0	14.00	−44.0	10.0	9.3	−25.00	TRUE	TCGA_GCC.vs.GSE84437
13	M338	421.0	595.5	2.00	0.0	2.9	2.3	−0.78	FALSE	TCGA_GCC.vs.GSE84437
14	M349	156.0	358.0	2.50	0.0	4.5	6.7	−3.60	FALSE	TCGA_GCC.vs.GSE84437
15	M395	345.0	315.0	5.60	−5.2	4.5	9.4	−3.00	TRUE	TCGA_GCC.vs.GSE84437
16	M417	387.5	274.5	2.60	0.0	3.1	14.0	−0.60	FALSE	TCGA_GCC.vs.GSE84437
17	M434	243.0	553.0	6.20	−6.7	6.7	4.5	−8.40	TRUE	TCGA_GCC.vs.GSE84437
18	M437	338.0	535.5	8.10	−13.0	7.2	5.3	−10.00	TRUE	TCGA_GCC.vs.GSE84437
19	M438	274.0	479.5	8.30	−14.0	6.7	11.0	−9.20	TRUE	TCGA_GCC.vs.GSE84437
20	M447	495.0	558.0	0.91	0.0	2.3	7.0	−0.67	FALSE	TCGA_GCC.vs.GSE84437
21	M458	308.0	491.0	7.60	−11.0	8.2	10.0	−14.00	TRUE	TCGA_GCC.vs.GSE84437
22	M467	458.5	544.0	5.00	−3.8	3.7	4.4	−2.00	TRUE	TCGA_GCC.vs.GSE84437
23	M475	494.0	584.5	9.40	−18.0	6.2	14.0	−9.00	TRUE	TCGA_GCC.vs.GSE84437
24	M614	61.0	138.5	−1.80	0.0	2.0	3.0	−2.80	FALSE	TCGA_GCC.vs.GSE84437
25	M666	286.0	477.0	6.70	−8.1	5.1	7.7	−4.70	TRUE	TCGA_GCC.vs.GSE84437
26	M667	225.0	390.5	7.90	−12.0	5.9	7.9	−6.90	TRUE	TCGA_GCC.vs.GSE84437
27	M685	282.0	446.5	8.90	−16.0	5.7	6.7	−7.80	TRUE	TCGA_GCC.vs.GSE84437
28	M690	354.0	417.0	2.00	0.0	3.1	6.4	−1.60	FALSE	TCGA_GCC.vs.GSE84437
29	M81	423.0	431.5	6.00	−6.2	5.0	6.8	−4.10	TRUE	TCGA_GCC.vs.GSE84437
30	M9	292.0	364.5	7.70	−12.0	6.8	11.0	−8.90	TRUE	TCGA_GCC.vs.GSE84437

	Module	medianRank.pres	medianRank.qual	Zconnectivity.pres	log.p.Bonfconnectivity.pres	Zsummary.pres	Zsummary.qual	log.p.Bonfsummary.pres	is.preserved	comparison
1	M110	386.0	570.0	5.200	−4.3	11.000	6.1	−40.00	TRUE	TCGA_GCC.vs.TCGA_vGCC
2	M119	379.0	569.5	27.000	−150.0	21.000	9.6	−100.00	TRUE	TCGA_GCC.vs.TCGA_vGCC
3	M121	526.0	561.5	21.000	−99.0	17.000	9.0	−69.00	TRUE	TCGA_GCC.vs.TCGA_vGCC
4	M122	510.0	577.0	24.000	−130.0	17.000	5.8	−77.00	TRUE	TCGA_GCC.vs.TCGA_vGCC
5	M126	535.0	596.5	16.000	−58.0	13.000	8.4	−39.00	TRUE	TCGA_GCC.vs.TCGA_vGCC
6	M127	544.0	573.5	18.000	−66.0	12.000	7.9	−37.00	TRUE	TCGA_GCC.vs.TCGA_vGCC
7	M129	597.5	575.0	−0.049	0.0	1.300	5.0	−0.77	FALSE	TCGA_GCC.vs.TCGA_vGCC
8	M158	503.0	485.5	17.000	−59.0	17.000	7.6	−75.00	TRUE	TCGA_GCC.vs.TCGA_vGCC
9	M203	441.0	544.5	13.000	−36.0	10.000	7.4	−25.00	TRUE	TCGA_GCC.vs.TCGA_vGCC
10	M226	486.0	543.5	21.000	−98.0	16.000	8.0	−62.00	TRUE	TCGA_GCC.vs.TCGA_vGCC
11	M258	260.0	435.0	12.000	−28.0	12.000	8.6	−31.00	TRUE	TCGA_GCC.vs.TCGA_vGCC
12	M28	517.0	572.0	25.000	−130.0	19.000	23.0	−88.00	TRUE	TCGA_GCC.vs.TCGA_vGCC
13	M338	590.0	597.5	2.700	0.0	2.800	2.6	−1.50	FALSE	TCGA_GCC.vs.TCGA_vGCC
14	M349	530.0	429.5	5.800	−5.8	6.100	6.0	−8.10	TRUE	TCGA_GCC.vs.TCGA_vGCC
15	M395	348.0	403.5	1.900	0.0	8.100	6.5	−23.00	FALSE	TCGA_GCC.vs.TCGA_vGCC
16	M417	232.0	368.5	8.500	−14.0	12.000	11.0	−33.00	TRUE	TCGA_GCC.vs.TCGA_vGCC
17	M434	335.0	549.5	13.000	−34.0	12.000	6.2	−28.00	TRUE	TCGA_GCC.vs.TCGA_vGCC
18	M437	487.0	529.5	13.000	−35.0	11.000	9.9	−25.00	TRUE	TCGA_GCC.vs.TCGA_vGCC
19	M438	455.0	472.0	16.000	−55.0	13.000	11.0	−39.00	TRUE	TCGA_GCC.vs.TCGA_vGCC
20	M447	457.0	558.5	11.000	−27.0	9.200	4.9	−18.00	TRUE	TCGA_GCC.vs.TCGA_vGCC
21	M458	459.0	506.5	15.000	−49.0	12.000	9.8	−33.00	TRUE	TCGA_GCC.vs.TCGA_vGCC
22	M467	597.5	575.0	−0.470	0.0	0.970	4.1	−0.67	FALSE	TCGA_GCC.vs.TCGA_vGCC
23	M475	560.0	590.0	17.000	−59.0	13.000	11.0	−38.00	TRUE	TCGA_GCC.vs.TCGA_vGCC
24	M614	592.0	554.5	−0.750	0.0	1.100	5.0	−1.00	FALSE	TCGA_GCC.vs.TCGA_vGCC
25	M666	495.0	478.0	8.600	−15.0	7.900	7.5	−13.00	TRUE	TCGA_GCC.vs.TCGA_vGCC
26	M667	399.0	384.5	6.700	−8.3	9.000	13.0	−17.00	TRUE	TCGA_GCC.vs.TCGA_vGCC
27	M685	401.0	427.5	8.300	−13.0	8.100	7.6	−13.00	TRUE	TCGA_GCC.vs.TCGA_vGCC
28	M690	601.0	437.0	0.530	0.0	0.023	3.5	0.00	FALSE	TCGA_GCC.vs.TCGA_vGCC
29	M81	502.0	435.0	7.600	−11.0	7.500	8.2	−11.00	TRUE	TCGA_GCC.vs.TCGA_vGCC
30	M9	336.0	339.5	15.000	−45.0	13.000	11.0	−38.00	TRUE	TCGA_GCC.vs.TCGA_vGCC

Supporting Material 2C: TCGA barcode list

	cohort	barcodes
TCGA−GCC	TCGA−GCC	TCGA−BR−8364,TCGA−BR−8363,TCGA−BR−8380,TCGA−BR−8372,TCGA−BR−8486,TCGA−BR−8589,TCGA−BR−8592,TCGA−BR−8678,TCGA−BR−8591,TCGA−IN−7808,<br>TCGA−BR−8686,TCGA−IN−8462,TCGA−BR−6707,TCGA−HU−A4GN,TCGA−HU−A4GP,TCGA−HU−A4GC,TCGA−D7−6527,TCGA−D7−6524,TCGA−D7−6528,TCGA−D7−6522,<br>TCGA−FP−8631,TCGA−HU−8238,TCGA−HU−A4GF,TCGA−HU−A4G3,TCGA−D7−5578,TCGA−D7−6820,TCGA−HU−A4GJ,TCGA−HU−8610,TCGA−HU−A4G9,TCGA−HU−A4GH,<br>TCGA−D7−6525,TCGA−BR−6705,TCGA−D7−6815,TCGA−BR−6852,TCGA−BR−6710,TCGA−BR−6564,TCGA−D7−6817,TCGA−HU−8602,TCGA−BR−4255,TCGA−BR−4187,<br>TCGA−BR−4257,TCGA−BR−4267,TCGA−HU−A4HB,TCGA−HU−A4H3,TCGA−HU−A4G8,TCGA−BR−A4PE,TCGA−HU−A4GX,TCGA−HU−A4H0,TCGA−HU−A4GU,TCGA−D7−6526,<br>TCGA−D7−8578,TCGA−BR−8286,TCGA−D7−8579,TCGA−BR−8365,TCGA−CD−8529,TCGA−BR−8366,TCGA−BR−8060,TCGA−HU−8244,TCGA−BR−8080,TCGA−HU−A4G6,<br>TCGA−BR−A4CR,TCGA−BR−8373,TCGA−CD−8532,TCGA−BR−7716,TCGA−FP−7916,TCGA−IP−7968,TCGA−F1−6875,TCGA−IN−7806,TCGA−BR−7717,TCGA−B7−5818,<br>TCGA−D7−5577,TCGA−CD−5801,TCGA−BR−6566,TCGA−FP−8209,TCGA−BR−8367,TCGA−BR−8289,TCGA−BR−8371,TCGA−CD−8526,TCGA−BR−8368,TCGA−D7−8573,<br>TCGA−BR−7722,TCGA−CD−5804,TCGA−BR−6563,TCGA−D7−6818,TCGA−BR−6709,TCGA−BR−6803,TCGA−D7−6822,TCGA−D7−6521,TCGA−CD−8524,TCGA−CD−8530,<br>TCGA−FP−8211,TCGA−CD−A487,TCGA−CD−A489,TCGA−D7−8570,TCGA−CD−8528,TCGA−CD−8531,TCGA−BR−8078,TCGA−BR−8077,TCGA−CD−8534,TCGA−FP−8099,<br>TCGA−BR−8059,TCGA−BR−8679,TCGA−HU−8604,TCGA−BR−8382,TCGA−BR−8588,TCGA−HU−8608,TCGA−IN−8663,TCGA−BR−8683,TCGA−BR−8687,TCGA−BR−8384,<br>TCGA−BR−8590,TCGA−BR−8381,TCGA−BR−8680,TCGA−BR−8677,TCGA−BR−8483,TCGA−HU−A4HD,TCGA−HU−A4H4,TCGA−HU−A4H6,TCGA−D7−A4YV,TCGA−HU−A4H5,<br>TCGA−BR−A4QI,TCGA−HU−A4H2,TCGA−BR−6801,TCGA−F1−6874,TCGA−BR−6706,TCGA−BR−6802,TCGA−CD−8535,TCGA−BR−8362,TCGA−D7−8576,TCGA−CD−A48C,<br>TCGA−CD−8533,TCGA−HU−A4GY,TCGA−BR−A44T,TCGA−BR−8297,TCGA−EQ−8122,TCGA−D7−8575,TCGA−BR−8284,TCGA−FP−7735,TCGA−BR−7723,TCGA−CD−5813,<br>TCGA−B7−5816,TCGA−D7−A4YY,TCGA−D7−A4YX,TCGA−D7−A4YU,TCGA−D7−6520,TCGA−BR−6565,TCGA−BR−8484,TCGA−BR−8487,TCGA−BR−8682,TCGA−BR−8485,<br>TCGA−BR−8058,TCGA−BR−8369,TCGA−CD−8536,TCGA−D7−8572,TCGA−CD−A486,TCGA−FP−A4BE,TCGA−BR−8361,TCGA−BR−A453,TCGA−F1−A448,TCGA−BR−A4CS,<br>TCGA−BR−8291,TCGA−BR−8081,TCGA−D7−8574,TCGA−FP−8210,TCGA−BR−4280,TCGA−BR−4292,TCGA−BR−4256,TCGA−BR−7901,TCGA−BR−7707,TCGA−BR−7715,<br>TCGA−CD−5803,TCGA−CD−5800,TCGA−BR−A4PF,TCGA−HU−A4H8,TCGA−D7−A4YT,TCGA−HU−A4GT,TCGA−BR−A4PD,TCGA−BR−A452,TCGA−HU−A4G2,TCGA−EQ−A4SO,<br>TCGA−CD−5798,TCGA−CD−5799,TCGA−D7−6518,TCGA−D7−6519,TCGA−F1−6177,TCGA−BR−6452,TCGA−BR−6453,TCGA−BR−6454,TCGA−BR−6455,TCGA−BR−6456,<br>TCGA−BR−6457,TCGA−BR−6458,TCGA−BR−7196,TCGA−BR−7703,TCGA−FP−7829,TCGA−BR−7197,TCGA−BR−7851,TCGA−BR−7957,TCGA−FP−7998,TCGA−BR−7958,<br>TCGA−BR−7959,TCGA−BR−A4J7,TCGA−BR−A4IU,TCGA−BR−A4J9,TCGA−CD−A4MH,TCGA−D7−A4Z0,TCGA−BR−A4IY,TCGA−BR−A4J6,TCGA−BR−A4J4,TCGA−CD−A4MJ
vTCGA−GCC	vTCGA−GCC	TCGA−VQ−A8PD,TCGA−IN−A7NR,TCGA−D7−A6EX,TCGA−VQ−A8E2,TCGA−VQ−A8PK,TCGA−BR−4367,TCGA−VQ−A94P,TCGA−VQ−A925,TCGA−VQ−A8DZ,TCGA−VQ−A91X,<br>TCGA−BR−4191,TCGA−HJ−7597,TCGA−VQ−A8P5,TCGA−D7−A6F2,TCGA−MX−A666,TCGA−VQ−A8P3,TCGA−R5−A7ZI,TCGA−FP−A4BF,TCGA−RD−A7BS,TCGA−IN−A6RS,<br>TCGA−VQ−A8P8,TCGA−FP−A8CX,TCGA−KB−A93J,TCGA−VQ−A8PE,TCGA−IN−A6RN,TCGA−IN−A6RJ,TCGA−VQ−AA6F,TCGA−ZA−A8F6,TCGA−HU−A4GD,TCGA−RD−A8N2,<br>TCGA−BR−4361,TCGA−R5−A805,TCGA−ZQ−A9CR,TCGA−D7−A6EV,TCGA−VQ−AA6A,TCGA−BR−4279,TCGA−D7−A6EY,TCGA−VQ−A928,TCGA−R5−A7ZE,TCGA−D7−A6EZ,<br>TCGA−VQ−A91A,TCGA−VQ−AA64,TCGA−IN−A6RO,TCGA−RD−A8N6,TCGA−VQ−A91U,TCGA−D7−A748,TCGA−R5−A7ZR,TCGA−VQ−A8E0,TCGA−BR−4370,TCGA−KB−A93G,<br>TCGA−MX−A5UG,TCGA−B7−A5TI,TCGA−D7−A74A,TCGA−BR−4363,TCGA−VQ−A8PH,TCGA−IN−A6RI,TCGA−VQ−A91K,TCGA−BR−A44U,TCGA−VQ−A8PU,TCGA−BR−4366,<br>TCGA−RD−A8N9,TCGA−VQ−A91E,TCGA−D7−A6F0,TCGA−IN−AB1X,TCGA−VQ−A8PC,TCGA−VQ−A8E7,TCGA−VQ−AA6K,TCGA−IN−AB1V,TCGA−R5−A7ZF,TCGA−IN−A7NU,<br>TCGA−RD−A8N0,TCGA−VQ−A92D,TCGA−MX−A5UJ,TCGA−IN−A6RL,TCGA−VQ−A91D,TCGA−BR−7704,TCGA−BR−8295,TCGA−VQ−A91Z,TCGA−RD−A7BW,TCGA−VQ−A94R,<br>TCGA−RD−A8N1,TCGA−SW−A7EA,TCGA−CD−8525,TCGA−BR−8690,TCGA−RD−A8MV,TCGA−RD−A8MW,TCGA−BR−4294,TCGA−VQ−A8PO,TCGA−RD−A7BT,TCGA−BR−8676,<br>TCGA−D7−A747,TCGA−BR−4357,TCGA−VQ−A8DV,TCGA−VQ−A923,TCGA−BR−4371,TCGA−RD−A8N4,TCGA−VQ−A94O,TCGA−VQ−A8PQ,TCGA−F1−A72C,TCGA−BR−4253,<br>TCGA−VQ−A927,TCGA−BR−4368,TCGA−VQ−A94U,TCGA−HU−8249,TCGA−KB−A93H,TCGA−RD−A8NB,TCGA−BR−4201,TCGA−B7−A5TK,TCGA−B7−A5TJ,TCGA−SW−A7EB,<br>TCGA−BR−4369,TCGA−RD−A7C1,TCGA−CD−A48A,TCGA−VQ−AA6G,TCGA−BR−A4QL,TCGA−BR−8296,TCGA−FP−A9TM,TCGA−VQ−AA69,TCGA−R5−A7O7,TCGA−VQ−A8E3,<br>TCGA−RD−A8N5,TCGA−CD−8527,TCGA−VQ−A91Y,TCGA−VQ−A91S,TCGA−MX−A663,TCGA−IN−A7NT,TCGA−VQ−AA6J,TCGA−VQ−AA68,TCGA−VQ−A8PM,TCGA−VQ−A8DU

Supporting Material 3A: TCGA patients with somatic mutations

	gene	barcodes	gene	barcodes
TP53	TP53	TCGA−BR−4256,TCGA−CD−8535,TCGA−BR−A4PE,TCGA−CD−A48C,TCGA−BR−8487,TCGA−FP−A4BE,TCGA−HU−A4GN,TCGA−BR−8289,TCGA−BR−8361,TCGA−CD−8524,TCGA−BR−7197,TCGA−D7−A4YU,TCGA−BR−8483,TCGA−BR−7723,TCGA−BR−8678,TCGA−CD−8533,TCGA−BR−8369,TCGA−BR−A4QI,TCGA−FP−7829,TCGA−BR−4292,TCGA−CD−8531,TCGA−BR−A452,TCGA−BR−8286,TCGA−BR−8590,TCGA−BR−6457,TCGA−CD−8528,TCGA−F1−6875,TCGA−HU−A4GX,TCGA−B7−5818,TCGA−D7−6815,TCGA−D7−5578,TCGA−BR−8297,TCGA−BR−7703,TCGA−HU−A4GH,TCGA−IN−7806,TCGA−D7−6520,TCGA−CD−8530,TCGA−HU−A4GF,TCGA−CD−5813,TCGA−D7−8578,TCGA−F1−6874,TCGA−FP−7735,TCGA−HU−A4GP,TCGA−IN−7808,TCGA−CD−A487,TCGA−BR−8080,TCGA−BR−A4PD,TCGA−BR−A4IY,TCGA−HU−8244,TCGA−FP−8099,TCGA−CD−5804,TCGA−D7−6525,TCGA−CD−5800,TCGA−BR−A4CR,TCGA−FP−8631,TCGA−BR−A4CS,TCGA−D7−6518,TCGA−BR−8077,TCGA−BR−6456,TCGA−BR−4255,TCGA−D7−6820,TCGA−CD−8526,TCGA−IP−7968,TCGA−BR−7715,TCGA−BR−6565,TCGA−CD−A489,TCGA−BR−8484,TCGA−D7−6817,TCGA−D7−8576,TCGA−BR−8682,TCGA−CD−5799	DMD	TCGA−BR−8487,TCGA−BR−8289,TCGA−CD−8524,TCGA−BR−A452,TCGA−HU−A4GX,TCGA−HU−A4GH,TCGA−BR−8080,TCGA−BR−8680,TCGA−BR−8591,TCGA−BR−6452,TCGA−HU−A4GU,TCGA−BR−8485,TCGA−HU−8604,TCGA−BR−7958,TCGA−D7−A4YV,TCGA−HU−A4GT,TCGA−BR−8284,TCGA−HU−8602,TCGA−HU−A4H0,TCGA−BR−8081,TCGA−FP−7916,TCGA−BR−8078,TCGA−D7−6818,TCGA−D7−6521
LRP1B	LRP1B	TCGA−BR−4256,TCGA−CD−8535,TCGA−BR−A4PE,TCGA−CD−A48C,TCGA−BR−8487,TCGA−FP−A4BE,TCGA−HU−A4GN,TCGA−BR−8289,TCGA−BR−8361,TCGA−CD−8524,TCGA−BR−7197,TCGA−D7−A4YU,TCGA−BR−8483,TCGA−BR−7723,TCGA−BR−8678,TCGA−CD−8533,TCGA−BR−8369,TCGA−BR−A4QI,TCGA−FP−7829,TCGA−BR−8680,TCGA−BR−8591,TCGA−HU−A4H3,TCGA−BR−4257,TCGA−BR−6452,TCGA−B7−5816,TCGA−HU−A4GU,TCGA−BR−7851,TCGA−F1−6177,TCGA−CD−5801,TCGA−BR−A4PF,TCGA−HU−A4H4,TCGA−D7−A4YY,TCGA−CD−A4MG,TCGA−BR−6566,TCGA−HU−A4GC,TCGA−CD−A4MJ,TCGA−BR−8059,TCGA−D7−6528,TCGA−BR−8683,TCGA−BR−8485,TCGA−HU−8604,TCGA−BR−7958,TCGA−BR−A4J5,TCGA−CD−A4MI,TCGA−BR−7959,TCGA−CD−A486	ERBB4	TCGA−BR−4256,TCGA−BR−8361,TCGA−CD−8524,TCGA−BR−7197,TCGA−BR−7703,TCGA−CD−5813,TCGA−F1−6874,TCGA−BR−A4PD,TCGA−D7−6820,TCGA−BR−8680,TCGA−BR−4257,TCGA−BR−6452,TCGA−B7−5816,TCGA−CD−5801,TCGA−CD−A4MG,TCGA−BR−8485,TCGA−BR−7958,TCGA−D7−A4Z0,TCGA−HU−A4GT,TCGA−CD−8536,TCGA−BR−8679,TCGA−BR−8363,TCGA−BR−8372,TCGA−BR−A4J8
CSMD3	CSMD3	TCGA−BR−4256,TCGA−CD−8535,TCGA−BR−A4PE,TCGA−CD−A48C,TCGA−BR−4292,TCGA−CD−8531,TCGA−BR−A452,TCGA−BR−8286,TCGA−BR−8590,TCGA−BR−6457,TCGA−CD−8528,TCGA−F1−6875,TCGA−BR−8680,TCGA−BR−8591,TCGA−HU−A4H3,TCGA−BR−4257,TCGA−D7−A4YV,TCGA−BR−8588,TCGA−HU−A4H8,TCGA−HU−A4G8,TCGA−D7−A4Z0,TCGA−BR−4280,TCGA−HU−A4H2,TCGA−D7−6822,TCGA−D7−6526,TCGA−CD−8534,TCGA−IN−8462,TCGA−BR−6709,TCGA−HU−A4G9,TCGA−IN−8663,TCGA−HU−A4GT,TCGA−HU−A4HD,TCGA−BR−6852,TCGA−D7−8572,TCGA−BR−8284,TCGA−BR−8677,TCGA−BR−7722,TCGA−BR−8384	PLEC	TCGA−BR−8487,TCGA−FP−A4BE,TCGA−BR−8361,TCGA−BR−8483,TCGA−HU−A4H3,TCGA−BR−6452,TCGA−F1−6177,TCGA−BR−A4PF,TCGA−D7−A4YY,TCGA−CD−A4MG,TCGA−CD−A4MJ,TCGA−CD−A4MI,TCGA−D7−A4YV,TCGA−HU−A4G8,TCGA−HU−A4G9,TCGA−HU−A4GT,TCGA−HU−8602,TCGA−BR−6455,TCGA−BR−8368,TCGA−BR−6458,TCGA−BR−7707,TCGA−F1−A448,TCGA−BR−8382,TCGA−BR−A4J7
PIK3CA	PIK3CA	TCGA−BR−8487,TCGA−HU−A4GX,TCGA−B7−5818,TCGA−D7−6815,TCGA−BR−8680,TCGA−BR−8591,TCGA−HU−A4H3,TCGA−BR−6452,TCGA−B7−5816,TCGA−HU−A4GU,TCGA−BR−7851,TCGA−F1−6177,TCGA−CD−5801,TCGA−BR−A4PF,TCGA−HU−A4H4,TCGA−D7−A4YV,TCGA−BR−8588,TCGA−HU−8602,TCGA−BR−6454,TCGA−HU−A4H0,TCGA−BR−6706,TCGA−BR−8589,TCGA−BR−8081,TCGA−CD−8536,TCGA−HU−8608,TCGA−D7−5577,TCGA−D7−A4YX,TCGA−BR−6707,TCGA−BR−6455,TCGA−BR−6802,TCGA−FP−7998,TCGA−BR−7196,TCGA−BR−8686,TCGA−HU−8238,TCGA−FP−7916,TCGA−BR−8366,TCGA−HU−A4G2	RNF213	TCGA−BR−4256,TCGA−BR−8487,TCGA−FP−A4BE,TCGA−BR−8361,TCGA−BR−4292,TCGA−HU−A4GX,TCGA−B7−5818,TCGA−F1−6874,TCGA−BR−8680,TCGA−HU−A4H3,TCGA−BR−6452,TCGA−HU−A4GU,TCGA−CD−5801,TCGA−CD−A4MG,TCGA−HU−A4G8,TCGA−BR−8284,TCGA−HU−8602,TCGA−BR−8081,TCGA−CD−8536,TCGA−BR−8078,TCGA−BR−6453,TCGA−BR−8363,TCGA−BR−7707,TCGA−BR−8687
FAT4	FAT4	TCGA−BR−4256,TCGA−FP−A4BE,TCGA−HU−A4GN,TCGA−BR−4292,TCGA−CD−8531,TCGA−D7−5578,TCGA−BR−8297,TCGA−BR−7703,TCGA−BR−8680,TCGA−BR−8591,TCGA−BR−4257,TCGA−BR−6452,TCGA−B7−5816,TCGA−HU−A4GU,TCGA−BR−7851,TCGA−D7−A4YY,TCGA−CD−A4MG,TCGA−BR−6566,TCGA−HU−A4GC,TCGA−CD−A4MJ,TCGA−HU−A4H8,TCGA−HU−A4G8,TCGA−D7−A4Z0,TCGA−BR−4280,TCGA−HU−A4H2,TCGA−HU−8602,TCGA−BR−6454,TCGA−HU−A4H0,TCGA−BR−6706,TCGA−BR−8368,TCGA−BR−8078,TCGA−D7−6818,TCGA−BR−6458,TCGA−BR−6453,TCGA−BR−8679,TCGA−HU−A4G6	UTRN	TCGA−BR−8487,TCGA−FP−A4BE,TCGA−BR−7197,TCGA−D7−A4YU,TCGA−BR−6457,TCGA−HU−A4GX,TCGA−BR−7703,TCGA−HU−A4GP,TCGA−CD−8526,TCGA−BR−8680,TCGA−BR−6452,TCGA−HU−A4GU,TCGA−F1−6177,TCGA−CD−5801,TCGA−HU−A4H4,TCGA−CD−A4MJ,TCGA−BR−7958,TCGA−HU−A4G8,TCGA−D7−6822,TCGA−BR−6802,TCGA−D7−6818,TCGA−BR−6458,TCGA−BR−8381,TCGA−CD−A4MH
RYR2	RYR2	TCGA−CD−8535,TCGA−BR−8487,TCGA−BR−8289,TCGA−BR−8361,TCGA−CD−8524,TCGA−HU−A4GX,TCGA−D7−5578,TCGA−HU−A4GH,TCGA−IN−7806,TCGA−D7−6520,TCGA−CD−8530,TCGA−BR−8680,TCGA−BR−4257,TCGA−BR−6452,TCGA−B7−5816,TCGA−F1−6177,TCGA−CD−5801,TCGA−D7−A4YY,TCGA−CD−A4MG,TCGA−BR−8059,TCGA−D7−6528,TCGA−BR−8683,TCGA−HU−A4H8,TCGA−HU−A4G8,TCGA−D7−6822,TCGA−D7−6526,TCGA−CD−8534,TCGA−BR−8589,TCGA−BR−8081,TCGA−CD−8536,TCGA−BR−8368,TCGA−BR−8363,TCGA−EQ−A4SO,TCGA−EQ−8122,TCGA−BR−7707,TCGA−BR−8380	CDH23	TCGA−BR−8487,TCGA−BR−8289,TCGA−BR−4292,TCGA−HU−A4GX,TCGA−BR−7703,TCGA−F1−6874,TCGA−BR−8680,TCGA−BR−4257,TCGA−BR−6452,TCGA−CD−A4MG,TCGA−D7−6528,TCGA−BR−7958,TCGA−BR−7959,TCGA−D7−A4YV,TCGA−HU−A4G8,TCGA−HU−A4HD,TCGA−BR−8589,TCGA−CD−8536,TCGA−BR−6453,TCGA−BR−7707,TCGA−BR−8367,TCGA−HU−8610,TCGA−FP−8211
HMCN1	HMCN1	TCGA−BR−A4PE,TCGA−BR−8487,TCGA−FP−A4BE,TCGA−BR−8289,TCGA−BR−8361,TCGA−BR−7197,TCGA−B7−5818,TCGA−HU−A4GH,TCGA−HU−A4GF,TCGA−CD−5813,TCGA−D7−8578,TCGA−F1−6874,TCGA−FP−7735,TCGA−BR−8680,TCGA−BR−6452,TCGA−HU−A4GU,TCGA−BR−A4PF,TCGA−D7−A4YY,TCGA−BR−8485,TCGA−D7−A4YV,TCGA−BR−8588,TCGA−HU−A4H8,TCGA−D7−A4Z0,TCGA−D7−6822,TCGA−IN−8462,TCGA−BR−6709,TCGA−HU−8602,TCGA−BR−8078,TCGA−BR−8363,TCGA−EQ−A4SO,TCGA−BR−8486,TCGA−HU−A4G3,TCGA−BR−7717,TCGA−BR−8367	PCDH10	TCGA−BR−8487,TCGA−FP−A4BE,TCGA−BR−8361,TCGA−CD−8524,TCGA−CD−8531,TCGA−BR−7703,TCGA−F1−6874,TCGA−IP−7968,TCGA−BR−8680,TCGA−BR−4257,TCGA−F1−6177,TCGA−CD−A4MI,TCGA−D7−A4YV,TCGA−HU−A4G8,TCGA−BR−8284,TCGA−HU−8602,TCGA−HU−A4H0,TCGA−D7−A4YX,TCGA−BR−8078,TCGA−BR−8363,TCGA−D7−8570,TCGA−BR−8060,TCGA−FP−8211
PCLO	PCLO	TCGA−BR−4256,TCGA−CD−8535,TCGA−BR−8487,TCGA−FP−A4BE,TCGA−BR−7197,TCGA−D7−A4YU,TCGA−BR−8483,TCGA−BR−7723,TCGA−BR−4292,TCGA−BR−A452,TCGA−HU−A4GX,TCGA−B7−5818,TCGA−BR−8297,TCGA−HU−A4GH,TCGA−HU−A4GP,TCGA−IN−7808,TCGA−BR−8680,TCGA−BR−8591,TCGA−HU−A4GU,TCGA−CD−A4MG,TCGA−BR−8059,TCGA−HU−8604,TCGA−HU−A4H8,TCGA−HU−A4G8,TCGA−BR−4280,TCGA−D7−6822,TCGA−HU−A4G9,TCGA−IN−8663,TCGA−BR−8589,TCGA−BR−8486,TCGA−D7−6527,TCGA−D7−8579,TCGA−F1−A448,TCGA−HU−A4H5	XIRP2	TCGA−BR−A4PE,TCGA−D7−6520,TCGA−HU−A4GP,TCGA−CD−A487,TCGA−BR−A4PD,TCGA−CD−5800,TCGA−BR−8680,TCGA−BR−4257,TCGA−BR−6452,TCGA−HU−A4GU,TCGA−CD−5801,TCGA−HU−A4H4,TCGA−CD−A4MG,TCGA−BR−8059,TCGA−BR−4280,TCGA−D7−6526,TCGA−HU−8602,TCGA−BR−6454,TCGA−BR−6706,TCGA−BR−8081,TCGA−BR−8078,TCGA−BR−8679,TCGA−BR−A4J9
CSMD1	CSMD1	TCGA−BR−A4PE,TCGA−CD−A48C,TCGA−FP−A4BE,TCGA−HU−A4GN,TCGA−BR−8289,TCGA−B7−5818,TCGA−HU−A4GF,TCGA−CD−A487,TCGA−BR−8080,TCGA−BR−A4PD,TCGA−BR−A4IY,TCGA−BR−8680,TCGA−HU−A4H3,TCGA−BR−4257,TCGA−BR−6452,TCGA−CD−A4MG,TCGA−BR−6566,TCGA−BR−7958,TCGA−HU−A4G8,TCGA−D7−6822,TCGA−HU−A4G9,TCGA−HU−A4GT,TCGA−HU−8602,TCGA−BR−8081,TCGA−HU−8608,TCGA−D7−5577,TCGA−BR−8078,TCGA−D7−6818,TCGA−EQ−8122,TCGA−HU−A4G3,TCGA−D7−6527,TCGA−BR−8372,TCGA−BR−8291	CUBN	TCGA−CD−A48C,TCGA−FP−A4BE,TCGA−BR−8289,TCGA−BR−7197,TCGA−BR−A4QI,TCGA−BR−4292,TCGA−HU−A4GX,TCGA−F1−6874,TCGA−BR−8080,TCGA−BR−7715,TCGA−BR−8680,TCGA−BR−4257,TCGA−HU−A4GU,TCGA−BR−8485,TCGA−BR−A4J5,TCGA−CD−A486,TCGA−HU−A4G8,TCGA−HU−A4H2,TCGA−IN−8663,TCGA−BR−8589,TCGA−BR−8081,TCGA−D7−6527
SPTA1	SPTA1	TCGA−BR−4256,TCGA−HU−A4GN,TCGA−D7−A4YU,TCGA−HU−A4GX,TCGA−D7−5578,TCGA−BR−8297,TCGA−BR−7703,TCGA−HU−A4GH,TCGA−HU−A4GF,TCGA−CD−A487,TCGA−BR−8080,TCGA−BR−8680,TCGA−HU−A4H3,TCGA−BR−4257,TCGA−BR−6452,TCGA−HU−A4GU,TCGA−HU−A4H4,TCGA−D7−A4YY,TCGA−BR−6566,TCGA−HU−A4GC,TCGA−BR−8485,TCGA−HU−A4G8,TCGA−D7−A4Z0,TCGA−BR−8081,TCGA−D7−A4YX,TCGA−BR−8368,TCGA−BR−8363,TCGA−D7−6527,TCGA−CD−8529,TCGA−D7−6524,TCGA−D7−8570,TCGA−BR−A4IU	FAT2	TCGA−BR−8361,TCGA−BR−4292,TCGA−CD−8531,TCGA−D7−6815,TCGA−BR−7703,TCGA−HU−A4GH,TCGA−D7−8578,TCGA−BR−6565,TCGA−BR−8680,TCGA−BR−8591,TCGA−BR−6452,TCGA−F1−6177,TCGA−BR−A4PF,TCGA−CD−A4MG,TCGA−BR−6566,TCGA−D7−A4YV,TCGA−BR−8588,TCGA−D7−A4YX,TCGA−EQ−8122,TCGA−BR−8372,TCGA−BR−7716,TCGA−D7−8575
USH2A	USH2A	TCGA−BR−8487,TCGA−B7−5818,TCGA−BR−7703,TCGA−HU−A4GH,TCGA−CD−A487,TCGA−BR−8680,TCGA−BR−8591,TCGA−BR−4257,TCGA−BR−6452,TCGA−BR−7851,TCGA−CD−A4MG,TCGA−BR−8059,TCGA−D7−6528,TCGA−BR−8485,TCGA−HU−A4G8,TCGA−BR−4280,TCGA−HU−A4H2,TCGA−D7−6822,TCGA−HU−A4G9,TCGA−HU−A4GT,TCGA−HU−A4HD,TCGA−BR−8589,TCGA−BR−6707,TCGA−BR−8078,TCGA−D7−6818,TCGA−D7−6527,TCGA−CD−8529,TCGA−BR−8060,TCGA−BR−7716,TCGA−BR−8382,TCGA−BR−4187	GLI3	TCGA−FP−A4BE,TCGA−BR−8361,TCGA−BR−8483,TCGA−BR−4292,TCGA−BR−6457,TCGA−HU−A4GX,TCGA−F1−6874,TCGA−HU−A4GP,TCGA−FP−8631,TCGA−BR−8680,TCGA−HU−A4H3,TCGA−HU−A4GC,TCGA−CD−A4MJ,TCGA−BR−8683,TCGA−BR−8485,TCGA−BR−8588,TCGA−D7−A4Z0,TCGA−BR−8078,TCGA−BR−7707,TCGA−D7−8579,TCGA−BR−8382,TCGA−D7−6519
FAT3	FAT3	TCGA−BR−4256,TCGA−BR−A4PE,TCGA−D7−A4YU,TCGA−BR−4292,TCGA−CD−8531,TCGA−BR−A452,TCGA−BR−7703,TCGA−HU−A4GH,TCGA−CD−5813,TCGA−D7−8578,TCGA−HU−8244,TCGA−BR−8680,TCGA−BR−8591,TCGA−BR−4257,TCGA−BR−6452,TCGA−BR−7851,TCGA−F1−6177,TCGA−CD−A4MG,TCGA−BR−6566,TCGA−CD−A4MJ,TCGA−BR−8059,TCGA−BR−4280,TCGA−BR−6852,TCGA−D7−8572,TCGA−BR−6454,TCGA−BR−8078,TCGA−BR−8363,TCGA−BR−8060,TCGA−BR−7716	KMT2D	TCGA−BR−4256,TCGA−BR−8487,TCGA−HU−A4GN,TCGA−BR−8361,TCGA−HU−A4GX,TCGA−BR−8591,TCGA−HU−A4H3,TCGA−BR−6452,TCGA−HU−A4GU,TCGA−F1−6177,TCGA−CD−A4MI,TCGA−HU−A4G8,TCGA−BR−4280,TCGA−D7−6526,TCGA−HU−A4HD,TCGA−HU−8602,TCGA−BR−8589,TCGA−BR−8368,TCGA−BR−7707,TCGA−D7−8570,TCGA−BR−4267,TCGA−BR−8381
ARID1A	ARID1A	TCGA−BR−8487,TCGA−BR−8361,TCGA−D7−A4YU,TCGA−BR−4292,TCGA−FP−8099,TCGA−BR−8680,TCGA−BR−7851,TCGA−CD−A4MJ,TCGA−BR−8059,TCGA−BR−A4J5,TCGA−HU−A4H8,TCGA−HU−A4G9,TCGA−BR−8284,TCGA−BR−8589,TCGA−CD−8536,TCGA−HU−8608,TCGA−D7−5577,TCGA−BR−6455,TCGA−BR−6802,TCGA−FP−7998,TCGA−BR−7196,TCGA−D7−8579,TCGA−BR−8372,TCGA−D7−6524,TCGA−BR−8382,TCGA−BR−8687,TCGA−D7−6521,TCGA−CD−5803	MUC17	TCGA−CD−8535,TCGA−HU−A4GN,TCGA−BR−8678,TCGA−BR−A4QI,TCGA−HU−A4GH,TCGA−BR−A4IY,TCGA−BR−8680,TCGA−BR−8591,TCGA−B7−5816,TCGA−BR−7851,TCGA−CD−A4MG,TCGA−BR−8059,TCGA−D7−A4YV,TCGA−HU−A4G8,TCGA−BR−6852,TCGA−BR−8081,TCGA−BR−6455,TCGA−BR−8078,TCGA−BR−7707,TCGA−BR−8060,TCGA−BR−8382,TCGA−BR−4267
DNAH5	DNAH5	TCGA−CD−A48C,TCGA−BR−8487,TCGA−BR−8286,TCGA−B7−5818,TCGA−CD−5804,TCGA−D7−6525,TCGA−CD−5800,TCGA−BR−8680,TCGA−BR−6452,TCGA−BR−7851,TCGA−BR−6566,TCGA−HU−A4GC,TCGA−BR−8059,TCGA−BR−8485,TCGA−HU−A4H8,TCGA−D7−A4Z0,TCGA−HU−A4H2,TCGA−HU−8602,TCGA−BR−6454,TCGA−BR−8589,TCGA−BR−8363,TCGA−EQ−A4SO,TCGA−BR−7707,TCGA−BR−8380,TCGA−BR−7717,TCGA−BR−8060,TCGA−BR−8382,TCGA−BR−8373	PCDH17	TCGA−BR−8487,TCGA−FP−A4BE,TCGA−HU−A4GN,TCGA−BR−8361,TCGA−BR−7197,TCGA−BR−6457,TCGA−B7−5818,TCGA−HU−A4GH,TCGA−D7−6520,TCGA−HU−8244,TCGA−BR−6452,TCGA−BR−7851,TCGA−F1−6177,TCGA−CD−A4MG,TCGA−D7−6528,TCGA−HU−8604,TCGA−HU−A4H2,TCGA−HU−A4GT,TCGA−HU−8602,TCGA−BR−8078,TCGA−BR−7707,TCGA−BR−8382
DNAH9	DNAH9	TCGA−BR−8487,TCGA−FP−A4BE,TCGA−HU−A4GN,TCGA−BR−4292,TCGA−HU−A4GH,TCGA−HU−A4GF,TCGA−CD−A487,TCGA−BR−A4PD,TCGA−CD−5804,TCGA−BR−A4CR,TCGA−BR−8680,TCGA−BR−4257,TCGA−BR−6452,TCGA−B7−5816,TCGA−BR−8059,TCGA−CD−A4MI,TCGA−HU−A4G8,TCGA−D7−A4Z0,TCGA−BR−8284,TCGA−HU−8602,TCGA−BR−8589,TCGA−BR−8081,TCGA−CD−8536,TCGA−BR−8363,TCGA−F1−A448,TCGA−D7−8570,TCGA−BR−8382,TCGA−BR−7901	PEG3	TCGA−BR−8487,TCGA−BR−8289,TCGA−BR−8361,TCGA−BR−4292,TCGA−BR−8286,TCGA−HU−A4GH,TCGA−HU−A4GP,TCGA−D7−6525,TCGA−BR−8077,TCGA−BR−8680,TCGA−D7−A4YY,TCGA−BR−8059,TCGA−HU−8604,TCGA−D7−A4Z0,TCGA−D7−6526,TCGA−HU−A4G9,TCGA−HU−8602,TCGA−D7−5577,TCGA−BR−8363,TCGA−BR−7717,TCGA−BR−8367,TCGA−BR−8372
PCDH15	PCDH15	TCGA−CD−A48C,TCGA−BR−8487,TCGA−FP−A4BE,TCGA−HU−A4GN,TCGA−BR−4292,TCGA−CD−8531,TCGA−BR−8590,TCGA−HU−A4GX,TCGA−B7−5818,TCGA−CD−5813,TCGA−F1−6874,TCGA−CD−A487,TCGA−BR−8680,TCGA−BR−7851,TCGA−BR−8683,TCGA−HU−8604,TCGA−HU−A4G8,TCGA−D7−A4Z0,TCGA−BR−4280,TCGA−D7−6526,TCGA−HU−A4HD,TCGA−BR−8081,TCGA−BR−8686,TCGA−D7−6818,TCGA−BR−8363,TCGA−BR−8372,TCGA−CD−8529,TCGA−BR−8687	TENM1	TCGA−CD−A48C,TCGA−BR−8487,TCGA−CD−8524,TCGA−CD−8533,TCGA−CD−8531,TCGA−CD−8528,TCGA−BR−8680,TCGA−BR−8591,TCGA−BR−6452,TCGA−B7−5816,TCGA−CD−A4MG,TCGA−HU−A4H8,TCGA−D7−A4Z0,TCGA−BR−4280,TCGA−HU−8602,TCGA−BR−8081,TCGA−BR−6453,TCGA−EQ−8122,TCGA−BR−8486,TCGA−BR−8382,TCGA−BR−8687,TCGA−BR−8592
ZFHX4	ZFHX4	TCGA−BR−4256,TCGA−CD−A48C,TCGA−BR−8487,TCGA−BR−8361,TCGA−BR−8483,TCGA−BR−7703,TCGA−HU−A4GP,TCGA−BR−8680,TCGA−BR−8591,TCGA−BR−4257,TCGA−BR−6452,TCGA−B7−5816,TCGA−BR−7851,TCGA−F1−6177,TCGA−D7−A4YY,TCGA−CD−A4MJ,TCGA−BR−8059,TCGA−BR−8485,TCGA−HU−8604,TCGA−HU−A4H8,TCGA−HU−8602,TCGA−BR−8589,TCGA−HU−8608,TCGA−BR−8368,TCGA−BR−8078,TCGA−D7−6818,TCGA−D7−6527	TG	TCGA−BR−8487,TCGA−FP−A4BE,TCGA−BR−8361,TCGA−BR−7723,TCGA−HU−A4GH,TCGA−HU−A4GP,TCGA−FP−8099,TCGA−BR−8680,TCGA−BR−4257,TCGA−HU−A4GU,TCGA−BR−7851,TCGA−F1−6177,TCGA−HU−A4H4,TCGA−CD−A4MG,TCGA−HU−A4G8,TCGA−D7−A4Z0,TCGA−HU−A4GT,TCGA−BR−8677,TCGA−BR−6706,TCGA−BR−6458,TCGA−BR−7707,TCGA−HU−8610
DNAH11	DNAH11	TCGA−BR−8487,TCGA−FP−A4BE,TCGA−BR−8678,TCGA−BR−4292,TCGA−HU−A4GX,TCGA−B7−5818,TCGA−D7−6815,TCGA−D7−5578,TCGA−BR−8297,TCGA−HU−A4GH,TCGA−FP−8631,TCGA−BR−A4CS,TCGA−BR−8680,TCGA−BR−6452,TCGA−HU−A4GU,TCGA−CD−A4MG,TCGA−D7−6528,TCGA−BR−8683,TCGA−BR−8485,TCGA−BR−8284,TCGA−CD−8536,TCGA−BR−6458,TCGA−D7−8579,TCGA−HU−A4H5,TCGA−BR−6705,TCGA−CD−5798	TRRAP	TCGA−CD−A48C,TCGA−BR−8487,TCGA−FP−A4BE,TCGA−HU−A4GN,TCGA−BR−8680,TCGA−BR−8591,TCGA−BR−4257,TCGA−HU−A4GU,TCGA−BR−7851,TCGA−F1−6177,TCGA−D7−A4YY,TCGA−CD−A4MG,TCGA−CD−A4MJ,TCGA−BR−8059,TCGA−BR−8485,TCGA−BR−4280,TCGA−HU−A4G9,TCGA−HU−A4GT,TCGA−HU−8238,TCGA−BR−8363,TCGA−BR−8060,TCGA−BR−8382
LAMA1	LAMA1	TCGA−CD−8535,TCGA−BR−8487,TCGA−BR−7197,TCGA−B7−5818,TCGA−IN−7806,TCGA−IN−7808,TCGA−D7−6518,TCGA−BR−8680,TCGA−B7−5816,TCGA−F1−6177,TCGA−D7−A4YY,TCGA−CD−A4MG,TCGA−BR−8059,TCGA−HU−8604,TCGA−D7−A4YV,TCGA−HU−A4G8,TCGA−D7−6526,TCGA−IN−8663,TCGA−BR−6852,TCGA−HU−8602,TCGA−BR−8589,TCGA−D7−A4YX,TCGA−BR−6453,TCGA−D7−8579,TCGA−BR−8372,TCGA−BR−6801	AHNAK2	TCGA−BR−4256,TCGA−CD−A48C,TCGA−BR−8487,TCGA−BR−8289,TCGA−BR−8483,TCGA−D7−5578,TCGA−HU−A4GH,TCGA−F1−6874,TCGA−BR−6452,TCGA−HU−A4GU,TCGA−BR−7958,TCGA−CD−A4MI,TCGA−HU−A4H8,TCGA−HU−8602,TCGA−BR−8589,TCGA−BR−8081,TCGA−FP−7998,TCGA−BR−8078,TCGA−BR−8372,TCGA−BR−7716,TCGA−BR−8382
MDN1	MDN1	TCGA−BR−4256,TCGA−BR−8487,TCGA−FP−A4BE,TCGA−BR−8361,TCGA−BR−4292,TCGA−HU−A4GX,TCGA−D7−5578,TCGA−HU−8244,TCGA−BR−8680,TCGA−BR−8591,TCGA−HU−A4H3,TCGA−BR−4257,TCGA−BR−6452,TCGA−HU−A4GU,TCGA−CD−A4MG,TCGA−BR−6566,TCGA−BR−8059,TCGA−D7−6528,TCGA−CD−A4MI,TCGA−HU−8602,TCGA−BR−8589,TCGA−BR−8081,TCGA−BR−8363,TCGA−F1−A448,TCGA−BR−8382,TCGA−D7−8573	COL6A3	TCGA−BR−4256,TCGA−FP−7829,TCGA−BR−4292,TCGA−F1−6875,TCGA−HU−A4GP,TCGA−HU−8244,TCGA−CD−A489,TCGA−BR−8680,TCGA−BR−4257,TCGA−BR−6452,TCGA−B7−5816,TCGA−CD−5801,TCGA−HU−A4H4,TCGA−BR−8059,TCGA−D7−A4YV,TCGA−HU−A4G9,TCGA−BR−6852,TCGA−HU−8238,TCGA−BR−8078,TCGA−BR−8372,TCGA−D7−8570
NEB	NEB	TCGA−FP−A4BE,TCGA−HU−A4GN,TCGA−BR−8361,TCGA−BR−8483,TCGA−CD−8533,TCGA−BR−4292,TCGA−D7−5578,TCGA−F1−6874,TCGA−BR−A4PD,TCGA−HU−8244,TCGA−BR−8077,TCGA−BR−8680,TCGA−BR−8591,TCGA−BR−6452,TCGA−HU−A4GU,TCGA−CD−A4MG,TCGA−CD−A4MJ,TCGA−HU−A4H8,TCGA−HU−A4G9,TCGA−HU−A4GT,TCGA−BR−6852,TCGA−HU−8602,TCGA−BR−8589,TCGA−BR−6458,TCGA−BR−4267,TCGA−BR−A4J4	DNAH3	TCGA−BR−8487,TCGA−B7−5818,TCGA−F1−6874,TCGA−BR−A4PD,TCGA−BR−8680,TCGA−BR−4257,TCGA−BR−6452,TCGA−F1−6177,TCGA−CD−A4MG,TCGA−CD−A4MJ,TCGA−BR−8485,TCGA−D7−A4Z0,TCGA−D7−6526,TCGA−HU−A4G9,TCGA−D7−8572,TCGA−BR−8284,TCGA−BR−8078,TCGA−BR−8363,TCGA−CD−8529,TCGA−BR−8382,TCGA−BR−4267
OBSCN	OBSCN	TCGA−BR−8487,TCGA−FP−A4BE,TCGA−BR−8361,TCGA−CD−8531,TCGA−BR−8286,TCGA−HU−A4GX,TCGA−HU−A4GF,TCGA−HU−A4GP,TCGA−BR−8680,TCGA−HU−A4H3,TCGA−HU−A4GU,TCGA−BR−7851,TCGA−CD−A4MG,TCGA−BR−6566,TCGA−CD−A4MI,TCGA−HU−A4H8,TCGA−HU−A4G8,TCGA−HU−8602,TCGA−BR−8081,TCGA−BR−6707,TCGA−BR−6455,TCGA−HU−8238,TCGA−BR−8368,TCGA−BR−8078,TCGA−BR−8060,TCGA−HU−8610	FBN1	TCGA−BR−8487,TCGA−BR−8289,TCGA−BR−8361,TCGA−D7−A4YU,TCGA−F1−6874,TCGA−BR−8484,TCGA−BR−8680,TCGA−BR−8591,TCGA−BR−4257,TCGA−HU−A4GU,TCGA−CD−A4MG,TCGA−BR−6566,TCGA−CD−A4MI,TCGA−CD−A486,TCGA−HU−A4G9,TCGA−D7−8572,TCGA−HU−8602,TCGA−BR−8078,TCGA−BR−8363,TCGA−BR−8382,TCGA−D7−A4YT
ADGRV1	ADGRV1	TCGA−HU−A4GN,TCGA−BR−8289,TCGA−BR−7723,TCGA−BR−4292,TCGA−B7−5818,TCGA−HU−A4GH,TCGA−CD−A487,TCGA−HU−8244,TCGA−D7−6525,TCGA−BR−8077,TCGA−BR−6456,TCGA−BR−4255,TCGA−BR−8680,TCGA−HU−A4H3,TCGA−HU−A4GU,TCGA−F1−6177,TCGA−D7−A4YY,TCGA−CD−A4MJ,TCGA−BR−8485,TCGA−BR−8588,TCGA−HU−A4GT,TCGA−HU−8602,TCGA−BR−6802,TCGA−BR−8078,TCGA−BR−A4J1	LRP2	TCGA−BR−8487,TCGA−HU−A4GN,TCGA−HU−8244,TCGA−BR−8077,TCGA−BR−8680,TCGA−BR−8591,TCGA−BR−6452,TCGA−BR−7851,TCGA−F1−6177,TCGA−CD−A4MG,TCGA−BR−6566,TCGA−BR−8059,TCGA−D7−6528,TCGA−HU−8604,TCGA−IN−8663,TCGA−HU−A4HD,TCGA−BR−7722,TCGA−D7−6818,TCGA−BR−8363,TCGA−D7−6524,TCGA−BR−8687
RYR3	RYR3	TCGA−BR−4256,TCGA−BR−A4PE,TCGA−BR−8487,TCGA−FP−A4BE,TCGA−BR−8369,TCGA−BR−4292,TCGA−CD−A487,TCGA−BR−8680,TCGA−BR−8591,TCGA−HU−A4H3,TCGA−CD−A4MG,TCGA−HU−A4GC,TCGA−CD−A4MJ,TCGA−BR−8485,TCGA−CD−A4MI,TCGA−HU−A4H8,TCGA−HU−A4GT,TCGA−HU−8602,TCGA−CD−8536,TCGA−BR−8679,TCGA−HU−A4G6,TCGA−BR−8363,TCGA−BR−7707,TCGA−D7−6527,TCGA−BR−4267	MACF1	TCGA−HU−A4GN,TCGA−BR−8361,TCGA−IN−7808,TCGA−BR−8077,TCGA−BR−8680,TCGA−BR−8591,TCGA−BR−4257,TCGA−B7−5816,TCGA−F1−6177,TCGA−HU−A4H4,TCGA−CD−A4MG,TCGA−CD−A4MJ,TCGA−CD−A4MI,TCGA−HU−A4H8,TCGA−BR−6852,TCGA−BR−8363,TCGA−D7−6527,TCGA−F1−A448,TCGA−BR−8372,TCGA−CD−8529,TCGA−BR−7716
APOB	APOB	TCGA−BR−8487,TCGA−FP−A4BE,TCGA−HU−A4GN,TCGA−BR−8369,TCGA−BR−8297,TCGA−HU−A4GH,TCGA−D7−6520,TCGA−F1−6874,TCGA−BR−8680,TCGA−BR−6452,TCGA−HU−A4GU,TCGA−BR−7851,TCGA−CD−A4MG,TCGA−CD−A4MJ,TCGA−BR−8589,TCGA−D7−5577,TCGA−BR−8078,TCGA−BR−6458,TCGA−BR−8363,TCGA−BR−7707,TCGA−BR−8380,TCGA−D7−6527,TCGA−HU−A4H5,TCGA−BR−6705	PREX2	TCGA−BR−4256,TCGA−BR−A4PE,TCGA−FP−A4BE,TCGA−BR−8289,TCGA−CD−8533,TCGA−BR−A4QI,TCGA−BR−4292,TCGA−BR−8286,TCGA−HU−A4GH,TCGA−CD−A487,TCGA−D7−6817,TCGA−BR−8680,TCGA−BR−6452,TCGA−CD−A4MG,TCGA−BR−8485,TCGA−CD−A4MI,TCGA−D7−5577,TCGA−BR−8078,TCGA−D7−6527,TCGA−BR−8382,TCGA−BR−7901

Supporting Material 3B:SMG enrichments in modules (NEB/PIK3CA)

	module id	mutation signature id	cohort	mutated.gene	comparison	DEG.ID	num.mutated	num.wildtype	Background	set1_size	set2_size	expected.overlap	actual.overlap	enrichment.foldchange	odds.ratio	FET_pvalue	corrected.FET.pvalue
1	M109	NEB__mutant_vs_wildtype__DN	TCGA−GCC	NEB	mutant_vs_wildtype	DN	26	187	26540	866	989	32.2710625	223	6.910216	11.273516967886	5.390552e−126	5.482130e−120
2	M116	NEB__mutant_vs_wildtype__DN	TCGA−GCC	NEB	mutant_vs_wildtype	DN	26	187	26540	455	989	16.9553504	151	8.905743	14.959989305776899	1.329752e−101	6.761714e−96
3	M633	NEB__mutant_vs_wildtype__DN	TCGA−GCC	NEB	mutant_vs_wildtype	DN	26	187	26540	387	989	14.4213640	129	8.945062	14.700513287721	9.016302e−87	1.833895e−81
4	M384	NEB__mutant_vs_wildtype__DN	TCGA−GCC	NEB	mutant_vs_wildtype	DN	26	187	26540	442	989	16.4709118	134	8.135554	12.8430440396834	3.890013e−84	6.593498e−79
5	M111	NEB__mutant_vs_wildtype__DN	TCGA−GCC	NEB	mutant_vs_wildtype	DN	26	187	26540	787	989	29.3271665	172	5.864869	8.5345770672724104	1.521651e−83	2.443423e−78
6	M399	NEB__mutant_vs_wildtype__DN	TCGA−GCC	NEB	mutant_vs_wildtype	DN	26	187	26540	644	989	23.9983421	149	6.208762	8.97688004798432	1.227786e−75	1.628666e−70
7	M635	NEB__mutant_vs_wildtype__DN	TCGA−GCC	NEB	mutant_vs_wildtype	DN	26	187	26540	499	989	18.5949887	127	6.829797	9.9705236698733195	1.577531e−69	1.552578e−64
8	M112	NEB__mutant_vs_wildtype__DN	TCGA−GCC	NEB	mutant_vs_wildtype	DN	26	187	26540	604	989	22.5077619	119	5.287065	7.0665818940304304	4.105862e−52	1.957320e−47
9	M729	NEB__mutant_vs_wildtype__DN	TCGA−GCC	NEB	mutant_vs_wildtype	DN	26	187	26540	339	989	12.6326677	91	7.203546	10.3349850521869	6.830510e−52	3.110396e−47
10	M403	NEB__mutant_vs_wildtype__DN	TCGA−GCC	NEB	mutant_vs_wildtype	DN	26	187	26540	285	989	10.6203843	78	7.344367	10.4795397611738	3.176008e−45	9.989579e−41
11	M423	NEB__mutant_vs_wildtype__DN	TCGA−GCC	NEB	mutant_vs_wildtype	DN	26	187	26540	153	989	5.7014695	58	10.172816	16.687636633380102	7.691884e−43	1.939477e−38
12	M113	NEB__mutant_vs_wildtype__DN	TCGA−GCC	NEB	mutant_vs_wildtype	DN	26	187	26540	319	989	11.8873775	72	6.056845	8.0429643934902106	9.847683e−36	1.155575e−31
13	M424	NEB__mutant_vs_wildtype__DN	TCGA−GCC	NEB	mutant_vs_wildtype	DN	26	187	26540	112	989	4.1736247	45	10.781995	18.125373492831802	8.050711e−35	8.440703e−31
14	M409	NEB__mutant_vs_wildtype__DN	TCGA−GCC	NEB	mutant_vs_wildtype	DN	26	187	26540	118	989	4.3972118	41	9.324090	14.303378099744201	7.350447e−29	4.346117e−25
15	M386	NEB__mutant_vs_wildtype__DN	TCGA−GCC	NEB	mutant_vs_wildtype	DN	26	187	26540	131	989	4.8816503	42	8.603648	12.6814513048863	6.504945e−28	3.556696e−24
16	M640	NEB__mutant_vs_wildtype__DN	TCGA−GCC	NEB	mutant_vs_wildtype	DN	26	187	26540	85	989	3.1674830	35	11.049783	18.705188833975001	8.309353e−28	4.527063e−24
17	M26	NEB__mutant_vs_wildtype__UP	TCGA−GCC	NEB	mutant_vs_wildtype	UP	26	187	26540	821	580	17.9419744	65	3.622790	4.2073491210954597	2.761216e−19	6.854660e−16
18	M226	NEB__mutant_vs_wildtype__UP	TCGA−GCC	NEB	mutant_vs_wildtype	UP	26	187	26540	223	580	4.8733986	32	6.566259	7.8771595321437804	3.333702e−17	6.687056e−14
19	M13	NEB__mutant_vs_wildtype__UP	TCGA−GCC	NEB	mutant_vs_wildtype	UP	26	187	26540	104	580	2.2727958	23	10.119695	13.1853214715501	5.534589e−17	1.088005e−13
20	M421	NEB__mutant_vs_wildtype__DN	TCGA−GCC	NEB	mutant_vs_wildtype	DN	26	187	26540	96	989	3.5773926	27	7.547396	10.363679360218301	8.599854e−17	1.649143e−13
21	M387	NEB__mutant_vs_wildtype__DN	TCGA−GCC	NEB	mutant_vs_wildtype	DN	26	187	26540	50	989	1.8632253	20	10.734075	17.5520366273512	3.607359e−16	6.398797e−13
22	M137	NEB__mutant_vs_wildtype__UP	TCGA−GCC	NEB	mutant_vs_wildtype	UP	26	187	26540	243	580	5.3104748	32	6.025827	7.1233581886602897	4.242675e−16	7.477908e−13
23	M163	NEB__mutant_vs_wildtype__UP	TCGA−GCC	NEB	mutant_vs_wildtype	UP	26	187	26540	24	580	0.5244913	13	24.785920	54.037107637824299	4.548118e−16	7.997783e−13
24	M28	NEB__mutant_vs_wildtype__UP	TCGA−GCC	NEB	mutant_vs_wildtype	UP	26	187	26540	244	580	5.3323286	32	6.001131	7.0895823259607198	4.784879e−16	8.394770e−13
25	M367	NEB__mutant_vs_wildtype__DN	TCGA−GCC	NEB	mutant_vs_wildtype	DN	26	187	26540	134	989	4.9934439	30	6.007878	7.65437369664504	1.610319e−15	2.692070e−12
26	M656	NEB__mutant_vs_wildtype__DN	TCGA−GCC	NEB	mutant_vs_wildtype	DN	26	187	26540	55	989	2.0495479	20	9.758250	15.041868492388801	3.239204e−15	5.193222e−12
27	M211	NEB__mutant_vs_wildtype__UP	TCGA−GCC	NEB	mutant_vs_wildtype	UP	26	187	26540	173	580	3.7807084	26	6.877018	8.2394390552120793	1.028487e−14	1.545754e−11
28	M104	NEB__mutant_vs_wildtype__DN	TCGA−GCC	NEB	mutant_vs_wildtype	DN	26	187	26540	244	989	9.0925396	38	4.179250	4.9160509121180702	7.986523e−14	1.084406e−10
29	M126	NEB__mutant_vs_wildtype__UP	TCGA−GCC	NEB	mutant_vs_wildtype	UP	26	187	26540	246	580	5.3760362	28	5.208298	5.9886839014078896	1.172393e−12	1.361084e−09
30	M727	NEB__mutant_vs_wildtype__DN	TCGA−GCC	NEB	mutant_vs_wildtype	DN	26	187	26540	110	989	4.0990957	24	5.854950	7.3615292108890102	1.952760e−12	2.212330e−09
31	M476	NEB__mutant_vs_wildtype__UP	TCGA−GCC	NEB	mutant_vs_wildtype	UP	26	187	26540	60	580	1.3112283	15	11.439655	15.2866341044934	2.225357e−12	2.494302e−09
32	M442	NEB__mutant_vs_wildtype__UP	TCGA−GCC	NEB	mutant_vs_wildtype	UP	26	187	26540	61	580	1.3330821	15	11.252120	14.943852807102701	2.892216e−12	3.177549e−09
33	M400	NEB__mutant_vs_wildtype__DN	TCGA−GCC	NEB	mutant_vs_wildtype	DN	26	187	26540	112	989	4.1736247	23	5.510797	6.8104333079166501	2.116996e−11	2.028544e−08
34	M410	NEB__mutant_vs_wildtype__DN	TCGA−GCC	NEB	mutant_vs_wildtype	DN	26	187	26540	172	989	6.4094951	28	4.368519	5.1404399041015703	5.126647e−11	4.608493e−08
35	M634	NEB__mutant_vs_wildtype__DN	TCGA−GCC	NEB	mutant_vs_wildtype	DN	26	187	26540	100	989	3.7264506	21	5.635389	6.9935754721081898	1.007440e−10	8.633889e−08
36	M388	NEB__mutant_vs_wildtype__DN	TCGA−GCC	NEB	mutant_vs_wildtype	DN	26	187	26540	34	989	1.2669932	13	10.260513	16.187149097140299	1.108442e−10	9.456989e−08
37	M610	NEB__mutant_vs_wildtype__UP	TCGA−GCC	NEB	mutant_vs_wildtype	UP	26	187	26540	46	580	1.0052751	12	11.937031	16.1059498997238	2.096022e−10	1.716289e−07
38	M414	NEB__mutant_vs_wildtype__DN	TCGA−GCC	NEB	mutant_vs_wildtype	DN	26	187	26540	43	989	1.6023738	14	8.737038	12.630999039419001	2.599188e−10	2.086851e−07
39	M764	NEB__mutant_vs_wildtype__DN	TCGA−GCC	NEB	mutant_vs_wildtype	DN	26	187	26540	67	989	2.4967219	17	6.808928	8.9184182532654095	2.622214e−10	2.103677e−07
40	M672	NEB__mutant_vs_wildtype__UP	TCGA−GCC	NEB	mutant_vs_wildtype	UP	26	187	26540	51	580	1.1145441	12	10.766734	14.030895775597299	7.741879e−10	5.715028e−07
41	M78	NEB__mutant_vs_wildtype__UP	TCGA−GCC	NEB	mutant_vs_wildtype	UP	26	187	26540	73	580	1.5953278	13	8.148795	9.8941763425745002	5.901035e−09	3.709841e−06
42	M122	NEB__mutant_vs_wildtype__UP	TCGA−GCC	NEB	mutant_vs_wildtype	UP	26	187	26540	134	580	2.9284099	17	5.805198	6.6682609038924099	6.222958e−09	3.890580e−06
43	M332	NEB__mutant_vs_wildtype__UP	TCGA−GCC	NEB	mutant_vs_wildtype	UP	26	187	26540	61	580	1.3330821	12	9.001696	11.1700053834081	6.963736e−09	4.317440e−06
44	M456	NEB__mutant_vs_wildtype__UP	TCGA−GCC	NEB	mutant_vs_wildtype	UP	26	187	26540	106	580	2.3165034	15	6.475277	7.5467175401415902	1.075925e−08	6.426827e−06
45	M425	NEB__mutant_vs_wildtype__DN	TCGA−GCC	NEB	mutant_vs_wildtype	DN	26	187	26540	42	989	1.5651093	12	7.667196	10.4478299123006	2.641187e−08	1.448009e−05
46	M722	NEB__mutant_vs_wildtype__UP	TCGA−GCC	NEB	mutant_vs_wildtype	UP	26	187	26540	17	580	0.3715147	7	18.841785	31.662544759221699	3.692984e−08	1.965318e−05
47	M469	NEB__mutant_vs_wildtype__UP	TCGA−GCC	NEB	mutant_vs_wildtype	UP	26	187	26540	316	580	6.9058026	25	3.620144	3.9730288467110602	3.746546e−08	1.991044e−05
48	M740	NEB__mutant_vs_wildtype__DN	TCGA−GCC	NEB	mutant_vs_wildtype	DN	26	187	26540	24	989	0.8943482	9	10.063195	15.6287895716848	1.054612e−07	5.057505e−05
49	M114	NEB__mutant_vs_wildtype__DN	TCGA−GCC	NEB	mutant_vs_wildtype	DN	26	187	26540	112	989	4.1736247	18	4.312798	5.0196801708898304	1.718149e−07	7.797137e−05
50	M728	NEB__mutant_vs_wildtype__DN	TCGA−GCC	NEB	mutant_vs_wildtype	DN	26	187	26540	14	989	0.5217031	7	13.417594	25.999761758786399	2.665575e−07	1.155360e−04

	module id	mutation signature id	cohort	mutated.gene	comparison	DEG.ID	num.mutated	num.wildtype	Background	set1_size	set2_size	expected.overlap	actual.overlap	enrichment.foldchange	odds.ratio	FET_pvalue	corrected.FET.pvalue
1	M475	NEB__mutant_vs_wildtype__UP	TCGA−GCC	NEB	mutant_vs_wildtype	UP	26	187	26540	154	580	3.3654861	16	4.754142	5.3070933790110297	2.918030e−07	1.251613e−04
2	M458	NEB__mutant_vs_wildtype__UP	TCGA−GCC	NEB	mutant_vs_wildtype	UP	26	187	26540	101	580	2.2072344	13	5.889723	6.7394044610959201	3.194893e−07	1.355139e−04
3	M131	NEB__mutant_vs_wildtype__UP	TCGA−GCC	NEB	mutant_vs_wildtype	UP	26	187	26540	384	580	8.3918613	26	3.098240	3.35595626578609	4.321577e−07	1.771699e−04
4	M684	NEB__mutant_vs_wildtype__UP	TCGA−GCC	NEB	mutant_vs_wildtype	UP	26	187	26540	47	580	1.0271289	9	8.762289	10.7500681622176	6.948183e−07	2.689501e−04
5	M422	NEB__mutant_vs_wildtype__DN	TCGA−GCC	NEB	mutant_vs_wildtype	DN	26	187	26540	30	989	1.1179352	9	8.050556	11.160817350872399	9.432337e−07	3.518061e−04
6	M14	NEB__mutant_vs_wildtype__UP	TCGA−GCC	NEB	mutant_vs_wildtype	UP	26	187	26540	280	580	6.1190656	21	3.431897	3.72773845780881	1.097076e−06	4.019144e−04
7	M177	NEB__mutant_vs_wildtype__UP	TCGA−GCC	NEB	mutant_vs_wildtype	UP	26	187	26540	28	580	0.6119066	7	11.439655	15.0769748317986	1.824398e−06	6.291599e−04
8	M534	NEB__mutant_vs_wildtype__UP	TCGA−GCC	NEB	mutant_vs_wildtype	UP	26	187	26540	186	580	4.0648078	16	3.936225	4.3031355402095004	3.632568e−06	1.149911e−03
9	M165	NEB__mutant_vs_wildtype__UP	TCGA−GCC	NEB	mutant_vs_wildtype	UP	26	187	26540	21	580	0.4589299	6	13.073892	18.073005936827599	4.355546e−06	1.345002e−03
10	M284	NEB__mutant_vs_wildtype__UP	TCGA−GCC	NEB	mutant_vs_wildtype	UP	26	187	26540	13	580	0.2840995	5	17.599469	28.187735913048801	5.453414e−06	1.634238e−03
11	M667	NEB__mutant_vs_wildtype__UP	TCGA−GCC	NEB	mutant_vs_wildtype	UP	26	187	26540	60	580	1.3112283	9	6.863793	8.0060279657824207	5.857371e−06	1.738727e−03
12	M581	NEB__mutant_vs_wildtype__UP	TCGA−GCC	NEB	mutant_vs_wildtype	UP	26	187	26540	22	580	0.4807837	6	12.479624	16.944228906685002	5.878447e−06	1.744032e−03
13	M52	NEB__mutant_vs_wildtype__UP	TCGA−GCC	NEB	mutant_vs_wildtype	UP	26	187	26540	33	580	0.7211756	7	9.706374	12.1809501921279	5.987390e−06	1.772666e−03
14	M121	NEB__mutant_vs_wildtype__UP	TCGA−GCC	NEB	mutant_vs_wildtype	UP	26	187	26540	194	580	4.2396383	16	3.773907	4.1085734287919502	6.243826e−06	1.838774e−03
15	M209	NEB__mutant_vs_wildtype__UP	TCGA−GCC	NEB	mutant_vs_wildtype	UP	26	187	26540	243	580	5.3104748	18	3.389527	3.6633710468567098	7.550026e−06	2.163508e−03
16	M648	NEB__mutant_vs_wildtype__DN	TCGA−GCC	NEB	mutant_vs_wildtype	DN	26	187	26540	30	989	1.1179352	8	7.156050	9.4609069595907194	1.018503e−05	2.807569e−03
17	M427	NEB__mutant_vs_wildtype__DN	TCGA−GCC	NEB	mutant_vs_wildtype	DN	26	187	26540	500	989	18.6322532	39	2.093145	2.2342311933321799	1.320344e−05	3.512972e−03
18	M642	NEB__mutant_vs_wildtype__DN	TCGA−GCC	NEB	mutant_vs_wildtype	DN	26	187	26540	52	989	1.9377543	10	5.160613	6.2034783562260198	1.880340e−05	4.730255e−03
19	M81	NEB__mutant_vs_wildtype__UP	TCGA−GCC	NEB	mutant_vs_wildtype	UP	26	187	26540	54	580	1.1801055	8	6.779055	7.8779373312737997	2.129345e−05	5.266769e−03
20	M156	NEB__mutant_vs_wildtype__UP	TCGA−GCC	NEB	mutant_vs_wildtype	UP	26	187	26540	17	580	0.3715147	5	13.458418	18.794690575445902	2.438575e−05	5.915097e−03
21	M402	NEB__mutant_vs_wildtype__DN	TCGA−GCC	NEB	mutant_vs_wildtype	DN	26	187	26540	25	989	0.9316127	7	7.513852	10.108936995407801	2.605204e−05	6.252862e−03
22	M400	NEB__mutant_vs_wildtype__UP	TCGA−GCC	NEB	mutant_vs_wildtype	UP	26	187	26540	112	580	2.4476262	11	4.494150	4.9488630942659402	3.535960e−05	8.083392e−03
23	M721	NEB__mutant_vs_wildtype__UP	TCGA−GCC	NEB	mutant_vs_wildtype	UP	26	187	26540	10	580	0.2185381	4	18.303448	30.011190949114798	4.269321e−05	9.493151e−03
24	M676	NEB__mutant_vs_wildtype__UP	TCGA−GCC	NEB	mutant_vs_wildtype	UP	26	187	26540	20	580	0.4370761	5	11.439655	15.027992110776699	5.786882e−05	1.220994e−02
25	M750	NEB__mutant_vs_wildtype__UP	TCGA−GCC	NEB	mutant_vs_wildtype	UP	26	187	26540	20	580	0.4370761	5	11.439655	15.027992110776699	5.786882e−05	1.220994e−02
26	M404	NEB__mutant_vs_wildtype__DN	TCGA−GCC	NEB	mutant_vs_wildtype	DN	26	187	26540	61	989	2.2731349	10	4.399211	5.1065722782901899	7.917063e−05	1.584641e−02
27	M578	NEB__mutant_vs_wildtype__UP	TCGA−GCC	NEB	mutant_vs_wildtype	UP	26	187	26540	24	580	0.5244913	5	9.533046	11.8669664445039	1.475851e−04	2.637518e−02
28	M168	NEB__mutant_vs_wildtype__UP	TCGA−GCC	NEB	mutant_vs_wildtype	UP	26	187	26540	90	580	1.9668425	9	4.575862	5.0350570954149596	1.567510e−04	2.777247e−02
29	M438	NEB__mutant_vs_wildtype__UP	TCGA−GCC	NEB	mutant_vs_wildtype	UP	26	187	26540	112	580	2.4476262	10	4.085591	4.4468905754270196	1.769127e−04	3.069820e−02
30	M680	NEB__mutant_vs_wildtype__DN	TCGA−GCC	NEB	mutant_vs_wildtype	DN	26	187	26540	80	989	2.9811605	11	3.689838	4.1533242151612502	1.817609e−04	3.136042e−02
31	M497	NEB__mutant_vs_wildtype__UP	TCGA−GCC	NEB	mutant_vs_wildtype	UP	26	187	26540	14	580	0.3059533	4	13.073892	18.013804055383801	1.898047e−04	3.247827e−02
32	M243	NEB__mutant_vs_wildtype__UP	TCGA−GCC	NEB	mutant_vs_wildtype	UP	26	187	26540	76	580	1.6608892	8	4.816697	5.3241460578014399	2.535501e−04	4.119130e−02
33	M659	NEB__mutant_vs_wildtype__DN	TCGA−GCC	NEB	mutant_vs_wildtype	DN	26	187	26540	479	989	17.8496986	34	1.904794	2.00852662762951	2.807548e−04	4.469933e−02
34	M293	NEB__mutant_vs_wildtype__UP	TCGA−GCC	NEB	mutant_vs_wildtype	UP	26	187	26540	28	580	0.6119066	5	8.171182	9.8043943193619896	3.175101e−04	4.944176e−02
35	M6	PIK3CA__mutant_vs_wildtype__UP	TCGA−GCC	PIK3CA	mutant_vs_wildtype	UP	37	176	26540	2276	747	64.0607385	347	5.416734	10.7301467930907	8.169669e−173	2.492538e−166
36	M102	PIK3CA__mutant_vs_wildtype__UP	TCGA−GCC	PIK3CA	mutant_vs_wildtype	UP	37	176	26540	322	747	9.0630746	130	14.343918	28.090791448486101	2.478834e−116	1.890710e−110
37	M357	PIK3CA__mutant_vs_wildtype__UP	TCGA−GCC	PIK3CA	mutant_vs_wildtype	UP	37	176	26540	138	747	3.8841748	68	17.506936	36.767393092509401	6.600084e−68	6.292697e−63
38	M355	PIK3CA__mutant_vs_wildtype__UP	TCGA−GCC	PIK3CA	mutant_vs_wildtype	UP	37	176	26540	183	747	5.1507536	65	12.619513	20.728267552822299	1.504959e−53	7.527178e−49
39	M105	PIK3CA__mutant_vs_wildtype__UP	TCGA−GCC	PIK3CA	mutant_vs_wildtype	UP	37	176	26540	328	747	9.2319518	77	8.340598	11.6914855058012	2.911988e−48	1.070407e−43
40	M101	PIK3CA__mutant_vs_wildtype__UP	TCGA−GCC	PIK3CA	mutant_vs_wildtype	UP	37	176	26540	236	747	6.6425019	66	9.936015	14.5998735749857	1.033739e−46	3.504335e−42
41	M112	PIK3CA__mutant_vs_wildtype__DN	TCGA−GCC	PIK3CA	mutant_vs_wildtype	DN	37	176	26540	604	631	14.3603617	84	5.849435	7.4971363015034402	7.775272e−40	1.560664e−35
42	M109	PIK3CA__mutant_vs_wildtype__DN	TCGA−GCC	PIK3CA	mutant_vs_wildtype	DN	37	176	26540	866	631	20.5895252	99	4.808270	6.0990771798146799	2.030156e−39	3.776791e−35
43	M26	PIK3CA__mutant_vs_wildtype__UP	TCGA−GCC	PIK3CA	mutant_vs_wildtype	UP	37	176	26540	821	747	23.1080256	96	4.154401	5.09749772978072	7.084702e−33	6.054673e−29
44	M116	PIK3CA__mutant_vs_wildtype__DN	TCGA−GCC	PIK3CA	mutant_vs_wildtype	DN	37	176	26540	455	631	10.8178222	66	6.101043	7.6626465403224104	1.805784e−32	1.461376e−28
45	M358	PIK3CA__mutant_vs_wildtype__UP	TCGA−GCC	PIK3CA	mutant_vs_wildtype	UP	37	176	26540	65	747	1.8295026	32	17.491093	34.910772410985203	1.881911e−32	1.514947e−28
46	M620	PIK3CA__mutant_vs_wildtype__UP	TCGA−GCC	PIK3CA	mutant_vs_wildtype	UP	37	176	26540	117	747	3.2931047	39	11.842927	18.154127950831398	2.763946e−31	2.036885e−27
47	M211	PIK3CA__mutant_vs_wildtype__UP	TCGA−GCC	PIK3CA	mutant_vs_wildtype	UP	37	176	26540	173	747	4.8692916	41	8.420116	11.2827318719674	2.726702e−26	1.273978e−22
48	M423	PIK3CA__mutant_vs_wildtype__DN	TCGA−GCC	PIK3CA	mutant_vs_wildtype	DN	37	176	26540	153	631	3.6376413	36	9.896523	13.3313989611345	1.313943e−25	5.662139e−22
49	M106	PIK3CA__mutant_vs_wildtype__UP	TCGA−GCC	PIK3CA	mutant_vs_wildtype	UP	37	176	26540	549	747	15.4522607	67	4.335935	5.1732964735058502	3.805363e−24	1.433337e−20
50	M633	PIK3CA__mutant_vs_wildtype__DN	TCGA−GCC	PIK3CA	mutant_vs_wildtype	DN	37	176	26540	387	631	9.2010927	48	5.216772	6.2091151571269796	7.045391e−21	1.979305e−17

	module id	mutation signature id	cohort	mutated.gene	comparison	DEG.ID	num.mutated	num.wildtype	Background	set1_size	set2_size	expected.overlap	actual.overlap	enrichment.foldchange	odds.ratio	FET_pvalue	corrected.FET.pvalue
1	M388	PIK3CA__mutant_vs_wildtype__DN	TCGA−GCC	PIK3CA	mutant_vs_wildtype	DN	37	176	26540	34	631	0.8083647	18	22.267176	47.480545782407297	7.176583e−21	2.014306e−17
2	M384	PIK3CA__mutant_vs_wildtype__DN	TCGA−GCC	PIK3CA	mutant_vs_wildtype	DN	37	176	26540	442	631	10.5087415	51	4.853103	5.7377945687867298	1.040529e−20	2.891274e−17
3	M378	PIK3CA__mutant_vs_wildtype__UP	TCGA−GCC	PIK3CA	mutant_vs_wildtype	UP	37	176	26540	464	747	13.0598342	54	4.134815	4.8237188528869197	1.032920e−18	2.446743e−15
4	M154	PIK3CA__mutant_vs_wildtype__UP	TCGA−GCC	PIK3CA	mutant_vs_wildtype	UP	37	176	26540	11	747	0.3096081	11	35.528782	Inf	8.173152e−18	1.753587e−14
5	M114	PIK3CA__mutant_vs_wildtype__DN	TCGA−GCC	PIK3CA	mutant_vs_wildtype	DN	37	176	26540	112	631	2.6628485	24	9.012905	11.6004284791949	1.676868e−16	3.108182e−13
6	M414	PIK3CA__mutant_vs_wildtype__DN	TCGA−GCC	PIK3CA	mutant_vs_wildtype	DN	37	176	26540	43	631	1.0223436	15	14.672170	22.495872720593301	3.046798e−14	4.345805e−11
7	M14	PIK3CA__mutant_vs_wildtype__UP	TCGA−GCC	PIK3CA	mutant_vs_wildtype	UP	37	176	26540	280	747	7.8809344	35	4.441098	5.1252769033202599	1.740489e−13	2.259647e−10
8	M404	PIK3CA__mutant_vs_wildtype__DN	TCGA−GCC	PIK3CA	mutant_vs_wildtype	DN	37	176	26540	61	631	1.4503014	16	11.032189	14.9417394993597	6.501842e−13	7.819038e−10
9	M403	PIK3CA__mutant_vs_wildtype__DN	TCGA−GCC	PIK3CA	mutant_vs_wildtype	DN	37	176	26540	285	631	6.7759985	31	4.574972	5.2176727703365602	2.201959e−12	2.473528e−09
10	M168	PIK3CA__mutant_vs_wildtype__UP	TCGA−GCC	PIK3CA	mutant_vs_wildtype	UP	37	176	26540	90	747	2.5331575	19	7.500521	9.4527593072626797	6.094758e−12	6.382834e−09
11	M421	PIK3CA__mutant_vs_wildtype__DN	TCGA−GCC	PIK3CA	mutant_vs_wildtype	DN	37	176	26540	96	631	2.2824416	18	7.886292	9.7210347815924205	1.130937e−11	1.135390e−08
12	M640	PIK3CA__mutant_vs_wildtype__DN	TCGA−GCC	PIK3CA	mutant_vs_wildtype	DN	37	176	26540	85	631	2.0209118	17	8.412044	10.516956726400201	1.426386e−11	1.412481e−08
13	M656	PIK3CA__mutant_vs_wildtype__DN	TCGA−GCC	PIK3CA	mutant_vs_wildtype	DN	37	176	26540	55	631	1.3076488	14	10.706238	14.3057659748993	2.836349e−11	2.659375e−08
14	M680	PIK3CA__mutant_vs_wildtype__DN	TCGA−GCC	PIK3CA	mutant_vs_wildtype	DN	37	176	26540	80	631	1.9020347	16	8.412044	10.501579781922601	5.689279e−11	5.085789e−08
15	M210	PIK3CA__mutant_vs_wildtype__UP	TCGA−GCC	PIK3CA	mutant_vs_wildtype	UP	37	176	26540	59	747	1.6606255	15	9.032741	11.988145800196	6.059004e−11	5.381606e−08
16	M373	PIK3CA__mutant_vs_wildtype__UP	TCGA−GCC	PIK3CA	mutant_vs_wildtype	UP	37	176	26540	71	747	1.9983798	15	7.506081	9.4154325624816302	1.015523e−09	7.357700e−07
17	M740	PIK3CA__mutant_vs_wildtype__DN	TCGA−GCC	PIK3CA	mutant_vs_wildtype	DN	37	176	26540	24	631	0.5706104	9	15.772583	24.9467693692987	2.180510e−09	1.483645e−06
18	M642	PIK3CA__mutant_vs_wildtype__DN	TCGA−GCC	PIK3CA	mutant_vs_wildtype	DN	37	176	26540	52	631	1.2363225	12	9.706205	12.5329092283232	2.547295e−09	1.710962e−06
19	M399	PIK3CA__mutant_vs_wildtype__DN	TCGA−GCC	PIK3CA	mutant_vs_wildtype	DN	37	176	26540	644	631	15.3113791	42	2.743058	2.99739788752666	4.578815e−09	2.942251e−06
20	M427	PIK3CA__mutant_vs_wildtype__DN	TCGA−GCC	PIK3CA	mutant_vs_wildtype	DN	37	176	26540	500	631	11.8877167	36	3.028336	3.3176653517175199	4.709757e−09	3.018762e−06
21	M413	PIK3CA__mutant_vs_wildtype__DN	TCGA−GCC	PIK3CA	mutant_vs_wildtype	DN	37	176	26540	26	631	0.6181613	9	14.559308	22.026302450524199	4.993835e−09	3.187452e−06
22	M452	PIK3CA__mutant_vs_wildtype__DN	TCGA−GCC	PIK3CA	mutant_vs_wildtype	DN	37	176	26540	107	631	2.5439714	16	6.289379	7.3813040200839799	5.054955e−09	3.223766e−06
23	M627	PIK3CA__mutant_vs_wildtype__UP	TCGA−GCC	PIK3CA	mutant_vs_wildtype	UP	37	176	26540	135	747	3.7997362	19	5.000347	5.7766561835703198	8.461670e−09	5.161189e−06
24	M170	PIK3CA__mutant_vs_wildtype__UP	TCGA−GCC	PIK3CA	mutant_vs_wildtype	UP	37	176	26540	72	747	2.0265260	14	6.908374	8.4730109014750905	1.145743e−08	6.789879e−06
25	M386	PIK3CA__mutant_vs_wildtype__DN	TCGA−GCC	PIK3CA	mutant_vs_wildtype	DN	37	176	26540	131	631	3.1145818	17	5.458197	6.2637358717488301	1.518662e−08	8.795343e−06
26	M124	PIK3CA__mutant_vs_wildtype__DN	TCGA−GCC	PIK3CA	mutant_vs_wildtype	DN	37	176	26540	160	631	3.8040693	18	4.731775	5.3272141909661004	5.507104e−08	2.816290e−05
27	M111	PIK3CA__mutant_vs_wildtype__DN	TCGA−GCC	PIK3CA	mutant_vs_wildtype	DN	37	176	26540	787	631	18.7112660	45	2.404968	2.6044608197108601	6.575955e−08	3.320592e−05
28	M402	PIK3CA__mutant_vs_wildtype__DN	TCGA−GCC	PIK3CA	mutant_vs_wildtype	DN	37	176	26540	25	631	0.5943858	8	13.459271	19.548962447584799	7.392028e−08	3.682695e−05
29	M635	PIK3CA__mutant_vs_wildtype__DN	TCGA−GCC	PIK3CA	mutant_vs_wildtype	DN	37	176	26540	499	631	11.8639412	33	2.781538	3.01273104203915	1.535529e−07	7.068266e−05
30	M727	PIK3CA__mutant_vs_wildtype__DN	TCGA−GCC	PIK3CA	mutant_vs_wildtype	DN	37	176	26540	110	631	2.6152977	14	5.353119	6.1000647811085598	3.601141e−07	1.507127e−04
31	M64	PIK3CA__mutant_vs_wildtype__UP	TCGA−GCC	PIK3CA	mutant_vs_wildtype	UP	37	176	26540	171	747	4.8129992	19	3.947642	4.4020068610863099	3.923152e−07	1.624733e−04
32	M371	PIK3CA__mutant_vs_wildtype__UP	TCGA−GCC	PIK3CA	mutant_vs_wildtype	UP	37	176	26540	59	747	1.6606255	11	6.624010	8.0152521787070903	6.673746e−07	2.593805e−04
33	M117	PIK3CA__mutant_vs_wildtype__DN	TCGA−GCC	PIK3CA	mutant_vs_wildtype	DN	37	176	26540	638	631	15.1687265	37	2.439229	2.6228471029975	7.074824e−07	2.730634e−04
34	M374	PIK3CA__mutant_vs_wildtype__UP	TCGA−GCC	PIK3CA	mutant_vs_wildtype	UP	37	176	26540	30	747	0.8443858	8	9.474342	12.6768828427898	1.282536e−06	4.621440e−04
35	M659	PIK3CA__mutant_vs_wildtype__DN	TCGA−GCC	PIK3CA	mutant_vs_wildtype	DN	37	176	26540	479	631	11.3884326	29	2.546443	2.7252196990739601	4.968543e−06	1.506406e−03
36	M185	PIK3CA__mutant_vs_wildtype__UP	TCGA−GCC	PIK3CA	mutant_vs_wildtype	UP	37	176	26540	225	747	6.3328937	20	3.158114	3.4335171781859901	6.507529e−06	1.906496e−03
37	M10	PIK3CA__mutant_vs_wildtype__UP	TCGA−GCC	PIK3CA	mutant_vs_wildtype	UP	37	176	26540	142	747	3.9967596	15	3.753040	4.1408445204824904	1.198530e−05	3.233993e−03
38	M700	PIK3CA__mutant_vs_wildtype__UP	TCGA−GCC	PIK3CA	mutant_vs_wildtype	UP	37	176	26540	180	747	5.0663150	17	3.355496	3.6610521886396499	1.436974e−05	3.773592e−03
39	M290	PIK3CA__mutant_vs_wildtype__UP	TCGA−GCC	PIK3CA	mutant_vs_wildtype	UP	37	176	26540	42	747	1.1821402	8	6.767387	8.2003341258519793	1.919269e−05	4.813503e−03
40	M209	PIK3CA__mutant_vs_wildtype__UP	TCGA−GCC	PIK3CA	mutant_vs_wildtype	UP	37	176	26540	243	747	6.8395252	20	2.924180	3.1541535469265498	2.022376e−05	5.031969e−03
41	M513	PIK3CA__mutant_vs_wildtype__UP	TCGA−GCC	PIK3CA	mutant_vs_wildtype	UP	37	176	26540	188	747	5.2914846	17	3.212709	3.48902214965113	2.528851e−05	6.095789e−03
42	M606	PIK3CA__mutant_vs_wildtype__UP	TCGA−GCC	PIK3CA	mutant_vs_wildtype	UP	37	176	26540	134	747	3.7715901	14	3.711962	4.08578619222531	2.631518e−05	6.303919e−03
43	M579	PIK3CA__mutant_vs_wildtype__DN	TCGA−GCC	PIK3CA	mutant_vs_wildtype	DN	37	176	26540	18	631	0.4279578	5	11.683395	15.9073004320327	4.955180e−05	1.074949e−02
44	M424	PIK3CA__mutant_vs_wildtype__DN	TCGA−GCC	PIK3CA	mutant_vs_wildtype	DN	37	176	26540	112	631	2.6628485	11	4.130915	4.5326708082580103	7.530086e−05	1.520352e−02
45	M311	PIK3CA__mutant_vs_wildtype__UP	TCGA−GCC	PIK3CA	mutant_vs_wildtype	UP	37	176	26540	148	747	4.1656368	14	3.360831	3.6566242262764002	7.899377e−05	1.581932e−02
46	M534	PIK3CA__mutant_vs_wildtype__UP	TCGA−GCC	PIK3CA	mutant_vs_wildtype	UP	37	176	26540	186	747	5.2351922	16	3.056239	3.2987155144709401	7.900304e−05	1.582014e−02
47	M20	PIK3CA__mutant_vs_wildtype__UP	TCGA−GCC	PIK3CA	mutant_vs_wildtype	UP	37	176	26540	496	747	13.9605124	30	2.148918	2.2737991590750699	8.316756e−05	1.648742e−02
48	M6	TP53__mutant_vs_wildtype__DN	TCGA−GCC	TP53	mutant_vs_wildtype	DN	71	142	26540	2276	219	18.7808591	168	8.945278	37.820004177451501	7.283606e−134	1.111102e−127
49	M102	TP53__mutant_vs_wildtype__DN	TCGA−GCC	TP53	mutant_vs_wildtype	DN	71	142	26540	322	219	2.6570460	56	21.076037	33.627672191515003	4.756236e−58	3.154590e−53
50	M357	TP53__mutant_vs_wildtype__DN	TCGA−GCC	TP53	mutant_vs_wildtype	DN	71	142	26540	138	219	1.1387340	32	28.101383	42.266519400378897	2.223445e−37	3.245768e−33

	module id	mutation signature id	cohort	mutated.gene	comparison	DEG.ID	num.mutated	num.wildtype	Background	set1_size	set2_size	expected.overlap	actual.overlap	enrichment.foldchange	odds.ratio	FET_pvalue	corrected.FET.pvalue
1	M378	TP53__mutant_vs_wildtype__DN	TCGA−GCC	TP53	mutant_vs_wildtype	DN	71	142	26540	464	219	3.82878674	45	11.753070	15.9826871121568	8.424652e−35	8.802509e−31
2	M105	TP53__mutant_vs_wildtype__DN	TCGA−GCC	TP53	mutant_vs_wildtype	DN	71	142	26540	328	219	2.70655614	39	14.409455	19.504255160965901	1.389736e−33	1.300625e−29
3	M106	TP53__mutant_vs_wildtype__DN	TCGA−GCC	TP53	mutant_vs_wildtype	DN	71	142	26540	549	219	4.53018086	46	10.154120	13.6378780982413	9.798406e−33	8.101519e−29
4	M373	TP53__mutant_vs_wildtype__DN	TCGA−GCC	TP53	mutant_vs_wildtype	DN	71	142	26540	71	219	0.58587038	22	37.550968	59.791627691439103	4.333316e−29	2.617980e−25
5	M371	TP53__mutant_vs_wildtype__DN	TCGA−GCC	TP53	mutant_vs_wildtype	DN	71	142	26540	59	219	0.48685004	11	22.594226	28.9316888586083	1.856410e−12	2.107085e−09
6	M104	TP53__mutant_vs_wildtype__DN	TCGA−GCC	TP53	mutant_vs_wildtype	DN	71	142	26540	244	219	2.01341372	18	8.940040	10.3365781439313	2.331568e−12	2.602830e−09
7	M367	TP53__mutant_vs_wildtype__DN	TCGA−GCC	TP53	mutant_vs_wildtype	DN	71	142	26540	134	219	1.10572720	13	11.756969	13.663624906633601	9.450133e−11	8.128569e−08
8	M358	TP53__mutant_vs_wildtype__DN	TCGA−GCC	TP53	mutant_vs_wildtype	DN	71	142	26540	65	219	0.53636021	10	18.644187	22.830091268705299	1.435294e−10	1.206013e−07
9	M77	TP53__mutant_vs_wildtype__UP	TCGA−GCC	TP53	mutant_vs_wildtype	UP	71	142	26540	60	134	0.30293896	8	26.407960	32.148707569543497	7.016499e−10	5.208540e−07
10	M227	TP53__mutant_vs_wildtype__UP	TCGA−GCC	TP53	mutant_vs_wildtype	UP	71	142	26540	12	134	0.06058779	5	82.524876	145.142061492067	2.343103e−09	1.586843e−06
11	M29	TP53__mutant_vs_wildtype__UP	TCGA−GCC	TP53	mutant_vs_wildtype	UP	71	142	26540	71	134	0.35847777	8	22.316586	26.539252229016501	2.785063e−09	1.856844e−06
12	M207	TP53__mutant_vs_wildtype__UP	TCGA−GCC	TP53	mutant_vs_wildtype	UP	71	142	26540	27	134	0.13632253	6	44.013267	58.770992654912398	4.015251e−09	2.605912e−06
13	M627	TP53__mutant_vs_wildtype__DN	TCGA−GCC	TP53	mutant_vs_wildtype	DN	71	142	26540	135	219	1.11397890	11	9.874514	11.1700202955536	1.717203e−08	9.816615e−06
14	M185	TP53__mutant_vs_wildtype__UP	TCGA−GCC	TP53	mutant_vs_wildtype	UP	71	142	26540	225	134	1.13602110	11	9.682919	10.945482882921	2.099211e−08	1.177320e−05
15	M700	TP53__mutant_vs_wildtype__UP	TCGA−GCC	TP53	mutant_vs_wildtype	UP	71	142	26540	180	134	0.90881688	10	11.003317	12.4410424011924	2.827960e−08	1.543196e−05
16	M513	TP53__mutant_vs_wildtype__UP	TCGA−GCC	TP53	mutant_vs_wildtype	UP	71	142	26540	188	134	0.94920874	10	10.535091	11.878503822106	4.269007e−08	2.239442e−05
17	M301	TP53__mutant_vs_wildtype__UP	TCGA−GCC	TP53	mutant_vs_wildtype	UP	71	142	26540	113	134	0.57053504	8	14.021926	15.8961163984391	1.121849e−07	5.343832e−05
18	M163	TP53__mutant_vs_wildtype__UP	TCGA−GCC	TP53	mutant_vs_wildtype	UP	71	142	26540	24	134	0.12117558	5	41.262438	53.7255687551771	1.197739e−07	5.648881e−05
19	M70	TP53__mutant_vs_wildtype__UP	TCGA−GCC	TP53	mutant_vs_wildtype	UP	71	142	26540	47	134	0.23730219	6	25.284217	30.120044722386599	1.340860e−07	6.248372e−05
20	M60	TP53__mutant_vs_wildtype__UP	TCGA−GCC	TP53	mutant_vs_wildtype	UP	71	142	26540	147	134	0.74220045	8	10.778759	11.9935761854332	8.461680e−07	3.191137e−04
21	M361	TP53__mutant_vs_wildtype__DN	TCGA−GCC	TP53	mutant_vs_wildtype	DN	71	142	26540	27	219	0.22279578	5	22.442077	27.890070470919699	2.544649e−06	8.446999e−04
22	M759	TP53__mutant_vs_wildtype__UP	TCGA−GCC	TP53	mutant_vs_wildtype	UP	71	142	26540	20	134	0.10097965	4	39.611940	50.646847939864898	2.827030e−06	9.240595e−04
23	M600	TP53__mutant_vs_wildtype__UP	TCGA−GCC	TP53	mutant_vs_wildtype	UP	71	142	26540	48	134	0.24235117	5	20.631219	23.729181382959499	4.378364e−06	1.351091e−03
24	M20	TP53__mutant_vs_wildtype__UP	TCGA−GCC	TP53	mutant_vs_wildtype	UP	71	142	26540	496	134	2.50429540	12	4.791767	5.26664750557297	8.525780e−06	2.408060e−03
25	M30	TP53__mutant_vs_wildtype__UP	TCGA−GCC	TP53	mutant_vs_wildtype	UP	71	142	26540	59	134	0.29788998	5	16.784720	18.904041670224601	1.224456e−05	3.290559e−03
26	M715	TP53__mutant_vs_wildtype__UP	TCGA−GCC	TP53	mutant_vs_wildtype	UP	71	142	26540	30	134	0.15146948	4	26.407960	31.192203607239801	1.537656e−05	3.995346e−03
27	M464	TP53__mutant_vs_wildtype__UP	TCGA−GCC	TP53	mutant_vs_wildtype	UP	71	142	26540	19	134	0.09593067	3	31.272584	37.663010657186803	1.149392e−04	2.149539e−02
28	M13	TP53__mutant_vs_wildtype__UP	TCGA−GCC	TP53	mutant_vs_wildtype	UP	71	142	26540	104	134	0.52509420	5	9.522101	10.293991296924499	1.875979e−04	3.217284e−02
29	M26	TP53__mutant_vs_wildtype__UP	TCGA−GCC	TP53	mutant_vs_wildtype	UP	71	142	26540	821	134	4.14521477	13	3.136146	3.4034050164151401	2.738634e−04	4.379870e−02
30	M413	TP53__mutant_vs_wildtype__UP	TCGA−GCC	TP53	mutant_vs_wildtype	UP	71	142	26540	26	134	0.13127355	3	22.853042	26.264486881509999	3.005306e−04	4.731944e−02

Supporting Material 4:GKN2 Knock−down signature

	gene.id	gene.name	AGSGKN2_2_count	AGSGKN2_1_count	AGScon_2_count	AGScon_1_count	AGSGKN2_2_FPKM	AGSGKN2_1_FPKM	AGScon_2_FPKM	AGScon_1_FPKM	AGSGKN2	AGScon	log2FC	Pvalue	Qvalue	updown
1	ENSG00000183607	GKN2	3755	3506	1	1	1.618244e+02	159.51837400	0.03909708	0.04143064	160.67139200	0.04026386	11.96233994	9.43e−211	2.21000e−206	UP
2	ENSG00000132967	HMGB1P5	3955	3498	373	391	2.089950e+02	195.15245770	17.88591898	19.85471224	202.07371330	18.87031561	3.4206912040000002	9.94e−67	1.16000e−62	UP
3	ENSG00000258941	RP11−407N17.3	9	11	171	228	1.761708e−01	0.22734531	3.03755576	4.28891223	0.20175807	3.66323399	−4.1824195959999999	1.90e−28	1.49000e−24	DN
4	ENSG00000267645	RP11−577H5.5	0	2	97	82	0.000000e+00	0.11358198	4.73570124	4.23945094	0.05679099	4.48757609	−6.3041305200000002	6.72e−26	3.94000e−22	DN
5	ENSG00000267368	UPK3BL	58	60	0	0	3.715780e+00	4.05828421	0.00000000	0.00000000	3.88703186	0.00000000	11.96233994	9.03e−24	4.23000e−20	UP
6	ENSG00000148677	ANKRD1	5388	3777	1103	1315	2.340770e+02	173.23791860	43.48305190	54.89775658	203.65744830	49.19040424	2.0496957629999999	1.64e−21	6.42000e−18	UP
7	ENSG00000280987	MATR3	679	811	3245	2673	4.031330e+00	5.08351406	17.48261885	15.25018646	4.55742218	16.36640266	−1.844447323	5.48e−21	1.84000e−17	DN
8	ENSG00000070669	ASNS	1539	1690	9069	5824	1.362688e+01	15.79825530	72.86692477	49.55379476	14.71256798	61.21035976	−2.0567267660000002	2.26e−20	6.61000e−17	DN
9	ENSG00000186480	INSIG1	1780	1524	535	575	1.895197e+01	17.13105889	5.16894022	5.88302193	18.04151354	5.52598108	1.707017848	9.71e−20	2.53000e−16	UP
10	ENSG00000015479	MATR3	4743	4712	1374	1783	1.625155e+01	17.04557830	4.27210380	5.87071173	16.64856334	5.07140777	1.7149395039999999	1.17e−17	2.74000e−14	UP
11	ENSG00000139211	AMIGO2	4470	3047	476	928	3.971401e+01	28.58073817	3.83757234	7.92285765	34.14737263	5.88021500	2.5378337690000001	4.84e−17	9.45000e−14	UP
12	ENSG00000218336	TENM3	1350	982	382	391	8.505605e+00	6.53202570	2.18397574	2.36725904	7.51881531	2.27561739	1.724247353	4.66e−17	9.45000e−14	UP
13	ENSG00000172575	RASGRP1	903	613	171	230	7.112153e+00	5.09727963	1.22214592	1.74075921	6.10471648	1.48145257	2.0429118590000002	8.94e−17	1.61000e−13	UP
14	ENSG00000113657	DPYSL3	4982	2906	1190	1033	3.604882e+01	22.19973543	7.81353887	7.18266555	29.12428011	7.49810221	1.957624993	3.03e−16	5.08000e−13	UP
15	ENSG00000258559	AC005519.4	34	35	0	0	2.138410e+00	2.32400471	0.00000000	0.00000000	2.23120738	0.00000000	11.96233994	3.82e−16	5.97000e−13	UP
16	ENSG00000052802	MSMO1	1863	1588	519	680	3.409391e+01	30.68167857	8.61875353	11.95833492	32.38779369	10.28854422	1.6544113300000001	8.27e−16	1.21000e−12	UP
17	ENSG00000281844	FBF1	0	0	47	30	0.000000e+00	0.00000000	1.07783322	0.72855438	0.00000000	0.90319380	NA	2.08e−15	2.87000e−12	NA
18	ENSG00000134531	EMP1	561	445	1660	1474	3.941532e+00	3.30085401	10.58335455	9.95170448	3.62119286	10.26752952	−1.5035521759999999	2.45e−15	3.19000e−12	DN
19	ENSG00000104419	NDRG1	368	358	1397	1016	1.930039e+00	1.98228619	6.64856859	5.12047182	1.95616263	5.88452020	−1.5888984690000001	6.51e−15	8.02000e−12	DN
20	ENSG00000186063	AIDA	276	227	810	775	3.896445e+00	3.38337826	10.37667212	10.51379107	3.63991145	10.44523160	−1.520869222	8.24e−15	9.65000e−12	DN
21	ENSG00000100292	HMOX1	2603	2185	1069	968	6.131381e+01	54.33756822	22.84942080	21.91075176	57.82568774	22.38008628	1.369494921	5.69e−14	6.35000e−11	UP
22	ENSG00000134852	CLOCK	1378	1424	3881	3850	6.593289e+00	7.19329242	16.85039338	17.70156476	6.89329060	17.27597907	−1.3255027319999999	9.33e−14	9.94000e−11	DN
23	ENSG00000133106	EPSTI1	667	582	261	243	7.868541e+00	7.24864016	2.79396954	2.75469472	7.55859039	2.77433213	1.4459766970000001	1.69e−13	1.72000e−10	UP
24	ENSG00000240224	UGT1A5	26	29	0	0	9.532572e−01	1.12252068	0.00000000	0.00000000	1.03788895	0.00000000	11.96233994	2.12e−13	2.07000e−10	UP
25	ENSG00000198848	CES1	5	9	65	67	7.544327e−02	0.14336700	0.88998798	0.97146599	0.10940514	0.93072699	−3.0886775430000002	5.47e−13	5.12000e−10	DN
26	ENSG00000102100	SLC35A2	359	473	143	141	1.827436e+00	2.54198669	0.66053526	0.68970726	2.18471118	0.67512126	1.6942240049999999	1.13e−12	1.02000e−09	UP
27	ENSG00000135069	PSAT1	2646	2620	7462	6314	8.545918e+01	89.33773971	218.69431450	195.96176840	87.39846138	207.32804140	−1.24623547	3.29e−12	2.85000e−09	DN
28	ENSG00000274049	INO80B−WBP1	0	0	27	25	0.000000e+00	0.00000000	0.71501301	0.70109905	0.00000000	0.70805603	NA	4.17e−12	3.49000e−09	NA
29	ENSG00000100889	PCK2	799	953	3609	2246	5.915846e+00	7.44951013	24.24767921	15.98003068	6.68267783	20.11385495	−1.5896913800000001	6.26e−12	5.05000e−09	DN
30	ENSG00000272405	RP11−284F21.10	179	183	728	482	4.776440e+00	5.15545082	17.62771409	12.35937211	4.96594538	14.99354310	−1.5942010470000001	1.06e−11	8.27000e−09	DN
31	ENSG00000109107	ALDOC	50	58	196	217	9.529501e−01	1.16706317	3.38979736	3.97431239	1.06000662	3.68205487	−1.7964378510000001	1.90e−11	1.44000e−08	DN
32	ENSG00000163993	S100P	259	393	2024	992	1.398730e+01	22.40736937	99.18766145	51.48059725	18.19733648	75.33412935	−2.04957631	2.72e−11	1.99000e−08	DN
33	ENSG00000228705	LINC00659	56	65	655	231	8.916005e+00	10.92596879	94.63199360	35.34218829	9.92098705	64.98709094	−2.7115976009999998	4.13e−11	2.93000e−08	DN
34	ENSG00000137819	PAQR5	72	53	327	206	5.077329e−01	0.39458795	2.09248690	1.39594356	0.45116042	1.74421523	−1.9508656520000001	6.03e−11	4.15000e−08	DN
35	ENSG00000113161	HMGCR	2753	2411	1039	1319	2.220790e+01	20.53351392	7.60554365	10.22454434	21.37070494	8.91504399	1.261320676	1.60e−10	1.07000e−07	UP
36	ENSG00000112972	HMGCS1	3979	3456	1733	1911	4.952204e+01	45.41121043	19.57206214	22.85510279	47.46662646	21.21358247	1.16192524	1.88e−10	1.23000e−07	UP
37	ENSG00000134324	LPIN1	1030	741	427	294	4.075437e+00	3.09542302	1.53312735	1.11784535	3.58543020	1.32548635	1.4356244170000001	2.11e−10	1.34000e−07	UP
38	ENSG00000104549	SQLE	1697	1423	649	792	2.535207e+01	22.44405998	8.79811117	11.36984223	23.89806720	10.08397670	1.2448292519999999	2.23e−10	1.38000e−07	UP
39	ENSG00000165828	PRAP1	42	43	158	151	1.620747e+00	1.75184620	5.53264366	5.59931816	1.68629672	5.56598091	−1.7227775439999999	4.12e−10	2.47000e−07	DN
40	ENSG00000116761	CTH	75	101	280	301	1.749382e+00	2.48717724	5.92642747	6.74660630	2.11827962	6.33651689	−1.580796981	5.69e−10	3.33000e−07	DN
41	ENSG00000102445	KIAA0226L	43	50	223	150	3.459295e−01	0.42467620	1.62794088	1.15960606	0.38530286	1.39377347	−1.8549313110000001	6.91e−10	3.95000e−07	DN
42	ENSG00000116741	RGS2	127	129	628	334	3.562451e+00	3.82033932	15.98524334	9.00307080	3.69139528	12.49415707	−1.759015435	8.75e−10	4.88000e−07	DN
43	ENSG00000151012	SLC7A11	1104	1068	2582	2434	9.621640e+00	9.82688792	20.41970431	20.38441645	9.72426388	20.40206038	−1.0690539080000001	1.10e−09	5.98000e−07	DN
44	ENSG00000178150	ZNF114	40	40	8	4	3.805052e−01	0.40172733	0.06905777	0.03656230	0.39111628	0.05281003	2.8887135979999998	1.17e−09	6.24000e−07	UP
45	ENSG00000148175	STOM	11	7	94	49	1.981481e−01	0.13312407	1.53647123	0.84816775	0.16563608	1.19231949	−2.8476819739999999	1.47e−09	7.63000e−07	DN
46	ENSG00000267426	RP11−552F3.12	32	42	2	7	4.993081e−01	0.69189031	0.02831168	0.10495994	0.59559923	0.06663581	3.1599723900000001	1.55e−09	7.91000e−07	UP
47	ENSG00000131797	CLUHP3	274	263	111	116	2.562538e+00	2.59681124	0.94201474	1.04250168	2.57967477	0.99225821	1.3784017	1.84e−09	9.15000e−07	UP
48	ENSG00000099337	KCNK6	14	20	130	71	1.941770e−01	0.29285462	1.63610674	0.94625961	0.24351583	1.29118317	−2.4066062339999998	3.85e−09	1.88000e−06	DN
49	ENSG00000151718	WWC2	1193	1043	521	607	4.577148e+00	4.22477337	1.81386848	2.23790303	4.40096085	2.02588576	1.119265717	4.49e−09	2.15000e−06	UP
50	ENSG00000067064	IDI1	1947	1695	637	965	3.256715e+01	29.93287084	9.66866024	15.51096591	31.25001012	12.58981307	1.3115997939999999	5.41e−09	2.53000e−06	UP
51	ENSG00000173926	43527	73	76	224	220	9.361879e−01	1.02901607	2.60678405	2.71121754	0.98260197	2.65900079	−1.4362051730000001	5.58e−09	2.56000e−06	DN
52	ENSG00000150995	ITPR1	26	28	111	96	1.249728e−01	0.14208717	0.48414723	0.44341148	0.13352998	0.46377935	−1.7962749119999999	7.16e−09	3.22000e−06	DN
53	ENSG00000142765	SYTL1	172	146	535	371	1.520917e+00	1.36299949	4.29283064	3.15245122	1.44195848	3.72264093	−1.3682968449999999	8.14e−09	3.60000e−06	DN
54	ENSG00000100031	GGT1	21	29	105	96	1.534386e−01	0.22370281	0.69616680	0.67402916	0.18857072	0.68509798	−1.861204614	8.48e−09	3.68000e−06	DN
55	ENSG00000122884	P4HA1	508	495	1253	1055	9.684199e+00	9.96254214	21.67528729	19.32638376	9.82337059	20.50083552	−1.061392677	8.76e−09	3.73000e−06	DN
56	ENSG00000006788	MYH13	18	35	135	105	2.312567e−01	0.47473318	1.57386378	1.29630235	0.35299494	1.43508307	−2.0234148260000002	9.35e−09	3.91000e−06	DN
57	ENSG00000187210	GCNT1	13	11	68	57	9.155423e−02	0.08179068	0.43456320	0.38574745	0.08667246	0.41015532	−2.2425248309999999	9.97e−09	4.10000e−06	DN
58	ENSG00000140297	GCNT3	281	449	1031	1024	2.261253e+00	3.81464339	7.52861663	7.91846479	3.03794804	7.72354071	−1.346165182	1.07e−08	4.32000e−06	DN
59	ENSG00000196689	TRPV1	33	32	124	109	3.275831e−01	0.33536690	1.11697914	1.03976315	0.33147500	1.07837114	−1.7018818339999999	1.10e−08	4.36000e−06	DN
60	ENSG00000204387	C6orf48	447	453	1187	925	9.036261e+00	9.66817527	21.77436997	17.96889198	9.35221826	19.87163097	−1.0873297829999999	1.71e−08	6.68000e−06	DN
61	ENSG00000147124	ZNF41	51	44	141	195	8.119939e−01	0.73961316	2.03711911	2.98342247	0.77580353	2.51027079	−1.69407975	2.19e−08	8.40000e−06	DN
62	ENSG00000183044	ABAT	186	123	29	55	1.104767e+00	0.77131168	0.15630418	0.31391916	0.93803921	0.23511167	1.996302077	2.22e−08	8.40000e−06	UP
63	ENSG00000121236	TRIM6	59	46	9	14	3.349125e−01	0.27567774	0.04635830	0.07636726	0.30529514	0.06136278	2.3147689480000002	2.62e−08	9.75000e−06	UP
64	ENSG00000100439	ABHD4	447	405	216	212	5.601402e+00	5.35808634	2.45616326	2.55283763	5.47974416	2.50450045	1.1295856689999999	2.86e−08	1.05000e−05	UP
65	ENSG00000170802	FOXN2	265	196	560	580	3.349542e+00	2.61553472	6.42305547	7.04475493	2.98253853	6.73390520	−1.174902635	2.96e−08	1.07000e−05	DN
66	ENSG00000102934	PLLP	413	480	227	211	3.591393e+00	4.40675194	1.79122851	1.76316020	3.99907223	1.77719435	1.1700638779999999	3.39e−08	1.20000e−05	UP
67	ENSG00000120885	CLU	284	266	597	747	2.218129e+00	2.19338549	4.23112922	5.60643811	2.20575734	4.91878366	−1.1570275160000001	3.56e−08	1.25000e−05	DN
68	ENSG00000237399	PITRM1−AS1	21	22	2	1	3.905500e−01	0.43195107	0.03374375	0.01787890	0.41125051	0.02581132	3.9939415980000001	3.70e−08	1.27000e−05	UP
69	ENSG00000244257	PKD1P1	34	91	402	241	4.295675e−01	1.21381813	4.60880136	2.92592312	0.82169280	3.76736224	−2.1968837369999998	5.04e−08	1.71000e−05	DN
70	ENSG00000162772	ATF3	836	578	331	353	7.401489e+00	5.40263516	2.65921865	3.00321024	6.40206220	2.83121445	1.177115664	5.39e−08	1.80000e−05	UP
71	ENSG00000272068	RP11−284F21.9	9	15	92	51	2.401435e−01	0.42256630	2.22767842	1.30773734	0.33135488	1.76770788	−2.415430797	6.12e−08	2.02000e−05	DN
72	ENSG00000130294	KIF1A	1	3	32	20	5.978266e−03	0.01892693	0.17353002	0.11484907	0.01245260	0.14418955	−3.5334479390000002	6.28e−08	2.04000e−05	DN
73	ENSG00000248713	RP11−766F14.2	0	0	18	13	0.000000e+00	0.00000000	0.13923117	0.10648467	0.00000000	0.12285792	NA	7.06e−08	2.26000e−05	NA
74	ENSG00000167617	CDC42EP5	37	56	215	136	2.761414e+00	4.41242879	14.56046885	9.75351692	3.58692146	12.15699288	−1.760968351	9.56e−08	3.03000e−05	DN
75	ENSG00000162244	RPL29	1913	2107	5744	3871	3.753353e+01	43.64493296	102.26625960	72.98366153	40.58923296	87.62496056	−1.110244813	1.08e−07	3.36000e−05	DN
76	ENSG00000229953	RP11−284F21.7	43	41	166	116	4.877241e+00	4.90977484	17.08558598	12.64342732	4.89350798	14.86450665	−1.6029306240000001	1.09e−07	3.36000e−05	DN
77	ENSG00000108797	CNTNAP1	29	40	125	110	3.285887e−01	0.47849389	1.28520756	1.19767522	0.40354127	1.24144139	−1.6212280100000001	1.11e−07	3.38000e−05	DN
78	ENSG00000169994	MYO7B	7	15	56	58	4.568851e−02	0.10336385	0.33168322	0.36379130	0.07452618	0.34773726	−2.2221784680000001	1.34e−07	4.02000e−05	DN
79	ENSG00000280780	JAKMIP2−AS1	30	17	2	2	7.074204e−01	0.42323206	0.04278589	0.04531679	0.56532621	0.04405134	3.6818257779999999	1.36e−07	4.05000e−05	UP
80	ENSG00000161888	SPC24	184	187	91	70	3.060469e+00	3.28379996	1.37349220	1.11884163	3.17213457	1.24616692	1.3479566540000001	1.45e−07	4.24000e−05	UP
81	ENSG00000139832	RAB20	682	573	369	293	3.862685e+01	34.26290701	18.96463645	15.94670006	36.44487622	17.45566826	1.0620204120000001	1.47e−07	4.26000e−05	UP
82	ENSG00000075223	SEMA3C	581	608	143	296	6.427178e+00	7.10088764	1.43546584	3.14654785	6.76403276	2.29100684	1.5619018790000001	1.57e−07	4.50000e−05	UP
83	ENSG00000197142	ACSL5	267	229	566	552	3.022065e+00	2.73647678	5.81328234	6.00384272	2.87927088	5.90856253	−1.0371036629999999	1.62e−07	4.56000e−05	DN
84	ENSG00000089225	TBX5	183	215	89	86	2.522212e+00	3.12849738	1.11309818	1.13901505	2.82535449	1.12605662	1.3271525200000001	1.64e−07	4.58000e−05	UP
85	ENSG00000116133	DHCR24	3760	3309	1220	2006	4.081171e+01	37.91913790	12.01628211	20.92308803	39.36542514	16.46968507	1.2571160910000001	1.70e−07	4.65000e−05	UP
86	ENSG00000149150	SLC43A1	27	44	191	109	4.043393e−01	0.69568212	2.59559400	1.56860977	0.55001073	2.08210188	−1.9205090039999999	1.71e−07	4.65000e−05	DN
87	ENSG00000023445	BIRC3	345	134	58	66	2.070335e+00	0.84896669	0.31583766	0.38059322	1.45965068	0.34821544	2.067571074	1.82e−07	4.91000e−05	UP
88	ENSG00000105516	DBP	46	29	165	110	3.931303e−01	0.26166113	1.27960220	0.90337570	0.32739574	1.09148895	−1.7371900689999999	1.90e−07	5.07000e−05	DN
89	ENSG00000172893	DHCR7	1272	1035	558	677	1.448112e+01	12.43999456	5.76451615	7.40630009	13.46055644	6.58540812	1.031393293	2.04e−07	5.38000e−05	UP
90	ENSG00000089472	HEPH	247	334	931	612	1.694543e+00	2.41917590	5.79588383	4.03464651	2.05685962	4.91526517	−1.256825917	2.33e−07	6.01000e−05	DN
91	ENSG00000095383	TBC1D2	843	832	2249	1563	9.453209e+00	9.85008094	22.88517490	16.84256940	9.65164516	19.86387215	−1.0413000990000001	2.32e−07	6.01000e−05	DN
92	ENSG00000088340	FER1L4	52	53	144	166	2.973579e−01	0.31997447	0.74721910	0.91217782	0.30866620	0.82969846	−1.426539593	2.39e−07	6.10000e−05	DN
93	ENSG00000008516	MMP25	1	2	32	15	1.770468e−02	0.03736188	0.51391034	0.25509044	0.02753328	0.38450039	−3.8037368040000001	3.93e−07	9.80000e−05	DN
94	ENSG00000079459	FDFT1	1515	1363	597	869	7.728805e+00	7.34108751	2.76367830	4.26007617	7.53494642	3.51187724	1.1013548390000001	3.99e−07	9.83000e−05	UP
95	ENSG00000187840	EIF4EBP1	549	620	1578	1106	4.398952e+01	52.44856069	114.73546000	85.15891934	48.21903953	99.94718967	−1.0515630869999999	4.26e−07	1.03927e−04	DN
96	ENSG00000174428	GTF2IRD2B	116	102	288	252	1.329056e+00	1.23381304	2.99426383	2.77448952	1.28143461	2.88437668	−1.1704997189999999	4.77e−07	1.15256e−04	DN
97	ENSG00000143772	ITPKB	176	154	62	80	1.662281e+00	1.53559832	0.53137187	0.72607026	1.59893983	0.62872107	1.3466236389999999	5.29e−07	1.26527e−04	UP
98	ENSG00000167508	MVD	399	365	198	209	3.510478e+00	3.39040307	1.58077909	1.76700162	3.45044071	1.67389035	1.043575616	5.56e−07	1.31477e−04	UP
99	ENSG00000270136	MINOS1−NBL1	17	17	74	58	5.314766e−01	0.56112876	2.09937975	1.74249417	0.54630266	1.92093696	−1.8140378159999999	6.51e−07	1.51011e−04	DN
100	ENSG00000104218	CSPP1	367	325	171	190	3.893535e+00	3.64020325	1.64621729	1.93700153	3.76686906	1.79160941	1.0721097390000001	7.01e−07	1.58352e−04	UP

	gene.id	gene.name	AGSGKN2_2_count	AGSGKN2_1_count	AGScon_2_count	AGScon_1_count	AGSGKN2_2_FPKM	AGSGKN2_1_FPKM	AGScon_2_FPKM	AGScon_1_FPKM	AGSGKN2	AGScon	log2FC	Pvalue	Qvalue	updown
1	ENSG00000169174	PCSK9	285	421	153	167	3.66594487	5.71725565	1.7858557	2.06422017	4.691600e+00	1.92503792	1.285193228	7.03e−07	0.000158352	UP
2	ENSG00000141738	GRB7	366	315	163	187	4.07606182	3.70369226	1.6472540	2.00124109	3.889877e+00	1.82424754	1.092423046	7.82e−07	0.000171199	UP
3	ENSG00000165376	CLDN2	349	352	189	187	4.82483305	5.13764498	2.3709970	2.48425490	4.981239e+00	2.42762596	1.0369584860000001	7.72e−07	0.000171199	UP
4	ENSG00000106384	MOGAT3	6	10	43	38	0.16294539	0.28670338	1.0596072	0.99163783	2.248244e−01	1.02562254	−2.1896294510000001	8.13e−07	0.000176335	DN
5	ENSG00000139725	RHOF	451	391	1373	776	5.10535496	4.67294642	14.1037037	8.44128486	4.889151e+00	11.27249430	−1.2051510050000001	8.44e−07	0.000178397	DN
6	ENSG00000175874	CREG2	433	447	220	254	4.81973084	5.25299663	2.2221399	2.71685583	5.036364e+00	2.46949784	1.0281647739999999	8.45e−07	0.000178397	UP
7	ENSG00000214736	TOMM6	134	129	26	58	10.22254435	10.38983142	1.7998489	4.25187130	1.030619e+01	3.02586008	1.768093618	8.36e−07	0.000178397	UP
8	ENSG00000105388	CEACAM5	463	555	1551	953	6.17256252	7.81164250	18.7633188	12.20887185	6.992103e+00	15.48609532	−1.1471751859999999	9.22e−07	0.000191206	DN
9	ENSG00000008300	CELSR3	103	107	279	236	0.49216604	0.53978429	1.2097378	1.08363730	5.159752e−01	1.14668757	−1.1520988320000001	9.38e−07	0.000192808	DN
10	ENSG00000167995	BEST1	2	0	75	10	0.01180050	0.00000000	0.4015987	0.05670614	5.900248e−03	0.22915242	−5.2793880729999998	1.07e−06	0.000215400	DN
11	ENSG00000197632	SERPINB2	2545	1543	5718	4256	52.29654278	33.47464354	106.6209949	84.03975778	4.288559e+01	95.33037632	−1.152442916	1.09e−06	0.000217902	DN
12	ENSG00000164251	F2RL1	1474	1778	4207	3031	35.99220127	45.83606635	93.2173588	71.12048538	4.091413e+01	82.16892206	−1.005993532	1.10e−06	0.000218145	DN
13	ENSG00000168916	ZNF608	125	102	42	51	0.69695070	0.60042367	0.2124979	0.27325095	6.486872e−01	0.24287444	1.4173123009999999	1.17e−06	0.000229859	UP
14	ENSG00000164099	PRSS12	118	159	441	302	1.58716690	2.25789248	5.3826056	3.90341755	1.922530e+00	4.64301158	−1.2720550020000001	1.18e−06	0.000230234	DN
15	ENSG00000090382	LYZ	3331	3973	6987	12521	110.48836390	139.13144600	210.3032702	399.09733460	1.248099e+02	304.70030240	−1.2876585	1.22e−06	0.000236524	DN
16	ENSG00000120437	ACAT2	578	480	240	314	9.98675321	8.75593980	3.7628800	5.21344020	9.371347e+00	4.48816012	1.0621322070000001	1.27e−06	0.000242278	UP
17	ENSG00000165949	IFI27	322	318	188	141	3.91518020	4.08213916	2.0742797	1.64745520	3.998660e+00	1.86086745	1.103541205	1.27e−06	0.000242278	UP
18	ENSG00000138678	GPAT3	159	147	502	304	2.30915310	2.25391054	6.6156448	4.24254561	2.281532e+00	5.42909520	−1.2507090089999999	1.33e−06	0.000245144	DN
19	ENSG00000179715	PCED1B	88	78	33	31	1.12739028	1.05498849	0.3836351	0.38164150	1.091189e+00	0.38263830	1.511848334	1.32e−06	0.000245144	UP
20	ENSG00000180340	FZD2	167	177	70	87	6.79829768	7.60713024	2.5858060	3.40328921	7.202714e+00	2.99454760	1.266202547	1.32e−06	0.000245144	UP
21	ENSG00000224259	LINC01133	123	138	344	282	3.07057103	3.63712113	7.7926502	6.76489878	3.353846e+00	7.27877447	−1.1178790830000001	1.30e−06	0.000245144	DN
22	ENSG00000198574	SH2D1B	3	3	29	22	0.08307328	0.08769782	0.7286644	0.58536509	8.538555e−02	0.65701476	−2.943861912	1.45e−06	0.000263615	DN
23	ENSG00000151715	TMEM45B	193	231	533	434	4.03339643	5.09671197	10.1077285	8.71566514	4.565054e+00	9.41169681	−1.043822858	1.49e−06	0.000269195	DN
24	ENSG00000166866	MYO1A	21	31	101	77	0.29329312	0.45709981	1.2800069	1.03340183	3.751965e−01	1.15670438	−1.6243020619999999	2.00e−06	0.000354109	DN
25	ENSG00000126562	WNK4	4	7	33	31	0.05281316	0.09758888	0.3954193	0.39336448	7.520102e−02	0.39439189	−2.3908058350000001	2.41e−06	0.000422078	DN
26	ENSG00000118322	ATP10B	70	89	189	244	0.38006608	0.51016914	0.9311797	1.27305248	4.451176e−01	1.10211609	−1.308017711	2.46e−06	0.000426663	DN
27	ENSG00000105855	ITGB8	37	23	120	83	0.17909859	0.11753786	0.5270850	0.38606557	1.483182e−01	0.45657530	−1.622156937	2.56e−06	0.000440559	DN
28	ENSG00000005448	WDR54	91	86	222	204	1.01095404	1.00868674	2.2379901	2.17780535	1.009820e+00	2.20789774	−1.1285746409999999	2.71e−06	0.000457293	DN
29	ENSG00000154263	ABCA10	8	5	38	31	0.05466251	0.03606499	0.2356072	0.20354416	4.536375e−02	0.21957570	−2.2751066569999998	2.69e−06	0.000457293	DN
30	ENSG00000146250	PRSS35	207	294	572	581	7.29385693	10.93700850	18.2892785	19.67257262	9.115433e+00	18.98092554	−1.058167294	2.77e−06	0.000462799	DN
31	ENSG00000258790	RP11−561B11.2	0	696	0	0	0.00000000	16.37952538	0.0000000	0.00000000	8.189763e+00	0.00000000	11.96233994	2.83e−06	0.000470875	UP
32	ENSG00000110921	MVK	255	189	69	114	1.82912026	1.43129201	0.4491259	0.78579116	1.630206e+00	0.61745855	1.4006401930000001	3.13e−06	0.000516831	UP
33	ENSG00000254166	CASC19	27	31	95	82	6.32510113	7.66731997	20.1952405	18.45951664	6.996211e+00	19.32737855	−1.46600036	3.19e−06	0.000522414	DN
34	ENSG00000276850	CH17−360D5.2	289	196	101	122	8.09086356	5.79318370	2.5658491	3.28212081	6.942024e+00	2.92398496	1.247420384	3.28e−06	0.000530223	UP
35	ENSG00000020181	ADGRA2	53	61	323	129	0.43924237	0.53373084	2.4291043	1.02734936	4.864866e−01	1.72822684	−1.828820613	3.33e−06	0.000532316	DN
36	ENSG00000168765	GSTM4	751	888	2434	1452	6.50231357	8.11718918	19.1232421	12.08070150	7.309751e+00	15.60197182	−1.0938341300000001	3.34e−06	0.000532316	DN
37	ENSG00000124466	LYPD3	71	86	201	192	1.59089659	2.03446095	4.0869029	4.13413745	1.812679e+00	4.11052020	−1.1811977010000001	3.49e−06	0.000552075	DN
38	ENSG00000272734	ADIRF−AS1	114	145	355	276	1.07046952	1.43748868	3.0249131	2.49044922	1.253979e+00	2.75768118	−1.1369423649999999	3.52e−06	0.000553963	DN
39	ENSG00000108960	MMD	260	258	103	148	6.27913237	6.57825522	2.2572464	3.43468678	6.428694e+00	2.84596661	1.175606897	4.14e−06	0.000642355	UP
40	ENSG00000137962	ARHGAP29	1844	1601	5181	2938	9.56440664	8.76704456	24.3850780	14.64356442	9.165726e+00	19.51432121	−1.09021228	4.49e−06	0.000688090	DN
41	ENSG00000186907	RTN4RL2	71	81	211	171	2.48342970	2.99118369	6.6971473	5.74763942	2.737307e+00	6.22239336	−1.1847125140000001	4.53e−06	0.000689686	DN
42	ENSG00000257046	RP11−545J16.1	4	0	18	22	0.09111459	0.00000000	0.3720947	0.48160006	4.555729e−02	0.42684740	−3.22796642	5.18e−06	0.000768212	DN
43	ENSG00000112494	UNC93A	9	16	114	40	0.16758550	0.31455628	1.9263456	0.71577873	2.410709e−01	1.32106215	−2.4541689870000001	5.75e−06	0.000835995	DN
44	ENSG00000020577	SAMD4A	502	252	169	157	2.04288058	1.08268969	0.6240768	0.61395556	1.562785e+00	0.61901617	1.3360704290000001	6.10e−06	0.000881865	UP
45	ENSG00000019144	PHLDB1	335	222	149	123	0.80221523	0.56125885	0.3237776	0.28304173	6.817370e−01	0.30340966	1.167948319	6.25e−06	0.000892301	UP
46	ENSG00000133794	ARNTL	179	177	98	83	1.49734282	1.56316979	0.7438849	0.66718201	1.530256e+00	0.70553347	1.1169868810000001	6.84e−06	0.000965200	UP
47	ENSG00000007255	TRAPPC6A	129	179	548	306	6.22386740	9.11771217	23.9918110	14.18692202	7.670790e+00	19.08936649	−1.315322195	6.89e−06	0.000966044	DN
48	ENSG00000183077	AFMID	80	82	196	188	0.92236796	0.99813963	2.0506069	2.08289555	9.602538e−01	2.06675124	−1.1058770899999999	7.05e−06	0.000982917	DN
49	ENSG00000231312	AC007246.3	27	29	86	79	0.29971751	0.33987230	0.8663022	0.84271745	3.197949e−01	0.85450982	−1.417950115	7.29e−06	0.001010207	DN
50	ENSG00000185479	KRT6B	4	3	32	19	0.15068644	0.11932591	1.0940300	0.68788899	1.350062e−01	0.89095949	−2.72233444	8.61e−06	0.001154465	DN
51	ENSG00000070731	ST6GALNAC2	10	9	52	32	0.08021567	0.07621806	0.3785090	0.24666608	7.821687e−02	0.31258753	−1.998708607	9.27e−06	0.001226631	DN
52	ENSG00000114779	ABHD14B	112	116	301	229	0.96215885	1.05209186	2.3464399	1.89044331	1.007125e+00	2.11844161	−1.0727600989999999	9.55e−06	0.001250131	DN
53	ENSG00000179532	DNHD1	25	26	110	64	0.05058195	0.05553878	0.2019595	0.12443306	5.306036e−02	0.16319628	−1.620901723	1.08e−05	0.001385570	DN
54	ENSG00000137033	IL33	110	113	239	261	1.59914652	1.73436484	3.1528733	3.64614874	1.666756e+00	3.39951100	−1.028284596	1.17e−05	0.001468516	DN
55	ENSG00000155792	DEPTOR	4	11	87	27	0.11067475	0.32137130	2.1845861	0.71794748	2.160230e−01	1.45126681	−2.748055779	1.17e−05	0.001468516	DN
56	ENSG00000165475	CRYL1	34	45	105	115	1.03111342	1.44078213	2.8895219	3.35134525	1.235948e+00	3.12043359	−1.336128728	1.16e−05	0.001468516	DN
57	ENSG00000133454	MYO18B	2	0	14	15	0.01289628	0.00000000	0.0819242	0.09295150	6.448141e−03	0.08743785	−3.7613027849999998	1.21e−05	0.001505913	DN
58	ENSG00000107796	ACTA2	29	37	85	113	0.56080314	0.75540033	1.4915507	2.09982878	6.581017e−01	1.79568974	−1.4481555690000001	1.26e−05	0.001555362	DN
59	ENSG00000120899	PTK2B	751	524	1778	1115	4.97902327	3.66774954	10.6966839	7.10356838	4.323386e+00	8.90012613	−1.041663998	1.29e−05	0.001572553	DN
60	ENSG00000132196	HSD17B7	65	71	26	29	0.86042656	0.99224428	0.3123063	0.36888189	9.263354e−01	0.34059411	1.4434811830000001	1.28e−05	0.001572553	UP
61	ENSG00000158055	GRHL3	396	363	164	239	6.57775347	6.36580822	2.4719473	3.81485352	6.471781e+00	3.14340043	1.0418366880000001	1.42e−05	0.001712907	UP
62	ENSG00000052795	FNIP2	477	340	195	243	3.40449602	2.56198869	1.2629402	1.66662918	2.983242e+00	1.46478467	1.0261925869999999	1.48e−05	0.001772330	UP
63	ENSG00000183426	NPIPA1	93	99	459	183	0.83194173	0.93499441	3.7259287	1.57310697	8.834681e−01	2.64951783	−1.5844799380000001	1.48e−05	0.001772330	DN
64	ENSG00000070526	ST6GALNAC1	62	104	284	189	0.75141358	1.33070722	3.1233273	2.20112859	1.041060e+00	2.66222794	−1.3545803199999999	1.66e−05	0.001969043	DN
65	ENSG00000147883	CDKN2B	533	379	151	270	7.84812502	5.89173165	2.0175689	3.82032505	6.869928e+00	2.91894696	1.2348470549999999	1.69e−05	0.001978394	UP
66	ENSG00000275410	HNF1B	178	162	75	96	1.69082755	1.62465478	0.6464803	0.87629003	1.657741e+00	0.76138515	1.122520422	1.68e−05	0.001978394	UP
67	ENSG00000168679	SLC16A4	208	195	589	354	2.12160639	2.09991231	5.4516700	3.46977644	2.110759e+00	4.46072321	−1.0795155279999999	1.73e−05	0.002015561	DN
68	ENSG00000101104	PABPC1L	245	296	1060	463	2.15931152	2.75426921	8.4775236	3.92128490	2.456790e+00	6.19940428	−1.335354827	1.79e−05	0.002080345	DN
69	ENSG00000107551	RASSF4	19	33	113	69	0.12530331	0.22976225	0.6762245	0.43726611	1.775328e−01	0.55674530	−1.648932077	1.80e−05	0.002080345	DN
70	ENSG00000165171	WBSCR27	12	15	44	56	0.61629915	0.81332654	2.0506527	2.76381955	7.148128e−01	2.40723611	−1.751740184	1.87e−05	0.002120811	DN
71	ENSG00000129991	TNNI3	4	5	25	26	0.08801291	0.11615197	0.4992145	0.54980597	1.020824e−01	0.52451023	−2.3612361800000001	1.96e−05	0.002191946	DN
72	ENSG00000179841	AKAP5	174	123	64	76	1.64610626	1.22851414	0.5494141	0.69090752	1.437310e+00	0.62016082	1.2126571820000001	2.05e−05	0.002268832	UP
73	ENSG00000204516	MICB	351	274	123	188	6.37195312	5.25147516	2.0261994	3.27959293	5.811714e+00	2.65289615	1.1313955410000001	2.05e−05	0.002268832	UP
74	ENSG00000141682	PMAIP1	264	233	156	117	5.65462459	5.26890310	3.0320517	2.40814370	5.461764e+00	2.72009768	1.005708477	2.46e−05	0.002634992	UP
75	ENSG00000171431	KRT20	6	10	44	28	0.25238997	0.44408166	1.6794484	1.13175976	3.482358e−01	1.40560410	−2.0130538229999999	2.44e−05	0.002634992	DN
76	ENSG00000186567	CEACAM19	42	45	144	96	0.38815703	0.43906453	1.2076147	0.85255306	4.136108e−01	1.03008386	−1.316416094	2.46e−05	0.002634992	DN
77	ENSG00000213859	KCTD11	185	32	637	454	4.55616573	0.83202885	14.2357243	10.74436550	2.694097e+00	12.49004491	−2.2129048079999998	2.48e−05	0.002636548	DN
78	ENSG00000165078	CPA6	170	164	86	93	4.38259288	4.46364451	2.0118472	2.30389960	4.423119e+00	2.15787341	1.0354537269999999	2.80e−05	0.002926992	UP
79	ENSG00000164088	PPM1M	16	21	135	45	0.20053012	0.27787223	1.5353202	0.54194918	2.392012e−01	1.03863468	−2.1183918940000002	3.03e−05	0.003140791	DN
80	ENSG00000240583	AQP1	93	58	21	34	1.63279394	1.07507085	0.3345638	0.57361488	1.353932e+00	0.45408934	1.576107632	3.08e−05	0.003165567	UP
81	ENSG00000111271	ACAD10	98	128	258	250	0.48946600	0.67494727	1.1693072	1.19986897	5.822066e−01	1.18458808	−1.024782289	3.10e−05	0.003167238	DN
82	ENSG00000107902	LHPP	54	56	172	115	0.87301798	0.95583264	2.5233064	1.78658589	9.144253e−01	2.15494616	−1.2367145820000001	3.22e−05	0.003246484	DN
83	ENSG00000171747	LGALS4	318	493	1111	751	6.09052634	9.96871487	19.3087779	13.82181349	8.029621e+00	16.56529567	−1.044760226	3.46e−05	0.003416973	DN
84	ENSG00000264230	ANXA8L1	115	74	33	45	1.01940698	0.69253823	0.2654475	0.38331492	8.559726e−01	0.32438120	1.3998744279999999	3.69e−05	0.003620972	UP
85	ENSG00000146592	CREB5	364	237	1142	505	2.10374187	1.44611969	5.9892178	2.80465916	1.774931e+00	4.39693849	−1.3087365879999999	4.11e−05	0.003944827	DN
86	ENSG00000109072	VTN	1064	1421	3465	2078	29.14252479	41.09078361	86.1196351	54.69264557	3.511665e+01	70.40614032	−1.00354586	4.28e−05	0.004078569	DN
87	ENSG00000281991	TMEM265	18	226	14	2	1.55534661	20.61706117	1.0977020	0.16605531	1.108620e+01	0.63187866	4.1329740920000004	4.35e−05	0.004118580	UP
88	ENSG00000166401	SERPINB8	302	355	1031	534	3.01845339	3.74602602	9.3508184	5.12880697	3.382240e+00	7.23981270	−1.0979734619999999	4.48e−05	0.004215138	DN
89	ENSG00000084710	EFR3B	20	24	67	63	0.14001328	0.17738826	0.4256289	0.42382242	1.587008e−01	0.42472568	−1.4202221770000001	4.52e−05	0.004235657	DN
90	ENSG00000235884	LINC00941	94	81	198	187	2.72570518	2.47970641	5.2099067	5.21065133	2.602706e+00	5.21027899	−1.001348385	4.63e−05	0.004317285	DN
91	ENSG00000198626	RYR2	44	55	105	211	0.18818815	0.24834943	0.4075114	0.86719554	2.182688e−01	0.63735349	−1.5459878970000001	4.72e−05	0.004367264	DN
92	ENSG00000105707	HPN	55	80	243	139	0.63142195	0.96963857	2.5314681	1.53343980	8.005303e−01	2.03245394	−1.3441948239999999	4.82e−05	0.004390848	DN
93	ENSG00000226950	DANCR	157	292	96	102	4.92636976	9.67328348	2.7334496	3.07556913	7.299827e+00	2.90450937	1.329567715	4.78e−05	0.004390848	UP
94	ENSG00000115255	REEP6	60	78	210	142	1.46634302	2.01251500	4.6570749	3.33477163	1.739429e+00	3.99592325	−1.1999150730000001	4.92e−05	0.004453507	DN
95	ENSG00000260914	RP11−343C2.11	7	9	0	0	0.46869860	0.63621899	0.0000000	0.00000000	5.524588e−01	0.00000000	11.96233994	5.02e−05	0.004514113	UP
96	ENSG00000116667	C1orf21	104	168	426	272	0.78036895	1.33089142	2.9006219	1.96125877	1.055630e+00	2.43094033	−1.2034099709999999	5.06e−05	0.004520187	DN
97	ENSG00000198894	CIPC	385	339	246	146	4.88500243	4.54117614	2.8323846	1.78014461	4.713089e+00	2.30626459	1.0311149719999999	5.14e−05	0.004581262	UP
98	ENSG00000130303	BST2	121	134	71	28	6.72905135	7.86749401	3.5829372	1.49632404	7.298273e+00	2.53963062	1.522936375	5.18e−05	0.004599603	UP
99	ENSG00000262585	RP11−353N14.5	14	20	73	45	0.22836339	0.34441393	1.0804816	0.70532562	2.863887e−01	0.89290359	−1.6405300540000001	5.22e−05	0.004612265	DN
100	ENSG00000173295	FAM86B3P	116	75	214	228	1.33528419	0.91146419	2.2353275	2.52201131	1.123374e+00	2.37866943	−1.0823162289999999	5.38e−05	0.004737173	DN

	gene.id	gene.name	AGSGKN2_2_count	AGSGKN2_1_count	AGScon_2_count	AGScon_1_count	AGSGKN2_2_FPKM	AGSGKN2_1_FPKM	AGScon_2_FPKM	AGScon_1_FPKM	AGSGKN2	AGScon	log2FC	Pvalue	Qvalue	updown
1	ENSG00000113578	FGF1	11	5	46	26	0.06610476	0.03171914	0.250841541	0.15014272	0.04891195	0.200492132	−2.0352866600000001	0.000054100	0.004750914	DN
2	ENSG00000180574	EIF2S3L	252	56	21	42	5.78685318	1.35767040	0.437595870	0.92680968	3.57226179	0.682202775	2.3885652830000002	0.000054500	0.004764512	UP
3	ENSG00000117724	CENPF	5666	5041	1847	3587	38.30919363	35.98387522	11.332002420	23.30535297	37.14653442	17.318677700	1.1008988390000001	0.000055200	0.004794176	UP
4	ENSG00000181577	C6orf223	43	52	18	18	0.36636099	0.46774537	0.139162180	0.14736979	0.41705318	0.143265985	1.5415352449999999	0.000056000	0.004824374	UP
5	ENSG00000166816	LDHD	3	6	23	25	0.06613911	0.13964191	0.460111194	0.52959943	0.10289051	0.494855313	−2.265896803	0.000059300	0.005034335	DN
6	ENSG00000253595	LINC01300	1	2	26	9	0.09072713	0.19145992	2.139693736	0.78433995	0.14109353	1.462016844	−3.3732362349999998	0.000061100	0.005150046	DN
7	ENSG00000137757	CASP5	0	0	15	5	0.00000000	0.00000000	0.472271811	0.16670872	0.00000000	0.319490265	NA	0.000064300	0.005357034	NA
8	ENSG00000160460	SPTBN4	16	16	58	45	0.07541454	0.07961769	0.248067874	0.20381401	0.07751612	0.225940943	−1.5433775009999999	0.000064600	0.005357034	DN
9	ENSG00000260342	RP11−1035H13.3	25	141	7	17	2.81332315	16.75202476	0.714850052	1.83840007	9.78267395	1.276625059	2.9378939869999998	0.000064700	0.005357034	UP
10	ENSG00000163216	SPRR2D	31	19	107	59	0.87328440	0.56507473	2.735212783	1.59712643	0.71917957	2.166169604	−1.590722272	0.000067300	0.005477836	DN
11	ENSG00000176046	NUPR1	7	10	40	28	0.09274295	0.13988333	0.480924725	0.35649822	0.11631314	0.418711473	−1.847942384	0.000067300	0.005477836	DN
12	ENSG00000178773	CPNE7	118	162	357	270	1.90627036	2.76301211	5.233390072	4.19144644	2.33464123	4.712418255	−1.013266727	0.000067100	0.005477836	DN
13	ENSG00000255439	RP11−196G11.1	10	3	25	37	0.26116390	0.08271620	0.592476000	0.92857571	0.17194005	0.760525855	−2.1450916740000001	0.000069100	0.005598789	DN
14	ENSG00000186951	PPARA	41	26	95	83	0.14766937	0.09886512	0.310487757	0.28726475	0.12326724	0.298876251	−1.277758776	0.000071200	0.005731995	DN
15	ENSG00000119950	MXI1	79	125	265	221	0.77473349	1.29419876	2.358220840	2.08264780	1.03446613	2.220434320	−1.101955491	0.000073400	0.005863944	DN
16	ENSG00000149927	DOC2A	37	48	133	93	0.25716646	0.35221658	0.838827734	0.62114027	0.30469152	0.729984003	−1.2605155109999999	0.000073100	0.005863944	DN
17	ENSG00000160182	TFF1	145	107	29	67	22.62519591	17.62674911	4.106176129	10.04612201	20.12597251	7.076149069	1.5080221519999999	0.000079700	0.006267310	UP
18	ENSG00000176153	GPX2	346	361	1055	548	6.66689695	7.34377394	18.446457880	10.14671807	7.00533544	14.296587970	−1.0291448350000001	0.000081300	0.006351919	DN
19	ENSG00000166670	MMP10	83	70	156	193	3.95129590	3.51822830	6.739011822	8.82904557	3.73476210	7.784028698	−1.0595006810000001	0.000084200	0.006552043	DN
20	ENSG00000261068	RP11−7K24.3	68	54	266	114	2.88422676	2.41816180	10.238084930	4.64651844	2.65119428	7.442301682	−1.4891064780000001	0.000085200	0.006609886	DN
21	ENSG00000130707	ASS1	37	60	215	100	0.70786584	1.21188638	3.732456634	1.83841442	0.95987611	2.785435526	−1.536982804	0.000096500	0.007291997	DN
22	ENSG00000197273	GUCA2A	29	11	4	2	4.38195212	1.75483414	0.548457037	0.29037792	3.06839313	0.419417477	2.8710244500000002	0.000098100	0.007378899	UP
23	ENSG00000111962	UST	37	69	168	125	0.63293854	1.24614457	2.607813671	2.05476963	0.93954155	2.331291653	−1.311100631	0.000106303	0.007868142	DN
24	ENSG00000229180	GS1−124K5.11	32	27	88	68	0.33881624	0.30182352	0.845505888	0.69186997	0.32031988	0.768687927	−1.2628846659999999	0.000107243	0.007900251	DN
25	ENSG00000099284	H2AFY2	20	10	1	3	0.76456341	0.40360289	0.034681489	0.11021751	0.58408315	0.072449497	3.0111261809999998	0.000119637	0.008722237	UP
26	ENSG00000229927	RHEBP1	127	57	262	221	19.63833286	9.30560375	36.763536140	32.83916055	14.47196830	34.801348340	−1.2658820500000001	0.000120703	0.008754167	DN
27	ENSG00000223745	RP4−717I23.3	15	22	88	43	0.12103494	0.18741528	0.644346111	0.33341589	0.15422511	0.488881002	−1.664445647	0.000122617	0.008865530	DN
28	ENSG00000264424	MYH4	181	337	708	519	2.58670590	5.08467654	9.181538321	7.12745482	3.83569122	8.154496568	−1.088109226	0.000124940	0.008978070	DN
29	ENSG00000256206	RP11−140L24.4	7	12	1	0	0.49288207	0.89206135	0.063880973	0.00000000	0.69247171	0.031940487	4.4382969269999997	0.000126086	0.009032727	UP
30	ENSG00000126583	PRKCG	46	65	174	113	1.01070568	1.50780045	3.469209886	2.38585196	1.25925306	2.927530925	−1.2171161720000001	0.000136410	0.009596249	DN
31	ENSG00000203857	HSD3B1	10	8	0	1	0.20349149	0.17188140	0.000000000	0.01956311	0.18768644	0.009781555	4.2621168789999997	0.000140767	0.009873096	UP
32	ENSG00000213443	RP11−75L1.2	9	4	10	223	0.91675620	0.43021262	0.924338205	21.82914490	0.67348441	11.376741550	−4.0782990520000002	0.000142931	0.009994930	DN
33	ENSG00000132932	ATP8A2	30	19	7	7	0.22001661	0.14711320	0.046587515	0.04933287	0.18356490	0.047960193	1.936380894	0.000153181	0.010461871	UP
34	ENSG00000260953	RP11−426C22.6	4	7	44	17	0.61295269	1.13262242	6.119059884	2.50363217	0.87278755	4.311346029	−2.3044359289999998	0.000160978	0.010899048	DN
35	ENSG00000136867	SLC31A2	100	71	40	42	3.23218081	2.42279195	1.173203211	1.30450204	2.82748638	1.238852628	1.190515499	0.000162575	0.010943883	UP
36	ENSG00000059804	SLC2A3	8604	6354	1677	4599	55.66117010	43.39742593	9.844604703	28.58994077	49.52929801	19.217272740	1.3658785659999999	0.000166785	0.011099716	UP
37	ENSG00000001630	CYP51A1	1160	1047	395	777	18.65546573	17.77703812	5.764464117	12.00789612	18.21625193	8.886180119	1.035590859	0.000167715	0.011129975	UP
38	ENSG00000128394	APOBEC3F	131	127	53	79	2.71721711	2.78112052	0.997571863	1.57463128	2.74916882	1.286101573	1.0959909130000001	0.000175517	0.011510770	UP
39	ENSG00000234667	ACTBP13	17	15	57	41	0.31539934	0.29380851	0.959647697	0.73097136	0.30460393	0.845309530	−1.472545175	0.000175909	0.011510770	DN
40	ENSG00000243742	RPLP0P2	6	15	38	36	0.08111401	0.21407369	0.466139281	0.46764420	0.14759385	0.466891739	−1.6614554859999999	0.000190341	0.012317500	DN
41	ENSG00000214290	COLCA2	3	8	34	19	0.07334164	0.20648248	0.754214891	0.44633571	0.13991206	0.600275299	−2.1011039569999999	0.000198872	0.012659722	DN
42	ENSG00000213240	RP11−458D21.5	0	0	36	1	0.00000000	0.00000000	0.823409114	0.02423164	0.00000000	0.423820379	NA	0.000200167	0.012707612	NA
43	ENSG00000213822	CEACAM18	16	17	41	63	0.93200315	1.04546347	2.167153936	3.52639780	0.98873331	2.846775870	−1.5256755719999999	0.000206293	0.012958626	DN
44	ENSG00000276600	RAB7B	65	35	155	108	0.60116938	0.34175069	1.300845168	0.95984666	0.47146003	1.130345913	−1.261556956	0.000213255	0.013251209	DN
45	ENSG00000271013	LRRC37A9P	2	2	10	21	0.03198163	0.03376191	0.145115596	0.32272837	0.03287177	0.233921983	−2.8311065540000002	0.000216623	0.013424890	DN
46	ENSG00000127311	HELB	85	78	45	38	0.47023831	0.45557093	0.225905107	0.20201566	0.46290462	0.213960383	1.1133712680000001	0.000223826	0.013762087	UP
47	ENSG00000267022	AC084219.2	0	0	0	56	0.00000000	0.00000000	0.000000000	1.22852733	0.00000000	0.614263664	NA	0.000226076	0.013858157	NA
48	ENSG00000205420	KRT6A	147	68	402	207	3.18590915	1.55591754	7.905947373	4.31104482	2.37091334	6.108496094	−1.3653743009999999	0.000234007	0.014206159	DN
49	ENSG00000140488	CELF6	3	0	15	11	0.04020302	0.00000000	0.182401737	0.14164266	0.02010151	0.162022199	−3.010815864	0.000237324	0.014328752	DN
50	ENSG00000270580	PKD1P6	87	109	179	284	2.34406357	3.10057480	4.376398937	7.35303554	2.72231919	5.864717240	−1.1072253219999999	0.000242973	0.014594567	DN
51	ENSG00000100271	TTLL1	11	7	36	28	0.35783612	0.24040907	1.062674418	0.87526181	0.29912260	0.968968113	−1.6957122870000001	0.000246335	0.014721037	DN
52	ENSG00000120915	EPHX2	43	62	157	106	0.47439353	0.72215083	1.571760734	1.12376614	0.59827218	1.347763435	−1.171693409	0.000247782	0.014769837	DN
53	ENSG00000073605	GSDMB	65	110	210	199	0.53880356	0.96265334	1.579597897	1.58513446	0.75072845	1.582366180	−1.0757204309999999	0.000249476	0.014833072	DN
54	ENSG00000172878	METAP1D	47	54	111	116	0.65492717	0.79442690	1.403556153	1.55327681	0.72467703	1.478416481	−1.0286426660000001	0.000250398	0.014850169	DN
55	ENSG00000137103	TMEM8B	47	43	126	89	0.43553574	0.42068477	1.059512140	0.79251691	0.42811026	0.926014523	−1.113052411	0.000261987	0.015343241	DN
56	ENSG00000104953	TLE6	29	26	84	60	0.36585924	0.34629630	0.961619333	0.72737843	0.35607777	0.844498881	−1.2459031380000001	0.000264673	0.015461947	DN
57	ENSG00000254614	AP003068.23	4	3	17	21	0.16076038	0.12730328	0.620049970	0.81112095	0.14403183	0.715585461	−2.312736369	0.000272457	0.015798441	DN
58	ENSG00000130304	SLC27A1	32	63	169	104	0.23045635	0.47901819	1.104445483	0.71974237	0.35473727	0.912093929	−1.362431494	0.000277467	0.015937096	DN
59	ENSG00000250067	YJEFN3	52	51	27	20	1.17682980	1.21853667	0.554469158	0.43493909	1.19768323	0.494704121	1.2756085669999999	0.000277190	0.015937096	UP
60	ENSG00000261796	ISY1−RAB43	0	45	0	0	0.00000000	0.83907505	0.000000000	0.00000000	0.41953753	0.000000000	11.96233994	0.000277569	0.015937096	UP
61	ENSG00000185920	PTCH1	103	88	37	57	0.26813854	0.24186189	0.087405291	0.14259083	0.25500021	0.114998062	1.1488889170000001	0.000291799	0.016591473	UP
62	ENSG00000118194	TNNT2	6	6	26	23	0.04153664	0.04384891	0.163319619	0.15299620	0.04269278	0.158157907	−1.8893018150000001	0.000295582	0.016765845	DN
63	ENSG00000182185	RAD51B	51	68	132	135	0.37673057	0.53031402	0.884809726	0.95827798	0.45352229	0.921543851	−1.022879345	0.000304541	0.017190768	DN
64	ENSG00000155629	PIK3AP1	83	137	228	273	0.95670202	1.66717354	2.384763685	3.02383123	1.31193778	2.704297455	−1.0435545429999999	0.000308817	0.017265729	DN
65	ENSG00000160323	ADAMTS13	12	15	51	35	0.14036528	0.18523927	0.541341586	0.39342125	0.16280227	0.467381419	−1.521479534	0.000308300	0.017265729	DN
66	ENSG00000260597	AC012531.25	34	42	20	8	1.09074871	1.42250583	0.582223982	0.24663559	1.25662727	0.414429785	1.6003571919999999	0.000312892	0.017451912	UP
67	ENSG00000227953	LINC01341	3	8	26	22	0.04199651	0.11823492	0.330255562	0.29592293	0.08011572	0.313089245	−1.9664167800000001	0.000315163	0.017536863	DN
68	ENSG00000175938	ORAI3	70	88	24	45	1.15670696	1.53521635	0.359875519	0.71454380	1.34596165	0.537209658	1.3250801619999999	0.000319847	0.017713306	UP
69	ENSG00000023171	GRAMD1B	83	110	31	56	0.37316532	0.52212731	0.126470762	0.24194080	0.44764631	0.184205781	1.281040964	0.000329671	0.018171464	UP
70	ENSG00000227827	RP11−958N24.2	57	68	157	121	0.49342048	0.62145840	1.233259303	1.00652659	0.55743944	1.119892947	−1.006473841	0.000336395	0.018498557	DN
71	ENSG00000216775	RP1−152L7.5	70	68	16	39	1.73189821	1.77620860	0.359219631	0.92722195	1.75405341	0.643220789	1.447306733	0.000341079	0.018668485	UP
72	ENSG00000145362	ANK2	27	24	103	51	0.06696608	0.06284633	0.231819679	0.12155348	0.06490620	0.176686578	−1.4447641419999999	0.000344467	0.018766231	DN
73	ENSG00000005187	ACSM3	58	97	250	149	0.46395386	0.81918830	1.814692029	1.14533456	0.64157108	1.480013294	−1.2059291160000001	0.000360642	0.019466348	DN
74	ENSG00000258727	RP11−66N24.3	21	18	66	44	0.55674142	0.50382417	1.587773729	1.12093755	0.53028280	1.354355639	−1.3527727709999999	0.000364752	0.019580664	DN
75	ENSG00000162433	AK4	126	56	288	189	0.67969564	0.31893073	1.409772604	0.97972181	0.49931319	1.194747208	−1.2586884840000001	0.000370236	0.019767137	DN
76	ENSG00000263020	XXbac−BPG32J3.22	17	21	33	173	0.46472514	0.60610605	0.818624834	4.54464951	0.53541559	2.681637174	−2.3243829900000001	0.000370433	0.019767137	DN
77	ENSG00000160161	CILP2	8	10	41	24	0.14383487	0.18981658	0.668908241	0.41465221	0.16682572	0.541780228	−1.6993659759999999	0.000376581	0.020004043	DN
78	ENSG00000074660	SCARF1	22	20	84	43	0.16799328	0.16124041	0.582061973	0.31552947	0.16461685	0.448795723	−1.446946946	0.000380269	0.020108754	DN
79	ENSG00000273117	AC144652.1	25	16	6	6	1.32350917	0.89427731	0.288220069	0.30521866	1.10889324	0.296719364	1.901949487	0.000395852	0.020745498	UP
80	ENSG00000145882	PCYOX1L	33	28	75	71	0.32491952	0.29105740	0.670097684	0.67176630	0.30798846	0.670931990	−1.123290235	0.000397719	0.020750460	DN
81	ENSG00000152154	TMEM178A	1	2	16	9	0.01930624	0.04074164	0.280199375	0.16690332	0.03002394	0.223551346	−2.8964209059999999	0.000397427	0.020750460	DN
82	ENSG00000175600	SUGCT	10	9	27	38	0.20402267	0.19385505	0.499864340	0.74502263	0.19893886	0.622443486	−1.645617721	0.000404479	0.020916807	DN
83	ENSG00000105270	CLIP3	2	3	11	20	0.03190448	0.05052918	0.159253571	0.30661228	0.04121683	0.232932926	−2.4986092530000001	0.000410056	0.021112037	DN
84	ENSG00000243708	PLA2G4B	8	7	24	31	0.11500056	0.10623661	0.313063422	0.42822206	0.11061858	0.370642740	−1.744435473	0.000412180	0.021174865	DN
85	ENSG00000272589	ZSWIM8−AS1	4	9	0	0	0.23552497	0.55952410	0.000000000	0.00000000	0.39752453	0.000000000	11.96233994	0.000416972	0.021327458	UP
86	ENSG00000148671	ADIRF	10	5	26	29	0.64017242	0.33790449	1.510372049	1.78398202	0.48903845	1.647177036	−1.751975807	0.000423891	0.021634130	DN
87	ENSG00000260019	RP11−218F4.1	0	0	7	7	0.00000000	0.00000000	0.407571224	0.43158898	0.00000000	0.419580104	NA	0.000426970	0.021743904	NA
88	ENSG00000173482	PTPRM	53	41	109	100	0.30077230	0.24565078	0.561311337	0.54533560	0.27321154	0.553323470	−1.018104699	0.000440200	0.022081638	DN
89	ENSG00000162600	OMA1	50	72	133	140	0.85872109	1.30551824	2.072772488	2.31052768	1.08211967	2.191650083	−1.01815743	0.000451249	0.022443651	DN
90	ENSG00000165030	NFIL3	93	111	54	57	3.83491221	4.83234446	2.020591866	2.25861142	4.33362834	2.139601644	1.0182332110000001	0.000455823	0.022623107	UP
91	ENSG00000157654	PALM2−AKAP2	35	27	90	64	0.30364586	0.24730646	0.708535068	0.53355540	0.27547616	0.621045236	−1.1727709040000001	0.000459399	0.022656581	DN
92	ENSG00000074590	NUAK1	19	5	0	2	0.20594796	0.05721202	0.000000000	0.02083019	0.13157999	0.010415093	3.659192467	0.000464572	0.022863564	UP
93	ENSG00000221878	PSG7	26	27	1	13	0.23248967	0.25489412	0.008112186	0.11170092	0.24369190	0.059906553	2.0242725560000001	0.000479991	0.023354689	UP
94	ENSG00000182796	TMEM198B	47	65	143	111	0.54710271	0.79881169	1.510484771	1.24162113	0.67295720	1.376052953	−1.0319493280000001	0.000494728	0.023945255	DN
95	ENSG00000137699	TRIM29	47	33	94	89	0.25387326	0.18818938	0.460738657	0.46195714	0.22103132	0.461347900	−1.061604271	0.000500007	0.024150848	DN
96	ENSG00000107159	CA9	0	0	11	4	0.00000000	0.00000000	0.319991608	0.12321032	0.00000000	0.221600963	NA	0.000513169	0.024634216	NA
97	ENSG00000197261	C6orf141	87	66	373	116	1.10731412	0.88687207	4.307977821	1.41875913	0.99709309	2.863368474	−1.5219132209999999	0.000512704	0.024634216	DN
98	ENSG00000196275	GTF2IRD2	25	33	71	70	0.24547498	0.34209804	0.632620023	0.66049479	0.29378651	0.646557408	−1.1380103319999999	0.000528485	0.025317580	DN
99	ENSG00000232133	IMPDH1P10	35	47	101	89	1.95144646	2.76667496	5.110066564	4.76846423	2.35906071	4.939265398	−1.0660839449999999	0.000546374	0.026014963	DN
100	ENSG00000086730	LAT2	34	35	91	71	0.37831067	0.41114461	0.918801760	0.75913851	0.39472764	0.838970137	−1.0877619089999999	0.000564286	0.026704994	DN

	gene.id	gene.name	AGSGKN2_2_count	AGSGKN2_1_count	AGScon_2_count	AGScon_1_count	AGSGKN2_2_FPKM	AGSGKN2_1_FPKM	AGScon_2_FPKM	AGScon_1_FPKM	AGSGKN2	AGScon	log2FC	Pvalue	Qvalue	updown
1	ENSG00000133943	C14orf159	8	15	47	29	0.04205297	0.083242152	0.22418615	0.14648373	0.062647559	0.18533494	−1.564804656	0.000570285	0.02689975	DN
2	ENSG00000115590	IL1R2	24	27	5	12	0.44760412	0.531628412	0.08461404	0.21506148	0.489616264	0.14983776	1.7082502239999999	0.000579672	0.02710457	UP
3	ENSG00000012124	CD22	45	50	88	152	0.23722374	0.278282820	0.42096436	0.77000031	0.257753281	0.59548234	−1.2080679219999999	0.000591541	0.02727843	DN
4	ENSG00000054392	HHAT	49	40	104	93	0.60226360	0.519058128	1.15994225	1.09842818	0.560660863	1.12918522	−1.010081875	0.000590113	0.02727843	DN
5	ENSG00000163629	PTPN13	56	76	235	113	0.39477229	0.565638223	1.50328144	0.76548366	0.480205257	1.13438255	−1.24018414	0.000588479	0.02727843	DN
6	ENSG00000272636	DOC2B	15	19	58	39	0.19071685	0.255044082	0.66918162	0.47650048	0.222880465	0.57284105	−1.3618647049999999	0.000589114	0.02727843	DN
7	ENSG00000171385	KCND3	13	19	3	5	0.11754066	0.181365873	0.02461598	0.04344350	0.149453265	0.03402974	2.134826388	0.000595131	0.02734712	UP
8	ENSG00000100170	SLC5A1	7	8	24	29	0.06813189	0.082214298	0.21198147	0.27124282	0.075173095	0.24161215	−1.6844046699999999	0.000613862	0.02781497	DN
9	ENSG00000156804	FBXO32	36	29	14	13	0.32597575	0.277231278	0.11503038	0.11311262	0.301603515	0.11407150	1.402714813	0.000617129	0.02790902	UP
10	ENSG00000256664	RP11−611O2.3	1	84	2	2	0.19592100	17.368180470	0.35534701	0.37636682	8.782050738	0.36585692	4.5852064349999999	0.000629976	0.02838041	UP
11	ENSG00000125246	CLYBL	32	22	74	60	0.32481557	0.235762669	0.68161680	0.58525195	0.280289120	0.63343437	−1.1762794130000001	0.000641344	0.02862269	DN
12	ENSG00000103269	RHBDL1	15	11	66	27	0.42802102	0.331397485	1.70897783	0.74034611	0.379709252	1.22466197	−1.6894165329999999	0.000647174	0.02876793	DN
13	ENSG00000003137	CYP26B1	47	42	89	112	0.45377885	0.428109558	0.77973122	1.03909990	0.440944203	0.90941556	−1.044343577	0.000656733	0.02886423	DN
14	ENSG00000064270	ATP2C2	19	13	77	33	0.14427088	0.104213073	0.53053875	0.24078611	0.124241978	0.38566243	−1.6341859240000001	0.000652706	0.02886423	DN
15	ENSG00000117983	MUC5B	0	1	9	8	0.00000000	0.004696515	0.03631836	0.03418939	0.002348257	0.03525387	−3.9081191789999998	0.000653114	0.02886423	DN
16	ENSG00000261611	AC010547.9	35	31	63	112	0.81836564	0.765272349	1.33670841	2.51650383	0.791818992	1.92660612	−1.2828190779999999	0.000654411	0.02886423	DN
17	ENSG00000273154	RP4−583P15.15	39	33	99	71	0.81504188	0.728101710	1.87742472	1.42582968	0.771571796	1.65162720	−1.0980157690000001	0.000660813	0.02898914	DN
18	ENSG00000138835	RGS3	280	273	80	187	0.90942162	0.936125898	0.23578118	0.58364177	0.922773760	0.40971147	1.1713686809999999	0.000670093	0.02917771	UP
19	ENSG00000261771	DYX1C1−CCPG1	0	0	0	36	0.00000000	0.000000000	0.00000000	0.61311422	0.000000000	0.30655711	NA	0.000674458	0.02925897	NA
20	ENSG00000111644	ACRBP	3	1	11	15	0.04690717	0.016511679	0.15606792	0.22535541	0.031709426	0.19071166	−2.5884093969999999	0.000679802	0.02943632	DN
21	ENSG00000153048	CARHSP1	557	532	153	381	3.86572260	3.898092908	0.96356630	2.54096639	3.881907753	1.75226635	1.1475437530000001	0.000691173	0.02981847	UP
22	ENSG00000240602	AADACP1	0	0	6	6	0.00000000	0.000000000	0.11049797	0.11701490	0.000000000	0.11375643	NA	0.000694884	0.02986855	NA
23	ENSG00000257529	RPL36A−HNRNPH2	108	371	76	99	9.67231175	35.078773150	6.17634547	8.51997943	22.375542450	7.34816245	1.606467232	0.000702879	0.03010172	UP
24	ENSG00000115257	PCSK4	24	26	64	58	0.25844877	0.295592342	0.62538521	0.60018273	0.277020556	0.61278397	−1.1453855260000001	0.000720383	0.03079507	DN
25	ENSG00000105519	CAPS	12	10	30	37	0.14204664	0.124976305	0.32225000	0.42088273	0.133511475	0.37156636	−1.476656151	0.000740436	0.03142294	DN
26	ENSG00000165071	TMEM71	0	0	10	4	0.00000000	0.000000000	0.13473946	0.05707255	0.000000000	0.09590600	NA	0.000743429	0.03143606	NA
27	ENSG00000221968	FADS3	139	113	375	194	0.99622235	0.855037817	2.43885169	1.33610457	0.925630081	1.88747813	−1.027952274	0.000748630	0.03154211	DN
28	ENSG00000116151	MORN1	39	37	87	81	0.22437649	0.224739687	0.45419628	0.44780994	0.224558090	0.45100311	−1.0060486740000001	0.000757287	0.03179250	DN
29	ENSG00000266997	RP11−886H22.1	0	0	5	7	0.00000000	0.000000000	0.41674223	0.61787312	0.000000000	0.51730767	NA	0.000758836	0.03180051	NA
30	ENSG00000265787	CYP4F35P	19	16	69	36	0.46159344	0.410371957	1.52111432	0.84041173	0.435982701	1.18076302	−1.437376652	0.000763123	0.03192309	DN
31	ENSG00000042832	TG	4	3	23	12	0.02340023	0.018530230	0.12211185	0.06746740	0.020965233	0.09478963	−2.1767303409999998	0.000784781	0.03259625	DN
32	ENSG00000268614	CTD−2207O23.10	0	0	2	17	0.00000000	0.000000000	0.16560227	1.49101661	0.000000000	0.82830944	NA	0.000798223	0.03286323	NA
33	ENSG00000257354	RP11−631N16.2	13	9	30	37	0.12857404	0.093972757	0.26923981	0.35164744	0.111273397	0.31044363	−1.4802226089999999	0.000804854	0.03307807	DN
34	ENSG00000182871	COL18A1	36	28	103	60	0.27114672	0.222646804	0.70396823	0.43426053	0.246896760	0.56911438	−1.204810736	0.000826178	0.03377671	DN
35	ENSG00000007952	NOX1	9	9	29	28	0.21065675	0.222408162	0.61598232	0.62981676	0.216532455	0.62289954	−1.5244162219999999	0.000849048	0.03453092	DN
36	ENSG00000129455	KLK8	41	67	166	102	0.85268881	1.471122058	3.13277072	2.03847591	1.161905434	2.58562332	−1.1540194610000001	0.000848039	0.03453092	DN
37	ENSG00000197565	COL4A6	59	59	103	175	0.39276697	0.414666643	0.62220653	1.11948492	0.403716806	0.87084573	−1.1090735169999999	0.000888354	0.03581854	DN
38	ENSG00000261114	RP11−325K4.2	18	11	1	5	2.21397694	1.428470134	0.11158608	0.59099314	1.821223536	0.35128961	2.3741751999999998	0.000923125	0.03671499	UP
39	ENSG00000273259	SERPINA3	2	4	31	8	0.04802604	0.101424427	0.67555877	0.18463223	0.074725234	0.43009550	−2.5249896079999998	0.000979931	0.03858130	DN
40	ENSG00000205177	C11orf91	3	2	9	21	0.23406765	0.164704179	0.63715316	1.57439902	0.199385914	1.10577609	−2.471423884	0.000988580	0.03870002	DN
41	ENSG00000100298	APOBEC3H	18	34	54	101	0.60641453	1.209329214	1.65083622	3.26975129	0.907871872	2.46029376	−1.4382699720000001	0.001030654	0.03984175	DN
42	ENSG00000185989	RASA3	16	14	38	44	0.27446861	0.253551945	0.59150954	0.72529938	0.264010276	0.65840446	−1.318380025	0.001038476	0.04001208	DN
43	ENSG00000259426	RP11−253M7.1	18	16	56	37	0.48681028	0.456843773	1.37430996	0.96158265	0.471827027	1.16794631	−1.3076439849999999	0.001061659	0.04077120	DN
44	ENSG00000131471	AOC3	4	5	27	14	0.03728762	0.049209036	0.22841339	0.12542382	0.043248329	0.17691861	−2.0323694840000002	0.001087694	0.04156661	DN
45	ENSG00000128254	C22orf24	19	26	79	45	0.61924913	0.894620644	2.33637795	1.40931439	0.756934886	1.87284617	−1.306991303	0.001093566	0.04165509	DN
46	ENSG00000241288	RP11−379B18.5	17	6	73	24	0.10983396	0.040926088	0.42798664	0.14900931	0.075380026	0.28849798	−1.9363070019999999	0.001112688	0.04231467	DN
47	ENSG00000182489	XKRX	7	5	26	19	0.11807122	0.089033889	0.39796540	0.30797777	0.103552554	0.35297159	−1.7691889119999999	0.001116283	0.04238256	DN
48	ENSG00000242852	ZNF709	60	56	28	32	0.87094289	0.858199893	0.36881722	0.44635607	0.864571391	0.40758665	1.084878314	0.001119608	0.04244003	UP
49	ENSG00000256552	RP11−113C12.4	8	12	1	2	0.69546848	1.101321443	0.07886620	0.16706272	0.898394961	0.12296446	2.8691084290000002	0.001129429	0.04274315	UP
50	ENSG00000135519	KCNH3	13	23	53	44	0.23169447	0.432775193	0.85715907	0.75356560	0.332234829	0.80536234	−1.277434685	0.001134248	0.04285627	DN
51	ENSG00000261487	AC135048.13	2	0	9	10	0.08844302	0.000000000	0.36118456	0.42500433	0.044221509	0.39309444	−3.1520557880000002	0.001144210	0.04316308	DN
52	ENSG00000230461	PROX1−AS1	8	3	26	18	0.03929043	0.015554766	0.11587054	0.08494851	0.027422599	0.10040952	−1.8724588600000001	0.001151901	0.04331085	DN
53	ENSG00000279696	RP11−178H8.7	22	7	2	3	0.60007498	0.201586667	0.04949154	0.07864187	0.400830824	0.06406671	2.6453467590000002	0.001178710	0.04410937	UP
54	ENSG00000139625	MAP3K12	3	1	18	8	0.01811140	0.006375349	0.09860171	0.04641085	0.012243374	0.07250628	−2.5661047830000001	0.001212773	0.04509591	DN
55	ENSG00000132010	ZNF20	82	64	18	45	1.16935331	0.963562259	0.23292180	0.61664810	1.066457782	0.42478495	1.328022297	0.001224098	0.04544486	UP
56	ENSG00000174564	IL20RB	9	7	70	14	0.17856964	0.146642321	1.26037901	0.26694172	0.162605978	0.76366037	−2.231550849	0.001232582	0.04561527	DN
57	ENSG00000125122	LRRC29	3	2	17	10	0.04601187	0.032376740	0.23658346	0.14737931	0.039194306	0.19198139	−2.292250466	0.001283362	0.04682872	DN
58	ENSG00000203688	RP11−351J23.1	13	5	36	24	0.43711150	0.177475844	1.09841552	0.77546613	0.307293671	0.93694082	−1.608339878	0.001365099	0.04927396	DN

Supporting Material 5A: ESTIMATE score

	ID	Stromal_score	Immune_score	ESTIMATE_score
1	TCGA−D7−6521−01	1015.80	1339.97	2355.77
2	TCGA−VQ−A91K−01	−86.84	631.61	544.77
3	TCGA−CG−4462−01	2073.37	1920.57	3993.94
4	TCGA−D7−6519−01	−539.10	−117.69	−656.79
5	TCGA−HU−A4GN−01	−822.75	89.01	−733.74
6	TCGA−IN−A6RS−01	−716.06	673.48	−42.59
7	TCGA−FP−7998−01	1254.44	2454.52	3708.97
8	TCGA−EQ−A4SO−01	−1437.50	−560.20	−1997.69
9	TCGA−CG−5719−01	1063.59	878.70	1942.29
10	TCGA−BR−6458−01	812.20	1257.51	2069.72
11	TCGA−BR−4369−01	213.68	578.74	792.42
12	TCGA−BR−7717−01	−18.78	74.72	55.94
13	TCGA−FP−8211−01	−1044.69	348.68	−696.02
14	TCGA−CD−5804−01	580.83	924.03	1504.86
15	TCGA−BR−8296−01	27.70	1364.22	1391.92
16	TCGA−CG−4475−01	757.51	197.33	954.84
17	TCGA−IN−A6RO−01	−1534.45	−348.01	−1882.46
18	TCGA−CD−8527−01	−613.25	72.24	−541.01
19	TCGA−CG−5726−01	−1392.16	−339.94	−1732.10
20	TCGA−CD−8530−01	1130.03	481.83	1611.87
21	TCGA−VQ−A8DT−01	−1348.88	−336.03	−1684.90
22	TCGA−CG−5721−01	344.15	2297.62	2641.77
23	TCGA−VQ−A91Y−01	959.31	1182.34	2141.65
24	TCGA−BR−7958−01	612.09	2111.97	2724.06
25	TCGA−BR−8373−01	503.98	14.33	518.30
26	TCGA−VQ−A91D−01	−161.63	407.01	245.38
27	TCGA−VQ−AA68−01	−1034.73	193.61	−841.12
28	TCGA−RD−A8NB−01	394.73	1091.46	1486.19
29	TCGA−CD−A48A−01	−551.69	45.93	−505.75
30	TCGA−VQ−A927−01	−298.29	161.53	−136.76
31	TCGA−HU−A4G9−01	−1834.98	−717.51	−2552.49
32	TCGA−CG−4436−01	−814.33	625.09	−189.24
33	TCGA−BR−4280−01	−572.60	690.15	117.55
34	TCGA−RD−A7BW−01	1593.70	1755.11	3348.81
35	TCGA−BR−4255−01	1367.70	937.26	2304.96
36	TCGA−VQ−A94O−01	−706.22	−110.41	−816.63
37	TCGA−BR−A4PF−01	−292.83	1062.97	770.14
38	TCGA−VQ−A8PP−01	−95.17	358.76	263.59
39	TCGA−HU−A4GH−01	−1838.38	−62.50	−1900.88
40	TCGA−FP−7829−01	−11.62	−46.16	−57.78
41	TCGA−F1−A72C−01	234.30	570.52	804.83
42	TCGA−HJ−7597−01	−1050.87	434.51	−616.37
43	TCGA−F1−A448−01	568.15	1262.05	1830.21
44	TCGA−CD−8525−01	95.69	1010.20	1105.89
45	TCGA−IN−A6RJ−01	−1443.63	−271.51	−1715.14
46	TCGA−HU−8604−01	507.86	1743.54	2251.40
47	TCGA−BR−8678−01	−327.97	−510.93	−838.90
48	TCGA−VQ−A8E3−01	−717.00	810.42	93.42
49	TCGA−BR−A4J4−01	36.83	513.74	550.57
50	TCGA−VQ−A91V−01	−1453.79	−523.75	−1977.55
51	TCGA−BR−4292−01	−504.81	873.48	368.67
52	TCGA−HU−A4H0−01	−769.08	1102.75	333.67
53	TCGA−D7−6518−01	1472.57	875.67	2348.23
54	TCGA−HU−A4G2−01	−300.64	404.88	104.23
55	TCGA−BR−A4J7−01	1136.15	1612.96	2749.11
56	TCGA−BR−4361−01	970.42	1074.69	2045.11
57	TCGA−BR−6452−01	36.37	702.88	739.26
58	TCGA−CD−A486−01	55.79	383.23	439.02
59	TCGA−BR−6802−01	−122.55	1184.86	1062.31
60	TCGA−KB−A93H−01	−1674.34	−268.78	−1943.12
61	TCGA−BR−6563−01	1529.82	2026.70	3556.53
62	TCGA−RD−A7BT−01	−1340.26	−123.59	−1463.85
63	TCGA−R5−A7ZI−01	−389.24	1775.92	1386.68
64	TCGA−BR−A4CS−01	69.37	253.83	323.20
65	TCGA−CG−4305−01	798.73	1390.92	2189.65
66	TCGA−CG−4460−01	−486.96	−58.20	−545.17
67	TCGA−KB−A93J−01	−313.82	1100.60	786.78
68	TCGA−CG−4449−01	1142.72	502.23	1644.96
69	TCGA−BR−6803−01	1148.95	1085.54	2234.49
70	TCGA−RD−A7BS−01	118.64	961.59	1080.22
71	TCGA−BR−8080−01	1379.09	1179.53	2558.63
72	TCGA−BR−8362−01	752.73	806.33	1559.07
73	TCGA−BR−4267−01	−304.89	417.28	112.39
74	TCGA−BR−6801−01	95.27	−181.43	−86.16
75	TCGA−VQ−A8P8−01	19.53	589.26	608.80
76	TCGA−BR−7704−01	−29.53	1658.79	1629.26
77	TCGA−D7−8575−01	−233.50	984.56	751.05
78	TCGA−CD−A4MH−01	−401.18	17.10	−384.08
79	TCGA−BR−A4J8−01	379.26	79.37	458.63
80	TCGA−BR−4363−01	850.93	1145.91	1996.84
81	TCGA−HU−A4H6−01	−271.89	794.49	522.60
82	TCGA−B7−A5TN−01	517.68	132.77	650.45
83	TCGA−FP−7916−01	914.94	1828.31	2743.25
84	TCGA−D7−5577−01	−58.92	1640.37	1581.45
85	TCGA−CG−5722−01	231.49	2198.65	2430.14
86	TCGA−BR−6565−01	424.76	981.34	1406.10
87	TCGA−VQ−AA64−01	262.48	−320.29	−57.81
88	TCGA−CD−8526−01	−28.22	194.54	166.32
89	TCGA−D7−A747−01	810.55	1141.05	1951.60
90	TCGA−BR−4256−01	1678.99	2123.52	3802.51
91	TCGA−BR−4187−01	2085.81	1705.01	3790.82
92	TCGA−D7−5578−01	302.42	924.30	1226.72
93	TCGA−BR−7196−01	1258.71	1272.84	2531.54
94	TCGA−BR−4191−01	942.20	1851.53	2793.73
95	TCGA−VQ−A924−01	−246.08	449.95	203.87
96	TCGA−VQ−A8PD−01	486.58	1626.47	2113.06
97	TCGA−VQ−A91S−01	−623.97	525.84	−98.13
98	TCGA−D7−8570−01	768.92	2255.26	3024.18
99	TCGA−IN−AB1V−01	−1414.23	−299.70	−1713.93
100	TCGA−SW−A7EB−01	−727.36	401.92	−325.44

	ID	Stromal_score	Immune_score	ESTIMATE_score
1	TCGA−CG−4465−01	556.75	1001.07	1557.82
2	TCGA−R5−A7ZR−01	−1263.69	−175.26	−1438.95
3	TCGA−IN−A7NT−01	−393.95	−278.21	−672.17
4	TCGA−CG−4441−01	421.75	451.68	873.43
5	TCGA−ZQ−A9CR−01	801.81	847.55	1649.36
6	TCGA−VQ−A8PE−01	−580.05	267.32	−312.72
7	TCGA−BR−8486−01	255.03	744.67	999.70
8	TCGA−BR−6453−01	911.58	2404.23	3315.82
9	TCGA−BR−4294−01	−897.55	−637.19	−1534.75
10	TCGA−VQ−A91Q−01	43.35	−472.76	−429.41
11	TCGA−VQ−AA6D−01	−1529.45	−727.36	−2256.81
12	TCGA−CG−5732−01	−878.92	1180.33	301.41
13	TCGA−RD−A8N4−01	666.93	605.61	1272.54
14	TCGA−HU−8608−01	319.12	2093.47	2412.59
15	TCGA−HF−A5NB−01	−1202.22	103.92	−1098.31
16	TCGA−BR−8687−01	−243.05	−273.15	−516.20
17	TCGA−BR−8683−01	590.02	268.52	858.54
18	TCGA−CD−A4MJ−01	−276.80	−41.16	−317.96
19	TCGA−CD−5800−01	−826.55	−160.59	−987.14
20	TCGA−BR−8382−01	387.21	814.77	1201.98
21	TCGA−BR−8588−01	798.81	1196.87	1995.67
22	TCGA−BR−8487−01	−364.36	515.76	151.40
23	TCGA−BR−6457−01	1507.37	1035.79	2543.17
24	TCGA−CD−8533−01	−482.03	−1143.65	−1625.68
25	TCGA−BR−A452−01	−1061.49	−736.32	−1797.81
26	TCGA−BR−8368−01	−1036.93	138.15	−898.78
27	TCGA−BR−A44U−01	−120.80	−428.33	−549.12
28	TCGA−BR−A4J5−01	1204.34	694.71	1899.05
29	TCGA−MX−A666−01	−942.84	35.98	−906.86
30	TCGA−D7−6818−01	1110.53	1061.51	2172.04
31	TCGA−VQ−A922−01	283.75	−596.63	−312.88
32	TCGA−ZA−A8F6−01	715.89	908.31	1624.20
33	TCGA−R5−A7O7−01	−246.51	328.39	81.89
34	TCGA−VQ−A8PM−01	168.08	1039.59	1207.67
35	TCGA−VQ−A8DU−01	−374.57	−201.18	−575.75
36	TCGA−BR−4201−01	1373.95	1185.14	2559.09
37	TCGA−CD−A489−01	686.70	714.09	1400.79
38	TCGA−D7−8572−01	902.04	699.71	1601.75
39	TCGA−IN−A6RR−01	−1001.88	−192.66	−1194.54
40	TCGA−BR−7197−01	−410.89	−721.90	−1132.78
41	TCGA−D7−6524−01	1210.13	1086.67	2296.79
42	TCGA−CG−5725−01	−1036.02	−989.65	−2025.67
43	TCGA−FP−A4BF−01	1171.61	2130.93	3302.54
44	TCGA−VQ−A91E−01	−1388.70	419.58	−969.12
45	TCGA−CG−4474−01	922.56	1297.28	2219.84
46	TCGA−BR−4279−01	2053.43	1643.55	3696.98
47	TCGA−BR−8384−01	1465.35	903.76	2369.11
48	TCGA−D7−A6EY−01	207.98	1535.89	1743.87
49	TCGA−EQ−8122−01	53.17	−233.94	−180.76
50	TCGA−BR−8363−01	384.88	1678.52	2063.40
51	TCGA−D7−A74A−01	−1340.73	31.22	−1309.51
52	TCGA−BR−4257−01	734.80	1217.14	1951.95
53	TCGA−B7−A5TK−01	886.36	2084.89	2971.24
54	TCGA−CG−4440−01	−795.98	147.02	−648.97
55	TCGA−BR−8590−01	1146.88	1037.22	2184.10
56	TCGA−HF−7136−01	−127.63	−443.50	−571.14
57	TCGA−RD−A8N1−01	461.44	1875.80	2337.24
58	TCGA−HU−A4GF−01	−884.98	161.37	−723.61
59	TCGA−BR−A4J1−01	−1039.91	−1169.12	−2209.03
60	TCGA−IN−7806−01	−863.28	−316.70	−1179.98
61	TCGA−CG−5718−01	−169.07	940.40	771.33
62	TCGA−FP−A9TM−01	−1071.55	1017.92	−53.62
63	TCGA−D7−6526−01	−741.60	−347.55	−1089.15
64	TCGA−CD−8532−01	303.13	1503.41	1806.55
65	TCGA−CG−5734−01	140.59	1936.28	2076.87
66	TCGA−VQ−A8PF−01	642.29	1962.04	2604.34
67	TCGA−BR−6564−01	1271.10	877.02	2148.11
68	TCGA−HU−8244−01	−1892.02	−518.85	−2410.86
69	TCGA−R5−A805−01	583.90	1622.05	2205.95
70	TCGA−VQ−A91Z−01	−1769.32	−1110.86	−2880.18
71	TCGA−BR−8059−01	628.99	−316.14	312.85
72	TCGA−HU−8610−01	325.12	1381.18	1706.30
73	TCGA−BR−A44T−01	749.38	2509.85	3259.23
74	TCGA−HU−8602−01	−78.44	1178.07	1099.63
75	TCGA−CD−A487−01	35.16	−231.45	−196.29
76	TCGA−BR−A4QM−01	−186.21	1280.99	1094.78
77	TCGA−HU−A4H8−01	−1426.50	−143.95	−1570.45
78	TCGA−BR−8289−01	194.72	−115.95	78.78
79	TCGA−CD−8529−01	1070.99	1749.14	2820.13
80	TCGA−BR−A4PD−01	−333.94	825.26	491.32
81	TCGA−D7−A6EV−01	−1166.87	−488.03	−1654.90
82	TCGA−BR−8364−01	1658.21	1008.33	2666.54
83	TCGA−CG−4301−01	703.08	776.66	1479.74
84	TCGA−BR−8361−01	−505.76	633.49	127.73
85	TCGA−VQ−AA6F−01	−702.05	565.72	−136.33
86	TCGA−BR−7723−01	22.12	346.53	368.65
87	TCGA−HF−7134−01	−500.86	263.55	−237.32
88	TCGA−RD−A8N9−01	1318.02	986.08	2304.10
89	TCGA−CG−4469−01	−336.22	659.11	322.89
90	TCGA−CG−4438−01	−40.18	1109.38	1069.20
91	TCGA−VQ−A8PX−01	−1526.91	107.99	−1418.92
92	TCGA−CD−5799−01	−283.60	−194.53	−478.13
93	TCGA−CD−8535−01	−790.96	−896.89	−1687.85
94	TCGA−FP−8210−01	1546.01	2008.71	3554.72
95	TCGA−RD−A8N0−01	871.28	2029.42	2900.70
96	TCGA−BR−8372−01	−250.32	1277.21	1026.90
97	TCGA−CD−5798−01	987.66	835.64	1823.30
98	TCGA−CG−5717−01	−225.17	924.68	699.51
99	TCGA−BR−8297−01	556.08	139.83	695.92
100	TCGA−CG−4476−01	1168.02	1209.50	2377.51

	ID	Stromal_score	Immune_score	ESTIMATE_score
1	TCGA−BR−8592−01	1306.04	864.02	2170.05
2	TCGA−D7−6817−01	−585.77	367.32	−218.45
3	TCGA−VQ−A8PJ−01	−1228.91	−560.77	−1789.69
4	TCGA−BR−8381−01	819.99	1854.68	2674.67
5	TCGA−RD−A8MW−01	893.97	1485.32	2379.29
6	TCGA−VQ−AA6I−01	−831.74	232.23	−599.51
7	TCGA−D7−6522−01	1904.47	2754.43	4658.90
8	TCGA−D7−6528−01	−1051.70	−422.65	−1474.35
9	TCGA−CD−A4MI−01	1306.12	1971.69	3277.81
10	TCGA−BR−4368−01	552.74	1142.07	1694.81
11	TCGA−HU−A4G8−01	−518.96	587.49	68.53
12	TCGA−BR−8484−01	423.83	1324.47	1748.30
13	TCGA−B7−5818−01	−232.11	1182.91	950.80
14	TCGA−F1−6177−01	−915.47	−155.87	−1071.34
15	TCGA−BR−A4J9−01	748.32	428.48	1176.80
16	TCGA−3M−AB47−01	932.88	635.88	1568.76
17	TCGA−VQ−A91X−01	−1957.19	−900.36	−2857.55
18	TCGA−D7−6820−01	−970.81	168.23	−802.58
19	TCGA−BR−7901−01	853.88	708.76	1562.64
20	TCGA−BR−8483−01	−800.36	−808.39	−1608.75
21	TCGA−D7−6520−01	342.14	102.69	444.84
22	TCGA−HU−A4GC−01	−276.74	17.09	−259.65
23	TCGA−VQ−A8P5−01	−6.74	150.20	143.45
24	TCGA−RD−A8N5−01	1317.24	1221.90	2539.15
25	TCGA−D7−8573−01	−1066.25	−137.91	−1204.16
26	TCGA−CG−4443−01	−343.67	−663.34	−1007.01
27	TCGA−BR−8077−01	−38.01	1041.87	1003.86
28	TCGA−BR−A4IU−01	1051.71	1163.72	2215.43
29	TCGA−VQ−A8PU−01	−1209.35	−489.64	−1698.99
30	TCGA−BR−7959−01	1114.63	303.65	1418.29
31	TCGA−BR−A4J6−01	−183.18	−145.69	−328.87
32	TCGA−BR−A4IV−01	1430.55	218.87	1649.42
33	TCGA−VQ−A94P−01	1261.31	711.74	1973.04
34	TCGA−HU−A4GQ−01	31.28	303.82	335.10
35	TCGA−BR−4362−01	−37.81	1064.20	1026.39
36	TCGA−BR−7707−01	−345.39	409.22	63.83
37	TCGA−IN−7808−01	−43.63	2591.55	2547.91
38	TCGA−D7−8578−01	885.42	345.58	1230.99
39	TCGA−CD−5801−01	−94.05	1802.27	1708.22
40	TCGA−BR−6705−01	1676.19	622.60	2298.80
41	TCGA−BR−8295−01	−1294.23	−1568.74	−2862.97
42	TCGA−D7−A6EZ−01	125.37	1276.29	1401.66
43	TCGA−HU−A4GX−01	−706.36	1064.96	358.60
44	TCGA−R5−A7ZF−01	−1416.21	−759.08	−2175.29
45	TCGA−CG−5720−01	330.71	1198.36	1529.07
46	TCGA−MX−A5UJ−01	212.26	97.85	310.11
47	TCGA−CG−5723−01	16.03	1484.80	1500.84
48	TCGA−D7−6525−01	125.64	195.75	321.39
49	TCGA−BR−8485−01	319.10	570.96	890.06
50	TCGA−CG−4466−01	−1112.21	−518.91	−1631.12
51	TCGA−BR−6709−01	1080.72	2140.85	3221.57
52	TCGA−BR−A4IZ−01	1935.09	1148.97	3084.07
53	TCGA−IN−A6RL−01	−785.32	−25.10	−810.41
54	TCGA−VQ−A94T−01	−870.18	−824.36	−1694.54
55	TCGA−F1−6874−01	241.14	928.26	1169.41
56	TCGA−VQ−A91N−01	−1140.32	−375.30	−1515.62
57	TCGA−VQ−A8E7−01	−529.27	−419.00	−948.27
58	TCGA−BR−8591−01	−37.11	542.60	505.50
59	TCGA−SW−A7EA−01	−326.29	296.78	−29.50
60	TCGA−D7−8576−01	−136.15	173.40	37.25
61	TCGA−VQ−A8E2−01	216.64	−371.25	−154.61
62	TCGA−FP−7735−01	−169.70	734.22	564.52
63	TCGA−BR−8060−01	810.20	672.91	1483.11
64	TCGA−BR−6566−01	509.75	1165.88	1675.63
65	TCGA−D7−A4YT−01	−679.77	−567.28	−1247.05
66	TCGA−FP−8209−01	1676.52	2014.94	3691.46
67	TCGA−HU−8243−01	−637.05	−360.38	−997.44
68	TCGA−HU−8249−01	−805.15	264.65	−540.50
69	TCGA−VQ−A8DV−01	−1339.78	−1043.68	−2383.45
70	TCGA−BR−6454−01	−148.48	1475.74	1327.26
71	TCGA−CD−A4MG−01	308.66	604.31	912.98
72	TCGA−HU−A4GU−01	−929.31	−297.23	−1226.55
73	TCGA−VQ−A94R−01	508.11	293.67	801.78
74	TCGA−BR−8589−01	−694.29	1709.75	1015.46
75	TCGA−D7−A4YV−01	364.29	1216.88	1581.17
76	TCGA−VQ−A91A−01	1118.53	567.30	1685.82
77	TCGA−HU−A4GP−01	−682.48	−138.95	−821.43
78	TCGA−D7−6815−01	−10.55	−77.16	−87.71
79	TCGA−VQ−A92D−01	−690.29	−753.39	−1443.68
80	TCGA−BR−8677−01	1072.90	1118.04	2190.94
81	TCGA−BR−7703−01	−879.57	333.31	−546.26
82	TCGA−CG−4304−01	700.63	1099.29	1799.92
83	TCGA−HU−A4G3−01	−650.70	−146.44	−797.13
84	TCGA−HU−A4HB−01	−328.54	2005.36	1676.82
85	TCGA−BR−6707−01	−12.01	1391.93	1379.91
86	TCGA−VQ−A8DZ−01	−624.87	−178.37	−803.24
87	TCGA−BR−A4QL−01	−983.80	26.01	−957.78
88	TCGA−CD−A48C−01	72.99	191.49	264.48
89	TCGA−BR−7722−01	−44.35	429.60	385.25
90	TCGA−CD−5803−01	1590.27	2238.05	3828.32
91	TCGA−CD−5813−01	1795.77	1958.23	3754.00
92	TCGA−BR−7715−01	202.71	−533.40	−330.69
93	TCGA−BR−A453−01	1419.12	522.85	1941.97
94	TCGA−CG−4442−01	−381.92	−154.29	−536.21
95	TCGA−D7−A6F2−01	−73.64	799.16	725.52
96	TCGA−R5−A7ZE−01	−1482.38	−871.12	−2353.50
97	TCGA−CG−4477−01	348.14	1566.67	1914.81
98	TCGA−HU−A4GD−01	−1692.88	−723.45	−2416.33
99	TCGA−VQ−A923−01	−37.37	1714.50	1677.13
100	TCGA−KB−A93G−01	1632.26	1038.85	2671.11

	ID	Stromal_score	Immune_score	ESTIMATE_score
1	TCGA−VQ−A8P2−01	−1308.98	−776.42	−2085.40
2	TCGA−VQ−A8PC−01	124.65	960.99	1085.63
3	TCGA−BR−8284−01	909.19	1516.58	2425.76
4	TCGA−VQ−A8P3−01	−163.15	−43.09	−206.23
5	TCGA−CD−8534−01	−93.98	427.04	333.06
6	TCGA−BR−8682−01	870.13	600.63	1470.76
7	TCGA−RD−A8N2−01	971.71	544.85	1516.56
8	TCGA−HU−A4H2−01	−682.65	310.76	−371.89
9	TCGA−BR−A4IY−01	961.13	1140.00	2101.13
10	TCGA−CD−8531−01	251.40	2039.54	2290.94
11	TCGA−BR−6456−01	1078.36	682.77	1761.13
12	TCGA−HU−8238−01	−267.09	581.52	314.43
13	TCGA−BR−8365−01	1356.11	793.37	2149.48
14	TCGA−IN−8462−01	−20.83	239.84	219.02
15	TCGA−BR−8367−01	814.64	213.41	1028.05
16	TCGA−BR−8679−01	205.15	−332.51	−127.36
17	TCGA−HF−7133−01	158.05	1078.55	1236.61
18	TCGA−FP−A8CX−01	−895.82	288.33	−607.49
19	TCGA−HU−A4H3−01	−766.48	−16.15	−782.63
20	TCGA−VQ−A91W−01	−735.06	833.52	98.46
21	TCGA−D7−A6EX−01	−702.78	−814.41	−1517.19
22	TCGA−BR−7851−01	252.39	433.80	686.19
23	TCGA−BR−A4J2−01	1249.58	1506.79	2756.37
24	TCGA−B7−5816−01	1388.12	1409.47	2797.59
25	TCGA−HF−7131−01	−350.24	642.34	292.10
26	TCGA−F1−6875−01	−1024.92	−1024.20	−2049.12
27	TCGA−IN−AB1X−01	−769.73	1034.82	265.09
28	TCGA−BR−8686−01	693.98	1530.31	2224.29
29	TCGA−HF−7132−01	280.19	1374.35	1654.53
30	TCGA−BR−8369−01	25.65	−331.49	−305.84
31	TCGA−FP−A4BE−01	−256.39	1182.10	925.71
32	TCGA−CG−4306−01	351.55	874.20	1225.76
33	TCGA−HU−A4GY−01	1690.03	2639.91	4329.94
34	TCGA−IP−7968−01	358.85	380.39	739.24
35	TCGA−BR−4357−01	573.62	1478.24	2051.85
36	TCGA−IN−A7NU−01	28.42	814.17	842.58
37	TCGA−BR−4370−01	1214.64	1267.19	2481.83
38	TCGA−VQ−A925−01	−979.12	−329.95	−1309.07
39	TCGA−RD−A8N6−01	−213.32	−412.93	−626.25
40	TCGA−VQ−AA6A−01	−1171.28	−476.94	−1648.22
41	TCGA−BR−4371−01	−943.27	235.04	−708.22
42	TCGA−VQ−AA6B−01	−845.57	−807.02	−1652.59
43	TCGA−VQ−A8PH−01	−1311.44	−64.94	−1376.37
44	TCGA−VQ−A8E0−01	−1029.14	243.61	−785.53
45	TCGA−BR−8058−01	1278.35	1878.15	3156.50
46	TCGA−BR−8371−01	528.56	899.06	1427.62
47	TCGA−B7−A5TJ−01	−607.21	−306.28	−913.49
48	TCGA−IN−8663−01	−902.61	−480.63	−1383.24
49	TCGA−VQ−AA6K−01	−244.98	212.42	−32.56
50	TCGA−FP−8099−01	−67.07	198.42	131.35
51	TCGA−RD−A7C1−01	−8.98	1398.23	1389.25
52	TCGA−BR−4253−01	409.37	2494.30	2903.67
53	TCGA−CG−5716−01	−685.45	997.74	312.29
54	TCGA−D7−A4YX−01	−546.54	854.39	307.86
55	TCGA−MX−A5UG−01	1500.55	1777.14	3277.69
56	TCGA−VQ−A8PO−01	−155.62	1049.10	893.48
57	TCGA−BR−8291−01	1693.88	1298.16	2992.04
58	TCGA−D7−A4YU−01	445.43	1611.66	2057.09
59	TCGA−VQ−A8PK−01	−437.84	−72.90	−510.74
60	TCGA−IN−A6RI−01	−1591.93	−481.57	−2073.50
61	TCGA−BR−6706−01	1203.26	2011.70	3214.96
62	TCGA−VQ−A91U−01	−541.96	661.32	119.36
63	TCGA−BR−4366−01	−72.23	170.60	98.37
64	TCGA−D7−8579−01	995.41	832.87	1828.28
65	TCGA−VQ−A8PB−01	−379.61	−194.47	−574.08
66	TCGA−D7−A748−01	1772.94	1873.30	3646.24
67	TCGA−HU−A4HD−01	−416.98	−387.60	−804.58
68	TCGA−CG−4437−01	420.00	1316.50	1736.50
69	TCGA−BR−4367−01	1091.84	1380.01	2471.85
70	TCGA−HU−A4G6−01	−1642.40	−499.09	−2141.49
71	TCGA−VQ−AA6G−01	−942.95	−211.90	−1154.85
72	TCGA−BR−8081−01	937.58	1812.79	2750.37
73	TCGA−BR−8380−01	925.69	94.23	1019.92
74	TCGA−VQ−A928−01	207.01	−202.88	4.13
75	TCGA−CG−4444−01	344.91	818.44	1163.35
76	TCGA−BR−6455−01	111.51	929.45	1040.96
77	TCGA−BR−8286−01	116.33	375.34	491.67
78	TCGA−BR−6852−01	627.39	1969.87	2597.25
79	TCGA−HU−A4GJ−01	206.33	2826.73	3033.07
80	TCGA−KB−A6F7−01	−870.15	885.83	15.68
81	TCGA−BR−7716−01	161.52	1371.45	1532.97
82	TCGA−CG−4472−01	1139.18	1354.19	2493.37
83	TCGA−BR−8680−01	−1221.67	−1184.83	−2406.49
84	TCGA−VQ−A94U−01	226.56	−398.46	−171.90
85	TCGA−VQ−A8PQ−01	809.74	2520.08	3329.82
86	TCGA−D7−A6F0−01	−619.00	245.08	−373.92
87	TCGA−FP−8631−01	−215.86	−390.21	−606.07
88	TCGA−RD−A8MV−01	3.90	1840.36	1844.26
89	TCGA−D7−6822−01	−534.14	−153.73	−687.86
90	TCGA−HU−A4GT−01	−873.20	−8.34	−881.54
91	TCGA−HU−A4H4−01	−383.12	1135.91	752.79
92	TCGA−CD−8528−01	−104.10	29.81	−74.29
93	TCGA−BR−A4PE−01	6.25	873.28	879.54
94	TCGA−BR−A4CR−01	−1056.28	−1182.70	−2238.98
95	TCGA−IN−A7NR−01	−556.35	548.71	−7.65
96	TCGA−BR−8366−01	1077.74	2134.92	3212.66
97	TCGA−BR−8676−01	−766.66	1039.38	272.72
98	TCGA−CG−5724−01	−62.08	533.85	471.77
99	TCGA−CD−8524−01	471.35	461.36	932.71
100	TCGA−D7−8574−01	1544.35	2335.58	3879.93

	ID	Stromal_score	Immune_score	ESTIMATE_score
1	TCGA−IN−A6RN−01	−1136.54	−286.57	−1423.11
2	TCGA−MX−A663−01	1006.36	−146.67	859.69
3	TCGA−BR−A4QI−01	−533.80	98.75	−435.05
4	TCGA−BR−7957−01	1323.58	187.30	1510.88
5	TCGA−HU−A4H5−01	−957.75	−37.39	−995.14
6	TCGA−D7−A4Z0−01	442.38	1068.63	1511.00
7	TCGA−BR−8690−01	423.53	1396.69	1820.22
8	TCGA−D7−A4YY−01	397.18	790.12	1187.31
9	TCGA−B7−A5TI−01	326.09	130.14	456.23
10	TCGA−D7−6527−01	−281.06	94.33	−186.73
11	TCGA−CD−8536−01	210.21	501.14	711.36
12	TCGA−VQ−AA6J−01	−66.87	1245.47	1178.60
13	TCGA−3M−AB46−01	−432.99	−350.21	−783.20
14	TCGA−VQ−AA69−01	−1148.41	140.99	−1007.41
15	TCGA−BR−8078−01	459.92	1294.43	1754.35

Supporting Material 5B: CIBERSORT resilts

	Input Sample	B cells naive	B cells memory	Plasma cells	T cells CD8	T cells CD4 naive	T cells CD4 memory resting	T cells CD4 memory activated	T cells follicular helper	T cells regulatory (Tregs)	T cells gamma delta	NK cells resting
1	TCGA−BR−8364	0.017259821	0.029591676	0.003254790	0.13460695	0	0.122810881	0.000000000	0.063631946	0.092646910	0.000000000	0.000000000
2	TCGA−BR−8363	0.025571686	0.000000000	0.030706600	0.24392878	0	0.077938640	0.043974025	0.095100043	0.067305242	0.000000000	0.000000000
3	TCGA−BR−8380	0.036920578	0.000000000	0.016507059	0.09828457	0	0.175744210	0.000000000	0.088759553	0.079432091	0.000000000	0.000000000
4	TCGA−BR−8372	0.000000000	0.009251768	0.000000000	0.14591183	0	0.065898958	0.068528516	0.069155990	0.064864879	0.007874613	0.000000000
5	TCGA−BR−8486	0.020457754	0.000700000	0.013876034	0.03332112	0	0.290173838	0.000000000	0.101206046	0.119740677	0.000000000	0.001255020
6	TCGA−BR−8589	0.018864021	0.000000000	0.000600000	0.29545490	0	0.004256270	0.079728983	0.092579270	0.052478399	0.000000000	0.000000000
7	TCGA−BR−8592	0.122929278	0.056717271	0.000000000	0.05981825	0	0.288757814	0.000000000	0.017603216	0.053461665	0.000000000	0.000000000
8	TCGA−BR−8678	0.002077324	0.000000000	0.004996554	0.17012475	0	0.000000000	0.000000000	0.123181369	0.016672021	0.000000000	0.036505171
9	TCGA−BR−8591	0.026310716	0.056269026	0.000000000	0.05668784	0	0.217555548	0.000000000	0.079414970	0.073841856	0.000300000	0.000000000
10	TCGA−IN−7808	0.049746234	0.111385545	0.000000000	0.12500921	0	0.154974383	0.000000000	0.092891756	0.081589739	0.062234109	0.000000000
11	TCGA−BR−8686	0.060824242	0.022410332	0.000000000	0.16525277	0	0.205707318	0.000000000	0.034162372	0.047936823	0.000000000	0.000000000
12	TCGA−IN−8462	0.017903403	0.011150958	0.000000000	0.12852108	0	0.223808694	0.000000000	0.044029677	0.120281451	0.000000000	0.000000000
13	TCGA−BR−6707	0.013684674	0.022378584	0.000000000	0.26047478	0	0.001569801	0.008018284	0.079313829	0.065658015	0.052676405	0.000000000
14	TCGA−HU−A4GN	0.000000000	0.047814502	0.000000000	0.13605862	0	0.073042326	0.000000000	0.094028779	0.099289269	0.000000000	0.000000000
15	TCGA−HU−A4GP	0.011227873	0.010421671	0.000000000	0.08116944	0	0.146270197	0.000000000	0.032337398	0.083585025	0.000000000	0.008317556
16	TCGA−HU−A4GC	0.001884001	0.011974670	0.010660133	0.14171596	0	0.243647223	0.000000000	0.022712279	0.060590509	0.000000000	0.000000000
17	TCGA−D7−6527	0.007444748	0.000000000	0.081266861	0.13265709	0	0.099413593	0.000000000	0.064000020	0.037965084	0.000000000	0.000000000
18	TCGA−D7−6524	0.000000000	0.000000000	0.000700000	0.04843712	0	0.190660405	0.000000000	0.016258689	0.052514593	0.000000000	0.013856399
19	TCGA−D7−6528	0.016637655	0.000000000	0.000000000	0.04778661	0	0.092026858	0.000000000	0.038014334	0.072892700	0.000000000	0.069503194
20	TCGA−D7−6522	0.063521161	0.080028538	0.000000000	0.12383017	0	0.114269882	0.000000000	0.063420427	0.064160425	0.007032110	0.000000000
21	TCGA−FP−8631	0.085095025	0.000000000	0.054547990	0.06593248	0	0.126182059	0.000000000	0.068419683	0.084214438	0.000000000	0.000000000
22	TCGA−HU−8238	0.081926217	0.013092686	0.000000000	0.03828271	0	0.158518510	0.000000000	0.064748548	0.074490463	0.000000000	0.000000000
23	TCGA−HU−A4GF	0.023373091	0.000000000	0.017991920	0.07688167	0	0.105062001	0.000000000	0.094733834	0.095479930	0.000000000	0.000000000
24	TCGA−HU−A4G3	0.000000000	0.000000000	0.015951514	0.07887002	0	0.165480542	0.000000000	0.029158282	0.044468153	0.000000000	0.000000000
25	TCGA−D7−5578	0.006794291	0.085596273	0.000000000	0.13079554	0	0.210916713	0.000000000	0.034729922	0.111488049	0.000000000	0.002045556
26	TCGA−D7−6820	0.036279772	0.002877483	0.000000000	0.00543516	0	0.195650088	0.000000000	0.073079947	0.096549632	0.000000000	0.000000000
27	TCGA−HU−A4GJ	0.169846341	0.136495366	0.006464953	0.08762979	0	0.210736315	0.000000000	0.060068070	0.058723820	0.000000000	0.000000000
28	TCGA−HU−8610	0.021296517	0.000000000	0.001197958	0.03290605	0	0.196362706	0.011750879	0.042770065	0.089217138	0.000000000	0.018677997
29	TCGA−HU−A4G9	0.000400000	0.014479965	0.010576848	0.12846709	0	0.195613676	0.000000000	0.072320981	0.097094580	0.000000000	0.000000000
30	TCGA−HU−A4GH	0.038940307	0.000000000	0.043329627	0.11647854	0	0.181987326	0.000000000	0.087276115	0.108408758	0.000000000	0.000000000
31	TCGA−D7−6525	0.004142630	0.000000000	0.025918084	0.11954349	0	0.084805830	0.000000000	0.038587394	0.082407833	0.000000000	0.000000000
32	TCGA−BR−6705	0.135132572	0.000000000	0.000000000	0.08715864	0	0.149245837	0.000000000	0.031366194	0.104290570	0.000000000	0.000000000
33	TCGA−D7−6815	0.052216639	0.000000000	0.040441479	0.05468275	0	0.142067550	0.000000000	0.008929666	0.104004740	0.000000000	0.000000000
34	TCGA−BR−6852	0.039660493	0.000000000	0.004210117	0.22886846	0	0.050671744	0.000000000	0.085797230	0.079386755	0.000000000	0.000000000
35	TCGA−BR−6710	0.008746992	0.003412662	0.031956883	0.16528036	0	0.223512650	0.000000000	0.105687571	0.059364516	0.000000000	0.000000000
36	TCGA−BR−6564	0.017259821	0.029591676	0.003254790	0.13460695	0	0.122810881	0.000000000	0.063631946	0.092646910	0.000000000	0.000000000
37	TCGA−D7−6817	0.067755044	0.103445488	0.004438085	0.05948852	0	0.315961701	0.000000000	0.039361085	0.067431532	0.000000000	0.000000000
38	TCGA−HU−8602	0.000000000	0.011587678	0.000000000	0.03491481	0	0.187705524	0.053247767	0.015650523	0.002025599	0.000000000	0.044690333
39	TCGA−BR−4255	0.015692241	0.001396341	0.034463787	0.16646842	0	0.099835732	0.000000000	0.057705771	0.112951768	0.012595257	0.000000000
40	TCGA−BR−4187	0.095645252	0.026379039	0.000000000	0.07126691	0	0.153171645	0.000000000	0.010850482	0.026700817	0.004420253	0.000000000
41	TCGA−BR−4257	0.013208410	0.002512112	0.000000000	0.17990144	0	0.051207187	0.079211282	0.100262415	0.021107468	0.000000000	0.000000000
42	TCGA−BR−4267	0.003278373	0.049594827	0.003152611	0.05462918	0	0.224621965	0.000000000	0.046526118	0.095489988	0.000000000	0.000500000
43	TCGA−HU−A4HB	0.106380617	0.162307483	0.000000000	0.09287700	0	0.214468064	0.000000000	0.049925378	0.053351135	0.009557489	0.000000000
44	TCGA−HU−A4H3	0.030173845	0.000000000	0.016636940	0.17249078	0	0.032989954	0.047691373	0.082128552	0.064318802	0.000000000	0.000000000
45	TCGA−HU−A4G8	0.071400694	0.028713060	0.000000000	0.08587177	0	0.244520793	0.000000000	0.119982851	0.023297257	0.000000000	0.000000000
46	TCGA−BR−A4PE	0.022103019	0.000000000	0.004330627	0.02763304	0	0.073024669	0.000000000	0.043991642	0.077371344	0.000000000	0.000000000
47	TCGA−HU−A4GX	0.000000000	0.010649458	0.002860447	0.29559453	0	0.005229298	0.025907469	0.109650415	0.044359637	0.037727542	0.000000000
48	TCGA−HU−A4H0	0.027048613	0.000000000	0.018063005	0.15445425	0	0.096159154	0.030458502	0.110707839	0.040670910	0.000000000	0.000000000
49	TCGA−HU−A4GU	0.022395647	0.000000000	0.000000000	0.05427777	0	0.140482449	0.000000000	0.057262894	0.094554793	0.000000000	0.026207537
50	TCGA−D7−6526	0.167152776	0.000000000	0.007055463	0.13469365	0	0.150447380	0.000000000	0.068629592	0.089240197	0.000000000	0.000000000
51	TCGA−D7−8578	0.000000000	0.028028180	0.007366241	0.16868378	0	0.155337265	0.000000000	0.072213239	0.086281353	0.000000000	0.000000000
52	TCGA−BR−8286	0.000000000	0.001085550	0.000000000	0.06635271	0	0.253123909	0.000000000	0.041592716	0.137909162	0.000000000	0.000000000
53	TCGA−D7−8579	0.036249492	0.000000000	0.006372341	0.05895032	0	0.306836339	0.000000000	0.000000000	0.049182281	0.000000000	0.000000000
54	TCGA−BR−8365	0.012886496	0.048251162	0.000000000	0.11031847	0	0.297445050	0.000000000	0.020370863	0.052183196	0.000000000	0.000000000
55	TCGA−CD−8529	0.070521262	0.001000000	0.023497609	0.07760790	0	0.259480502	0.000000000	0.050363203	0.073883049	0.000000000	0.000000000
56	TCGA−BR−8366	0.000000000	0.057009776	0.000000000	0.16487669	0	0.029596281	0.050697879	0.051284471	0.049084313	0.004565387	0.000000000
57	TCGA−BR−8060	0.010995659	0.000000000	0.026096002	0.08339080	0	0.097501490	0.016627080	0.028712846	0.047389876	0.000000000	0.000000000
58	TCGA−HU−8244	0.020809370	0.024329956	0.000000000	0.06188904	0	0.297212137	0.000000000	0.049404787	0.089409179	0.000000000	0.000000000
59	TCGA−BR−8080	0.066674376	0.013048272	0.000000000	0.10250449	0	0.143587949	0.000000000	0.031769886	0.089669404	0.000000000	0.000000000
60	TCGA−HU−A4G6	0.011881527	0.023773162	0.000000000	0.08032629	0	0.178824859	0.000000000	0.063442588	0.125783923	0.000000000	0.046426554
61	TCGA−BR−A4CR	0.025272537	0.000000000	0.033880251	0.04970677	0	0.155096015	0.000000000	0.009344789	0.149033798	0.000000000	0.044477312
62	TCGA−BR−8373	0.035828317	0.000000000	0.073484034	0.01976649	0	0.232448318	0.000000000	0.013254144	0.068282515	0.000000000	0.003693872
63	TCGA−CD−8532	0.006230321	0.004164078	0.005241538	0.14425300	0	0.215745669	0.000000000	0.125377113	0.046656804	0.007439453	0.000000000
64	TCGA−BR−7716	0.025110113	0.000000000	0.000000000	0.08722746	0	0.118674251	0.055866048	0.094715207	0.075013451	0.000000000	0.000000000
65	TCGA−FP−7916	0.015756015	0.005705010	0.015076788	0.12556119	0	0.116441397	0.000000000	0.065554520	0.074403513	0.002872572	0.000000000
66	TCGA−IP−7968	0.021878155	0.009434300	0.009974004	0.19276620	0	0.134935959	0.000000000	0.139602074	0.026892188	0.000000000	0.000000000
67	TCGA−F1−6875	0.055316413	0.032588510	0.022723827	0.17431045	0	0.053903387	0.000000000	0.103813811	0.097547787	0.000000000	0.000000000
68	TCGA−IN−7806	0.026537828	0.044173648	0.000000000	0.13609332	0	0.136641075	0.000000000	0.041356613	0.080059946	0.000000000	0.000000000
69	TCGA−BR−7717	0.077756208	0.000600000	0.000000000	0.06902836	0	0.157349806	0.000000000	0.038550647	0.115140379	0.000000000	0.000000000
70	TCGA−B7−5818	0.045827626	0.000000000	0.030394596	0.20355179	0	0.032659887	0.053174294	0.104998235	0.021889344	0.000000000	0.000000000
71	TCGA−D7−5577	0.033318812	0.000000000	0.022052024	0.28708443	0	0.000000000	0.055843532	0.084014167	0.029229950	0.000000000	0.000000000
72	TCGA−CD−5801	0.000000000	0.002025899	0.002830235	0.28842668	0	0.077248587	0.063972236	0.092627195	0.065453905	0.000000000	0.000000000
73	TCGA−BR−6566	0.000000000	0.026180696	0.000000000	0.06705181	0	0.243187889	0.000000000	0.060142849	0.041703052	0.000000000	0.000000000
74	TCGA−FP−8209	0.183158825	0.068163357	0.000000000	0.09882399	0	0.290664396	0.023341722	0.006834626	0.011010002	0.000000000	0.034378134
75	TCGA−BR−8367	0.032710909	0.000000000	0.002256132	0.06738450	0	0.218027876	0.000000000	0.021057658	0.031208580	0.000000000	0.020684405
76	TCGA−BR−8289	0.021670057	0.055091582	0.015209963	0.04893257	0	0.147868061	0.000000000	0.013586040	0.115978328	0.000000000	0.000000000
77	TCGA−BR−8371	0.374746247	0.000000000	0.000000000	0.15773003	0	0.100459975	0.000000000	0.038468681	0.091738298	0.000000000	0.000000000
78	TCGA−CD−8526	0.161469526	0.000000000	0.021355461	0.06874360	0	0.218870504	0.000000000	0.052575956	0.099142399	0.000000000	0.000000000
79	TCGA−BR−8368	0.017259821	0.029591676	0.003254790	0.13460695	0	0.122810881	0.000000000	0.063631946	0.092646910	0.000000000	0.000000000
80	TCGA−D7−8573	0.079372773	0.000000000	0.012911351	0.15819650	0	0.292905075	0.061950198	0.056883764	0.040958797	0.000000000	0.036460928
81	TCGA−BR−7722	0.000000000	0.023301259	0.000900000	0.05427707	0	0.222883710	0.000000000	0.001969175	0.071881345	0.000000000	0.000000000
82	TCGA−CD−5804	0.007928437	0.050055067	0.000000000	0.18157600	0	0.006176354	0.000000000	0.052306470	0.107987137	0.000000000	0.000000000
83	TCGA−BR−6563	0.064785550	0.000000000	0.000000000	0.15731368	0	0.153452693	0.000000000	0.016749608	0.066460011	0.000000000	0.000000000
84	TCGA−D7−6818	0.015634255	0.000000000	0.010773454	0.01321795	0	0.132383897	0.000000000	0.017001967	0.054825014	0.000000000	0.000000000
85	TCGA−BR−6709	0.020567730	0.023305565	0.000000000	0.12991304	0	0.153025380	0.000000000	0.063724852	0.085183433	0.000000000	0.000000000
86	TCGA−BR−6803	0.053871013	0.000000000	0.000000000	0.15972027	0	0.201065259	0.000000000	0.102512112	0.065349204	0.000000000	0.000000000
87	TCGA−D7−6822	0.060913513	0.004142856	0.000000000	0.08354708	0	0.085196893	0.000000000	0.196657011	0.000000000	0.000000000	0.000000000
88	TCGA−D7−6521	0.042286901	0.062235268	0.000000000	0.14713454	0	0.184004883	0.000000000	0.063121641	0.045860040	0.036956532	0.000000000
89	TCGA−CD−8524	0.128250167	0.000000000	0.033788281	0.14361620	0	0.151316594	0.000000000	0.089601646	0.048906703	0.011545410	0.000000000
90	TCGA−CD−8530	0.029435495	0.009836811	0.000000000	0.08799264	0	0.192430638	0.000000000	0.039701295	0.043367168	0.000000000	0.000000000
91	TCGA−FP−8211	0.000000000	0.010531232	0.000000000	0.12285331	0	0.224163796	0.000000000	0.089926706	0.086538852	0.012216132	0.000000000
92	TCGA−CD−A487	0.000000000	0.000000000	0.003598087	0.09189069	0	0.051076045	0.000000000	0.056298840	0.064442951	0.000000000	0.000000000
93	TCGA−CD−A489	0.061855432	0.042163309	0.000000000	0.11284270	0	0.244159421	0.000000000	0.050643165	0.008326244	0.035816715	0.000000000
94	TCGA−D7−8570	0.017259821	0.029591676	0.003254790	0.13460695	0	0.122810881	0.000000000	0.063631946	0.092646910	0.000000000	0.000000000
95	TCGA−CD−8528	0.017259821	0.029591676	0.003254790	0.13460695	0	0.122810881	0.000000000	0.063631946	0.092646910	0.000000000	0.000000000
96	TCGA−CD−8531	0.000000000	0.171566964	0.049694750	0.08412849	0	0.029545297	0.037898511	0.133046084	0.069139032	0.000000000	0.000000000
97	TCGA−BR−8078	0.000000000	0.000000000	0.002397505	0.13555490	0	0.062957896	0.000000000	0.044615158	0.043081913	0.010745659	0.000000000
98	TCGA−BR−8077	0.000000000	0.000000000	0.030316030	0.07862499	0	0.118137445	0.000000000	0.065193905	0.113248165	0.000000000	0.000000000
99	TCGA−CD−8534	0.037237733	0.016008417	0.000000000	0.07853215	0	0.108800859	0.000000000	0.042399893	0.081178493	0.011566720	0.000000000
100	TCGA−FP−8099	0.022909777	0.020428031	0.022420022	0.05476441	0	0.177238323	0.000000000	0.051010301	0.175338520	0.000000000	0.041544149

	Input Sample	NK cells activated	Monocytes	Macrophages M0	Macrophages M1	Macrophages M2	Dendritic cells resting	Dendritic cells activated	Mast cells resting	Mast cells activated	Eosinophils	Neutrophils	P−value	Pearson Correlation	RMSE
1	TCGA−BR−8364	0.060502514	0.021263925	0.083978187	0.095916934	0.22055376	0.007199252	0.000400000	0.005969530	0.040446787	0e+00	0.000000000	0.01	0.223068412	0.9998417
2	TCGA−BR−8363	0.061864066	0.003417182	0.060759374	0.086438836	0.13586421	0.000000000	0.010961744	0.000000000	0.027653092	0e+00	0.028516480	0.01	0.327725150	0.9541466
3	TCGA−BR−8380	0.052590690	0.060374335	0.058083804	0.075710245	0.18475634	0.000000000	0.000000000	0.022902900	0.049933621	0e+00	0.000000000	0.11	0.119667443	1.0391994
4	TCGA−BR−8372	0.013658283	0.004045710	0.142485455	0.078757507	0.25497457	0.036974574	0.000000000	0.000000000	0.030516071	0e+00	0.007101277	0.01	0.323384356	0.9532375
5	TCGA−BR−8486	0.007636831	0.011517106	0.115147002	0.039158893	0.10636446	0.000000000	0.003041009	0.055844378	0.080546317	0e+00	0.000000000	0.04	0.179693179	1.0178556
6	TCGA−BR−8589	0.046852557	0.015305547	0.000000000	0.095026149	0.16286105	0.078189803	0.006564186	0.000000000	0.051197001	0e+00	0.000000000	0.01	0.337964856	0.9484507
7	TCGA−BR−8592	0.044602278	0.030304754	0.000000000	0.006985050	0.18175622	0.000000000	0.004942058	0.120675323	0.007921353	0e+00	0.003525465	0.03	0.200163290	1.0042197
8	TCGA−BR−8678	0.060578936	0.000000000	0.238967037	0.134318488	0.04650663	0.000000000	0.000000000	0.000000000	0.142657471	0e+00	0.023414248	0.01	0.391791086	0.9289081
9	TCGA−BR−8591	0.090956144	0.000000000	0.059464035	0.094096024	0.15094362	0.000000000	0.000000000	0.057404634	0.018183353	0e+00	0.018531596	0.03	0.192362613	1.0125521
10	TCGA−IN−7808	0.104967244	0.004654169	0.000000000	0.048886141	0.08125473	0.000000000	0.000000000	0.000000000	0.073484645	0e+00	0.008922097	0.01	0.259261585	0.9959068
11	TCGA−BR−8686	0.073918231	0.035088554	0.000000000	0.084783912	0.15016655	0.002594041	0.000000000	0.082584296	0.034570558	0e+00	0.000000000	0.01	0.291216940	0.9693968
12	TCGA−IN−8462	0.000000000	0.024485157	0.015685744	0.086572421	0.14658965	0.014153449	0.002599625	0.000000000	0.164218688	0e+00	0.000000000	0.05	0.159527262	1.0310822
13	TCGA−BR−6707	0.121279641	0.000000000	0.053423880	0.095263192	0.16247211	0.003227230	0.000000000	0.060559577	0.000000000	0e+00	0.000000000	0.00	0.421664412	0.9101415
14	TCGA−HU−A4GN	0.129726607	0.036840026	0.090898121	0.134379177	0.04122602	0.000000000	0.021642210	0.000000000	0.095054345	0e+00	0.000000000	0.02	0.208818745	1.0237388
15	TCGA−HU−A4GP	0.018621051	0.059602720	0.154075940	0.046346661	0.18940057	0.010292281	0.000000000	0.071494803	0.072918110	0e+00	0.003918710	0.15	0.094110653	1.0490154
16	TCGA−HU−A4GC	0.024797631	0.000000000	0.000000000	0.088177448	0.25727980	0.024517289	0.000000000	0.110578651	0.000000000	0e+00	0.001464406	0.05	0.159900046	1.0237315
17	TCGA−D7−6527	0.011320297	0.018697144	0.143890430	0.084836050	0.11309403	0.000000000	0.012292802	0.083807244	0.042665149	0e+00	0.066649464	0.05	0.171491477	1.0136027
18	TCGA−D7−6524	0.004440849	0.021931593	0.092658475	0.012792724	0.04692258	0.017343943	0.014907379	0.000000000	0.368669312	0e+00	0.097897994	0.01	0.286926781	0.9958396
19	TCGA−D7−6528	0.000000000	0.000000000	0.332505619	0.033397151	0.15864186	0.000000000	0.002486398	0.000000000	0.136107622	0e+00	0.000000000	0.05	0.149170551	1.0543155
20	TCGA−D7−6522	0.035614824	0.004814687	0.061608075	0.048498751	0.21316781	0.034657293	0.000000000	0.074075355	0.011300492	0e+00	0.000000000	0.00	0.451684809	0.8925605
21	TCGA−FP−8631	0.058870185	0.002991800	0.027673542	0.005404669	0.08285754	0.000000000	0.132156302	0.145151625	0.029772539	0e+00	0.030730126	0.19	0.067778098	1.0506733
22	TCGA−HU−8238	0.051860577	0.022048654	0.102663873	0.087085669	0.09743706	0.000000000	0.018655810	0.079312016	0.096721557	0e+00	0.013155654	0.01	0.287086798	0.9675695
23	TCGA−HU−A4GF	0.040639709	0.018991398	0.231907693	0.094555217	0.13086905	0.000000000	0.006649011	0.009005631	0.000000000	0e+00	0.053859848	0.03	0.191985384	1.0167942
24	TCGA−HU−A4G3	0.152129963	0.014175706	0.067817759	0.070935911	0.13922137	0.000000000	0.000000000	0.199044177	0.010702026	0e+00	0.012044578	0.04	0.180516680	1.0219975
25	TCGA−D7−5578	0.034566154	0.027618896	0.046171131	0.016014391	0.12390567	0.023791036	0.000000000	0.034653600	0.110912773	0e+00	0.000000000	0.11	0.123451773	1.0408982
26	TCGA−D7−6820	0.019774365	0.006933973	0.202426887	0.000000000	0.09986221	0.000000000	0.058389923	0.000000000	0.161901352	0e+00	0.040839213	0.05	0.161817681	1.0300912
27	TCGA−HU−A4GJ	0.024874940	0.000000000	0.116648868	0.017230603	0.07538183	0.000000000	0.014071051	0.020167550	0.001660499	0e+00	0.000000000	0.01	0.219045181	1.0038090
28	TCGA−HU−8610	0.000000000	0.007643044	0.138230321	0.010532936	0.13296280	0.008537069	0.066464415	0.000000000	0.202301118	0e+00	0.019148987	0.00	0.456070304	0.8891595
29	TCGA−HU−A4G9	0.092601616	0.028924204	0.092382621	0.062623602	0.10804361	0.000000000	0.003485399	0.089188043	0.000000000	0e+00	0.003830803	0.15	0.093276597	1.0579405
30	TCGA−HU−A4GH	0.103861605	0.005083274	0.020247074	0.048579833	0.13352499	0.016315844	0.000000000	0.075076272	0.000000000	0e+00	0.020890441	0.22	0.063877345	1.0690210
31	TCGA−D7−6525	0.064417399	0.000000000	0.093315796	0.026026828	0.33032738	0.000000000	0.023870933	0.087933903	0.000000000	0e+00	0.018702496	0.06	0.136231496	1.0328257
32	TCGA−BR−6705	0.050356392	0.037996018	0.023657979	0.060950590	0.15804443	0.027739016	0.013459701	0.120602064	0.000000000	0e+00	0.000000000	0.06	0.136824770	1.0246181
33	TCGA−D7−6815	0.015216321	0.000000000	0.221361600	0.047001668	0.20575253	0.013961710	0.000000000	0.078281727	0.016081624	0e+00	0.000000000	0.12	0.108153586	1.0448094
34	TCGA−BR−6852	0.094471187	0.001859988	0.145461939	0.098475911	0.13862540	0.000000000	0.000000000	0.031426354	0.000000000	0e+00	0.001084423	0.01	0.367246040	0.9363512
35	TCGA−BR−6710	0.031645639	0.009991061	0.029315216	0.049687381	0.14612124	0.000000000	0.005791041	0.043344469	0.079186691	0e+00	0.006955627	0.05	0.141893998	1.0369260
36	TCGA−BR−6564	0.060502514	0.021263925	0.083978187	0.095916934	0.22055376	0.007199252	0.000400000	0.005969530	0.040446787	0e+00	0.000000000	0.01	0.223068412	0.9998417
37	TCGA−D7−6817	0.012468276	0.009373587	0.065213660	0.033302490	0.07704965	0.000000000	0.003042348	0.103126007	0.034534756	0e+00	0.004007765	0.14	0.100335947	1.0479394
38	TCGA−HU−8602	0.000000000	0.000000000	0.309660845	0.077227211	0.13688228	0.000000000	0.000000000	0.000000000	0.099101675	0e+00	0.027305753	0.01	0.363665478	0.9405834
39	TCGA−BR−4255	0.075142368	0.000300000	0.041553530	0.047488458	0.17399177	0.013515300	0.010017108	0.136846248	0.000000000	0e+00	0.000000000	0.04	0.184620651	1.0117651
40	TCGA−BR−4187	0.026925057	0.037284328	0.000000000	0.019914460	0.27996684	0.000000000	0.006586027	0.233261583	0.000000000	0e+00	0.007627309	0.01	0.371213158	0.9286697
41	TCGA−BR−4257	0.051851456	0.014653393	0.110148500	0.058097336	0.25724227	0.024080698	0.000000000	0.000000000	0.036516040	0e+00	0.000000000	0.01	0.285356844	0.9708599
42	TCGA−BR−4267	0.016491879	0.000000000	0.187716762	0.064139182	0.12752028	0.004444570	0.000000000	0.000000000	0.099996602	0e+00	0.021892791	0.05	0.144606052	1.0342446
43	TCGA−HU−A4HB	0.043669388	0.000000000	0.051137889	0.034270565	0.10574756	0.000000000	0.003815387	0.072492049	0.000000000	0e+00	0.000000000	0.01	0.367621088	0.9321361
44	TCGA−HU−A4H3	0.067294826	0.027434004	0.053933340	0.103211191	0.09381016	0.000000000	0.000000000	0.000000000	0.203027253	0e+00	0.004858975	0.01	0.222455159	1.0105756
45	TCGA−HU−A4G8	0.058732526	0.000000000	0.061123912	0.135954315	0.14442013	0.009790832	0.000000000	0.000000000	0.016191858	0e+00	0.000000000	0.01	0.293171097	0.9726872
46	TCGA−BR−A4PE	0.023908373	0.000000000	0.317092607	0.102694822	0.12720873	0.000000000	0.000000000	0.000000000	0.173219029	0e+00	0.007422093	0.01	0.225569817	1.0212432
47	TCGA−HU−A4GX	0.092132050	0.006631387	0.000000000	0.145672040	0.14036607	0.000000000	0.000000000	0.079358950	0.003201049	0e+00	0.000700000	0.01	0.340308950	0.9577025
48	TCGA−HU−A4H0	0.042205940	0.005601790	0.008382827	0.149749527	0.24884109	0.013916791	0.002146117	0.051593648	0.000000000	0e+00	0.000000000	0.01	0.384140276	0.9253624
49	TCGA−HU−A4GU	0.015010143	0.004905611	0.207616601	0.024560211	0.19645400	0.000000000	0.000000000	0.000000000	0.140260593	0e+00	0.016011753	0.17	0.083013818	1.0675803
50	TCGA−D7−6526	0.000000000	0.019948385	0.065097335	0.021462363	0.13797398	0.009689262	0.026500704	0.000000000	0.094947608	0e+00	0.007161308	0.11	0.113223155	1.0405653
51	TCGA−D7−8578	0.100028622	0.006816267	0.012229012	0.038976682	0.17561448	0.000000000	0.012520096	0.058511204	0.077393577	0e+00	0.000000000	0.15	0.095644187	1.0633477
52	TCGA−BR−8286	0.000000000	0.000000000	0.070328244	0.045950020	0.16149124	0.031301377	0.022835310	0.000000000	0.154817576	0e+00	0.013212189	0.07	0.132660603	1.0394746
53	TCGA−D7−8579	0.064187551	0.059082475	0.032070848	0.067208039	0.11523839	0.000000000	0.000000000	0.162025157	0.031176215	0e+00	0.011420556	0.02	0.201599418	1.0047072
54	TCGA−BR−8365	0.061137208	0.021184385	0.000000000	0.058514967	0.19724797	0.000000000	0.002118844	0.117535660	0.000800000	0e+00	0.000000000	0.03	0.189272767	1.0125754
55	TCGA−CD−8529	0.036319431	0.012497213	0.095612061	0.072840806	0.19544421	0.000500000	0.000000000	0.011688604	0.008087976	0e+00	0.010616415	0.03	0.194383117	1.0096496
56	TCGA−BR−8366	0.131726282	0.000000000	0.075519390	0.132913121	0.23649569	0.013296786	0.000000000	0.002933937	0.000000000	0e+00	0.000000000	0.00	0.446821898	0.8952809
57	TCGA−BR−8060	0.051028073	0.050745952	0.100269310	0.106529573	0.21316487	0.001903073	0.000000000	0.000000000	0.146180852	0e+00	0.019464544	0.01	0.241857617	0.9938281
58	TCGA−HU−8244	0.048466794	0.000000000	0.005340870	0.014827026	0.26167081	0.017314296	0.000000000	0.000000000	0.109325739	0e+00	0.000000000	0.17	0.081940070	1.0636652
59	TCGA−BR−8080	0.042658030	0.036466416	0.146788204	0.046175925	0.14892211	0.000000000	0.000000000	0.000000000	0.131658141	0e+00	0.000100000	0.01	0.230862138	0.9952631
60	TCGA−HU−A4G6	0.001135651	0.055859990	0.104696550	0.081900807	0.12983489	0.014210332	0.034621619	0.000000000	0.047281257	0e+00	0.000000000	0.18	0.075354387	1.0584039
61	TCGA−BR−A4CR	0.000000000	0.019688952	0.144368564	0.027474547	0.19256801	0.000000000	0.006126107	0.000000000	0.142962347	0e+00	0.000000000	0.57	0.008542091	1.0869015
62	TCGA−BR−8373	0.024079083	0.046477435	0.178476159	0.082290455	0.09415352	0.000000000	0.008491563	0.000000000	0.093205352	0e+00	0.026068738	0.21	0.064341185	1.0611576
63	TCGA−CD−8532	0.048614561	0.005660773	0.000000000	0.118076212	0.12184321	0.000000000	0.014846341	0.000000000	0.135850929	0e+00	0.000000000	0.01	0.355774543	0.9448650
64	TCGA−BR−7716	0.080744106	0.000000000	0.063111497	0.135099921	0.23947104	0.004465255	0.000000000	0.000000000	0.020501652	0e+00	0.000000000	0.01	0.350469289	0.9437464
65	TCGA−FP−7916	0.041907331	0.012267448	0.037588032	0.116380607	0.23511872	0.000000000	0.003773599	0.099823076	0.023554796	0e+00	0.008215392	0.01	0.223191854	0.9961048
66	TCGA−IP−7968	0.072513272	0.011075366	0.045586381	0.089671161	0.10625401	0.046975632	0.000000000	0.000000000	0.092441304	0e+00	0.000000000	0.01	0.277325343	0.9796545
67	TCGA−F1−6875	0.053650813	0.026811791	0.000000000	0.014834485	0.20600791	0.000000000	0.029008940	0.129481882	0.000000000	0e+00	0.000000000	0.05	0.162979322	1.0165192
68	TCGA−IN−7806	0.058942868	0.000000000	0.156573324	0.093483180	0.16177444	0.003760972	0.000000000	0.000000000	0.060602790	0e+00	0.000000000	0.02	0.203216733	1.0111747
69	TCGA−BR−7717	0.010400183	0.000000000	0.244486037	0.075349674	0.07984546	0.000000000	0.000000000	0.000000000	0.131475116	0e+00	0.000000000	0.01	0.278882518	0.9778577
70	TCGA−B7−5818	0.037981014	0.017684989	0.061795917	0.078580687	0.27767168	0.025503750	0.000000000	0.000000000	0.008286183	0e+00	0.000000000	0.03	0.200127582	1.0060623
71	TCGA−D7−5577	0.062558483	0.005941314	0.000000000	0.125195689	0.23385661	0.008302008	0.004112922	0.004588668	0.043901391	0e+00	0.000000000	0.01	0.383158200	0.9277526
72	TCGA−CD−5801	0.037142052	0.000000000	0.008377910	0.107952923	0.18736874	0.002111370	0.000000000	0.000000000	0.061894855	0e+00	0.002567416	0.01	0.367896929	0.9370779
73	TCGA−BR−6566	0.072542631	0.001617174	0.128232924	0.088218641	0.19065348	0.020045370	0.000000000	0.000000000	0.049662570	0e+00	0.010760915	0.01	0.295463204	0.9684070
74	TCGA−FP−8209	0.000000000	0.013146670	0.041342865	0.030665709	0.09556855	0.000000000	0.026453503	0.008750282	0.057619135	0e+00	0.010078232	0.01	0.289468303	0.9688927
75	TCGA−BR−8367	0.009128285	0.015914735	0.048163865	0.046869156	0.21554726	0.034549914	0.000000000	0.210883286	0.025613437	0e+00	0.000000000	0.12	0.108784473	1.0383859
76	TCGA−BR−8289	0.015305919	0.000000000	0.121197973	0.030270566	0.24105452	0.000000000	0.000000000	0.149341735	0.023543599	0e+00	0.000900000	0.09	0.128462275	1.0300198
77	TCGA−BR−8371	0.041427342	0.014220497	0.040045021	0.017079242	0.07925202	0.000000000	0.027887382	0.016945270	0.000000000	0e+00	0.000000000	0.01	0.326457182	0.9597628
78	TCGA−CD−8526	0.053083166	0.028953208	0.067658913	0.049355495	0.09252506	0.001837253	0.028361707	0.056067750	0.000000000	0e+00	0.000000000	0.07	0.131687125	1.0332688
79	TCGA−BR−8368	0.060502514	0.021263925	0.083978187	0.095916934	0.22055376	0.007199252	0.000400000	0.005969530	0.040446787	0e+00	0.000000000	0.01	0.223068412	0.9998417
80	TCGA−D7−8573	0.009850768	0.043551892	0.007755584	0.084905684	0.02322030	0.014436526	0.000000000	0.000000000	0.076639861	0e+00	0.000000000	0.18	0.073412964	1.0776201
81	TCGA−BR−7722	0.028318383	0.000000000	0.044913672	0.018740379	0.27728162	0.015417175	0.008462240	0.190135240	0.041548724	0e+00	0.000000000	0.01	0.210865505	1.0002097
82	TCGA−CD−5804	0.047833434	0.032725842	0.121373549	0.116848632	0.23813559	0.000000000	0.000000000	0.010371837	0.000000000	0e+00	0.026681651	0.01	0.271091748	0.9797156
83	TCGA−BR−6563	0.034549630	0.022369446	0.061831495	0.056875699	0.15100289	0.015425546	0.000000000	0.199183743	0.000000000	0e+00	0.000000000	0.01	0.298384145	0.9605764
84	TCGA−D7−6818	0.034351498	0.098806298	0.040536771	0.003552177	0.43075578	0.007581424	0.000000000	0.076078555	0.029160658	0e+00	0.035340305	0.04	0.185584446	1.0190774
85	TCGA−BR−6709	0.070016921	0.029186658	0.020912573	0.070245289	0.30375766	0.000000000	0.018703061	0.011457841	0.000000000	0e+00	0.000000000	0.01	0.297023745	0.9665611
86	TCGA−BR−6803	0.057897106	0.008918347	0.059149878	0.076941202	0.15874461	0.007694552	0.000000000	0.000000000	0.048136453	0e+00	0.000000000	0.01	0.240300518	0.9964005
87	TCGA−D7−6822	0.096236372	0.015343083	0.160637734	0.089121995	0.13681105	0.028800811	0.000000000	0.000000000	0.042591598	0e+00	0.000000000	0.05	0.148684467	1.0354313
88	TCGA−D7−6521	0.025012452	0.005146631	0.042291186	0.058964323	0.19165262	0.007040314	0.000000000	0.055553003	0.025437005	0e+00	0.007302661	0.01	0.297598455	0.9631565
89	TCGA−CD−8524	0.000000000	0.000000000	0.000000000	0.155123543	0.14303343	0.029919534	0.000000000	0.034258977	0.030639517	0e+00	0.000000000	0.01	0.219393328	1.0034273
90	TCGA−CD−8530	0.027096865	0.015958412	0.015183682	0.042114372	0.27118568	0.050134403	0.000000000	0.036083765	0.139478769	0e+00	0.000000000	0.01	0.300510703	0.9622539
91	TCGA−FP−8211	0.111257455	0.005766465	0.024227614	0.058753795	0.06612386	0.001111254	0.014780266	0.000000000	0.162456386	0e+00	0.009292873	0.03	0.191480796	1.0363438
92	TCGA−CD−A487	0.110335552	0.000000000	0.010026399	0.052272978	0.07533230	0.000000000	0.092912569	0.311471631	0.000000000	0e+00	0.080341954	0.05	0.155841530	1.0339679
93	TCGA−CD−A489	0.046272419	0.000000000	0.000000000	0.063512935	0.25881976	0.008385389	0.000000000	0.067202513	0.000000000	0e+00	0.000000000	0.04	0.185533824	1.0136753
94	TCGA−D7−8570	0.060502514	0.021263925	0.083978187	0.095916934	0.22055376	0.007199252	0.000400000	0.005969530	0.040446787	0e+00	0.000000000	0.01	0.223068412	0.9998417
95	TCGA−CD−8528	0.060502514	0.021263925	0.083978187	0.095916934	0.22055376	0.007199252	0.000400000	0.005969530	0.040446787	0e+00	0.000000000	0.01	0.223068412	0.9998417
96	TCGA−CD−8531	0.041997414	0.000000000	0.054441962	0.063114923	0.15195304	0.051163216	0.051901041	0.000000000	0.010409276	0e+00	0.000000000	0.01	0.296084648	0.9602556
97	TCGA−BR−8078	0.067817120	0.008300238	0.248016337	0.100978779	0.17698830	0.000000000	0.000000000	0.000000000	0.077114659	0e+00	0.021431529	0.01	0.393100593	0.9250149
98	TCGA−BR−8077	0.070088549	0.013377714	0.136180620	0.049692797	0.15211910	0.019546517	0.024043581	0.027579281	0.092338169	0e+00	0.009513135	0.03	0.188866461	1.0119997
99	TCGA−CD−8534	0.037174954	0.000000000	0.143078515	0.168492582	0.25897279	0.000000000	0.000000000	0.001777751	0.009861015	0e+00	0.004918123	0.01	0.347014589	0.9485210
100	TCGA−FP−8099	0.000500000	0.036024170	0.120487426	0.032389383	0.07895560	0.000000000	0.012783385	0.000000000	0.149844768	9e−04	0.002491354	0.15	0.092352416	1.0526933

	Input Sample	B cells naive	B cells memory	Plasma cells	T cells CD8	T cells CD4 naive	T cells CD4 memory resting	T cells CD4 memory activated	T cells follicular helper	T cells regulatory (Tregs)	T cells gamma delta	NK cells resting
1	TCGA−BR−8059	0.000000000	0.000000000	0.038098330	0.05524027	0	0.11895393	0.000000000	0.018160493	0.08815364	0.000000000	0.032184418
2	TCGA−BR−8679	0.014447518	0.000000000	0.000000000	0.03886943	0	0.24182546	0.000000000	0.000100000	0.13761681	0.000000000	0.000000000
3	TCGA−HU−8604	0.038491382	0.000000000	0.015361649	0.12182109	0	0.13566951	0.047221185	0.113311547	0.07439332	0.000000000	0.068729316
4	TCGA−BR−8382	0.000000000	0.037774043	0.000000000	0.07266210	0	0.19594863	0.015279375	0.070247009	0.02749524	0.000000000	0.000000000
5	TCGA−BR−8588	0.042846788	0.050054682	0.000000000	0.15930277	0	0.08902804	0.000000000	0.113369002	0.06218572	0.000000000	0.000000000
6	TCGA−HU−8608	0.005248345	0.016289973	0.028640965	0.17154532	0	0.10443501	0.034392440	0.077492867	0.07155343	0.000000000	0.031762184
7	TCGA−IN−8663	0.000000000	0.000000000	0.011188264	0.22544055	0	0.08069007	0.014218058	0.025689795	0.05941537	0.000000000	0.000000000
8	TCGA−BR−8683	0.063277827	0.001918881	0.000000000	0.17064511	0	0.17172455	0.000000000	0.093774545	0.06561203	0.000000000	0.000000000
9	TCGA−BR−8687	0.084094244	0.000000000	0.000000000	0.06277715	0	0.25546487	0.000000000	0.043466775	0.04033251	0.000000000	0.000000000
10	TCGA−BR−8384	0.066315087	0.017696670	0.000000000	0.12675926	0	0.20723980	0.000000000	0.008015429	0.03997484	0.000000000	0.000000000
11	TCGA−BR−8590	0.038743255	0.013835556	0.010522899	0.06501975	0	0.24138030	0.000000000	0.027673935	0.03188496	0.000000000	0.000000000
12	TCGA−BR−8381	0.004410640	0.056365737	0.000000000	0.24975800	0	0.07843233	0.065540658	0.056204825	0.09374483	0.000000000	0.000000000
13	TCGA−BR−8680	0.160443488	0.000000000	0.049021392	0.17040971	0	0.00000000	0.006711672	0.165311406	0.06339987	0.000000000	0.019480447
14	TCGA−BR−8677	0.013623845	0.021336061	0.011674262	0.25483477	0	0.05609033	0.000000000	0.094071271	0.10970550	0.000000000	0.000000000
15	TCGA−BR−8483	0.004824277	0.024643534	0.081702524	0.08189206	0	0.15039930	0.000000000	0.046066377	0.05515418	0.000000000	0.000000000
16	TCGA−HU−A4HD	0.000000000	0.042235105	0.000000000	0.03685320	0	0.14688292	0.000000000	0.023515531	0.07118900	0.000000000	0.000000000
17	TCGA−HU−A4H4	0.000000000	0.015696482	0.008232158	0.17760794	0	0.05241291	0.045907727	0.095596060	0.07450275	0.000000000	0.000000000
18	TCGA−HU−A4H6	0.174971482	0.000000000	0.000000000	0.07435014	0	0.11883372	0.000000000	0.047777544	0.05769619	0.000000000	0.000000000
19	TCGA−D7−A4YV	0.017521652	0.016864867	0.005251893	0.20486488	0	0.17815780	0.000000000	0.067636436	0.04771344	0.011267824	0.000000000
20	TCGA−HU−A4H5	0.012098399	0.000000000	0.070794191	0.05903878	0	0.07505878	0.000000000	0.040889149	0.06582847	0.000000000	0.041643893
21	TCGA−BR−A4QI	0.000000000	0.000000000	0.000000000	0.13912108	0	0.06494312	0.014492336	0.096590756	0.09812571	0.000000000	0.000000000
22	TCGA−HU−A4H2	0.000000000	0.040965767	0.020368233	0.13229905	0	0.12082254	0.000000000	0.052452176	0.12325146	0.000000000	0.000000000
23	TCGA−BR−6801	0.010735184	0.040645637	0.000000000	0.01780122	0	0.15211049	0.000000000	0.087120143	0.07243380	0.000000000	0.057064498
24	TCGA−F1−6874	0.025868158	0.002289085	0.000000000	0.07714535	0	0.31679271	0.090518366	0.000000000	0.00000000	0.000000000	0.025831050
25	TCGA−BR−6706	0.008117787	0.000000000	0.008877342	0.24817074	0	0.09946555	0.063692675	0.073925353	0.06186078	0.000000000	0.000000000
26	TCGA−BR−6802	0.031013019	0.026541388	0.000000000	0.21799207	0	0.01650570	0.007341547	0.162629469	0.08784157	0.000000000	0.000000000
27	TCGA−CD−8535	0.000000000	0.006334801	0.001228591	0.00000000	0	0.16062733	0.000000000	0.091306524	0.06198283	0.000000000	0.032561023
28	TCGA−BR−8362	0.014224035	0.000000000	0.009450959	0.15876942	0	0.13227161	0.000000000	0.027344540	0.09923933	0.000000000	0.000000000
29	TCGA−D7−8576	0.000000000	0.000000000	0.052267996	0.08236038	0	0.08840111	0.000000000	0.044739631	0.01604028	0.000000000	0.000000000
30	TCGA−CD−A48C	0.000000000	0.040521512	0.000000000	0.19777476	0	0.11529304	0.012686409	0.092168022	0.03937671	0.001959560	0.000000000
31	TCGA−CD−8533	0.000000000	0.000000000	0.050848583	0.03462699	0	0.25126818	0.000000000	0.000000000	0.12729348	0.000000000	0.043340759
32	TCGA−HU−A4GY	0.055770417	0.042837085	0.000000000	0.15090421	0	0.16377876	0.001357341	0.053363190	0.07705802	0.008577246	0.000000000
33	TCGA−BR−A44T	0.230266929	0.180997496	0.000000000	0.11132613	0	0.17191593	0.000000000	0.026143360	0.05284194	0.000000000	0.000000000
34	TCGA−BR−8297	0.147333039	0.010598314	0.000000000	0.03523975	0	0.23286882	0.000000000	0.068816918	0.08932348	0.000000000	0.000000000
35	TCGA−EQ−8122	0.016002741	0.020443445	0.003939265	0.13269178	0	0.07161212	0.000000000	0.072459885	0.06659392	0.000000000	0.000000000
36	TCGA−D7−8575	0.000000000	0.062663755	0.009072922	0.07448171	0	0.13922137	0.000000000	0.034174875	0.03432851	0.000000000	0.000000000
37	TCGA−BR−8284	0.023600706	0.000000000	0.001887573	0.28224270	0	0.12870635	0.018021386	0.041046618	0.06695596	0.001299322	0.000000000
38	TCGA−FP−7735	0.082060908	0.083342667	0.000000000	0.07889897	0	0.12881929	0.000000000	0.100255482	0.05073680	0.033702340	0.000000000
39	TCGA−BR−7723	0.011997526	0.009686579	0.003983895	0.12190024	0	0.13721866	0.000000000	0.099203952	0.03827879	0.000000000	0.000000000
40	TCGA−CD−5813	0.084578755	0.000000000	0.000000000	0.07053389	0	0.08341758	0.000000000	0.086302470	0.02306305	0.036829457	0.000000000
41	TCGA−B7−5816	0.021435672	0.057941079	0.000000000	0.06371543	0	0.10225360	0.000000000	0.105244328	0.06061294	0.000000000	0.003564771
42	TCGA−D7−A4YY	0.000000000	0.023488984	0.000000000	0.09214375	0	0.07321929	0.000000000	0.036505084	0.09735253	0.000000000	0.000000000
43	TCGA−D7−A4YX	0.013589541	0.000000000	0.007008052	0.26540268	0	0.02078620	0.080231132	0.088715680	0.03787235	0.033150958	0.000000000
44	TCGA−D7−A4YU	0.086353756	0.041778666	0.000000000	0.10403167	0	0.20176027	0.058465593	0.047817725	0.03998991	0.000000000	0.000000000
45	TCGA−D7−6520	0.038693435	0.000000000	0.040632374	0.06576761	0	0.21333254	0.000000000	0.098644823	0.07288093	0.000000000	0.000000000
46	TCGA−BR−6565	0.009037616	0.029020979	0.000000000	0.17739877	0	0.05107016	0.097931860	0.079096173	0.03925564	0.003914007	0.000000000
47	TCGA−BR−8484	0.043685713	0.024632185	0.000000000	0.08436194	0	0.26908890	0.001041498	0.049075737	0.04738968	0.000000000	0.000000000
48	TCGA−BR−8487	0.024303525	0.075483112	0.005411890	0.07851221	0	0.18040742	0.000000000	0.077687622	0.05699029	0.009105578	0.000000000
49	TCGA−BR−8682	0.048800662	0.029545568	0.000000000	0.05963439	0	0.08223309	0.000000000	0.048832292	0.07781876	0.000000000	0.000000000
50	TCGA−BR−8485	0.052195449	0.000000000	0.016028773	0.27453610	0	0.23953211	0.000000000	0.047249092	0.04898173	0.004568132	0.000000000
51	TCGA−BR−8058	0.017180916	0.000000000	0.000500000	0.14775159	0	0.30422751	0.000000000	0.039864561	0.06646168	0.000000000	0.000000000
52	TCGA−BR−8369	0.099123686	0.012898212	0.003755656	0.06283107	0	0.19485739	0.000000000	0.066603059	0.12377505	0.000000000	0.000000000
53	TCGA−CD−8536	0.035338491	0.000000000	0.006405932	0.21294837	0	0.16281746	0.096320133	0.060970090	0.00000000	0.000000000	0.003606934
54	TCGA−D7−8572	0.014371667	0.000000000	0.005420585	0.08764196	0	0.14983491	0.000000000	0.048487903	0.04687111	0.000000000	0.000000000
55	TCGA−CD−A486	0.000000000	0.013516201	0.022295646	0.05833845	0	0.07511821	0.000000000	0.064466256	0.06141996	0.000000000	0.000000000
56	TCGA−FP−A4BE	0.000000000	0.000000000	0.008029078	0.11136247	0	0.07230833	0.101195664	0.104716241	0.05048301	0.003442091	0.021540694
57	TCGA−BR−8361	0.005485149	0.000000000	0.000000000	0.26825615	0	0.01223394	0.014888906	0.120308652	0.11160825	0.027178676	0.000000000
58	TCGA−BR−A453	0.013831529	0.000000000	0.004917410	0.12666564	0	0.12759810	0.000000000	0.050149205	0.02563804	0.079906291	0.000000000
59	TCGA−F1−A448	0.033199681	0.063480543	0.000000000	0.07089720	0	0.16554123	0.000000000	0.056989299	0.03798825	0.000000000	0.000000000
60	TCGA−BR−A4CS	0.007334367	0.000000000	0.043044078	0.04384885	0	0.14433981	0.000000000	0.045166207	0.04707664	0.000000000	0.000000000
61	TCGA−BR−8291	0.037317544	0.000000000	0.006141847	0.10731052	0	0.16818940	0.000000000	0.035269783	0.04420968	0.000000000	0.000000000
62	TCGA−BR−8081	0.070333087	0.000000000	0.000000000	0.09626058	0	0.13172662	0.024745109	0.095181794	0.05116266	0.000000000	0.000000000
63	TCGA−D7−8574	0.013824385	0.312481360	0.000000000	0.12728036	0	0.21695897	0.000000000	0.006808652	0.10728222	0.000000000	0.000000000
64	TCGA−FP−8210	0.056887480	0.082610163	0.000000000	0.10804438	0	0.15047594	0.000000000	0.117581960	0.04250105	0.040497544	0.000000000
65	TCGA−BR−4280	0.018822490	0.000000000	0.060440385	0.09139405	0	0.15104398	0.000000000	0.139501745	0.10710306	0.000000000	0.000000000
66	TCGA−BR−4292	0.005395327	0.100094289	0.000000000	0.14831402	0	0.17661501	0.000000000	0.036891007	0.08972874	0.000000000	0.000000000
67	TCGA−BR−4256	0.059905755	0.000000000	0.003246766	0.09225677	0	0.20901938	0.000000000	0.047843119	0.04526946	0.000000000	0.000000000
68	TCGA−BR−7901	0.006924397	0.045202848	0.000000000	0.07257846	0	0.15661003	0.000000000	0.028264362	0.09674924	0.000000000	0.017650977
69	TCGA−BR−7707	0.000000000	0.005698322	0.000000000	0.05279580	0	0.02715505	0.000000000	0.072881575	0.12314256	0.022319038	0.000000000
70	TCGA−BR−7715	0.007611316	0.000000000	0.009765664	0.02816629	0	0.08904491	0.000000000	0.050684841	0.05040566	0.002163216	0.000000000
71	TCGA−CD−5803	0.019816579	0.077514210	0.000000000	0.14436197	0	0.18294500	0.000000000	0.074696374	0.08613745	0.000000000	0.000000000
72	TCGA−CD−5800	0.113980441	0.000000000	0.014940825	0.06865266	0	0.08773742	0.000000000	0.097664908	0.05525449	0.000000000	0.000000000
73	TCGA−BR−A4PF	0.053223752	0.000000000	0.015957999	0.04905419	0	0.13305069	0.029577617	0.050502562	0.07435044	0.000000000	0.003077512
74	TCGA−HU−A4H8	0.000000000	0.073423899	0.000000000	0.07235097	0	0.18001231	0.009782162	0.110077525	0.05205886	0.000000000	0.000000000
75	TCGA−D7−A4YT	0.062278073	0.000000000	0.061324147	0.03750461	0	0.41219634	0.000000000	0.066422704	0.10204945	0.000000000	0.000000000
76	TCGA−HU−A4GT	0.009399068	0.000000000	0.000000000	0.07209187	0	0.22520958	0.000000000	0.048247765	0.11725455	0.000000000	0.000000000
77	TCGA−BR−A4PD	0.000000000	0.012826825	0.022636069	0.04394684	0	0.23073488	0.000000000	0.046234068	0.08931934	0.000000000	0.000000000
78	TCGA−BR−A452	0.040571885	0.107439619	0.000000000	0.15083500	0	0.09199258	0.000000000	0.092351803	0.19017305	0.000000000	0.000000000
79	TCGA−HU−A4G2	0.025518758	0.000000000	0.000000000	0.16308449	0	0.14823106	0.036502725	0.058911460	0.04187429	0.000000000	0.000000000
80	TCGA−EQ−A4SO	0.011639413	0.022011690	0.000200000	0.10445896	0	0.31516209	0.000000000	0.057669488	0.12918973	0.000000000	0.000000000
81	TCGA−CD−5798	0.009477173	0.056856979	0.000000000	0.06255822	0	0.18243524	0.000000000	0.035932018	0.04735218	0.000000000	0.000000000
82	TCGA−CD−5799	0.002470999	0.017957086	0.023054112	0.05949450	0	0.16643772	0.000000000	0.062640753	0.09199879	0.000000000	0.000000000
83	TCGA−D7−6518	0.037167064	0.012337212	0.000000000	0.07000774	0	0.17320335	0.000000000	0.097698021	0.03117748	0.011151752	0.000000000
84	TCGA−D7−6519	0.004107668	0.003240558	0.000000000	0.19820619	0	0.05630208	0.000000000	0.051101583	0.05609723	0.000000000	0.000000000
85	TCGA−F1−6177	0.049194097	0.026180870	0.000000000	0.12943109	0	0.21017278	0.000000000	0.048039662	0.05531749	0.000000000	0.000000000
86	TCGA−BR−6452	0.017300033	0.000000000	0.000000000	0.06683990	0	0.12710458	0.001138927	0.064603815	0.06592489	0.000000000	0.032426889
87	TCGA−BR−6453	0.174178737	0.030396790	0.000000000	0.15609849	0	0.07307696	0.014751614	0.084673912	0.11047184	0.000000000	0.000000000
88	TCGA−BR−6454	0.076147659	0.000000000	0.005683919	0.17111953	0	0.19618034	0.000000000	0.167730244	0.04216363	0.018127492	0.000000000
89	TCGA−BR−6455	0.000000000	0.010001317	0.000000000	0.20964505	0	0.09841421	0.010473958	0.088479934	0.07590662	0.000000000	0.000000000
90	TCGA−BR−6456	0.048065787	0.000000000	0.000000000	0.06270262	0	0.15122048	0.000000000	0.034558090	0.07954553	0.000000000	0.000000000
91	TCGA−BR−6457	0.052688896	0.104998378	0.000000000	0.08267641	0	0.15029461	0.000000000	0.112116651	0.05598274	0.000500000	0.000000000
92	TCGA−BR−6458	0.009245089	0.019824442	0.000000000	0.07822654	0	0.23511481	0.000000000	0.066629143	0.04243223	0.000000000	0.000000000
93	TCGA−BR−7196	0.095082521	0.000000000	0.000000000	0.16185440	0	0.20108577	0.000000000	0.017965607	0.04718490	0.000000000	0.027268478
94	TCGA−BR−7703	0.017259821	0.029591676	0.003254790	0.13460695	0	0.12281088	0.000000000	0.063631946	0.09264691	0.000000000	0.000000000
95	TCGA−FP−7829	0.017837904	0.012893279	0.000000000	0.00000000	0	0.13167972	0.000000000	0.063080684	0.05170673	0.000000000	0.000000000
96	TCGA−BR−7197	0.010848098	0.014038896	0.000000000	0.00000000	0	0.09191760	0.000000000	0.032779697	0.14488773	0.000000000	0.049643380
97	TCGA−BR−7851	0.084323828	0.000000000	0.000000000	0.03975152	0	0.17369203	0.000000000	0.093607100	0.06167295	0.000000000	0.028824767
98	TCGA−BR−7957	0.037199507	0.000000000	0.000200000	0.10105724	0	0.18304039	0.000000000	0.064435271	0.06848792	0.000000000	0.000000000
99	TCGA−FP−7998	0.032599875	0.000000000	0.009760754	0.23235219	0	0.11282128	0.111046941	0.089120986	0.05231239	0.000000000	0.000000000
100	TCGA−BR−7958	0.013242347	0.000000000	0.016224285	0.21167869	0	0.00000000	0.024015962	0.084081224	0.06988187	0.000000000	0.000000000

	Input Sample	NK cells activated	Monocytes	Macrophages M0	Macrophages M1	Macrophages M2	Dendritic cells resting	Dendritic cells activated	Mast cells resting	Mast cells activated	Eosinophils	Neutrophils	P−value	Pearson Correlation	RMSE
1	TCGA−BR−8059	0.006369288	0.006580198	0.265860143	0.041397635	0.12768511	0.000000000	0.000000000	0.000000000	0.201316553	0	0.000000000	0.05	0.15816924	1.0435658
2	TCGA−BR−8679	0.046974243	0.000000000	0.078882930	0.023843948	0.36182999	0.016631376	0.000000000	0.038946871	0.000000000	0	0.000000000	0.12	0.10738626	1.0523922
3	TCGA−HU−8604	0.000000000	0.000000000	0.146461751	0.066803419	0.09993638	0.000000000	0.000000000	0.000000000	0.062507884	0	0.009291565	0.05	0.15030577	1.0304616
4	TCGA−BR−8382	0.149887953	0.031801313	0.029347900	0.085454929	0.11976084	0.015544834	0.008380619	0.000000000	0.110042593	0	0.030372627	0.01	0.38159428	0.9302497
5	TCGA−BR−8588	0.088530992	0.003674595	0.060012330	0.056903322	0.13121123	0.000000000	0.000500000	0.079324452	0.020383028	0	0.042654773	0.01	0.27520429	0.9743434
6	TCGA−HU−8608	0.002323288	0.000000000	0.056218227	0.184853934	0.18971392	0.000000000	0.019936857	0.000000000	0.002967772	0	0.002625459	0.01	0.37706060	0.9330743
7	TCGA−IN−8663	0.072435659	0.023348409	0.050718141	0.079992996	0.25664511	0.011366726	0.000000000	0.000000000	0.076942584	0	0.011908274	0.01	0.27208484	0.9807077
8	TCGA−BR−8683	0.075205898	0.007635479	0.000000000	0.081322348	0.14445139	0.017109991	0.000000000	0.107321953	0.000000000	0	0.000000000	0.15	0.09230799	1.0597620
9	TCGA−BR−8687	0.056742333	0.012177170	0.000000000	0.047188995	0.27287172	0.021999434	0.000000000	0.102884797	0.000000000	0	0.000000000	0.05	0.14578393	1.0251045
10	TCGA−BR−8384	0.074088161	0.020447904	0.000000000	0.031062375	0.18186770	0.000100000	0.011243347	0.215149746	0.000000000	0	0.000000000	0.01	0.26587273	0.9768457
11	TCGA−BR−8590	0.043263836	0.013013791	0.078608837	0.046521224	0.24277730	0.000000000	0.007235382	0.138380839	0.000000000	0	0.001138130	0.04	0.17782916	1.0103218
12	TCGA−BR−8381	0.034127042	0.020977475	0.073439104	0.124448829	0.11945308	0.014318390	0.000000000	0.008744864	0.000000000	0	0.000000000	0.01	0.27446332	0.9808115
13	TCGA−BR−8680	0.000000000	0.006167211	0.071354161	0.063987875	0.12646037	0.000000000	0.000000000	0.026369358	0.070883044	0	0.000000000	0.15	0.09087913	1.0476693
14	TCGA−BR−8677	0.102668912	0.017663157	0.000000000	0.140898986	0.15637425	0.000000000	0.000000000	0.021058655	0.000000000	0	0.000000000	0.01	0.31539597	0.9701728
15	TCGA−BR−8483	0.070089834	0.000000000	0.168566845	0.051029339	0.13232740	0.009088042	0.008456660	0.069085291	0.046674341	0	0.000000000	0.19	0.06669255	1.0579574
16	TCGA−HU−A4HD	0.054772293	0.032235492	0.090627136	0.031894573	0.20081577	0.014892499	0.000000000	0.173545171	0.075036160	0	0.005505146	0.05	0.17111645	1.0170566
17	TCGA−HU−A4H4	0.040171337	0.000000000	0.190259207	0.158128304	0.11523473	0.000000000	0.014283536	0.002792185	0.000000000	0	0.009174664	0.01	0.37588259	0.9334577
18	TCGA−HU−A4H6	0.064940276	0.004500365	0.131456320	0.060305980	0.07094649	0.000000000	0.000000000	0.077336691	0.065668614	0	0.051216191	0.01	0.27612317	0.9723218
19	TCGA−D7−A4YV	0.096713449	0.004948580	0.003969615	0.092405219	0.17458109	0.000000000	0.000000000	0.000000000	0.078103258	0	0.000000000	0.01	0.37420844	0.9354959
20	TCGA−HU−A4H5	0.000000000	0.000400000	0.367612950	0.033719076	0.13774025	0.000000000	0.000000000	0.000000000	0.081993660	0	0.013202760	0.01	0.23312466	1.0095089
21	TCGA−BR−A4QI	0.063351295	0.019290105	0.119146776	0.045887208	0.27803818	0.010726592	0.000500000	0.000000000	0.049809379	0	0.000000000	0.19	0.06695062	1.0707294
22	TCGA−HU−A4H2	0.067892453	0.017228450	0.098511084	0.061991718	0.10613524	0.027334875	0.000000000	0.102786355	0.021567424	0	0.006393163	0.11	0.11174588	1.0399435
23	TCGA−BR−6801	0.000000000	0.002640559	0.283792652	0.040144169	0.14036075	0.000000000	0.002841101	0.000000000	0.092309793	0	0.000000000	0.11	0.11651586	1.0539149
24	TCGA−F1−6874	0.016850950	0.016652949	0.092773231	0.079945326	0.15591006	0.000800000	0.007439310	0.000000000	0.072662371	0	0.018494306	0.03	0.19425509	1.0109135
25	TCGA−BR−6706	0.030057047	0.012020597	0.011795507	0.160960555	0.14797785	0.003696280	0.000000000	0.058571663	0.010810268	0	0.000000000	0.04	0.18483741	1.0279267
26	TCGA−BR−6802	0.085226577	0.000400000	0.021172855	0.059739661	0.08903099	0.000000000	0.108359596	0.000000000	0.072744423	0	0.013507470	0.01	0.32263978	0.9599046
27	TCGA−CD−8535	0.021916785	0.000000000	0.446827217	0.001447166	0.06094481	0.000000000	0.000000000	0.000000000	0.114822932	0	0.000000000	0.05	0.15158852	1.0691619
28	TCGA−BR−8362	0.108249902	0.000000000	0.045801156	0.063779041	0.23738651	0.016664467	0.000000000	0.068233128	0.018585891	0	0.000000000	0.03	0.19408715	1.0148776
29	TCGA−D7−8576	0.063460322	0.032135207	0.085937083	0.143990960	0.30535338	0.015234986	0.000000000	0.070078667	0.000000000	0	0.000000000	0.06	0.13847667	1.0397717
30	TCGA−CD−A48C	0.070112712	0.010796247	0.035960391	0.118367490	0.13329366	0.000600000	0.008434569	0.000000000	0.122615287	0	0.000000000	0.01	0.26223690	0.9907039
31	TCGA−CD−8533	0.000000000	0.024362853	0.032989433	0.119627292	0.23810268	0.000000000	0.000000000	0.000000000	0.077539751	0	0.000000000	0.26	0.05176438	1.0706761
32	TCGA−HU−A4GY	0.055279142	0.012473845	0.009323696	0.088744919	0.22728179	0.007711236	0.000000000	0.045539101	0.000000000	0	0.000000000	0.00	0.44608861	0.8941151
33	TCGA−BR−A44T	0.064559086	0.000000000	0.065945954	0.034376967	0.05456522	0.000000000	0.000000000	0.007060991	0.000000000	0	0.000000000	0.00	0.44970944	0.8939520
34	TCGA−BR−8297	0.102249635	0.003082455	0.007301074	0.059989610	0.09585294	0.000000000	0.000000000	0.147343952	0.000000000	0	0.000000000	0.05	0.14711335	1.0337334
35	TCGA−EQ−8122	0.020094858	0.036490362	0.122343838	0.059048649	0.26648617	0.014748784	0.000000000	0.027252264	0.057116334	0	0.012675579	0.01	0.25534466	0.9820423
36	TCGA−D7−8575	0.001030868	0.000000000	0.300581141	0.029999981	0.19471920	0.000000000	0.000000000	0.000000000	0.114867210	0	0.004858463	0.02	0.20606164	1.0198948
37	TCGA−BR−8284	0.035414657	0.000000000	0.053878487	0.084858379	0.18292099	0.017227907	0.000000000	0.046952234	0.014986725	0	0.000000000	0.01	0.24045196	0.9961439
38	TCGA−FP−7735	0.071489120	0.011174724	0.052544413	0.053938406	0.17963915	0.000000000	0.003422436	0.046529803	0.019805826	0	0.003639665	0.01	0.28838438	0.9666444
39	TCGA−BR−7723	0.121876772	0.000000000	0.117903489	0.102347611	0.20174408	0.016979055	0.000000000	0.016879350	0.000000000	0	0.000000000	0.02	0.20211438	1.0172927
40	TCGA−CD−5813	0.074037522	0.037087263	0.101805422	0.078912526	0.24202172	0.032974261	0.000000000	0.000000000	0.048436082	0	0.000000000	0.04	0.17589101	1.0186153
41	TCGA−B7−5816	0.031552882	0.000000000	0.166326288	0.070505017	0.12651041	0.000000000	0.033520497	0.000000000	0.133568468	0	0.023248623	0.04	0.17353228	1.0208621
42	TCGA−D7−A4YY	0.057531584	0.000000000	0.320599565	0.095861904	0.19396325	0.000000000	0.000000000	0.000000000	0.009334064	0	0.000000000	0.01	0.27685086	0.9894682
43	TCGA−D7−A4YX	0.028739606	0.000000000	0.042465957	0.144238589	0.17914547	0.000000000	0.013988734	0.000000000	0.035966214	0	0.008698848	0.01	0.37978787	0.9307886
44	TCGA−D7−A4YU	0.016813546	0.023854573	0.029179330	0.201414852	0.13298179	0.000000000	0.000000000	0.015558325	0.000000000	0	0.000000000	0.00	0.47663183	0.8785432
45	TCGA−D7−6520	0.065257830	0.018633985	0.000000000	0.123044296	0.20306004	0.000000000	0.008974395	0.051077745	0.000000000	0	0.000000000	0.17	0.08053181	1.0610886
46	TCGA−BR−6565	0.059417662	0.000000000	0.140038901	0.099758437	0.18380416	0.003969199	0.000000000	0.026286433	0.000000000	0	0.000000000	0.01	0.27944820	0.9745296
47	TCGA−BR−8484	0.022315566	0.000000000	0.119493895	0.055835026	0.18053566	0.002492515	0.000000000	0.044866332	0.055185352	0	0.000000000	0.11	0.11549681	1.0427143
48	TCGA−BR−8487	0.034610189	0.011133228	0.074297689	0.068116399	0.25406055	0.000000000	0.000000000	0.000000000	0.041670976	0	0.008209334	0.05	0.16069190	1.0222717
49	TCGA−BR−8682	0.054442717	0.021442566	0.144593993	0.025365851	0.23077766	0.036736462	0.000000000	0.125271578	0.000000000	0	0.014504418	0.04	0.17222374	1.0125841
50	TCGA−BR−8485	0.045171184	0.022721001	0.011079744	0.043279333	0.09093447	0.020351509	0.001530424	0.052242107	0.028468607	0	0.001130235	0.02	0.20872471	1.0182406
51	TCGA−BR−8058	0.038015238	0.009464080	0.041388548	0.076690584	0.14778218	0.009735527	0.000000000	0.080270554	0.000000000	0	0.020620055	0.01	0.21295420	1.0070326
52	TCGA−BR−8369	0.070053256	0.000000000	0.136687172	0.026852795	0.07453486	0.000000000	0.011443744	0.039227819	0.058821411	0	0.018534820	0.18	0.07844713	1.0605603
53	TCGA−CD−8536	0.035756771	0.018124745	0.002156662	0.058636952	0.16340607	0.001902747	0.000000000	0.000000000	0.130450269	0	0.011158373	0.03	0.20072912	1.0125247
54	TCGA−D7−8572	0.041445621	0.004475762	0.189884864	0.061075217	0.12119694	0.001213132	0.000000000	0.000000000	0.212648751	0	0.015431592	0.04	0.17637581	1.0334600
55	TCGA−CD−A486	0.059566307	0.000000000	0.198264970	0.102617632	0.26606435	0.000000000	0.000000000	0.000000000	0.064175757	0	0.014156260	0.05	0.16470455	1.0349157
56	TCGA−FP−A4BE	0.016930013	0.000000000	0.106585988	0.106016518	0.15677533	0.010533592	0.000000000	0.000000000	0.122444058	0	0.007636918	0.01	0.36875223	0.9321894
57	TCGA−BR−8361	0.074978605	0.015845860	0.000000000	0.124360851	0.13628330	0.000000000	0.034938404	0.000000000	0.053633260	0	0.000000000	0.04	0.18048699	1.0404754
58	TCGA−BR−A453	0.102916728	0.001868013	0.000000000	0.074990464	0.23293108	0.014419360	0.000000000	0.114010455	0.030157681	0	0.000000000	0.01	0.24013917	0.9965416
59	TCGA−F1−A448	0.065216038	0.031204277	0.116096254	0.039800778	0.16268880	0.005002422	0.014683482	0.000000000	0.118859979	0	0.018351771	0.11	0.11598198	1.0424322
60	TCGA−BR−A4CS	0.014700590	0.019080331	0.122164576	0.027723465	0.14118068	0.000000000	0.046426420	0.000000000	0.261811177	0	0.036102802	0.01	0.29512518	0.9725292
61	TCGA−BR−8291	0.049548317	0.033461390	0.063110802	0.013439347	0.29225100	0.033534178	0.003374273	0.112841923	0.000000000	0	0.000000000	0.01	0.24660363	0.9829946
62	TCGA−BR−8081	0.063663386	0.004823005	0.063831865	0.103464685	0.15479543	0.000200000	0.004101340	0.000000000	0.129135439	0	0.006534996	0.01	0.37844478	0.9282923
63	TCGA−D7−8574	0.042785253	0.011803037	0.039864239	0.015536574	0.08235774	0.000000000	0.000000000	0.023017208	0.000000000	0	0.000000000	0.01	0.39336050	0.9225738
64	TCGA−FP−8210	0.073802497	0.000000000	0.028371521	0.070188771	0.13349322	0.017661360	0.007542961	0.024522926	0.031833137	0	0.013985103	0.00	0.44718067	0.8935870
65	TCGA−BR−4280	0.056494803	0.000000000	0.102356398	0.036795437	0.15255578	0.000000000	0.000000000	0.000000000	0.083491875	0	0.000000000	0.18	0.07595308	1.0616890
66	TCGA−BR−4292	0.036301624	0.017559789	0.121329000	0.072847983	0.05457356	0.008157753	0.000000000	0.000000000	0.132191897	0	0.000000000	0.04	0.17685449	1.0221834
67	TCGA−BR−4256	0.023983084	0.088500195	0.078048566	0.073250810	0.14105642	0.000000000	0.028907891	0.063315998	0.028177932	0	0.017217852	0.03	0.19967610	1.0014577
68	TCGA−BR−7901	0.007850484	0.000000000	0.263348720	0.021498408	0.13401891	0.006516272	0.021118035	0.000000000	0.118787051	0	0.002881803	0.01	0.25352042	0.9905433
69	TCGA−BR−7707	0.076263717	0.000000000	0.171177665	0.181225651	0.09553561	0.000000000	0.031345204	0.000000000	0.106762179	0	0.033697630	0.01	0.38081539	0.9357746
70	TCGA−BR−7715	0.060930381	0.000000000	0.363412654	0.046091914	0.17826667	0.000000000	0.000000000	0.037989049	0.075467436	0	0.000000000	0.01	0.27072582	0.9959770
71	TCGA−CD−5803	0.070615701	0.042940525	0.008404408	0.070601570	0.16519867	0.008728917	0.002047548	0.045991084	0.000000000	0	0.000000000	0.01	0.38914585	0.9221697
72	TCGA−CD−5800	0.017486954	0.000000000	0.386321554	0.062300024	0.09566071	0.000000000	0.000000000	0.000000000	0.000000000	0	0.000000000	0.01	0.22909292	1.0114643
73	TCGA−BR−A4PF	0.019995437	0.000000000	0.122247735	0.122409300	0.21052356	0.000000000	0.000000000	0.000000000	0.019925592	0	0.096103621	0.22	0.06297986	1.0697347
74	TCGA−HU−A4H8	0.048701104	0.022877182	0.040854229	0.068456801	0.17101924	0.004813420	0.026506305	0.119065991	0.000000000	0	0.000000000	0.15	0.09299633	1.0437022
75	TCGA−D7−A4YT	0.038073241	0.008495866	0.098839253	0.062523001	0.05029331	0.000000000	0.000000000	0.000000000	0.000000000	0	0.000000000	0.23	0.05855872	1.0847116
76	TCGA−HU−A4GT	0.011221078	0.017872133	0.107474380	0.042142658	0.18092363	0.010167466	0.000000000	0.096519153	0.058707690	0	0.002768973	0.05	0.14380167	1.0287994
77	TCGA−BR−A4PD	0.040943656	0.024330382	0.047301259	0.080527644	0.29097942	0.014375747	0.006037062	0.000000000	0.049806812	0	0.000000000	0.06	0.13963038	1.0332194
78	TCGA−BR−A452	0.038366013	0.025043835	0.072055443	0.049510489	0.00000000	0.017199568	0.000000000	0.000000000	0.124460715	0	0.000000000	0.13	0.10400147	1.0584462
79	TCGA−HU−A4G2	0.051990086	0.014982997	0.010261262	0.120691933	0.27179826	0.019285997	0.018801885	0.000000000	0.018064794	0	0.000000000	0.01	0.34201907	0.9470094
80	TCGA−EQ−A4SO	0.073156043	0.000400000	0.011321044	0.063154091	0.13291704	0.015177756	0.000000000	0.040382269	0.020179775	0	0.002894464	0.01	0.25641652	0.9932493
81	TCGA−CD−5798	0.080106287	0.028891125	0.000000000	0.052594108	0.26889224	0.014986344	0.014287461	0.111516858	0.034113765	0	0.000000000	0.06	0.14162139	1.0264792
82	TCGA−CD−5799	0.076573591	0.000000000	0.221173784	0.059372596	0.14972211	0.000000000	0.005629439	0.033678035	0.000000000	0	0.029796473	0.05	0.14964199	1.0332898
83	TCGA−D7−6518	0.087810459	0.017590938	0.000000000	0.133126829	0.19544170	0.000400000	0.000000000	0.132848815	0.000000000	0	0.000000000	0.07	0.13113588	1.0407903
84	TCGA−D7−6519	0.110983050	0.008909823	0.000000000	0.034097442	0.21491726	0.000000000	0.004122992	0.257914112	0.000000000	0	0.000000000	0.06	0.14164309	1.0395436
85	TCGA−F1−6177	0.066221953	0.000000000	0.000000000	0.097868270	0.20374591	0.008797502	0.013551147	0.000000000	0.091479220	0	0.000000000	0.05	0.14313010	1.0398133
86	TCGA−BR−6452	0.000000000	0.000000000	0.186100028	0.044743086	0.20240986	0.002573064	0.010312756	0.000000000	0.171406964	0	0.007115210	0.02	0.20711537	1.0123864
87	TCGA−BR−6453	0.013265069	0.000000000	0.142276442	0.050999154	0.11677975	0.000000000	0.000000000	0.000000000	0.033031247	0	0.000000000	0.00	0.47975974	0.8773442
88	TCGA−BR−6454	0.051155689	0.000700000	0.011286017	0.127950601	0.08535314	0.003726617	0.000000000	0.000000000	0.031554153	0	0.011146324	0.17	0.08691680	1.0826862
89	TCGA−BR−6455	0.032963300	0.017506107	0.066592959	0.086018629	0.21870379	0.031524757	0.000000000	0.035559333	0.015266684	0	0.002943353	0.01	0.23080575	0.9956560
90	TCGA−BR−6456	0.031173869	0.000000000	0.190672911	0.052058104	0.20977301	0.000000000	0.000000000	0.096854790	0.042239545	0	0.001135259	0.01	0.27269654	0.9753258
91	TCGA−BR−6457	0.062888235	0.001023532	0.033722892	0.081557322	0.15337476	0.016727012	0.000000000	0.089584782	0.000000000	0	0.001851439	0.01	0.22368361	0.9934916
92	TCGA−BR−6458	0.073251983	0.018869256	0.017842037	0.107216706	0.25041210	0.065883284	0.000000000	0.014758579	0.000000000	0	0.000300000	0.01	0.26810998	0.9799337
93	TCGA−BR−7196	0.000000000	0.025626915	0.009374751	0.159325202	0.16152580	0.000000000	0.003642654	0.082761596	0.007301404	0	0.000000000	0.09	0.12789084	1.0466650
94	TCGA−BR−7703	0.060502514	0.021263925	0.083978187	0.095916934	0.22055376	0.007199252	0.000400000	0.005969530	0.040446787	0	0.000000000	0.01	0.22306841	0.9998417
95	TCGA−FP−7829	0.000000000	0.006647179	0.374407041	0.023521664	0.15526850	0.005545129	0.000000000	0.000000000	0.127325419	0	0.030086754	0.03	0.19975445	1.0365421
96	TCGA−BR−7197	0.000000000	0.000000000	0.394445749	0.007734285	0.12138830	0.000000000	0.000000000	0.000000000	0.132316257	0	0.000000000	0.01	0.22640558	1.0224972
97	TCGA−BR−7851	0.002130653	0.018399615	0.151803356	0.060775253	0.14561675	0.000000000	0.000000000	0.000000000	0.121377326	0	0.018024851	0.08	0.13063771	1.0357966
98	TCGA−BR−7957	0.024068788	0.018908887	0.007078007	0.072114322	0.15864222	0.029110146	0.011494265	0.153597001	0.049802035	0	0.020741961	0.01	0.22847315	0.9906555
99	TCGA−FP−7998	0.013426096	0.003460223	0.030968494	0.132036263	0.15705486	0.009743796	0.000000000	0.000000000	0.012572130	0	0.000700000	0.01	0.24762173	0.9941154
100	TCGA−BR−7958	0.122821525	0.005282458	0.014195783	0.178723893	0.15884279	0.022860163	0.014905732	0.000000000	0.054583766	0	0.008659512	0.00	0.48953044	0.8728023

	Input Sample	B cells naive	B cells memory	Plasma cells	T cells CD8	T cells CD4 naive	T cells CD4 memory resting	T cells CD4 memory activated	T cells follicular helper	T cells regulatory (Tregs)	T cells gamma delta	NK cells resting
1	TCGA−BR−7959	0.000000000	0.00000000	0.013499055	0.06130388	0	0.20073860	0.00000000	0.00000000	0.07387292	0.00000000	0.03342120
2	TCGA−BR−A4J7	0.005782058	0.00000000	0.019653699	0.23362570	0	0.07598142	0.01742029	0.06323109	0.05854923	0.00000000	0.00000000
3	TCGA−BR−A4IU	0.017259821	0.02959168	0.003254790	0.13460695	0	0.12281088	0.00000000	0.06363195	0.09264691	0.00000000	0.00000000
4	TCGA−BR−A4J9	0.077509321	0.09440084	0.000000000	0.04543263	0	0.19925248	0.00000000	0.04990764	0.09571860	0.01815577	0.00000000
5	TCGA−CD−A4MH	0.017259821	0.02959168	0.003254790	0.13460695	0	0.12281088	0.00000000	0.06363195	0.09264691	0.00000000	0.00000000
6	TCGA−D7−A4Z0	0.037945819	0.02179156	0.000000000	0.02719157	0	0.27606929	0.00000000	0.01139587	0.03221782	0.00000000	0.00000000
7	TCGA−BR−A4IY	0.039397908	0.00000000	0.027243242	0.10745268	0	0.04367774	0.07797613	0.05013658	0.03493716	0.00000000	0.00000000
8	TCGA−BR−A4J6	0.000000000	0.09740036	0.042737064	0.11817360	0	0.18358385	0.00000000	0.09935995	0.03738772	0.00000000	0.00000000
9	TCGA−BR−A4J4	0.017259821	0.02959168	0.003254790	0.13460695	0	0.12281088	0.00000000	0.06363195	0.09264691	0.00000000	0.00000000
10	TCGA−CD−A4MJ	0.001675407	0.00000000	0.000000000	0.01291004	0	0.20611271	0.00000000	0.00000000	0.06575115	0.00000000	0.09608625
11	TCGA−BR−A4J2	0.263742892	0.08202392	0.000000000	0.07821518	0	0.12575068	0.00000000	0.09277594	0.05648314	0.00000000	0.00000000
12	TCGA−CD−A4MG	0.046823668	0.00000000	0.000000000	0.03889448	0	0.20127277	0.00000000	0.05988425	0.02535254	0.00000000	0.00000000
13	TCGA−BR−A4J8	0.004664470	0.07603116	0.004971531	0.06656304	0	0.19074187	0.00000000	0.02223517	0.08048852	0.00000000	0.00000000
14	TCGA−BR−A4IV	0.017259821	0.02959168	0.003254790	0.13460695	0	0.12281088	0.00000000	0.06363195	0.09264691	0.00000000	0.00000000
15	TCGA−CD−A4MI	0.019570867	0.00000000	0.000100000	0.10756023	0	0.05081598	0.06300651	0.08710662	0.02907621	0.04851653	0.00000000
16	TCGA−BR−A4J1	0.000100000	0.00000000	0.029690068	0.12503948	0	0.09426738	0.00000000	0.04161475	0.17976603	0.00000000	0.00000000
17	TCGA−BR−A4J5	0.000000000	0.03674224	0.000000000	0.04169479	0	0.16831559	0.00000000	0.01462238	0.04822440	0.00000000	0.00000000
18	TCGA−BR−A4IZ	0.042696745	0.00298960	0.000000000	0.07183424	0	0.19194877	0.00000000	0.06031186	0.03623201	0.02617297	0.00000000

	Input Sample	NK cells activated	Monocytes	Macrophages M0	Macrophages M1	Macrophages M2	Dendritic cells resting	Dendritic cells activated	Mast cells resting	Mast cells activated	Eosinophils	Neutrophils	P−value	Pearson Correlation	RMSE
1	TCGA−BR−7959	0.00090000	0.047049158	0.08136749	0.057711202	0.16214876	0.000000000	0.011410103	0.21136015	0.02222080	0	0.022949561	0.03	0.18972198	1.0055649
2	TCGA−BR−A4J7	0.07960540	0.021825498	0.08423538	0.126399233	0.20201451	0.000000000	0.000000000	0.00000000	0.01167650	0	0.000000000	0.05	0.14709298	1.0450556
3	TCGA−BR−A4IU	0.06050251	0.021263925	0.08397819	0.095916934	0.22055376	0.007199252	0.000400000	0.00596953	0.04044679	0	0.000000000	0.01	0.22306841	0.9998417
4	TCGA−BR−A4J9	0.05791999	0.000000000	0.15392781	0.075115135	0.11733044	0.000000000	0.000000000	0.00000000	0.01532935	0	0.000000000	0.01	0.21026112	1.0050493
5	TCGA−CD−A4MH	0.06050251	0.021263925	0.08397819	0.095916934	0.22055376	0.007199252	0.000400000	0.00596953	0.04044679	0	0.000000000	0.01	0.22306841	0.9998417
6	TCGA−D7−A4Z0	0.06906254	0.016436501	0.10319297	0.008294847	0.10260902	0.032690165	0.052433720	0.14918476	0.00000000	0	0.059483559	0.17	0.08525675	1.0458535
7	TCGA−BR−A4IY	0.05557602	0.000000000	0.03212820	0.212234937	0.23974328	0.010544678	0.019153448	0.00000000	0.04010595	0	0.009692052	0.01	0.28318639	0.9853278
8	TCGA−BR−A4J6	0.05144151	0.014118877	0.05581859	0.003718340	0.13499257	0.000700000	0.021080163	0.09875616	0.04051240	0	0.000200000	0.19	0.06899529	1.0555269
9	TCGA−BR−A4J4	0.06050251	0.021263925	0.08397819	0.095916934	0.22055376	0.007199252	0.000400000	0.00596953	0.04044679	0	0.000000000	0.01	0.22306841	0.9998417
10	TCGA−CD−A4MJ	0.05003544	0.000000000	0.17688320	0.109947316	0.07221580	0.000000000	0.000000000	0.00000000	0.18524813	0	0.023134562	0.19	0.06849664	1.0920726
11	TCGA−BR−A4J2	0.01495415	0.002745121	0.08480037	0.015858941	0.08787102	0.000000000	0.015205902	0.03991599	0.02562099	0	0.014035777	0.01	0.36893601	0.9323168
12	TCGA−CD−A4MG	0.06645736	0.015114429	0.12916424	0.049621811	0.16263818	0.044151334	0.000000000	0.00000000	0.12186433	0	0.038760606	0.02	0.20541307	1.0063779
13	TCGA−BR−A4J8	0.10304790	0.019253589	0.07769931	0.052853458	0.12286202	0.000000000	0.000000000	0.17858796	0.00000000	0	0.000000000	0.05	0.15035084	1.0248428
14	TCGA−BR−A4IV	0.06050251	0.021263925	0.08397819	0.095916934	0.22055376	0.007199252	0.000400000	0.00596953	0.04044679	0	0.000000000	0.01	0.22306841	0.9998417
15	TCGA−CD−A4MI	0.08141871	0.000000000	0.09026356	0.116477817	0.25903005	0.000000000	0.002591608	0.00743877	0.02693568	0	0.010049887	0.00	0.50935010	0.8605160
16	TCGA−BR−A4J1	0.09467492	0.012465881	0.10456901	0.000000000	0.11610444	0.000000000	0.014787392	0.00000000	0.18262041	0	0.004275761	0.26	0.04884165	1.0913880
17	TCGA−BR−A4J5	0.02796844	0.000000000	0.36691524	0.034896270	0.21296268	0.000000000	0.000000000	0.04375521	0.00030000	0	0.003608411	0.01	0.26536071	0.9972268
18	TCGA−BR−A4IZ	0.07396168	0.010073569	0.01024237	0.047191201	0.28845245	0.000000000	0.000000000	0.00000000	0.13218154	0	0.005711002	0.01	0.27754232	0.9769880

Supporting Material 5C:
Stromal score correlated genes (TCGA−GCC)

	gene	feature	p.value	estimate	adj.p.value	COR.ID	gene	feature	p.value	estimate	adj.p.value	COR.ID
1	DES	Stromal_score	4.667034e−12	0.4470284	1.238584e−07	POS	CCDC92	Stromal_score	1.432342e−07	0.3479315	3.801294e−03	POS
2	MYH11	Stromal_score	1.006945e−17	0.5386074	2.672330e−13	POS	CCT5	Stromal_score	4.064986e−12	−0.4481560	1.078807e−07	NEG
3	ACTG2	Stromal_score	1.151534e−14	0.4925919	3.056057e−10	POS	BC048192	Stromal_score	2.206306e−08	−0.3684912	5.855315e−04	NEG
4	HSPB6	Stromal_score	1.531922e−22	0.5992362	4.065567e−18	POS	STAT5B	Stromal_score	4.934125e−14	0.4821547	1.309468e−09	POS
5	CNN1	Stromal_score	7.290887e−18	0.5405589	1.934928e−13	POS	RCCD1	Stromal_score	3.771813e−13	−0.4669445	1.001001e−08	NEG
6	FDCSP	Stromal_score	1.231572e−06	0.3223050	3.268469e−02	POS	CYP2U1	Stromal_score	1.207327e−17	0.5375050	3.204125e−13	POS
7	APOD	Stromal_score	1.029472e−14	0.4933811	2.732115e−10	POS	IDI2−AS1	Stromal_score	2.475811e−11	−0.4330665	6.570554e−07	NEG
8	C7	Stromal_score	3.373656e−21	0.5835580	8.953345e−17	POS	SNORD60	Stromal_score	1.289910e−09	−0.3971882	3.423291e−05	NEG
9	SYNM	Stromal_score	4.523845e−13	0.4655476	1.200583e−08	POS	ANKRD44	Stromal_score	2.790905e−11	0.4320392	7.406783e−07	POS
10	THBS4	Stromal_score	1.900711e−25	0.6304662	5.044296e−21	POS	CCDC88A	Stromal_score	5.448322e−07	0.3322940	1.445930e−02	POS
11	SFRP2	Stromal_score	1.584275e−22	0.5990703	4.204508e−18	POS	LINC00341	Stromal_score	1.330259e−08	0.3738124	3.530373e−04	POS
12	SYNPO2	Stromal_score	5.342642e−19	0.5559041	1.417884e−14	POS	BC038570	Stromal_score	7.463989e−14	−0.4791198	1.980868e−09	NEG
13	CCL19	Stromal_score	9.026099e−18	0.5392700	2.395436e−13	POS	C20orf72	Stromal_score	4.855507e−08	−0.3599979	1.288603e−03	NEG
14	FLNC	Stromal_score	5.164728e−19	0.5560979	1.370667e−14	POS	SLC25A1	Stromal_score	2.511065e−07	−0.3414646	6.664117e−03	NEG
15	C2orf40	Stromal_score	1.071466e−15	0.5088973	2.843564e−11	POS	ZFHX4	Stromal_score	3.086367e−22	0.5957585	8.190909e−18	POS
16	OGN	Stromal_score	5.548685e−26	0.6358483	1.472566e−21	POS	ERG	Stromal_score	6.231214e−23	0.6036410	1.653702e−18	POS
17	CD79A	Stromal_score	2.832146e−07	0.3400593	7.516232e−03	POS	USP11	Stromal_score	1.827589e−07	0.3451423	4.850240e−03	POS
18	LMOD1	Stromal_score	4.530315e−20	0.5697010	1.202300e−15	POS	SNORD38B	Stromal_score	1.080587e−09	−0.3988896	2.867770e−05	NEG
19	CHRDL2	Stromal_score	5.856115e−19	0.5553784	1.554154e−14	POS	PWWP2B	Stromal_score	7.542704e−07	−0.3283526	2.001758e−02	NEG
20	CLU	Stromal_score	3.752450e−07	0.3367458	9.958628e−03	POS	PHF1	Stromal_score	2.136780e−09	0.3922847	5.670801e−05	POS
21	CCL21	Stromal_score	1.262145e−13	0.4752245	3.349605e−09	POS	LOC81691	Stromal_score	1.025869e−11	−0.4405175	2.722553e−07	NEG
22	CR2	Stromal_score	1.982192e−07	0.3442068	5.260538e−03	POS	BCL7A	Stromal_score	1.114019e−07	−0.3507802	2.956495e−03	NEG
23	PLN	Stromal_score	5.605841e−20	0.5685350	1.487734e−15	POS	SPATA2	Stromal_score	2.371582e−07	−0.3421296	6.293941e−03	NEG
24	HSPB7	Stromal_score	3.093188e−17	0.5317246	8.209012e−13	POS	MTHFD1L	Stromal_score	5.772552e−07	−0.3315975	1.531978e−02	NEG
25	MGP	Stromal_score	1.125389e−24	0.6225010	2.986669e−20	POS	SNORD44	Stromal_score	6.868249e−11	−0.4242039	1.822765e−06	NEG
26	FHL1	Stromal_score	3.590267e−23	0.6063074	9.528210e−19	POS	TRAIP	Stromal_score	1.107192e−15	−0.5086779	2.938377e−11	NEG
27	TACR2	Stromal_score	2.131855e−08	0.3688553	5.657730e−04	POS	KIAA0408	Stromal_score	1.402304e−10	0.4178472	3.721575e−06	POS
28	C3	Stromal_score	8.939665e−15	0.4943722	2.372498e−10	POS	S1PR2	Stromal_score	2.730870e−11	0.4322259	7.247455e−07	POS
29	MYL9	Stromal_score	5.025229e−23	0.6046842	1.333645e−18	POS	HDDC3	Stromal_score	2.872215e−08	−0.3656785	7.622572e−04	NEG
30	PTGDS	Stromal_score	2.639680e−19	0.5599123	7.005447e−15	POS	WDHD1	Stromal_score	5.544372e−10	−0.4052166	1.471421e−05	NEG
31	SFRP4	Stromal_score	4.667493e−17	0.5291617	1.238706e−12	POS	KDM1B	Stromal_score	6.560284e−07	−0.3300503	1.741034e−02	NEG
32	TAGLN	Stromal_score	8.210384e−25	0.6239300	2.178954e−20	POS	CLIC1	Stromal_score	1.066748e−10	−0.4202986	2.831042e−06	NEG
33	PGM5	Stromal_score	9.564274e−19	0.5525525	2.538263e−14	POS	TTF2	Stromal_score	4.238853e−11	−0.4284280	1.124949e−06	NEG
34	PDK4	Stromal_score	4.179331e−11	0.4285509	1.109153e−06	POS	CENPI	Stromal_score	2.665129e−13	−0.4695956	7.072985e−09	NEG
35	HAND2	Stromal_score	4.241781e−19	0.5572218	1.125726e−14	POS	NUDT14	Stromal_score	8.921740e−08	−0.3532740	2.367740e−03	NEG
36	GREM1	Stromal_score	3.591415e−18	0.5447946	9.531257e−14	POS	PIP5K1C	Stromal_score	1.239466e−16	0.5229894	3.289419e−12	POS
37	MYLK	Stromal_score	8.101929e−23	0.6023624	2.150171e−18	POS	SS18L1	Stromal_score	3.433582e−09	−0.3876025	9.112383e−05	NEG
38	ADH1B	Stromal_score	2.887062e−16	0.5175424	7.661974e−12	POS	SLC35D2	Stromal_score	2.034300e−07	−0.3439072	5.398828e−03	NEG
39	PRUNE2	Stromal_score	1.748448e−13	0.4727827	4.640207e−09	POS	AHDC1	Stromal_score	7.765704e−09	0.3793698	2.060940e−04	POS
40	BARX1	Stromal_score	3.152143e−14	0.4854068	8.365473e−10	POS	TOR2A	Stromal_score	3.156075e−07	−0.3387886	8.375907e−03	NEG
41	TPM2	Stromal_score	3.469612e−16	0.5163454	9.208003e−12	POS	PPM1K	Stromal_score	3.725291e−13	0.4670397	9.886549e−09	POS
42	SFRP1	Stromal_score	6.395242e−22	0.5920961	1.697233e−17	POS	ATP5B	Stromal_score	1.379328e−07	−0.3483607	3.660598e−03	NEG
43	TNC	Stromal_score	2.663863e−18	0.5465640	7.069627e−14	POS	ZNF761	Stromal_score	1.829930e−06	−0.3173230	4.856450e−02	NEG
44	DARC	Stromal_score	2.627185e−21	0.5848581	6.972286e−17	POS	ERC1	Stromal_score	7.016511e−11	0.4240157	1.862112e−06	POS
45	VPREB3	Stromal_score	1.151396e−06	0.3231431	3.055690e−02	POS	BC033949	Stromal_score	8.483481e−08	−0.3538366	2.251431e−03	NEG
46	HSPB8	Stromal_score	5.255796e−23	0.6044670	1.394836e−18	POS	IDH3A	Stromal_score	1.203738e−06	−0.3225899	3.194601e−02	NEG
47	MS4A1	Stromal_score	3.938473e−08	0.3622759	1.045231e−03	POS	FREM1	Stromal_score	9.040303e−07	0.3261338	2.399206e−02	POS
48	RGMA	Stromal_score	5.109130e−23	0.6046041	1.355912e−18	POS	RBMS2	Stromal_score	2.856145e−10	0.4113769	7.579924e−06	POS
49	SPARCL1	Stromal_score	2.379971e−27	0.6491464	6.316204e−23	POS	SLC25A33	Stromal_score	7.903470e−10	−0.4018718	2.097502e−05	NEG
50	SMOC2	Stromal_score	9.098246e−15	0.4942489	2.414583e−10	POS	PCDH12	Stromal_score	2.848071e−09	0.3894569	7.558495e−05	POS
51	PLIN4	Stromal_score	4.165288e−14	0.4833882	1.105426e−09	POS	RRP9	Stromal_score	8.350546e−13	−0.4607905	2.216151e−08	NEG
52	CCL14	Stromal_score	7.985897e−20	0.5665877	2.119377e−15	POS	VRK1	Stromal_score	7.521777e−09	−0.3796960	1.996204e−04	NEG
53	CCDC80	Stromal_score	1.766275e−37	0.7305771	4.687517e−33	POS	CCDC99	Stromal_score	1.191108e−09	−0.3979551	3.161082e−05	NEG
54	RBPMS2	Stromal_score	2.461611e−16	0.5185769	6.532870e−12	POS	RFC5	Stromal_score	4.050599e−11	−0.4288226	1.074988e−06	NEG
55	PPP1R12B	Stromal_score	3.795615e−17	0.5304525	1.007318e−12	POS	ARRDC1	Stromal_score	1.029367e−10	−0.4206169	2.731836e−06	NEG
56	MFAP4	Stromal_score	1.282292e−25	0.6321981	3.403075e−21	POS	MTFR1	Stromal_score	1.343405e−06	−0.3212191	3.565262e−02	NEG
57	PTGIS	Stromal_score	4.075769e−26	0.6371804	1.081668e−21	POS	MRM1	Stromal_score	1.473652e−09	−0.3959022	3.910924e−05	NEG
58	FABP4	Stromal_score	4.471379e−12	0.4473785	1.186659e−07	POS	TNFSF8	Stromal_score	1.438945e−10	0.4176149	3.818816e−06	POS
59	SPON1	Stromal_score	1.229148e−23	0.6114208	3.262036e−19	POS	IRF2BPL	Stromal_score	1.111085e−15	0.5086544	2.948709e−11	POS
60	NGFR	Stromal_score	5.602086e−18	0.5421420	1.486737e−13	POS	ADCY4	Stromal_score	1.281933e−16	0.5227743	3.402122e−12	POS
61	PRELP	Stromal_score	4.420472e−24	0.6162142	1.173149e−19	POS	AMIGO3	Stromal_score	1.670589e−11	−0.4364159	4.433576e−07	NEG
62	TNS1	Stromal_score	1.676645e−28	0.6598568	4.449649e−24	POS	DLG3	Stromal_score	3.472924e−11	−0.4301554	9.216792e−07	NEG
63	CCL5	Stromal_score	1.215222e−07	0.3497978	3.225078e−03	POS	RUSC1−AS1	Stromal_score	5.731967e−08	−0.3581792	1.521207e−03	NEG
64	KCNMB1	Stromal_score	6.100892e−15	0.4970395	1.619116e−10	POS	EXOSC4	Stromal_score	6.109863e−07	−0.3309117	1.621497e−02	NEG
65	SEPP1	Stromal_score	4.693939e−10	0.4067749	1.245725e−05	POS	FGFR1OP	Stromal_score	2.427634e−14	−0.4872865	6.442698e−10	NEG
66	COL14A1	Stromal_score	3.208053e−30	0.6750547	8.513851e−26	POS	GTF3C5	Stromal_score	1.678343e−11	−0.4363767	4.454154e−07	NEG
67	NDE1	Stromal_score	3.955189e−12	0.4483791	1.049668e−07	POS	THOP1	Stromal_score	2.944620e−07	−0.3396030	7.814728e−03	NEG
68	CRYAB	Stromal_score	8.723988e−24	0.6130368	2.315259e−19	POS	MEGF8	Stromal_score	9.330620e−08	0.3527725	2.476253e−03	POS
69	SORBS1	Stromal_score	4.527638e−12	0.4472763	1.201590e−07	POS	ALG3	Stromal_score	2.684343e−09	−0.3900418	7.123978e−05	NEG
70	PRSS3	Stromal_score	1.038179e−08	−0.3763850	2.755223e−04	NEG	TMEM180	Stromal_score	3.702265e−08	−0.3629456	9.825440e−04	NEG
71	MRGPRF	Stromal_score	1.402777e−27	0.6513155	3.722829e−23	POS	NIPSNAP1	Stromal_score	5.322253e−07	−0.3325755	1.412473e−02	NEG
72	FLNA	Stromal_score	1.460484e−21	0.5878879	3.875980e−17	POS	CRTC3	Stromal_score	2.538032e−11	0.4328539	6.735684e−07	POS
73	CILP	Stromal_score	3.314785e−23	0.6066915	8.797109e−19	POS	ZDHHC23	Stromal_score	4.595378e−15	−0.4990029	1.219567e−10	NEG
74	IGF1	Stromal_score	1.178619e−23	0.6116191	3.127936e−19	POS	AK093279	Stromal_score	1.073763e−06	−0.3240095	2.849659e−02	NEG
75	TCEAL2	Stromal_score	4.636872e−15	0.4989408	1.230579e−10	POS	AX748330	Stromal_score	4.293580e−10	−0.4076059	1.139473e−05	NEG
76	CCNE1	Stromal_score	2.022468e−09	−0.3928228	5.367428e−05	NEG	RAD54B	Stromal_score	5.421766e−14	−0.4814663	1.438883e−09	NEG
77	CYP1B1	Stromal_score	1.783493e−30	0.6772351	4.733213e−26	POS	LATS2	Stromal_score	2.194642e−13	0.4710683	5.824362e−09	POS
78	SIX2	Stromal_score	2.498106e−08	0.3671700	6.629724e−04	POS	SNHG15	Stromal_score	2.046475e−12	−0.4536986	5.431141e−08	NEG
79	FBLN1	Stromal_score	8.708433e−25	0.6236637	2.311131e−20	POS	SLC25A15	Stromal_score	4.013054e−13	−0.4664689	1.065024e−08	NEG
80	GJB1	Stromal_score	6.154342e−08	−0.3573965	1.633301e−03	NEG	AK057719	Stromal_score	3.740648e−18	0.5445526	9.927305e−14	POS
81	AOC3	Stromal_score	6.645442e−28	0.6543512	1.763634e−23	POS	ARID5A	Stromal_score	1.094464e−19	0.5648425	2.904598e−15	POS
82	TPSAB1	Stromal_score	2.244966e−10	0.4135825	5.957915e−06	POS	LNX2	Stromal_score	6.331339e−08	−0.3570838	1.680274e−03	NEG
83	SCRG1	Stromal_score	3.353603e−15	0.5011709	8.900126e−11	POS	TP73−AS1	Stromal_score	4.510350e−07	0.3345583	1.197002e−02	POS
84	ACTA2	Stromal_score	1.556080e−27	0.6508913	4.129681e−23	POS	NOP2	Stromal_score	7.923264e−08	−0.3545980	2.102755e−03	NEG
85	CPE	Stromal_score	1.171006e−20	0.5770007	3.107732e−16	POS	43354	Stromal_score	5.739244e−09	0.3824468	1.523138e−04	POS
86	CHRDL1	Stromal_score	2.891056e−22	0.5960848	7.672573e−18	POS	ADAMTS10	Stromal_score	1.225546e−26	0.6423075	3.252477e−22	POS
87	C2orf82	Stromal_score	1.630006e−07	−0.3464552	4.325873e−03	NEG	LEPRE1	Stromal_score	5.572760e−07	0.3320221	1.478955e−02	POS
88	APOE	Stromal_score	1.391433e−06	0.3207790	3.692723e−02	POS	ST8SIA4	Stromal_score	1.833809e−11	0.4356256	4.866747e−07	POS
89	ENTPD8	Stromal_score	4.741265e−07	−0.3339616	1.258284e−02	NEG	ZNF518A	Stromal_score	3.727286e−07	−0.3368255	9.891843e−03	NEG
90	FXYD1	Stromal_score	2.447384e−19	0.5603391	6.495114e−15	POS	FEZ2	Stromal_score	1.231422e−09	0.3976350	3.268070e−05	POS
91	PTPRCAP	Stromal_score	3.084765e−08	0.3649125	8.186659e−04	POS	CPOX	Stromal_score	1.094299e−06	−0.3237747	2.904160e−02	NEG
92	EFEMP1	Stromal_score	1.308555e−28	0.6608356	3.472774e−24	POS	PLBD2	Stromal_score	1.349653e−09	0.3967516	3.581844e−05	POS
93	CCDC152	Stromal_score	1.799518e−10	0.4155944	4.775740e−06	POS	FAM168A	Stromal_score	6.070817e−07	0.3309892	1.611134e−02	POS
94	AX748062	Stromal_score	2.204080e−26	0.6398154	5.849408e−22	POS	NSDHL	Stromal_score	1.082856e−06	−0.3239050	2.873790e−02	NEG
95	GRB7	Stromal_score	9.159934e−07	−0.3259721	2.430955e−02	NEG	POLR2H	Stromal_score	2.533838e−10	−0.4124757	6.724552e−06	NEG
96	AHNAK2	Stromal_score	3.878281e−11	0.4291997	1.029257e−06	POS	HPCA	Stromal_score	1.181407e−09	−0.3980337	3.135337e−05	NEG
97	IL33	Stromal_score	2.321721e−13	0.4706422	6.161616e−09	POS	FAM13B	Stromal_score	5.268288e−12	0.4460356	1.398151e−07	POS
98	GGH	Stromal_score	5.728315e−07	−0.3316903	1.520238e−02	NEG	PHLPP1	Stromal_score	3.901683e−07	−0.3362834	1.035468e−02	NEG
99	CD52	Stromal_score	2.219684e−10	0.4136858	5.890819e−06	POS	ATP10A	Stromal_score	6.367281e−14	0.4802886	1.689813e−09	POS
100	FOXA2	Stromal_score	7.187486e−09	−0.3801602	1.907487e−04	NEG	GPR146	Stromal_score	9.463428e−09	0.3773404	2.511499e−04	POS
101	CD79B	Stromal_score	3.253672e−10	0.4101766	8.634919e−06	POS	CCR2	Stromal_score	6.538497e−10	0.4036652	1.735252e−05	POS
102	CFD	Stromal_score	9.774784e−16	0.5095105	2.594130e−11	POS	PRKCZ	Stromal_score	2.150927e−11	−0.4342684	5.708346e−07	NEG
103	GZMK	Stromal_score	4.674365e−11	0.4275767	1.240530e−06	POS	SUN2	Stromal_score	2.415706e−19	0.5604126	6.411043e−15	POS
104	SOD3	Stromal_score	1.036979e−20	0.5776477	2.752039e−16	POS	HMBS	Stromal_score	1.166152e−11	−0.4394448	3.094850e−07	NEG
105	TCL1A	Stromal_score	5.181579e−07	0.3328973	1.375139e−02	POS	ALKBH2	Stromal_score	6.341043e−09	−0.3814357	1.682849e−04	NEG
106	DD181999	Stromal_score	6.114821e−13	0.4632181	1.622812e−08	POS	PTER	Stromal_score	7.267592e−07	−0.3288056	1.928746e−02	NEG
107	DPYSL3	Stromal_score	2.343357e−30	0.6762235	6.219035e−26	POS	TUBA1A	Stromal_score	1.782246e−16	0.5206617	4.729904e−12	POS
108	FAM188B	Stromal_score	1.404293e−11	0.4378828	3.726854e−07	POS	COX5A	Stromal_score	4.727062e−09	−0.3844045	1.254515e−04	NEG
109	PPP1R14A	Stromal_score	2.332916e−11	0.4335750	6.191326e−07	POS	PLEKHJ1	Stromal_score	1.550845e−09	−0.3954077	4.115787e−05	NEG
110	TCRBV3S1	Stromal_score	9.473095e−09	0.3773299	2.514065e−04	POS	NUAK1	Stromal_score	3.769587e−12	0.4487703	1.000411e−07	POS
111	LTB	Stromal_score	2.078429e−09	0.3925558	5.515942e−05	POS	TMEM43	Stromal_score	1.028314e−11	0.4404977	2.729042e−07	POS
112	PDLIM3	Stromal_score	2.295018e−28	0.6586118	6.090749e−24	POS	FKSG63	Stromal_score	1.501717e−06	−0.3198211	3.985407e−02	NEG
113	TPSB2	Stromal_score	3.032556e−07	0.3392577	8.048101e−03	POS	GALNTL1	Stromal_score	1.853874e−13	0.4723420	4.919996e−09	POS
114	DPT	Stromal_score	1.182539e−21	0.5889695	3.138340e−17	POS	HCN3	Stromal_score	2.541422e−11	−0.4328425	6.744679e−07	NEG
115	CPA3	Stromal_score	5.639243e−11	0.4259366	1.496599e−06	POS	C22orf15	Stromal_score	5.905213e−07	−0.3313233	1.567184e−02	NEG
116	HLA−DOA	Stromal_score	1.677355e−07	0.3461272	4.451533e−03	POS	PTX3	Stromal_score	8.124893e−08	0.3543182	2.156265e−03	POS
117	CPXM2	Stromal_score	1.683695e−24	0.6206644	4.468358e−20	POS	MIR4292	Stromal_score	7.990863e−07	−0.3276474	2.120695e−02	NEG
118	TMEM45B	Stromal_score	3.462440e−08	−0.3636691	9.188970e−04	NEG	NPR3	Stromal_score	2.968485e−08	0.3653250	7.878063e−04	POS
119	JPH2	Stromal_score	2.304286e−15	0.5037336	6.115343e−11	POS	UCK2	Stromal_score	3.007858e−09	−0.3889167	7.982555e−05	NEG
120	FNDC1	Stromal_score	5.152868e−22	0.5931868	1.367520e−17	POS	FHL5	Stromal_score	1.000048e−12	0.4593771	2.654028e−08	POS

	gene	feature	p.value	estimate	adj.p.value	COR.ID	gene	feature	p.value	estimate	adj.p.value	COR.ID
121	COL1A1	Stromal_score	1.914226e−13	0.4721006	5.080165e−09	POS	LETM1	Stromal_score	1.566902e−06	−0.3192860	4.158401e−02	NEG
122	CCL11	Stromal_score	2.888599e−11	0.4317435	7.666054e−07	POS	CHORDC1	Stromal_score	3.524661e−10	−0.4094373	9.354099e−06	NEG
123	ELN	Stromal_score	2.273577e−18	0.5474981	6.033847e−14	POS	TAF4	Stromal_score	9.405923e−08	−0.3526824	2.496238e−03	NEG
124	CD22	Stromal_score	1.458808e−08	0.3728493	3.871530e−04	POS	DQ579335	Stromal_score	1.547311e−06	−0.3194445	4.106409e−02	NEG
125	FAM129A	Stromal_score	9.364475e−20	0.5657072	2.485238e−15	POS	MAP3K3	Stromal_score	1.403832e−28	0.6605585	3.725631e−24	POS
126	NEXN	Stromal_score	3.656590e−22	0.5949104	9.704225e−18	POS	BMPR1B	Stromal_score	1.081951e−06	0.3239154	2.871391e−02	POS
127	VIP	Stromal_score	1.481702e−09	0.3958495	3.932289e−05	POS	LRRC45	Stromal_score	2.288550e−13	−0.4707512	6.073584e−09	NEG
128	ABI3BP	Stromal_score	2.894557e−24	0.6181750	7.681864e−20	POS	USP9Y	Stromal_score	3.344530e−08	0.3640427	8.876047e−04	POS
129	CALD1	Stromal_score	2.282846e−28	0.6586329	6.058444e−24	POS	DKC1	Stromal_score	6.519769e−11	−0.4246625	1.730281e−06	NEG
130	AKAP12	Stromal_score	3.385390e−20	0.5712882	8.984487e−16	POS	PAK4	Stromal_score	1.159511e−07	−0.3503284	3.077227e−03	NEG
131	FN1	Stromal_score	1.704755e−17	0.5353980	4.524250e−13	POS	DPP3	Stromal_score	8.227426e−11	−0.4226084	2.183477e−06	NEG
132	PODN	Stromal_score	1.761781e−28	0.6596608	4.675591e−24	POS	LOC388630	Stromal_score	2.867740e−14	0.4860887	7.610696e−10	POS
133	CASQ2	Stromal_score	6.003831e−13	0.4633602	1.593357e−08	POS	ACTL6A	Stromal_score	1.288397e−12	−0.4573800	3.419277e−08	NEG
134	CCL15	Stromal_score	2.503405e−09	0.3907297	6.643786e−05	POS	PA2G4	Stromal_score	1.163622e−09	−0.3981794	3.088135e−05	NEG
135	KCNMA1	Stromal_score	9.493088e−18	0.5389646	2.519371e−13	POS	FHOD3	Stromal_score	5.997111e−07	0.3311368	1.591573e−02	POS
136	RAP1GAP	Stromal_score	1.362218e−07	−0.3485027	3.615190e−03	NEG	SHROOM4	Stromal_score	1.086755e−10	0.4201327	2.884139e−06	POS
137	ITM2A	Stromal_score	2.720626e−18	0.5464395	7.220270e−14	POS	TPTEP1	Stromal_score	3.975934e−10	0.4083205	1.055173e−05	POS
138	IGFBP5	Stromal_score	1.680115e−22	0.5987801	4.458857e−18	POS	ACP6	Stromal_score	7.940733e−10	−0.4018272	2.107391e−05	NEG
139	SRPX	Stromal_score	1.155609e−23	0.6117123	3.066870e−19	POS	CHST10	Stromal_score	8.012918e−07	0.3276137	2.126548e−02	POS
140	C6orf223	Stromal_score	8.152395e−07	−0.3274025	2.163564e−02	NEG	CAST	Stromal_score	1.177529e−08	0.3750807	3.125044e−04	POS
141	F13A1	Stromal_score	4.658096e−16	0.5144171	1.236212e−11	POS	WHSC1	Stromal_score	3.616348e−10	−0.4091996	9.597425e−06	NEG
142	CD37	Stromal_score	1.815177e−14	0.4893656	4.817298e−10	POS	CPNE8	Stromal_score	1.238872e−07	0.3495796	3.287842e−03	POS
143	TSPAN7	Stromal_score	2.109454e−10	0.4141502	5.598280e−06	POS	ADRM1	Stromal_score	6.208769e−07	−0.3307174	1.647745e−02	NEG
144	FRZB	Stromal_score	1.033707e−15	0.5091371	2.743355e−11	POS	CPT1C	Stromal_score	5.241728e−11	0.4265765	1.391102e−06	POS
145	CCND2	Stromal_score	2.776916e−11	0.4320823	7.369658e−07	POS	CCNI	Stromal_score	4.988595e−07	0.3333527	1.323923e−02	POS
146	C1QB	Stromal_score	1.303193e−09	0.3970894	3.458543e−05	POS	ANGPTL7	Stromal_score	1.114458e−11	0.4398247	2.957661e−07	POS
147	ASPN	Stromal_score	1.227495e−25	0.6323897	3.257650e−21	POS	RALGPS1	Stromal_score	1.282010e−10	−0.4186532	3.402327e−06	NEG
148	ETV4	Stromal_score	7.146799e−07	−0.3290097	1.896689e−02	NEG	CTDSP2	Stromal_score	1.499991e−08	0.3725581	3.980826e−04	POS
149	DJ417913	Stromal_score	5.586494e−08	0.3584617	1.482600e−03	POS	RAE1	Stromal_score	4.830460e−12	−0.4467467	1.281956e−07	NEG
150	PI16	Stromal_score	2.931388e−12	0.4508089	7.779611e−08	POS	DEGS1	Stromal_score	8.258475e−09	0.3787398	2.191717e−04	POS
151	COL8A1	Stromal_score	6.618451e−28	0.6543676	1.756471e−23	POS	NR2F6	Stromal_score	5.045645e−12	−0.4463897	1.339064e−07	NEG
152	ANGPTL1	Stromal_score	1.421786e−20	0.5759648	3.773277e−16	POS	MRPL13	Stromal_score	1.479256e−06	−0.3200106	3.925797e−02	NEG
153	MATN2	Stromal_score	2.765893e−11	0.4321165	7.340403e−07	POS	QPCTL	Stromal_score	1.226388e−07	−0.3496943	3.254712e−03	NEG
154	HLA−DPB1	Stromal_score	1.044314e−07	0.3515080	2.771504e−03	POS	MYH9	Stromal_score	3.168247e−07	0.3387433	8.408211e−03	POS
155	THBS2	Stromal_score	2.315218e−20	0.5733456	6.144357e−16	POS	ZNF92	Stromal_score	1.924800e−07	−0.3445456	5.108228e−03	NEG
156	MYLK−AS1	Stromal_score	1.568613e−17	0.5359076	4.162942e−13	POS	PTPN13	Stromal_score	7.889447e−19	0.5536647	2.093780e−14	POS
157	IGFBP4	Stromal_score	1.834910e−24	0.6202708	4.869668e−20	POS	RBFOX2	Stromal_score	1.197750e−18	0.5512474	3.178709e−14	POS
158	EPS8L3	Stromal_score	7.477913e−11	−0.4234535	1.984563e−06	NEG	CCDC85B	Stromal_score	1.519710e−06	0.3196712	4.033157e−02	POS
159	CYBRD1	Stromal_score	2.096497e−24	0.6196597	5.563892e−20	POS	MAST4	Stromal_score	4.522111e−12	0.4472863	1.200123e−07	POS
160	SLC2A1	Stromal_score	1.384972e−06	−0.3208374	3.675576e−02	NEG	ZNF793	Stromal_score	2.654277e−07	0.3408177	7.044185e−03	POS
161	CDH1	Stromal_score	4.318232e−09	−0.3853129	1.146016e−04	NEG	DCLRE1A	Stromal_score	2.172326e−13	−0.4711456	5.765136e−09	NEG
162	FGL2	Stromal_score	9.609809e−24	0.6125819	2.550347e−19	POS	PTGFR	Stromal_score	5.196018e−19	0.5560633	1.378971e−14	POS
163	DJ417906	Stromal_score	9.374275e−07	0.3256873	2.487839e−02	POS	RDH13	Stromal_score	1.651544e−07	−0.3463049	4.383032e−03	NEG
164	CARTPT	Stromal_score	1.298546e−07	0.3490463	3.446211e−03	POS	BARD1	Stromal_score	5.686741e−07	−0.3317781	1.509204e−02	NEG
165	SERPINE2	Stromal_score	1.854363e−15	0.5052073	4.921294e−11	POS	PCBP3	Stromal_score	6.363241e−11	0.4248763	1.688741e−06	POS
166	AX747968	Stromal_score	8.398737e−20	0.5663093	2.228941e−15	POS	CNRIP1	Stromal_score	1.823300e−31	0.6855303	4.838855e−27	POS
167	SELL	Stromal_score	4.394926e−11	0.4281135	1.166369e−06	POS	HDGF	Stromal_score	4.115178e−10	−0.4080007	1.092127e−05	NEG
168	CXCL12	Stromal_score	7.321212e−25	0.6244477	1.942976e−20	POS	CCDC86	Stromal_score	2.847884e−11	−0.4318655	7.557998e−07	NEG
169	CST7	Stromal_score	3.907342e−08	0.3623620	1.036970e−03	POS	LOC728613	Stromal_score	2.577517e−09	−0.3904423	6.840472e−05	NEG
170	MSRB3	Stromal_score	7.956135e−26	0.6342839	2.111479e−21	POS	VASH1	Stromal_score	2.700619e−17	0.5325654	7.167173e−13	POS
171	ENTPD2	Stromal_score	8.778788e−07	−0.3264946	2.329803e−02	NEG	EPHA10	Stromal_score	1.808035e−07	−0.3452660	4.798343e−03	NEG
172	CLDN4	Stromal_score	1.862697e−06	−0.3170976	4.943412e−02	NEG	MIPEP	Stromal_score	1.292229e−06	−0.3217049	3.429447e−02	NEG
173	TMC5	Stromal_score	7.867288e−07	−0.3278380	2.087900e−02	NEG	FAM190B	Stromal_score	1.123053e−08	0.3755719	2.980470e−04	POS
174	SLCO4A1	Stromal_score	1.286533e−06	−0.3217601	3.414330e−02	NEG	DOK5	Stromal_score	1.911186e−19	0.5617303	5.072095e−15	POS
175	CPVL	Stromal_score	4.128076e−07	0.3356135	1.095550e−02	POS	EEF1E1	Stromal_score	1.029363e−06	−0.3245324	2.731828e−02	NEG
176	SGCA	Stromal_score	2.342555e−12	0.4526152	6.216906e−08	POS	C3orf45	Stromal_score	1.746197e−14	−0.4896415	4.634232e−10	NEG
177	PTPRC	Stromal_score	2.877115e−11	0.4317778	7.635575e−07	POS	SNHG12	Stromal_score	1.021161e−07	−0.3517601	2.710060e−03	NEG
178	LIMS2	Stromal_score	3.419323e−22	0.5952463	9.074540e−18	POS	PLEKHG1	Stromal_score	3.643098e−08	0.3631198	9.668418e−04	POS
179	SLC9A2	Stromal_score	1.179246e−08	−0.3750656	3.129601e−04	NEG	HOXA2	Stromal_score	1.126180e−07	0.3506577	2.988769e−03	POS
180	SVIL	Stromal_score	3.095775e−14	0.4855371	8.215878e−10	POS	CORO1C	Stromal_score	2.007434e−10	0.4146014	5.327528e−06	POS
181	SLIT3	Stromal_score	5.316010e−23	0.6044118	1.410816e−18	POS	SNORD34	Stromal_score	5.949382e−08	−0.3577697	1.578906e−03	NEG
182	THBS1	Stromal_score	3.164968e−26	0.6382676	8.399509e−22	POS	ARHGAP12	Stromal_score	5.008180e−07	−0.3333057	1.329121e−02	NEG
183	EVI2B	Stromal_score	3.283174e−14	0.4851127	8.713215e−10	POS	DVL1	Stromal_score	1.098946e−08	−0.3757967	2.916493e−04	NEG
184	MYOC	Stromal_score	4.554895e−08	0.3606954	1.208824e−03	POS	ZNF385D	Stromal_score	4.462211e−19	0.5569330	1.184226e−14	POS
185	C11orf96	Stromal_score	1.616282e−21	0.5873671	4.289451e−17	POS	SMPD2	Stromal_score	1.478051e−09	−0.3958733	3.922599e−05	NEG
186	GAS1	Stromal_score	1.074452e−28	0.6616114	2.851488e−24	POS	ANO6	Stromal_score	2.106303e−08	0.3689831	5.589916e−04	POS
187	GPX3	Stromal_score	2.389553e−18	0.5472051	6.341634e−14	POS	P2RX7	Stromal_score	1.904887e−14	0.4890216	5.055380e−10	POS
188	POSTN	Stromal_score	1.207866e−11	0.4391500	3.205555e−07	POS	PPAPDC3	Stromal_score	1.547253e−21	0.5875915	4.106254e−17	POS
189	ADCY5	Stromal_score	1.414399e−15	0.5070348	3.753673e−11	POS	PSMG4	Stromal_score	1.339126e−06	−0.3212590	3.553907e−02	NEG
190	CCDC69	Stromal_score	2.799552e−23	0.6075024	7.429731e−19	POS	MYO5A	Stromal_score	3.832316e−20	0.5706137	1.017058e−15	POS
191	SCARA3	Stromal_score	5.199133e−16	0.5136947	1.379798e−11	POS	POLR1C	Stromal_score	1.120175e−09	−0.3985446	2.972832e−05	NEG
192	DCN	Stromal_score	1.786858e−32	0.6937044	4.742143e−28	POS	AK094859	Stromal_score	2.796697e−10	−0.4115702	7.422154e−06	NEG
193	CLEC3B	Stromal_score	8.785547e−14	0.4779163	2.331596e−09	POS	RELN	Stromal_score	4.596899e−07	0.3343313	1.219971e−02	POS
194	CD3E	Stromal_score	6.727329e−08	0.3564135	1.785366e−03	POS	SOGA3	Stromal_score	6.979593e−11	0.4240622	1.852314e−06	POS
195	SDPR	Stromal_score	2.386299e−21	0.5853566	6.332999e−17	POS	PRR22	Stromal_score	1.185511e−07	−0.3500778	3.146227e−03	NEG
196	TFPI2	Stromal_score	1.608021e−09	0.3950565	4.267528e−05	POS	SERINC1	Stromal_score	3.185768e−14	0.4853302	8.454710e−10	POS
197	NGFRAP1	Stromal_score	8.803568e−09	0.3780839	2.336379e−04	POS	STX2	Stromal_score	4.183248e−07	0.3354555	1.110192e−02	POS
198	SERPINE1	Stromal_score	2.441526e−08	0.3674141	6.479567e−04	POS	SNORD50B	Stromal_score	3.143350e−07	−0.3388361	8.342138e−03	NEG
199	EPN3	Stromal_score	1.410546e−06	−0.3206079	3.743447e−02	NEG	MXD4	Stromal_score	1.974061e−07	0.3442542	5.238961e−03	POS
200	SMTN	Stromal_score	1.593904e−11	0.4368136	4.230063e−07	POS	SFXN4	Stromal_score	8.600649e−09	−0.3783233	2.282526e−04	NEG
201	ITGA7	Stromal_score	5.320537e−18	0.5424509	1.412017e−13	POS	PGAM5	Stromal_score	5.023725e−11	−0.4269478	1.333246e−06	NEG
202	FCRLA	Stromal_score	2.369368e−07	0.3421405	6.288066e−03	POS	MRPL50	Stromal_score	1.375657e−06	−0.3209220	3.650855e−02	NEG
203	RGS1	Stromal_score	2.309929e−07	0.3424356	6.130321e−03	POS	POLA2	Stromal_score	6.123117e−11	−0.4252144	1.625014e−06	NEG
204	COL3A1	Stromal_score	2.066859e−20	0.5739572	5.485238e−16	POS	NSUN5P2	Stromal_score	1.037230e−06	−0.3244382	2.752704e−02	NEG
205	RGS5	Stromal_score	7.295332e−11	0.4236718	1.936108e−06	POS	CR1	Stromal_score	5.300611e−09	0.3832506	1.406729e−04	POS
206	ISLR	Stromal_score	3.757014e−23	0.6060888	9.970740e−19	POS	LOC100130231	Stromal_score	1.606762e−06	0.3189692	4.264187e−02	POS
207	CCR7	Stromal_score	1.042652e−08	0.3763406	2.767093e−04	POS	NPR2	Stromal_score	3.580534e−19	0.5581859	9.502380e−15	POS
208	C1QC	Stromal_score	1.028842e−10	0.4206214	2.730443e−06	POS	PTGES2	Stromal_score	1.994708e−09	−0.3929579	5.293756e−05	NEG
209	DSG2	Stromal_score	1.157655e−07	−0.3503465	3.072302e−03	NEG	C6orf225	Stromal_score	7.332196e−11	0.4236273	1.945891e−06	POS
210	FBXO32	Stromal_score	1.221890e−16	0.5230806	3.242773e−12	POS	HSP90AA1	Stromal_score	3.576618e−08	−0.3633188	9.491987e−04	NEG
211	CYR61	Stromal_score	3.707591e−12	0.4489051	9.839575e−08	POS	BMPR2	Stromal_score	3.126492e−13	0.4683797	8.297398e−09	POS
212	SHISA3	Stromal_score	3.860264e−13	0.4667668	1.024476e−08	POS	GPATCH4	Stromal_score	3.743249e−07	−0.3367748	9.934208e−03	NEG
213	C1QA	Stromal_score	1.289721e−11	0.4385992	3.422791e−07	POS	DNMBP	Stromal_score	1.327559e−06	−0.3213676	3.523208e−02	NEG
214	FCGR3A	Stromal_score	7.611572e−07	0.3282417	2.020035e−02	POS	ZNF664	Stromal_score	1.816028e−08	−0.3705500	4.819558e−04	NEG
215	NCS1	Stromal_score	5.508043e−10	0.4052783	1.461779e−05	POS	GNAI2	Stromal_score	7.029425e−20	0.5672912	1.865539e−15	POS
216	SNCG	Stromal_score	4.659612e−09	0.3845490	1.236614e−04	POS	AX747770	Stromal_score	3.342762e−09	−0.3878691	8.871355e−05	NEG
217	SSC5D	Stromal_score	2.069906e−28	0.6590219	5.493324e−24	POS	TIMM17B	Stromal_score	1.115974e−08	−0.3756374	2.961684e−04	NEG
218	BAIAP2L2	Stromal_score	7.786280e−07	−0.3279645	2.066401e−02	NEG	MAPK10	Stromal_score	6.540107e−22	0.5919828	1.735679e−17	POS
219	EGR1	Stromal_score	1.940968e−07	0.3444492	5.151134e−03	POS	C10orf2	Stromal_score	6.777687e−11	−0.4243209	1.798730e−06	NEG
220	AJ004954	Stromal_score	2.030993e−07	0.3439260	5.390053e−03	POS	RTKN2	Stromal_score	1.476607e−08	−0.3727225	3.918766e−04	NEG
221	RSPO3	Stromal_score	9.825612e−23	0.6014194	2.607619e−18	POS	SMPD1	Stromal_score	7.804447e−14	0.4787910	2.071222e−09	POS
222	ITGB2	Stromal_score	5.468461e−10	0.4053459	1.451275e−05	POS	C6orf130	Stromal_score	2.504441e−08	−0.3671430	6.646537e−04	NEG
223	PCDH7	Stromal_score	3.809644e−07	0.3365665	1.011041e−02	POS	GAB1	Stromal_score	6.399911e−10	0.4038672	1.698472e−05	POS
224	RNF128	Stromal_score	3.836574e−08	−0.3625600	1.018188e−03	NEG	RFWD3	Stromal_score	9.235216e−08	−0.3528876	2.450934e−03	NEG
225	PRIMA1	Stromal_score	7.544800e−11	0.4233748	2.002314e−06	POS	PTPRB	Stromal_score	6.554913e−09	0.3810985	1.739608e−04	POS
226	CD53	Stromal_score	2.761425e−12	0.4512911	7.328545e−08	POS	MAMLD1	Stromal_score	2.729411e−13	0.4694144	7.243583e−09	POS
227	CAV1	Stromal_score	2.457855e−20	0.5730228	6.522901e−16	POS	RMI1	Stromal_score	1.037985e−12	−0.4590844	2.754707e−08	NEG
228	SMYD1	Stromal_score	1.743384e−07	0.3456843	4.626766e−03	POS	AMMECR1	Stromal_score	1.150760e−08	−0.3753193	3.054002e−04	NEG
229	BC073897	Stromal_score	7.276164e−09	0.3800350	1.931021e−04	POS	TIAM1	Stromal_score	3.590767e−07	0.3372670	9.529537e−03	POS
230	ATP1A2	Stromal_score	4.537794e−13	0.4655239	1.204285e−08	POS	CENPL	Stromal_score	2.889145e−15	−0.5021915	7.667503e−11	NEG
231	CACNA1H	Stromal_score	2.899103e−14	0.4860104	7.693929e−10	POS	PCMTD1	Stromal_score	1.051575e−07	0.3514300	2.790774e−03	POS
232	FAIM3	Stromal_score	1.353363e−08	0.3736328	3.591690e−04	POS	KAT2A	Stromal_score	2.203666e−08	−0.3685039	5.848310e−04	NEG
233	C2orf72	Stromal_score	1.603573e−08	−0.3718583	4.255723e−04	NEG	PEA15	Stromal_score	1.339421e−22	0.5998981	3.554690e−18	POS
234	MRVI1	Stromal_score	9.101112e−23	0.6017942	2.415344e−18	POS	RAPGEF1	Stromal_score	3.386605e−11	0.4303728	8.987711e−07	POS
235	NUPR1	Stromal_score	1.458864e−13	0.4741417	3.871678e−09	POS	RRAGA	Stromal_score	5.667931e−10	0.4050097	1.504212e−05	POS
236	PDZRN3	Stromal_score	2.836838e−14	0.4861667	7.528685e−10	POS	ADHFE1	Stromal_score	3.690697e−11	0.4296293	9.794740e−07	POS
237	SERPINF1	Stromal_score	1.150248e−29	0.6702469	3.052643e−25	POS	RAP1A	Stromal_score	2.688579e−12	0.4515067	7.135219e−08	POS
238	MMP2	Stromal_score	4.074762e−23	0.6056974	1.081401e−18	POS	SHE	Stromal_score	2.369898e−23	0.6082999	6.289473e−19	POS
239	TGM2	Stromal_score	9.728871e−07	0.3252295	2.581945e−02	POS	SIGLEC9	Stromal_score	6.279709e−07	0.3305799	1.666572e−02	POS
240	CFL2	Stromal_score	5.149487e−19	0.5561148	1.366622e−14	POS	NRIP2	Stromal_score	1.192658e−13	0.4756468	3.165195e−09	POS

	gene	feature	p.value	estimate	adj.p.value	COR.ID	gene	feature	p.value	estimate	adj.p.value	COR.ID
241	DNAJB5	Stromal_score	1.551169e−19	0.5628994	4.116648e−15	POS	NCKAP5L	Stromal_score	2.178104e−15	0.5041163	5.780469e−11	POS
242	CDC6	Stromal_score	2.763431e−13	−0.4693201	7.333869e−09	NEG	MVK	Stromal_score	6.512173e−10	−0.4037032	1.728266e−05	NEG
243	CSRP1	Stromal_score	1.655801e−14	0.4900198	4.394331e−10	POS	PEG3	Stromal_score	1.130887e−19	0.5646607	3.001262e−15	POS
244	UCHL1	Stromal_score	2.031904e−12	0.4537558	5.392470e−08	POS	ESRRA	Stromal_score	6.739303e−10	−0.4033798	1.788544e−05	NEG
245	CD27	Stromal_score	2.609462e−07	0.3410164	6.925252e−03	POS	BC061632	Stromal_score	4.103069e−07	−0.3356857	1.088913e−02	NEG
246	TGFB1I1	Stromal_score	8.377469e−27	0.6439106	2.223296e−22	POS	TRIM38	Stromal_score	3.546707e−08	0.3634095	9.412607e−04	POS
247	PPP1R1A	Stromal_score	3.690412e−07	0.3369432	9.793986e−03	POS	ZNF711	Stromal_score	9.594776e−07	−0.3254007	2.546358e−02	NEG
248	FOLR2	Stromal_score	7.870576e−14	0.4787288	2.088772e−09	POS	TMEM41B	Stromal_score	4.289694e−09	−0.3853793	1.138442e−04	NEG
249	CXCR4	Stromal_score	5.805123e−08	0.3580398	1.540622e−03	POS	ITPRIPL2	Stromal_score	2.270306e−07	0.3426365	6.025164e−03	POS
250	FERMT2	Stromal_score	2.185143e−29	0.6677962	5.799150e−25	POS	ZBTB42	Stromal_score	9.045817e−07	−0.3261263	2.400669e−02	NEG
251	CD44	Stromal_score	5.489808e−07	0.3322027	1.456940e−02	POS	ALYREF	Stromal_score	3.101058e−07	−0.3389953	8.229897e−03	NEG
252	RGS2	Stromal_score	2.348353e−11	0.4335187	6.232293e−07	POS	SCARNA12	Stromal_score	1.396831e−07	−0.3482173	3.707050e−03	NEG
253	FMOD	Stromal_score	1.139614e−12	0.4583489	3.024422e−08	POS	PCDHGC5	Stromal_score	1.786192e−13	0.4726220	4.740375e−09	POS
254	GPNMB	Stromal_score	2.939034e−17	0.5320416	7.799902e−13	POS	PINK1	Stromal_score	6.094336e−09	0.3818385	1.617376e−04	POS
255	C5orf4	Stromal_score	1.553265e−12	0.4558979	4.122211e−08	POS	MAD2L1BP	Stromal_score	3.991600e−09	−0.3861004	1.059331e−04	NEG
256	COL1A2	Stromal_score	8.591879e−18	0.5395681	2.280199e−13	POS	SNORD24	Stromal_score	4.579762e−09	−0.3847227	1.215423e−04	NEG
257	PSD	Stromal_score	1.122425e−12	0.4584687	2.978804e−08	POS	LRRC8C	Stromal_score	1.567054e−18	0.5496809	4.158806e−14	POS
258	SEMA3C	Stromal_score	2.462264e−10	0.4127381	6.534602e−06	POS	TM6SF1	Stromal_score	7.769539e−25	0.6241794	2.061958e−20	POS
259	TOX3	Stromal_score	4.617723e−08	−0.3605460	1.225498e−03	NEG	C4orf3	Stromal_score	1.739940e−17	0.5352728	4.617626e−13	POS
260	CSPG4	Stromal_score	1.496816e−13	0.4739493	3.972400e−09	POS	CLIP1	Stromal_score	3.854053e−10	0.4086095	1.022827e−05	POS
261	MAP1B	Stromal_score	4.466483e−20	0.5697785	1.185360e−15	POS	ZNF440	Stromal_score	5.908660e−07	−0.3313163	1.568099e−02	NEG
262	MFAP5	Stromal_score	2.048355e−27	0.6497639	5.436128e−23	POS	ZNF816	Stromal_score	1.773157e−07	−0.3454898	4.705781e−03	NEG
263	OVOL1	Stromal_score	1.811674e−06	−0.3174503	4.808002e−02	NEG	ESYT1	Stromal_score	5.183993e−07	0.3328917	1.375780e−02	POS
264	CCL2	Stromal_score	1.748802e−22	0.5985820	4.641146e−18	POS	CCDC120	Stromal_score	5.541435e−08	−0.3585507	1.470641e−03	NEG
265	C16orf89	Stromal_score	1.969960e−08	0.3696913	5.228076e−04	POS	AK093299	Stromal_score	1.080917e−13	0.4763791	2.868646e−09	POS
266	POF1B	Stromal_score	7.520860e−08	−0.3551776	1.995961e−03	NEG	GAP43	Stromal_score	1.341698e−12	0.4570592	3.560732e−08	POS
267	KCTD12	Stromal_score	1.468115e−16	0.5219066	3.896232e−12	POS	C20orf160	Stromal_score	8.545738e−15	0.4946881	2.267953e−10	POS
268	C7orf58	Stromal_score	9.939961e−20	0.5653768	2.637966e−15	POS	RNF152	Stromal_score	8.797531e−09	0.3780909	2.334777e−04	POS
269	POPDC2	Stromal_score	4.105452e−15	0.4997805	1.089546e−10	POS	CPSF4	Stromal_score	2.428263e−07	−0.3418550	6.444366e−03	NEG
270	APLNR	Stromal_score	1.260701e−17	0.5372416	3.345775e−13	POS	DAK	Stromal_score	3.499167e−10	−0.4095044	9.286439e−06	NEG
271	AB231710	Stromal_score	2.976651e−08	0.3652956	7.899735e−04	POS	RTN4IP1	Stromal_score	7.662999e−11	−0.4232374	2.033683e−06	NEG
272	NTN1	Stromal_score	8.175843e−11	0.4226641	2.169787e−06	POS	RRP1	Stromal_score	1.620707e−09	−0.3949803	4.301195e−05	NEG
273	LCP1	Stromal_score	3.192101e−10	0.4103528	8.471517e−06	POS	PET117	Stromal_score	1.319723e−06	−0.3214416	3.502414e−02	NEG
274	HNF4A	Stromal_score	2.461855e−09	−0.3908944	6.533518e−05	NEG	GGTA1P	Stromal_score	1.109190e−16	0.5236976	2.943680e−12	POS
275	HCST	Stromal_score	4.554335e−11	0.4278034	1.208675e−06	POS	POLA1	Stromal_score	2.953907e−07	−0.3395660	7.839374e−03	NEG
276	UGT8	Stromal_score	1.431672e−08	−0.3730456	3.799515e−04	NEG	KLHL17	Stromal_score	1.520148e−08	−0.3724183	4.034321e−04	NEG
277	NNMT	Stromal_score	1.484508e−19	0.5631448	3.939735e−15	POS	RDM1	Stromal_score	3.122169e−10	−0.4105571	8.285924e−06	NEG
278	BC127952	Stromal_score	1.610886e−07	0.3465903	4.275131e−03	POS	ZNF385B	Stromal_score	3.146442e−09	0.3884701	8.350344e−05	POS
279	PTRF	Stromal_score	3.389463e−30	0.6748494	8.995297e−26	POS	SEPN1	Stromal_score	1.821095e−08	0.3705206	4.833003e−04	POS
280	MYOCD	Stromal_score	3.148082e−13	0.4683272	8.354695e−09	POS	TMEM241	Stromal_score	2.525207e−09	−0.3906443	6.701647e−05	NEG
281	JUP	Stromal_score	2.510019e−08	−0.3671193	6.661339e−04	NEG	ABCA6	Stromal_score	3.338259e−22	0.5953663	8.859407e−18	POS
282	SPOCK1	Stromal_score	2.000517e−22	0.5979161	5.309173e−18	POS	ANKRD33B	Stromal_score	4.181556e−13	0.4661529	1.109743e−08	POS
283	DHCR24	Stromal_score	9.145504e−09	−0.3776921	2.427125e−04	NEG	MSH2	Stromal_score	1.027106e−09	−0.3993756	2.725837e−05	NEG
284	PRR15L	Stromal_score	4.779677e−14	−0.4823867	1.268479e−09	NEG	TMEM201	Stromal_score	2.833563e−09	−0.3895074	7.519992e−05	NEG
285	PPAP2C	Stromal_score	1.283609e−08	−0.3741842	3.406571e−04	NEG	RAB9B	Stromal_score	3.175742e−08	0.3646001	8.428103e−04	POS
286	DUSP1	Stromal_score	1.393168e−10	0.4179060	3.697329e−06	POS	ZBTB38	Stromal_score	6.772180e−07	0.3296644	1.797269e−02	POS
287	MIR143HG	Stromal_score	2.563974e−15	0.5030065	6.804530e−11	POS	KIF24	Stromal_score	1.723029e−11	−0.4361541	4.572746e−07	NEG
288	ASB2	Stromal_score	1.154820e−16	0.5234407	3.064778e−12	POS	ANKRD50	Stromal_score	1.188056e−09	0.3979797	3.152981e−05	POS
289	CRLF1	Stromal_score	1.084210e−09	0.3988575	2.877384e−05	POS	TMEM68	Stromal_score	1.988007e−09	−0.3929908	5.275973e−05	NEG
290	BC005081	Stromal_score	6.978652e−09	−0.3804608	1.852064e−04	NEG	TSEN54	Stromal_score	2.907477e−16	−0.5174966	7.716152e−12	NEG
291	CLMP	Stromal_score	2.441200e−31	0.6844840	6.478701e−27	POS	ZNF714	Stromal_score	2.241622e−07	−0.3427840	5.949040e−03	NEG
292	FBLN2	Stromal_score	3.772191e−26	0.6375136	1.001102e−21	POS	NSUN2	Stromal_score	8.769511e−09	−0.3781237	2.327341e−04	NEG
293	ADAMTS1	Stromal_score	5.599331e−23	0.6041600	1.486007e−18	POS	BC035106	Stromal_score	1.390723e−08	−0.3733487	3.690841e−04	NEG
294	PALLD	Stromal_score	2.373367e−24	0.6190897	6.298678e−20	POS	AK130759	Stromal_score	2.718163e−09	−0.3899182	7.213732e−05	NEG
295	SLC2A4	Stromal_score	3.550570e−09	0.3872690	9.422856e−05	POS	PIGW	Stromal_score	3.504226e−08	−0.3635396	9.299866e−04	NEG
296	CLIP3	Stromal_score	4.818440e−30	0.6735328	1.278766e−25	POS	CEP76	Stromal_score	9.561429e−10	−0.4000597	2.537508e−05	NEG
297	UBE2C	Stromal_score	8.276352e−14	−0.4783576	2.196461e−09	NEG	FDPS	Stromal_score	2.112866e−07	−0.3434691	5.607336e−03	NEG
298	TGFBI	Stromal_score	4.134692e−14	0.4834417	1.097306e−09	POS	SNORD52	Stromal_score	2.264287e−10	−0.4135043	6.009191e−06	NEG
299	CPXM1	Stromal_score	3.186218e−14	0.4853292	8.455903e−10	POS	TMEM71	Stromal_score	3.740168e−11	0.4295140	9.926032e−07	POS
300	FBLN5	Stromal_score	8.030681e−29	0.6627530	2.131262e−24	POS	WDR77	Stromal_score	1.221934e−06	−0.3224029	3.242890e−02	NEG
301	C1QTNF1	Stromal_score	8.080194e−11	0.4227683	2.144403e−06	POS	SLBP	Stromal_score	2.137454e−07	−0.3433352	5.672589e−03	NEG
302	STEAP4	Stromal_score	4.007133e−13	0.4664802	1.063453e−08	POS	BC069792	Stromal_score	8.346005e−10	−0.4013545	2.214946e−05	NEG
303	C10orf10	Stromal_score	8.591331e−10	0.4010790	2.280053e−05	POS	CDK2	Stromal_score	1.053537e−06	−0.3242451	2.795982e−02	NEG
304	DMPK	Stromal_score	2.100199e−08	0.3690139	5.573718e−04	POS	POP7	Stromal_score	8.935738e−11	−0.4218758	2.371456e−06	NEG
305	SPOCK2	Stromal_score	9.225220e−08	0.3528997	2.448281e−03	POS	BRCA2	Stromal_score	5.450266e−13	−0.4641095	1.446446e−08	NEG
306	CD48	Stromal_score	1.128030e−09	0.3984776	2.993679e−05	POS	BC031953	Stromal_score	4.971162e−08	−0.3597406	1.319297e−03	NEG
307	CD2	Stromal_score	2.564639e−07	0.3412186	6.806295e−03	POS	RHOQ	Stromal_score	8.023804e−22	0.5909462	2.129437e−17	POS
308	COL15A1	Stromal_score	1.009591e−25	0.6332450	2.679353e−21	POS	KATNAL1	Stromal_score	6.655655e−12	0.4441113	1.766344e−07	POS
309	KRT18	Stromal_score	4.146249e−08	−0.3617181	1.100373e−03	NEG	PRKD1	Stromal_score	4.500051e−28	0.6559214	1.194269e−23	POS
310	GNAO1	Stromal_score	6.411325e−17	0.5271692	1.701501e−12	POS	FCHSD2	Stromal_score	2.424198e−10	0.4128806	6.433578e−06	POS
311	LAPTM5	Stromal_score	5.707410e−12	0.4453779	1.514690e−07	POS	C17orf103	Stromal_score	1.946809e−07	0.3444145	5.166635e−03	POS
312	AEBP1	Stromal_score	6.130906e−26	0.6354160	1.627081e−21	POS	SAC3D1	Stromal_score	9.555063e−12	−0.4411106	2.535818e−07	NEG
313	MYBL2	Stromal_score	8.147329e−09	−0.3788786	2.162220e−04	NEG	ADAT3	Stromal_score	1.021301e−06	−0.3246296	2.710432e−02	NEG
314	BAG2	Stromal_score	3.909861e−08	0.3623550	1.037638e−03	POS	MTHFS	Stromal_score	1.939554e−11	−0.4351492	5.147381e−07	NEG
315	IGFBP6	Stromal_score	2.799621e−17	0.5323426	7.429914e−13	POS	REXO2	Stromal_score	8.557397e−07	0.3268082	2.271048e−02	POS
316	SERPING1	Stromal_score	2.213349e−28	0.6587558	5.874006e−24	POS	TECR	Stromal_score	1.725232e−08	−0.3710900	4.578593e−04	NEG
317	SLCO2A1	Stromal_score	3.822651e−14	0.4840113	1.014493e−09	POS	ARHGEF5	Stromal_score	5.209404e−07	−0.3328330	1.382524e−02	NEG
318	PTPRH	Stromal_score	8.206123e−09	−0.3788050	2.177823e−04	NEG	ODZ2	Stromal_score	1.379278e−06	0.3208890	3.660467e−02	POS
319	AX746920	Stromal_score	2.257718e−19	0.5607936	5.991758e−15	POS	TSPYL4	Stromal_score	1.910067e−07	0.3446342	5.069126e−03	POS
320	GREM2	Stromal_score	2.811824e−10	0.4115207	7.462300e−06	POS	C11orf83	Stromal_score	3.813387e−08	−0.3626256	1.012035e−03	NEG
321	ITGAL	Stromal_score	5.163858e−09	0.3835144	1.370436e−04	POS	DHX34	Stromal_score	1.088627e−07	−0.3510401	2.889108e−03	NEG
322	SRGN	Stromal_score	3.881420e−17	0.5303132	1.030090e−12	POS	TMEM79	Stromal_score	5.990529e−07	−0.3311501	1.589826e−02	NEG
323	ITGBL1	Stromal_score	1.422781e−18	0.5502448	3.775919e−14	POS	HSPA7	Stromal_score	3.478216e−10	0.4095600	9.230838e−06	POS
324	OMD	Stromal_score	1.882721e−25	0.6305081	4.996553e−21	POS	MEIS3	Stromal_score	4.929068e−18	0.5429080	1.308125e−13	POS
325	PRR15	Stromal_score	2.919560e−08	−0.3655033	7.748219e−04	NEG	THNSL1	Stromal_score	6.178905e−09	−0.3816986	1.639820e−04	NEG
326	DDR2	Stromal_score	5.269768e−32	0.6899320	1.398544e−27	POS	SNORD4B	Stromal_score	1.695615e−07	−0.3460031	4.499992e−03	NEG
327	RERG	Stromal_score	2.845492e−18	0.5461743	7.551650e−14	POS	ENDOG	Stromal_score	1.486205e−10	−0.4173238	3.944238e−06	NEG
328	ATP2B4	Stromal_score	4.427752e−23	0.6052964	1.175081e−18	POS	EXOSC8	Stromal_score	8.875821e−07	−0.3263596	2.355554e−02	NEG
329	ITPKA	Stromal_score	6.903130e−10	−0.4031530	1.832022e−05	NEG	ESYT2	Stromal_score	1.696695e−09	0.3945351	4.502858e−05	POS
330	CTSF	Stromal_score	9.392767e−10	0.4002295	2.492746e−05	POS	LHX6	Stromal_score	1.098204e−10	0.4200390	2.914525e−06	POS
331	TOP2A	Stromal_score	7.906840e−13	−0.4612173	2.098396e−08	NEG	DZIP1	Stromal_score	1.617773e−20	0.5752734	4.293409e−16	POS
332	LDOC1	Stromal_score	1.572791e−18	0.5496595	4.174031e−14	POS	STX11	Stromal_score	2.084083e−09	0.3925292	5.530948e−05	POS
333	AX746851	Stromal_score	3.071622e−09	−0.3887088	8.151778e−05	NEG	ACVR2B	Stromal_score	2.672759e−08	−0.3664487	7.093234e−04	NEG
334	TSPAN2	Stromal_score	4.829701e−16	0.5141795	1.281754e−11	POS	MRPL23	Stromal_score	5.489805e−07	−0.3322027	1.456939e−02	NEG
335	CYBB	Stromal_score	9.290151e−12	0.4413450	2.465513e−07	POS	CCZ1B	Stromal_score	2.384884e−09	−0.3912068	6.329244e−05	NEG
336	PNCK	Stromal_score	3.754291e−07	0.3367400	9.963513e−03	POS	TMEM121	Stromal_score	7.304377e−08	0.3555018	1.938509e−03	POS
337	BOC	Stromal_score	1.903805e−32	0.6934848	5.052508e−28	POS	MRPS18A	Stromal_score	1.292634e−07	−0.3490981	3.430520e−03	NEG
338	A2M	Stromal_score	2.343429e−24	0.6191481	6.219227e−20	POS	UFSP1	Stromal_score	5.513031e−07	−0.3321519	1.463103e−02	NEG
339	COL6A3	Stromal_score	8.927483e−29	0.6623384	2.369265e−24	POS	RHPN1−AS1	Stromal_score	7.133326e−10	−0.4028429	1.893113e−05	NEG
340	ANGPTL2	Stromal_score	9.251946e−26	0.6336262	2.455374e−21	POS	YARS2	Stromal_score	2.393692e−07	−0.3420218	6.352620e−03	NEG
341	RRAD	Stromal_score	1.240776e−10	0.4189465	3.292896e−06	POS	MEF2D	Stromal_score	2.697038e−12	0.4514813	7.157670e−08	POS
342	ZCCHC24	Stromal_score	7.609137e−31	0.6803656	2.019389e−26	POS	RAB8B	Stromal_score	3.924232e−14	0.4838211	1.041452e−09	POS
343	SELM	Stromal_score	7.208853e−17	0.5264300	1.913157e−12	POS	PIGO	Stromal_score	1.686782e−07	−0.3460630	4.476550e−03	NEG
344	BHLHE41	Stromal_score	1.824428e−09	0.3938287	4.841849e−05	POS	MAP3K9	Stromal_score	4.192147e−09	−0.3856098	1.112554e−04	NEG
345	CAV2	Stromal_score	1.027654e−07	0.3516889	2.727290e−03	POS	PFDN6	Stromal_score	1.413535e−06	−0.3205814	3.751380e−02	NEG
346	SLC4A11	Stromal_score	1.438355e−07	−0.3478838	3.817251e−03	NEG	HSD17B7	Stromal_score	2.353171e−12	−0.4525789	6.245081e−08	NEG
347	AK309441	Stromal_score	7.968978e−16	0.5108705	2.114887e−11	POS	KCTD10	Stromal_score	1.016408e−20	0.5777542	2.697445e−16	POS
348	ITGA5	Stromal_score	4.825203e−20	0.5693563	1.280561e−15	POS	MSI2	Stromal_score	8.862976e−08	−0.3533478	2.352145e−03	NEG
349	KRT8	Stromal_score	8.224086e−08	−0.3541830	2.182590e−03	NEG	ASPA	Stromal_score	9.460776e−11	0.4213684	2.510795e−06	POS
350	S1PR3	Stromal_score	1.776568e−25	0.6307642	4.714833e−21	POS	INO80C	Stromal_score	3.249512e−07	−0.3384452	8.623880e−03	NEG
351	FOXF1	Stromal_score	7.868600e−19	0.5536799	2.088248e−14	POS	JDP2	Stromal_score	3.853185e−08	0.3625132	1.022597e−03	POS
352	NAPSB	Stromal_score	1.558556e−09	0.3953596	4.136252e−05	POS	YWHAZ	Stromal_score	1.015698e−07	−0.3518204	2.695562e−03	NEG
353	CECR1	Stromal_score	1.446031e−14	0.4909817	3.837621e−10	POS	HDAC1	Stromal_score	1.293217e−07	−0.3490930	3.432068e−03	NEG
354	LOC100127983	Stromal_score	1.451997e−17	0.5363799	3.853456e−13	POS	SLC25A19	Stromal_score	8.802865e−10	−0.4008476	2.336192e−05	NEG
355	MCAM	Stromal_score	2.444837e−19	0.5603450	6.488353e−15	POS	RNF38	Stromal_score	1.314181e−09	0.3970085	3.487706e−05	POS
356	C1R	Stromal_score	5.441500e−37	0.7272254	1.444120e−32	POS	CCDC138	Stromal_score	6.161617e−16	−0.5125746	1.635232e−11	NEG
357	SULF1	Stromal_score	3.266729e−13	0.4680447	8.669571e−09	POS	RGS9	Stromal_score	1.358602e−10	0.4181319	3.605594e−06	POS
358	FA2H	Stromal_score	4.626243e−07	−0.3342553	1.227759e−02	NEG	CNTN4	Stromal_score	5.893888e−13	0.4635035	1.564179e−08	POS
359	METTL7A	Stromal_score	9.189551e−10	0.4004381	2.438815e−05	POS	CASQ1	Stromal_score	5.478298e−07	0.3322279	1.453885e−02	POS
360	FOXF2	Stromal_score	6.872698e−07	0.3294854	1.823945e−02	POS	SNCA	Stromal_score	1.529744e−09	0.3955404	4.059787e−05	POS

	gene	feature	p.value	estimate	adj.p.value	COR.ID	gene	feature	p.value	estimate	adj.p.value	COR.ID
361	HOPX	Stromal_score	2.642777e−16	0.5181165	7.013667e−12	POS	NLE1	Stromal_score	9.036465e−08	−0.3531311	2.398188e−03	NEG
362	BC047373	Stromal_score	2.247380e−19	0.5608195	5.964322e−15	POS	SIRT7	Stromal_score	1.216249e−09	−0.3977543	3.227803e−05	NEG
363	CORO1A	Stromal_score	3.799064e−09	0.3865944	1.008234e−04	POS	PPM1M	Stromal_score	2.593878e−15	0.5029274	6.883893e−11	POS
364	CHI3L2	Stromal_score	4.436066e−08	0.3609833	1.177287e−03	POS	GLP2R	Stromal_score	7.496735e−10	0.4023726	1.989559e−05	POS
365	LOC100128338	Stromal_score	1.129576e−09	−0.3984645	2.997782e−05	NEG	C20orf166−AS1	Stromal_score	4.101111e−08	0.3618370	1.088394e−03	POS
366	INMT	Stromal_score	1.657475e−24	0.6207362	4.398772e−20	POS	EZH1	Stromal_score	3.894752e−10	0.4085120	1.033628e−05	POS
367	BGN	Stromal_score	1.563965e−22	0.5991340	4.150606e−18	POS	GLS2	Stromal_score	2.160541e−07	−0.3432109	5.733860e−03	NEG
368	EPCAM	Stromal_score	3.596058e−13	−0.4673101	9.543577e−09	NEG	AK021888	Stromal_score	4.289248e−08	0.3613496	1.138324e−03	POS
369	TPM1	Stromal_score	2.494057e−10	0.4126206	6.618978e−06	POS	CERS2	Stromal_score	1.055295e−06	−0.3242245	2.800648e−02	NEG
370	RAB34	Stromal_score	9.839141e−08	0.3521775	2.611209e−03	POS	THAP10	Stromal_score	1.879430e−07	−0.3448204	4.987819e−03	NEG
371	PLEK	Stromal_score	8.567889e−08	0.3537261	2.273832e−03	POS	ZFP82	Stromal_score	4.157828e−12	0.4479719	1.103446e−07	POS
372	ITIH5	Stromal_score	3.045841e−19	0.5591031	8.083357e−15	POS	TBCEL	Stromal_score	1.381998e−08	0.3734144	3.667684e−04	POS
373	SMPD3	Stromal_score	2.079103e−07	−0.3436554	5.517732e−03	NEG	FAM117B	Stromal_score	1.402524e−07	−0.3481710	3.722159e−03	NEG
374	C1S	Stromal_score	2.408434e−35	0.7155569	6.391743e−31	POS	BACH2	Stromal_score	1.578060e−12	0.4557720	4.188012e−08	POS
375	SASH3	Stromal_score	2.178907e−10	0.4138549	5.782602e−06	POS	MED14	Stromal_score	2.911022e−07	−0.3397375	7.725562e−03	NEG
376	BIK	Stromal_score	8.783817e−11	−0.4220281	2.331137e−06	NEG	PTGR2	Stromal_score	3.347044e−07	−0.3380968	8.882720e−03	NEG
377	TRIM22	Stromal_score	6.475280e−12	0.4443381	1.718475e−07	POS	LINC00294	Stromal_score	1.244348e−06	0.3221763	3.302376e−02	POS
378	WWTR1	Stromal_score	7.263011e−18	0.5405819	1.927531e−13	POS	AFAP1L1	Stromal_score	9.845434e−13	0.4594998	2.612880e−08	POS
379	DACT3	Stromal_score	7.970214e−19	0.5536059	2.115215e−14	POS	WDR4	Stromal_score	8.461265e−07	−0.3269468	2.245535e−02	NEG
380	MT1A	Stromal_score	5.984252e−07	0.3311627	1.588161e−02	POS	ACTR5	Stromal_score	1.234093e−13	−0.4753922	3.275159e−09	NEG
381	GYPC	Stromal_score	6.494210e−33	0.6971827	1.723498e−28	POS	MOCS3	Stromal_score	1.173706e−06	−0.3229044	3.114899e−02	NEG
382	SELP	Stromal_score	1.438852e−20	0.5759010	3.818568e−16	POS	COL21A1	Stromal_score	2.006321e−13	0.4717461	5.324575e−09	POS
383	ANXA1	Stromal_score	1.110218e−09	0.3986303	2.946409e−05	POS	TACR1	Stromal_score	3.834857e−11	0.4292973	1.017733e−06	POS
384	CD163	Stromal_score	6.757455e−14	0.4798517	1.793361e−09	POS	TMOD2	Stromal_score	3.034176e−08	0.3650901	8.052401e−04	POS
385	MPEG1	Stromal_score	4.131868e−15	0.4997363	1.096556e−10	POS	FAM55C	Stromal_score	3.521422e−13	0.4674707	9.345501e−09	POS
386	C17orf110	Stromal_score	1.532586e−10	−0.4170466	4.067331e−06	NEG	LSM5	Stromal_score	8.689652e−09	−0.3782176	2.306147e−04	NEG
387	FGF7	Stromal_score	1.116670e−28	0.6614599	2.963531e−24	POS	ARHGAP33	Stromal_score	1.331732e−06	−0.3213283	3.534283e−02	NEG
388	MAL2	Stromal_score	4.121582e−09	−0.3857798	1.093827e−04	NEG	SRPK1	Stromal_score	5.202470e−10	−0.4058132	1.380684e−05	NEG
389	MYB	Stromal_score	7.499596e−07	−0.3284225	1.990318e−02	NEG	SKI	Stromal_score	7.103100e−17	0.5265233	1.885092e−12	POS
390	SAPCD2	Stromal_score	2.140219e−12	−0.4533400	5.679927e−08	NEG	BX538254	Stromal_score	2.897852e−12	0.4509018	7.690611e−08	POS
391	CFH	Stromal_score	5.814752e−24	0.6149372	1.543177e−19	POS	DUSP26	Stromal_score	2.801931e−08	0.3659439	7.436044e−04	POS
392	ALDH1A3	Stromal_score	2.878584e−17	0.5321703	7.639475e−13	POS	PTGER3	Stromal_score	1.016362e−25	0.6332158	2.697323e−21	POS
393	MT2A	Stromal_score	7.269512e−07	0.3288024	1.929256e−02	POS	NSUN5	Stromal_score	8.466031e−11	−0.4223550	2.246800e−06	NEG
394	MXRA8	Stromal_score	7.122918e−22	0.5915504	1.890351e−17	POS	CCDC51	Stromal_score	3.393546e−07	−0.3379341	9.006132e−03	NEG
395	EHD2	Stromal_score	1.793273e−26	0.6406939	4.759167e−22	POS	ANKRD35	Stromal_score	3.928859e−14	0.4838125	1.042680e−09	POS
396	TIMP3	Stromal_score	9.427947e−27	0.6434138	2.502083e−22	POS	CHL1	Stromal_score	5.746345e−12	0.4453220	1.525022e−07	POS
397	PPP1R3C	Stromal_score	5.134775e−11	0.4267568	1.362718e−06	POS	ENTPD1	Stromal_score	2.801899e−29	0.6668405	7.435961e−25	POS
398	HIST1H2BD	Stromal_score	4.803325e−07	−0.3338060	1.274754e−02	NEG	ZNF25	Stromal_score	3.213830e−19	0.5587989	8.529184e−15	POS
399	PLAC9	Stromal_score	1.510541e−17	0.5361383	4.008825e−13	POS	TOPBP1	Stromal_score	2.914224e−07	−0.3397246	7.734060e−03	NEG
400	C13orf33	Stromal_score	8.775058e−24	0.6130094	2.328813e−19	POS	GPR176	Stromal_score	5.948434e−10	0.4045558	1.578655e−05	POS
401	SLC16A2	Stromal_score	7.382586e−11	0.4235668	1.959264e−06	POS	SNX29	Stromal_score	1.706770e−19	0.5623645	4.529598e−15	POS
402	DKFZp547J0510	Stromal_score	1.145446e−17	0.5378250	3.039898e−13	POS	KANK3	Stromal_score	9.203165e−16	0.5099124	2.442428e−11	POS
403	C11orf9	Stromal_score	4.701850e−07	−0.3340615	1.247824e−02	NEG	FOXRED1	Stromal_score	3.358959e−08	−0.3639963	8.914340e−04	NEG
404	GEM	Stromal_score	4.304977e−22	0.5940915	1.142498e−17	POS	MNF1	Stromal_score	1.636917e−12	−0.4554807	4.344215e−08	NEG
405	TREM2	Stromal_score	3.924756e−08	0.3623138	1.041591e−03	POS	LIN9	Stromal_score	3.513488e−17	−0.5309333	9.324445e−13	NEG
406	PTN	Stromal_score	9.710225e−15	0.4937920	2.576997e−10	POS	KCTD20	Stromal_score	1.338226e−08	0.3737501	3.551517e−04	POS
407	COL12A1	Stromal_score	1.273106e−12	0.4574744	3.378697e−08	POS	TBC1D2B	Stromal_score	1.050284e−13	0.4765927	2.787349e−09	POS
408	JAM2	Stromal_score	1.320085e−27	0.6515637	3.503374e−23	POS	LCN12	Stromal_score	5.993073e−08	−0.3576891	1.590502e−03	NEG
409	CAP2	Stromal_score	1.061474e−12	0.4589084	2.817047e−08	POS	MCCC1	Stromal_score	2.251765e−07	−0.3427316	5.975959e−03	NEG
410	LUM	Stromal_score	3.082633e−24	0.6178844	8.180999e−20	POS	NR2C2AP	Stromal_score	1.514532e−08	−0.3724571	4.019417e−04	NEG
411	MXRA5	Stromal_score	6.248127e−08	0.3572298	1.658190e−03	POS	HOOK2	Stromal_score	3.042408e−12	−0.4505084	8.074247e−08	NEG
412	CTHRC1	Stromal_score	1.153778e−08	0.3752921	3.062012e−04	POS	GNL3	Stromal_score	8.498949e−11	−0.4223206	2.255536e−06	NEG
413	TMEM119	Stromal_score	9.166196e−28	0.6530487	2.432617e−23	POS	NUPL1	Stromal_score	5.276710e−10	−0.4056805	1.400386e−05	NEG
414	ELF3	Stromal_score	1.901223e−13	−0.4721520	5.045655e−09	NEG	PPIH	Stromal_score	8.750295e−07	−0.3265346	2.322241e−02	NEG
415	COL6A2	Stromal_score	1.303741e−33	0.7026004	3.459998e−29	POS	HIST1H2AJ	Stromal_score	1.848059e−07	−0.3450142	4.904564e−03	NEG
416	USH1C	Stromal_score	6.205688e−08	−0.3573049	1.646928e−03	NEG	ATXN1	Stromal_score	3.777743e−16	0.5157897	1.002575e−11	POS
417	IL7R	Stromal_score	3.226585e−10	0.4102537	8.563034e−06	POS	C11orf84	Stromal_score	3.529012e−07	−0.3374720	9.365646e−03	NEG
418	NDN	Stromal_score	4.712353e−32	0.6903247	1.250611e−27	POS	AIFM1	Stromal_score	8.672773e−09	−0.3782376	2.301667e−04	NEG
419	LAD1	Stromal_score	1.629357e−09	−0.3949286	4.324151e−05	NEG	KCNK2	Stromal_score	2.869721e−11	0.4317999	7.615953e−07	POS
420	CTF1	Stromal_score	2.641733e−08	0.3665734	7.010895e−04	POS	GMPS	Stromal_score	1.089732e−06	−0.3238265	2.892040e−02	NEG
421	C1orf162	Stromal_score	2.088325e−18	0.5479980	5.542206e−14	POS	COQ3	Stromal_score	1.007808e−06	−0.3247941	2.674621e−02	NEG
422	TXNIP	Stromal_score	2.879426e−12	0.4509534	7.641707e−08	POS	PTPN14	Stromal_score	1.752402e−08	0.3709256	4.650699e−04	POS
423	FBN1	Stromal_score	9.370843e−35	0.7112262	2.486928e−30	POS	PI4K2B	Stromal_score	5.622700e−07	−0.3319146	1.492208e−02	NEG
424	DAAM2	Stromal_score	4.858470e−20	0.5693187	1.289389e−15	POS	MS4A2	Stromal_score	3.331119e−08	0.3640859	8.840457e−04	POS
425	LDB3	Stromal_score	7.097840e−10	0.4028901	1.883696e−05	POS	PEG3−AS1	Stromal_score	2.172034e−17	0.5339098	5.764360e−13	POS
426	OLFML2B	Stromal_score	3.254417e−24	0.6176337	8.636897e−20	POS	PTHLH	Stromal_score	1.046173e−10	0.4204724	2.776438e−06	POS
427	DD182001	Stromal_score	9.531569e−18	0.5389401	2.529583e−13	POS	GAS8	Stromal_score	4.942372e−07	−0.3334643	1.311656e−02	NEG
428	HEYL	Stromal_score	1.645788e−19	0.5625682	4.367758e−15	POS	LOC100294145	Stromal_score	2.306894e−07	−0.3424509	6.122265e−03	NEG
429	MAMDC2	Stromal_score	1.289982e−15	0.5076537	3.423482e−11	POS	AZI1	Stromal_score	1.642352e−11	−0.4365603	4.358639e−07	NEG
430	NR2F1	Stromal_score	3.441552e−16	0.5163984	9.133536e−12	POS	WDR3	Stromal_score	5.005038e−08	−0.3596664	1.328287e−03	NEG
431	EMILIN1	Stromal_score	5.225334e−39	0.7407482	1.386751e−34	POS	BRIP1	Stromal_score	1.304378e−13	−0.4749788	3.461688e−09	NEG
432	PRKCB	Stromal_score	8.062067e−19	0.5535398	2.139592e−14	POS	PDHX	Stromal_score	2.258253e−07	−0.3426982	5.993177e−03	NEG
433	MXRA7	Stromal_score	1.578450e−19	0.5628019	4.189047e−15	POS	C19orf73	Stromal_score	1.304013e−06	−0.3215914	3.460719e−02	NEG
434	BASP1	Stromal_score	5.266511e−18	0.5425120	1.397679e−13	POS	ZNF473	Stromal_score	8.066603e−09	−0.3789806	2.140796e−04	NEG
435	IGSF9	Stromal_score	1.883920e−10	−0.4151785	4.999734e−06	NEG	MIR25	Stromal_score	3.010719e−08	−0.3651734	7.990146e−04	NEG
436	LPP	Stromal_score	9.224834e−15	0.4941520	2.448179e−10	POS	ANGPT1	Stromal_score	1.256856e−13	0.4752559	3.335571e−09	POS
437	SQLE	Stromal_score	5.623047e−08	−0.3583901	1.492301e−03	NEG	MRTO4	Stromal_score	7.114634e−10	−0.4028677	1.888153e−05	NEG
438	SPEG	Stromal_score	3.832227e−11	0.4293033	1.017035e−06	POS	WDR5	Stromal_score	6.596287e−07	−0.3299839	1.750589e−02	NEG
439	TMEM52	Stromal_score	5.448010e−09	−0.3829736	1.445848e−04	NEG	TNFRSF1A	Stromal_score	4.722157e−08	0.3603020	1.253213e−03	POS
440	FYB	Stromal_score	1.485215e−10	0.4173298	3.941613e−06	POS	GPR65	Stromal_score	1.770875e−08	0.3708152	4.699725e−04	POS
441	IL17RB	Stromal_score	1.519392e−09	−0.3956062	4.032315e−05	NEG	TPP1	Stromal_score	2.858915e−09	0.3894193	7.587276e−05	POS
442	PID1	Stromal_score	1.282036e−06	0.3218038	3.402396e−02	POS	FANCE	Stromal_score	1.143370e−08	−0.3753861	3.034390e−04	NEG
443	CDC20	Stromal_score	4.458555e−12	−0.4474020	1.183256e−07	NEG	XRCC3	Stromal_score	3.657197e−08	−0.3630781	9.705836e−04	NEG
444	LHFP	Stromal_score	1.738402e−30	0.6773298	4.613545e−26	POS	CORO2B	Stromal_score	4.719445e−11	0.4274930	1.252494e−06	POS
445	CDCA7	Stromal_score	1.728743e−06	−0.3180442	4.587911e−02	NEG	OTUD3	Stromal_score	1.142671e−08	−0.3753924	3.032535e−04	NEG
446	C16orf45	Stromal_score	7.486201e−17	0.5261916	1.986763e−12	POS	C5orf34	Stromal_score	3.539474e−17	−0.5308874	9.393410e−13	NEG
447	RNASE6	Stromal_score	1.009306e−17	0.5385932	2.678598e−13	POS	URB2	Stromal_score	4.110398e−08	−0.3618124	1.090858e−03	NEG
448	MN1	Stromal_score	1.609134e−20	0.5753021	4.270482e−16	POS	MDH2	Stromal_score	4.808746e−07	−0.3337925	1.276193e−02	NEG
449	HTRA3	Stromal_score	1.515322e−15	0.5065708	4.021513e−11	POS	NDOR1	Stromal_score	8.579757e−09	−0.3783483	2.276982e−04	NEG
450	RAI2	Stromal_score	3.622013e−15	0.5006424	9.612461e−11	POS	MRPS30	Stromal_score	3.560211e−09	−0.3872419	9.448443e−05	NEG
451	SYNPO	Stromal_score	8.666867e−22	0.5905543	2.300100e−17	POS	ABCA9	Stromal_score	2.893091e−17	0.5321392	7.677975e−13	POS
452	CBX6	Stromal_score	5.881930e−12	0.4451301	1.561005e−07	POS	TBRG4	Stromal_score	2.751840e−13	−0.4693521	7.303108e−09	NEG
453	MST1R	Stromal_score	6.738210e−07	−0.3297255	1.788254e−02	NEG	ADAMTS16	Stromal_score	1.461757e−06	0.3201602	3.879357e−02	POS
454	COL5A1	Stromal_score	2.411454e−19	0.5604225	6.399759e−15	POS	CRY2	Stromal_score	9.561095e−09	0.3772346	2.537419e−04	POS
455	RBPMS	Stromal_score	6.021592e−12	0.4449369	1.598070e−07	POS	C19orf40	Stromal_score	8.849564e−07	−0.3263960	2.348586e−02	NEG
456	HP08874	Stromal_score	1.212106e−08	−0.3747802	3.216807e−04	NEG	TFB2M	Stromal_score	1.192140e−08	−0.3749527	3.163821e−04	NEG
457	AMH	Stromal_score	1.376645e−07	−0.3483829	3.653479e−03	NEG	NUDCD1	Stromal_score	5.296097e−08	−0.3590474	1.405531e−03	NEG
458	JA483376	Stromal_score	2.175745e−12	0.4532080	5.774209e−08	POS	ABCD3	Stromal_score	1.092483e−07	−0.3510003	2.899342e−03	NEG
459	LTBP1	Stromal_score	1.411000e−21	0.5880648	3.744654e−17	POS	NOC2L	Stromal_score	2.918323e−07	−0.3397081	7.744937e−03	NEG
460	SLC1A5	Stromal_score	2.812999e−08	−0.3659017	7.465417e−04	NEG	MAP4	Stromal_score	1.534428e−15	0.5064864	4.072219e−11	POS
461	VSIG4	Stromal_score	4.825733e−12	0.4467548	1.280701e−07	POS	ATL1	Stromal_score	1.004530e−08	0.3767252	2.665922e−04	POS
462	TYROBP	Stromal_score	2.624786e−12	0.4517002	6.965918e−08	POS	PEX7	Stromal_score	2.097845e−15	−0.5043712	5.567471e−11	NEG
463	KANK2	Stromal_score	3.267175e−22	0.5954740	8.670755e−18	POS	TMEM53	Stromal_score	1.332229e−09	−0.3968770	3.535601e−05	NEG
464	CYTIP	Stromal_score	1.732756e−06	0.3180148	4.598562e−02	POS	NINJ1	Stromal_score	1.018648e−12	0.4592323	2.703390e−08	POS
465	CTGF	Stromal_score	1.358409e−18	0.5505147	3.605081e−14	POS	LSM4	Stromal_score	5.270099e−10	−0.4056922	1.398632e−05	NEG
466	INHBA	Stromal_score	1.264287e−08	0.3743420	3.355291e−04	POS	KIAA1984	Stromal_score	1.532152e−07	−0.3471632	4.066179e−03	NEG
467	TSPAN18	Stromal_score	1.118079e−13	0.4761276	2.967270e−09	POS	NIPA1	Stromal_score	3.963390e−11	−0.4290115	1.051844e−06	NEG
468	C3orf70	Stromal_score	1.214645e−11	0.4391030	3.223547e−07	POS	TOR1AIP1	Stromal_score	6.932901e−11	0.4241214	1.839923e−06	POS
469	FCER1G	Stromal_score	4.308283e−08	0.3613014	1.143375e−03	POS	WBP1L	Stromal_score	1.158162e−19	0.5645282	3.073646e−15	POS
470	AF268386	Stromal_score	3.283980e−36	0.7217654	8.715355e−32	POS	PRKAR1A	Stromal_score	5.458473e−11	0.4262220	1.448624e−06	POS
471	GIMAP7	Stromal_score	3.157642e−19	0.5588989	8.380066e−15	POS	MYO9B	Stromal_score	3.574579e−14	0.4844975	9.486576e−10	POS
472	FGFR1	Stromal_score	2.281347e−25	0.6296592	6.054467e−21	POS	RPUSD1	Stromal_score	1.182603e−08	−0.3750361	3.138510e−04	NEG
473	HSPA2	Stromal_score	6.731301e−13	0.4624718	1.786420e−08	POS	AK128593	Stromal_score	8.949910e−07	−0.3262574	2.375217e−02	NEG
474	VWF	Stromal_score	1.258795e−19	0.5640646	3.340716e−15	POS	ZNF57	Stromal_score	6.906378e−15	−0.4961763	1.832884e−10	NEG
475	SSPN	Stromal_score	3.827060e−20	0.5706212	1.015663e−15	POS	NISCH	Stromal_score	4.978601e−14	0.4820893	1.321271e−09	POS
476	GGT5	Stromal_score	1.394569e−32	0.6945608	3.701048e−28	POS	LOC643733	Stromal_score	5.456763e−11	0.4262248	1.448170e−06	POS
477	FPR3	Stromal_score	2.717547e−11	0.4322679	7.212097e−07	POS	PLXDC1	Stromal_score	4.686863e−13	0.4652749	1.243847e−08	POS
478	TSC22D3	Stromal_score	4.604735e−13	0.4654111	1.222051e−08	POS	MFSD9	Stromal_score	1.217664e−09	−0.3977431	3.231560e−05	NEG
479	PCOLCE	Stromal_score	3.764585e−23	0.6060791	9.990831e−19	POS	NOTCH4	Stromal_score	1.063397e−09	0.3990432	2.822148e−05	POS
480	CFI	Stromal_score	3.773020e−07	0.3366810	1.001322e−02	POS	MTBP	Stromal_score	3.724201e−16	−0.5158830	9.883658e−12	NEG

	gene	feature	p.value	estimate	adj.p.value	COR.ID	gene	feature	p.value	estimate	adj.p.value	COR.ID
481	PDLIM7	Stromal_score	7.106104e−18	0.5407135	1.885889e−13	POS	RBM43	Stromal_score	1.033183e−07	0.3516285	2.741965e−03	POS
482	CXCR5	Stromal_score	2.772807e−07	0.3403071	7.358751e−03	POS	BC067230	Stromal_score	1.676693e−06	0.3184310	4.449777e−02	POS
483	JA202352	Stromal_score	4.286425e−08	0.3613567	1.137574e−03	POS	CENPQ	Stromal_score	4.033100e−11	−0.4288602	1.070344e−06	NEG
484	B3GNT3	Stromal_score	5.982099e−11	−0.4254190	1.587589e−06	NEG	MIR142	Stromal_score	1.438729e−06	0.3203596	3.818243e−02	POS
485	KLF9	Stromal_score	1.950564e−20	0.5742689	5.176601e−16	POS	RRP12	Stromal_score	7.574241e−08	−0.3550990	2.010128e−03	NEG
486	KCNK5	Stromal_score	1.682546e−06	−0.3183870	4.465308e−02	NEG	YPEL5	Stromal_score	4.089568e−10	0.4080587	1.085331e−05	POS
487	CSF1R	Stromal_score	1.171621e−20	0.5769979	3.109365e−16	POS	MRPL48	Stromal_score	6.754556e−09	−0.3807932	1.792592e−04	NEG
488	C4orf19	Stromal_score	5.200659e−09	−0.3834427	1.380203e−04	NEG	STIP1	Stromal_score	1.220845e−11	−0.4390603	3.240000e−07	NEG
489	CLDN5	Stromal_score	2.427122e−18	0.5471131	6.441339e−14	POS	LOC100287216	Stromal_score	1.248273e−10	0.4188925	3.312793e−06	POS
490	PRRX1	Stromal_score	2.377431e−27	0.6491508	6.309463e−23	POS	TADA2A	Stromal_score	1.031829e−06	−0.3245028	2.738371e−02	NEG
491	TJP3	Stromal_score	1.721700e−07	−0.3458279	4.569220e−03	NEG	C6orf136	Stromal_score	4.530385e−11	−0.4278493	1.202319e−06	NEG
492	PALM	Stromal_score	1.753617e−18	0.5490228	4.653925e−14	POS	HIVEP2	Stromal_score	2.187735e−10	0.4138181	5.806031e−06	POS
493	BEX4	Stromal_score	2.921388e−23	0.6072981	7.753071e−19	POS	HIST1H3B	Stromal_score	4.135737e−09	−0.3857455	1.097583e−04	NEG
494	ADAMTS2	Stromal_score	2.483573e−13	0.4701313	6.591155e−09	POS	CCDC102A	Stromal_score	3.022557e−11	0.4313535	8.021565e−07	POS
495	FSTL3	Stromal_score	1.690982e−10	0.4161579	4.487696e−06	POS	CTPS2	Stromal_score	1.326564e−07	−0.3488040	3.520568e−03	NEG
496	CD4	Stromal_score	6.638023e−13	0.4625804	1.761665e−08	POS	RAB12	Stromal_score	3.946852e−09	0.3862131	1.047455e−04	POS
497	FXYD6−FXYD2	Stromal_score	2.899196e−17	0.5321262	7.694176e−13	POS	AVL9	Stromal_score	4.264484e−09	−0.3854384	1.131751e−04	NEG
498	ALDH1B1	Stromal_score	1.573979e−06	0.3192292	4.177183e−02	POS	GPRC5D	Stromal_score	2.498634e−08	−0.3671678	6.631125e−04	NEG
499	ARHGEF25	Stromal_score	2.725178e−24	0.6184532	7.232350e−20	POS	RILPL1	Stromal_score	3.209922e−09	0.3882719	8.518813e−05	POS
500	RHOH	Stromal_score	6.317204e−08	0.3571085	1.676523e−03	POS	LRRC8B	Stromal_score	4.566498e−12	−0.4472065	1.211903e−07	NEG
501	CTSG	Stromal_score	2.817203e−12	0.4511297	7.476574e−08	POS	PUS1	Stromal_score	4.128841e−09	−0.3857622	1.095753e−04	NEG
502	AK123947	Stromal_score	4.567428e−26	0.6366893	1.212150e−21	POS	RAD9A	Stromal_score	4.698839e−08	−0.3603561	1.247025e−03	NEG
503	COLEC12	Stromal_score	2.646104e−26	0.6390346	7.022495e−22	POS	GPIHBP1	Stromal_score	3.678068e−10	0.4090428	9.761225e−06	POS
504	GXYLT2	Stromal_score	1.401344e−24	0.6215027	3.719027e−20	POS	NFIL3	Stromal_score	5.847921e−07	0.3314410	1.551980e−02	POS
505	LY86	Stromal_score	1.953384e−15	0.5048550	5.184085e−11	POS	SLC22A5	Stromal_score	1.200104e−07	−0.3499395	3.184957e−03	NEG
506	AX747977	Stromal_score	1.069969e−08	0.3760732	2.839591e−04	POS	DQ583960	Stromal_score	1.340810e−06	−0.3212433	3.558375e−02	NEG
507	GUCY1A3	Stromal_score	2.639887e−32	0.6923498	7.005997e−28	POS	TMEM33	Stromal_score	4.349863e−08	−0.3611969	1.154410e−03	NEG
508	SCARA5	Stromal_score	8.962591e−11	0.4218492	2.378582e−06	POS	GTPBP4	Stromal_score	1.496206e−06	−0.3198673	3.970782e−02	NEG
509	AK097351	Stromal_score	1.266667e−10	0.4187613	3.361608e−06	POS	TARBP1	Stromal_score	2.900540e−07	−0.3397798	7.697743e−03	NEG
510	TGFBR3	Stromal_score	1.076847e−09	0.3989228	2.857845e−05	POS	HIST1H4I	Stromal_score	3.510179e−07	−0.3375352	9.315663e−03	NEG
511	KRTCAP3	Stromal_score	8.712719e−07	−0.3265874	2.312268e−02	NEG	FAM133B	Stromal_score	1.060827e−07	−0.3513314	2.815330e−03	NEG
512	NEGR1	Stromal_score	3.429741e−19	0.5584302	9.102191e−15	POS	C3orf58	Stromal_score	1.056364e−07	0.3513789	2.803484e−03	POS
513	COL6A1	Stromal_score	1.491687e−19	0.5631179	3.958789e−15	POS	FBLN7	Stromal_score	8.363907e−13	0.4607780	2.219697e−08	POS
514	PHLDA3	Stromal_score	4.116094e−10	0.4079986	1.092370e−05	POS	ITGB1BP2	Stromal_score	2.305703e−07	0.3424569	6.119106e−03	POS
515	LTBP4	Stromal_score	2.954916e−15	0.5020376	7.842051e−11	POS	BRCC3	Stromal_score	1.736402e−09	−0.3943102	4.608238e−05	NEG
516	KLF5	Stromal_score	4.932463e−08	−0.3598261	1.309026e−03	NEG	AARD	Stromal_score	2.870712e−09	0.3893786	7.618584e−05	POS
517	LOC113230	Stromal_score	7.174269e−09	−0.3801789	1.903979e−04	NEG	CHST14	Stromal_score	7.589065e−13	0.4615375	2.014062e−08	POS
518	FNBP1	Stromal_score	1.093110e−23	0.6119747	2.901005e−19	POS	AFF1	Stromal_score	6.065840e−10	0.4043720	1.609813e−05	POS
519	TOX	Stromal_score	2.441542e−08	0.3674141	6.479609e−04	POS	MIR939	Stromal_score	8.276244e−09	−0.3787177	2.196433e−04	NEG
520	PLS1	Stromal_score	4.430830e−07	−0.3347705	1.175898e−02	NEG	ELOVL4	Stromal_score	9.737498e−15	0.4937723	2.584235e−10	POS
521	OLFML3	Stromal_score	4.954561e−27	0.6461083	1.314891e−22	POS	OCLN	Stromal_score	6.879903e−08	−0.3561654	1.825857e−03	NEG
522	APBB1IP	Stromal_score	5.392759e−07	0.3324173	1.431184e−02	POS	FAM171B	Stromal_score	1.008222e−08	0.3766874	2.675720e−04	POS
523	JA202363	Stromal_score	5.026652e−11	0.4269427	1.334023e−06	POS	C4orf46	Stromal_score	1.959818e−07	−0.3443377	5.201161e−03	NEG
524	STON1−GTF2A1L	Stromal_score	5.832138e−26	0.6356325	1.547791e−21	POS	HDAC7	Stromal_score	8.715744e−12	0.4418764	2.313071e−07	POS
525	WAS	Stromal_score	7.433006e−13	0.4616995	1.972645e−08	POS	C3orf26	Stromal_score	1.636295e−12	−0.4554838	4.342564e−08	NEG
526	NBLA00301	Stromal_score	5.639062e−14	0.4811789	1.496551e−09	POS	ADAM23	Stromal_score	4.045686e−23	0.6057320	1.073685e−18	POS
527	PBX1	Stromal_score	5.198238e−09	0.3834474	1.379560e−04	POS	ANKRD18A	Stromal_score	2.332863e−07	−0.3423209	6.191186e−03	NEG
528	MS4A6A	Stromal_score	1.988614e−17	0.5344527	5.277582e−13	POS	CACYBP	Stromal_score	8.526376e−12	−0.4420590	2.262815e−07	NEG
529	LOC572558	Stromal_score	8.852731e−13	0.4603334	2.349426e−08	POS	WDR12	Stromal_score	1.445571e−14	−0.4909839	3.836401e−10	NEG
530	EFNA3	Stromal_score	8.183187e−08	−0.3542385	2.171736e−03	NEG	GET4	Stromal_score	1.374547e−06	−0.3209321	3.647910e−02	NEG
531	HCLS1	Stromal_score	4.145627e−15	0.4997134	1.100208e−10	POS	KIAA0430	Stromal_score	8.031789e−10	0.4017190	2.131556e−05	POS
532	THY1	Stromal_score	3.311536e−25	0.6280043	8.788485e−21	POS	ACTR3C	Stromal_score	5.947421e−07	−0.3312373	1.578386e−02	NEG
533	GFRA1	Stromal_score	3.984212e−13	0.4665243	1.057370e−08	POS	PRAM1	Stromal_score	4.603909e−07	0.3343131	1.221831e−02	POS
534	FSTL1	Stromal_score	6.585182e−38	0.7334752	1.747642e−33	POS	DOT1L	Stromal_score	4.512126e−07	−0.3345536	1.197473e−02	NEG
535	PCP4L1	Stromal_score	1.103945e−08	0.3757497	2.929759e−04	POS	IPW	Stromal_score	5.915605e−12	0.4450831	1.569942e−07	POS
536	PDE3A	Stromal_score	1.586402e−21	0.5874630	4.210151e−17	POS	ATG4D	Stromal_score	6.896053e−11	−0.4241683	1.830143e−06	NEG
537	TPX2	Stromal_score	3.903720e−14	−0.4838591	1.036008e−09	NEG	SNX18	Stromal_score	1.074915e−11	0.4401271	2.852718e−07	POS
538	CRISPLD2	Stromal_score	6.092922e−31	0.6811760	1.617001e−26	POS	NARS2	Stromal_score	1.021295e−13	−0.4768006	2.710415e−09	NEG
539	RGS4	Stromal_score	4.464027e−11	0.4279778	1.184708e−06	POS	ST3GAL3	Stromal_score	4.705924e−08	0.3603396	1.248905e−03	POS
540	FZD7	Stromal_score	1.103429e−09	0.3986891	2.928389e−05	POS	MAGI2−AS3	Stromal_score	9.280608e−35	0.7112574	2.462980e−30	POS
541	GPR183	Stromal_score	7.669640e−19	0.5538276	2.035446e−14	POS	PPP2R3B	Stromal_score	4.038128e−08	−0.3620049	1.071679e−03	NEG
542	PBK	Stromal_score	1.924256e−08	−0.3699393	5.106782e−04	NEG	DPEP2	Stromal_score	1.761788e−08	0.3708694	4.675610e−04	POS
543	WLS	Stromal_score	9.604786e−09	0.3771877	2.549014e−04	POS	FAM72B	Stromal_score	1.286772e−15	−0.5076704	3.414964e−11	NEG
544	COX7A1	Stromal_score	2.133391e−18	0.5478725	5.661807e−14	POS	POP1	Stromal_score	1.291100e−10	−0.4185898	3.426450e−06	NEG
545	RARRES2	Stromal_score	1.165870e−22	0.6005805	3.094102e−18	POS	PHF16	Stromal_score	2.394818e−08	−0.3676198	6.355608e−04	NEG
546	IL10RA	Stromal_score	1.753674e−14	0.4896111	4.654075e−10	POS	AK056084	Stromal_score	5.845147e−08	−0.3579642	1.551244e−03	NEG
547	BX649128	Stromal_score	4.040351e−17	0.5300630	1.072269e−12	POS	RSPO1	Stromal_score	7.564921e−18	0.5403365	2.007654e−13	POS
548	SLCO2B1	Stromal_score	5.828719e−10	0.4047470	1.546884e−05	POS	EIF3B	Stromal_score	1.766397e−06	−0.3177712	4.687840e−02	NEG
549	LSP1	Stromal_score	3.660258e−19	0.5580608	9.713960e−15	POS	CREBL2	Stromal_score	1.159619e−13	0.4758561	3.077512e−09	POS
550	ABCA8	Stromal_score	3.382260e−16	0.5165117	8.976179e−12	POS	PBX4	Stromal_score	1.367942e−06	−0.3209924	3.630381e−02	NEG
551	PLD4	Stromal_score	9.047064e−10	0.4005870	2.401000e−05	POS	MRRF	Stromal_score	1.179762e−07	−0.3501328	3.130971e−03	NEG
552	RHPN2	Stromal_score	2.198039e−08	−0.3685311	5.833376e−04	NEG	FAM206A	Stromal_score	1.727711e−09	−0.3943590	4.585173e−05	NEG
553	IFI16	Stromal_score	1.316796e−12	0.4572075	3.494645e−08	POS	YARS	Stromal_score	9.620918e−07	−0.3253672	2.553295e−02	NEG
554	C14orf132	Stromal_score	4.696721e−20	0.5695039	1.246463e−15	POS	CCT6A	Stromal_score	9.606602e−11	−0.4212323	2.549496e−06	NEG
555	CYS1	Stromal_score	2.186496e−12	0.4531685	5.802743e−08	POS	NUCB1	Stromal_score	7.113534e−08	0.3557955	1.887861e−03	POS
556	AMOTL1	Stromal_score	2.786816e−16	0.5177721	7.395930e−12	POS	PTPN21	Stromal_score	7.472419e−09	0.3797633	1.983105e−04	POS
557	NCKAP1L	Stromal_score	8.132673e−13	0.4609972	2.158330e−08	POS	CALU	Stromal_score	5.679223e−14	0.4811269	1.507209e−09	POS
558	S1PR1	Stromal_score	1.176093e−23	0.6116293	3.121234e−19	POS	SPATA5L1	Stromal_score	1.251134e−07	−0.3494680	3.320385e−03	NEG
559	NPTX1	Stromal_score	6.266142e−07	0.3306061	1.662972e−02	POS	MRPS17	Stromal_score	4.628062e−07	−0.3342506	1.228241e−02	NEG
560	ANTXR2	Stromal_score	9.376243e−10	0.4002463	2.488361e−05	POS	BNIP2	Stromal_score	8.100394e−15	0.4950627	2.149764e−10	POS
561	ADAMTS8	Stromal_score	1.826277e−14	0.4893221	4.846758e−10	POS	MASTL	Stromal_score	4.089068e−09	−0.3858591	1.085198e−04	NEG
562	SGCE	Stromal_score	2.502542e−14	0.4870684	6.641497e−10	POS	FAM69A	Stromal_score	2.064999e−12	0.4536265	5.480302e−08	POS
563	ENPP2	Stromal_score	1.597726e−10	0.4166708	4.240206e−06	POS	CXorf21	Stromal_score	1.612419e−08	0.3718006	4.279200e−04	POS
564	GNG11	Stromal_score	1.785858e−26	0.6407115	4.739489e−22	POS	NEIL3	Stromal_score	5.972551e−10	−0.4045178	1.585055e−05	NEG
565	RAMP2	Stromal_score	2.344029e−20	0.5732789	6.220818e−16	POS	TBXA2R	Stromal_score	6.810613e−13	0.4623807	1.807469e−08	POS
566	CHRNA3	Stromal_score	6.786947e−08	0.3563159	1.801188e−03	POS	SLC16A7	Stromal_score	1.062342e−06	0.3241420	2.819349e−02	POS
567	ROR2	Stromal_score	1.701964e−24	0.6206151	4.516842e−20	POS	CHSY1	Stromal_score	5.188885e−10	0.4058377	1.377078e−05	POS
568	CSF2RB	Stromal_score	1.617010e−15	0.5061329	4.291382e−11	POS	C16orf55	Stromal_score	5.195745e−13	−0.4644793	1.378899e−08	NEG
569	IGFBP7	Stromal_score	1.287504e−25	0.6321803	3.416908e−21	POS	CSGALNACT2	Stromal_score	4.156405e−22	0.5942678	1.103068e−17	POS
570	AHNAK	Stromal_score	7.494996e−17	0.5261842	1.989097e−12	POS	LRR1	Stromal_score	5.090445e−09	−0.3836588	1.350953e−04	NEG
571	PRKCDBP	Stromal_score	2.680277e−10	0.4119606	7.113188e−06	POS	CNPY4	Stromal_score	6.227712e−09	0.3816188	1.652773e−04	POS
572	RASSF2	Stromal_score	2.977834e−24	0.6180441	7.902874e−20	POS	SNORD4A	Stromal_score	2.741886e−10	−0.4117520	7.276691e−06	NEG
573	ID4	Stromal_score	9.801459e−15	0.4937263	2.601209e−10	POS	TET3	Stromal_score	1.537170e−07	−0.3471258	4.079496e−03	NEG
574	PTPRS	Stromal_score	2.659534e−09	0.3901334	7.058137e−05	POS	SNRPA1	Stromal_score	1.926165e−12	−0.4541832	5.111848e−08	NEG
575	SMAD9	Stromal_score	1.582124e−11	0.4368763	4.198798e−07	POS	VKORC1	Stromal_score	8.143798e−07	0.3274154	2.161282e−02	POS
576	SEMA3A	Stromal_score	5.064890e−08	0.3595363	1.344171e−03	POS	43347	Stromal_score	4.471100e−16	0.5146861	1.186585e−11	POS
577	CPZ	Stromal_score	2.231478e−18	0.5476081	5.922120e−14	POS	RCBTB2	Stromal_score	4.442274e−16	0.5147285	1.178935e−11	POS
578	LOC255130	Stromal_score	2.961727e−29	0.6666267	7.860127e−25	POS	ISCU	Stromal_score	2.536114e−10	0.4124675	6.730593e−06	POS
579	PDLIM4	Stromal_score	2.304936e−13	0.4706971	6.117069e−09	POS	NMUR1	Stromal_score	4.879347e−08	0.3599444	1.294930e−03	POS
580	JAM3	Stromal_score	1.251606e−22	0.6002317	3.321638e−18	POS	GRPEL1	Stromal_score	9.607420e−10	−0.4000139	2.549713e−05	NEG
581	COL4A2	Stromal_score	8.238427e−20	0.5664158	2.186396e−15	POS	AK055370	Stromal_score	2.070823e−15	0.5044592	5.495756e−11	POS
582	TGFB3	Stromal_score	2.274002e−25	0.6296734	6.034973e−21	POS	C1QTNF2	Stromal_score	8.534840e−18	0.5396084	2.265061e−13	POS
583	CADM3	Stromal_score	9.224847e−19	0.5527616	2.448182e−14	POS	ITGB3BP	Stromal_score	4.840813e−10	−0.4064871	1.284703e−05	NEG
584	DIO2	Stromal_score	7.996239e−15	0.4951532	2.122122e−10	POS	DENND2A	Stromal_score	6.477334e−09	0.3812196	1.719020e−04	POS
585	PLTP	Stromal_score	1.156809e−11	0.4395122	3.070055e−07	POS	STAT3	Stromal_score	6.909357e−14	0.4796883	1.833674e−09	POS
586	FILIP1L	Stromal_score	1.842459e−20	0.5745754	4.889702e−16	POS	DSEL	Stromal_score	1.727893e−21	0.5870235	4.585655e−17	POS
587	GPT2	Stromal_score	1.503942e−11	−0.4373045	3.991313e−07	NEG	BICD2	Stromal_score	3.412569e−09	0.3876636	9.056617e−05	POS
588	ANXA6	Stromal_score	1.291569e−21	0.5885181	3.427695e−17	POS	JA611288	Stromal_score	7.749793e−13	0.4613739	2.056718e−08	POS
589	DSP	Stromal_score	1.553287e−07	−0.3470067	4.122269e−03	NEG	ACTR3B	Stromal_score	1.471961e−13	−0.4740748	3.906438e−09	NEG
590	VCAM1	Stromal_score	5.006662e−22	0.5933318	1.328718e−17	POS	SLC25A39	Stromal_score	4.467950e−14	−0.4828780	1.185749e−09	NEG
591	CH25H	Stromal_score	1.683448e−14	0.4899020	4.467704e−10	POS	FKBPL	Stromal_score	3.810776e−07	−0.3365630	1.011342e−02	NEG
592	IKZF1	Stromal_score	4.798968e−10	0.4065682	1.273598e−05	POS	CCT8	Stromal_score	2.797121e−07	−0.3402049	7.423280e−03	NEG
593	ITGA9	Stromal_score	9.677356e−16	0.5095774	2.568273e−11	POS	C1orf182	Stromal_score	1.306882e−14	−0.4916981	3.468334e−10	NEG
594	SLIT2	Stromal_score	1.252858e−24	0.6220130	3.324959e−20	POS	FLT4	Stromal_score	3.298813e−10	0.4100493	8.754719e−06	POS
595	EML1	Stromal_score	1.574506e−20	0.5754187	4.178581e−16	POS	SASS6	Stromal_score	9.898040e−17	−0.5244221	2.626841e−12	NEG
596	TIMP2	Stromal_score	7.098520e−28	0.6540846	1.883876e−23	POS	CREB5	Stromal_score	1.392663e−06	0.3207680	3.695988e−02	POS
597	LTBP2	Stromal_score	3.435343e−19	0.5584209	9.117058e−15	POS	AK7	Stromal_score	5.766129e−10	−0.4048484	1.530273e−05	NEG
598	BCHE	Stromal_score	1.523482e−16	0.5216693	4.043168e−12	POS	FAM173B	Stromal_score	1.215732e−06	−0.3224663	3.226431e−02	NEG
599	RAB23	Stromal_score	3.784643e−17	0.5304705	1.004406e−12	POS	PPP1R37	Stromal_score	1.847373e−06	−0.3172026	4.902742e−02	NEG
600	TNFSF13B	Stromal_score	3.524786e−08	0.3634765	9.354430e−04	POS	MCAT	Stromal_score	8.572919e−12	−0.4420138	2.275167e−07	NEG

	gene	feature	p.value	estimate	adj.p.value	COR.ID	gene	feature	p.value	estimate	adj.p.value	COR.ID
601	RRM2	Stromal_score	2.033265e−08	−0.3693568	5.396083e−04	NEG	SNRK	Stromal_score	4.153347e−14	0.4834090	1.102257e−09	POS
602	ISM1	Stromal_score	3.772441e−20	0.5706994	1.001168e−15	POS	DSE	Stromal_score	8.626348e−15	0.4946223	2.289347e−10	POS
603	FOXM1	Stromal_score	5.501801e−09	−0.3828742	1.460123e−04	NEG	PDCD6	Stromal_score	2.420399e−10	−0.4128950	6.423496e−06	NEG
604	TMEM47	Stromal_score	2.163793e−23	0.6087345	5.742491e−19	POS	TNRC6C	Stromal_score	1.146948e−09	0.3983180	3.043886e−05	POS
605	CDT1	Stromal_score	1.228217e−11	−0.4390097	3.259564e−07	NEG	CKAP5	Stromal_score	1.316071e−06	−0.3214763	3.492722e−02	NEG
606	EPHA7	Stromal_score	3.393747e−10	0.4097873	9.006665e−06	POS	AQP11	Stromal_score	4.166805e−10	−0.4078847	1.105828e−05	NEG
607	CHST15	Stromal_score	1.261635e−14	0.4919472	3.348252e−10	POS	ALDH18A1	Stromal_score	7.175095e−09	−0.3801778	1.904198e−04	NEG
608	ALOX5AP	Stromal_score	4.728399e−11	0.4274765	1.254870e−06	POS	LOC100288748	Stromal_score	1.270302e−11	−0.4387267	3.371254e−07	NEG
609	AK074396	Stromal_score	3.758976e−08	0.3627812	9.975947e−04	POS	TDG	Stromal_score	9.261932e−07	−0.3258358	2.458024e−02	NEG
610	RCN3	Stromal_score	7.147206e−12	0.4435223	1.896797e−07	POS	COX19	Stromal_score	1.248556e−10	−0.4188904	3.313543e−06	NEG
611	P2RY10	Stromal_score	1.465414e−06	0.3201288	3.889063e−02	POS	AK027179	Stromal_score	6.127585e−09	0.3817833	1.626200e−04	POS
612	SH3BGRL	Stromal_score	5.171261e−18	0.5426212	1.372401e−13	POS	B3GNT4	Stromal_score	2.916887e−08	−0.3655131	7.741125e−04	NEG
613	ZEB1	Stromal_score	1.301007e−27	0.6516231	3.452743e−23	POS	PARD3B	Stromal_score	9.380747e−09	0.3774308	2.489556e−04	POS
614	CD248	Stromal_score	1.386090e−21	0.5881561	3.678545e−17	POS	C17orf58	Stromal_score	1.487086e−08	−0.3726485	3.946577e−04	NEG
615	GPRC5C	Stromal_score	1.630025e−10	−0.4164899	4.325922e−06	NEG	OSTM1	Stromal_score	4.463686e−24	0.6161689	1.184618e−19	POS
616	VCAN	Stromal_score	1.556118e−21	0.5875621	4.129782e−17	POS	P4HTM	Stromal_score	4.583241e−07	−0.3343668	1.216346e−02	NEG
617	REEP2	Stromal_score	6.261386e−08	0.3572064	1.661709e−03	POS	PPP3CB	Stromal_score	9.632230e−07	0.3253527	2.556297e−02	POS
618	HDGFRP3	Stromal_score	2.591558e−18	0.5467265	6.877737e−14	POS	C1orf96	Stromal_score	1.068265e−06	−0.3240732	2.835068e−02	NEG
619	MKI67	Stromal_score	3.140339e−08	−0.3647207	8.334146e−04	NEG	TCP1	Stromal_score	6.244774e−07	−0.3306474	1.657301e−02	NEG
620	AURKA	Stromal_score	1.046648e−14	−0.4932647	2.777700e−10	NEG	RAD18	Stromal_score	4.806974e−12	−0.4467866	1.275723e−07	NEG
621	CXADR	Stromal_score	1.876413e−12	−0.4543922	4.979811e−08	NEG	PRDM6	Stromal_score	7.745460e−17	0.5259765	2.055568e−12	POS
622	HOXB2	Stromal_score	1.420713e−10	0.4177298	3.770430e−06	POS	NLN	Stromal_score	2.891160e−10	−0.4112649	7.672850e−06	NEG
623	MRC2	Stromal_score	3.523546e−29	0.6659563	9.351138e−25	POS	PGAP2	Stromal_score	8.963299e−12	−0.4416433	2.378770e−07	NEG
624	AK091265	Stromal_score	4.315481e−07	−0.3350850	1.145286e−02	NEG	FAM103A1	Stromal_score	8.382336e−11	−0.4224431	2.224588e−06	NEG
625	NRP2	Stromal_score	5.651229e−20	0.5684908	1.499780e−15	POS	SUV39H2	Stromal_score	2.313253e−12	−0.4527163	6.139141e−08	NEG
626	ADAM33	Stromal_score	2.631781e−23	0.6077985	6.984484e−19	POS	CYB5R3	Stromal_score	1.480347e−13	0.4740322	3.928692e−09	POS
627	NOV	Stromal_score	3.242642e−08	0.3643758	8.605647e−04	POS	MUTYH	Stromal_score	7.620700e−11	−0.4232863	2.022458e−06	NEG
628	LRP1	Stromal_score	1.465915e−15	0.5067941	3.890392e−11	POS	KCNN3	Stromal_score	4.187852e−19	0.5572947	1.111414e−14	POS
629	SOCS3	Stromal_score	3.611781e−08	0.3632131	9.585307e−04	POS	IGDCC4	Stromal_score	1.433662e−08	0.3730311	3.804795e−04	POS
630	GSN	Stromal_score	1.289861e−19	0.5639288	3.423162e−15	POS	FBXL5	Stromal_score	2.435662e−13	0.4702791	6.464004e−09	POS
631	SDC1	Stromal_score	1.741398e−06	−0.3179518	4.621495e−02	NEG	unknown	Stromal_score	1.378405e−10	0.4180018	3.658150e−06	POS
632	NPTXR	Stromal_score	3.348712e−07	0.3380909	8.887146e−03	POS	PKNOX2	Stromal_score	1.958134e−21	0.5863788	5.196692e−17	POS
633	ATP1B1	Stromal_score	5.983704e−07	−0.3311638	1.588015e−02	NEG	GAN	Stromal_score	6.824955e−07	−0.3295701	1.811275e−02	NEG
634	APOBEC3C	Stromal_score	5.627809e−08	0.3583808	1.493564e−03	POS	APCDD1L	Stromal_score	1.974195e−08	0.3696686	5.239315e−04	POS
635	FLRT2	Stromal_score	8.619392e−26	0.6339351	2.287500e−21	POS	TIMM8A	Stromal_score	3.373888e−14	−0.4849156	8.953962e−10	NEG
636	GJA1	Stromal_score	1.980424e−18	0.5483096	5.255848e−14	POS	ZNF789	Stromal_score	9.097684e−08	−0.3530555	2.414434e−03	NEG
637	TSPYL5	Stromal_score	3.536035e−07	0.3374485	9.384283e−03	POS	COX6A1	Stromal_score	5.683165e−08	−0.3582732	1.508255e−03	NEG
638	TSPAN13	Stromal_score	1.585294e−07	−0.3467735	4.207211e−03	NEG	LAPTM4A	Stromal_score	1.223619e−09	0.3976961	3.247362e−05	POS
639	ARHGDIB	Stromal_score	9.419120e−15	0.4940057	2.499740e−10	POS	COG3	Stromal_score	2.480295e−08	−0.3672463	6.582455e−04	NEG
640	FMO2	Stromal_score	4.368729e−12	0.4475682	1.159417e−07	POS	CRLF2	Stromal_score	4.669453e−08	0.3604245	1.239226e−03	POS
641	MOXD1	Stromal_score	9.607295e−29	0.6620507	2.549680e−24	POS	TFAP4	Stromal_score	1.448033e−08	−0.3729268	3.842934e−04	NEG
642	HTRA1	Stromal_score	4.187351e−18	0.5438813	1.111281e−13	POS	IGIP	Stromal_score	6.256395e−10	0.4040808	1.660385e−05	POS
643	PER1	Stromal_score	5.167094e−13	0.4645221	1.371295e−08	POS	STARD8	Stromal_score	1.164702e−16	0.5233864	3.091003e−12	POS
644	COL4A1	Stromal_score	2.084850e−13	0.4714562	5.532982e−09	POS	WASF2	Stromal_score	1.140072e−11	0.4396344	3.025638e−07	POS
645	TMC4	Stromal_score	1.138669e−07	−0.3505332	3.021913e−03	NEG	FBXO22−AS1	Stromal_score	6.871140e−08	−0.3561795	1.823532e−03	NEG
646	IL1R1	Stromal_score	3.150816e−28	0.6573490	8.361950e−24	POS	LOC100130015	Stromal_score	2.068157e−07	−0.3437165	5.488683e−03	NEG
647	GIMAP4	Stromal_score	2.731707e−20	0.5724517	7.249678e−16	POS	LOC100133957	Stromal_score	1.033397e−06	−0.3244840	2.742533e−02	NEG
648	SOX9	Stromal_score	5.195300e−11	−0.4266543	1.378781e−06	NEG	TSSC1	Stromal_score	8.222234e−12	−0.4423608	2.182099e−07	NEG
649	TIMP1	Stromal_score	2.456391e−08	0.3673495	6.519017e−04	POS	RCE1	Stromal_score	1.964194e−11	−0.4350419	5.212774e−07	NEG
650	KCNK3	Stromal_score	2.737878e−09	0.3898468	7.266056e−05	POS	CLNS1A	Stromal_score	8.286727e−07	−0.3272023	2.199214e−02	NEG
651	GAL3ST1	Stromal_score	1.911703e−07	−0.3446243	5.073467e−03	NEG	OLA1	Stromal_score	5.232777e−11	−0.4265915	1.388727e−06	NEG
652	LOC100499467	Stromal_score	3.216610e−14	−0.4852606	8.536561e−10	NEG	C1QTNF4	Stromal_score	3.463597e−14	0.4847258	9.192039e−10	POS
653	NRXN3	Stromal_score	1.741072e−11	0.4360658	4.620631e−07	POS	ROCK1	Stromal_score	2.570092e−07	0.3411938	6.820767e−03	POS
654	MS4A7	Stromal_score	2.180255e−18	0.5477447	5.786178e−14	POS	C5orf28	Stromal_score	1.909965e−09	−0.3933819	5.068857e−05	NEG
655	FPR1	Stromal_score	7.945191e−10	0.4018219	2.108574e−05	POS	PIGM	Stromal_score	2.354489e−08	−0.3678005	6.248578e−04	NEG
656	HSPG2	Stromal_score	1.334084e−18	0.5506200	3.540526e−14	POS	ADPRH	Stromal_score	5.723325e−15	0.4974832	1.518913e−10	POS
657	AP1M2	Stromal_score	6.344141e−15	−0.4967676	1.683672e−10	NEG	BC033983	Stromal_score	8.638148e−11	−0.4221765	2.292478e−06	NEG
658	PLEKHA4	Stromal_score	3.022155e−09	0.3888697	8.020497e−05	POS	DPH2	Stromal_score	6.996012e−13	−0.4621717	1.856672e−08	NEG
659	RHOB	Stromal_score	2.261551e−11	0.4338404	6.001931e−07	POS	LEPROT	Stromal_score	1.627867e−14	0.4901408	4.320195e−10	POS
660	COL5A2	Stromal_score	2.159590e−16	0.5194237	5.731337e−12	POS	C12orf66	Stromal_score	3.213940e−07	−0.3385748	8.529475e−03	NEG
661	FAM83H	Stromal_score	3.300013e−08	−0.3641870	8.757906e−04	NEG	TARS2	Stromal_score	2.802127e−10	−0.4115524	7.436564e−06	NEG
662	RAB25	Stromal_score	5.411511e−12	−0.4458154	1.436161e−07	NEG	NUP37	Stromal_score	7.071575e−10	−0.4029251	1.876725e−05	NEG
663	ANTXR1	Stromal_score	5.245538e−23	0.6044764	1.392113e−18	POS	POMC	Stromal_score	3.161746e−07	0.3387674	8.390957e−03	POS
664	BC078172	Stromal_score	6.513452e−31	0.6809329	1.728605e−26	POS	SNORD42B	Stromal_score	5.078811e−09	−0.3836818	1.347866e−04	NEG
665	LOC144571	Stromal_score	7.500516e−16	0.5112727	1.990562e−11	POS	PTGDR2	Stromal_score	1.383260e−13	−0.4745400	3.671034e−09	NEG
666	PRSS23	Stromal_score	2.934290e−23	0.6072770	7.787313e−19	POS	GEN1	Stromal_score	6.777449e−13	−0.4624187	1.798667e−08	NEG
667	KIAA1644	Stromal_score	1.347086e−09	0.3967700	3.575032e−05	POS	ABCB9	Stromal_score	9.547256e−08	−0.3525153	2.533746e−03	NEG
668	PROS1	Stromal_score	1.344883e−11	0.4382469	3.569186e−07	POS	TLR6	Stromal_score	1.458170e−07	0.3477278	3.869838e−03	POS
669	DIXDC1	Stromal_score	5.710749e−15	0.4974985	1.515576e−10	POS	TGDS	Stromal_score	1.061428e−10	−0.4203432	2.816923e−06	NEG
670	PYGL	Stromal_score	5.486211e−08	0.3586606	1.455985e−03	POS	YRDC	Stromal_score	3.521307e−11	−0.4300358	9.345197e−07	NEG
671	PLXDC2	Stromal_score	1.469724e−34	0.7097738	3.900501e−30	POS	PPP2R1B	Stromal_score	3.988035e−07	−0.3360236	1.058385e−02	NEG
672	EFCAB4A	Stromal_score	7.410373e−10	−0.4024824	1.966639e−05	NEG	COASY	Stromal_score	2.290048e−07	−0.3425360	6.077558e−03	NEG
673	OSMR	Stromal_score	4.791769e−10	0.4065822	1.271688e−05	POS	PXMP4	Stromal_score	3.183630e−08	−0.3645734	8.449037e−04	NEG
674	CD14	Stromal_score	8.760423e−18	0.5394507	2.324929e−13	POS	SFMBT2	Stromal_score	3.421769e−13	0.4676903	9.081033e−09	POS
675	NXN	Stromal_score	2.832122e−11	0.4319132	7.516168e−07	POS	LRRC42	Stromal_score	1.499703e−06	−0.3198380	3.980061e−02	NEG
676	JA202350	Stromal_score	1.850279e−10	0.4153420	4.910456e−06	POS	HGF	Stromal_score	1.704429e−17	0.5353992	4.523383e−13	POS
677	GPR35	Stromal_score	8.905883e−09	−0.3779651	2.363532e−04	NEG	PEAK1	Stromal_score	8.035826e−15	0.4951187	2.132628e−10	POS
678	43344	Stromal_score	1.095647e−06	0.3237594	2.907737e−02	POS	FGF14	Stromal_score	1.492305e−11	0.4373701	3.960427e−07	POS
679	FAM127A	Stromal_score	6.901311e−13	0.4622778	1.831539e−08	POS	LOC100505666	Stromal_score	5.179423e−16	−0.5137197	1.374567e−11	NEG
680	PRICKLE2	Stromal_score	5.618476e−22	0.5927505	1.491087e−17	POS	RMND5A	Stromal_score	5.845547e−08	−0.3579634	1.551350e−03	NEG
681	MS4A4A	Stromal_score	2.220844e−14	0.4879247	5.893898e−10	POS	GSTCD	Stromal_score	8.438702e−09	−0.3785184	2.239547e−04	NEG
682	FCER1A	Stromal_score	4.035149e−10	0.4081832	1.070888e−05	POS	PTRH2	Stromal_score	2.249153e−09	−0.3917823	5.969026e−05	NEG
683	EMP3	Stromal_score	2.063488e−25	0.6301032	5.476291e−21	POS	EIF2AK1	Stromal_score	3.129151e−10	−0.4105365	8.304453e−06	NEG
684	CTSK	Stromal_score	1.977978e−24	0.6199267	5.249357e−20	POS	PIF1	Stromal_score	3.133850e−12	−0.4502688	8.316925e−08	NEG
685	AOX1	Stromal_score	4.317065e−14	0.4831280	1.145706e−09	POS	LOC100288842	Stromal_score	1.730931e−06	−0.3180282	4.593718e−02	NEG
686	CSRP2	Stromal_score	1.527734e−14	0.4905918	4.054453e−10	POS	NAP1L2	Stromal_score	2.920108e−16	0.5174685	7.749674e−12	POS
687	PRNP	Stromal_score	6.058207e−14	0.4806536	1.607788e−09	POS	SCARF1	Stromal_score	1.845209e−12	0.4545261	4.897000e−08	POS
688	BAIAP2L1	Stromal_score	5.508162e−09	−0.3828626	1.461811e−04	NEG	NDUFAF6	Stromal_score	3.497149e−12	−0.4493798	9.281084e−08	NEG
689	POU2F2	Stromal_score	3.884362e−13	0.4667190	1.030871e−08	POS	INADL	Stromal_score	2.814437e−09	−0.3895744	7.469234e−05	NEG
690	NEURL1B	Stromal_score	4.031388e−11	0.4288639	1.069890e−06	POS	C17orf100	Stromal_score	2.084893e−08	−0.3690913	5.533097e−04	NEG
691	LTBP3	Stromal_score	2.240342e−16	0.5191864	5.945643e−12	POS	SNORD50A	Stromal_score	7.195843e−09	−0.3801483	1.909705e−04	NEG
692	LYVE1	Stromal_score	3.194068e−14	0.4853114	8.476738e−10	POS	BC045163	Stromal_score	1.409548e−08	0.3732083	3.740800e−04	POS
693	LMO7	Stromal_score	2.913065e−07	−0.3397293	7.730983e−03	NEG	VARS2	Stromal_score	3.052387e−11	−0.4312689	8.100730e−07	NEG
694	CLIC4	Stromal_score	1.766498e−23	0.6097010	4.688108e−19	POS	NEK9	Stromal_score	8.955041e−07	0.3262504	2.376578e−02	POS
695	AXL	Stromal_score	6.005269e−34	0.7051728	1.593738e−29	POS	DAPK3	Stromal_score	5.326956e−08	0.3589838	1.413721e−03	POS
696	RCSD1	Stromal_score	1.546747e−17	0.5359935	4.104912e−13	POS	SIGLEC6	Stromal_score	5.701099e−09	0.3825143	1.513015e−04	POS
697	PDGFRB	Stromal_score	3.201179e−28	0.6572856	8.495610e−24	POS	DNM2	Stromal_score	4.292774e−07	−0.3351478	1.139259e−02	NEG
698	FOXF1−AS1	Stromal_score	4.168973e−11	0.4285724	1.106404e−06	POS	AAED1	Stromal_score	1.103265e−08	0.3757560	2.927956e−04	POS
699	OR7E14P	Stromal_score	1.113625e−08	−0.3756592	2.955450e−04	NEG	TUBG1	Stromal_score	1.346455e−07	−0.3486350	3.573356e−03	NEG
700	SLC22A17	Stromal_score	1.387631e−18	0.5503907	3.682634e−14	POS	LOC283481	Stromal_score	1.618097e−09	0.3949959	4.294268e−05	POS
701	VLDLR	Stromal_score	1.149327e−08	0.3753322	3.050200e−04	POS	WDR67	Stromal_score	9.858037e−14	−0.4770631	2.616225e−09	NEG
702	P2RY8	Stromal_score	3.063560e−11	0.4312375	8.130381e−07	POS	SLC35B1	Stromal_score	1.842766e−06	−0.3172343	4.890516e−02	NEG
703	RGMB	Stromal_score	6.010930e−09	0.3819782	1.595241e−04	POS	TOP1	Stromal_score	8.950191e−07	−0.3262570	2.375291e−02	NEG
704	ADAMTSL4	Stromal_score	3.138891e−13	0.4683495	8.330302e−09	POS	PNPLA3	Stromal_score	1.522527e−09	−0.3955863	4.040634e−05	NEG
705	THBD	Stromal_score	8.150862e−15	0.4950192	2.163157e−10	POS	STIM1	Stromal_score	2.990805e−07	0.3394204	7.937297e−03	POS
706	C1QTNF3	Stromal_score	4.507532e−13	0.4655754	1.196254e−08	POS	LRPPRC	Stromal_score	8.708086e−07	−0.3265939	2.311039e−02	NEG
707	LPAR1	Stromal_score	2.507471e−16	0.5184573	6.654577e−12	POS	XRCC6BP1	Stromal_score	1.828503e−06	−0.3173329	4.852664e−02	NEG
708	TMEM35	Stromal_score	1.425894e−12	0.4565770	3.784179e−08	POS	MAP3K12	Stromal_score	4.565297e−09	0.3847545	1.211584e−04	POS
709	EPR−1	Stromal_score	6.285160e−15	−0.4968326	1.668019e−10	NEG	C10orf12	Stromal_score	7.057974e−12	−0.4436263	1.873116e−07	NEG
710	MEOX2	Stromal_score	1.540541e−24	0.6210706	4.088441e−20	POS	C1orf131	Stromal_score	2.620691e−10	−0.4121668	6.955051e−06	NEG
711	EFEMP2	Stromal_score	1.211288e−36	0.7248110	3.214638e−32	POS	FAM162B	Stromal_score	6.204698e−09	0.3816564	1.646665e−04	POS
712	CD180	Stromal_score	2.908085e−09	0.3892507	7.717768e−05	POS	MED13L	Stromal_score	6.670759e−07	0.3298476	1.770353e−02	POS
713	LOC100652770	Stromal_score	1.737343e−09	−0.3943049	4.610734e−05	NEG	GTPBP3	Stromal_score	8.329318e−12	−0.4422534	2.210518e−07	NEG
714	ITGA11	Stromal_score	1.845222e−10	0.4153669	4.897034e−06	POS	POU6F1	Stromal_score	1.918738e−19	0.5617082	5.092138e−15	POS
715	RCOR2	Stromal_score	2.813680e−08	−0.3658991	7.467224e−04	NEG	SFMBT1	Stromal_score	7.580333e−10	−0.4022676	2.011744e−05	NEG
716	MYOF	Stromal_score	5.364654e−07	0.3324801	1.423725e−02	POS	MIR559	Stromal_score	4.420841e−08	−0.3610208	1.173247e−03	NEG
717	WIPF1	Stromal_score	5.280986e−26	0.6360622	1.401521e−21	POS	CLEC1A	Stromal_score	1.265789e−19	0.5640338	3.359277e−15	POS
718	COPZ2	Stromal_score	1.122610e−25	0.6327809	2.979296e−21	POS	DNAJC9	Stromal_score	4.268737e−11	−0.4283669	1.132880e−06	NEG
719	SPARC	Stromal_score	2.156587e−22	0.5975435	5.723368e−18	POS	KIF9	Stromal_score	2.819777e−13	−0.4691666	7.483406e−09	NEG
720	PRKAR2B	Stromal_score	4.473253e−21	0.5820843	1.187157e−16	POS	EBNA1BP2	Stromal_score	1.079735e−08	−0.3759792	2.865509e−04	NEG

	gene	feature	p.value	estimate	adj.p.value	COR.ID	gene	feature	p.value	estimate	adj.p.value	COR.ID
721	PLK1	Stromal_score	2.090738e−11	−0.4345103	5.548608e−07	NEG	MMAB	Stromal_score	3.741042e−10	−0.4088855	9.928353e−06	NEG
722	RCAN2	Stromal_score	9.091759e−17	0.5249616	2.412862e−12	POS	C5orf22	Stromal_score	8.646181e−07	−0.3266815	2.294610e−02	NEG
723	CD36	Stromal_score	3.859236e−09	0.3864375	1.024203e−04	POS	ZDBF2	Stromal_score	2.313639e−09	0.3915048	6.140165e−05	POS
724	EMP1	Stromal_score	9.537471e−17	0.5246578	2.531149e−12	POS	TUB	Stromal_score	2.013401e−17	0.5343765	5.343366e−13	POS
725	PPARG	Stromal_score	1.817884e−08	−0.3705392	4.824482e−04	NEG	SLC26A1	Stromal_score	1.786442e−07	−0.3454041	4.741038e−03	NEG
726	OSR1	Stromal_score	9.686718e−07	0.3252831	2.570758e−02	POS	AP1M1	Stromal_score	8.415068e−12	0.4421682	2.233275e−07	POS
727	HOXA4	Stromal_score	4.844391e−14	0.4822886	1.285653e−09	POS	JAK1	Stromal_score	1.621578e−11	0.4366680	4.303507e−07	POS
728	GBP2	Stromal_score	4.579130e−10	0.4070059	1.215255e−05	POS	PFDN2	Stromal_score	2.410189e−07	−0.3419419	6.396400e−03	NEG
729	TAGAP	Stromal_score	6.281249e−09	0.3815319	1.666981e−04	POS	BC038731	Stromal_score	6.113709e−07	−0.3309041	1.622517e−02	NEG
730	C19orf46	Stromal_score	2.375840e−07	−0.3421088	6.305243e−03	NEG	AK311356	Stromal_score	8.822907e−07	−0.3264331	2.341511e−02	NEG
731	PYGM	Stromal_score	4.182620e−13	0.4661510	1.110025e−08	POS	IFRD2	Stromal_score	2.396061e−12	−0.4524338	6.358905e−08	NEG
732	DKK3	Stromal_score	1.082151e−14	0.4930298	2.871921e−10	POS	ESCO1	Stromal_score	7.194635e−11	−0.4237945	1.909384e−06	NEG
733	GINS2	Stromal_score	1.324114e−12	−0.4571637	3.514066e−08	NEG	NPHP4	Stromal_score	7.942672e−07	−0.3277214	2.107906e−02	NEG
734	FAM20C	Stromal_score	9.285091e−20	0.5657544	2.464170e−15	POS	CTU2	Stromal_score	8.985030e−07	−0.3262093	2.384537e−02	NEG
735	RASEF	Stromal_score	1.189699e−08	−0.3749740	3.157341e−04	NEG	SNORA71A	Stromal_score	1.086543e−07	−0.3510617	2.883578e−03	NEG
736	FCGR2A	Stromal_score	9.771012e−17	0.5245042	2.593129e−12	POS	CAMKMT	Stromal_score	3.015556e−11	−0.4313734	8.002983e−07	NEG
737	PENK	Stromal_score	6.905259e−11	0.4241566	1.832587e−06	POS	KIAA0247	Stromal_score	4.572631e−18	0.5433564	1.213530e−13	POS
738	STOM	Stromal_score	4.849459e−24	0.6157834	1.286998e−19	POS	ISL2	Stromal_score	2.062750e−07	0.3437467	5.474332e−03	POS
739	GFPT2	Stromal_score	5.799641e−30	0.6728364	1.539167e−25	POS	CXorf38	Stromal_score	1.236351e−07	−0.3496027	3.281151e−03	NEG
740	RRAS	Stromal_score	7.402294e−09	0.3798596	1.964495e−04	POS	DUS1L	Stromal_score	1.103184e−12	−0.4586049	2.927739e−08	NEG
741	C3AR1	Stromal_score	4.322246e−13	0.4658985	1.147081e−08	POS	RNF32	Stromal_score	1.456068e−07	−0.3477443	3.864260e−03	NEG
742	SORD	Stromal_score	4.285173e−09	−0.3853899	1.137242e−04	NEG	ANKRD13A	Stromal_score	2.419839e−07	0.3418954	6.422011e−03	POS
743	ACTN1	Stromal_score	2.839119e−12	0.4510672	7.534739e−08	POS	HIST1H2BL	Stromal_score	1.585449e−10	−0.4167405	4.207623e−06	NEG
744	CD34	Stromal_score	1.484597e−24	0.6212394	3.939973e−20	POS	CHST12	Stromal_score	9.019420e−07	0.3261623	2.393664e−02	POS
745	CDC42EP3	Stromal_score	1.327262e−15	0.5074623	3.522420e−11	POS	CDK16	Stromal_score	4.101322e−11	−0.4287145	1.088450e−06	NEG
746	C5AR1	Stromal_score	1.699652e−16	0.5209668	4.510706e−12	POS	TMEM191A	Stromal_score	8.852558e−09	−0.3780269	2.349380e−04	NEG
747	ACAP1	Stromal_score	5.693825e−07	0.3317631	1.511084e−02	POS	APITD1−CORT	Stromal_score	9.412124e−12	−0.4412363	2.497884e−07	NEG
748	CBX7	Stromal_score	1.645697e−15	0.5060142	4.367516e−11	POS	TRPC4	Stromal_score	4.641570e−08	0.3604898	1.231826e−03	POS
749	SCARF2	Stromal_score	1.562619e−24	0.6210056	4.147034e−20	POS	SELRC1	Stromal_score	1.678750e−09	−0.3946385	4.455234e−05	NEG
750	SEMA4G	Stromal_score	5.245113e−08	−0.3591535	1.392000e−03	NEG	PMS2P1	Stromal_score	5.580107e−07	−0.3320062	1.480905e−02	NEG
751	HIST1H2BC	Stromal_score	3.916368e−07	−0.3362389	1.039365e−02	NEG	PMEL	Stromal_score	2.302597e−09	−0.3915518	6.110862e−05	NEG
752	MYADM	Stromal_score	2.104491e−14	0.4883097	5.585109e−10	POS	C1orf159	Stromal_score	1.104187e−15	−0.5086960	2.930401e−11	NEG
753	LRRC32	Stromal_score	3.159726e−28	0.6573377	8.385597e−24	POS	CHCHD8	Stromal_score	7.157520e−07	−0.3289915	1.899534e−02	NEG
754	SPI1	Stromal_score	5.696005e−12	0.4453944	1.511663e−07	POS	DQ581019	Stromal_score	1.048453e−06	−0.3243050	2.782489e−02	NEG
755	TCEAL3	Stromal_score	3.062811e−11	0.4312396	8.128394e−07	POS	DDX31	Stromal_score	6.156124e−12	−0.4447549	1.633774e−07	NEG
756	PLEKHO1	Stromal_score	3.075667e−30	0.6752118	8.162513e−26	POS	ARL6IP6	Stromal_score	6.736115e−08	−0.3563991	1.787697e−03	NEG
757	PDGFRL	Stromal_score	2.118029e−17	0.5340648	5.621036e−13	POS	GLRB	Stromal_score	1.316503e−10	0.4184148	3.493867e−06	POS
758	NID1	Stromal_score	1.030657e−15	0.5091568	2.735261e−11	POS	MMACHC	Stromal_score	2.721792e−10	−0.4118196	7.223365e−06	NEG
759	FAP	Stromal_score	1.579113e−19	0.5627995	4.190807e−15	POS	FASTKD1	Stromal_score	2.763501e−08	−0.3660917	7.334056e−04	NEG
760	AIF1	Stromal_score	2.239497e−14	0.4878648	5.943401e−10	POS	PIP4K2B	Stromal_score	2.854102e−07	0.3399689	7.574503e−03	POS
761	MIR3606	Stromal_score	1.084314e−19	0.5648943	2.877660e−15	POS	FASTKD3	Stromal_score	3.341632e−16	−0.5165905	8.868356e−12	NEG
762	SELPLG	Stromal_score	1.875210e−09	0.3935610	4.976621e−05	POS	FCGR2C	Stromal_score	1.120925e−09	0.3985382	2.974823e−05	POS
763	TENC1	Stromal_score	1.739491e−25	0.6308572	4.616435e−21	POS	GMCL1	Stromal_score	1.569578e−07	−0.3468874	4.165502e−03	NEG
764	NR4A3	Stromal_score	1.440755e−11	0.4376667	3.823619e−07	POS	KL	Stromal_score	2.953250e−08	0.3653802	7.837630e−04	POS
765	SIGLEC1	Stromal_score	2.524380e−08	0.3670585	6.699452e−04	POS	INTS7	Stromal_score	1.930699e−12	−0.4541644	5.123882e−08	NEG
766	PLCG2	Stromal_score	4.862333e−08	0.3599826	1.290415e−03	POS	MGAT1	Stromal_score	2.096716e−18	0.5479744	5.564475e−14	POS
767	LY96	Stromal_score	4.609464e−15	0.4989818	1.223306e−10	POS	C20orf94	Stromal_score	4.519679e−07	−0.3345336	1.199477e−02	NEG
768	PTGS1	Stromal_score	4.105540e−15	0.4997804	1.089569e−10	POS	SNRPC	Stromal_score	2.851823e−08	−0.3657549	7.568453e−04	NEG
769	CDCP1	Stromal_score	1.027527e−06	−0.3245545	2.726955e−02	NEG	DL490307	Stromal_score	1.531415e−08	0.3723410	4.064221e−04	POS
770	CYFIP2	Stromal_score	1.137607e−06	0.3232928	3.019097e−02	POS	ARMC7	Stromal_score	2.060940e−07	−0.3437569	5.469528e−03	NEG
771	MYH10	Stromal_score	6.766097e−14	0.4798423	1.795655e−09	POS	PCDHB4	Stromal_score	2.179759e−16	0.5193636	5.784862e−12	POS
772	KIT	Stromal_score	9.428580e−11	0.4213987	2.502251e−06	POS	ZFP106	Stromal_score	1.738815e−06	0.3179706	4.614640e−02	POS
773	AKR1B1	Stromal_score	2.157278e−07	0.3432284	5.725200e−03	POS	HNRNPF	Stromal_score	8.617755e−07	−0.3267220	2.287066e−02	NEG
774	CD69	Stromal_score	4.577837e−07	0.3343809	1.214912e−02	POS	ARSB	Stromal_score	3.204521e−19	0.5588154	8.504477e−15	POS
775	LOC401093	Stromal_score	2.946149e−14	0.4858943	7.818786e−10	POS	AF007131	Stromal_score	1.068613e−10	−0.4202830	2.835993e−06	NEG
776	AURKB	Stromal_score	1.161798e−10	−0.4195357	3.083295e−06	NEG	KIAA0845	Stromal_score	1.249242e−08	0.3744666	3.315362e−04	POS
777	TMEM100	Stromal_score	2.856999e−13	0.4690667	7.582190e−09	POS	DLEU2	Stromal_score	5.090418e−09	−0.3836588	1.350946e−04	NEG
778	COL18A1	Stromal_score	5.560620e−12	0.4455921	1.475733e−07	POS	NLGN3	Stromal_score	4.256894e−08	0.3614319	1.129737e−03	POS
779	MSR1	Stromal_score	7.898954e−12	0.4426938	2.096303e−07	POS	ATAT1	Stromal_score	1.297374e−07	−0.3490566	3.443100e−03	NEG
780	PKIG	Stromal_score	1.251955e−15	0.5078545	3.322564e−11	POS	PDIA3P	Stromal_score	8.286374e−07	−0.3272028	2.199121e−02	NEG
781	EGR3	Stromal_score	3.128275e−07	0.3388926	8.302129e−03	POS	SNX2	Stromal_score	1.386827e−08	0.3733780	3.680499e−04	POS
782	FILIP1	Stromal_score	2.372629e−16	0.5188153	6.296720e−12	POS	CEP78	Stromal_score	2.640516e−07	−0.3408784	7.007665e−03	NEG
783	ARL4D	Stromal_score	3.759841e−11	0.4294685	9.978241e−07	POS	MIR3120	Stromal_score	1.412315e−12	0.4566529	3.748143e−08	POS
784	WASF3	Stromal_score	1.750844e−06	0.3178833	4.646565e−02	POS	MECP2	Stromal_score	1.661583e−07	0.3462355	4.409674e−03	POS
785	CENPF	Stromal_score	2.885786e−13	−0.4689904	7.658587e−09	NEG	PIDD	Stromal_score	6.326446e−09	−0.3814591	1.678975e−04	NEG
786	HMGCS1	Stromal_score	1.261992e−08	−0.3743609	3.349201e−04	NEG	GTPBP10	Stromal_score	1.883145e−06	−0.3169589	4.997678e−02	NEG
787	FAM84B	Stromal_score	1.786344e−06	−0.3176289	4.740779e−02	NEG	PDE8B	Stromal_score	3.538590e−07	0.3374400	9.391064e−03	POS
788	CYP51A1	Stromal_score	1.502518e−08	−0.3725405	3.987531e−04	NEG	CCNJ	Stromal_score	1.131614e−09	−0.3984472	3.003190e−05	NEG
789	DTX1	Stromal_score	6.007469e−13	0.4633555	1.594322e−08	POS	HN1L	Stromal_score	1.163007e−07	−0.3502944	3.086505e−03	NEG
790	EPHA3	Stromal_score	2.697971e−19	0.5597890	7.160144e−15	POS	CYP2R1	Stromal_score	1.696650e−06	−0.3182814	4.502739e−02	NEG
791	RASSF8	Stromal_score	4.784287e−20	0.5694029	1.269702e−15	POS	CCT3	Stromal_score	8.426987e−12	−0.4421565	2.236438e−07	NEG
792	PDPN	Stromal_score	2.621336e−16	0.5181694	6.956764e−12	POS	FAM86C2P	Stromal_score	4.055007e−13	−0.4663890	1.076158e−08	NEG
793	CKS2	Stromal_score	6.202108e−10	−0.4041629	1.645977e−05	NEG	LOC100507564	Stromal_score	1.229438e−13	−0.4754204	3.262805e−09	NEG
794	STARD10	Stromal_score	2.285324e−08	−0.3681176	6.065020e−04	NEG	ILF2	Stromal_score	2.421486e−08	−0.3675019	6.426380e−04	NEG
795	FAM83F	Stromal_score	1.005124e−07	−0.3519380	2.667498e−03	NEG	AASS	Stromal_score	7.653018e−10	0.4021772	2.031034e−05	POS
796	TMEM125	Stromal_score	8.595378e−14	−0.4780781	2.281127e−09	NEG	ZNF671	Stromal_score	1.191989e−08	0.3749541	3.163419e−04	POS
797	PPP1R16B	Stromal_score	3.261245e−12	0.4499462	8.655018e−08	POS	FANCF	Stromal_score	4.099411e−09	−0.3858338	1.087943e−04	NEG
798	MAP1A	Stromal_score	2.934274e−28	0.6576332	7.787271e−24	POS	CASKIN1	Stromal_score	4.971075e−11	−0.4270398	1.319274e−06	NEG
799	C1orf210	Stromal_score	3.647977e−12	−0.4490369	9.681365e−08	NEG	CREBRF	Stromal_score	1.477752e−12	0.4562936	3.921807e−08	POS
800	ARHGEF26	Stromal_score	6.522467e−11	0.4246589	1.730997e−06	POS	CCDC77	Stromal_score	1.699564e−09	−0.3945187	4.510474e−05	NEG
801	NFASC	Stromal_score	2.941112e−21	0.5842721	7.805417e−17	POS	KIAA0355	Stromal_score	1.483496e−10	0.4173402	3.937051e−06	POS
802	CORO2A	Stromal_score	1.036709e−07	−0.3515902	2.751322e−03	NEG	RORA	Stromal_score	2.117404e−17	0.5340666	5.619379e−13	POS
803	DHCR7	Stromal_score	8.066717e−12	−0.4425194	2.140826e−07	NEG	BC095475	Stromal_score	4.914380e−09	−0.3840134	1.304227e−04	NEG
804	LAIR1	Stromal_score	2.229413e−13	0.4709493	5.916639e−09	POS	LRWD1	Stromal_score	1.250336e−06	−0.3221164	3.318268e−02	NEG
805	CELF2	Stromal_score	3.344461e−22	0.5953571	8.875865e−18	POS	F8	Stromal_score	8.181593e−10	0.4015435	2.171313e−05	POS
806	ERBB3	Stromal_score	4.452387e−11	−0.4280005	1.181619e−06	NEG	CTAGE5	Stromal_score	1.137427e−08	−0.3754401	3.018618e−04	NEG
807	RECQL4	Stromal_score	4.030277e−13	−0.4664360	1.069595e−08	NEG	KIAA1755	Stromal_score	2.074973e−14	0.4884107	5.506771e−10	POS
808	ITPKB	Stromal_score	4.920939e−19	0.5563743	1.305968e−14	POS	ARAP1	Stromal_score	1.397484e−09	0.3964153	3.708782e−05	POS
809	PTTG1	Stromal_score	7.046927e−09	−0.3803616	1.870184e−04	NEG	PPIL1	Stromal_score	8.627292e−07	−0.3267084	2.289597e−02	NEG
810	KIAA0101	Stromal_score	2.613561e−11	−0.4326026	6.936128e−07	NEG	UQCC	Stromal_score	8.364740e−08	−0.3539939	2.219918e−03	NEG
811	CRNDE	Stromal_score	2.172860e−08	−0.3686533	5.766553e−04	NEG	PAK1IP1	Stromal_score	4.003741e−08	−0.3620977	1.062553e−03	NEG
812	MMRN1	Stromal_score	2.892082e−14	0.4860278	7.675297e−10	POS	KIAA1432	Stromal_score	9.210241e−07	0.3259047	2.444306e−02	POS
813	CD209	Stromal_score	2.760324e−07	0.3403599	7.325623e−03	POS	NRK	Stromal_score	1.747670e−19	0.5622319	4.638142e−15	POS
814	HMGA1	Stromal_score	1.208296e−11	−0.4391470	3.206698e−07	NEG	ISLR2	Stromal_score	3.213817e−09	0.3882599	8.529150e−05	POS
815	NCAM1	Stromal_score	3.227571e−07	0.3385250	8.565651e−03	POS	PPM1F	Stromal_score	2.309260e−17	0.5335323	6.128544e−13	POS
816	GSTO2	Stromal_score	1.348126e−07	−0.3486209	3.577790e−03	NEG	PRCD	Stromal_score	2.262655e−10	0.4135108	6.004861e−06	POS
817	HMOX1	Stromal_score	1.243680e−07	0.3495357	3.300602e−03	POS	TIGD1	Stromal_score	1.020415e−14	−0.4934432	2.708080e−10	NEG
818	ECT2	Stromal_score	1.356952e−09	−0.3966995	3.601214e−05	NEG	SFR1	Stromal_score	7.850554e−07	−0.3278640	2.083458e−02	NEG
819	PVRIG	Stromal_score	1.610057e−06	0.3189433	4.272931e−02	POS	TBC1D7	Stromal_score	2.473598e−14	−0.4871519	6.564682e−10	NEG
820	LOC100128252	Stromal_score	9.779033e−24	0.6124997	2.595257e−19	POS	GUF1	Stromal_score	3.134258e−07	−0.3388701	8.318008e−03	NEG
821	CDH11	Stromal_score	2.933727e−35	0.7149330	7.785819e−31	POS	HAUS8	Stromal_score	3.225145e−11	−0.4307945	8.559212e−07	NEG
822	AK130904	Stromal_score	4.541369e−13	0.4655178	1.205234e−08	POS	AIMP2	Stromal_score	2.931555e−11	−0.4316166	7.780054e−07	NEG
823	KCND3	Stromal_score	1.236114e−08	0.3745764	3.280523e−04	POS	ORC5	Stromal_score	3.054609e−07	−0.3391726	8.106626e−03	NEG
824	NEK2	Stromal_score	2.323974e−16	−0.5189494	6.167594e−12	NEG	ADAMTS5	Stromal_score	5.274826e−10	0.4056838	1.399886e−05	POS
825	LCP2	Stromal_score	1.135322e−10	0.4197419	3.013030e−06	POS	WBSCR22	Stromal_score	3.077463e−07	−0.3390850	8.167278e−03	NEG
826	CCNO	Stromal_score	5.820923e−07	−0.3314969	1.544815e−02	NEG	MYCBP2	Stromal_score	3.464526e−07	0.3376898	9.194504e−03	POS
827	CD93	Stromal_score	1.171641e−22	0.6005562	3.109417e−18	POS	STARD9	Stromal_score	5.301733e−16	0.5135660	1.407027e−11	POS
828	APOL3	Stromal_score	7.704760e−08	0.3549091	2.044766e−03	POS	C8orf42	Stromal_score	1.663789e−16	0.5211039	4.415529e−12	POS
829	GADD45B	Stromal_score	1.022474e−11	0.4405452	2.713544e−07	POS	COX5B	Stromal_score	7.090161e−07	−0.3291066	1.881658e−02	NEG
830	RASAL3	Stromal_score	3.200681e−09	0.3883005	8.494289e−05	POS	FAM136A	Stromal_score	3.918389e−16	−0.5155506	1.039901e−11	NEG
831	BVES	Stromal_score	5.596797e−12	0.4455388	1.485334e−07	POS	UCN	Stromal_score	8.695306e−07	−0.3266120	2.307647e−02	NEG
832	CDKN3	Stromal_score	1.076842e−07	−0.3511627	2.857832e−03	NEG	LCORL	Stromal_score	5.363734e−12	−0.4458882	1.423481e−07	NEG
833	NNAT	Stromal_score	1.624267e−07	0.3464956	4.310643e−03	POS	ZDHHC11	Stromal_score	3.390078e−08	−0.3638969	8.996928e−04	NEG
834	TST	Stromal_score	5.307054e−07	−0.3326099	1.408439e−02	NEG	GPM6A	Stromal_score	1.182295e−10	0.4193791	3.137692e−06	POS
835	PKP3	Stromal_score	1.968156e−10	−0.4147811	5.223288e−06	NEG	LOC100506939	Stromal_score	4.956030e−13	0.4648441	1.315281e−08	POS
836	TGFBR2	Stromal_score	1.118403e−09	0.3985598	2.968130e−05	POS	ERI2	Stromal_score	3.649713e−10	−0.4091145	9.685972e−06	NEG
837	COL16A1	Stromal_score	2.959552e−22	0.5959680	7.854356e−18	POS	ZRANB2−AS1	Stromal_score	5.606904e−13	0.4638902	1.488016e−08	POS
838	LAMC1	Stromal_score	1.039382e−13	0.4766702	2.758416e−09	POS	MTRF1	Stromal_score	3.733424e−11	−0.4295296	9.908135e−07	NEG
839	FOXS1	Stromal_score	1.696266e−11	0.4362868	4.501719e−07	POS	LOC100506730	Stromal_score	1.421894e−06	−0.3205074	3.773565e−02	NEG
840	LSR	Stromal_score	7.649048e−08	−0.3549898	2.029981e−03	NEG	CBX3	Stromal_score	9.579775e−07	−0.3254200	2.542377e−02	NEG

	gene	feature	p.value	estimate	adj.p.value	COR.ID	gene	feature	p.value	estimate	adj.p.value	COR.ID
841	MAFB	Stromal_score	3.608487e−32	0.6912595	9.576565e−28	POS	MYCBP	Stromal_score	4.520747e−08	−0.3607774	1.199761e−03	NEG
842	ASB5	Stromal_score	3.096366e−07	0.3390131	8.217445e−03	POS	B4GALT3	Stromal_score	6.769890e−08	−0.3563438	1.796661e−03	NEG
843	MIR568	Stromal_score	7.730877e−20	0.5667669	2.051698e−15	POS	LOC100505678	Stromal_score	1.635283e−08	0.3716528	4.339878e−04	POS
844	SMARCA1	Stromal_score	2.916861e−07	0.3397140	7.741059e−03	POS	TPCN1	Stromal_score	3.040589e−07	0.3392266	8.069418e−03	POS
845	ARHGAP23	Stromal_score	1.659150e−12	0.4553734	4.403219e−08	POS	GEMIN6	Stromal_score	1.177246e−08	−0.3750832	3.124293e−04	NEG
846	SLC15A3	Stromal_score	8.513410e−09	0.3784280	2.259374e−04	POS	ATIC	Stromal_score	5.534227e−13	−0.4639912	1.468729e−08	NEG
847	OVOL2	Stromal_score	5.307028e−09	−0.3832384	1.408432e−04	NEG	SNAPC4	Stromal_score	2.064083e−07	−0.3437392	5.477871e−03	NEG
848	PDE5A	Stromal_score	5.930003e−13	0.4634561	1.573763e−08	POS	HHIPL1	Stromal_score	1.119159e−09	0.3985534	2.970135e−05	POS
849	LXN	Stromal_score	2.039041e−11	0.4347236	5.411411e−07	POS	DNAJC18	Stromal_score	1.464853e−14	0.4908900	3.887575e−10	POS
850	CLEC10A	Stromal_score	3.443763e−14	0.4847674	9.139404e−10	POS	FKBP14	Stromal_score	1.446834e−10	0.4175657	3.839753e−06	POS
851	SAMHD1	Stromal_score	8.336322e−13	0.4608039	2.212377e−08	POS	FAM210A	Stromal_score	3.126269e−07	−0.3389001	8.296804e−03	NEG
852	TACC1	Stromal_score	2.960752e−14	0.4858587	7.857540e−10	POS	LOC646762	Stromal_score	9.221299e−09	−0.3776072	2.447241e−04	NEG
853	FCRL1	Stromal_score	7.513391e−07	0.3284001	1.993979e−02	POS	EPN2	Stromal_score	4.969326e−07	0.3333991	1.318809e−02	POS
854	CAMSAP3	Stromal_score	9.754162e−16	−0.5095246	2.588657e−11	NEG	CCDC137	Stromal_score	2.591397e−07	−0.3410975	6.877308e−03	NEG
855	KIF2C	Stromal_score	1.816959e−12	−0.4546493	4.822028e−08	NEG	GDF10	Stromal_score	8.665883e−09	0.3782458	2.299839e−04	POS
856	BC015662	Stromal_score	3.069722e−12	−0.4504361	8.146736e−08	NEG	CHST7	Stromal_score	1.569720e−10	0.4168305	4.165880e−06	POS
857	ABLIM3	Stromal_score	2.066354e−07	0.3437265	5.483896e−03	POS	SNORA65	Stromal_score	1.503084e−07	−0.3473818	3.989034e−03	NEG
858	TRIP13	Stromal_score	1.252287e−14	−0.4919998	3.323444e−10	NEG	UCKL1	Stromal_score	3.812778e−07	−0.3365568	1.011873e−02	NEG
859	LOX	Stromal_score	6.063556e−19	0.5551788	1.609207e−14	POS	IFFO1	Stromal_score	4.326634e−27	0.6466724	1.148245e−22	POS
860	DOCK2	Stromal_score	7.552862e−11	0.4233654	2.004454e−06	POS	IQCH	Stromal_score	3.609673e−08	−0.3632195	9.579712e−04	NEG
861	CDCA8	Stromal_score	3.767846e−15	−0.5003711	9.999485e−11	NEG	KLHL14	Stromal_score	3.176430e−07	0.3387129	8.429927e−03	POS
862	MCF2L−AS1	Stromal_score	3.481972e−07	−0.3376305	9.240806e−03	NEG	PRND	Stromal_score	2.269612e−07	0.3426400	6.023324e−03	POS
863	GPR155	Stromal_score	3.566909e−08	0.3633482	9.466220e−04	POS	NOP14	Stromal_score	1.470981e−08	−0.3727624	3.903836e−04	NEG
864	AOAH	Stromal_score	3.194387e−09	0.3883201	8.477585e−05	POS	NANP	Stromal_score	1.240552e−09	−0.3975639	3.292301e−05	NEG
865	C20orf151	Stromal_score	9.487260e−09	−0.3773145	2.517824e−04	NEG	NFYA	Stromal_score	2.242975e−08	−0.3683163	5.952630e−04	NEG
866	C4B	Stromal_score	7.640002e−19	0.5538499	2.027580e−14	POS	HS3ST3A1	Stromal_score	8.638657e−07	0.3266922	2.292613e−02	POS
867	ICA1	Stromal_score	1.686179e−08	−0.3713308	4.474952e−04	NEG	RPS6KB2	Stromal_score	2.477658e−07	−0.3416206	6.575456e−03	NEG
868	ZSCAN18	Stromal_score	2.033725e−13	0.4716437	5.397302e−09	POS	GLRX2	Stromal_score	1.570547e−08	−0.3720766	4.168075e−04	NEG
869	CNTNAP1	Stromal_score	2.977386e−23	0.6072070	7.901686e−19	POS	INPP4A	Stromal_score	6.874863e−09	0.3806135	1.824520e−04	POS
870	BIRC5	Stromal_score	3.993332e−15	−0.4999712	1.059790e−10	NEG	DUS3L	Stromal_score	3.332072e−07	−0.3381497	8.842985e−03	NEG
871	GMFG	Stromal_score	2.110149e−13	0.4713651	5.600125e−09	POS	SNORA6	Stromal_score	1.298025e−06	−0.3216489	3.444828e−02	NEG
872	NOTCH3	Stromal_score	1.330104e−12	0.4571279	3.529962e−08	POS	FAM172A	Stromal_score	3.836161e−09	0.3864974	1.018079e−04	POS
873	PYCR1	Stromal_score	1.542592e−10	−0.4169879	4.093885e−06	NEG	NCL	Stromal_score	1.672326e−07	−0.3461616	4.438185e−03	NEG
874	PMP22	Stromal_score	1.089982e−27	0.6523445	2.892704e−23	POS	C6orf72	Stromal_score	4.054576e−11	0.4288141	1.076044e−06	POS
875	WNT9A	Stromal_score	1.185167e−11	0.4393092	3.145315e−07	POS	PDLIM5	Stromal_score	5.538995e−09	0.3828061	1.469994e−04	POS
876	BC058547	Stromal_score	4.504750e−14	0.4828183	1.195516e−09	POS	ZNF626	Stromal_score	1.009617e−12	0.4593023	2.679422e−08	POS
877	ANXA4	Stromal_score	3.778810e−07	−0.3366628	1.002858e−02	NEG	SMARCD2	Stromal_score	7.584746e−09	−0.3796108	2.012916e−04	NEG
878	SCPEP1	Stromal_score	2.935121e−10	0.4111261	7.789517e−06	POS	LOC100129269	Stromal_score	9.160180e−07	0.3259718	2.431020e−02	POS
879	MYO5B	Stromal_score	2.160124e−07	−0.3432131	5.732753e−03	NEG	LOC100507050	Stromal_score	3.511338e−08	0.3635177	9.318740e−04	POS
880	RIPK4	Stromal_score	2.119181e−08	−0.3689185	5.624096e−04	NEG	TRPC1	Stromal_score	1.518058e−18	0.5498665	4.028774e−14	POS
881	EPB41L4B	Stromal_score	1.059611e−13	−0.4765270	2.812101e−09	NEG	CEP68	Stromal_score	3.558951e−07	0.3373722	9.445100e−03	POS
882	MSN	Stromal_score	8.571369e−25	0.6237354	2.274756e−20	POS	ZNF138	Stromal_score	1.359516e−10	−0.4181259	3.608021e−06	NEG
883	ASF1B	Stromal_score	4.886588e−12	−0.4466522	1.296851e−07	NEG	AK097472	Stromal_score	1.300831e−06	−0.3216219	3.452276e−02	NEG
884	RBP5	Stromal_score	1.244142e−11	0.4389015	3.301829e−07	POS	FIGN	Stromal_score	7.934029e−07	0.3277347	2.105612e−02	POS
885	SVEP1	Stromal_score	5.185255e−25	0.6259997	1.376115e−20	POS	NEFL	Stromal_score	2.666135e−07	0.3407656	7.075656e−03	POS
886	MELK	Stromal_score	7.867721e−12	−0.4427266	2.088014e−07	NEG	OXSM	Stromal_score	1.048379e−11	−0.4403361	2.782293e−07	NEG
887	TSTA3	Stromal_score	6.993921e−08	−0.3559834	1.856117e−03	NEG	ZNF823	Stromal_score	2.255554e−13	−0.4708611	5.986015e−09	NEG
888	PECAM1	Stromal_score	8.304266e−28	0.6534494	2.203869e−23	POS	ATAD5	Stromal_score	8.323503e−15	−0.4948726	2.208974e−10	NEG
889	PXDN	Stromal_score	2.664980e−14	0.4866165	7.072592e−10	POS	PRMT3	Stromal_score	1.083718e−10	−0.4201577	2.876080e−06	NEG
890	PLVAP	Stromal_score	6.947029e−14	0.4796483	1.843672e−09	POS	LOC148709	Stromal_score	2.028457e−07	−0.3439404	5.383323e−03	NEG
891	CCNB2	Stromal_score	8.692649e−10	−0.4009675	2.306942e−05	NEG	KIAA0907	Stromal_score	1.823087e−09	−0.3938358	4.838291e−05	NEG
892	SSBP2	Stromal_score	3.722290e−16	0.5158863	9.878585e−12	POS	PMPCA	Stromal_score	1.246992e−08	−0.3744853	3.309393e−04	NEG
893	DMD	Stromal_score	8.493358e−12	0.4420913	2.254052e−07	POS	ARHGAP1	Stromal_score	5.289302e−19	0.5559615	1.403728e−14	POS
894	LAMA2	Stromal_score	1.167804e−25	0.6326081	3.099236e−21	POS	GRIN2A	Stromal_score	1.020097e−06	0.3246442	2.707235e−02	POS
895	LIMD2	Stromal_score	2.572570e−07	0.3411825	6.827345e−03	POS	NUP50	Stromal_score	8.566297e−07	−0.3267955	2.273410e−02	NEG
896	MARVELD1	Stromal_score	1.666178e−21	0.5872107	4.421869e−17	POS	ITIH3	Stromal_score	2.731482e−10	0.4117869	7.249080e−06	POS
897	ESRP1	Stromal_score	1.945088e−09	−0.3932040	5.162068e−05	NEG	SLITRK5	Stromal_score	6.977729e−14	0.4796158	1.851820e−09	POS
898	NFIB	Stromal_score	1.954837e−09	0.3931552	5.187943e−05	POS	CIC	Stromal_score	5.254225e−09	0.3833393	1.394419e−04	POS
899	FMO1	Stromal_score	1.677431e−14	0.4899275	4.451733e−10	POS	ENOX1	Stromal_score	1.020186e−23	0.6123003	2.707473e−19	POS
900	VIM	Stromal_score	6.447872e−34	0.7049380	1.711201e−29	POS	AK092965	Stromal_score	2.176160e−14	0.4880702	5.775312e−10	POS
901	NPTX2	Stromal_score	1.609905e−06	0.3189445	4.272528e−02	POS	C6orf52	Stromal_score	1.469600e−09	−0.3959288	3.900172e−05	NEG
902	SCG5	Stromal_score	1.607898e−06	0.3189602	4.267201e−02	POS	AUP1	Stromal_score	7.979884e−12	−0.4426092	2.117781e−07	NEG
903	HMGB3	Stromal_score	5.091737e−08	−0.3594785	1.351296e−03	NEG	MIR3607	Stromal_score	1.868818e−06	−0.3170559	4.959657e−02	NEG
904	DANCR	Stromal_score	7.236646e−07	−0.3288576	1.920534e−02	NEG	MED20	Stromal_score	1.132211e−07	−0.3505974	3.004776e−03	NEG
905	TTK	Stromal_score	9.415969e−15	−0.4940081	2.498904e−10	NEG	TXNDC15	Stromal_score	1.795962e−16	0.5206124	4.766304e−12	POS
906	CERCAM	Stromal_score	1.520404e−16	0.5216823	4.035000e−12	POS	E2F3	Stromal_score	6.762364e−07	−0.3296820	1.794664e−02	NEG
907	AK024736	Stromal_score	1.439451e−11	−0.4376743	3.820159e−07	NEG	FAM212A	Stromal_score	5.962864e−13	0.4634133	1.582484e−08	POS
908	SKA3	Stromal_score	1.313487e−18	−0.5507106	3.485862e−14	NEG	CREBBP	Stromal_score	4.212968e−09	0.3855602	1.118080e−04	POS
909	GPR124	Stromal_score	6.743776e−35	0.7122823	1.789731e−30	POS	FBXO45	Stromal_score	4.233403e−13	−0.4660582	1.123503e−08	NEG
910	ST3GAL4	Stromal_score	1.722308e−06	0.3180914	4.570833e−02	POS	BRAT1	Stromal_score	2.972742e−10	−0.4110089	7.889360e−06	NEG
911	LGALS1	Stromal_score	1.137127e−19	0.5646301	3.017820e−15	POS	EXOSC2	Stromal_score	1.798108e−09	−0.3939702	4.771998e−05	NEG
912	AL832909	Stromal_score	3.437068e−10	0.4096700	9.121634e−06	POS	NAP1L3	Stromal_score	1.357503e−31	0.6865836	3.602677e−27	POS
913	RAMP3	Stromal_score	6.465829e−19	0.5548100	1.715966e−14	POS	HSPA14	Stromal_score	6.905264e−12	−0.4438071	1.832588e−07	NEG
914	BRI3BP	Stromal_score	1.966483e−09	−0.3930971	5.218850e−05	NEG	SERHL	Stromal_score	1.431457e−07	−0.3479385	3.798943e−03	NEG
915	CCNA2	Stromal_score	1.125606e−11	−0.4397414	2.987246e−07	NEG	CIRH1A	Stromal_score	2.338230e−07	−0.3422942	6.205427e−03	NEG
916	SAMSN1	Stromal_score	1.599389e−10	0.4166614	4.244618e−06	POS	PDZD2	Stromal_score	6.062558e−08	0.3575621	1.608942e−03	POS
917	NR_026754	Stromal_score	4.456576e−17	0.5294508	1.182731e−12	POS	RSU1	Stromal_score	6.369330e−12	0.4444742	1.690356e−07	POS
918	KIAA1274	Stromal_score	2.126510e−09	0.3923319	5.643546e−05	POS	SNRPG	Stromal_score	8.034001e−09	−0.3790221	2.132144e−04	NEG
919	AF339817	Stromal_score	4.623197e−08	−0.3605331	1.226950e−03	NEG	MMP23B	Stromal_score	8.032706e−12	0.4425545	2.131800e−07	POS
920	CRIP2	Stromal_score	4.985199e−12	0.4464885	1.323022e−07	POS	PNPO	Stromal_score	1.126764e−07	−0.3506519	2.990318e−03	NEG
921	CCNB1	Stromal_score	1.582245e−09	−0.3952133	4.199120e−05	NEG	FUT11	Stromal_score	1.442314e−09	0.3961101	3.827757e−05	POS
922	ARHGAP9	Stromal_score	7.191054e−07	0.3289346	1.908434e−02	POS	ZNF131	Stromal_score	1.183783e−09	−0.3980144	3.141643e−05	NEG
923	ADAP1	Stromal_score	5.036567e−10	−0.4061165	1.336654e−05	NEG	ZNF692	Stromal_score	1.409567e−13	−0.4743990	3.740850e−09	NEG
924	CGNL1	Stromal_score	6.619717e−10	0.4035487	1.756807e−05	POS	RNF11	Stromal_score	5.619228e−14	0.4812046	1.491287e−09	POS
925	RNF223	Stromal_score	3.892128e−08	−0.3624042	1.032932e−03	NEG	CEP112	Stromal_score	9.353779e−13	0.4599019	2.482399e−08	POS
926	DM074112	Stromal_score	5.782212e−18	0.5419523	1.534541e−13	POS	ZNF154	Stromal_score	1.680780e−09	0.3946268	4.460621e−05	POS
927	CACNA2D1	Stromal_score	9.480621e−12	0.4411758	2.516062e−07	POS	LOC100507501	Stromal_score	9.957784e−08	−0.3520429	2.642696e−03	NEG
928	TAC1	Stromal_score	4.843352e−07	0.3337067	1.285377e−02	POS	PHB	Stromal_score	2.401465e−09	−0.3911387	6.373248e−05	NEG
929	LST1	Stromal_score	6.168304e−11	0.4251498	1.637006e−06	POS	USP37	Stromal_score	1.720471e−10	−0.4160014	4.565958e−06	NEG
930	EDNRA	Stromal_score	1.250198e−25	0.6323093	3.317901e−21	POS	ZNF570	Stromal_score	6.675377e−08	0.3564993	1.771578e−03	POS
931	IQGAP3	Stromal_score	4.009158e−11	−0.4289118	1.063990e−06	NEG	TRIM10	Stromal_score	4.667217e−08	−0.3604297	1.238633e−03	NEG
932	SLA	Stromal_score	1.586166e−09	0.3951893	4.209526e−05	POS	AK125727	Stromal_score	1.948078e−07	0.3444070	5.170004e−03	POS
933	MCM4	Stromal_score	2.308367e−07	−0.3424435	6.126175e−03	NEG	ENOPH1	Stromal_score	3.909214e−12	−0.4484743	1.037466e−07	NEG
934	GAS7	Stromal_score	1.049509e−30	0.6791884	2.785291e−26	POS	EFCAB10	Stromal_score	9.008049e−08	−0.3531663	2.390646e−03	NEG
935	ADAM12	Stromal_score	7.499105e−08	0.3552098	1.990187e−03	POS	NAT9	Stromal_score	1.298428e−10	−0.4185390	3.445897e−06	NEG
936	MAD2L1	Stromal_score	2.849077e−13	−0.4690879	7.561165e−09	NEG	TMEM206	Stromal_score	1.664338e−08	−0.3714678	4.416988e−04	NEG
937	RAB31	Stromal_score	1.001342e−36	0.7253877	2.657463e−32	POS	WRNIP1	Stromal_score	1.388913e−09	−0.3964748	3.686037e−05	NEG
938	DPYD	Stromal_score	9.990011e−12	0.4407392	2.651249e−07	POS	AVPR1A	Stromal_score	4.504663e−14	0.4828184	1.195493e−09	POS
939	CHDH	Stromal_score	9.893753e−11	−0.4209700	2.625703e−06	NEG	SMYD2	Stromal_score	1.617747e−06	−0.3188832	4.293339e−02	NEG
940	ELMO3	Stromal_score	6.812764e−11	−0.4242754	1.808040e−06	NEG	CDK5RAP3	Stromal_score	2.919493e−07	−0.3397034	7.748042e−03	NEG
941	F2RL3	Stromal_score	4.948246e−08	0.3597912	1.313215e−03	POS	MMS22L	Stromal_score	6.370691e−12	−0.4444724	1.690718e−07	NEG
942	AK092048	Stromal_score	2.394893e−13	0.4704071	6.355806e−09	POS	MPRIP	Stromal_score	1.456676e−10	0.4175046	3.865872e−06	POS
943	KIFC1	Stromal_score	1.629884e−12	−0.4555150	4.325549e−08	NEG	RALY	Stromal_score	2.839820e−07	−0.3400276	7.536597e−03	NEG
944	LOC283070	Stromal_score	2.732487e−14	0.4864365	7.251748e−10	POS	LOC100287042	Stromal_score	9.348679e−12	−0.4412927	2.481046e−07	NEG
945	TK1	Stromal_score	2.591259e−07	−0.3410981	6.876943e−03	NEG	SMUG1	Stromal_score	4.211334e−09	−0.3855641	1.117646e−04	NEG
946	HEG1	Stromal_score	1.266483e−32	0.6948929	3.361119e−28	POS	SPATA17	Stromal_score	5.014970e−07	−0.3332895	1.330923e−02	NEG
947	RNF150	Stromal_score	1.832498e−23	0.6095265	4.863267e−19	POS	STRBP	Stromal_score	4.102507e−10	−0.4080293	1.088764e−05	NEG
948	CEP55	Stromal_score	1.617366e−10	−0.4165604	4.292328e−06	NEG	DNAJC11	Stromal_score	4.674680e−07	−0.3341308	1.240613e−02	NEG
949	TTC39A	Stromal_score	7.350672e−08	−0.3554317	1.950795e−03	NEG	MAP1LC3B	Stromal_score	9.385593e−08	0.3527067	2.490842e−03	POS
950	TWIST2	Stromal_score	4.243064e−25	0.6268980	1.126067e−20	POS	TMEM109	Stromal_score	3.539842e−13	0.4674308	9.394387e−09	POS
951	FGF10	Stromal_score	5.040692e−17	0.5286801	1.337749e−12	POS	ALG10	Stromal_score	2.559711e−10	−0.4123826	6.793217e−06	NEG
952	RASD2	Stromal_score	3.323961e−07	0.3381784	8.821461e−03	POS	TXNRD2	Stromal_score	2.804573e−10	−0.4115444	7.443055e−06	NEG
953	LIX1L	Stromal_score	1.170444e−27	0.6520544	3.106242e−23	POS	ZFYVE27	Stromal_score	2.295574e−11	−0.4337129	6.092224e−07	NEG
954	S100B	Stromal_score	8.846033e−09	0.3780344	2.347649e−04	POS	PLSCR3	Stromal_score	1.177086e−11	0.4393665	3.123869e−07	POS
955	DEPDC1B	Stromal_score	3.170876e−10	−0.4104144	8.415187e−06	NEG	SPRYD4	Stromal_score	3.373563e−08	−0.3639495	8.953099e−04	NEG
956	ADAMTS4	Stromal_score	2.509106e−12	0.4520631	6.658915e−08	POS	COX4NB	Stromal_score	7.159652e−08	−0.3557238	1.900100e−03	NEG
957	MFNG	Stromal_score	2.695675e−09	0.3900002	7.154053e−05	POS	SNORA56	Stromal_score	8.352286e−08	−0.3540105	2.216613e−03	NEG
958	FAM198B	Stromal_score	9.268184e−20	0.5657645	2.459683e−15	POS	CHCHD2	Stromal_score	2.771827e−08	−0.3660595	7.356151e−04	NEG
959	PCNA−AS1	Stromal_score	2.974023e−08	−0.3653050	7.892759e−04	NEG	PCNX	Stromal_score	7.663673e−13	0.4614611	2.033862e−08	POS
960	GALE	Stromal_score	9.119527e−11	−0.4216950	2.420231e−06	NEG	LOC100125556	Stromal_score	6.056005e−10	−0.4043873	1.607203e−05	NEG

	gene	feature	p.value	estimate	adj.p.value	COR.ID	gene	feature	p.value	estimate	adj.p.value	COR.ID
961	SLC52A3	Stromal_score	8.249619e−07	−0.3272573	2.189366e−02	NEG	GLE1	Stromal_score	4.243728e−08	−0.3614656	1.126243e−03	NEG
962	PRSS16	Stromal_score	1.707396e−09	−0.3944740	4.531258e−05	NEG	HEATR2	Stromal_score	1.184222e−13	−0.4756997	3.142806e−09	NEG
963	RASGRP2	Stromal_score	1.492080e−17	0.5362135	3.959831e−13	POS	RRNAD1	Stromal_score	1.307719e−09	−0.3970560	3.470556e−05	NEG
964	PALMD	Stromal_score	1.392832e−24	0.6215305	3.696437e−20	POS	LRRK2	Stromal_score	5.362846e−22	0.5929854	1.423246e−17	POS
965	MCM2	Stromal_score	1.452311e−08	−0.3728960	3.854289e−04	NEG	TRIM8	Stromal_score	6.603315e−07	0.3299709	1.752454e−02	POS
966	CDCA5	Stromal_score	8.171734e−14	−0.4784516	2.168697e−09	NEG	CYLD	Stromal_score	3.659484e−09	0.3869679	9.711906e−05	POS
967	CLEC11A	Stromal_score	2.396426e−22	0.5970195	6.359875e−18	POS	PDE10A	Stromal_score	1.298717e−09	0.3971226	3.446666e−05	POS
968	TMEM97	Stromal_score	1.012689e−10	−0.4207625	2.687577e−06	NEG	FLAD1	Stromal_score	3.773117e−11	−0.4294380	1.001347e−06	NEG
969	DDX3Y	Stromal_score	1.134724e−08	0.3754648	3.011444e−04	POS	WBSCR17	Stromal_score	1.397927e−11	0.4379211	3.709958e−07	POS
970	PAPLN	Stromal_score	1.492772e−21	0.5877756	3.961667e−17	POS	SNORA5A	Stromal_score	5.245062e−09	−0.3833569	1.391987e−04	NEG
971	AK097288	Stromal_score	9.752431e−15	0.4937615	2.588198e−10	POS	ZFAND5	Stromal_score	1.427275e−07	0.3479718	3.787846e−03	POS
972	PKMYT1	Stromal_score	1.437819e−08	−0.3730008	3.815829e−04	NEG	NKRF	Stromal_score	6.283734e−12	−0.4445858	1.667640e−07	NEG
973	HOXB4	Stromal_score	1.635324e−09	0.3948931	4.339986e−05	POS	HECTD2	Stromal_score	2.167026e−09	0.3921470	5.751070e−05	POS
974	FMNL1	Stromal_score	2.704566e−17	0.5325563	7.177648e−13	POS	CCDC47	Stromal_score	1.637379e−07	−0.3464035	4.345440e−03	NEG
975	IRX5	Stromal_score	3.848733e−07	−0.3364455	1.021415e−02	NEG	AAGAB	Stromal_score	1.388998e−06	−0.3208010	3.686261e−02	NEG
976	PI15	Stromal_score	1.864245e−08	0.3702737	4.947520e−04	POS	BC104003	Stromal_score	2.452365e−10	−0.4127749	6.508332e−06	NEG
977	ANKRD65	Stromal_score	1.595337e−08	0.3719123	4.233865e−04	POS	ZNHIT2	Stromal_score	2.452365e−10	−0.4127749	6.508332e−06	NEG
978	EGFL7	Stromal_score	6.288469e−09	0.3815202	1.668897e−04	POS	SFT2D3	Stromal_score	8.013557e−08	−0.3544719	2.126718e−03	NEG
979	PLSCR4	Stromal_score	4.224873e−20	0.5700821	1.121239e−15	POS	COX8A	Stromal_score	9.750001e−07	−0.3252028	2.587553e−02	NEG
980	BUB1	Stromal_score	3.376764e−12	−0.4496640	8.961595e−08	NEG	LRRC24	Stromal_score	1.437597e−06	−0.3203695	3.815240e−02	NEG
981	ELOVL6	Stromal_score	6.244990e−08	−0.3572353	1.657358e−03	NEG	KLRG1	Stromal_score	4.046967e−08	0.3619812	1.074024e−03	POS
982	KIAA1244	Stromal_score	2.394739e−10	−0.4129925	6.355398e−06	NEG	LOC100506314	Stromal_score	1.277666e−09	−0.3972801	3.390797e−05	NEG
983	ATP8B2	Stromal_score	2.669164e−33	0.7001978	7.083695e−29	POS	BC038201	Stromal_score	9.582747e−10	−0.4000384	2.543165e−05	NEG
984	TCF7L1	Stromal_score	3.090303e−14	0.4855498	8.201356e−10	POS	C21orf58	Stromal_score	4.475207e−12	−0.4473715	1.187675e−07	NEG
985	GPR133	Stromal_score	4.425522e−21	0.5821405	1.174489e−16	POS	ADD1	Stromal_score	1.046503e−15	0.5090548	2.777313e−11	POS
986	PHYHIPL	Stromal_score	3.070579e−08	0.3649621	8.149011e−04	POS	NGF	Stromal_score	7.454944e−11	0.4234807	1.978468e−06	POS
987	CCDC3	Stromal_score	7.451226e−11	0.4234851	1.977481e−06	POS	DLG4	Stromal_score	3.382762e−17	0.5311690	8.977511e−13	POS
988	ABCC9	Stromal_score	2.772727e−22	0.5962933	7.358541e−18	POS	FAM63B	Stromal_score	4.615735e−08	0.3605507	1.224970e−03	POS
989	SIM2	Stromal_score	4.453489e−12	−0.4474113	1.181912e−07	NEG	DAAM1	Stromal_score	9.542436e−07	0.3254682	2.532467e−02	POS
990	ARHGAP30	Stromal_score	1.029335e−11	0.4404894	2.731752e−07	POS	ZNF232	Stromal_score	2.718600e−08	−0.3662669	7.214892e−04	NEG
991	HCK	Stromal_score	9.194304e−08	0.3529373	2.440076e−03	POS	SUSD5	Stromal_score	1.617444e−12	0.4555760	4.292535e−08	POS
992	MEF2C	Stromal_score	1.088173e−28	0.6615615	2.887903e−24	POS	IL11RA	Stromal_score	7.359468e−08	0.3554185	1.953129e−03	POS
993	ABHD11	Stromal_score	1.003177e−07	−0.3519598	2.662331e−03	NEG	FARSB	Stromal_score	1.186750e−09	−0.3979903	3.149515e−05	NEG
994	SPINT2	Stromal_score	6.131887e−09	−0.3817762	1.627342e−04	NEG	RNF180	Stromal_score	6.941826e−16	0.5117859	1.842291e−11	POS
995	ENG	Stromal_score	6.687744e−25	0.6248557	1.774860e−20	POS	L2HGDH	Stromal_score	1.526328e−11	−0.4371797	4.050722e−07	NEG
996	FAM113B	Stromal_score	9.325412e−08	0.3527787	2.474871e−03	POS	NUFIP1	Stromal_score	5.418480e−12	−0.4458048	1.438010e−07	NEG
997	WNT2B	Stromal_score	1.821566e−11	0.4356824	4.834254e−07	POS	PDIK1L	Stromal_score	1.090095e−07	−0.3510249	2.893003e−03	NEG
998	NGEF	Stromal_score	1.411155e−06	−0.3206025	3.745065e−02	NEG	TXLNG	Stromal_score	3.030941e−10	−0.4108303	8.043813e−06	NEG
999	HMMR	Stromal_score	8.683026e−11	−0.4221305	2.304388e−06	NEG	FBXW9	Stromal_score	3.756401e−07	−0.3367333	9.969112e−03	NEG
1000	F2R	Stromal_score	1.576014e−13	0.4735627	4.182583e−09	POS	UTP14A	Stromal_score	4.071301e−10	−0.4081003	1.080483e−05	NEG
1001	STMN2	Stromal_score	1.600523e−06	0.3190183	4.247629e−02	POS	GIT2	Stromal_score	1.983424e−18	0.5483007	5.263808e−14	POS
1002	C16orf54	Stromal_score	1.599982e−08	0.3718818	4.246191e−04	POS	FARSA	Stromal_score	1.335487e−06	−0.3212931	3.544248e−02	NEG
1003	SKP2	Stromal_score	1.672254e−09	−0.3946762	4.437994e−05	NEG	C8orf76	Stromal_score	1.402952e−07	−0.3481675	3.723293e−03	NEG
1004	CHMP4C	Stromal_score	1.016394e−11	−0.4405951	2.697409e−07	NEG	PRIM2	Stromal_score	4.926994e−13	−0.4648895	1.307575e−08	NEG
1005	LARP6	Stromal_score	2.096012e−08	0.3690350	5.562607e−04	POS	GNA12	Stromal_score	1.702998e−08	0.3712265	4.519586e−04	POS
1006	CDK1	Stromal_score	2.066326e−13	−0.4715236	5.483823e−09	NEG	RBM28	Stromal_score	6.270361e−09	−0.3815495	1.664091e−04	NEG
1007	ANLN	Stromal_score	1.067327e−11	−0.4401864	2.832579e−07	NEG	REXO4	Stromal_score	8.113658e−10	−0.4016227	2.153284e−05	NEG
1008	RASL12	Stromal_score	4.513726e−22	0.5938535	1.197898e−17	POS	DPH3P1	Stromal_score	1.012241e−07	−0.3518587	2.686386e−03	NEG
1009	FKBP5	Stromal_score	1.804611e−11	0.4357618	4.789257e−07	POS	RAD1	Stromal_score	1.130439e−09	−0.3984571	3.000073e−05	NEG
1010	SLC22A18AS	Stromal_score	6.176074e−07	−0.3307813	1.639068e−02	NEG	METAP1	Stromal_score	1.053214e−08	−0.3762364	2.795125e−04	NEG
1011	PRC1	Stromal_score	1.455032e−13	−0.4741614	3.861511e−09	NEG	CCPG1	Stromal_score	5.701596e−07	0.3317467	1.513147e−02	POS
1012	NUF2	Stromal_score	2.478662e−14	−0.4871372	6.578121e−10	NEG	C21orf59	Stromal_score	7.814872e−15	−0.4953136	2.073989e−10	NEG
1013	ELOVL5	Stromal_score	5.553776e−09	0.3827792	1.473917e−04	POS	NDUFAF5	Stromal_score	6.146810e−09	−0.3817515	1.631302e−04	NEG
1014	GUCY1B3	Stromal_score	1.345971e−33	0.7024940	3.572072e−29	POS	HDAC6	Stromal_score	1.215320e−06	−0.3224706	3.225337e−02	NEG
1015	VAMP5	Stromal_score	1.466788e−18	0.5500671	3.892707e−14	POS	C9orf117	Stromal_score	7.227254e−09	−0.3801039	1.918041e−04	NEG
1016	LOXL1	Stromal_score	1.004527e−16	0.5243283	2.665913e−12	POS	AP1AR	Stromal_score	1.945591e−08	−0.3698228	5.163403e−04	NEG
1017	DL490908	Stromal_score	2.102604e−10	−0.4141798	5.580101e−06	NEG	DOM3Z	Stromal_score	1.025105e−08	−0.3765159	2.720527e−04	NEG
1018	ARHGAP15	Stromal_score	2.121447e−16	0.5195388	5.630109e−12	POS	LOC728377	Stromal_score	1.790333e−06	−0.3176006	4.751364e−02	NEG
1019	MEIS1	Stromal_score	1.858526e−16	0.5203920	4.932342e−12	POS	PPP1CA	Stromal_score	2.180702e−07	−0.3431033	5.787366e−03	NEG
1020	MARVELD3	Stromal_score	6.450820e−09	−0.3812613	1.711983e−04	NEG	TXNL4B	Stromal_score	8.487232e−09	−0.3784596	2.252427e−04	NEG
1021	NKX3−2	Stromal_score	1.013693e−10	0.4207537	2.690240e−06	POS	VTA1	Stromal_score	1.632409e−06	−0.3187692	4.332251e−02	NEG
1022	ADAMTS12	Stromal_score	1.744804e−06	0.3179271	4.630536e−02	POS	C14orf43	Stromal_score	4.154655e−07	0.3355371	1.102604e−02	POS
1023	MNDA	Stromal_score	1.125377e−12	0.4584480	2.986639e−08	POS	C1orf74	Stromal_score	2.378707e−11	−0.4334088	6.312851e−07	NEG
1024	NCF4	Stromal_score	4.698035e−08	0.3603579	1.246811e−03	POS	MEN1	Stromal_score	1.589504e−10	−0.4167174	4.218384e−06	NEG
1025	RFTN1	Stromal_score	1.461407e−27	0.6511481	3.878427e−23	POS	TERF2IP	Stromal_score	3.258502e−09	0.3881228	8.647738e−05	POS
1026	NUSAP1	Stromal_score	7.540358e−12	−0.4430790	2.001136e−07	NEG	NFXL1	Stromal_score	4.041640e−15	−0.4998884	1.072611e−10	NEG
1027	PHLDB2	Stromal_score	2.477820e−21	0.5851616	6.575888e−17	POS	SNHG11	Stromal_score	9.083636e−09	−0.3777620	2.410706e−04	NEG
1028	FAM111B	Stromal_score	1.927562e−11	−0.4352020	5.115558e−07	NEG	ZNF485	Stromal_score	1.771630e−10	−0.4157359	4.701728e−06	NEG
1029	SLC37A1	Stromal_score	1.243645e−06	−0.3221833	3.300511e−02	NEG	PIGA	Stromal_score	6.533511e−11	−0.4246440	1.733929e−06	NEG
1030	SOX18	Stromal_score	2.150558e−15	0.5042028	5.707365e−11	POS	FAM199X	Stromal_score	1.157200e−06	−0.3230806	3.071093e−02	NEG
1031	LLGL2	Stromal_score	1.193090e−12	−0.4579872	3.166341e−08	NEG	EXOSC9	Stromal_score	1.106591e−06	−0.3236361	2.936782e−02	NEG
1032	TNFAIP6	Stromal_score	6.929480e−08	0.3560859	1.839015e−03	POS	DCUN1D2	Stromal_score	1.432696e−06	−0.3204124	3.802231e−02	NEG
1033	ZNF853	Stromal_score	1.507168e−18	0.5499085	3.999874e−14	POS	HYAL2	Stromal_score	3.177894e−07	0.3387075	8.433813e−03	POS
1034	UBE2T	Stromal_score	5.429219e−17	−0.5282145	1.440860e−12	NEG	TSSK6	Stromal_score	4.446637e−14	−0.4829128	1.180093e−09	NEG
1035	METTL24	Stromal_score	2.055970e−14	0.4884765	5.456338e−10	POS	LAS1L	Stromal_score	1.410098e−09	−0.3963285	3.742260e−05	NEG
1036	CCR5	Stromal_score	1.087787e−07	0.3510488	2.886877e−03	POS	AF086285	Stromal_score	8.381409e−07	−0.3270631	2.224342e−02	NEG
1037	LAMA4	Stromal_score	5.931355e−30	0.6727519	1.574122e−25	POS	ALG6	Stromal_score	1.172732e−10	−0.4194518	3.112313e−06	NEG
1038	GPSM3	Stromal_score	6.517811e−10	0.4036951	1.729762e−05	POS	SEPSECS	Stromal_score	1.306131e−07	−0.3489802	3.466340e−03	NEG
1039	TLN1	Stromal_score	4.933283e−20	0.5692351	1.309244e−15	POS	ABCB6	Stromal_score	2.695630e−07	−0.3406371	7.153932e−03	NEG
1040	CDS1	Stromal_score	2.646899e−09	−0.3901804	7.024605e−05	NEG	DAGLB	Stromal_score	2.182452e−07	−0.3430940	5.792010e−03	NEG
1041	CDH5	Stromal_score	4.946393e−21	0.5815573	1.312723e−16	POS	POLR3B	Stromal_score	8.466128e−07	−0.3269397	2.246826e−02	NEG
1042	GAPT	Stromal_score	7.630660e−12	0.4429803	2.025101e−07	POS	FTO	Stromal_score	4.497989e−16	0.5146467	1.193721e−11	POS
1043	ARHGEF16	Stromal_score	2.269160e−15	−0.5038380	6.022123e−11	NEG	ZBTB9	Stromal_score	2.842665e−09	−0.3894757	7.544149e−05	NEG
1044	SPON2	Stromal_score	2.152690e−11	0.4342614	5.713024e−07	POS	DCLRE1B	Stromal_score	1.708344e−06	−0.3181945	4.533773e−02	NEG
1045	ETS1	Stromal_score	3.916520e−18	0.5442794	1.039405e−13	POS	NOL12	Stromal_score	2.638956e−11	−0.4325197	7.003526e−07	NEG
1046	EGR2	Stromal_score	6.089294e−08	0.3575136	1.616038e−03	POS	C1orf27	Stromal_score	7.723507e−10	−0.4020902	2.049741e−05	NEG
1047	GAS6	Stromal_score	2.773596e−09	0.3897188	7.360846e−05	POS	ZNF620	Stromal_score	1.142269e−07	−0.3504976	3.031468e−03	NEG
1048	BICC1	Stromal_score	2.515010e−23	0.6080157	6.674584e−19	POS	C3orf33	Stromal_score	1.478272e−06	−0.3200190	3.923187e−02	NEG
1049	FAM108C1	Stromal_score	1.963590e−09	−0.3931115	5.211171e−05	NEG	C9orf86	Stromal_score	5.417563e−07	−0.3323621	1.437767e−02	NEG
1050	GLB1L2	Stromal_score	2.772615e−08	−0.3660564	7.358243e−04	NEG	CENPJ	Stromal_score	9.280683e−09	−0.3775412	2.463000e−04	NEG
1051	SMO	Stromal_score	5.322848e−12	0.4459510	1.412631e−07	POS	POLR1B	Stromal_score	3.185850e−10	−0.4103709	8.454926e−06	NEG
1052	GPBAR1	Stromal_score	8.614912e−13	0.4605467	2.286312e−08	POS	GLG1	Stromal_score	4.617188e−10	0.4069287	1.225356e−05	POS
1053	E2F1	Stromal_score	2.589198e−13	−0.4698152	6.871472e−09	NEG	C14orf142	Stromal_score	1.218244e−06	−0.3224406	3.233097e−02	NEG
1054	NR2F2	Stromal_score	3.405405e−11	0.4303250	9.037603e−07	POS	ZC2HC1C	Stromal_score	6.020955e−08	−0.3576380	1.597901e−03	NEG
1055	DST	Stromal_score	1.041918e−07	0.3515338	2.765146e−03	POS	HECA	Stromal_score	2.022777e−08	0.3694115	5.368248e−04	POS
1056	IL20RA	Stromal_score	1.805598e−09	−0.3939298	4.791876e−05	NEG	POLR3A	Stromal_score	1.078678e−07	−0.3511435	2.862705e−03	NEG
1057	HJURP	Stromal_score	1.815953e−17	−0.5350105	4.819358e−13	NEG	RANGAP1	Stromal_score	1.384890e−06	−0.3208381	3.675361e−02	NEG
1058	KIF20A	Stromal_score	2.843696e−08	−0.3657854	7.546884e−04	NEG	SH3YL1	Stromal_score	1.039446e−10	−0.4205299	2.758586e−06	NEG
1059	AK056897	Stromal_score	1.307031e−11	0.4384871	3.468729e−07	POS	TMEM209	Stromal_score	1.735915e−06	−0.3179918	4.606944e−02	NEG
1060	GLIS2	Stromal_score	1.080286e−20	0.5774301	2.866970e−16	POS	VIT	Stromal_score	1.572060e−09	0.3952759	4.172090e−05	POS
1061	AKR7L	Stromal_score	6.744433e−09	−0.3808085	1.789905e−04	NEG	ZNF195	Stromal_score	1.491441e−10	−0.4172921	3.958136e−06	NEG
1062	WDFY4	Stromal_score	1.699044e−09	0.3945217	4.509092e−05	POS	ATN1	Stromal_score	1.370212e−09	0.3966057	3.636405e−05	POS
1063	AGTR1	Stromal_score	1.904444e−13	0.4721392	5.054205e−09	POS	DOCK4	Stromal_score	2.276440e−11	0.4337844	6.041443e−07	POS
1064	COL5A3	Stromal_score	1.352231e−06	0.3211371	3.588686e−02	POS	TARBP2	Stromal_score	1.257114e−07	−0.3494140	3.336254e−03	NEG
1065	VCL	Stromal_score	4.718341e−11	0.4274951	1.252201e−06	POS	PSMC3IP	Stromal_score	3.570239e−12	−0.4492118	9.475056e−08	NEG
1066	SLFN11	Stromal_score	1.848117e−06	0.3171974	4.904717e−02	POS	MTCH2	Stromal_score	1.082113e−08	−0.3759564	2.871821e−04	NEG
1067	CDC45	Stromal_score	1.790657e−13	−0.4726032	4.752225e−09	NEG	GCSH	Stromal_score	3.093207e−08	−0.3648832	8.209062e−04	NEG
1068	43349	Stromal_score	1.065214e−11	0.4402029	2.826972e−07	POS	CDK17	Stromal_score	8.820666e−13	0.4603619	2.340917e−08	POS
1069	PDZRN4	Stromal_score	1.556511e−14	0.4904593	4.130825e−10	POS	PICK1	Stromal_score	3.941053e−10	−0.4084023	1.045916e−05	NEG
1070	DAB2	Stromal_score	1.767885e−24	0.6204412	4.691790e−20	POS	TPM3	Stromal_score	4.213077e−07	−0.3353710	1.118109e−02	NEG
1071	PARD6B	Stromal_score	7.298032e−10	−0.4026270	1.936825e−05	NEG	C11orf68	Stromal_score	2.996309e−08	0.3652249	7.951905e−04	POS
1072	ASPSCR1	Stromal_score	7.001869e−07	−0.3292590	1.858226e−02	NEG	INTS2	Stromal_score	8.993822e−09	−0.3778641	2.386870e−04	NEG
1073	LRFN4	Stromal_score	1.451610e−07	−0.3477792	3.852428e−03	NEG	REEP5	Stromal_score	3.423220e−07	0.3378314	9.084883e−03	POS
1074	FAM64A	Stromal_score	2.027642e−12	−0.4537726	5.381160e−08	NEG	SNRNP48	Stromal_score	2.600626e−10	−0.4122373	6.901801e−06	NEG
1075	RSPH1	Stromal_score	5.613462e−09	−0.3826711	1.489757e−04	NEG	CUL4A	Stromal_score	1.729628e−07	−0.3457752	4.590260e−03	NEG
1076	HNF1A	Stromal_score	5.134460e−13	−0.4645710	1.362634e−08	NEG	JAKMIP2	Stromal_score	1.881514e−08	0.3701764	4.993350e−04	POS
1077	MMRN2	Stromal_score	7.368228e−25	0.6244188	1.955454e−20	POS	AX746755	Stromal_score	8.968127e−10	0.4006705	2.380051e−05	POS
1078	SLC24A3	Stromal_score	5.377041e−19	0.5558674	1.427013e−14	POS	MRVI1−AS1	Stromal_score	7.758824e−16	0.5110480	2.059114e−11	POS
1079	JAK3	Stromal_score	4.806509e−09	0.3842369	1.275599e−04	POS	TRMT11	Stromal_score	1.675473e−08	−0.3713977	4.446539e−04	NEG
1080	CNN3	Stromal_score	1.275614e−18	0.5508810	3.385353e−14	POS	NFS1	Stromal_score	3.142267e−09	−0.3884833	8.339264e−05	NEG

	gene	feature	p.value	estimate	adj.p.value	COR.ID	gene	feature	p.value	estimate	adj.p.value	COR.ID
1081	LDLRAD2	Stromal_score	7.820068e−17	0.5259158	2.075368e−12	POS	ZC3H8	Stromal_score	8.868497e−14	−0.4778468	2.353610e−09	NEG
1082	ZWINT	Stromal_score	4.301837e−13	−0.4659349	1.141664e−08	NEG	MRPS5	Stromal_score	6.778818e−13	−0.4624171	1.799031e−08	NEG
1083	CPQ	Stromal_score	2.067112e−14	0.4884379	5.485909e−10	POS	FAM72D	Stromal_score	6.078383e−15	−0.4970652	1.613142e−10	NEG
1084	SDC2	Stromal_score	3.696099e−24	0.6170447	9.809077e−20	POS	CREM	Stromal_score	2.232186e−08	0.3683674	5.923999e−04	POS
1085	ZNF488	Stromal_score	9.175343e−08	−0.3529604	2.435044e−03	NEG	RHOG	Stromal_score	2.549597e−10	0.4124189	6.766375e−06	POS
1086	IL18BP	Stromal_score	2.334288e−08	0.3678922	6.194966e−04	POS	SECISBP2L	Stromal_score	3.857392e−11	0.4292466	1.023713e−06	POS
1087	SPIRE2	Stromal_score	2.177084e−15	−0.5041195	5.777762e−11	NEG	CAPN10	Stromal_score	1.274117e−14	−0.4918777	3.381380e−10	NEG
1088	CDCA2	Stromal_score	4.573310e−09	−0.3847369	1.213711e−04	NEG	PRPF3	Stromal_score	6.576520e−07	−0.3300203	1.745343e−02	NEG
1089	MAN1C1	Stromal_score	3.759211e−21	0.5829936	9.976571e−17	POS	WRAP53	Stromal_score	3.092833e−08	−0.3648845	8.208070e−04	NEG
1090	TUSC3	Stromal_score	1.647813e−10	0.4163918	4.373132e−06	POS	RNF6	Stromal_score	1.161141e−06	−0.3230383	3.081553e−02	NEG
1091	KCNE4	Stromal_score	4.216508e−23	0.6055324	1.119019e−18	POS	DDX28	Stromal_score	4.927378e−07	−0.3335007	1.307677e−02	NEG
1092	LOC100507424	Stromal_score	2.796025e−09	−0.3896392	7.420370e−05	NEG	DDX52	Stromal_score	1.165956e−06	−0.3229868	3.094332e−02	NEG
1093	KIRREL	Stromal_score	1.076347e−27	0.6523958	2.856518e−23	POS	LOC100288637	Stromal_score	8.181647e−11	−0.4226578	2.171327e−06	NEG
1094	CRISPLD1	Stromal_score	2.655195e−15	0.5027681	7.046623e−11	POS	PRPF4	Stromal_score	1.171985e−07	−0.3502075	3.110331e−03	NEG
1095	SATB1	Stromal_score	2.559648e−07	0.3412413	6.793050e−03	POS	PPCDC	Stromal_score	2.959246e−08	−0.3653585	7.853542e−04	NEG
1096	LAMB2	Stromal_score	1.547537e−12	0.4559272	4.107008e−08	POS	MAPK11	Stromal_score	5.797332e−07	0.3315459	1.538554e−02	POS
1097	CENPA	Stromal_score	2.248710e−13	−0.4708841	5.967852e−09	NEG	SNHG10	Stromal_score	6.861587e−16	−0.5118629	1.820997e−11	NEG
1098	RHOJ	Stromal_score	2.030943e−25	0.6301734	5.389920e−21	POS	ZSWIM3	Stromal_score	1.291574e−06	−0.3217112	3.427708e−02	NEG
1099	HLF	Stromal_score	3.213058e−08	0.3644745	8.527135e−04	POS	DFFB	Stromal_score	2.960931e−09	−0.3890724	7.858015e−05	NEG
1100	FAM127C	Stromal_score	2.145870e−18	0.5478382	5.694923e−14	POS	LOC100506668	Stromal_score	3.074328e−08	−0.3649489	8.158960e−04	NEG
1101	TROAP	Stromal_score	5.702554e−14	−0.4810969	1.513401e−09	NEG	BC107568	Stromal_score	8.701029e−11	−0.4221121	2.309166e−06	NEG
1102	UHRF1	Stromal_score	1.025338e−08	−0.3765135	2.721146e−04	NEG	TMEM182	Stromal_score	3.516943e−13	−0.4674804	9.333616e−09	NEG
1103	DLGAP5	Stromal_score	7.395497e−11	−0.4235514	1.962691e−06	NEG	NAA40	Stromal_score	2.442124e−12	−0.4522807	6.481152e−08	NEG
1104	NFIX	Stromal_score	7.330246e−11	0.4236297	1.945374e−06	POS	C1orf112	Stromal_score	6.999417e−17	−0.5266161	1.857575e−12	NEG
1105	RAB17	Stromal_score	6.318745e−14	−0.4803448	1.676932e−09	NEG	NCOA1	Stromal_score	1.323062e−10	0.4183702	3.511275e−06	POS
1106	SRPX2	Stromal_score	3.584338e−17	0.5308091	9.512474e−13	POS	KIAA1841	Stromal_score	4.216441e−09	−0.3855519	1.119001e−04	NEG
1107	GZMM	Stromal_score	5.293332e−07	0.3326410	1.404797e−02	POS	MIR1178	Stromal_score	4.486240e−07	−0.3346222	1.190603e−02	NEG
1108	PLAT	Stromal_score	1.308999e−10	0.4184662	3.473952e−06	POS	C2orf48	Stromal_score	5.959474e−07	−0.3312129	1.581585e−02	NEG
1109	ZNF423	Stromal_score	7.387358e−23	0.6028127	1.960531e−18	POS	LOC100132891	Stromal_score	4.799533e−19	0.5565170	1.273748e−14	POS
1110	TNFRSF1B	Stromal_score	2.280626e−07	0.3425838	6.052553e−03	POS	HBT8	Stromal_score	3.884716e−10	0.4085360	1.030965e−05	POS
1111	ITGA1	Stromal_score	2.103835e−17	0.5341062	5.583369e−13	POS	PAXIP1	Stromal_score	4.753341e−07	−0.3339312	1.261489e−02	NEG
1112	BMP3	Stromal_score	1.538099e−07	0.3471189	4.081960e−03	POS	DBF4B	Stromal_score	3.704435e−10	−0.4089766	9.831200e−06	NEG
1113	DTX3	Stromal_score	3.659074e−10	0.4090908	9.710817e−06	POS	LOC100130950	Stromal_score	4.828064e−08	0.3600599	1.281320e−03	POS
1114	CNTN1	Stromal_score	7.905511e−12	0.4426869	2.098044e−07	POS	MAGEE1	Stromal_score	5.704178e−09	0.3825089	1.513832e−04	POS
1115	GINS1	Stromal_score	1.679225e−14	−0.4899199	4.456495e−10	NEG	SYNGAP1	Stromal_score	1.423514e−10	0.4177120	3.777864e−06	POS
1116	TPRN	Stromal_score	2.796115e−11	−0.4320232	7.420610e−07	NEG	FAM124B	Stromal_score	5.008712e−15	0.4984075	1.329262e−10	POS
1117	PCNA	Stromal_score	1.186013e−07	−0.3500730	3.147559e−03	NEG	SIK3	Stromal_score	9.324752e−08	0.3527795	2.474696e−03	POS
1118	RBP7	Stromal_score	2.019546e−09	0.3928369	5.359674e−05	POS	SYPL2	Stromal_score	1.029567e−07	0.3516680	2.732368e−03	POS
1119	HVCN1	Stromal_score	3.765462e−13	0.4669575	9.993159e−09	POS	UMPS	Stromal_score	2.029381e−13	−0.4716599	5.385774e−09	NEG
1120	ITGAX	Stromal_score	1.483257e−07	0.3475333	3.936415e−03	POS	EBF2	Stromal_score	1.170816e−10	0.4194665	3.107227e−06	POS
1121	PRR7	Stromal_score	7.804261e−07	−0.3279363	2.071173e−02	NEG	GMEB2	Stromal_score	1.168841e−06	−0.3229561	3.101988e−02	NEG
1122	AK123675	Stromal_score	4.245163e−21	0.5823583	1.126624e−16	POS	ARMC6	Stromal_score	1.515552e−08	−0.3724500	4.022123e−04	NEG
1123	SDC3	Stromal_score	8.956286e−15	0.4943592	2.376909e−10	POS	NOVA2	Stromal_score	1.054561e−10	0.4204011	2.798700e−06	POS
1124	BSPRY	Stromal_score	8.017797e−15	−0.4951344	2.127843e−10	NEG	FAM89B	Stromal_score	1.037688e−09	0.3992775	2.753921e−05	POS
1125	ST14	Stromal_score	4.728936e−11	−0.4274755	1.255012e−06	NEG	PNPT1	Stromal_score	6.065962e−13	−0.4632803	1.609846e−08	NEG
1126	RBFOX3	Stromal_score	5.860316e−07	0.3314155	1.555269e−02	POS	PHACTR1	Stromal_score	2.224542e−07	0.3428726	5.903711e−03	POS
1127	GIMAP6	Stromal_score	1.820658e−23	0.6095573	4.831845e−19	POS	PDCD2	Stromal_score	1.062548e−07	−0.3513132	2.819896e−03	NEG
1128	LBH	Stromal_score	8.569207e−14	0.4781007	2.274182e−09	POS	BCMS	Stromal_score	7.460610e−17	−0.5262132	1.979971e−12	NEG
1129	PPM1H	Stromal_score	1.005516e−12	−0.4593342	2.668539e−08	NEG	AK056973	Stromal_score	6.494620e−09	−0.3811925	1.723607e−04	NEG
1130	CCR4	Stromal_score	5.105144e−09	0.3836297	1.354854e−04	POS	CACNA1C	Stromal_score	3.844394e−10	0.4086328	1.020264e−05	POS
1131	DDR1	Stromal_score	3.726036e−08	−0.3628764	9.888527e−04	NEG	HCFC2	Stromal_score	6.476218e−10	0.4037554	1.718724e−05	POS
1132	KDM5D	Stromal_score	1.658765e−06	0.3185669	4.402196e−02	POS	TMEM140	Stromal_score	3.227109e−08	0.3644275	8.564425e−04	POS
1133	EDNRB	Stromal_score	2.132528e−10	0.4140511	5.659516e−06	POS	C12orf52	Stromal_score	1.560782e−06	−0.3193353	4.142159e−02	NEG
1134	SLC2A3	Stromal_score	1.682990e−10	0.4162007	4.466487e−06	POS	GFM1	Stromal_score	1.838302e−06	−0.3172651	4.878669e−02	NEG
1135	SMAGP	Stromal_score	1.310594e−08	−0.3739675	3.478186e−04	NEG	CSNK2A1	Stromal_score	1.210450e−08	−0.3747944	3.212414e−04	NEG
1136	C1orf38	Stromal_score	1.421531e−11	0.4377800	3.772600e−07	POS	GREB1	Stromal_score	3.984299e−12	0.4483194	1.057393e−07	POS
1137	CDC42BPG	Stromal_score	3.613681e−14	−0.4844188	9.590347e−10	NEG	CLECL1	Stromal_score	1.327894e−06	0.3213644	3.524097e−02	POS
1138	BC073932	Stromal_score	1.369681e−21	0.5882172	3.634996e−17	POS	TRAK2	Stromal_score	1.525097e−08	0.3723843	4.047455e−04	POS
1139	DACT1	Stromal_score	1.350213e−19	0.5636740	3.583329e−15	POS	SNORA68	Stromal_score	3.482472e−08	−0.3636068	9.242131e−04	NEG
1140	FCGR2B	Stromal_score	3.240631e−15	0.5014058	8.600312e−11	POS	PA2G4P4	Stromal_score	2.321011e−14	−0.4876086	6.159732e−10	NEG
1141	PTAFR	Stromal_score	1.259768e−07	0.3493901	3.343299e−03	POS	FOXD3	Stromal_score	1.773423e−06	0.3177209	4.706486e−02	POS
1142	RARA	Stromal_score	9.300379e−07	0.3257848	2.468227e−02	POS	EAF1	Stromal_score	1.171741e−07	−0.3502099	3.109685e−03	NEG
1143	CYGB	Stromal_score	2.177134e−16	0.5193714	5.777895e−12	POS	PIGG	Stromal_score	7.077172e−07	−0.3291289	1.878211e−02	NEG
1144	BUB1B	Stromal_score	3.187034e−12	−0.4501326	8.458070e−08	NEG	AK026965	Stromal_score	1.993443e−07	−0.3441414	5.290399e−03	NEG
1145	HIST1H2BJ	Stromal_score	1.004296e−07	−0.3519472	2.665302e−03	NEG	PTGES3	Stromal_score	1.550759e−06	−0.3194165	4.115559e−02	NEG
1146	SPG20	Stromal_score	1.260408e−22	0.6001972	3.344998e−18	POS	C2orf47	Stromal_score	8.284083e−12	−0.4422986	2.198513e−07	NEG
1147	DOK2	Stromal_score	7.105667e−10	0.4028796	1.885773e−05	POS	GAB3	Stromal_score	1.571242e−12	0.4558064	4.169919e−08	POS
1148	TMEM141	Stromal_score	7.246421e−07	−0.3288411	1.923128e−02	NEG	METTL21A	Stromal_score	6.433378e−09	−0.3812888	1.707354e−04	NEG
1149	GPX8	Stromal_score	1.895190e−17	0.5347483	5.029644e−13	POS	ADAMTSL1	Stromal_score	3.364426e−11	0.4304296	8.928851e−07	POS
1150	MSMO1	Stromal_score	4.540851e−07	−0.3344778	1.205096e−02	NEG	TIMM44	Stromal_score	1.953243e−07	−0.3443765	5.183712e−03	NEG
1151	LPHN2	Stromal_score	1.294049e−15	0.5076325	3.434278e−11	POS	LOC100287015	Stromal_score	6.901822e−08	−0.3561302	1.831675e−03	NEG
1152	NRN1	Stromal_score	6.140274e−08	0.3574218	1.629567e−03	POS	PIEZO2	Stromal_score	5.124134e−11	0.4267749	1.359894e−06	POS
1153	CEP72	Stromal_score	6.704757e−14	−0.4799093	1.779375e−09	NEG	C12orf73	Stromal_score	8.353388e−09	−0.3786226	2.216906e−04	NEG
1154	ZBTB16	Stromal_score	1.030526e−11	0.4404797	2.734912e−07	POS	MEX3B	Stromal_score	1.461235e−08	0.3728319	3.877973e−04	POS
1155	ARMCX2	Stromal_score	5.233400e−20	0.5689118	1.388892e−15	POS	NR6A1	Stromal_score	6.312415e−11	−0.4249468	1.675252e−06	NEG
1156	CTSO	Stromal_score	6.027209e−22	0.5923958	1.599561e−17	POS	ACVR1	Stromal_score	3.109817e−08	0.3648256	8.253144e−04	POS
1157	HOOK1	Stromal_score	2.370458e−15	−0.5035410	6.290959e−11	NEG	TRMU	Stromal_score	4.824246e−12	−0.4467573	1.280307e−07	NEG
1158	IL6ST	Stromal_score	2.404469e−24	0.6190298	6.381221e−20	POS	SSH1	Stromal_score	3.354585e−16	0.5165653	8.902732e−12	POS
1159	SHISA4	Stromal_score	1.599784e−07	0.3466694	4.245667e−03	POS	NUP35	Stromal_score	1.892068e−10	−0.4151393	5.021360e−06	NEG
1160	PAK6	Stromal_score	3.579563e−09	−0.3871879	9.499801e−05	NEG	MRPL9	Stromal_score	3.286291e−07	−0.3383127	8.721488e−03	NEG
1161	SESN3	Stromal_score	9.596777e−12	0.4410743	2.546889e−07	POS	C1orf43	Stromal_score	4.403309e−07	−0.3348448	1.168594e−02	NEG
1162	SLC16A4	Stromal_score	2.847396e−09	0.3894593	7.556704e−05	POS	MUSK	Stromal_score	8.136960e−09	0.3788917	2.159468e−04	POS
1163	KCNJ8	Stromal_score	1.335654e−25	0.6320192	3.544693e−21	POS	NUPL2	Stromal_score	2.672962e−07	−0.3407358	7.093774e−03	NEG
1164	LPXN	Stromal_score	6.016484e−07	0.3310978	1.596715e−02	POS	NEFH	Stromal_score	2.001597e−09	0.3929242	5.312037e−05	POS
1165	HAVCR2	Stromal_score	3.595747e−07	0.3372506	9.542753e−03	POS	LOC440461	Stromal_score	3.038021e−07	0.3392365	8.062604e−03	POS
1166	RERGL	Stromal_score	2.381451e−07	0.3420814	6.320133e−03	POS	MTIF2	Stromal_score	7.968893e−14	−0.4786371	2.114864e−09	NEG
1167	CDON	Stromal_score	6.120254e−12	0.4448030	1.624254e−07	POS	C11orf63	Stromal_score	2.550079e−08	0.3669504	6.767654e−04	POS
1168	A4GALT	Stromal_score	3.991198e−12	0.4483053	1.059224e−07	POS	AX747269	Stromal_score	1.464905e−12	−0.4563629	3.887712e−08	NEG
1169	CLEC14A	Stromal_score	2.935703e−23	0.6072747	7.791061e−19	POS	CISD2	Stromal_score	2.007512e−08	−0.3694916	5.327735e−04	NEG
1170	SNAI2	Stromal_score	2.681809e−16	0.5180214	7.117252e−12	POS	AX747493	Stromal_score	1.240469e−12	−0.4576796	3.292081e−08	NEG
1171	EBP	Stromal_score	2.238774e−13	−0.4709176	5.941484e−09	NEG	PSPC1	Stromal_score	4.300277e−09	−0.3853546	1.141251e−04	NEG
1172	C1QTNF5	Stromal_score	9.852374e−15	0.4936899	2.614722e−10	POS	LOC727677	Stromal_score	1.508747e−06	−0.3197623	4.004063e−02	NEG
1173	SPINT1	Stromal_score	1.190641e−08	−0.3749658	3.159842e−04	NEG	LINC00478	Stromal_score	4.057094e−22	0.5943892	1.076712e−17	POS
1174	HSPB2	Stromal_score	2.638685e−18	0.5466201	7.002807e−14	POS	PSMA5	Stromal_score	1.696326e−06	−0.3182838	4.501880e−02	NEG
1175	SOX10	Stromal_score	1.041231e−12	0.4590598	2.763322e−08	POS	TMEM177	Stromal_score	5.860487e−15	−0.4973188	1.555315e−10	NEG
1176	GLT8D2	Stromal_score	6.556650e−30	0.6723744	1.740069e−25	POS	ZSCAN16	Stromal_score	5.442716e−13	−0.4641203	1.444442e−08	NEG
1177	EPB41L3	Stromal_score	9.169679e−20	0.5658236	2.433541e−15	POS	PCDH9	Stromal_score	5.505885e−12	0.4456734	1.461207e−07	POS
1178	CENPM	Stromal_score	3.022412e−12	−0.4505617	8.021180e−08	NEG	FBXO22	Stromal_score	7.329798e−10	−0.4025859	1.945255e−05	NEG
1179	CD109	Stromal_score	9.287574e−15	0.4941044	2.464829e−10	POS	CCDC42B	Stromal_score	1.734610e−07	−0.3457422	4.603483e−03	NEG
1180	FCN1	Stromal_score	9.020916e−12	0.4415900	2.394061e−07	POS	ZNF366	Stromal_score	6.225613e−11	0.4250685	1.652215e−06	POS
1181	ZFPM1	Stromal_score	1.010552e−06	−0.3247605	2.681903e−02	NEG	THSD1	Stromal_score	9.619800e−13	0.4596819	2.552999e−08	POS
1182	SPN	Stromal_score	8.637091e−09	0.3782799	2.292198e−04	POS	AL832007	Stromal_score	1.469749e−12	0.4563367	3.900566e−08	POS
1183	FAM117A	Stromal_score	2.782510e−09	0.3896871	7.384502e−05	POS	C1orf216	Stromal_score	2.540276e−07	0.3413298	6.741638e−03	POS
1184	NLGN2	Stromal_score	1.837223e−25	0.6306161	4.875805e−21	POS	SNTB2	Stromal_score	2.995463e−11	0.4314310	7.949658e−07	POS
1185	GRIK5	Stromal_score	1.009961e−15	0.5092923	2.680336e−11	POS	DND1	Stromal_score	3.707092e−08	−0.3629316	9.838251e−04	NEG
1186	NOXA1	Stromal_score	3.811438e−10	−0.4087126	1.011518e−05	NEG	MRPL37	Stromal_score	4.124228e−08	−0.3617759	1.094529e−03	NEG
1187	TGFB1	Stromal_score	1.165080e−29	0.6701982	3.092005e−25	POS	LARP1B	Stromal_score	1.777237e−11	−0.4358915	4.716609e−07	NEG
1188	PABPC1L	Stromal_score	4.673244e−08	−0.3604157	1.240232e−03	NEG	LIMS1	Stromal_score	9.895190e−10	0.3997320	2.626085e−05	POS
1189	GNB4	Stromal_score	2.675417e−34	0.7078266	7.100290e−30	POS	43350	Stromal_score	2.482137e−11	0.4330447	6.587343e−07	POS
1190	KIF18B	Stromal_score	3.614167e−11	−0.4298106	9.591639e−07	NEG	FANCB	Stromal_score	5.022823e−13	−0.4647408	1.333007e−08	NEG
1191	TMTC1	Stromal_score	1.588371e−20	0.5753717	4.215377e−16	POS	ZNF695	Stromal_score	3.403430e−14	−0.4848526	9.032364e−10	NEG
1192	PBX3	Stromal_score	3.407553e−16	0.5164631	9.043306e−12	POS	CNNM3	Stromal_score	3.705728e−07	−0.3368941	9.834633e−03	NEG
1193	MEOX1	Stromal_score	5.211114e−17	0.5284717	1.382977e−12	POS	PCGF1	Stromal_score	1.583837e−06	−0.3191505	4.203345e−02	NEG
1194	EFHD1	Stromal_score	6.851124e−14	0.4797505	1.818220e−09	POS	SLC39A13	Stromal_score	6.709475e−11	0.4244100	1.780628e−06	POS
1195	IDH1	Stromal_score	3.929846e−08	−0.3622997	1.042942e−03	NEG	BHLHE22	Stromal_score	3.350026e−25	0.6279528	8.890633e−21	POS
1196	PDE4B	Stromal_score	1.638341e−15	0.5060444	4.347994e−11	POS	PAIP2	Stromal_score	1.570082e−11	0.4369409	4.166840e−07	POS
1197	NACC2	Stromal_score	1.520419e−06	0.3196653	4.035039e−02	POS	TRIT1	Stromal_score	3.656076e−08	−0.3630814	9.702860e−04	NEG
1198	PPAP2A	Stromal_score	3.870696e−17	0.5303305	1.027244e−12	POS	ZNF765	Stromal_score	8.090842e−07	−0.3274952	2.147229e−02	NEG
1199	SYT11	Stromal_score	3.278388e−25	0.6280491	8.700513e−21	POS	EXOSC7	Stromal_score	1.643022e−09	−0.3948475	4.360417e−05	NEG
1200	CENPV	Stromal_score	4.401013e−11	−0.4281015	1.167985e−06	NEG	LIN54	Stromal_score	1.041414e−06	−0.3243884	2.763808e−02	NEG

	gene	feature	p.value	estimate	adj.p.value	COR.ID	gene	feature	p.value	estimate	adj.p.value	COR.ID
1201	EPAS1	Stromal_score	2.527511e−19	0.5601574	6.707763e−15	POS	ZBED4	Stromal_score	9.555934e−08	−0.3525051	2.536049e−03	NEG
1202	RHPN1	Stromal_score	3.979619e−11	−0.4289760	1.056151e−06	NEG	TRMT2A	Stromal_score	1.288539e−07	−0.3491341	3.419654e−03	NEG
1203	BIN2	Stromal_score	4.046659e−08	0.3619820	1.073943e−03	POS	STX18	Stromal_score	7.115798e−07	−0.3290626	1.888462e−02	NEG
1204	DTL	Stromal_score	1.876333e−13	−0.4722513	4.979600e−09	NEG	WARS2	Stromal_score	1.785080e−06	−0.3176379	4.737425e−02	NEG
1205	LAX1	Stromal_score	9.692491e−07	0.3252758	2.572290e−02	POS	NOD1	Stromal_score	7.692038e−09	0.3794672	2.041390e−04	POS
1206	C19orf48	Stromal_score	2.155528e−07	−0.3432378	5.720556e−03	NEG	KLHDC1	Stromal_score	3.138891e−07	0.3388528	8.330304e−03	POS
1207	APOLD1	Stromal_score	9.425471e−13	0.4598420	2.501426e−08	POS	ADD2	Stromal_score	4.218089e−07	0.3353568	1.119439e−02	POS
1208	CTSL1	Stromal_score	6.785420e−15	0.4962994	1.800783e−10	POS	CNKSR2	Stromal_score	2.836950e−13	0.4691203	7.528982e−09	POS
1209	FOXP2	Stromal_score	7.270268e−18	0.5405759	1.929456e−13	POS	LRCH3	Stromal_score	4.236418e−07	0.3353052	1.124303e−02	POS
1210	SLFN5	Stromal_score	6.028567e−09	0.3819485	1.599922e−04	POS	DNAJA3	Stromal_score	1.571030e−07	−0.3468768	4.169355e−03	NEG
1211	TUBB6	Stromal_score	1.846572e−21	0.5866813	4.900618e−17	POS	PDP2	Stromal_score	2.012267e−10	−0.4145796	5.340356e−06	NEG
1212	SGCD	Stromal_score	1.463456e−21	0.5878774	3.883866e−17	POS	MIR1292	Stromal_score	5.224715e−10	−0.4057732	1.386587e−05	NEG
1213	ESPL1	Stromal_score	2.465447e−11	−0.4331024	6.543050e−07	NEG	BTBD19	Stromal_score	1.873615e−10	0.4152283	4.972387e−06	POS
1214	MAGIX	Stromal_score	7.453612e−09	−0.3797890	1.978114e−04	NEG	EDA2R	Stromal_score	5.017581e−08	0.3596390	1.331616e−03	POS
1215	PILRA	Stromal_score	2.351132e−08	0.3678157	6.239668e−04	POS	NAGK	Stromal_score	8.541995e−13	0.4606132	2.266960e−08	POS
1216	EPB41L2	Stromal_score	8.672562e−08	0.3535905	2.301611e−03	POS	NEK4	Stromal_score	3.511860e−08	−0.3635161	9.320125e−04	NEG
1217	FASN	Stromal_score	1.877544e−07	−0.3448320	4.982814e−03	NEG	ANKRD54	Stromal_score	7.923407e−07	−0.3277511	2.102793e−02	NEG
1218	PCSK6	Stromal_score	1.502180e−08	−0.3725428	3.986637e−04	NEG	LUZP1	Stromal_score	1.234813e−07	0.3496168	3.277070e−03	POS
1219	CD84	Stromal_score	3.051308e−09	0.3887746	8.097866e−05	POS	C1orf109	Stromal_score	1.640019e−12	−0.4554657	4.352446e−08	NEG
1220	FAM107A	Stromal_score	1.753001e−17	0.5352269	4.652289e−13	POS	ZNF33B	Stromal_score	9.969600e−09	−0.3768033	2.645832e−04	NEG
1221	TMEM173	Stromal_score	6.879829e−13	0.4623020	1.825838e−08	POS	PALB2	Stromal_score	3.925166e−08	−0.3623126	1.041700e−03	NEG
1222	TONSL	Stromal_score	1.019282e−12	−0.4592274	2.705073e−08	NEG	CIAPIN1	Stromal_score	7.423827e−07	−0.3285464	1.970209e−02	NEG
1223	NFIA	Stromal_score	9.627696e−14	0.4772384	2.555094e−09	POS	SNORA41	Stromal_score	8.561813e−09	−0.3783698	2.272219e−04	NEG
1224	CKAP2	Stromal_score	9.836106e−09	−0.3769423	2.610404e−04	NEG	SWSAP1	Stromal_score	2.059027e−13	−0.4715504	5.464452e−09	NEG
1225	PTCH2	Stromal_score	4.888886e−15	0.4985750	1.297462e−10	POS	NAA25	Stromal_score	3.596677e−10	−0.4092501	9.545222e−06	NEG
1226	LOC100289019	Stromal_score	3.060635e−13	−0.4685421	8.122620e−09	NEG	AK125463	Stromal_score	3.364087e−08	−0.3639798	8.927950e−04	NEG
1227	ECSCR	Stromal_score	3.879978e−19	0.5577294	1.029707e−14	POS	DHX33	Stromal_score	4.225661e−07	−0.3353355	1.121448e−02	NEG
1228	AKT3	Stromal_score	3.783018e−26	0.6375012	1.003975e−21	POS	SSRP1	Stromal_score	6.495154e−09	−0.3811916	1.723749e−04	NEG
1229	LOC728392	Stromal_score	6.611268e−22	0.5919280	1.754564e−17	POS	BMPER	Stromal_score	8.467059e−08	0.3538582	2.247073e−03	POS
1230	KIAA1462	Stromal_score	6.654451e−18	0.5411086	1.766025e−13	POS	MRPL3	Stromal_score	4.239561e−07	−0.3352964	1.125137e−02	NEG
1231	ARHGAP25	Stromal_score	6.360155e−10	0.4039259	1.687922e−05	POS	DUS4L	Stromal_score	1.697543e−13	−0.4730049	4.505110e−09	NEG
1232	TNFAIP3	Stromal_score	4.214956e−07	0.3353657	1.118607e−02	POS	HIST1H2AH	Stromal_score	2.207882e−09	−0.3919639	5.859499e−05	NEG
1233	LGI2	Stromal_score	5.072468e−25	0.6260984	1.346182e−20	POS	CDK5RAP1	Stromal_score	8.320688e−13	−0.4608185	2.208228e−08	NEG
1234	PFKFB2	Stromal_score	9.883559e−07	−0.3250348	2.622998e−02	NEG	ZW10	Stromal_score	6.053545e−09	−0.3819066	1.606550e−04	NEG
1235	TMEM88	Stromal_score	4.217415e−12	0.4478559	1.119260e−07	POS	ZNF436	Stromal_score	9.335267e−20	0.5657245	2.477486e−15	POS
1236	ARHGEF17	Stromal_score	3.060722e−19	0.5590755	8.122851e−15	POS	ARRDC5	Stromal_score	3.114485e−07	0.3389445	8.265532e−03	POS
1237	COL8A2	Stromal_score	1.598825e−28	0.6600448	4.243121e−24	POS	TTC30B	Stromal_score	5.120481e−07	−0.3330397	1.358924e−02	NEG
1238	ABI3	Stromal_score	1.808063e−13	0.4725304	4.798418e−09	POS	DDX49	Stromal_score	1.800342e−08	−0.3706414	4.777928e−04	NEG
1239	P2RY14	Stromal_score	1.768854e−09	0.3941300	4.694361e−05	POS	ZC2HC1B	Stromal_score	1.310337e−11	−0.4384658	3.477503e−07	NEG
1240	CLDN11	Stromal_score	4.511261e−09	0.3848741	1.197244e−04	POS	HSPE1−MOB4	Stromal_score	2.068882e−09	−0.3926009	5.490605e−05	NEG
1241	LAYN	Stromal_score	2.055145e−26	0.6401137	5.454149e−22	POS	NLGN4Y	Stromal_score	4.580665e−18	0.5433459	1.215663e−13	POS
1242	CXorf69	Stromal_score	5.161989e−11	0.4267106	1.369940e−06	POS	LCLAT1	Stromal_score	5.372262e−09	−0.3831150	1.425745e−04	NEG
1243	APBB1	Stromal_score	3.308937e−18	0.5452808	8.781587e−14	POS	MRPL42	Stromal_score	1.259430e−06	−0.3220259	3.342400e−02	NEG
1244	CBFA2T3	Stromal_score	4.284722e−08	0.3613611	1.137122e−03	POS	MKL2	Stromal_score	8.685441e−08	0.3535739	2.305029e−03	POS
1245	NUP62CL	Stromal_score	1.669305e−09	−0.3946933	4.430167e−05	NEG	WDSUB1	Stromal_score	2.302930e−07	−0.3424709	6.111746e−03	NEG
1246	RDX	Stromal_score	6.497538e−12	0.4443098	1.724382e−07	POS	RPUSD4	Stromal_score	5.501211e−07	−0.3321777	1.459966e−02	NEG
1247	CLIP4	Stromal_score	4.836184e−19	0.5564736	1.283475e−14	POS	NUP43	Stromal_score	1.275611e−08	−0.3742492	3.385345e−04	NEG
1248	GPX7	Stromal_score	9.876397e−13	0.4594752	2.621097e−08	POS	HLCS	Stromal_score	5.355980e−08	−0.3589242	1.421424e−03	NEG
1249	GABARAPL1	Stromal_score	6.210493e−19	0.5550414	1.648203e−14	POS	PAFAH1B1	Stromal_score	7.473272e−11	0.4234590	1.983332e−06	POS
1250	C1orf172	Stromal_score	8.047519e−18	−0.5399635	2.135731e−13	NEG	PTBP1	Stromal_score	2.075557e−07	−0.3436752	5.508321e−03	NEG
1251	SKA1	Stromal_score	1.132344e−10	−0.4197654	3.005127e−06	NEG	ARFIP2	Stromal_score	2.900775e−10	−0.4112343	7.698366e−06	NEG
1252	CMTM4	Stromal_score	3.882157e−10	−0.4085421	1.030286e−05	NEG	CARD8	Stromal_score	2.711501e−09	0.3899424	7.196054e−05	POS
1253	hCG_1995134	Stromal_score	2.157558e−30	0.6765300	5.725942e−26	POS	ZFP64	Stromal_score	4.207669e−10	−0.4077940	1.116673e−05	NEG
1254	ICAM2	Stromal_score	5.853722e−09	0.3822468	1.553519e−04	POS	OAZ3	Stromal_score	2.364455e−09	−0.3912914	6.275026e−05	NEG
1255	CCNF	Stromal_score	1.640434e−10	−0.4164324	4.353547e−06	NEG	LRCH2	Stromal_score	1.034629e−26	0.6430223	2.745802e−22	POS
1256	GTSE1	Stromal_score	1.000186e−13	−0.4769556	2.654393e−09	NEG	ATPBD4	Stromal_score	9.855396e−07	−0.3250701	2.615524e−02	NEG
1257	CDC25A	Stromal_score	4.271072e−13	−0.4659901	1.133500e−08	NEG	TMEM200C	Stromal_score	1.244375e−07	0.3495294	3.302446e−03	POS
1258	PDE2A	Stromal_score	1.876052e−19	0.5618344	4.978854e−15	POS	IMP4	Stromal_score	6.377725e−10	−0.4038999	1.692585e−05	NEG
1259	IRF6	Stromal_score	3.280533e−09	−0.3880558	8.706205e−05	NEG	FRMD4A	Stromal_score	3.849424e−17	0.5303648	1.021599e−12	POS
1260	CHST3	Stromal_score	4.575347e−13	0.4654604	1.214251e−08	POS	PHF21A	Stromal_score	1.085636e−15	0.5088094	2.881169e−11	POS
1261	CALCRL	Stromal_score	4.792160e−20	0.5693939	1.271791e−15	POS	SLC25A17	Stromal_score	6.751256e−07	−0.3297020	1.791716e−02	NEG
1262	FRMD6	Stromal_score	4.469861e−27	0.6465370	1.186256e−22	POS	FAM72A	Stromal_score	5.057437e−17	−0.5286593	1.342193e−12	NEG
1263	CLEC2B	Stromal_score	9.978238e−25	0.6230471	2.648125e−20	POS	ZNF696	Stromal_score	1.836661e−09	−0.3937636	4.874315e−05	NEG
1264	AP1S3	Stromal_score	2.685653e−08	−0.3663973	7.127454e−04	NEG	CCT7	Stromal_score	2.645949e−07	−0.3408544	7.022083e−03	NEG
1265	SLC29A2	Stromal_score	6.625911e−13	−0.4625946	1.758450e−08	NEG	SYT15	Stromal_score	3.737923e−18	0.5445570	9.920075e−14	POS
1266	C18orf56	Stromal_score	1.804559e−09	−0.3939354	4.789119e−05	NEG	TFE3	Stromal_score	2.346718e−09	0.3913654	6.227954e−05	POS
1267	SOBP	Stromal_score	1.130287e−07	0.3506166	2.999669e−03	POS	SNORA71B	Stromal_score	3.293169e−08	−0.3642094	8.739742e−04	NEG
1268	TSHZ3	Stromal_score	6.347016e−28	0.6545367	1.684435e−23	POS	WDR81	Stromal_score	1.023404e−07	0.3517355	2.716012e−03	POS
1269	PBXIP1	Stromal_score	1.813663e−10	0.4155234	4.813281e−06	POS	GIPC3	Stromal_score	1.476939e−10	0.4173801	3.919648e−06	POS
1270	FUT4	Stromal_score	5.164759e−07	−0.3329364	1.370675e−02	NEG	TAF5	Stromal_score	2.057001e−11	−0.4346489	5.459075e−07	NEG
1271	PRPH	Stromal_score	1.240602e−06	0.3222139	3.292433e−02	POS	NOL11	Stromal_score	4.766767e−10	−0.4066311	1.265052e−05	NEG
1272	PAM	Stromal_score	2.487531e−15	0.5032128	6.601658e−11	POS	AX747750	Stromal_score	3.635714e−10	0.4091501	9.648822e−06	POS
1273	DOCK11	Stromal_score	9.364074e−14	0.4774442	2.485132e−09	POS	DDX56	Stromal_score	2.757639e−10	−0.4116994	7.318498e−06	NEG
1274	LRRN2	Stromal_score	2.215303e−17	0.5337883	5.879192e−13	POS	TOE1	Stromal_score	6.456777e−10	−0.4037838	1.713564e−05	NEG
1275	PLEKHN1	Stromal_score	8.458024e−07	−0.3269515	2.244675e−02	NEG	GPATCH1	Stromal_score	1.000744e−06	−0.3248810	2.655875e−02	NEG
1276	KLHL6	Stromal_score	5.078720e−08	0.3595065	1.347842e−03	POS	TMEM132C	Stromal_score	2.174120e−09	0.3921150	5.769898e−05	POS
1277	CEBPD	Stromal_score	1.333582e−10	0.4182990	3.539193e−06	POS	WDR43	Stromal_score	9.965859e−09	−0.3768072	2.644839e−04	NEG
1278	DOK3	Stromal_score	1.446632e−06	0.3202909	3.839216e−02	POS	METTL2A	Stromal_score	4.354357e−10	−0.4074750	1.155603e−05	NEG
1279	LOC100128881	Stromal_score	1.710895e−07	−0.3459002	4.540545e−03	NEG	ZNF749	Stromal_score	6.580480e−09	−0.3810589	1.746394e−04	NEG
1280	CRB3	Stromal_score	5.162276e−12	−0.4462023	1.370016e−07	NEG	SOCS5	Stromal_score	2.924314e−14	0.4859480	7.760836e−10	POS
1281	CCDC64B	Stromal_score	1.698937e−07	−0.3459806	4.508809e−03	NEG	CLDND1	Stromal_score	4.611424e−09	0.3846535	1.223826e−04	POS
1282	MYO1F	Stromal_score	3.960862e−12	0.4483674	1.051173e−07	POS	TIGD2	Stromal_score	3.091784e−07	−0.3390305	8.205286e−03	NEG
1283	EFNA1	Stromal_score	3.666346e−08	−0.3630511	9.730116e−04	NEG	METAP1D	Stromal_score	5.917644e−09	−0.3821368	1.570484e−04	NEG
1284	FZD8	Stromal_score	1.400716e−10	0.4178574	3.717359e−06	POS	DHX15	Stromal_score	9.051012e−08	−0.3531131	2.402048e−03	NEG
1285	AX747844	Stromal_score	1.034125e−06	0.3244753	2.744465e−02	POS	EBF3	Stromal_score	1.153550e−15	0.5084033	3.061405e−11	POS
1286	KIF4A	Stromal_score	1.289877e−14	−0.4917907	3.423203e−10	NEG	SUPV3L1	Stromal_score	8.717358e−10	−0.4009405	2.313500e−05	NEG
1287	IRS1	Stromal_score	9.588561e−07	0.3254087	2.544708e−02	POS	NARF	Stromal_score	4.721247e−07	−0.3340122	1.252972e−02	NEG
1288	GLIPR2	Stromal_score	1.434255e−25	0.6317064	3.806370e−21	POS	AX746733	Stromal_score	2.128807e−09	−0.3923214	5.649640e−05	NEG
1289	E2F2	Stromal_score	1.004781e−07	−0.3519418	2.666588e−03	NEG	MSTO1	Stromal_score	1.547465e−13	−0.4736998	4.106817e−09	NEG
1290	GP1BB	Stromal_score	1.923193e−13	0.4720653	5.103961e−09	POS	EXOC6	Stromal_score	1.173521e−08	−0.3751161	3.114408e−04	NEG
1291	OIP5	Stromal_score	1.339917e−13	−0.4747780	3.556005e−09	NEG	MKI67IP	Stromal_score	3.189479e−09	−0.3883353	8.464559e−05	NEG
1292	RMI2	Stromal_score	1.271189e−09	−0.3973290	3.373608e−05	NEG	SGIP1	Stromal_score	1.024876e−08	0.3765182	2.719919e−04	POS
1293	TMOD1	Stromal_score	5.168122e−09	0.3835060	1.371568e−04	POS	PASK	Stromal_score	1.450029e−08	−0.3729124	3.848232e−04	NEG
1294	CNKSR1	Stromal_score	2.807442e−10	−0.4115350	7.450670e−06	NEG	EXD3	Stromal_score	5.026003e−10	−0.4061362	1.333851e−05	NEG
1295	HSD11B1	Stromal_score	3.189190e−20	0.5716124	8.463792e−16	POS	FBXL19−AS1	Stromal_score	1.333707e−08	−0.3737854	3.539526e−04	NEG
1296	PLA2G4C	Stromal_score	3.029219e−16	0.5172299	8.039244e−12	POS	RTTN	Stromal_score	2.330240e−07	−0.3423340	6.184225e−03	NEG
1297	HSD17B14	Stromal_score	1.241665e−06	0.3222032	3.295256e−02	POS	APTX	Stromal_score	1.026155e−08	−0.3765053	2.723312e−04	NEG
1298	KLC3	Stromal_score	1.790651e−06	−0.3175983	4.752208e−02	NEG	FANCC	Stromal_score	5.768047e−08	−0.3581103	1.530782e−03	NEG
1299	VASN	Stromal_score	7.999666e−21	0.5790242	2.123031e−16	POS	ARID1B	Stromal_score	1.018213e−06	0.3246671	2.702236e−02	POS
1300	FRK	Stromal_score	1.548849e−07	−0.3470394	4.110490e−03	NEG	APBA1	Stromal_score	7.211056e−07	0.3289007	1.913742e−02	POS
1301	OLFM1	Stromal_score	1.422328e−09	0.3962450	3.774716e−05	POS	BC035400	Stromal_score	4.308681e−21	0.5822806	1.143481e−16	POS
1302	C5orf62	Stromal_score	5.093771e−11	0.4268268	1.351836e−06	POS	ZNF471	Stromal_score	1.423443e−19	0.5633794	3.777676e−15	POS
1303	JA429539	Stromal_score	1.297748e−11	−0.4385470	3.444093e−07	NEG	WDR86	Stromal_score	1.031188e−07	0.3516503	2.736671e−03	POS
1304	E2F8	Stromal_score	8.785512e−12	−0.4418100	2.331587e−07	NEG	ARL2BP	Stromal_score	4.354180e−11	0.4281946	1.155556e−06	POS
1305	SNORD104	Stromal_score	1.006758e−07	−0.3519197	2.671834e−03	NEG	SPECC1L	Stromal_score	7.041657e−10	0.4029652	1.868785e−05	POS
1306	SIRPA	Stromal_score	1.699794e−06	0.3182579	4.511083e−02	POS	RMND1	Stromal_score	5.308616e−07	−0.3326064	1.408854e−02	NEG
1307	CHKA	Stromal_score	1.620531e−08	−0.3717479	4.300728e−04	NEG	C17orf75	Stromal_score	2.109018e−10	−0.4141521	5.597123e−06	NEG
1308	HIST1H2AM	Stromal_score	3.848172e−10	−0.4086237	1.021266e−05	NEG	ANKRD46	Stromal_score	3.291279e−12	−0.4498719	8.734725e−08	NEG
1309	NFIC	Stromal_score	1.371205e−16	0.5223439	3.639042e−12	POS	CCDC163P	Stromal_score	2.142195e−13	−0.4712512	5.685172e−09	NEG
1310	FAM65B	Stromal_score	3.452503e−11	0.4302063	9.162599e−07	POS	LOC100505495	Stromal_score	7.296912e−14	0.4792866	1.936528e−09	POS
1311	TMEM55A	Stromal_score	5.893258e−10	0.4046435	1.564012e−05	POS	NIT2	Stromal_score	6.054808e−08	−0.3575762	1.606886e−03	NEG
1312	OLFML2A	Stromal_score	5.213170e−08	0.3592204	1.383523e−03	POS	MRPL46	Stromal_score	9.438544e−11	−0.4213893	2.504895e−06	NEG
1313	LILRB2	Stromal_score	4.290566e−10	0.4076124	1.138673e−05	POS	DPY19L1P1	Stromal_score	3.590849e−14	−0.4844647	9.529755e−10	NEG
1314	GNG7	Stromal_score	7.282886e−09	0.3800256	1.932805e−04	POS	MRS2	Stromal_score	1.387567e−06	−0.3208139	3.682464e−02	NEG
1315	BTK	Stromal_score	5.534074e−10	0.4052340	1.468688e−05	POS	AK054921	Stromal_score	7.478062e−10	0.4023963	1.984603e−05	POS
1316	MEIS3P1	Stromal_score	1.473791e−06	0.3200571	3.911294e−02	POS	BC112312	Stromal_score	6.959674e−08	−0.3560377	1.847028e−03	NEG
1317	PLP1	Stromal_score	5.113725e−14	0.4818938	1.357132e−09	POS	METTL17	Stromal_score	2.551264e−07	−0.3412795	6.770798e−03	NEG
1318	FBXL7	Stromal_score	2.630623e−35	0.7152781	6.981410e−31	POS	NOG	Stromal_score	1.741279e−07	0.3456982	4.621180e−03	POS
1319	MFI2−AS1	Stromal_score	4.688942e−08	−0.3603791	1.244398e−03	NEG	PPM1G	Stromal_score	4.790218e−09	−0.3842710	1.271276e−04	NEG
1320	HTR2B	Stromal_score	6.292225e−07	0.3305558	1.669893e−02	POS	FNDC8	Stromal_score	1.976046e−08	−0.3696587	5.244228e−04	NEG

	gene	feature	p.value	estimate	adj.p.value	COR.ID	gene	feature	p.value	estimate	adj.p.value	COR.ID
1321	EBF1	Stromal_score	6.523576e−20	0.5677023	1.731292e−15	POS	BC024173	Stromal_score	1.316385e−07	0.3488915	3.493554e−03	POS
1322	PAFAH1B3	Stromal_score	5.857723e−07	−0.3314208	1.554581e−02	NEG	LRRC57	Stromal_score	6.170680e−07	−0.3307919	1.637637e−02	NEG
1323	VWA7	Stromal_score	9.021427e−07	−0.3261595	2.394197e−02	NEG	MOB3C	Stromal_score	5.826061e−08	0.3580002	1.546178e−03	POS
1324	TACC3	Stromal_score	4.013233e−09	−0.3860464	1.065072e−04	NEG	BC021024	Stromal_score	2.009326e−15	−0.5046637	5.332550e−11	NEG
1325	CHST11	Stromal_score	1.116243e−14	0.4928113	2.962398e−10	POS	PARK2	Stromal_score	8.885191e−08	0.3533199	2.358041e−03	POS
1326	IL16	Stromal_score	5.596184e−13	0.4639050	1.485171e−08	POS	NUDT13	Stromal_score	1.016890e−06	−0.3246832	2.698723e−02	NEG
1327	FBXL22	Stromal_score	8.559781e−08	0.3537367	2.271680e−03	POS	ZFYVE20	Stromal_score	1.970221e−07	0.3442767	5.228770e−03	POS
1328	KIF23	Stromal_score	1.257427e−12	−0.4575724	3.337084e−08	NEG	SEC61A2	Stromal_score	1.780058e−07	−0.3454452	4.724097e−03	NEG
1329	MAPK4	Stromal_score	4.535870e−07	0.3344909	1.203774e−02	POS	NPAS1	Stromal_score	1.711016e−07	−0.3458994	4.540865e−03	NEG
1330	GIMAP1−GIMAP5	Stromal_score	1.675650e−18	0.5492891	4.447008e−14	POS	MS4A14	Stromal_score	3.570815e−09	0.3872123	9.476586e−05	POS
1331	EPS8L2	Stromal_score	1.068075e−09	−0.3990012	2.834565e−05	NEG	PLCL1	Stromal_score	2.232030e−18	0.5476066	5.923584e−14	POS
1332	ARHGAP11A	Stromal_score	1.575012e−11	−0.4369144	4.179924e−07	NEG	AK054623	Stromal_score	2.711802e−08	−0.3662937	7.196850e−04	NEG
1333	MRPS35	Stromal_score	1.463386e−10	−0.4174632	3.883679e−06	NEG	SPIN1	Stromal_score	1.265184e−08	0.3743347	3.357673e−04	POS
1334	ZNF385A	Stromal_score	4.840368e−10	0.4064880	1.284585e−05	POS	EPB41L5	Stromal_score	3.425957e−12	−0.4495467	9.092148e−08	NEG
1335	HIST1H2AD	Stromal_score	1.496748e−06	−0.3198628	3.972220e−02	NEG	LSM12	Stromal_score	5.153855e−10	−0.4059011	1.367782e−05	NEG
1336	KIF11	Stromal_score	8.119661e−14	−0.4784988	2.154877e−09	NEG	PES1	Stromal_score	1.594133e−06	−0.3190687	4.230670e−02	NEG
1337	AX795351	Stromal_score	3.076141e−12	−0.4504192	8.163770e−08	NEG	ORC2	Stromal_score	4.675709e−07	−0.3341281	1.240886e−02	NEG
1338	CSF2RA	Stromal_score	1.794483e−10	0.4156198	4.762377e−06	POS	HSF2BP	Stromal_score	6.034831e−08	−0.3576126	1.601584e−03	NEG
1339	ZNF880	Stromal_score	8.378570e−10	0.4013175	2.223589e−05	POS	TRUB1	Stromal_score	2.147307e−08	−0.3687788	5.698739e−04	NEG
1340	LRRN4CL	Stromal_score	1.022201e−18	0.5521673	2.712819e−14	POS	MRPS23	Stromal_score	1.052257e−08	−0.3762458	2.792586e−04	NEG
1341	PREX1	Stromal_score	4.003395e−12	0.4482804	1.062461e−07	POS	TAF1A	Stromal_score	3.333031e−12	−0.4497697	8.845530e−08	NEG
1342	DOCK8	Stromal_score	1.657964e−09	0.3947596	4.400069e−05	POS	KIAA1614	Stromal_score	9.141230e−08	0.3530021	2.425991e−03	POS
1343	RPP25	Stromal_score	7.894977e−08	−0.3546378	2.095248e−03	NEG	LURAP1	Stromal_score	3.904393e−17	0.5302764	1.036187e−12	POS
1344	MSC	Stromal_score	4.621061e−21	0.5819140	1.226383e−16	POS	PSTK	Stromal_score	1.688321e−09	−0.3945832	4.480636e−05	NEG
1345	EMCN	Stromal_score	7.554319e−20	0.5668943	2.004841e−15	POS	ZC3H15	Stromal_score	1.695482e−06	−0.3182901	4.499639e−02	NEG
1346	THEM6	Stromal_score	2.097639e−08	−0.3690268	5.566925e−04	NEG	BMX	Stromal_score	9.399979e−08	0.3526895	2.494660e−03	POS
1347	EVC	Stromal_score	1.208215e−28	0.6611499	3.206481e−24	POS	NPRL2	Stromal_score	1.718164e−07	−0.3458515	4.559836e−03	NEG
1348	MPP1	Stromal_score	1.671980e−11	0.4364089	4.437267e−07	POS	AHRR	Stromal_score	1.515133e−06	−0.3197092	4.021011e−02	NEG
1349	CCR1	Stromal_score	5.459736e−09	0.3829518	1.448959e−04	POS	HNRNPA2B1	Stromal_score	6.127975e−07	−0.3308759	1.626303e−02	NEG
1350	CRIM1	Stromal_score	4.672170e−16	0.5143973	1.239947e−11	POS	CSTF1	Stromal_score	1.021382e−08	−0.3765535	2.710644e−04	NEG
1351	EVL	Stromal_score	1.605673e−19	0.5627063	4.261296e−15	POS	LINC00511	Stromal_score	1.058728e−08	−0.3761824	2.809759e−04	NEG
1352	CENPW	Stromal_score	7.447458e−07	−0.3285076	1.976481e−02	NEG	MTX2	Stromal_score	1.494539e−09	−0.3957660	3.966357e−05	NEG
1353	HIC1	Stromal_score	1.930743e−33	0.7012867	5.123998e−29	POS	C2orf29	Stromal_score	5.325429e−12	−0.4459470	1.413316e−07	NEG
1354	NCAPH	Stromal_score	1.367696e−13	−0.4746246	3.629729e−09	NEG	CHSY3	Stromal_score	4.715661e−15	0.4988244	1.251489e−10	POS
1355	RTN4R	Stromal_score	7.832854e−09	−0.3792817	2.078761e−04	NEG	PELO	Stromal_score	5.560911e−09	0.3827662	1.475810e−04	POS
1356	NDC80	Stromal_score	1.873434e−11	−0.4354440	4.971906e−07	NEG	ZNF707	Stromal_score	1.135429e−06	−0.3233166	3.013316e−02	NEG
1357	FAM166A	Stromal_score	3.660223e−07	−0.3370404	9.713867e−03	NEG	TDP1	Stromal_score	9.471252e−10	−0.4001501	2.513575e−05	NEG
1358	DLC1	Stromal_score	1.504226e−22	0.5993262	3.992064e−18	POS	MKL1	Stromal_score	3.252495e−09	0.3881411	8.631797e−05	POS
1359	BST1	Stromal_score	3.782163e−12	0.4487432	1.003748e−07	POS	UTP6	Stromal_score	8.361614e−07	−0.3270920	2.219089e−02	NEG
1360	SNORA76	Stromal_score	4.363658e−11	−0.4281757	1.158071e−06	NEG	TECPR2	Stromal_score	9.634652e−08	0.3524131	2.556940e−03	POS
1361	EXO1	Stromal_score	3.778353e−12	−0.4487514	1.002737e−07	NEG	C6orf99	Stromal_score	1.552757e−11	−0.4370347	4.120862e−07	NEG
1362	STAB1	Stromal_score	1.176056e−23	0.6116294	3.121134e−19	POS	PTCD3	Stromal_score	3.890979e−11	−0.4291714	1.032627e−06	NEG
1363	TSHZ2	Stromal_score	3.081823e−15	0.5017499	8.178850e−11	POS	FTSJ2	Stromal_score	3.135441e−09	−0.3885049	8.321146e−05	NEG
1364	DPYSL2	Stromal_score	7.299429e−12	0.4433479	1.937195e−07	POS	DKFZp686D0853	Stromal_score	1.536632e−10	0.4170228	4.078067e−06	POS
1365	SRD5A1	Stromal_score	2.583916e−09	−0.3904178	6.857455e−05	NEG	AK094916	Stromal_score	2.060103e−08	−0.3692180	5.467307e−04	NEG
1366	ARMCX1	Stromal_score	1.419287e−18	0.5502591	3.766645e−14	POS	HEATR3	Stromal_score	1.233924e−07	−0.3496249	3.274712e−03	NEG
1367	UBE2E2	Stromal_score	6.644972e−17	0.5269437	1.763509e−12	POS	UBAC2−AS1	Stromal_score	2.395403e−11	−0.4333490	6.357160e−07	NEG
1368	BLVRA	Stromal_score	1.544543e−08	0.3722515	4.099064e−04	POS	ATG16L1	Stromal_score	1.348582e−06	−0.3211710	3.579002e−02	NEG
1369	PRKACB	Stromal_score	1.805461e−10	0.4155645	4.791512e−06	POS	C16orf93	Stromal_score	1.490326e−10	−0.4172988	3.955175e−06	NEG
1370	DIRAS3	Stromal_score	1.308096e−11	0.4384802	3.471557e−07	POS	SLC45A1	Stromal_score	1.139541e−15	0.5084851	3.024228e−11	POS
1371	CDK14	Stromal_score	5.885567e−14	0.4808655	1.561971e−09	POS	CCDC15	Stromal_score	2.281833e−10	−0.4134338	6.055757e−06	NEG
1372	DCHS1	Stromal_score	9.085856e−24	0.6128457	2.411295e−19	POS	PDE1C	Stromal_score	5.505538e−13	0.4640315	1.461115e−08	POS
1373	CMTM3	Stromal_score	1.650569e−20	0.5751658	4.380445e−16	POS	DQ574760	Stromal_score	1.307957e−08	−0.3739885	3.471187e−04	NEG
1374	SHMT2	Stromal_score	1.626363e−06	−0.3188161	4.316205e−02	NEG	DPYSL4	Stromal_score	2.716828e−08	0.3662739	7.210190e−04	POS
1375	SPC25	Stromal_score	8.978757e−17	−0.5250409	2.382872e−12	NEG	SETD8	Stromal_score	2.887747e−08	−0.3656207	7.663792e−04	NEG
1376	MAF	Stromal_score	1.165116e−21	0.5890454	3.092102e−17	POS	NIF3L1	Stromal_score	5.975713e−13	−0.4633966	1.585894e−08	NEG
1377	TCN2	Stromal_score	4.360714e−13	0.4658303	1.157290e−08	POS	CAMTA2	Stromal_score	6.681971e−08	0.3564884	1.773328e−03	POS
1378	TSPAN11	Stromal_score	1.001442e−18	0.5522862	2.657728e−14	POS	ZNF653	Stromal_score	8.849879e−11	−0.4219616	2.348669e−06	NEG
1379	EVI2A	Stromal_score	4.401524e−12	0.4475071	1.168120e−07	POS	POLR2B	Stromal_score	1.198873e−11	0.4392127	3.181690e−07	POS
1380	ASPM	Stromal_score	1.634016e−11	−0.4366033	4.336515e−07	NEG	MTHFD2L	Stromal_score	5.846359e−12	−0.4451800	1.551565e−07	NEG
1381	PCDHB5	Stromal_score	4.424800e−09	0.3850683	1.174298e−04	POS	LOC100129518	Stromal_score	2.065813e−13	−0.4715255	5.482460e−09	NEG
1382	PNMA1	Stromal_score	8.198130e−08	0.3542182	2.175702e−03	POS	ADAMTS9−AS2	Stromal_score	2.211752e−16	0.5192695	5.869767e−12	POS
1383	C9orf71	Stromal_score	1.631734e−11	0.4366151	4.330459e−07	POS	ADAT2	Stromal_score	1.333143e−12	−0.4571099	3.538029e−08	NEG
1384	TNFAIP8	Stromal_score	2.652371e−12	0.4516160	7.039127e−08	POS	MLX	Stromal_score	2.121335e−07	−0.3434228	5.629812e−03	NEG
1385	CDCA3	Stromal_score	1.258390e−11	−0.4388059	3.339641e−07	NEG	NUP85	Stromal_score	5.410185e−08	−0.3588137	1.435809e−03	NEG
1386	STIL	Stromal_score	1.967929e−12	−0.4540117	5.222688e−08	NEG	SLC10A7	Stromal_score	1.448475e−06	−0.3202749	3.844107e−02	NEG
1387	SOWAHB	Stromal_score	1.112773e−10	−0.4199213	2.953189e−06	NEG	BC035377	Stromal_score	4.357584e−07	−0.3349693	1.156459e−02	NEG
1388	SH3PXD2B	Stromal_score	1.774470e−19	0.5621466	4.709266e−15	POS	PRPF19	Stromal_score	1.183242e−07	−0.3500995	3.140206e−03	NEG
1389	MCM7	Stromal_score	8.227779e−09	−0.3787779	2.183570e−04	NEG	HIST1H4B	Stromal_score	4.486498e−09	−0.3849294	1.190672e−04	NEG
1390	LOC100129845	Stromal_score	1.110820e−17	0.5380116	2.948004e−13	POS	PARP2	Stromal_score	5.802874e−09	−0.3823352	1.540025e−04	NEG
1391	COL4A4	Stromal_score	1.758083e−07	0.3455879	4.665776e−03	POS	RG9MTD2	Stromal_score	2.181438e−11	−0.4341483	5.789318e−07	NEG
1392	PIK3CG	Stromal_score	1.205835e−08	0.3748341	3.200167e−04	POS	C6orf89	Stromal_score	1.721484e−06	0.3180975	4.568646e−02	POS
1393	HIST1H3D	Stromal_score	1.625998e−07	−0.3464834	4.315236e−03	NEG	ETV2	Stromal_score	4.901298e−09	−0.3840403	1.300755e−04	NEG
1394	FAM201A	Stromal_score	1.090726e−08	−0.3758744	2.894679e−04	NEG	ALPK2	Stromal_score	4.468156e−10	0.4072347	1.185804e−05	POS
1395	CD72	Stromal_score	1.755173e−06	0.3178520	4.658053e−02	POS	MRPS11	Stromal_score	4.517308e−08	−0.3607857	1.198848e−03	NEG
1396	PLOD2	Stromal_score	2.660803e−08	0.3664966	7.061505e−04	POS	EFHA2	Stromal_score	2.290509e−20	0.5734035	6.078781e−16	POS
1397	OTX1	Stromal_score	1.020445e−12	−0.4592184	2.708160e−08	NEG	NOS1AP	Stromal_score	9.979891e−07	−0.3249150	2.648563e−02	NEG
1398	SEMA6B	Stromal_score	8.073136e−16	0.5107842	2.142529e−11	POS	SLC9B1	Stromal_score	2.262796e−11	−0.4338357	6.005233e−07	NEG
1399	TEAD1	Stromal_score	1.289503e−08	0.3741365	3.422212e−04	POS	RNF146	Stromal_score	2.846259e−07	0.3400011	7.553688e−03	POS
1400	MFGE8	Stromal_score	1.172758e−15	0.5082926	3.112383e−11	POS	CHD9	Stromal_score	1.644220e−08	0.3715955	4.363595e−04	POS
1401	BDKRB1	Stromal_score	1.141642e−10	0.4196923	3.029803e−06	POS	EFCAB11	Stromal_score	4.295173e−15	−0.4994692	1.139896e−10	NEG
1402	BC039364	Stromal_score	1.577455e−19	0.5628054	4.186409e−15	POS	KATNB1	Stromal_score	1.193123e−07	−0.3500054	3.166429e−03	NEG
1403	NRP1	Stromal_score	6.866723e−26	0.6349242	1.822360e−21	POS	ERAL1	Stromal_score	1.797901e−08	−0.3706557	4.771450e−04	NEG
1404	RGCC	Stromal_score	3.242564e−08	0.3643761	8.605440e−04	POS	LCTL	Stromal_score	5.074203e−16	−0.5138547	1.346643e−11	NEG
1405	PODXL	Stromal_score	5.424302e−07	0.3323471	1.439555e−02	POS	TBCE	Stromal_score	1.072739e−07	−0.3512057	2.846942e−03	NEG
1406	NUDT16P1	Stromal_score	5.684544e−10	−0.4049822	1.508621e−05	NEG	RECQL5	Stromal_score	3.590253e−08	−0.3632777	9.528174e−04	NEG
1407	GNA14	Stromal_score	4.523909e−07	0.3345224	1.200600e−02	POS	PICALM	Stromal_score	2.995326e−08	0.3652285	7.949295e−04	POS
1408	MMP14	Stromal_score	3.452464e−10	0.4096287	9.162494e−06	POS	TSN	Stromal_score	3.964552e−10	−0.4083471	1.052152e−05	NEG
1409	LOC645249	Stromal_score	1.523487e−10	−0.4171003	4.043183e−06	NEG	HBP1	Stromal_score	6.091001e−08	0.3575106	1.616491e−03	POS
1410	LYL1	Stromal_score	2.776471e−13	0.4692843	7.368477e−09	POS	IMMP1L	Stromal_score	2.616054e−07	−0.3409870	6.942747e−03	NEG
1411	MAGEH1	Stromal_score	2.596971e−19	0.5600044	6.892101e−15	POS	FUBP1	Stromal_score	2.274292e−07	−0.3426161	6.035742e−03	NEG
1412	MOCS1	Stromal_score	2.033245e−09	0.3927708	5.396030e−05	POS	PTH1R	Stromal_score	2.851498e−13	0.4690814	7.567590e−09	POS
1413	FIBIN	Stromal_score	1.750085e−29	0.6686468	4.644550e−25	POS	BC096759	Stromal_score	1.859267e−10	−0.4152981	4.934308e−06	NEG
1414	ELTD1	Stromal_score	7.529786e−21	0.5793444	1.998330e−16	POS	ABCA11P	Stromal_score	2.811429e−09	−0.3895849	7.461250e−05	NEG
1415	RASSF7	Stromal_score	4.134509e−13	−0.4662399	1.097257e−08	NEG	CCDC41	Stromal_score	1.238646e−09	−0.3975787	3.287242e−05	NEG
1416	CKAP2L	Stromal_score	1.493841e−12	−0.4562077	3.964505e−08	NEG	TFB1M	Stromal_score	5.916440e−12	−0.4450820	1.570164e−07	NEG
1417	KCTD15	Stromal_score	1.609149e−08	0.3718219	4.270519e−04	POS	DDX55	Stromal_score	1.191670e−12	−0.4579966	3.162574e−08	NEG
1418	ID3	Stromal_score	1.780952e−06	0.3176672	4.726468e−02	POS	ACP1	Stromal_score	1.354268e−16	−0.5224234	3.594093e−12	NEG
1419	ORC1	Stromal_score	1.312901e−11	−0.4384494	3.484307e−07	NEG	TMEM183B	Stromal_score	8.262578e−07	−0.3272380	2.192806e−02	NEG
1420	CMTM8	Stromal_score	7.843348e−21	−0.5791286	2.081546e−16	NEG	CA5B	Stromal_score	2.105247e−07	0.3435109	5.587115e−03	POS
1421	C1orf54	Stromal_score	1.430908e−21	0.5879929	3.797487e−17	POS	AK001875	Stromal_score	1.948166e−10	0.4148739	5.170238e−06	POS
1422	ANK2	Stromal_score	1.490962e−21	0.5877818	3.956863e−17	POS	TMEM240	Stromal_score	6.884214e−16	0.5118411	1.827002e−11	POS
1423	ADAM19	Stromal_score	8.096267e−25	0.6239933	2.148668e−20	POS	ARHGAP11B	Stromal_score	2.264556e−09	−0.3917153	6.009906e−05	NEG
1424	VAT1	Stromal_score	2.175711e−10	0.4138683	5.774120e−06	POS	BC044305	Stromal_score	1.102300e−10	0.4200058	2.925394e−06	POS
1425	GRHL2	Stromal_score	4.275572e−10	−0.4076450	1.134694e−05	NEG	TRIM11	Stromal_score	8.254661e−07	−0.3272498	2.190705e−02	NEG
1426	GMNN	Stromal_score	6.869056e−12	−0.4438506	1.822979e−07	NEG	ATF7	Stromal_score	9.394988e−13	0.4598674	2.493336e−08	POS
1427	LTC4S	Stromal_score	1.730762e−12	0.4550368	4.593270e−08	POS	POU2F1	Stromal_score	4.599330e−08	−0.3605895	1.220616e−03	NEG
1428	PLXND1	Stromal_score	1.985518e−12	0.4539406	5.269367e−08	POS	ZNF124	Stromal_score	4.919185e−14	−0.4821769	1.305502e−09	NEG
1429	DENND5A	Stromal_score	1.402383e−23	0.6107968	3.721783e−19	POS	EPS15	Stromal_score	2.343750e−11	0.4335354	6.220078e−07	POS
1430	GJA4	Stromal_score	1.364185e−15	0.5072779	3.620409e−11	POS	TTC27	Stromal_score	9.175343e−08	−0.3529604	2.435044e−03	NEG
1431	FYCO1	Stromal_score	2.328740e−13	0.4706193	6.180244e−09	POS	AX747991	Stromal_score	2.992147e−10	−0.4109490	7.940860e−06	NEG
1432	PTPRM	Stromal_score	3.900086e−26	0.6373701	1.035044e−21	POS	AR	Stromal_score	9.732481e−19	0.5524516	2.582903e−14	POS
1433	NFATC1	Stromal_score	2.417441e−09	0.3910735	6.415648e−05	POS	NTNG2	Stromal_score	1.697228e−13	0.4730063	4.504273e−09	POS
1434	MPDZ	Stromal_score	5.061910e−26	0.6362454	1.343380e−21	POS	HERC1	Stromal_score	7.053589e−10	0.4029492	1.871952e−05	POS
1435	ELMO1	Stromal_score	1.350445e−15	0.5073460	3.583946e−11	POS	MARK2	Stromal_score	6.215264e−09	−0.3816391	1.649469e−04	NEG
1436	TWIST1	Stromal_score	5.365033e−08	0.3589057	1.423826e−03	POS	CFP	Stromal_score	1.463474e−07	0.3476865	3.883914e−03	POS
1437	PLEKHA6	Stromal_score	9.908029e−07	−0.3250043	2.629492e−02	NEG	ABT1	Stromal_score	1.185265e−08	−0.3750128	3.145576e−04	NEG
1438	RBM47	Stromal_score	8.511646e−07	−0.3268740	2.258906e−02	NEG	ING5	Stromal_score	1.052138e−08	−0.3762469	2.792269e−04	NEG
1439	ALDH1A2	Stromal_score	5.711217e−07	0.3317264	1.515700e−02	POS	ANKRD40	Stromal_score	1.725738e−07	0.3458011	4.579936e−03	POS
1440	FAM19A5	Stromal_score	3.570499e−10	0.4093177	9.475747e−06	POS	BC132841	Stromal_score	8.064821e−23	0.6023848	2.140323e−18	POS

	gene	feature	p.value	estimate	adj.p.value	COR.ID	gene	feature	p.value	estimate	adj.p.value	COR.ID
1441	C1QTNF7	Stromal_score	1.524908e−09	0.3955711	4.046953e−05	POS	PIGC	Stromal_score	5.630919e−07	−0.3318970	1.494390e−02	NEG
1442	SALL2	Stromal_score	1.148115e−17	0.5378109	3.046983e−13	POS	LINC00473	Stromal_score	3.851877e−10	0.4086147	1.022250e−05	POS
1443	NCALD	Stromal_score	4.887031e−14	0.4822247	1.296969e−09	POS	TCP11L1	Stromal_score	8.752456e−08	0.3534881	2.322814e−03	POS
1444	WISP1	Stromal_score	1.534966e−20	0.5755549	4.073646e−16	POS	QTRTD1	Stromal_score	4.336727e−11	−0.4282295	1.150924e−06	NEG
1445	HIST1H3H	Stromal_score	4.545662e−08	−0.3607175	1.206373e−03	NEG	GRIN3B	Stromal_score	2.346418e−07	−0.3422536	6.227158e−03	NEG
1446	GLIPR1	Stromal_score	1.807345e−25	0.6306884	4.796512e−21	POS	WHSC2	Stromal_score	3.430606e−10	−0.4096874	9.104486e−06	NEG
1447	GRTP1	Stromal_score	5.420128e−10	−0.4054291	1.438448e−05	NEG	PSMD14	Stromal_score	1.230143e−12	−0.4577457	3.264675e−08	NEG
1448	ARL4C	Stromal_score	2.336713e−07	0.3423018	6.201401e−03	POS	THUMPD2	Stromal_score	2.416364e−08	−0.3675245	6.412789e−04	NEG
1449	TRAF3IP3	Stromal_score	4.981269e−07	0.3333703	1.321979e−02	POS	LOC642846	Stromal_score	7.077287e−10	−0.4029175	1.878241e−05	NEG
1450	SPAG1	Stromal_score	6.549829e−07	−0.3300696	1.738259e−02	NEG	BCCIP	Stromal_score	1.055201e−10	−0.4203957	2.800397e−06	NEG
1451	ITGA4	Stromal_score	4.014998e−11	0.4288992	1.065540e−06	POS	FAT3	Stromal_score	9.879988e−07	0.3250393	2.622050e−02	POS
1452	ELK3	Stromal_score	6.715668e−19	0.5545921	1.782271e−14	POS	RGS7BP	Stromal_score	6.095023e−10	0.4043268	1.617558e−05	POS
1453	CHRM2	Stromal_score	3.169474e−10	0.4104184	8.411467e−06	POS	OXNAD1	Stromal_score	3.739306e−07	−0.3367873	9.923745e−03	NEG
1454	PKD2	Stromal_score	1.042041e−26	0.6429922	2.765473e−22	POS	GNL2	Stromal_score	2.404399e−08	−0.3675773	6.381036e−04	NEG
1455	GNG2	Stromal_score	6.191537e−21	0.5803770	1.643172e−16	POS	ZDHHC16	Stromal_score	1.222970e−06	−0.3223924	3.245641e−02	NEG
1456	CLDN12	Stromal_score	1.134337e−07	−0.3505763	3.010416e−03	NEG	ZFAND2B	Stromal_score	4.417642e−07	−0.3348061	1.172398e−02	NEG
1457	GIMAP2	Stromal_score	3.194289e−07	0.3386470	8.477324e−03	POS	NOL7	Stromal_score	1.834942e−06	−0.3172883	4.869753e−02	NEG
1458	LAT2	Stromal_score	2.030013e−09	0.3927864	5.387451e−05	POS	BC037861	Stromal_score	9.078451e−09	0.3777678	2.409330e−04	POS
1459	NPR1	Stromal_score	7.117616e−18	0.5407037	1.888944e−13	POS	TMX2	Stromal_score	2.386211e−07	−0.3420582	6.332764e−03	NEG
1460	EID1	Stromal_score	8.029923e−15	0.4951238	2.131061e−10	POS	ZNF335	Stromal_score	1.238716e−06	−0.3222328	3.287429e−02	NEG
1461	TCEAL7	Stromal_score	6.655393e−22	0.5918943	1.766275e−17	POS	KCND1	Stromal_score	1.453738e−12	0.4564237	3.858074e−08	POS
1462	ATAD2	Stromal_score	2.629095e−10	−0.4121375	6.977356e−06	NEG	CTNNA3	Stromal_score	1.310371e−08	0.3739693	3.477593e−04	POS
1463	GPR34	Stromal_score	1.526141e−16	0.5216581	4.050226e−12	POS	FAM160B1	Stromal_score	3.619713e−11	0.4297974	9.606356e−07	POS
1464	WTIP	Stromal_score	1.506562e−09	0.3956884	3.998264e−05	POS	ATP6V1G2	Stromal_score	2.426953e−08	0.3674779	6.440890e−04	POS
1465	PHLDB1	Stromal_score	1.002162e−22	0.6013226	2.659638e−18	POS	SRRT	Stromal_score	2.586010e−09	−0.3904098	6.863012e−05	NEG
1466	NID2	Stromal_score	3.103681e−20	0.5717599	8.236860e−16	POS	TXLNB	Stromal_score	1.853315e−20	0.5745439	4.918511e−16	POS
1467	TIMELESS	Stromal_score	1.476796e−10	−0.4173810	3.919269e−06	NEG	TACO1	Stromal_score	8.360352e−08	−0.3539997	2.218754e−03	NEG
1468	ZYX	Stromal_score	4.658400e−14	0.4825740	1.236293e−09	POS	AAMP	Stromal_score	2.168473e−09	−0.3921405	5.754910e−05	NEG
1469	CD86	Stromal_score	1.604438e−10	0.4166329	4.258019e−06	POS	NLRP3	Stromal_score	5.922915e−11	0.4255062	1.571882e−06	POS
1470	GJC1	Stromal_score	3.888944e−13	0.4667100	1.032087e−08	POS	PHOSPHO2	Stromal_score	1.579712e−06	−0.3191833	4.192398e−02	NEG
1471	CLCN2	Stromal_score	5.905800e−13	−0.4634878	1.567340e−08	NEG	PCDHB7	Stromal_score	9.475776e−11	0.4213543	2.514776e−06	POS
1472	ITPR3	Stromal_score	6.842553e−08	−0.3562256	1.815945e−03	NEG	RNLS	Stromal_score	6.930857e−11	0.4241240	1.839380e−06	POS
1473	VIPR2	Stromal_score	1.254176e−10	0.4188502	3.328457e−06	POS	GRID1	Stromal_score	9.096394e−08	0.3530571	2.414092e−03	POS
1474	PDZD4	Stromal_score	2.102930e−18	0.5479570	5.580965e−14	POS	MDGA1	Stromal_score	2.936535e−12	0.4507947	7.793271e−08	POS
1475	DNMT3B	Stromal_score	1.120108e−09	−0.3985452	2.972656e−05	NEG	NEK8	Stromal_score	1.707575e−10	−0.4160695	4.531733e−06	NEG
1476	SETBP1	Stromal_score	1.267363e−21	0.5886150	3.363456e−17	POS	METTL12	Stromal_score	5.408598e−10	−0.4054491	1.435388e−05	NEG
1477	CITED2	Stromal_score	1.735317e−15	0.5056560	4.605357e−11	POS	RTN3	Stromal_score	9.440547e−07	−0.3256005	2.505427e−02	NEG
1478	AF289551	Stromal_score	1.071509e−11	−0.4401536	2.843677e−07	NEG	SNX1	Stromal_score	4.194984e−12	0.4478994	1.113307e−07	POS
1479	CLSTN3	Stromal_score	4.893637e−13	0.4649419	1.298722e−08	POS	COG2	Stromal_score	1.408173e−07	−0.3481253	3.737149e−03	NEG
1480	ESCO2	Stromal_score	1.075660e−09	−0.3989334	2.854694e−05	NEG	VPS33B	Stromal_score	6.733254e−08	−0.3564038	1.786938e−03	NEG
1481	KPNA2	Stromal_score	6.239879e−10	−0.4041057	1.656001e−05	NEG	BCS1L	Stromal_score	1.039446e−10	−0.4205299	2.758586e−06	NEG
1482	CD59	Stromal_score	1.966817e−07	0.3442966	5.219736e−03	POS	SMG7	Stromal_score	1.337117e−06	−0.3212778	3.548575e−02	NEG
1483	PARVA	Stromal_score	2.228064e−18	0.5476171	5.913059e−14	POS	BC031657	Stromal_score	2.305092e−09	−0.3915412	6.117484e−05	NEG
1484	AGPAT2	Stromal_score	9.325412e−08	−0.3527787	2.474871e−03	NEG	WDR85	Stromal_score	6.658447e−10	−0.4034937	1.767085e−05	NEG
1485	CCNE2	Stromal_score	1.916176e−10	−0.4150243	5.085339e−06	NEG	TNNI1	Stromal_score	8.411038e−12	−0.4421722	2.232205e−07	NEG
1486	SLC19A1	Stromal_score	3.388447e−11	−0.4303681	8.992600e−07	NEG	NAA35	Stromal_score	9.998537e−09	−0.3767734	2.653512e−04	NEG
1487	OGFRL1	Stromal_score	2.608912e−07	0.3410189	6.923793e−03	POS	ZDHHC6	Stromal_score	2.838972e−07	−0.3400311	7.534348e−03	NEG
1488	DYSF	Stromal_score	4.724375e−17	0.5290859	1.253802e−12	POS	PARS2	Stromal_score	3.692323e−13	−0.4671078	9.799055e−09	NEG
1489	NCAPG	Stromal_score	2.062754e−12	−0.4536352	5.474342e−08	NEG	P2RY12	Stromal_score	1.205136e−09	0.3978425	3.198311e−05	POS
1490	CD99L2	Stromal_score	2.316280e−15	0.5036983	6.147177e−11	POS	LOC644554	Stromal_score	1.556757e−10	0.4169054	4.131478e−06	POS
1491	DHRS13	Stromal_score	1.887665e−12	−0.4543445	5.009673e−08	NEG	SYCP2L	Stromal_score	1.425777e−09	−0.3962216	3.783869e−05	NEG
1492	NLRP1	Stromal_score	9.525279e−15	0.4939270	2.527914e−10	POS	ARF1	Stromal_score	4.587857e−07	−0.3343548	1.217571e−02	NEG
1493	DEPDC1	Stromal_score	4.158821e−14	−0.4833995	1.103710e−09	NEG	ZNF670	Stromal_score	2.844017e−08	−0.3657842	7.547737e−04	NEG
1494	FLI1	Stromal_score	4.867755e−22	0.5934735	1.291854e−17	POS	AK056368	Stromal_score	4.691115e−12	−0.4469863	1.244975e−07	NEG
1495	PNKD	Stromal_score	5.613110e−07	−0.3319352	1.489663e−02	NEG	C9orf114	Stromal_score	7.357132e−08	−0.3554220	1.952509e−03	NEG
1496	FAAH	Stromal_score	2.663326e−11	−0.4324408	7.068200e−07	NEG	SNX3	Stromal_score	2.128020e−07	0.3433864	5.647553e−03	POS
1497	SAMD10	Stromal_score	7.490617e−09	−0.3797384	1.987935e−04	NEG	MRPS9	Stromal_score	2.803591e−07	−0.3401779	7.440451e−03	NEG
1498	CDO1	Stromal_score	4.278317e−22	0.5941227	1.135422e−17	POS	DDIT4L	Stromal_score	2.440401e−12	0.4522864	6.476581e−08	POS
1499	CCDC8	Stromal_score	4.543007e−19	0.5568306	1.205669e−14	POS	CDAN1	Stromal_score	7.937819e−09	−0.3791454	2.106618e−04	NEG
1500	KIFC2	Stromal_score	1.128367e−06	−0.3233941	2.994572e−02	NEG	NGDN	Stromal_score	8.326104e−07	−0.3271442	2.209665e−02	NEG
1501	LMO3	Stromal_score	7.989425e−14	0.4786182	2.120314e−09	POS	MAP3K15	Stromal_score	7.475801e−10	−0.4023991	1.984003e−05	NEG
1502	PARVG	Stromal_score	4.382375e−08	0.3611159	1.163039e−03	POS	FAM181B	Stromal_score	2.380285e−07	0.3420871	6.317038e−03	POS
1503	MARCKSL1	Stromal_score	2.842897e−08	−0.3657884	7.544765e−04	NEG	SLC25A14	Stromal_score	2.128452e−07	−0.3433841	5.648699e−03	NEG
1504	PAPPA	Stromal_score	8.971112e−10	0.4006673	2.380843e−05	POS	TCF15	Stromal_score	4.123269e−07	0.3356273	1.094274e−02	POS
1505	PIK3IP1	Stromal_score	8.268309e−12	0.4423144	2.194326e−07	POS	ZNF354C	Stromal_score	4.867084e−13	0.4649839	1.291675e−08	POS
1506	PIK3CD	Stromal_score	1.319995e−14	0.4916275	3.503134e−10	POS	GEMIN2	Stromal_score	1.798118e−09	−0.3939702	4.772024e−05	NEG
1507	CCDC24	Stromal_score	1.369378e−09	−0.3966115	3.634194e−05	NEG	DDX24	Stromal_score	1.772226e−08	0.3708072	4.703311e−04	POS
1508	KCNA3	Stromal_score	1.318623e−07	0.3488722	3.499493e−03	POS	PSMD12	Stromal_score	1.715811e−06	−0.3181393	4.553590e−02	NEG
1509	FHL3	Stromal_score	2.107950e−14	0.4882980	5.594288e−10	POS	ZNF443	Stromal_score	8.680570e−13	−0.4604872	2.303737e−08	NEG
1510	MAPK13	Stromal_score	2.913484e−12	−0.4508584	7.732095e−08	NEG	AHCTF1	Stromal_score	7.307375e−09	−0.3799914	1.939304e−04	NEG
1511	DOK7	Stromal_score	3.085576e−09	−0.3886639	8.188810e−05	NEG	HIVEP1	Stromal_score	1.748677e−06	0.3178990	4.640814e−02	POS
1512	CAMK1D	Stromal_score	3.585755e−13	0.4673321	9.516234e−09	POS	LOC151009	Stromal_score	3.174497e−10	−0.4104038	8.424798e−06	NEG
1513	IL34	Stromal_score	2.108848e−10	0.4141528	5.596673e−06	POS	LOC401588	Stromal_score	8.904570e−13	−0.4602877	2.363184e−08	NEG
1514	SCUBE2	Stromal_score	4.055935e−12	0.4481742	1.076405e−07	POS	COX15	Stromal_score	1.320970e−06	−0.3214298	3.505721e−02	NEG
1515	NRXN2	Stromal_score	6.958888e−21	0.5797609	1.846819e−16	POS	GCFC1	Stromal_score	2.780641e−07	−0.3402741	7.379543e−03	NEG
1516	E2F7	Stromal_score	3.044806e−09	−0.3887957	8.080611e−05	NEG	GTF2H4	Stromal_score	9.732702e−07	−0.3252247	2.582962e−02	NEG
1517	PPP1R12C	Stromal_score	3.737900e−08	0.3628420	9.920012e−04	POS	CD1D	Stromal_score	3.862239e−09	0.3864297	1.025000e−04	POS
1518	PALM2−AKAP2	Stromal_score	2.365138e−28	0.6584921	6.276840e−24	POS	ZFP62	Stromal_score	2.437594e−07	−0.3418104	6.469132e−03	NEG
1519	CXCR7	Stromal_score	3.211160e−12	0.4500715	8.522097e−08	POS	TSEN2	Stromal_score	1.129646e−13	−0.4760511	2.997968e−09	NEG
1520	LINC00467	Stromal_score	2.529653e−07	−0.3413787	6.713445e−03	NEG	ARHGAP20	Stromal_score	3.274743e−29	0.6662392	8.690841e−25	POS
1521	CAND2	Stromal_score	2.405927e−15	0.5034399	6.385089e−11	POS	SNORD18B	Stromal_score	1.263433e−06	−0.3219863	3.353024e−02	NEG
1522	BC073817	Stromal_score	3.329982e−13	0.4678982	8.837439e−09	POS	GEMIN8P4	Stromal_score	1.226724e−09	−0.3976718	3.255602e−05	NEG
1523	C15orf42	Stromal_score	1.916165e−12	−0.4542248	5.085312e−08	NEG	MKS1	Stromal_score	3.761404e−12	−0.4487879	9.982390e−08	NEG
1524	RGS10	Stromal_score	4.932694e−07	0.3334878	1.309088e−02	POS	RRP15	Stromal_score	1.523543e−06	−0.3196395	4.043330e−02	NEG
1525	PIM1	Stromal_score	2.592048e−10	0.4122676	6.879035e−06	POS	FLJ42709	Stromal_score	1.156024e−17	0.5377691	3.067971e−13	POS
1526	OSBP2	Stromal_score	4.878591e−07	−0.3336199	1.294729e−02	NEG	C12orf65	Stromal_score	5.566869e−07	−0.3320348	1.477391e−02	NEG
1527	MNX1	Stromal_score	2.304871e−11	−0.4336784	6.116898e−07	NEG	EIF2B5	Stromal_score	1.234511e−07	−0.3496195	3.276270e−03	NEG
1528	MARVELD2	Stromal_score	1.780375e−10	−0.4156913	4.724938e−06	NEG	RPS17L	Stromal_score	1.872260e−10	−0.4152349	4.968790e−06	NEG
1529	RASA3	Stromal_score	3.424583e−07	0.3378267	9.088501e−03	POS	CAMK4	Stromal_score	8.408134e−07	0.3270240	2.231435e−02	POS
1530	OLFML1	Stromal_score	2.331087e−32	0.6927824	6.186471e−28	POS	BC030152	Stromal_score	1.180449e−10	−0.4193931	3.132793e−06	NEG
1531	SLC37A2	Stromal_score	4.681684e−07	0.3341129	1.242472e−02	POS	TRO	Stromal_score	3.512407e−23	0.6064130	9.321576e−19	POS
1532	CDH13	Stromal_score	2.152069e−08	0.3687553	5.711377e−04	POS	ARHGAP28	Stromal_score	2.007259e−08	0.3694930	5.327066e−04	POS
1533	RACGAP1	Stromal_score	3.571762e−11	−0.4299127	9.479099e−07	NEG	WRAP73	Stromal_score	3.465413e−12	−0.4494538	9.196859e−08	NEG
1534	MND1	Stromal_score	4.112629e−11	−0.4286906	1.091451e−06	NEG	CORO6	Stromal_score	6.894825e−08	0.3561414	1.829818e−03	POS
1535	RAB19	Stromal_score	7.337409e−07	−0.3286891	1.947275e−02	NEG	PIH1D2	Stromal_score	9.850507e−08	−0.3521645	2.614226e−03	NEG
1536	TMEM184A	Stromal_score	3.183853e−08	−0.3645727	8.449628e−04	NEG	SRSF9	Stromal_score	2.728105e−07	−0.3404971	7.240117e−03	NEG
1537	LAMB1	Stromal_score	1.517937e−11	0.4372263	4.028452e−07	POS	LTV1	Stromal_score	2.971563e−09	−0.3890369	7.886231e−05	NEG
1538	ARHGAP10	Stromal_score	3.990995e−12	0.4483057	1.059170e−07	POS	BC046483	Stromal_score	6.259136e−09	−0.3815677	1.661112e−04	NEG
1539	CREB3L4	Stromal_score	9.082598e−09	−0.3777631	2.410431e−04	NEG	PRKACA	Stromal_score	1.806800e−09	0.3939233	4.795068e−05	POS
1540	CD200	Stromal_score	4.158114e−25	0.6269884	1.103522e−20	POS	GUCY1A2	Stromal_score	5.957385e−08	0.3577549	1.581030e−03	POS
1541	ABHD3	Stromal_score	4.099398e−10	−0.4080364	1.087939e−05	NEG	ARNT	Stromal_score	2.218038e−07	0.3429066	5.886451e−03	POS
1542	ARHGEF6	Stromal_score	3.828846e−22	0.5946797	1.016138e−17	POS	LRRC70	Stromal_score	2.232111e−14	0.4878884	5.923798e−10	POS
1543	BC049825	Stromal_score	1.458958e−07	−0.3477217	3.871928e−03	NEG	RFT1	Stromal_score	4.006176e−10	−0.4082501	1.063199e−05	NEG
1544	UTRN	Stromal_score	1.006935e−14	0.4935367	2.672305e−10	POS	C15orf40	Stromal_score	8.172751e−07	−0.3273719	2.168966e−02	NEG
1545	TNFAIP8L3	Stromal_score	4.360861e−19	0.5570640	1.157329e−14	POS	NDUFS3	Stromal_score	1.138116e−06	−0.3232873	3.020445e−02	NEG
1546	HMCN2	Stromal_score	1.225768e−10	0.4190556	3.253065e−06	POS	TRPV1	Stromal_score	1.953554e−09	−0.3931616	5.184536e−05	NEG
1547	ASAP3	Stromal_score	1.021759e−10	0.4206830	2.711647e−06	POS	CPSF3	Stromal_score	4.865083e−08	−0.3599764	1.291144e−03	NEG
1548	PYHIN1	Stromal_score	1.386708e−06	0.3208217	3.680184e−02	POS	DNM3OS	Stromal_score	2.049134e−20	0.5740036	5.438196e−16	POS
1549	BHMT2	Stromal_score	2.831197e−19	0.5595166	7.513713e−15	POS	KLHL4	Stromal_score	6.933520e−17	0.5266757	1.840087e−12	POS
1550	MAP7	Stromal_score	5.481920e−18	−0.5422719	1.454847e−13	NEG	TINF2	Stromal_score	6.423058e−12	0.4444049	1.704615e−07	POS
1551	DCXR	Stromal_score	1.021648e−09	−0.3994265	2.711351e−05	NEG	LOC257396	Stromal_score	8.843366e−08	−0.3533726	2.346941e−03	NEG
1552	ST6GALNAC6	Stromal_score	1.210707e−24	0.6221687	3.213096e−20	POS	PHKG2	Stromal_score	8.908702e−10	−0.4007338	2.364281e−05	NEG
1553	LEPR	Stromal_score	5.600996e−14	0.4812284	1.486448e−09	POS	OAZ2	Stromal_score	1.290304e−17	0.5371002	3.424339e−13	POS
1554	RAD51AP1	Stromal_score	5.849707e−12	−0.4451753	1.552454e−07	NEG	UBLCP1	Stromal_score	2.976109e−07	0.3394782	7.898296e−03	POS
1555	KLHL5	Stromal_score	2.718237e−19	0.5597467	7.213929e−15	POS	ZNF408	Stromal_score	4.975312e−09	−0.3838893	1.320398e−04	NEG
1556	NXPH3	Stromal_score	4.573915e−13	0.4654628	1.213871e−08	POS	KIAA1731	Stromal_score	3.701223e−08	−0.3629487	9.822677e−04	NEG
1557	SOD2	Stromal_score	6.021125e−10	0.4044416	1.597946e−05	POS	COIL	Stromal_score	4.130308e−09	−0.3857586	1.096142e−04	NEG
1558	NOS3	Stromal_score	5.566978e−08	0.3585002	1.477420e−03	POS	ZIK1	Stromal_score	2.696058e−14	0.4865331	7.155068e−10	POS
1559	BCL6	Stromal_score	3.589510e−11	0.4298699	9.526200e−07	POS	ZYG11B	Stromal_score	1.457246e−10	0.4175011	3.867384e−06	POS
1560	SLC8A1	Stromal_score	7.242419e−23	0.6029093	1.922066e−18	POS	RNH1	Stromal_score	9.057739e−09	0.3777913	2.403833e−04	POS

	gene	feature	p.value	estimate	adj.p.value	COR.ID	gene	feature	p.value	estimate	adj.p.value	COR.ID
1561	KLF7	Stromal_score	2.232629e−12	0.4530010	5.925174e−08	POS	FAM161A	Stromal_score	4.906173e−07	−0.3335524	1.302049e−02	NEG
1562	LRP8	Stromal_score	1.862739e−08	−0.3702822	4.943523e−04	NEG	DDX10	Stromal_score	1.581383e−09	−0.3952186	4.196833e−05	NEG
1563	RBMS3	Stromal_score	2.175122e−28	0.6588250	5.772555e−24	POS	ZNF662	Stromal_score	4.056534e−13	0.4663861	1.076564e−08	POS
1564	MLF1IP	Stromal_score	3.389340e−13	−0.4677631	8.994971e−09	NEG	SGTB	Stromal_score	3.535762e−11	0.4300003	9.383559e−07	POS
1565	LOC100131825	Stromal_score	9.335997e−18	0.5390657	2.477680e−13	POS	AGPAT4	Stromal_score	2.223663e−07	0.3428772	5.901379e−03	POS
1566	NME1−NME2	Stromal_score	1.787006e−06	−0.3176242	4.742535e−02	NEG	THUMPD3	Stromal_score	1.486736e−06	−0.3199472	3.945648e−02	NEG
1567	ESRP2	Stromal_score	2.012052e−12	−0.4538344	5.339785e−08	NEG	CCDC43	Stromal_score	8.090311e−07	−0.3274961	2.147088e−02	NEG
1568	TMEM204	Stromal_score	2.064806e−27	0.6497310	5.479789e−23	POS	DIEXF	Stromal_score	5.389822e−11	−0.4263328	1.430405e−06	NEG
1569	EZH2	Stromal_score	2.460246e−10	−0.4127456	6.529246e−06	NEG	CEP95	Stromal_score	1.838974e−09	−0.3937513	4.880453e−05	NEG
1570	HDHD3	Stromal_score	1.040976e−07	−0.3515440	2.762646e−03	NEG	SH3GLB1	Stromal_score	4.153212e−07	0.3355413	1.102221e−02	POS
1571	FAM109A	Stromal_score	1.704494e−07	−0.3459432	4.523556e−03	NEG	CIRBP−AS1	Stromal_score	1.016667e−08	−0.3766012	2.698132e−04	NEG
1572	CHAF1B	Stromal_score	8.723274e−14	−0.4779689	2.315070e−09	NEG	HBS1L	Stromal_score	1.860915e−06	−0.3171098	4.938683e−02	NEG
1573	GPM6B	Stromal_score	2.006911e−07	0.3440637	5.326142e−03	POS	BC035465	Stromal_score	5.757557e−10	−0.4048624	1.527998e−05	NEG
1574	MCM10	Stromal_score	3.643239e−13	−0.4672103	9.668792e−09	NEG	TUT1	Stromal_score	1.289050e−09	−0.3971946	3.421010e−05	NEG
1575	CENPK	Stromal_score	1.757088e−08	−0.3708975	4.663135e−04	NEG	PRMT7	Stromal_score	8.228586e−07	−0.3272886	2.183784e−02	NEG
1576	KIF18A	Stromal_score	1.466938e−15	−0.5067894	3.893107e−11	NEG	AK097794	Stromal_score	2.793381e−08	−0.3659766	7.413354e−04	NEG
1577	ESAM	Stromal_score	6.816301e−11	0.4242708	1.808978e−06	POS	RFWD2	Stromal_score	9.312804e−07	−0.3257683	2.471525e−02	NEG
1578	TRPV2	Stromal_score	5.351687e−09	0.3831538	1.420284e−04	POS	ATP6V1E2	Stromal_score	1.306553e−06	−0.3215671	3.467462e−02	NEG
1579	SHROOM1	Stromal_score	7.193858e−08	−0.3556710	1.909178e−03	NEG	DIABLO	Stromal_score	8.152478e−08	−0.3542804	2.163586e−03	NEG
1580	MYO1G	Stromal_score	3.352049e−07	0.3380792	8.896004e−03	POS	FGF1	Stromal_score	6.135597e−13	0.4631918	1.628326e−08	POS
1581	SORBS3	Stromal_score	3.465010e−11	0.4301751	9.195789e−07	POS	MAGI2	Stromal_score	3.083279e−14	0.4855663	8.182713e−10	POS
1582	TPST1	Stromal_score	4.215637e−11	0.4284757	1.118788e−06	POS	ANKZF1	Stromal_score	2.760728e−14	−0.4863625	7.326695e−10	NEG
1583	LRRC8E	Stromal_score	2.224339e−07	−0.3428737	5.903173e−03	NEG	DDX12P	Stromal_score	1.413093e−13	−0.4743803	3.750207e−09	NEG
1584	CYYR1	Stromal_score	6.004508e−24	0.6147872	1.593536e−19	POS	RAD21−AS1	Stromal_score	1.836071e−07	−0.3450890	4.872748e−03	NEG
1585	DSCC1	Stromal_score	1.378154e−10	−0.4180034	3.657484e−06	NEG	GPR17	Stromal_score	1.371537e−07	0.3484252	3.639921e−03	POS
1586	PPAP2B	Stromal_score	9.277846e−17	0.5248330	2.462248e−12	POS	CAPZB	Stromal_score	1.661792e−09	0.3947372	4.410230e−05	POS
1587	ACACB	Stromal_score	7.516015e−08	0.3551847	1.994675e−03	POS	LOC100506599	Stromal_score	9.193112e−09	−0.3776387	2.439760e−04	NEG
1588	CMKLR1	Stromal_score	6.920373e−16	0.5118064	1.836598e−11	POS	LOC400456	Stromal_score	1.059702e−14	0.4931774	2.812344e−10	POS
1589	VGLL3	Stromal_score	6.472638e−24	0.6144363	1.717773e−19	POS	RASGRF2	Stromal_score	1.057121e−17	0.5383124	2.805493e−13	POS
1590	FLVCR1	Stromal_score	1.422512e−10	−0.4177184	3.775204e−06	NEG	FAM185A	Stromal_score	4.842476e−07	−0.3337089	1.285145e−02	NEG
1591	FGD5	Stromal_score	1.684197e−14	0.4898988	4.469690e−10	POS	NOX4	Stromal_score	6.218862e−08	0.3572815	1.650424e−03	POS
1592	MMEL1	Stromal_score	7.722550e−10	−0.4020914	2.049488e−05	NEG	FIZ1	Stromal_score	3.871630e−07	−0.3363752	1.027492e−02	NEG
1593	FAM54A	Stromal_score	1.680503e−16	−0.5210397	4.459886e−12	NEG	CNOT10	Stromal_score	4.327952e−11	−0.4282471	1.148595e−06	NEG
1594	GM2A	Stromal_score	6.046737e−10	0.4044017	1.604743e−05	POS	CDNF	Stromal_score	6.092766e−08	−0.3575074	1.616959e−03	NEG
1595	FBXO16	Stromal_score	1.412212e−06	−0.3205931	3.747870e−02	NEG	POLR2D	Stromal_score	4.183633e−09	−0.3856302	1.110294e−04	NEG
1596	SYDE1	Stromal_score	1.141212e−32	0.6952514	3.028663e−28	POS	ARHGAP22	Stromal_score	1.558097e−11	0.4370057	4.135034e−07	POS
1597	RFC3	Stromal_score	3.949077e−14	−0.4837753	1.048046e−09	NEG	INE2	Stromal_score	1.344792e−06	0.3212062	3.568943e−02	POS
1598	PKD1	Stromal_score	3.067005e−13	0.4685262	8.139524e−09	POS	PREX2	Stromal_score	1.065873e−12	0.4588758	2.828722e−08	POS
1599	C10orf54	Stromal_score	2.047808e−15	0.5045350	5.434679e−11	POS	C10orf128	Stromal_score	7.761234e−13	0.4613624	2.059754e−08	POS
1600	HAS1	Stromal_score	1.066317e−10	0.4203022	2.829899e−06	POS	LRRC4	Stromal_score	5.297846e−10	0.4056430	1.405995e−05	POS
1601	WISP2	Stromal_score	3.999879e−12	0.4482876	1.061528e−07	POS	ZRANB3	Stromal_score	1.236205e−06	−0.3222582	3.280764e−02	NEG
1602	SPC24	Stromal_score	8.964906e−11	−0.4218469	2.379196e−06	NEG	TUBD1	Stromal_score	2.710981e−09	−0.3899443	7.194673e−05	NEG
1603	FEZ1	Stromal_score	6.964677e−20	0.5673422	1.848356e−15	POS	PACRGL	Stromal_score	1.368756e−07	−0.3484482	3.632542e−03	NEG
1604	C16orf62	Stromal_score	5.696899e−13	0.4637669	1.511900e−08	POS	SMYD5	Stromal_score	8.590815e−09	−0.3783351	2.279916e−04	NEG
1605	CLIC2	Stromal_score	1.127979e−24	0.6224905	2.993543e−20	POS	IQCC	Stromal_score	2.181680e−16	−0.5193579	5.789961e−12	NEG
1606	PCDH18	Stromal_score	1.330104e−12	0.4571279	3.529962e−08	POS	AK055694	Stromal_score	6.325443e−07	−0.3304921	1.678709e−02	NEG
1607	SH3BP5	Stromal_score	7.980405e−25	0.6240584	2.117920e−20	POS	AX747325	Stromal_score	3.933356e−08	−0.3622900	1.043873e−03	NEG
1608	TRAF1	Stromal_score	8.117558e−10	0.4016181	2.154319e−05	POS	ZNF77	Stromal_score	2.166854e−14	−0.4881008	5.750614e−10	NEG
1609	MDFIC	Stromal_score	1.024434e−28	0.6617987	2.718745e−24	POS	POLG2	Stromal_score	1.894733e−08	−0.3701025	5.028432e−04	NEG
1610	LILRB1	Stromal_score	3.485840e−09	0.3874522	9.251070e−05	POS	PNO1	Stromal_score	8.937486e−09	−0.3779287	2.371919e−04	NEG
1611	CASC5	Stromal_score	2.432638e−09	−0.3910119	6.455977e−05	NEG	PLEKHM2	Stromal_score	2.085397e−14	0.4883749	5.534435e−10	POS
1612	FOXD2	Stromal_score	2.266205e−08	−0.3682068	6.014282e−04	NEG	THSD7A	Stromal_score	8.037566e−18	0.5399709	2.133090e−13	POS
1613	PIK3R5	Stromal_score	1.004223e−10	0.4208373	2.665107e−06	POS	LOC440434	Stromal_score	2.765678e−07	−0.3403372	7.339832e−03	NEG
1614	ITGAM	Stromal_score	1.202976e−08	0.3748588	3.192577e−04	POS	COMMD5	Stromal_score	1.038609e−06	−0.3244218	2.756364e−02	NEG
1615	SH3PXD2A	Stromal_score	1.009469e−13	0.4768870	2.679029e−09	POS	KLF8	Stromal_score	4.416787e−13	0.4657320	1.172171e−08	POS
1616	C20orf194	Stromal_score	2.416599e−11	0.4332737	6.413411e−07	POS	LRP5L	Stromal_score	1.145441e−08	−0.3753673	3.039887e−04	NEG
1617	LEF1	Stromal_score	1.671890e−08	0.3714202	4.437028e−04	POS	WDR75	Stromal_score	4.540767e−08	−0.3607292	1.205074e−03	NEG
1618	MICAL2	Stromal_score	2.139259e−07	0.3433255	5.677379e−03	POS	UCHL5	Stromal_score	5.854753e−13	−0.4635551	1.553793e−08	NEG
1619	PTPLA	Stromal_score	1.515038e−06	0.3197100	4.020758e−02	POS	CEP57L1	Stromal_score	1.248753e−09	−0.3975005	3.314065e−05	NEG
1620	FGF2	Stromal_score	1.541410e−17	0.5360146	4.090748e−13	POS	SIRT5	Stromal_score	1.140929e−15	−0.5084769	3.027912e−11	NEG
1621	EFS	Stromal_score	4.990772e−25	0.6261712	1.324501e−20	POS	DNAAF2	Stromal_score	1.657405e−08	−0.3715117	4.398586e−04	NEG
1622	ELANE	Stromal_score	1.121541e−07	0.3507043	2.976457e−03	POS	ZUFSP	Stromal_score	3.571982e−09	−0.3872091	9.479683e−05	NEG
1623	BC069212	Stromal_score	6.814799e−14	−0.4797896	1.808580e−09	NEG	SPAST	Stromal_score	3.557183e−07	−0.3373781	9.440408e−03	NEG
1624	ITGB3	Stromal_score	1.115083e−13	0.4761476	2.959319e−09	POS	DQ576853	Stromal_score	4.955428e−07	0.3334327	1.315121e−02	POS
1625	PSRC1	Stromal_score	5.205685e−12	−0.4461337	1.381537e−07	NEG	NOL10	Stromal_score	2.276143e−14	−0.4877485	6.040657e−10	NEG
1626	UBE2Q2	Stromal_score	6.205960e−08	0.3573044	1.647000e−03	POS	SNORD43	Stromal_score	1.477433e−06	−0.3200261	3.920960e−02	NEG
1627	ITGB1	Stromal_score	6.187180e−10	0.4041856	1.642016e−05	POS	PNMAL2	Stromal_score	7.800010e−24	0.6135625	2.070045e−19	POS
1628	RAB3IL1	Stromal_score	6.281645e−14	0.4803880	1.667086e−09	POS	DDX18	Stromal_score	8.384621e−07	−0.3270584	2.225195e−02	NEG
1629	TSPAN4	Stromal_score	5.693633e−23	0.6040790	1.511033e−18	POS	SNORD65	Stromal_score	3.299397e−12	−0.4498519	8.756270e−08	NEG
1630	RAD51	Stromal_score	2.587296e−12	−0.4518161	6.866425e−08	NEG	C3orf24	Stromal_score	9.276637e−15	−0.4941127	2.461927e−10	NEG
1631	PDE1B	Stromal_score	5.109703e−18	0.5426928	1.356064e−13	POS	RBM15	Stromal_score	7.428304e−08	−0.3553151	1.971398e−03	NEG
1632	FZD1	Stromal_score	2.660712e−17	0.5326575	7.061263e−13	POS	TRIM27	Stromal_score	9.236184e−08	−0.3528864	2.451191e−03	NEG
1633	GPR68	Stromal_score	1.429975e−12	0.4565544	3.795010e−08	POS	HTR2A	Stromal_score	7.595759e−21	0.5792983	2.015838e−16	POS
1634	MGC12982	Stromal_score	1.244795e−07	−0.3495256	3.303562e−03	NEG	DJ031130	Stromal_score	1.025950e−06	−0.3245735	2.722768e−02	NEG
1635	H2AFX	Stromal_score	1.013197e−07	−0.3518481	2.688925e−03	NEG	FGFBP3	Stromal_score	1.258939e−07	−0.3493976	3.341099e−03	NEG
1636	ARHGAP6	Stromal_score	1.094921e−06	0.3237676	2.905811e−02	POS	BC042378	Stromal_score	3.534926e−08	0.3634455	9.381341e−04	POS
1637	ZNF542	Stromal_score	1.386022e−20	0.5761010	3.678363e−16	POS	PCDHB12	Stromal_score	3.275418e−10	0.4101151	8.692631e−06	POS
1638	SMARCD3	Stromal_score	4.080650e−16	0.5152850	1.082964e−11	POS	PAR5	Stromal_score	1.361410e−14	0.4914089	3.613046e−10	POS
1639	FRRS1L	Stromal_score	3.576070e−11	0.4299023	9.490533e−07	POS	EVC2	Stromal_score	2.875108e−18	0.5461131	7.630249e−14	POS
1640	PNMAL1	Stromal_score	8.473696e−15	0.4947474	2.248834e−10	POS	LOC254128	Stromal_score	3.211922e−08	−0.3644783	8.524120e−04	NEG
1641	SNORD49A	Stromal_score	8.392520e−12	−0.4421905	2.227291e−07	NEG	ZNHIT3	Stromal_score	1.100516e−08	−0.3757819	2.920659e−04	NEG
1642	CENPE	Stromal_score	2.416233e−13	−0.4703398	6.412442e−09	NEG	SCYL3	Stromal_score	3.072795e−12	−0.4504280	8.154891e−08	NEG
1643	SNORD88B	Stromal_score	2.573834e−09	−0.3904564	6.830697e−05	NEG	BC069739	Stromal_score	3.697212e−10	−0.4089947	9.812031e−06	NEG
1644	RNASEH2A	Stromal_score	2.536116e−09	−0.3906018	6.730598e−05	NEG	ZNF835	Stromal_score	5.847083e−21	0.5806784	1.551757e−16	POS
1645	43348	Stromal_score	6.121923e−13	0.4632091	1.624697e−08	POS	AVPR2	Stromal_score	7.188018e−11	0.4238026	1.907628e−06	POS
1646	RNF130	Stromal_score	7.921535e−13	0.4612028	2.102296e−08	POS	CCDC57	Stromal_score	4.923966e−08	−0.3598449	1.306771e−03	NEG
1647	RAD54L	Stromal_score	6.841283e−14	−0.4797611	1.815608e−09	NEG	RASGRP4	Stromal_score	7.557292e−15	0.4955478	2.005630e−10	POS
1648	ROBO4	Stromal_score	1.391781e−13	0.4744940	3.693647e−09	POS	NRG2	Stromal_score	1.502866e−09	0.3957122	3.988457e−05	POS
1649	ITPR1	Stromal_score	4.264495e−20	0.5700312	1.131754e−15	POS	MACF1	Stromal_score	1.437486e−16	0.5220417	3.814945e−12	POS
1650	ATP1B2	Stromal_score	3.346283e−18	0.5452142	8.880701e−14	POS	TRIM45	Stromal_score	2.330258e−11	−0.4335848	6.184271e−07	NEG
1651	HIST1H4C	Stromal_score	5.745278e−09	−0.3824362	1.524739e−04	NEG	TNNT3	Stromal_score	9.246847e−08	0.3528735	2.454021e−03	POS
1652	BC032795	Stromal_score	1.812499e−06	−0.3174445	4.810192e−02	NEG	ABCA1	Stromal_score	4.114369e−18	0.5439860	1.091912e−13	POS
1653	ROBO1	Stromal_score	4.030248e−20	0.5703393	1.069587e−15	POS	ADAM1	Stromal_score	5.890468e−07	−0.3313535	1.563271e−02	NEG
1654	AKAP6	Stromal_score	1.415769e−10	0.4177611	3.757310e−06	POS	LOC644656	Stromal_score	2.307145e−13	−0.4706899	6.122931e−09	NEG
1655	RGL1	Stromal_score	4.610649e−23	0.6051008	1.223620e−18	POS	C1orf124	Stromal_score	4.772526e−10	−0.4066198	1.266581e−05	NEG
1656	SFXN2	Stromal_score	1.008898e−08	−0.3766804	2.677513e−04	NEG	SNX17	Stromal_score	5.086143e−07	0.3331205	1.349811e−02	POS
1657	KDR	Stromal_score	3.172538e−09	0.3883882	8.419599e−05	POS	IQCA1	Stromal_score	4.169981e−08	0.3616561	1.106671e−03	POS
1658	CELSR3	Stromal_score	8.099329e−12	−0.4424859	2.149481e−07	NEG	C16orf72	Stromal_score	2.034797e−07	0.3439044	5.400148e−03	POS
1659	PODNL1	Stromal_score	6.232968e−07	0.3306703	1.654167e−02	POS	TADA1	Stromal_score	1.743175e−15	−0.5056255	4.626212e−11	NEG
1660	PDGFC	Stromal_score	1.085585e−14	0.4930075	2.881035e−10	POS	PLEKHA8	Stromal_score	3.088139e−08	−0.3649008	8.195613e−04	NEG
1661	SLC16A13	Stromal_score	1.091925e−08	−0.3758630	2.897859e−04	NEG	C15orf44	Stromal_score	1.370749e−10	−0.4180519	3.637830e−06	NEG
1662	POC1A	Stromal_score	2.712046e−14	−0.4864905	7.197499e−10	NEG	RIC3	Stromal_score	8.137278e−11	0.4227060	2.159552e−06	POS
1663	FGF13	Stromal_score	1.306475e−08	0.3740003	3.467255e−04	POS	LINC00092	Stromal_score	7.042060e−08	0.3559074	1.868892e−03	POS
1664	LOC100129480	Stromal_score	1.024605e−06	−0.3245897	2.719198e−02	NEG	LOC407835	Stromal_score	3.026883e−08	−0.3651160	8.033046e−04	NEG
1665	ZNF331	Stromal_score	3.496185e−07	0.3375824	9.278526e−03	POS	DNAH11	Stromal_score	5.586213e−07	−0.3319931	1.482525e−02	NEG
1666	FKBP4	Stromal_score	5.734092e−08	−0.3581752	1.521771e−03	NEG	DNASE1L2	Stromal_score	1.201616e−08	−0.3748705	3.188968e−04	NEG
1667	IL6R	Stromal_score	1.319213e−06	0.3214465	3.501060e−02	POS	METTL15	Stromal_score	6.039635e−07	−0.3310514	1.602859e−02	NEG
1668	RBMS1	Stromal_score	9.162936e−13	0.4600636	2.431752e−08	POS	POLR3E	Stromal_score	4.041604e−09	−0.3859759	1.072601e−04	NEG
1669	FAM49A	Stromal_score	1.834630e−27	0.6502164	4.868925e−23	POS	ZNF641	Stromal_score	9.889111e−10	0.3997379	2.624471e−05	POS
1670	FAM78A	Stromal_score	2.957459e−07	0.3395519	7.848800e−03	POS	LOC154822	Stromal_score	1.245008e−08	0.3745019	3.304127e−04	POS
1671	DIAPH3	Stromal_score	3.931854e−10	−0.4084240	1.043475e−05	NEG	TMEM128	Stromal_score	1.131893e−06	−0.3233554	3.003930e−02	NEG
1672	CRMP1	Stromal_score	1.777379e−10	0.4157066	4.716985e−06	POS	SIN3B	Stromal_score	3.227437e−07	0.3385255	8.565295e−03	POS
1673	MPZL3	Stromal_score	1.581721e−10	−0.4167617	4.197730e−06	NEG	ENO1−AS1	Stromal_score	5.360326e−07	−0.3324898	1.422577e−02	NEG
1674	PDSS1	Stromal_score	2.114255e−08	−0.3689432	5.611022e−04	NEG	MMP16	Stromal_score	6.239882e−12	0.4446435	1.656002e−07	POS
1675	PDE7B	Stromal_score	1.033224e−20	0.5776670	2.742073e−16	POS	MAPKAPK5	Stromal_score	1.142471e−11	−0.4396168	3.032005e−07	NEG
1676	LZTS1	Stromal_score	7.480492e−19	0.5539715	1.985248e−14	POS	C14orf149	Stromal_score	5.946209e−08	0.3577755	1.578064e−03	POS
1677	GLI3	Stromal_score	2.433387e−33	0.7005092	6.457965e−29	POS	ACTR1A	Stromal_score	6.197325e−10	0.4041702	1.644708e−05	POS
1678	PRR11	Stromal_score	3.966059e−13	−0.4665593	1.052552e−08	NEG	AP1S2	Stromal_score	1.181209e−13	0.4757187	3.134809e−09	POS
1679	SYNDIG1	Stromal_score	9.763593e−10	0.3998599	2.591160e−05	POS	PDE11A	Stromal_score	2.026602e−07	−0.3439510	5.378399e−03	NEG
1680	SLC37A4	Stromal_score	2.221087e−07	−0.3428907	5.894542e−03	NEG	GTF3C3	Stromal_score	5.101001e−10	−0.4059976	1.353755e−05	NEG

	gene	feature	p.value	estimate	adj.p.value	COR.ID	gene	feature	p.value	estimate	adj.p.value	COR.ID
1681	SETD7	Stromal_score	3.173688e−10	0.4104062	8.422651e−06	POS	NARFL	Stromal_score	5.907637e−08	−0.3578472	1.567828e−03	NEG
1682	TIE1	Stromal_score	4.387884e−21	0.5821852	1.164501e−16	POS	C12orf60	Stromal_score	6.538684e−07	−0.3300903	1.735301e−02	NEG
1683	CHST2	Stromal_score	9.749999e−08	0.3522796	2.587552e−03	POS	RRP7B	Stromal_score	8.932991e−08	−0.3532599	2.370727e−03	NEG
1684	PCYT2	Stromal_score	7.625750e−13	−0.4614998	2.023798e−08	NEG	ANKMY1	Stromal_score	2.908981e−07	−0.3397457	7.720145e−03	NEG
1685	VSTM4	Stromal_score	1.208488e−33	0.7028534	3.207207e−29	POS	GOLGA2P5	Stromal_score	3.243270e−07	−0.3384679	8.607314e−03	NEG
1686	PDCD1LG2	Stromal_score	1.197318e−09	0.3979051	3.177561e−05	POS	INTS4	Stromal_score	1.371112e−07	−0.3484287	3.638793e−03	NEG
1687	LPPR2	Stromal_score	2.038939e−07	0.3438809	5.411140e−03	POS	DRG1	Stromal_score	1.073494e−06	−0.3240127	2.848945e−02	NEG
1688	LPPR4	Stromal_score	1.037171e−12	0.4590906	2.752547e−08	POS	AK2	Stromal_score	3.807768e−07	−0.3365724	1.010543e−02	NEG
1689	GPRASP1	Stromal_score	9.006510e−22	0.5903587	2.390238e−17	POS	GRIK3	Stromal_score	1.168961e−06	0.3229548	3.102306e−02	POS
1690	TEX30	Stromal_score	4.424777e−08	−0.3610111	1.174292e−03	NEG	VPS54	Stromal_score	2.869022e−07	−0.3399078	7.614099e−03	NEG
1691	BEND5	Stromal_score	8.050897e−15	0.4951056	2.136627e−10	POS	HIST1H4K	Stromal_score	7.479166e−07	−0.3284558	1.984896e−02	NEG
1692	IDS	Stromal_score	1.069873e−09	0.3989851	2.839336e−05	POS	CCDC18	Stromal_score	1.865293e−07	−0.3449073	4.950300e−03	NEG
1693	HELLS	Stromal_score	1.544536e−11	−0.4370795	4.099043e−07	NEG	STK11IP	Stromal_score	1.076664e−07	−0.3511646	2.857359e−03	NEG
1694	PPP1R18	Stromal_score	9.381588e−21	0.5781797	2.489780e−16	POS	C9orf47	Stromal_score	9.016632e−19	0.5528936	2.392924e−14	POS
1695	TSPAN9	Stromal_score	1.843830e−17	0.5349170	4.893340e−13	POS	AK131020	Stromal_score	2.968622e−07	−0.3395077	7.878427e−03	NEG
1696	KAZALD1	Stromal_score	7.800947e−13	−0.4613225	2.070293e−08	NEG	LOC100288846	Stromal_score	3.013007e−08	−0.3651653	7.996219e−04	NEG
1697	RTN1	Stromal_score	1.444233e−12	0.4564757	3.832851e−08	POS	OTUB1	Stromal_score	5.788154e−10	−0.4048126	1.536118e−05	NEG
1698	ENTPD6	Stromal_score	1.162522e−07	−0.3502991	3.085216e−03	NEG	PIK3R6	Stromal_score	7.377781e−09	0.3798935	1.957989e−04	POS
1699	PDGFB	Stromal_score	2.887939e−07	0.3398308	7.664302e−03	POS	HRC	Stromal_score	3.708059e−07	0.3368867	9.840817e−03	POS
1700	FAM180A	Stromal_score	3.916413e−22	0.5945663	1.039377e−17	POS	METTL13	Stromal_score	4.875694e−11	−0.4272089	1.293960e−06	NEG
1701	USP43	Stromal_score	2.451039e−10	−0.4127799	6.504812e−06	NEG	LOC100132774	Stromal_score	9.222379e−14	−0.4775571	2.447527e−09	NEG
1702	HIST1H2BO	Stromal_score	8.562828e−10	−0.4011106	2.272489e−05	NEG	RBM12B−AS1	Stromal_score	1.639712e−06	−0.3187129	4.351631e−02	NEG
1703	GPR162	Stromal_score	3.109839e−12	0.4503311	8.253202e−08	POS	ILKAP	Stromal_score	3.156680e−08	−0.3646649	8.377513e−04	NEG
1704	NREP	Stromal_score	5.324806e−17	0.5283363	1.413150e−12	POS	ZFYVE1	Stromal_score	4.697180e−12	0.4469757	1.246585e−07	POS
1705	TTC28	Stromal_score	3.878680e−26	0.6373938	1.029363e−21	POS	THSD7B	Stromal_score	7.003988e−07	0.3292553	1.858788e−02	POS
1706	TOMM34	Stromal_score	1.675956e−09	−0.3946547	4.447819e−05	NEG	C21orf7	Stromal_score	5.951913e−07	0.3312282	1.579578e−02	POS
1707	CYTH4	Stromal_score	2.183604e−09	0.3920723	5.795066e−05	POS	HINFP	Stromal_score	4.176587e−09	−0.3856471	1.108424e−04	NEG
1708	PLXNC1	Stromal_score	1.588952e−13	0.4735014	4.216920e−09	POS	CPSF4L	Stromal_score	1.687990e−07	−0.3460547	4.479757e−03	NEG
1709	GRASP	Stromal_score	4.871905e−18	0.5429778	1.292955e−13	POS	PGBD2	Stromal_score	2.419185e−11	−0.4332645	6.420274e−07	NEG
1710	GAL3ST4	Stromal_score	1.352221e−11	0.4382011	3.588659e−07	POS	ZNF202	Stromal_score	5.695961e−09	−0.3825235	1.511651e−04	NEG
1711	CYB5B	Stromal_score	6.337213e−08	−0.3570736	1.681833e−03	NEG	LGALS8−AS1	Stromal_score	3.362874e−07	−0.3380412	8.924731e−03	NEG
1712	RAP1GAP2	Stromal_score	2.817012e−08	−0.3658864	7.476069e−04	NEG	ATXN7L2	Stromal_score	1.670722e−11	−0.4364153	4.433928e−07	NEG
1713	LRRC17	Stromal_score	3.953385e−13	0.4665839	1.049189e−08	POS	FLJ46906	Stromal_score	1.285582e−06	−0.3217693	3.411806e−02	NEG
1714	LRRC25	Stromal_score	8.520637e−10	0.4011576	2.261292e−05	POS	ALS2	Stromal_score	5.075457e−10	−0.4060445	1.346976e−05	NEG
1715	SAMD4A	Stromal_score	8.953867e−16	0.5100953	2.376267e−11	POS	AF198444	Stromal_score	1.423504e−08	0.3731054	3.777838e−04	POS
1716	NOTCH2	Stromal_score	6.803718e−18	0.5409752	1.805639e−13	POS	C4orf21	Stromal_score	2.774083e−12	−0.4512542	7.362138e−08	NEG
1717	CDC25C	Stromal_score	5.527722e−16	−0.5132910	1.467002e−11	NEG	CEP152	Stromal_score	1.036092e−08	−0.3764058	2.749684e−04	NEG
1718	TRPS1	Stromal_score	1.072630e−13	0.4764363	2.846652e−09	POS	ZFP28	Stromal_score	9.015849e−16	0.5100494	2.392716e−11	POS
1719	AF070581	Stromal_score	1.534672e−06	0.3195479	4.072866e−02	POS	ZNF200	Stromal_score	1.010798e−08	−0.3766610	2.682557e−04	NEG
1720	CD1C	Stromal_score	2.825722e−09	0.3895348	7.499183e−05	POS	RTCA	Stromal_score	7.333866e−10	−0.4025806	1.946335e−05	NEG
1721	C1orf226	Stromal_score	5.000092e−07	−0.3333251	1.326974e−02	NEG	TAMM41	Stromal_score	2.848377e−07	−0.3399924	7.559307e−03	NEG
1722	GPC6	Stromal_score	1.821112e−22	0.5983816	4.833048e−18	POS	ASMT	Stromal_score	7.646230e−09	−0.3795283	2.029233e−04	NEG
1723	ZNF296	Stromal_score	2.291870e−07	−0.3425268	6.082393e−03	NEG	OGFOD2	Stromal_score	1.822779e−09	−0.3938375	4.837474e−05	NEG
1724	ARID5B	Stromal_score	1.607016e−11	0.4367443	4.264860e−07	POS	CNTLN	Stromal_score	1.424266e−06	0.3204865	3.779860e−02	POS
1725	RCC1	Stromal_score	3.863804e−11	−0.4292322	1.025415e−06	NEG	COPS7B	Stromal_score	1.312362e−07	−0.3489262	3.482879e−03	NEG
1726	HSP90AB1	Stromal_score	4.365095e−07	−0.3349487	1.158453e−02	NEG	SLC5A10	Stromal_score	3.967446e−10	−0.4083404	1.052920e−05	NEG
1727	MTHFD2	Stromal_score	6.021334e−08	−0.3576373	1.598002e−03	NEG	GLIS1	Stromal_score	6.941149e−07	0.3293649	1.842111e−02	POS
1728	LOC100233209	Stromal_score	3.605760e−07	0.3372177	9.569327e−03	POS	LOC388906	Stromal_score	1.692350e−07	−0.3460252	4.491326e−03	NEG
1729	GSG2	Stromal_score	2.116830e−11	−0.4344046	5.617855e−07	NEG	TMEM116	Stromal_score	2.099775e−08	−0.3690160	5.572593e−04	NEG
1730	MIR210HG	Stromal_score	8.954294e−08	−0.3532332	2.376380e−03	NEG	PRDM2	Stromal_score	1.340344e−07	0.3486866	3.557140e−03	POS
1731	C19orf57	Stromal_score	4.460112e−08	−0.3609245	1.183669e−03	NEG	BC017347	Stromal_score	3.876255e−07	−0.3363610	1.028719e−02	NEG
1732	LMCD1	Stromal_score	3.870762e−10	0.4085694	1.027261e−05	POS	SNORD42A	Stromal_score	3.980979e−08	−0.3621596	1.056512e−03	NEG
1733	ITGAV	Stromal_score	1.309989e−15	0.5075503	3.476579e−11	POS	UCKL1−AS1	Stromal_score	3.469660e−08	−0.3636466	9.208130e−04	NEG
1734	NTM	Stromal_score	2.904489e−08	0.3655588	7.708223e−04	POS	NT5DC4	Stromal_score	1.443401e−12	−0.4564803	3.830643e−08	NEG
1735	PYCRL	Stromal_score	1.066598e−07	−0.3512704	2.830643e−03	NEG	ZNF135	Stromal_score	2.041701e−30	0.6767346	5.418469e−26	POS
1736	NECAB1	Stromal_score	2.140165e−13	0.4712583	5.679783e−09	POS	BC071794	Stromal_score	2.280553e−07	−0.3425842	6.052359e−03	NEG
1737	GLYCTK	Stromal_score	6.045982e−10	−0.4044029	1.604543e−05	NEG	GFRA2	Stromal_score	2.718656e−11	0.4322644	7.215042e−07	POS
1738	KIF15	Stromal_score	1.074823e−15	−0.5088763	2.852474e−11	NEG	MTPAP	Stromal_score	1.076187e−09	−0.3989287	2.856094e−05	NEG
1739	RIN3	Stromal_score	8.500561e−12	0.4420842	2.255964e−07	POS	VPS39	Stromal_score	5.805188e−11	0.4256824	1.540639e−06	POS
1740	PJA2	Stromal_score	1.593031e−22	0.5990431	4.227745e−18	POS	TEFM	Stromal_score	1.957411e−14	−0.4888274	5.194774e−10	NEG
1741	PRG4	Stromal_score	1.996259e−07	0.3441252	5.297872e−03	POS	CPSF6	Stromal_score	6.296395e−08	−0.3571449	1.671000e−03	NEG
1742	ALDH1L2	Stromal_score	2.312958e−12	0.4527174	6.138360e−08	POS	LOC100192378	Stromal_score	1.621265e−15	0.5061151	4.302674e−11	POS
1743	PRICKLE1	Stromal_score	2.815803e−19	0.5595474	7.472859e−15	POS	MEMO1	Stromal_score	1.446407e−08	−0.3729386	3.838619e−04	NEG
1744	BLM	Stromal_score	9.153741e−14	−0.4776124	2.429311e−09	NEG	PEX13	Stromal_score	1.274604e−07	−0.3492574	3.382671e−03	NEG
1745	ZBTB4	Stromal_score	2.796156e−19	0.5595870	7.420718e−15	POS	BX647243	Stromal_score	1.312394e−09	−0.3970216	3.482962e−05	NEG
1746	CENPH	Stromal_score	4.371551e−07	−0.3349311	1.160166e−02	NEG	LOC100630918	Stromal_score	1.290522e−08	−0.3741283	3.424915e−04	NEG
1747	GIMAP8	Stromal_score	2.576228e−23	0.6079006	6.837051e−19	POS	SPDYE3	Stromal_score	5.674553e−10	−0.4049987	1.505970e−05	NEG
1748	PSPH	Stromal_score	1.692272e−11	−0.4363067	4.491122e−07	NEG	ZNF678	Stromal_score	3.813929e−12	−0.4486751	1.012179e−07	NEG
1749	NR3C1	Stromal_score	1.406573e−20	0.5760223	3.732904e−16	POS	LCN6	Stromal_score	2.045544e−10	0.4144303	5.428671e−06	POS
1750	FOXN3	Stromal_score	2.875177e−20	0.5721745	7.630432e−16	POS	ZNF7	Stromal_score	6.706559e−10	−0.4034257	1.779854e−05	NEG
1751	CNR1	Stromal_score	1.862495e−12	0.4544517	4.942876e−08	POS	NCAM2	Stromal_score	5.158136e−19	0.5561052	1.368918e−14	POS
1752	BX647792	Stromal_score	2.299256e−08	0.3680530	6.101995e−04	POS	LOC388152	Stromal_score	2.027986e−11	−0.4347699	5.382071e−07	NEG
1753	GNAL	Stromal_score	2.040048e−12	0.4537238	5.414083e−08	POS	RBBP5	Stromal_score	6.198098e−10	−0.4041690	1.644913e−05	NEG
1754	TCF19	Stromal_score	6.428485e−08	−0.3569157	1.706056e−03	NEG	FAM86EP	Stromal_score	3.622873e−14	−0.4844004	9.614742e−10	NEG
1755	MAB21L2	Stromal_score	5.482900e−08	0.3586672	1.455107e−03	POS	AK095267	Stromal_score	3.146442e−09	−0.3884701	8.350344e−05	NEG
1756	PLEKHO2	Stromal_score	5.778440e−32	0.6896080	1.533540e−27	POS	MIR17HG	Stromal_score	5.261868e−07	−0.3327127	1.396447e−02	NEG
1757	FEN1	Stromal_score	3.568146e−12	−0.4492166	9.469504e−08	NEG	TBC1D22B	Stromal_score	2.500354e−07	−0.3415144	6.635690e−03	NEG
1758	ZNF28	Stromal_score	7.930302e−08	−0.3545881	2.104623e−03	NEG	KIF20B	Stromal_score	3.918477e−11	−0.4291103	1.039925e−06	NEG
1759	NOVA1	Stromal_score	1.860273e−14	0.4891906	4.936980e−10	POS	LOC440288	Stromal_score	1.230029e−12	−0.4577464	3.264375e−08	NEG
1760	MIF	Stromal_score	2.083975e−07	−0.3436284	5.530661e−03	NEG	ATRIP	Stromal_score	2.021929e−10	−0.4145360	5.365998e−06	NEG
1761	SCN1B	Stromal_score	4.577993e−14	0.4827009	1.214953e−09	POS	TAF6L	Stromal_score	9.188790e−11	−0.4216277	2.438613e−06	NEG
1762	TNXB	Stromal_score	1.321376e−15	0.5074922	3.506798e−11	POS	C10orf88	Stromal_score	1.733526e−07	−0.3457494	4.600605e−03	NEG
1763	LPAR2	Stromal_score	5.978807e−11	−0.4254238	1.586715e−06	NEG	E2F6	Stromal_score	8.894473e−11	−0.4219169	2.360504e−06	NEG
1764	C1orf53	Stromal_score	3.739690e−11	−0.4295151	9.924763e−07	NEG	PPP2R2B	Stromal_score	4.721176e−12	0.4469340	1.252953e−07	POS
1765	MYO5C	Stromal_score	2.477158e−07	−0.3416229	6.574129e−03	NEG	PMS2	Stromal_score	1.045346e−08	−0.3763139	2.774243e−04	NEG
1766	CYSLTR1	Stromal_score	4.870109e−16	0.5141247	1.292478e−11	POS	HIST1H2AK	Stromal_score	1.417938e−11	−0.4378013	3.763066e−07	NEG
1767	PDE1A	Stromal_score	2.756359e−26	0.6388599	7.315102e−22	POS	DCTN1	Stromal_score	3.220360e−07	0.3385513	8.546514e−03	POS
1768	GLI1	Stromal_score	2.522809e−20	0.5728819	6.695282e−16	POS	SMG6	Stromal_score	5.223402e−13	0.4644383	1.386239e−08	POS
1769	SH2D3A	Stromal_score	9.504330e−07	−0.3255175	2.522354e−02	NEG	CDR1	Stromal_score	9.201058e−11	0.4216159	2.441869e−06	POS
1770	AK289390	Stromal_score	5.423079e−13	−0.4641482	1.439231e−08	NEG	LOC100130417	Stromal_score	8.125864e−11	0.4227184	2.156523e−06	POS
1771	ARHGAP39	Stromal_score	4.774594e−11	−0.4273917	1.267130e−06	NEG	TOMM20L	Stromal_score	1.442057e−07	−0.3478545	3.827075e−03	NEG
1772	CKS1B	Stromal_score	7.912370e−12	−0.4426797	2.099864e−07	NEG	KLHL12	Stromal_score	1.733662e−06	−0.3180082	4.600967e−02	NEG
1773	DARS2	Stromal_score	3.785942e−10	−0.4087749	1.004751e−05	NEG	PTPN5	Stromal_score	3.642735e−16	0.5160275	9.667453e−12	POS
1774	PYGO1	Stromal_score	3.769197e−13	0.4669499	1.000307e−08	POS	EIF3C	Stromal_score	4.429104e−10	−0.4073165	1.175440e−05	NEG
1775	CHD3	Stromal_score	9.145965e−14	0.4776187	2.427248e−09	POS	MTHFSD	Stromal_score	3.204320e−07	−0.3386101	8.503946e−03	NEG
1776	LOC100287482	Stromal_score	3.744844e−07	−0.3367698	9.938442e−03	NEG	MYOT	Stromal_score	1.122544e−07	0.3506942	2.979119e−03	POS
1777	RAI14	Stromal_score	1.342235e−13	0.4747651	3.562157e−09	POS	ZCCHC4	Stromal_score	1.600429e−10	−0.4166555	4.247377e−06	NEG
1778	OSBPL5	Stromal_score	1.193820e−06	0.3226929	3.168278e−02	POS	GATC	Stromal_score	2.502720e−09	−0.3907324	6.641970e−05	NEG
1779	SLC5A6	Stromal_score	1.541820e−08	−0.3722700	4.091837e−04	NEG	LOC100506686	Stromal_score	1.048568e−09	−0.3991777	2.782795e−05	NEG
1780	ZEB2	Stromal_score	2.163270e−38	0.7367002	5.741101e−34	POS	SATL1	Stromal_score	1.071425e−06	−0.3240366	2.843454e−02	NEG
1781	EIF4E3	Stromal_score	1.479781e−06	0.3200062	3.927191e−02	POS	FAM114A2	Stromal_score	9.667026e−08	0.3523755	2.565532e−03	POS
1782	SNORD36A	Stromal_score	8.721056e−07	−0.3265757	2.314481e−02	NEG	PMS2CL	Stromal_score	2.534242e−08	−0.3670169	6.725624e−04	NEG
1783	PRR19	Stromal_score	3.823100e−09	−0.3865314	1.014613e−04	NEG	ATP5S	Stromal_score	1.092022e−06	−0.3238005	2.898117e−02	NEG
1784	NFKBIA	Stromal_score	4.836556e−10	0.4064953	1.283574e−05	POS	C14orf93	Stromal_score	3.993960e−08	−0.3621242	1.059957e−03	NEG
1785	FYN	Stromal_score	6.783611e−14	0.4798233	1.800302e−09	POS	CLP1	Stromal_score	6.242331e−08	−0.3572400	1.656652e−03	NEG
1786	TBC1D9	Stromal_score	1.381838e−09	0.3965241	3.667260e−05	POS	DYNC2H1	Stromal_score	1.244683e−08	0.3745046	3.303263e−04	POS
1787	C15orf23	Stromal_score	1.487558e−13	−0.4739958	3.947831e−09	NEG	LRP12	Stromal_score	8.452714e−07	0.3269592	2.243266e−02	POS
1788	RTKN	Stromal_score	9.224091e−14	−0.4775557	2.447982e−09	NEG	HIST1H4D	Stromal_score	5.896757e−08	−0.3578675	1.564940e−03	NEG
1789	NCAPG2	Stromal_score	8.674429e−10	−0.4009875	2.302107e−05	NEG	LOC100505815	Stromal_score	2.761595e−07	−0.3403545	7.328997e−03	NEG
1790	RASIP1	Stromal_score	1.695999e−14	0.4898492	4.501012e−10	POS	VPS33A	Stromal_score	5.047450e−07	−0.3332121	1.339543e−02	NEG
1791	EHD3	Stromal_score	3.158406e−18	0.5455568	8.382095e−14	POS	MRPL30	Stromal_score	3.128161e−09	−0.3885280	8.301826e−05	NEG
1792	GJA5	Stromal_score	5.445794e−11	0.4262424	1.445259e−06	POS	SUFU	Stromal_score	4.824458e−08	0.3600680	1.280363e−03	POS
1793	HIPK3	Stromal_score	4.297085e−11	0.4283094	1.140403e−06	POS	TTC31	Stromal_score	3.340049e−07	−0.3381215	8.864156e−03	NEG
1794	TLR4	Stromal_score	1.218714e−12	0.4578194	3.234345e−08	POS	HIST1H3J	Stromal_score	6.995179e−08	−0.3559814	1.856451e−03	NEG
1795	SLC9A9	Stromal_score	8.550661e−28	0.6533308	2.269260e−23	POS	ZNF273	Stromal_score	5.018722e−09	−0.3838018	1.331919e−04	NEG
1796	TTYH2	Stromal_score	9.517296e−08	0.3525505	2.525795e−03	POS	USP42	Stromal_score	4.862310e−07	−0.3336599	1.290408e−02	NEG
1797	C17orf72	Stromal_score	1.228212e−06	0.3223390	3.259553e−02	POS	METTL19	Stromal_score	3.104340e−07	−0.3389829	8.238607e−03	NEG
1798	LMNB2	Stromal_score	4.417027e−08	−0.3610302	1.172235e−03	NEG	SETD4	Stromal_score	3.519428e−15	−0.5008397	9.340209e−11	NEG
1799	BCAN	Stromal_score	1.528815e−09	−0.3955463	4.057322e−05	NEG	ARL10	Stromal_score	7.268821e−14	0.4793150	1.929072e−09	POS
1800	FAM13C	Stromal_score	1.880644e−18	0.5486130	4.991041e−14	POS	GORAB	Stromal_score	1.584682e−08	−0.3719826	4.205588e−04	NEG

	gene	feature	p.value	estimate	adj.p.value	COR.ID	gene	feature	p.value	estimate	adj.p.value	COR.ID
1801	ZBTB20	Stromal_score	2.115603e−12	0.4534326	5.614598e−08	POS	DCUN1D5	Stromal_score	1.872338e−08	−0.3702280	4.968997e−04	NEG
1802	GAS2L3	Stromal_score	1.853435e−06	−0.3171609	4.918831e−02	NEG	BOP1	Stromal_score	6.525501e−10	−0.4036839	1.731803e−05	NEG
1803	LIG1	Stromal_score	1.370325e−07	−0.3484352	3.636706e−03	NEG	WASH3P	Stromal_score	4.390540e−07	−0.3348795	1.165205e−02	NEG
1804	GINS4	Stromal_score	1.104665e−11	−0.4398986	2.931670e−07	NEG	RFTN2	Stromal_score	7.257043e−17	0.5263880	1.925947e−12	POS
1805	DUSP3	Stromal_score	4.327032e−08	0.3612542	1.148351e−03	POS	HPS4	Stromal_score	7.744984e−09	−0.3793971	2.055441e−04	NEG
1806	TNFAIP8L2	Stromal_score	3.207263e−09	0.3882802	8.511756e−05	POS	HDAC8	Stromal_score	2.586010e−09	−0.3904098	6.863012e−05	NEG
1807	ZFP36L1	Stromal_score	3.159749e−10	0.4104468	8.385658e−06	POS	C12orf47	Stromal_score	9.521635e−11	−0.4213113	2.526947e−06	NEG
1808	FZD4	Stromal_score	5.672321e−22	0.5927023	1.505377e−17	POS	WDR89	Stromal_score	1.543346e−07	−0.3470800	4.095886e−03	NEG
1809	C11orf52	Stromal_score	5.897463e−07	−0.3313392	1.565128e−02	NEG	SNRPN	Stromal_score	4.093631e−13	0.4663162	1.086409e−08	POS
1810	PLEKHA7	Stromal_score	6.329318e−08	−0.3570873	1.679738e−03	NEG	PP2D1	Stromal_score	1.636204e−07	−0.3464118	4.342322e−03	NEG
1811	TTLL12	Stromal_score	1.033934e−07	−0.3516204	2.743958e−03	NEG	ZNF20	Stromal_score	1.677849e−06	−0.3184223	4.452844e−02	NEG
1812	APOO	Stromal_score	1.518258e−07	−0.3472672	4.029305e−03	NEG	SLC26A10	Stromal_score	1.680294e−06	0.3184039	4.459332e−02	POS
1813	FGR	Stromal_score	2.453531e−07	0.3417345	6.511426e−03	POS	LOC541472	Stromal_score	1.073703e−06	0.3240102	2.849501e−02	POS
1814	CSF1	Stromal_score	2.110287e−16	0.5195728	5.600491e−12	POS	MAGEL2	Stromal_score	7.707412e−08	0.3549053	2.045470e−03	POS
1815	PXMP2	Stromal_score	1.693827e−09	−0.3945516	4.495249e−05	NEG	KLC1	Stromal_score	1.728364e−06	0.3180470	4.586906e−02	POS
1816	FAM83G	Stromal_score	6.038587e−07	−0.3310535	1.602581e−02	NEG	ZNF418	Stromal_score	3.531991e−10	0.4094180	9.373550e−06	POS
1817	MST1P2	Stromal_score	3.953381e−07	−0.3361272	1.049188e−02	NEG	AL832593	Stromal_score	7.763350e−10	−0.4020415	2.060315e−05	NEG
1818	THSD4	Stromal_score	7.338614e−07	0.3286871	1.947595e−02	POS	ANAPC7	Stromal_score	4.003846e−08	−0.3620974	1.062581e−03	NEG
1819	GLI2	Stromal_score	6.226979e−17	0.5273529	1.652578e−12	POS	SMPD4	Stromal_score	1.051570e−07	−0.3514301	2.790761e−03	NEG
1820	AX747246	Stromal_score	1.555139e−08	0.3721799	4.127182e−04	POS	FAM190A	Stromal_score	1.280128e−07	−0.3492084	3.397332e−03	NEG
1821	SAPCD1	Stromal_score	1.147352e−10	−0.4196476	3.044956e−06	NEG	GHR	Stromal_score	7.575365e−21	0.5793125	2.010426e−16	POS
1822	DCLK1	Stromal_score	2.500267e−23	0.6080438	6.635459e−19	POS	CSTF3	Stromal_score	1.543134e−06	−0.3194786	4.095322e−02	NEG
1823	SLC50A1	Stromal_score	8.619865e−07	−0.3267190	2.287626e−02	NEG	ZCCHC8	Stromal_score	9.448475e−09	−0.3773567	2.507531e−04	NEG
1824	ZNF467	Stromal_score	1.182619e−10	0.4193767	3.138551e−06	POS	FLJ38668	Stromal_score	3.076794e−10	−0.4106920	8.165504e−06	NEG
1825	HSPD1	Stromal_score	2.678262e−12	−0.4515377	7.107839e−08	NEG	SRSF1	Stromal_score	1.909195e−07	−0.3446394	5.066812e−03	NEG
1826	STX3	Stromal_score	3.777017e−13	−0.4669340	1.002382e−08	NEG	SPICE1	Stromal_score	1.919421e−09	−0.3933337	5.093952e−05	NEG
1827	ARL6IP1	Stromal_score	1.772819e−08	−0.3708037	4.704885e−04	NEG	FANCM	Stromal_score	1.220094e−09	−0.3977239	3.238008e−05	NEG
1828	SGOL1	Stromal_score	3.331942e−21	−0.5836228	8.842642e−17	NEG	LOC286367	Stromal_score	5.535248e−07	0.3321034	1.468999e−02	POS
1829	CALCOCO1	Stromal_score	3.761478e−10	0.4088350	9.982586e−06	POS	RUNDC3B	Stromal_score	6.155308e−10	0.4042342	1.633557e−05	POS
1830	DQ582443	Stromal_score	5.403611e−16	0.5134406	1.434064e−11	POS	LOC100616668	Stromal_score	2.458406e−13	−0.4702086	6.524364e−09	NEG
1831	GPR116	Stromal_score	8.668314e−15	0.4945883	2.300484e−10	POS	VTI1A	Stromal_score	1.752427e−07	−0.3456249	4.650767e−03	NEG
1832	VAMP2	Stromal_score	1.258787e−08	0.3743874	3.340694e−04	POS	ODZ4	Stromal_score	7.606564e−11	0.4233027	2.018706e−06	POS
1833	LRRC56	Stromal_score	2.497358e−09	−0.3907535	6.627737e−05	NEG	LOC145820	Stromal_score	7.913348e−12	0.4426787	2.100124e−07	POS
1834	LOC729970	Stromal_score	1.765198e−07	0.3455415	4.684659e−03	POS	TMEM81	Stromal_score	1.073181e−07	−0.3512011	2.848115e−03	NEG
1835	LSAMP	Stromal_score	4.745815e−19	0.5565813	1.259492e−14	POS	HARBI1	Stromal_score	1.793776e−09	−0.3939937	4.760503e−05	NEG
1836	ZBTB47	Stromal_score	1.310881e−18	0.5507222	3.478946e−14	POS	FAM86HP	Stromal_score	6.284231e−09	−0.3815271	1.667772e−04	NEG
1837	PRRG2	Stromal_score	3.385141e−10	−0.4098107	8.983826e−06	NEG	KCNIP1	Stromal_score	3.021759e−13	0.4686396	8.019446e−09	POS
1838	NXT2	Stromal_score	4.155476e−07	−0.3355348	1.102822e−02	NEG	PRDM10	Stromal_score	2.263201e−08	−0.3682209	6.006309e−04	NEG
1839	GALNTL2	Stromal_score	2.249448e−14	0.4878330	5.969809e−10	POS	POLDIP2	Stromal_score	4.390750e−07	−0.3348789	1.165261e−02	NEG
1840	KIAA1522	Stromal_score	7.985150e−07	−0.3276561	2.119179e−02	NEG	ANAPC1	Stromal_score	1.704839e−10	−0.4160840	4.524472e−06	NEG
1841	PRR24	Stromal_score	6.221386e−12	0.4446680	1.651094e−07	POS	PRPF40A	Stromal_score	7.531625e−10	−0.4023287	1.998818e−05	NEG
1842	KIAA1804	Stromal_score	2.776090e−16	−0.5177971	7.367465e−12	NEG	AY927515	Stromal_score	8.079191e−12	0.4425066	2.144136e−07	POS
1843	NUMBL	Stromal_score	1.120524e−17	0.5379587	2.973758e−13	POS	CDH23	Stromal_score	1.181922e−08	0.3750421	3.136702e−04	POS
1844	ARRDC3	Stromal_score	4.976112e−10	0.4062295	1.320610e−05	POS	HIST2H2BC	Stromal_score	1.486647e−08	−0.3726516	3.945412e−04	NEG
1845	SH2D3C	Stromal_score	2.042134e−21	0.5861619	5.419620e−17	POS	HIST2H2BA	Stromal_score	9.122594e−07	0.3260224	2.421045e−02	POS
1846	SEMA3G	Stromal_score	1.118499e−13	0.4761248	2.968386e−09	POS	TMEM234	Stromal_score	8.697715e−08	−0.3535582	2.308287e−03	NEG
1847	AX748080	Stromal_score	2.952983e−14	0.4858776	7.836922e−10	POS	ST6GAL2	Stromal_score	8.405201e−15	0.4948042	2.230656e−10	POS
1848	SPR	Stromal_score	1.012828e−09	−0.3995094	2.687945e−05	NEG	IQSEC3	Stromal_score	3.389850e−07	0.3379470	8.996323e−03	POS
1849	ECM2	Stromal_score	1.964268e−31	0.6852637	5.212970e−27	POS	ZNRD1−AS1	Stromal_score	4.366454e−10	−0.4074492	1.158813e−05	NEG
1850	ARHGAP31	Stromal_score	6.734779e−31	0.6808111	1.787343e−26	POS	PPIL6	Stromal_score	1.740456e−11	−0.4360688	4.618995e−07	NEG
1851	SOCS2	Stromal_score	9.295520e−09	0.3775247	2.466938e−04	POS	AGAP5	Stromal_score	4.884494e−07	−0.3336054	1.296296e−02	NEG
1852	C2CD4D	Stromal_score	8.317909e−08	−0.3540565	2.207490e−03	NEG	ZSCAN29	Stromal_score	6.729932e−07	−0.3297404	1.786057e−02	NEG
1853	FAM20A	Stromal_score	5.096897e−16	0.5138254	1.352666e−11	POS	FAM86C1	Stromal_score	4.657111e−09	−0.3845544	1.235951e−04	NEG
1854	VEGFB	Stromal_score	3.379135e−13	0.4677862	8.967885e−09	POS	ZMYM1	Stromal_score	2.112223e−07	−0.3434727	5.605629e−03	NEG
1855	PPIF	Stromal_score	4.786856e−07	−0.3338471	1.270384e−02	NEG	C8orf46	Stromal_score	3.699193e−08	0.3629546	9.817288e−04	POS
1856	FLT3LG	Stromal_score	4.083466e−07	0.3357426	1.083711e−02	POS	C2orf44	Stromal_score	2.922997e−07	−0.3396894	7.757341e−03	NEG
1857	KIAA0922	Stromal_score	2.402447e−12	0.4524124	6.375853e−08	POS	PCA3_1	Stromal_score	5.187752e−07	0.3328830	1.376778e−02	POS
1858	KAL1	Stromal_score	1.292585e−09	0.3971682	3.430391e−05	POS	NAV3	Stromal_score	2.367539e−18	0.5472596	6.283211e−14	POS
1859	SCIMP	Stromal_score	6.342985e−11	0.4249043	1.683365e−06	POS	ZNF167	Stromal_score	3.109187e−09	0.3885883	8.251473e−05	POS
1860	LIPT2	Stromal_score	3.056012e−09	−0.3887593	8.110350e−05	NEG	CYP11A1	Stromal_score	1.660726e−06	0.3185520	4.407400e−02	POS
1861	SERPINH1	Stromal_score	9.358302e−09	0.3774554	2.483600e−04	POS	GPATCH2	Stromal_score	3.672054e−10	−0.4090580	9.745265e−06	NEG
1862	C15orf59	Stromal_score	1.462101e−17	0.5363375	3.880271e−13	POS	PLEKHA8P1	Stromal_score	1.756844e−07	−0.3455960	4.662488e−03	NEG
1863	NFATC4	Stromal_score	4.571413e−20	0.5696517	1.213207e−15	POS	KCNA1	Stromal_score	1.237800e−11	0.4389445	3.284997e−07	POS
1864	DNA2	Stromal_score	2.250541e−14	−0.4878295	5.972710e−10	NEG	PLDN	Stromal_score	1.737364e−06	0.3179812	4.610791e−02	POS
1865	ILK	Stromal_score	9.723148e−18	0.5388196	2.580426e−13	POS	SNORD72	Stromal_score	3.189642e−07	−0.3386641	8.464991e−03	NEG
1866	ORC6	Stromal_score	4.971962e−16	−0.5139886	1.319509e−11	NEG	LOC100527964	Stromal_score	2.803467e−14	−0.4862520	7.440121e−10	NEG
1867	ZNF367	Stromal_score	1.794502e−10	−0.4156197	4.762429e−06	NEG	CHODL	Stromal_score	7.044765e−11	0.4239802	1.869610e−06	POS
1868	HMGCR	Stromal_score	9.692748e−09	−0.3770937	2.572358e−04	NEG	KRT222	Stromal_score	4.255699e−11	0.4283935	1.129420e−06	POS
1869	LDB2	Stromal_score	2.642258e−22	0.5965335	7.012288e−18	POS	DCAF5	Stromal_score	5.683028e−08	0.3582735	1.508219e−03	POS
1870	JAZF1	Stromal_score	1.561026e−16	0.5215132	4.142808e−12	POS	KIN	Stromal_score	1.393715e−07	−0.3482427	3.698782e−03	NEG
1871	C8ORFK29	Stromal_score	2.893374e−11	−0.4317293	7.678726e−07	NEG	AK125532	Stromal_score	1.296786e−10	0.4185503	3.441540e−06	POS
1872	LANCL2	Stromal_score	1.023236e−06	−0.3246062	2.715565e−02	NEG	LOC541467	Stromal_score	6.566729e−12	−0.4442224	1.742744e−07	NEG
1873	H6PD	Stromal_score	1.091943e−09	0.3987894	2.897907e−05	POS	GPN1	Stromal_score	3.633434e−08	−0.3631485	9.642770e−04	NEG
1874	BC044939	Stromal_score	1.011664e−06	−0.3247469	2.684856e−02	NEG	CYP2D7P1	Stromal_score	1.288102e−08	−0.3741478	3.418495e−04	NEG
1875	AACS	Stromal_score	1.675061e−07	−0.3461429	4.445446e−03	NEG	MIR1227	Stromal_score	1.941607e−08	−0.3698445	5.152830e−04	NEG
1876	TNN	Stromal_score	1.976299e−08	0.3696573	5.244899e−04	POS	CBWD2	Stromal_score	4.432170e−17	−0.5294852	1.176254e−12	NEG
1877	TLE4	Stromal_score	7.855789e−09	0.3792518	2.084848e−04	POS	DQ582057	Stromal_score	5.462403e−08	0.3587083	1.449667e−03	POS
1878	CHRNA5	Stromal_score	1.532471e−08	−0.3723338	4.067024e−04	NEG	AAK1	Stromal_score	2.193320e−07	0.3430365	5.820852e−03	POS
1879	FAM167B	Stromal_score	1.395096e−09	0.3964319	3.702444e−05	POS	FASTKD2	Stromal_score	3.179681e−09	−0.3883659	8.438557e−05	NEG
1880	ZNF521	Stromal_score	1.074313e−30	0.6791027	2.851119e−26	POS	FRMPD3	Stromal_score	1.834501e−11	0.4356224	4.868583e−07	POS
1881	ELL2	Stromal_score	1.614406e−08	0.3717876	4.284471e−04	POS	GDF6	Stromal_score	7.596371e−18	0.5403115	2.016001e−13	POS
1882	C10orf95	Stromal_score	3.387540e−12	−0.4496382	8.990191e−08	NEG	AK021804	Stromal_score	3.207632e−21	0.5838208	8.512735e−17	POS
1883	FBXL6	Stromal_score	1.545720e−10	−0.4169696	4.102186e−06	NEG	AX747870	Stromal_score	8.977871e−07	−0.3262191	2.382637e−02	NEG
1884	F11R	Stromal_score	1.205023e−07	−0.3498932	3.198012e−03	NEG	NUDT10	Stromal_score	9.903785e−22	0.5898750	2.628365e−17	POS
1885	SNRPD1	Stromal_score	1.291104e−08	−0.3741236	3.426461e−04	NEG	GTF3C2	Stromal_score	2.182231e−07	−0.3430952	5.791423e−03	NEG
1886	WDR62	Stromal_score	1.378162e−09	−0.3965498	3.657505e−05	NEG	TPO	Stromal_score	7.178672e−10	0.4027830	1.905148e−05	POS
1887	YOD1	Stromal_score	2.911666e−09	−0.3892385	7.727272e−05	NEG	DPY19L2	Stromal_score	8.379432e−10	0.4013165	2.223818e−05	POS
1888	PLS3	Stromal_score	1.575480e−08	0.3720437	4.181167e−04	POS	LOC390940	Stromal_score	2.778950e−10	0.4116287	7.375055e−06	POS
1889	PPP1R16A	Stromal_score	4.788060e−12	−0.4468189	1.270703e−07	NEG	SLC35B4	Stromal_score	6.399910e−07	0.3303503	1.698472e−02	POS
1890	TCF21	Stromal_score	3.252884e−19	0.5587305	8.632829e−15	POS	ZNF341	Stromal_score	9.935915e−14	−0.4770047	2.636892e−09	NEG
1891	BCL6B	Stromal_score	8.180525e−16	0.5106964	2.171030e−11	POS	SCN9A	Stromal_score	5.571925e−09	0.3827462	1.478733e−04	POS
1892	C1orf95	Stromal_score	7.799383e−15	0.4953275	2.069878e−10	POS	LOC100506660	Stromal_score	7.017491e−12	−0.4436738	1.862372e−07	NEG
1893	KLF12	Stromal_score	2.239302e−08	0.3683337	5.942885e−04	POS	USP39	Stromal_score	2.413542e−09	−0.3910894	6.405298e−05	NEG
1894	PLK4	Stromal_score	2.883917e−14	−0.4860482	7.653627e−10	NEG	HAUS3	Stromal_score	1.162287e−07	−0.3503014	3.084594e−03	NEG
1895	AX747485	Stromal_score	4.537129e−15	−0.4990910	1.204109e−10	NEG	LACE1	Stromal_score	4.090119e−07	−0.3357233	1.085477e−02	NEG
1896	MID2	Stromal_score	4.116524e−11	0.4286824	1.092484e−06	POS	ASTN1	Stromal_score	1.360335e−09	0.3966755	3.610192e−05	POS
1897	ASUN	Stromal_score	4.864560e−08	−0.3599776	1.291006e−03	NEG	TBC1D3F	Stromal_score	1.014247e−09	−0.3994961	2.691709e−05	NEG
1898	TOMM40	Stromal_score	1.563175e−08	−0.3721259	4.148509e−04	NEG	LOC100507392	Stromal_score	2.826914e−12	0.4511020	7.502347e−08	POS
1899	AK096792	Stromal_score	3.546548e−09	0.3872802	9.412184e−05	POS	FPGT−TNNI3K	Stromal_score	7.106754e−08	−0.3558060	1.886061e−03	NEG
1900	MZT1	Stromal_score	9.834846e−11	−0.4210232	2.610070e−06	NEG	DDX20	Stromal_score	2.961709e−08	−0.3653496	7.860081e−04	NEG
1901	CRTAP	Stromal_score	3.015905e−15	0.5018979	8.003911e−11	POS	AK026244	Stromal_score	5.819399e−09	−0.3823064	1.544410e−04	NEG
1902	LOC100505761	Stromal_score	4.536655e−11	−0.4278372	1.203983e−06	NEG	MCM3AP−AS1	Stromal_score	6.493120e−09	−0.3811948	1.723209e−04	NEG
1903	FAM60A	Stromal_score	3.483257e−09	−0.3874595	9.244217e−05	NEG	LINC00087	Stromal_score	5.218959e−07	0.3328110	1.385059e−02	POS
1904	DDX11	Stromal_score	3.783266e−12	−0.4487408	1.004041e−07	NEG	TRIM9	Stromal_score	3.210158e−08	0.3644842	8.519438e−04	POS
1905	MIS18A	Stromal_score	2.157122e−13	−0.4711987	5.724785e−09	NEG	LINC00602	Stromal_score	1.430549e−09	0.3961893	3.796535e−05	POS
1906	C1RL	Stromal_score	1.824108e−16	0.5205123	4.840999e−12	POS	PGR	Stromal_score	2.132331e−16	0.5195057	5.658992e−12	POS
1907	C16orf59	Stromal_score	7.157429e−14	−0.4794287	1.899510e−09	NEG	LOC143666	Stromal_score	1.158098e−08	−0.3752534	3.073475e−04	NEG
1908	SPAG5	Stromal_score	6.491462e−15	−0.4966078	1.722769e−10	NEG	GPR89A	Stromal_score	9.184387e−17	−0.5248973	2.437445e−12	NEG
1909	SCN7A	Stromal_score	9.046654e−18	0.5392562	2.400891e−13	POS	BRF1	Stromal_score	1.543421e−06	−0.3194763	4.096086e−02	NEG
1910	MEG3	Stromal_score	5.666301e−16	0.5131278	1.503780e−11	POS	DQ586551	Stromal_score	3.805721e−08	0.3626474	1.010000e−03	POS
1911	SLC52A2	Stromal_score	5.818372e−09	−0.3823082	1.544138e−04	NEG	USP51	Stromal_score	5.642996e−17	0.5279720	1.497595e−12	POS
1912	C20orf20	Stromal_score	1.360081e−09	−0.3966773	3.609519e−05	NEG	MRPL45P2	Stromal_score	3.380975e−08	−0.3639259	8.972770e−04	NEG
1913	RPS6KA2	Stromal_score	3.054606e−10	0.4107587	8.106619e−06	POS	ST8SIA1	Stromal_score	1.138520e−18	0.5515420	3.021519e−14	POS
1914	CIB1	Stromal_score	1.365444e−06	−0.3210153	3.623751e−02	NEG	MORN1	Stromal_score	1.455195e−07	−0.3477511	3.861941e−03	NEG
1915	CMA1	Stromal_score	8.551624e−09	0.3783820	2.269516e−04	POS	CHTOP	Stromal_score	4.093765e−12	−0.4480985	1.086444e−07	NEG
1916	RAB3IP	Stromal_score	1.113083e−16	−0.5236753	2.954010e−12	NEG	NUDT11	Stromal_score	1.173652e−17	0.5376771	3.114754e−13	POS
1917	SNHG1	Stromal_score	5.740098e−12	−0.4453309	1.523365e−07	NEG	PRPF38A	Stromal_score	2.246810e−10	−0.4135750	5.962810e−06	NEG
1918	MMD	Stromal_score	2.122098e−09	0.3923523	5.631835e−05	POS	MRPL19	Stromal_score	1.294369e−06	−0.3216842	3.435126e−02	NEG
1919	SMAD6	Stromal_score	8.306803e−08	−0.3540714	2.204542e−03	NEG	KERA	Stromal_score	2.435593e−13	0.4702793	6.463819e−09	POS
1920	SMARCA2	Stromal_score	1.537001e−09	0.3954946	4.079047e−05	POS	AK095439	Stromal_score	8.736043e−12	0.4418570	2.318458e−07	POS

	gene	feature	p.value	estimate	adj.p.value	COR.ID	gene	feature	p.value	estimate	adj.p.value	COR.ID
1921	TMEM150C	Stromal_score	8.997651e−08	0.3531792	2.387887e−03	POS	MGARP	Stromal_score	6.289743e−07	0.3305606	1.669235e−02	POS
1922	KLF10	Stromal_score	1.214475e−11	0.4391042	3.223096e−07	POS	TTC4	Stromal_score	9.857034e−08	−0.3521571	2.615958e−03	NEG
1923	PLAGL1	Stromal_score	7.615234e−07	0.3282358	2.021007e−02	POS	CATSPER2	Stromal_score	4.475429e−07	−0.3346510	1.187734e−02	NEG
1924	STK39	Stromal_score	1.364868e−06	−0.3210206	3.622222e−02	NEG	SNORA64	Stromal_score	1.066264e−07	−0.3512739	2.829758e−03	NEG
1925	MVD	Stromal_score	5.669186e−07	−0.3318154	1.504545e−02	NEG	AK094325	Stromal_score	6.228624e−07	−0.3306788	1.653014e−02	NEG
1926	SHANK3	Stromal_score	1.093623e−12	0.4586735	2.902366e−08	POS	C6orf165	Stromal_score	1.089579e−07	−0.3510303	2.891635e−03	NEG
1927	AK023457	Stromal_score	1.919590e−08	0.3699649	5.094399e−04	POS	BC043601	Stromal_score	1.088813e−08	−0.3758925	2.889601e−04	NEG
1928	ADCK5	Stromal_score	7.610350e−08	−0.3550462	2.019711e−03	NEG	AK091729	Stromal_score	4.014587e−11	−0.4289001	1.065431e−06	NEG
1929	SEMA6D	Stromal_score	1.111848e−07	0.3508022	2.950733e−03	POS	AMPH	Stromal_score	5.015164e−18	0.5428045	1.330974e−13	POS
1930	ODZ3	Stromal_score	5.812272e−11	0.4256717	1.542519e−06	POS	TRAM1L1	Stromal_score	1.832043e−11	0.4356337	4.862060e−07	POS
1931	CACFD1	Stromal_score	2.240822e−09	−0.3918187	5.946917e−05	NEG	GAPDHS	Stromal_score	1.191579e−06	−0.3227163	3.162331e−02	NEG
1932	CHN1	Stromal_score	4.109273e−14	0.4834865	1.090560e−09	POS	CLLU1	Stromal_score	4.790378e−07	0.3338383	1.271319e−02	POS
1933	PRDM8	Stromal_score	1.626561e−11	0.4366420	4.316731e−07	POS	DKFZP434I0714	Stromal_score	3.091049e−07	−0.3390333	8.203335e−03	NEG
1934	FANCA	Stromal_score	1.456068e−08	−0.3728690	3.864259e−04	NEG	HIST4H4	Stromal_score	6.961939e−07	−0.3293286	1.847629e−02	NEG
1935	C18orf1	Stromal_score	2.734843e−18	0.5464087	7.258000e−14	POS	SART3	Stromal_score	1.375998e−07	−0.3483882	3.651761e−03	NEG
1936	CD99	Stromal_score	2.217802e−11	0.4340072	5.885824e−07	POS	LOC284408	Stromal_score	1.071186e−07	0.3512220	2.842822e−03	POS
1937	FANCI	Stromal_score	5.613206e−12	−0.4455147	1.489689e−07	NEG	NLGN1	Stromal_score	1.484054e−13	0.4740135	3.938531e−09	POS
1938	DTNA	Stromal_score	5.320962e−12	0.4459539	1.412130e−07	POS	SNORA26	Stromal_score	1.966242e−08	−0.3697113	5.218210e−04	NEG
1939	CDH2	Stromal_score	3.919190e−08	0.3623292	1.040114e−03	POS	NRXN1	Stromal_score	1.338193e−11	0.4382888	3.551431e−07	POS
1940	AK126266	Stromal_score	1.125372e−17	0.5379325	2.986626e−13	POS	SENP8	Stromal_score	3.741474e−08	−0.3628317	9.929498e−04	NEG
1941	CHTF18	Stromal_score	1.062435e−15	−0.5089538	2.819597e−11	NEG	GDAP2	Stromal_score	1.687357e−08	−0.3713235	4.478077e−04	NEG
1942	NCAPD2	Stromal_score	6.849406e−07	−0.3295267	1.817764e−02	NEG	ZNF829	Stromal_score	1.776991e−11	0.4358926	4.715956e−07	POS
1943	FAM195A	Stromal_score	7.943978e−07	−0.3277194	2.108252e−02	NEG	LILRA1	Stromal_score	1.696561e−08	0.3712663	4.502504e−04	POS
1944	LOC388796	Stromal_score	2.921962e−10	−0.4111674	7.754594e−06	NEG	BCDIN3D	Stromal_score	5.927953e−07	−0.3312769	1.573219e−02	NEG
1945	BC080653	Stromal_score	7.479923e−07	−0.3284546	1.985097e−02	NEG	ZNF208	Stromal_score	7.445765e−13	0.4616862	1.976032e−08	POS
1946	TWSG1	Stromal_score	3.441843e−13	0.4676456	9.134307e−09	POS	ANKRD53	Stromal_score	9.510136e−11	0.4213221	2.523895e−06	POS
1947	UTP18	Stromal_score	7.666104e−07	−0.3281545	2.034507e−02	NEG	BC043188	Stromal_score	2.356671e−13	0.4705290	6.254368e−09	POS
1948	CYTL1	Stromal_score	4.182532e−17	0.5298472	1.110002e−12	POS	ORC4	Stromal_score	5.809181e−13	−0.4636157	1.541699e−08	NEG
1949	PLEKHA2	Stromal_score	4.638448e−09	0.3845948	1.230998e−04	POS	LINC00337	Stromal_score	1.015112e−06	−0.3247048	2.694005e−02	NEG
1950	TLR1	Stromal_score	4.352091e−12	0.4475994	1.155001e−07	POS	C19orf44	Stromal_score	4.374118e−09	−0.3851839	1.160847e−04	NEG
1951	LGMN	Stromal_score	9.497088e−07	0.3255269	2.520432e−02	POS	AK123872	Stromal_score	3.092712e−07	−0.3390270	8.207747e−03	NEG
1952	TTC7B	Stromal_score	4.159393e−14	0.4833985	1.103861e−09	POS	ANKRD23	Stromal_score	1.255913e−07	−0.3494249	3.333068e−03	NEG
1953	STRA13	Stromal_score	1.483403e−11	−0.4374206	3.936804e−07	NEG	SAMD12−AS1	Stromal_score	7.420999e−08	−0.3553260	1.969459e−03	NEG
1954	WWC3	Stromal_score	3.626969e−12	0.4490838	9.625613e−08	POS	ADAMTS13	Stromal_score	4.733004e−08	−0.3602770	1.256092e−03	NEG
1955	ZDHHC9	Stromal_score	5.852201e−09	−0.3822495	1.553116e−04	NEG	FTH1P3	Stromal_score	1.275333e−06	−0.3218693	3.384606e−02	NEG
1956	BEND3	Stromal_score	6.508169e−16	−0.5122129	1.727203e−11	NEG	NSUN4	Stromal_score	2.381855e−10	−0.4130418	6.321206e−06	NEG
1957	DNAJB4	Stromal_score	5.229651e−19	0.5560264	1.387897e−14	POS	LOC100133612	Stromal_score	8.288377e−10	−0.4014203	2.199652e−05	NEG
1958	MKX	Stromal_score	3.660229e−10	0.4090879	9.713882e−06	POS	AK098783	Stromal_score	6.621550e−10	−0.4035461	1.757293e−05	NEG
1959	CHEK1	Stromal_score	9.081128e−13	−0.4601339	2.410041e−08	NEG	TIGD3	Stromal_score	1.645286e−13	−0.4732398	4.366424e−09	NEG
1960	CCT2	Stromal_score	4.105096e−07	−0.3356798	1.089451e−02	NEG	HEATR8	Stromal_score	1.394995e−10	−0.4178942	3.702176e−06	NEG
1961	TRANK1	Stromal_score	1.268150e−07	0.3493150	3.365544e−03	POS	BEND6	Stromal_score	1.215087e−19	0.5642613	3.224719e−15	POS
1962	TEK	Stromal_score	1.001014e−21	0.5898205	2.656590e−17	POS	COLQ	Stromal_score	2.974243e−09	0.3890280	7.893344e−05	POS
1963	ARHGEF38	Stromal_score	4.011819e−09	−0.3860499	1.064697e−04	NEG	KCNA6	Stromal_score	1.327242e−12	0.4571450	3.522367e−08	POS
1964	ACAT2	Stromal_score	5.431930e−11	−0.4262647	1.441580e−06	NEG	PI4KAP2	Stromal_score	1.463374e−08	−0.3728166	3.883649e−04	NEG
1965	ZFPM2	Stromal_score	5.542579e−28	0.6550833	1.470945e−23	POS	NEFM	Stromal_score	4.647293e−11	0.4276273	1.233345e−06	POS
1966	PLCH1	Stromal_score	5.382338e−10	−0.4054947	1.428419e−05	NEG	EID3	Stromal_score	1.300956e−07	0.3490253	3.452607e−03	POS
1967	HMCN1	Stromal_score	2.960980e−21	0.5842371	7.858144e−17	POS	LOC254099	Stromal_score	9.634145e−07	−0.3253502	2.556806e−02	NEG
1968	E2F5	Stromal_score	4.677786e−07	−0.3341228	1.241438e−02	NEG	ZNF630	Stromal_score	1.325566e−06	−0.3213864	3.517920e−02	NEG
1969	CENPN	Stromal_score	3.922799e−09	−0.3862742	1.041072e−04	NEG	ZNF782	Stromal_score	1.085000e−07	−0.3510777	2.879481e−03	NEG
1970	FAM124A	Stromal_score	6.150912e−14	0.4805423	1.632391e−09	POS	ZNF300P1	Stromal_score	7.585597e−16	0.5111979	2.013142e−11	POS
1971	ITPRIP	Stromal_score	2.972769e−18	0.5459155	7.889430e−14	POS	C16orf86	Stromal_score	3.149916e−10	0.4104755	8.359563e−06	POS
1972	PTGFRN	Stromal_score	1.372719e−10	0.4180390	3.643060e−06	POS	ZNF727	Stromal_score	3.175725e−10	0.4104003	8.428055e−06	POS
1973	MBNL1	Stromal_score	1.924145e−16	0.5201685	5.106488e−12	POS	NPAS3	Stromal_score	3.551755e−12	0.4492540	9.426004e−08	POS
1974	CHML	Stromal_score	4.873328e−07	−0.3336328	1.293333e−02	NEG	AK307134	Stromal_score	4.743179e−11	0.4274493	1.258792e−06	POS
1975	AHCY	Stromal_score	3.561464e−09	−0.3872384	9.451769e−05	NEG	KLLN	Stromal_score	2.485190e−07	−0.3415853	6.595447e−03	NEG
1976	GVINP1	Stromal_score	3.088056e−08	0.3649011	8.195393e−04	POS	BC132896	Stromal_score	1.046106e−06	−0.3243328	2.776260e−02	NEG
1977	NCAPD3	Stromal_score	1.389973e−08	−0.3733543	3.688848e−04	NEG	ANKDD1A	Stromal_score	3.057098e−09	0.3887558	8.113234e−05	POS
1978	HIST1H2BN	Stromal_score	2.233837e−10	−0.4136278	5.928379e−06	NEG	AK126351	Stromal_score	8.387623e−11	−0.4224375	2.225991e−06	NEG
1979	ADAP2	Stromal_score	3.272995e−15	0.5013377	8.686201e−11	POS	PRPH2	Stromal_score	1.281319e−13	0.4751120	3.400493e−09	POS
1980	RNF144A	Stromal_score	1.686755e−11	0.4363344	4.476479e−07	POS	MIR4258	Stromal_score	4.032477e−07	−0.3358920	1.070179e−02	NEG
1981	SHCBP1	Stromal_score	1.065171e−08	−0.3761197	2.826857e−04	NEG	SWT1	Stromal_score	2.826186e−07	−0.3400839	7.500415e−03	NEG
1982	LRRC16A	Stromal_score	1.449924e−11	−0.4376132	3.847954e−07	NEG	BC070391	Stromal_score	4.593041e−10	−0.4069776	1.218947e−05	NEG
1983	SNORD28	Stromal_score	1.029063e−08	−0.3764761	2.731030e−04	NEG	FAM43B	Stromal_score	4.772668e−18	0.5431007	1.266618e−13	POS
1984	RCAN1	Stromal_score	4.268556e−08	0.3614022	1.132832e−03	POS	LOC644961	Stromal_score	2.513615e−08	−0.3671040	6.670884e−04	NEG
1985	ANKRD5	Stromal_score	2.634151e−10	−0.4121199	6.990772e−06	NEG	RADIL	Stromal_score	2.367625e−11	0.4334488	6.283441e−07	POS
1986	PTPLAD2	Stromal_score	1.645678e−24	0.6207689	4.367464e−20	POS	PABPC4L	Stromal_score	1.671851e−25	0.6310319	4.436926e−21	POS
1987	ST3GAL2	Stromal_score	4.700066e−09	0.3844621	1.247351e−04	POS	DIS3L2	Stromal_score	3.760247e−08	−0.3627775	9.979318e−04	NEG
1988	RGAG4	Stromal_score	1.712266e−17	0.5353711	4.544184e−13	POS	SNORD78	Stromal_score	5.900100e−12	−0.4451047	1.565828e−07	NEG
1989	LPIN3	Stromal_score	4.112612e−07	−0.3356581	1.091446e−02	NEG	MRC1	Stromal_score	3.368055e−07	0.3380230	8.938481e−03	POS
1990	DCLK2	Stromal_score	3.106249e−15	0.5016959	8.243675e−11	POS	BC046476	Stromal_score	1.094919e−07	−0.3509752	2.905805e−03	NEG
1991	SIPA1	Stromal_score	6.771747e−15	0.4963135	1.797154e−10	POS	SPATA12	Stromal_score	2.114936e−08	−0.3689398	5.612827e−04	NEG
1992	LOC389641	Stromal_score	3.021655e−07	−0.3393000	8.019169e−03	NEG	UBE3D	Stromal_score	2.045309e−15	−0.5045433	5.428045e−11	NEG
1993	TH1L	Stromal_score	2.898553e−10	−0.4112414	7.692470e−06	NEG	AURKAPS1	Stromal_score	4.900117e−15	−0.4985591	1.300442e−10	NEG
1994	YEATS4	Stromal_score	1.511647e−08	−0.3724770	4.011760e−04	NEG	MSTO2P	Stromal_score	6.957779e−09	−0.3804913	1.846525e−04	NEG
1995	NDST1	Stromal_score	9.409585e−15	0.4940128	2.497210e−10	POS	TCTE3	Stromal_score	7.816930e−15	−0.4953118	2.074535e−10	NEG
1996	PDGFRA	Stromal_score	5.642924e−17	0.5279721	1.497576e−12	POS	TCEAL5	Stromal_score	1.540630e−14	0.4905321	4.088677e−10	POS
1997	BNC2	Stromal_score	1.565546e−36	0.7240315	4.154801e−32	POS	ENTPD3−AS1	Stromal_score	1.110830e−07	−0.3508125	2.948033e−03	NEG
1998	MORN5	Stromal_score	2.828803e−07	0.3400731	7.507361e−03	POS	SORCS1	Stromal_score	6.898516e−11	0.4241652	1.830797e−06	POS
1999	PMCH	Stromal_score	1.065530e−11	−0.4402005	2.827810e−07	NEG	C12orf68	Stromal_score	3.299659e−12	0.4498513	8.756966e−08	POS
2000	NAP1L5	Stromal_score	1.238135e−10	0.4189656	3.285886e−06	POS	DNAJB7	Stromal_score	3.150776e−10	−0.4104730	8.361844e−06	NEG
2001	SNORA72	Stromal_score	1.134148e−09	−0.3984257	3.009914e−05	NEG	PARG	Stromal_score	3.505081e−11	−0.4300757	9.302136e−07	NEG
2002	ADPRHL1	Stromal_score	2.166684e−11	−0.4342062	5.750161e−07	NEG	ADCYAP1R1	Stromal_score	3.069622e−10	0.4107135	8.146471e−06	POS
2003	C11orf82	Stromal_score	2.497145e−13	−0.4700900	6.627172e−09	NEG	PPP1R27	Stromal_score	1.541462e−06	−0.3194923	4.090886e−02	NEG
2004	LMO2	Stromal_score	3.955472e−14	0.4837635	1.049743e−09	POS	CRSP8P	Stromal_score	6.040400e−10	−0.4044115	1.603062e−05	NEG
2005	FAM101B	Stromal_score	2.146018e−11	0.4342879	5.695317e−07	POS	STAMBP	Stromal_score	7.487373e−10	−0.4023845	1.987074e−05	NEG
2006	ZNF532	Stromal_score	5.964857e−14	0.4807675	1.583013e−09	POS	LOC375196	Stromal_score	1.000524e−10	0.4208702	2.655290e−06	POS
2007	C1orf135	Stromal_score	4.409280e−15	−0.4992883	1.170179e−10	NEG	ZNF781	Stromal_score	6.975486e−19	0.5543738	1.851224e−14	POS
2008	SNORA10	Stromal_score	1.455722e−06	−0.3202122	3.863341e−02	NEG	LOC100130557	Stromal_score	8.654776e−10	−0.4010090	2.296891e−05	NEG
2009	SEMA6C	Stromal_score	4.914928e−09	0.3840123	1.304373e−04	POS	ZNF660	Stromal_score	6.357550e−12	0.4444895	1.687230e−07	POS
2010	CHAC2	Stromal_score	1.318319e−08	−0.3739063	3.498687e−04	NEG	ZNF799	Stromal_score	4.047922e−11	−0.4288283	1.074278e−06	NEG
2011	CD81	Stromal_score	1.755671e−09	0.3942028	4.659375e−05	POS	CD300LB	Stromal_score	7.856319e−08	0.3546924	2.084989e−03	POS
2012	ARHGEF35	Stromal_score	1.490907e−06	−0.3199120	3.956718e−02	NEG	KCND2	Stromal_score	6.820081e−08	0.3562620	1.809981e−03	POS
2013	SLC25A5−AS1	Stromal_score	8.703481e−08	−0.3535508	2.309817e−03	NEG	ZNF568	Stromal_score	6.411649e−13	0.4628500	1.701588e−08	POS
2014	ST5	Stromal_score	1.106591e−06	0.3236361	2.936782e−02	POS	METTL6	Stromal_score	4.486890e−07	−0.3346205	1.190776e−02	NEG
2015	KAT2B	Stromal_score	1.824538e−09	0.3938281	4.842141e−05	POS	CNGA3	Stromal_score	3.416256e−07	0.3378554	9.066402e−03	POS
2016	SYNE1	Stromal_score	1.141979e−30	0.6788784	3.030697e−26	POS	TIPARP−AS1	Stromal_score	1.167723e−06	−0.3229680	3.099020e−02	NEG
2017	MOB3A	Stromal_score	1.390693e−20	0.5760830	3.690761e−16	POS	ABCD2	Stromal_score	1.271916e−11	0.4387160	3.375539e−07	POS
2018	LHFPL2	Stromal_score	1.025131e−19	0.5652058	2.720596e−15	POS	DZANK1	Stromal_score	2.805230e−10	−0.4115422	7.444800e−06	NEG
2019	LBX2	Stromal_score	1.349380e−07	−0.3486103	3.581120e−03	NEG	FLJ27354	Stromal_score	4.886366e−13	0.4649534	1.296793e−08	POS
2020	OSBPL3	Stromal_score	4.866463e−09	−0.3841121	1.291511e−04	NEG	NSL1	Stromal_score	2.295119e−07	−0.3425103	6.091017e−03	NEG
2021	FAM195B	Stromal_score	5.620643e−08	0.3583948	1.491663e−03	POS	BC071789	Stromal_score	5.284315e−11	0.4265058	1.402404e−06	POS
2022	PLXNA4	Stromal_score	6.950005e−11	0.4240996	1.844462e−06	POS	LOC285548	Stromal_score	2.269925e−08	0.3681894	6.024155e−04	POS
2023	NDFIP2	Stromal_score	1.169016e−06	−0.3229542	3.102452e−02	NEG	NTRK3	Stromal_score	7.818127e−14	0.4787781	2.074853e−09	POS
2024	CDC7	Stromal_score	1.636747e−08	−0.3716434	4.343763e−04	NEG	DUSP28	Stromal_score	1.441177e−13	−0.4742330	3.824740e−09	NEG
2025	ARHGAP24	Stromal_score	2.356093e−10	0.4131412	6.252836e−06	POS	SNORD116−20	Stromal_score	1.218029e−08	0.3747296	3.232526e−04	POS
2026	RAB11FIP4	Stromal_score	4.585013e−08	−0.3606235	1.216817e−03	NEG	PRDM15	Stromal_score	2.123950e−07	−0.3434086	5.636751e−03	NEG
2027	Z49982	Stromal_score	4.385563e−07	0.3348930	1.163885e−02	POS	MYOZ3	Stromal_score	2.148249e−10	0.4139841	5.701239e−06	POS
2028	TOX2	Stromal_score	4.895501e−09	0.3840522	1.299217e−04	POS	TMED10P1	Stromal_score	5.293594e−07	−0.3326404	1.404867e−02	NEG
2029	TMEM63B	Stromal_score	3.924048e−07	−0.3362156	1.041403e−02	NEG	NALCN	Stromal_score	2.801425e−12	0.4511751	7.434702e−08	POS
2030	TESPA1	Stromal_score	5.995069e−08	0.3576854	1.591031e−03	POS	NKAPL	Stromal_score	1.331629e−14	0.4915654	3.534009e−10	POS
2031	ECE2	Stromal_score	6.100031e−13	−0.4632369	1.618887e−08	NEG	TSGA10	Stromal_score	3.175817e−14	−0.4853528	8.428302e−10	NEG
2032	BMP1	Stromal_score	6.060799e−07	0.3310091	1.608475e−02	POS	HEATR7A	Stromal_score	2.708725e−09	−0.3899525	7.188684e−05	NEG
2033	CYTH3	Stromal_score	4.606389e−10	0.4069505	1.222489e−05	POS	LRRC39	Stromal_score	1.600892e−07	−0.3466615	4.248608e−03	NEG
2034	TBC1D30	Stromal_score	3.374824e−11	−0.4304029	8.956445e−07	NEG	TLL1	Stromal_score	1.519950e−17	0.5361004	4.033795e−13	POS
2035	MITF	Stromal_score	8.402111e−13	0.4607424	2.229836e−08	POS	OCLM	Stromal_score	2.229169e−07	−0.3428486	5.915992e−03	NEG
2036	TMEM130	Stromal_score	3.361611e−09	0.3878132	8.921379e−05	POS	NRL	Stromal_score	6.745326e−07	−0.3297127	1.790142e−02	NEG
2037	TUFT1	Stromal_score	8.978423e−09	−0.3778818	2.382784e−04	NEG	TMCC2	Stromal_score	1.346747e−10	0.4182107	3.574132e−06	POS
2038	BC007896	Stromal_score	9.393596e−09	0.3774167	2.492966e−04	POS	ZNF667	Stromal_score	2.771079e−22	0.5962962	7.354165e−18	POS
2039	CLSPN	Stromal_score	1.207872e−13	−0.4755523	3.205572e−09	NEG	AX747062	Stromal_score	4.154896e−09	0.3856992	1.102668e−04	POS
2040	MCM3	Stromal_score	1.343641e−09	−0.3967947	3.565890e−05	NEG	AKD1	Stromal_score	4.298433e−07	−0.3351321	1.140761e−02	NEG

	gene	feature	p.value	estimate	adj.p.value	COR.ID	gene	feature	p.value	estimate	adj.p.value	COR.ID
2041	SOX17	Stromal_score	3.918389e−16	0.5155506	1.039901e−11	POS	DPY19L2P3	Stromal_score	1.129106e−07	−0.3506284	2.996535e−03	NEG
2042	PRIM1	Stromal_score	2.624947e−08	−0.3666415	6.966347e−04	NEG	DHH	Stromal_score	4.679237e−13	0.4652874	1.241823e−08	POS
2043	C2orf15	Stromal_score	7.579080e−19	−0.5538960	2.011412e−14	NEG	C12orf76	Stromal_score	2.036911e−09	−0.3927532	5.405759e−05	NEG
2044	PRKG1	Stromal_score	2.832401e−19	0.5595142	7.516909e−15	POS	LOC100507266	Stromal_score	2.959386e−09	−0.3890776	7.853915e−05	NEG
2045	ZNF518B	Stromal_score	1.178747e−06	0.3228511	3.128276e−02	POS	WDR27	Stromal_score	3.740866e−09	−0.3867485	9.927884e−05	NEG
2046	EXPH5	Stromal_score	1.317233e−06	−0.3214653	3.495804e−02	NEG	RBM45	Stromal_score	5.305976e−07	−0.3326123	1.408153e−02	NEG
2047	ADAMTSL3	Stromal_score	1.336623e−19	0.5637304	3.547264e−15	POS	SNAP91	Stromal_score	9.378357e−08	0.3527153	2.488922e−03	POS
2048	KRBOX1	Stromal_score	9.941401e−09	0.3768325	2.638348e−04	POS	AK308605	Stromal_score	1.270419e−10	0.4187347	3.371565e−06	POS
2049	YDJC	Stromal_score	1.837491e−11	−0.4356085	4.876518e−07	NEG	SUGP1	Stromal_score	3.596701e−07	−0.3372475	9.545284e−03	NEG
2050	AMICA1	Stromal_score	3.657393e−10	0.4090950	9.706355e−06	POS	CBWD6	Stromal_score	8.013701e−11	−0.4228415	2.126756e−06	NEG
2051	CHRD	Stromal_score	2.515774e−19	0.5601837	6.676611e−15	POS	FBXO48	Stromal_score	1.530434e−07	−0.3471760	4.061620e−03	NEG
2052	SGCB	Stromal_score	4.112579e−10	0.4080065	1.091437e−05	POS	CASC2	Stromal_score	2.156621e−07	−0.3432319	5.723458e−03	NEG
2053	PLEKHG2	Stromal_score	3.530978e−12	0.4493016	9.370862e−08	POS	ZNF582	Stromal_score	2.644609e−21	0.5848239	7.018528e−17	POS
2054	PLD3	Stromal_score	1.040030e−10	0.4205249	2.760136e−06	POS	ZNF677	Stromal_score	1.481317e−22	0.5994019	3.931268e−18	POS
2055	TOM1L1	Stromal_score	3.582796e−09	−0.3871789	9.508382e−05	NEG	SHC4	Stromal_score	2.035828e−10	0.4144736	5.402884e−06	POS
2056	43161	Stromal_score	4.437505e−16	0.5147356	1.177669e−11	POS	LOC340037	Stromal_score	8.742443e−07	−0.3265456	2.320157e−02	NEG
2057	PIK3R1	Stromal_score	5.614765e−09	0.3826687	1.490103e−04	POS	ZGLP1	Stromal_score	6.401328e−07	−0.3303476	1.698848e−02	NEG
2058	SAMD12	Stromal_score	7.452480e−07	−0.3284994	1.977814e−02	NEG	LOC100130894	Stromal_score	3.059936e−07	−0.3391521	8.120765e−03	NEG
2059	TRAF4	Stromal_score	2.088324e−15	−0.5044021	5.542202e−11	NEG	SNORA27	Stromal_score	5.361763e−08	−0.3589123	1.422958e−03	NEG
2060	TSHZ1	Stromal_score	2.435381e−07	0.3418209	6.463258e−03	POS	DQ574505	Stromal_score	4.080297e−08	−0.3618922	1.082870e−03	NEG
2061	TCF4	Stromal_score	1.785250e−34	0.7091434	4.737874e−30	POS	SCN2B	Stromal_score	8.697220e−18	0.5394944	2.308155e−13	POS
2062	SIGLEC7	Stromal_score	4.044911e−07	0.3358554	1.073479e−02	POS	LOC400752	Stromal_score	3.408132e−10	−0.4097482	9.044841e−06	NEG
2063	LOC100506451	Stromal_score	1.601211e−14	0.4902581	4.249453e−10	POS	SNORD75	Stromal_score	5.470687e−09	−0.3829316	1.451866e−04	NEG
2064	CYB561	Stromal_score	1.697178e−07	−0.3459925	4.504142e−03	NEG	ZNF833P	Stromal_score	1.779064e−11	−0.4358828	4.721459e−07	NEG
2065	STARD13	Stromal_score	1.042912e−10	0.4205003	2.767784e−06	POS	USHBP1	Stromal_score	5.204438e−13	0.4644664	1.381206e−08	POS
2066	SLC25A5	Stromal_score	3.733358e−07	−0.3368062	9.907960e−03	NEG	LOC100286844	Stromal_score	7.463880e−07	−0.3284807	1.980839e−02	NEG
2067	KIAA1524	Stromal_score	1.965434e−09	−0.3931023	5.216066e−05	NEG	DLG2	Stromal_score	5.902223e−23	0.6039044	1.566391e−18	POS
2068	ACSS3	Stromal_score	1.220871e−14	0.4921793	3.240068e−10	POS	RBM14	Stromal_score	1.279504e−08	−0.3742175	3.395675e−04	NEG
2069	FAM176B	Stromal_score	3.546016e−15	0.5007880	9.410773e−11	POS	C9orf163	Stromal_score	1.188136e−09	−0.3979791	3.153193e−05	NEG
2070	RUSC2	Stromal_score	6.062090e−14	0.4806489	1.608818e−09	POS	C19orf35	Stromal_score	3.626537e−07	0.3371498	9.624467e−03	POS
2071	C3orf80	Stromal_score	1.882996e−07	0.3447986	4.997283e−03	POS	THAP9	Stromal_score	7.268446e−13	−0.4618741	1.928973e−08	NEG
2072	LRRC33	Stromal_score	1.035833e−12	0.4591007	2.748997e−08	POS	YPEL4	Stromal_score	5.054761e−16	0.5138800	1.341483e−11	POS
2073	VAV2	Stromal_score	1.332607e−07	−0.3487524	3.536605e−03	NEG	RPSAP9	Stromal_score	4.130864e−10	−0.4079653	1.096290e−05	NEG
2074	MRAS	Stromal_score	5.669416e−28	0.6549921	1.504606e−23	POS	ZNF676	Stromal_score	1.924898e−08	0.3699358	5.108486e−04	POS
2075	RTN4	Stromal_score	2.340226e−13	0.4705821	6.210725e−09	POS	ZNF2	Stromal_score	8.983794e−08	−0.3531964	2.384209e−03	NEG
2076	HIST2H2AC	Stromal_score	6.789748e−08	−0.3563114	1.801931e−03	NEG	KCNQ5	Stromal_score	2.632963e−12	0.4516751	6.987620e−08	POS
2077	POLE2	Stromal_score	3.490053e−11	−0.4301128	9.262251e−07	NEG	LOC339524	Stromal_score	1.668270e−13	0.4731356	4.427423e−09	POS
2078	BC071809	Stromal_score	2.314945e−12	0.4527105	6.143632e−08	POS	BC015159	Stromal_score	4.664865e−17	0.5291652	1.238008e−12	POS
2079	PUS7	Stromal_score	3.648325e−10	−0.4091180	9.682288e−06	NEG	CMTM5	Stromal_score	1.532447e−08	0.3723339	4.066962e−04	POS
2080	CCDC71L	Stromal_score	8.755894e−11	0.4220564	2.323727e−06	POS	AK126852	Stromal_score	8.919037e−07	0.3262999	2.367023e−02	POS
2081	LOC646576	Stromal_score	1.367654e−10	0.4180722	3.629616e−06	POS	SNORA58	Stromal_score	1.756305e−12	−0.4549200	4.661059e−08	NEG
2082	TMEM106C	Stromal_score	1.001612e−06	−0.3248703	2.658178e−02	NEG	LOC100272217	Stromal_score	6.565141e−09	−0.3810827	1.742323e−04	NEG
2083	TUBB4B	Stromal_score	5.392716e−11	−0.4263281	1.431173e−06	NEG	WDR20	Stromal_score	1.031992e−07	−0.3516415	2.738804e−03	NEG
2084	MAFG−AS1	Stromal_score	6.125744e−09	−0.3817863	1.625711e−04	NEG	KLHL38	Stromal_score	4.209398e−10	0.4077901	1.117132e−05	POS
2085	ACVRL1	Stromal_score	2.030448e−17	0.5343247	5.388607e−13	POS	WASH7P	Stromal_score	2.335138e−11	−0.4335669	6.197221e−07	NEG
2086	TRIL	Stromal_score	2.483098e−09	0.3908098	6.589893e−05	POS	BC043649	Stromal_score	1.736939e−06	−0.3179843	4.609663e−02	NEG
2087	DOCK10	Stromal_score	4.685184e−11	0.4275566	1.243401e−06	POS	PTGDR	Stromal_score	9.823398e−07	0.3251102	2.607031e−02	POS
2088	MREG	Stromal_score	4.415717e−08	−0.3610334	1.171887e−03	NEG	PCDH19	Stromal_score	3.989705e−07	0.3360186	1.058828e−02	POS
2089	FGD2	Stromal_score	1.419788e−07	0.3480317	3.767976e−03	POS	CIAO1	Stromal_score	1.568860e−06	−0.3192702	4.163599e−02	NEG
2090	EHBP1L1	Stromal_score	1.150515e−08	0.3753215	3.053353e−04	POS	RASGEF1C	Stromal_score	6.249137e−08	0.3572280	1.658458e−03	POS
2091	MIR22HG	Stromal_score	2.381774e−07	0.3420798	6.320990e−03	POS	C1orf213	Stromal_score	4.234455e−08	0.3614894	1.123782e−03	POS
2092	BCL2	Stromal_score	1.194121e−12	0.4579804	3.169077e−08	POS	CSMD2	Stromal_score	1.867914e−06	0.3170621	4.957256e−02	POS
2093	SNRPB	Stromal_score	8.921061e−09	−0.3779476	2.367560e−04	NEG	MAP1LC3C	Stromal_score	6.431624e−08	0.3569103	1.706889e−03	POS
2094	CYP7B1	Stromal_score	3.077908e−19	0.5590438	8.168461e−15	POS	TYW5	Stromal_score	7.108214e−16	−0.5116290	1.886449e−11	NEG
2095	PKIA	Stromal_score	3.002097e−13	0.4686893	7.967264e−09	POS	FAM216B	Stromal_score	1.282018e−07	0.3491916	3.402348e−03	POS
2096	SEC23B	Stromal_score	2.185018e−07	−0.3430804	5.798818e−03	NEG	C9orf43	Stromal_score	1.614150e−12	−0.4555922	4.283792e−08	NEG
2097	BC034929	Stromal_score	1.356501e−10	−0.4181459	3.600018e−06	NEG	ATP8B4	Stromal_score	1.116991e−13	0.4761349	2.964383e−09	POS
2098	PLEKHH1	Stromal_score	1.488254e−08	−0.3726403	3.949679e−04	NEG	ZNF169	Stromal_score	2.080485e−07	−0.3436478	5.521398e−03	NEG
2099	HSPA12B	Stromal_score	5.189841e−24	0.6154675	1.377332e−19	POS	LINC00115	Stromal_score	2.333088e−10	−0.4132309	6.191782e−06	NEG
2100	SPRY1	Stromal_score	3.821742e−09	0.3865350	1.014252e−04	POS	TMEM108	Stromal_score	1.913803e−13	0.4721022	5.079042e−09	POS
2101	ZHX2	Stromal_score	5.375934e−07	0.3324549	1.426719e−02	POS	LOC127841	Stromal_score	7.324535e−11	−0.4236365	1.943858e−06	NEG
2102	CALHM2	Stromal_score	1.460132e−22	0.5994729	3.875043e−18	POS	MPPED2	Stromal_score	1.796807e−09	0.3939773	4.768547e−05	POS
2103	MIR4785	Stromal_score	1.441676e−07	0.3478575	3.826063e−03	POS	FLT3	Stromal_score	1.772180e−07	0.3454962	4.703188e−03	POS
2104	LOC100129046	Stromal_score	2.586857e−07	−0.3411180	6.865260e−03	NEG	ZNF454	Stromal_score	4.208553e−23	0.6055416	1.116908e−18	POS
2105	RAB27A	Stromal_score	4.795552e−07	0.3338254	1.272692e−02	POS	LRRC4C	Stromal_score	4.617343e−16	0.5144748	1.225397e−11	POS
2106	ITGB5	Stromal_score	2.614468e−08	0.3666843	6.938536e−04	POS	CBWD3	Stromal_score	1.789079e−10	−0.4156471	4.748036e−06	NEG
2107	CREB3L2	Stromal_score	2.560797e−18	0.5467970	6.796100e−14	POS	SV2B	Stromal_score	4.443950e−16	0.5147260	1.179380e−11	POS
2108	RPS26	Stromal_score	1.455954e−07	−0.3477452	3.863957e−03	NEG	C9orf174	Stromal_score	1.827840e−06	0.3173375	4.850903e−02	POS
2109	IDI1	Stromal_score	8.523878e−07	−0.3268564	2.262152e−02	NEG	CACNA2D3	Stromal_score	2.055406e−12	0.4536638	5.454841e−08	POS
2110	MAP7D1	Stromal_score	3.467808e−22	0.5951759	9.203216e−18	POS	BC037839	Stromal_score	8.788987e−09	0.3781009	2.332509e−04	POS
2111	LNX1	Stromal_score	9.150381e−07	−0.3259849	2.428420e−02	NEG	LINC00471	Stromal_score	2.741423e−07	−0.3404402	7.275463e−03	NEG
2112	BACE1	Stromal_score	4.864936e−17	0.5289024	1.291105e−12	POS	LOC727896	Stromal_score	9.003341e−11	−0.4218089	2.389397e−06	NEG
2113	ATAD3B	Stromal_score	3.813736e−11	−0.4293452	1.012127e−06	NEG	BC017255	Stromal_score	1.470866e−06	−0.3200821	3.903532e−02	NEG
2114	WDR34	Stromal_score	2.749184e−11	−0.4321685	7.296060e−07	NEG	DKFZp779M0652	Stromal_score	1.427767e−06	0.3204557	3.789150e−02	POS
2115	ZBTB12	Stromal_score	8.496176e−07	−0.3268963	2.254800e−02	NEG	LOC100129722	Stromal_score	1.546174e−06	−0.3194538	4.103391e−02	NEG
2116	MCM8	Stromal_score	6.982680e−09	−0.3804549	1.853133e−04	NEG	AX747376	Stromal_score	4.453396e−08	0.3609409	1.181887e−03	POS
2117	DDX39A	Stromal_score	8.837730e−07	−0.3264124	2.345445e−02	NEG	LOC100287765	Stromal_score	4.342584e−07	−0.3350104	1.152478e−02	NEG
2118	SMC4	Stromal_score	1.717544e−08	−0.3711370	4.558190e−04	NEG	LHX4	Stromal_score	1.174452e−13	−0.4757614	3.116879e−09	NEG
2119	TGFBR1	Stromal_score	6.851913e−09	0.3806475	1.818429e−04	POS	ZNF382	Stromal_score	2.301721e−13	0.4707077	6.108537e−09	POS
2120	SV2A	Stromal_score	2.863672e−10	0.4113527	7.599899e−06	POS	LOC100506388	Stromal_score	2.133408e−12	0.4533655	5.661852e−08	POS
2121	NIN	Stromal_score	6.933229e−10	0.4031119	1.840010e−05	POS	PMP2	Stromal_score	1.457310e−08	0.3728601	3.867556e−04	POS
2122	SRF	Stromal_score	2.428870e−09	0.3910271	6.445979e−05	POS	LOC386758	Stromal_score	1.530491e−13	0.4737826	4.061771e−09	POS
2123	PEAR1	Stromal_score	1.634016e−13	0.4732914	4.336515e−09	POS	SHANK1	Stromal_score	6.337298e−14	0.4803232	1.681856e−09	POS
2124	CHST1	Stromal_score	1.697515e−09	0.3945305	4.505034e−05	POS	AK056431	Stromal_score	1.362537e−08	0.3735624	3.616036e−04	POS
2125	FAM198A	Stromal_score	1.338145e−13	0.4747879	3.551304e−09	POS	TAL1	Stromal_score	3.958103e−10	0.4083622	1.050441e−05	POS
2126	CA11	Stromal_score	4.789824e−08	0.3601467	1.271171e−03	POS	CADM2	Stromal_score	1.181249e−08	0.3750480	3.134917e−04	POS
2127	TMEM48	Stromal_score	5.113581e−11	−0.4267929	1.357093e−06	NEG	DKFZP564C196	Stromal_score	5.022000e−10	−0.4061436	1.332788e−05	NEG
2128	CSDC2	Stromal_score	5.533025e−17	0.5280956	1.468410e−12	POS	TTC18	Stromal_score	2.826935e−08	−0.3658487	7.502403e−04	NEG
2129	ARHGAP32	Stromal_score	1.030571e−08	−0.3764610	2.735031e−04	NEG	AK056073	Stromal_score	4.361067e−17	−0.5295862	1.157383e−12	NEG
2130	PCDH17	Stromal_score	3.598932e−17	0.5307838	9.551205e−13	POS	SNORD114−10	Stromal_score	1.118548e−06	0.3235027	2.968514e−02	POS
2131	TP53INP1	Stromal_score	7.818946e−08	0.3547455	2.075070e−03	POS	DCST1	Stromal_score	3.097967e−12	−0.4503620	8.221694e−08	NEG
2132	MIR4641	Stromal_score	4.863621e−10	−0.4064432	1.290756e−05	NEG	CAMK2A	Stromal_score	1.498239e−07	0.3474187	3.976175e−03	POS
2133	CHEK2	Stromal_score	1.176314e−08	−0.3750915	3.121819e−04	NEG	BAI3	Stromal_score	2.173766e−18	0.5477622	5.768958e−14	POS
2134	UPF3B	Stromal_score	1.221588e−06	−0.3224065	3.241974e−02	NEG	KCNT2	Stromal_score	8.691590e−20	0.5661199	2.306661e−15	POS
2135	TMPO	Stromal_score	2.501615e−07	−0.3415085	6.639035e−03	NEG	CECR5−AS1	Stromal_score	1.050022e−06	−0.3242865	2.786654e−02	NEG
2136	SULT1C4	Stromal_score	2.173083e−11	0.4341810	5.767146e−07	POS	DEPDC4	Stromal_score	5.918441e−13	−0.4634713	1.570695e−08	NEG
2137	KIF14	Stromal_score	7.636317e−15	−0.4954752	2.026602e−10	NEG	PRTG	Stromal_score	3.372868e−14	0.4849178	8.951254e−10	POS
2138	CXorf36	Stromal_score	2.691962e−21	0.5847318	7.144197e−17	POS	VWC2	Stromal_score	1.453399e−06	0.3202322	3.857177e−02	POS
2139	CC2D2A	Stromal_score	2.399973e−10	0.4129725	6.369289e−06	POS	FMN2	Stromal_score	1.520466e−08	0.3724161	4.035166e−04	POS
2140	DSN1	Stromal_score	2.879348e−12	−0.4509536	7.641501e−08	NEG	C14orf64	Stromal_score	1.011934e−10	0.4207691	2.685572e−06	POS
2141	HIST1H2AG	Stromal_score	7.398107e−09	−0.3798654	1.963384e−04	NEG	KLF17	Stromal_score	9.161879e−17	0.5249128	2.431471e−12	POS
2142	MAP6	Stromal_score	2.069154e−12	0.4536104	5.491328e−08	POS	BC047513	Stromal_score	4.823563e−13	−0.4650532	1.280125e−08	NEG
2143	ABL1	Stromal_score	2.864696e−10	0.4113494	7.602616e−06	POS	LOC100129662	Stromal_score	3.159359e−08	0.3646557	8.384622e−04	POS
2144	ZNF165	Stromal_score	5.243282e−13	−0.4644089	1.391515e−08	NEG	CMYA5	Stromal_score	3.805932e−16	0.5157411	1.010056e−11	POS
2145	BC070118	Stromal_score	3.889179e−14	0.4838862	1.032149e−09	POS	WHAMMP3	Stromal_score	2.852645e−10	0.4113882	7.570634e−06	POS
2146	INPP5J	Stromal_score	1.187949e−09	−0.3979806	3.152699e−05	NEG	ESR1	Stromal_score	1.326344e−19	0.5637734	3.519985e−15	POS
2147	SNORD16	Stromal_score	1.836580e−07	−0.3450858	4.874100e−03	NEG	CCDC150	Stromal_score	2.280625e−14	−0.4877344	6.052551e−10	NEG
2148	ADARB1	Stromal_score	7.708781e−13	0.4614153	2.045833e−08	POS	SNORD46	Stromal_score	2.982297e−08	−0.3652752	7.914719e−04	NEG
2149	NACAD	Stromal_score	1.821299e−19	0.5620006	4.833545e−15	POS	ABHD1	Stromal_score	1.509594e−07	−0.3473325	4.006312e−03	NEG
2150	PRCP	Stromal_score	2.811485e−17	0.5323164	7.461399e−13	POS	LOC100132077	Stromal_score	1.250675e−13	0.4752926	3.319167e−09	POS
2151	MMP17	Stromal_score	5.029727e−07	0.3332543	1.334839e−02	POS	SGCG	Stromal_score	1.050961e−06	0.3242754	2.789145e−02	POS
2152	NLGN4X	Stromal_score	1.762386e−12	0.4548925	4.677195e−08	POS	C1QTNF9	Stromal_score	1.041095e−06	0.3243922	2.762961e−02	POS
2153	AK094577	Stromal_score	4.527676e−13	−0.4655411	1.201600e−08	NEG	PCA3	Stromal_score	1.225952e−08	0.3746622	3.253555e−04	POS
2154	PACS1	Stromal_score	1.446769e−14	0.4909780	3.839580e−10	POS	WASH1	Stromal_score	1.996279e−07	−0.3441250	5.297924e−03	NEG
2155	RNF122	Stromal_score	1.658011e−14	0.4900103	4.400196e−10	POS	BC041434	Stromal_score	8.969751e−14	−0.4777627	2.380482e−09	NEG
2156	NOP16	Stromal_score	8.424070e−07	−0.3270008	2.235664e−02	NEG	CLUL1	Stromal_score	1.356650e−08	−0.3736075	3.600413e−04	NEG
2157	SLC10A5	Stromal_score	6.673347e−07	−0.3298429	1.771040e−02	NEG	DKFZP434K028	Stromal_score	1.570706e−06	−0.3192554	4.168497e−02	NEG
2158	BZW2	Stromal_score	1.245883e−11	−0.4388898	3.306449e−07	NEG	FAM203A	Stromal_score	7.947992e−15	−0.4951955	2.109317e−10	NEG
2159	TRAM2	Stromal_score	4.017175e−10	0.4082247	1.066118e−05	POS	MIR4723	Stromal_score	1.004346e−06	−0.3248366	2.665434e−02	NEG
2160	IL3RA	Stromal_score	2.489773e−21	0.5851367	6.607609e−17	POS	SLITRK2	Stromal_score	5.605076e−09	0.3826862	1.487531e−04	POS

	gene	feature	p.value	estimate	adj.p.value	COR.ID	gene	feature	p.value	estimate	adj.p.value	COR.ID
2161	ST6GALNAC5	Stromal_score	2.509559e−15	0.5031527	6.660119e−11	POS	HEPACAM	Stromal_score	1.580681e−07	0.3468068	4.194968e−03	POS
2162	EIF6	Stromal_score	1.207783e−06	−0.3225481	3.205335e−02	NEG	PABPC5	Stromal_score	6.933601e−29	0.6633270	1.840108e−24	POS
2163	MAPRE2	Stromal_score	7.348406e−07	0.3286709	1.950193e−02	POS	PPIP5K1	Stromal_score	2.107055e−11	−0.4344441	5.591913e−07	NEG
2164	KNTC1	Stromal_score	2.870141e−09	−0.3893806	7.617067e−05	NEG	BVES−AS1	Stromal_score	1.300635e−06	0.3216238	3.451755e−02	POS
2165	PNRC1	Stromal_score	1.307617e−13	0.4749603	3.470286e−09	POS	FGF8	Stromal_score	1.049636e−06	−0.3242910	2.785629e−02	NEG
2166	PRKCH	Stromal_score	2.404828e−17	0.5332821	6.382172e−13	POS	CPEB1	Stromal_score	1.176259e−15	0.5082727	3.121674e−11	POS
2167	RBM24	Stromal_score	2.067609e−10	0.4143327	5.487227e−06	POS	KLRF1	Stromal_score	3.049115e−07	0.3391937	8.092047e−03	POS
2168	BC022047	Stromal_score	1.239299e−06	0.3222270	3.288977e−02	POS	GPR88	Stromal_score	2.274395e−13	0.4707981	6.036017e−09	POS
2169	CSDA	Stromal_score	1.681844e−10	0.4162069	4.463447e−06	POS	ZCCHC5	Stromal_score	9.597142e−10	0.4000241	2.546986e−05	POS
2170	AL390170	Stromal_score	6.425191e−12	0.4444022	1.705181e−07	POS	LOC100507244	Stromal_score	1.790722e−07	0.3453766	4.752397e−03	POS
2171	HIST1H1B	Stromal_score	2.838842e−08	−0.3658037	7.534003e−04	NEG	AX746690	Stromal_score	1.551022e−08	−0.3722077	4.116258e−04	NEG
2172	PARPBP	Stromal_score	3.353087e−13	−0.4678453	8.898759e−09	NEG	FAM156B	Stromal_score	3.699030e−08	−0.3629551	9.816855e−04	NEG
2173	LOC389906	Stromal_score	1.019247e−07	−0.3517812	2.704979e−03	NEG	ST8SIA2	Stromal_score	6.909170e−10	0.4031447	1.833625e−05	POS
2174	SLC25A10	Stromal_score	1.987814e−13	−0.4718161	5.275459e−09	NEG	KIAA1045	Stromal_score	7.165175e−10	0.4028008	1.901566e−05	POS
2175	MSH5	Stromal_score	1.838975e−12	−0.4545531	4.880456e−08	NEG	DOK6	Stromal_score	2.103802e−19	0.5611909	5.583279e−15	POS
2176	LOC339803	Stromal_score	1.490128e−06	0.3199185	3.954650e−02	POS	LOC283143	Stromal_score	1.029417e−16	0.5241727	2.731969e−12	POS
2177	HIP1	Stromal_score	1.198704e−07	0.3499527	3.181239e−03	POS	AX746830	Stromal_score	5.893968e−11	−0.4255492	1.564200e−06	NEG
2178	CRTAC1	Stromal_score	2.023906e−08	0.3694056	5.371244e−04	POS	LOC100134868	Stromal_score	3.606754e−09	−0.3871125	9.571965e−05	NEG
2179	DQ593252	Stromal_score	4.468975e−10	0.4072329	1.186021e−05	POS	TPI1P2	Stromal_score	2.515638e−08	−0.3670954	6.676252e−04	NEG
2180	CMAHP	Stromal_score	3.323701e−20	0.5713881	8.820770e−16	POS	BC042825	Stromal_score	1.157200e−06	−0.3230806	3.071093e−02	NEG
2181	GPR4	Stromal_score	2.154654e−07	0.3432425	5.718235e−03	POS	PIPSL	Stromal_score	1.488094e−07	−0.3474962	3.949254e−03	NEG
2182	KLHDC8B	Stromal_score	1.422077e−12	0.4565983	3.774051e−08	POS	DCST2	Stromal_score	8.391004e−07	−0.3270490	2.226889e−02	NEG
2183	FAM65A	Stromal_score	2.376878e−12	0.4524984	6.307997e−08	POS	LINC00340	Stromal_score	5.171077e−07	0.3329217	1.372352e−02	POS
2184	43160	Stromal_score	6.590937e−10	0.4035898	1.749169e−05	POS	LPAR4	Stromal_score	2.004047e−21	0.5862591	5.318541e−17	POS
2185	FAM110B	Stromal_score	1.976039e−12	0.4539788	5.244211e−08	POS	AY343891	Stromal_score	6.636430e−08	0.3565640	1.761242e−03	POS
2186	LOC387647	Stromal_score	1.572898e−06	0.3192378	4.174315e−02	POS	PCDHB18	Stromal_score	3.208868e−13	0.4681812	8.516015e−09	POS
2187	THOC3	Stromal_score	1.812187e−08	−0.3705723	4.809364e−04	NEG	ZSCAN23	Stromal_score	1.724905e−12	0.4550638	4.577727e−08	POS
2188	PPP1R35	Stromal_score	1.627325e−06	−0.3188086	4.318757e−02	NEG	AX748440	Stromal_score	2.161903e−10	0.4139264	5.737473e−06	POS
2189	SCN4B	Stromal_score	9.787884e−16	0.5095016	2.597607e−11	POS	LRRC55	Stromal_score	5.742153e−12	0.4453280	1.523910e−07	POS
2190	EPT1	Stromal_score	9.041511e−11	−0.4217713	2.399526e−06	NEG	AF161442	Stromal_score	2.973496e−08	0.3653069	7.891362e−04	POS
2191	FANCD2	Stromal_score	1.680687e−15	−0.5058721	4.460374e−11	NEG	FGF5	Stromal_score	1.223465e−07	0.3497213	3.246955e−03	POS
2192	PPP1R12A	Stromal_score	7.754776e−11	0.4231321	2.058040e−06	POS	bK250D10.C22.8	Stromal_score	1.775292e−06	−0.3177076	4.711448e−02	NEG
2193	TOP1MT	Stromal_score	2.654789e−09	−0.3901510	7.045545e−05	NEG	TSLP	Stromal_score	3.268363e−07	0.3383771	8.673908e−03	POS
2194	TFDP1	Stromal_score	2.409700e−07	−0.3419443	6.395103e−03	NEG	SRSF10	Stromal_score	5.648727e−13	−0.4638327	1.499116e−08	NEG
2195	ADCY9	Stromal_score	6.141974e−11	0.4251873	1.630019e−06	POS	DPYS	Stromal_score	1.911051e−09	0.3933763	5.071737e−05	POS
2196	FRAT2	Stromal_score	2.337877e−09	−0.3914025	6.204491e−05	NEG	PRRG3	Stromal_score	1.356391e−13	0.4746866	3.599726e−09	POS
2197	PAICS	Stromal_score	1.551774e−09	−0.3954019	4.118252e−05	NEG	ZDHHC15	Stromal_score	1.086421e−24	0.6226611	2.883253e−20	POS
2198	LOC100506844	Stromal_score	4.174136e−08	0.3616453	1.107774e−03	POS	HIST1H2AB	Stromal_score	4.900364e−07	−0.3335665	1.300508e−02	NEG
2199	TMEM62	Stromal_score	5.767209e−10	−0.4048466	1.530559e−05	NEG	AL833634	Stromal_score	1.296990e−07	0.3490599	3.442081e−03	POS
2200	GOT1	Stromal_score	7.051745e−09	−0.3803546	1.871462e−04	NEG	KIAA1875	Stromal_score	2.592299e−07	−0.3410934	6.879702e−03	NEG
2201	MTL5	Stromal_score	7.401408e−15	−0.4956934	1.964260e−10	NEG	TRIM74	Stromal_score	1.244798e−09	−0.3975310	3.303568e−05	NEG
2202	RBBP8	Stromal_score	2.137109e−07	−0.3433371	5.671674e−03	NEG	AK308867	Stromal_score	7.050216e−07	−0.3291753	1.871057e−02	NEG
2203	INCENP	Stromal_score	3.761934e−12	−0.4487868	9.983797e−08	NEG	LOC401127	Stromal_score	7.607867e−07	−0.3282476	2.019052e−02	NEG
2204	ZNF774	Stromal_score	2.978610e−07	−0.3394683	7.904934e−03	NEG	KIAA2022	Stromal_score	1.929041e−12	0.4541713	5.119482e−08	POS
2205	PDCD2L	Stromal_score	1.525588e−08	−0.3723809	4.048757e−04	NEG	FXYD7	Stromal_score	8.356525e−08	0.3540048	2.217738e−03	POS
2206	FES	Stromal_score	8.460426e−11	0.4223609	2.245312e−06	POS	C10orf111	Stromal_score	5.037253e−11	−0.4269243	1.336836e−06	NEG
2207	MCM6	Stromal_score	6.824102e−07	−0.3295716	1.811048e−02	NEG	FAM27E3	Stromal_score	1.245989e−06	−0.3221598	3.306731e−02	NEG
2208	AFF3	Stromal_score	9.167202e−15	0.4941960	2.432884e−10	POS	BC039681	Stromal_score	1.876266e−08	−0.3702059	4.979423e−04	NEG
2209	KIF21A	Stromal_score	3.169844e−08	−0.3646201	8.412448e−04	NEG	AX747440	Stromal_score	2.892249e−07	−0.3398133	7.675740e−03	NEG
2210	PCBD1	Stromal_score	3.340784e−09	−0.3878750	8.866107e−05	NEG	CCDC89	Stromal_score	1.115115e−10	0.4199025	2.959403e−06	POS
2211	VEGFC	Stromal_score	9.894824e−29	0.6619349	2.625987e−24	POS	LOC100507632	Stromal_score	4.051214e−15	0.4998721	1.075152e−10	POS
2212	PTPN3	Stromal_score	7.610800e−13	−0.4615152	2.019830e−08	NEG	KCNC2	Stromal_score	9.177112e−07	0.3259490	2.435514e−02	POS
2213	PPAT	Stromal_score	1.066715e−12	−0.4588696	2.830954e−08	NEG	AX747174	Stromal_score	1.020018e−09	0.3994418	2.707026e−05	POS
2214	SPESP1	Stromal_score	2.434521e−08	0.3674448	6.460974e−04	POS	ZNF843	Stromal_score	1.292754e−09	0.3971670	3.430840e−05	POS
2215	DIP2C	Stromal_score	6.749087e−07	0.3297059	1.791140e−02	POS	D28359	Stromal_score	6.709221e−07	−0.3297778	1.780560e−02	NEG
2216	CHAF1A	Stromal_score	4.121728e−10	−0.4079859	1.093865e−05	NEG	C8orf48	Stromal_score	3.786559e−15	0.5003371	1.004915e−10	POS
2217	PHYHIP	Stromal_score	7.897462e−08	0.3546343	2.095908e−03	POS	CRHBP	Stromal_score	1.678557e−09	0.3946396	4.454723e−05	POS
2218	KCNB1	Stromal_score	7.087296e−09	0.3803033	1.880898e−04	POS	UG0898H09	Stromal_score	1.213641e−06	0.3224878	3.220883e−02	POS
2219	XYLT1	Stromal_score	2.574385e−07	0.3411743	6.832159e−03	POS	HTR7	Stromal_score	6.043922e−10	0.4044061	1.603996e−05	POS
2220	QKI	Stromal_score	2.027736e−31	0.6851498	5.381408e−27	POS	NHLRC4	Stromal_score	2.060618e−08	0.3692153	5.468674e−04	POS
2221	ARHGEF15	Stromal_score	1.437851e−18	0.5501833	3.815913e−14	POS	HDX	Stromal_score	6.975354e−13	0.4621947	1.851189e−08	POS
2222	STXBP2	Stromal_score	5.316979e−08	−0.3590043	1.411073e−03	NEG	BC065754	Stromal_score	1.691111e−09	0.3945672	4.488039e−05	POS
2223	GSTM5	Stromal_score	6.807141e−14	0.4797979	1.806547e−09	POS	AB075492	Stromal_score	1.222331e−07	−0.3497318	3.243945e−03	NEG
2224	BC040700	Stromal_score	9.434177e−08	−0.3526488	2.503736e−03	NEG	LRFN5	Stromal_score	7.426207e−18	0.5404481	1.970841e−13	POS
2225	TUBA1C	Stromal_score	9.856408e−09	−0.3769211	2.615792e−04	NEG	PPFIA2	Stromal_score	2.265679e−10	0.4134986	6.012886e−06	POS
2226	FRY	Stromal_score	3.819055e−09	0.3865420	1.013539e−04	POS	AK124950	Stromal_score	1.553666e−09	0.3953901	4.123274e−05	POS
2227	ABCB1	Stromal_score	5.728323e−09	0.3824661	1.520240e−04	POS	EMX2OS	Stromal_score	3.834869e−10	0.4086558	1.017736e−05	POS
2228	PANK1	Stromal_score	5.783519e−10	−0.4048201	1.534888e−05	NEG	TDRD10	Stromal_score	2.050865e−16	0.5197572	5.442790e−12	POS
2229	BC035392	Stromal_score	3.796899e−12	−0.4487115	1.007659e−07	NEG	DMGDH	Stromal_score	5.359090e−14	0.4815514	1.422249e−09	POS
2230	THBS3	Stromal_score	8.217818e−16	0.5106662	2.180927e−11	POS	TMEM155	Stromal_score	5.192868e−07	0.3328712	1.378135e−02	POS
2231	AFAP1	Stromal_score	2.821513e−18	0.5462243	7.488013e−14	POS	ADAMTS3	Stromal_score	7.360141e−18	0.5405019	1.953308e−13	POS
2232	CCDC90A	Stromal_score	3.511444e−08	−0.3635174	9.319020e−04	NEG	POLN	Stromal_score	1.018608e−06	−0.3246623	2.703283e−02	NEG
2233	SLC45A4	Stromal_score	1.071123e−08	−0.3760620	2.842654e−04	NEG	JPH4	Stromal_score	1.951191e−10	0.4148598	5.178266e−06	POS
2234	AL133111	Stromal_score	3.741688e−19	0.5579357	9.930066e−15	POS	XKR4	Stromal_score	8.973752e−10	0.4006645	2.381544e−05	POS
2235	MARCKS	Stromal_score	4.154984e−12	0.4479775	1.102691e−07	POS	DQ586822	Stromal_score	4.017786e−08	0.3620597	1.066280e−03	POS
2236	BC015688	Stromal_score	3.849869e−07	−0.3364420	1.021717e−02	NEG	SNORD74	Stromal_score	1.835398e−10	−0.4154153	4.870962e−06	NEG
2237	SMC2	Stromal_score	2.547558e−09	−0.3905575	6.760964e−05	NEG	AJ606316	Stromal_score	7.994489e−10	0.4017632	2.121657e−05	POS
2238	AK057379	Stromal_score	2.596254e−08	0.3667589	6.890197e−04	POS	GPR89B	Stromal_score	1.088889e−08	−0.3758918	2.889802e−04	NEG
2239	RECK	Stromal_score	2.194778e−33	0.7008563	5.824723e−29	POS	ACRV1	Stromal_score	3.938965e−09	−0.3862331	1.045362e−04	NEG
2240	NHSL2	Stromal_score	2.457928e−22	0.5968935	6.523095e−18	POS	C15orf56	Stromal_score	3.536146e−07	−0.3374482	9.384577e−03	NEG
2241	SWAP70	Stromal_score	5.807306e−14	0.4809636	1.541201e−09	POS	CTAGE7P	Stromal_score	7.033928e−10	−0.4029756	1.866734e−05	NEG
2242	FMNL3	Stromal_score	3.312791e−21	0.5836529	8.791817e−17	POS	AK298300	Stromal_score	9.160152e−12	0.4414624	2.431013e−07	POS
2243	HSPA12A	Stromal_score	6.968015e−08	0.3560245	1.849242e−03	POS	FAM218A	Stromal_score	1.426541e−10	0.4176929	3.785897e−06	POS
2244	SNORD79	Stromal_score	2.061408e−11	−0.4346307	5.470770e−07	NEG	LOC728558	Stromal_score	3.163165e−09	0.3884176	8.394724e−05	POS
2245	RFC4	Stromal_score	8.434663e−13	−0.4607121	2.238475e−08	NEG	ARMC4	Stromal_score	1.330875e−06	0.3213364	3.532009e−02	POS
2246	SASH1	Stromal_score	5.831813e−15	0.4973528	1.547705e−10	POS	LOC100128788	Stromal_score	1.011355e−07	−0.3518686	2.684036e−03	NEG
2247	KIF22	Stromal_score	1.194840e−08	−0.3749292	3.170987e−04	NEG	LCA5L	Stromal_score	4.285070e−08	−0.3613602	1.137215e−03	NEG
2248	LGI4	Stromal_score	4.288845e−18	0.5437385	1.138217e−13	POS	DAZL	Stromal_score	6.581041e−11	0.4245802	1.746542e−06	POS
2249	TDRKH	Stromal_score	5.084026e−07	−0.3331255	1.349250e−02	NEG	HPCAL4	Stromal_score	5.605188e−07	0.3319522	1.487561e−02	POS
2250	CDCA4	Stromal_score	7.330863e−08	−0.3554617	1.945538e−03	NEG	CYP27C1	Stromal_score	3.007755e−08	0.3651840	7.982282e−04	POS
2251	PIGU	Stromal_score	8.351615e−09	−0.3786248	2.216435e−04	NEG	ZNF252P−AS1	Stromal_score	2.033978e−09	−0.3927673	5.397974e−05	NEG
2252	LMO1	Stromal_score	1.164404e−08	0.3751970	3.090213e−04	POS	SCARNA15	Stromal_score	4.688241e−11	−0.4275509	1.244212e−06	NEG
2253	C17orf53	Stromal_score	1.895796e−12	−0.4543102	5.031253e−08	NEG	LOC100628307	Stromal_score	3.048762e−07	0.3391951	8.091110e−03	POS
2254	MERTK	Stromal_score	1.914906e−07	0.3446050	5.081969e−03	POS	HSP90AB3P	Stromal_score	4.190953e−08	−0.3616016	1.112237e−03	NEG
2255	KIAA1958	Stromal_score	7.487108e−09	−0.3797432	1.987004e−04	NEG	BC052775	Stromal_score	4.423404e−08	−0.3610145	1.173927e−03	NEG
2256	PRELID2	Stromal_score	2.029159e−09	−0.3927905	5.385186e−05	NEG	AK055876	Stromal_score	2.007972e−07	−0.3440576	5.328957e−03	NEG
2257	PRR5−ARHGAP8	Stromal_score	5.515240e−09	−0.3828496	1.463689e−04	NEG	KLHL31	Stromal_score	5.625160e−07	−0.3319094	1.492861e−02	NEG
2258	KDELC2	Stromal_score	2.596964e−10	0.4122502	6.892082e−06	POS	RORB	Stromal_score	1.938321e−12	0.4541329	5.144110e−08	POS
2259	DOLPP1	Stromal_score	1.572813e−08	−0.3720614	4.174088e−04	NEG	LOC728758	Stromal_score	1.601470e−09	−0.3950961	4.250141e−05	NEG
2260	AK298596	Stromal_score	1.976013e−13	−0.4718610	5.244141e−09	NEG	GPR62	Stromal_score	6.258740e−07	−0.3306204	1.661007e−02	NEG
2261	GINS3	Stromal_score	1.037707e−10	−0.4205449	2.753971e−06	NEG	DSCR9	Stromal_score	3.185692e−07	−0.3386787	8.454507e−03	NEG
2262	FGD1	Stromal_score	4.925256e−09	0.3839912	1.307114e−04	POS	DCAF12L2	Stromal_score	1.320298e−10	0.4183890	3.503938e−06	POS
2263	FNBP1L	Stromal_score	5.221918e−09	−0.3834016	1.385845e−04	NEG	CACNB4	Stromal_score	9.344382e−07	0.3257266	2.479906e−02	POS
2264	LYAR	Stromal_score	5.776077e−07	−0.3315902	1.532913e−02	NEG	BC036850	Stromal_score	9.360725e−19	0.5526770	2.484243e−14	POS
2265	FBXO5	Stromal_score	4.875555e−09	−0.3840933	1.293924e−04	NEG	DNAJC9−AS1	Stromal_score	1.319370e−09	−0.3969705	3.501476e−05	NEG
2266	STAT5A	Stromal_score	3.354501e−08	0.3640106	8.902510e−04	POS	RNF165	Stromal_score	9.569808e−18	0.5389159	2.539731e−13	POS
2267	MAP9	Stromal_score	1.074973e−07	0.3511823	2.852871e−03	POS	SLITRK4	Stromal_score	1.140743e−10	0.4196993	3.027417e−06	POS
2268	CD28	Stromal_score	4.668547e−08	0.3604266	1.238986e−03	POS	BET3L	Stromal_score	5.771831e−08	0.3581030	1.531786e−03	POS
2269	LOC100126784	Stromal_score	1.162614e−08	0.3752130	3.085461e−04	POS	LOC100130348	Stromal_score	2.590453e−09	−0.3903929	6.874803e−05	NEG
2270	FAM110D	Stromal_score	2.788114e−17	0.5323681	7.399376e−13	POS	BC146941	Stromal_score	3.476972e−07	−0.3376475	9.227536e−03	NEG
2271	ST3GAL5	Stromal_score	3.178313e−10	0.4103928	8.434924e−06	POS	LOC100507462	Stromal_score	1.707894e−09	−0.3944712	4.532581e−05	NEG
2272	ZMIZ1	Stromal_score	2.292637e−09	0.3915944	6.084429e−05	POS	AGAP8	Stromal_score	5.611202e−08	−0.3584133	1.489157e−03	NEG
2273	PUSL1	Stromal_score	3.552238e−07	−0.3373945	9.427284e−03	NEG	AL832163	Stromal_score	3.501471e−08	0.3635481	9.292555e−04	POS
2274	BRIX1	Stromal_score	1.396270e−12	−0.4567435	3.705562e−08	NEG	KIRREL3	Stromal_score	8.444241e−12	0.4421395	2.241017e−07	POS
2275	TTLL4	Stromal_score	1.886814e−12	−0.4543481	5.007415e−08	NEG	DGKI	Stromal_score	2.694700e−07	0.3406411	7.151463e−03	POS
2276	C11orf35	Stromal_score	4.930277e−11	−0.4271118	1.308446e−06	NEG	AK123878	Stromal_score	4.172395e−16	0.5151394	1.107312e−11	POS
2277	PCDH10	Stromal_score	7.982092e−12	0.4426069	2.118367e−07	POS	AK123543	Stromal_score	3.192793e−13	0.4682195	8.473354e−09	POS
2278	HSD17B6	Stromal_score	4.078298e−09	0.3858855	1.082340e−04	POS	PRDM12	Stromal_score	8.202589e−07	−0.3273273	2.176885e−02	NEG
2279	SUV39H1	Stromal_score	2.941998e−12	−0.4507797	7.807769e−08	NEG	AK123934	Stromal_score	2.088598e−09	0.3925080	5.542929e−05	POS
2280	TMTC4	Stromal_score	3.270211e−07	−0.3383705	8.678814e−03	NEG	TCTEX1D1	Stromal_score	3.165899e−12	0.4501865	8.401980e−08	POS

	gene	feature	p.value	estimate	adj.p.value	COR.ID	gene	feature	p.value	estimate	adj.p.value	COR.ID
2281	REM1	Stromal_score	1.031413e−16	0.5241603	2.737268e−12	POS	IRX1	Stromal_score	1.509068e−08	0.3724949	4.004914e−04	POS
2282	SGOL2	Stromal_score	1.416799e−13	−0.4743607	3.760042e−09	NEG	AK055957	Stromal_score	2.259945e−07	0.3426895	5.997667e−03	POS
2283	COX4I2	Stromal_score	1.567486e−06	0.3192813	4.159951e−02	POS	LEKR1	Stromal_score	1.280080e−09	−0.3972619	3.397204e−05	NEG
2284	SH2B3	Stromal_score	7.803440e−14	0.4787919	2.070955e−09	POS	AK094934	Stromal_score	1.360108e−07	−0.3485203	3.609592e−03	NEG
2285	MYCT1	Stromal_score	6.856106e−21	0.5798394	1.819542e−16	POS	HCG23	Stromal_score	1.258108e−06	0.3220390	3.338894e−02	POS
2286	POLE	Stromal_score	5.148278e−09	−0.3835448	1.366302e−04	NEG	ZCCHC18	Stromal_score	4.332837e−11	0.4282373	1.149892e−06	POS
2287	SPSB1	Stromal_score	1.721683e−20	0.5749395	4.569174e−16	POS	EXTL1	Stromal_score	1.154285e−15	0.5083990	3.063357e−11	POS
2288	FIGNL1	Stromal_score	1.011329e−11	−0.4406368	2.683965e−07	NEG	SNORA40	Stromal_score	7.730754e−07	−0.3280519	2.051665e−02	NEG
2289	CIRBP	Stromal_score	2.217115e−09	0.3919230	5.884002e−05	POS	LOC440300	Stromal_score	7.104495e−08	−0.3558095	1.885462e−03	NEG
2290	SORCS2	Stromal_score	1.329155e−18	0.5506416	3.527444e−14	POS	PHOX2B	Stromal_score	4.185060e−07	0.3354504	1.110673e−02	POS
2291	TBC1D1	Stromal_score	4.615012e−07	0.3342843	1.224778e−02	POS	SNORD77	Stromal_score	3.255955e−12	−0.4499593	8.640978e−08	NEG
2292	DBF4	Stromal_score	7.916485e−14	−0.4786858	2.100956e−09	NEG	ANP32C	Stromal_score	3.448901e−07	−0.3377432	9.153038e−03	NEG
2293	MRPL14	Stromal_score	1.651121e−10	−0.4163736	4.381911e−06	NEG	NTRK1	Stromal_score	5.760200e−07	0.3316234	1.528700e−02	POS
2294	POLQ	Stromal_score	1.600775e−11	−0.4367772	4.248298e−07	NEG	EPHA6	Stromal_score	2.934345e−12	0.4508007	7.787458e−08	POS
2295	ATAD3A	Stromal_score	1.332957e−10	−0.4183032	3.537534e−06	NEG	BC043578	Stromal_score	3.482608e−09	0.3874614	9.242492e−05	POS
2296	FAM221A	Stromal_score	4.644788e−11	−0.4276320	1.232680e−06	NEG	DQ570882	Stromal_score	4.783280e−09	0.3842856	1.269435e−04	POS
2297	BC011648	Stromal_score	5.163787e−07	−0.3329386	1.370417e−02	NEG	BC128131	Stromal_score	2.928508e−11	0.4316255	7.771968e−07	POS
2298	TMEM45A	Stromal_score	4.314318e−11	0.4282746	1.144977e−06	POS	ANKRD55	Stromal_score	2.925511e−07	0.3396793	7.764015e−03	POS
2299	TIPIN	Stromal_score	7.352570e−10	−0.4025565	1.951299e−05	NEG	TRIM61	Stromal_score	5.586714e−13	0.4639182	1.482658e−08	POS
2300	RPIA	Stromal_score	1.028305e−09	−0.3993644	2.729020e−05	NEG	ZBTB20−AS1	Stromal_score	4.474668e−11	0.4279570	1.187532e−06	POS
2301	CLN6	Stromal_score	7.750342e−10	−0.4020574	2.056863e−05	NEG	AL832797	Stromal_score	4.786657e−13	0.4651124	1.270331e−08	POS
2302	NIPAL4	Stromal_score	7.172981e−08	0.3557032	1.903637e−03	POS	GRIA1	Stromal_score	1.155158e−06	0.3231025	3.065675e−02	POS
2303	CYCS	Stromal_score	3.604972e−09	−0.3871175	9.567236e−05	NEG	NT5C1A	Stromal_score	1.364589e−06	0.3210232	3.621482e−02	POS
2304	FAM26E	Stromal_score	4.549631e−17	0.5293216	1.207427e−12	POS	UNQ2565	Stromal_score	3.162837e−11	0.4309627	8.393854e−07	POS
2305	LOC286190	Stromal_score	1.391994e−06	−0.3207740	3.694212e−02	NEG	AK094929	Stromal_score	4.994381e−12	0.4464734	1.325459e−07	POS
2306	GGCT	Stromal_score	1.779489e−08	−0.3707641	4.722586e−04	NEG	MAB21L1	Stromal_score	1.126099e−09	0.3984940	2.988554e−05	POS
2307	KCNJ11	Stromal_score	6.287131e−10	−0.4040347	1.668542e−05	NEG	LOC283856	Stromal_score	3.957390e−13	0.4665761	1.050252e−08	POS
2308	TOMM5	Stromal_score	9.070034e−08	−0.3530896	2.407096e−03	NEG	GDF5	Stromal_score	3.698926e−07	0.3369159	9.816579e−03	POS
2309	SEH1L	Stromal_score	1.689981e−06	−0.3183312	4.485041e−02	NEG	ATP6V0CP3	Stromal_score	5.845497e−10	0.4047199	1.551336e−05	POS
2310	MYO6	Stromal_score	1.277133e−06	−0.3218517	3.389383e−02	NEG	CACNA1G	Stromal_score	1.030660e−07	0.3516560	2.735267e−03	POS
2311	FANCG	Stromal_score	1.574996e−08	−0.3720469	4.179882e−04	NEG	WHAMMP2	Stromal_score	5.696005e−12	0.4453944	1.511663e−07	POS
2312	BRCA1	Stromal_score	4.067921e−10	−0.4081080	1.079586e−05	NEG	CCDC36	Stromal_score	7.335858e−12	0.4433067	1.946863e−07	POS
2313	AX746750	Stromal_score	1.626126e−11	−0.4366443	4.315575e−07	NEG	TMEM221	Stromal_score	6.021521e−08	0.3576369	1.598051e−03	POS
2314	SH3RF3	Stromal_score	2.542162e−17	0.5329392	6.746645e−13	POS	LOC100506795	Stromal_score	4.616938e−12	0.4471167	1.225289e−07	POS
2315	CIT	Stromal_score	5.504019e−10	−0.4052851	1.460712e−05	NEG	CCIN	Stromal_score	3.098226e−13	0.4684490	8.222383e−09	POS
2316	CBX8	Stromal_score	9.622797e−10	−0.3999986	2.553794e−05	NEG	DL490294	Stromal_score	9.147647e−10	0.4004816	2.427694e−05	POS
2317	CYP27B1	Stromal_score	1.727301e−06	−0.3180548	4.584083e−02	NEG	AK055351	Stromal_score	8.299533e−08	−0.3540812	2.202613e−03	NEG
2318	CCHCR1	Stromal_score	3.500824e−11	−0.4300862	9.290837e−07	NEG	CR933665	Stromal_score	1.677320e−10	−0.4162313	4.451439e−06	NEG
2319	CX3CR1	Stromal_score	1.363589e−06	0.3210324	3.618828e−02	POS	C10orf105	Stromal_score	1.626071e−06	0.3188184	4.315431e−02	POS
2320	MMP19	Stromal_score	7.396341e−19	0.5540366	1.962915e−14	POS	MYOZ2	Stromal_score	5.812744e−11	0.4256710	1.542644e−06	POS
2321	AP1G2	Stromal_score	3.317971e−09	−0.3879431	8.805563e−05	NEG	LOC644649	Stromal_score	8.529952e−07	−0.3268476	2.263764e−02	NEG
2322	TRMT6	Stromal_score	2.254812e−07	−0.3427159	5.984046e−03	NEG	BC050301	Stromal_score	6.522728e−07	0.3301199	1.731067e−02	POS
2323	CSGALNACT1	Stromal_score	6.321720e−08	0.3571006	1.677721e−03	POS	BX248253	Stromal_score	1.669489e−17	0.5355261	4.430656e−13	POS
2324	PDRG1	Stromal_score	1.026425e−09	−0.3993819	2.724031e−05	NEG	DNM1P46	Stromal_score	2.124027e−07	0.3434082	5.636956e−03	POS
2325	FAT4	Stromal_score	1.530517e−18	0.5498187	4.061839e−14	POS	AY660578	Stromal_score	4.633114e−09	−0.3846064	1.229582e−04	NEG
2326	TLR7	Stromal_score	7.324402e−10	0.4025928	1.943823e−05	POS	ABCG4	Stromal_score	3.841504e−08	0.3625461	1.019497e−03	POS
2327	SCARNA22	Stromal_score	1.100251e−06	−0.3237074	2.919957e−02	NEG	CNTNAP3B	Stromal_score	2.015776e−08	0.3694482	5.349667e−04	POS
2328	PIP4K2A	Stromal_score	2.505729e−16	0.5184618	6.649955e−12	POS	CASP12	Stromal_score	4.865433e−11	0.4272273	1.291237e−06	POS
2329	ZMYND19	Stromal_score	1.013442e−13	−0.4768579	2.689574e−09	NEG	C10orf140	Stromal_score	3.752519e−07	0.3367456	9.958810e−03	POS
2330	CSTF2	Stromal_score	8.191174e−10	−0.4015324	2.173856e−05	NEG	SLX1B	Stromal_score	9.620008e−09	−0.3771713	2.553054e−04	NEG
2331	PLA2G5	Stromal_score	1.844354e−08	0.3703868	4.894732e−04	POS	KIAA0825	Stromal_score	1.012263e−06	0.3247395	2.686445e−02	POS
2332	LRRC1	Stromal_score	2.495407e−08	−0.3671815	6.622561e−04	NEG	RCVRN	Stromal_score	6.354402e−13	0.4629197	1.686395e−08	POS
2333	STAT2	Stromal_score	1.602312e−08	0.3718665	4.252375e−04	POS	AK096335	Stromal_score	3.689614e−12	0.4489446	9.791868e−08	POS
2334	DCAF13	Stromal_score	3.309102e−08	−0.3641574	8.782026e−04	NEG	BC035247	Stromal_score	1.111954e−06	−0.3235761	2.951016e−02	NEG
2335	SLC35A2	Stromal_score	7.006240e−11	−0.4240286	1.859386e−06	NEG	LOC401463	Stromal_score	3.055877e−19	0.5590844	8.109991e−15	POS
2336	BORA	Stromal_score	5.974543e−15	−0.4971849	1.585584e−10	NEG	AX747213	Stromal_score	8.185775e−08	−0.3542350	2.172423e−03	NEG
2337	PCDHB15	Stromal_score	2.419121e−11	0.4332647	6.420105e−07	POS	USP44	Stromal_score	8.801779e−10	0.4008488	2.335904e−05	POS
2338	LITAF	Stromal_score	1.379401e−06	0.3208879	3.660791e−02	POS	RGS22	Stromal_score	2.017528e−11	0.4348139	5.354316e−07	POS
2339	MYO19	Stromal_score	2.816427e−10	−0.4115056	7.474514e−06	NEG	CYP1B1−AS1	Stromal_score	1.259664e−13	0.4752392	3.343022e−09	POS
2340	EME1	Stromal_score	5.934855e−09	−0.3821073	1.575051e−04	NEG	ARMC12	Stromal_score	5.053507e−07	0.3331977	1.341150e−02	POS
2341	CLCN5	Stromal_score	7.278949e−07	−0.3287866	1.931760e−02	NEG	C20orf152	Stromal_score	1.054787e−10	−0.4203992	2.799300e−06	NEG
2342	GYG1	Stromal_score	3.067258e−09	0.3887229	8.140195e−05	POS	STL	Stromal_score	2.380505e−11	0.4334024	6.317622e−07	POS
2343	SEMA4C	Stromal_score	2.556814e−07	0.3412542	6.785527e−03	POS	AL050132	Stromal_score	6.942224e−11	0.4241095	1.842397e−06	POS
2344	NUDT15	Stromal_score	3.876965e−11	−0.4292027	1.028908e−06	NEG	KHDRBS2	Stromal_score	5.902041e−07	0.3313298	1.566343e−02	POS
2345	QSOX2	Stromal_score	2.645713e−07	−0.3408554	7.021457e−03	NEG	SCN11A	Stromal_score	4.646794e−09	0.3845767	1.233213e−04	POS
2346	MCC	Stromal_score	3.984233e−28	0.6564099	1.057376e−23	POS	WNK3	Stromal_score	1.779660e−15	0.5054855	4.723041e−11	POS
2347	CACNB2	Stromal_score	1.530417e−15	0.5065040	4.061572e−11	POS	STMN4	Stromal_score	2.707891e−08	0.3663091	7.186472e−04	POS
2348	KCNA5	Stromal_score	3.232132e−11	0.4307758	8.577754e−07	POS	SULT1A3	Stromal_score	3.681888e−07	−0.3369705	9.771362e−03	NEG
2349	POLD2	Stromal_score	3.745710e−12	−0.4488219	9.940741e−08	NEG	CLEC4F	Stromal_score	6.532274e−07	0.3301021	1.733600e−02	POS
2350	SNORD58C	Stromal_score	1.126524e−08	−0.3755399	2.989681e−04	NEG	LOXHD1	Stromal_score	1.781035e−06	0.3176666	4.726688e−02	POS
2351	ERCC6L	Stromal_score	1.759171e−13	−0.4727367	4.668665e−09	NEG	AX747189	Stromal_score	8.135298e−08	0.3543039	2.159027e−03	POS
2352	B3GNT9	Stromal_score	1.112942e−08	0.3756656	2.953636e−04	POS	C20orf166	Stromal_score	6.765305e−08	0.3563513	1.795444e−03	POS
2353	DTYMK	Stromal_score	1.932001e−12	−0.4541590	5.127338e−08	NEG	RNF175	Stromal_score	4.548669e−07	0.3344573	1.207171e−02	POS
2354	SNORD110	Stromal_score	1.119649e−08	−0.3756034	2.971437e−04	NEG	SNORD113−4	Stromal_score	7.859427e−09	0.3792470	2.085813e−04	POS
2355	HPGDS	Stromal_score	9.523554e−07	0.3254926	2.527456e−02	POS	KLHL33	Stromal_score	2.903986e−08	0.3655606	7.706888e−04	POS
2356	BEND7	Stromal_score	4.477186e−07	−0.3346463	1.188200e−02	NEG	BC039516	Stromal_score	3.568697e−09	0.3872182	9.470966e−05	POS
2357	P4HA3	Stromal_score	3.741533e−11	0.4295108	9.929655e−07	POS	BC039319	Stromal_score	1.063818e−06	0.3241248	2.823267e−02	POS
2358	MEF2A	Stromal_score	4.066038e−16	0.5153085	1.079086e−11	POS	SLCO1C1	Stromal_score	1.994010e−08	0.3695630	5.291904e−04	POS
2359	C3orf64	Stromal_score	6.774506e−15	0.4963106	1.797886e−10	POS	LOC100506835	Stromal_score	2.603166e−07	0.3410446	6.908543e−03	POS
2360	RNFT2	Stromal_score	1.210104e−09	−0.3978030	3.211496e−05	NEG	LOC100128593	Stromal_score	1.744652e−07	0.3456760	4.630133e−03	POS
2361	MYL6	Stromal_score	3.604357e−10	0.4092303	9.565603e−06	POS	SNORA51	Stromal_score	1.559277e−07	−0.3469627	4.138164e−03	NEG
2362	SLC12A4	Stromal_score	2.294794e−15	0.5037617	6.090154e−11	POS	MS4A4E	Stromal_score	1.493432e−09	0.3957731	3.963420e−05	POS
2363	RANBP1	Stromal_score	9.196191e−07	−0.3259235	2.440577e−02	NEG	AK124194	Stromal_score	2.455370e−07	0.3417258	6.516306e−03	POS
2364	MRPS26	Stromal_score	1.691080e−06	−0.3183230	4.487957e−02	NEG	DIRC3	Stromal_score	5.597739e−17	0.5280226	1.485584e−12	POS
2365	NUP107	Stromal_score	7.747001e−09	−0.3793944	2.055977e−04	NEG	BC045735	Stromal_score	1.299445e−09	0.3971172	3.448598e−05	POS
2366	MCTP1	Stromal_score	5.989891e−12	0.4449804	1.589657e−07	POS	LOC440600	Stromal_score	1.925023e−10	−0.4149825	5.108819e−06	NEG
2367	AK296947	Stromal_score	5.932726e−08	−0.3578005	1.574486e−03	NEG	C18orf34	Stromal_score	2.359478e−09	0.3913121	6.261819e−05	POS
2368	XYLB	Stromal_score	8.800848e−13	−0.4603795	2.335657e−08	NEG	LOC284276	Stromal_score	5.281938e−08	0.3590768	1.401773e−03	POS
2369	MYO1C	Stromal_score	3.989815e−07	0.3360183	1.058857e−02	POS	LOC100302640	Stromal_score	8.962158e−10	0.4006768	2.378467e−05	POS
2370	CD46	Stromal_score	8.615788e−08	−0.3536639	2.286544e−03	NEG	OR7E2P	Stromal_score	1.272190e−09	0.3973214	3.376264e−05	POS
2371	NECAB3	Stromal_score	2.350040e−08	−0.3678207	6.236771e−04	NEG	PTPRQ	Stromal_score	1.221959e−09	0.3977092	3.242958e−05	POS
2372	PREP	Stromal_score	6.478201e−07	−0.3302029	1.719250e−02	NEG	AK126307	Stromal_score	3.405609e−15	0.5010653	9.038146e−11	POS
2373	PTPLAD1	Stromal_score	2.084333e−09	−0.3925281	5.531612e−05	NEG	LOC643699	Stromal_score	9.713176e−10	0.3999093	2.577780e−05	POS
2374	BPHL	Stromal_score	1.402899e−07	−0.3481680	3.723152e−03	NEG	IL5RA	Stromal_score	5.690477e−07	0.3317702	1.510196e−02	POS
2375	SNORD30	Stromal_score	3.538496e−07	−0.3374403	9.390815e−03	NEG	MGAT4C	Stromal_score	6.863846e−09	0.3806298	1.821596e−04	POS
2376	ADCYAP1	Stromal_score	3.691150e−09	0.3868819	9.795942e−05	POS	HFM1	Stromal_score	7.575588e−08	0.3550970	2.010485e−03	POS
2377	SHMT1	Stromal_score	1.426972e−10	−0.4176902	3.787041e−06	NEG	RFPL1	Stromal_score	4.392170e−11	0.4281190	1.165638e−06	POS
2378	MARS2	Stromal_score	3.009811e−13	−0.4686698	7.987737e−09	NEG	BC042024	Stromal_score	7.500636e−07	0.3284208	1.990594e−02	POS
2379	IL4R	Stromal_score	1.280666e−09	0.3972575	3.398761e−05	POS	FLJ37201	Stromal_score	9.030775e−08	−0.3531381	2.396677e−03	NEG
2380	ADRB2	Stromal_score	2.571138e−14	0.4868741	6.823543e−10	POS	TECTA	Stromal_score	1.913867e−07	0.3446113	5.079211e−03	POS
2381	NAV1	Stromal_score	6.258767e−09	0.3815683	1.661014e−04	POS	LOC100506474	Stromal_score	1.655534e−07	0.3462772	4.393622e−03	POS
2382	FNDC5	Stromal_score	5.723415e−09	0.3824748	1.518937e−04	POS	SNTG2	Stromal_score	1.350650e−09	0.3967445	3.584491e−05	POS
2383	FLJ41200	Stromal_score	6.371245e−12	0.4444717	1.690865e−07	POS	CBWD5	Stromal_score	6.097087e−08	−0.3574995	1.618106e−03	NEG
2384	PSMG3	Stromal_score	4.100873e−12	−0.4480844	1.088331e−07	NEG	LOC100288122	Stromal_score	1.226992e−06	0.3223514	3.256315e−02	POS
2385	PRR16	Stromal_score	1.626802e−06	0.3188127	4.317369e−02	POS	AK090688	Stromal_score	1.081439e−08	0.3759629	2.870032e−04	POS
2386	IKBKE	Stromal_score	1.580736e−07	−0.3468064	4.195114e−03	NEG	ADRA1A	Stromal_score	2.564415e−07	0.3412196	6.805700e−03	POS
2387	RNF217	Stromal_score	1.671619e−18	0.5493032	4.436310e−14	POS	LINC00167	Stromal_score	1.410737e−07	−0.3481046	3.743954e−03	NEG
2388	SNED1	Stromal_score	1.496913e−16	0.5217821	3.972657e−12	POS	Mir_324	Stromal_score	1.729487e−06	−0.3180388	4.589885e−02	NEG
2389	ENOSF1	Stromal_score	1.002172e−06	−0.3248634	2.659665e−02	NEG	CDK15	Stromal_score	5.935444e−08	0.3577955	1.575207e−03	POS
2390	WDR90	Stromal_score	8.454153e−08	−0.3538753	2.243648e−03	NEG	WNT9B	Stromal_score	1.378044e−08	0.3734443	3.657191e−04	POS
2391	XRCC2	Stromal_score	7.129139e−13	−0.4620249	1.892002e−08	NEG	COL25A1	Stromal_score	2.362618e−11	0.4334669	6.270151e−07	POS
2392	CDH19	Stromal_score	1.285271e−09	0.3972229	3.410980e−05	POS	ACSBG2	Stromal_score	2.080108e−07	−0.3436498	5.520399e−03	NEG
2393	GSS	Stromal_score	1.974413e−11	−0.4349977	5.239894e−07	NEG	FNDC9	Stromal_score	1.123341e−06	0.3234496	2.981234e−02	POS
2394	MIR192	Stromal_score	1.298857e−06	−0.3216409	3.447037e−02	NEG	AK094562	Stromal_score	9.308774e−08	0.3527987	2.470456e−03	POS
2395	HIF1A	Stromal_score	4.352641e−07	0.3349828	1.155147e−02	POS	BC042649	Stromal_score	1.121794e−07	0.3507017	2.977128e−03	POS
2396	MAP7D3	Stromal_score	2.409879e−13	0.4703598	6.395578e−09	POS	LINC00475	Stromal_score	4.986801e−10	0.4062094	1.323447e−05	POS
2397	CTIF	Stromal_score	1.231127e−12	0.4577394	3.267288e−08	POS	BC034416	Stromal_score	8.241944e−07	0.3272687	2.187330e−02	POS
2398	IQSEC1	Stromal_score	1.105750e−11	0.4398904	2.934551e−07	POS	CYP46A1	Stromal_score	2.974385e−07	0.3394850	7.893720e−03	POS
2399	SURF2	Stromal_score	3.786255e−08	−0.3627029	1.004834e−03	NEG	RFPL1−AS1	Stromal_score	5.593191e−12	0.4455441	1.484377e−07	POS
2400	IQCJ−SCHIP1	Stromal_score	2.648099e−14	0.4866622	7.027791e−10	POS	LHCGR	Stromal_score	4.323761e−08	0.3612624	1.147483e−03	POS

	gene	feature	p.value	estimate	adj.p.value	COR.ID	gene	feature	p.value	estimate	adj.p.value	COR.ID
2401	CEP85	Stromal_score	1.109502e−10	−0.4199476	2.944507e−06	NEG	KRT8P41	Stromal_score	2.125812e−07	−0.3433985	5.641693e−03	NEG
2402	STK10	Stromal_score	1.835758e−11	0.4356165	4.871919e−07	POS	PURG	Stromal_score	3.157162e−10	0.4104543	8.378791e−06	POS
2403	FKBP7	Stromal_score	1.602253e−17	0.5357778	4.252218e−13	POS	SULT6B1	Stromal_score	1.277898e−08	−0.3742306	3.391413e−04	NEG
2404	FAM70A	Stromal_score	2.901560e−11	0.4317050	7.700450e−07	POS	SLC24A4	Stromal_score	4.165921e−09	0.3856727	1.105594e−04	POS
2405	TIGD5	Stromal_score	1.376277e−08	−0.3734577	3.652501e−04	NEG	GALR1	Stromal_score	1.041679e−07	0.3515364	2.764512e−03	POS
2406	C14orf159	Stromal_score	1.397873e−06	0.3207211	3.709815e−02	POS	DBX2	Stromal_score	3.838457e−09	0.3864914	1.018688e−04	POS
2407	RIMKLB	Stromal_score	6.264942e−07	0.3306084	1.662653e−02	POS	BC043540	Stromal_score	4.145545e−07	0.3355632	1.100186e−02	POS
2408	C1orf31	Stromal_score	1.318669e−06	−0.3214516	3.499616e−02	NEG	OR5K2	Stromal_score	2.005465e−07	0.3440720	5.322304e−03	POS
2409	ARL6IP5	Stromal_score	1.542867e−13	0.4737222	4.094615e−09	POS	LOC643542	Stromal_score	3.807317e−07	0.3365738	1.010424e−02	POS
2410	BYSL	Stromal_score	4.304489e−11	−0.4282944	1.142368e−06	NEG	BC033330	Stromal_score	1.780158e−07	−0.3454446	4.724362e−03	NEG
2411	RUNX1T1	Stromal_score	6.086475e−28	0.6547059	1.615290e−23	POS	IMPG2	Stromal_score	7.317803e−09	0.3799768	1.942072e−04	POS
2412	ZWILCH	Stromal_score	6.923645e−10	−0.4031249	1.837466e−05	NEG	ANKRD30BP2	Stromal_score	7.934235e−07	−0.3277344	2.105667e−02	NEG
2413	PTGIR	Stromal_score	2.396229e−23	0.6082471	6.359352e−19	POS	TXNDC2	Stromal_score	4.029328e−08	0.3620286	1.069343e−03	POS
2414	HOOK3	Stromal_score	5.029577e−11	0.4269376	1.334799e−06	POS	OLFM3	Stromal_score	5.633151e−07	0.3318922	1.494982e−02	POS
2415	BC019904	Stromal_score	2.737272e−07	0.3404579	7.264447e−03	POS	BLID	Stromal_score	2.276803e−09	0.3916624	6.042406e−05	POS
2416	MIR937	Stromal_score	2.628174e−07	−0.3409331	6.974910e−03	NEG	LOC645434	Stromal_score	6.310232e−08	0.3571206	1.674673e−03	POS
2417	KIAA0895	Stromal_score	2.326301e−15	−0.5036689	6.173769e−11	NEG	KIF4B	Stromal_score	4.641190e−09	−0.3845888	1.231725e−04	NEG
2418	TNFSF14	Stromal_score	6.103761e−07	0.3309237	1.619877e−02	POS	MCF2	Stromal_score	4.087749e−07	0.3357302	1.084848e−02	POS
2419	SNORD22	Stromal_score	1.316257e−07	−0.3488926	3.493214e−03	NEG	AK055854	Stromal_score	1.566786e−06	0.3192869	4.158093e−02	POS
2420	MILR1	Stromal_score	2.301973e−09	0.3915545	6.109207e−05	POS	EXD1	Stromal_score	1.341832e−06	−0.3212338	3.561088e−02	NEG
2421	TMEM41A	Stromal_score	8.819783e−09	−0.3780650	2.340682e−04	NEG	BC038746	Stromal_score	6.473524e−07	0.3302117	1.718009e−02	POS
2422	TNK1	Stromal_score	2.743965e−12	−0.4513423	7.282209e−08	NEG	AK024141	Stromal_score	6.387079e−09	0.3813622	1.695067e−04	POS
2423	C3orf52	Stromal_score	8.098503e−10	−0.4016404	2.149262e−05	NEG	TTTY10	Stromal_score	1.452462e−08	0.3728949	3.854689e−04	POS
2424	NTAN1	Stromal_score	1.057124e−10	0.4203795	2.805501e−06	POS	CTAGE1	Stromal_score	8.714336e−07	−0.3265851	2.312698e−02	NEG
2425	RBL1	Stromal_score	1.731359e−10	−0.4159442	4.594853e−06	NEG	AK127472	Stromal_score	1.539552e−08	0.3722855	4.085816e−04	POS
2426	SEC23A	Stromal_score	7.051184e−15	0.4960316	1.871314e−10	POS	BC040219	Stromal_score	3.475416e−07	0.3376528	9.223407e−03	POS
2427	UNG	Stromal_score	8.993615e−07	−0.3261975	2.386816e−02	NEG	LOC254312	Stromal_score	1.856168e−08	0.3703195	4.926083e−04	POS
2428	HLX	Stromal_score	1.625891e−15	0.5060959	4.314952e−11	POS	GPR26	Stromal_score	9.191864e−08	0.3529402	2.439429e−03	POS
2429	TMEM59L	Stromal_score	1.397570e−11	0.4379232	3.709011e−07	POS	PZP	Stromal_score	1.986430e−11	0.4349461	5.271787e−07	POS
2430	CCDC50	Stromal_score	1.165828e−14	0.4925049	3.093991e−10	POS	GRM6	Stromal_score	2.843553e−07	0.3400122	7.546506e−03	POS
2431	HM13	Stromal_score	1.358866e−06	−0.3210758	3.606294e−02	NEG	LOC100132987	Stromal_score	6.468962e−10	0.4037660	1.716798e−05	POS
2432	AK055386	Stromal_score	7.533107e−11	0.4233885	1.999211e−06	POS	BC035181	Stromal_score	5.962200e−11	0.4254482	1.582308e−06	POS
2433	HCFC1R1	Stromal_score	2.772489e−10	0.4116501	7.357909e−06	POS	FAM48B1	Stromal_score	5.642314e−08	0.3583525	1.497414e−03	POS
2434	DCTPP1	Stromal_score	2.358644e−07	−0.3421932	6.259606e−03	NEG	FLJ34690	Stromal_score	5.211098e−07	0.3328291	1.382973e−02	POS
2435	RPL8	Stromal_score	6.048345e−10	−0.4043992	1.605170e−05	NEG	AX746487	Stromal_score	8.357648e−07	0.3270979	2.218036e−02	POS
2436	SUV420H2	Stromal_score	2.679959e−07	−0.3407052	7.112342e−03	NEG	SNORD112	Stromal_score	1.184062e−06	0.3227951	3.142382e−02	POS
2437	CCDC58	Stromal_score	6.685215e−09	−0.3808983	1.774189e−04	NEG	PCDH11Y	Stromal_score	1.808689e−06	0.3174712	4.800080e−02	POS
2438	AFMID	Stromal_score	2.269774e−11	−0.4338095	6.023754e−07	NEG	DKFZP434H168	Stromal_score	1.136240e−08	0.3754509	3.015468e−04	POS
2439	BID	Stromal_score	1.958463e−08	−0.3697531	5.197564e−04	NEG	LOC100507584	Stromal_score	1.865162e−06	0.3170808	4.949953e−02	POS
2440	ZNF362	Stromal_score	1.050983e−13	0.4765878	2.789205e−09	POS	MIR764	Stromal_score	1.783599e−06	0.3176484	4.733493e−02	POS
2441	FAM160A1	Stromal_score	4.490827e−09	−0.3849197	1.191821e−04	NEG	MIR4272	Stromal_score	1.535149e−06	0.3195439	4.074133e−02	POS
2442	ADCY2	Stromal_score	1.662490e−08	0.3714795	4.412081e−04	POS	MIR3119−1	Stromal_score	1.042586e−06	−0.3243745	2.766918e−02	NEG
2443	C14orf80	Stromal_score	7.112867e−10	−0.4028701	1.887684e−05	NEG	MIR3119−2	Stromal_score	1.042586e−06	−0.3243745	2.766918e−02	NEG

Supporting Material 5D:
Stromal score correlated genes (vTCGA−GCC)

	gene	feature	p.value	estimate	adj.p.value	COR.ID	gene	feature	p.value	estimate	adj.p.value	COR.ID
1	CNN1|ENSG00000130176	Stromal_score	1.802662e−07	0.4351771	4.122508e−03	POS	MEOX1|ENSG00000005102	Stromal_score	4.825790e−07	0.4209004	1.103610e−02	POS
2	HSPB6|ENSG00000004776	Stromal_score	4.719420e−08	0.4536338	1.079284e−03	POS	SEMA6B|ENSG00000167680	Stromal_score	5.356548e−08	0.4519364	1.224989e−03	POS
3	SFRP2|ENSG00000145423	Stromal_score	0.000000e+00	0.6054629	0.000000e+00	POS	TCEAL7|ENSG00000182916	Stromal_score	0.000000e+00	0.5602819	0.000000e+00	POS
4	CCL19|ENSG00000172724	Stromal_score	9.361620e−11	0.5207346	2.140909e−06	POS	TLR4|ENSG00000136869	Stromal_score	0.000000e+00	0.5337869	0.000000e+00	POS
5	CCL21|ENSG00000137077	Stromal_score	1.985165e−10	0.5152473	4.539873e−06	POS	NRP1|ENSG00000099250	Stromal_score	0.000000e+00	0.5925276	0.000000e+00	POS
6	OGN|ENSG00000106809	Stromal_score	3.110244e−09	0.4876305	7.112816e−05	POS	MMP19|ENSG00000123342	Stromal_score	0.000000e+00	0.5572627	0.000000e+00	POS
7	THBS4|ENSG00000113296	Stromal_score	1.622465e−10	0.5168325	3.710416e−06	POS	EBF1|ENSG00000164330	Stromal_score	0.000000e+00	0.6562384	0.000000e+00	POS
8	LMOD1|ENSG00000163431	Stromal_score	6.506914e−09	0.4788996	1.488066e−04	POS	OSCAR|ENSG00000170909	Stromal_score	8.398390e−07	0.4125841	1.920628e−02	POS
9	CHRDL2|ENSG00000054938	Stromal_score	8.489460e−09	0.4756560	1.941455e−04	POS	CYYR1|ENSG00000166265	Stromal_score	0.000000e+00	0.5638962	0.000000e+00	POS
10	C3|ENSG00000125730	Stromal_score	8.407667e−08	0.4458150	1.922749e−03	POS	BTK|ENSG00000010671	Stromal_score	2.087449e−08	0.4643352	4.773788e−04	POS
11	SFRP4|ENSG00000106483	Stromal_score	0.000000e+00	0.6781972	0.000000e+00	POS	PARVA|ENSG00000197702	Stromal_score	5.769479e−08	0.4509365	1.319422e−03	POS
12	HSPB7|ENSG00000173641	Stromal_score	1.199802e−06	0.4071164	2.743828e−02	POS	TRPV2|ENSG00000187688	Stromal_score	0.000000e+00	0.5542581	0.000000e+00	POS
13	MYL9|ENSG00000101335	Stromal_score	1.215801e−10	0.5189591	2.780416e−06	POS	PPP1R18|ENSG00000146112	Stromal_score	0.000000e+00	0.6005268	0.000000e+00	POS
14	TAGLN|ENSG00000149591	Stromal_score	0.000000e+00	0.5746952	0.000000e+00	POS	CDC25A|ENSG00000164045	Stromal_score	6.465083e−09	−0.4789777	1.478500e−04	NEG
15	PLN|ENSG00000198523	Stromal_score	6.450115e−10	0.5045996	1.475077e−05	POS	ESAM|ENSG00000149564	Stromal_score	1.757342e−07	0.4355380	4.018865e−03	POS
16	MFAP4|ENSG00000166482	Stromal_score	0.000000e+00	0.5485270	0.000000e+00	POS	LRRC25|ENSG00000175489	Stromal_score	0.000000e+00	0.5456492	0.000000e+00	POS
17	BARX1|ENSG00000131668	Stromal_score	4.552110e−09	0.4831773	1.041022e−04	POS	SH3PXD2A|ENSG00000107957	Stromal_score	1.559435e−06	0.4030387	3.566272e−02	POS
18	FHL1|ENSG00000022267	Stromal_score	2.680628e−07	0.4294996	6.130329e−03	POS	ANK2|ENSG00000145362	Stromal_score	1.664220e−06	0.4020193	3.805905e−02	POS
19	MGP|ENSG00000111341	Stromal_score	0.000000e+00	0.6713930	0.000000e+00	POS	CLCN2|ENSG00000114859	Stromal_score	5.800932e−09	−0.4802849	1.326615e−04	NEG
20	PTGDS|ENSG00000107317	Stromal_score	1.344112e−08	0.4699395	3.073849e−04	POS	BMP8A|ENSG00000183682	Stromal_score	3.452362e−07	0.4258267	7.895206e−03	POS
21	TNC|ENSG00000041982	Stromal_score	3.056188e−08	0.4593845	6.989196e−04	POS	EID1|ENSG00000255302	Stromal_score	1.330720e−06	0.4055117	3.043223e−02	POS
22	COL10A1|ENSG00000123500	Stromal_score	4.498670e−10	0.5080821	1.028801e−05	POS	GLI2|ENSG00000074047	Stromal_score	5.354604e−08	0.4519413	1.224544e−03	POS
23	MYLK|ENSG00000065534	Stromal_score	3.649372e−10	0.5100185	8.345749e−06	POS	DENND5A|ENSG00000184014	Stromal_score	1.199962e−08	0.4713638	2.744193e−04	POS
24	PRELP|ENSG00000188783	Stromal_score	0.000000e+00	0.6107404	0.000000e+00	POS	STX11|ENSG00000135604	Stromal_score	3.056771e−09	0.4878305	6.990529e−05	POS
25	SPARCL1|ENSG00000152583	Stromal_score	0.000000e+00	0.5944493	0.000000e+00	POS	GPR68|ENSG00000119714	Stromal_score	0.000000e+00	0.5936738	0.000000e+00	POS
26	GREM1|ENSG00000166923	Stromal_score	0.000000e+00	0.6150180	0.000000e+00	POS	CLIP4|ENSG00000115295	Stromal_score	1.237847e−06	0.4066335	2.830832e−02	POS
27	ACKR1|ENSG00000213088	Stromal_score	1.688517e−10	0.5165252	3.861470e−06	POS	NR3C1|ENSG00000113580	Stromal_score	7.659457e−10	0.5028778	1.751641e−05	POS
28	FBLN1|ENSG00000077942	Stromal_score	2.758124e−09	0.4890108	6.307554e−05	POS	GNG2|ENSG00000186469	Stromal_score	0.000000e+00	0.5769486	0.000000e+00	POS
29	SPON1|ENSG00000262655	Stromal_score	0.000000e+00	0.5653351	0.000000e+00	POS	LILRB2|ENSG00000131042	Stromal_score	7.131989e−08	0.4480636	1.631015e−03	POS
30	FN1|ENSG00000115414	Stromal_score	1.091641e−09	0.4992147	2.496474e−05	POS	FGR|ENSG00000000938	Stromal_score	3.748782e−07	0.4246220	8.573089e−03	POS
31	ACTA2|ENSG00000107796	Stromal_score	0.000000e+00	0.5974978	0.000000e+00	POS	RHOH|ENSG00000168421	Stromal_score	5.615464e−09	0.4806751	1.284201e−04	POS
32	SMOC2|ENSG00000112562	Stromal_score	8.007500e−09	0.4763730	1.831235e−04	POS	HSPD1|ENSG00000144381	Stromal_score	1.113552e−06	−0.4082675	2.546583e−02	NEG
33	FLNA|ENSG00000196924	Stromal_score	0.000000e+00	0.5624427	0.000000e+00	POS	ADGRF5|ENSG00000069122	Stromal_score	0.000000e+00	0.5844503	0.000000e+00	POS
34	SFRP1|ENSG00000104332	Stromal_score	1.958132e−07	0.4340016	4.478053e−03	POS	GRTP1|ENSG00000139835	Stromal_score	1.557978e−06	−0.4030534	3.562939e−02	NEG
35	COL1A1|ENSG00000108821	Stromal_score	0.000000e+00	0.5808604	0.000000e+00	POS	TRPS1|ENSG00000104447	Stromal_score	3.924820e−07	0.4239489	8.975671e−03	POS
36	FNDC1|ENSG00000164694	Stromal_score	0.000000e+00	0.6621744	0.000000e+00	POS	VSTM4|ENSG00000165633	Stromal_score	0.000000e+00	0.6203297	0.000000e+00	POS
37	TNS1|ENSG00000079308	Stromal_score	1.710974e−10	0.5164228	3.912827e−06	POS	CLIC2|ENSG00000155962	Stromal_score	0.000000e+00	0.6138133	0.000000e+00	POS
38	THBS2|ENSG00000186340	Stromal_score	0.000000e+00	0.6286362	0.000000e+00	POS	NREP|ENSG00000134986	Stromal_score	3.676857e−10	0.5099502	8.408605e−06	POS
39	SERPINE1|ENSG00000106366	Stromal_score	3.920911e−07	0.4239635	8.966731e−03	POS	MAP7|ENSG00000135525	Stromal_score	3.241071e−08	−0.4586138	7.412005e−04	NEG
40	HLA−DQA1|ENSG00000196735	Stromal_score	2.039209e−09	0.4924251	4.663468e−05	POS	SLC5A6|ENSG00000138074	Stromal_score	2.535088e−07	−0.4303044	5.797493e−03	NEG
41	HLA−DQB1|ENSG00000179344	Stromal_score	1.701928e−07	0.4359916	3.892140e−03	POS	PTPRM|ENSG00000173482	Stromal_score	4.517812e−11	0.5248756	1.033178e−06	POS
42	CCDC80|ENSG00000091986	Stromal_score	0.000000e+00	0.6890986	0.000000e+00	POS	HVCN1|ENSG00000122986	Stromal_score	2.953627e−09	0.4882255	6.754650e−05	POS
43	MRGPRF|ENSG00000172935	Stromal_score	1.490295e−10	0.5174763	3.408155e−06	POS	ELK3|ENSG00000111145	Stromal_score	0.000000e+00	0.5952444	0.000000e+00	POS
44	CCL11|ENSG00000172156	Stromal_score	3.426427e−08	0.4578822	7.835897e−04	POS	MPDZ|ENSG00000107186	Stromal_score	0.000000e+00	0.5494879	0.000000e+00	POS
45	HLA−DPA1|ENSG00000231389	Stromal_score	3.961489e−07	0.4238123	9.059529e−03	POS	ZBTB47|ENSG00000114853	Stromal_score	3.892355e−07	0.4240708	8.901426e−03	POS
46	DPYSL3|ENSG00000113657	Stromal_score	0.000000e+00	0.6036240	0.000000e+00	POS	TNFAIP8L2|ENSG00000163154	Stromal_score	3.601915e−09	0.4859282	8.237219e−05	POS
47	ASPN|ENSG00000106819	Stromal_score	0.000000e+00	0.6621061	0.000000e+00	POS	TMEM204|ENSG00000131634	Stromal_score	0.000000e+00	0.6483660	0.000000e+00	POS
48	PTGIS|ENSG00000124212	Stromal_score	0.000000e+00	0.5509999	0.000000e+00	POS	ELMO1|ENSG00000155849	Stromal_score	5.071117e−09	0.4818944	1.159714e−04	POS
49	COL3A1|ENSG00000168542	Stromal_score	0.000000e+00	0.6895035	0.000000e+00	POS	RIN3|ENSG00000100599	Stromal_score	1.256704e−06	0.4063994	2.873956e−02	POS
50	COL14A1|ENSG00000187955	Stromal_score	0.000000e+00	0.6351868	0.000000e+00	POS	PRKG1|ENSG00000185532	Stromal_score	1.363874e−11	0.5291191	3.119044e−07	POS
51	CD52|ENSG00000169442	Stromal_score	3.072482e−07	0.4275241	7.026459e−03	POS	OLFML1|ENSG00000183801	Stromal_score	0.000000e+00	0.7323090	0.000000e+00	POS
52	EFEMP1|ENSG00000115380	Stromal_score	0.000000e+00	0.6157985	0.000000e+00	POS	MYO1G|ENSG00000136286	Stromal_score	9.142398e−08	0.4446639	2.090775e−03	POS
53	HLA−DRB1|ENSG00000196126	Stromal_score	2.083395e−06	0.3984733	4.764516e−02	POS	CCR4|ENSG00000183813	Stromal_score	3.650211e−07	0.4250122	8.347667e−03	POS
54	IGFBP5|ENSG00000115461	Stromal_score	0.000000e+00	0.5798020	0.000000e+00	POS	CYTH4|ENSG00000100055	Stromal_score	2.891745e−10	0.5120915	6.613132e−06	POS
55	POSTN|ENSG00000133110	Stromal_score	0.000000e+00	0.5481465	0.000000e+00	POS	EMCN|ENSG00000164035	Stromal_score	0.000000e+00	0.5343235	0.000000e+00	POS
56	AOC3|ENSG00000131471	Stromal_score	0.000000e+00	0.5705834	0.000000e+00	POS	LZTS1|ENSG00000061337	Stromal_score	0.000000e+00	0.5717052	0.000000e+00	POS
57	ISLR|ENSG00000129009	Stromal_score	0.000000e+00	0.7055263	0.000000e+00	POS	FGD5|ENSG00000154783	Stromal_score	4.043717e−10	0.5090772	9.247577e−06	POS
58	DPT|ENSG00000143196	Stromal_score	4.510340e−10	0.5080578	1.031470e−05	POS	ANOS1|ENSG00000011201	Stromal_score	7.343000e−09	0.4774315	1.679271e−04	POS
59	HLA−DRA|ENSG00000204287	Stromal_score	4.303713e−07	0.4225929	9.842161e−03	POS	CSF2RA|ENSG00000198223	Stromal_score	7.821423e−09	0.4766608	1.788681e−04	POS
60	CALD1|ENSG00000122786	Stromal_score	0.000000e+00	0.6028729	0.000000e+00	POS	MACF1|ENSG00000127603	Stromal_score	1.518642e−06	0.4034533	3.472983e−02	POS
61	CRYAB|ENSG00000109846	Stromal_score	2.651817e−08	0.4612379	6.064441e−04	POS	PDGFB|ENSG00000100311	Stromal_score	2.115644e−06	0.3982294	4.838266e−02	POS
62	SOD3|ENSG00000109610	Stromal_score	3.088806e−11	0.5265535	7.063791e−07	POS	RNF144A|ENSG00000151692	Stromal_score	5.771536e−07	0.4182372	1.319892e−02	POS
63	CPXM2|ENSG00000121898	Stromal_score	4.019243e−09	0.4846454	9.191607e−05	POS	FZD1|ENSG00000157240	Stromal_score	4.563487e−08	0.4540825	1.043624e−03	POS
64	CHRDL1|ENSG00000101938	Stromal_score	4.482456e−08	0.4543215	1.025093e−03	POS	ITPRIP|ENSG00000148841	Stromal_score	9.227444e−10	0.5009706	2.110224e−05	POS
65	FABP4|ENSG00000170323	Stromal_score	4.254157e−07	0.4227636	9.728832e−03	POS	TIE1|ENSG00000066056	Stromal_score	0.000000e+00	0.6069701	0.000000e+00	POS
66	CYP1B1|ENSG00000138061	Stromal_score	0.000000e+00	0.5872110	0.000000e+00	POS	TRAV9−2|ENSG00000211793	Stromal_score	1.120272e−06	0.4081748	2.561949e−02	POS
67	HLA−DOA|ENSG00000204252	Stromal_score	4.656106e−08	0.4538143	1.064805e−03	POS	ABCA1|ENSG00000165029	Stromal_score	0.000000e+00	0.5473222	0.000000e+00	POS
68	FAM129A|ENSG00000135842	Stromal_score	2.000155e−07	0.4336992	4.574154e−03	POS	IL16|ENSG00000172349	Stromal_score	1.097622e−07	0.4421374	2.510151e−03	POS
69	SULF1|ENSG00000137573	Stromal_score	0.000000e+00	0.6546630	0.000000e+00	POS	RFLNB|ENSG00000183688	Stromal_score	3.215897e−08	0.4587162	7.354435e−04	POS
70	AKAP12|ENSG00000131016	Stromal_score	8.301709e−10	0.5020583	1.898518e−05	POS	IDS|ENSG00000010404	Stromal_score	1.894075e−07	0.4344747	4.331561e−03	POS
71	CYR61|ENSG00000142871	Stromal_score	1.488450e−08	0.4686518	3.403937e−04	POS	GAL3ST4|ENSG00000197093	Stromal_score	0.000000e+00	0.5523120	0.000000e+00	POS
72	COL1A2|ENSG00000164692	Stromal_score	0.000000e+00	0.6546288	0.000000e+00	POS	EVA1B|ENSG00000142694	Stromal_score	1.541376e−10	0.5172227	3.524973e−06	POS
73	MMP2|ENSG00000087245	Stromal_score	0.000000e+00	0.5985123	0.000000e+00	POS	SYNDIG1|ENSG00000101463	Stromal_score	0.000000e+00	0.5816896	0.000000e+00	POS
74	CST2|ENSG00000170369	Stromal_score	6.003857e−07	0.4176471	1.373022e−02	POS	ZNF469|ENSG00000225614	Stromal_score	1.264642e−06	0.4063018	2.892109e−02	POS
75	PDLIM3|ENSG00000154553	Stromal_score	0.000000e+00	0.6517852	0.000000e+00	POS	ECSCR|ENSG00000249751	Stromal_score	0.000000e+00	0.5355624	0.000000e+00	POS
76	NEXN|ENSG00000162614	Stromal_score	3.782845e−11	0.5256999	8.650987e−07	POS	PLPP3|ENSG00000162407	Stromal_score	2.464762e−07	0.4307092	5.636665e−03	POS
77	RGMA|ENSG00000182175	Stromal_score	1.293614e−06	0.4059506	2.958366e−02	POS	GIMAP8|ENSG00000171115	Stromal_score	0.000000e+00	0.5653985	0.000000e+00	POS
78	HLA−DPB1|ENSG00000223865	Stromal_score	5.267215e−10	0.5065798	1.204559e−05	POS	ST6GALNAC5|ENSG00000117069	Stromal_score	0.000000e+00	0.5735440	0.000000e+00	POS
79	TRAC|ENSG00000277734	Stromal_score	8.924630e−08	0.4449956	2.040974e−03	POS	SH3RF3|ENSG00000172985	Stromal_score	0.000000e+00	0.5347771	0.000000e+00	POS
80	THBS1|ENSG00000137801	Stromal_score	0.000000e+00	0.6173642	0.000000e+00	POS	CD99L2|ENSG00000102181	Stromal_score	5.659542e−07	0.4185299	1.294281e−02	POS
81	C1QB|ENSG00000173369	Stromal_score	4.161658e−11	0.5252658	9.517297e−07	POS	TNFRSF4|ENSG00000186827	Stromal_score	3.392877e−07	0.4260804	7.759170e−03	POS
82	ELN|ENSG00000049540	Stromal_score	0.000000e+00	0.5482148	0.000000e+00	POS	GLI3|ENSG00000106571	Stromal_score	0.000000e+00	0.5790411	0.000000e+00	POS
83	CPE|ENSG00000109472	Stromal_score	6.480740e−09	0.4789484	1.482080e−04	POS	AHCY|ENSG00000101444	Stromal_score	4.909054e−07	−0.4206468	1.122652e−02	NEG
84	CD37|ENSG00000104894	Stromal_score	1.334558e−10	0.5182909	3.052001e−06	POS	MYO1F|ENSG00000142347	Stromal_score	1.180062e−09	0.4983904	2.698683e−05	POS
85	PODN|ENSG00000174348	Stromal_score	0.000000e+00	0.6300166	0.000000e+00	POS	GPRASP1|ENSG00000198932	Stromal_score	1.641618e−06	0.4022339	3.754216e−02	POS
86	GAS1|ENSG00000180447	Stromal_score	0.000000e+00	0.5687884	0.000000e+00	POS	HSPA7|ENSG00000225217	Stromal_score	1.422573e−07	0.4385182	3.253281e−03	POS
87	CILP|ENSG00000138615	Stromal_score	7.541406e−08	0.4473027	1.724644e−03	POS	ARHGAP31|ENSG00000031081	Stromal_score	0.000000e+00	0.6451029	0.000000e+00	POS
88	CST7|ENSG00000077984	Stromal_score	2.580994e−07	0.4300458	5.902474e−03	POS	HAS2|ENSG00000170961	Stromal_score	1.043022e−07	0.4428446	2.385287e−03	POS
89	IGFBP4|ENSG00000141753	Stromal_score	0.000000e+00	0.5413911	0.000000e+00	POS	ZNF667−AS1|ENSG00000166770	Stromal_score	1.416839e−06	0.4045361	3.240170e−02	POS
90	FGL2|ENSG00000127951	Stromal_score	0.000000e+00	0.5700322	0.000000e+00	POS	SHANK3|ENSG00000251322	Stromal_score	4.620532e−08	0.4539167	1.056670e−03	POS
91	NGFR|ENSG00000064300	Stromal_score	2.363160e−07	0.4313140	5.404312e−03	POS	PSAP|ENSG00000197746	Stromal_score	2.040246e−07	0.4334163	4.665837e−03	POS
92	AEBP1|ENSG00000106624	Stromal_score	0.000000e+00	0.6737099	0.000000e+00	POS	TRANK1|ENSG00000168016	Stromal_score	1.815144e−06	0.4006536	4.151054e−02	POS
93	DCN|ENSG00000011465	Stromal_score	0.000000e+00	0.6978148	0.000000e+00	POS	CD200|ENSG00000091972	Stromal_score	0.000000e+00	0.5827285	0.000000e+00	POS
94	FBLN2|ENSG00000163520	Stromal_score	0.000000e+00	0.6053995	0.000000e+00	POS	ITPR1|ENSG00000150995	Stromal_score	1.137418e−06	0.4079407	2.601162e−02	POS
95	BGN|ENSG00000182492	Stromal_score	0.000000e+00	0.6911131	0.000000e+00	POS	STAT5A|ENSG00000126561	Stromal_score	7.959687e−07	0.4133987	1.820301e−02	POS
96	CTHRC1|ENSG00000164932	Stromal_score	3.845434e−11	0.5256268	8.794123e−07	POS	HMCN1|ENSG00000143341	Stromal_score	0.000000e+00	0.6007121	0.000000e+00	POS
97	PTPRC|ENSG00000081237	Stromal_score	7.608984e−09	0.4769974	1.740099e−04	POS	NFAM1|ENSG00000235568	Stromal_score	2.115644e−06	0.3982294	4.838266e−02	POS
98	SELL|ENSG00000188404	Stromal_score	1.042646e−09	0.4996976	2.384427e−05	POS	HIC1|ENSG00000177374	Stromal_score	0.000000e+00	0.7124622	0.000000e+00	POS
99	F13A1|ENSG00000124491	Stromal_score	7.407798e−08	0.4475466	1.694089e−03	POS	ZNF423|ENSG00000102935	Stromal_score	5.276775e−11	0.5240952	1.206746e−06	POS
100	MSRB3|ENSG00000174099	Stromal_score	0.000000e+00	0.5840698	0.000000e+00	POS	FAM49A|ENSG00000197872	Stromal_score	0.000000e+00	0.6116086	0.000000e+00	POS
101	CYBRD1|ENSG00000071967	Stromal_score	0.000000e+00	0.5661057	0.000000e+00	POS	TRAF1|ENSG00000056558	Stromal_score	9.209635e−07	0.4111794	2.106151e−02	POS
102	CAV1|ENSG00000105974	Stromal_score	3.134186e−10	0.5113843	7.167570e−06	POS	PDGFC|ENSG00000145431	Stromal_score	4.351214e−11	0.5250561	9.950791e−07	POS
103	SPOCK1|ENSG00000152377	Stromal_score	0.000000e+00	0.5418886	0.000000e+00	POS	TEK|ENSG00000120156	Stromal_score	0.000000e+00	0.5471369	0.000000e+00	POS
104	FBXO32|ENSG00000156804	Stromal_score	1.421584e−07	0.4385279	3.251021e−03	POS	PLXNC1|ENSG00000136040	Stromal_score	0.000000e+00	0.6046142	0.000000e+00	POS
105	SPOCK2|ENSG00000107742	Stromal_score	1.684412e−07	0.4361379	3.852082e−03	POS	MILR1|ENSG00000271605	Stromal_score	0.000000e+00	0.6546093	0.000000e+00	POS
106	FMOD|ENSG00000122176	Stromal_score	1.694901e−07	0.4360501	3.876069e−03	POS	RAI14|ENSG00000039560	Stromal_score	8.065096e−09	0.4762852	1.844407e−04	POS
107	MRVI1|ENSG00000072952	Stromal_score	0.000000e+00	0.5581553	0.000000e+00	POS	SH2D3C|ENSG00000095370	Stromal_score	0.000000e+00	0.5970783	0.000000e+00	POS
108	MXRA5|ENSG00000101825	Stromal_score	1.651035e−07	0.4364208	3.775752e−03	POS	TNFAIP8L3|ENSG00000183578	Stromal_score	3.596538e−08	0.4572432	8.224924e−04	POS
109	FCGR3A|ENSG00000203747	Stromal_score	0.000000e+00	0.5337479	0.000000e+00	POS	FAT4|ENSG00000196159	Stromal_score	1.772530e−07	0.4354161	4.053598e−03	POS
110	LUM|ENSG00000139329	Stromal_score	0.000000e+00	0.6964735	0.000000e+00	POS	LAT2|ENSG00000086730	Stromal_score	2.951013e−08	0.4598429	6.748671e−04	POS
111	C11orf96|ENSG00000187479	Stromal_score	0.000000e+00	0.5692323	0.000000e+00	POS	PRDM8|ENSG00000152784	Stromal_score	1.091849e−07	0.4422105	2.496950e−03	POS
112	LCP1|ENSG00000136167	Stromal_score	2.121294e−07	0.4328602	4.851187e−03	POS	NOTCH2|ENSG00000134250	Stromal_score	4.058634e−07	0.4234562	9.281691e−03	POS
113	C1QC|ENSG00000159189	Stromal_score	0.000000e+00	0.5510536	0.000000e+00	POS	CNRIP1|ENSG00000119865	Stromal_score	0.000000e+00	0.6575017	0.000000e+00	POS
114	C1QA|ENSG00000173372	Stromal_score	0.000000e+00	0.5430397	0.000000e+00	POS	FCGR1A|ENSG00000150337	Stromal_score	2.721234e−08	0.4609014	6.223190e−04	POS
115	COL12A1|ENSG00000111799	Stromal_score	0.000000e+00	0.5501268	0.000000e+00	POS	TICRR|ENSG00000140534	Stromal_score	9.664302e−07	−0.4104429	2.210129e−02	NEG
116	C10orf10|ENSG00000165507	Stromal_score	1.936312e−10	0.5154473	4.428153e−06	POS	DNAJB4|ENSG00000162616	Stromal_score	1.562205e−07	0.4372012	3.572608e−03	POS
117	SRPX|ENSG00000101955	Stromal_score	0.000000e+00	0.5392303	0.000000e+00	POS	PJA2|ENSG00000198961	Stromal_score	3.114024e−07	0.4273290	7.121462e−03	POS
118	ABI3BP|ENSG00000154175	Stromal_score	1.099750e−09	0.4991367	2.515018e−05	POS	CYP7B1|ENSG00000172817	Stromal_score	0.000000e+00	0.5425666	0.000000e+00	POS
119	FRZB|ENSG00000162998	Stromal_score	0.000000e+00	0.5732660	0.000000e+00	POS	TTC28|ENSG00000100154	Stromal_score	4.453036e−08	0.4544093	1.018365e−03	POS
120	RGS1|ENSG00000090104	Stromal_score	0.000000e+00	0.5363477	0.000000e+00	POS	VGLL3|ENSG00000206538	Stromal_score	0.000000e+00	0.5476490	0.000000e+00	POS

	gene	feature	p.value	estimate	adj.p.value	COR.ID	gene	feature	p.value	estimate	adj.p.value	COR.ID
121	ANGPTL2|ENSG00000136859	Stromal_score	0.000000e+00	0.6415813	0.000000e+00	POS	GJA5|ENSG00000265107	Stromal_score	1.711761e−08	0.4668764	3.914627e−04	POS
122	MFAP5|ENSG00000197614	Stromal_score	0.000000e+00	0.6125793	0.000000e+00	POS	CLEC7A|ENSG00000172243	Stromal_score	0.000000e+00	0.5400985	0.000000e+00	POS
123	RSPO3|ENSG00000146374	Stromal_score	0.000000e+00	0.5967710	0.000000e+00	POS	PDCD1LG2|ENSG00000197646	Stromal_score	0.000000e+00	0.5885231	0.000000e+00	POS
124	MXRA8|ENSG00000162576	Stromal_score	0.000000e+00	0.5940152	0.000000e+00	POS	MAN1C1|ENSG00000117643	Stromal_score	2.892006e−07	0.4284021	6.613729e−03	POS
125	COL8A1|ENSG00000144810	Stromal_score	0.000000e+00	0.7109892	0.000000e+00	POS	PDE2A|ENSG00000186642	Stromal_score	1.856583e−06	0.4002975	4.245820e−02	POS
126	CAVIN2|ENSG00000168497	Stromal_score	1.441888e−06	0.4042630	3.297454e−02	POS	RBMS3|ENSG00000144642	Stromal_score	0.000000e+00	0.5961028	0.000000e+00	POS
127	CD53|ENSG00000143119	Stromal_score	0.000000e+00	0.5412545	0.000000e+00	POS	CHST1|ENSG00000175264	Stromal_score	2.461155e−09	0.4903083	5.628415e−05	POS
128	CXCL12|ENSG00000107562	Stromal_score	0.000000e+00	0.5404692	0.000000e+00	POS	BNC2|ENSG00000173068	Stromal_score	0.000000e+00	0.5989513	0.000000e+00	POS
129	GPNMB|ENSG00000136235	Stromal_score	0.000000e+00	0.6242220	0.000000e+00	POS	WWC3|ENSG00000047644	Stromal_score	9.807009e−07	0.4102185	2.242765e−02	POS
130	EVI2B|ENSG00000185862	Stromal_score	0.000000e+00	0.5374402	0.000000e+00	POS	CLEC2D|ENSG00000069493	Stromal_score	1.163241e−06	0.4075944	2.660216e−02	POS
131	RGS5|ENSG00000143248	Stromal_score	5.070608e−08	0.4526729	1.159597e−03	POS	NCF1B|ENSG00000182487	Stromal_score	1.555067e−06	0.4030826	3.556283e−02	POS
132	COL6A3|ENSG00000163359	Stromal_score	0.000000e+00	0.6750415	0.000000e+00	POS	NPR1|ENSG00000169418	Stromal_score	1.105867e−09	0.4990781	2.529007e−05	POS
133	A2M|ENSG00000175899	Stromal_score	0.000000e+00	0.6714711	0.000000e+00	POS	LILRB1|ENSG00000104972	Stromal_score	3.794950e−09	0.4853185	8.678671e−05	POS
134	NNMT|ENSG00000166741	Stromal_score	0.000000e+00	0.6558824	0.000000e+00	POS	ECM2|ENSG00000106823	Stromal_score	0.000000e+00	0.6898303	0.000000e+00	POS
135	SERPINF1|ENSG00000132386	Stromal_score	0.000000e+00	0.6792410	0.000000e+00	POS	EPB41L3|ENSG00000082397	Stromal_score	1.111744e−10	0.5195835	2.542448e−06	POS
136	ITM2A|ENSG00000078596	Stromal_score	7.548433e−09	0.4770949	1.726251e−04	POS	DOCK10|ENSG00000135905	Stromal_score	3.233902e−10	0.5111062	7.395611e−06	POS
137	CCL2|ENSG00000108691	Stromal_score	0.000000e+00	0.5471564	0.000000e+00	POS	ZNF532|ENSG00000074657	Stromal_score	1.504594e−08	0.4685153	3.440856e−04	POS
138	CXCR4|ENSG00000121966	Stromal_score	6.538455e−09	0.4788411	1.495279e−04	POS	LHFPL2|ENSG00000145685	Stromal_score	1.797469e−10	0.5160375	4.110632e−06	POS
139	CAVIN1|ENSG00000177469	Stromal_score	0.000000e+00	0.6104331	0.000000e+00	POS	NHSL2|ENSG00000204131	Stromal_score	6.953425e−09	0.4780948	1.590179e−04	POS
140	ADAMDEC1|ENSG00000134028	Stromal_score	1.112495e−06	0.4082821	2.544165e−02	POS	PCDH17|ENSG00000118946	Stromal_score	0.000000e+00	0.5353965	0.000000e+00	POS
141	CLMP|ENSG00000166250	Stromal_score	0.000000e+00	0.5998000	0.000000e+00	POS	DCLK2|ENSG00000170390	Stromal_score	7.369494e−09	0.4773876	1.685329e−04	POS
142	COL5A1|ENSG00000130635	Stromal_score	0.000000e+00	0.6437031	0.000000e+00	POS	TCF21|ENSG00000118526	Stromal_score	6.724932e−10	0.5041850	1.537925e−05	POS
143	COL15A1|ENSG00000204291	Stromal_score	0.000000e+00	0.5930104	0.000000e+00	POS	CRMP1|ENSG00000072832	Stromal_score	1.909922e−08	0.4654765	4.367801e−04	POS
144	MPEG1|ENSG00000197629	Stromal_score	0.000000e+00	0.5740269	0.000000e+00	POS	CCDC71L|ENSG00000253276	Stromal_score	3.790179e−07	0.4244610	8.667760e−03	POS
145	PALLD|ENSG00000129116	Stromal_score	4.692335e−11	0.5246903	1.073090e−06	POS	LITAF|ENSG00000189067	Stromal_score	2.248977e−08	0.4633743	5.143186e−04	POS
146	CTGF|ENSG00000118523	Stromal_score	0.000000e+00	0.5342503	0.000000e+00	POS	MAP7D1|ENSG00000116871	Stromal_score	8.368880e−08	0.4458785	1.913879e−03	POS
147	MAP1B|ENSG00000131711	Stromal_score	1.016952e−10	0.5201883	2.325668e−06	POS	PLEKHO2|ENSG00000241839	Stromal_score	0.000000e+00	0.6610721	0.000000e+00	POS
148	SSC5D|ENSG00000179954	Stromal_score	0.000000e+00	0.6341576	0.000000e+00	POS	ACVRL1|ENSG00000139567	Stromal_score	1.842734e−08	0.4659350	4.214147e−04	POS
149	KCTD12|ENSG00000178695	Stromal_score	6.357630e−08	0.4496244	1.453926e−03	POS	CD300A|ENSG00000167851	Stromal_score	1.614939e−07	0.4367330	3.693203e−03	POS
150	APLNR|ENSG00000134817	Stromal_score	1.024845e−09	0.4998781	2.343719e−05	POS	OGFRL1|ENSG00000119900	Stromal_score	4.852638e−10	0.5073651	1.109750e−05	POS
151	TGFBI|ENSG00000120708	Stromal_score	5.442724e−08	0.4517218	1.244697e−03	POS	MRAS|ENSG00000158186	Stromal_score	3.549547e−11	0.5259780	8.117460e−07	POS
152	SERPING1|ENSG00000149131	Stromal_score	0.000000e+00	0.6769730	0.000000e+00	POS	VAMP2|ENSG00000220205	Stromal_score	1.149694e−07	0.4414935	2.629235e−03	POS
153	EHD2|ENSG00000024422	Stromal_score	4.390212e−10	0.5083114	1.003998e−05	POS	FAM78A|ENSG00000126882	Stromal_score	5.968740e−07	0.4177349	1.364991e−02	POS
154	TSPAN2|ENSG00000134198	Stromal_score	8.028562e−10	0.5023998	1.836052e−05	POS	IL3RA|ENSG00000185291	Stromal_score	2.912741e−08	0.4600137	6.661147e−04	POS
155	OLFML2B|ENSG00000162745	Stromal_score	0.000000e+00	0.6745342	0.000000e+00	POS	MAPK13|ENSG00000156711	Stromal_score	2.502656e−07	−0.4304897	5.723324e−03	NEG
156	C1S|ENSG00000182326	Stromal_score	0.000000e+00	0.7267779	0.000000e+00	POS	GRASP|ENSG00000161835	Stromal_score	1.224510e−08	0.4711101	2.800332e−04	POS
157	SRGN|ENSG00000122862	Stromal_score	0.000000e+00	0.5842991	0.000000e+00	POS	AC018755.4|ENSG00000273837	Stromal_score	6.946395e−07	0.4154570	1.588571e−02	POS
158	COL6A2|ENSG00000142173	Stromal_score	0.000000e+00	0.6898888	0.000000e+00	POS	PLCB2|ENSG00000137841	Stromal_score	2.779240e−07	0.4289777	6.355844e−03	POS
159	CD48|ENSG00000117091	Stromal_score	1.226418e−08	0.4710906	2.804695e−04	POS	FLI1|ENSG00000151702	Stromal_score	0.000000e+00	0.6005170	0.000000e+00	POS
160	LDOC1|ENSG00000182195	Stromal_score	3.080168e−08	0.4592820	7.044037e−04	POS	GPR132|ENSG00000183484	Stromal_score	6.015606e−07	0.4176178	1.375709e−02	POS
161	LAPTM5|ENSG00000162511	Stromal_score	0.000000e+00	0.6067311	0.000000e+00	POS	VEGFC|ENSG00000150630	Stromal_score	0.000000e+00	0.6457809	0.000000e+00	POS
162	CPXM1|ENSG00000088882	Stromal_score	0.000000e+00	0.5749537	0.000000e+00	POS	GALNT15|ENSG00000131386	Stromal_score	1.146448e−06	0.4078187	2.621812e−02	POS
163	HTRA3|ENSG00000170801	Stromal_score	0.000000e+00	0.6681982	0.000000e+00	POS	EBPL|ENSG00000123179	Stromal_score	9.596752e−07	−0.4105502	2.194681e−02	NEG
164	SASH3|ENSG00000122122	Stromal_score	4.405940e−07	0.4222466	1.007594e−02	POS	MARVELD2|ENSG00000152939	Stromal_score	4.605225e−07	−0.4215930	1.053169e−02	NEG
165	CYBB|ENSG00000165168	Stromal_score	0.000000e+00	0.5963711	0.000000e+00	POS	RUNX1|ENSG00000159216	Stromal_score	5.422186e−07	0.4191689	1.240000e−02	POS
166	PLEK|ENSG00000115956	Stromal_score	2.176065e−11	0.5278168	4.976442e−07	POS	AC004816.1|ENSG00000259153	Stromal_score	1.212741e−06	−0.4069505	2.773417e−02	NEG
167	HLA−DMB|ENSG00000242574	Stromal_score	2.269428e−07	0.4318944	5.189955e−03	POS	NFATC4|ENSG00000100968	Stromal_score	9.994557e−08	0.4434348	2.285655e−03	POS
168	ADAMTS2|ENSG00000087116	Stromal_score	0.000000e+00	0.6175641	0.000000e+00	POS	RTL5|ENSG00000242732	Stromal_score	9.210670e−08	0.4445615	2.106388e−03	POS
169	CCL4|ENSG00000275302	Stromal_score	7.826728e−08	0.4467954	1.789894e−03	POS	SLC9A9|ENSG00000181804	Stromal_score	0.000000e+00	0.5747829	0.000000e+00	POS
170	CPED1|ENSG00000106034	Stromal_score	3.250595e−07	0.4267047	7.433786e−03	POS	FZD4|ENSG00000174804	Stromal_score	1.139654e−07	0.4416155	2.606274e−03	POS
171	CCL4L2|ENSG00000276070	Stromal_score	1.095012e−06	0.4085260	2.504184e−02	POS	MEG3|ENSG00000214548	Stromal_score	2.652993e−09	0.4894547	6.067130e−05	POS
172	RCAN2|ENSG00000172348	Stromal_score	1.431021e−10	0.5177788	3.272601e−06	POS	GLI1|ENSG00000111087	Stromal_score	0.000000e+00	0.6048873	0.000000e+00	POS
173	ANTXR1|ENSG00000169604	Stromal_score	0.000000e+00	0.6832797	0.000000e+00	POS	SIPA1|ENSG00000213445	Stromal_score	7.494325e−09	0.4771827	1.713877e−04	POS
174	CLIP3|ENSG00000105270	Stromal_score	0.000000e+00	0.6243196	0.000000e+00	POS	NIN|ENSG00000100503	Stromal_score	1.019529e−06	0.4096235	2.331560e−02	POS
175	ITGB2|ENSG00000160255	Stromal_score	0.000000e+00	0.5559653	0.000000e+00	POS	FMO1|ENSG00000010932	Stromal_score	7.383989e−08	0.4475905	1.688645e−03	POS
176	FOLR2|ENSG00000165457	Stromal_score	2.488878e−11	0.5273632	5.691816e−07	POS	FAM180A|ENSG00000189320	Stromal_score	5.758707e−09	0.4803726	1.316959e−04	POS
177	GJA1|ENSG00000152661	Stromal_score	8.078296e−07	0.4131743	1.847425e−02	POS	PRR16|ENSG00000184838	Stromal_score	1.682361e−09	0.4945566	3.847392e−05	POS
178	LTBP1|ENSG00000049323	Stromal_score	0.000000e+00	0.5689055	0.000000e+00	POS	SNX20|ENSG00000167208	Stromal_score	1.023720e−07	0.4431031	2.341146e−03	POS
179	FNBP1|ENSG00000187239	Stromal_score	1.849710e−06	0.4003561	4.230101e−02	POS	ZEB2|ENSG00000169554	Stromal_score	0.000000e+00	0.7475271	0.000000e+00	POS
180	ADA2|ENSG00000093072	Stromal_score	1.416175e−08	0.4692810	3.238650e−04	POS	NIPSNAP1|ENSG00000184117	Stromal_score	1.595300e−06	−0.4026827	3.648291e−02	NEG
181	COL6A1|ENSG00000142156	Stromal_score	0.000000e+00	0.5465320	0.000000e+00	POS	ARHGEF39|ENSG00000137135	Stromal_score	2.098955e−07	−0.4330114	4.800100e−03	NEG
182	OMD|ENSG00000127083	Stromal_score	0.000000e+00	0.5896352	0.000000e+00	POS	CALHM2|ENSG00000138172	Stromal_score	0.000000e+00	0.6312116	0.000000e+00	POS
183	EMILIN1|ENSG00000138080	Stromal_score	0.000000e+00	0.6892352	0.000000e+00	POS	FAM167B|ENSG00000183615	Stromal_score	3.277929e−07	0.4265828	7.496296e−03	POS
184	DDR2|ENSG00000162733	Stromal_score	0.000000e+00	0.6724954	0.000000e+00	POS	AC117498.2|ENSG00000257660	Stromal_score	1.691839e−06	−0.4017608	3.869066e−02	NEG
185	FBN1|ENSG00000166147	Stromal_score	0.000000e+00	0.7144522	0.000000e+00	POS	BCL6B|ENSG00000161940	Stromal_score	0.000000e+00	0.5413033	0.000000e+00	POS
186	ITGA5|ENSG00000161638	Stromal_score	2.707976e−09	0.4892206	6.192870e−05	POS	LYL1|ENSG00000104903	Stromal_score	1.122187e−07	0.4418301	2.566329e−03	POS
187	GUCY1A3|ENSG00000164116	Stromal_score	0.000000e+00	0.6834309	0.000000e+00	POS	TRGV7|ENSG00000249978	Stromal_score	9.566540e−11	0.5205931	2.187772e−06	POS
188	HCST|ENSG00000126264	Stromal_score	0.000000e+00	0.5334260	0.000000e+00	POS	PPP1R16A|ENSG00000160972	Stromal_score	1.922221e−07	−0.4342649	4.395926e−03	NEG
189	FOXF1|ENSG00000103241	Stromal_score	1.752168e−08	0.4665789	4.007034e−04	POS	SPSB1|ENSG00000171621	Stromal_score	1.235039e−08	0.4710028	2.824410e−04	POS
190	INHBA|ENSG00000122641	Stromal_score	0.000000e+00	0.5400400	0.000000e+00	POS	FMNL3|ENSG00000161791	Stromal_score	0.000000e+00	0.6552678	0.000000e+00	POS
191	LIMS2|ENSG00000072163	Stromal_score	1.937438e−09	0.4929958	4.430728e−05	POS	QKI|ENSG00000112531	Stromal_score	0.000000e+00	0.5907472	0.000000e+00	POS
192	COL5A2|ENSG00000204262	Stromal_score	0.000000e+00	0.6308994	0.000000e+00	POS	ARSI|ENSG00000183876	Stromal_score	2.232051e−08	0.4634719	5.104477e−04	POS
193	TUBA1A|ENSG00000167552	Stromal_score	0.000000e+00	0.6390791	0.000000e+00	POS	FYN|ENSG00000010810	Stromal_score	2.303097e−08	0.4630670	5.266953e−04	POS
194	TNFAIP6|ENSG00000123610	Stromal_score	2.505990e−09	0.4901034	5.730949e−05	POS	TLR8|ENSG00000101916	Stromal_score	7.509132e−08	0.4473612	1.717263e−03	POS
195	IGFBP3|ENSG00000146674	Stromal_score	1.549851e−08	0.4681397	3.544355e−04	POS	HSPA12B|ENSG00000132622	Stromal_score	9.166541e−11	0.5208711	2.096296e−06	POS
196	BCL2A1|ENSG00000140379	Stromal_score	4.074819e−07	0.4233977	9.318705e−03	POS	ZNF521|ENSG00000198795	Stromal_score	0.000000e+00	0.6467710	0.000000e+00	POS
197	MEDAG|ENSG00000102802	Stromal_score	7.768400e−11	0.5219101	1.776555e−06	POS	PAMR1|ENSG00000149090	Stromal_score	5.681773e−07	0.4184714	1.299365e−02	POS
198	GEM|ENSG00000164949	Stromal_score	2.375745e−09	0.4907082	5.433091e−05	POS	PLPPR4|ENSG00000117600	Stromal_score	0.000000e+00	0.5773339	0.000000e+00	POS
199	SELENOM|ENSG00000198832	Stromal_score	1.186013e−08	0.4715101	2.712292e−04	POS	JAZF1|ENSG00000153814	Stromal_score	2.952113e−10	0.5119110	6.751186e−06	POS
200	C1R|ENSG00000159403	Stromal_score	0.000000e+00	0.7191347	0.000000e+00	POS	TOX2|ENSG00000124191	Stromal_score	1.167126e−09	0.4985075	2.669100e−05	POS
201	TMEM119|ENSG00000183160	Stromal_score	0.000000e+00	0.6542142	0.000000e+00	POS	SLC37A4|ENSG00000137700	Stromal_score	1.002484e−06	−0.4098820	2.292581e−02	NEG
202	FBLN5|ENSG00000140092	Stromal_score	0.000000e+00	0.5815238	0.000000e+00	POS	CHN1|ENSG00000128656	Stromal_score	0.000000e+00	0.5372939	0.000000e+00	POS
203	ZCCHC24|ENSG00000165424	Stromal_score	0.000000e+00	0.6167886	0.000000e+00	POS	SH3YL1|ENSG00000035115	Stromal_score	2.017188e−06	−0.3989855	4.613107e−02	NEG
204	TRIM22|ENSG00000132274	Stromal_score	0.000000e+00	0.5401473	0.000000e+00	POS	TMEM200B|ENSG00000253304	Stromal_score	7.344358e−10	0.5033021	1.679581e−05	POS
205	VSIG4|ENSG00000155659	Stromal_score	0.000000e+00	0.5609892	0.000000e+00	POS	ST6GALNAC6|ENSG00000160408	Stromal_score	1.164447e−09	0.4985319	2.662974e−05	POS
206	VCAN|ENSG00000038427	Stromal_score	0.000000e+00	0.6745586	0.000000e+00	POS	NTM|ENSG00000182667	Stromal_score	3.596538e−08	0.4572432	8.224924e−04	POS
207	TGFB1I1|ENSG00000140682	Stromal_score	0.000000e+00	0.5825822	0.000000e+00	POS	CXorf36|ENSG00000147113	Stromal_score	0.000000e+00	0.5454443	0.000000e+00	POS
208	CFH|ENSG00000000971	Stromal_score	0.000000e+00	0.6266267	0.000000e+00	POS	SH2B3|ENSG00000111252	Stromal_score	0.000000e+00	0.5460784	0.000000e+00	POS
209	HSPG2|ENSG00000142798	Stromal_score	1.574687e−07	0.4370891	3.601153e−03	POS	NLRP1|ENSG00000091592	Stromal_score	5.495675e−09	0.4809336	1.256806e−04	POS
210	FERMT2|ENSG00000073712	Stromal_score	0.000000e+00	0.5686031	0.000000e+00	POS	HIP1|ENSG00000127946	Stromal_score	1.043022e−07	0.4428446	2.385287e−03	POS
211	SPARC|ENSG00000113140	Stromal_score	0.000000e+00	0.6640035	0.000000e+00	POS	PLA2G4C|ENSG00000105499	Stromal_score	4.515362e−08	0.4542240	1.032618e−03	POS
212	FCER1G|ENSG00000158869	Stromal_score	0.000000e+00	0.5414789	0.000000e+00	POS	RUNX2|ENSG00000124813	Stromal_score	2.999037e−07	0.4278753	6.858498e−03	POS
213	TREM2|ENSG00000095970	Stromal_score	0.000000e+00	0.5504780	0.000000e+00	POS	ARHGAP24|ENSG00000138639	Stromal_score	3.225641e−07	0.4268169	7.376718e−03	POS
214	OLFML3|ENSG00000116774	Stromal_score	1.272899e−10	0.5186323	2.910993e−06	POS	DCLK1|ENSG00000133083	Stromal_score	1.389609e−09	0.4966394	3.177897e−05	POS
215	LTBP2|ENSG00000119681	Stromal_score	0.000000e+00	0.5469027	0.000000e+00	POS	AFAP1|ENSG00000196526	Stromal_score	1.390865e−09	0.4966296	3.180769e−05	POS
216	IL2RB|ENSG00000100385	Stromal_score	9.487187e−07	0.4107258	2.169625e−02	POS	INAFM1|ENSG00000257704	Stromal_score	3.670948e−07	0.4249293	8.395091e−03	POS
217	TIMP2|ENSG00000035862	Stromal_score	0.000000e+00	0.6559458	0.000000e+00	POS	MCC|ENSG00000171444	Stromal_score	0.000000e+00	0.5398205	0.000000e+00	POS
218	TSC22D3|ENSG00000157514	Stromal_score	4.022894e−08	0.4557604	9.199956e−04	POS	LRRC17|ENSG00000128606	Stromal_score	3.567409e−09	0.4860404	8.158307e−05	POS
219	DACT3|ENSG00000197380	Stromal_score	8.372285e−09	0.4758267	1.914658e−04	POS	PIK3R5|ENSG00000141506	Stromal_score	1.087887e−08	0.4725880	2.487889e−04	POS
220	BASP1|ENSG00000176788	Stromal_score	2.123577e−09	0.4919715	4.856409e−05	POS	MOB3A|ENSG00000172081	Stromal_score	1.312704e−08	0.4702371	3.002023e−04	POS
221	GZMK|ENSG00000113088	Stromal_score	6.265431e−07	0.4170081	1.432841e−02	POS	CSRP2|ENSG00000175183	Stromal_score	1.411526e−06	0.4045947	3.228019e−02	POS
222	IGFBP6|ENSG00000167779	Stromal_score	9.313595e−09	0.4745147	2.129926e−04	POS	PALMD|ENSG00000099260	Stromal_score	2.730029e−09	0.4891279	6.243304e−05	POS
223	IL7R|ENSG00000168685	Stromal_score	2.245920e−09	0.4913423	5.136195e−05	POS	CMAHP|ENSG00000168405	Stromal_score	0.000000e+00	0.6269144	0.000000e+00	POS
224	SELP|ENSG00000174175	Stromal_score	1.609078e−10	0.5168959	3.679801e−06	POS	SOX17|ENSG00000164736	Stromal_score	1.478055e−09	0.4959711	3.380164e−05	POS
225	ADAMTS1|ENSG00000154734	Stromal_score	0.000000e+00	0.5359916	0.000000e+00	POS	ADAP2|ENSG00000184060	Stromal_score	0.000000e+00	0.5692079	0.000000e+00	POS
226	TYROBP|ENSG00000011600	Stromal_score	0.000000e+00	0.6106819	0.000000e+00	POS	FLT1|ENSG00000102755	Stromal_score	9.926411e−07	0.4100332	2.270071e−02	POS
227	NR2F1|ENSG00000175745	Stromal_score	7.407798e−08	0.4475466	1.694089e−03	POS	SYNE1|ENSG00000131018	Stromal_score	0.000000e+00	0.5495415	0.000000e+00	POS
228	AC104083.1|ENSG00000260244	Stromal_score	0.000000e+00	0.6757194	0.000000e+00	POS	TLR1|ENSG00000174125	Stromal_score	0.000000e+00	0.5357868	0.000000e+00	POS
229	ICAM1|ENSG00000090339	Stromal_score	1.704570e−08	0.4669301	3.898180e−04	POS	CDO1|ENSG00000129596	Stromal_score	5.708871e−10	0.5057994	1.305562e−05	POS
230	CTSK|ENSG00000143387	Stromal_score	0.000000e+00	0.6779875	0.000000e+00	POS	C3orf80|ENSG00000180044	Stromal_score	1.836036e−06	0.4004731	4.198831e−02	POS
231	GPR183|ENSG00000169508	Stromal_score	0.000000e+00	0.5741537	0.000000e+00	POS	MYCT1|ENSG00000120279	Stromal_score	0.000000e+00	0.5427617	0.000000e+00	POS
232	HEYL|ENSG00000163909	Stromal_score	0.000000e+00	0.5888255	0.000000e+00	POS	C20orf24|ENSG00000101084	Stromal_score	2.147708e−06	−0.3979904	4.911594e−02	NEG
233	AGMAT|ENSG00000116771	Stromal_score	1.684412e−07	−0.4361379	3.852082e−03	NEG	CLEC4A|ENSG00000111729	Stromal_score	4.907655e−09	0.4822847	1.122332e−04	POS
234	MCAM|ENSG00000076706	Stromal_score	3.038708e−09	0.4878987	6.949221e−05	POS	ARHGEF15|ENSG00000198844	Stromal_score	0.000000e+00	0.5492586	0.000000e+00	POS
235	RNASE6|ENSG00000169413	Stromal_score	0.000000e+00	0.6109160	0.000000e+00	POS	MAP6|ENSG00000171533	Stromal_score	5.307971e−07	0.4194859	1.213880e−02	POS
236	SLIT3|ENSG00000184347	Stromal_score	1.394624e−10	0.5179690	3.189366e−06	POS	TCF4|ENSG00000196628	Stromal_score	0.000000e+00	0.6615062	0.000000e+00	POS
237	CD163|ENSG00000177575	Stromal_score	0.000000e+00	0.5489367	0.000000e+00	POS	IGF1|ENSG00000017427	Stromal_score	7.888306e−07	0.4135353	1.803977e−02	POS
238	NDN|ENSG00000182636	Stromal_score	0.000000e+00	0.6324944	0.000000e+00	POS	TNFAIP8|ENSG00000145779	Stromal_score	2.394676e−07	0.4311238	5.476385e−03	POS
239	TMEM47|ENSG00000147027	Stromal_score	0.000000e+00	0.6482148	0.000000e+00	POS	EOGT|ENSG00000163378	Stromal_score	6.325657e−08	0.4496927	1.446614e−03	POS
240	FSTL1|ENSG00000163430	Stromal_score	0.000000e+00	0.6947712	0.000000e+00	POS	RNF122|ENSG00000133874	Stromal_score	7.694527e−09	0.4768608	1.759661e−04	POS

	gene	feature	p.value	estimate	adj.p.value	COR.ID	gene	feature	p.value	estimate	adj.p.value	COR.ID
241	VWF|ENSG00000110799	Stromal_score	1.241783e−08	0.4709345	2.839834e−04	POS	TNFSF8|ENSG00000106952	Stromal_score	2.585334e−10	0.5130573	5.912400e−06	POS
242	PCOLCE|ENSG00000106333	Stromal_score	0.000000e+00	0.6293630	0.000000e+00	POS	LDLRAD4|ENSG00000168675	Stromal_score	1.970520e−06	0.3993562	4.506383e−02	POS
243	STC1|ENSG00000159167	Stromal_score	2.786750e−07	0.4289386	6.373020e−03	POS	RECK|ENSG00000122707	Stromal_score	0.000000e+00	0.6482880	0.000000e+00	POS
244	PDLIM7|ENSG00000196923	Stromal_score	2.663159e−10	0.5128036	6.090377e−06	POS	NUAK1|ENSG00000074590	Stromal_score	2.014583e−07	0.4335967	4.607149e−03	POS
245	MN1|ENSG00000169184	Stromal_score	1.272627e−06	0.4062043	2.910370e−02	POS	PTGER3|ENSG00000050628	Stromal_score	3.994938e−10	0.5091893	9.136024e−06	POS
246	ISM1|ENSG00000101230	Stromal_score	2.884617e−08	0.4601405	6.596831e−04	POS	FEZ1|ENSG00000149557	Stromal_score	9.414806e−07	0.4108428	2.153072e−02	POS
247	FILIP1L|ENSG00000168386	Stromal_score	1.322692e−09	0.4971710	3.024865e−05	POS	SWAP70|ENSG00000133789	Stromal_score	1.019694e−08	0.4733928	2.331938e−04	POS
248	PLA2G7|ENSG00000146070	Stromal_score	1.865043e−07	0.4346942	4.265167e−03	POS	NRROS|ENSG00000174004	Stromal_score	6.731519e−10	0.5041752	1.539431e−05	POS
249	WWTR1|ENSG00000018408	Stromal_score	2.040100e−09	0.4924203	4.665504e−05	POS	MYO5A|ENSG00000197535	Stromal_score	0.000000e+00	0.5616769	0.000000e+00	POS
250	CSF2RB|ENSG00000100368	Stromal_score	6.991612e−11	0.5225393	1.598912e−06	POS	BORA|ENSG00000136122	Stromal_score	1.706606e−06	−0.4016242	3.902836e−02	NEG
251	FPR3|ENSG00000187474	Stromal_score	0.000000e+00	0.6020730	0.000000e+00	POS	PEAR1|ENSG00000187800	Stromal_score	1.062297e−07	0.4425910	2.429367e−03	POS
252	HOPX|ENSG00000171476	Stromal_score	2.466499e−08	0.4621793	5.640636e−04	POS	TRAF3IP3|ENSG00000009790	Stromal_score	1.147173e−06	0.4078090	2.623471e−02	POS
253	FYB1|ENSG00000082074	Stromal_score	3.392935e−10	0.5106770	7.759304e−06	POS	PHLDB1|ENSG00000019144	Stromal_score	2.500677e−10	0.5133402	5.718797e−06	POS
254	FGF7|ENSG00000140285	Stromal_score	0.000000e+00	0.6260023	0.000000e+00	POS	REEP3|ENSG00000165476	Stromal_score	1.339102e−06	0.4054141	3.062392e−02	POS
255	GIMAP7|ENSG00000179144	Stromal_score	2.034957e−10	0.5150473	4.653744e−06	POS	RELB|ENSG00000104856	Stromal_score	6.582567e−07	0.4162667	1.505367e−02	POS
256	HTRA1|ENSG00000166033	Stromal_score	2.142135e−09	0.4918740	4.898848e−05	POS	ANKRD44|ENSG00000065413	Stromal_score	4.071976e−08	0.4555995	9.312201e−04	POS
257	PRRX1|ENSG00000116132	Stromal_score	0.000000e+00	0.6582772	0.000000e+00	POS	NECAB3|ENSG00000125967	Stromal_score	1.874673e−07	−0.4346210	4.287190e−03	NEG
258	KANK2|ENSG00000197256	Stromal_score	5.332430e−09	0.4812945	1.219473e−04	POS	PACS1|ENSG00000175115	Stromal_score	4.857367e−08	0.4532485	1.110831e−03	POS
259	ALOX5AP|ENSG00000132965	Stromal_score	0.000000e+00	0.5483270	0.000000e+00	POS	CD28|ENSG00000178562	Stromal_score	1.007245e−07	0.4433275	2.303469e−03	POS
260	IGFBP7|ENSG00000163453	Stromal_score	0.000000e+00	0.6668666	0.000000e+00	POS	SORCS2|ENSG00000184985	Stromal_score	3.318955e−08	0.4583016	7.590119e−04	POS
261	MRC2|ENSG00000011028	Stromal_score	0.000000e+00	0.6333528	0.000000e+00	POS	TRAV2|ENSG00000211776	Stromal_score	1.243505e−07	0.4404009	2.843771e−03	POS
262	PDGFRB|ENSG00000113721	Stromal_score	0.000000e+00	0.7191932	0.000000e+00	POS	LATS2|ENSG00000150457	Stromal_score	2.559242e−07	0.4301678	5.852730e−03	POS
263	DIO2|ENSG00000211448	Stromal_score	3.885892e−07	0.4240952	8.886647e−03	POS	TSPAN4|ENSG00000214063	Stromal_score	0.000000e+00	0.5912448	0.000000e+00	POS
264	CSF1R|ENSG00000182578	Stromal_score	0.000000e+00	0.6231587	0.000000e+00	POS	PLXNA4|ENSG00000221866	Stromal_score	5.299281e−07	0.4195103	1.211893e−02	POS
265	NAPSB|ENSG00000131401	Stromal_score	6.519827e−08	0.4492830	1.491019e−03	POS	FKBP7|ENSG00000079150	Stromal_score	1.043881e−10	0.5200127	2.387252e−06	POS
266	ADAM12|ENSG00000148848	Stromal_score	1.043127e−06	0.4092723	2.385526e−02	POS	REM1|ENSG00000088320	Stromal_score	0.000000e+00	0.6272364	0.000000e+00	POS
267	WAS|ENSG00000015285	Stromal_score	1.662071e−10	0.5166472	3.800990e−06	POS	C1orf54|ENSG00000118292	Stromal_score	0.000000e+00	0.6063799	0.000000e+00	POS
268	LHFPL6|ENSG00000183722	Stromal_score	0.000000e+00	0.7312945	0.000000e+00	POS	BMP6|ENSG00000153162	Stromal_score	3.974377e−08	0.4559214	9.089004e−04	POS
269	LOX|ENSG00000113083	Stromal_score	0.000000e+00	0.5459858	0.000000e+00	POS	CALU|ENSG00000128595	Stromal_score	1.216571e−06	0.4069018	2.782177e−02	POS
270	PRKCB|ENSG00000166501	Stromal_score	2.217473e−07	0.4322261	5.071139e−03	POS	SLC12A4|ENSG00000124067	Stromal_score	2.702200e−09	0.4892450	6.179660e−05	POS
271	RARRES2|ENSG00000106538	Stromal_score	0.000000e+00	0.6062872	0.000000e+00	POS	PIP4K2A|ENSG00000150867	Stromal_score	0.000000e+00	0.5497025	0.000000e+00	POS
272	SGCE|ENSG00000127990	Stromal_score	1.927258e−06	0.3997073	4.407447e−02	POS	CCDC58|ENSG00000160124	Stromal_score	6.910509e−07	−0.4155351	1.580364e−02	NEG
273	LGALS1|ENSG00000100097	Stromal_score	0.000000e+00	0.6308750	0.000000e+00	POS	ZFHX4|ENSG00000091656	Stromal_score	2.710289e−09	0.4892108	6.198161e−05	POS
274	CD4|ENSG00000010610	Stromal_score	0.000000e+00	0.5738562	0.000000e+00	POS	RN7SL138P|ENSG00000266274	Stromal_score	1.347534e−06	0.4053166	3.081676e−02	POS
275	KLF9|ENSG00000119138	Stromal_score	1.238406e−08	0.4709687	2.832112e−04	POS	ERG|ENSG00000157554	Stromal_score	0.000000e+00	0.6206029	0.000000e+00	POS
276	CYTIP|ENSG00000115165	Stromal_score	5.158008e−08	0.4524437	1.179585e−03	POS	RAB7B|ENSG00000276600	Stromal_score	2.097526e−06	0.3983660	4.796833e−02	POS
277	NRP2|ENSG00000118257	Stromal_score	0.000000e+00	0.5475905	0.000000e+00	POS	GIMAP1|ENSG00000213203	Stromal_score	0.000000e+00	0.6108087	0.000000e+00	POS
278	SLAMF8|ENSG00000158714	Stromal_score	0.000000e+00	0.5664325	0.000000e+00	POS	TTLL4|ENSG00000135912	Stromal_score	1.343734e−06	−0.4053605	3.072984e−02	NEG
279	CAVIN3|ENSG00000170955	Stromal_score	9.168529e−07	0.4112477	2.096751e−02	POS	VASH1|ENSG00000071246	Stromal_score	7.902562e−10	0.5025607	1.807237e−05	POS
280	DAAM2|ENSG00000146122	Stromal_score	5.381872e−08	0.4518730	1.230780e−03	POS	PANK1|ENSG00000152782	Stromal_score	1.350498e−06	−0.4052824	3.088453e−02	NEG
281	PDGFRA|ENSG00000134853	Stromal_score	1.163609e−06	0.4075895	2.661057e−02	POS	PNRC1|ENSG00000146278	Stromal_score	1.496525e−06	0.4036826	3.422403e−02	POS
282	THY1|ENSG00000154096	Stromal_score	0.000000e+00	0.6930104	0.000000e+00	POS	ST8SIA4|ENSG00000113532	Stromal_score	0.000000e+00	0.5837284	0.000000e+00	POS
283	BOC|ENSG00000144857	Stromal_score	0.000000e+00	0.5564579	0.000000e+00	POS	AC138207.5|ENSG00000265743	Stromal_score	5.807998e−09	0.4802702	1.328231e−04	POS
284	TIMP1|ENSG00000102265	Stromal_score	0.000000e+00	0.5628085	0.000000e+00	POS	FHL5|ENSG00000112214	Stromal_score	5.133691e−08	0.4525071	1.174024e−03	POS
285	OSMR|ENSG00000145623	Stromal_score	1.120626e−06	0.4081699	2.562760e−02	POS	PIP5K1C|ENSG00000186111	Stromal_score	9.289406e−07	0.4110477	2.124394e−02	POS
286	SCARF2|ENSG00000244486	Stromal_score	0.000000e+00	0.6503366	0.000000e+00	POS	MARCH2|ENSG00000099785	Stromal_score	1.289507e−07	0.4398937	2.948972e−03	POS
287	CRISPLD2|ENSG00000103196	Stromal_score	0.000000e+00	0.7102234	0.000000e+00	POS	TLR7|ENSG00000196664	Stromal_score	0.000000e+00	0.5373817	0.000000e+00	POS
288	CD248|ENSG00000174807	Stromal_score	0.000000e+00	0.5948054	0.000000e+00	POS	NUMBL|ENSG00000105245	Stromal_score	6.955261e−08	0.4484050	1.590599e−03	POS
289	SYNPO|ENSG00000171992	Stromal_score	3.001804e−10	0.5117647	6.864825e−06	POS	RTN1|ENSG00000139970	Stromal_score	5.136728e−07	0.4199737	1.174718e−02	POS
290	CHST15|ENSG00000182022	Stromal_score	3.098087e−10	0.5114867	7.085015e−06	POS	HGF|ENSG00000019991	Stromal_score	1.580959e−06	0.4028241	3.615495e−02	POS
291	AL136084.3|ENSG00000270412	Stromal_score	1.654938e−06	0.4021071	3.784678e−02	POS	LMCD1|ENSG00000071282	Stromal_score	6.062823e−07	0.4175007	1.386507e−02	POS
292	RASSF2|ENSG00000101265	Stromal_score	0.000000e+00	0.6295776	0.000000e+00	POS	CSDC2|ENSG00000172346	Stromal_score	0.000000e+00	0.5396498	0.000000e+00	POS
293	S1PR1|ENSG00000170989	Stromal_score	0.000000e+00	0.6167984	0.000000e+00	POS	TRBV11−2|ENSG00000241657	Stromal_score	2.183580e−06	0.3977271	4.993629e−02	POS
294	TUBB6|ENSG00000176014	Stromal_score	1.922320e−08	0.4653936	4.396153e−04	POS	SH3BP5|ENSG00000131370	Stromal_score	8.786654e−07	0.4118964	2.009420e−02	POS
295	MXRA7|ENSG00000182534	Stromal_score	1.070130e−08	0.4727929	2.447281e−04	POS	PTGFR|ENSG00000122420	Stromal_score	2.090449e−06	0.3984197	4.780649e−02	POS
296	AMOTL1|ENSG00000166025	Stromal_score	8.843346e−09	0.4751536	2.022385e−04	POS	HMBS|ENSG00000256269	Stromal_score	6.289928e−07	−0.4169496	1.438444e−02	NEG
297	PDPN|ENSG00000162493	Stromal_score	0.000000e+00	0.5428787	0.000000e+00	POS	KCNN3|ENSG00000143603	Stromal_score	3.579106e−07	0.4253000	8.185058e−03	POS
298	COL4A2|ENSG00000134871	Stromal_score	0.000000e+00	0.5712272	0.000000e+00	POS	SNX18|ENSG00000178996	Stromal_score	1.160632e−07	0.4413618	2.654249e−03	POS
299	CLDN5|ENSG00000184113	Stromal_score	2.533034e−09	0.4899815	5.792796e−05	POS	CERKL|ENSG00000188452	Stromal_score	9.635058e−09	0.4740952	2.203441e−04	POS
300	GYPC|ENSG00000136732	Stromal_score	0.000000e+00	0.6874939	0.000000e+00	POS	HCN3|ENSG00000143630	Stromal_score	1.877249e−07	−0.4346015	4.293081e−03	NEG
301	GSN|ENSG00000148180	Stromal_score	1.173132e−08	0.4716467	2.682835e−04	POS	GHR|ENSG00000112964	Stromal_score	1.094924e−07	0.4421715	2.503983e−03	POS
302	IL10RA|ENSG00000110324	Stromal_score	7.527482e−12	0.5302507	1.721460e−07	POS	TRAV8−4|ENSG00000211790	Stromal_score	6.068750e−07	0.4174861	1.387862e−02	POS
303	TIMP3|ENSG00000100234	Stromal_score	0.000000e+00	0.5638279	0.000000e+00	POS	PCDHGB7|ENSG00000254122	Stromal_score	2.052141e−07	0.4333333	4.693041e−03	POS
304	SLC2A3|ENSG00000059804	Stromal_score	2.962035e−08	0.4597942	6.773879e−04	POS	RHOQ|ENSG00000119729	Stromal_score	1.673523e−08	0.4671642	3.827179e−04	POS
305	FGFR1|ENSG00000077782	Stromal_score	4.232766e−10	0.5086528	9.679913e−06	POS	KATNAL1|ENSG00000102781	Stromal_score	2.988970e−07	0.4279241	6.835476e−03	POS
306	GBP2|ENSG00000162645	Stromal_score	1.888397e−06	0.4000293	4.318575e−02	POS	ZBTB20|ENSG00000181722	Stromal_score	1.431998e−06	0.4043703	3.274836e−02	POS
307	LSP1|ENSG00000130592	Stromal_score	0.000000e+00	0.5751732	0.000000e+00	POS	KANK3|ENSG00000186994	Stromal_score	5.006833e−07	0.4203541	1.145013e−02	POS
308	RERG|ENSG00000134533	Stromal_score	7.319899e−07	0.4146669	1.673988e−02	POS	HPGDS|ENSG00000163106	Stromal_score	1.830367e−06	0.4005219	4.185867e−02	POS
309	OLR1|ENSG00000173391	Stromal_score	7.757213e−08	0.4469174	1.773997e−03	POS	CSGALNACT2|ENSG00000169826	Stromal_score	0.000000e+00	0.6889816	0.000000e+00	POS
310	AXL|ENSG00000167601	Stromal_score	0.000000e+00	0.7138035	0.000000e+00	POS	THBS3|ENSG00000169231	Stromal_score	1.753112e−07	0.4355721	4.009191e−03	POS
311	LRRC32|ENSG00000137507	Stromal_score	0.000000e+00	0.7026388	0.000000e+00	POS	AVPR1A|ENSG00000166148	Stromal_score	2.324916e−07	0.4315481	5.316849e−03	POS
312	ID4|ENSG00000172201	Stromal_score	1.384595e−09	0.4966784	3.166431e−05	POS	DZIP1|ENSG00000134874	Stromal_score	9.140840e−10	0.5010682	2.090419e−05	POS
313	EPHA3|ENSG00000044524	Stromal_score	6.586036e−09	0.4787533	1.506160e−04	POS	STK10|ENSG00000072786	Stromal_score	3.652763e−08	0.4570383	8.353504e−04	POS
314	MRC1|ENSG00000260314	Stromal_score	1.429668e−09	0.4963321	3.269507e−05	POS	IL10|ENSG00000136634	Stromal_score	8.044606e−07	0.4132377	1.839721e−02	POS
315	LY86|ENSG00000112799	Stromal_score	0.000000e+00	0.6342308	0.000000e+00	POS	FGD2|ENSG00000146192	Stromal_score	3.110890e−07	0.4273437	7.114294e−03	POS
316	MYADM|ENSG00000179820	Stromal_score	4.308996e−09	0.4838260	9.854244e−05	POS	MARS2|ENSG00000247626	Stromal_score	3.332141e−07	−0.4263438	7.620274e−03	NEG
317	CLIC4|ENSG00000169504	Stromal_score	0.000000e+00	0.6149400	0.000000e+00	POS	PDE1A|ENSG00000115252	Stromal_score	0.000000e+00	0.6398156	0.000000e+00	POS
318	IFI16|ENSG00000163565	Stromal_score	0.000000e+00	0.5431812	0.000000e+00	POS	TMIGD3|ENSG00000121933	Stromal_score	6.210645e−07	0.4171398	1.420312e−02	POS
319	FAM20C|ENSG00000177706	Stromal_score	9.295342e−07	0.4110379	2.125752e−02	POS	S1PR2|ENSG00000267534	Stromal_score	1.682410e−06	0.4018486	3.847504e−02	POS
320	ZEB1|ENSG00000148516	Stromal_score	0.000000e+00	0.6653790	0.000000e+00	POS	ENOX1|ENSG00000120658	Stromal_score	1.095689e−09	0.4991757	2.505730e−05	POS
321	IFIT2|ENSG00000119922	Stromal_score	1.690815e−07	0.4360843	3.866724e−03	POS	ARID5A|ENSG00000196843	Stromal_score	0.000000e+00	0.5392450	0.000000e+00	POS
322	C5AR1|ENSG00000197405	Stromal_score	2.450664e−10	0.5135109	5.604424e−06	POS	AL583785.1|ENSG00000226197	Stromal_score	3.900123e−07	0.4240416	8.919191e−03	POS
323	RAMP2|ENSG00000131477	Stromal_score	3.219482e−08	0.4587016	7.362634e−04	POS	PRKCH|ENSG00000027075	Stromal_score	1.127321e−07	0.4417667	2.578070e−03	POS
324	PRKAR2B|ENSG00000005249	Stromal_score	4.219495e−10	0.5086821	9.649563e−06	POS	LDB2|ENSG00000169744	Stromal_score	0.000000e+00	0.5900985	0.000000e+00	POS
325	COL4A1|ENSG00000187498	Stromal_score	6.139391e−11	0.5232807	1.404017e−06	POS	SELENOI|ENSG00000138018	Stromal_score	4.734945e−08	−0.4535899	1.082834e−03	NEG
326	CELF2|ENSG00000048740	Stromal_score	9.741965e−09	0.4739586	2.227890e−04	POS	SUSD6|ENSG00000100647	Stromal_score	1.991507e−08	0.4649400	4.554377e−04	POS
327	PECAM1|ENSG00000261371	Stromal_score	0.000000e+00	0.6433714	0.000000e+00	POS	NOTCH4|ENSG00000204301	Stromal_score	2.879315e−09	0.4885182	6.584707e−05	POS
328	SPI1|ENSG00000066336	Stromal_score	0.000000e+00	0.6022583	0.000000e+00	POS	TESPA1|ENSG00000135426	Stromal_score	1.139219e−06	0.4079163	2.605280e−02	POS
329	FXYD6|ENSG00000137726	Stromal_score	1.027713e−09	0.4998488	2.350278e−05	POS	CORO2B|ENSG00000103647	Stromal_score	2.440633e−07	0.4308506	5.581484e−03	POS
330	EGR2|ENSG00000122877	Stromal_score	3.912752e−11	0.5255487	8.948073e−07	POS	POLD2|ENSG00000106628	Stromal_score	9.023687e−09	−0.4749049	2.063627e−04	NEG
331	ARHGDIB|ENSG00000111348	Stromal_score	2.242536e−10	0.5142523	5.128455e−06	POS	GSS|ENSG00000100983	Stromal_score	2.202410e−07	−0.4323237	5.036690e−03	NEG
332	FMO2|ENSG00000094963	Stromal_score	1.263699e−08	0.4707151	2.889954e−04	POS	TRAV8−6|ENSG00000211795	Stromal_score	3.648995e−07	0.4250171	8.344886e−03	POS
333	MIR100HG|ENSG00000255248	Stromal_score	0.000000e+00	0.5648278	0.000000e+00	POS	DSE|ENSG00000111817	Stromal_score	0.000000e+00	0.5605112	0.000000e+00	POS
334	IL4I1|ENSG00000104951	Stromal_score	1.246456e−06	0.4065262	2.850520e−02	POS	BMPR2|ENSG00000204217	Stromal_score	6.346193e−08	0.4496488	1.451311e−03	POS
335	RCN3|ENSG00000142552	Stromal_score	1.157008e−06	0.4076773	2.645961e−02	POS	RUNX1T1|ENSG00000079102	Stromal_score	0.000000e+00	0.5700468	0.000000e+00	POS
336	C14orf132|ENSG00000227051	Stromal_score	4.338012e−07	0.4224759	9.920601e−03	POS	AL161785.1|ENSG00000224307	Stromal_score	7.782378e−07	0.4137401	1.779752e−02	POS
337	DKK3|ENSG00000050165	Stromal_score	0.000000e+00	0.5412594	0.000000e+00	POS	PEA15|ENSG00000162734	Stromal_score	7.152457e−08	0.4480246	1.635695e−03	POS
338	ROR2|ENSG00000169071	Stromal_score	7.530356e−09	0.4771242	1.722117e−04	POS	TRAV8−2|ENSG00000211786	Stromal_score	1.307104e−06	0.4057897	2.989215e−02	POS
339	CD14|ENSG00000170458	Stromal_score	0.000000e+00	0.6074529	0.000000e+00	POS	TMEM140|ENSG00000146859	Stromal_score	2.087881e−06	0.3984392	4.774776e−02	POS
340	AIF1|ENSG00000204472	Stromal_score	0.000000e+00	0.5933470	0.000000e+00	POS	AFAP1L1|ENSG00000157510	Stromal_score	3.691797e−07	0.4248464	8.442771e−03	POS
341	C3AR1|ENSG00000171860	Stromal_score	0.000000e+00	0.6664325	0.000000e+00	POS	ATP10A|ENSG00000206190	Stromal_score	6.649583e−10	0.5042971	1.520693e−05	POS
342	SELPLG|ENSG00000110876	Stromal_score	0.000000e+00	0.5430738	0.000000e+00	POS	THOC3|ENSG00000051596	Stromal_score	2.468108e−07	−0.4306897	5.644317e−03	NEG
343	IKZF1|ENSG00000185811	Stromal_score	2.985956e−07	0.4279387	6.828583e−03	POS	AP001189.3|ENSG00000254810	Stromal_score	0.000000e+00	0.6259584	0.000000e+00	POS
344	PLXDC2|ENSG00000120594	Stromal_score	0.000000e+00	0.6831968	0.000000e+00	POS	PARVG|ENSG00000138964	Stromal_score	6.607791e−11	0.5228661	1.511136e−06	POS
345	SIGLEC10|ENSG00000142512	Stromal_score	7.884038e−09	0.4765633	1.803001e−04	POS	RAB8B|ENSG00000166128	Stromal_score	0.000000e+00	0.5465028	0.000000e+00	POS
346	GIMAP4|ENSG00000133574	Stromal_score	0.000000e+00	0.6369818	0.000000e+00	POS	CPNE8|ENSG00000139117	Stromal_score	2.005423e−06	0.3990781	4.586202e−02	POS
347	PMP22|ENSG00000109099	Stromal_score	0.000000e+00	0.6407570	0.000000e+00	POS	CETP|ENSG00000087237	Stromal_score	5.339368e−07	0.4193981	1.221060e−02	POS
348	MAFB|ENSG00000204103	Stromal_score	0.000000e+00	0.6269827	0.000000e+00	POS	ADAMTS10|ENSG00000142303	Stromal_score	0.000000e+00	0.6493123	0.000000e+00	POS
349	ANXA6|ENSG00000197043	Stromal_score	0.000000e+00	0.5875768	0.000000e+00	POS	JAML|ENSG00000160593	Stromal_score	1.951180e−06	0.3995122	4.462154e−02	POS
350	LY96|ENSG00000154589	Stromal_score	0.000000e+00	0.6707882	0.000000e+00	POS	MEIS3|ENSG00000105419	Stromal_score	0.000000e+00	0.6140133	0.000000e+00	POS
351	ADAM33|ENSG00000149451	Stromal_score	4.225772e−08	0.4551068	9.663918e−04	POS	GNAI2|ENSG00000114353	Stromal_score	0.000000e+00	0.5710711	0.000000e+00	POS
352	ADGRA2|ENSG00000020181	Stromal_score	0.000000e+00	0.6745439	0.000000e+00	POS	SEH1L|ENSG00000085415	Stromal_score	1.886066e−06	−0.4000488	4.313244e−02	NEG
353	C16orf45|ENSG00000166780	Stromal_score	5.879102e−09	0.4801239	1.344492e−04	POS	AC136628.3|ENSG00000253520	Stromal_score	0.000000e+00	0.5346405	0.000000e+00	POS
354	ITGA11|ENSG00000137809	Stromal_score	0.000000e+00	0.5891913	0.000000e+00	POS	ENTPD1|ENSG00000138185	Stromal_score	0.000000e+00	0.7042581	0.000000e+00	POS
355	INMT|ENSG00000241644	Stromal_score	0.000000e+00	0.5912935	0.000000e+00	POS	PLXDC1|ENSG00000161381	Stromal_score	0.000000e+00	0.6624671	0.000000e+00	POS
356	HCLS1|ENSG00000180353	Stromal_score	1.801934e−10	0.5160179	4.120844e−06	POS	SIGLEC7|ENSG00000168995	Stromal_score	2.070112e−08	0.4644425	4.734140e−04	POS
357	MAP1A|ENSG00000166963	Stromal_score	0.000000e+00	0.5690957	0.000000e+00	POS	OSTM1|ENSG00000081087	Stromal_score	0.000000e+00	0.5792996	0.000000e+00	POS
358	TNFSF13B|ENSG00000102524	Stromal_score	0.000000e+00	0.5353136	0.000000e+00	POS	P2RX7|ENSG00000089041	Stromal_score	0.000000e+00	0.5744610	0.000000e+00	POS
359	GGT5|ENSG00000099998	Stromal_score	0.000000e+00	0.7346259	0.000000e+00	POS	KIF21A|ENSG00000139116	Stromal_score	1.197911e−06	−0.4071408	2.739502e−02	NEG
360	NID1|ENSG00000116962	Stromal_score	6.957646e−07	0.4154326	1.591144e−02	POS	MAP3K3|ENSG00000198909	Stromal_score	0.000000e+00	0.5865672	0.000000e+00	POS

	gene	feature	p.value	estimate	adj.p.value	COR.ID	gene	feature	p.value	estimate	adj.p.value	COR.ID
361	SPON2|ENSG00000159674	Stromal_score	4.774569e−09	0.4826115	1.091896e−04	POS	PLEKHJ1|ENSG00000104886	Stromal_score	2.135876e−06	−0.3980782	4.884535e−02	NEG
362	THBD|ENSG00000178726	Stromal_score	3.749721e−08	0.4566920	8.575237e−04	POS	ARHGAP15|ENSG00000075884	Stromal_score	0.000000e+00	0.5766364	0.000000e+00	POS
363	CD109|ENSG00000156535	Stromal_score	4.225772e−08	0.4551068	9.663918e−04	POS	PLPP7|ENSG00000160539	Stromal_score	1.371562e−07	0.4390303	3.136624e−03	POS
364	CH25H|ENSG00000138135	Stromal_score	4.635746e−08	0.4538728	1.060149e−03	POS	FCHSD2|ENSG00000137478	Stromal_score	1.752838e−08	0.4665740	4.008566e−04	POS
365	SERPINB9|ENSG00000170542	Stromal_score	1.691314e−06	0.4017657	3.867865e−02	POS	CCR2|ENSG00000121807	Stromal_score	5.265936e−08	0.4521656	1.204267e−03	POS
366	STOM|ENSG00000148175	Stromal_score	1.412373e−09	0.4964638	3.229956e−05	POS	DLG4|ENSG00000132535	Stromal_score	1.969306e−06	0.3993659	4.503607e−02	POS
367	CD93|ENSG00000125810	Stromal_score	0.000000e+00	0.5784655	0.000000e+00	POS	C10orf128|ENSG00000204161	Stromal_score	0.000000e+00	0.6329626	0.000000e+00	POS
368	RAB31|ENSG00000168461	Stromal_score	0.000000e+00	0.6867671	0.000000e+00	POS	SLAMF1|ENSG00000117090	Stromal_score	8.073104e−07	0.4131841	1.846238e−02	POS
369	SSPN|ENSG00000123096	Stromal_score	3.221793e−12	0.5311531	7.367918e−08	POS	TFEC|ENSG00000105967	Stromal_score	6.898759e−11	0.5226173	1.577677e−06	POS
370	S1PR3|ENSG00000213694	Stromal_score	0.000000e+00	0.5971271	0.000000e+00	POS	GGTA1P|ENSG00000204136	Stromal_score	0.000000e+00	0.5321042	0.000000e+00	POS
371	LAMP5|ENSG00000125869	Stromal_score	1.452298e−06	0.4041508	3.321260e−02	POS	CHST7|ENSG00000147119	Stromal_score	1.070976e−06	0.4088674	2.449214e−02	POS
372	ARHGEF25|ENSG00000240771	Stromal_score	2.012657e−09	0.4925715	4.602746e−05	POS	CD1D|ENSG00000158473	Stromal_score	2.641949e−07	0.4297093	6.041872e−03	POS
373	GUCY1B3|ENSG00000061918	Stromal_score	0.000000e+00	0.7243440	0.000000e+00	POS	RCBTB2|ENSG00000136161	Stromal_score	4.843225e−08	0.4532875	1.107597e−03	POS
374	ENPP2|ENSG00000136960	Stromal_score	3.454449e−08	0.4577749	7.899979e−04	POS	MYO9B|ENSG00000099331	Stromal_score	4.825602e−08	0.4533363	1.103567e−03	POS
375	FCGR2A|ENSG00000143226	Stromal_score	0.000000e+00	0.5580236	0.000000e+00	POS	ZNF25|ENSG00000175395	Stromal_score	4.865081e−10	0.5073407	1.112595e−05	POS
376	PDE3A|ENSG00000172572	Stromal_score	1.037880e−08	0.4731733	2.373527e−04	POS	TRAV6|ENSG00000211780	Stromal_score	2.270200e−07	0.4318896	5.191722e−03	POS
377	SRPX2|ENSG00000102359	Stromal_score	3.361724e−10	0.5107599	7.687926e−06	POS	TNFSF4|ENSG00000117586	Stromal_score	1.380180e−07	0.4389425	3.156334e−03	POS
378	FIBIN|ENSG00000176971	Stromal_score	0.000000e+00	0.6098234	0.000000e+00	POS	LGI4|ENSG00000153902	Stromal_score	3.124493e−07	0.4272803	7.145404e−03	POS
379	MS4A7|ENSG00000166927	Stromal_score	0.000000e+00	0.6590967	0.000000e+00	POS	ARSB|ENSG00000113273	Stromal_score	0.000000e+00	0.5574968	0.000000e+00	POS
380	DHRS11|ENSG00000278535	Stromal_score	1.049107e−06	−0.4091845	2.399203e−02	NEG	TRBV5−4|ENSG00000230099	Stromal_score	1.024077e−06	0.4095552	2.341962e−02	POS
381	RNF150|ENSG00000170153	Stromal_score	1.187123e−09	0.4983270	2.714830e−05	POS	ZNF57|ENSG00000171970	Stromal_score	1.543901e−08	−0.4681885	3.530748e−04	NEG
382	IL1R1|ENSG00000115594	Stromal_score	0.000000e+00	0.6494196	0.000000e+00	POS	HACD4|ENSG00000188921	Stromal_score	3.374547e−10	0.5107258	7.717251e−06	POS
383	CERCAM|ENSG00000167123	Stromal_score	1.763145e−10	0.5161887	4.032136e−06	POS	PCDH12|ENSG00000113555	Stromal_score	1.315405e−07	0.4396156	3.008199e−03	POS
384	C1orf162|ENSG00000143110	Stromal_score	0.000000e+00	0.6074042	0.000000e+00	POS	SNX29|ENSG00000048471	Stromal_score	4.510412e−08	0.4542386	1.031486e−03	POS
385	LOXL1|ENSG00000129038	Stromal_score	4.513266e−11	0.5248805	1.032139e−06	POS	HAS2−AS1|ENSG00000248690	Stromal_score	2.887141e−07	0.4284265	6.602602e−03	POS
386	MOXD1|ENSG00000079931	Stromal_score	0.000000e+00	0.6160960	0.000000e+00	POS	ZFPM2|ENSG00000169946	Stromal_score	2.192473e−11	0.5277924	5.013967e−07	POS
387	EMP3|ENSG00000142227	Stromal_score	0.000000e+00	0.6539850	0.000000e+00	POS	TMEM255A|ENSG00000125355	Stromal_score	1.258126e−09	0.4977075	2.877208e−05	POS
388	FAS|ENSG00000026103	Stromal_score	5.563182e−09	0.4807872	1.272244e−04	POS	CHSY1|ENSG00000131873	Stromal_score	9.747823e−07	0.4103112	2.229230e−02	POS
389	WIPF1|ENSG00000115935	Stromal_score	0.000000e+00	0.6821920	0.000000e+00	POS	AC015911.3|ENSG00000267074	Stromal_score	8.315457e−08	0.4459662	1.901662e−03	POS
390	SLC24A3|ENSG00000185052	Stromal_score	1.252399e−09	0.4977563	2.864112e−05	POS	AC090559.1|ENSG00000255197	Stromal_score	6.209637e−10	0.5049751	1.420082e−05	POS
391	COL16A1|ENSG00000084636	Stromal_score	2.274302e−09	0.4912009	5.201100e−05	POS	SCN1B|ENSG00000105711	Stromal_score	2.929242e−07	0.4282168	6.698884e−03	POS
392	SIGLEC1|ENSG00000088827	Stromal_score	9.012287e−10	0.5012145	2.061020e−05	POS	TRAIP|ENSG00000183763	Stromal_score	3.284664e−08	−0.4584382	7.511697e−04	NEG
393	MSN|ENSG00000147065	Stromal_score	0.000000e+00	0.5827188	0.000000e+00	POS	KIAA1755|ENSG00000149633	Stromal_score	0.000000e+00	0.5743098	0.000000e+00	POS
394	PRSS23|ENSG00000150687	Stromal_score	1.224090e−07	0.4406204	2.799372e−03	POS	STARD8|ENSG00000130052	Stromal_score	1.410714e−08	0.4693298	3.226161e−04	POS
395	GLIPR1|ENSG00000139278	Stromal_score	0.000000e+00	0.5782314	0.000000e+00	POS	AC037198.2|ENSG00000276107	Stromal_score	4.558371e−07	0.4217442	1.042454e−02	POS
396	CDH11|ENSG00000140937	Stromal_score	0.000000e+00	0.7544825	0.000000e+00	POS	BTBD19|ENSG00000222009	Stromal_score	4.275328e−07	0.4226905	9.777248e−03	POS
397	POU2F2|ENSG00000028277	Stromal_score	1.258684e−06	0.4063750	2.878484e−02	POS	WBP1L|ENSG00000166272	Stromal_score	1.789073e−07	0.4352844	4.091432e−03	POS
398	MARVELD1|ENSG00000155254	Stromal_score	4.919741e−09	0.4822554	1.125096e−04	POS	FCGR2B|ENSG00000072694	Stromal_score	0.000000e+00	0.5601551	0.000000e+00	POS
399	TAGAP|ENSG00000164691	Stromal_score	5.297737e−09	0.4813725	1.211539e−04	POS	NXPE3|ENSG00000144815	Stromal_score	6.106415e−07	0.4173934	1.396476e−02	POS
400	COL18A1|ENSG00000182871	Stromal_score	1.789920e−09	0.4938738	4.093368e−05	POS	KCTD10|ENSG00000110906	Stromal_score	3.232133e−07	0.4267876	7.391565e−03	POS
401	GXYLT2|ENSG00000172986	Stromal_score	4.187706e−09	0.4841625	9.576865e−05	POS	P3H1|ENSG00000117385	Stromal_score	2.717095e−07	0.4293045	6.213724e−03	POS
402	VIM|ENSG00000026025	Stromal_score	0.000000e+00	0.7491025	0.000000e+00	POS	CXorf21|ENSG00000120280	Stromal_score	3.592883e−09	0.4859575	8.216565e−05	POS
403	SAMSN1|ENSG00000155307	Stromal_score	0.000000e+00	0.5707785	0.000000e+00	POS	PPM1M|ENSG00000164088	Stromal_score	7.833908e−09	0.4766413	1.791537e−04	POS
404	NOTCH3|ENSG00000074181	Stromal_score	1.336565e−09	0.4970588	3.056590e−05	POS	SLC8A1|ENSG00000183023	Stromal_score	9.910621e−07	0.4100576	2.266460e−02	POS
405	CLEC11A|ENSG00000105472	Stromal_score	0.000000e+00	0.6391328	0.000000e+00	POS	ITGB2−AS1|ENSG00000227039	Stromal_score	9.682803e−07	0.4104136	2.214360e−02	POS
406	ADAMTS12|ENSG00000151388	Stromal_score	1.053723e−10	0.5199493	2.409760e−06	POS	CREBL2|ENSG00000111269	Stromal_score	1.007922e−06	0.4097990	2.305016e−02	POS
407	VCAM1|ENSG00000162692	Stromal_score	0.000000e+00	0.6775924	0.000000e+00	POS	P4HA3|ENSG00000149380	Stromal_score	1.626710e−08	0.4675251	3.720122e−04	POS
408	RASAL3|ENSG00000105122	Stromal_score	8.812009e−07	0.4118525	2.015218e−02	POS	CEP170|ENSG00000143702	Stromal_score	2.046692e−08	0.4645888	4.680580e−04	POS
409	MMP14|ENSG00000157227	Stromal_score	4.006659e−08	0.4558141	9.162829e−04	POS	PSTPIP1|ENSG00000140368	Stromal_score	3.041432e−08	0.4594479	6.955452e−04	POS
410	FAM198B|ENSG00000164125	Stromal_score	0.000000e+00	0.5702322	0.000000e+00	POS	PRKD1|ENSG00000184304	Stromal_score	0.000000e+00	0.5970686	0.000000e+00	POS
411	JAM3|ENSG00000166086	Stromal_score	0.000000e+00	0.5934933	0.000000e+00	POS	ADPRH|ENSG00000144843	Stromal_score	7.503820e−11	0.5221198	1.716049e−06	POS
412	HAPLN3|ENSG00000140511	Stromal_score	8.049781e−07	0.4132280	1.840904e−02	POS	RIC1|ENSG00000107036	Stromal_score	2.011607e−06	0.3990294	4.600344e−02	POS
413	CD69|ENSG00000110848	Stromal_score	7.123329e−10	0.5036094	1.629034e−05	POS	UQCC1|ENSG00000101019	Stromal_score	1.596623e−07	−0.4368940	3.651316e−03	NEG
414	NCF4|ENSG00000100365	Stromal_score	5.434835e−08	0.4517413	1.242892e−03	POS	GPR65|ENSG00000140030	Stromal_score	7.577080e−11	0.5220613	1.732802e−06	POS
415	ALDH1A3|ENSG00000184254	Stromal_score	7.796509e−09	0.4766998	1.782984e−04	POS	TRMT61A|ENSG00000166166	Stromal_score	1.590836e−06	−0.4027266	3.638083e−02	NEG
416	SYT11|ENSG00000132718	Stromal_score	0.000000e+00	0.5841479	0.000000e+00	POS	HS3ST3A1|ENSG00000153976	Stromal_score	5.674353e−07	0.4184909	1.297668e−02	POS
417	JAM2|ENSG00000154721	Stromal_score	0.000000e+00	0.5766364	0.000000e+00	POS	TMEM109|ENSG00000110108	Stromal_score	7.174745e−07	0.4149693	1.640792e−02	POS
418	EDNRA|ENSG00000151617	Stromal_score	0.000000e+00	0.6393327	0.000000e+00	POS	SH3RF3−AS1|ENSG00000259863	Stromal_score	9.319118e−07	0.4109989	2.131189e−02	POS
419	MS4A6A|ENSG00000110077	Stromal_score	0.000000e+00	0.6437616	0.000000e+00	POS	AL133415.1|ENSG00000234961	Stromal_score	9.861313e−11	0.5203931	2.255184e−06	POS
420	LBH|ENSG00000213626	Stromal_score	1.317300e−09	0.4972149	3.012532e−05	POS	SFMBT2|ENSG00000198879	Stromal_score	2.309590e−12	0.5313579	5.281802e−08	POS
421	TWIST2|ENSG00000233608	Stromal_score	0.000000e+00	0.5633645	0.000000e+00	POS	MARCH1|ENSG00000145416	Stromal_score	3.369581e−09	0.4867037	7.705896e−05	POS
422	EML1|ENSG00000066629	Stromal_score	5.111341e−08	0.4525656	1.168913e−03	POS	PCDHB15|ENSG00000113248	Stromal_score	9.171459e−07	0.4112428	2.097421e−02	POS
423	C4A|ENSG00000244731	Stromal_score	4.274079e−08	0.4549556	9.774390e−04	POS	C1QTNF2|ENSG00000145861	Stromal_score	3.476666e−07	0.4257243	7.950787e−03	POS
424	CAMSAP3|ENSG00000076826	Stromal_score	7.638324e−07	−0.4140230	1.746808e−02	NEG	FLT4|ENSG00000037280	Stromal_score	4.204056e−10	0.5087162	9.614255e−06	POS
425	LPAR1|ENSG00000198121	Stromal_score	1.504020e−06	0.4036045	3.439542e−02	POS	IFRD2|ENSG00000214706	Stromal_score	2.120129e−08	−0.4641352	4.848522e−04	NEG
426	PXDN|ENSG00000130508	Stromal_score	0.000000e+00	0.5485123	0.000000e+00	POS	STAT3|ENSG00000168610	Stromal_score	1.896677e−07	0.4344552	4.337510e−03	POS
427	DPYD|ENSG00000188641	Stromal_score	5.635365e−08	0.4512535	1.288752e−03	POS	AP000695.1|ENSG00000230479	Stromal_score	3.045764e−07	0.4276510	6.965358e−03	POS
428	F2R|ENSG00000181104	Stromal_score	0.000000e+00	0.5747293	0.000000e+00	POS	CCM2L|ENSG00000101331	Stromal_score	2.670762e−08	0.4611453	6.107765e−04	POS
429	STEAP4|ENSG00000127954	Stromal_score	7.969935e−07	0.4133792	1.822644e−02	POS	OSBPL8|ENSG00000091039	Stromal_score	3.120302e−07	0.4272998	7.135818e−03	POS
430	ERBB3|ENSG00000065361	Stromal_score	1.825844e−06	−0.4005609	4.175523e−02	NEG	TAF4|ENSG00000130699	Stromal_score	3.960174e−07	−0.4238172	9.056521e−03	NEG
431	JAK3|ENSG00000105639	Stromal_score	1.156975e−07	0.4414057	2.645886e−03	POS	BACH2|ENSG00000112182	Stromal_score	1.926840e−07	0.4342308	4.406491e−03	POS
432	MS4A4A|ENSG00000110079	Stromal_score	0.000000e+00	0.6127549	0.000000e+00	POS	LRRK2|ENSG00000188906	Stromal_score	1.468101e−10	0.5175885	3.357401e−06	POS
433	SIM2|ENSG00000159263	Stromal_score	1.907771e−07	−0.4343723	4.362882e−03	NEG	TBRG4|ENSG00000136270	Stromal_score	8.307245e−07	−0.4127500	1.899784e−02	NEG
434	AC093010.3|ENSG00000259976	Stromal_score	4.057807e−09	0.4845332	9.279798e−05	POS	CALHM5|ENSG00000178033	Stromal_score	0.000000e+00	0.5808165	0.000000e+00	POS
435	FZD8|ENSG00000177283	Stromal_score	7.757357e−07	0.4137889	1.774030e−02	POS	PRICKLE1|ENSG00000139174	Stromal_score	0.000000e+00	0.5530436	0.000000e+00	POS
436	CNTNAP1|ENSG00000108797	Stromal_score	1.480712e−09	0.4959516	3.386241e−05	POS	MAGI2−AS3|ENSG00000234456	Stromal_score	0.000000e+00	0.5978783	0.000000e+00	POS
437	COX7A1|ENSG00000161281	Stromal_score	0.000000e+00	0.5992781	0.000000e+00	POS	PDE1B|ENSG00000123360	Stromal_score	1.155380e−09	0.4986148	2.642238e−05	POS
438	CDC42EP3|ENSG00000163171	Stromal_score	5.805642e−09	0.4802751	1.327692e−04	POS	CFLAR|ENSG00000003402	Stromal_score	7.007357e−07	0.4153253	1.602512e−02	POS
439	STON1|ENSG00000243244	Stromal_score	0.000000e+00	0.5366696	0.000000e+00	POS	TSEN54|ENSG00000182173	Stromal_score	5.288871e−07	−0.4195396	1.209512e−02	NEG
440	PLAC9|ENSG00000189129	Stromal_score	2.751953e−08	0.4607550	6.293440e−04	POS	NAP1L3|ENSG00000186310	Stromal_score	0.000000e+00	0.5506390	0.000000e+00	POS
441	LOXL2|ENSG00000134013	Stromal_score	4.283825e−07	0.4226612	9.796679e−03	POS	SNRK|ENSG00000163788	Stromal_score	4.441017e−07	0.4221295	1.015616e−02	POS
442	PRICKLE2|ENSG00000163637	Stromal_score	1.604388e−08	0.4677007	3.669076e−04	POS	CYLD|ENSG00000083799	Stromal_score	2.690610e−07	0.4294459	6.153155e−03	POS
443	GPSM3|ENSG00000213654	Stromal_score	6.591128e−10	0.5043849	1.507325e−05	POS	ADAMTS5|ENSG00000154736	Stromal_score	2.815088e−07	0.4287923	6.437824e−03	POS
444	CCDC8|ENSG00000169515	Stromal_score	4.319745e−08	0.4548142	9.878826e−04	POS	POU6F1|ENSG00000184271	Stromal_score	7.944853e−08	0.4465906	1.816908e−03	POS
445	LAMA2|ENSG00000196569	Stromal_score	0.000000e+00	0.5886109	0.000000e+00	POS	NLE1|ENSG00000073536	Stromal_score	5.745216e−07	−0.4183055	1.313873e−02	NEG
446	TRBV19|ENSG00000211746	Stromal_score	1.368408e−06	0.4050776	3.129413e−02	POS	SIGLEC9|ENSG00000129450	Stromal_score	1.334771e−08	0.4700273	3.052487e−04	POS
447	LAMA4|ENSG00000112769	Stromal_score	0.000000e+00	0.6656229	0.000000e+00	POS	LRRC8C|ENSG00000171488	Stromal_score	0.000000e+00	0.6560823	0.000000e+00	POS
448	TNS2|ENSG00000111077	Stromal_score	0.000000e+00	0.5840064	0.000000e+00	POS	FBXO45|ENSG00000174013	Stromal_score	1.845141e−06	−0.4003951	4.219653e−02	NEG
449	ABCC9|ENSG00000069431	Stromal_score	0.000000e+00	0.5678714	0.000000e+00	POS	PEAK1|ENSG00000173517	Stromal_score	8.981787e−08	0.4449078	2.054045e−03	POS
450	LPXN|ENSG00000110031	Stromal_score	6.634021e−07	0.4161496	1.517134e−02	POS	TRPC1|ENSG00000144935	Stromal_score	1.446573e−08	0.4690128	3.308167e−04	POS
451	GAS7|ENSG00000007237	Stromal_score	0.000000e+00	0.6323237	0.000000e+00	POS	PEX7|ENSG00000112357	Stromal_score	1.221183e−06	−0.4068432	2.792724e−02	NEG
452	RAMP3|ENSG00000122679	Stromal_score	4.153660e−10	0.5088284	9.499006e−06	POS	EBF3|ENSG00000108001	Stromal_score	4.466610e−09	0.4834016	1.021469e−04	POS
453	CD209|ENSG00000090659	Stromal_score	4.188467e−07	0.4229929	9.578605e−03	POS	TPM4|ENSG00000167460	Stromal_score	1.895407e−06	0.3999707	4.334605e−02	POS
454	MNDA|ENSG00000163563	Stromal_score	0.000000e+00	0.5681641	0.000000e+00	POS	CYB5R3|ENSG00000100243	Stromal_score	1.179896e−06	0.4073749	2.698303e−02	POS
455	CHST3|ENSG00000122863	Stromal_score	2.029677e−08	0.4646961	4.641669e−04	POS	TLR6|ENSG00000174130	Stromal_score	9.802331e−08	0.4437031	2.241695e−03	POS
456	MEOX2|ENSG00000106511	Stromal_score	1.145177e−10	0.5193786	2.618904e−06	POS	IFFO1|ENSG00000010295	Stromal_score	0.000000e+00	0.6746805	0.000000e+00	POS
457	SNAI2|ENSG00000019549	Stromal_score	0.000000e+00	0.5503414	0.000000e+00	POS	FLRT2|ENSG00000185070	Stromal_score	9.491743e−09	0.4742806	2.170667e−04	POS
458	COPZ2|ENSG00000005243	Stromal_score	2.815119e−09	0.4887767	6.437895e−05	POS	PREX2|ENSG00000046889	Stromal_score	7.133721e−10	0.5035948	1.631411e−05	POS
459	TGFB3|ENSG00000119699	Stromal_score	0.000000e+00	0.6402205	0.000000e+00	POS	TMEM177|ENSG00000144120	Stromal_score	7.096314e−07	−0.4151351	1.622856e−02	NEG
460	FOXS1|ENSG00000179772	Stromal_score	0.000000e+00	0.5888352	0.000000e+00	POS	PYM1|ENSG00000170473	Stromal_score	3.710288e−07	−0.4247732	8.485057e−03	NEG
461	TNFAIP3|ENSG00000118503	Stromal_score	1.272068e−07	0.4400839	2.909093e−03	POS	DOK5|ENSG00000101134	Stromal_score	0.000000e+00	0.5869281	0.000000e+00	POS
462	COL5A3|ENSG00000080573	Stromal_score	2.881312e−07	0.4284558	6.589273e−03	POS	FAM212A|ENSG00000185614	Stromal_score	8.240829e−07	0.4128719	1.884595e−02	POS
463	DACT1|ENSG00000165617	Stromal_score	0.000000e+00	0.6309189	0.000000e+00	POS	DPEP2|ENSG00000167261	Stromal_score	2.261892e−11	0.5276900	5.172721e−07	POS
464	SGCD|ENSG00000170624	Stromal_score	0.000000e+00	0.5336260	0.000000e+00	POS	FASTKD1|ENSG00000138399	Stromal_score	8.016204e−07	−0.4132914	1.833226e−02	NEG
465	NCKAP1L|ENSG00000123338	Stromal_score	3.122097e−09	0.4875866	7.139925e−05	POS	SCARF1|ENSG00000074660	Stromal_score	7.016721e−10	0.5037606	1.604654e−05	POS
466	ARHGAP30|ENSG00000186517	Stromal_score	4.348066e−07	0.4224417	9.943591e−03	POS	HRH2|ENSG00000113749	Stromal_score	1.159570e−06	0.4076432	2.651822e−02	POS
467	A4GALT|ENSG00000128274	Stromal_score	3.489462e−07	0.4256707	7.980050e−03	POS	GSTM5|ENSG00000134201	Stromal_score	2.399421e−09	0.4905960	5.487236e−05	POS
468	CHI3L2|ENSG00000064886	Stromal_score	1.465190e−07	0.4381036	3.350744e−03	POS	ILVBL|ENSG00000105135	Stromal_score	1.231621e−06	−0.4067115	2.816595e−02	NEG
469	WISP1|ENSG00000104415	Stromal_score	0.000000e+00	0.5721393	0.000000e+00	POS	TRMT10C|ENSG00000174173	Stromal_score	8.479604e−07	−0.4124378	1.939201e−02	NEG
470	SDC2|ENSG00000169439	Stromal_score	0.000000e+00	0.5788216	0.000000e+00	POS	ADCY4|ENSG00000129467	Stromal_score	1.014377e−07	0.4432299	2.319778e−03	POS
471	APOL3|ENSG00000128284	Stromal_score	5.475688e−07	0.4190225	1.252235e−02	POS	FUT11|ENSG00000196968	Stromal_score	1.148664e−08	0.4719101	2.626880e−04	POS
472	ARL4C|ENSG00000188042	Stromal_score	7.546501e−11	0.5220857	1.725809e−06	POS	RGS18|ENSG00000150681	Stromal_score	8.505846e−10	0.5018096	1.945202e−05	POS
473	S1PR4|ENSG00000125910	Stromal_score	7.815019e−07	0.4136767	1.787217e−02	POS	DOCK4|ENSG00000128512	Stromal_score	9.052289e−09	0.4748659	2.070168e−04	POS
474	F2RL3|ENSG00000127533	Stromal_score	1.646217e−06	0.4021900	3.764734e−02	POS	PLBD2|ENSG00000151176	Stromal_score	1.193383e−06	0.4071993	2.729146e−02	POS
475	RGS4|ENSG00000117152	Stromal_score	2.135623e−09	0.4919081	4.883956e−05	POS	FCGR2C|ENSG00000244682	Stromal_score	0.000000e+00	0.5354404	0.000000e+00	POS
476	PLSCR4|ENSG00000114698	Stromal_score	3.409971e−08	0.4579456	7.798263e−04	POS	PTGIR|ENSG00000160013	Stromal_score	0.000000e+00	0.6565945	0.000000e+00	POS
477	PLEKHO1|ENSG00000023902	Stromal_score	0.000000e+00	0.5917423	0.000000e+00	POS	SMUG1|ENSG00000123415	Stromal_score	1.758171e−06	−0.4011560	4.020761e−02	NEG
478	ITGA1|ENSG00000213949	Stromal_score	9.319160e−10	0.5008682	2.131199e−05	POS	RSU1|ENSG00000148484	Stromal_score	7.475083e−07	0.4143498	1.709477e−02	POS
479	SNRPN|ENSG00000128739	Stromal_score	1.670500e−10	0.5166081	3.820266e−06	POS	ARMC9|ENSG00000135931	Stromal_score	9.351906e−07	0.4109453	2.138687e−02	POS
480	HEG1|ENSG00000173706	Stromal_score	0.000000e+00	0.6760560	0.000000e+00	POS	RNF180|ENSG00000164197	Stromal_score	3.182622e−08	0.4588528	7.278339e−04	POS

	gene	feature	p.value	estimate	adj.p.value	COR.ID	gene	feature	p.value	estimate	adj.p.value	COR.ID
481	RAB25|ENSG00000132698	Stromal_score	1.600273e−06	−0.4026339	3.659665e−02	NEG	TENM4|ENSG00000149256	Stromal_score	5.140021e−09	0.4817335	1.175471e−04	POS
482	ETS1|ENSG00000134954	Stromal_score	0.000000e+00	0.6428153	0.000000e+00	POS	NLRP3|ENSG00000162711	Stromal_score	6.866010e−08	0.4485806	1.570188e−03	POS
483	SVEP1|ENSG00000165124	Stromal_score	1.285659e−11	0.5292557	2.940174e−07	POS	AC074117.1|ENSG00000234072	Stromal_score	9.698246e−07	−0.4103892	2.217892e−02	NEG
484	PLVAP|ENSG00000130300	Stromal_score	2.874942e−08	0.4601844	6.574706e−04	POS	TBXA2R|ENSG00000006638	Stromal_score	4.937658e−08	0.4530290	1.129193e−03	POS
485	NID2|ENSG00000087303	Stromal_score	0.000000e+00	0.5608965	0.000000e+00	POS	LEPROT|ENSG00000213625	Stromal_score	0.000000e+00	0.5359380	0.000000e+00	POS
486	GMFG|ENSG00000130755	Stromal_score	0.000000e+00	0.5740074	0.000000e+00	POS	AC121338.2|ENSG00000278385	Stromal_score	8.578048e−07	−0.4122622	1.961714e−02	NEG
487	RASSF8|ENSG00000123094	Stromal_score	1.089593e−08	0.4725685	2.491789e−04	POS	MINDY2|ENSG00000128923	Stromal_score	1.220414e−06	0.4068530	2.790964e−02	POS
488	SOX18|ENSG00000203883	Stromal_score	5.571443e−07	0.4187640	1.274133e−02	POS	CNPY4|ENSG00000166997	Stromal_score	2.065540e−06	0.3986099	4.723684e−02	POS
489	GPX7|ENSG00000116157	Stromal_score	1.955450e−07	0.4340211	4.471919e−03	POS	MRPS25|ENSG00000131368	Stromal_score	4.747000e−07	−0.4211443	1.085591e−02	NEG
490	ITGBL1|ENSG00000198542	Stromal_score	1.547352e−10	0.5171934	3.538639e−06	POS	TMEM71|ENSG00000165071	Stromal_score	2.159277e−07	0.4326066	4.938051e−03	POS
491	PCED1B−AS1|ENSG00000247774	Stromal_score	3.522863e−09	0.4861867	8.056436e−05	POS	FXYD1|ENSG00000266964	Stromal_score	9.797641e−07	0.4102331	2.240623e−02	POS
492	P2RY14|ENSG00000174944	Stromal_score	1.219278e−08	0.4711638	2.788367e−04	POS	SHE|ENSG00000169291	Stromal_score	9.227444e−10	0.5009706	2.110224e−05	POS
493	VASN|ENSG00000168140	Stromal_score	9.556025e−08	0.4440542	2.185367e−03	POS	TGDS|ENSG00000088451	Stromal_score	1.407994e−06	−0.4046337	3.219942e−02	NEG
494	EFEMP2|ENSG00000172638	Stromal_score	0.000000e+00	0.6635889	0.000000e+00	POS	LRCH2|ENSG00000130224	Stromal_score	0.000000e+00	0.5377817	0.000000e+00	POS
495	ADGRL2|ENSG00000117114	Stromal_score	4.972401e−07	0.4204565	1.137138e−02	POS	MMAB|ENSG00000139428	Stromal_score	8.060139e−07	−0.4132085	1.843273e−02	NEG
496	HCK|ENSG00000101336	Stromal_score	0.000000e+00	0.5380890	0.000000e+00	POS	EIPR1|ENSG00000032389	Stromal_score	1.638049e−06	−0.4022681	3.746055e−02	NEG
497	LAMB2|ENSG00000172037	Stromal_score	5.372701e−07	0.4193054	1.228683e−02	POS	GNGT2|ENSG00000167083	Stromal_score	4.560152e−08	0.4540923	1.042861e−03	POS
498	LILRB4|ENSG00000186818	Stromal_score	1.461395e−10	0.5176227	3.342064e−06	POS	HS3ST3B1|ENSG00000125430	Stromal_score	1.067245e−06	0.4089211	2.440681e−02	POS
499	ITGAX|ENSG00000140678	Stromal_score	0.000000e+00	0.5374012	0.000000e+00	POS	ACVR1|ENSG00000115170	Stromal_score	1.303015e−09	0.4973320	2.979866e−05	POS
500	NCF1C|ENSG00000165178	Stromal_score	1.669371e−07	0.4362648	3.817684e−03	POS	PCDHGA12|ENSG00000253159	Stromal_score	3.618169e−10	0.5100966	8.274390e−06	POS
501	GFPT2|ENSG00000131459	Stromal_score	0.000000e+00	0.5856258	0.000000e+00	POS	RASGRP3|ENSG00000152689	Stromal_score	1.721614e−08	0.4668032	3.937160e−04	POS
502	LGI2|ENSG00000153012	Stromal_score	1.466183e−10	0.5175983	3.353014e−06	POS	MGAT1|ENSG00000131446	Stromal_score	1.324050e−06	0.4055897	3.027970e−02	POS
503	VCL|ENSG00000035403	Stromal_score	1.796702e−06	0.4008146	4.108878e−02	POS	CYSLTR1|ENSG00000173198	Stromal_score	8.892519e−11	0.5210662	2.033630e−06	POS
504	NCF2|ENSG00000116701	Stromal_score	2.692859e−08	0.4610379	6.158298e−04	POS	GIPC3|ENSG00000179855	Stromal_score	5.119716e−10	0.5068530	1.170828e−05	POS
505	AKT3|ENSG00000117020	Stromal_score	0.000000e+00	0.5977173	0.000000e+00	POS	AC090877.2|ENSG00000259721	Stromal_score	3.169640e−08	0.4589064	7.248650e−04	POS
506	GPC6|ENSG00000183098	Stromal_score	0.000000e+00	0.5833772	0.000000e+00	POS	GAB3|ENSG00000160219	Stromal_score	2.281876e−11	0.5276607	5.218422e−07	POS
507	TWIST1|ENSG00000122691	Stromal_score	9.951724e−07	0.4099941	2.275860e−02	POS	ZNF436|ENSG00000125945	Stromal_score	1.792797e−08	0.4662862	4.099947e−04	POS
508	ARMCX2|ENSG00000184867	Stromal_score	2.177769e−07	0.4324846	4.980339e−03	POS	MAP1LC3B|ENSG00000140941	Stromal_score	3.820559e−07	0.4243440	8.737237e−03	POS
509	LCP2|ENSG00000043462	Stromal_score	0.000000e+00	0.5893913	0.000000e+00	POS	SSH1|ENSG00000084112	Stromal_score	7.266501e−10	0.5034094	1.661776e−05	POS
510	RCSD1|ENSG00000198771	Stromal_score	1.544422e−09	0.4954931	3.531939e−05	POS	DSEL|ENSG00000171451	Stromal_score	6.060685e−10	0.5052141	1.386018e−05	POS
511	CD96|ENSG00000153283	Stromal_score	1.986981e−06	0.3992245	4.544026e−02	POS	RGS7BP|ENSG00000186479	Stromal_score	1.562841e−06	0.4030046	3.574060e−02	POS
512	PRDM1|ENSG00000057657	Stromal_score	9.429842e−07	0.4108185	2.156511e−02	POS	CCR8|ENSG00000179934	Stromal_score	1.989430e−06	0.3992050	4.549628e−02	POS
513	ENG|ENSG00000106991	Stromal_score	0.000000e+00	0.6129987	0.000000e+00	POS	PHACTR1|ENSG00000112137	Stromal_score	6.281752e−07	0.4169691	1.436574e−02	POS
514	ACTA2−AS1|ENSG00000180139	Stromal_score	1.208533e−09	0.4981368	2.763794e−05	POS	CARD8−AS1|ENSG00000268001	Stromal_score	6.603960e−07	0.4162179	1.510260e−02	POS
515	SLFN11|ENSG00000172716	Stromal_score	1.075401e−06	0.4088040	2.459335e−02	POS	TFE3|ENSG00000068323	Stromal_score	4.158052e−07	0.4231002	9.509049e−03	POS
516	NR2F2|ENSG00000185551	Stromal_score	2.275616e−07	0.4318554	5.204107e−03	POS	CHSY3|ENSG00000198108	Stromal_score	6.872913e−09	0.4782363	1.571767e−04	POS
517	VAMP5|ENSG00000168899	Stromal_score	2.008263e−09	0.4925958	4.592696e−05	POS	SLC39A13|ENSG00000165915	Stromal_score	1.035844e−08	0.4731977	2.368871e−04	POS
518	SAMHD1|ENSG00000101347	Stromal_score	5.372118e−08	0.4518974	1.228550e−03	POS	MCTP1|ENSG00000175471	Stromal_score	1.416200e−09	0.4964345	3.238708e−05	POS
519	HSD11B1|ENSG00000117594	Stromal_score	0.000000e+00	0.5639206	0.000000e+00	POS	CNTN4|ENSG00000144619	Stromal_score	2.857362e−09	0.4886060	6.534502e−05	POS
520	CTSL|ENSG00000135047	Stromal_score	4.308636e−10	0.5084870	9.853419e−06	POS	AC093278.2|ENSG00000261269	Stromal_score	6.447515e−08	0.4494342	1.474482e−03	POS
521	KCNJ8|ENSG00000121361	Stromal_score	0.000000e+00	0.6133792	0.000000e+00	POS	C1orf27|ENSG00000157181	Stromal_score	1.755993e−06	−0.4011755	4.015781e−02	NEG
522	PLXND1|ENSG00000004399	Stromal_score	3.229322e−09	0.4871964	7.385137e−05	POS	MAPK10|ENSG00000109339	Stromal_score	5.103912e−08	0.4525851	1.167214e−03	POS
523	AOAH|ENSG00000136250	Stromal_score	5.768065e−09	0.4803531	1.319099e−04	POS	CEP76|ENSG00000101624	Stromal_score	1.618805e−06	−0.4024534	3.702044e−02	NEG
524	CDK14|ENSG00000058091	Stromal_score	1.646798e−08	0.4673690	3.766063e−04	POS	CDK5RAP1|ENSG00000101391	Stromal_score	6.989241e−07	−0.4153644	1.598370e−02	NEG
525	RFTN1|ENSG00000131378	Stromal_score	0.000000e+00	0.7529851	0.000000e+00	POS	AP001528.3|ENSG00000280339	Stromal_score	1.831523e−08	0.4660131	4.188511e−04	POS
526	MSR1|ENSG00000038945	Stromal_score	0.000000e+00	0.5862160	0.000000e+00	POS	MRPS5|ENSG00000144029	Stromal_score	2.019673e−06	−0.3989660	4.618790e−02	NEG
527	SLA|ENSG00000155926	Stromal_score	1.430956e−09	0.4963223	3.272454e−05	POS	VTA1|ENSG00000009844	Stromal_score	5.245705e−07	−0.4196615	1.199640e−02	NEG
528	ATP8B2|ENSG00000143515	Stromal_score	0.000000e+00	0.5963613	0.000000e+00	POS	NFS1|ENSG00000244005	Stromal_score	3.475919e−09	−0.4863428	7.949079e−05	NEG
529	RHOJ|ENSG00000126785	Stromal_score	0.000000e+00	0.5903278	0.000000e+00	POS	PCAT19|ENSG00000267107	Stromal_score	1.561125e−07	0.4372110	3.570136e−03	POS
530	STAB1|ENSG00000010327	Stromal_score	0.000000e+00	0.6152229	0.000000e+00	POS	CCDC138|ENSG00000163006	Stromal_score	1.063526e−06	−0.4089747	2.432177e−02	NEG
531	DCHS1|ENSG00000166341	Stromal_score	0.000000e+00	0.5604283	0.000000e+00	POS	MTIF2|ENSG00000085760	Stromal_score	3.683199e−07	−0.4248805	8.423107e−03	NEG
532	CHST11|ENSG00000171310	Stromal_score	0.000000e+00	0.6113647	0.000000e+00	POS	MAIP1|ENSG00000162972	Stromal_score	1.913630e−06	−0.3998195	4.376281e−02	NEG
533	SLC15A3|ENSG00000110446	Stromal_score	1.740479e−07	0.4356746	3.980302e−03	POS	OXSM|ENSG00000151093	Stromal_score	9.230266e−08	−0.4445322	2.110870e−03	NEG
534	LIX1L|ENSG00000271601	Stromal_score	0.000000e+00	0.5668959	0.000000e+00	POS	RORA|ENSG00000069667	Stromal_score	2.421452e−08	0.4624183	5.537617e−04	POS
535	GIMAP6|ENSG00000133561	Stromal_score	0.000000e+00	0.6127061	0.000000e+00	POS	SNHG10|ENSG00000247092	Stromal_score	2.443119e−07	−0.4308360	5.587169e−03	NEG
536	COL8A2|ENSG00000171812	Stromal_score	0.000000e+00	0.5933470	0.000000e+00	POS	ABCA9|ENSG00000154258	Stromal_score	7.461290e−11	0.5221539	1.706322e−06	POS
537	SH3PXD2B|ENSG00000174705	Stromal_score	1.473868e−10	0.5175593	3.370588e−06	POS	LIMS1|ENSG00000169756	Stromal_score	4.009606e−08	0.4558043	9.169569e−04	POS
538	IL18BP|ENSG00000137496	Stromal_score	3.103677e−09	0.4876549	7.097798e−05	POS	AC093535.2|ENSG00000279118	Stromal_score	2.098554e−08	0.4642669	4.799183e−04	POS
539	IGSF6|ENSG00000140749	Stromal_score	8.540151e−09	0.4755829	1.953047e−04	POS	CREBRF|ENSG00000164463	Stromal_score	5.556888e−07	0.4188030	1.270805e−02	POS
540	CDH5|ENSG00000179776	Stromal_score	0.000000e+00	0.5507707	0.000000e+00	POS	MKL1|ENSG00000196588	Stromal_score	8.023941e−07	0.4132768	1.834995e−02	POS
541	CLEC10A|ENSG00000132514	Stromal_score	4.740750e−07	0.4211638	1.084162e−02	POS	ISLR2|ENSG00000167178	Stromal_score	2.033532e−08	0.4646717	4.650485e−04	POS
542	TGFB1|ENSG00000105329	Stromal_score	1.330517e−09	0.4971076	3.042760e−05	POS	CAMKMT|ENSG00000143919	Stromal_score	4.671694e−09	−0.4828700	1.068370e−04	NEG
543	PBX3|ENSG00000167081	Stromal_score	1.700790e−06	0.4016779	3.889536e−02	POS	RASGRF2|ENSG00000113319	Stromal_score	0.000000e+00	0.5472881	0.000000e+00	POS
544	BICC1|ENSG00000122870	Stromal_score	0.000000e+00	0.6810019	0.000000e+00	POS	AL031651.2|ENSG00000277829	Stromal_score	3.154968e−10	0.5113257	7.215095e−06	POS
545	GPX8|ENSG00000164294	Stromal_score	0.000000e+00	0.5903814	0.000000e+00	POS	ZNF366|ENSG00000178175	Stromal_score	4.880078e−07	0.4207346	1.116025e−02	POS
546	GNG11|ENSG00000127920	Stromal_score	0.000000e+00	0.5910789	0.000000e+00	POS	UMPS|ENSG00000114491	Stromal_score	1.102261e−07	−0.4420788	2.520760e−03	NEG
547	SPN|ENSG00000197471	Stromal_score	5.626350e−07	0.4186177	1.286690e−02	POS	DNM3OS|ENSG00000230630	Stromal_score	0.000000e+00	0.6077066	0.000000e+00	POS
548	CLEC14A|ENSG00000176435	Stromal_score	0.000000e+00	0.5788069	0.000000e+00	POS	MRPS22|ENSG00000175110	Stromal_score	1.273428e−06	−0.4061945	2.912203e−02	NEG
549	PAPLN|ENSG00000100767	Stromal_score	9.825771e−07	0.4101892	2.247055e−02	POS	BHLHE22|ENSG00000180828	Stromal_score	0.000000e+00	0.5852210	0.000000e+00	POS
550	PDGFRL|ENSG00000104213	Stromal_score	2.295840e−07	0.4317286	5.250356e−03	POS	AC008105.3|ENSG00000267121	Stromal_score	7.913729e−07	0.4134865	1.809791e−02	POS
551	LST1|ENSG00000204482	Stromal_score	0.000000e+00	0.5449176	0.000000e+00	POS	UCHL5|ENSG00000116750	Stromal_score	4.564391e−07	−0.4217247	1.043831e−02	NEG
552	FAP|ENSG00000078098	Stromal_score	0.000000e+00	0.5959906	0.000000e+00	POS	LINC01943|ENSG00000280721	Stromal_score	7.216601e−07	0.4148815	1.650365e−02	POS
553	MSC|ENSG00000178860	Stromal_score	0.000000e+00	0.6442737	0.000000e+00	POS	MIR99AHG|ENSG00000215386	Stromal_score	7.601485e−07	0.4140962	1.738384e−02	POS
554	SPART|ENSG00000133104	Stromal_score	1.314946e−07	0.4396205	3.007150e−03	POS	TXNDC15|ENSG00000113621	Stromal_score	3.023546e−10	0.5117013	6.914548e−06	POS
555	CMTM3|ENSG00000140931	Stromal_score	0.000000e+00	0.6585797	0.000000e+00	POS	ADAMTSL1|ENSG00000178031	Stromal_score	1.216832e−07	0.4407033	2.782772e−03	POS
556	ARHGAP9|ENSG00000123329	Stromal_score	3.495293e−07	0.4256463	7.993385e−03	POS	LINC01094|ENSG00000251442	Stromal_score	7.064693e−10	0.5036923	1.615625e−05	POS
557	EVC|ENSG00000072840	Stromal_score	0.000000e+00	0.5707492	0.000000e+00	POS	PIEZO2|ENSG00000154864	Stromal_score	7.110095e−07	0.4151058	1.626008e−02	POS
558	GLIS2|ENSG00000126603	Stromal_score	8.416642e−08	0.4458004	1.924802e−03	POS	SLC45A1|ENSG00000162426	Stromal_score	1.915018e−08	0.4654424	4.379454e−04	POS
559	PAM|ENSG00000145730	Stromal_score	4.405940e−07	0.4222466	1.007594e−02	POS	PRAM1|ENSG00000133246	Stromal_score	4.155051e−08	0.4553312	9.502186e−04	POS
560	CNN3|ENSG00000117519	Stromal_score	5.206974e−08	0.4523168	1.190783e−03	POS	ZNF823|ENSG00000197933	Stromal_score	7.742382e−07	−0.4138182	1.770605e−02	NEG
561	CCR5|ENSG00000160791	Stromal_score	4.231685e−07	0.4228417	9.677440e−03	POS	HLX|ENSG00000136630	Stromal_score	1.561083e−09	0.4953761	3.570040e−05	POS
562	LAYN|ENSG00000204381	Stromal_score	0.000000e+00	0.5340308	0.000000e+00	POS	THSD7A|ENSG00000005108	Stromal_score	0.000000e+00	0.5554629	0.000000e+00	POS
563	FMNL1|ENSG00000184922	Stromal_score	2.565843e−09	0.4898351	5.867827e−05	POS	NOX4|ENSG00000086991	Stromal_score	9.567470e−12	0.5298556	2.187985e−07	POS
564	SLIT2|ENSG00000145147	Stromal_score	0.000000e+00	0.5867184	0.000000e+00	POS	HIRA|ENSG00000100084	Stromal_score	9.411802e−07	−0.4108477	2.152385e−02	NEG
565	PLPP1|ENSG00000067113	Stromal_score	4.891583e−09	0.4823237	1.118656e−04	POS	SGTB|ENSG00000197860	Stromal_score	6.333428e−09	0.4792264	1.448392e−04	POS
566	KLHL5|ENSG00000109790	Stromal_score	5.189974e−08	0.4523607	1.186895e−03	POS	NLGN4Y|ENSG00000165246	Stromal_score	3.573142e−07	0.4253244	8.171417e−03	POS
567	CTSO|ENSG00000256043	Stromal_score	0.000000e+00	0.6098429	0.000000e+00	POS	PCDHB7|ENSG00000113212	Stromal_score	1.787825e−06	0.4008926	4.088577e−02	POS
568	RASL12|ENSG00000103710	Stromal_score	0.000000e+00	0.5521949	0.000000e+00	POS	KLRD1|ENSG00000134539	Stromal_score	7.640786e−07	0.4140181	1.747371e−02	POS
569	KIRREL1|ENSG00000183853	Stromal_score	0.000000e+00	0.5713443	0.000000e+00	POS	LINC01711|ENSG00000268941	Stromal_score	1.653396e−06	0.4021217	3.781151e−02	POS
570	CALCRL|ENSG00000064989	Stromal_score	0.000000e+00	0.6072968	0.000000e+00	POS	PLCL1|ENSG00000115896	Stromal_score	1.639866e−08	0.4674227	3.750210e−04	POS
571	GPR34|ENSG00000171659	Stromal_score	0.000000e+00	0.5661984	0.000000e+00	POS	P2RY12|ENSG00000169313	Stromal_score	6.642634e−07	0.4161301	1.519104e−02	POS
572	MMRN2|ENSG00000173269	Stromal_score	0.000000e+00	0.5841625	0.000000e+00	POS	PHC2|ENSG00000134686	Stromal_score	7.355436e−07	0.4145937	1.682115e−02	POS
573	CCR1|ENSG00000163823	Stromal_score	0.000000e+00	0.5638474	0.000000e+00	POS	AL021453.1|ENSG00000184068	Stromal_score	1.367550e−06	−0.4050873	3.127451e−02	NEG
574	CYGB|ENSG00000161544	Stromal_score	0.000000e+00	0.5738416	0.000000e+00	POS	RNLS|ENSG00000184719	Stromal_score	1.263448e−06	0.4063165	2.889379e−02	POS
575	KCNE4|ENSG00000152049	Stromal_score	0.000000e+00	0.6026095	0.000000e+00	POS	GIT2|ENSG00000139436	Stromal_score	1.001528e−06	0.4098966	2.290394e−02	POS
576	GJA4|ENSG00000187513	Stromal_score	1.210194e−09	0.4981221	2.767593e−05	POS	AC087286.2|ENSG00000259367	Stromal_score	5.280559e−10	0.5065555	1.207611e−05	POS
577	SKP2|ENSG00000145604	Stromal_score	1.830137e−07	−0.4349624	4.185340e−03	NEG	AL109741.1|ENSG00000225938	Stromal_score	0.000000e+00	0.5443030	0.000000e+00	POS
578	MAF|ENSG00000178573	Stromal_score	0.000000e+00	0.5611306	0.000000e+00	POS	KCND1|ENSG00000102057	Stromal_score	2.970931e−07	0.4280119	6.794221e−03	POS
579	PILRA|ENSG00000085514	Stromal_score	2.024292e−08	0.4647303	4.629353e−04	POS	AMPH|ENSG00000078053	Stromal_score	1.825943e−08	0.4660521	4.175748e−04	POS
580	ABI3|ENSG00000108798	Stromal_score	0.000000e+00	0.5494440	0.000000e+00	POS	TMEM260|ENSG00000070269	Stromal_score	1.597287e−06	−0.4026632	3.652836e−02	NEG
581	ARMCX1|ENSG00000126947	Stromal_score	4.319401e−07	0.4225393	9.878039e−03	POS	PPIAP39|ENSG00000230018	Stromal_score	3.755026e−07	0.4245976	8.587370e−03	POS
582	CD84|ENSG00000066294	Stromal_score	0.000000e+00	0.5558482	0.000000e+00	POS	NECAP2|ENSG00000157191	Stromal_score	1.785612e−06	0.4009121	4.083516e−02	POS
583	DAB2|ENSG00000153071	Stromal_score	5.429269e−10	0.5062872	1.241619e−05	POS	PHTF2|ENSG00000006576	Stromal_score	2.522238e−07	0.4303775	5.768107e−03	POS
584	ADAMTS4|ENSG00000158859	Stromal_score	5.252566e−08	0.4521998	1.201209e−03	POS	Z97200.1|ENSG00000271811	Stromal_score	2.489892e−09	0.4901766	5.694133e−05	POS
585	JCAD|ENSG00000165757	Stromal_score	0.000000e+00	0.5533070	0.000000e+00	POS	TSEN2|ENSG00000154743	Stromal_score	3.366880e−07	−0.4261926	7.699718e−03	NEG
586	CD36|ENSG00000135218	Stromal_score	7.119297e−07	0.4150863	1.628112e−02	POS	AC004921.1|ENSG00000273341	Stromal_score	4.523902e−07	0.4218564	1.034571e−02	POS
587	AC105243.1|ENSG00000261780	Stromal_score	7.253048e−08	0.4478344	1.658700e−03	POS	ZNF471|ENSG00000196263	Stromal_score	2.080835e−06	0.3984928	4.758662e−02	POS
588	OLFML2A|ENSG00000185585	Stromal_score	9.766476e−07	0.4102819	2.233495e−02	POS	CD33|ENSG00000105383	Stromal_score	3.873218e−09	0.4850795	8.857662e−05	POS
589	IL2RA|ENSG00000134460	Stromal_score	7.071115e−07	0.4151888	1.617093e−02	POS	EBF2|ENSG00000221818	Stromal_score	2.551454e−07	0.4302117	5.834921e−03	POS
590	FAM19A5|ENSG00000219438	Stromal_score	1.524268e−07	0.4375476	3.485849e−03	POS	CCDC102B|ENSG00000150636	Stromal_score	6.482905e−07	0.4164960	1.482576e−02	POS
591	ARHGEF17|ENSG00000110237	Stromal_score	1.615644e−09	0.4950005	3.694817e−05	POS	AP002004.1|ENSG00000235505	Stromal_score	0.000000e+00	0.5788947	0.000000e+00	POS
592	GLIPR2|ENSG00000122694	Stromal_score	0.000000e+00	0.5712857	0.000000e+00	POS	PIK3R6|ENSG00000276231	Stromal_score	3.209931e−08	0.4587406	7.340791e−04	POS
593	APOLD1|ENSG00000178878	Stromal_score	1.307173e−07	0.4397034	2.989374e−03	POS	AC131235.3|ENSG00000272721	Stromal_score	3.832012e−07	−0.4243001	8.763428e−03	NEG
594	NLGN2|ENSG00000169992	Stromal_score	2.167567e−06	0.3978441	4.957009e−02	POS	ARHGAP20|ENSG00000137727	Stromal_score	2.679859e−10	0.5127500	6.128569e−06	POS
595	MFGE8|ENSG00000140545	Stromal_score	7.613877e−11	0.5220320	1.741217e−06	POS	MAP3K12|ENSG00000139625	Stromal_score	3.245887e−08	0.4585943	7.423019e−04	POS
596	CD34|ENSG00000174059	Stromal_score	0.000000e+00	0.6076578	0.000000e+00	POS	TM6SF1|ENSG00000136404	Stromal_score	0.000000e+00	0.6133353	0.000000e+00	POS
597	PTCH2|ENSG00000117425	Stromal_score	5.035960e−09	0.4819774	1.151674e−04	POS	IPO4|ENSG00000196497	Stromal_score	2.415090e−07	−0.4310019	5.523069e−03	NEG
598	FRMD6|ENSG00000139926	Stromal_score	0.000000e+00	0.5604624	0.000000e+00	POS	FAM124B|ENSG00000124019	Stromal_score	2.437966e−09	0.4904156	5.575385e−05	POS
599	MAGEH1|ENSG00000187601	Stromal_score	1.192375e−07	0.4409862	2.726842e−03	POS	AL049838.1|ENSG00000259969	Stromal_score	5.150514e−08	0.4524632	1.177871e−03	POS
600	TSHZ3|ENSG00000121297	Stromal_score	0.000000e+00	0.6018437	0.000000e+00	POS	SIRPB2|ENSG00000196209	Stromal_score	8.590695e−11	0.5212857	1.964606e−06	POS

	gene	feature	p.value	estimate	adj.p.value	COR.ID	gene	feature	p.value	estimate	adj.p.value	COR.ID
601	ADAM19|ENSG00000135074	Stromal_score	0.000000e+00	0.6044679	0.000000e+00	POS	BATF3|ENSG00000123685	Stromal_score	9.098469e−07	0.4113647	2.080729e−02	POS
602	SCIMP|ENSG00000161929	Stromal_score	1.432891e−09	0.4963077	3.276879e−05	POS	MMP23B|ENSG00000189409	Stromal_score	2.884617e−08	0.4601405	6.596831e−04	POS
603	DOCK8|ENSG00000107099	Stromal_score	7.724057e−08	0.4469759	1.766415e−03	POS	SGIP1|ENSG00000118473	Stromal_score	0.000000e+00	0.5919227	0.000000e+00	POS
604	TSPAN11|ENSG00000110900	Stromal_score	6.894864e−07	0.4155692	1.576787e−02	POS	PIANP|ENSG00000139200	Stromal_score	1.758552e−07	0.4355282	4.021633e−03	POS
605	LEF1|ENSG00000138795	Stromal_score	2.738601e−08	0.4608185	6.262906e−04	POS	EIF2B5|ENSG00000145191	Stromal_score	1.679279e−06	−0.4018779	3.840342e−02	NEG
606	FAM107A|ENSG00000168309	Stromal_score	2.898831e−07	0.4283680	6.629338e−03	POS	CLEC1A|ENSG00000150048	Stromal_score	0.000000e+00	0.5566823	0.000000e+00	POS
607	CADM3|ENSG00000162706	Stromal_score	3.054988e−07	0.4276071	6.986451e−03	POS	MMP16|ENSG00000156103	Stromal_score	4.184124e−08	0.4552385	9.568673e−04	POS
608	DOK2|ENSG00000147443	Stromal_score	2.837155e−10	0.5122573	6.488290e−06	POS	TNFRSF8|ENSG00000120949	Stromal_score	1.768003e−06	0.4010682	4.043247e−02	POS
609	MFNG|ENSG00000100060	Stromal_score	1.067635e−09	0.4994488	2.441573e−05	POS	ZNF677|ENSG00000197928	Stromal_score	6.068750e−07	0.4174861	1.387862e−02	POS
610	THEMIS2|ENSG00000130775	Stromal_score	0.000000e+00	0.5586723	0.000000e+00	POS	RASGRP4|ENSG00000171777	Stromal_score	3.405378e−07	0.4260267	7.787759e−03	POS
611	FOXP3|ENSG00000049768	Stromal_score	1.678757e−06	0.4018827	3.839150e−02	POS	MSC−AS1|ENSG00000235531	Stromal_score	0.000000e+00	0.6645010	0.000000e+00	POS
612	DOCK2|ENSG00000134516	Stromal_score	8.970288e−09	0.4749781	2.051415e−04	POS	NR2F1−AS1|ENSG00000237187	Stromal_score	7.020138e−10	0.5037557	1.605435e−05	POS
613	ROBO1|ENSG00000169855	Stromal_score	1.836437e−07	0.4349137	4.199747e−03	POS	HTR2A|ENSG00000102468	Stromal_score	1.933195e−09	0.4930202	4.421023e−05	POS
614	CMTM8|ENSG00000170293	Stromal_score	5.319739e−08	−0.4520291	1.216571e−03	NEG	ZNF77|ENSG00000175691	Stromal_score	6.086564e−07	−0.4174422	1.391936e−02	NEG
615	IL6ST|ENSG00000134352	Stromal_score	0.000000e+00	0.5343918	0.000000e+00	POS	MDGA1|ENSG00000112139	Stromal_score	1.663046e−07	0.4363184	3.803220e−03	POS
616	HAVCR2|ENSG00000135077	Stromal_score	1.804170e−10	0.5160082	4.125957e−06	POS	EVC2|ENSG00000173040	Stromal_score	4.569074e−10	0.5079358	1.044902e−05	POS
617	TSHZ2|ENSG00000182463	Stromal_score	8.536763e−09	0.4755877	1.952272e−04	POS	TRPC6|ENSG00000137672	Stromal_score	3.796489e−07	0.4244366	8.682191e−03	POS
618	ITGAV|ENSG00000138448	Stromal_score	5.106720e−10	0.5068774	1.167856e−05	POS	LINC01638|ENSG00000233521	Stromal_score	1.928180e−08	0.4653546	4.409555e−04	POS
619	COLEC12|ENSG00000158270	Stromal_score	0.000000e+00	0.5731929	0.000000e+00	POS	TRO|ENSG00000067445	Stromal_score	3.531654e−07	0.4254951	8.076540e−03	POS
620	ADGRL4|ENSG00000162618	Stromal_score	0.000000e+00	0.6086089	0.000000e+00	POS	CASS4|ENSG00000087589	Stromal_score	7.536018e−08	0.4473125	1.723412e−03	POS
621	LAIR1|ENSG00000167613	Stromal_score	0.000000e+00	0.5835528	0.000000e+00	POS	ZNF16|ENSG00000170631	Stromal_score	4.017106e−07	−0.4236075	9.186720e−03	NEG
622	GLT8D2|ENSG00000120820	Stromal_score	0.000000e+00	0.6748805	0.000000e+00	POS	SEPT4|ENSG00000108387	Stromal_score	0.000000e+00	0.5687933	0.000000e+00	POS
623	LINC00578|ENSG00000228221	Stromal_score	1.863763e−07	0.4347039	4.262239e−03	POS	HSPB2|ENSG00000170276	Stromal_score	1.703464e−10	0.5164569	3.895651e−06	POS
624	EVI2A|ENSG00000126860	Stromal_score	0.000000e+00	0.6175300	0.000000e+00	POS	SOX5|ENSG00000134532	Stromal_score	1.796473e−07	0.4352258	4.108355e−03	POS
625	FBXL7|ENSG00000183580	Stromal_score	0.000000e+00	0.6573066	0.000000e+00	POS	PCDHGB6|ENSG00000253305	Stromal_score	1.206255e−06	0.4070335	2.758585e−02	POS
626	DOK3|ENSG00000146094	Stromal_score	1.777337e−06	0.4009853	4.064593e−02	POS	PABPC4L|ENSG00000254535	Stromal_score	1.014019e−07	0.4432348	2.318960e−03	POS
627	SYDE1|ENSG00000105137	Stromal_score	0.000000e+00	0.6801385	0.000000e+00	POS	GUCY1A2|ENSG00000152402	Stromal_score	4.187080e−07	0.4229978	9.575432e−03	POS
628	PIK3CG|ENSG00000105851	Stromal_score	1.050107e−06	0.4091698	2.401489e−02	POS	MS4A14|ENSG00000166928	Stromal_score	1.163484e−07	0.4413277	2.660771e−03	POS
629	SIGLEC14|ENSG00000254415	Stromal_score	3.563618e−07	0.4253634	8.149638e−03	POS	KCNK13|ENSG00000152315	Stromal_score	1.707181e−08	0.4669105	3.904153e−04	POS
630	PDE4B|ENSG00000184588	Stromal_score	0.000000e+00	0.5346747	0.000000e+00	POS	GPR141|ENSG00000187037	Stromal_score	3.543760e−08	0.4574383	8.104224e−04	POS
631	PIK3IP1|ENSG00000100100	Stromal_score	1.308085e−07	0.4396937	2.991460e−03	POS	PTH1R|ENSG00000160801	Stromal_score	3.334372e−07	0.4263340	7.625375e−03	POS
632	TGFB2|ENSG00000092969	Stromal_score	1.280257e−06	0.4061116	2.927821e−02	POS	AP000473.1|ENSG00000228798	Stromal_score	1.532444e−06	0.4033119	3.504547e−02	POS
633	MEF2C|ENSG00000081189	Stromal_score	0.000000e+00	0.6583748	0.000000e+00	POS	AL157394.1|ENSG00000261438	Stromal_score	2.005639e−07	0.4336601	4.586697e−03	POS
634	TNFSF12|ENSG00000239697	Stromal_score	0.000000e+00	0.5806653	0.000000e+00	POS	LINC00924|ENSG00000259134	Stromal_score	1.701310e−08	0.4669544	3.890726e−04	POS
635	DLC1|ENSG00000164741	Stromal_score	2.096931e−09	0.4921130	4.795472e−05	POS	SPRYD4|ENSG00000176422	Stromal_score	9.127599e−07	−0.4113160	2.087391e−02	NEG
636	ITGAM|ENSG00000169896	Stromal_score	4.139403e−09	0.4842991	9.466401e−05	POS	AL035409.1|ENSG00000230498	Stromal_score	6.724514e−08	0.4488635	1.537829e−03	POS
637	EFS|ENSG00000100842	Stromal_score	9.653368e−10	0.5005024	2.207629e−05	POS	CAMK4|ENSG00000152495	Stromal_score	2.741051e−07	0.4291776	6.268511e−03	POS
638	PCDH18|ENSG00000189184	Stromal_score	2.277173e−08	0.4632133	5.207666e−04	POS	TXLNB|ENSG00000164440	Stromal_score	3.513748e−08	0.4575505	8.035589e−04	POS
639	DYSF|ENSG00000135636	Stromal_score	1.072099e−09	0.4994049	2.451784e−05	POS	AC120049.1|ENSG00000267414	Stromal_score	5.006833e−07	0.4203541	1.145013e−02	POS
640	MDFIC|ENSG00000135272	Stromal_score	0.000000e+00	0.6407229	0.000000e+00	POS	NOVA2|ENSG00000104967	Stromal_score	4.261203e−07	0.4227392	9.744945e−03	POS
641	CLEC2B|ENSG00000110852	Stromal_score	0.000000e+00	0.6220710	0.000000e+00	POS	ABCA6|ENSG00000154262	Stromal_score	5.081029e−11	0.5242903	1.161981e−06	POS
642	ARHGEF6|ENSG00000129675	Stromal_score	0.000000e+00	0.6038143	0.000000e+00	POS	NAV3|ENSG00000067798	Stromal_score	0.000000e+00	0.5902058	0.000000e+00	POS
643	ARHGAP25|ENSG00000163219	Stromal_score	2.427838e−08	0.4623842	5.552224e−04	POS	GIMAP5|ENSG00000196329	Stromal_score	1.837172e−06	0.4004634	4.201429e−02	POS
644	LRRN4CL|ENSG00000177363	Stromal_score	1.419609e−07	0.4385475	3.246505e−03	POS	AL356417.2|ENSG00000233682	Stromal_score	2.245582e−08	0.4633938	5.135422e−04	POS
645	GAPT|ENSG00000175857	Stromal_score	1.236136e−07	0.4404839	2.826919e−03	POS	ZNF835|ENSG00000127903	Stromal_score	1.719026e−07	0.4358502	3.931241e−03	POS
646	TCN2|ENSG00000185339	Stromal_score	2.798053e−07	0.4288801	6.398866e−03	POS	C14orf37|ENSG00000139971	Stromal_score	4.721899e−09	0.4827431	1.079851e−04	POS
647	HOOK1|ENSG00000134709	Stromal_score	8.640286e−10	−0.5016486	1.975947e−05	NEG	INTS6|ENSG00000102786	Stromal_score	1.700262e−06	−0.4016828	3.888329e−02	NEG
648	BIN2|ENSG00000110934	Stromal_score	2.935647e−08	0.4599112	6.713531e−04	POS	TLL1|ENSG00000038295	Stromal_score	6.423505e−09	0.4790557	1.468991e−04	POS
649	CD180|ENSG00000134061	Stromal_score	2.517067e−08	0.4619159	5.756280e−04	POS	ZNF385D|ENSG00000151789	Stromal_score	5.156995e−07	0.4199151	1.179353e−02	POS
650	EVL|ENSG00000196405	Stromal_score	1.626086e−08	0.4675300	3.718695e−04	POS	NUDT10|ENSG00000122824	Stromal_score	3.639278e−07	0.4250561	8.322665e−03	POS
651	GJC1|ENSG00000182963	Stromal_score	2.057300e−06	0.3986733	4.704840e−02	POS	TMCC2|ENSG00000133069	Stromal_score	9.372828e−07	0.4109111	2.143472e−02	POS
652	ESPL1|ENSG00000135476	Stromal_score	4.485238e−07	−0.4219832	1.025729e−02	NEG	AL135818.1|ENSG00000258875	Stromal_score	4.634170e−07	0.4215003	1.059788e−02	POS
653	CD86|ENSG00000114013	Stromal_score	0.000000e+00	0.5782753	0.000000e+00	POS	PRDM6|ENSG00000061455	Stromal_score	0.000000e+00	0.5457711	0.000000e+00	POS
654	TMEM173|ENSG00000184584	Stromal_score	2.083270e−09	0.4921861	4.764230e−05	POS	AC083964.1|ENSG00000272293	Stromal_score	4.645915e−08	0.4538435	1.062474e−03	POS
655	RNF144B|ENSG00000137393	Stromal_score	1.264642e−06	0.4063018	2.892109e−02	POS	AC106786.1|ENSG00000223652	Stromal_score	1.578505e−07	0.4370549	3.609884e−03	POS
656	FGF2|ENSG00000138685	Stromal_score	3.274637e−07	0.4265974	7.488768e−03	POS	AC092645.1|ENSG00000278962	Stromal_score	5.145793e−10	0.5068042	1.176791e−05	POS
657	GNB4|ENSG00000114450	Stromal_score	0.000000e+00	0.6796313	0.000000e+00	POS	AP005019.1|ENSG00000256633	Stromal_score	3.323143e−09	0.4868647	7.599696e−05	POS
658	PKD2|ENSG00000118762	Stromal_score	0.000000e+00	0.5762023	0.000000e+00	POS	RFTN2|ENSG00000162944	Stromal_score	1.994002e−07	0.4337430	4.560082e−03	POS
659	RGL1|ENSG00000143344	Stromal_score	3.792561e−10	0.5096673	8.673207e−06	POS	SENCR|ENSG00000254703	Stromal_score	1.662309e−09	0.4946883	3.801534e−05	POS
660	CMKLR1|ENSG00000174600	Stromal_score	0.000000e+00	0.5452346	0.000000e+00	POS	FBLN7|ENSG00000144152	Stromal_score	8.611599e−09	0.4754804	1.969387e−04	POS
661	P2RY13|ENSG00000181631	Stromal_score	2.323007e−09	0.4909619	5.312486e−05	POS	ANKDD1A|ENSG00000166839	Stromal_score	1.325714e−06	0.4055702	3.031776e−02	POS
662	LSAMP|ENSG00000185565	Stromal_score	4.510340e−10	0.5080578	1.031470e−05	POS	AL445426.1|ENSG00000231246	Stromal_score	4.643346e−07	0.4214711	1.061887e−02	POS
663	DRAM1|ENSG00000136048	Stromal_score	8.635540e−09	0.4754463	1.974862e−04	POS	ARHGAP22|ENSG00000128805	Stromal_score	0.000000e+00	0.5477514	0.000000e+00	POS
664	EFHD1|ENSG00000115468	Stromal_score	5.249146e−07	0.4196517	1.200427e−02	POS	SHC4|ENSG00000185634	Stromal_score	5.798761e−08	0.4508682	1.326119e−03	POS
665	C1orf53|ENSG00000203724	Stromal_score	1.648265e−06	−0.4021705	3.769418e−02	NEG	PRPH2|ENSG00000112619	Stromal_score	1.466442e−06	0.4039996	3.353605e−02	POS
666	KDR|ENSG00000128052	Stromal_score	3.863997e−07	0.4241781	8.836575e−03	POS	NTNG2|ENSG00000196358	Stromal_score	0.000000e+00	0.5369525	0.000000e+00	POS
667	BDH1|ENSG00000161267	Stromal_score	9.681850e−08	−0.4438738	2.214142e−03	NEG	TCTE3|ENSG00000184786	Stromal_score	2.629471e−07	−0.4297776	6.013337e−03	NEG
668	NCF1|ENSG00000158517	Stromal_score	9.369837e−07	0.4109160	2.142788e−02	POS	GPR162|ENSG00000250510	Stromal_score	3.616523e−08	0.4571700	8.270627e−04	POS
669	ITGA4|ENSG00000115232	Stromal_score	1.979157e−10	0.5152717	4.526134e−06	POS	FGF1|ENSG00000113578	Stromal_score	2.517969e−07	0.4304019	5.758343e−03	POS
670	CSF1|ENSG00000184371	Stromal_score	1.467142e−10	0.5175934	3.355207e−06	POS	HINFP|ENSG00000172273	Stromal_score	2.119546e−06	−0.3982002	4.847189e−02	NEG

Supporting Material 5E: M102 hub genes correlating to immune cell abundances

	gene	cell type	estimate.rho	p.value	adj.p.value	gene	cell type	estimate.rho	p.value	adj.p.value
1	CD8A	T.cells.CD8	0.75899282803757395	3.983590e−42	2.821975e−38	NKG7	T.cells.gamma.delta	0.41768344675271002	1.294624e−10	9.171115e−07
2	KLRK1	T.cells.CD8	0.72465323573000295	8.772582e−37	6.214497e−33	TCRA	Macrophages.M1	0.41365537697683502	2.023130e−10	1.433185e−06
3	GZMH	T.cells.CD8	0.70478666678532897	4.777558e−34	3.384422e−30	TRIM22	Macrophages.M1	0.411273738654925	2.626950e−10	1.860931e−06
4	NKG7	T.cells.CD8	0.65087125767485798	1.184451e−27	8.390652e−24	TRIM22	T.cells.CD8	0.406033563161175	4.633407e−10	3.282306e−06
5	FASLG	T.cells.CD8	0.64025924250188104	1.522171e−26	1.078306e−22	CD247	Macrophages.M1	0.401062596254816	7.866259e−10	5.572458e−06
6	FASLG	Macrophages.M1	0.61589830548769098	3.718837e−24	2.634424e−20	CD3E	T.cells.CD4.memory.activated	0.40000474318817197	8.794236e−10	6.229837e−06
7	TBX21	Macrophages.M1	0.58249601532516104	3.353293e−21	2.375473e−17	PYHIN1	Macrophages.M1	0.399606960607163	9.169910e−10	6.495964e−06
8	SH2D1A	T.cells.CD8	0.57670170314467395	1.009539e−20	7.151571e−17	TRIM22	Macrophages.M0	−0.38987799887693397	2.507728e−09	1.776475e−05
9	GZMH	Macrophages.M1	0.57570148266338395	1.218406e−20	8.631189e−17	CD3E	T.cells.gamma.delta	0.38349614631659701	4.767139e−09	3.377041e−05
10	CD244	T.cells.CD8	0.57278567848837703	2.100327e−20	1.487871e−16	CD3E	Macrophages.M1	0.38024747714805301	6.576423e−09	4.658738e−05
11	CD96	T.cells.CD8	0.57208453930540104	2.392243e−20	1.694665e−16	TCRBV3S1	T.cells.CD4.memory.activated	0.37692472967076701	9.106129e−09	6.450782e−05
12	CCR5	T.cells.CD8	0.56759448452436001	5.464762e−20	3.871237e−16	CD3E	Macrophages.M0	−0.37646471842796803	9.523075e−09	6.746146e−05
13	PYHIN1	T.cells.CD8	0.56237827930338802	1.404633e−19	9.950417e−16	FASLG	T.cells.CD4.memory.resting	−0.37634048361287598	9.638802e−09	6.828127e−05
14	TBX21	T.cells.CD8	0.55358729964050801	6.643918e−19	4.706552e−15	GZMH	T.cells.gamma.delta	0.37622921558320699	9.743602e−09	6.902368e−05
15	TCRA	T.cells.CD8	0.54719089731836401	2.000348e−18	1.417046e−14	SH2D1A	T.cells.gamma.delta	0.37217732914151602	1.440604e−08	1.020524e−04
16	SIRPG	T.cells.CD8	0.54705748644250396	2.046355e−18	1.449638e−14	TCRBV3S1	T.cells.gamma.delta	0.37159045293262	1.523874e−08	1.079512e−04
17	CD3E	T.cells.CD8	0.54162350494315303	5.122835e−18	3.629017e−14	CD2	T.cells.gamma.delta	0.36825401616657699	2.093004e−08	1.482684e−04
18	TBX21	T.cells.CD4.memory.activated	0.54097487108187803	5.709548e−18	4.044644e−14	TCRBV3S1	Macrophages.M0	−0.36610882344086798	2.561888e−08	1.814841e−04
19	CD2	T.cells.CD8	0.54094806778885496	5.735159e−18	4.062787e−14	PYHIN1	Macrophages.M0	−0.36107171333716898	4.094376e−08	2.900456e−04
20	FASLG	T.cells.CD4.memory.activated	0.53712080706523402	1.082249e−17	7.666652e−14	NKG7	Macrophages.M0	−0.35813324819559	5.362451e−08	3.798760e−04
21	AV1S4A1N1T	T.cells.CD8	0.53222971758203297	2.408301e−17	1.706041e−13	CD2	Macrophages.M0	−0.35757219991567402	5.644191e−08	3.998345e−04
22	CD244	T.cells.CD4.memory.activated	0.52866734971986995	4.277698e−17	3.030322e−13	CD96	Macrophages.M0	−0.35630263167829301	6.335277e−08	4.487910e−04
23	UBASH3A	T.cells.CD8	0.52073708591719903	1.500416e−16	1.062894e−12	TCRBV3S1	Macrophages.M1	0.348113340968701	1.318598e−07	9.340946e−04
24	TCRBV3S1	T.cells.CD8	0.52043781156399704	1.572178e−16	1.113731e−12	PYHIN1	T.cells.gamma.delta	0.345313156479919	1.686190e−07	1.194497e−03
25	GZMH	T.cells.CD4.memory.activated	0.51527043420120999	3.497120e−16	2.477360e−12	KLRK1	T.cells.gamma.delta	0.34501835458466801	1.730172e−07	1.225654e−03
26	CCR5	Macrophages.M1	0.51080250503240199	6.905780e−16	4.892055e−12	SIRPG	T.cells.follicular.helper	0.34175859228553201	2.296030e−07	1.626508e−03
27	CD96	T.cells.CD4.memory.activated	0.51061136741437096	7.108174e−16	5.035431e−12	CD8A	T.cells.gamma.delta	0.34103923654592999	2.442913e−07	1.730560e−03
28	NKG7	Macrophages.M1	0.50785840987959496	1.075425e−15	7.618313e−12	TRIM22	T.cells.regulatory..Tregs.	−0.33580648496911503	3.817439e−07	2.704274e−03
29	CD247	T.cells.CD8	0.50333487887805595	2.106500e−15	1.492245e−11	GZMH	Macrophages.M0	−0.333553555714654	4.614713e−07	3.269063e−03
30	TCRA	T.cells.CD4.memory.activated	0.49304386624003399	9.374883e−15	6.641167e−11	CD96	T.cells.gamma.delta	0.33260232012449298	4.997212e−07	3.540025e−03
31	SIRPG	T.cells.CD4.memory.activated	0.49182027797369099	1.115893e−14	7.904988e−11	SH2D1A	Macrophages.M0	−0.33095685417387599	5.731597e−07	4.060264e−03
32	CCR5	T.cells.CD4.memory.activated	0.49097243757921	1.258547e−14	8.915550e−11	CCR5	T.cells.regulatory..Tregs.	−0.32820159374685298	7.197975e−07	5.099045e−03
33	AV1S4A1N1T	T.cells.CD4.memory.activated	0.48750997525619799	2.049968e−14	1.452198e−10	SIRPG	T.cells.gamma.delta	0.32644302714914603	8.314778e−07	5.890189e−03
34	NKG7	T.cells.CD4.memory.activated	0.48703773298692699	2.190081e−14	1.551454e−10	TCRA	T.cells.follicular.helper	0.325532703189165	8.956171e−07	6.344552e−03
35	CD8A	T.cells.CD4.memory.activated	0.48077585470497802	5.212532e−14	3.692557e−10	TRIM22	T.cells.CD4.memory.activated	0.32307223334621599	1.093513e−06	7.746449e−03
36	SIRPG	Macrophages.M1	0.47753199468560797	8.112180e−14	5.746668e−10	TCRA	T.cells.gamma.delta	0.32060855968350199	1.333139e−06	9.443957e−03
37	KLRK1	T.cells.CD4.memory.activated	0.470480554903832	2.088192e−13	1.479275e−09	CD247	T.cells.gamma.delta	0.32031591255939501	1.364729e−06	9.667742e−03
38	SH2D1A	T.cells.CD4.memory.activated	0.47012672374854098	2.188408e−13	1.550268e−09	TBX21	T.cells.follicular.helper	0.31857110311741799	1.568433e−06	1.111078e−02
39	CD244	Macrophages.M1	0.46955763302671799	2.359512e−13	1.671478e−09	CCR5	T.cells.gamma.delta	0.31645926754868797	1.853905e−06	1.313306e−02
40	CD8A	Macrophages.M1	0.46635734241684201	3.593801e−13	2.545849e−09	CD244	T.cells.follicular.helper	0.31438848684192899	2.181526e−06	1.545393e−02
41	UBASH3A	T.cells.CD4.memory.activated	0.46462749359594702	4.503361e−13	3.190181e−09	CD8A	T.cells.follicular.helper	0.31323070297612599	2.388047e−06	1.691693e−02
42	KLRK1	Macrophages.M1	0.46384214265961299	4.986999e−13	3.532790e−09	FASLG	T.cells.gamma.delta	0.31300207032384503	2.430976e−06	1.722104e−02
43	CD247	T.cells.CD4.memory.activated	0.462122007232804	6.229878e−13	4.413246e−09	UBASH3A	T.cells.gamma.delta	0.312450703325028	2.537550e−06	1.797600e−02
44	PYHIN1	T.cells.CD4.memory.activated	0.45972711656980098	8.474836e−13	6.003574e−09	KLRK1	T.cells.follicular.helper	0.31165465896517702	2.699303e−06	1.912186e−02
45	UBASH3A	Macrophages.M1	0.45487611791696603	1.569208e−12	1.111627e−08	CD244	T.cells.CD4.memory.resting	−0.31105032291689599	2.828613e−06	2.003790e−02
46	CD96	Macrophages.M1	0.45183798179558199	2.296704e−12	1.626985e−08	UBASH3A	T.cells.follicular.helper	0.30974281948823001	3.128873e−06	2.216493e−02
47	CD2	T.cells.CD4.memory.activated	0.44584663874682401	4.814718e−12	3.410746e−08	GZMH	T.cells.follicular.helper	0.309368662607928	3.220234e−06	2.281213e−02
48	FASLG	T.cells.follicular.helper	0.44337874480336997	6.503855e−12	4.607331e−08	TRIM22	Mast.cells.activated	−0.30849420441623698	3.443767e−06	2.439565e−02
49	SH2D1A	Macrophages.M1	0.43374046662648003	2.057142e−11	1.457279e−07	NKG7	T.cells.follicular.helper	0.30816510533661401	3.531659e−06	2.501827e−02
50	CD2	Macrophages.M1	0.42924150358600599	3.478225e−11	2.463975e−07	TCRA	Macrophages.M0	−0.30295557682197399	5.241835e−06	3.713316e−02
51	AV1S4A1N1T	Macrophages.M1	0.42913224415433099	3.522535e−11	2.495364e−07	KLRK1	T.cells.regulatory..Tregs.	−0.30195883007500901	5.648378e−06	4.001311e−02
52	CD8A	Macrophages.M0	−0.42607659527269798	5.009720e−11	3.548885e−07	NKG7	NK.cells.activated	0.30152532589874098	5.834390e−06	4.133082e−02
53	KLRK1	Macrophages.M0	−0.41958004932915199	1.047036e−10	7.417201e−07	GZMH	T.cells.CD4.memory.resting	−0.300894817512743	6.115356e−06	4.332118e−02

Supporting Material 6A: DEG between EBV subtype and rest in TCGA−GCC

	Gene	logFC	AveExpr	t	P.Value	adj.P.Val	B	Gene	logFC	AveExpr	t	P.Value	adj.P.Val	B	Gene	logFC	AveExpr	t	P.Value	adj.P.Val	B	Gene	logFC	AveExpr	t	P.Value	adj.P.Val	B
1	CLDN3	−4.47209501	5.71146299	−10.915433	8.542404e−22	2.267069e−17	38.48389	GPR113	0.14920039	0.361339973	4.132303	5.405022e−05	0.001360670	1.551910	SNORD36B	0.33874401	1.083812935	3.438905	0.0007194648	0.009062114	−0.8348539	NRTN	−0.52909624	1.840822052	−3.006825	0.003001244	0.02520271	−2.127317
2	CAMK2N2	1.87505869	1.16060466	10.624172	6.036205e−21	8.009742e−17	36.60969	SPN	0.66422779	1.792554830	4.132117	5.409048e−05	0.001360670	1.551218	FLJ38109	0.21895405	0.733885866	3.438517	0.0007204340	0.009069367	−0.8360822	ITGA4	0.44954443	2.338095750	3.006762	0.003001837	0.02520271	−2.127494
3	APOBEC3H	0.96502799	0.80887202	9.400439	1.910791e−17	1.690350e−13	28.87881	FAM50B	−0.76429252	2.604620406	−4.131737	5.417234e−05	0.001361439	1.549812	RGN	−0.63966692	1.114670436	−3.438309	0.0007209553	0.009069367	−0.8367422	C18orf56	0.48050167	2.602389488	3.006527	0.003004052	0.02521333	−2.128155
4	ZNF683	1.76150173	1.29482577	9.310303	3.418003e−17	2.267759e−13	28.32085	CCDC134	0.40059099	2.283033229	4.131496	5.422446e−05	0.001361460	1.548917	C1orf185	0.01528984	0.005617097	3.438265	0.0007210658	0.009069367	−0.8368820	ZNF273	0.19442821	0.926671525	3.006334	0.003005866	0.02522057	−2.128696
5	C15orf53	0.13633412	0.06567045	9.261845	4.668778e−17	2.478094e−13	28.02166	MID2	−0.51844774	1.567515447	−4.130741	5.438798e−05	0.001364275	1.546117	PION	0.36937256	1.874322873	3.437364	0.0007233252	0.009093476	−0.8397365	MCCC2	0.31068052	4.342752924	3.005924	0.003009738	0.02524275	−2.129850
6	CXCR2P1	1.33296099	0.83636583	9.157566	9.115780e−17	4.032061e−13	27.37968	LOC100130238	0.21473873	0.188261284	4.129206	5.472160e−05	0.001370183	1.540431	PYROXD1	−0.38467061	3.198095139	−3.437190	0.0007237625	0.009094666	−0.8402879	LYN	0.43462283	4.489330017	3.005852	0.003010411	0.02524275	−2.130050
7	FCRL6	0.80256333	0.56571593	8.862699	5.955588e−16	2.257934e−12	25.57893	ZNF45	0.31808448	1.819535868	4.129183	5.472678e−05	0.001370183	1.540343	RFC4	0.41331582	3.244977093	3.436087	0.0007265385	0.009125227	−0.8437805	MGAM	0.41445985	0.586461043	3.005593	0.003012858	0.02525506	−2.130779
8	C19orf63	−1.10508817	6.19057754	−8.840935	6.834193e−16	2.267158e−12	25.44692	DQ575954	0.09409023	0.020038866	4.126521	5.531026e−05	0.001383486	1.530483	AX746823	0.32500283	1.015875955	3.435661	0.0007276133	0.009134404	−0.8451292	FZD9	−0.23597986	0.303010288	−3.005495	0.003013782	0.02525506	−2.131053
9	MLYCD	−0.73174710	2.61770401	−8.810222	8.297106e−16	2.446632e−12	25.26083	MYO1C	−0.46158846	5.695765995	−4.125701	5.549120e−05	0.001386705	1.527446	ALS2CL	−0.53285324	2.001475067	−3.434525	0.0007304858	0.009166129	−0.8487237	FAM90A1	−0.26181108	0.448892512	−3.003869	0.003029189	0.02537615	−2.135623
10	IFNG	0.96939685	0.56849252	8.686574	1.806731e−15	4.794885e−12	24.51429	SLC16A4	−0.64617355	1.807771637	−4.125056	5.563390e−05	0.001387554	1.525059	ATP6V0E2	−0.55526089	2.958181585	−3.434346	0.0007309398	0.009167491	−0.8492904	SUGT1P1	0.21074541	0.465346622	3.003723	0.003030572	0.02537973	−2.136032
11	ATP2C1	0.72376484	3.90068104	8.658538	2.154063e−15	4.933707e−12	24.34561	CXCR3	0.84397849	2.231812756	4.124995	5.564735e−05	0.001387554	1.524834	LOC100133991	0.22359085	1.320211133	3.433935	0.0007319829	0.009174339	−0.8505914	RAB11FIP4	0.36811747	2.807733715	3.003279	0.003034789	0.02540703	−2.137278
12	FASLG	0.90467729	0.75428138	8.652949	2.230848e−15	4.933707e−12	24.31201	KLK1	−1.30621440	2.035911772	−4.124839	5.568203e−05	0.001387554	1.524255	ARPC1B	−0.50406487	7.130216320	−3.433859	0.0007321772	0.009174339	−0.8508335	ADAMTS9	−0.38956532	1.793128638	−3.002759	0.003039741	0.02543405	−2.138739
13	C1orf115	−1.60878152	2.95750960	−8.544312	4.398955e−15	8.980297e−12	23.66071	IL2	0.09473981	0.102508006	4.122970	5.609791e−05	0.001396607	1.517336	DBF4	0.39584838	2.394689914	3.432834	0.0007347851	0.009200525	−0.8540771	CCNC	0.26529450	3.773730779	3.002739	0.003039934	0.02543405	−2.138796
14	C17orf66	0.11184177	0.07966878	8.399744	1.079795e−14	2.046905e−11	22.79942	C11orf74	−0.36284067	1.612866368	−4.121362	5.645798e−05	0.001404253	1.511389	TRAF3IP3	0.52353332	1.650187099	3.432707	0.0007351085	0.009200525	−0.8544784	HOXA1	−0.33054524	0.921818288	−3.001830	0.003048596	0.02549849	−2.141346
15	CRAT	−1.44233589	4.28852028	−8.286866	2.167037e−14	3.834066e−11	22.13138	TMEM120B	−0.58354666	3.074600954	−4.120063	5.675063e−05	0.001410211	1.506582	PPP1R8	0.19192626	3.785808849	3.432608	0.0007353601	0.009200525	−0.8547906	ZHX2	−0.37589409	2.870112836	−3.000876	0.003057723	0.02556677	−2.144024
16	TRIAP1	0.68042450	4.13183903	8.254799	2.639259e−14	4.330862e−11	21.94234	PBX4	0.39635975	1.180617166	4.118959	5.700036e−05	0.001415091	1.502500	CRISP3	0.77576300	0.456879805	3.432493	0.0007356537	0.009200525	−0.8551548	ARHGAP15	0.51327889	1.986589009	3.000404	0.003062247	0.02559653	−2.145349
17	ECHDC2	−0.97428783	2.62962780	−8.246679	2.774206e−14	4.330862e−11	21.89452	THNSL2	−0.62778050	1.294488587	−4.114676	5.797949e−05	0.001438054	1.486668	STAT2	0.37778757	4.156866666	3.431375	0.0007385116	0.009230776	−0.8586916	KARS	0.23413094	5.678307745	2.999460	0.003071307	0.02566417	−2.147996
18	SLC6A8	−1.91096024	3.86311339	−8.170682	4.419484e−14	6.516038e−11	21.44802	MXRA7	−0.84207320	4.101031184	−4.113790	5.818407e−05	0.001441780	1.483395	BC045791	0.03003283	0.011105563	3.431275	0.0007387682	0.009230776	−0.8590085	SEMA4C	−0.35187768	2.957405585	−2.998184	0.003083598	0.02575877	−2.151574
19	CXCL10	2.60177789	3.98717905	8.090651	7.201627e−14	9.907559e−11	20.97987	AASDH	0.21209625	1.282307070	4.110987	5.883575e−05	0.001456569	1.473042	HIST1H1B	0.42677396	0.944755785	3.431055	0.0007393314	0.009233467	−0.8597037	ERLIN2	−0.32755550	3.325426387	−2.997591	0.003089331	0.02579854	−2.153238
20	GZMH	1.63539100	2.06456003	8.084718	7.466415e−14	9.907559e−11	20.94526	ZNF227	0.34445179	2.156834378	4.109649	5.914927e−05	0.001462966	1.468103	AFG3L2	−0.37761554	4.583005095	−3.430731	0.0007401634	0.009239510	−0.8607296	MSX1	−0.49377080	1.922053909	−2.997485	0.003090354	0.02579897	−2.153535
21	ITGAE	0.89073904	2.21048092	8.053036	9.052550e−14	1.144027e−10	20.76058	MIR4632	0.49813015	1.797416828	4.109141	5.926854e−05	0.001463852	1.466230	RPL39L	−0.84288198	2.992146279	−3.430453	0.0007408758	0.009239866	−0.8616070	AK309441	−0.65105105	3.841700824	−2.997373	0.003091431	0.02579984	−2.153847
22	AK090973	0.36377417	0.25769165	8.013484	1.150792e−13	1.388221e−10	20.53052	CD300A	0.54844657	2.134447297	4.109027	5.929543e−05	0.001463852	1.465809	SLC35F2	0.49802562	2.335496100	3.430448	0.0007408883	0.009239866	−0.8616224	DQ576627	0.18523622	0.574101512	2.996999	0.003095052	0.02581532	−2.154895
23	CXCL11	2.35856552	2.51762304	7.989004	1.334718e−13	1.540091e−10	20.38839	FOXO3	−0.42040854	3.538449375	−4.108398	5.944372e−05	0.001466150	1.463487	DMKN	0.87552476	1.977421741	3.429216	0.0007440592	0.009275053	−0.8655180	MESP1	−0.53190725	1.238837622	−2.996981	0.003095232	0.02581532	−2.154947
24	SCNN1A	−2.37831159	4.20089108	−7.939401	1.801280e−13	1.991841e−10	20.10105	IFITM10	0.58296747	1.586116218	4.107068	5.975849e−05	0.001472545	1.458579	AK095633	0.06090556	0.055014505	3.428596	0.0007456599	0.009290642	−0.8674780	FLJ22184	−0.64430028	0.955438142	−2.996500	0.003099891	0.02584606	−2.156295
25	CD244	0.58911195	0.57292945	7.922843	1.990471e−13	2.113005e−10	20.00532	PRRX2	−0.83887853	2.504521445	−4.106580	5.987435e−05	0.001472955	1.456779	NCAPD3	0.42980882	3.067089652	3.426485	0.0007511336	0.009354451	−0.8741488	SLC7A9	−0.68484159	0.992593352	−2.996278	0.003102043	0.02585588	−2.156917
26	HSD17B12	−1.03515879	4.94744287	−7.868535	2.760074e−13	2.817293e−10	19.69203	PDLIM4	−0.78753774	2.951111811	−4.106530	5.988616e−05	0.001472955	1.456596	ZNF259	0.29057986	4.296979832	3.426083	0.0007521806	0.009363096	−0.8754191	RPS3A	0.32523772	4.613551374	2.995975	0.003104989	0.02587231	−2.157767
27	DNAJA4	−1.10900538	3.21026198	−7.856252	2.971418e−13	2.920684e−10	19.62132	C1orf88	−0.65871516	0.893428760	−4.105421	6.015019e−05	0.001478080	1.452507	LOC100335030	0.08046536	0.181375752	3.425835	0.0007528257	0.009366733	−0.8762009	AK128361	−0.31426550	1.556596762	−2.995473	0.003109868	0.02589902	−2.159173
28	C10orf131	0.19833019	0.23914227	7.840020	3.275479e−13	3.065068e−10	19.52795	CMKLR1	0.56859972	1.799792482	4.105166	6.021108e−05	0.001478207	1.451567	PRPF40B	−0.28725441	2.379054734	−3.425580	0.0007534915	0.009370623	−0.8770071	GAL	−1.04619704	2.220147982	−2.995410	0.003110473	0.02589902	−2.159348
29	ACPL2	−0.83651948	2.47852178	−7.836305	3.349296e−13	3.065068e−10	19.50659	TRAV12−1	0.53187372	0.877775310	4.103169	6.069006e−05	0.001487094	1.444204	RPL13AP6	0.13804654	0.291415064	3.425394	0.0007539768	0.009372267	−0.8775944	BC044946	0.03152302	0.004486194	2.995344	0.003111122	0.02589902	−2.159534
30	CLEC9A	0.32457684	0.25802034	7.818055	3.736469e−13	3.305405e−10	19.40176	ERP29	0.34960968	6.334140327	4.103114	6.070336e−05	0.001487094	1.444000	NUB1	0.32225543	3.901383822	3.424463	0.0007564118	0.009394923	−0.8805349	PRKAB1	−0.32335020	4.281026799	−2.994247	0.003121812	0.02597986	−2.162607
31	ATP2A1	0.44315500	0.60162647	7.770136	4.976850e−13	4.260666e−10	19.12708	CA9	−1.60816471	3.290595079	−4.102957	6.074119e−05	0.001487094	1.443421	FAM18B2	0.23724253	1.129095349	3.424411	0.0007565475	0.009394923	−0.8806985	ABCC4	0.43783244	2.177551346	2.994138	0.003122869	0.02598051	−2.162910
32	LOC79015	0.74992618	0.42019323	7.701963	7.471843e−13	6.196726e−10	18.73774	CD33	0.29806743	0.545152000	4.102441	6.086549e−05	0.001488764	1.441521	SCIMP	0.45273668	1.320364295	3.424260	0.0007569422	0.009394923	−0.8811742	MAFK	−0.32989190	3.970427277	−2.993572	0.003128403	0.02601839	−2.164496
33	LOC284215	1.24202898	0.44625848	7.660517	9.557489e−13	7.686248e−10	18.50189	ESR2	0.12889446	0.257471558	4.100245	6.139785e−05	0.001499458	1.433429	PPP1R11	−0.26551507	5.817077874	−3.424156	0.0007572154	0.009394923	−0.8815032	POU2F3	0.44666827	0.876252359	2.992681	0.003137123	0.02607541	−2.166989
34	DDB2	0.91287620	3.00503146	7.639909	1.079940e−12	8.429563e−10	18.38486	PIGM	−0.37790292	2.703073093	−4.100172	6.141569e−05	0.001499458	1.433159	DTX2	0.46136360	3.856794366	3.422403	0.0007618227	0.009447669	−0.8870350	DQ599973	0.06130498	0.026196473	2.992671	0.003137224	0.02607541	−2.167018
35	PPP1R1B	−3.29766148	4.95411955	−7.592667	1.428092e−12	1.082861e−09	18.11719	LINC00607	0.05085119	0.048718987	4.099454	6.159070e−05	0.001501168	1.430515	RBM12	0.20206784	3.787498036	3.421809	0.0007633902	0.009462687	−0.8889094	AKR7L	−0.48894967	1.567901782	−2.992131	0.003142522	0.02611127	−2.168529
36	CYSLTR2	0.36111712	0.32415556	7.535736	1.997583e−12	1.472607e−09	17.79576	SERTAD4	−0.70757772	1.542036709	−4.099218	6.164825e−05	0.001501168	1.429647	FAM173A	0.38036524	4.036220041	3.421265	0.0007648289	0.009476095	−0.8906263	ACSM3	0.60764653	2.856468054	2.991646	0.003147292	0.02614272	−2.169888
37	DUSP23	−1.23233622	5.00484649	−7.521522	2.171737e−12	1.557723e−09	17.71571	AX747758	0.42649425	0.940651109	4.099189	6.165543e−05	0.001501168	1.429539	HRH1	−0.38968792	1.848263845	−3.420541	0.0007667457	0.009495410	−0.8929086	NANOS3	−0.54882980	1.038498511	−2.991398	0.003149728	0.02615477	−2.170580
38	SIGLEC8	0.79005294	0.67820497	7.495835	2.525375e−12	1.763708e−09	17.57123	SNORD92	0.16350121	0.117013703	4.097498	6.206996e−05	0.001509876	1.423313	AX748322	0.13746857	0.492177797	3.420370	0.0007671991	0.009496594	−0.8934477	ARID3C	0.09616431	0.120104145	2.990314	0.003160414	0.02623367	−2.173614
39	JAKMIP1	0.47440686	0.41805698	7.478763	2.791302e−12	1.899445e−09	17.47536	VANGL2	−0.93111266	1.834584697	−4.096133	6.240665e−05	0.001516364	1.418286	APLP1	−0.73804865	1.631130103	−3.420119	0.0007678675	0.009500436	−0.8942417	TRAPPC4	0.26859945	4.139694288	2.990233	0.003161208	0.02623367	−2.173838
40	CASP4	0.83856609	4.51498785	7.462865	3.063733e−12	2.032711e−09	17.38618	PXT1	0.04694900	0.079558258	4.095954	6.245097e−05	0.001516364	1.417627	LOC550643	−0.57548614	5.177916730	−3.419009	0.0007708197	0.009532318	−0.8977406	PSMA4	0.27789766	5.076664258	2.989816	0.003165332	0.02625482	−2.175006
41	PPIC	−1.10708502	4.11659724	−7.454578	3.216006e−12	2.081697e−09	17.33974	SOX2	1.44573950	1.886293253	4.093695	6.301218e−05	0.001528593	1.409315	ADCK2	0.34420836	3.625180698	3.418880	0.0007711627	0.009532318	−0.8981462	MOB3C	0.22806148	2.797354818	2.989775	0.003165734	0.02625482	−2.175120
42	FAM115C	0.28400465	0.27727997	7.365051	5.421547e−12	3.425773e−09	16.83973	SUMO2	0.26247393	6.578325815	4.092736	6.325179e−05	0.001533004	1.405788	IL7	0.45888070	1.754314878	3.418706	0.0007716269	0.009533615	−0.8986949	COL12A1	−0.63876098	4.267891424	−2.989508	0.003168375	0.02626851	−2.175867
43	MAP1LC3A	−1.20940851	4.18688305	−7.292945	8.236876e−12	5.083685e−09	16.43938	ZSCAN5A	0.31609859	1.325755600	4.090943	6.370231e−05	0.001542514	1.399194	HEMGN	0.04128973	0.036953709	3.418335	0.0007726193	0.009541434	−0.8998667	PLSCR2	−0.08391880	0.126922712	−2.989122	0.003172198	0.02629199	−2.176946
44	AK026512	0.31461802	0.29356855	7.239630	1.120634e−11	6.759207e−09	16.14475	SERINC2	−0.87856840	6.103909755	−4.090586	6.379234e−05	0.001543286	1.397882	ME1	−0.61029800	2.784727743	−3.418027	0.0007734409	0.009547138	−0.9008358	LOC729041	−0.09216480	0.151542951	−2.988112	0.003182219	0.02636682	−2.179771
45	CD101	0.52971611	0.87161738	7.194575	1.452267e−11	8.564828e−09	15.89668	RNF34	0.25292841	3.164063287	4.090280	6.386971e−05	0.001543751	1.396756	SLC7A5P1	0.29720730	0.947615388	3.417443	0.0007750031	0.009560702	−0.9026755	AKT2	0.24841920	4.016431373	2.987676	0.003186552	0.02639448	−2.180989
46	IL9R	0.38533293	0.34199557	7.166885	1.702352e−11	9.717409e−09	15.74466	DQ571132	0.04490483	0.010814694	4.085986	6.496392e−05	0.001568769	1.380976	SP1	0.25379569	4.535831973	3.417348	0.0007752602	0.009560702	−0.9029780	GABRB2	−0.17923895	0.174163346	−2.987186	0.003191423	0.02641950	−2.182357
47	RTBDN	1.03039555	0.57743859	7.164992	1.720932e−11	9.717409e−09	15.73428	ZXDA	−0.29767970	1.035568363	−4.085560	6.507332e−05	0.001569983	1.379413	TAF1B	0.23538307	2.420726305	3.417141	0.0007758136	0.009563083	−0.9036285	LOC283547	0.11647230	0.123548001	2.987172	0.003191565	0.02641950	−2.182396
48	CRTAM	0.67417749	0.59862664	7.117709	2.255527e−11	1.241772e−08	15.47547	LOC100653515	−0.11229475	0.202782496	−4.084989	6.522059e−05	0.001572106	1.377313	abParts	1.26977517	4.983387645	3.416569	0.0007773494	0.009577566	−0.9054316	PPP1R10	−0.19541683	4.327118570	−2.986296	0.003200306	0.02647763	−2.184845
49	GBP5	1.75773087	2.14443297	7.114844	2.292732e−11	1.241772e−08	15.45982	GPHB5	0.05172777	0.012439649	4.084509	6.534428e−05	0.001573659	1.375553	PHC1	0.26563425	2.091799265	3.415497	0.0007802334	0.009608638	−0.9088077	TGFB1I1	−0.69657118	4.846637315	−2.986268	0.003200582	0.02647763	−2.184923
50	IL18RAP	0.46817376	0.53445530	7.105561	2.417483e−11	1.283152e−08	15.40914	EPDR1	−0.72006803	2.383269853	−4.083718	6.554897e−05	0.001577157	1.372648	GATA2	−0.44657339	1.511845042	−3.412616	0.0007880356	0.009698546	−0.9178785	ARHGAP6	−0.41551635	1.363327130	−2.985810	0.003205162	0.02650726	−2.186203
51	XCL2	0.89993690	0.98743695	7.084813	2.721049e−11	1.415959e−08	15.29599	DL476309	0.77252466	4.952993446	4.082980	6.574060e−05	0.001580165	1.369936	IL4I1	0.33474440	2.947140470	3.412532	0.0007882649	0.009698546	−0.9181438	AF387615	0.15251598	0.069041602	2.985542	0.003207842	0.02651788	−2.186951
52	AX747415	0.11049157	0.06480756	7.073467	2.902660e−11	1.481417e−08	15.23419	C6orf130	0.43015447	3.059851055	4.082778	6.579308e−05	0.001580165	1.369195	GPR89C	0.01657356	0.024455340	3.412050	0.0007895775	0.009705734	−0.9196603	FRG1B	−0.39237522	2.563585961	−2.985481	0.003208444	0.02651788	−2.187119
53	FAM211B	−1.23590415	2.41011806	−7.061907	3.099978e−11	1.552270e−08	15.17129	GTF2H1	0.23503032	3.448767143	4.081023	6.625089e−05	0.001589722	1.362754	BC042032	−0.26463395	1.368300480	−3.412049	0.0007895806	0.009705734	−0.9196640	IGFBPL1	−0.65541432	0.977592238	−2.985230	0.003210963	0.02653043	−2.187822
54	PHYH	−1.03274427	3.99334549	−7.042191	3.467484e−11	1.704140e−08	15.06413	BC036874	0.03387424	0.025387100	4.080555	6.637349e−05	0.001591225	1.361037	PRPF19	0.26358897	5.784878886	3.411694	0.0007905480	0.009713127	−0.9207801	EPHB1	−0.48667132	0.942113078	−2.985061	0.003212653	0.02653614	−2.188293
55	WARS	1.67819452	6.11725015	7.038469	3.541534e−11	1.708887e−08	15.04393	SLC25A29	−0.45248617	3.258829266	−4.080298	6.644092e−05	0.001591404	1.360094	CRYBB1	0.20907784	0.575627224	3.411262	0.0007917295	0.009723142	−0.9221414	YES1	−0.35491030	3.821394435	−2.984622	0.003217060	0.02656426	−2.189520
56	TBX21	0.57075327	0.61307002	7.034052	3.631447e−11	1.720982e−08	15.01995	IGFLR1	0.48993065	3.003649307	4.078928	6.680137e−05	0.001598595	1.355069	ZNF431	0.31025709	1.763004951	3.410955	0.0007925699	0.009728960	−0.9231085	KIAA1967	0.26134893	4.467462960	2.983913	0.003224183	0.02661480	−2.191500
57	SYCE2	0.79618423	0.85070988	7.028722	3.742941e−11	1.742700e−08	14.99103	TMEM171	−0.89163514	3.145671150	−4.077737	6.711616e−05	0.001604681	1.350702	CCNE2	0.51126448	2.249164230	3.409886	0.0007954993	0.009760404	−0.9264712	HINT3	−0.26451996	3.384810234	−2.982644	0.003236957	0.02671194	−2.195040
58	FCGR1B	0.24927658	0.25619620	7.020839	3.914035e−11	1.759938e−08	14.94828	ZNF286B	0.16931952	0.583340458	4.076168	6.753309e−05	0.001613196	1.344950	CTPS1	0.36292676	3.096874837	3.409287	0.0007971443	0.009774939	−0.9283542	SMAD2	−0.21228718	2.745421948	−2.982363	0.003239799	0.02672708	−2.195826
59	ZNF480	−0.81784253	2.26418340	−7.019478	3.944352e−11	1.759938e−08	14.94090	SLFN12	0.40799391	1.260361186	4.075364	6.774784e−05	0.001616870	1.342002	DTX4	0.48059063	3.617015892	3.409186	0.0007974205	0.009774939	−0.9286699	NXT2	0.40873401	2.708706868	2.981277	0.003250784	0.02680937	−2.198856
60	MIAT	0.60639264	0.76685744	7.017939	3.978910e−11	1.759938e−08	14.93256	C6orf99	0.30667423	0.508444320	4.074853	6.788464e−05	0.001617344	1.340128	NCKAP1L	0.67675705	2.727421494	3.409011	0.0007979024	0.009776330	−0.9292205	ZNF141	−0.33584102	1.183961467	−2.981128	0.003252291	0.02681347	−2.199271
61	CXCR6	1.27611808	2.05081281	7.004705	4.288637e−11	1.825414e−08	14.86088	ZBTB7B	−0.41126833	4.659119932	−4.074699	6.792578e−05	0.001617344	1.339566	METRN	−0.62941073	3.790178431	−3.407483	0.0008021194	0.009823464	−0.9340249	ELP2	−0.24399434	3.482267880	−2.980730	0.003256323	0.02683838	−2.200380
62	CXCL9	2.55358388	4.36452190	7.003625	4.314955e−11	1.825414e−08	14.85503	PLIN2	−0.75484084	4.346370281	−4.074607	6.795051e−05	0.001617344	1.339228	ATP13A4	0.47954550	0.600731350	3.407088	0.0008032147	0.009832340	−0.9352686	CCDC74A	−0.39395063	1.552266982	−2.980615	0.003257495	0.02683970	−2.200702
63	PREPL	−0.45990148	3.32878994	−7.002876	4.333286e−11	1.825414e−08	14.85097	BC114455	0.28995225	0.861495386	4.074053	6.809918e−05	0.001619430	1.337198	ARSG	0.27139821	0.961680085	3.406766	0.0008041069	0.009838725	−0.9362804	PAK1	0.30463466	4.566419780	2.980403	0.003259641	0.02684904	−2.201291
64	GZMB	1.69503029	3.05028272	6.999642	4.413322e−11	1.830080e−08	14.83347	EZH2	0.57007474	2.578206563	4.073190	6.833142e−05	0.001623498	1.334036	AK123100	0.21560351	1.394415687	3.406468	0.0008049315	0.009840196	−0.9372145	PAQR7	−0.36266120	2.315780281	−2.980022	0.003263514	0.02687260	−2.202354
65	NMNAT3	−1.01094905	1.79713976	−6.980043	4.930590e−11	2.013122e−08	14.72750	ZNF235	0.26229393	1.312479284	4.072826	6.842960e−05	0.001624377	1.332703	AK094325	−0.18952554	0.680458865	−3.406455	0.0008049688	0.009840196	−0.9372567	SLC9B2	−0.52695716	1.947525514	−2.979001	0.003273912	0.02694986	−2.205201
66	DNAJC5B	0.45783728	0.40406660	6.958244	5.576331e−11	2.242276e−08	14.60982	MIP	0.04875695	0.062668350	4.069576	6.931232e−05	0.001643860	1.320800	ALDH3B2	0.78734626	1.211390992	3.406088	0.0008059889	0.009848131	−0.9384110	SCARB1	−0.47554461	4.201363299	−2.978871	0.003275231	0.02695236	−2.205562
67	KLRC4	0.58519048	0.48012675	6.946627	5.953800e−11	2.358327e−08	14.54720	RFX7	0.34572585	2.316245307	4.068698	6.955270e−05	0.001648089	1.317586	LPAR1	−0.62642857	2.112364563	−3.405815	0.0008067467	0.009852854	−0.9392675	PVRIG	0.54306745	1.960288078	2.977806	0.003286113	0.02703352	−2.208531
68	PRKCQ	0.82716256	1.04502742	6.927053	6.647616e−11	2.594428e−08	14.44182	HOMER1	0.44878047	1.481273243	4.068069	6.972526e−05	0.001649448	1.315285	SAMD9L	0.71189077	2.814805188	3.404893	0.0008093154	0.009879679	−0.9421646	EXTL2	−0.24822598	2.090285396	−2.977345	0.003290835	0.02706398	−2.209816
69	GQ868703	0.18801689	0.13549088	6.920250	6.906938e−11	2.656569e−08	14.40523	DUOXA2	−1.54868550	2.310939034	−4.068036	6.973436e−05	0.001649448	1.315163	SEC22A	0.17568210	1.980292258	3.404707	0.0008098341	0.009881466	−0.9427485	DIRAS3	−0.43208757	0.975438020	−2.976451	0.003300008	0.02713102	−2.212307
70	KCNK6	−0.92765273	3.38656515	−6.876051	8.852009e−11	3.356050e−08	14.16806	ESYT3	−0.38947076	0.646647820	−4.067301	6.993674e−05	0.001652762	1.312472	LOC100294362	0.39624657	1.869181020	3.404201	0.0008112482	0.009894171	−0.9443383	MZB1	1.06533831	3.844642243	2.975800	0.003306691	0.02717754	−2.214117
71	CAMK2N1	−1.59267863	5.46722086	−6.862348	9.558114e−11	3.561498e−08	14.09471	STAC3	0.39546364	1.248343536	4.065006	7.057186e−05	0.001666287	1.304078	STMN1	0.47533578	4.945386382	3.404021	0.0008117516	0.009895763	−0.9449037	RHOBTB3	−0.57073559	2.794266525	−2.974691	0.003318121	0.02725614	−2.217205
72	TOR4A	−1.40669120	4.32713128	−6.860411	9.662303e−11	3.561498e−08	14.08434	BC127858	−0.56266529	1.611077036	−4.064040	7.084087e−05	0.001671152	1.300546	TEX21P	0.08087985	0.151623913	3.403687	0.0008126849	0.009902592	−0.9459509	OLFM2	−0.67169205	2.625930407	−2.974673	0.003318309	0.02725614	−2.217256
73	TOMM20	−0.69873941	5.59121633	−6.852525	1.009817e−10	3.609342e−08	14.04217	PARP9	0.47850412	3.590557642	4.063632	7.095485e−05	0.001672354	1.299053	LOC645166	−0.34854139	1.266275989	−3.403308	0.0008137472	0.009910985	−0.9471412	FAM201A	−0.45515319	0.982160875	−2.974213	0.003323060	0.02728673	−2.218536
74	TP53I13	−0.77539510	4.32605507	−6.850223	1.022899e−10	3.609342e−08	14.02987	HOXA−AS3	−0.55284935	0.878020440	−4.062754	7.120065e−05	0.001676659	1.295842	SCLY	0.27392992	3.503857805	3.403021	0.0008145529	0.009916248	−0.9480431	PLA2G16	−0.63366733	4.788679276	−2.973873	0.003326574	0.02730713	−2.219482
75	FAM71E1	−0.82527966	3.16290915	−6.848374	1.033527e−10	3.609342e−08	14.01999	HOXB−AS5	−0.55918577	0.672481455	−4.062370	7.130830e−05	0.001677229	1.294440	LOC400604	0.20318018	0.893870639	3.402297	0.0008165875	0.009936458	−0.9503163	ARHGEF17	−0.46144499	2.992837953	−2.973758	0.003327765	0.02730846	−2.219802
76	AK124194	0.10850435	0.10536971	6.848360	1.033611e−10	3.609342e−08	14.01992	MAGIX	−0.63173828	1.481591061	−4.062217	7.135127e−05	0.001677229	1.293880	LILRB5	0.32010119	0.702412175	3.401846	0.0008178561	0.009947334	−0.9517308	CEP120	0.24632986	2.454726921	2.973493	0.003330515	0.02732177	−2.220541
77	AX747832	0.55706915	0.51199472	6.837997	1.095240e−10	3.774881e−08	13.96457	CASP1	0.85906042	3.392698081	4.061725	7.148975e−05	0.001678997	1.292080	KIAA1524	0.42756128	1.918167546	3.401497	0.0008188414	0.009954756	−0.9528280	FAM83F	−0.52343337	2.416118291	−2.973403	0.003331446	0.02732177	−2.220791
78	WNK2	−1.55829434	2.80717292	−6.818212	1.223121e−10	4.161591e−08	13.85903	DAZAP1	0.27073308	5.625660493	4.060966	7.170372e−05	0.001682533	1.289305	ZBTB2	0.21381138	3.052384653	3.400489	0.0008216895	0.009984806	−0.9559918	C7orf49	0.18699270	2.859641363	2.973169	0.003333872	0.02733322	−2.221442
79	KLRK1	1.04645230	1.36963643	6.813884	1.253000e−10	4.209286e−08	13.83597	SIGMAR1	−0.49997795	6.171922348	−4.059162	7.221442e−05	0.001692579	1.282716	SMC4	0.41840342	4.206797984	3.399656	0.0008240496	0.010008903	−0.9586052	BC058547	−0.50488663	2.635779053	−2.972634	0.003339417	0.02737022	−2.222929
80	SARM1	−0.64169095	2.55546543	−6.806341	1.306802e−10	4.335153e−08	13.79580	PCDP1	0.53620272	0.682455483	4.059004	7.225938e−05	0.001692579	1.282138	PPL	−0.78564760	3.285139213	−3.399506	0.0008244746	0.010009484	−0.9590750	ZNF85	0.24530712	0.777839424	2.972147	0.003344481	0.02739805	−2.224285
81	ZNF32	−0.80042617	4.10872492	−6.765878	1.636643e−10	5.362329e−08	13.58075	BC043220	−0.16277733	0.228028549	−4.058541	7.239117e−05	0.001694127	1.280447	FLJ34208	−0.31488131	0.758939794	−3.398553	0.0008271846	0.010037793	−0.9620650	DNASE1L3	0.42174661	1.012516900	2.972109	0.003344877	0.02739805	−2.224391
82	APOC1P1	0.41779496	0.37914841	6.758164	1.708242e−10	5.528661e−08	13.53985	CHORDC1	0.42711616	3.021594648	4.058323	7.245314e−05	0.001694127	1.279652	ENPP5	−0.56901781	1.109290047	−3.397667	0.0008297097	0.010063833	−0.9648420	FIZ1	0.20248408	2.124675131	2.971738	0.003348735	0.02742119	−2.225423
83	IQCB1	0.60797315	2.83841726	6.750021	1.787177e−10	5.714443e−08	13.49669	FLJ30679	0.06350617	0.056348210	4.057437	7.270611e−05	0.001698545	1.276417	SERBP1	0.22565503	5.361810821	3.396723	0.0008324098	0.010091970	−0.9678020	FCRL5	0.40253597	0.842171710	2.971435	0.003351892	0.02743857	−2.226266
84	AK124820	0.61832668	0.64605759	6.744413	1.843616e−10	5.824729e−08	13.46699	MIR196A1	−0.44026175	0.508706217	−4.056440	7.299192e−05	0.001701390	1.272775	DTX3L	0.36630068	4.196773705	3.396142	0.0008340749	0.010107541	−0.9696227	PKMYT1	0.51177129	3.648562920	2.970222	0.003364555	0.02753374	−2.229639
85	SLC26A9	1.74438007	1.22229442	6.714705	2.173167e−10	6.785139e−08	13.30989	LRRC49	−0.31768744	0.956459757	−4.056419	7.299795e−05	0.001701390	1.272698	ASAP3	−0.49201438	2.047410411	−3.395840	0.0008349416	0.010113425	−0.9705688	FSCN1	−0.70435263	4.752616178	−2.969615	0.003370899	0.02757716	−2.231325
86	HAPLN3	1.13160198	2.57640812	6.701268	2.340667e−10	7.223135e−08	13.23897	AK098656	0.01779217	0.009182860	4.056341	7.302022e−05	0.001701390	1.272415	LOC647979	−0.39221456	5.977347336	−3.395000	0.0008373588	0.010133685	−0.9732025	SNHG1	0.38138050	3.262978179	2.969048	0.003376842	0.02761726	−2.232900
87	AK125001	0.32837680	0.31725818	6.693071	2.449023e−10	7.470645e−08	13.19574	LOC283888	0.50790614	0.908126670	4.055198	7.334930e−05	0.001707559	1.268240	ENDOD1	−0.39673089	3.303946900	−3.394993	0.0008373778	0.010133685	−0.9732232	DQ576223	0.03577158	0.010821535	2.968899	0.003378412	0.02762159	−2.233316
88	CERK	−0.90484249	3.76799128	−6.689192	2.502003e−10	7.545530e−08	13.17530	BTF3	0.34691469	7.486936815	4.053300	7.389857e−05	0.001718838	1.261315	CCNH	0.24332000	2.825668112	3.394743	0.0008380989	0.010137788	−0.9740073	FAM113B	0.52048308	2.443611362	2.968598	0.003381567	0.02763887	−2.234151
89	VRTN	0.69306903	0.37050929	6.686979	2.532730e−10	7.552373e−08	13.16364	EME2	0.36353081	1.328732252	4.052436	7.415015e−05	0.001723179	1.258160	SELPLG	0.62448505	3.223442805	3.393346	0.0008421350	0.010181969	−0.9783839	NGRN	−0.32343250	5.657141029	−2.968251	0.003385212	0.02765587	−2.235115
90	GTF2E1	0.37040475	2.17902264	6.684619	2.565909e−10	7.566296e−08	13.15121	BC067230	−0.39434036	3.980895328	−4.050997	7.457063e−05	0.001729389	1.252911	EIF3F	0.27079717	5.754070290	3.393196	0.0008425680	0.010182564	−0.9788520	TPCN2	−0.21212780	2.031355746	−2.968202	0.003385732	0.02765587	−2.235252
91	BAX	0.72891776	5.09164733	6.682503	2.596032e−10	7.571000e−08	13.14007	ZNF284	0.28835745	1.044324137	4.050855	7.461204e−05	0.001729389	1.252396	PRUNE	−0.28317791	3.475395729	−3.392140	0.0008456317	0.010214938	−0.9821583	C7orf73	0.27433853	3.657391572	2.967894	0.003388968	0.02767379	−2.236107
92	FBXW10	0.19085764	0.13509009	6.659280	2.950274e−10	8.510579e−08	13.01791	NPM1	0.47361737	6.930512793	4.050852	7.461285e−05	0.001729389	1.252386	TSPAN18	−0.68398732	3.228665175	−3.391630	0.0008471159	0.010224209	−0.9837557	ZNF571	0.13973882	0.561135985	2.967393	0.003394247	0.02770837	−2.237499
93	GBP1P1	0.84243559	1.12288667	6.638339	3.310231e−10	9.446260e−08	12.90798	SNORD115−44	0.03332819	0.005994484	4.049792	7.492437e−05	0.001735094	1.248519	BC171896	0.07513082	0.152729097	3.391612	0.0008471696	0.010224209	−0.9838135	KLHDC9	−0.30169525	1.219075177	−2.967063	0.003397722	0.02772821	−2.238414
94	TMEM143	0.50130211	1.79417905	6.635953	3.353900e−10	9.469059e−08	12.89546	PIDD	0.36877134	2.082666105	4.049473	7.501829e−05	0.001735470	1.247356	ACAP1	0.63436167	2.082716674	3.390730	0.0008497397	0.010247037	−0.9865726	FBXO42	0.16259637	2.503980849	2.966392	0.003404816	0.02777756	−2.240280
95	ANKRD32	0.44184635	1.34341305	6.605077	3.972695e−10	1.109804e−07	12.73379	CRABP2	−1.38977316	3.012355564	−4.049293	7.507141e−05	0.001735470	1.246699	ABCC6P2	−0.43977833	0.817875045	−3.390593	0.0008501415	0.010247037	−0.9870032	PON1	−0.57321326	0.859752876	−2.965882	0.003410204	0.02781297	−2.241694
96	TXNRD3	−0.65340706	1.28890414	−6.599098	4.104907e−10	1.134793e−07	12.70254	C4orf32	0.41558254	2.198454540	4.046928	7.577176e−05	0.001750136	1.238080	TSPYL1	0.29004322	4.071004193	3.390566	0.0008502195	0.010247037	−0.9870868	ZFP14	0.18595292	1.032876052	2.965502	0.003414227	0.02783668	−2.242749
97	GOLT1A	−1.37039245	2.06250741	−6.572713	4.741893e−10	1.290695e−07	12.56482	OR10A4	0.04102132	0.040549556	4.045599	7.616814e−05	0.001757762	1.233238	NMU	−1.02884482	2.398792552	−3.390093	0.0008516022	0.010259042	−0.9885668	LYPD6	0.47721551	1.273707938	2.965410	0.003415209	0.02783668	−2.243006
98	LOC400043	1.97946821	3.27033444	6.571069	4.784647e−10	1.290695e−07	12.55625	MCM2	0.69896302	4.137691884	4.045138	7.630600e−05	0.001759272	1.231559	hCG_1980662	0.24965042	1.384985999	3.389795	0.0008524739	0.010264885	−0.9894988	BACE1	−0.36405753	2.840061284	−2.964929	0.003420304	0.02786965	−2.244339
99	SKA2	0.52028674	3.52631434	6.569920	4.814756e−10	1.290695e−07	12.55027	LRRC72	0.01974011	0.007948513	4.044727	7.642920e−05	0.001759272	1.230062	MBOAT1	0.50916026	3.009921679	3.389582	0.0008530988	0.010267750	−0.9901662	ANKRD22	0.62214687	2.991961660	2.964306	0.003426922	0.02791501	−2.246068
100	BC045668	0.09188714	0.05635801	6.557928	5.140351e−10	1.362468e−07	12.48780	LOC643770	0.12364010	0.211462997	4.044716	7.643243e−05	0.001759272	1.230023	OVOL2	0.60071057	3.238291783	3.388990	0.0008548355	0.010282516	−0.9920185	AGER	0.19282521	0.889165213	2.963302	0.003437610	0.02799347	−2.248853

	Gene	logFC	AveExpr	t	P.Value	adj.P.Val	B	Gene	logFC	AveExpr	t	P.Value	adj.P.Val	B	Gene	logFC	AveExpr	t	P.Value	adj.P.Val	B	Gene	logFC	AveExpr	t	P.Value	adj.P.Val	B
101	DQ586608	−0.95846643	1.66771924	−6.556336	5.185171e−10	1.362468e−07	12.479516	HTR1D	−0.98689733	1.474033384	−4.043420	7.682221e−05	0.001766711	1.2253021	RALBP1	−0.31269187	4.540428533	−3.388900	0.0008551005	0.01028252	−0.9923008	RPH3A	0.059740453	0.058854966	2.963050	0.003440303	0.02800681	−2.249553
102	SLA2	0.80910581	1.58545132	6.545988	5.485998e−10	1.427381e−07	12.425685	ICAM1	0.80415074	4.833894802	4.041376	7.744082e−05	0.001779396	1.2178590	DQ582785	0.01244212	0.004285885	3.388533	0.0008561769	0.01028804	−0.9934466	F2RL2	0.619111639	2.667381646	2.962563	0.003445501	0.02804053	−2.250904
103	TIFAB	0.60271302	0.64361456	6.541279	5.628545e−10	1.450252e−07	12.401201	PAQR4	0.65995957	4.139539625	4.040315	7.776367e−05	0.001785213	1.2139982	TBC1D3B	0.02045968	0.023439336	3.388479	0.0008563356	0.01028804	−0.9936154	LOC100287177	0.227895740	0.890167667	2.962088	0.003450586	0.02807330	−2.252223
104	DSC2	−1.57223414	3.33350608	−6.518954	6.355372e−10	1.621781e−07	12.285288	PTK6	−0.87185543	3.082889251	−4.040102	7.782853e−05	0.001785213	1.2132245	BCKDHB	−0.33412730	2.628483395	−3.388110	0.0008574216	0.01029643	−0.9947697	CD109	−0.477064693	1.532385894	−2.961723	0.003454489	0.02809644	−2.253234
105	LAP3	0.84521555	5.57423221	6.515672	6.469725e−10	1.635238e−07	12.268269	GPRC5B	1.12625443	3.989048765	4.039424	7.803578e−05	0.001788421	1.2107567	JA429839	0.02522269	0.005329819	3.387712	0.0008585957	0.01030587	−0.9960161	AHSP	0.071579027	0.104767202	2.961498	0.003456904	0.02810747	−2.253858
106	TRBV9	0.96497479	1.06216359	6.496056	7.196620e−10	1.789299e−07	12.166657	cytochrome_b	−0.44655524	12.787737275	−4.038763	7.823816e−05	0.001791512	1.2083532	ZNF777	−0.23448358	3.292368975	−3.386457	0.0008623024	0.01034373	−0.9999394	CCT2	0.374381042	5.777244395	2.960491	0.003467713	0.02818672	−2.256650
107	LZTFL1	−0.58039961	2.48835131	−6.495609	7.214100e−10	1.789299e−07	12.164342	E2F6	0.25292784	1.818080418	4.038496	7.832014e−05	0.001791843	1.2073814	PTP4A3	−0.61711914	4.245946115	−3.386380	0.0008625294	0.01034373	−1.0001791	FAM127C	−0.479795217	2.558890782	−2.960361	0.003469110	0.02818944	−2.257010
108	ZNF669	−0.51309295	1.82433058	−6.489528	7.455914e−10	1.832153e−07	12.132881	MRPL11	0.35626683	4.380819550	4.035617	7.920870e−05	0.001810611	1.1969131	FMO5	−0.75344825	2.245188459	−3.386148	0.0008632161	0.01034729	−1.0009039	GBF1	−0.207001575	3.760021793	−2.960069	0.003472246	0.02820044	−2.257818
109	C5orf20	0.38337090	0.39000902	6.481691	7.779322e−10	1.894086e−07	12.092364	ZNF689	0.33501558	2.469137045	4.034823	7.945545e−05	0.001813974	1.1940270	AX748287	0.05340950	0.120092778	3.385892	0.0008639755	0.01035171	−1.0017047	DPEP1	−1.325142636	2.383020415	−2.959984	0.003473165	0.02820044	−2.258055
110	KIR2DL2	0.07384135	0.04756036	6.476514	8.000473e−10	1.930223e−07	12.065617	HOXB6	−0.86119557	2.181163835	−4.034591	7.952779e−05	0.001813974	1.1931827	AK056490	0.35775374	0.619899087	3.385118	0.0008662737	0.01036997	−1.0041239	MEIS2	0.465437622	2.186288344	2.959939	0.003473651	0.02820044	−2.258180
111	PPARA	−0.64178815	2.51282558	−6.468871	8.338334e−10	1.993613e−07	12.026150	ITGAL	0.90579118	2.574461439	4.034345	7.960451e−05	0.001813974	1.1922880	MICB	0.59457542	2.225758648	3.385116	0.0008662803	0.01036997	−1.0041309	RPL13A	0.380635135	8.950595975	2.959518	0.003478185	0.02822861	−2.259346
112	LGALS17A	0.60691494	0.28922986	6.455226	8.976697e−10	2.127076e−07	11.955768	SNX21	−0.42127069	2.637233556	−4.034102	7.968027e−05	0.001813974	1.1914054	ZNF624	0.15585092	0.977863062	3.384452	0.0008682576	0.01038486	−1.0062072	CEP57L1	0.211979548	1.522323294	2.959346	0.003480035	0.02823499	−2.259822
113	XCR1	0.54134431	0.50234938	6.418199	1.096111e−09	2.566916e−07	11.765238	AK126253	0.22553912	0.360489688	4.034047	7.969756e−05	0.001813974	1.1912041	UTP15	0.23936581	2.123924538	3.384365	0.0008685162	0.01038486	−1.0064783	RNASET2	−0.394196288	4.337578823	−2.958960	0.003484197	0.02825038	−2.260890
114	LOC100130275	0.68006574	0.77270664	6.415544	1.111892e−09	2.566916e−07	11.751602	INTS12	0.30963423	3.410395787	4.033115	7.998882e−05	0.001819043	1.1878194	SNORD30	0.38084648	1.013482294	3.384304	0.0008686986	0.01038486	−1.0066696	TRAM2	−0.361748486	3.386492218	−2.958909	0.003484755	0.02825038	−2.261034
115	GPR171	0.93160957	1.38946486	6.415474	1.112308e−09	2.566916e−07	11.751246	CXCL17	2.29275205	3.914407026	4.032587	8.015435e−05	0.001821247	1.1859012	BC022047	−0.41345316	2.037120149	−3.383913	0.0008698623	0.01039191	−1.0078887	PIK3CG	0.453545059	1.363453100	2.958838	0.003485517	0.02825038	−2.261229
116	NMB	−1.00938890	2.77880439	−6.403159	1.188506e−09	2.719117e−07	11.688044	CECR1	0.88428540	3.484419455	4.032032	8.032876e−05	0.001823648	1.1838846	BC062758	−0.79237183	1.502799891	−3.383843	0.0008700714	0.01039191	−1.0081075	AHRR	0.236702571	0.842388190	2.958704	0.003486968	0.02825038	−2.261601
117	LOC388906	0.38890292	0.63793083	6.401030	1.202187e−09	2.720220e−07	11.677127	GNG12	−0.56429025	5.104204516	−4.028621	8.140840e−05	0.001845487	1.1714979	WIPI1	−0.45604707	3.612877731	−3.383237	0.0008718821	0.01040839	−1.0100006	PI15	−0.487451445	0.817702299	−2.958590	0.003488197	0.02825038	−2.261916
118	TMEM117	−0.63417555	2.14056278	−6.399904	1.209488e−09	2.720220e−07	11.671352	MAP3K5	0.36392962	2.724426823	4.028554	8.142979e−05	0.001845487	1.1712542	PRSS21	−1.27050876	1.831911239	−3.383119	0.0008722359	0.01040839	−1.0103701	LOC100289473	−0.084221871	0.120078633	−2.958521	0.003488945	0.02825038	−2.262108
119	FAM26F	1.31187772	2.43298096	6.369212	1.426101e−09	3.180444e−07	11.514229	ALDH3A1	1.34353831	2.676766051	4.028295	8.151229e−05	0.001845780	1.1703148	MON2	0.25972512	2.189213672	3.382485	0.0008741344	0.01042636	−1.0123499	CAMK4	0.223806786	0.588501366	2.958479	0.003489393	0.02825038	−2.262223
120	IDO1	2.51625771	3.00612490	6.366561	1.446506e−09	3.199068e−07	11.500681	bK250D10.C22.8	0.15315023	0.192874940	4.027955	8.162083e−05	0.001846663	1.1690802	NR2C1	0.26025795	2.469252373	3.381735	0.0008763853	0.01044851	−1.0146917	MRI1	−0.342071070	3.005073137	−2.958382	0.003490448	0.02825038	−2.262493
121	CLEC12A	0.69440581	0.72461061	6.363682	1.468992e−09	3.221949e−07	11.485971	DL476366	0.76107229	4.955142480	4.027501	8.176600e−05	0.001848371	1.1674316	THBS3	−0.38508228	2.588722068	−3.381462	0.0008772062	0.01045360	−1.0155442	AX748230	0.215599420	2.146916330	2.958218	0.003492223	0.02825613	−2.262948
122	HOXA10	−1.68949690	2.22210611	−6.348679	1.591838e−09	3.462770e−07	11.409386	AVEN	0.30482314	2.875253826	4.026876	8.196606e−05	0.001850009	1.1651645	TYROBP	0.68089237	6.517094132	3.380893	0.0008789192	0.01046521	−1.0173206	ALKBH2	0.319921615	3.111367739	2.957561	0.003499336	0.02829952	−2.264767
123	UBE2L6	1.05043819	5.60225069	6.345419	1.619841e−09	3.491019e−07	11.392757	MIR196B	−0.83149015	1.045018495	−4.026781	8.199664e−05	0.001850009	1.1648185	LOC170425	−0.23910280	0.302512421	−3.380877	0.0008789688	0.01046521	−1.0173720	TOMM22	−0.253780423	4.981941594	−2.957525	0.003499719	0.02829952	−2.264865
124	CISD1	−0.60545491	3.49867428	−6.344120	1.631133e−09	3.491019e−07	11.386133	HEBP1	−0.54600113	4.595333951	−4.026622	8.204760e−05	0.001850009	1.1642421	FGF18	−0.52113826	1.265471175	−3.380720	0.0008794427	0.01046616	−1.0178627	CCDC43	0.209662539	3.470371415	2.957263	0.003502565	0.02831391	−2.265591
125	CD8A	1.45518670	2.28839935	6.321986	1.835845e−09	3.884771e−07	11.273405	GDPD1	0.51017960	1.429694520	4.026271	8.216029e−05	0.001850978	1.1629688	TNNC1	−0.84530593	1.626665811	−3.380449	0.0008802578	0.01046993	−1.0187062	EEF1A2	−1.005218070	1.466626832	−2.957113	0.003504196	0.02831847	−2.266008
126	RCL1	0.64937778	2.40087045	6.321116	1.844384e−09	3.884771e−07	11.268981	BC028022	0.27592237	1.359970850	4.025501	8.240796e−05	0.001854983	1.1601765	KIR3DL3	0.01052348	0.007728418	3.380340	0.0008805879	0.01046993	−1.0190477	IMPAD1	−0.251690801	4.341066269	−2.956498	0.003510870	0.02836377	−2.267709
127	KLRD1	0.76491860	1.03900257	6.298761	2.077776e−09	4.341897e−07	11.155387	GPC1	−0.77960353	4.388414712	−4.023378	8.309499e−05	0.001867412	1.1524748	NFATC4	−0.43568349	1.486263916	−3.380222	0.0008809430	0.01046993	−1.0194147	C11orf53	−0.686343944	1.325985735	−2.956128	0.003514901	0.02838769	−2.268735
128	AX747104	0.51966335	1.10486867	6.288600	2.193213e−09	4.547318e−07	11.103845	C12orf5	0.35252966	1.894886354	4.023360	8.310084e−05	0.001867412	1.1524094	DBR1	0.45392859	3.075462887	3.379877	0.0008819872	0.01047764	−1.0204934	LYZ	1.217307642	9.595487268	2.955981	0.003516496	0.02839194	−2.269140
129	ARHGEF3	0.57735031	2.78386942	6.285227	2.232914e−09	4.591325e−07	11.086744	SEMA6A	−0.85141125	2.018425641	−4.023036	8.320611e−05	0.001868195	1.1512351	ENO4	−0.07930331	0.234879570	−3.379453	0.0008832685	0.01048817	−1.0218151	NUDT11	−0.169137658	0.429573751	−2.955683	0.003519744	0.02840952	−2.269966
130	PTCRA	0.20459219	0.18713638	6.283873	2.249038e−09	4.591325e−07	11.079885	NUP37	0.36932583	3.572465125	4.022446	8.339831e−05	0.001870273	1.1490948	MEX3D	0.34494977	4.200515057	3.378527	0.0008860770	0.01051681	−1.0247055	SHMT2	0.455998915	5.229539325	2.953727	0.003541119	0.02857138	−2.275378
131	DAZAP2	−0.49433181	6.61331311	−6.272072	2.394574e−09	4.826593e−07	11.020118	AK307207	−0.57218365	0.602053383	−4.022319	8.343959e−05	0.001870273	1.1486358	PIK3R3	0.37802461	2.498804491	3.378119	0.0008873158	0.01052340	−1.0259775	DQ589976	0.024283559	0.006783741	2.953651	0.003541951	0.02857138	−2.275588
132	OR56B1	0.58797328	0.86088260	6.271595	2.400657e−09	4.826593e−07	11.017700	ERN2	−1.17476614	3.663332128	−4.021948	8.356058e−05	0.001871404	1.1472916	DUS2L	0.29469365	2.676726936	3.378083	0.0008874252	0.01052340	−1.0260897	MYBBP1A	0.294072909	3.861925139	2.952862	0.003550605	0.02862447	−2.277769
133	MUC3A	−2.31768143	4.42265827	−6.255908	2.609032e−09	5.206097e−07	10.938367	ADAM5P	0.01149287	0.005176725	4.021363	8.375194e−05	0.001874109	1.1451698	ZNF530	−0.28003025	0.716151996	−3.377380	0.0008895659	0.01054408	−1.0282833	ARF6	0.257563712	5.243027324	2.952854	0.003550690	0.02862447	−2.277791
134	THEMIS	0.68112330	0.79662215	6.241658	2.813646e−09	5.572488e−07	10.866412	ACOT7	0.51841607	4.619562528	4.020385	8.407263e−05	0.001879700	1.1416249	HNRNPH1	0.28983497	5.224946295	3.376880	0.0008910895	0.01055742	−1.0298412	CAP2	−0.628588134	2.070199196	−2.952731	0.003552044	0.02862669	−2.278131
135	DPY19L1	0.74342175	3.24326032	6.237602	2.874702e−09	5.651239e−07	10.845953	SLC6A20	−1.07604348	1.924398644	−4.019232	8.445201e−05	0.001886593	1.1374486	OSBPL7	0.48281197	2.640660139	3.375902	0.0008940792	0.01058812	−1.0328906	C1orf94	−0.014423864	0.018059047	−2.952376	0.003555948	0.02864946	−2.279113
136	CENPH	0.80241353	2.65536581	6.232784	2.948934e−09	5.754542e−07	10.821658	TLR7	0.46502128	0.957446090	4.018377	8.473467e−05	0.001891315	1.1343494	UBN2	0.23762768	1.731101588	3.375540	0.0008951900	0.01059654	−1.0340209	TCEAL4	−0.452800051	4.918079969	−2.952088	0.003559118	0.02866629	−2.279910
137	GNGT2	0.60642667	1.23928979	6.226996	3.040590e−09	5.890088e−07	10.792491	NUP62	0.33517305	4.732592468	4.015628	8.564896e−05	0.001910116	1.1243952	FLJ46906	0.22186143	0.891557118	3.375356	0.0008957530	0.01059848	−1.0345933	TSC22D4	0.296521873	4.569179964	2.950511	0.003576510	0.02878940	−2.284267
138	FAS	0.89482157	1.96960706	6.221065	3.137418e−09	6.033619e−07	10.762620	TNFAIP8L2	0.52968501	2.044352523	4.013793	8.626444e−05	0.001921578	1.1177542	ST7−OT4	0.19373887	0.388723670	3.375200	0.0008962310	0.01059941	−1.0350790	ADA	0.494529218	3.157751991	2.950506	0.003576572	0.02878940	−2.284282
139	FBXO44	−0.71864497	2.05997242	−6.212547	3.281806e−09	6.265889e−07	10.719747	C6orf141	0.72604621	1.399076771	4.013665	8.630772e−05	0.001921578	1.1172891	OR1E2	0.02719895	0.010759325	3.374749	0.0008976176	0.01060392	−1.0364863	PIM2	0.588072260	4.245322633	2.950030	0.003581831	0.02882299	−2.285595
140	PDE4A	−0.72032641	2.41025881	−6.209101	3.342046e−09	6.335325e−07	10.702416	RANBP6	0.40087009	2.201400205	4.013100	8.649800e−05	0.001924200	1.1152467	BRCA1	0.38633590	1.979025753	3.374636	0.0008979656	0.01060392	−1.0368392	SPEF2	−0.179959816	0.450097043	−2.949561	0.003587028	0.02885606	−2.286891
141	GPR82	0.34434236	0.62897176	6.199094	3.523198e−09	6.631358e−07	10.652123	FOXJ1	−1.20355636	1.554962493	−4.012023	8.686247e−05	0.001930689	1.1113471	FANCA	0.42237367	2.589025681	3.374573	0.0008981589	0.01060392	−1.0370351	PELI3	−0.307479588	1.985335845	−2.948487	0.003598959	0.02894326	−2.289859
142	C4orf50	0.05353766	0.04230488	6.193173	3.634898e−09	6.793419e−07	10.622386	OR52H1	0.04558627	0.024417438	4.009715	8.764761e−05	0.001946510	1.1030024	PDK2	−0.36686158	4.126873357	−3.374484	0.0008984313	0.01060392	−1.0373112	MIR4715	0.063676073	0.048047951	2.947739	0.003607289	0.02900147	−2.291925
143	SLC46A1	−0.64872500	2.66741912	−6.161927	4.284360e−09	7.951234e−07	10.465783	FAM27E3	0.14185502	0.199236391	4.009257	8.780449e−05	0.001948364	1.1013441	HCST	0.77170911	4.039734710	3.374426	0.0008986103	0.01060392	−1.0374926	CD163	0.638123890	2.699767143	2.947191	0.003613399	0.02903671	−2.293437
144	HAS3	0.73543938	2.49403866	6.157398	4.387483e−09	8.086070e−07	10.443127	TBX3	−0.75645599	2.350742077	−4.007884	8.827562e−05	0.001957182	1.0963817	TNFRSF10D	−0.53698813	1.874494437	−3.372702	0.0009039287	0.01066194	−1.0428642	CC2D2B	0.050684993	0.167405884	2.947149	0.003613860	0.02903671	−2.293551
145	LAG3	1.14833169	1.86457245	6.147473	4.622080e−09	8.407725e−07	10.393513	CLEC1B	0.03830077	0.036195098	4.007531	8.839704e−05	0.001958238	1.0951070	HNRNPR	0.22053545	5.567565608	3.372504	0.0009045432	0.01066445	−1.0434828	VAMP3	0.238407397	5.569382801	2.946541	0.003620662	0.02907478	−2.295231
146	TRD	0.68846448	0.55131324	6.147337	4.625374e−09	8.407725e−07	10.392835	NPW	−1.29528999	1.981933365	−4.007284	8.848235e−05	0.001958493	1.0942126	UBA2	0.34751087	4.983916903	3.371905	0.0009063973	0.01068156	−1.0453467	RFX5	0.318708817	4.054721032	2.946529	0.003620790	0.02907478	−2.295263
147	LOC285972	0.39114903	0.53276754	6.145881	4.660825e−09	8.414533e−07	10.385562	SSFA2	0.48280253	4.305919544	4.006817	8.864361e−05	0.001958931	1.0925242	RPS9	0.25862163	8.302385547	3.371642	0.0009072133	0.01068241	−1.0461658	SLC26A10	−0.192119000	0.435923437	−2.946311	0.003623234	0.02908560	−2.295866
148	SPINK14	0.15524886	0.06118063	6.137956	4.858564e−09	8.712258e−07	10.345989	WDR72	−0.96440227	1.437859251	−4.006799	8.864978e−05	0.001958931	1.0924596	PDE6G	0.35800688	0.787782297	3.371623	0.0009072739	0.01068241	−1.0462266	TARBP2	0.251976874	2.936583720	2.946051	0.003626144	0.02910016	−2.296583
149	B3GALT4	−0.62106184	2.73808697	−6.135795	4.913882e−09	8.740861e−07	10.335206	TFAM	0.36927375	3.810434147	4.006460	8.876702e−05	0.001959890	1.0912341	TIGD4	0.13209792	0.309887232	3.370200	0.0009117011	0.01072977	−1.0506573	RAB1A	−0.200019313	6.016644371	−2.945068	0.003637169	0.02917982	−2.299296
150	TCEA3	−1.53108503	3.87367370	−6.134768	4.940386e−09	8.740861e−07	10.330083	SLC20A2	−0.47974075	3.464134511	−4.005700	8.902999e−05	0.001964062	1.0884911	VTI1A	0.21374275	1.954641585	3.369747	0.0009131145	0.01073831	−1.0520672	SH2D4A	0.511301254	3.529052139	2.944960	0.003638383	0.02918073	−2.299594
151	TRBV21−1	0.33590893	0.26492639	6.131541	5.024617e−09	8.831013e−07	10.313982	OSBPL5	−0.52450964	2.808385140	−4.004956	8.928875e−05	0.001968135	1.0858000	ZNF234	0.22237074	1.372893056	3.369640	0.0009134478	0.01073831	−1.0523994	TRAM1	0.324311322	6.468117903	2.944062	0.003648473	0.02925282	−2.302068
152	GBP4	1.64188910	3.79175704	6.127566	5.130274e−09	8.957391e−07	10.294164	CXorf65	0.31552104	0.493426427	4.002377	9.019003e−05	0.001986064	1.0764877	AMELX	0.17894119	0.148475362	3.369578	0.0009136403	0.01073831	−1.0525912	DEFB136	−0.029266851	0.018365549	−2.943827	0.003651129	0.02926527	−2.302718
153	PPIE	−0.36954143	2.00261418	−6.123285	5.246535e−09	9.100510e−07	10.272823	FAM222A	−0.80487480	2.054623910	−4.002201	9.025183e−05	0.001986064	1.0758525	TRNP1	1.09658378	4.921156298	3.368958	0.0009155799	0.01075311	−1.0545214	ALDOC	−0.586875646	3.345197974	−2.943283	0.003657260	0.02930556	−2.304217
154	DBN1	−1.03718315	3.96506102	−6.111314	5.585468e−09	9.625502e−07	10.213210	PDE9A	−0.70747238	1.538740701	−4.000687	9.078539e−05	0.001996150	1.0703874	TTLL2	−0.14767752	0.220540509	−3.368917	0.0009157096	0.01075311	−1.0546503	SLC22A17	−0.550119137	2.059014134	−2.942635	0.003664576	0.02935057	−2.306002
155	AGBL3	0.15111970	0.23483153	6.098497	5.972155e−09	1.022549e−06	10.149469	TRBV4−2	0.44399954	0.707347419	3.999628	9.116039e−05	0.002002737	1.0665655	SNORA2A	0.16530441	0.250430253	3.368132	0.0009181688	0.01077721	−1.0570913	APOBEC3C	0.558758108	4.589131358	2.942543	0.003665620	0.02935057	−2.306257
156	FAM213A	−0.78730512	3.87619830	−6.095444	6.068061e−09	1.027992e−06	10.134300	DTD1	−0.53851107	3.810982418	−3.999330	9.126602e−05	0.002003398	1.0654919	IFI30	0.62677372	7.877735044	3.367821	0.0009191473	0.01078393	−1.0580606	ERCC6L	0.331092240	1.260428665	2.942492	0.003666194	0.02935057	−2.306397
157	FCGR1C	0.16780195	0.14602938	6.093143	6.141363e−09	1.027992e−06	10.122867	C2orf65	−0.56378238	0.810008588	−3.998570	9.153651e−05	0.002007676	1.0627482	G0S2	−0.82810789	3.547266167	−3.367090	0.0009214447	0.01080262	−1.0603325	ASF1B	0.528920181	4.102564010	2.942254	0.003668885	0.02936036	−2.307052
158	CCR5	0.96255646	1.67132751	6.092700	6.155573e−09	1.027992e−06	10.120666	GRWD1	0.31880791	3.260343821	3.998232	9.165700e−05	0.002008658	1.0615287	LOC100128531	0.34793824	1.337000245	3.367056	0.0009215541	0.01080262	−1.0604405	UBE2D3	0.188144514	5.583797971	2.942189	0.003669630	0.02936036	−2.307234
159	PRKCG	1.05865048	0.86696928	6.092597	6.158888e−09	1.027992e−06	10.120153	RAPGEF2	0.29401579	2.716895429	3.997285	9.199499e−05	0.002014402	1.0581163	HIST1H2BO	0.45473434	1.043802370	3.366333	0.0009238317	0.01082453	−1.0626870	FAM170A	0.023833186	0.021372365	2.941947	0.003672369	0.02937341	−2.307900
160	BMP8B	−0.71240429	1.42816555	−6.090636	6.222201e−09	1.032069e−06	10.110415	TTC26	0.36027837	1.347301024	3.996273	9.235791e−05	0.002019703	1.0544662	RFPL3−AS1	0.08193227	0.157859731	3.366168	0.0009243537	0.01082587	−1.0632011	OXNAD1	0.217266754	1.969516318	2.941722	0.003674912	0.02938491	−2.308519
161	TRAV14DV4	0.75842269	0.74845557	6.085944	6.376274e−09	1.051056e−06	10.087126	ASCL2	−1.56079000	3.453998589	−3.996186	9.238928e−05	0.002019703	1.0541514	ATP7A	0.22877517	1.379899872	3.365452	0.0009266181	0.01084760	−1.0654277	TPST1	−0.420030144	2.387512438	−2.941095	0.003682027	0.02943293	−2.310247
162	FAM179A	0.17448343	0.20719916	6.076596	6.694526e−09	1.096704e−06	10.040754	ZBED2	0.48353569	0.630821350	3.995624	9.259126e−05	0.002022452	1.0521268	COMMD8	0.27606845	3.681408644	3.364849	0.0009285293	0.01086519	−1.0673027	AX748292	0.080487901	0.264896573	2.940790	0.003685499	0.02945181	−2.311089
163	ZNF416	−0.45237261	1.35869953	−6.054807	7.498003e−09	1.220795e−06	9.932851	FOXP3	0.57528011	1.885895505	3.994777	9.289672e−05	0.002027456	1.0490736	VWA1	−0.50932322	5.046622993	−3.364547	0.0009294868	0.01087160	−1.0682406	TAS2R41	0.019287756	0.015867306	2.940288	0.003691207	0.02948429	−2.312471
164	ACAD8	0.39353040	2.09506572	6.040252	8.086609e−09	1.308601e−06	9.860915	DQ582939	−0.06423416	0.168650316	−3.993622	9.331506e−05	0.002034912	1.0449083	SULT1A2	−0.63169896	1.220768937	−3.363757	0.0009320001	0.01089195	−1.0706979	IGFL2	0.630606742	0.803100699	2.940237	0.003691785	0.02948429	−2.312611
165	NOL3	−0.79308784	2.76255394	−6.020188	8.972803e−09	1.443207e−06	9.761942	C10orf81	1.05783564	2.765334669	3.992920	9.357006e−05	0.002038798	1.0423786	HMGB2	0.43672098	5.791948067	3.363742	0.0009320481	0.01089195	−1.0707447	CD1D	0.220417545	0.662457862	2.940029	0.003694152	0.02949431	−2.313183
166	FUT6	−1.60202204	2.16316148	−6.011255	9.397337e−09	1.502385e−06	9.717948	FAM186A	0.03665138	0.074135906	3.990731	9.436930e−05	0.002053457	1.0344945	APOE	0.97185242	7.564197960	3.363504	0.0009328048	0.01089600	−1.0714832	ECHDC1	0.278198431	4.226858727	2.939184	0.003703792	0.02956239	−2.315511
167	IRF1	1.00325480	4.78973914	5.997179	1.010655e−08	1.606094e−06	9.648712	CEACAM21	0.36331357	0.742318643	3.990654	9.439760e−05	0.002053457	1.0342166	IGFBP4	−0.85276804	7.497592749	−3.362013	0.0009375644	0.01094678	−1.0761143	NCAPG2	0.393819075	2.480286811	2.938777	0.003708441	0.02959059	−2.316632
168	GFI1B	0.62155599	0.32848644	5.989855	1.049605e−08	1.658064e−06	9.612729	LOC389332	−0.98863237	1.344948108	−3.987179	9.568047e−05	0.002078197	1.0217049	FMO3	0.47945738	1.415014528	3.361301	0.0009398485	0.01096034	−1.0783283	SNRNP40	0.212674851	3.820185965	2.938559	0.003710930	0.02960155	−2.317231
169	HLA−DPB2	0.20802944	0.19820375	5.984046	1.081536e−08	1.698396e−06	9.584214	IL4	0.09657929	0.043387957	3.987149	9.569150e−05	0.002078197	1.0215980	TMEM136	−0.19492701	0.596117653	−3.361267	0.0009399546	0.01096034	−1.0784310	LOC100507582	0.192159786	0.820235470	2.937980	0.003717561	0.02964554	−2.318825
170	CTSC	0.84838479	4.60901506	5.982534	1.090002e−08	1.701621e−06	9.576796	SLC25A23	−0.61245424	4.678757439	−3.985743	9.621538e−05	0.002086178	1.0165377	MAT1A	0.48245547	0.448011128	3.361262	0.0009399730	0.01096034	−1.0784489	HS3ST1	0.611501667	3.628747502	2.937533	0.003722687	0.02967750	−2.320056
171	FDXR	0.92064771	2.61685960	5.973163	1.143946e−08	1.775390e−06	9.530837	MIR636	0.31623684	4.098655739	3.985740	9.621621e−05	0.002086178	1.0165297	LOC100507086	0.13066942	0.292759717	3.361136	0.0009403780	0.01096034	−1.0788407	PRKAA2	−0.446913704	0.733604147	−2.937357	0.003724709	0.02968470	−2.320541
172	CLEC12B	0.04404834	0.02909856	5.970666	1.158758e−08	1.787924e−06	9.518597	KLHL35	−0.66798103	1.260650497	−3.985297	9.638189e−05	0.002088064	1.0149351	ARF1	−0.24354301	8.066854529	−3.360489	0.0009424552	0.01097638	−1.0808483	SDCCAG3	0.265218569	3.750873911	2.936989	0.003728935	0.02970945	−2.321554
173	C22orf24	0.15288281	0.20890755	5.960028	1.223980e−08	1.877642e−06	9.466501	PPA1	0.45111146	6.845000774	3.983934	9.689305e−05	0.002097426	1.0100327	TRBV3−1	0.49014790	0.982110946	3.360450	0.0009425815	0.01097638	−1.0809703	C3orf23	0.202893998	2.553053309	2.936175	0.003738294	0.02977509	−2.323793
174	DQ575504	0.10769434	0.11530558	5.956329	1.247493e−08	1.902714e−06	9.448400	AKR1E2	−0.48197346	0.819191241	−3.983335	9.711857e−05	0.002100524	1.0078782	ZNF71	−0.34837607	1.466911917	−3.359784	0.0009447251	0.01099652	−1.0830371	GJB5	−0.756127738	1.220284543	−2.936077	0.003739424	0.02977515	−2.324062
175	TTC3P1	−0.51479655	0.88854637	−5.942972	1.336131e−08	2.023289e−06	9.383104	RAB6B	−0.46334305	1.166624467	−3.983134	9.719445e−05	0.002100524	1.0071544	MT−ND5	−0.53013097	10.902456025	−3.359517	0.0009455857	0.01100171	−1.0838655	DQ574214	0.021505961	0.002029128	2.935466	0.003746468	0.02982229	−2.325743
176	TPD52L1	−1.05715847	1.73852021	−5.942148	1.341794e−08	2.023289e−06	9.379080	MLXIPL	−1.07977419	1.755486138	−3.982270	9.752051e−05	0.002105856	1.0040505	MYO7A	0.39627181	1.222794290	3.357376	0.0009525169	0.01107750	−1.0905100	BC039500	0.198805037	0.922445683	2.935029	0.003751513	0.02985349	−2.326945
177	LTA4H	0.54856638	4.93381918	5.936787	1.379237e−08	2.057686e−06	9.352901	RFPL4A	0.05726841	0.045758149	3.980604	9.815295e−05	0.002116558	0.9980599	C7orf31	0.38429759	1.590979083	3.356356	0.0009558367	0.01111124	−1.0936752	HAGHL	0.517516945	1.886705427	2.934414	0.003758620	0.02990109	−2.328635
178	LOC100272216	0.19460232	0.28424845	5.936238	1.383125e−08	2.057686e−06	9.350223	NKAPP1	0.18774617	0.602036207	3.980471	9.820344e−05	0.002116558	0.9975833	WWTR1	−0.70784436	4.263405341	−3.355948	0.0009571680	0.01111927	−1.0949413	NOSIP	0.257627106	4.440121672	2.933584	0.003768235	0.02996859	−2.330917
179	SLC28A3	1.11411063	1.30153154	5.935572	1.387866e−08	2.057686e−06	9.346969	MFAP3L	−0.50617924	0.829561175	−3.980335	9.825536e−05	0.002116558	0.9970935	SLC34A2	−0.94478607	1.283994988	−3.355887	0.0009573662	0.01111927	−1.0951297	MAP2K4	0.250710774	3.067886963	2.933442	0.003769886	0.02997206	−2.331308
180	AIPL1	0.05879730	0.02720971	5.932735	1.408214e−08	2.076255e−06	9.333125	ANKRD36BP2	0.33047108	0.444963115	3.977684	9.927034e−05	0.002136687	0.9875700	TTC27	0.23054125	2.677971974	3.355287	0.0009593270	0.01113717	−1.0969910	MIR4799	0.053305837	0.026782439	2.933352	0.003770930	0.02997206	−2.331555
181	RASGEF1B	0.57004130	1.47037575	5.928024	1.442649e−08	2.115275e−06	9.310146	PROS1	−0.73277119	2.689478186	−3.977364	9.939340e−05	0.002137139	0.9864219	PSME1	0.37653809	7.036691260	3.355131	0.0009598358	0.01113821	−1.0974733	ICT1	0.256849721	4.574464038	2.933173	0.003773010	0.02997961	−2.332047
182	C19orf38	0.73012616	1.16801865	5.926495	1.454003e−08	2.120208e−06	9.302690	NPL	0.51344645	2.403300176	3.977211	9.945236e−05	0.002137139	0.9858724	GNL1	−0.23791228	2.769700096	−3.354880	0.0009606582	0.01114129	−1.0982524	LOC286094	0.008829082	0.005072076	2.932484	0.003781016	0.03003423	−2.333940
183	YBX2	−1.49302505	2.28118006	−5.922346	1.485256e−08	2.153945e−06	9.282464	BC068088	0.23231069	0.732200969	3.973866	1.007496e−04	0.002163263	0.9738645	MLH3	0.20402998	1.619281917	3.354794	0.0009609411	0.01114129	−1.0985202	TNFAIP8	0.454160703	3.018149526	2.932208	0.003784224	0.03005073	−2.334697
184	LOC100506776	0.44983245	0.58677084	5.917618	1.521668e−08	2.194758e−06	9.259429	TMEM192	−0.36865496	2.281189382	−3.973003	1.010870e−04	0.002168754	0.9707659	ADAT2	0.25421295	1.093974344	3.353665	0.0009646427	0.01117793	−1.1020175	AF007131	0.272056795	1.498416898	2.930911	0.003799358	0.03015914	−2.338261
185	LOC100131733	0.87757629	1.69856050	5.897759	1.684391e−08	2.416328e−06	9.162812	RAB34	−0.84116684	4.112240316	−3.971950	1.015001e−04	0.002175858	0.9669872	NUDT4	0.42319957	3.518503874	3.353574	0.0009649435	0.01117793	−1.1023012	HMGN1	0.306119129	5.452389430	2.930843	0.003800149	0.03015914	−2.338447
186	CD160	0.25298693	0.41424966	5.895739	1.701872e−08	2.428278e−06	9.152994	BMP7	−1.09234844	1.687859162	−3.971513	1.016718e−04	0.002177780	0.9654213	PLEC	−0.52690059	6.136662960	−3.353187	0.0009662176	0.01118354	−1.1035014	ZNF668	0.173440344	2.289922077	2.930609	0.003802895	0.03017190	−2.339092
187	TMEM244	0.10827018	0.08251960	5.882820	1.817914e−08	2.579980e−06	9.090274	KPNA4	−0.31500732	3.755662314	−3.971178	1.018038e−04	0.002178849	0.9642191	LOC641298	0.05282272	0.245444027	3.353171	0.0009662702	0.01118354	−1.1035510	KCNJ3	−0.616558225	0.969404413	−2.930361	0.003805786	0.03017508	−2.339770
188	GPR25	0.90229937	1.07423772	5.878205	1.861225e−08	2.627397e−06	9.067887	MRPS18C	0.28864024	3.974746843	3.969697	1.023892e−04	0.002189610	0.9589074	SNORD33	0.31629055	1.252712748	3.352855	0.0009673104	0.01119069	−1.1045296	SPERT	−0.190908221	0.185799999	−2.930290	0.003806623	0.03017508	−2.339966
189	C5orf63	−0.33259803	0.68330247	−5.874032	1.901243e−08	2.669687e−06	9.047662	IRF2	0.32816717	4.507443075	3.969461	1.024824e−04	0.002189840	0.9580640	BRI3BP	0.57846575	4.483501877	3.352571	0.0009682460	0.01119664	−1.1054090	C17orf85	0.186879515	2.656245170	2.930199	0.003807688	0.03017508	−2.340216
190	AK096803	−1.77326310	2.99191211	−5.868078	1.959810e−08	2.724971e−06	9.018816	CNEP1R1	0.25006084	2.438285539	3.969211	1.025819e−04	0.002190202	0.9571653	SLAMF1	0.34495190	0.829099914	3.352409	0.0009687813	0.01119728	−1.1059117	CCT8	0.281701868	5.944760337	2.930186	0.003807842	0.03017508	−2.340252
191	ADAMDEC1	1.24115782	2.37615948	5.867944	1.961149e−08	2.724971e−06	9.018167	SLBP	0.41303708	5.018554786	3.968337	1.029291e−04	0.002195849	0.9540347	MLKL	0.37412883	3.293571993	3.352298	0.0009691457	0.01119728	−1.1062537	DQ584035	0.037943209	0.008350028	2.930038	0.003809578	0.03017981	−2.340659
192	HOXA6	−0.72153405	0.97134181	−5.859138	2.051081e−08	2.835086e−06	8.975542	SCARA3	−0.95025243	2.586338466	−3.967699	1.031838e−04	0.002199515	0.9517448	HIST1H3B	0.33672749	0.709117268	3.352042	0.0009699913	0.01119737	−1.1070470	SRPX	−0.755759984	2.450350852	−2.929894	0.003811266	0.03018418	−2.341055
193	CCL5	1.67235822	4.97845665	5.847399	2.177276e−08	2.988222e−06	8.918783	SNX20	0.43976267	1.015204317	3.966135	1.038100e−04	0.002209973	0.9461400	ZNF826P	0.25050039	0.383183912	3.352041	0.0009699972	0.01119737	−1.1070525	TM7SF3	0.455139539	4.330636560	2.929492	0.003815975	0.03020162	−2.342157
194	RAB11FIP5	−0.60826106	3.04815365	−5.846757	2.184389e−08	2.988222e−06	8.915682	MAPK8IP1	−0.76209126	1.774577803	−3.965904	1.039027e−04	0.002209973	0.9453134	BC045769	0.31150641	0.763341898	3.351229	0.0009726828	0.01121176	−1.1095674	BC025370	−0.387042680	0.426405004	−2.929452	0.003816444	0.03020162	−2.342267
195	PODXL2	−1.59360132	3.94924709	−5.843554	2.220236e−08	3.021684e−06	8.900210	FABP1	−2.43467596	3.329974237	−3.965850	1.039243e−04	0.002209973	0.9451207	COTL1	0.56336983	6.846587035	3.351197	0.0009727894	0.01121176	−1.1096671	IKBKG	0.101261310	0.707017190	2.929415	0.003816882	0.03020162	−2.342369
196	CDH17	−2.83821466	4.06170900	−5.839384	2.267765e−08	3.070623e−06	8.880076	CLCN5	0.45151499	1.610784245	3.965441	1.040887e−04	0.002211697	0.9436562	C20orf195	−0.29341357	0.491022873	−3.351179	0.0009728475	0.01121176	−1.1097214	PYCR2	−0.405810635	4.551032751	−2.929081	0.003820797	0.03022358	−2.343284
197	FDXACB1	0.37942603	1.07965939	5.832153	2.352546e−08	3.169249e−06	8.845190	MAP2K6	0.53393741	1.325389563	3.963373	1.049242e−04	0.002227667	0.9362498	ZBTB39	0.29652128	1.794802747	3.351153	0.0009729332	0.01121176	−1.1098015	GLMN	0.251891389	1.861028639	2.928511	0.003827504	0.03026762	−2.344850
198	IL18BP	0.89960074	3.18106120	5.820343	2.497739e−08	3.347854e−06	8.788270	HNRNPA1	0.31008067	5.665004377	3.962748	1.051782e−04	0.002231274	0.9340103	RNF4	0.21467165	4.161570840	3.349673	0.0009778492	0.01126352	−1.1143855	HIPK2	0.417933859	3.445497103	2.927832	0.003835499	0.03032181	−2.346713
199	LOC440131	0.03900025	0.01477965	5.815642	2.557942e−08	3.410741e−06	8.765635	CDKN2AIPNL	0.33481442	3.517877220	3.961972	1.054941e−04	0.002235068	0.9312324	RXFP4	−0.29845258	0.441469384	−3.348946	0.0009802736	0.01128654	−1.1166376	DOHH	0.237781316	3.263670477	2.927071	0.003844477	0.03037815	−2.348800
200	JAK2	0.97720657	2.70838472	5.814685	2.570361e−08	3.410741e−06	8.761032	SYPL1	0.39992946	5.886781133	3.961895	1.055254e−04	0.002235068	0.9309571	IPO13	−0.20499095	3.218882510	−3.348105	0.0009830814	0.01131384	−1.1192388	MAP6D1	−0.251962217	0.929941511	−2.926954	0.003845859	0.03037815	−2.349121

	Gene	logFC	AveExpr	t	P.Value	adj.P.Val	B	Gene	logFC	AveExpr	t	P.Value	adj.P.Val	B	Gene	logFC	AveExpr	t	P.Value	adj.P.Val	B	Gene	logFC	AveExpr	t	P.Value	adj.P.Val	B
201	SUCNR1	0.97023623	1.04351428	5.807043	2.671746e−08	3.527635e−06	8.724268	DQ570835	0.05402425	0.115978534	3.960316	0.0001061713	0.002246953	0.9253055	OLFM1	−0.521271486	1.354774709	−3.347981	0.0009834968	0.01131384	−1.119623	DQ590971	0.009990129	0.002113954	2.926937	0.003846059	0.03037815	−2.349167
202	FCGR1A	0.68165963	1.17678371	5.800505	2.761580e−08	3.628197e−06	8.692842	CNPY2	0.38292933	5.252981523	3.959312	0.0001065836	0.002253883	0.9217148	ZNF605	−0.337081381	1.120148872	−3.347295	0.0009857967	0.01133538	−1.121747	PBX1	−0.580074779	3.126158147	−2.926760	0.003848161	0.03038570	−2.349655
203	FAM114A1	−0.65861129	3.96694925	−5.790390	2.906414e−08	3.799671e−06	8.644270	EBI3	0.70047353	1.728278033	3.958933	0.0001067398	0.002255388	0.9203586	NAP1L4	0.215926146	4.380338077	3.346969	0.0009868892	0.01134240	−1.122755	POLR3K	0.316486146	3.925468214	2.926545	0.003850700	0.03039671	−2.350244
204	B2M	0.91059831	10.67383732	5.787940	2.942608e−08	3.821792e−06	8.632510	TM4SF20	−1.54796222	2.246767157	−3.958546	0.0001068996	0.002256968	0.9189728	WDHD1	0.374376230	1.668234931	3.346821	0.0009873872	0.01134240	−1.123213	BC021061	0.289733251	0.804797846	2.925996	0.003857199	0.03043896	−2.351749
205	C10orf116	−1.72230181	4.89016943	−5.787299	2.952136e−08	3.821792e−06	8.629439	ZNF155	0.34028341	1.400635518	3.958292	0.0001070045	0.002257387	0.9180643	PPP3R2	0.030909769	0.008965007	3.346618	0.0009880700	0.01134240	−1.123842	DJ417906	0.734222939	6.434055027	2.925649	0.003861309	0.03046233	−2.352700
206	SIDT1	0.83730112	1.40412629	5.776022	3.124993e−08	4.025932e−06	8.575374	SYCP3	0.52648785	1.992641370	3.957426	0.0001073629	0.002261801	0.9149672	ALYREF	0.350493681	5.555103733	3.346604	0.0009881168	0.01134240	−1.123885	STEAP1	0.609163791	3.772751742	2.925548	0.003862518	0.03046281	−2.352979
207	LOC439990	−0.90063997	1.90393213	−5.770678	3.210332e−08	4.115894e−06	8.549777	ZNF195	0.33868075	2.670102805	3.957326	0.0001074044	0.002261801	0.9146094	RPS2	0.346829762	8.491052528	3.346014	0.0009901005	0.01136026	−1.125709	PRIM2	0.255907681	2.067100647	2.925227	0.003866320	0.03048374	−2.353857
208	HENMT1	−0.86112680	2.34062710	−5.769489	3.229627e−08	4.120724e−06	8.544084	TPPP3	−1.17512187	3.678982828	−3.957169	0.0001074694	0.002261801	0.9140489	VPS29	0.235839377	4.659454312	3.345260	0.0009926451	0.01138047	−1.128043	SRSF1	0.187093603	4.989755813	2.924727	0.003872261	0.03052151	−2.355228
209	MDM2	0.90264654	3.65495437	5.763612	3.326657e−08	4.224218e−06	8.515963	CCBL2	0.34769992	3.340607870	3.954541	0.0001085654	0.002283055	0.9046521	BAG3	−0.493189464	4.935190713	−3.345237	0.0009927201	0.01138047	−1.128112	ZFP3	−0.339762014	1.384242278	−2.924532	0.003874585	0.03053076	−2.355763
210	LOC344967	0.11482764	0.15974256	5.762040	3.353090e−08	4.237507e−06	8.508444	RHEBL1	0.36545679	0.909996913	3.953749	0.0001088974	0.002288225	0.9018240	AF339817	−0.591598264	1.519541443	−3.344997	0.0009935319	0.01138486	−1.128855	TRNA_Phe	0.027479604	0.032425803	2.924211	0.003878402	0.03055177	−2.356642
211	NKG7	1.50340549	3.78554341	5.759221	3.401017e−08	4.277705e−06	8.494962	PTGES3	0.32427278	6.196327438	3.953270	0.0001090987	0.002290641	0.9001134	MIR936	0.603088719	0.766010086	3.344582	0.0009949368	0.01139604	−1.130140	LIMCH1	−0.346254533	1.433104830	−2.923691	0.003884607	0.03059157	−2.358069
212	SESN2	0.77445306	3.07419317	5.757311	3.433868e−08	4.298652e−06	8.485830	TIGIT	0.62054107	1.264952805	3.951538	0.0001098301	0.002302360	0.8939258	AX747894	0.018318777	0.016694476	3.344229	0.0009961297	0.01140478	−1.131229	SNORA8	0.311461939	2.381795586	2.923486	0.003887046	0.03059920	−2.358629
213	FUT3	−1.94263980	3.75228962	−5.756359	3.450359e−08	4.299018e−06	8.481279	TMED5	0.31714070	3.907454179	3.951538	0.0001098304	0.002302360	0.8939234	DNASE1L2	0.219439012	0.488507767	3.343341	0.0009991411	0.01143433	−1.133974	ZNF282	0.207785826	3.632256570	2.923414	0.003887909	0.03059920	−2.358827
214	PSMB10	0.83934526	3.79872295	5.750623	3.551347e−08	4.404169e−06	8.453876	CCR2	0.43151748	0.785942114	3.951220	0.0001099649	0.002302865	0.8927897	CCDC104	0.306572731	4.204342684	3.342429	0.0010022451	0.01146216	−1.136795	NEB	0.433482941	0.567543756	2.923320	0.003889036	0.03059920	−2.359086
215	IKZF2	0.45898791	0.84903020	5.743669	3.677672e−08	4.539616e−06	8.420675	PKP4	0.35852710	3.767849134	3.951072	0.0001100280	0.002302865	0.8922584	MIRLET7G	0.089846097	0.050148669	3.342373	0.0010024367	0.01146216	−1.136968	DQ588801	0.016030794	0.001703613	2.922775	0.003895541	0.03064130	−2.360577
216	ACER3	0.49262865	2.90206099	5.742317	3.702742e−08	4.549401e−06	8.414222	FBXO7	0.19465911	3.677982552	3.950532	0.0001102570	0.002305840	0.8903328	CLEC4C	0.090250527	0.099273646	3.341674	0.0010048178	0.01148444	−1.139126	UFL1	0.215268709	3.777442949	2.922450	0.003899433	0.03066230	−2.361469
217	AX747531	0.14807651	0.24944158	5.722328	4.093411e−08	4.987494e−06	8.318956	TMPRSS9	0.11986090	0.375242448	3.947076	0.0001117358	0.002334927	0.8779952	C21orf91	0.259020229	1.668672686	3.339965	0.0010106695	0.01154634	−1.144405	MAP9	−0.337689262	1.086556293	−2.922359	0.003900521	0.03066230	−2.361717
218	A2LD1	−0.72513457	2.11042472	−5.722158	4.096890e−08	4.987494e−06	8.318150	DQ576037	0.03418585	0.005687487	3.946705	0.0001118957	0.002336429	0.8766711	PTMA	0.296836680	9.107276493	3.339608	0.0010118986	0.01155541	−1.145510	MRPS22	0.255116042	4.520133639	2.921674	0.003908727	0.03071771	−2.363593
219	CD226	0.44266568	0.62889637	5.716831	4.207725e−08	5.099032e−06	8.292799	ARHGEF10	−0.49909042	1.708603518	−3.945469	0.0001124297	0.002345732	0.8722632	XRCC2	0.378109069	1.384375210	3.338474	0.0010158009	0.01159498	−1.149009	IFNE	0.248258145	0.242223333	2.921464	0.003911247	0.03072842	−2.364168
220	C4orf36	0.38048843	0.57531075	5.714395	4.259379e−08	5.138167e−06	8.281212	KIAA0753	0.28255487	2.102610293	3.945061	0.0001126066	0.002346092	0.8708074	CHAF1A	0.404351249	3.130644836	3.337259	0.0010200006	0.01163382	−1.152760	ZIC5	−0.492942500	0.629362574	−2.921095	0.003915684	0.03075417	−2.365180
221	GBP1	1.26074991	4.48103977	5.711495	4.321668e−08	5.182739e−06	8.267426	CACNG4	−0.88514227	0.909642928	−3.945022	0.0001126237	0.002346092	0.8706664	RAB13	−0.375818244	4.945859376	−3.337236	0.0010200802	0.01163382	−1.152831	LOC100506241	0.013894489	0.007099907	2.920308	0.003925142	0.03081933	−2.367333
222	IL21	0.27962660	0.17374075	5.710862	4.335386e−08	5.182739e−06	8.264416	ZW10	0.32347305	2.922860167	3.944696	0.0001127650	0.002346463	0.8695061	C3AR1	0.603690775	2.992200640	3.336353	0.0010231423	0.01165522	−1.155556	MGC13053	0.029306225	0.038790370	2.920041	0.003928359	0.03083229	−2.368064
223	C5orf56	0.84529996	3.64724462	5.706794	4.424543e−08	5.254597e−06	8.245086	CCDC153	0.31279535	0.807712907	3.944373	0.0001129055	0.002346463	0.8683524	RWDD2B	0.322925265	3.130225898	3.336326	0.0010232355	0.01165522	−1.155638	BC035080	0.216821497	2.247667196	2.919945	0.003929518	0.03083229	−2.368327
224	NINL	−0.83162708	1.37359623	−5.706318	4.435094e−08	5.254597e−06	8.242825	SELM	−0.85041658	6.221675391	−3.944370	0.0001129068	0.002346463	0.8683421	CCNE1	−1.009965312	2.682994584	−3.336315	0.0010232735	0.01165522	−1.155672	MIR3914−2	0.021469899	0.004710407	2.919852	0.003930635	0.03083229	−2.368580
225	MIR4754	0.28222024	0.32692090	5.703319	4.502133e−08	5.310316e−06	8.228579	RAD51C	−0.32408616	2.274343276	−3.944150	0.0001130027	0.002346619	0.8675558	NCRUPAR	0.041166768	0.028570111	3.335923	0.0010246386	0.01166576	−1.156884	CRYBG3	−0.277239985	2.784716790	−2.919558	0.003934187	0.03083229	−2.369387
226	NDRG1	−0.84870958	5.21602270	−5.699550	4.587786e−08	5.371257e−06	8.210684	CKMT1B	−0.68597117	2.165957555	−3.942780	0.0001136003	0.002355762	0.8626720	MIR4318	0.019057373	0.002747905	3.334427	0.0010298513	0.01172008	−1.161497	RAB8B	0.304023393	2.857271410	2.919502	0.003934865	0.03083229	−2.369540
227	RCOR2	−0.97218981	1.58006779	−5.698869	4.603423e−08	5.371257e−06	8.207453	AX746478	−0.32489393	1.202712971	−3.942734	0.0001136206	0.002355762	0.8625073	DQ599950	−0.147329958	0.164032229	−3.334068	0.0010311069	0.01172584	−1.162604	SELT	0.220895933	4.589235792	2.919490	0.003935002	0.03083229	−2.369571
228	TSHR	0.12475029	0.12712174	5.698388	4.614517e−08	5.371257e−06	8.205167	GPR65	0.39518447	1.012446508	3.941679	0.0001140826	0.002363496	0.8587498	FAM63B	−0.291914237	2.114349970	−3.333979	0.0010314188	0.01172584	−1.162879	ASB13	−0.306886942	3.312391328	−2.919476	0.003935175	0.03083229	−2.369611
229	C14orf105	0.83672238	0.57042537	5.693904	4.719086e−08	5.468988e−06	8.183891	TMEM55A	−0.58877147	2.211759380	−3.940807	0.0001144664	0.002368900	0.8556402	TGM2	0.777544645	5.675920074	3.333903	0.0010316830	0.01172584	−1.163112	DQ582443	−0.381268452	3.138259105	−2.919397	0.003936130	0.03083229	−2.369827
230	AJ606331	0.06962958	0.09129545	5.684582	4.943929e−08	5.704649e−06	8.139700	SNORA10	0.47843556	1.865346975	3.940663	0.0001145299	0.002368900	0.8551265	MTHFD1	0.349572067	4.548059150	3.333100	0.0010344965	0.01175278	−1.165587	P2RY2	−0.425113665	1.239603060	−2.919304	0.003937248	0.03083229	−2.370080
231	UBXN10	−1.08346182	1.71716504	−5.674346	5.202906e−08	5.950779e−06	8.091228	CBFB	0.36647378	4.186280914	3.940478	0.0001146112	0.002368900	0.8544696	EIF2S2	0.323978660	5.157121959	3.332882	0.0010352638	0.01175647	−1.166261	TARS2	0.259913126	3.704781711	2.918504	0.003946921	0.03089625	−2.372270
232	IGKV3−11	0.06613005	0.03184738	5.673328	5.229373e−08	5.950779e−06	8.086411	EIF2B1	0.23594664	4.394385965	3.939478	0.0001150532	0.002375445	0.8509061	SNHG12	0.354420690	2.152181503	3.330500	0.0010436569	0.01184671	−1.173600	C6orf228	−0.234216994	2.032377997	−2.918339	0.003948917	0.03089625	−2.372721
233	CDX2	−2.09461350	3.25198320	−5.673259	5.231178e−08	5.950779e−06	8.086083	CXorf28	0.07225892	0.068095474	3.939357	0.0001151069	0.002375445	0.8504740	CAAP1	0.356427257	3.015157835	3.329369	0.0010476666	0.01188616	−1.177085	C16orf87	0.224111616	2.398696296	2.918301	0.003949383	0.03089625	−2.372826
234	RIPK3	−0.71465914	2.69300253	−5.672655	5.246928e−08	5.950779e−06	8.083229	LOC389043	0.07000945	0.041583891	3.938697	0.0001153996	0.002377241	0.8481232	UBE2Q2P1	−0.266107465	0.816082979	−3.329267	0.0010480280	0.01188616	−1.177399	RPL23P8	0.093089248	0.178170571	2.918146	0.003951258	0.03089625	−2.373250
235	CD40	1.01463670	2.65583183	5.667203	5.391405e−08	6.088617e−06	8.057443	ABHD11	−0.67515476	4.697867084	−3.938647	0.0001154220	0.002377241	0.8479435	ZNF581	0.365938126	3.597369003	3.327800	0.0010532491	0.01194027	−1.181915	POLR2D	0.204588157	3.781223168	2.918072	0.003952155	0.03089625	−2.373452
236	UBASH3A	0.60660574	0.85857138	5.664076	5.476016e−08	6.157966e−06	8.042662	MIR4483	0.32396192	0.346381604	3.938555	0.0001154627	0.002377241	0.8476173	RBM45	0.168113321	2.225230281	3.327238	0.0010552580	0.01195382	−1.183646	ANGEL2	−0.163506413	2.301337556	−2.918052	0.003952401	0.03089625	−2.373508
237	MS4A14	0.43052526	0.75677657	5.659098	5.613391e−08	6.285814e−06	8.019143	CCL17	0.49352190	0.892212959	3.937980	0.0001157185	0.002380661	0.8455685	MNX1	0.479098474	1.769541771	3.327213	0.0010553446	0.01195382	−1.183721	LOC100288069	0.093122439	0.339223798	2.917466	0.003959499	0.03094024	−2.375109
238	HPS5	0.42726041	2.33681842	5.656071	5.698585e−08	6.354401e−06	8.004845	ODF4	0.03722013	0.018508971	3.937560	0.0001159055	0.002382146	0.8440737	RGS19	0.494948803	3.701388782	3.326416	0.0010581995	0.01197727	−1.186176	LRP12	−0.188955742	0.886235486	−2.917395	0.003960360	0.03094024	−2.375303
239	ACSM5	0.19864051	0.23112429	5.651845	5.819618e−08	6.462211e−06	7.984897	TCRBV12S3	0.41138129	0.419098666	3.937204	0.0001160645	0.002382146	0.8428044	P2RY10	0.660686808	1.521792236	3.326352	0.0010584294	0.01197727	−1.186373	MXI1	−0.380567101	3.175751742	−2.916844	0.003967058	0.03097974	−2.376810
240	TXNRD3NB	−0.43319946	0.83962301	−5.646542	5.975049e−08	6.607159e−06	7.959880	PRKY	0.39012723	0.571635868	3.937119	0.0001161024	0.002382146	0.8425018	BC033962	0.008886259	0.002621702	3.326257	0.0010587693	0.01197727	−1.186665	FOXF2	−0.657913766	3.008280077	−2.916787	0.003967751	0.03097974	−2.376966
241	LOC338739	0.06990613	0.06768977	5.635474	6.312609e−08	6.951466e−06	7.907722	UTP18	0.36594128	3.886674638	3.937013	0.0001161497	0.002382146	0.8421251	SLC25A27	−0.297021960	0.614883528	−3.325852	0.0010602222	0.01198860	−1.187911	ZBTB7C	−0.678049091	1.860677130	−2.916088	0.003976258	0.03103703	−2.378877
242	APC2	0.35977419	0.56727412	5.632747	6.398618e−08	7.017063e−06	7.894879	ISYNA1	−0.72024445	3.331365972	−3.936239	0.0001164959	0.002385540	0.8393701	KIAA0825	0.075566722	0.289572954	3.325379	0.0010619225	0.01199788	−1.189367	NUDT10	−0.170462726	0.292413157	−2.915782	0.003979991	0.03105704	−2.379714
243	SLC9A7	0.97076267	2.29033669	5.627468	6.568327e−08	7.151352e−06	7.870037	DUOX1	−0.72463341	1.339008680	−3.936043	0.0001165838	0.002385540	0.8386720	PIK3R5	0.477200940	1.641682637	3.325372	0.0010619470	0.01199788	−1.189388	MICAL2	−0.416646533	3.837680835	−2.915670	0.003981362	0.03105747	−2.380021
244	TTC22	−0.96200755	2.63286685	−5.626777	6.590856e−08	7.151352e−06	7.866788	PNPLA8	−0.28321344	3.090843564	−3.936040	0.0001165849	0.002385540	0.8386632	DPY19L1P2	0.136601343	0.312194640	3.325007	0.0010632603	0.01199894	−1.190511	DJ417913	0.739187964	6.334218435	2.915585	0.003982387	0.03105747	−2.380251
245	PEX3	0.47093974	2.55724091	5.626439	6.601912e−08	7.151352e−06	7.865197	FYB	0.81157074	3.065359412	3.935635	0.0001167666	0.002387366	0.8372212	COL1A1	−0.849547556	9.034709348	−3.325001	0.0010632811	0.01199894	−1.190529	HNRNPF	0.277784016	6.779362585	2.915258	0.003986381	0.03107491	−2.381145
246	PCYOX1	−0.54361231	4.13017697	−5.623907	6.685301e−08	7.212244e−06	7.853286	TRBV5−1	0.64563764	1.213421826	3.935441	0.0001168540	0.002387366	0.8365284	ZDHHC14	0.287479136	1.775699923	3.324969	0.0010633978	0.01199894	−1.190629	HOXB13	−0.683874968	1.743470068	−2.915211	0.003986965	0.03107491	−2.381276
247	C7orf69	0.08926841	0.06636722	5.621719	6.758188e−08	7.261358e−06	7.842996	ADM	−0.75259213	3.044515773	−3.934738	0.0001171701	0.002390802	0.8340280	MOCS1	−0.481107049	2.172492457	−3.324748	0.0010641931	0.01200281	−1.191308	TMEM128	0.204078510	1.859934786	2.914988	0.003989689	0.03108701	−2.381885
248	D21847	0.44158279	0.36131803	5.619161	6.844341e−08	7.324272e−06	7.830976	NSUN5	−0.39311742	3.682934214	−3.934667	0.0001172024	0.002390802	0.8337730	LOC729020	0.029999880	0.047371386	3.324538	0.0010649498	0.01200387	−1.191954	C3orf77	0.007819433	0.004881730	2.914732	0.003992815	0.03110223	−2.382584
249	LACTB2	−1.00619747	4.54914928	−5.612945	7.058289e−08	7.522889e−06	7.801769	TRAFD1	0.36331854	3.529539266	3.933541	0.0001177108	0.002399328	0.8297663	CENPW	0.524127096	4.119978062	3.324471	0.0010651915	0.01200387	−1.192160	MOB1A	0.254024017	4.821821937	2.914157	0.003999847	0.03114787	−2.384154
250	KDELC2	0.62186237	3.48909207	5.601094	7.484363e−08	7.945100e−06	7.746157	HMGN2	0.39160305	5.014430882	3.932799	0.0001180467	0.002403305	0.8271279	NOL8	0.251196537	2.688574699	3.324035	0.0010667649	0.01201650	−1.193501	MYEF2	−0.438720310	0.885234999	−2.913079	0.004013084	0.03124179	−2.387101
251	MAPRE3	−0.80770543	2.34058316	−5.597324	7.625068e−08	8.062218e−06	7.728486	LOC220906	−0.32823206	1.992521392	−3.932710	0.0001180870	0.002403305	0.8268125	UBE3D	0.171715603	0.735010862	3.323807	0.0010675883	0.01202067	−1.194202	GINS2	0.538181987	3.195222368	2.912928	0.004014930	0.03124699	−2.387511
252	APOL4	0.79984590	2.01989011	5.593862	7.756602e−08	8.168748e−06	7.712260	PRMT1	0.35129909	5.531174217	3.931963	0.0001184266	0.002408371	0.8241538	COL5A1	−0.663717481	5.335000611	−3.323266	0.0010695445	0.01203759	−1.195866	SPP1	−1.127888993	5.072249869	−2.912764	0.004016956	0.03125231	−2.387961
253	CD274	0.97307635	1.26366688	5.592507	7.808651e−08	8.191058e−06	7.705916	SNORA27	0.19371926	0.597632814	3.931328	0.0001187159	0.002412406	0.8218953	METTL12	0.251225016	1.263748115	3.323000	0.0010705064	0.01204331	−1.196682	ACPT	0.087105593	0.142385273	2.912681	0.004017969	0.03125231	−2.388186
254	C19orf54	0.56944964	2.86926078	5.587463	8.005508e−08	8.364496e−06	7.682296	TP53	0.70658286	4.377956264	3.930805	0.0001189545	0.002415405	0.8200371	JA611291	0.020445451	0.001729024	3.321138	0.0010772722	0.01211429	−1.202406	CAMKK1	−0.244251376	1.370305383	−2.912523	0.004019920	0.03125832	−2.388619
255	H2BFXP	0.05956111	0.05020139	5.581827	8.231196e−08	8.566577e−06	7.655923	NCK1	0.34509453	3.801325261	3.929918	0.0001193606	0.002419867	0.8168827	MYOM3	−0.478150559	0.668392501	−3.320938	0.0010780000	0.01211734	−1.203020	CADPS	−0.573641685	0.761291472	−2.912074	0.004025450	0.03128384	−2.389845
256	LOC100128420	0.41750472	0.48230147	5.576290	8.458978e−08	8.769250e−06	7.630030	ZNF79	0.21629401	1.727590643	3.929839	0.0001193968	0.002419867	0.8166022	ZNF304	0.345766634	1.584289721	3.320269	0.0010804430	0.01213966	−1.205077	ARID2	0.207256476	2.324270378	2.912065	0.004025559	0.03128384	−2.389869
257	ARPP21	0.25579651	0.36198132	5.569611	8.741919e−08	8.992295e−06	7.598822	LOC400643	−0.94947918	1.178021747	−3.929728	0.0001194478	0.002419867	0.8162064	LOC100506083	0.205444476	1.749824327	3.319766	0.0010822800	0.01215224	−1.206620	BC033164	0.056061557	0.125208844	2.911623	0.004031004	0.03131698	−2.391075
258	COX20	−0.56439176	4.27337629	−5.569307	8.754998e−08	8.992295e−06	7.597404	TCRBV3S1	1.08774173	4.210042922	3.928953	0.0001198038	0.002425227	0.8134522	LOC100128788	0.112691373	0.362198478	3.319712	0.0010824787	0.01215224	−1.206787	IKZF1	0.583514730	1.907854247	2.911430	0.004033386	0.03132632	−2.391602
259	SYBU	−0.85938212	2.22102590	−5.568826	8.775781e−08	8.992295e−06	7.595155	ANKRD9	−0.41606045	3.946712353	−3.928265	0.0001201207	0.002429788	0.8110075	LINC00294	−0.329701351	2.193099663	−3.319429	0.0010835169	0.01215390	−1.207658	5_8S_rRNA	0.064141809	0.048666493	2.911148	0.004036872	0.03134181	−2.392372
260	FAM188A	−0.50230591	2.88907956	−5.565968	8.900129e−08	9.084636e−06	7.581809	UIMC1	0.24755095	2.316587388	3.927072	0.0001206721	0.002439083	0.8067685	OR6A2	0.018142818	0.011182635	3.319241	0.0010842051	0.01215390	−1.208234	PSMD5	−0.258828397	3.960618151	−2.911077	0.004037743	0.03134181	−2.392565
261	AMH	1.05300357	1.11612087	5.562433	9.056303e−08	9.208629e−06	7.565311	TRGV3	0.40045302	0.711432088	3.925805	0.0001212608	0.002449117	0.8022644	LZTS2	−0.361893336	3.469901244	−3.319205	0.0010843351	0.01215390	−1.208343	LAPTM4A	−0.265113739	6.977987382	−2.910600	0.004043643	0.03137843	−2.393867
262	SCML4	0.52985169	0.62409489	5.560960	9.122146e−08	9.227008e−06	7.558440	TM6SF2	−1.02459802	1.683033937	−3.925551	0.0001213788	0.002449635	0.8013643	IL10RA	0.659714487	2.545268129	3.319172	0.0010844579	0.01215390	−1.208446	WLS	−0.580771366	4.306174000	−2.910302	0.004047339	0.03139794	−2.394682
263	WDR64	0.02627626	0.03061018	5.559990	9.165781e−08	9.227008e−06	7.553914	DCUN1D3	0.33219523	1.868731007	3.925259	0.0001215147	0.002450265	0.8003282	U2AF2	0.255168844	5.754349239	3.318733	0.0010860685	0.01216421	−1.209794	BC087859	−0.247410655	0.623266440	−2.910117	0.004049627	0.03140444	−2.395186
264	C9orf129	−0.58051905	0.85982015	−5.559673	9.180077e−08	9.227008e−06	7.552436	MEIS3P1	−0.57956148	1.849929066	−3.925088	0.0001215946	0.002450265	0.7997201	NXF4	0.008923778	0.004170170	3.318671	0.0010862948	0.01216421	−1.209984	CD72	0.455543283	1.487956555	2.910043	0.004050544	0.03140444	−2.395388
265	F12	−1.12603354	2.05391445	−5.558361	9.239499e−08	9.227008e−06	7.546317	REG4	−2.39187710	5.212610315	−3.924402	0.0001219151	0.002453131	0.7972837	TBCEL	−0.333344788	1.645532612	−3.318185	0.0010880819	0.01217438	−1.211477	UCP2	0.589298060	6.162388882	2.909897	0.004052350	0.03140927	−2.395786
266	LOC100306975	0.04373425	0.04083593	5.558169	9.248216e−08	9.227008e−06	7.545423	TECTB	0.06168766	0.039910261	3.924388	0.0001219217	0.002453131	0.7972338	CRABP1	−1.007690878	1.178157455	−3.317856	0.0010892904	0.01217438	−1.212485	KCNJ1	0.018632445	0.029570206	2.909104	0.004062194	0.03147637	−2.397949
267	BAIAP2	−0.79537284	2.92878926	−5.545738	9.830762e−08	9.771483e−06	7.487491	BAI2	−0.51808528	0.950959709	−3.922934	0.0001226036	0.002464982	0.7920722	MOAP1	−0.325339503	3.312396924	−3.317851	0.0010893091	0.01217438	−1.212501	PIGY	0.190352469	5.373860286	2.908906	0.004064651	0.03148621	−2.398488
268	RAB36	−0.73522104	1.55259177	−5.544497	9.890825e−08	9.792896e−06	7.481715	FUT8	0.46210930	3.484162982	3.920385	0.0001238082	0.002487317	0.7830241	PARVG	0.493397572	1.856017231	3.317739	0.0010897239	0.01217438	−1.212847	MIR3914−1	0.021258812	0.004695092	2.908600	0.004068465	0.03150657	−2.399325
269	HV303525	1.03443026	2.98703372	5.543772	9.926105e−08	9.792896e−06	7.478339	LOC100271722	−0.21461815	0.643139641	−3.918119	0.0001248879	0.002507111	0.7749892	C9orf24	−0.319611392	0.484509343	−3.317694	0.0010898895	0.01217438	−1.212985	DDAH1	0.445963096	4.870865439	2.908328	0.004071843	0.03152353	−2.400065
270	HTRA4	0.35829626	0.39643256	5.541339	1.004534e−07	9.873827e−06	7.467015	CRYM−AS1	0.10989384	0.233488356	3.916807	0.0001255179	0.002516959	0.7703337	FPR3	0.670072472	3.228307567	3.317598	0.0010902409	0.01217438	−1.213278	UBE2L3	0.185821932	5.082354422	2.907960	0.004076437	0.03154349	−2.401071
271	KIAA1467	0.53303601	0.95511709	5.538154	1.020355e−07	9.992324e−06	7.452197	CCDC65	0.11839718	0.371668733	3.916465	0.0001256820	0.002516959	0.7691242	NACA2	0.040326403	0.057764466	3.317551	0.0010904146	0.01217438	−1.213422	MUC12	−0.666076986	0.750529595	−2.907931	0.004076799	0.03154349	−2.401150
272	SLFN12L	0.50236084	0.76192976	5.531964	1.051806e−07	1.026245e−05	7.423411	SIPA1L2	−0.60107349	2.550160556	−3.916325	0.0001257498	0.002516959	0.7686258	NFRKB	0.249466124	2.943821017	3.317104	0.0010920636	0.01218300	−1.214795	VHDJH	0.801832917	1.281769636	2.907312	0.004084515	0.03158271	−2.402836
273	VRK2	0.45841144	3.07496457	5.530719	1.058242e−07	1.028743e−05	7.417627	C8orf80	0.43212332	0.651145543	3.916114	0.0001258512	0.002516959	0.7678799	ABP1	−1.015901172	4.085025542	−3.317093	0.0010921039	0.01218300	−1.214829	BC016751	−0.217547996	0.358327899	−2.907263	0.004085127	0.03158271	−2.402969
274	DD413682	0.06094772	0.05619889	5.528107	1.071876e−07	1.038194e−05	7.405489	QPRT	−1.21418158	2.803364793	−3.916041	0.0001258867	0.002516959	0.7676186	FAM132A	−0.640611207	1.396029840	−3.316368	0.0010947835	0.01220776	−1.217055	LOC441454	0.093775103	0.288568207	2.907238	0.004085438	0.03158271	−2.403037
275	RHOD	−1.29135009	4.15865078	−5.520961	1.110056e−07	1.071265e−05	7.372304	IL12RB2	0.32390841	0.526187202	3.915914	0.0001259476	0.002516959	0.7671715	LOC100129716	0.104275681	0.323872125	3.316166	0.0010955296	0.01221095	−1.217673	EVI2B	0.730725636	4.009338353	2.906408	0.004095824	0.03165378	−2.405301
276	TRIM24	0.78411005	3.67962187	5.514371	1.146437e−07	1.102366e−05	7.341730	BTBD1	−0.32350603	4.512349670	−3.914407	0.0001266767	0.002529625	0.7618307	POLK	−0.215902520	1.846000499	−3.315358	0.0010985236	0.01223918	−1.220152	PET112	0.213477634	1.606456106	2.906291	0.004097288	0.03165587	−2.405620
277	BC045731	0.34127036	0.88608631	5.510631	1.167599e−07	1.114834e−05	7.324390	CTDSP1	−0.26354445	5.634101405	−3.914131	0.0001268110	0.002530403	0.7608503	VMAC	−0.305304099	2.091480038	−3.314641	0.0011011902	0.01226374	−1.222355	NDOR1	0.271939637	3.313169882	2.905485	0.004107390	0.03172468	−2.407816
278	SMA	0.13176842	0.16183635	5.510595	1.167806e−07	1.114834e−05	7.324222	ADAMTS17	−0.34795739	0.611272370	−3.913336	0.0001271977	0.002536213	0.7580330	BC040935	0.197329856	0.506888321	3.313642	0.0011049078	0.01229749	−1.225416	GOLGA5	−0.183970545	4.204701936	−2.904844	0.004115444	0.03174858	−2.409562
279	FAM210B	−0.87538844	4.82313550	−5.509568	1.173684e−07	1.116430e−05	7.319462	CASP2	0.38606176	3.687728751	3.911403	0.0001281421	0.002553125	0.7511893	RFC5	0.370906819	3.140328309	3.313477	0.0011055269	0.01229749	−1.225924	AASDHPPT	0.237079063	3.554036099	2.904834	0.004115569	0.03174858	−2.409590
280	KBTBD10	−0.44929000	1.22835993	−5.507823	1.183734e−07	1.120358e−05	7.311379	KCTD11	−0.34933098	2.659045175	−3.911151	0.0001282661	0.002553679	0.7502943	SCARNA20	0.062063818	0.156667740	3.313454	0.0011056112	0.01229749	−1.225994	TMEM43	−0.315629258	4.344196054	−2.904776	0.004116294	0.03174858	−2.409747
281	RBMXL1	0.39878893	2.61056516	5.507388	1.186256e−07	1.120358e−05	7.309362	RAD9B	0.18778462	0.793158879	3.910143	0.0001287617	0.002559386	0.7467262	SLC9A3R2	−0.432850006	4.943626774	−3.312996	0.0011073224	0.01230942	−1.227398	LRRC61	0.392861517	3.376425556	2.904673	0.004117598	0.03174858	−2.410029
282	TCRA	0.41509397	0.58204633	5.505979	1.194451e−07	1.120681e−05	7.302835	SLC44A1	0.37733429	4.844940978	3.910052	0.0001288064	0.002559386	0.7464051	PBXIP1	−0.520325135	4.812968414	−3.312919	0.0011076114	0.01230942	−1.227635	LINC00307	0.007408718	0.001886717	2.904568	0.004118916	0.03174858	−2.410314
283	SUSD4	1.02637112	1.05027290	5.505878	1.195044e−07	1.120681e−05	7.302365	CCDC62	0.05482385	0.109876566	3.909980	0.0001288420	0.002559386	0.7461493	ZNF702P	−0.391537317	1.011594050	−3.312257	0.0011100882	0.01233178	−1.229664	ABLIM3	−0.527206906	2.134674218	−2.904528	0.004119412	0.03174858	−2.410422
284	FHDC1	0.66960583	1.88088879	5.500277	1.228195e−07	1.147713e−05	7.276426	ARSH	0.10196742	0.102542700	3.909524	0.0001290669	0.002561934	0.7445362	RPS11	0.317244569	10.535147351	3.312010	0.0011110142	0.01233691	−1.230421	FGD1	−0.348424687	1.858951502	−2.904482	0.004120001	0.03174858	−2.410549
285	APOL6	0.70606556	4.08764557	5.494948	1.260563e−07	1.173827e−05	7.251769	SHC1	−0.28738575	4.789956584	−3.907324	0.0001301576	0.002581653	0.7367515	ST13	0.243732413	5.379105822	3.311749	0.0011119938	0.01234262	−1.231222	SFRP4	−0.937834918	4.285661707	−2.904478	0.004120053	0.03174858	−2.410560
286	UBE2Z	0.43488478	5.23502357	5.487732	1.305722e−07	1.211628e−05	7.218406	IFT57	0.37706182	3.338443224	3.905405	0.0001311165	0.002598730	0.7299614	GNAL	−0.440476789	1.067307275	−3.309912	0.0011189084	0.01241009	−1.236851	GORASP1	−0.150452919	3.835829259	−2.903450	0.004133000	0.03183862	−2.413358
287	RAET1K	0.21726623	0.24409175	5.485788	1.318156e−07	1.218904e−05	7.209423	OR7E14P	0.71865538	2.769739945	3.904503	0.0001315692	0.002605757	0.7267730	AK097759	−0.103118718	0.252043163	−3.309885	0.0011190081	0.01241009	−1.236932	GIMAP6	0.479085930	2.598254742	2.903355	0.004134209	0.03183862	−2.413618
288	HYLS1	−0.54526301	1.53902615	−5.484148	1.328739e−07	1.224424e−05	7.201844	PIK3C2A	0.30955746	3.486678274	3.903532	0.0001320584	0.002613328	0.7233400	OPN3	−0.443708310	2.756598968	−3.309612	0.0011200388	0.01241634	−1.237768	BC041466	0.168443914	0.309015329	2.903265	0.004135337	0.03183862	−2.413862
289	FARP1	−0.78670199	3.70008067	−5.480023	1.355715e−07	1.244959e−05	7.182795	RCC2	0.40181778	5.586590465	3.903354	0.0001321484	0.002613328	0.7227096	CYTH4	0.453247147	1.807130874	3.308927	0.0011226319	0.01243989	−1.239868	GPR18	0.347960578	0.755981506	2.903118	0.004137204	0.03184154	−2.414264
290	C2orf70	−1.08502725	1.74022559	−5.477968	1.369351e−07	1.248137e−05	7.173310	LOC100129148	0.23208409	3.784542067	3.903029	0.0001323125	0.002614625	0.7215618	C12orf61	0.126203424	0.419815041	3.308416	0.0011245695	0.01245112	−1.241434	C20orf94	0.274128491	1.477145810	2.903045	0.004138116	0.03184154	−2.414461
291	ANXA5	−0.69456492	7.35199053	−5.477766	1.370702e−07	1.248137e−05	7.172376	ZNF616	0.31275272	1.619446073	3.901781	0.0001329450	0.002622769	0.7171507	DCHS1	−0.491985094	2.567464175	−3.308325	0.0011249132	0.01245112	−1.241712	SATB2	−0.398468907	1.198633249	−2.902360	0.004146790	0.03189903	−2.416327
292	COL22A1	0.92850027	0.77323010	5.477379	1.373285e−07	1.248137e−05	7.170592	SPIN3	−0.42224713	1.080181570	−3.901560	0.0001330571	0.002622769	0.7163710	C14orf23	0.010041018	0.005534064	3.308288	0.0011250533	0.01245112	−1.241825	MIR31	0.063962746	0.028703627	2.901973	0.004151684	0.03192360	−2.417379
293	LOC148189	−0.61043710	1.30479041	−5.473711	1.398034e−07	1.266294e−05	7.153664	PTAFR	0.63157188	2.834217249	3.901462	0.0001331072	0.002622769	0.7160232	RFX3	0.285042816	0.873968002	3.307903	0.0011265143	0.01246059	−1.243003	LOC100505933	0.271748267	1.396722468	2.901918	0.004152390	0.03192360	−2.417530
294	BC024732	−0.73969384	1.72208587	−5.471962	1.409984e−07	1.267938e−05	7.145597	BC063132	−0.62119844	1.748912400	−3.901436	0.0001331200	0.002622769	0.7159342	FLT3	0.157482054	0.291885987	3.307816	0.0011268475	0.01246059	−1.243272	AK310665	0.130780417	0.222898504	2.900886	0.004165493	0.03201506	−2.420339
295	GOLGA2P5	−0.34470929	0.70418907	−5.471762	1.411357e−07	1.267938e−05	7.144675	LAX1	0.61976931	1.279565576	3.900989	0.0001333474	0.002625302	0.7143550	LPXN	0.545050315	2.621677101	3.304781	0.0011384318	0.01258344	−1.252559	PLA2G15	0.238443383	2.961680247	2.900654	0.004168433	0.03202839	−2.420968
296	PSPC1	0.44189599	3.90384849	5.471351	1.414181e−07	1.267938e−05	7.142781	GFPT1	−0.51976581	4.467128199	−3.899930	0.0001338882	0.002629173	0.7106118	SATB1	−0.541090899	2.407008934	−3.304583	0.0011391924	0.01258661	−1.253165	DQ583960	0.291817870	1.623415110	2.900484	0.004170603	0.03203579	−2.421432
297	CD200R1	0.42236731	0.66573802	5.469042	1.430160e−07	1.277947e−05	7.132133	AX748308	0.10040991	0.183725731	3.899888	0.0001339096	0.002629173	0.7104638	ENO1−AS1	0.229661511	0.645003609	3.303671	0.0011426992	0.01262010	−1.255956	EIF4EBP3	−0.353811594	3.989433931	−2.900342	0.004172413	0.03204041	−2.421819
298	KIAA1653	0.15723682	0.09855246	5.467649	1.439885e−07	1.282319e−05	7.125711	ADRA2C	−0.89965147	1.363372053	−3.899854	0.0001339268	0.002629173	0.7103448	SEMA4D	0.320926679	2.380823883	3.302656	0.0011466113	0.01265804	−1.259059	SNORD54	0.347920162	1.327836874	2.898584	0.004194850	0.03219892	−2.426599
299	SFMBT2	0.49780313	1.06491399	5.460823	1.488472e−07	1.321156e−05	7.094262	AF075112	0.10811450	0.088490155	3.899690	0.0001340109	0.002629173	0.7097646	PARM1	−0.689397453	2.866134756	−3.302394	0.0011476248	0.01266396	−1.259861	CASKIN2	−0.248039115	3.387795102	−2.898535	0.004195480	0.03219892	−2.426733
300	PAPD4	0.40891895	3.60521627	5.456136	1.522759e−07	1.347044e−05	7.072682	ZNF655	0.60576928	2.432498887	3.899634	0.0001340394	0.002629173	0.7095680	TRDMT1	0.182883350	0.778751350	3.301576	0.0011507906	0.01269361	−1.262362	PTPRN2	−0.639633541	2.187727757	−2.898143	0.004200500	0.03222812	−2.427799

	Gene	logFC	AveExpr	t	P.Value	adj.P.Val	B	Gene	logFC	AveExpr	t	P.Value	adj.P.Val	B	Gene	logFC	AveExpr	t	P.Value	adj.P.Val	B	Gene	logFC	AveExpr	t	P.Value	adj.P.Val	B
301	MFSD6L	−0.80577183	0.99641495	−5.455457	1.527790e−07	1.347044e−05	7.069557	B3GNT8	−0.82938996	2.446002849	−3.899120	0.0001343030	0.002632399	0.7077504	ABCA12	0.434514121	0.502418256	3.300627	0.001154475	0.01272891	−1.265263	TRIM47	−0.382460597	4.586294547	−2.897675	0.004206494	0.03226478	−2.429069
302	FAH	−0.56077610	1.66069999	−5.452330	1.551162e−07	1.363122e−05	7.055171	NEK4	0.32052961	2.999859880	3.898775	0.0001344800	0.002633922	0.7065326	MPZL2	0.532246274	3.871364433	3.300504	0.001154950	0.01272891	−1.265637	ZNF184	0.212308246	1.828716617	2.896775	0.004218053	0.03234410	−2.431515
303	V_alpha_immunoglobulin	0.21903409	0.17842095	5.451350	1.558562e−07	1.365105e−05	7.050662	AV4S1	0.30902832	0.425748936	3.898481	0.0001346311	0.002634938	0.7054938	RIF1	0.265921617	2.617913386	3.298992	0.001160842	0.01278854	−1.270256	C20orf118	0.385994811	3.095406047	2.896638	0.004219814	0.03234825	−2.431887
304	AK130294	−0.76530135	2.69611262	−5.449293	1.574200e−07	1.371732e−05	7.041203	ORC2	0.29667541	3.144979634	3.896936	0.0001354272	0.002648566	0.7000409	RCC1	0.452966240	4.124928675	3.298529	0.001162655	0.01280320	−1.271673	GPR39	0.431508925	2.550038525	2.896434	0.004222449	0.03235910	−2.432443
305	LEPROT	−0.48303501	4.19343339	−5.448997	1.576466e−07	1.371732e−05	7.039840	LRIG3	0.55123419	3.464558398	3.896654	0.0001355732	0.002649468	0.6990446	TFDP1	0.397211882	5.068307563	3.297691	0.001165938	0.01283274	−1.274233	LOC375295	−0.369256655	1.005133314	−2.896243	0.004224907	0.03236860	−2.432962
306	POLQ	0.60082350	1.19355914	5.448305	1.581765e−07	1.371845e−05	7.036661	DKFZP564C196	0.16490039	0.378594958	3.895574	0.0001361332	0.002657499	0.6952326	DHX58	0.397420179	2.515823566	3.297597	0.001166305	0.01283274	−1.274518	SLC30A10	−0.155798712	0.181073372	−2.895977	0.004228333	0.03238549	−2.433685
307	DUT	0.57823571	4.27231397	5.438187	1.661315e−07	1.436145e−05	6.990171	BC048141	0.06572952	0.053611798	3.895475	0.0001361844	0.002657499	0.6948844	KLRB1	0.677810047	2.423802329	3.297361	0.001167232	0.01283761	−1.275239	LAMB2	−0.484260490	5.107354676	−2.894925	0.004241905	0.03248007	−2.436540
308	AB231723	0.40997759	0.21847163	5.436549	1.674552e−07	1.442887e−05	6.982653	EOMES	0.56872854	0.993587740	3.894907	0.0001364802	0.002661314	0.6928781	CDC14B	0.289301484	2.380496237	3.296605	0.001170203	0.01286094	−1.277547	AK123440	0.014637955	0.011811951	2.893664	0.004258234	0.03259569	−2.439964
309	ANKRD24	0.26999209	0.41397215	5.432246	1.709822e−07	1.468510e−05	6.962906	SLC7A4	−0.71755914	0.954308292	−3.894576	0.0001366524	0.002662715	0.6917122	N4BP2	0.283492138	1.374366707	3.296575	0.001170322	0.01286094	−1.277639	LHX2	0.148779595	0.214691349	2.893034	0.004266421	0.03264895	−2.441676
310	PKN3	−0.70821402	2.78661857	−5.427343	1.750902e−07	1.498941e−05	6.940415	PALB2	0.29722200	2.354336197	3.892840	0.0001375606	0.002678445	0.6855865	BC034630	0.025979366	0.010145965	3.296301	0.001171402	0.01286749	−1.278476	LTA	0.353655702	0.833139079	2.892616	0.004271855	0.03268111	−2.442810
311	B3GALNT1	−0.71989911	1.80862770	−5.426027	1.762085e−07	1.503664e−05	6.934384	DL476313	0.74676140	4.815715438	3.892520	0.0001377284	0.002679747	0.6844588	CHST5	−0.987117205	1.871685332	−3.295542	0.001174396	0.01289406	−1.280793	SSX2IP	0.329708322	2.319559262	2.892419	0.004274413	0.03268424	−2.443343
312	ZNF793	0.54336397	0.57520182	5.423960	1.779793e−07	1.513908e−05	6.924912	LIPA	0.57965738	5.076466115	3.892122	0.0001379377	0.002681852	0.6830551	CAPZA3	0.124319078	0.101194234	3.295441	0.001174793	0.01289406	−1.281100	CALM3	0.277485552	7.394591023	2.892395	0.004274728	0.03268424	−2.443409
313	LOC440335	−1.38281540	4.77380579	−5.415828	1.851176e−07	1.569596e−05	6.887663	ALPK1	0.32228500	1.561127165	3.891925	0.0001380412	0.002681900	0.6823614	SERP2	−0.467409408	1.148247312	−3.295090	0.001176180	0.01290101	−1.282171	RET	−0.487828705	0.681850688	−2.892278	0.004276252	0.03268648	−2.443726
314	ETV7	1.04403050	2.97145425	5.414027	1.867356e−07	1.578272e−05	6.879420	MTHFD1L	0.42926461	2.527997544	3.891376	0.0001383303	0.002683205	0.6804263	TNFRSF17	0.590543017	1.276317149	3.295035	0.001176399	0.01290101	−1.282340	LMBR1L	0.210415199	2.450321852	2.891956	0.004280452	0.03270916	−2.444601
315	HV303528	1.01783397	2.84925357	5.413032	1.876356e−07	1.580845e−05	6.874866	KRT76	0.02634310	0.007008906	3.891296	0.0001383726	0.002683205	0.6801437	PLXNB3	−0.614480613	1.126331154	−3.293139	0.001183919	0.01297812	−1.288123	TNFSF10	0.470551536	4.598964812	2.891385	0.004287895	0.03275660	−2.446149
316	C19orf57	0.68386869	1.04406421	5.408185	1.920803e−07	1.613171e−05	6.852691	JAG2	−0.58224733	2.726126257	−3.891222	0.0001384117	0.002683205	0.6798828	WNK4	−0.614371694	1.234104979	−3.292035	0.001188318	0.01302096	−1.291488	TNNC2	−0.604514645	1.093182671	−2.891012	0.004292772	0.03278443	−2.447162
317	CCRL1	0.96282521	1.58344757	5.405071	1.949897e−07	1.632439e−05	6.838453	CCL4L2	0.12716280	0.101209913	3.890125	0.0001389920	0.002692237	0.6760137	CEP85	0.374469712	2.172628984	3.290452	0.001194655	0.01308500	−1.296315	PLCZ1	0.081429197	0.072678413	2.890416	0.004300561	0.03283446	−2.448777
318	IRS2	−1.27886346	3.66732350	−5.397888	2.018655e−07	1.684688e−05	6.805633	AX229788	0.14825892	0.091860314	3.889958	0.0001390805	0.002692237	0.6754254	FLT3LG	0.433525495	2.834281016	3.289396	0.001198900	0.01312607	−1.299534	P2RX3	0.269617370	0.378144588	2.890223	0.004303086	0.03284429	−2.449300
319	ATF5	0.63935723	4.27132369	5.395819	2.038892e−07	1.694269e−05	6.796186	KIR2DL1	0.06546546	0.068198663	3.888035	0.0001401034	0.002710061	0.6686497	SLC24A1	0.204049572	1.495510849	3.288878	0.001200986	0.01314182	−1.301111	ZNF641	−0.183230463	2.219101946	−2.889265	0.004315661	0.03293080	−2.451899
320	FN3K	−0.83252304	3.30562631	−5.394767	2.049263e−07	1.694269e−05	6.791381	CCDC24	−0.53385759	2.637907599	−3.886547	0.0001409000	0.002723485	0.6634074	SPON1	−0.998658477	3.624634558	−3.288768	0.001201428	0.01314182	−1.301445	WDR91	0.188519214	1.489164551	2.888886	0.004320638	0.03295931	−2.452926
321	CCDC148	0.25926472	0.28470465	5.394764	2.049288e−07	1.694269e−05	6.791369	YBEY	0.46507119	2.218810889	3.886303	0.0001410306	0.002724025	0.6625507	AGPAT5	0.429236376	3.016615190	3.288670	0.001201824	0.01314182	−1.301744	SH3GLB1	−0.202986449	4.108145817	−2.888630	0.004324007	0.03297553	−2.453621
322	C7orf29	0.61226582	2.02304822	5.394020	2.056656e−07	1.695080e−05	6.787971	ZNF202	0.26391484	1.819694927	3.886019	0.0001411832	0.002724991	0.6615505	ZMYND15	0.434654187	1.735556094	3.288301	0.001203315	0.01315271	−1.302869	RAB35	−0.178995275	4.376737765	−2.888341	0.004327798	0.03299495	−2.454401
323	FAM174A	−0.61469596	2.91572678	−5.390001	2.096870e−07	1.713254e−05	6.769633	APCDD1	−1.14833993	3.292951038	−3.885700	0.0001413552	0.002726327	0.6604249	SACS	−0.368291952	1.253986631	−3.287857	0.001205110	0.01316691	−1.304222	C17orf58	0.283343944	2.780854677	2.887951	0.004332943	0.03302156	−2.455460
324	TRIM26	0.36309705	4.21804207	5.389930	2.097588e−07	1.713254e−05	6.769309	EAF2	0.44025745	1.694184682	3.885153	0.0001416498	0.002730024	0.6585004	GNPDA1	0.328922899	3.942743680	3.287594	0.001206176	0.01316707	−1.305024	FRMD8P1	0.028962759	0.068786820	2.887888	0.004333776	0.03302156	−2.455631
325	PRKCDBP	−1.01086485	5.14482851	−5.389882	2.098072e−07	1.713254e−05	6.769090	EPHX1	−0.58772445	5.292167006	−3.884539	0.0001419817	0.002734435	0.6563366	MT1H	−1.120450222	2.300480004	−3.287577	0.001206244	0.01316707	−1.305075	RPP30	0.193954976	2.052401052	2.887650	0.004336905	0.03303591	−2.456274
326	HNRPLL	0.28066604	3.61173977	5.387151	2.125857e−07	1.730617e−05	6.756632	ZNF649	−0.51294717	1.509962612	−3.884107	0.0001422150	0.002736942	0.6548185	DIAPH1	0.347559293	5.004461955	3.287486	0.001206613	0.01316707	−1.305353	HIST2H2AC	0.360561471	1.631816028	2.887411	0.004340062	0.03304371	−2.456922
327	NAIP	0.11408884	0.16876781	5.383664	2.161851e−07	1.754537e−05	6.740733	HOXA13	−1.08854941	2.004232981	−3.883290	0.0001426585	0.002741762	0.6519399	POLR2E	0.224429286	5.269674083	3.286923	0.001208892	0.01318651	−1.307065	OR5J2	0.010929265	0.003747157	2.887324	0.004341209	0.03304371	−2.457157
328	AK8	0.49121449	0.51388691	5.381401	2.185523e−07	1.768341e−05	6.730421	CENPP	0.25087955	1.636224845	3.883265	0.0001426720	0.002741762	0.6518519	AGPAT1	−0.299703077	4.802811282	−3.285994	0.001212668	0.01322227	−1.309894	SNRNP25	0.324117847	3.918700213	2.887290	0.004341664	0.03304371	−2.457251
329	GOLPH3	0.38215521	3.32782575	5.380513	2.194881e−07	1.770515e−05	6.726375	LOC100131691	0.27859300	1.191661908	3.882860	0.0001428920	0.002744003	0.6504275	DNAJC7	0.236765222	4.852510018	3.285730	0.001213743	0.01322855	−1.310698	MME	−0.743949111	1.546887930	−2.887154	0.004343460	0.03304790	−2.457619
330	ALDH16A1	0.60218217	3.94638416	5.378064	2.220898e−07	1.784880e−05	6.715218	PPM1D	0.25473188	2.252983328	3.882043	0.0001433370	0.002750558	0.6475528	RPS7	0.334865373	5.545096457	3.284710	0.001217904	0.01326173	−1.313803	SDK2	−0.336223308	0.571963457	−2.886935	0.004346354	0.03306044	−2.458212
331	GRAP2	0.62267446	1.16729889	5.377107	2.231140e−07	1.784880e−05	6.710861	CTSF	−0.87577512	3.355698858	−3.881315	0.0001437346	0.002756194	0.6449923	PCDH20	−0.369723359	0.484215115	−3.284622	0.001218263	0.01326173	−1.314070	LOC285141	0.422339900	0.813094039	2.886798	0.004348166	0.03306475	−2.458584
332	LINS	0.40466439	1.97694247	5.376705	2.235465e−07	1.784880e−05	6.709028	RBM14	0.18772450	3.735532008	3.880796	0.0001440188	0.002759649	0.6431663	PPP2R3A	−0.445752655	1.425368702	−3.284616	0.001218287	0.01326173	−1.314088	MYO18A	−0.283251334	4.078717712	−2.886161	0.004356589	0.03311931	−2.460307
333	GZMA	1.26290165	2.93878605	5.376321	2.239590e−07	1.784880e−05	6.707282	TRAV38−2DV8	0.41716050	0.723480048	3.880570	0.0001441425	0.002760027	0.6423726	AMPD2	−0.250575290	3.559753523	−3.284431	0.001219042	0.01326452	−1.314650	CENPB	−0.343522776	5.662827647	−2.885100	0.004370665	0.03321680	−2.463180
334	TFF3	−2.65279105	5.18368835	−5.374297	2.261495e−07	1.796940e−05	6.698066	HSD11B1L	−0.43336685	1.358096163	−3.879998	0.0001444565	0.002764045	0.6403608	PDE12	0.303823997	3.441067615	3.283894	0.001221238	0.01328297	−1.316283	HOXB8	−0.683149801	1.239748765	−2.884898	0.004373355	0.03322773	−2.463728
335	CD8B	0.92797136	1.33127383	5.372438	2.281801e−07	1.807663e−05	6.689603	CXorf61	−1.27768354	1.695771800	−3.877072	0.0001460731	0.002792881	0.6300732	MND1	0.481301097	2.033298286	3.283596	0.001222459	0.01328543	−1.317190	SLC16A8	0.180113314	0.445470513	2.884361	0.004380499	0.03327248	−2.465182
336	RPUSD4	0.42262254	3.09689635	5.362772	2.390261e−07	1.878344e−05	6.645637	JA429722	0.14491936	0.298493547	3.876891	0.0001461740	0.002792881	0.6294347	BCORL1	−0.339916196	2.219291754	−3.283595	0.001222466	0.01328543	−1.317195	POT1	0.250025507	2.338866782	2.883951	0.004385958	0.03330442	−2.466291
337	LOC100506585	0.33034791	0.61127570	5.362497	2.393413e−07	1.878344e−05	6.644390	TBC1D10C	0.82238068	2.177173403	3.875384	0.0001470135	0.002806900	0.6241414	TRAF3IP2	0.339906676	3.716881992	3.283221	0.001223999	0.01329665	−1.318332	EHD2	−0.620659566	5.291903507	−2.883751	0.004388624	0.03331513	−2.466833
338	STX11	0.52405356	1.25647480	5.362484	2.393572e−07	1.878344e−05	6.644327	TSPY26P	−0.45621516	1.106617035	−3.875164	0.0001471366	0.002807231	0.6233676	SIT1	0.679196499	1.885877978	3.282817	0.001225658	0.01330923	−1.319561	FAM46C	0.555893975	2.608533259	2.882784	0.004401538	0.03340361	−2.469449
339	GPR31	0.12703441	0.11015927	5.361983	2.399331e−07	1.878344e−05	6.642052	GSDMC	0.38341159	0.336845228	3.874860	0.0001473069	0.002807966	0.6222986	MIR25	0.323267540	0.961383431	3.282451	0.001227161	0.01332009	−1.320672	CDSN	−0.412704435	0.907250878	−2.882650	0.004403327	0.03340763	−2.469811
340	TCRGV	0.45005414	0.59942601	5.361180	2.408592e−07	1.880047e−05	6.638405	CCR6	0.37851075	1.788514907	3.874717	0.0001473867	0.002807966	0.6217975	LILRB2	0.491989699	1.902521131	3.280744	0.001234205	0.01339107	−1.325864	BC045656	0.098119713	0.436101849	2.882421	0.004406386	0.03342128	−2.470430
341	STAT1	0.95343236	5.92204548	5.358663	2.437867e−07	1.897318e−05	6.626968	TUBB6	−0.60707091	4.394282639	−3.874364	0.0001475846	0.002809718	0.6205577	QTRTD1	0.243539117	2.161813361	3.280484	0.001235282	0.01339728	−1.326656	BX248273	−0.205151544	0.204638941	−2.882300	0.004408016	0.03342409	−2.470759
342	GZMM	0.85685264	1.29339422	5.355245	2.478180e−07	1.923053e−05	6.611442	BCORP1	0.05755986	0.057761560	3.873158	0.0001482624	0.002820599	0.6163223	KIAA0664L3	0.263469635	1.614943734	3.279466	0.001239502	0.01343756	−1.329749	CCT5	0.338442137	5.739788439	2.881812	0.004414553	0.03346410	−2.472079
343	TLR8	0.64739177	0.87703986	5.351429	2.523938e−07	1.952851e−05	6.594122	SIGLEC7	0.47293842	1.441377854	3.872492	0.0001486381	0.002825720	0.6139833	IAH1	−0.278016870	4.167237087	−3.278518	0.001243441	0.01347476	−1.332627	BZW2	0.345740818	4.746320221	2.881044	0.004424853	0.03353260	−2.474154
344	ZNF434	0.28136195	2.18324629	5.344764	2.605866e−07	2.010380e−05	6.563884	PURA	−0.30746792	3.241933446	−3.872248	0.0001487758	0.002826314	0.6131271	AK125500	0.187542221	1.515004370	3.278212	0.001244719	0.01348310	−1.333558	ERAP1	0.351458824	3.754854646	2.880327	0.004434505	0.03358969	−2.476094
345	SYNE2	0.68720123	2.98383107	5.343844	2.617371e−07	2.013403e−05	6.559714	BC045767	0.04781085	0.022520354	3.869397	0.0001503956	0.002854022	0.6031191	ETNK2	−0.559375154	1.357073668	−3.277389	0.001248154	0.01351409	−1.336058	DCTD	0.194152565	4.866942582	2.880296	0.004434918	0.03358969	−2.476177
346	MAPK14	−0.38623175	3.26297966	−5.342480	2.634521e−07	2.020739e−05	6.553532	TRBV19	0.65958574	1.248256882	3.869303	0.0001504494	0.002854022	0.6027886	ANO7	−0.476472958	0.961000149	−3.277283	0.001248598	0.01351409	−1.336381	ZFP112	0.188996157	0.798167346	2.879755	0.004442212	0.03363251	−2.477640
347	SLC22A3	−0.98429759	1.45331737	−5.338709	2.682527e−07	2.051631e−05	6.536440	FIGNL1	0.44920291	2.304414955	3.868868	0.0001506979	0.002856694	0.6012633	IGF2	−1.362162329	3.869203819	−3.277083	0.001249434	0.01351763	−1.336988	AGFG1	0.214959460	3.571750905	2.879688	0.004443105	0.03363251	−2.477819
348	PAK2	−0.39663516	4.53581435	−5.336699	2.708448e−07	2.065503e−05	6.527338	BST2	0.95588866	6.659060569	3.868506	0.0001509051	0.002858153	0.5999935	RABL2A	0.181926789	1.015869695	3.274862	0.001258756	0.01361293	−1.343730	FLJ31485	−0.233345234	0.409377282	−2.878297	0.004461901	0.03376190	−2.481578
349	TMEM86A	0.52936852	1.68406365	5.335690	2.721566e−07	2.069560e−05	6.522765	TRAV39	0.22384959	0.338455842	3.868348	0.0001509956	0.002858153	0.5994395	ARHGEF4	−0.494725365	0.949096622	−3.273151	0.001265984	0.01368552	−1.348922	BC031238	0.006780606	0.002753160	2.878091	0.004464689	0.03376190	−2.482134
350	SMTNL1	0.28884413	0.33816073	5.333988	2.743814e−07	2.080516e−05	6.515059	TDRD5	−0.48343558	0.627070310	−3.868170	0.0001510980	0.002858153	0.5988131	HOXC8	−0.441519930	0.832908154	−3.272844	0.001267285	0.01369401	−1.349853	MXRA8	−0.601236638	5.062867336	−2.878088	0.004464732	0.03376190	−2.482143
351	PRF1	0.97569545	2.18816029	5.330385	2.791498e−07	2.110552e−05	6.498753	LOC341056	0.09460018	0.184575064	3.867724	0.0001513537	0.002859086	0.5972504	SNORD36C	0.379276434	1.572029048	3.271761	0.001271882	0.01373808	−1.353137	BRSK2	−0.402343582	0.671322758	−2.878047	0.004465288	0.03376190	−2.482253
352	MT1G	−2.07604175	5.08213902	−5.329799	2.799330e−07	2.110552e−05	6.496101	GYS1	0.30982639	4.410712938	3.867708	0.0001513628	0.002859086	0.5971948	HOMER2	−0.864281301	1.656275646	−3.271427	0.001273303	0.01374147	−1.354149	PTRF	−0.662442302	5.661475023	−2.877715	0.004469786	0.03378629	−2.483150
353	C1QTNF1	−1.14637500	3.59585835	−5.325185	2.861764e−07	2.151512e−05	6.475226	SLC35B1	0.36724315	4.561462242	3.866866	0.0001518474	0.002866201	0.5942406	MMADHC	0.207559313	5.605621610	3.271396	0.001273436	0.01374147	−1.354244	CCDC136	−0.436094707	0.861896947	−2.877340	0.004474875	0.03381512	−2.484163
354	LHFPL1	0.06980400	0.05004230	5.323366	2.886739e−07	2.164157e−05	6.467002	POLE	0.43059741	2.867667298	3.865956	0.0001523726	0.002874069	0.5910500	CLDN20	0.090808732	0.264453724	3.271225	0.001274165	0.01374147	−1.354763	FAM49B	0.325694539	4.746279253	2.876964	0.004479982	0.03384338	−2.485178
355	HOXB7	−1.21581569	3.57550864	−5.322215	2.902652e−07	2.169957e−05	6.461800	LOC442497	−0.34588444	0.346732314	−3.865007	0.0001529222	0.002882388	0.5877224	CCDC66	0.201420643	1.493565475	3.271201	0.001274267	0.01374147	−1.354835	A4GNT	0.797057706	1.192372113	2.876877	0.004481165	0.03384338	−2.485413
356	KRT7	−2.58688319	4.71385038	−5.316837	2.978153e−07	2.220146e−05	6.437500	TRNA_Sup	0.12784330	0.264387641	3.862894	0.0001541529	0.002903523	0.5803156	MILR1	0.362123136	1.342538864	3.270974	0.001275235	0.01374633	−1.355524	CCIN	−0.067590308	0.145697359	−2.876653	0.004484217	0.03385340	−2.486020
357	CD38	1.20887605	2.14448138	5.315418	2.998395e−07	2.228975e−05	6.431090	GLOD5	−0.34934489	0.675463366	−3.862500	0.0001543832	0.002905336	0.5789360	HIATL1	0.247566720	4.478707197	3.270151	0.001278747	0.01377494	−1.358018	NUP62CL	−0.456400096	1.274248595	−2.876592	0.004485043	0.03385340	−2.486183
358	AMIGO1	−0.62924546	1.59890187	−5.313153	3.030984e−07	2.246907e−05	6.420861	ZP3	−0.58860110	1.359239713	−3.862355	0.0001544681	0.002905336	0.5784279	RILPL1	−0.318851425	2.172362318	−3.270109	0.001278927	0.01377494	−1.358146	HIP1R	0.436981984	3.734757316	2.875716	0.004496968	0.03393376	−2.488547
359	SCRN1	−1.10586456	3.52411131	−5.300208	3.223914e−07	2.383272e−05	6.362472	LINC00336	−0.10830949	0.115595483	−3.861924	0.0001547208	0.002906120	0.5769173	C11orf45	0.234826136	0.598428756	3.269649	0.001280896	0.01379055	−1.359541	GGNBP1	0.045170089	0.076781381	2.874539	0.004513055	0.03403426	−2.491726
360	PLOD2	−0.74935743	3.25883669	−5.298324	3.252978e−07	2.398077e−05	6.353981	BC014138	−0.37041951	3.884629011	−3.861910	0.0001547288	0.002906120	0.5768699	FBLN1	−0.942580673	3.988550944	−3.269292	0.001282423	0.01379763	−1.360620	METTL4	−0.212920509	2.013271136	−2.874510	0.004513440	0.03403426	−2.491802
361	CD96	0.86917370	1.61527920	5.296981	3.273849e−07	2.406777e−05	6.347930	IL26	0.17796603	0.221592227	3.860908	0.0001553175	0.002915114	0.5733609	SF3B5	0.263430864	6.453053847	3.269252	0.001282594	0.01379763	−1.360741	H19	−1.221531741	5.153270664	−2.874395	0.004515019	0.03403426	−2.492113
362	CD300LF	0.69495139	1.36576128	5.293039	3.335849e−07	2.443662e−05	6.330181	TBK1	0.29330522	3.446658733	3.858853	0.0001565320	0.002935775	0.5661639	PRPF31	0.262145436	3.949238695	3.268181	0.001287195	0.01384152	−1.363988	FJX1	−0.347607564	1.845863975	−2.874366	0.004515416	0.03403426	−2.492192
363	C12orf59	0.24100811	0.36622167	5.292625	3.342437e−07	2.443662e−05	6.328314	SETD7	−0.51319812	3.607017389	−3.858672	0.0001566395	0.002935775	0.5655294	DGCR8	0.183999375	2.982059354	3.267556	0.001289886	0.01386484	−1.365882	PPP1R3G	−0.299534437	0.758527709	−2.874253	0.004516966	0.03403628	−2.492497
364	MATN2	−1.28008835	2.99913372	−5.287423	3.426167e−07	2.491818e−05	6.304907	CUTA	−0.38530025	6.102460197	−3.857841	0.0001571332	0.002942949	0.5626221	LRRC46	0.166224428	0.486642441	3.267271	0.001291114	0.01387104	−1.366744	RNASEH2A	0.403130993	4.324717345	2.873654	0.004525169	0.03408010	−2.494112
365	ABCD2	0.26161033	0.32424036	5.287367	3.427083e−07	2.491818e−05	6.304655	RNF166	0.36994784	2.855089671	3.857507	0.0001573323	0.002944599	0.5614523	NRSN2	−0.607389489	3.415418698	−3.267174	0.001291530	0.01387104	−1.367036	HNRNPK	0.162852077	6.698425274	2.873641	0.004525350	0.03408010	−2.494148
366	ADORA3	0.63819744	1.18944074	5.277964	3.583703e−07	2.598576e−05	6.262382	C1orf233	−0.70781531	2.743794909	−3.856957	0.0001576602	0.002945042	0.5595285	RAD54L	0.452043313	2.090583029	3.267030	0.001292151	0.01387104	−1.367472	LOC728752	0.201500857	0.850881472	2.873207	0.004531298	0.03411521	−2.495317
367	LDLRAD3	−0.78864645	2.15661493	−5.276862	3.602502e−07	2.605090e−05	6.257433	IL6R	0.54293303	2.106593630	3.856918	0.0001576836	0.002945042	0.5593913	TNFRSF4	0.442922243	1.977667553	3.266937	0.001292554	0.01387104	−1.367755	GGPS1	−0.232344101	3.561463414	−2.872806	0.004536820	0.03414115	−2.496401
368	EI24	0.39061799	5.86721170	5.267826	3.760377e−07	2.711865e−05	6.216864	HIST1H2AH	0.31358527	0.553573653	3.856735	0.0001577928	0.002945042	0.5587517	SLC16A6	0.285516772	0.682079851	3.266491	0.001294482	0.01388525	−1.369106	SLC26A11	−0.300374132	2.156425291	−2.872723	0.004537951	0.03414115	−2.496623
369	ARL13B	−0.32924765	1.97776806	−5.260547	3.892411e−07	2.795473e−05	6.184226	X04930	0.69810248	1.389150941	3.856723	0.0001577998	0.002945042	0.5587108	AK097794	0.232352481	1.069240778	3.266389	0.001294925	0.01388525	−1.369416	DNAAF1	0.087987759	0.188136549	2.872676	0.004538603	0.03414115	−2.496751
370	CDKN1C	−1.18223443	3.33985621	−5.260278	3.897378e−07	2.795473e−05	6.183020	MPND	0.34842524	3.430422147	3.856152	0.0001581415	0.002949344	0.5567128	IL17RD	−0.430246813	0.996850002	−3.266005	0.001296586	0.01388896	−1.370578	LOC730091	−0.239388784	0.524156482	−2.872268	0.004544218	0.03416663	−2.497851
371	DQ580250	−0.61445201	2.24532502	−5.259535	3.911119e−07	2.797768e−05	6.179691	HEATR3	0.29410652	2.632923083	3.855554	0.0001585003	0.002951414	0.5546190	NAA16	0.288374460	2.249919493	3.265998	0.001296616	0.01388896	−1.370599	TMEM132E	−0.132819275	0.277717376	−2.872243	0.004544564	0.03416663	−2.497919
372	AK092619	0.09208032	0.05291600	5.258955	3.921890e−07	2.797931e−05	6.177090	SLC38A1	−0.68450548	4.004726226	−3.855414	0.0001585840	0.002951414	0.5541311	ZNF596	0.189149133	0.715954624	3.265944	0.001296850	0.01388896	−1.370763	DQ600552	−0.134852210	0.145426856	−2.872054	0.004547167	0.03417651	−2.498429
373	ZFYVE28	0.42056788	1.40180255	5.258239	3.935206e−07	2.799905e−05	6.173884	TPK1	0.46787561	1.643011614	3.855411	0.0001585861	0.002951414	0.5541188	KIF12	−0.728124546	2.703592464	−3.265825	0.001297364	0.01388896	−1.371122	GSG2	0.399153818	1.858296461	2.871769	0.004551083	0.03419405	−2.499195
374	TSNAX	−0.36789892	3.90342332	−5.256808	3.961977e−07	2.811414e−05	6.167473	MIR4511	0.08099978	0.053717886	3.854556	0.0001591001	0.002958905	0.5511294	BTF3L4	0.225971059	3.081545026	3.265327	0.001299523	0.01390120	−1.372630	MCF2L	−0.430063612	2.550526821	−2.871697	0.004552077	0.03419405	−2.499389
375	EMR4P	0.17415858	0.19717560	5.248287	4.125063e−07	2.919334e−05	6.129326	TRBV7−3	0.48650627	1.019489501	3.854105	0.0001593720	0.002961887	0.5495515	BC037494	0.058741527	0.051019909	3.265320	0.001299555	0.01390120	−1.372652	CNTD1	0.217059125	3.011998950	2.871538	0.004554272	0.03420086	−2.499818
376	TMEM65	−0.61430106	2.39395388	−5.245564	4.178540e−07	2.949316e−05	6.117146	PGLYRP2	0.07637745	0.099809303	3.853780	0.0001595680	0.002963454	0.5484163	KRT25	0.028304436	0.012357447	3.264749	0.001302031	0.01392208	−1.374378	IER3	−0.627004963	6.395525015	−2.871204	0.004558879	0.03422576	−2.500718
377	LTBP3	−0.91454321	4.33815663	−5.242062	4.248318e−07	2.990613e−05	6.101486	MFAP2	−0.84694741	3.678286520	−3.853232	0.0001598992	0.002967528	0.5465007	UBN1	0.215197644	3.444682704	3.264471	0.001303243	0.01392943	−1.375221	MIS18BP1	0.249643885	2.630831286	2.870788	0.004564633	0.03425187	−2.501841
378	CHRM3	−0.98313769	1.30375283	−5.238245	4.325652e−07	3.036997e−05	6.084428	C9orf156	0.24023383	1.758839129	3.851998	0.0001606481	0.002979343	0.5421843	CYP2C19	0.860858871	2.421180257	3.264075	0.001304966	0.01394223	−1.376419	B3GNT5	0.410649286	3.244651850	2.870766	0.004564938	0.03425187	−2.501900
379	SLAMF7	1.30646374	3.12589853	5.233012	4.433897e−07	3.104781e−05	6.061059	BCL11B	0.43405057	1.803050510	3.850796	0.0001613802	0.002990831	0.5379839	UBXN11	0.363162628	2.746804796	3.263049	0.001309444	0.01398444	−1.379524	RORC	−0.649944742	2.026253952	−2.870509	0.004568490	0.03426884	−2.502592
380	AK093552	0.37548711	0.11118518	5.230877	4.478824e−07	3.125549e−05	6.051527	NOL11	0.29631385	4.140854452	3.850444	0.0001615955	0.002992730	0.5367524	AX747844	0.512891491	1.325891689	3.262740	0.001310793	0.01399321	−1.380457	TAX1BP1	−0.259700576	5.303500191	−2.869959	0.004576105	0.03431626	−2.504074
381	ACPP	0.89910782	1.40148752	5.230485	4.487111e−07	3.125549e−05	6.049779	ZBTB49	0.19256717	1.234298157	3.849126	0.0001624030	0.003005588	0.5321481	TRAM1L1	−0.182463359	0.428916176	−3.262261	0.001312889	0.01400996	−1.381906	GALM	0.433827370	4.301290350	2.869707	0.004579600	0.03433277	−2.504754
382	ZC3H12B	−0.31970859	0.58501884	−5.221328	4.685208e−07	3.254993e−05	6.008936	PRDM5	−0.26423170	0.520983555	−3.848612	0.0001627188	0.003009334	0.5303533	CHRNA6	0.062740565	0.099150883	3.262018	0.001313955	0.01401399	−1.382641	DCBLD2	−0.491040765	2.139719709	−2.869505	0.004582399	0.03434405	−2.505298
383	P2RY13	0.67673986	1.37227215	5.218905	4.739021e−07	3.283783e−05	5.998140	ST8SIA4	0.42302611	1.437709657	3.848098	0.0001630350	0.003013082	0.5285602	FKBP14	−0.284423039	2.354249504	−3.261934	0.001314323	0.01401399	−1.382895	EXOSC9	0.249032557	3.166424810	2.869134	0.004587549	0.03437294	−2.506297
384	CD3G	0.85658590	1.55525536	5.217117	4.779130e−07	3.302951e−05	5.990173	PRMT10	0.24422036	1.678042871	3.847223	0.0001635755	0.003019827	0.5255029	ZNF92	0.363935448	2.417278114	3.261723	0.001315250	0.01401771	−1.383534	C17orf101	0.227720070	2.733775230	2.868672	0.004593964	0.03440319	−2.507541
385	CENPK	0.72184367	2.36679072	5.209783	4.947101e−07	3.410159e−05	5.957520	GTF2H3	0.39368461	3.415347618	3.847139	0.0001636275	0.003019827	0.5252093	GPR146	−0.358340562	1.688531193	−3.261614	0.001315729	0.01401771	−1.383864	C10orf125	−0.553824338	3.562356847	−2.868657	0.004594178	0.03440319	−2.507582
386	FKBP10	−1.41334743	4.57547411	−5.206378	5.027018e−07	3.456270e−05	5.942372	BC030750	−0.39529676	1.293283114	−3.845808	0.0001644527	0.003032385	0.5205629	AF086132	0.352371376	2.998693751	3.261371	0.001316795	0.01402344	−1.384598	ATP2A2	0.263632132	5.588906393	2.868221	0.004600238	0.03443885	−2.508755
387	ADM2	−0.89310114	2.02003953	−5.205794	5.040846e−07	3.456822e−05	5.939776	FAM3A	−0.37209501	3.736699564	−3.845673	0.0001645365	0.003032385	0.5200926	DD181999	−0.869870676	10.060574695	−3.260954	0.001318629	0.01403659	−1.385860	MIR320C2	0.098895900	0.100524798	2.867575	0.004609240	0.03448965	−2.510495
388	KIR3DL2	0.06109901	0.05061596	5.201955	5.132712e−07	3.510749e−05	5.922705	CD276	−0.50304818	4.465765465	−3.843216	0.0001660709	0.003057159	0.5115192	LOC649133	0.043205151	0.014968023	3.260849	0.001319088	0.01403659	−1.386175	MRPL23	0.296979394	5.767920586	2.867472	0.004610677	0.03448965	−2.510772
389	BBC3	0.72919002	3.14270203	5.198598	5.214370e−07	3.557434e−05	5.907787	SOX21	1.02357210	1.156087088	3.843152	0.0001661111	0.003057159	0.5112954	MIR302B	0.099623082	0.219744102	3.259130	0.001326673	0.01411166	−1.391372	FIBCD1	−0.689903511	0.788639496	−2.867454	0.004610922	0.03448965	−2.510819
390	SNTA1	−0.91149690	2.84477753	−5.191950	5.379794e−07	3.660881e−05	5.878270	MSX2	0.88689834	2.543715775	3.842779	0.0001663454	0.003059123	0.5099938	SCN10A	0.008763452	0.007134478	3.258199	0.001330799	0.01414987	−1.394186	ZNF573	0.176729529	0.975520375	2.867072	0.004616261	0.03451985	−2.511849
391	C7orf41	−0.83768202	2.36396026	−5.190738	5.410504e−07	3.672362e−05	5.872890	CFLAR−AS1	0.30956691	1.879600294	3.842615	0.0001664484	0.003059123	0.5094223	HOXA9	−0.716012671	1.065748076	−3.257994	0.001331707	0.01415385	−1.394804	SV2A	−0.359569951	1.089629020	−2.865619	0.004636590	0.03466210	−2.515759
392	CD74	1.41956734	10.64293955	5.188983	5.455276e−07	3.693305e−05	5.865102	FLJ46066	0.04328632	0.016378072	3.841666	0.0001670463	0.003067988	0.5061104	RNaseP_nuc	0.039563010	0.010059969	3.257676	0.001333122	0.01416287	−1.395767	PCBP3	−0.303188486	0.778207812	−2.864540	0.004651746	0.03476561	−2.518662
393	SLC44A4	−1.71779769	5.89029069	−5.185974	5.532872e−07	3.736307e−05	5.851755	TIMP4	−0.61886224	1.110744913	−3.840073	0.0001680536	0.003084354	0.5005583	IGLL5	1.464372939	7.005471711	3.257563	0.001333623	0.01416287	−1.396107	POP1	0.277387865	1.588113048	2.863938	0.004660212	0.03481908	−2.520280
394	APOL3	0.88195051	3.01970815	5.183931	5.586158e−07	3.762717e−05	5.842697	CRSP8P	0.20155566	0.702101660	3.838955	0.0001687649	0.003095266	0.4966582	KIAA1598	−0.345292454	2.489757999	−3.257331	0.001334654	0.01416815	−1.396807	FAM19A5	−0.419461180	1.713058563	−2.863797	0.004662201	0.03482413	−2.520659
395	DQ570500	0.14073339	0.02805282	5.180747	5.670227e−07	3.809675e−05	5.828582	CCDC109B	0.51909440	3.084139014	3.838762	0.0001688874	0.003095374	0.4959881	ABHD14B	−0.359343098	4.820994230	−3.256966	0.001336278	0.01417972	−1.397910	ZDHHC3	0.261622044	3.140479452	2.862394	0.004682011	0.03496227	−2.524431
396	KLRC1	0.34663511	0.43096500	5.178281	5.736168e−07	3.844247e−05	5.817656	CDHR2	−1.18523576	2.497378847	−3.838233	0.0001692249	0.003099420	0.4941443	ZNF404	0.201807625	0.603156769	3.256741	0.001337282	0.01418471	−1.398591	DNAH11	−0.195901066	0.709721484	−2.861947	0.004688337	0.03499966	−2.525632
397	GRIN3A	0.15606455	0.18716394	5.175078	5.822922e−07	3.892557e−05	5.803473	ZC2HC1C	−0.31369510	1.116566204	−3.837933	0.0001694164	0.003100788	0.4930997	JTB	−0.295394801	6.324914462	−3.256437	0.001338635	0.01419338	−1.399507	PALM	−0.567198006	2.430668043	−2.861661	0.004692396	0.03502010	−2.526402
398	C1QA	1.18115059	6.49502189	5.174034	5.851485e−07	3.901823e−05	5.798849	TBXAS1	0.55482617	2.288149032	3.837642	0.0001696025	0.003102055	0.4920858	PRR21	0.012035581	0.006541161	3.256092	0.001340176	0.01420404	−1.400549	NFATC2IP	0.216185441	3.467352625	2.861147	0.004699679	0.03506336	−2.527782
399	C2CD4B	−1.43757541	2.76447994	−5.173336	5.870653e−07	3.904794e−05	5.795759	DNAJC4	−0.42206872	4.513847617	−3.836927	0.0001700607	0.003108292	0.4895947	ERO1LB	−0.472668296	1.640238889	−3.255626	0.001342258	0.01422043	−1.401956	immunoglobulin_heavy_chain	1.262543975	4.514450239	2.861066	0.004700834	0.03506336	−2.528000
400	HOXA5	−0.86413867	2.33386449	−5.168609	6.002084e−07	3.982232e−05	5.774839	CEPT1	0.25348730	3.074215587	3.836626	0.0001702543	0.003108567	0.4885441	DQ583972	−0.179701776	2.312660848	−3.254508	0.001347266	0.01426620	−1.405331	CYP2B6	−0.880363563	1.659889526	−2.860655	0.004706674	0.03509706	−2.529105

	Gene	logFC	AveExpr	t	P.Value	adj.P.Val	B	Gene	logFC	AveExpr	t	P.Value	adj.P.Val	B	Gene	logFC	AveExpr	t	P.Value	adj.P.Val	B	Gene	logFC	AveExpr	t	P.Value	adj.P.Val	B
401	NELF	−0.98338449	4.47845523	−5.163881	6.136369e−07	4.061175e−05	5.753934	NOB1	0.35606112	5.23218642	3.836539	0.0001703100	0.003108567	0.4882421	C4BPB	−0.841111371	1.870709905	−3.254422	0.001347653	0.01426620	−1.405591	WRAP53	0.24721828	2.35359232	2.860453	0.004709550	0.03510864	−2.529648
402	PRMT6	−0.66689187	2.82374048	−5.162689	6.170700e−07	4.073737e−05	5.748663	LINC00582	0.16552038	0.25283243	3.835833	0.0001707645	0.003114721	0.4857808	TMEM156	0.443804203	0.953782666	3.253563	0.001351515	0.01429647	−1.408184	ZNF17	0.17490186	1.57976012	2.860333	0.004711244	0.03511141	−2.529968
403	LOC283104	−0.44810653	1.72290741	−5.159922	6.251095e−07	4.116571e−05	5.736434	CCDC38	0.02583925	0.03541877	3.835628	0.0001708961	0.003114980	0.4850698	FAM156B	0.126203192	0.403536272	3.253537	0.001351631	0.01429647	−1.408262	HAUS6	0.29907799	1.87768150	2.860001	0.004715974	0.03513679	−2.530860
404	AV1S4A1N1T	0.34605007	0.41085694	5.159017	6.277594e−07	4.123789e−05	5.732437	LMNB1	0.67755451	4.20055278	3.835027	0.0001712841	0.003119910	0.4829755	TOR3A	0.298992898	4.480405855	3.253426	0.001352128	0.01429647	−1.408595	CD48	0.66549293	2.54019601	2.859902	0.004717391	0.03513748	−2.531127
405	C1QB	1.26145470	7.28351743	5.157323	6.327521e−07	4.146323e−05	5.724953	TRIM68	−0.31458317	2.07104768	−3.834448	0.0001716587	0.003124589	0.4809582	MRPS23	0.257002344	4.045237926	3.252732	0.001355260	0.01432387	−1.410691	GFM1	0.25079510	3.93936627	2.859527	0.004722738	0.03516744	−2.532135
406	EPPK1	−1.11905544	3.77365370	−5.152309	6.477520e−07	4.234160e−05	5.702820	BEGAIN	−0.31057910	0.67941610	−3.831012	0.0001738963	0.003163148	0.4690005	LOC100505812	0.275666321	1.423303049	3.252524	0.001356197	0.01432807	−1.411318	PELI2	0.42034671	2.12265499	2.859225	0.004727037	0.03518958	−2.532944
407	PPP3CA	−0.56565333	3.86810505	−5.151452	6.503525e−07	4.240714e−05	5.699035	LOC100270746	−0.34938935	0.71385340	−3.830286	0.0001743726	0.003169468	0.4664751	AK128707	0.238744678	0.338962475	3.251924	0.001358907	0.01435079	−1.413126	ZNF467	−0.37100812	2.15700781	−2.858408	0.004738707	0.03526656	−2.535137
408	OSBP2	−0.73488879	1.37212855	−5.148059	6.607392e−07	4.297882e−05	5.684068	FAM133B	0.38653173	1.73440147	3.830119	0.0001744826	0.003169468	0.4658929	PSMA5	0.271146432	4.548978572	3.251727	0.001359801	0.01435079	−1.413722	RAD54L2	0.20717409	2.03775774	2.858217	0.004741442	0.03527702	−2.535650
409	BC039551	0.31953638	0.54375628	5.145256	6.694411e−07	4.332992e−05	5.671709	RANBP17	−0.26903102	0.41769102	−3.828632	0.0001754624	0.003185087	0.4607227	DDX6	0.284019749	4.537546345	3.251681	0.001360008	0.01435079	−1.413860	SRMS	−0.55400776	1.55655937	−2.858078	0.004743427	0.03528189	−2.536023
410	LYRM2	−0.32725007	2.12014705	−5.144807	6.708461e−07	4.332992e−05	5.669728	TDRD6	0.19842258	0.33948250	3.825419	0.0001775981	0.003221652	0.4495535	FADS1	−0.564534291	2.368425726	−3.251570	0.001360511	0.01435079	−1.414195	C17orf82	−0.19116357	0.39066042	−2.857509	0.004751584	0.03533267	−2.537551
411	CIB2	−1.03280230	2.32436141	−5.144493	6.718296e−07	4.332992e−05	5.668345	BCL2L10	−0.58737106	0.80409556	−3.823427	0.0001789347	0.003241501	0.4426323	RAB3IL1	−0.439756451	2.485771251	−3.251330	0.001361596	0.01435654	−1.414918	PPWD1	0.20790484	2.96612858	2.857389	0.004753307	0.03533557	−2.537873
412	TRBV7−6	0.50676145	0.59778512	5.144226	6.726676e−07	4.332992e−05	5.667167	C16orf95	0.33906893	1.42115564	3.823424	0.0001789366	0.003241501	0.4426222	PRKAR1B	0.386103725	3.200830610	3.251038	0.001362919	0.01436478	−1.415798	TMEM19	0.24444739	2.83773178	2.857228	0.004755611	0.03534280	−2.538304
413	ULK2	−0.44898517	1.29376166	−5.143159	6.760267e−07	4.344085e−05	5.662462	PTPN4	0.22094280	1.71165580	3.821699	0.0001801015	0.003260378	0.4366325	WBP11	0.248359066	5.057958834	3.250574	0.001365028	0.01438130	−1.417199	MIR3688−1	0.02901003	0.01071631	2.856957	0.004759500	0.03536181	−2.539031
414	ZNF267	0.41819403	2.69777184	5.140529	6.843706e−07	4.383433e−05	5.650875	HDAC5	−0.41226904	3.13047398	−3.820330	0.0001810316	0.003274982	0.4318777	CCR1	0.476275887	2.347021655	3.250179	0.001366822	0.01439135	−1.418388	GHRLOS2	0.15746997	0.80865021	2.856666	0.004763690	0.03538303	−2.539813
415	SLC35D2	0.54830264	3.50363538	5.140190	6.854533e−07	4.383433e−05	5.649382	BC032899	−0.35165457	0.62897397	−3.819391	0.0001816720	0.003284327	0.4286185	WBSCR27	−0.644441390	1.885857390	−3.250125	0.001367067	0.01439135	−1.418551	MCM6	0.35211612	3.91660232	2.856532	0.004765614	0.03538742	−2.540173
416	CASS4	0.33252626	0.61473552	5.138099	6.921710e−07	4.415751e−05	5.640170	ILVBL	−0.37824229	4.39669248	−3.818571	0.0001822325	0.003292218	0.4257752	NAA50	0.278529123	4.404472914	3.249826	0.001368427	0.01439996	−1.419452	DEFB123	0.04423781	0.02345370	2.856367	0.004767992	0.03539517	−2.540616
417	CX3CL1	1.15452539	3.31649530	5.137164	6.951964e−07	4.424416e−05	5.636051	TCRBV21S1	0.19959579	0.22531638	3.817935	0.0001826685	0.003297849	0.4235697	DHX33	0.270051423	2.492480653	3.249534	0.001369759	0.01440716	−1.420333	PRKD3	0.31777262	2.22772046	2.855896	0.004774765	0.03543349	−2.541878
418	MFSD11	0.32576385	4.55377215	5.134640	7.034225e−07	4.463206e−05	5.624941	CKMT1A	−0.72435230	2.26622166	−3.817013	0.0001833029	0.003307053	0.4203699	SPHAR	−0.337798821	2.882017988	−3.249366	0.001370524	0.01440716	−1.420839	LINC00494	−0.24140754	0.35308973	−2.855823	0.004775823	0.03543349	−2.542076
419	ZNF222	0.32841509	0.99420305	5.134265	7.046547e−07	4.463206e−05	5.623288	EWSR1	0.20292146	5.19965620	3.816820	0.0001834357	0.003307200	0.4197015	TMEM62	0.391460425	3.545845292	3.249319	0.001370740	0.01440716	−1.420982	SSBP4	0.24000652	4.76726848	2.854853	0.004789811	0.03552733	−2.544676
420	BRIP1	0.48195801	1.03753265	5.132485	7.105221e−07	4.489654e−05	5.615456	SMAD5	−0.30218007	3.23065056	−3.816362	0.0001837516	0.003310648	0.4181132	IFFO2	0.460735547	2.682981794	3.247710	0.001378093	0.01447870	−1.425828	UHRF1	0.47801217	2.37058194	2.854541	0.004794312	0.03553939	−2.545511
421	AK055967	−0.63382051	1.04173025	−5.130127	7.183707e−07	4.528465e−05	5.605081	TMEM106A	0.40209168	2.03333877	3.815925	0.0001840537	0.003313840	0.4165972	PML	0.367289643	3.656510633	3.247581	0.001378684	0.01447918	−1.426217	ANKRD6	−0.23993696	1.13720709	−2.854525	0.004794546	0.03553939	−2.545555
422	IL12RB1	0.70599434	1.49239029	5.124036	7.390352e−07	4.637983e−05	5.578298	HLA−DQA1	1.22802247	4.21949318	3.815267	0.0001845092	0.003319789	0.4143160	ST3GAL4	−0.564236418	2.606713966	−3.246733	0.001382579	0.01451243	−1.428773	CEP112	−0.25057411	1.08509263	−2.854462	0.004795454	0.03553939	−2.545723
423	EED	0.37469205	2.78187023	5.123977	7.392392e−07	4.637983e−05	5.578037	LOC100129794	−0.34756144	0.62553392	−3.812949	0.0001861227	0.003345546	0.4062806	DQ596663	0.018633263	0.002898322	3.246654	0.001382943	0.01451243	−1.429011	LRRC37A	0.09006545	0.17361275	2.854308	0.004797689	0.03554599	−2.546137
424	HSPB1	−1.05308609	8.82273008	−5.119643	7.542997e−07	4.721311e−05	5.558992	MIR298	0.05146323	0.01941935	3.812849	0.0001861928	0.003345546	0.4059329	TRA2B	0.191528435	3.847449949	3.244220	0.001394174	0.01462450	−1.436337	LINC00305	0.05581908	0.01011492	2.854216	0.004799024	0.03554599	−2.546385
425	KCNAB3	0.16914010	0.35482366	5.116305	7.660985e−07	4.783880e−05	5.544335	BC018779	−0.22565596	1.19212707	−3.812084	0.0001867280	0.003352891	0.4032844	FGD2	0.412874913	1.341568860	3.243927	0.001395533	0.01463298	−1.437220	GMPR	−0.52809242	2.18173196	−2.853378	0.004811156	0.03562591	−2.548630
426	GABBR1	1.52737113	4.23789532	5.111329	7.840225e−07	4.884313e−05	5.522497	ROCK1	−0.35153348	3.82792239	−3.811526	0.0001871199	0.003357657	0.4013495	KLLN	0.180270694	0.459653443	3.242226	0.001403441	0.01471008	−1.442338	ZNF615	−0.27129617	1.36960654	−2.852956	0.004817277	0.03566128	−2.549760
427	LEPREL4	−0.88885038	3.57227126	−5.108996	7.925649e−07	4.925967e−05	5.512265	GOLGA8A	−0.51351220	1.73107200	−3.809639	0.0001884497	0.003378276	0.3948148	ITGA5	−0.717552624	4.949634348	−3.241936	0.001404791	0.01471842	−1.443209	PPP3CC	0.32934343	2.54050316	2.852579	0.004822759	0.03569191	−2.550771
428	CTSW	1.07985313	2.27642161	5.105826	8.043179e−07	4.987335e−05	5.498367	BC037833	0.60691539	0.66131815	3.809344	0.0001886585	0.003378276	0.3937927	LOC283033	0.008413107	0.005183989	3.241658	0.001406087	0.01472618	−1.444044	GALNTL2	−0.35542811	0.85293729	−2.852196	0.004828320	0.03572311	−2.551796
429	PRPS2	0.57874746	4.53020209	5.095607	8.433701e−07	5.217296e−05	5.453605	LCP2	0.67587521	3.31565661	3.809030	0.0001888804	0.003378276	0.3927079	KBTBD7	−0.318197094	2.008582279	−3.241249	0.001408000	0.01474040	−1.445275	DIRAS1	−0.33721411	0.65262031	−2.852062	0.004830281	0.03572766	−2.552157
430	HOXC4	0.84965131	2.05813351	5.092399	8.560052e−07	5.283145e−05	5.439567	EPHA4	0.51476362	1.14101312	3.809002	0.0001889009	0.003378276	0.3926081	CAPN13	−0.527458800	1.024377399	−3.240517	0.001411424	0.01477042	−1.447475	COL6A2	−0.58842569	6.31549627	−2.851596	0.004837061	0.03576783	−2.553404
431	SOWAHA	−1.08161803	1.85805837	−5.081372	9.008539e−07	5.547045e−05	5.391361	ALKBH8	0.23199336	1.44834884	3.808995	0.0001889054	0.003378276	0.3925858	AX747163	0.030852905	0.038814579	3.240252	0.001412667	0.01477760	−1.448272	CHRNA10	0.11146705	0.40284815	2.851209	0.004842712	0.03579696	−2.554442
432	OBSL1	−0.97249497	2.14871147	−5.078834	9.114946e−07	5.599573e−05	5.380278	PARPBP	0.45220040	1.72587760	3.808741	0.0001890857	0.003379223	0.3917056	BTN3A1	0.504192536	3.516557274	3.239591	0.001415772	0.01480425	−1.450261	WNT5B	−0.49637810	1.65438738	−2.851141	0.004843698	0.03579696	−2.554623
433	TCRVA15	0.58565333	0.79062270	5.075703	9.247869e−07	5.668111e−05	5.366613	GTF2H2C	0.15750607	0.44465765	3.808417	0.0001893155	0.003381052	0.3905850	DQ586243	−0.149983965	0.226372967	−3.239135	0.001417915	0.01481797	−1.451630	NAPG	−0.29506238	3.05319941	−2.850395	0.004854595	0.03586751	−2.556621
434	CCNI2	−0.79224438	2.12352484	−5.072737	9.375530e−07	5.733115e−05	5.353673	BNIP3	−0.67030989	2.07205267	−3.807240	0.0001901536	0.003393736	0.3865092	APOA1BP	−0.363244910	5.964078382	−3.239074	0.001418201	0.01481797	−1.451813	HDAC6	−0.25169572	2.60840811	−2.849964	0.004860907	0.03589716	−2.557776
435	HOXB9	−1.38660300	2.17706903	−5.070707	9.463916e−07	5.773859e−05	5.344817	TMEM61	−0.65720893	1.05254576	−3.806750	0.0001905034	0.003396707	0.3848133	TMEM151A	−0.664666428	1.109428258	−3.238810	0.001419444	0.01482512	−1.452606	B3GNT9	−0.32146741	3.22168093	−2.849849	0.004862588	0.03589716	−2.558083
436	MORC2	−0.33513725	3.26217249	−5.068309	9.569334e−07	5.824784e−05	5.334363	C11orf57	0.24758707	2.68083787	3.806648	0.0001905760	0.003396707	0.3844617	MYH10	−0.575204043	2.812200387	−3.238590	0.001420485	0.01482746	−1.453270	DCAF8	−0.17607340	4.12603657	−2.849829	0.004862871	0.03589716	−2.558135
437	PCBP1−AS1	0.32219546	1.30683071	5.064915	9.720498e−07	5.903256e−05	5.319571	KCNH2	−0.91470517	1.78630868	−3.806167	0.0001909198	0.003399651	0.3827988	SAT1	0.473555039	7.096853307	3.238448	0.001421151	0.01482746	−1.453695	AK097878	0.30250920	1.61403899	2.849751	0.004864019	0.03589716	−2.558345
438	ITGB7	0.76561746	2.17693077	5.063616	9.778992e−07	5.925221e−05	5.313909	BCAT2	0.45504179	3.22960780	3.806059	0.0001909974	0.003399651	0.3824238	MPHOSPH9	0.359143172	1.979577329	3.238407	0.001421345	0.01482746	−1.453818	SAMD1	0.27339268	5.03367420	2.849366	0.004869665	0.03591845	−2.559376
439	LGMN	0.61430196	6.11947011	5.052745	1.028185e−06	6.215719e−05	5.266591	N6AMT1	0.22605144	1.29464694	3.805864	0.0001911370	0.003399855	0.3817497	DPYSL3	−0.862439041	4.645724772	−3.237575	0.001425276	0.01486263	−1.456319	NQO2	−0.29311398	2.71847081	−2.849303	0.004870584	0.03591845	−2.559543
440	ZXDB	−0.45180932	2.09850930	−5.051907	1.032163e−06	6.225585e−05	5.262947	FBXL2	−0.32963869	0.91311978	−3.805653	0.0001912886	0.003400273	0.3810180	AK090854	0.007569289	0.003992679	3.236285	0.001431386	0.01492048	−1.460192	MCM9	0.18850523	1.63426015	2.849258	0.004871238	0.03591845	−2.559663
441	OMP	0.44099232	0.60285308	5.049483	1.043754e−06	6.281101e−05	5.252410	TEX9	0.27944733	0.66012654	3.805304	0.0001915390	0.003402446	0.3798109	PRKX	0.415102388	2.560069805	3.235800	0.001433688	0.01493861	−1.461648	FGF20	0.41919835	0.35483308	2.849185	0.004872317	0.03591845	−2.559860
442	ZNF511	−1.02176736	4.52192458	−5.048996	1.046101e−06	6.281101e−05	5.250291	DQ599403	0.05341203	0.01395735	3.802760	0.0001933741	0.003432746	0.3710137	SNAPC2	−0.313797109	3.465491739	−3.235522	0.001435011	0.01494653	−1.462483	MIR7−3	0.02828459	0.01106321	2.848985	0.004875255	0.03593013	−2.560396
443	ESPN	−1.26557055	2.37260821	−5.037787	1.101496e−06	6.598782e−05	5.201607	GPN1	0.21401736	4.15845418	3.801341	0.0001944053	0.003447975	0.3661070	AK027145	0.152113709	0.158396506	3.235403	0.001435581	0.01494660	−1.462842	EMR1	0.21842624	0.49492250	2.848240	0.004886198	0.03600078	−2.562388
444	BC042098	0.17623477	0.29014739	5.036224	1.109442e−06	6.631415e−05	5.194826	VEGFB	−0.48483701	5.12250524	−3.801222	0.0001944918	0.003447975	0.3656962	DQ594785	0.019844664	0.002989305	3.235081	0.001437113	0.01495668	−1.463808	FLRT3	0.68930212	2.25448024	2.847958	0.004890346	0.03601815	−2.563143
445	DSG2	−1.19747906	5.71400128	−5.034191	1.119860e−06	6.678641e−05	5.186009	CALCOCO2	0.33473889	5.00321531	3.800891	0.0001947329	0.003449944	0.3645534	FATE1	0.161406899	0.222278102	3.234883	0.001438056	0.01495689	−1.464403	TMCO2	0.03311290	0.03123212	2.847895	0.004891270	0.03601815	−2.563311
446	SIRPG	0.97725138	1.72183126	5.031125	1.135757e−06	6.743260e−05	5.172712	AX748236	0.03249733	0.03876615	3.800330	0.0001951424	0.003454893	0.3626155	GAL3ST4	0.435852103	2.137023970	3.234840	0.001438260	0.01495689	−1.464531	CCDC73	0.29253828	1.81596081	2.847274	0.004900428	0.03607558	−2.564973
447	PCNA	0.80588228	5.98254559	5.031121	1.135777e−06	6.743260e−05	5.172695	JA040723	−0.52515107	11.40303303	−3.799424	0.0001958058	0.003464326	0.3594847	PILRA	0.515141691	2.368655973	3.233825	0.001443106	0.01498305	−1.467576	GEMIN2	0.21013525	2.11036384	2.846683	0.004909151	0.03612977	−2.566554
448	GRIN2D	−1.14446242	2.95324720	−5.029990	1.141691e−06	6.763247e−05	5.167795	IQCF2	0.02384053	0.01282813	3.799039	0.0001960886	0.003467018	0.3581534	TRAK1	0.411626946	3.822998542	3.233788	0.001443286	0.01498305	−1.467689	RAD51	0.40497353	2.36008807	2.846458	0.004912473	0.03614420	−2.567155
449	SULF2	−1.04510815	4.94231093	−5.027578	1.154416e−06	6.823398e−05	5.157339	EIF3L	−0.34833917	6.52588916	−3.797598	0.0001971490	0.003483448	0.3531779	ZNF317	0.209643267	3.122272818	3.233551	0.001444419	0.01498305	−1.468400	FAM176A	−0.48836367	1.17678476	−2.845781	0.004922484	0.03620782	−2.568965
450	GGT6	−1.70819849	2.47724653	−5.022609	1.181055e−06	6.965339e−05	5.135820	CCL4	0.93455077	3.80284686	3.797000	0.0001975908	0.003486865	0.3511131	ADRA2A	−1.118345889	2.932897165	−3.233545	0.001444450	0.01498305	−1.468419	PIK3R4	0.18541428	2.86542280	2.845102	0.004932557	0.03626830	−2.570781
451	IGSF6	0.88879437	2.50108117	5.021122	1.189147e−06	6.997508e−05	5.129380	TBC1D21	0.02583103	0.01193423	3.796981	0.0001976052	0.003486865	0.3510457	AF086294	0.024252756	0.008305843	3.233499	0.001444666	0.01498305	−1.468555	RAB3B	−0.44836213	0.80483650	−2.845015	0.004933836	0.03626830	−2.571012
452	PVRL1	0.86497390	3.72459358	5.020287	1.193709e−06	7.008814e−05	5.125768	SLC16A2	−0.80979192	2.47576563	−3.794952	0.0001991114	0.003511107	0.3440417	MGLL	−0.642748934	4.338098526	−3.233497	0.001444678	0.01498305	−1.468562	FAM86FP	−0.16451421	0.50700401	−2.844950	0.004934807	0.03626830	−2.571186
453	RAB33A	0.27728406	0.47319484	5.014682	1.224796e−06	7.166960e−05	5.101519	ZRANB3	0.26923461	0.98264459	3.794366	0.0001995480	0.003514930	0.3420214	SMO	−0.531218259	2.081010986	−3.233487	0.001444727	0.01498305	−1.468593	ING5	0.20833065	1.81023505	2.844807	0.004936930	0.03627072	−2.571569
454	HES4	−0.79898496	3.78295860	−5.014460	1.226045e−06	7.166960e−05	5.100558	NIT1	−0.23734830	3.52650900	−3.794306	0.0001995930	0.003514930	0.3418131	FAM81A	0.465657446	1.457226746	3.231113	0.001456135	0.01509546	−1.475714	SnoU83B	0.05956096	0.03048824	2.844744	0.004937869	0.03627072	−2.571738
455	RIBC2	−0.68399152	1.03717009	−5.011482	1.242897e−06	7.249505e−05	5.087682	MIB2	−0.42875799	2.97463601	−3.791621	0.0002016078	0.003548056	0.3325493	FBXL19	0.310006152	3.301196528	3.230689	0.001458183	0.01511078	−1.476986	TRIM35	0.21279153	2.62893662	2.844478	0.004941822	0.03628004	−2.572449
456	BV6S4−BJ2S2	0.87939426	1.46622628	5.007690	1.264676e−06	7.354334e−05	5.071300	NUDT16P1	−0.53192676	2.20022282	−3.790763	0.0002022555	0.003557096	0.3295908	FAM103A1	−0.310115746	1.994625681	−3.230396	0.001459598	0.01511955	−1.477864	ZNF69	0.41465273	1.82918260	2.844474	0.004941872	0.03628004	−2.572458
457	TAF2	−0.37075596	3.32319160	−5.007391	1.266412e−06	7.354334e−05	5.070006	SLFN14	0.03496511	0.04396262	3.789462	0.0002032413	0.003571666	0.3251063	ESPNP	−0.229556298	0.265206529	−3.229934	0.001461834	0.01513680	−1.479250	MBTD1	0.22209115	2.20053161	2.844277	0.004944802	0.03629151	−2.572985
458	SPRN	0.29790451	1.10659025	5.005419	1.277899e−06	7.403772e−05	5.061491	DIP2C	−0.44144278	1.89389215	−3.789315	0.0002033531	0.003571666	0.3245994	SCARNA15	0.104813492	0.228850299	3.229595	0.001463475	0.01514788	−1.480265	LAPTM5	0.64869657	6.37335828	2.843916	0.004950169	0.03632086	−2.573949
459	ZDHHC18	0.35593094	3.49284652	5.004974	1.280505e−06	7.403772e−05	5.059570	ILDR1	−0.59695705	2.06033031	−3.788673	0.0002038412	0.003577872	0.3223882	BC041470	0.142704574	0.323160952	3.229425	0.001464298	0.01515049	−1.480774	ABCF2	0.22468363	4.03450615	2.843564	0.004955412	0.03634928	−2.574889
460	IDO2	0.06581942	0.06908082	5.002701	1.293904e−06	7.464980e−05	5.049753	MS4A4A	0.69955143	2.70316266	3.788032	0.0002043305	0.003584090	0.3201770	KIF18A	0.458091449	1.864061673	3.229012	0.001466304	0.01516533	−1.482013	PRPH2	−0.15291364	0.43958730	−2.843342	0.004958724	0.03636195	−2.575483
461	RTCA	0.32335121	3.43993656	5.002093	1.297505e−06	7.469520e−05	5.047132	MAP4K1	0.78965920	1.98718504	3.787338	0.0002048605	0.003591013	0.3177881	CYP2W1	−1.360871418	1.922855449	−3.228778	0.001467440	0.01517117	−1.482715	SPARC	−0.51111215	8.13966995	−2.843265	0.004959880	0.03636195	−2.575690
462	CHPF	−0.68788720	5.94030340	−4.998015	1.321948e−06	7.593763e−05	5.029532	SRSF9	0.26617033	5.70522835	3.786825	0.0002052539	0.003595534	0.3160187	XCL1	0.322537535	0.649178576	3.225166	0.001485086	0.01534762	−1.493534	GLCCI1	0.31912236	2.02571092	2.843017	0.004963576	0.03636999	−2.576352
463	MB21D1	0.62874098	2.95784456	4.992353	1.356616e−06	7.776077e−05	5.005122	FAM172BP	0.05549716	0.09251206	3.785410	0.0002063415	0.003611891	0.3111454	AKR1B1	0.590058308	4.795692721	3.224466	0.001488528	0.01537231	−1.495629	COASY	0.26309337	4.41676256	2.843007	0.004963717	0.03636999	−2.576377
464	BC038465	0.09286335	0.08411028	4.988454	1.381007e−06	7.898827e−05	4.988320	ORC1	0.57275606	1.98575071	3.785251	0.0002064638	0.003611891	0.3105989	APOL1	0.687176897	5.450229803	3.224444	0.001488633	0.01537231	−1.495693	DTL	0.45982468	2.52313301	2.842855	0.004965990	0.03637660	−2.576784
465	LPAR6	0.62482171	2.17057406	4.985753	1.398147e−06	7.979663e−05	4.976691	MDK	0.80450490	6.73711759	3.784930	0.0002067116	0.003611891	0.3094927	JAK3	0.536257425	2.274135255	3.224104	0.001490310	0.01538364	−1.496712	OSBPL3	0.36095350	3.02547483	2.842714	0.004968094	0.03638197	−2.577161
466	GFI1	0.62332542	1.13410097	4.984382	1.406925e−06	8.012529e−05	4.970790	LOC389641	0.45332677	1.74964636	3.784904	0.0002067320	0.003611891	0.3094016	NDP	−0.209081636	0.283184745	−3.223979	0.001490926	0.01538402	−1.497086	SEMA6C	−0.36148263	1.62901722	−2.842613	0.004969601	0.03638296	−2.577430
467	MGA	−0.42918391	2.08187792	−4.983663	1.411553e−06	8.021672e−05	4.967694	ST7−AS1	0.35735318	1.58722480	3.782880	0.0002082999	0.003636889	0.3024346	CHP2	−1.029703133	1.383936233	−3.222268	0.001499385	0.01546529	−1.502206	GNAI3	0.15904238	4.25375340	2.840863	0.004995808	0.03656065	−2.582103
468	HOXC11	−1.01895003	1.56418856	−4.982478	1.419210e−06	8.047953e−05	4.962593	MTG1	0.31271271	3.91759360	3.782304	0.0002087480	0.003642317	0.3004528	DTNB	0.406044084	1.914189266	3.222039	0.001500518	0.01547096	−1.502890	NRL	0.14607197	0.92380630	2.840808	0.004996626	0.03656065	−2.582249
469	PUS10	0.32131359	1.36022593	4.977101	1.454460e−06	8.230259e−05	4.939463	RRM1	0.42190507	4.18341455	3.781847	0.0002091047	0.003646144	0.2988785	PAQR6	0.320848195	0.937994276	3.220727	0.001507038	0.01553151	−1.506814	DACT2	−0.65070892	0.79261070	−2.840558	0.005000390	0.03657657	−2.582918
470	TUG1	−0.39207803	4.44540414	−4.974597	1.471161e−06	8.307053e−05	4.928700	UNC93B1	−0.57421332	5.31953757	−3.781389	0.0002094620	0.003649975	0.2973044	BC069739	0.223892315	2.195548967	3.220622	0.001507561	0.01553151	−1.507128	ACTL10	−0.32701467	1.50791583	−2.840480	0.005001559	0.03657657	−2.583126
471	HEPH	−1.20665007	3.44243683	−4.972494	1.485337e−06	8.369291e−05	4.919660	HSPA1A	−0.89086514	4.55871662	−3.780455	0.0002101933	0.003660315	0.2940910	DEFB116	0.041330365	0.011284300	3.220436	0.001508486	0.01553501	−1.507682	OR6C75	0.03967324	0.01154585	2.840171	0.005006210	0.03660050	−2.583951
472	MCM5	0.60991632	3.96624316	4.971801	1.490035e−06	8.377974e−05	4.916683	KHSRP	0.29750811	5.96793896	3.779931	0.0002106045	0.003665071	0.2922889	DKK1	1.290678949	1.889781239	3.220069	0.001510319	0.01554785	−1.508781	SRGAP3	0.17939830	0.48091767	2.839877	0.005010626	0.03662269	−2.584735
473	GTPBP8	0.37547424	2.63162464	4.969450	1.506081e−06	8.442285e−05	4.906586	JA429044	0.07952847	0.02568292	3.779484	0.0002109561	0.003667468	0.2907509	NR3C2	−0.416080230	1.747616247	−3.219817	0.001511576	0.01555086	−1.509534	RELL2	−0.30645196	1.68713956	−2.839692	0.005013411	0.03663072	−2.585228
474	SOX2−OT	0.21684927	0.19681278	4.968820	1.510414e−06	8.442285e−05	4.903878	AK125860	0.20133229	0.61111991	3.779405	0.0002110187	0.003667468	0.2904775	PHTF2	0.227194887	1.907483356	3.219775	0.001511785	0.01555086	−1.509659	FGFR1	−0.58394048	2.97467585	−2.839621	0.005014484	0.03663072	−2.585419
475	KIAA0101	0.88415362	4.03537318	4.968732	1.511016e−06	8.442285e−05	4.903502	ZGLP1	0.18144256	0.43843009	3.777733	0.0002123382	0.003687986	0.2847301	GPC4	0.600290977	3.469859343	3.219556	0.001512883	0.01555086	−1.510316	CD40LG	0.38688167	0.96099062	2.839316	0.005019076	0.03665417	−2.586232
476	LOC439949	0.61421263	1.07990567	4.964537	1.540163e−06	8.587056e−05	4.885492	LGALS9	0.63087836	6.34054380	3.776817	0.0002130647	0.003698184	0.2815811	CCDC34	0.445084344	2.955588699	3.219436	0.001513481	0.01555086	−1.510674	C16orf52	0.12984851	1.40992878	2.838902	0.005025311	0.03668961	−2.587335
477	AK124173	0.24724809	0.54555279	4.962259	1.556218e−06	8.658382e−05	4.875717	AK131347	0.04891757	0.08977267	3.776371	0.0002134190	0.003701913	0.2800491	RASGEF1A	−0.547250789	1.339038428	−3.219424	0.001513542	0.01555086	−1.510710	MIR877	0.14131548	0.38130759	2.838585	0.005030111	0.03671314	−2.588183
478	CACNG8	0.33669138	0.24443754	4.959609	1.575098e−06	8.745089e−05	4.864351	TEAD2	−0.63766153	3.20078795	−3.774516	0.0002148997	0.003723186	0.2736751	AOC2	0.142841188	0.338201462	3.219268	0.001514320	0.01555284	−1.511175	SLC35B2	−0.35590159	4.85075226	−2.838506	0.005031301	0.03671314	−2.588393
479	PLCH1	0.58679335	1.47203775	4.957967	1.586905e−06	8.792249e−05	4.857313	MT1L	−0.73387970	1.88111230	−3.774483	0.0002149260	0.003723186	0.2735622	C11orf58	0.190111132	4.687558177	3.218995	0.001515689	0.01556088	−1.511993	NKRF	0.24921737	2.19597650	2.837629	0.005044567	0.03679983	−2.590732
480	NRAP	0.60549527	0.63469383	4.956952	1.594249e−06	8.814536e−05	4.852961	HNRNPC	0.22060794	5.99961759	3.773017	0.0002161036	0.003741143	0.2685250	CEP78	0.291726556	1.878671160	3.218589	0.001517719	0.01557569	−1.513204	C7orf25	0.18522601	4.42851671	2.837458	0.005047149	0.03680854	−2.591187
481	AK095267	0.31016399	0.62784159	4.956410	1.598185e−06	8.817929e−05	4.850637	PPP2R5A	−0.28313826	4.52157760	−3.772095	0.0002168470	0.003751565	0.2653593	UCP3	0.093734306	0.328981648	3.218368	0.001518829	0.01558106	−1.513866	HDAC9	0.42211454	1.27822551	2.837121	0.005052256	0.03683567	−2.592085
482	NKD2	−1.25664080	1.66443161	−4.954248	1.613975e−06	8.886572e−05	4.841371	PHGDH	−0.87751496	2.70873844	−3.771202	0.0002175691	0.003759171	0.2622943	NCAPH2	0.289404812	3.927524159	3.217960	0.001520876	0.01559603	−1.515084	SNORD91B	0.14399824	0.23728568	2.836674	0.005059047	0.03687505	−2.593278
483	X06774	0.38005275	0.70094979	4.952602	1.626096e−06	8.934775e−05	4.834319	ARHGAP9	0.66057527	2.01659364	3.771163	0.0002176008	0.003759171	0.2621604	EGR4	−0.349784197	0.483577700	−3.216953	0.001525941	0.01564193	−1.518092	ARAP2	0.30827776	1.71991605	2.836047	0.005068561	0.03692222	−2.594947
484	CIITA	1.18097140	2.73939815	4.949053	1.652534e−06	9.061282e−05	4.819120	ADIPOR1	−0.27573331	6.04563914	−3.770863	0.0002178438	0.003759171	0.2611312	LOC389634	0.052001983	0.108516940	3.216311	0.001529179	0.01566906	−1.520010	HSPH1	0.39947140	4.67816482	2.835996	0.005069340	0.03692222	−2.595084
485	MYO7B	−1.58469020	2.57411115	−4.947035	1.667750e−06	9.108618e−05	4.810482	MOB3B	0.55405738	2.73045262	3.770693	0.0002179822	0.003759171	0.2605459	GGA3	0.206713176	3.368688284	3.215314	0.001534220	0.01571465	−1.522987	ACE	−0.41552214	3.02020977	−2.835973	0.005069692	0.03692222	−2.595145
486	ENDOV	0.39728420	1.19809803	4.946998	1.668031e−06	9.108618e−05	4.810324	LOC402160	0.14241705	0.16791459	3.770586	0.0002180688	0.003759171	0.2601798	UGT2A3	1.120731023	1.559612488	3.214950	0.001536063	0.01572746	−1.524074	BC038532	0.03011347	0.04323504	2.835619	0.005075085	0.03695135	−2.596090
487	AV2S1A1	0.76422875	1.06420593	4.945106	1.682421e−06	9.158744e−05	4.802229	IKZF3	0.82264214	1.90450550	3.770503	0.0002181365	0.003759171	0.2598938	MIR191	0.051698638	0.038583617	3.214816	0.001536741	0.01572834	−1.524473	FMO2	0.53062657	1.51658852	2.835467	0.005077402	0.03695808	−2.596495
488	BC030111	0.09198101	0.13161139	4.944885	1.684113e−06	9.158744e−05	4.801282	VLDLR	−0.70355475	1.62588879	−3.770200	0.0002183826	0.003760970	0.2588545	RPL35	0.324217484	9.744512784	3.214064	0.001540563	0.01576137	−1.526720	KIAA1841	0.24449752	1.29846693	2.835311	0.005079766	0.03696515	−2.596909
489	AY006148	0.89879619	1.53770015	4.942675	1.701096e−06	9.227484e−05	4.791826	OASL	0.87026268	3.35973588	3.768057	0.0002201316	0.003785930	0.2515013	HLA−E	0.417716641	8.107890559	3.213826	0.001541769	0.01576322	−1.527428	CERKL	0.30951553	1.59976337	2.834950	0.005085278	0.03699512	−2.597872
490	AL157440	−1.12579201	2.63753967	−4.942337	1.703707e−06	9.227484e−05	4.790381	RMI2	0.58987702	2.85087969	3.767807	0.0002203369	0.003785930	0.2506421	HEXIM2	0.306860524	1.857811821	3.213729	0.001542264	0.01576322	−1.527719	DIO3AS	−0.34634156	0.54962053	−2.834835	0.005087032	0.03699774	−2.598178
491	CHPT1	0.64880301	3.66773566	4.940395	1.718788e−06	9.290207e−05	4.782076	C2orf44	0.22458195	1.53611078	3.767763	0.0002203729	0.003785930	0.2504915	C6orf123	−0.336880780	0.549550321	−3.213620	0.001542817	0.01576322	−1.528043	HEXDC	0.29134515	2.65786269	2.834666	0.005089606	0.03700632	−2.598627
492	AMZ1	0.16686527	0.23323213	4.930612	1.796742e−06	9.691817e−05	4.740282	DQ584235	0.07984239	0.02961106	3.767727	0.0002204025	0.003785930	0.2503677	HAUS3	0.192702464	1.548890124	3.213561	0.001543119	0.01576322	−1.528220	SOWAHC	−0.38512174	3.13504931	−2.834299	0.005095223	0.03703003	−2.599607
493	mir−34	0.54034880	1.10232474	4.929327	1.807237e−06	9.728653e−05	4.734795	SLC29A4	−1.00185930	1.73813146	−3.767019	0.0002209835	0.003793454	0.2479414	NPM2	−0.376519495	0.646687717	−3.213140	0.001545265	0.01577906	−1.529477	NEURL3	0.50736268	1.03481736	2.834257	0.005095861	0.03703003	−2.599718
494	C4orf48	−1.04236529	3.89719486	−4.928798	1.811573e−06	9.732255e−05	4.732537	BV13S6J2.1	0.45112005	0.86080131	3.765537	0.0002222054	0.003811728	0.2428591	SIGLEC9	0.338835922	1.182683967	3.212415	0.001548962	0.01581074	−1.531639	C3orf17	0.16623462	2.73082788	2.834168	0.005097223	0.03703003	−2.599956
495	BCL2L11	0.50404476	2.64227439	4.918074	1.901677e−06	1.017487e−04	4.686807	FAM207A	0.40607164	3.54707997	3.765380	0.0002223352	0.003811728	0.2423206	TEX34	0.079573788	0.220727813	3.212128	0.001550428	0.01581961	−1.532495	AK123474	−0.36606115	0.90127882	−2.834072	0.005098689	0.03703003	−2.600211
496	PSMA1	0.32105812	5.69319047	4.917317	1.908205e−06	1.017487e−04	4.683579	GPR87	0.53228646	0.37224499	3.764180	0.0002233299	0.003826309	0.2382064	TNFRSF11B	−0.816619905	1.699033605	−3.211684	0.001552702	0.01583672	−1.533820	WDR45L	0.28843097	5.39906468	2.833996	0.005099844	0.03703003	−2.600412
497	TCRBV14S1	0.34834739	0.39117620	4.917207	1.909148e−06	1.017487e−04	4.683114	GPR89B	0.13006990	0.35994168	3.762500	0.0002247289	0.003847793	0.2324514	CTSS	0.548717876	5.476831168	3.210103	0.001560815	0.01591335	−1.538534	SELS	−0.27895680	4.86198560	−2.833713	0.005104176	0.03704189	−2.601166
498	IGSF8	−0.66860088	4.38601012	−4.917190	1.909298e−06	1.017487e−04	4.683039	RRM2	0.71520687	4.47712646	3.762014	0.0002251350	0.003852262	0.2307873	AK131521	−0.790047741	1.209989123	−3.209272	0.001565097	0.01595088	−1.541012	LINC00086	−0.17803145	0.38903500	−2.833707	0.005104269	0.03704189	−2.601183
499	ISY1−RAB43	0.31312069	2.59544217	4.915296	1.925716e−06	1.024180e−04	4.674974	PAK6	−0.59815309	1.79555231	−3.761809	0.0002253068	0.003852718	0.2300842	PIK3CB	0.292554236	3.456396825	3.209128	0.001565837	0.01595230	−1.541440	GCNT3	−1.11816677	4.56029651	−2.833329	0.005110056	0.03707375	−2.602189
500	TFEC	0.48923054	0.86814251	4.908926	1.981964e−06	1.050704e−04	4.647854	LINC00085	−0.31981617	0.72897590	−3.760861	0.0002261025	0.003863835	0.2268354	DIABLO	0.225148353	4.106058534	3.208950	0.001566757	0.01595270	−1.541971	LOC100287765	0.12611751	0.54902707	2.833111	0.005113406	0.03708792	−2.602771

	Gene	logFC	AveExpr	t	P.Value	adj.P.Val	B	Gene	logFC	AveExpr	t	P.Value	adj.P.Val	B	Gene	logFC	AveExpr	t	P.Value	adj.P.Val	B	Gene	logFC	AveExpr	t	P.Value	adj.P.Val	B
501	MTF2	0.32834992	2.429709416	4.908470	1.986045e−06	0.0001050704	4.645917	RBP4	−1.49515426	2.949688739	−3.760555	0.0002263600	0.003865744	0.225786794	OXT	−0.39881060	0.672731108	−3.208850	0.001567271	0.01595270	−1.542268	PSORS1C2	−0.280825939	0.540059142	−2.832848	0.005117427	0.03710255	−2.603469
502	C4orf21	0.31375658	0.790869641	4.908312	1.987464e−06	0.0001050704	4.645244	SLC10A5	0.44660432	0.896747430	3.759662	0.0002271117	0.003876089	0.222731339	SNORA62	0.13814869	0.523815334	3.208772	0.001567679	0.01595270	−1.542503	MIR3688−2	0.028104956	0.010297908	2.832797	0.005118220	0.03710255	−2.603607
503	STAT4	0.39806062	0.772878766	4.896518	2.096112e−06	0.0001105938	4.595114	TRIM21	0.37494557	3.845393096	3.758302	0.0002282623	0.003893222	0.218074676	JA429130	0.03683333	0.020334338	3.208352	0.001569849	0.01596591	−1.543754	UBE2V2	−0.275615555	4.429087052	−2.831682	0.005135355	0.03719845	−2.606575
504	BC038731	0.44823348	0.690167733	4.894387	2.116353e−06	0.0001114402	4.586064	RPA3	0.43193456	3.845917942	3.757619	0.0002288422	0.003900606	0.215736642	ZNF2	0.15821905	1.195973810	3.208288	0.001570181	0.01596591	−1.543946	ULBP1	−0.244027586	0.349973509	−2.831672	0.005135497	0.03719845	−2.606599
505	IL18R1	0.51600350	1.194046063	4.892562	2.133825e−06	0.0001121378	4.578321	NPNT	−0.95109360	3.129263524	−3.756675	0.0002296453	0.003910222	0.212508666	ORAI3	−0.34673873	3.365054616	−3.205988	0.001582118	0.01607578	−1.550795	SLC5A9	−0.364821892	0.610662269	−2.831662	0.005135654	0.03719845	−2.606626
506	KCNK15	−1.22639597	1.475063116	−4.891414	2.144899e−06	0.0001124970	4.573446	SLC39A13	−0.28903332	3.571701204	−3.756610	0.0002297010	0.003910222	0.212284845	DQ580140	0.04274127	0.097417959	3.205973	0.001582198	0.01607578	−1.550841	DQ576756	0.275940438	3.637811603	2.831324	0.005140862	0.03722601	−2.607526
507	AK096230	0.31227026	0.607335729	4.890450	2.154235e−06	0.0001127638	4.569356	DIP2A	0.23926007	2.076716351	3.754469	0.0002315342	0.003938902	0.204960424	LOC729421	−0.31928028	1.318956889	−3.205699	0.001583626	0.01608414	−1.551657	ATP10D	0.258505767	1.851870753	2.830691	0.005150615	0.03727089	−2.609210
508	RPRD1A	−0.53597460	3.526618598	−4.887219	2.185814e−06	0.0001141916	4.555652	AX747402	0.01427399	0.004936887	3.754135	0.0002318208	0.003941250	0.203820766	RAVER2	0.38537613	3.201530692	3.204902	0.001587784	0.01612020	−1.554028	MLF1IP	0.416515329	2.878333680	2.830634	0.005151498	0.03727089	−2.609362
509	GABPB1	0.28754071	2.793250200	4.886460	2.193290e−06	0.0001142768	4.552437	TUBA1B	0.36236758	7.702869979	3.753480	0.0002323855	0.003948146	0.201579012	CAV2	−0.70566461	3.757715920	−3.204532	0.001589719	0.01613050	−1.555129	JAZF1−AS1	0.126919828	0.483642711	2.830573	0.005152450	0.03727089	−2.609526
510	SNORA11C	0.09535715	0.031476572	4.886180	2.196057e−06	0.0001142768	4.551250	GIMAP1−GIMAP5	0.57442323	2.119479896	3.753194	0.0002326323	0.003948146	0.200600968	CDC7	0.40458894	1.864366982	3.204476	0.001590014	0.01613050	−1.555297	AK097472	0.258470957	5.652567206	2.830558	0.005152677	0.03727089	−2.609565
511	C11orf93	1.08918151	2.745493866	4.885257	2.205205e−06	0.0001145283	4.547335	TEX10	0.33151940	2.726937416	3.753147	0.0002326727	0.003948146	0.200441061	SNORA6	0.29033127	1.387026692	3.204163	0.001591656	0.01614098	−1.556231	CDCA5	0.490954824	3.324616598	2.830466	0.005154095	0.03727098	−2.609809
512	LSM12	0.37444045	2.343073985	4.883978	2.217942e−06	0.0001149648	4.541912	SHPK	0.35942485	2.862637935	3.752551	0.0002331878	0.003954358	0.198403700	TTC9C	0.21252319	3.347938288	3.203715	0.001594003	0.01615861	−1.557563	GPX6	0.016305585	0.003865797	2.830106	0.005159650	0.03730099	−2.610766
513	TRIM10	0.41964950	0.665254451	4.883216	2.225553e−06	0.0001151344	4.538687	GU228584	−0.84484227	3.588820380	−3.750731	0.0002347671	0.003977637	0.192184660	GSTK1	0.43688524	6.816340881	3.203536	0.001594940	0.01616194	−1.558095	OR14A16	0.007963293	0.002640518	2.829839	0.005163781	0.03732069	−2.611476
514	KDM2B	0.37637280	2.864475059	4.880920	2.248670e−06	0.0001161040	4.528956	DL476358	0.73562050	4.745456463	3.750624	0.0002348603	0.003977637	0.191819135	NAPA	0.24669291	5.484451871	3.202673	0.001599479	0.01620175	−1.560664	CSN1S2AP	0.016951122	0.005057426	2.829628	0.005167044	0.03733411	−2.612037
515	SAMHD1	0.81779862	5.026918507	4.879401	2.264084e−06	0.0001166729	4.522524	FOXD1	−0.88945689	1.508446542	−3.750221	0.0002352118	0.003980710	0.190441509	PLA2G4F	−0.65370674	1.504261340	−3.202045	0.001602784	0.01622903	−1.562531	FAM219A	−0.269286558	2.755588591	−2.829462	0.005169625	0.03734259	−2.612481
516	CCDC149	−0.38899039	1.291778314	−4.878771	2.270515e−06	0.0001167775	4.519853	RPL24	0.30007365	8.934244346	3.750072	0.0002353417	0.003980710	0.189932605	CCDC96	−0.18508760	0.706781280	−3.201713	0.001604536	0.01624058	−1.563519	SNORA18	0.322512222	2.519628086	2.829176	0.005174047	0.03736436	−2.613240
517	MIR3918	0.47148872	1.373165748	4.876979	2.288883e−06	0.0001174945	4.512267	MARK2P9	0.07157804	0.104747850	3.749670	0.0002356929	0.003982081	0.188558737	SLC30A4	−0.27135466	1.206979499	−3.201320	0.001606610	0.01625088	−1.564687	RARG	0.408524150	3.464723170	2.829005	0.005176695	0.03736606	−2.613694
518	MEI1	0.40425993	0.689368296	4.874473	2.314812e−06	0.0001185962	4.501661	PSMB9	0.78825299	5.403753421	3.749439	0.0002358945	0.003982081	0.187771088	OBSCN	−0.26127020	0.738966076	−3.201275	0.001606852	0.01625088	−1.564823	B4GALNT4	−0.632496383	1.037416890	−2.828979	0.005177098	0.03736606	−2.613763
519	GAB3	0.40318589	1.004405916	4.870001	2.361799e−06	0.0001207703	4.482742	AKAP5	0.28461170	0.726185628	3.749362	0.0002359620	0.003982081	0.187507837	TM4SF4	−1.32199499	2.443904349	−3.201100	0.001607776	0.01625088	−1.565342	TBC1D3P2	0.006677316	0.002690114	2.828176	0.005189564	0.03744585	−2.615899
520	RASGRP1	0.74224647	1.466406715	4.865425	2.410830e−06	0.0001230404	4.463398	TCRBV2S1	0.77446009	1.972859056	3.749293	0.0002360230	0.003982081	0.187269696	AB231784	0.03341165	0.005790024	3.201057	0.001608004	0.01625088	−1.565471	AK057451	0.022413407	0.038271104	2.828022	0.005191956	0.03745293	−2.616308
521	FADS3	−0.61885192	3.279536411	−4.862465	2.443075e−06	0.0001244468	4.450891	ANPEP	−2.03605212	4.987597813	−3.747335	0.0002377417	0.004008531	0.180585343	MCM4	0.55877842	4.518652960	3.200875	0.001608966	0.01625442	−1.566012	ANTXR2	−0.563422969	3.192849769	−2.827889	0.005194033	0.03745773	−2.616663
522	NCR1	0.30236401	0.376697546	4.860637	2.463184e−06	0.0001252307	4.443174	TRBV30	0.47943296	0.781147262	3.746272	0.0002386806	0.004021807	0.176954423	LOC339593	0.01503622	0.007313754	3.200651	0.001610153	0.01626022	−1.566678	FRG1	0.242593870	4.012325469	2.827699	0.005196983	0.03746657	−2.617167
523	FBXO2	−1.31740360	2.137713555	−4.859914	2.471188e−06	0.0001253632	4.440120	PSMB6	0.27789551	6.299867311	3.745829	0.0002390721	0.004025848	0.175444669	TAF15	0.24437268	5.265410745	3.199844	0.001614430	0.01629721	−1.569077	TMEM126A	0.240315878	4.208574926	2.827628	0.005198082	0.03746657	−2.617355
524	GPR162	−0.65068466	1.993267972	−4.859549	2.475237e−06	0.0001253632	4.438579	ZMYND8	0.45558789	3.190255200	3.745205	0.0002396258	0.004031025	0.173314057	COPA	−0.26144302	5.767589422	−3.199714	0.001615118	0.01629796	−1.569462	AK124066	0.183608042	0.287243462	2.827532	0.005199581	0.03746719	−2.617611
525	TMEM101	0.38126705	3.668928081	4.857870	2.493945e−06	0.0001258612	4.431491	C11orf65	0.12328064	0.414452461	3.745140	0.0002396834	0.004031025	0.173092462	MIR124−3	0.03468401	0.014893134	3.199500	0.001616256	0.01630323	−1.570099	LOC100129858	0.057280660	0.162874264	2.827220	0.005204445	0.03748950	−2.618441
526	LEPRE1	−0.49462968	2.896402293	−4.857815	2.494555e−06	0.0001258612	4.431261	EGFR	−0.77940768	2.974268747	−3.744639	0.0002401286	0.004035954	0.171383190	BC023201	0.17628330	0.740569941	3.199096	0.001618406	0.01631872	−1.571301	PFKFB1	0.085902244	0.175032688	2.827103	0.005206268	0.03748950	−2.618752
527	BC007984	0.07249337	0.063254900	4.856275	2.511848e−06	0.0001262930	4.424758	FAM21A	0.25475710	1.156109510	3.740491	0.0002438450	0.004093824	0.157237031	TNFSF8	0.36407148	0.890789802	3.198436	0.001621920	0.01633550	−1.573262	C6	0.369753586	0.512520505	2.827061	0.005206915	0.03748950	−2.618862
528	MLLT3	0.55356450	2.245842302	4.856179	2.512926e−06	0.0001262930	4.424355	RABGAP1L	0.39130520	2.559406799	3.740452	0.0002438802	0.004093824	0.157103944	STYX	−0.21830271	2.659236995	−3.198337	0.001622446	0.01633550	−1.573555	CCDC28B	0.310119840	2.253764002	2.826603	0.005214065	0.03752808	−2.620080
529	OR51I2	0.02296141	0.005903689	4.855668	2.518688e−06	0.0001262930	4.422198	HOXC5	0.56384249	1.186797424	3.738981	0.0002452113	0.004113566	0.152090806	YPEL3	−0.41860392	4.129700735	−3.198331	0.001622478	0.01633550	−1.573573	CD27	0.684724065	3.154906469	2.826486	0.005215881	0.03752808	−2.620390
530	H2AFX	0.67218377	5.743301883	4.855362	2.522149e−06	0.0001262930	4.420906	RAB15	−0.68459258	3.325304749	−3.737290	0.0002467502	0.004135207	0.146328924	SYT1	−0.60130995	0.856291151	−3.198321	0.001622532	0.01633550	−1.573603	STARD6	0.019010092	0.015956393	2.826446	0.005216515	0.03752808	−2.620497
531	RQCD1	0.43214802	4.026597941	4.853589	2.542268e−06	0.0001270607	4.413428	ZBP1	0.54838357	1.552139210	3.737091	0.0002469323	0.004135207	0.145649396	TRAF5	−0.44645309	2.763171351	−3.198166	0.001623362	0.01633765	−1.574065	XPC	0.202264232	3.456438789	2.825877	0.005225403	0.03758184	−2.622009
532	PTPN22	0.70503790	1.520493133	4.852462	2.555138e−06	0.0001272528	4.408675	PCGF6	0.30242610	1.936937393	3.737051	0.0002469687	0.004135207	0.145513592	GBA	−0.30774466	3.889445004	−3.197351	0.001627713	0.01637523	−1.576485	GTF3C6	0.267483006	5.189167005	2.825699	0.005228186	0.03758765	−2.622481
533	ProSAPiP1	−1.06415403	3.026974550	−4.852191	2.558242e−06	0.0001272528	4.407532	SNORA52	0.31605495	0.939373922	3.736642	0.0002473424	0.004138852	0.144121390	SGCE	−0.62050466	2.778695903	−3.195730	0.001636406	0.01644130	−1.581300	KRT27	0.203931930	0.072636162	2.825644	0.005229044	0.03758765	−2.622627
534	CCDC129	0.29622923	0.168739707	4.851994	2.560496e−06	0.0001272528	4.406703	TMEM176A	−0.98818010	6.796178553	−3.735986	0.0002479432	0.004146292	0.141886995	LRRC37BP1	0.20427424	1.396169459	3.195691	0.001636615	0.01644130	−1.581416	RNF151	0.036056357	0.038802080	2.825492	0.005231430	0.03759462	−2.623032
535	EIF4E	0.37706767	2.239795602	4.848265	2.603620e−06	0.0001291541	4.390983	HSPA1L	−0.79547630	3.651367859	−3.734861	0.0002489768	0.004160954	0.138056151	DAG1	0.41426629	5.480937818	3.195689	0.001636623	0.01644130	−1.581420	TRMT1L	−0.183099350	2.581032794	−2.825398	0.005232896	0.03759497	−2.623281
536	TRPM2	−0.77232472	1.724525964	−4.845478	2.636316e−06	0.0001305321	4.379238	GEMIN7	0.36547259	3.298260281	3.734337	0.0002494593	0.004166394	0.136273096	TTC5	0.21147749	2.451507672	3.195643	0.001636875	0.01644130	−1.581559	MPEG1	0.620769762	3.368352141	2.825180	0.005236313	0.03760934	−2.623860
537	RHOF	−0.96966395	4.200447248	−4.844838	2.643873e−06	0.0001306625	4.376544	FLJ31813	0.11938510	0.235253819	3.733891	0.0002498708	0.004170642	0.134755482	DDX26B	0.33909125	1.372696236	3.195549	0.001637377	0.01644130	−1.581836	IGSF9B	0.369067066	0.912684029	2.824963	0.005239706	0.03762353	−2.624435
538	LACTB	0.41055151	3.118987736	4.843543	2.659238e−06	0.0001311775	4.371090	CR627240	0.01721506	0.010278108	3.733502	0.0002502307	0.004174024	0.133429986	BC031243	0.02572463	0.036944547	3.194884	0.001640960	0.01647105	−1.583812	DAPK3	−0.267177115	4.059292318	−2.824319	0.005249810	0.03768588	−2.626145
539	TRIM22	1.00629162	3.836542863	4.840818	2.691862e−06	0.0001324167	4.359615	LOC100505746	0.43913830	0.669817628	3.732688	0.0002509846	0.004181490	0.130659644	FAM131C	−0.39090333	0.571148452	−3.194725	0.001641819	0.01647221	−1.584285	CD7	0.633056039	3.174971544	2.823631	0.005260624	0.03775330	−2.627972
540	PLCXD2	0.32208206	0.746466622	4.840612	2.694338e−06	0.0001324167	4.358749	PATL2	0.26308217	0.589054690	3.732678	0.0002509934	0.004181490	0.130627482	SLC25A34	−0.29467884	0.679808593	−3.194632	0.001642317	0.01647221	−1.584559	AJUBA	0.459199397	2.561609762	2.823404	0.005264194	0.03775976	−2.628574
541	ZNF91	−0.53717075	2.495972031	−4.839746	2.704789e−06	0.0001326847	4.355106	RB1	0.39446562	4.210345841	3.731916	0.0002517011	0.004190650	0.128034603	ATP6V0E1	−0.26155774	7.219105437	−3.194345	0.001643864	0.01648149	−1.585411	STARD4	−0.400349277	1.711867405	−2.823393	0.005264370	0.03775976	−2.628604
542	ADAM28	1.19168382	2.768243139	4.832608	2.792484e−06	0.0001364739	4.325080	RGS10	0.52328067	4.425332479	3.731463	0.0002521229	0.004195041	0.126492883	AX747742	−0.32130504	2.481447548	−3.194044	0.001645491	0.01649157	−1.586305	SART3	0.163581406	2.901684965	2.823010	0.005270401	0.03779281	−2.629620
543	TTC14	−0.56490168	2.067987294	−4.832315	2.796144e−06	0.0001364739	4.323848	IFITM2	−0.68631541	7.518084716	−3.731053	0.0002525050	0.004198765	0.125098637	IQCF6	0.06859986	0.054337850	3.193728	0.001647199	0.01649889	−1.587242	ERCC4	0.133207047	1.252737917	2.822393	0.005280130	0.03785234	−2.631257
544	SH3PXD2A	−0.64427011	3.301401575	−4.832209	2.797460e−06	0.0001364739	4.323405	FAM78A	0.52680351	1.817530969	3.730103	0.0002533927	0.004208889	0.121867192	U2AF1	0.26586272	3.369172516	3.193670	0.001647513	0.01649889	−1.587414	ALPI	−0.730464057	1.030632676	−2.822237	0.005282598	0.03785980	−2.631672
545	SPTLC3	0.71528730	1.335872401	4.830480	2.819155e−06	0.0001372799	4.316136	MLH1	0.44766230	3.001935337	3.730062	0.0002534310	0.004208889	0.121727995	RBX1	0.27514980	5.254129052	3.193501	0.001648428	0.01649889	−1.587916	SRPR	0.227782542	5.997177411	2.821821	0.005289177	0.03789673	−2.632776
546	SCAND3	0.28318752	0.196785369	4.829215	2.835119e−06	0.0001377985	4.310822	LOC440354	0.07346525	0.268271515	3.729147	0.0002542887	0.004219740	0.118616935	HSPA12A	−0.37893336	1.056563690	−3.193449	0.001648708	0.01649889	−1.588070	AS3MT	−0.351196783	1.874046167	−2.821061	0.005301202	0.03797263	−2.634792
547	NR1I2	−1.06737141	1.443122195	−4.828815	2.840190e−06	0.0001377985	4.309141	AK093519	0.07450436	0.078725100	3.729026	0.0002544024	0.004219740	0.118205619	43344	0.61606756	2.311756017	3.192819	0.001652123	0.01652684	−1.589941	UTP20	0.233072256	2.188303503	2.820332	0.005312760	0.03804515	−2.636725
548	AK056105	0.18851983	0.045685814	4.826942	2.864036e−06	0.0001387019	4.301274	ANKMY2	0.42502800	3.334178505	3.728107	0.0002552675	0.004231446	0.115079796	LRRC25	0.43125364	2.159642051	3.192409	0.001654346	0.01654284	−1.591156	NR4A1	−0.713022647	4.867749473	−2.820071	0.005316906	0.03806457	−2.637417
549	LINC00161	0.09071379	0.061287883	4.824209	2.899190e−06	0.0001401486	4.289795	DQ572814	0.03937300	0.007296653	3.727290	0.0002560388	0.004239252	0.112302038	MAGI2	−0.22206333	0.601273843	−3.192161	0.001655694	0.01655008	−1.591892	TMX4	−0.355808873	4.220181871	−2.819251	0.005329945	0.03814763	−2.639590
550	PCNA−AS1	0.81250829	6.681378667	4.822444	2.922102e−06	0.0001409994	4.282389	TOR1AIP2	−0.27153696	2.750995675	−3.727189	0.0002561334	0.004239252	0.111962026	TUB	−0.28554217	0.595900838	−3.191623	0.001658619	0.01657308	−1.593487	TMEM48	0.348861386	3.499614385	2.819146	0.005331612	0.03814927	−2.639868
551	LOC100507463	0.85930554	5.060346093	4.821698	2.931838e−06	0.0001410433	4.279260	IL10	0.26396206	0.540720404	3.727100	0.0002562177	0.004239252	0.111659132	VAMP4	−0.20681375	2.064422752	−3.191490	0.001659345	0.01657409	−1.593883	CDK5RAP1	0.230765185	2.610538223	2.819007	0.005333827	0.03815484	−2.640237
552	SLC24A4	0.05334200	0.081867354	4.821561	2.933640e−06	0.0001410433	4.278681	BATF	0.68378568	3.235487003	3.726857	0.0002564481	0.004240421	0.110831603	ARVCF	−0.37816900	1.944333162	−3.191305	0.001660351	0.01657790	−1.594430	TREM2	0.613806642	3.062191549	2.818145	0.005347589	0.03824297	−2.642523
553	KHDRBS3	−0.81323643	2.138304125	−4.819451	2.961374e−06	0.0001420042	4.269829	LRRC58	−0.30102098	3.460265922	−3.725653	0.0002575895	0.004255983	0.106742781	TGFBI	−0.72617605	5.865792001	−3.188448	0.001675984	0.01672770	−1.602900	POM121L12	0.006557119	0.001635063	2.817807	0.005352981	0.03827101	−2.643417
554	SPATS2	0.32538448	2.481177029	4.819227	2.964330e−06	0.0001420042	4.268890	C17orf100	−0.34327575	1.420217653	−3.725527	0.0002577100	0.004255983	0.106312435	LOC100287559	0.19266005	0.963475203	3.188141	0.001677672	0.01673800	−1.603809	SASH3	0.632347966	2.908697556	2.817688	0.005354885	0.03827101	−2.643733
555	UBD	1.87588022	6.357675415	4.815883	3.008847e−06	0.0001434623	4.254867	AX746590	0.18570265	0.913283430	3.725055	0.0002581588	0.004260743	0.104710491	PHF15	0.42642164	2.907434143	3.188031	0.001678277	0.01673800	−1.604136	AK096499	0.010365369	0.004870559	2.817568	0.005356806	0.03827101	−2.644051
556	NUP35	0.38619429	3.070553395	4.815874	3.008963e−06	0.0001434623	4.254830	SOBP	−0.57344051	1.357048991	−3.724362	0.0002588195	0.004267359	0.102357194	PCDHB2	−0.46655177	0.937145648	−3.187589	0.001680711	0.01675597	−1.605445	LRBA	0.246331020	2.930753984	2.817538	0.005357278	0.03827101	−2.644129
557	FAIM	−0.43156102	1.668287203	−4.815724	3.010983e−06	0.0001434623	4.254199	TIMP2	−0.71025555	6.278053382	−3.724282	0.0002588965	0.004267359	0.102083287	FUBP3	−0.20397979	3.951770594	−3.187285	0.001682389	0.01676559	−1.606347	ADRA1D	−0.424403557	0.675860285	−2.817339	0.005360467	0.03828349	−2.644657
558	PDCD1LG2	0.60820051	1.249151745	4.815037	3.020211e−06	0.0001436441	4.251320	MATL2963	0.23286190	1.227526328	3.724129	0.0002590420	0.004267359	0.101566236	PRNP	−0.59339983	5.132412438	−3.187120	0.001683298	0.01676559	−1.606835	NBPF15	−0.179308912	0.896580021	−2.816733	0.005370170	0.03834247	−2.646262
559	ASTE1	0.32850128	2.296941910	4.813174	3.045382e−06	0.0001445821	4.243512	TCRDV2	0.38883982	0.748390000	3.723447	0.0002596948	0.004275232	0.099249237	BC035340	−0.14787403	0.165564667	−3.186957	0.001684199	0.01676559	−1.607318	CLGN	−0.383228668	0.561606453	−2.816544	0.005373196	0.03835375	−2.646762
560	C19orf43	−0.39893374	7.027621420	−4.810942	3.075810e−06	0.0001457237	4.234159	CD209	0.65027583	1.783741783	3.723293	0.0002598421	0.004275232	0.098727178	ULBP3	−0.44661613	1.233491386	−3.186908	0.001684468	0.01676559	−1.607463	KIFAP3	−0.278165515	2.507170145	−2.816210	0.005378566	0.03836658	−2.647648
561	ENTPD8	−1.34662430	1.766060311	−4.810607	3.080409e−06	0.0001457237	4.232754	LILRP2	0.04198746	0.047244974	3.722368	0.0002607303	0.004287188	0.095585722	CHIT1	0.63909669	0.729101978	3.186760	0.001685289	0.01676559	−1.607903	LOC100507240	0.026596891	0.049195442	2.816199	0.005378743	0.03836658	−2.647677
562	HES6	−1.04749844	3.111588215	−4.806247	3.140775e−06	0.0001483150	4.214498	NDNL2	−0.30386655	3.600931178	−3.722171	0.0002609202	0.004287654	0.094915354	SNORA40	0.09148091	0.683749583	3.186655	0.001685869	0.01676559	−1.608214	MIR3606	−0.489105553	2.120118941	−2.816147	0.005379568	0.03836658	−2.647813
563	SEPN1	−0.48278860	5.795484869	−4.803080	3.185340e−06	0.0001501523	4.201245	CD84	0.59059279	1.720394529	3.721700	0.0002613731	0.004292439	0.093318795	MPP6	−0.48605917	1.445246227	−3.186613	0.001686098	0.01676559	−1.608337	MYH3	0.089196987	0.253032384	2.816072	0.005380776	0.03836658	−2.648013
564	PRR22	0.48170496	0.935509814	4.802193	3.197927e−06	0.0001503516	4.197536	GSTO1	0.35577662	5.650135723	3.721291	0.0002617679	0.004296264	0.091929406	JUND	−0.36887043	7.302119857	−3.186269	0.001688001	0.01677823	−1.609356	DUS3L	0.258305594	3.473945465	2.815590	0.005388520	0.03839637	−2.649288
565	APOBEC3F	0.64421442	1.893823167	4.801829	3.203108e−06	0.0001503516	4.196013	SNORA71D	0.20155186	0.351398212	3.719621	0.0002633845	0.004320124	0.086262213	MIR4695	0.27917354	0.843500654	3.184168	0.001699659	0.01688778	−1.615575	ITGA7	−0.681535331	2.589305475	−2.815433	0.005391046	0.03839637	−2.649704
566	LOC388588	−0.65986330	1.314034199	−4.801587	3.206564e−06	0.0001503516	4.194999	LILRB1	0.51557165	1.653419502	3.718975	0.0002640119	0.004327740	0.084072222	RPL28	0.37058717	8.053152475	3.183580	0.001702938	0.01691377	−1.617316	NPC2	0.293428844	7.271513727	2.815413	0.005391367	0.03839637	−2.649757
567	IL27	0.16661411	0.221473572	4.799916	3.230474e−06	0.0001512056	4.188011	LTK	−0.71976008	1.182019838	−3.717979	0.0002649830	0.004340978	0.080692629	TAPBP	0.34207937	6.557543286	3.183429	0.001703779	0.01691377	−1.617763	CTBP2	−0.276763887	4.120326662	−2.815406	0.005391475	0.03839637	−2.649775
568	BUD13	0.31169239	3.099370109	4.797712	3.262276e−06	0.0001523382	4.178797	DQ571461	0.05595173	0.114500450	3.717007	0.0002659333	0.004351364	0.077397471	PDGFRL	−0.56713694	1.524226833	−3.183356	0.001704187	0.01691377	−1.617979	TROVE2	−0.192806356	2.774741554	−2.815362	0.005392188	0.03839637	−2.649892
569	RRM2B	0.52011672	2.970707573	4.797445	3.266154e−06	0.0001523382	4.177680	TNIP3	0.33196644	0.513269347	3.716995	0.0002659449	0.004351364	0.077357450	BEX5	−0.63473441	1.438062147	−3.182958	0.001706414	0.01692954	−1.619159	DDX12P	0.201923677	0.713762425	2.815270	0.005393670	0.03839662	−2.650136
570	RGS18	0.45019641	0.959277927	4.795601	3.293026e−06	0.0001532176	4.169974	SNRPC	0.33075136	5.533832671	3.714390	0.0002685089	0.004388878	0.068526221	COL9A3	−1.03099524	1.515775357	−3.182447	0.001709272	0.01694818	−1.620670	ZNF577	−0.316576816	0.922607947	−2.814914	0.005399398	0.03842626	−2.651078
571	NHLRC1	−0.62110820	1.204450870	−4.795360	3.296555e−06	0.0001532176	4.168966	CYTIP	0.77809343	2.658256408	3.714329	0.0002685684	0.004388878	0.068322281	FAM169A	0.41433562	1.075798361	3.182393	0.001709570	0.01694818	−1.620828	LRRC43	−0.165934530	0.294240826	−2.814832	0.005400730	0.03842626	−2.651296
572	ANKH	−0.73010372	3.399088254	−4.794623	3.307365e−06	0.0001532442	4.165887	SHC2	−0.67625780	2.625315908	−3.713171	0.0002697161	0.004404921	0.064397768	SNORD65	0.21347213	0.865584965	3.182180	0.001710764	0.01694928	−1.621459	AP1S1	0.396834638	5.194024447	2.814608	0.005404343	0.03844166	−2.651890
573	TAP1	0.98479224	6.178111126	4.794244	3.312943e−06	0.0001532442	4.164302	U4	0.03042115	0.085733418	3.712334	0.0002705482	0.004413201	0.061562921	SRRM1	0.22301973	4.831842194	3.182145	0.001710959	0.01694928	−1.621562	KLHDC5	−0.374388685	2.886260843	−2.814482	0.005406365	0.03844574	−2.652222
574	AEN	0.59677308	3.298578487	4.794142	3.314449e−06	0.0001532442	4.163875	ZNF277	0.25840526	2.397045763	3.712326	0.0002705557	0.004413201	0.061537272	AB062083	0.09151428	0.457780720	3.181999	0.001711780	0.01695109	−1.621995	PABPC4	−0.293481036	5.773364899	−2.813364	0.005424432	0.03856334	−2.655181
575	LONRF3	−0.66980696	1.386911474	−4.792310	3.341527e−06	0.0001542275	4.156223	DQ582047	0.02081364	0.001917523	3.712139	0.0002707429	0.004413541	0.060900989	WDR90	0.35825902	2.273696308	3.181800	0.001712893	0.01695579	−1.622583	CCDC90A	−0.346142230	3.122551145	−2.813279	0.005425808	0.03856334	−2.655406
576	AK126351	0.24837021	0.744848541	4.791566	3.352583e−06	0.0001544691	4.153116	DTX2P1−UPK3BP1−PMS2P11	0.28214014	1.247664037	3.711729	0.0002711512	0.004417485	0.059513867	NACAD	−0.38138236	0.840915317	−3.180908	0.001717901	0.01699408	−1.625220	AK055370	−0.265816145	0.763788361	−2.812848	0.005432786	0.03860260	−2.656546
577	PINK1	−0.48782556	4.535273072	−4.789616	3.381727e−06	0.0001555419	4.144976	PIGW	0.38780010	2.336257903	3.711210	0.0002716698	0.004423218	0.057755470	JA429672	0.09342548	0.060468116	3.180840	0.001718288	0.01699408	−1.625423	TMEM140	0.236477988	3.981206973	2.811968	0.005447056	0.03869364	−2.658873
578	SPATA6L	0.34300840	0.527123582	4.788679	3.395832e−06	0.0001559204	4.141062	GAR1	0.34565917	3.425477148	3.708865	0.0002740233	0.004458801	0.049817681	PTK2B	0.37648876	3.309133859	3.180769	0.001718682	0.01699408	−1.625631	MGST2	0.367460655	5.165205551	2.811374	0.005456705	0.03875181	−2.660443
579	GPR141	0.40946676	0.583314037	4.782985	3.482715e−06	0.0001596335	4.117305	NT5C3L	−0.64245205	3.925345045	−3.707356	0.0002755479	0.004474186	0.044712300	GALNT11	−0.23078673	2.196041806	−3.180111	0.001722391	0.01702441	−1.627578	CDHR5	−0.909901187	4.043929653	−2.810853	0.005465188	0.03880167	−2.661821
580	SYTL3	0.40292116	1.336973101	4.779791	3.532374e−06	0.0001616305	4.103992	TRAV20	0.36503855	0.502789304	3.707209	0.0002756969	0.004474186	0.044214901	BAAT	0.71222991	0.839740363	3.179958	0.001723252	0.01702658	−1.628030	LCE1A	0.052694565	0.010997144	2.810583	0.005469590	0.03882253	−2.662535
581	ZNF718	−0.47917546	1.302076514	−4.779036	3.544229e−06	0.0001618938	4.100841	PLAC1	0.43569079	0.391130730	3.707180	0.0002757257	0.004474186	0.044118623	THEG5	0.03471827	0.004174422	3.179202	0.001727520	0.01706240	−1.630264	ZMAT3	0.308561802	2.567129684	2.810449	0.005471774	0.03882416	−2.662889
582	KLRC2	0.43777049	0.493140201	4.777648	3.566095e−06	0.0001626127	4.095058	PDCD1	0.61153220	1.351530956	3.707133	0.0002757732	0.004474186	0.043960160	GMFG	0.56293133	3.247760108	3.178805	0.001729768	0.01707824	−1.631439	EMR2	0.355743909	1.356868709	2.810337	0.005473600	0.03882416	−2.663185
583	SIRPD	0.19840185	0.189165050	4.772629	3.646264e−06	0.0001657847	4.074154	WDR76	0.48214387	2.009349359	3.706991	0.0002759179	0.004474186	0.043477365	BPIFB1	1.81327977	3.103598099	3.177495	0.001737197	0.01714521	−1.635310	MIR4788	0.029926719	0.009345582	2.810299	0.005474209	0.03882416	−2.663284
584	SLAMF8	0.91226192	2.893543091	4.772387	3.650170e−06	0.0001657847	4.073148	GNAI1	−0.62106988	2.362292731	−3.706854	0.0002760566	0.004474186	0.043015134	SIX2	−0.91386221	1.898505231	−3.177300	0.001738302	0.01714973	−1.635884	RFT1	0.208678921	2.346953588	2.810184	0.005476097	0.03882718	−2.663590
585	CPOX	0.49407547	3.162413784	4.772126	3.654398e−06	0.0001657847	4.072059	CDC45	0.61390994	2.661719295	3.706640	0.0002762742	0.004474186	0.042289969	LETMD1	0.26717192	3.745275206	3.176992	0.001740054	0.01716064	−1.636794	ZNF274	−0.274368840	2.230812392	−2.809823	0.005481991	0.03885859	−2.664544
586	SHF	−0.54602615	1.281348097	−4.771506	3.664434e−06	0.0001659563	4.069481	FAR1	0.43938851	3.991882206	3.706565	0.0002763505	0.004474186	0.042035939	LIPE	−0.42179539	1.672855856	−3.176337	0.001743786	0.01719106	−1.638729	SMAGP	0.458474385	4.900476569	2.808977	0.005495816	0.03894342	−2.666778
587	CFL1P1	0.09822741	0.128827294	4.766022	3.754458e−06	0.0001694982	4.046663	OR10A2	0.06103631	0.054674940	3.706305	0.0002766140	0.004474186	0.041158987	LOC100132707	0.25640511	2.435384081	3.175760	0.001747077	0.01721425	−1.640433	HINFP	0.195467593	2.140775331	2.808911	0.005496893	0.03894342	−2.666952
588	CCDC171	0.20610428	0.388217363	4.765965	3.755414e−06	0.0001694982	4.046424	LOC727896	0.16709612	0.528444125	3.706265	0.0002766547	0.004474186	0.041023443	LOC153910	0.08341058	0.022532862	3.175697	0.001747435	0.01721425	−1.640618	IFNA21	0.033584014	0.012892264	2.808066	0.005510754	0.03903120	−2.669185
589	AF258550	0.32587311	0.810925761	4.764346	3.782391e−06	0.0001704259	4.039694	BIN2	0.59015647	2.211579179	3.705378	0.0002775583	0.004486066	0.038023140	ARHGAP30	0.54060235	2.651658684	3.175029	0.001751256	0.01722868	−1.642590	SPINK5	0.745212693	1.384685435	2.807728	0.005516304	0.03906008	−2.670078
590	HNRNPD	0.36081738	6.225440770	4.761935	3.822940e−06	0.0001719610	4.029670	ST6GALNAC2	−0.69697190	1.893828047	−3.704059	0.0002789068	0.004505117	0.033563752	VSTM2L	−0.88776697	1.559442422	−3.175018	0.001751318	0.01722868	−1.642623	DLEU2L	0.051329290	0.156040486	2.807157	0.005525694	0.03911463	−2.671586
591	PYHIN1	0.67791124	1.040728843	4.761009	3.838623e−06	0.0001723743	4.025821	BATF2	0.84136889	3.024400246	3.703744	0.0002792296	0.004507588	0.032499452	GRIP1	−0.24402706	0.543118743	−3.174968	0.001751609	0.01722868	−1.642773	KRT13	0.841433449	0.864672535	2.807080	0.005526955	0.03911463	−2.671788
592	ZNF226	0.31285438	1.437603172	4.759285	3.867976e−06	0.0001733990	4.018660	BC043511	0.37766259	0.933332426	3.702066	0.0002809562	0.004532702	0.026827956	BC030768	−0.30561133	0.410687944	−3.174918	0.001751895	0.01722868	−1.642920	PRDM12	0.082370571	0.138401774	2.806843	0.005530851	0.03913177	−2.672413
593	MMP25	0.53937320	1.352940710	4.757774	3.893882e−06	0.0001740262	4.012385	HSD17B11	−0.51503329	5.229121112	−3.700483	0.0002825933	0.004554853	0.021482460	PDF	0.27876328	3.784395550	3.174874	0.001752146	0.01722868	−1.643049	LOC729176	0.095316164	0.336470429	2.806522	0.005536152	0.03914968	−2.673263
594	ALG9	0.30400596	2.172468385	4.757165	3.904373e−06	0.0001740262	4.009856	SNX19	−0.51168349	3.641259128	−3.700407	0.0002826724	0.004554853	0.021225147	SPICE1	0.19789965	1.289770990	3.174391	0.001754916	0.01724355	−1.644476	ATP5L	0.253193728	6.607492658	2.806511	0.005536333	0.03914968	−2.673292
595	SNORD43	0.31769589	0.463130236	4.757071	3.905996e−06	0.0001740262	4.009466	TMPRSS12	0.02442736	0.018202553	3.699925	0.0002831729	0.004560149	0.019597594	DNAH3	−0.10170872	0.190076888	−3.174383	0.001754958	0.01724355	−1.644498	C1orf133	−0.557791931	1.748026515	−2.805876	0.005546795	0.03921321	−2.674966
596	VEZT	0.34902643	3.236313224	4.756944	3.908196e−06	0.0001740262	4.008936	TRBV2	0.49758893	0.955951892	3.698706	0.0002844435	0.004577832	0.015479001	KLK10	−1.41954384	2.674481056	−3.172688	0.001764705	0.01732529	−1.649500	CAPNS1	0.225590793	7.582202326	2.805560	0.005552012	0.03923964	−2.675799
597	RPS6KB2	0.42752039	4.879129213	4.756212	3.920854e−06	0.0001742974	4.005897	APOBEC3D	0.50406965	1.725219578	3.697718	0.0002854763	0.004591670	0.012144716	ESCO2	0.46020044	1.787896764	3.172676	0.001764775	0.01732529	−1.649536	OR8U8	0.006261144	0.001346111	2.805440	0.005554000	0.03923991	−2.676117
598	C12orf26	0.27349659	1.442347953	4.755513	3.932968e−06	0.0001745435	4.002997	TFAP2C	−0.73018407	1.038784298	−3.697090	0.0002861352	0.004599481	0.010023862	CCNO	0.55214910	2.043668196	3.172596	0.001765236	0.01732529	−1.649771	MAPK8IP2	−0.635494356	1.605840702	−2.805379	0.005555008	0.03923991	−2.676278
599	PRAM1	0.45452107	1.052400508	4.754228	3.955360e−06	0.0001752442	3.997660	SCO2	0.55436297	4.960646756	3.696035	0.0002872447	0.004612703	0.006463879	IL27RA	0.48735639	2.714002796	3.172178	0.001767649	0.01734257	−1.651005	BC032901	−0.185024024	0.368595151	−2.804935	0.005562350	0.03927666	−2.677449
600	PTGS2	−1.12271558	2.128051389	−4.753028	3.976364e−06	0.0001755491	3.992682	AK293020	1.00329793	2.425754638	3.695977	0.0002873054	0.004612703	0.006269485	PTPN7	0.55472577	1.832383509	3.171967	0.001768865	0.01734808	−1.651626	CNTROB	0.189563908	2.684912010	2.804886	0.005563169	0.03927666	−2.677579

	Gene	logFC	AveExpr	t	P.Value	adj.P.Val	B	Gene	logFC	AveExpr	t	P.Value	adj.P.Val	B	Gene	logFC	AveExpr	t	P.Value	adj.P.Val	B	Gene	logFC	AveExpr	t	P.Value	adj.P.Val	B
601	HAT1	0.31403786	3.761002413	4.752761	3.981066e−06	0.0001755491	3.991571	MIR204	0.04233153	0.009861486	3.695035	0.0002882995	0.004623710	0.0030921461	KIAA1614	−0.25389038	0.774494021	−3.171369	0.001772327	0.01737561	−1.653392	SSBP2	−0.477316321	2.4299816956	−2.804173	0.005574972	0.03934639	−2.679459
602	WDFY1	0.40521020	4.073802775	4.752703	3.982085e−06	0.0001755491	3.991331	HDAC3	0.23946003	4.092089212	3.694967	0.0002883716	0.004623710	0.0028623501	C7orf44	0.23089110	3.324268613	3.170908	0.001774996	0.01739067	−1.654751	B9D2	0.271261746	2.4741313226	2.804111	0.005576012	0.03934639	−2.679624
603	PIK3R6	0.31292684	0.821516901	4.750196	4.026393e−06	0.0001772080	3.980930	CCNG1	0.50485677	4.484481660	3.694833	0.0002885136	0.004623710	0.0024092957	MORC4	0.48344050	2.613684578	3.170803	0.001775603	0.01739067	−1.655060	LOC100129534	0.167706645	0.5821889270	2.803221	0.005590792	0.03944020	−2.681971
604	SLC38A9	0.38817457	1.995740123	4.747790	4.069360e−06	0.0001788026	3.970953	LOC100128191	0.44538179	2.889204590	3.694043	0.0002893506	0.004634325	−0.0002552919	KANSL2	0.19690531	2.854262580	3.170764	0.001775829	0.01739067	−1.655174	KRT17	−1.398445449	3.4967208490	−2.803013	0.005594247	0.03945383	−2.682518
605	SNX33	−0.39131233	3.705499251	−4.740392	4.204287e−06	0.0001844257	3.940297	CAPN9	−0.84644842	1.238969738	−3.691025	0.0002925686	0.004683039	−0.0104281076	RALGPS1	0.33178259	2.114075162	3.169772	0.001781591	0.01744067	−1.658100	DIP2B	0.243363948	2.9645148160	2.802926	0.005595697	0.03945383	−2.682748
606	TMEM220	−0.52420780	1.402414693	−4.732693	4.349290e−06	0.0001904716	3.908432	EPSTI1	0.74994063	4.331912440	3.690519	0.0002931115	0.004688900	−0.0121330668	MAPK3	−0.33491573	5.576501130	−3.168892	0.001786717	0.01748439	−1.660694	BC042022	0.037069767	0.0512830128	2.802645	0.005600380	0.03947636	−2.683490
607	BTN2A2	0.43446123	1.477230908	4.730695	4.387700e−06	0.0001918372	3.900170	OPA3	0.23428151	2.111230712	3.689622	0.0002940765	0.004699528	−0.0151561462	ZNF287	−0.17492190	0.653840293	−3.168528	0.001788840	0.01749872	−1.661766	LOC731275	0.133297514	0.6236796294	2.802324	0.005605715	0.03950347	−2.684334
608	GPN3	0.31307425	3.140584602	4.728566	4.428989e−06	0.0001933239	3.891369	C5orf62	−0.55757023	3.179254901	−3.689572	0.0002941300	0.004699528	−0.0153234568	ZNF485	0.26509936	1.441730764	3.168046	0.001791655	0.01751979	−1.663186	RLN1	0.103031499	0.1494092318	2.801864	0.005613401	0.03954713	−2.685549
609	DQ577785	−0.68370534	2.322161703	−4.727049	4.458636e−06	0.0001942984	3.885101	KRBA1	−0.38418181	1.499277406	−3.689028	0.0002947172	0.004706077	−0.0171577382	RASA3	−0.46138564	2.745661714	−3.166626	0.001799974	0.01759466	−1.667368	ERAL1	0.214528961	4.3786758941	2.801624	0.005617409	0.03956487	−2.686181
610	SPTSSA	−0.57169929	4.457948084	−4.722527	4.548165e−06	0.0001978180	3.866421	ACSS1	−0.68424800	3.603993640	−3.688849	0.0002949097	0.004706320	−0.0177584099	LOC145845	0.01132567	0.008975993	3.166489	0.001800780	0.01759606	−1.667773	C6orf225	−0.291231728	1.1871536599	−2.801329	0.005622332	0.03958904	−2.686958
611	PPP2R2B	0.30100431	0.493088709	4.722220	4.554309e−06	0.0001978180	3.865153	MARVELD1	−0.63877410	4.254721287	−3.682739	0.0003015799	0.004808367	−0.0383259840	MPDU1	0.32279094	4.759737195	3.165596	0.001806034	0.01764090	−1.670403	METTL21D	0.179447378	1.9734508856	2.800902	0.005629464	0.03962874	−2.688082
612	SLC4A10	0.15208711	0.088361409	4.720625	4.586347e−06	0.0001988841	3.858567	KISS1	−0.53974976	0.842242606	−3.682660	0.0003016666	0.004808367	−0.0385904011	LIMK2	0.32438581	3.628640323	3.165378	0.001807315	0.01764692	−1.671043	CCDC59	0.235946534	3.7346943915	2.800715	0.005632608	0.03964036	−2.688577
613	ZCCHC8	0.28145679	2.546845448	4.719070	4.617776e−06	0.0001999203	3.852150	SPINK4	−1.98427983	2.915866812	−3.682342	0.0003020183	0.004808923	−0.0396617745	SNORD119	0.19981001	0.420952253	3.165263	0.001807998	0.01764710	−1.671384	PCNP	0.195728526	5.3742024909	2.800438	0.005637247	0.03965750	−2.689306
614	TNFSF14	0.50155487	1.088612655	4.716795	4.664146e−06	0.0002015990	3.842763	CTF1	−0.77311586	2.033694507	−3.682284	0.0003020820	0.004808923	−0.0398556924	VWA5A	−0.48352154	2.922611225	−3.165059	0.001809198	0.01765232	−1.671983	DQ576593	0.017739124	0.0028847069	2.800391	0.005638033	0.03965750	−2.689430
615	F11R	−0.56667505	5.286890312	−4.708030	4.847043e−06	0.0002089412	3.806630	SLA	0.64818669	2.137733482	3.682063	0.0003023270	0.004808923	−0.0406011543	C1QBP	0.41023835	6.524497127	3.163833	0.001816448	0.01771654	−1.675593	TMX1	0.262332942	3.6269582570	2.800282	0.005639861	0.03965985	−2.689717
616	FAM110A	0.65795536	3.546670759	4.707902	4.849759e−06	0.0002089412	3.806104	ASTL	0.26093817	0.274946748	3.681920	0.0003024850	0.004808923	−0.0410815267	GIMAP4	0.59941027	3.772579804	3.163556	0.001818085	0.01772600	−1.676407	RN7SK	0.009819434	0.0536395503	2.799406	0.005654553	0.03975263	−2.692024
617	LOC100049716	−0.43502843	1.435045609	−4.707234	4.863978e−06	0.0002092141	3.803354	KCNIP3	−0.36117005	0.578208365	−3.681809	0.0003026075	0.004808923	−0.0414538084	IRF4	0.50670475	1.228575911	3.162760	0.001822810	0.01776554	−1.678750	MRPS34	0.253688005	5.9618485752	2.799221	0.005657669	0.03976162	−2.692512
618	AX747684	−1.18699791	2.466887123	−4.706655	4.876355e−06	0.0002094071	3.800966	HLA−DRB6	1.14408369	3.428600583	3.681528	0.0003029188	0.004810238	−0.0423990963	SNORD14	0.33468963	0.937703009	3.162505	0.001824326	0.01776953	−1.679500	CXXC11	0.095589146	0.1140428374	2.799152	0.005658828	0.03976162	−2.692693
619	C12orf75	1.01714739	5.672370484	4.706096	4.888312e−06	0.0002095814	3.798666	MANEA	0.38539741	2.712565325	3.681407	0.0003030528	0.004810238	−0.0428057423	CCL20	1.30040618	4.355701439	3.162466	0.001824559	0.01776953	−1.679616	LOC644649	0.069190961	0.1819860215	2.797848	0.005680791	0.03990537	−2.696127
620	PSME2	0.68834204	6.934730981	4.705072	4.910295e−06	0.0002101844	3.794451	SIRPB2	0.29516558	0.744522182	3.681051	0.0003034471	0.004813618	−0.0440014527	NEIL1	−0.40925903	1.585467632	−3.162286	0.001825628	0.01777055	−1.680144	PP12613	0.021859009	0.0328260636	2.797742	0.005682579	0.03990737	−2.696406
621	CLEC7A	0.61797091	1.693213944	4.702997	4.955151e−06	0.0002117629	3.785910	AK095221	0.22993442	1.374588471	3.680683	0.0003038555	0.004817217	−0.0452380847	KCNK9	−0.33129846	0.462803101	−3.162223	0.001826003	0.01777055	−1.680330	NASP	0.254907361	3.3637508538	2.796520	0.005703222	0.04004175	−2.699619
622	RPE	0.32399492	3.684394453	4.701160	4.995209e−06	0.0002126173	3.778347	OTOA	0.07246650	0.123383484	3.679815	0.0003048214	0.004829645	−0.0481561530	CISH	0.38561395	2.974255100	3.161412	0.001830834	0.01780745	−1.682715	POLB	0.313593856	3.2778921496	2.796083	0.005710632	0.04008317	−2.700770
623	TMEM155	0.15867172	0.159124139	4.700643	5.006539e−06	0.0002126173	3.776219	ZKSCAN2	0.24385825	0.889081189	3.679587	0.0003050763	0.004830800	−0.0489248876	HOXA7	−0.63876642	1.283472714	−3.161361	0.001831137	0.01780745	−1.682864	BOLA3−AS1	−0.182016962	0.5632413054	−2.795982	0.005712339	0.04008455	−2.701035
624	CBX2	0.83600327	1.579065826	4.700531	5.008980e−06	0.0002126173	3.775761	MGC3771	0.23642785	1.338416614	3.679206	0.0003055014	0.004834647	−0.0502051122	RPA1	0.23948771	4.131226980	3.161034	0.001833090	0.01781851	−1.683826	DL490795	−0.209764283	2.4632613634	−2.795493	0.005720649	0.04010170	−2.702323
625	ESD	−0.51798991	4.517814332	−4.700471	5.010305e−06	0.0002126173	3.775513	ARGFX	0.03587486	0.024736072	3.677636	0.0003072591	0.004859565	−0.0554796931	ARHGDIG	−0.66391806	1.066753107	−3.160946	0.001833617	0.01781851	−1.684085	HCLS1	0.570460009	3.5132109055	2.795454	0.005721309	0.04010170	−2.702425
626	LOC652276	0.23321665	0.825009301	4.700248	5.015200e−06	0.0002126173	3.774596	ABCB11	0.09770867	0.067554842	3.676893	0.0003080944	0.004869873	−0.0579755232	H2AFY2	−0.63654939	2.414452420	−3.160630	0.001835503	0.01782483	−1.685013	CD1B	0.138923808	0.2416614926	2.795310	0.005723746	0.04010170	−2.702803
627	DQ596646	−0.45478319	0.786847165	−4.695850	5.112711e−06	0.0002164055	3.756510	BC033323	0.10694377	0.360702182	3.675663	0.0003094818	0.004888891	−0.0621063545	L37717	0.02632062	0.007242626	3.160612	0.001835610	0.01782483	−1.685066	TEAD4	0.383829665	3.5754250363	2.795279	0.005724274	0.04010170	−2.702885
628	HLA−DQB2	1.24836977	2.534956612	4.692554	5.186989e−06	0.0002191999	3.742964	BRD3	−0.31370002	3.456954876	−3.674306	0.0003110199	0.004910266	−0.0666639722	ZNF527	0.16398608	0.789325999	3.160211	0.001838012	0.01784163	−1.686247	SALL2	−0.411832609	1.0128987273	−2.795268	0.005724470	0.04010170	−2.702915
629	LOC643387	0.08229920	0.152361303	4.688588	5.277744e−06	0.0002226805	3.726675	MIR610	0.06739113	0.026927134	3.674018	0.0003113469	0.004912507	−0.0676301078	ZNF860	0.27973345	0.988803212	3.159927	0.001839709	0.01785156	−1.687079	HIST1H1E	0.453597313	2.6450948229	2.795239	0.005724953	0.04010170	−2.702990
630	NEURL1B	−0.85572347	2.953589686	−4.687369	5.305952e−06	0.0002235153	3.721669	BC039523	0.18164962	0.520120852	3.673810	0.0003115832	0.004913314	−0.0683274981	TPM2	−1.01762894	6.454082390	−3.159763	0.001840696	0.01785462	−1.687564	MARS2	0.310440386	2.4588344753	2.795215	0.005725361	0.04010170	−2.703053
631	HOXC10	−1.58740277	2.009926377	−4.686431	5.327744e−06	0.0002237554	3.717820	RASAL3	0.66429654	2.132032899	3.673478	0.0003119607	0.004916345	−0.0694405827	RAB11FIP3	0.34220210	3.376531449	3.159591	0.001841723	0.01785805	−1.688067	IRF8	0.562816245	3.7876226957	2.794593	0.005735946	0.04016524	−2.704690
632	EIF4A1	0.44103682	6.546749720	4.686398	5.328512e−06	0.0002237554	3.717685	PLEKHA1	−0.38034229	4.028589698	−3.672951	0.0003125620	0.004922898	−0.0712107625	TERF1	−0.22139977	3.216561627	−3.159040	0.001845034	0.01788362	−1.689688	DQ585433	0.020530867	0.0023455182	2.794480	0.005737858	0.04016804	−2.704985
633	RUFY4	0.24328031	0.210345625	4.684977	5.361697e−06	0.0002247931	3.711855	IL23R	0.13356646	0.190706245	3.670432	0.0003154485	0.004965414	−0.0796608433	ZNF607	0.26543205	1.185513365	3.158763	0.001846699	0.01788546	−1.690502	PA2G4	0.309723922	6.0528368216	2.793964	0.005746651	0.04021898	−2.706342
634	NLRP9	0.12390227	0.172321339	4.682764	5.413804e−06	0.0002266198	3.702773	EIF2C4	−0.29360683	2.080524039	−3.669686	0.0003163087	0.004976003	−0.0821638925	CENPV	0.48063749	4.415771398	3.158719	0.001846964	0.01788546	−1.690632	AK093746	−0.356785538	0.9345846671	−2.793779	0.005749819	0.04022815	−2.706830
635	AOAH	0.81719278	2.057073230	4.678877	5.506490e−06	0.0002298280	3.686834	MIR4422	0.07092451	0.022603691	3.669452	0.0003165787	0.004977300	−0.0829482215	SNRNP70	0.20870926	5.101215615	3.158672	0.001847246	0.01788546	−1.690769	COQ5	−0.241881260	3.4594121293	−2.793710	0.005750993	0.04022815	−2.707011
636	ZNF230	0.23828895	0.971889407	4.678824	5.507766e−06	0.0002298280	3.686616	TRIM66	0.24691253	1.056339213	3.668692	0.0003174575	0.004988161	−0.0854959975	AVIL	0.49200373	1.056964317	3.158485	0.001848372	0.01788984	−1.691320	NUP88	0.217457037	3.6426454903	2.793580	0.005753215	0.04023309	−2.707353
637	AGBL2	0.21577255	0.354530979	4.676998	5.551826e−06	0.0002307642	3.679135	ALOXE3	0.19516520	0.204805038	3.668109	0.0003181332	0.004991880	−0.0874504253	SPI1	0.56552479	3.982502447	3.157849	0.001852199	0.01792035	−1.693187	NUP205	0.279233600	3.6527397556	2.793468	0.005755115	0.04023577	−2.707646
638	TMEM168	−0.49943312	3.298157396	−4.676829	5.555921e−06	0.0002307642	3.678443	ABHD8	−0.47776452	2.749419466	−3.668090	0.0003181558	0.004991880	−0.0875157782	KIAA1033	0.24295596	3.375860261	3.157632	0.001853511	0.01792650	−1.693825	TDP1	0.223350419	1.9937183978	2.793235	0.005759094	0.04025299	−2.708258
639	BC094791	0.02026942	0.011084287	4.676814	5.556289e−06	0.0002307642	3.678380	LRRC3	−0.47640876	1.709945977	−3.668001	0.0003182584	0.004991880	−0.0878121186	ADV21S1A1N	0.22213468	0.306431724	3.156769	0.001858724	0.01797037	−1.696360	CLDN22	0.238447310	0.8413560769	2.792981	0.005763433	0.04027271	−2.708926
640	LRRC8E	−0.64442388	1.752607766	−4.673537	5.636313e−06	0.0002337221	3.664954	DACT1	−0.56903259	2.265674639	−3.665143	0.0003215919	0.005041185	−0.0973885867	FAM71C	0.01899995	0.010053041	3.156302	0.001861553	0.01798393	−1.697733	LOC100506422	0.015029810	0.0157837452	2.792760	0.005767222	0.04028858	−2.709508
641	PSTK	0.35619357	1.273805902	4.667463	5.787568e−06	0.0002396198	3.640092	CNOT4	0.18402850	2.544221576	3.664788	0.0003220089	0.005044743	−0.0985797058	ZNF232	0.27695305	1.840329624	3.156256	0.001861826	0.01798393	−1.697865	SYNJ2BP−COX16	−0.193801595	3.5944623319	−2.792238	0.005776159	0.04034039	−2.710880
642	IPO7	−0.46879137	4.939529156	−4.665163	5.845879e−06	0.0002412361	3.630681	DYM	−0.30458149	3.510728931	−3.663897	0.0003230558	0.005056336	−0.1015627379	DQ570651	0.05194869	0.021061281	3.156201	0.001862160	0.01798393	−1.698027	DSEL	−0.263601492	0.7566878946	−2.792112	0.005778313	0.04034482	−2.711210
643	ATR	0.38687231	1.897849570	4.664935	5.851691e−06	0.0002412361	3.629749	GOLGA6L1	0.01052358	0.004940162	3.663571	0.0003234398	0.005056336	−0.1026545581	NEURL2	−0.35817314	1.607256172	−3.155469	0.001866601	0.01802028	−1.700177	KLK15	−0.234593204	0.2317518950	−2.791533	0.005788242	0.04040351	−2.712731
644	C1QC	1.07401107	6.893069984	4.664662	5.858639e−06	0.0002412361	3.628635	PIK3R2	0.47694619	6.207804290	3.663526	0.0003234925	0.005056336	−0.1028041041	LOC100500773	0.04025649	0.063265539	3.155199	0.001868243	0.01802628	−1.700970	CLNK	0.148124680	0.2185767906	2.790994	0.005797502	0.04045751	−2.714148
645	SRSF2	0.30786185	6.026252161	4.664493	5.862968e−06	0.0002412361	3.627941	ZNF212	0.24757051	2.328328243	3.663426	0.0003236108	0.005056336	−0.1031401596	MRPL37	0.26099729	5.876598845	3.155143	0.001868581	0.01802628	−1.701133	DACH1	−0.571934513	1.2739493271	−2.790659	0.005803264	0.04048707	−2.715028
646	TAS2R39	0.02351213	0.008548432	4.663604	5.885694e−06	0.0002417963	3.624310	ITM2C	−0.70812427	5.674468803	−3.663349	0.0003237015	0.005056336	−0.1033977046	GRAMD4	−0.37362255	3.473377358	−3.154942	0.001869803	0.01803151	−1.701723	SEC1	0.161427119	0.4843503682	2.790472	0.005806466	0.04049876	−2.715516
647	IRAK3	0.71456594	1.446868003	4.660455	5.966962e−06	0.0002445411	3.611437	E2F2	0.57160809	1.777053943	3.660421	0.0003271725	0.005107547	−0.1131990863	API5	−0.23075692	4.746746180	−3.154149	0.001874633	0.01806261	−1.704051	PIGG	0.231659896	2.7008984860	2.790379	0.005808076	0.04049935	−2.715762
648	NDNF	−0.90669065	1.261125765	−4.660302	5.970934e−06	0.0002445411	3.610812	LARP1	0.30623476	5.173930301	3.659521	0.0003282461	0.005121294	−0.1162094294	ZNF876P	−0.15156382	0.306015880	−3.154035	0.001875330	0.01806261	−1.704387	MFSD2B	0.064535225	0.1530019784	2.789953	0.005815406	0.04053981	−2.716879
649	HPRT1	0.52544982	4.596383224	4.658831	6.009290e−06	0.0002456279	3.604802	THG1L	0.35954632	3.033547475	3.658059	0.0003299975	0.005145596	−0.1210993895	ACTRT3	−0.26552008	0.824129304	−3.153995	0.001875570	0.01806261	−1.704502	EEF2K	0.248063614	3.0120303604	2.789667	0.005820337	0.04055341	−2.717630
650	PLEKHG3	−0.63446301	2.894920452	−4.658576	6.015982e−06	0.0002456279	3.603757	AK093898	−0.33423431	0.965483168	−3.656517	0.0003318545	0.005167729	−0.1262554976	TOP1P1	0.12414248	0.410914293	3.153966	0.001875750	0.01806261	−1.704589	CAPN7	−0.217385664	3.0282345205	−2.789663	0.005820413	0.04055341	−2.717642
651	CD47	0.49431042	4.346620326	4.657712	6.038621e−06	0.0002461735	3.600231	CAPRIN1	0.33774662	5.386182440	3.656463	0.0003319193	0.005167729	−0.1264348254	LGALS8	−0.24203248	3.819815784	−3.153437	0.001878978	0.01808124	−1.706140	INTS2	0.213584018	2.0343855638	2.789559	0.005822207	0.04055526	−2.717915
652	KYNU	1.03455510	2.362159935	4.657170	6.052889e−06	0.0002463767	3.598016	BC033124	−0.38761981	0.511417303	−3.656395	0.0003320012	0.005167729	−0.1266613780	AK055458	0.27451380	1.749751612	3.153426	0.001879048	0.01808124	−1.706174	LRRC2	−0.217385688	0.4743521782	−2.789411	0.005824766	0.04056244	−2.718304
653	LOC100287722	0.38097191	1.760782924	4.656711	6.065002e−06	0.0002464917	3.596139	RDM1	0.37515143	0.869414331	3.656001	0.0003324780	0.005172118	−0.1279801310	PSMA2	0.24900958	4.379431378	3.151734	0.001889414	0.01817439	−1.711138	APOOL	−0.269649649	2.7650688860	−2.789045	0.005831083	0.04058810	−2.719264
654	ACAD9	0.31876219	3.458702686	4.655135	6.106715e−06	0.0002478075	3.589706	TOPBP1	0.36841749	3.363606775	3.655785	0.0003327386	0.005173140	−0.1287000358	CASP7	0.40198358	4.008165958	3.150923	0.001894401	0.01821123	−1.713516	C14orf132	−0.553039798	1.6184121400	−2.789020	0.005831509	0.04058810	−2.719329
655	CCDC78	0.37510939	0.491139712	4.651628	6.200570e−06	0.0002512320	3.575390	KHDRBS1	0.18404785	5.540793219	3.655483	0.0003331044	0.005175795	−0.1297095907	GRID1	−0.23247724	0.536902608	−3.150877	0.001894681	0.01821123	−1.713649	AQR	0.206796328	3.4922379591	2.788784	0.005835583	0.04059957	−2.719947
656	FLYWCH2	−0.55977292	3.959036827	−4.648407	6.287990e−06	0.0002541565	3.562251	CDK5	0.40616786	3.640823595	3.654556	0.0003342301	0.005189859	−0.1328092285	LOC286190	−0.36425220	1.487201440	−3.150776	0.001895302	0.01821123	−1.713945	TSPAN17	0.252725647	3.5881233122	2.788748	0.005836217	0.04059957	−2.720044
657	CDCA7L	−0.76516943	3.310958967	−4.647996	6.299224e−06	0.0002541565	3.560575	TTC16	0.09315908	0.248996216	3.654257	0.0003345928	0.005189859	−0.1338056884	PGAM5	0.33185549	4.205633797	3.150498	0.001897018	0.01821618	−1.714762	NDUFB6	0.282402543	5.1697105687	2.788367	0.005842804	0.04062604	−2.721043
658	RIMKLA	−0.52217503	0.817059856	−4.647914	6.301479e−06	0.0002541565	3.560239	TLE6	0.35729307	0.736047866	3.654184	0.0003346817	0.005189859	−0.1340496985	AX746765	0.03013003	0.058477255	3.150470	0.001897190	0.01821618	−1.714844	SMPD4	0.177578270	4.1561684767	2.788351	0.005843083	0.04062604	−2.721085
659	ALOX15B	0.61702497	0.791548352	4.647295	6.318433e−06	0.0002544536	3.557718	LOC100652770	−0.67249687	2.491812972	−3.653968	0.0003349456	0.005189859	−0.1347740536	SPDYE7P	0.02278311	0.031638251	3.150241	0.001898600	0.01822312	−1.715514	CCDC58	0.309758775	4.3771395364	2.787646	0.005855292	0.04070026	−2.722934
660	IL2RA	0.68915455	1.577417900	4.644833	6.386377e−06	0.0002568001	3.547680	MINPP1	0.36168577	2.847446378	3.653933	0.0003349873	0.005189859	−0.1348883867	RAB8A	0.20054856	4.771585927	3.149703	0.001901925	0.01824844	−1.717092	VGLL4	−0.208225889	4.4526297935	−2.787078	0.005865157	0.04075816	−2.724425
661	ZNF847P	0.05623559	0.027648696	4.643965	6.410495e−06	0.0002573799	3.544143	AGPAT4	−0.25932390	0.860116683	−3.652572	0.0003366494	0.005212565	−0.1394353427	PRADC1	−0.34234999	3.555590333	−3.149457	0.001903441	0.01825639	−1.717811	TYR	0.008634229	0.0061939403	2.786593	0.005873580	0.04079262	−2.725696
662	TTC24	0.22527232	0.195682108	4.642121	6.462015e−06	0.0002590565	3.536631	LOC643733	0.34506762	1.093527688	3.652355	0.0003369157	0.005213648	−0.1401619112	BC127870	0.14567027	0.273846835	3.149316	0.001904318	0.01825820	−1.718227	LOC285819	0.060175285	0.1577417316	2.786464	0.005875819	0.04079262	−2.726034
663	TPI1P3	0.06686397	0.086997782	4.638915	6.552569e−06	0.0002622906	3.523572	SNED1	−0.40805465	1.549483333	−3.651736	0.0003376738	0.005221981	−0.1422267539	MAGED2	−0.40842874	5.248150884	−3.149160	0.001905284	0.01826086	−1.718684	BEST4	−0.183535482	0.3805489559	−2.786449	0.005876085	0.04079262	−2.726074
664	LOC127841	0.20147739	0.320923237	4.638296	6.570186e−06	0.0002625997	3.521052	C14orf93	0.23134562	2.171297067	3.651595	0.0003378478	0.005221981	−0.1426999356	HAPLN4	−0.50339671	0.817171800	−3.148750	0.001907824	0.01827860	−1.719886	ATG10	0.151694427	1.0668921608	2.786390	0.005877120	0.04079262	−2.726230
665	CXorf26	−0.55344035	4.275522591	−4.632749	6.730147e−06	0.0002685665	3.498481	PTGR1	−0.55641711	3.204523248	−3.650812	0.0003388100	0.005233807	−0.1453128212	MIR4738	0.09333530	0.069822302	3.148466	0.001909578	0.01828503	−1.720715	DDX11L2	0.230642810	0.5048272177	2.786350	0.005877801	0.04079262	−2.726333
666	VSIG8	0.58697340	1.556680425	4.632421	6.739714e−06	0.0002685665	3.497148	LRRN4	−0.46939059	0.501280766	−3.650602	0.0003390680	0.005234745	−0.1460121247	HLA−G	0.61697967	3.613972353	3.148419	0.001909872	0.01828503	−1.720854	SLC41A3	−0.218235746	3.7231039851	−2.786132	0.005881609	0.04080837	−2.726906
667	LOC653786	0.11794928	0.146902649	4.630816	6.786783e−06	0.0002700366	3.490618	MLLT4−AS1	−0.28252962	0.544823260	−3.650403	0.0003393144	0.005235503	−0.1466793986	TRIM54	−0.70295148	1.065443886	−3.147790	0.001913779	0.01831499	−1.722698	GSDMD	0.289917700	3.9674946561	2.785106	0.005899502	0.04092140	−2.729596
668	UNC5CL	1.10194920	3.231612909	4.629138	6.836295e−06	0.0002715994	3.483798	LOC100129083	0.70361166	1.824375497	3.649026	0.0003410162	0.005258703	−0.1512750966	P27_CRE	0.28911746	0.755159931	3.147693	0.001914382	0.01831499	−1.722982	KDM5B	−0.289096539	3.4472533015	−2.785021	0.005900983	0.04092140	−2.729819
669	KCTD5	0.40650756	4.372823230	4.626644	6.910558e−06	0.0002739625	3.473661	PIK3C2G	0.97021610	1.295897966	3.648782	0.0003413185	0.005260309	−0.1520891811	LINC00094	−0.25607078	2.582117306	−3.147317	0.001916720	0.01833075	−1.724083	SHISA2	−0.504289702	1.4299669040	−2.784295	0.005913670	0.04099434	−2.731721
670	SLC38A6	0.37121369	1.581846341	4.626448	6.916421e−06	0.0002739625	3.472865	GBE1	−0.30470184	2.689752362	−3.648335	0.0003418727	0.005265792	−0.1535793491	TP73−AS1	−0.34983545	1.240689054	−3.147045	0.001918414	0.01834035	−1.724880	BCYRN1	0.266119423	0.5845587967	2.784242	0.005914590	0.04099434	−2.731859
671	NUDC	0.39356228	6.228320657	4.624849	6.964467e−06	0.0002754545	3.466370	CKB	−1.20293584	5.371967837	−3.647678	0.0003426893	0.005275308	−0.1557709330	EFNA1	−0.49496244	4.683082411	−3.145503	0.001928040	0.01842573	−1.729395	NOTUM	−1.233422252	1.5890354677	−2.783870	0.005921119	0.04102887	−2.732835
672	IL2RB	0.98478800	3.028589488	4.623649	7.000734e−06	0.0002764769	3.461498	AMMECR1L	−0.19220733	2.691856367	−3.647504	0.0003429058	0.005275581	−0.1563511473	MYO1G	0.45843955	1.436521826	3.145122	0.001930428	0.01844191	−1.730511	DQ596074	0.255868474	0.4281947849	2.782593	0.005943519	0.04116802	−2.736179
673	SNRPA	0.35017844	5.558960558	4.621412	7.068852e−06	0.0002787522	3.452413	BTN3A3	0.56673834	3.458152900	3.645765	0.0003450774	0.005302184	−0.1621497949	P2RY12	0.23895630	0.403823616	3.144971	0.001931369	0.01844426	−1.730951	FCF1	−0.150795307	3.6679993095	−2.782549	0.005944302	0.04116802	−2.736296
674	GLB1L2	−0.73717348	1.947552914	−4.617784	7.180661e−06	0.0002827412	3.437691	FAM104A	0.21835344	3.885954451	3.645669	0.0003451979	0.005302184	−0.1624706362	TSPAN7	−0.81434172	3.049798857	−3.144435	0.001934734	0.01846975	−1.732521	ARMCX2	−0.443538728	2.1941639395	−2.782247	0.005949603	0.04119397	−2.737085
675	PLA2G2D	1.15125984	1.427916362	4.617131	7.200993e−06	0.0002831217	3.435039	RGS1	0.86503433	4.691054711	3.645640	0.0003452343	0.005302184	−0.1625674859	AATK	−0.61812367	2.191249742	−3.144291	0.001935638	0.01847173	−1.732942	PSMD12	0.199585840	4.2211891576	2.781997	0.005954007	0.04121371	−2.737740
676	ICA1	−0.78708280	3.638402704	−4.613257	7.322579e−06	0.0002874762	3.419333	TMEM161B	0.33094934	2.218031902	3.644718	0.0003463913	0.005316875	−0.1656403373	MESP2	−0.34641993	0.498976314	−3.143779	0.001938854	0.01849578	−1.734440	STIM2	0.232162996	2.7332731109	2.781717	0.005958941	0.04123711	−2.738474
677	FCGR3A	1.06057676	4.032623752	4.612759	7.338373e−06	0.0002876707	3.417312	C17orf39	−0.30755133	2.077127672	−3.644540	0.0003466160	0.005317250	−0.1662360747	ZNF239	0.32664419	1.722410772	3.143659	0.001939611	0.01849635	−1.734792	DFNB31	−0.363121737	1.4155126246	−2.781491	0.005962917	0.04125388	−2.739065
678	SSBP1	0.40447864	5.073108336	4.611259	7.386083e−06	0.0002891140	3.411233	TRBV25−1	0.40992043	0.391253604	3.643902	0.0003474191	0.005326490	−0.1683616071	SLC12A5	0.05190704	0.087968026	3.143422	0.001941105	0.01849943	−1.735486	SERPINB1	0.452179054	6.7795641974	2.781358	0.005965264	0.04125936	−2.739413
679	ABTB1	−0.60838036	3.574311439	−4.610130	7.422199e−06	0.0002900998	3.406658	WDR77	0.35681340	4.207010060	3.643379	0.0003480785	0.005333519	−0.1701032986	SENP2	0.28648820	3.374313843	3.143385	0.001941334	0.01849943	−1.735593	IYD	−0.602383833	1.5806071640	−2.781157	0.005968809	0.04127311	−2.739939
680	IL15RA	0.57632412	3.661330835	4.609473	7.443292e−06	0.0002904964	3.403996	XKRX	0.36760064	0.458004208	3.641920	0.0003499257	0.005358730	−0.1749645228	MIR1197	0.02467085	0.004651540	3.143276	0.001942025	0.01849943	−1.735914	PSMC4	0.265706993	6.1939439996	2.780924	0.005972930	0.04129086	−2.740550
681	CEP128	0.25261859	0.753886178	4.607864	7.495199e−06	0.0002920926	3.397478	ARHGEF37	−0.52813622	1.981570750	−3.641611	0.0003503190	0.005361659	−0.1759961066	IPP	−0.20040816	2.415412457	−3.143144	0.001942852	0.01850066	−1.736298	MEF2A	−0.324231017	3.6824907791	−2.780811	0.005974921	0.04129386	−2.740845
682	MS4A6A	0.89080971	3.503547144	4.605123	7.584400e−06	0.0002951355	3.386382	ZNF780A	0.23969024	1.340747179	3.641390	0.0003505999	0.005362865	−0.1767322723	CHRD	−0.38801505	1.556423764	−3.142917	0.001944285	0.01850767	−1.736963	LOC100505738	−0.350979761	1.3604122270	−2.780613	0.005978415	0.04130725	−2.741363
683	ZNF286A	0.29755542	1.222071211	4.602788	7.661217e−06	0.0002976882	3.376930	ODF3B	0.57117026	3.962026733	3.641085	0.0003509878	0.005365706	−0.1777477310	MLF1	−0.61919175	1.505272223	−3.141854	0.001951000	0.01856493	−1.740072	LUM	−0.579047712	7.4570580523	−2.780057	0.005988256	0.04136448	−2.742819
684	LOC728758	0.14715227	0.261845911	4.600915	7.723390e−06	0.0002992357	3.369350	STT3A	0.29861960	5.596341101	3.640640	0.0003515548	0.005371280	−0.1792301661	LOC286442	0.08320183	0.120117044	3.141355	0.001954157	0.01858831	−1.741531	BAZ1A	0.261320417	3.8817572310	2.779796	0.005992878	0.04138563	−2.743502
685	GAD1	0.69600325	0.748462021	4.600909	7.723593e−06	0.0002992357	3.369325	MMP17	−0.43488776	0.958695156	−3.638887	0.0003537956	0.005402405	−0.1850651166	MEP1A	−1.08149841	1.503660202	−3.140771	0.001957865	0.01861690	−1.743240	KIAA1279	−0.205954391	3.6911684360	−2.779366	0.006000491	0.04142743	−2.744626
686	TMEM202	0.02420598	0.009342101	4.595277	7.913435e−06	0.0003061438	3.346554	CYTH1	0.33023088	3.771375654	3.637745	0.0003552639	0.005421708	−0.1888685577	L3MBTL2	0.18965898	3.521462245	3.140595	0.001958977	0.01862081	−1.743752	PATZ1	0.254322243	3.4590242977	2.778743	0.006011547	0.04149046	−2.746256
687	ATIC	0.39329205	5.169692512	4.594767	7.930841e−06	0.0003063706	3.344493	HLTF	−0.55312191	2.604639284	−3.636906	0.0003563448	0.005434944	−0.1916581828	LRRC20	0.36423468	2.679143730	3.140211	0.001961421	0.01863736	−1.744877	JA611286	0.005554886	0.0007483904	2.778663	0.006012973	0.04149046	−2.746466
688	C15orf55	0.07338154	0.078847366	4.593590	7.971149e−06	0.0003074801	3.339739	LARP4	−0.33947379	3.158279037	−3.636600	0.0003567409	0.005434944	−0.1926783636	TNFRSF9	0.33979813	0.891892561	3.140015	0.001962667	0.01863756	−1.745449	ERI3	−0.182943740	4.6709921735	−2.778588	0.006014311	0.04149046	−2.746663
689	TMEM218	0.29543293	2.314513838	4.591172	8.054596e−06	0.0003102481	3.329974	OR10S1	0.02286081	0.003574146	3.636596	0.0003567457	0.005434944	−0.1926905623	PRMT3	0.28390443	2.795745394	3.139987	0.001962846	0.01863756	−1.745532	C12orf32	0.298275783	4.1008998244	2.777331	0.006036678	0.04163344	−2.749949
690	C2orf16	0.17761050	0.341837943	4.589864	8.100096e−06	0.0003114478	3.324692	SMA4	0.02961733	0.078740727	3.636194	0.0003572664	0.005439114	−0.1940299113	NLGN2	−0.49399068	2.477090427	−3.139702	0.001964658	0.01864809	−1.746364	SMAD9	−0.528801192	1.5335707006	−2.777247	0.006038174	0.04163344	−2.750168
691	AV8S2	0.49231227	0.671908264	4.589603	8.109215e−06	0.0003114478	3.323637	MAMSTR	−0.33726667	0.954388746	−3.635948	0.0003575839	0.005439114	−0.1948455945	PITHD1	0.22981687	4.215413581	3.138497	0.001972343	0.01871435	−1.749884	MSR1	0.502934518	2.3619692193	2.777050	0.006041699	0.04164692	−2.750685
692	NUDT19	−0.69010952	2.898762785	−4.584501	8.289264e−06	0.0003179029	3.303046	SLC6A14	1.10593258	2.306387217	3.635910	0.0003576342	0.005439114	−0.1949747970	TMEM158	−0.55144516	3.038683766	−3.137701	0.001977438	0.01875598	−1.752211	SOCS5	−0.217095728	2.6775825071	−2.776682	0.006048260	0.04168132	−2.751646
693	C8A	0.27067710	0.124301855	4.583732	8.316736e−06	0.0003184962	3.299944	KIAA2012	0.05942011	0.065653094	3.635541	0.0003581116	0.005443255	−0.1961997748	SLC30A7	0.24046367	3.168748881	3.136504	0.001985117	0.01882208	−1.755705	LOC728228	0.065133095	0.1050091888	2.776556	0.006050508	0.04168262	−2.751975
694	TRBV29−1	0.82082566	1.274223313	4.579699	8.462233e−06	0.0003236011	3.283684	ZNF618	0.36616764	1.836212925	3.635356	0.0003583518	0.005443788	−0.1968153852	TMEM231	−0.30461482	1.729039333	−3.136121	0.001987580	0.01883871	−1.756823	SLC16A11	−0.209681664	0.5073341057	−2.776496	0.006051590	0.04168262	−2.752133
695	DQ574682	0.12513307	0.219638463	4.577213	8.553116e−06	0.0003266060	3.273669	TMEM198	−0.50476702	1.691128995	−3.635022	0.0003587863	0.005447271	−0.1979282072	PLGRKT	0.40231497	3.264523834	3.135663	0.001990531	0.01885994	−1.758161	BC037892	0.018248585	0.0192541889	2.776400	0.006053295	0.04168355	−2.752382
696	RPS19	0.50066900	9.190546236	4.575869	8.602686e−06	0.0003276271	3.268252	TRIM5	0.34610205	2.630388631	3.634319	0.0003597004	0.005457804	−0.2002646369	AK056524	0.01798043	0.028493298	3.135053	0.001994465	0.01889047	−1.759941	XPNPEP2	−0.811333609	1.4024080254	−2.776217	0.006056579	0.04169534	−2.752862
697	TTC12	0.35790712	1.633634395	4.575818	8.604547e−06	0.0003276271	3.268049	LUC7L2	0.20000197	4.089889882	3.634157	0.0003599111	0.005457804	−0.2008021918	MXRA5	−0.66333320	4.595807906	−3.134256	0.001999623	0.01892606	−1.762270	TRB	0.363479319	0.8077014849	2.775782	0.006064350	0.04173801	−2.753997
698	LOC126536	0.08854554	0.021359573	4.574018	8.671344e−06	0.0003296974	3.260799	ATG4C	0.21541418	2.335865068	3.634015	0.0003600970	0.005457804	−0.2012763852	COPS3	0.25926798	4.351453270	3.134175	0.002000142	0.01892606	−1.762504	ZNF852	0.191081691	1.3128987395	2.775246	0.006073952	0.04179326	−2.755398
699	P4HA2	−0.53725771	3.939565925	−4.572824	8.715919e−06	0.0003309181	3.255993	CD4	0.73766030	3.848643272	3.632666	0.0003618603	0.005481398	−0.2057614059	SYT6	0.04318101	0.068946586	3.134141	0.002000362	0.01892606	−1.762603	PMS1	0.190175066	2.2332090022	2.773933	0.006097533	0.04194464	−2.758827
700	LOC113386	0.37633627	4.343762745	4.568390	8.883433e−06	0.0003367963	3.238148	ZNF497	0.23389462	0.979687658	3.632345	0.0003622809	0.005484640	−0.2068281211	NCF1	0.27331089	0.813231655	3.132986	0.002007857	0.01899020	−1.765975	BC005081	−0.651158108	2.2896606260	−2.773732	0.006101162	0.04195873	−2.759354

	Gene	logFC	AveExpr	t	P.Value	adj.P.Val	B	Gene	logFC	AveExpr	t	P.Value	adj.P.Val	B	Gene	logFC	AveExpr	t	P.Value	adj.P.Val	B	Gene	logFC	AveExpr	t	P.Value	adj.P.Val	B
701	GPR153	−0.77461233	2.756770660	−4.564278	9.041510e−06	0.0003420216	3.221615	CEP152	0.23869441	1.059967750	3.631463	0.0003634393	0.005496146	−0.2097593	NT5DC3	−0.42320705	1.752850041	−3.132776	0.002009220	0.01899632	−1.766586	CACNA2D2	−0.33998043	0.746634237	−2.773637	0.006102874	0.04195963	−2.759602
702	NECAP2	0.36809139	4.751564114	4.564136	9.047032e−06	0.0003420216	3.221043	DDX47	0.27286119	3.752441682	3.631451	0.0003634551	0.005496146	−0.2097992	SEMA4F	−0.26531045	1.279853957	−3.132506	0.002010978	0.01900618	−1.767375	ATP2C2	−0.69168335	2.374355215	−2.773189	0.006110930	0.04200414	−2.760770
703	MIR4723	0.18513990	0.440564870	4.563605	9.067664e−06	0.0003423140	3.218908	ZNF280C	0.26586151	1.306566066	3.631107	0.0003639073	0.005499849	−0.2109406	CYBB	0.69255945	3.668653694	3.132373	0.002011843	0.01900758	−1.767763	STEAP2	0.45173600	2.827510750	2.772776	0.006118382	0.04203591	−2.761848
704	CTLA4	0.63626588	1.254547542	4.562842	9.097372e−06	0.0003429477	3.215842	KCNQ4	−0.46396345	0.955254975	−3.630608	0.0003645659	0.005506667	−0.2126008	SNORD38A	0.28157134	1.057600117	3.131641	0.002016612	0.01904586	−1.769897	GRB2	0.17525884	5.357605993	2.772758	0.006118720	0.04203591	−2.761897
705	LOC643401	0.51636191	0.356332231	4.561342	9.156076e−06	0.0003446711	3.209814	GLS2	0.37439408	0.892530176	3.629564	0.0003659454	0.005524360	−0.2160682	NR0B2	−0.98185204	2.749978570	−3.131070	0.002020338	0.01907426	−1.771561	NUFIP1	0.24226320	1.869867297	2.771970	0.006132959	0.04212282	−2.763954
706	TCRBV5S2	0.49567588	0.685821594	4.558044	9.286370e−06	0.0003490807	3.196571	INF2	−0.42650518	5.092591039	−3.628878	0.0003668547	0.005532383	−0.2183466	NACA	0.27191012	5.918425280	3.130501	0.002024057	0.01909925	−1.773219	AK296947	0.32255387	2.909316679	2.771768	0.006136603	0.04213695	−2.764480
707	GNRHR2	−0.33744313	2.692491274	−4.555119	9.403452e−06	0.0003525742	3.184829	SNORD28	0.44995376	1.341769760	3.628848	0.0003668938	0.005532383	−0.2184444	FABP2	−0.85857519	1.081045398	−3.130445	0.002024424	0.01909925	−1.773382	OAZ1	0.20527370	8.653045477	2.771232	0.006146312	0.04219270	−2.765879
708	TAP2	0.69101792	3.957701762	4.554748	9.418400e−06	0.0003525742	3.183340	OR51V1	0.02848590	0.008175214	3.627204	0.0003690823	0.005562223	−0.2239042	TTC28	−0.42232410	1.788015279	−3.130068	0.002026893	0.01911574	−1.774481	C14orf80	0.31585766	2.447341358	2.771137	0.006148031	0.04219358	−2.766126
709	NMI	0.50713851	3.623346047	4.554729	9.419161e−06	0.0003525742	3.183264	NR_026754	−0.62900947	4.350643799	−3.626871	0.0003695273	0.005563105	−0.2250104	FERMT1	0.67766788	3.928089930	3.129071	0.002033442	0.01916208	−1.777389	ANKRD13C	−0.15065621	2.260681375	−2.771000	0.006150520	0.04219976	−2.766484
710	PIGZ	−0.61260683	1.586479863	−4.552656	9.503137e−06	0.0003552165	3.174946	TRAPPC11	0.24015633	3.077018044	3.626846	0.0003695601	0.005563105	−0.2250918	DOCK2	0.56648637	1.929672879	3.129027	0.002033726	0.01916208	−1.777515	MAP4K3	−0.26479144	3.330341298	−2.769914	0.006170245	0.04232415	−2.769318
711	BC043356	0.05551057	0.075300679	4.550824	9.577961e−06	0.0003575099	3.167597	ZNF841	−0.37103094	1.948710970	−3.626269	0.0003703319	0.005571564	−0.2270072	OR7A17	0.01499768	0.004498237	3.128990	0.002033971	0.01916208	−1.777624	DQ583891	0.02616479	0.005363396	2.769696	0.006174197	0.04234031	−2.769884
712	CD2	1.02242394	2.912550986	4.543788	9.870584e−06	0.0003679149	3.139397	CILP2	−0.58651332	0.934158747	−3.625705	0.0003710888	0.005579788	−0.2288813	TSGA13	0.10407740	0.292542301	3.128765	0.002035455	0.01916925	−1.778281	MIR769	0.03138371	0.011370120	2.769566	0.006176566	0.04234561	−2.770223
713	MAP3K2	−0.27247787	2.955470646	−4.543206	9.895140e−06	0.0003681919	3.137069	PSTPIP1	0.40692096	1.354039755	3.624960	0.0003720889	0.005588755	−0.2313519	MTERFD2	0.18031703	2.257424338	3.128379	0.002037995	0.01918636	−1.779405	ANKS1B	0.12879263	0.737235099	2.769195	0.006183330	0.04238104	−2.771192
714	TCRAV5.1a	0.48111201	0.576600873	4.542955	9.905762e−06	0.0003681919	3.136064	BC053951	0.05835411	0.030112663	3.624947	0.0003721063	0.005588755	−0.2313949	SNORD22	0.35301648	1.457027177	3.127009	0.002047039	0.01926467	−1.783396	MUC2	−1.44304305	2.114163824	−2.768879	0.006189084	0.04240384	−2.772015
715	HDAC11	−0.52827586	2.565663250	−4.542235	9.936300e−06	0.0003688104	3.133180	SLC25A17	0.29868898	2.955057599	3.624402	0.0003728397	0.005596602	−0.2332022	MEX3C	−0.29733609	3.327567496	−3.126536	0.002050167	0.01928242	−1.784772	SNORA71A	0.25791510	0.877486158	2.768837	0.006189852	0.04240384	−2.772125
716	TMCO5A	0.06106694	0.018183864	4.541756	9.956683e−06	0.0003690508	3.131260	COL3A1	−0.84026618	8.273482385	−3.623755	0.0003737127	0.005605186	−0.2353491	CES4A	−0.33520130	0.734368508	−3.126504	0.002050378	0.01928242	−1.784865	OPRM1	0.01642697	0.025745627	2.768452	0.006196883	0.04244105	−2.773129
717	FAM59B	−0.43697060	1.300191673	−4.539068	1.007166e−05	0.0003727917	3.120503	RIPK2	0.41060164	3.484388596	3.623665	0.0003738340	0.005605186	−0.2356469	BC024248	−0.48764176	0.834342430	−3.126178	0.002052541	0.01929592	−1.785815	CCDC7	0.10446301	0.585795972	2.768328	0.006199151	0.04244563	−2.773453
718	DPP4	−1.21260119	2.770054474	−4.533989	1.029248e−05	0.0003804347	3.100184	RABL2B	0.25968897	2.245468222	3.623112	0.0003745826	0.005612364	−0.2374833	ZNF66	0.18657333	0.442574777	3.125625	0.002056211	0.01932358	−1.787425	ATG16L2	0.21335826	1.858174615	2.768032	0.006204553	0.04246548	−2.774224
719	NLRC4	0.25733781	0.602032779	4.532868	1.034185e−05	0.0003813893	3.095701	SLC39A4	−0.80067314	3.882573002	−3.622966	0.0003747805	0.005612364	−0.2379679	IGHG1	1.56010508	9.013422490	3.124330	0.002064828	0.01939769	−1.791194	FKBP1B	−0.57117868	2.635198241	−2.767994	0.006205250	0.04246548	−2.774323
720	HDAC1	0.40059706	5.513465066	4.532750	1.034705e−05	0.0003813893	3.095230	PHKA1	−0.39003644	1.250753483	−3.622843	0.0003749471	0.005612364	−0.2383760	SEC14L1	0.30404829	3.359971637	3.123490	0.002070435	0.01944348	−1.793638	ZNF518A	0.30511745	2.281726396	2.767615	0.006212186	0.04249405	−2.775311
721	C12orf76	0.22318107	0.817409434	4.531309	1.041086e−05	0.0003832094	3.089470	MGST3	−0.42881450	5.935362984	−3.622535	0.0003753639	0.005615436	−0.2393958	ID2B	0.15380556	0.360452259	3.122752	0.002075370	0.01948293	−1.795783	GLIS2	−0.45361404	3.454974333	−2.767547	0.006213432	0.04249405	−2.775489
722	SLC25A4	−0.72442712	2.879219764	−4.527698	1.057241e−05	0.0003881368	3.075045	KEL	0.31248918	0.334999587	3.621925	0.0003761926	0.005624662	−0.2414198	AHNAK2	−0.89811957	2.512189475	−3.122234	0.002078845	0.01950478	−1.797290	SLC5A2	−0.24482902	3.492892859	−2.767503	0.006214228	0.04249405	−2.775602
723	IGH	0.17836491	0.198747380	4.527663	1.057398e−05	0.0003881368	3.074906	RPS27L	0.47488705	5.315634769	3.621530	0.0003767290	0.005627997	−0.2427277	C9orf91	0.23484182	2.329566780	3.122186	0.002079168	0.01950478	−1.797430	PROL1	0.02229929	0.006622438	2.767372	0.006216636	0.04249956	−2.775945
724	HLA−DPA1	1.27204424	7.086896172	4.526802	1.061288e−05	0.0003890265	3.071467	RANBP1	0.40964430	5.736226645	3.621449	0.0003768398	0.005627997	−0.2429976	IGSF3	−0.43496628	2.317497780	−3.121795	0.002081789	0.01951981	−1.798566	BET3L	0.08376713	0.218210854	2.767118	0.006221283	0.04251868	−2.776606
725	HESX1	0.24490422	0.464783220	4.520916	1.088242e−05	0.0003983566	3.047974	FOXRED2	0.66355762	3.382546309	3.621029	0.0003774114	0.005633363	−0.2443887	FAM40B	0.41567722	1.108048325	3.121728	0.002082240	0.01951981	−1.798761	TAF5	0.21943667	1.791771857	2.766989	0.006223635	0.04251868	−2.776940
726	RARRES3	1.36575451	6.041367023	4.518168	1.101054e−05	0.0004024914	3.037012	APOC4	0.85135350	2.358205389	3.620189	0.0003785575	0.005647295	−0.2471719	GABRB3	−0.55590351	0.927009983	−3.121436	0.002084204	0.01953132	−1.799610	FLJ33534	−0.02132847	0.031495513	−2.766957	0.006224238	0.04251868	−2.777026
727	NFAM1	0.59698501	1.572166760	4.516035	1.111096e−05	0.0004056036	3.028509	MT1M	−1.00811306	2.251088164	−3.619623	0.0003793322	0.005650741	−0.2490482	TNRC18	−0.36439038	4.743080650	−3.121276	0.002085283	0.01953453	−1.800077	MAPKAP1	−0.21203520	3.943147094	−2.766600	0.006230774	0.04255237	−2.777955
728	TK1	0.75356178	5.360235287	4.514799	1.116952e−05	0.0004071812	3.023586	RCN3	−0.68251659	4.380294747	−3.619601	0.0003793622	0.005650741	−0.2491206	ZBTB46	0.27169020	1.585448890	3.118290	0.002105462	0.01971660	−1.808754	ROCK2	−0.31378721	3.443801098	−2.765937	0.006242962	0.04261901	−2.779683
729	UGT2B28	0.11124379	0.027344652	4.514383	1.118932e−05	0.0004073435	3.021927	RPS13	0.29279725	9.161195857	3.619554	0.0003794272	0.005650741	−0.2492781	PHGR1	−1.80677460	5.100808993	−3.117973	0.002107613	0.01972328	−1.809674	FLJ35390	−0.23549130	0.992491751	−2.765894	0.006243743	0.04261901	−2.779794
730	THAP10	−0.44936225	1.576438294	−4.513375	1.123740e−05	0.0004085332	3.017912	PRIM1	0.44814962	2.881726602	3.616630	0.0003834531	0.005706145	−0.2589645	CSMD2	−0.14518758	0.295231987	−3.117796	0.002108814	0.01972328	−1.810187	FSTL3	−0.56703967	3.589388051	−2.765330	0.006254115	0.04267882	−2.781262
731	AX748120	0.16938634	0.318772741	4.505911	1.159974e−05	0.0004205937	2.988193	SNX4	0.28566389	3.904089238	3.616540	0.0003835775	0.005706145	−0.2592622	KLHL6	0.49440151	1.318833802	3.117769	0.002108999	0.01972328	−1.810266	KIF20B	0.18327146	1.246494478	2.765064	0.006259014	0.04270128	−2.781955
732	FAM3B	−1.38727141	2.332583832	−4.505888	1.160084e−05	0.0004205937	2.988104	GSTZ1	0.42633000	2.454211488	3.615726	0.0003847046	0.005719416	−0.2619549	RPL41	0.34481890	4.701809308	3.117662	0.002109726	0.01972328	−1.810577	AK096023	0.02403796	0.055360125	2.764931	0.006261474	0.04270708	−2.782302
733	APOC1	1.28383402	5.630217051	4.504137	1.168745e−05	0.0004229198	2.981139	SRSF3	0.20229363	5.557454380	3.615585	0.0003849006	0.005719416	−0.2624222	AK098198	0.05363671	0.097765181	3.117638	0.002109890	0.01972328	−1.810647	BFSP2	0.10003394	0.195829315	2.764637	0.006266898	0.04273310	−2.783068
734	OR6C65	0.04707704	0.008899628	4.503948	1.169687e−05	0.0004229198	2.980385	CDCA4	0.44066599	3.353310335	3.615177	0.0003854681	0.005724644	−0.2637743	CENPO	0.26737855	3.138380479	3.117488	0.002110915	0.01972590	−1.811084	RBL1	0.32106746	2.100733744	2.763880	0.006280862	0.04281732	−2.785037
735	AK055459	0.86866753	0.800495950	4.502278	1.178013e−05	0.0004253509	2.973743	FAM20C	−0.64458707	4.096243710	−3.614723	0.0003860989	0.005728955	−0.2652746	PCOLCE	−0.62313652	4.847650359	−3.116854	0.002115229	0.01975926	−1.812923	SMG9	0.23173315	3.352175815	2.763644	0.006285226	0.04283366	−2.785652
736	AK127292	0.10525185	0.044213096	4.499202	1.193493e−05	0.0004294033	2.961519	NOV	−0.68169085	1.779529870	−3.614658	0.0003861902	0.005728955	−0.2654916	SCARF2	−0.54618326	3.188152927	−3.116719	0.002116148	0.01976089	−1.813315	TIGD5	−0.31101994	2.773438194	−2.763559	0.006286810	0.04283366	−2.785874
737	HLA−DOA	1.20330588	2.848668930	4.499180	1.193604e−05	0.0004294033	2.961431	CD99P1	0.29791132	1.245304980	3.614282	0.0003867146	0.005733530	−0.2667369	LOC282997	0.23322626	0.963328300	3.116334	0.002118775	0.01977846	−1.814432	AJ004954	0.66897803	1.640457821	2.763489	0.006288102	0.04283366	−2.786056
738	NUDT21	0.35871477	4.416183509	4.499084	1.194090e−05	0.0004294033	2.961050	XRN1	0.34704491	2.511943272	3.613113	0.0003883479	0.005752950	−0.2706045	C1orf162	0.66045987	3.963332057	3.115977	0.002121220	0.01979433	−1.815471	DBF4B	0.23268568	1.507359589	2.763376	0.006290193	0.04283691	−2.786350
739	TMIGD2	0.34834412	0.618012712	4.493273	1.223895e−05	0.0004395258	2.937968	MtDNA_ssA	0.06672480	0.176050246	3.613034	0.0003884580	0.005752950	−0.2708645	FXYD4	−0.60728967	0.803976123	−3.115634	0.002123564	0.01980923	−1.816466	CXorf21	0.27430107	1.084571684	2.763250	0.006292526	0.04284180	−2.786678
740	GGTA1P	0.45125577	1.019344985	4.484690	1.269227e−05	0.0004551894	2.903921	SH2D1B	0.20570307	0.357320171	3.611971	0.0003899497	0.005771822	−0.2743815	AMBRA1	0.21008380	3.056395993	3.114947	0.002128267	0.01984612	−1.818459	ITGB3BP	0.27296503	2.680113810	2.762577	0.006304992	0.04291567	−2.788429
741	ARHGAP19	0.35946119	1.560420795	4.483835	1.273826e−05	0.0004562223	2.900535	LOC728024	0.15117245	0.635606757	3.611485	0.0003906336	0.005778720	−0.2759892	TCR	0.26732862	0.430871223	3.114389	0.002132095	0.01987484	−1.820078	CDK11B	−0.20330703	3.472136417	−2.762414	0.006308022	0.04292528	−2.788853
742	ARPC3	0.30484198	7.711753511	4.481404	1.287003e−05	0.0004603203	2.890903	WNT7B	−0.73682738	0.968410066	−3.610665	0.0003917899	0.005792412	−0.2787014	NCOA3	0.32208695	3.276514858	3.114191	0.002133459	0.01988058	−1.820654	FGFR1OP	0.30416735	2.793437982	2.762006	0.006315591	0.04295920	−2.789914
743	GSTP1	−0.70293019	8.806115205	−4.481048	1.288942e−05	0.0004603933	2.889494	DQ596940	0.47093359	1.655831562	3.610519	0.0003919956	0.005792412	−0.2791829	DEPTOR	−0.48753288	3.836894412	−3.113590	0.002137593	0.01991210	−1.822398	C1QTNF2	−0.26266280	0.799661693	−2.761971	0.006316244	0.04295920	−2.790006
744	AP4B1	0.37639556	2.199035609	4.479917	1.295125e−05	0.0004619802	2.885014	ZBTB32	0.28143271	0.544249475	3.609505	0.0003934304	0.005810379	−0.2825353	PCDH7	0.73800638	2.448961535	3.112916	0.002142239	0.01994838	−1.824353	LOC100506540	0.25790427	2.014746995	2.761731	0.006320704	0.04297852	−2.790630
745	TNFSF13B	0.81927990	2.686726761	4.478077	1.305248e−05	0.0004649662	2.877726	ARNTL	0.23757857	2.007044903	3.608687	0.0003945920	0.005824292	−0.2852401	BC067244	−0.30316829	3.480204362	−3.112750	0.002143382	0.01995202	−1.824834	C2orf47	0.22791880	3.450161933	2.761183	0.006330886	0.04302615	−2.792053
746	GPR85	0.10912852	0.235353919	4.477466	1.308623e−05	0.0004655435	2.875310	TSFM	0.39889165	3.372794403	3.608113	0.0003954081	0.005833094	−0.2871358	WFS1	−0.55792668	4.025799849	−3.112165	0.002147418	0.01997897	−1.826528	BC027846	0.04026509	0.049047084	2.761180	0.006330952	0.04302615	−2.792063
747	C7orf61	0.20262629	0.326434136	4.475381	1.320212e−05	0.0004690376	2.867057	SYT7	−0.64954173	0.891325498	−3.607423	0.0003963921	0.005842906	−0.2894160	LOC388499	0.04561607	0.040728168	3.112113	0.002147783	0.01997897	−1.826681	SNUPN	−0.21169239	2.943340985	−2.760655	0.006340732	0.04308159	−2.793428
748	MRPL44	0.33106009	4.019705114	4.474504	1.325116e−05	0.0004701503	2.863588	T−cell_receptor_IGR_b_14_Vbeta_13	0.48912731	0.844573950	3.607338	0.0003965136	0.005842906	−0.2896971	TOR2A	0.34832775	3.300200452	3.111886	0.002149349	0.01998653	−1.827338	AK172755	0.02913418	0.048428849	2.760248	0.006348324	0.04312213	−2.794486
749	GTF2H2	0.17060018	0.387681448	4.472223	1.337952e−05	0.0004739717	2.854565	PPP2R4	0.28811980	4.745411802	3.607157	0.0003967726	0.005843478	−0.2902963	FAM160A1	0.34605745	1.337536002	3.111722	0.002150488	0.01999012	−1.827815	RPL26L1	0.29998069	3.442539857	2.760059	0.006351852	0.04313209	−2.794977
750	ATP11C	0.44031194	2.051059692	4.471957	1.339458e−05	0.0004739717	2.853512	FAM135A	0.42363036	2.350671016	3.605525	0.0003991105	0.005873996	−0.2956863	PKIG	−0.54624522	4.594615560	−3.110864	0.002156436	0.02003840	−1.830303	IRF3	0.24655028	4.125518326	2.759939	0.006354098	0.04313209	−2.795290
751	PLA2G7	0.84476489	2.339032642	4.468804	1.357420e−05	0.0004796882	2.841046	C15orf54	0.09668762	0.049635824	3.605295	0.0003994414	0.005873996	−0.2964464	NAT2	−0.65903891	0.894573414	−3.110447	0.002159329	0.02005826	−1.831510	SLC5A10	0.17993874	0.827733891	2.759883	0.006355135	0.04313209	−2.795434
752	TPBG	−0.78071850	2.917941619	−4.467173	1.366802e−05	0.0004823611	2.834601	CLEC4E	0.47524419	1.027481701	3.605157	0.0003996398	0.005873996	−0.2969022	NAT10	0.29158808	4.111735770	3.109879	0.002163282	0.02008794	−1.833157	FASTKD3	0.26441060	2.164433955	2.759769	0.006357263	0.04313209	−2.795730
753	BAHCC1	−0.57222385	1.936736522	−4.465117	1.378716e−05	0.0004859194	2.826479	PCDHGC5	−0.37593480	1.542856160	−3.605094	0.0003997301	0.005873996	−0.2971093	JA611269	0.36175871	2.307896415	3.109417	0.002166500	0.02011078	−1.834496	DLGAP5	0.45834676	2.791557736	2.759734	0.006357917	0.04313209	−2.795821
754	IL12B	0.11745525	0.113992338	4.462528	1.393861e−05	0.0004906059	2.816256	HOXA11−AS	−0.70379343	1.029392966	−3.604681	0.0004003252	0.005877978	−0.2984742	PAQR5	−0.44529709	1.266644632	−3.109256	0.002167620	0.02011415	−1.834961	RRNAD1	−0.24223833	3.252165533	−2.758963	0.006372340	0.04321889	−2.797825
755	ANKRD31	0.09517431	0.084449322	4.454233	1.443474e−05	0.0005073954	2.783531	NACAP1	0.32794595	1.201920513	3.604598	0.0004004441	0.005877978	−0.2987464	MX2	−0.49770063	2.516426470	−3.108994	0.002169446	0.02012406	−1.835720	ARHGAP44	0.40399630	1.848163952	2.758522	0.006380599	0.04326385	−2.798970
756	IL29	0.27832129	0.201917131	4.453289	1.449224e−05	0.0005087427	2.779812	PLCB1	−0.57385737	1.942745060	−3.604147	0.0004010947	0.005884274	−0.3002357	XYLT1	−0.38423226	2.099824920	−3.108883	0.002170220	0.02012421	−1.836041	TNFAIP8L1	−0.30712521	3.310196478	−2.758310	0.006384579	0.04326954	−2.799522
757	MUC20	−1.38934946	2.828084538	−4.452564	1.453658e−05	0.0005096251	2.776954	CFI	0.67957582	3.285773588	3.603793	0.0004016058	0.005887750	−0.3014040	BC039678	−0.14899648	0.262948089	−3.108576	0.002172368	0.02013709	−1.836932	SNAR−B2	0.01659454	0.007522181	2.758304	0.006384699	0.04326954	−2.799538
758	EIF3M	0.34733515	6.625364864	4.449810	1.470616e−05	0.0005143807	2.766103	MMAA	0.24984297	1.626982451	3.603676	0.0004017753	0.005887750	−0.3017911	ANKFN1	0.17499370	0.188981187	3.108210	0.002174925	0.02015375	−1.837991	OTX1	0.40938581	1.685724428	2.758126	0.006388028	0.04328105	−2.799999
759	KBTBD11	−0.77490904	1.926451035	−4.449732	1.471099e−05	0.0005143807	2.765796	SIGLEC12	0.52597393	1.082658503	3.601922	0.0004043186	0.005921750	−0.3075796	TUBBP5	−0.37647171	0.671070722	−3.108049	0.002176049	0.02015690	−1.838457	LOC100506013	−0.11426788	0.159868600	−2.757940	0.006391532	0.04329374	−2.800484
760	PLA1A	0.89753014	2.255241270	4.448472	1.478924e−05	0.0005164365	2.760833	HIST1H2AL	0.33814266	0.620036117	3.601586	0.0004048074	0.005925640	−0.3086880	ERGIC1	−0.32908771	4.684583905	−3.107944	0.002176785	0.02015690	−1.838761	IQCF1	0.01315618	0.019550175	2.757045	0.006408361	0.04338875	−2.802809
761	AK090553	0.18784815	0.355596119	4.443632	1.509352e−05	0.0005263693	2.741782	JA040725	−0.33647843	13.072254787	−3.601126	0.0004054774	0.005929361	−0.3102049	TYMP	0.64729443	5.728002710	3.107799	0.002177805	0.02015932	−1.839183	L1CAM	−0.79017069	1.671330162	−2.757020	0.006408829	0.04338875	−2.802874
762	GTF2B	0.29325693	4.013006155	4.442832	1.514435e−05	0.0005274488	2.738637	SAMD10	0.53996855	2.971685353	3.601105	0.0004055085	0.005929361	−0.3102752	CTAGE1	0.02414127	0.063051875	3.107540	0.002179619	0.02016145	−1.839933	TNNI3	−0.52509724	0.958556382	−2.756851	0.006411999	0.04339914	−2.803311
763	FAM84A	−1.31185008	2.762533861	−4.442179	1.518603e−05	0.0005278018	2.736066	DEF6	0.52585452	3.175722876	3.600538	0.0004063359	0.005938187	−0.3121449	PDCD11	0.29273667	3.325592772	3.107468	0.002180119	0.02016145	−1.840139	CERS4	−0.66759312	2.502422903	−2.756641	0.006415956	0.04341486	−2.803856
764	BC041855	0.03040566	0.013648598	4.442050	1.519426e−05	0.0005278018	2.735559	PLEKHG5	−0.41853734	2.225672909	−3.600265	0.0004067356	0.005940272	−0.3130467	LOC100329135	0.01689305	0.016727433	3.107441	0.002180314	0.02016145	−1.840220	BFSP1	0.23324615	0.656793199	2.755867	0.006430564	0.04350261	−2.805867
765	IL18	0.88005633	3.486903289	4.441425	1.523426e−05	0.0005284995	2.733100	MATK	0.36166904	0.978655424	3.600135	0.0004069262	0.005940272	−0.3134764	PXDN	−0.54505970	3.634956501	−3.107175	0.002182178	0.02017095	−1.840989	PDIA2	−0.77347370	1.467586847	−2.755778	0.006432251	0.04350293	−2.806099
766	LOC654342	−0.65277412	2.620636632	−4.439558	1.535428e−05	0.0005319678	2.725760	ATXN7L3B	0.34980352	5.277094648	3.599690	0.0004075776	0.005946510	−0.3149435	ECE1	−0.35807745	4.824306022	−3.107077	0.002182862	0.02017095	−1.841271	SSX8	0.03361166	0.011038938	2.755653	0.006434603	0.04350775	−2.806422
767	LINC00426	0.43078464	0.645481663	4.439185	1.537838e−05	0.0005321081	2.724293	LINC00158	0.05773177	0.061740954	3.598847	0.0004088137	0.005961268	−0.3177212	AX747031	0.01297536	0.009422326	3.106636	0.002185963	0.02019258	−1.842550	PRRT3	−0.19306800	1.088920653	−2.755370	0.006439961	0.04353289	−2.807158
768	PLA2R1	−0.48614227	1.116801191	−4.438045	1.545223e−05	0.0005339672	2.719812	WDR26	−0.26210237	3.519901734	−3.598528	0.0004092833	0.005961789	−0.3187742	BC073918	0.23338714	0.846030778	3.105981	0.002190573	0.02022812	−1.844447	BRCA2	0.28949835	1.272384922	2.755172	0.006443703	0.04354368	−2.807671
769	Y00482	0.37605993	0.549452553	4.436742	1.553704e−05	0.0005361995	2.714693	SRSF7	0.21806915	4.968726101	3.598381	0.0004094990	0.005961789	−0.3192575	KLRF1	0.12632336	0.274363715	3.105513	0.002193868	0.02025150	−1.845800	NECAB2	−0.06906035	0.114214187	−2.755112	0.006444839	0.04354368	−2.807827
770	C11orf40	0.02712566	0.007622428	4.435712	1.560443e−05	0.0005378261	2.710644	NPTXR	−0.66965349	1.518290115	−3.598365	0.0004095234	0.005961789	−0.3193120	GALNT3	−0.60706229	4.245475203	−3.104356	0.002202037	0.02031776	−1.849147	KIAA1324L	−0.33996218	0.887390941	−2.754959	0.006447734	0.04355215	−2.808224
771	DTHD1	0.19228844	0.219935862	4.435258	1.563421e−05	0.0005381535	2.708861	OSR1	−0.61411918	1.475623529	−3.598053	0.0004099834	0.005965214	−0.3203417	MIR212	0.07894654	0.081518124	3.104280	0.002202578	0.02031776	−1.849368	WAS	0.56355464	3.074495432	2.754823	0.006450295	0.04355417	−2.808575
772	ARRDC4	−0.64049497	2.911402098	−4.434866	1.565995e−05	0.0005383411	2.707323	CROCCP3	−0.18776454	0.668745497	−3.597440	0.0004108877	0.005975095	−0.3223625	PSMD4	−0.21180796	6.197971526	−3.103408	0.002208754	0.02036766	−1.851889	LOC285419	0.09617878	0.184496192	2.754770	0.006451315	0.04355417	−2.808715
773	LIG1	0.58032072	3.231606564	4.434033	1.571486e−05	0.0005395300	2.704049	LYSMD2	0.35587649	3.154552488	3.595926	0.0004131288	0.006004395	−0.3273513	DNALI1	−0.57665348	2.000659736	−3.101347	0.002223426	0.02049583	−1.857850	ATXN7L3	0.18954325	4.312823460	2.754526	0.006455929	0.04357017	−2.809347
774	CD3D	1.10574847	3.258144230	4.433113	1.577571e−05	0.0005409193	2.700435	DEAF1	0.30756740	3.389926189	3.595086	0.0004143761	0.006019227	−0.3301161	MAP2K4P1	0.03310452	0.063634220	3.101151	0.002224826	0.02050162	−1.858416	CDC37	0.21391891	6.502715639	2.754400	0.006458318	0.04357017	−2.809674
775	MPHOSPH6	0.47107519	3.991018246	4.432720	1.580172e−05	0.0005411119	2.698894	43160	0.42947732	1.560165528	3.594020	0.0004159666	0.006037833	−0.3336295	RASGRP3	0.29338178	1.588999555	3.100047	0.002232723	0.02056725	−1.861606	LINC00311	−0.05114314	0.090866591	−2.754344	0.006459378	0.04357017	−2.809819
776	CERCAM	−0.72646918	3.298244463	−4.428821	1.606248e−05	0.0005493328	2.683587	FRAT2	0.43157428	3.769111835	3.593922	0.0004161120	0.006037833	−0.3339500	C15orf56	−0.11073534	0.225118468	−3.099739	0.002234933	0.02058046	−1.862497	POLA1	0.29877629	2.356547871	2.754257	0.006461026	0.04357017	−2.810045
777	43161	−0.65265680	3.104332914	−4.422684	1.648125e−05	0.0005629291	2.659519	RPL23AP53	0.26954241	1.074729449	3.593596	0.0004166001	0.006041613	−0.3350252	D28359	−0.11010917	0.287871582	−3.099568	0.002236160	0.02058462	−1.862991	CNOT2	0.21882350	3.520941926	2.754211	0.006461893	0.04357017	−2.810163
778	PPP2R5B	−0.35685262	2.976838438	−4.421702	1.654921e−05	0.0005645239	2.655671	SIGLEC1	0.64255678	1.658390963	3.593319	0.0004170141	0.006044313	−0.3359360	OGFOD2	0.20652483	1.846685153	3.099285	0.002238194	0.02059358	−1.863809	TRBV5−5	0.21317978	0.398137848	2.753656	0.006472436	0.04363017	−2.811604
779	PANK2	0.34689488	3.117736618	4.420716	1.661772e−05	0.0005661331	2.651808	DDX20	0.20255560	2.064685903	3.593021	0.0004174606	0.006047482	−0.3369174	AKR1C4	−0.62455121	0.962258361	−3.099216	0.002238686	0.02059358	−1.864007	GRHL1	0.44046665	1.627859822	2.753537	0.006474686	0.04363425	−2.811912
780	LZIC	−0.33915077	3.733432700	−4.419885	1.667572e−05	0.0005673806	2.648551	NEK3	−0.49012490	1.935559486	−3.592561	0.0004181505	0.006054172	−0.3384317	ZNF815P	0.15977924	0.754657649	3.098803	0.002241654	0.02061374	−1.865200	METAP1	0.24157301	3.047364272	2.752990	0.006485100	0.04369334	−2.813332
781	QSER1	0.45103295	2.553759084	4.419267	1.671890e−05	0.0005681216	2.646132	DOCK10	0.45003361	1.392498565	3.591601	0.0004195954	0.006071778	−0.3415948	LTB4R	0.27589923	1.146541690	3.098383	0.002244678	0.02063440	−1.866413	BCAN	−0.37333027	1.273341229	−2.752696	0.006490684	0.04371987	−2.814093
782	SLC4A3	−0.61434163	0.932005042	−4.417628	1.683412e−05	0.0005713053	2.639711	LINC00346	−0.35040490	0.700981719	−3.591323	0.0004200140	0.006074524	−0.3425092	TPRG1	0.31994280	0.468399209	3.098103	0.002246695	0.02064091	−1.867221	SF1	0.17199242	5.679029140	2.752460	0.006495192	0.04373913	−2.814707
783	RNF144A	−0.54069811	1.841986949	−4.417047	1.687513e−05	0.0005719656	2.637436	VPS13C	0.30390854	2.595722927	3.589187	0.0004232450	0.006117919	−0.3495361	LOC100507410	−0.20150831	0.231813924	−3.098069	0.002246942	0.02064091	−1.867320	NSA2	0.27722219	5.927028841	2.751952	0.006504874	0.04376142	−2.816023
784	AIFM2	−0.54169707	2.708187688	−4.412644	1.718905e−05	0.0005818624	2.620204	DMXL2	0.31642616	1.873250981	3.588666	0.0004240372	0.006126034	−0.3512509	EPHX4	−0.55042647	1.141033153	−3.097476	0.002251218	0.02067304	−1.869032	MIR2276	0.08203342	0.089891368	2.751945	0.006505005	0.04376142	−2.816041
785	CLUAP1	0.33345643	1.783356159	4.411527	1.726954e−05	0.0005838426	2.615836	DDO	0.38352868	0.759207081	3.588132	0.0004248493	0.006133715	−0.3530051	LOC284576	0.09335014	0.082777159	3.096800	0.002256105	0.02071075	−1.870984	ABCE1	0.24154627	4.147498966	2.751943	0.006505037	0.04376142	−2.816046
786	BC070093	0.11184635	0.294287096	4.410879	1.731644e−05	0.0005846832	2.613300	TMEM109	0.31455256	5.547043841	3.588013	0.0004250312	0.006133715	−0.3533977	AHCY	0.38128843	6.010817956	3.096433	0.002258768	0.02072802	−1.872045	PTPRC	0.70352948	3.130096328	2.751940	0.006505099	0.04376142	−2.816054
787	CLP1	0.26883922	2.697713577	4.408206	1.751112e−05	0.0005905053	2.602849	ARNT	−0.25689054	3.522957068	−3.587275	0.0004261583	0.006145905	−0.3558259	C10orf136	−0.04899094	0.064469294	−3.096262	0.002260003	0.02073218	−1.872537	LOC339290	−0.30510030	2.307394988	−2.751581	0.006511947	0.04379639	−2.816984
788	LOC100286979	0.11690216	0.134157818	4.406468	1.763884e−05	0.0005940572	2.596055	CYP2R1	−0.33096629	1.956076837	−3.587156	0.0004263390	0.006145905	−0.3562146	PAXIP1	0.26247424	2.722155265	3.095733	0.002263841	0.02076022	−1.874064	43351	0.20796367	3.627455610	2.751456	0.006514343	0.04380141	−2.817309
789	ABCD1	−0.63344849	3.684926083	−4.405917	1.767951e−05	0.0005946725	2.593902	LSM6	0.31227260	3.341019021	3.586901	0.0004267296	0.006148196	−0.3570543	SCG5	−0.54930953	1.810070843	−3.094649	0.002271723	0.02082531	−1.877193	RNASE6	0.57395549	3.767660776	2.751042	0.006522261	0.04384354	−2.818383
790	GCA	0.42922003	2.992313149	4.404252	1.780296e−05	0.0005980667	2.587398	HPS1	−0.30897594	4.308407746	−3.585962	0.0004281679	0.006165571	−0.3601393	FKBP9	−0.30142937	4.906484613	−3.094527	0.002272611	0.02082625	−1.877544	EARS2	0.24154740	2.863311888	2.750595	0.006530811	0.04388989	−2.819541
791	ICOS	0.52441802	0.873642899	4.403897	1.782939e−05	0.0005981976	2.586011	FBXO5	0.41754859	2.537280643	3.584878	0.0004298352	0.006186223	−0.3637026	MYO3B	0.22161662	0.156790349	3.094090	0.002275798	0.02084825	−1.878805	COL6A3	−0.60209297	5.545849335	−2.749800	0.006546047	0.04398115	−2.821600
792	UNQ6494	0.08561678	0.105711569	4.403544	1.785570e−05	0.0005983237	2.584633	C6orf195	−0.03401776	0.050187006	−3.583797	0.0004315042	0.006206878	−0.3672555	DENR	0.24099093	4.389818825	3.093890	0.002277255	0.02085440	−1.879381	DPH1	0.19218900	3.942168156	2.749606	0.006549780	0.04399509	−2.822104
793	DQ575742	−0.48648277	1.354762952	−4.398904	1.820510e−05	0.0006092624	2.566519	DENND2D	0.47259652	4.279725491	3.583320	0.0004322425	0.006214129	−0.3688227	CAPS	−0.88330288	2.791970394	−3.093403	0.002280810	0.02087976	−1.880785	SAR1A	−0.21043732	4.588068446	−2.749434	0.006553064	0.04399771	−2.822547
794	SLC36A4	−0.56302258	1.865525184	−4.396209	1.841106e−05	0.0006153791	2.556004	PDZRN3	−0.82742134	2.525726843	−3.582980	0.0004327688	0.006218328	−0.3699385	CSTF2	0.35184200	3.443597834	3.093159	0.002282597	0.02088626	−1.881490	MMP24	−0.36074422	1.975915635	−2.749375	0.006554199	0.04399771	−2.822700
795	RCN1	0.61353285	4.449199149	4.392217	1.872029e−05	0.0006242802	2.540436	GPX5	0.03274550	0.008892158	3.582006	0.0004342821	0.006236694	−0.3731384	CENPN	0.39478819	2.849217010	3.093091	0.002283094	0.02088626	−1.881686	AURKC	0.19999203	0.526370806	2.749326	0.006555143	0.04399771	−2.822827
796	CPQ	−0.69754592	3.615449949	−4.392164	1.872441e−05	0.0006242802	2.540231	ZNF80	0.14990837	0.186969888	3.581060	0.0004357556	0.006254471	−0.3762436	TMEM2	−0.43983951	4.211082977	−3.092925	0.002284307	0.02089015	−1.882164	CRISP1	0.01259618	0.007414237	2.749140	0.006558731	0.04401066	−2.823311
797	TMSB15B	0.29197300	0.584384861	4.391027	1.881338e−05	0.0006264595	2.535801	PI4K2B	0.34522445	3.047775462	3.580530	0.0004365843	0.006262979	−0.3779854	CD52	0.88659134	4.316858885	3.092421	0.002288001	0.02091673	−1.883618	KDELR3	−0.50346958	4.946379848	−2.748924	0.006562874	0.04402134	−2.823869
798	ALG1L	−0.59489020	1.563823489	−4.389831	1.890746e−05	0.0006277510	2.531139	PRKD2	0.27998760	4.281536061	3.579956	0.0004373421	0.006270186	−0.3798684	C3orf70	−0.63157361	1.583778953	−3.092297	0.002288908	0.02091782	−1.883975	ELMOD2	−0.18332967	2.542837267	−2.748884	0.006563640	0.04402134	−2.823972
799	C6orf211	0.33957841	3.327174475	4.389653	1.892152e−05	0.0006277510	2.530444	MR1	0.35185544	1.922016049	3.579753	0.0004378002	0.006270186	−0.3805348	MAST4	0.32100664	2.115077174	3.092104	0.002290326	0.02092568	−1.884532	AK298596	0.32163218	1.669771165	2.748729	0.006566624	0.04403022	−2.824374
800	SHISA4	−0.69367850	2.539507474	−4.389550	1.892958e−05	0.0006277510	2.530046	XRCC1	0.27057914	4.201164092	3.579522	0.0004381624	0.006270186	−0.3812928	AK055408	0.22861265	0.719237463	3.091588	0.002294115	0.02095097	−1.886019	PPP1R16B	0.50595492	1.868813884	2.748512	0.006570801	0.04404711	−2.824936

	Gene	logFC	AveExpr	t	P.Value	adj.P.Val	B	Gene	logFC	AveExpr	t	P.Value	adj.P.Val	B	Gene	logFC	AveExpr	t	P.Value	adj.P.Val	B	Gene	logFC	AveExpr	t	P.Value	adj.P.Val	B
801	HLA−DRA	1.22831981	8.891520613	4.389333	1.894678e−05	0.0006277510	2.529198	TNFRSF1B	0.601607389	4.348164682	3.579464	0.0004382538	0.006270186	−0.3814840	SNRPD2	0.30063450	6.957499840	3.091264	0.002296497	0.02096148	−1.886953	C17orf53	0.32839784	1.709302329	2.748213	0.006576564	0.04407460	−2.825710
802	HIST2H2AB	0.33460657	0.563952485	4.388497	1.901290e−05	0.0006284193	2.525942	ARNTL2	0.568202390	2.716023824	3.579455	0.0004382680	0.006270186	−0.3815138	AFG3L1P	0.22376278	1.564593473	3.091216	0.002296846	0.02096148	−1.887090	ERCC8	0.15484791	1.291491510	2.747896	0.006582676	0.04410443	−2.826531
803	HNRNPU−AS1	−0.43822158	3.662954123	−4.388479	1.901431e−05	0.0006284193	2.525873	FAM175A	0.251461196	2.381648816	3.578747	0.0004393799	0.006282706	−0.3838362	FAM132B	−0.35088417	0.728790628	−3.090951	0.002298802	0.02097212	−1.887856	AK125726	−0.25175187	0.534214768	−2.747454	0.006591197	0.04415037	−2.827674
804	AK025726	−0.39683749	1.372408335	−4.388041	1.904904e−05	0.0006287842	2.524167	DCUN1D5	0.234346002	1.503778516	3.578550	0.0004396896	0.006283750	−0.3844823	STK32C	−0.44136458	1.724841772	−3.090691	0.002300715	0.02098236	−1.888604	BC051708	0.14343356	0.276152303	2.747264	0.006594876	0.04416387	−2.828167
805	ACAA2	−0.86778176	5.106966727	−4.387415	1.909882e−05	0.0006296443	2.521728	BC038442	0.237104936	0.888372229	3.578382	0.0004399546	0.006284152	−0.3850344	M55536	−0.29949933	3.271163034	−3.090481	0.002302265	0.02098929	−1.889211	CCDC74B	−0.17840926	0.422510688	−2.746989	0.006600189	0.04418830	−2.828879
806	SNRPF	0.48771272	5.885723143	4.386717	1.915447e−05	0.0006306953	2.519009	H2AFZ	0.396584609	6.039523228	3.577699	0.0004410309	0.006296138	−0.3872742	RNPS1	0.20752780	5.560545668	3.090373	0.002303056	0.02098929	−1.889520	NUDT12	0.37532919	2.126262778	2.746522	0.006609228	0.04423765	−2.830088
807	DQ571064	−0.39317096	0.777986585	−4.385757	1.923123e−05	0.0006324382	2.515272	NR1H3	0.401471328	3.147408558	3.577542	0.0004412792	0.006296294	−0.3877900	OSBPL10	0.29522485	2.339805954	3.089666	0.002308280	0.02102968	−1.891558	EFCAB4B	−0.41265018	1.498990797	−2.746281	0.006613880	0.04424861	−2.830710
808	IL21R	0.65384167	1.185087577	4.384251	1.935221e−05	0.0006355899	2.509412	FBL	0.417461503	5.976582129	3.576926	0.0004422519	0.006306783	−0.3898083	DHRS2	0.70173095	0.914833654	3.089366	0.002310495	0.02103398	−1.892421	PSME3	0.21611361	5.260242462	2.746208	0.006615302	0.04424861	−2.830900
809	CTRL	0.25325022	1.025416762	4.383969	1.937497e−05	0.0006355899	2.508314	ATAD5	0.316749176	1.428303419	3.575111	0.0004451339	0.006344473	−0.3957619	ECT2	0.56037136	4.100641314	3.089215	0.002311611	0.02103398	−1.892856	KLC4	−0.33981574	3.039844718	−2.746179	0.006615866	0.04424861	−2.830975
810	FAM122C	0.32726240	0.972001349	4.383493	1.941342e−05	0.0006360652	2.506461	PCNXL3	0.308409990	4.039971283	3.574933	0.0004454169	0.006345099	−0.3963443	FAM171B	−0.31050727	1.178376309	−3.089177	0.002311892	0.02103398	−1.892965	PRR11	0.38668844	2.869606237	2.745977	0.006619781	0.04426363	−2.831498
811	GSTTP1	0.06182130	0.029499505	4.383057	1.944874e−05	0.0006364367	2.504762	ZNF844	−0.476055382	1.530878120	−3.573907	0.0004470548	0.006365015	−0.3997084	HCK	0.52007884	2.998026897	3.089173	0.002311923	0.02103398	−1.892977	DLX6	−0.34834427	0.446432777	−2.745801	0.006623187	0.04427345	−2.831952
812	VSTM5	0.44838406	0.736171780	4.380236	1.967849e−05	0.0006431617	2.493789	ADAP2	0.445681484	2.619281506	3.572461	0.0004493718	0.006394573	−0.4044462	MUC16	0.40506620	0.306164398	3.088718	0.002315293	0.02105742	−1.894288	TMPRSS7	0.05900654	0.098524365	2.745729	0.006624586	0.04427345	−2.832139
813	NME8	0.06741175	0.110538480	4.379914	1.970489e−05	0.0006432324	2.492537	KIAA1586	−0.363941522	1.397115377	−3.572290	0.0004496455	0.006395039	−0.4050042	PIGL	0.27790053	1.797089623	3.088405	0.002317618	0.02107135	−1.895191	LOC100507341	0.05632717	0.104649692	2.744660	0.006645351	0.04439517	−2.834904
814	EMP2	−0.67678394	4.525053329	−4.378093	1.985473e−05	0.0006469563	2.485458	ZNF543	0.282552900	1.601812452	3.571878	0.0004503086	0.006398069	−0.4063546	BC043280	0.00744190	0.003376363	3.088213	0.002319042	0.02107707	−1.895743	AK097101	0.01180509	0.013311671	2.744619	0.006646144	0.04439517	−2.835009
815	MOV10	0.45662575	3.969089086	4.377796	1.987929e−05	0.0006469563	2.484303	PATL1	0.288572759	4.526201885	3.571816	0.0004504094	0.006398069	−0.4065597	VPS45	−0.21471829	3.237217859	−3.087056	0.002327640	0.02114798	−1.899073	CSTF3	0.17970460	3.211732367	2.744305	0.006652259	0.04442484	−2.835822
816	BIRC3	1.01479785	3.803406707	4.377641	1.989210e−05	0.0006469563	2.483701	MSRA	−0.548749354	2.545417099	−3.571708	0.0004505818	0.006398069	−0.4069106	OAS2	0.68189550	4.090976961	3.086240	0.002333726	0.02119179	−1.901422	SNAI3	0.28174268	1.153414271	2.744141	0.006655459	0.04443502	−2.836247
817	SYNJ1	0.24835699	1.706548822	4.375906	2.003624e−05	0.0006508468	2.476955	DAOA	0.016282080	0.006328578	3.570893	0.0004518971	0.006413313	−0.4095817	TEKT4P2	−0.28328101	0.705891885	−3.086095	0.002334811	0.02119179	−1.901841	ITGB2	0.66235791	4.398013949	2.743830	0.006661520	0.04444896	−2.837051
818	ERVMER34−1	−0.78976210	1.000159126	−4.373389	2.024698e−05	0.0006568883	2.467180	AK123834	0.032722420	0.054484718	3.570601	0.0004523685	0.006416572	−0.4105371	GBP2	0.54963463	4.450351966	3.086047	0.002335170	0.02119179	−1.901979	NT5E	−0.64082453	3.498914948	−2.743793	0.006662236	0.04444896	−2.837146
819	VWA2	−1.00073941	1.558472245	−4.373092	2.027206e−05	0.0006568989	2.466024	PPP1R9A	−0.572914917	1.213290350	−3.570132	0.0004531271	0.006423899	−0.4120726	LOC729059	0.06096403	0.077055720	3.085982	0.002335656	0.02119179	−1.902166	FLJ20021	−0.33247844	2.582070799	−2.743769	0.006662699	0.04444896	−2.837207
820	CD80	0.37542897	0.677011379	4.372735	2.030211e−05	0.0006570704	2.464640	LCA5	0.257986389	1.049095023	3.569501	0.0004541508	0.006434975	−0.4141407	BC114339	0.35903478	3.758752677	3.085755	0.002337345	0.02119987	−1.902817	KRT20	−1.52027738	3.039881506	−2.743690	0.006664246	0.04444896	−2.837412
821	AX746880	−0.53411527	0.644370606	−4.372128	2.035343e−05	0.0006577038	2.462281	HK3	0.412775516	0.931958015	3.566468	0.0004590959	0.006501470	−0.4240643	CLU	−1.14574506	5.775721582	−3.085389	0.002340087	0.02121748	−1.903871	TRMT11	0.23160389	2.728652226	2.743326	0.006671351	0.04447853	−2.838354
822	ASCC3	0.41256654	2.714215413	4.371918	2.037125e−05	0.0006577038	2.461464	STMN3	−0.798582705	3.161255486	−3.566323	0.0004593336	0.006501470	−0.4245387	CNPY4	−0.28451994	2.410364825	−3.085254	0.002341094	0.02121936	−1.904258	HLA−F	0.54916528	4.754980173	2.743291	0.006672032	0.04447853	−2.838444
823	ITGAD	0.17697408	0.150758306	4.371363	2.041823e−05	0.0006584198	2.459311	RELL1	0.335823333	3.271203019	3.565771	0.0004602400	0.006510826	−0.4263449	MEF2D	−0.31535954	4.126187375	−3.084950	0.002343377	0.02123281	−1.905135	GNE	−0.54939932	2.856558308	−2.742851	0.006680634	0.04451077	−2.839582
824	AGFG2	0.63301734	3.048360088	4.370707	2.047396e−05	0.0006594155	2.456765	ENC1	0.636561528	4.527692442	3.565479	0.0004607196	0.006514138	−0.4272993	MYRIP	−0.32385165	0.718046759	−3.084716	0.002345128	0.02123621	−1.905807	TFCP2L1	−0.57968107	1.690528608	−2.742747	0.006682656	0.04451077	−2.839849
825	DOK2	0.68917136	2.467011117	4.370391	2.050088e−05	0.0006594690	2.455537	LCE1F	0.041903340	0.010473365	3.564752	0.0004619178	0.006527602	−0.4296790	KCNMB4	−0.36013921	0.835164747	−3.084686	0.002345353	0.02123621	−1.905893	SLC5A1	−0.81347422	2.837033103	−2.742640	0.006684760	0.04451077	−2.840127
826	Mir_207	0.12468235	0.082778835	4.370104	2.052532e−05	0.0006594690	2.454425	MIB1	−0.346944184	2.731499083	−3.563685	0.0004636800	0.006549017	−0.4331677	DRAM1	0.43926092	3.357908315	3.084340	0.002347947	0.02124629	−1.906888	UAP1L1	−0.42458462	2.665936904	−2.742620	0.006685135	0.04451077	−2.840177
827	TRAPPC9	−0.38304067	3.629430674	−4.369409	2.058474e−05	0.0006605786	2.451724	SNORD114−20	0.055309541	0.011781981	3.562796	0.0004651524	0.006566319	−0.4360726	BRWD3	0.22637881	1.763197803	3.084324	0.002348067	0.02124629	−1.906934	IGKV	0.73078179	1.337936109	2.742614	0.006685253	0.04451077	−2.840193
828	TRBV4−1	0.66286728	0.930315949	4.368689	2.064641e−05	0.0006617574	2.448929	PITRM1	−0.295573518	4.041296281	−3.561805	0.0004668002	0.006586077	−0.4393123	RMND5A	−0.29456605	3.305852737	−3.084050	0.002350129	0.02125769	−1.907723	PLLP	0.71750190	4.593371780	2.742143	0.006694489	0.04455907	−2.841412
829	ZNF514	−0.40821158	1.635122415	−4.367172	2.077688e−05	0.0006644305	2.443045	CLEC2D	0.524466952	1.238366476	3.561344	0.0004675681	0.006593405	−0.4408181	ZC3H7B	0.22937297	3.693737853	3.083305	0.002355732	0.02129757	−1.909865	C2orf15	0.34299513	1.860257633	2.742072	0.006695865	0.04455907	−2.841594
830	HAVCR2	0.67914625	2.344106059	4.367137	2.077988e−05	0.0006644305	2.442910	GPRIN1	−0.553720989	1.692115682	−3.561063	0.0004680358	0.006595547	−0.4417340	AK127609	0.16714355	0.696839110	3.083251	0.002356142	0.02129757	−1.910022	ORM1	−0.76851407	1.098855853	−2.741842	0.006700383	0.04457622	−2.842190
831	CEP135	0.22660328	1.209444310	4.361381	2.128238e−05	0.0006796788	2.420590	MMP2	−0.824212314	6.244193133	−3.560955	0.0004682170	0.006595547	−0.4420887	BMS1P5	0.02944347	0.079907886	3.083054	0.002357625	0.02130371	−1.910588	LHX8	0.07941816	0.025543125	2.741769	0.006701802	0.04457622	−2.842377
832	MCM3	0.55069383	5.290558141	4.360175	2.138917e−05	0.0006822682	2.415915	AX747192	−0.284397853	3.247394743	−3.560721	0.0004686085	0.006597561	−0.4428545	C7orf63	0.20564021	0.778204981	3.082461	0.002362095	0.02133684	−1.912292	SNORA76	0.41189874	1.285622911	2.741459	0.006707894	0.04459690	−2.843179
833	ANKMY1	0.28706240	1.465123807	4.358411	2.154619e−05	0.0006854717	2.409083	MCM7	0.550542592	5.225620841	3.559925	0.0004699390	0.006612784	−0.4454517	C9orf64	0.29288651	3.156354198	3.082198	0.002364081	0.02134751	−1.913047	SPTB	−0.20166912	0.534162922	−2.741439	0.006708271	0.04459690	−2.843229
834	AK094188	−0.32585005	0.745575681	−4.357817	2.159932e−05	0.0006854717	2.406783	OGDH	−0.331996139	5.317854933	−3.559053	0.0004714029	0.006629869	−0.4483011	KLHL24	−0.32369525	3.119118898	−3.081816	0.002366967	0.02136630	−1.914145	DQ266889	−0.03541772	0.038727084	−2.741319	0.006710636	0.04460145	−2.843540
835	MIR3653	0.43713695	1.151438752	4.357765	2.160403e−05	0.0006854717	2.406579	SAMSN1	0.610087030	2.469585682	3.557273	0.0004744033	0.006666920	−0.4541131	ALDH1B1	−0.61924619	4.656120735	−3.081564	0.002368876	0.02137626	−1.914870	SAAL1	0.27011705	2.884942881	2.740705	0.006722693	0.04465828	−2.845126
836	ZNRF1	0.37963101	2.993390158	4.357671	2.161242e−05	0.0006854717	2.406216	C11orf35	0.409204224	1.517036775	3.557192	0.0004745398	0.006666920	−0.4543765	GRINA	−0.37154320	6.619637751	−3.081074	0.002372588	0.02140248	−1.916278	AK097866	0.02554849	0.030339237	2.740689	0.006723004	0.04465828	−2.845167
837	ZNF185	−0.85200587	2.520437184	−4.357601	2.161874e−05	0.0006854717	2.405943	FCER1G	0.709613559	6.371021787	3.557024	0.0004748244	0.006667389	−0.4549258	LOC100233209	0.42688276	1.113893290	3.080257	0.002378790	0.02145114	−1.918626	PTGER1	−0.49816394	1.438672768	−2.740627	0.006724235	0.04465828	−2.845328
838	C16orf5	−0.65806797	3.002151053	−4.356137	2.175029e−05	0.0006880244	2.400278	OR8S1	0.035511041	0.016137719	3.554827	0.0004785539	0.006716204	−0.4620923	OR9Q1	0.02447746	0.014423565	3.079976	0.002380927	0.02146312	−1.919434	ZSWIM4	−0.29724006	2.863505215	−2.740433	0.006728051	0.04467244	−2.845830
839	TCR−alpha	0.41796948	0.720043652	4.356128	2.175110e−05	0.0006880244	2.400243	ARMCX3	−0.536839612	3.480419276	−3.553430	0.0004809418	0.006746150	−0.4666513	GGT1	−0.81186165	2.502718200	−3.079501	0.002384536	0.02147953	−1.920796	OTOL1	0.01212679	0.007928362	2.740267	0.006731311	0.04468291	−2.846257
840	CCL1	0.17320579	0.122572055	4.354863	2.186546e−05	0.0006894272	2.395345	PF4	−0.729458163	1.016799132	−3.552168	0.0004831060	0.006772927	−0.4707634	TNS4	1.43526281	3.666055060	3.079438	0.002385023	0.02147953	−1.920980	MRGPRF	−0.80179030	3.210745996	−2.740082	0.006734956	0.04469593	−2.846736
841	FDPSP2	0.18024073	0.342985702	4.354816	2.186972e−05	0.0006894272	2.395163	MOB1B	−0.292794552	1.949162332	−3.551837	0.0004836766	0.006777347	−0.4718446	NGFRAP1	−0.75426845	4.294341047	−3.079417	0.002385176	0.02147953	−1.921037	IGKV1−12	0.07709932	0.106041757	2.739890	0.006738723	0.04470175	−2.847229
842	AMICA1	0.53538511	1.599372108	4.354776	2.187338e−05	0.0006894272	2.395007	MIR1278	0.066825211	0.033492787	3.551453	0.0004843383	0.006783037	−0.4730966	ULBP2	−0.49970210	1.067727192	−3.079128	0.002387378	0.02149207	−1.921867	MIR1911	0.01614069	0.002502480	2.739866	0.006739202	0.04470175	−2.847292
843	E2F7	0.61442310	1.377548869	4.352897	2.204434e−05	0.0006939915	2.387736	TMEM225	0.043248171	0.026128106	3.551042	0.0004850473	0.006788944	−0.4744362	NEU4	−0.43864230	0.625295479	−3.078158	0.002394788	0.02155147	−1.924654	PGM2	0.24646873	3.389280576	2.739584	0.006744759	0.04472742	−2.848020
844	HLA−DPB1	1.09757574	5.245336978	4.351506	2.217178e−05	0.0006971765	2.382352	C12orf45	0.367054711	3.857257397	3.550912	0.0004852718	0.006788944	−0.4748600	C5orf58	0.31900842	1.035152986	3.077596	0.002399084	0.02158281	−1.926266	HNRNPL	0.16813289	5.735952511	2.739167	0.006752990	0.04476416	−2.849097
845	MAPK15	−0.66318633	1.155133808	−4.349029	2.240037e−05	0.0007035307	2.372773	LOC339529	0.022308408	0.017971243	3.549720	0.0004873329	0.006814187	−0.4787416	ASPHD2	0.48425853	2.115677164	3.077385	0.002400707	0.02159009	−1.926874	DQ597063	0.01587793	0.003710987	2.739120	0.006753918	0.04476416	−2.849219
846	FAM117B	0.43286448	2.318647200	4.348255	2.247228e−05	0.0007049550	2.369780	KCNA3	0.524310443	1.032580762	3.548951	0.0004886679	0.006829256	−0.4812469	SNORD8	0.16606804	0.323818583	3.076912	0.002404333	0.02160742	−1.928232	NUAK2	−0.47562170	2.526339309	−2.739047	0.006755359	0.04476416	−2.849407
847	AX747795	0.19565872	0.544592558	4.347312	2.256014e−05	0.0007067912	2.366137	BIN3	0.339985666	3.572302492	3.548746	0.0004890252	0.006830653	−0.4819163	FXYD5	0.56540520	5.026211375	3.076814	0.002405081	0.02160742	−1.928511	C11orf92	0.45274176	1.414245233	2.738936	0.006757544	0.04476747	−2.849693
848	FAM109B	−0.41466833	3.063145813	−4.347056	2.258408e−05	0.0007067912	2.365146	CEP41	0.321219918	1.449527727	3.547992	0.0004903383	0.006845391	−0.4843719	MEIS3	−0.30571583	1.287815829	−3.076712	0.002405866	0.02160742	−1.928805	COX6B2	−0.32955270	0.548938207	−2.738352	0.006769090	0.04483277	−2.851200
849	ACHE	−1.18606997	2.152693111	−4.346159	2.266804e−05	0.0007085833	2.361681	METTL19	0.226419470	1.322289653	3.547661	0.0004909159	0.006849851	−0.4854499	BC089413	0.51470326	0.806699712	3.076709	0.002405891	0.02160742	−1.928814	DQ571410	0.01131199	0.001358809	2.738196	0.006772172	0.04484199	−2.851602
850	ACY1	−0.60211351	4.415621742	−4.345686	2.271245e−05	0.0007091361	2.359853	TFR2	−0.628003976	1.176897868	−3.546541	0.0004928750	0.006873574	−0.4890971	DQ595015	−0.11184957	0.082812252	−3.076525	0.002407299	0.02161276	−1.929340	RGMB	−0.49656251	2.947627353	−2.738018	0.006775698	0.04485414	−2.852062
851	PRKRA	−0.28823284	3.415188184	−4.338635	2.338451e−05	0.0007292615	2.332625	POLD1	0.507010960	3.425317884	3.546014	0.0004937980	0.006879622	−0.4908103	SLC25A38	0.23568480	3.891602458	3.076198	0.002409817	0.02162805	−1.930280	DQ598729	0.02290151	0.002577499	2.737475	0.006786450	0.04491411	−2.853462
852	PAOX	−0.42900095	2.070867746	−4.337462	2.349823e−05	0.0007311635	2.328096	ZNF473	0.354282003	1.936522569	3.545998	0.0004938272	0.006879622	−0.4908644	SAMD12−AS1	0.17222968	0.393515126	3.075399	0.002415968	0.02167592	−1.932573	PFKFB2	−0.43293432	2.544268312	−2.737127	0.006793367	0.04494418	−2.854362
853	DUOX2	−1.86517401	2.745896806	−4.337437	2.350060e−05	0.0007311635	2.328002	MRC1	0.183121731	0.349297168	3.545681	0.0004943845	0.006883773	−0.4918972	ZNF587B	0.25440936	1.899261969	3.075084	0.002418398	0.02168432	−1.933477	FASTKD5	0.24600345	3.207538309	2.737076	0.006794379	0.04494418	−2.854494
854	EXOC6	0.34669948	1.923021273	4.335608	2.367891e−05	0.0007358486	2.320944	RFPL1−AS1	0.042227026	0.099272992	3.545037	0.0004955164	0.006895914	−0.4939912	MBD3	0.24689191	4.640327205	3.074857	0.002420153	0.02168432	−1.934129	NOXRED1	0.07265370	0.222526248	2.736585	0.006804117	0.04499737	−2.855758
855	AMOT	0.73367410	2.018259845	4.331906	2.404373e−05	0.0007463119	2.306670	FIGN	0.320084516	0.489264377	3.542629	0.0004997743	0.006950801	−0.5018255	SI	−1.15102061	1.590671596	−3.074846	0.002420231	0.02168432	−1.934158	KALRN	0.34352168	1.817357726	2.735954	0.006816684	0.04505953	−2.857388
856	AK055364	0.11838061	0.169864997	4.328652	2.436880e−05	0.0007555183	2.294134	DST	−0.571209316	2.244333946	−3.542511	0.0004999841	0.006950801	−0.5022099	SLC25A22	0.40249847	3.576110581	3.074774	0.002420790	0.02168432	−1.934365	GOLGA8B	−0.31418519	1.092165787	−2.735942	0.006816911	0.04505953	−2.857417
857	DEPDC1B	0.72843949	2.643771362	4.327613	2.447349e−05	0.0007578787	2.290132	OR10A5	0.042875988	0.035338669	3.541489	0.0005018022	0.006971430	−0.5055331	C20orf72	0.34489594	3.516026711	3.074701	0.002421351	0.02168432	−1.934574	LIAS	0.24011753	2.027550693	2.735626	0.006823204	0.04508989	−2.858232
858	UNC45A	−0.32970463	3.676710998	−4.326785	2.455719e−05	0.0007595841	2.286945	HLA−J	0.804478410	2.531844780	3.541382	0.0005019934	0.006971430	−0.5058819	HOTAIR	−0.49527650	0.734431541	−3.074642	0.002421806	0.02168432	−1.934743	HLA−C	0.47948241	9.487212255	2.735455	0.006826608	0.04510115	−2.858672
859	KLF7	−0.60929322	2.346068705	−4.325382	2.469972e−05	0.0007631034	2.281543	SLC47A1	0.355822353	0.737974163	3.540737	0.0005031439	0.006983753	−0.5079777	TMEM41A	0.34589890	2.657915034	3.073066	0.002434014	0.02178133	−1.939264	PTGDR	0.13434013	0.320262772	2.735314	0.006829420	0.04510851	−2.859036
860	HLA−DMB	1.00705329	3.668071368	4.324191	2.482138e−05	0.0007655697	2.276957	AK055601	0.369589868	2.976733860	3.539606	0.0005051676	0.007001877	−0.5116526	JN120858	0.02921112	0.024654792	3.073031	0.002434283	0.02178133	−1.939363	CST7	0.69922218	3.471047035	2.735093	0.006833828	0.04512639	−2.859606
861	TCRBV15S1	0.53514236	0.642162913	4.324036	2.483724e−05	0.0007655697	2.276360	LST1	0.594271173	3.241800740	3.539597	0.0005051839	0.007001877	−0.5116823	UCHL1	−0.72665465	2.094161999	−3.072577	0.002437810	0.02180554	−1.940665	ADAM11	0.08339731	0.181338309	2.734974	0.006836202	0.04512868	−2.859912
862	RFFL	0.29857906	3.060428113	4.323634	2.487840e−05	0.0007659489	2.274815	IKBKE	0.410276820	2.804650513	3.539565	0.0005052411	0.007001877	−0.5117859	C21orf90	−0.39336224	0.707211795	−3.072264	0.002440247	0.02181999	−1.941563	LRRC10B	−0.38873028	0.759142459	−2.734827	0.006839143	0.04512868	−2.860292
863	MTAP	0.56987008	2.038176759	4.322488	2.499624e−05	0.0007678359	2.270404	C3orf38	0.234747683	3.203138462	3.538969	0.0005063110	0.007013042	−0.5137225	FZD5	0.47113349	3.808767337	3.072095	0.002441558	0.02182435	−1.942046	COL5A2	−0.50043280	4.675444124	−2.734820	0.006839276	0.04512868	−2.860309
864	CABIN1	0.49395890	4.190360078	4.322475	2.499756e−05	0.0007678359	2.270355	TP53INP2	−0.620917536	4.259972115	−3.538625	0.0005069303	0.007017957	−0.5148417	NMUR1	0.29007645	0.695627330	3.071602	0.002445402	0.02185136	−1.943460	PLD3	0.34023942	6.049482061	2.733878	0.006858114	0.04524173	−2.862738
865	SNORD1B	0.24679469	0.355453966	4.321845	2.506253e−05	0.0007689416	2.267932	NOC3L	0.298216893	2.465090976	3.538084	0.0005079049	0.007027783	−0.5165999	FAM57A	0.41753755	4.060272110	3.071323	0.002447572	0.02186338	−1.944258	DQ580359	0.03873250	0.003807988	2.733349	0.006868713	0.04530039	−2.864101
866	LOC728723	0.10425138	0.231059415	4.319189	2.533836e−05	0.0007765067	2.257716	NEU3	0.297552001	1.505791053	3.536925	0.0005099972	0.007053056	−0.5203632	SOWAHD	0.30207150	1.008291017	3.071181	0.002448687	0.02186586	−1.944667	B4GALNT1	−0.25489543	0.645152107	−2.732995	0.006875816	0.04533002	−2.865014
867	MAGED1	−0.84435832	5.500419469	−4.318700	2.538946e−05	0.0007768091	2.255836	TRA@	0.399520741	0.675444489	3.536505	0.0005107585	0.007058274	−0.5217287	MTHFD2	0.41432440	4.752852976	3.071077	0.002449496	0.02186586	−1.944964	YAF2	0.13872176	1.398716202	2.732955	0.006876622	0.04533002	−2.865117
868	FAS−AS1	0.10192075	0.178568657	4.318375	2.542345e−05	0.0007768091	2.254587	CEACAM4	0.178133244	0.360186124	3.536421	0.0005109097	0.007058274	−0.5219997	ARHGAP25	0.49577892	1.989840268	3.070965	0.002450373	0.02186632	−1.945286	FAM110C	−0.51035973	1.919965479	−2.732722	0.006881282	0.04534947	−2.865715
869	SCO1	0.42303553	3.642899882	4.318255	2.543604e−05	0.0007768091	2.254125	GABPB2	−0.310112040	2.430215697	−3.536276	0.0005111723	0.007058274	−0.5224701	LEFTY1	−1.23299532	1.279687920	−3.070838	0.002451357	0.02186776	−1.945647	PRRG4	0.40468740	2.942888452	2.732531	0.006885133	0.04536359	−2.866209
870	ARPC5L	0.38915128	4.515710892	4.317604	2.550436e−05	0.0007780004	2.251621	C9orf11	0.062520160	0.084046630	3.536026	0.0005116273	0.007060882	−0.5232846	PEG10	−1.05269005	1.634926138	−3.070543	0.002453664	0.02187825	−1.946493	ZNF207	0.15875306	4.957717553	2.732147	0.006892847	0.04540315	−2.867198
871	LRRC6	−0.56301342	1.045771759	−4.317086	2.555880e−05	0.0007787657	2.249631	ZWINT	0.581175445	4.159068241	3.534665	0.0005141031	0.007091363	−0.5277035	RGPD3	0.02522673	0.041576556	3.070441	0.002454465	0.02187825	−1.946786	C1orf216	−0.22045936	2.143243798	−2.731662	0.006902602	0.04545612	−2.868446
872	MANEAL	0.90740363	2.873171112	4.316257	2.564622e−05	0.0007805334	2.246445	PHF5A	0.296870392	4.420413852	3.534026	0.0005152679	0.007102565	−0.5297751	TSSC4	0.17591838	3.845815462	3.070372	0.002455006	0.02187825	−1.946984	RPUSD2	0.21931197	2.422193244	2.730994	0.006916070	0.04553351	−2.870167
873	FLJ90757	−0.43826245	2.203033823	−4.315461	2.573038e−05	0.0007821976	2.243387	THAP5	−0.276697530	2.581315373	−3.533926	0.0005154505	0.007102565	−0.5300993	CPSF7	0.20825070	4.373268221	3.069757	0.002459822	0.02191380	−1.948746	PTPN18	−0.30974389	4.581790151	−2.730830	0.006919377	0.04554399	−2.870589
874	AK057177	0.24941464	1.694042316	4.314641	2.581742e−05	0.0007839455	2.240236	RTN4	−0.423904776	5.739413899	−3.532252	0.0005185194	0.007141145	−0.5355327	RGS11	−0.37027293	0.850798625	−3.069395	0.002462661	0.02193098	−1.949783	FAM3D	−1.21868591	4.398325620	−2.730450	0.006927043	0.04558314	−2.871566
875	OR51Q1	0.01949247	0.007575702	4.312822	2.601134e−05	0.0007884220	2.233252	WTIP	−0.517664657	2.138272761	−3.532099	0.0005188002	0.007141306	−0.5360283	TRIP4	−0.18188021	3.461249194	−3.069219	0.002464042	0.02193098	−1.950287	SNORD75	0.13544933	0.317797344	2.729692	0.006942365	0.04564984	−2.873517
876	HLA−DQB1	1.39402898	5.642371289	4.312306	2.606672e−05	0.0007884220	2.231267	LOC285484	0.047519586	0.053873315	3.529729	0.0005231747	0.007197789	−0.5437133	ZNF470	−0.22339237	0.561574631	−3.069121	0.002464807	0.02193098	−1.950566	MIR1226	0.11683181	0.267686075	2.729685	0.006942510	0.04564984	−2.873535
877	IL28RA	−0.57142453	1.417337502	−4.312272	2.607035e−05	0.0007884220	2.231137	ABLIM2	−0.499967182	1.366164276	−3.528081	0.0005262379	0.007236180	−0.5490560	TRMT61B	0.19534711	2.923762306	3.069089	0.002465056	0.02193098	−1.950657	CDKL1	−0.18094699	0.789840692	−2.729651	0.006943193	0.04564984	−2.873622
878	M6PR	0.40147715	4.778061008	4.312148	2.608367e−05	0.0007884220	2.230660	NEK6	0.446258370	4.358764180	3.527714	0.0005269217	0.007238567	−0.5502444	PPIF	0.39616356	5.443374931	3.068080	0.002472992	0.02199421	−1.953546	HIP1	−0.33282205	2.796086790	−2.729608	0.006944061	0.04564984	−2.873732
879	FANCM	0.26821841	0.916263026	4.310867	2.622151e−05	0.0007910455	2.225742	EVI2A	0.530449372	2.022435113	3.527695	0.0005269570	0.007238567	−0.5503058	DQ571986	−0.15639806	0.188185998	−3.067107	0.002480667	0.02205508	−1.956331	MSH2	0.29140306	3.040294706	2.729069	0.006954977	0.04571029	−2.875119
880	SUN1	−0.45094982	4.078738876	−4.310787	2.623008e−05	0.0007910455	2.225437	C4BPA	0.839553578	1.045104381	3.527216	0.0005278525	0.007247118	−0.5518597	FAM92A1	−0.38731418	1.866204008	−3.066917	0.002482172	0.02206107	−1.956876	SH3BP4	−0.42939289	4.602170526	−2.728977	0.006956848	0.04571126	−2.875356
881	AK096766	0.10034867	0.098900449	4.309594	2.635911e−05	0.0007932013	2.220858	BC141902	0.074408392	0.135102254	3.526818	0.0005285960	0.007253573	−0.5531478	RPL27A	0.30599344	6.074882694	3.065995	0.002489468	0.02211851	−1.959515	APBB1	−0.42280543	2.008914926	−2.728470	0.006967137	0.04576754	−2.876661
882	LAPTM4B	−0.96438732	6.183418165	−4.309574	2.636134e−05	0.0007932013	2.220779	LCK	0.800864472	2.833497401	3.526341	0.0005294903	0.007259585	−0.5546946	TSPAN2	−0.67999301	2.221188445	−3.064909	0.002498085	0.02218764	−1.962620	RARRES2	−0.52696265	5.747303839	−2.728385	0.006968865	0.04576756	−2.876879
883	PLK1S1	−0.57976195	2.925716490	−4.304868	2.687631e−05	0.0008077807	2.202726	KCNJ10	0.440313913	0.471880143	3.526292	0.0005295813	0.007259585	−0.5548518	LMBR1	0.26451551	3.300967585	3.064022	0.002505145	0.02223644	−1.965156	DQ595965	0.03226040	0.003176059	2.727894	0.006978850	0.04582180	−2.878143
884	YWHAH	0.33189431	6.487219655	4.303473	2.703079e−05	0.0008115046	2.197378	PAPOLG	0.161428535	1.975755858	3.525969	0.0005301871	0.007264138	−0.5558980	MARK1	−0.41102278	0.924562696	−3.064008	0.002505255	0.02223644	−1.965196	TSPAN14	0.23955997	4.482070212	2.727250	0.006991958	0.04589651	−2.879799
885	TMEM52	−0.84851362	1.944864786	−4.301959	2.719944e−05	0.0008156451	2.191575	ALAS1	0.401150337	4.948052953	3.523403	0.0005350243	0.007326631	−0.5642089	KIF26B	−0.49203230	1.672427933	−3.063736	0.002507423	0.02224283	−1.965973	VIPR1	−0.61009802	2.127092951	−2.726921	0.006998650	0.04592907	−2.880643
886	BC047609	0.18019535	0.279385402	4.301042	2.730206e−05	0.0008177984	2.188061	SNORA64	0.201498210	0.818920253	3.522761	0.0005362402	0.007339494	−0.5662859	DRD3	0.00992959	0.007536808	3.063708	0.002507651	0.02224283	−1.966055	UNC119	−0.24981673	3.760029840	−2.726416	0.007008948	0.04598528	−2.881941
887	LOC643201	0.28778186	0.266492275	4.298820	2.755228e−05	0.0008234492	2.179550	RNF133	0.062000667	0.078669072	3.522407	0.0005369121	0.007344902	−0.5674317	Mir_500	0.04893201	0.024497635	3.063553	0.002508886	0.02224635	−1.966497	BC064148	0.06631793	0.072885664	2.725741	0.007022755	0.04606449	−2.883678
888	GPR174	0.51911110	0.669453172	4.298816	2.755277e−05	0.0008234492	2.179533	KNTC1	0.421258497	2.351467423	3.521879	0.0005379156	0.007354839	−0.5691403	IL15	0.28996518	1.299701974	3.063300	0.002510907	0.02225683	−1.967221	L32131	0.02092130	0.051884282	2.725604	0.007025541	0.04607137	−2.884028
889	DNM1	−0.77332637	2.200637635	−4.298005	2.764463e−05	0.0008251827	2.176428	LOC100505715	0.186537306	0.443645809	3.521153	0.0005392975	0.007369937	−0.5714879	ADAT1	0.23561929	2.628345567	3.063114	0.002512394	0.02226258	−1.967753	SNHG15	0.30392685	2.398582563	2.724990	0.007038121	0.04614246	−2.885606
890	ZNF225	0.24526680	0.999742955	4.297489	2.770330e−05	0.0008251827	2.174450	FRMD5	0.512071966	1.142645506	3.520468	0.0005406056	0.007384010	−0.5737043	HIST1H2AB	0.11640639	0.171515580	3.062781	0.002515055	0.02227418	−1.968705	AK022914	−0.19432300	0.489923880	−2.724646	0.007045164	0.04617723	−2.886489
891	SCMH1	−0.48586704	3.455457328	−4.297365	2.771742e−05	0.0008251827	2.173974	NAA25	0.283276256	2.600898611	3.519575	0.0005423159	0.007403561	−0.5765944	MAP3K1	0.34182736	2.655090324	3.062740	0.002515382	0.02227418	−1.968821	MIR4674	0.08621383	0.115785202	2.724219	0.007053938	0.04621084	−2.887587
892	LPCAT2	−0.67886325	2.594077839	−4.297209	2.773514e−05	0.0008251827	2.173378	SMOC1	−0.978962303	1.361304064	−3.519117	0.0005431939	0.007411735	−0.5780744	CD300C	0.31451966	0.955620994	3.062573	0.002516715	0.02227855	−1.969297	LOC100132735	0.03414200	0.029448424	2.724210	0.007054126	0.04621084	−2.887611
893	LOC728175	0.12003077	0.201810175	4.296363	2.783155e−05	0.0008271237	2.170141	C12orf12	0.008833726	0.003320027	3.518840	0.0005437265	0.007411791	−0.5789709	BEND5	−0.41998124	0.955074747	−3.061916	0.002521983	0.02230644	−1.971177	CARD14	−0.32976188	0.864462611	−2.724056	0.007057276	0.04621084	−2.888005
894	WFDC2	−2.00951328	3.964297212	−4.295810	2.789483e−05	0.0008280771	2.168022	ZSCAN22	0.199365824	1.205349552	3.518824	0.0005437566	0.007411791	−0.5790216	BC035392	0.36730405	1.271909247	3.061838	0.002522608	0.02230644	−1.971399	SLC16A9	−0.39699769	0.798749335	−2.724026	0.007057901	0.04621084	−2.888083
895	GUCY2C	−1.54548157	2.273878109	−4.295509	2.792929e−05	0.0008281735	2.166870	PUS3	0.248802538	2.879679409	3.518061	0.0005452236	0.007427971	−0.5814864	MAP4K2	−0.34234416	2.770508537	−3.061807	0.002522857	0.02230644	−1.971488	USP1	0.24624878	3.636552485	2.723972	0.007058998	0.04621084	−2.888220
896	SH2D1A	0.65651355	1.104468601	4.295187	2.796624e−05	0.0008283438	2.165637	TLCD1	−0.607804200	3.360654955	−3.517183	0.0005469182	0.007443730	−0.5843255	CCDC107	−0.35944716	3.718369792	−3.061761	0.002523227	0.02230644	−1.971620	BC070106	−0.14435438	0.225739240	−2.723536	0.007067975	0.04625819	−2.889341
897	ZNHIT3	0.28508746	3.214262612	4.292638	2.826012e−05	0.0008356284	2.155885	PARP14	0.497306080	3.743105100	3.517171	0.0005469412	0.007443730	−0.5843640	AL832593	0.19538984	1.776413283	3.061574	0.002524728	0.02231227	−1.972154	IGF2BP2	0.64884405	3.655632922	2.723445	0.007069854	0.04625908	−2.889576
898	REC8	1.31515093	2.699427605	4.292509	2.827515e−05	0.0008356284	2.155389	F10	−0.755067337	1.523382846	−3.516751	0.0005477541	0.007450971	−0.5857226	DGKD	−0.32790539	2.875536734	−3.061373	0.002526337	0.02231906	−1.972727	SLX4	0.16859318	1.471465937	2.723071	0.007077536	0.04629794	−2.890534
899	MED17	0.27935588	2.659103608	4.290534	2.850504e−05	0.0008414852	2.147835	C1orf229	−0.136588498	0.189656678	−3.516297	0.0005486327	0.007459100	−0.5871888	ZNF837	0.24116902	1.303886544	3.060187	0.002535879	0.02239591	−1.976114	ELN	−0.71583000	4.209733553	−2.722757	0.007084015	0.04631751	−2.891341
900	MRPL28	0.37136067	5.155891466	4.287976	2.880547e−05	0.0008494094	2.138055	ARFGAP2	0.216993594	4.330538582	3.515602	0.0005499821	0.007470659	−0.5894361	ANGPTL4	−0.65018221	2.325405274	−3.059934	0.002537919	0.02240647	−1.976837	CSNK1G2	0.18035461	4.381024561	2.722757	0.007084019	0.04631751	−2.891342

	Gene	logFC	AveExpr	t	P.Value	adj.P.Val	B	Gene	logFC	AveExpr	t	P.Value	adj.P.Val	B	Gene	logFC	AveExpr	t	P.Value	adj.P.Val	B	Gene	logFC	AveExpr	t	P.Value	adj.P.Val	B
901	RAB43	0.39578561	2.28277954	4.286732	2.895260e−05	0.0008528004	2.133303	MSI1	−0.85153913	1.374980560	−3.515569	0.0005500459	0.007470659	−0.5895422	CHST6	−0.304080970	0.529033953	−3.058975	0.002545671	0.02246743	−1.979577	H1FX	0.301290158	6.309701619	2.722438	0.007090588	0.04634904	−2.892160
902	KCNJ15	0.90026129	1.27140979	4.282282	2.948509e−05	0.0008675221	2.116304	FANCL	0.30987452	3.423014577	3.513969	0.0005531625	0.007509145	−0.5947106	EXOC2	0.209276597	3.008220514	3.058780	0.002547247	0.02247386	−1.980133	JAK1	0.250285611	4.398561511	2.722054	0.007098512	0.04638941	−2.893145
903	TRBV5−4	0.46593090	0.67415505	4.281246	2.961047e−05	0.0008702461	2.112347	CCT6B	0.22297177	0.621281314	3.512773	0.0005555013	0.007537040	−0.5985700	LOC100526771	0.077140403	0.118512406	3.058571	0.002548936	0.02248129	−1.980729	CBX5	−0.367184621	3.540797714	−2.721755	0.007104689	0.04641835	−2.893912
904	ABI3	0.66171640	3.17029308	4.279620	2.980818e−05	0.0008750876	2.106141	ACTR3B	0.33123979	1.883384753	3.512401	0.0005562324	0.007543103	−0.5997731	AF143871	0.157708678	0.377471256	3.058405	0.002550283	0.02248570	−1.981203	LOC100130557	0.144500399	0.985277091	2.721387	0.007112305	0.04645636	−2.894858
905	NUP50	0.37994992	3.37707553	4.278938	2.989146e−05	0.0008765629	2.103538	PRAME	−0.96885926	1.078032788	−3.512164	0.0005566965	0.007544125	−0.6005359	PIP5K1P1	0.023863909	0.044528796	3.058171	0.002552181	0.02249375	−1.981872	SPATS1	0.039274682	0.053993163	2.721305	0.007114007	0.04645636	−2.895069
906	CD86	0.60783116	2.18082614	4.278010	3.000507e−05	0.0008789235	2.100000	SLC25A51P1	0.03580407	0.014200193	3.512073	0.0005568763	0.007544125	−0.6008314	DERL2	0.234124384	4.466723558	3.058083	0.002552891	0.02249375	−1.982122	ZNF827	−0.320071828	2.283886969	−2.720621	0.007128169	0.04653739	−2.896823
907	PRDM1	0.65870196	3.29314094	4.276647	3.017286e−05	0.0008828637	2.094800	FAM18B1	0.35416845	3.611223853	3.511507	0.0005579897	0.007555351	−0.6026582	HOXA2	−0.326125842	0.850707223	−3.057869	0.002554632	0.02250162	−1.982734	CCT7	0.216191204	6.632697013	2.720419	0.007132355	0.04655326	−2.897341
908	LRRN1	−0.97236083	1.12120355	−4.275527	3.031137e−05	0.0008859399	2.090529	UMPS	0.28819541	2.366096822	3.510693	0.0005595952	0.007573226	−0.6052861	GGT7	−0.514758732	2.191732182	−3.057628	0.002556586	0.02251136	−1.983421	LILRB3	0.271745574	1.061724855	2.720174	0.007137437	0.04657498	−2.897969
909	F8A3	−0.18418746	1.06518221	−4.275242	3.034671e−05	0.0008859971	2.089442	MYO1F	0.54495264	2.598083605	3.510546	0.0005598838	0.007573270	−0.6057578	AP1B1	0.251422272	4.805856587	3.056676	0.002564327	0.02257203	−1.986137	NME1−NME2	0.295803601	6.964733552	2.720035	0.007140317	0.04658232	−2.898325
910	PLCD1	−0.48927156	2.42497494	−4.274486	3.044062e−05	0.0008877622	2.086561	MCF2L2	0.08142428	0.219162034	3.509947	0.0005610694	0.007583463	−0.6076926	FAM169B	0.167495135	0.149273166	3.056474	0.002565970	0.02257901	−1.986713	NIF3L1	0.215923296	3.307917364	2.718556	0.007171081	0.04677152	−2.902118
911	PPP1R14D	−1.30208937	2.59455786	−4.273282	3.059092e−05	0.0008911661	2.081968	BCAM	−0.82748434	4.750421992	−3.509876	0.0005612089	0.007583463	−0.6079198	NIT2	0.239374613	3.528363466	3.055738	0.002571975	0.02262435	−1.988813	FAM63A	−0.314320308	2.939167894	−2.718422	0.007173869	0.04677821	−2.902460
912	CCDC122	−0.45943855	1.27903606	−4.272467	3.069289e−05	0.0008929357	2.078864	COL16A1	−0.58643705	2.893829809	−3.509026	0.0005628940	0.007598521	−0.6106620	ELL2	0.408176348	2.895167940	3.055624	0.002572907	0.02262505	−1.989138	MIR3607	0.244351942	0.621689554	2.718249	0.007177483	0.04678213	−2.902905
913	TBC1D3P1−DHX40P1	0.14364683	0.12606870	4.272260	3.071895e−05	0.0008929357	2.078073	TSPO2	0.31252135	0.504043007	3.509025	0.0005628958	0.007598521	−0.6106650	ARSI	−0.371392741	0.978360952	−3.055359	0.002575076	0.02263662	−1.989895	DQ593272	0.009472538	0.001062707	2.718224	0.007177996	0.04678213	−2.902968
914	TCRAVN1	0.37678935	0.46729587	4.270281	3.096837e−05	0.0008990817	2.070532	PID1	−0.74345724	2.057791334	−3.508178	0.0005645805	0.007617388	−0.6133981	ATE1	−0.395819819	3.005719392	−3.054818	0.002579504	0.02266803	−1.991439	ABHD14A	−0.328802703	3.293194191	−2.717868	0.007185434	0.04681911	−2.903881
915	CD247	0.69058103	1.68463609	4.269931	3.101268e−05	0.0008990817	2.069199	SLAMF6	0.65835037	1.488145980	3.507832	0.0005652697	0.007622811	−0.6145138	SCUBE2	−0.432560913	1.067941740	−3.054570	0.002581534	0.02267836	−1.992145	SERPINF1	−0.639061153	5.701413888	−2.717661	0.007189762	0.04683581	−2.904412
916	SNX5	0.39234115	4.63887246	4.269778	3.103202e−05	0.0008990817	2.068618	KIF7	−0.36820607	1.228284316	−3.507158	0.0005666130	0.007637046	−0.6166846	TMEM30A	0.276611190	4.971222114	3.054302	0.002583725	0.02269010	−1.992908	ARHGEF10L	−0.385157394	3.217410972	−2.717403	0.007195150	0.04685400	−2.905073
917	RPSA	0.34933569	3.72335105	4.269363	3.108472e−05	0.0008993355	2.067036	TAF1D	0.34496883	3.424282871	3.506779	0.0005673705	0.007640846	−0.6179064	LOXL2	−0.521378692	4.137667115	−3.053957	0.002586562	0.02270750	−1.993893	AK057887	−0.299737484	2.477020983	−2.717358	0.007196085	0.04685400	−2.905188
918	RAP2C	0.31392343	3.14506526	4.269175	3.110856e−05	0.0008993355	2.066321	CDV3	0.30429938	5.156749738	3.506685	0.0005675590	0.007640846	−0.6182101	C21orf88	−0.298967309	0.338504353	−3.053693	0.002588726	0.02271898	−1.994644	SOGA2	−0.452376312	1.845921242	−2.717121	0.007201053	0.04687485	−2.905797
919	MIR575	0.13196210	0.03971497	4.268187	3.123435e−05	0.0009019895	2.062558	RAN	0.35645532	7.024642353	3.506585	0.0005677587	0.007640846	−0.6185319	TLX1	−0.492560215	0.955175677	−3.053558	0.002589834	0.02272119	−1.995029	SLC9B1	0.212466346	1.308439996	2.717010	0.007203368	0.04687659	−2.906080
920	TCRB	0.44393797	0.95448975	4.266807	3.141087e−05	0.0009061011	2.057303	HECTD3	−0.27064362	4.353502747	−3.505878	0.0005691761	0.007656039	−0.6208121	PSME4	0.227568797	3.592593603	3.053358	0.002591478	0.02272810	−1.995599	KITLG	0.462762673	3.373445312	2.716932	0.007204998	0.04687659	−2.906280
921	CCDC160	0.19443900	0.16317363	4.266271	3.147967e−05	0.0009070998	2.055263	ESYT1	−0.36827488	4.805643214	−3.505681	0.0005695706	0.007657464	−0.6214457	ULK1	−0.317650171	3.431694446	−3.052605	0.002597679	0.02277153	−1.997746	FCGR2C	0.255206768	0.736570195	2.716703	0.007209798	0.04687659	−2.906867
922	SCLT1	0.22079227	1.09971564	4.264391	3.172222e−05	0.0009117825	2.048107	LY9	0.53402806	1.039550031	3.505441	0.0005700534	0.007660074	−0.6222205	RBM8A	−0.192247682	3.937637619	−3.052548	0.002598145	0.02277153	−1.997907	FBLN2	−0.606234877	3.619310909	−2.716620	0.007211534	0.04687659	−2.907079
923	UBE2N	0.28645660	5.25075468	4.264250	3.174045e−05	0.0009117825	2.047571	BDKRB2	−0.50194050	1.933610608	−3.503887	0.0005731822	0.007697750	−0.6272259	PERP	−0.585740195	5.803550728	−3.052005	0.002602632	0.02280332	−1.999457	POGK	−0.220640932	4.227801054	−2.716458	0.007214934	0.04687659	−2.907495
924	RFC2	0.46599041	4.10657803	4.264213	3.174524e−05	0.0009117825	2.047431	RPL6	0.33471585	6.562316665	3.503761	0.0005734373	0.007697750	−0.6276328	GZMK	0.854114445	2.304569257	3.051249	0.002608876	0.02285049	−2.001609	UBASH3B	0.338697874	1.435316615	2.716340	0.007217410	0.04687659	−2.907797
925	SKAP2	−0.51760216	3.63700267	−4.261735	3.206785e−05	0.0009193542	2.038004	AK056351	0.23044871	0.680056704	3.503459	0.0005740470	0.007702039	−0.6286045	SNORD24	0.311498943	0.988282073	3.050787	0.002612698	0.02287641	−2.002924	C5orf52	−0.230994329	0.266333991	−2.716303	0.007218182	0.04687659	−2.907892
926	NLRC3	0.59490038	1.28136044	4.261656	3.207815e−05	0.0009193542	2.037704	CNPPD1	−0.27535846	4.856456017	−3.502812	0.0005753581	0.007715729	−0.6306906	CCDC15	0.242810250	0.802393143	3.050402	0.002615890	0.02289680	−2.004021	OPN5	0.008956084	0.007433857	2.716223	0.007219847	0.04687659	−2.908095
927	TCRBV14.1−J2.1	0.85327777	1.76466688	4.259422	3.237174e−05	0.0009267677	2.029210	PAFAH1B3	0.51820654	4.705606070	3.502266	0.0005764654	0.007721954	−0.6324487	TMEM33	0.294356707	3.684640809	3.050148	0.002617997	0.02290769	−2.004744	RB1CC1	−0.260527889	3.375261226	−2.716181	0.007220730	0.04687659	−2.908203
928	CHEK1	0.54423553	2.32940502	4.258179	3.253618e−05	0.0009294938	2.024487	TOP1	0.32457553	5.118210236	3.502203	0.0005765934	0.007721954	−0.6326518	C2orf63	0.142285390	0.476180487	3.049915	0.002619931	0.02291706	−2.005407	FBXL14	0.317847213	3.787447066	2.716119	0.007222035	0.04687659	−2.908363
929	DNA2	0.55227209	1.57458660	4.258173	3.253701e−05	0.0009294938	2.024463	TANC2	−0.44804138	1.439172896	−3.502153	0.0005766951	0.007721954	−0.6328131	VPS33A	0.192241567	4.041335956	3.048933	0.002628101	0.02298095	−2.008203	TBC1D30	0.334782204	1.336841291	2.716073	0.007223006	0.04687659	−2.908481
930	PHACTR4	0.29292927	3.61617448	4.257554	3.261927e−05	0.0009308418	2.022109	PXMP4	−0.33979616	2.283745263	−3.501431	0.0005781632	0.007734320	−0.6351378	LINC00317	0.008832551	0.001228564	3.048530	0.002631461	0.02300275	−2.009351	PCLO	−0.275308544	0.402141128	−2.715897	0.007226687	0.04687659	−2.908930
931	SLC16A10	−0.49378801	0.90932570	−4.255102	3.294678e−05	0.0009378210	2.012796	LOC100505681	0.22280297	3.037743763	3.501412	0.0005782016	0.007734320	−0.6351984	CYLD	0.264731468	2.034706319	3.048419	0.002632385	0.02300325	−2.009666	SPTSSB	0.741491150	1.841083202	2.715893	0.007226767	0.04687659	−2.908940
932	KIAA1383	−0.34176943	0.73304085	−4.255045	3.295443e−05	0.0009378210	2.012580	PLD2	−0.31299451	2.987790250	−3.501132	0.0005787720	0.007738050	−0.6361000	MIR155HG	0.399047411	1.066468733	3.048072	0.002635287	0.02301750	−2.010656	GALNS	−0.323244888	3.328836556	−2.715881	0.007227028	0.04687659	−2.908972
933	TMEM9	−0.47226406	4.14775389	−4.254802	3.298712e−05	0.0009378210	2.011655	PHACTR2	0.31837917	2.867632625	3.500986	0.0005790692	0.007738126	−0.6365695	SDC2	−0.504431554	3.761244747	−3.047945	0.002636347	0.02301750	−2.011017	EGFL7	−0.469695853	3.364527590	−2.715844	0.007227815	0.04687659	−2.909068
934	TBC1D12	0.30844456	1.97000776	4.254668	3.300519e−05	0.0009378210	2.011145	LOC100505483	−0.48775295	1.293557339	−3.500606	0.0005798435	0.007744573	−0.6377912	RFPL3	0.045659422	0.088586906	3.047912	0.002636617	0.02301750	−2.011109	MIR1304	0.283882464	1.441848622	2.715601	0.007232917	0.04689821	−2.909690
935	CPSF4	0.44158857	3.37300885	4.254179	3.307091e−05	0.0009386833	2.009291	WISP3	−0.65231364	0.839751995	−3.500372	0.0005803224	0.007747071	−0.6385461	CLEC2B	0.456474434	2.476432618	3.047772	0.002637790	0.02301940	−2.011508	KCNK3	−0.516243227	1.030492050	−2.715448	0.007236130	0.04690759	−2.910082
936	DNAJB7	0.22065523	0.64635585	4.253246	3.319693e−05	0.0009412537	2.005745	FAM171A2	−0.40910254	0.978667104	−3.500201	0.0005806704	0.007747818	−0.6390942	FNDC8	0.240317491	0.750394954	3.047679	0.002638570	0.02301940	−2.011773	DQ591572	0.341144702	1.824652444	2.714817	0.007249383	0.04698202	−2.911696
937	CLRN3	−1.19553351	2.77855798	−4.251738	3.340135e−05	0.0009460389	2.000023	BATF3	0.34099675	1.409026822	3.499033	0.0005830632	0.007775836	−0.6428541	PLSCR4	−0.503507485	2.624325441	−3.047495	0.002640109	0.02301945	−2.012297	IL1R1	−0.494588801	3.946268136	−2.714673	0.007252417	0.04699021	−2.912065
938	DD413671	0.20238441	0.14695722	4.250186	3.361319e−05	0.0009502918	1.994130	CRTC2	−0.23855616	4.157821965	−3.498810	0.0005835204	0.007778025	−0.6435707	METTL15	0.206761478	1.719584498	3.047471	0.002640310	0.02301945	−2.012365	MAGEE2	−0.025115330	0.035414519	−2.714360	0.007259020	0.04702151	−2.912867
939	AQP11	0.40816709	1.38036385	4.250096	3.362550e−05	0.0009502918	1.993789	TIFA	0.35248336	2.023670726	3.497860	0.0005854748	0.007800159	−0.6466279	NCF1C	0.416711823	0.992009301	3.047256	0.002642110	0.02302758	−2.012978	BTNL3	−0.617871480	1.133719786	−2.714054	0.007265471	0.04704655	−2.913650
940	AK2	0.28790337	4.02674553	4.249852	3.365893e−05	0.0009502918	1.992862	C8orf47	−0.67717746	1.320774956	−3.497589	0.0005860330	0.007803678	−0.6474992	FUS	0.214015992	5.041094953	3.047007	0.002644189	0.02303813	−2.013684	SRBD1	0.174767251	2.457840431	2.713953	0.007267600	0.04704655	−2.913909
941	SOGA1	−0.65497958	2.18955325	−4.249044	3.376973e−05	0.0009524070	1.989799	SGMS1	0.31915334	3.038670086	3.497040	0.0005871662	0.007814846	−0.6492651	KIF4B	0.028966142	0.057029458	3.046883	0.002645233	0.02303965	−2.014038	TXNDC11	0.253513774	4.431158819	2.713924	0.007268204	0.04704655	−2.913982
942	LOC389247	0.17044944	0.40159057	4.248644	3.382479e−05	0.0009529471	1.988281	C16orf45	−0.71205211	2.818030091	−3.496727	0.0005878137	0.007819543	−0.6502728	NBEA	−0.410348704	0.781240793	−3.046381	0.002649443	0.02306875	−2.015466	RPL27	0.247108991	10.447475685	2.713543	0.007276249	0.04708715	−2.914958
943	C9orf116	0.56565049	1.43105801	4.247408	3.399542e−05	0.0009567387	1.983591	DQ571433	0.07327311	0.029815779	3.496081	0.0005891505	0.007833399	−0.6523495	SULT4A1	−0.329278610	0.453307208	−3.045636	0.002655698	0.02311061	−2.017584	NACC1	0.206451423	4.799362420	2.713374	0.007279819	0.04709876	−2.915390
944	RNF19B	0.45303900	4.22645016	4.246448	3.412847e−05	0.0009594657	1.979950	PLK4	0.44809876	1.963814206	3.493806	0.0005938813	0.007887247	−0.6596608	MIR3908	0.038338662	0.012960936	3.045601	0.002655992	0.02311061	−2.017683	CPNE4	0.121398667	0.184358103	2.713219	0.007283093	0.04710846	−2.915787
945	LOC100505702	0.29770850	0.71933847	4.245758	3.422436e−05	0.0009611432	1.977335	WDR45	−0.32022785	3.904746028	−3.493773	0.0005939507	0.007887247	−0.6597677	MIR4722	0.263804318	0.661188762	3.045315	0.002658402	0.02312400	−2.018497	ANAPC5	0.199034702	4.448587588	2.712804	0.007291872	0.04714520	−2.916849
946	ITK	0.72369459	1.31512401	4.245364	3.427931e−05	0.0009616687	1.975840	ACP5	0.66596775	4.682333772	3.493593	0.0005943261	0.007887247	−0.6603452	AAMP	0.229404070	5.671121115	3.044901	0.002661884	0.02314368	−2.019673	PKN2	0.233394511	3.421915404	2.712782	0.007292327	0.04714520	−2.916904
947	P2RX7	0.44177349	1.27609904	4.244167	3.444653e−05	0.0009653395	1.971304	LPPR2	−0.46798360	3.238948050	−3.493563	0.0005943892	0.007887247	−0.6604422	CSDC2	−0.353986222	0.978529231	−3.044839	0.002662408	0.02314368	−2.019850	GDPD5	−0.447790589	1.961590955	−2.712578	0.007296651	0.04715558	−2.917427
948	COBL	−0.56543270	2.17433922	−4.243806	3.449714e−05	0.0009657379	1.969936	CCDC102B	0.24595416	0.806096740	3.492645	0.0005963097	0.007908777	−0.6633911	SEBOX	0.065298563	0.123296648	3.044534	0.002664983	0.02315847	−2.020717	CIDEC	−0.458880690	2.037316097	−2.712539	0.007297485	0.04715558	−2.917527
949	ART3	0.31619988	0.23020643	4.242904	3.462392e−05	0.0009682658	1.966517	CA13	0.60457743	2.065070627	3.492177	0.0005972901	0.007917823	−0.6648927	GPR34	0.446069274	2.128783165	3.044074	0.002668867	0.02318463	−2.022025	TUBA4A	−0.430584221	5.248755360	−2.712372	0.007301017	0.04716692	−2.917954
950	THYN1	0.36607143	4.13379440	4.242088	3.473901e−05	0.0009704617	1.963425	ABHD15	−0.28997118	3.047310109	−3.491744	0.0005981993	0.007925917	−0.6662831	PLEKHB1	−0.894269486	3.567870920	−3.043886	0.002670453	0.02318652	−2.022559	C1orf198	−0.271709506	4.353461395	−2.711868	0.007311700	0.04721837	−2.919243
951	HLA−DMA	1.04507016	5.66686614	4.240967	3.489764e−05	0.0009738679	1.959179	ZNF280D	−0.21472115	1.814065571	−3.491277	0.0005991833	0.007934993	−0.6677853	CHN1	−0.381464441	2.129320804	−3.043793	0.002671244	0.02318652	−2.022824	NSL1	0.141689359	1.574123780	2.711828	0.007312540	0.04721837	−2.919344
952	MRPL50	0.43650920	4.06421775	4.239705	3.507713e−05	0.0009778487	1.954398	TTC30A	−0.34152472	1.271936051	−3.490443	0.0006009408	0.007954299	−0.6704625	SPATA17	−0.266337299	0.646786071	−3.043738	0.002671705	0.02318652	−2.022979	KAAG1	−0.362706148	0.567096902	−2.711692	0.007315430	0.04722554	−2.919693
953	ANKRD34A	0.22894079	0.53380284	4.238041	3.531501e−05	0.0009834470	1.948100	FKBP7	−0.37721070	2.228947357	−3.489965	0.0006019504	0.007963689	−0.6719967	TTC19	0.288980387	3.167771323	3.043492	0.002673788	0.02319701	−2.023679	DQ575010	0.114415813	0.734616179	2.711504	0.007319423	0.04723982	−2.920174
954	CCR8	0.36073538	0.56770838	4.237708	3.536288e−05	0.0009837478	1.946837	TEC	0.22095967	0.917639304	3.488466	0.0006051271	0.008001727	−0.6768074	CCDC41	0.222449375	1.448457417	3.042344	0.002683524	0.02327387	−2.026942	AK091889	0.022832242	0.013748248	2.711406	0.007321510	0.04724181	−2.920425
955	ST6GALNAC4	−0.57790427	4.35836976	−4.235747	3.564567e−05	0.0009905764	1.939415	SNORD88B	0.48627760	1.738301731	3.487292	0.0006076252	0.008029404	−0.6805727	CCL28	−0.776931459	2.284599748	−3.042180	0.002684916	0.02327834	−2.027408	KIF18B	0.438424769	2.601586230	2.710989	0.007330366	0.04728745	−2.921491
956	CHAF1B	0.60102378	2.52863674	4.231037	3.633379e−05	0.0010086427	1.921599	MIR378F	0.03390720	0.008290142	3.487199	0.0006078252	0.008029404	−0.6808735	PMCH	0.382291888	1.785863430	3.042010	0.002686360	0.02328325	−2.027891	HCP5	0.563499572	4.423914193	2.710366	0.007343629	0.04736150	−2.923085
957	HOXA11	−0.87824405	1.25568894	−4.230389	3.642945e−05	0.0010102415	1.919149	GVINP1	0.45285111	0.959397874	3.486827	0.0006086181	0.008035879	−0.6820649	PVT1	0.453113237	1.974631483	3.041501	0.002690692	0.02331318	−2.029337	TIMELESS	0.403527452	3.086140723	2.710099	0.007349307	0.04738660	−2.923766
958	LOC100499466	−0.40623619	2.51272672	−4.229998	3.648722e−05	0.0010107873	1.917672	GPR158	−0.42436125	0.568436790	−3.486574	0.0006091603	0.008039038	−0.6828788	CHST10	−0.325725354	1.383870491	−3.040988	0.002695060	0.02333652	−2.030794	MIR548J	0.060058584	0.055837872	2.709279	0.007366791	0.04748780	−2.925861
959	LARP6	−0.70656643	2.01069424	−4.228507	3.670865e−05	0.0010158612	1.912035	PGM1	−0.46227834	4.092913559	−3.486155	0.0006100546	0.008046839	−0.6842195	NKX6−3	−0.731628741	1.249723955	−3.040978	0.002695144	0.02333652	−2.030822	MIR4439	0.067215808	0.045342866	2.709099	0.007370640	0.04750107	−2.926321
960	C19orf76	0.38242830	0.92046392	4.227884	3.680141e−05	0.0010173674	1.909684	LOC401127	0.12292099	0.375411231	3.483928	0.0006148399	0.008105930	−0.6913599	CDK12	0.322099834	3.492104947	3.040632	0.002698091	0.02335200	−2.031803	BGN	−0.581066984	7.517188810	−2.708571	0.007381943	0.04756191	−2.927672
961	C11orf24	−0.43828629	4.92316713	−4.226866	3.695371e−05	0.0010202505	1.905836	CDT1	0.67982563	3.686979055	3.481969	0.0006190801	0.008157779	−0.6976401	ATP11B	0.312600230	3.577106478	3.040461	0.002699551	0.02335200	−2.032288	ADAMTSL4	−0.491110764	2.497100770	−2.708490	0.007383664	0.04756191	−2.927878
962	RIOK3	−0.50891721	4.34886868	−4.226049	3.707632e−05	0.0010202505	1.902750	ZUFSP	0.23493661	2.048011373	3.481726	0.0006196067	0.008160666	−0.6984171	TRAP1	0.332885814	5.271316501	3.040459	0.002699572	0.02335200	−2.032295	AK056211	0.017674247	0.018607158	2.708284	0.007388067	0.04757872	−2.928403
963	AX746857	0.12925355	0.17578682	4.225994	3.708452e−05	0.0010202505	1.902544	BC041449	0.09780709	0.157129187	3.480001	0.0006233645	0.008206086	−0.7039419	SH3RF3	−0.353070761	1.843056501	−3.040104	0.002702607	0.02337064	−2.033304	AKR7A3	−0.861277030	4.969355018	−2.708039	0.007393324	0.04760102	−2.929030
964	C9orf139	0.08715600	0.12732076	4.225866	3.710388e−05	0.0010202505	1.902058	GPR37L1	−0.25658276	0.518313088	−3.477942	0.0006278777	0.008261401	−0.7105331	SNORD77	0.081937592	0.337443859	3.038937	0.002712595	0.02344937	−2.036615	CSDE1	−0.294398261	6.566387900	−2.707781	0.007398861	0.04761578	−2.929690
965	KLF11	−0.45521011	2.70710605	−4.225479	3.716208e−05	0.0010202505	1.900598	ZNF836	0.24566138	1.319200762	3.476379	0.0006313252	0.008302646	−0.7155358	TMEM176B	−0.679782054	6.360727624	−3.038746	0.002714236	0.02345052	−2.037158	UGCG	0.326262807	3.708878590	2.707765	0.007399204	0.04761578	−2.929731
966	TBC1D3	0.16066173	0.24893008	4.225427	3.716985e−05	0.0010202505	1.900403	FMNL2	0.36960365	3.133154194	3.475380	0.0006335354	0.008325326	−0.7187286	SUMO1	0.219854371	5.316478598	3.038716	0.002714496	0.02345052	−2.037244	LOC100509575	0.162113212	0.054119549	2.707673	0.007401175	0.04761691	−2.929966
967	AIF1	0.73217259	3.58198128	4.225394	3.717481e−05	0.0010202505	1.900279	PRODH	1.02155896	1.959761743	3.475192	0.0006339526	0.008325326	−0.7193299	LOC286059	0.020665086	0.040428679	3.038388	0.002717307	0.02346717	−2.038173	SLC6A1	0.142815238	0.415512185	2.706540	0.007425497	0.04776182	−2.932858
968	MIR3185	−0.70180216	0.84073825	−4.222933	3.754745e−05	0.0010294131	1.890986	SH3PXD2B	−0.52165978	3.116778184	−3.475175	0.0006339909	0.008325326	−0.7193851	PKD2L1	0.135428801	0.222086307	3.037668	0.002723498	0.02351298	−2.040216	ETAA1	0.158606800	2.168410026	2.706074	0.007435534	0.04781479	−2.934048
969	USP28	0.35766821	2.73289387	4.219979	3.799943e−05	0.0010407296	1.879839	REEP6	−1.00583747	3.633989906	−3.475020	0.0006343357	0.008325735	−0.7198819	CCDC157	0.177799508	1.405387978	3.036979	0.002729434	0.02355657	−2.042170	PAQR3	−0.218932697	2.060102005	−2.705674	0.007444143	0.04785855	−2.935068
970	NLRC5	0.67968887	3.15232077	4.218670	3.820142e−05	0.0010451831	1.874901	RWDD4	0.26856121	2.111449491	3.474594	0.0006352814	0.008334025	−0.7212428	AMPD1	0.243948819	0.413926845	3.036321	0.002735118	0.02359795	−2.044037	FLVCR1	0.372358739	2.595603820	2.705569	0.007446401	0.04786148	−2.935335
971	AK302694	0.08491559	0.09839086	4.215797	3.864839e−05	0.0010563229	1.864066	C12orf53	−0.24565007	0.450163331	−3.473656	0.0006373712	0.008357309	−0.7242430	DQ573754	0.042965370	0.004717991	3.035485	0.002742351	0.02365267	−2.046408	PHF1	−0.306755335	3.964979455	−2.705299	0.007452243	0.04788352	−2.936026
972	ASAH2B	−0.28677802	0.65735776	−4.215019	3.877013e−05	0.0010585600	1.861136	MOCS3	−0.34002505	2.593092855	−3.473275	0.0006382219	0.008364332	−0.7254615	RPA2	0.226459999	4.114058610	3.034995	0.002746602	0.02367700	−2.047798	LTV1	0.200069705	2.907741381	2.705159	0.007455259	0.04788352	−2.936383
973	TRAT1	0.67515836	1.08233881	4.214385	3.886976e−05	0.0010601896	1.858746	CDR2	−0.29953460	3.673853337	−3.472632	0.0006396593	0.008379032	−0.7275165	ZNF490	0.169433783	1.419486046	3.034954	0.002746957	0.02367700	−2.047914	DKKL1	−0.163309644	0.219136230	−2.705110	0.007456316	0.04788352	−2.936508
974	GCSHP3	−0.42506247	2.66750186	−4.213830	3.895713e−05	0.0010614819	1.856654	RAPH1	0.37145170	2.848092833	3.472442	0.0006400849	0.008380471	−0.7281241	IPO11	0.209669113	2.466680060	3.033993	0.002755300	0.02373396	−2.050636	SDHAF2	0.150273706	4.906638203	2.705076	0.007457047	0.04788352	−2.936594
975	TRA	0.50120032	0.81067187	4.211832	3.927327e−05	0.0010681055	1.849126	LAIR1	0.61970491	3.051427503	3.472126	0.0006407922	0.008385594	−0.7291329	TAOK2	0.195110801	3.382069866	3.033987	0.002755353	0.02373396	−2.050653	NCKAP5L	−0.283126766	2.556486542	−2.704855	0.007461830	0.04790264	−2.937159
976	C19orf31	0.34845408	0.67677813	4.211785	3.928072e−05	0.0010681055	1.848950	TRIM16L	0.43665053	1.645698459	3.471935	0.0006412205	0.008387062	−0.7297432	KIF26A	−0.740530254	1.674003692	−3.033788	0.002757090	0.02374121	−2.051219	BC041818	0.009510285	0.004816420	2.704600	0.007467324	0.04792631	−2.937808
977	TALDO1	0.34611980	6.42396508	4.209764	3.960306e−05	0.0010757683	1.841338	LOC100288846	0.22684870	0.952691491	3.471774	0.0006415809	0.008387643	−0.7302565	RELT	0.307126361	1.790532145	3.032624	0.002767240	0.02381736	−2.054516	PYGO2	−0.194680467	4.337533331	−2.704413	0.007471381	0.04794076	−2.938287
978	RNMT	−0.36454131	2.87894010	−4.208538	3.979984e−05	0.0010798246	1.836722	SGK1	0.62650352	3.655188130	3.470857	0.0006436439	0.008409568	−0.7331890	NUP85	0.233850233	3.485265897	3.032568	0.002767729	0.02381736	−2.054675	PTPRU	−0.647380029	2.732342071	−2.703799	0.007484678	0.04800248	−2.939854
979	HIST1H2AJ	0.41551635	0.72604090	4.208327	3.983376e−05	0.0010798246	1.835928	FCHSD2	0.35507463	3.083968467	3.470747	0.0006438917	0.008409568	−0.7335406	SLC25A3	0.222961980	6.667976552	3.032168	0.002771221	0.02382742	−2.055806	AASS	−0.242979272	1.147808793	−2.703758	0.007485564	0.04800248	−2.939958
980	BMP4	−1.03978782	3.92018784	−4.207227	4.001122e−05	0.0010834007	1.831789	HMGCS2	−1.77046793	3.972734134	−3.470225	0.0006450685	0.008418541	−0.7352083	CCNJL	−0.275264196	0.779270889	−3.032135	0.002771514	0.02382742	−2.055901	CCT4	0.201667319	5.925663809	2.703718	0.007486426	0.04800248	−2.940060
981	LILRB4	0.71845678	2.08327074	4.207004	4.004733e−05	0.0010834007	1.830948	FBXO41	−0.45288038	1.639054160	−3.470160	0.0006452132	0.008418541	−0.7354132	DQ594967	−0.242545737	1.855230469	−3.032126	0.002771590	0.02382742	−2.055926	OR2B3	0.026301448	0.010286460	2.703229	0.007497033	0.04805888	−2.941307
982	GANC	0.28252692	1.79113304	4.206315	4.015884e−05	0.0010853110	1.828359	SCIN	−0.69703703	1.762831578	−3.469302	0.0006471527	0.008439699	−0.7381548	SH3GL1P1	0.097895289	0.347416998	3.031766	0.002774744	0.02384467	−2.056946	TSPAN9	−0.366269344	3.165062575	−2.703067	0.007500550	0.04806982	−2.941721
983	MPZL1	−0.32916520	4.97221367	−4.203576	4.060560e−05	0.0010962685	1.818056	RUSC2	−0.43126878	2.830013301	−3.468076	0.0006499317	0.008471776	−0.7420685	PCDHAC2	−0.205244497	0.292370518	−3.031692	0.002775394	0.02384467	−2.057157	AEBP2	0.247998421	2.854094124	2.702249	0.007518308	0.04815429	−2.943804
984	NFKB1	0.30744052	3.89261698	4.202150	4.084008e−05	0.0011014786	1.812694	GPSM1	−0.45299054	1.603445691	−3.466972	0.0006524451	0.008500364	−0.7455937	DEPDC7	−0.431775734	0.879732948	−3.031476	0.002777284	0.02385131	−2.057767	NIP7	0.250042215	3.766179204	2.702228	0.007518777	0.04815429	−2.943859
985	HCG26	0.58658298	1.55412993	4.200312	4.114432e−05	0.0011085575	1.805783	TRIM25	0.35107537	3.697090302	3.466829	0.0006527704	0.008500428	−0.7460489	PISRT1	0.029335402	0.014518047	3.031398	0.002777965	0.02385131	−2.057987	ESPNL	−0.263181877	0.397716675	−2.702209	0.007519174	0.04815429	−2.943906
986	DPF3	0.14236636	0.28948187	4.198994	4.136372e−05	0.0011133385	1.800831	RNF213	0.51605268	4.256985514	3.466241	0.0006541127	0.008513731	−0.7479250	COL6A1	−0.594251738	6.577530340	−3.030989	0.002781554	0.02387440	−2.059146	NUP54	0.185945600	3.710963087	2.701694	0.007530393	0.04820198	−2.945220
987	CSNK1G1	0.25566408	2.03437997	4.197998	4.153018e−05	0.0011166864	1.797091	BC042046	−0.02940116	0.025834277	−3.465783	0.0006551599	0.008523181	−0.7493859	SLC3A1	−0.576354216	1.902940699	−3.030743	0.002783716	0.02388523	−2.059843	IGSF21	−0.206634098	0.568358320	−2.701692	0.007530434	0.04820198	−2.945224
988	STRADB	0.48229237	2.49365600	4.197349	4.163908e−05	0.0011184812	1.794653	BC073897	−0.80321327	4.019945628	−3.464263	0.0006586486	0.008564367	−0.7542359	AFAP1−AS1	−0.922731446	2.159233276	−3.029771	0.002792266	0.02395085	−2.062595	C12orf74	0.104128349	0.251736021	2.701617	0.007532070	0.04820198	−2.945416
989	FAM65A	−0.49038227	3.86536873	−4.193212	4.233957e−05	0.0011345138	1.779119	MYH15	0.09279211	0.137380624	3.463088	0.0006613559	0.008595359	−0.7579819	LMNB2	0.401732534	4.704681791	3.029611	0.002793675	0.02395519	−2.063047	DQ658414	0.270018082	0.697973465	2.701426	0.007536233	0.04820697	−2.945903
990	CITED4	−1.06615869	3.94015886	−4.193100	4.235870e−05	0.0011345138	1.778699	ATAD2B	0.19064214	1.518573056	3.462388	0.0006629750	0.008610253	−0.7602148	RARRES1	−0.865852415	3.697632791	−3.029332	0.002796133	0.02396683	−2.063836	TIMM8B	0.255684880	4.536907277	2.701415	0.007536483	0.04820697	−2.945932
991	SOCS1	0.79160248	3.54957354	4.193068	4.236419e−05	0.0011345138	1.778578	MSH6	0.32206131	3.484541133	3.462254	0.0006632848	0.008610253	−0.7606414	GLIS1	−0.198504782	0.532660502	−3.029252	0.002796838	0.02396683	−2.064063	AX746542	−0.047519266	0.119109589	−2.701264	0.007539773	0.04821639	−2.946316
992	DCLRE1B	0.36468346	2.19637789	4.192484	4.246395e−05	0.0011351775	1.776388	ASB9	0.39510998	1.097944004	3.462171	0.0006634779	0.008610253	−0.7609072	C9orf100	−0.440398491	2.906393212	−3.028961	0.002799409	0.02397618	−2.064887	C17orf51	−0.194000838	1.006040766	−2.701017	0.007545151	0.04823916	−2.946945
993	THEM4	−0.44646531	2.28770984	−4.192422	4.247452e−05	0.0011351775	1.776157	PTPRCAP	1.00343846	4.286373757	3.462032	0.0006637996	0.008610253	−0.7613498	HYI	−0.298374523	3.581453084	−3.028912	0.002799837	0.02397618	−2.065024	FAXC	−0.272809689	0.468227034	−2.700598	0.007554296	0.04828600	−2.948012
994	MS4A7	0.78494700	2.77125761	4.191326	4.266256e−05	0.0011390560	1.772044	DQ588762	0.02775825	0.004513282	3.461723	0.0006645156	0.008615330	−0.7623342	PECR	−0.355695094	2.527222610	−3.028821	0.002800640	0.02397618	−2.065281	ATMIN	0.164958879	3.480603071	2.700487	0.007556723	0.04828988	−2.948295
995	CNTD2	−0.72508938	0.93327602	−4.190233	4.285092e−05	0.0011429351	1.767943	TMEM141	−0.56328341	5.235635270	−3.460787	0.0006666913	0.008639317	−0.7653190	PRLR	−0.552036964	1.066245521	−3.028634	0.002802295	0.02397722	−2.065811	TRIM6−TRIM34	0.187987671	1.423087858	2.700367	0.007559352	0.04829505	−2.948601
996	HOXB5	−0.81007031	2.36678769	−4.189542	4.297025e−05	0.0011449674	1.765354	TCTA	−0.34532845	4.152874017	−3.460592	0.0006671442	0.008640967	−0.7659391	CELSR1	−0.554325725	2.166936091	−3.028577	0.002802802	0.02397722	−2.065974	TRIM2	−0.452632027	3.585558936	−2.699542	0.007577394	0.04839135	−2.950702
997	ZNF311	0.20458348	0.27134670	4.185655	4.364815e−05	0.0011615686	1.750782	APOL2	0.50078914	4.145073939	3.459999	0.0006685255	0.008654634	−0.7678277	MCM8	0.377868042	2.138971098	3.028405	0.002804324	0.02397722	−2.066461	LOC100287482	−0.358227529	0.912502117	−2.699512	0.007578072	0.04839135	−2.950780
998	ZNF37A	−0.31684915	2.18037835	−4.185469	4.368083e−05	0.0011615686	1.750085	AX748181	0.08107174	0.089820286	3.459225	0.0006703330	0.008673801	−0.7702930	SCGB1A1	0.473203068	0.293982521	3.028181	0.002806303	0.02397722	−2.067094	ZNF503−AS1	−0.414783034	0.806765613	−2.699248	0.007583840	0.04839509	−2.951451
999	HLA−DRB5	1.26017793	6.97926630	4.182390	4.422551e−05	0.0011748757	1.738550	SPTY2D1	0.28115249	3.063214308	3.458283	0.0006725405	0.008693200	−0.7732948	LOC554201	0.019938186	0.022435115	3.028137	0.002806691	0.02397722	−2.067218	GPR173	−0.183702501	0.468429573	−2.699187	0.007585178	0.04839509	−2.951606
1000	TOP1P2	0.07619716	0.09366822	4.179108	4.481306e−05	0.0011892938	1.726265	MAFIP	−0.27415385	0.781993567	−3.458226	0.0006726744	0.008693200	−0.7734767	PPP1R2P9	0.024326131	0.013573249	3.028134	0.002806716	0.02397722	−2.067226	KIF11	0.420101045	3.207584879	2.699156	0.007585864	0.04839509	−2.951686

	Gene	logFC	AveExpr	t	P.Value	adj.P.Val	B	Gene	logFC	AveExpr	t	P.Value	adj.P.Val	B	Gene	logFC	AveExpr	t	P.Value	adj.P.Val	B	Gene	logFC	AveExpr	t	P.Value	adj.P.Val	B
1001	CMTM1	0.47205264	2.993496796	4.178169	4.498262e−05	0.001192601	1.722750	GXYLT2	−0.68054619	2.02345203	−3.458166	0.0006728148	0.008693200	−0.7736672	POMT1	−0.278306328	2.728611881	−3.028092	0.002807085	0.02397722	−2.067344	UGT8	0.605764324	2.833292978	2.699152	0.007585951	0.04839509	−2.951696
1002	DQ574306	0.03305656	0.004695953	4.177893	4.503254e−05	0.001192733	1.721718	GINS3	0.41003668	2.13049827	3.457889	0.0006734661	0.008697381	−0.7745506	ILKAP	0.197862805	3.277008378	3.027503	0.002812311	0.02401413	−2.069012	C10orf62	0.032024638	0.034458799	2.698942	0.007590561	0.04841286	−2.952231
1003	KCNH8	−0.43936387	0.501768666	−4.177471	4.510901e−05	0.001193165	1.720138	FAM168B	−0.34926311	4.20521773	−3.457660	0.0006740020	0.008700067	−0.7752768	DQ583348	0.102343737	0.047168792	3.027372	0.002813466	0.02401627	−2.069381	ZC3HAV1	0.199317549	3.558731098	2.698771	0.007594312	0.04842514	−2.952666
1004	ZC4H2	−0.37734362	1.379632709	−4.177307	4.513878e−05	0.001193165	1.719524	LAMP3	0.66657471	2.54610890	3.457457	0.0006744815	0.008702025	−0.7759261	FEM1B	−0.283816698	3.216912996	−3.027122	0.002815684	0.02402747	−2.070088	RTP4	0.442579708	3.384710090	2.698295	0.007604768	0.04848017	−2.953878
1005	FLOT1	−0.39116673	6.118698673	−4.176374	4.530835e−05	0.001196456	1.716034	C3orf37	0.29786640	4.40970082	3.457291	0.0006748716	0.008702751	−0.7764540	XKR9	0.460940399	1.101934127	3.026271	0.002823247	0.02408426	−2.072494	TIPIN	0.317862605	2.490057105	2.698067	0.007609781	0.04850047	−2.954459
1006	TRMT112	0.39428083	6.618113798	4.175834	4.540686e−05	0.001196955	1.714013	PROC	−0.60959489	1.09115979	−3.457154	0.0006751936	0.008702751	−0.7768896	CLDN23	−0.873445598	3.633568194	−3.024903	0.002835448	0.02418058	−2.076363	CCDC79	0.022867429	0.030692062	2.697958	0.007612175	0.04850408	−2.954736
1007	CLDN18	2.45668801	5.753129057	4.175776	4.541743e−05	0.001196955	1.713796	TMEM181	−0.36606625	3.51899580	−3.456684	0.0006763002	0.008712782	−0.7783847	ZNF90	0.313422595	2.701213351	3.023414	0.002848779	0.02428646	−2.080570	BC105019	0.006644132	0.003016489	2.697820	0.007615208	0.04851176	−2.955087
1008	ACADM	−0.47323655	3.652299780	−4.174696	4.561493e−05	0.001200967	1.709758	SAMD3	0.30811453	0.65088954	3.455973	0.0006779801	0.008730186	−0.7806496	MUC6	−1.681418146	3.164712955	−3.023140	0.002851239	0.02429842	−2.081345	SCN3B	−0.168644313	0.327211005	−2.697595	0.007620168	0.04853171	−2.955661
1009	CCDC97	0.33598889	3.893589258	4.173102	4.590789e−05	0.001207482	1.703800	ARHGEF26	−0.62467707	1.33022004	−3.455375	0.0006793949	0.008739746	−0.7825527	TYMS	0.533942428	4.759379384	3.023053	0.002852013	0.02429842	−2.081588	AK096314	0.274264369	0.530668375	2.697423	0.007623959	0.04854421	−2.956099
1010	ASS1	−1.29605533	5.904164452	−4.171689	4.616917e−05	0.001212231	1.698518	C11orf68	−0.28959176	4.63297322	−3.455229	0.0006797392	0.008739746	−0.7830153	USP48	0.236638947	2.973443262	3.022928	0.002853141	0.02430023	−2.081943	KLHL29	−0.234182602	0.990681844	−2.696709	0.007639693	0.04863272	−2.957915
1011	OIP5−AS1	−0.33704328	4.209571260	−4.171632	4.617979e−05	0.001212231	1.698304	ANKRD16	−0.24950015	1.54389751	−3.455176	0.0006798649	0.008739746	−0.7831841	HOXA4	−0.547788819	1.934676732	−3.022719	0.002855015	0.02430839	−2.082532	AFAP1L2	−0.457323204	2.090918960	−2.696579	0.007642571	0.04863937	−2.958246
1012	ZNF8	0.28044036	3.042608478	4.169805	4.651971e−05	0.001219947	1.691479	PTP4A2	−0.28058498	5.79675535	−3.454871	0.0006805895	0.008739746	−0.7841566	HTT	0.268717383	3.136372576	3.022260	0.002859149	0.02433578	−2.083829	AGA	0.261390894	2.868170298	2.696244	0.007649961	0.04866520	−2.959098
1013	PSMD9	0.28317050	3.828570420	4.167753	4.690438e−05	0.001228821	1.683816	COL1A2	−0.77040171	8.15598170	−3.454867	0.0006805990	0.008739746	−0.7841694	INPP5A	−0.357867092	3.587639384	−3.021870	0.002862660	0.02435725	−2.084930	DQ578778	0.022241120	0.004847025	2.696229	0.007650296	0.04866520	−2.959136
1014	CDX1	−2.21492756	3.075596593	−4.166426	4.715482e−05	0.001234163	1.678861	CUL1	0.22582297	3.93483997	3.454825	0.0006806984	0.008739746	−0.7843026	CNIH4	−0.263010430	3.173112105	−3.021776	0.002863508	0.02435725	−2.085196	AK021933	0.142338319	0.457243996	2.695841	0.007658878	0.04870811	−2.960124
1015	TMPO	0.49663526	4.357666714	4.164577	4.750592e−05	0.001242128	1.671958	LOC149837	−0.35348706	0.78140322	−3.454614	0.0006811986	0.008741939	−0.7849732	RASSF10	−0.668438113	1.260174221	−3.021239	0.002868349	0.02439062	−2.086711	GNPTAB	−0.202621555	2.990993171	−2.695359	0.007669548	0.04876429	−2.961350
1016	BC070363	−0.72330050	2.299498885	−4.163653	4.768213e−05	0.001245508	1.668513	GM2A	0.48279787	4.50946871	3.453208	0.0006845433	0.008780617	−0.7894446	CD53	0.728869149	4.506133287	3.020752	0.002872750	0.02441165	−2.088086	DKK3	−0.502761660	4.643075415	−2.694091	0.007697658	0.04892220	−2.964572
1017	HABP2	1.43221228	2.392611700	4.162768	4.785176e−05	0.001248710	1.665209	LYSMD1	−0.27569078	2.01070706	−3.452824	0.0006854600	0.008787623	−0.7906662	LPCAT1	−0.495300283	4.096398157	−3.020735	0.002872909	0.02441165	−2.088136	NUDT22	−0.230150442	4.110847165	−2.694073	0.007698071	0.04892220	−2.964619
1018	ZNF550	−0.40237828	1.251068090	−4.162029	4.799367e−05	0.001251183	1.662454	CCDC18	0.22915616	1.12147223	3.452549	0.0006861176	0.008787623	−0.7915416	TTC17	−0.233496089	3.302540360	−3.020660	0.002873582	0.02441165	−2.088346	CCDC138	0.275986477	1.392324260	2.693910	0.007701687	0.04893346	−2.965033
1019	AX748339	0.35565600	0.955649982	4.161699	4.805726e−05	0.001251335	1.661222	PIH1D2	0.25499087	1.15471715	3.452521	0.0006861850	0.008787623	−0.7916312	OSTBETA	−0.546765696	1.119409041	−3.020346	0.002876429	0.02442801	−2.089234	RPS15	0.268241295	9.216911505	2.693807	0.007703973	0.04893627	−2.965294
1020	HOXB3	−0.72729840	2.455143205	−4.161509	4.809380e−05	0.001251335	1.660514	ZNF831	0.29779550	0.43253345	3.452425	0.0006864140	0.008787623	−0.7919359	PLEKHA7	0.395452648	2.747016013	3.019938	0.002880123	0.02444752	−2.090385	SEC14L4	−0.467757221	0.726687219	−2.693613	0.007708301	0.04895204	−2.965789
1021	SLC31A2	0.45342610	2.562875744	4.161242	4.814539e−05	0.001251450	1.659517	ZNF740	0.25974051	2.72889635	3.451988	0.0006874589	0.008796755	−0.7933243	CHD3	0.396101607	4.029720244	3.019889	0.002880568	0.02444752	−2.090523	GNA15	0.452138278	2.311363999	2.692940	0.007723285	0.04903132	−2.967499
1022	JPH1	−0.90794777	1.721127992	−4.160961	4.819958e−05	0.001251633	1.658470	ADCK3	−0.41951930	3.38443169	−3.451587	0.0006884201	0.008802338	−0.7945997	HIF1AN	0.242113502	2.856483487	3.018741	0.002890988	0.02452811	−2.093762	ZNF749	0.210753826	1.423443472	2.692886	0.007724480	0.04903132	−2.967635
1023	GNLY	0.92849986	2.645295878	4.160576	4.827400e−05	0.001252340	1.657035	PRKAB2	−0.40992122	2.62439040	−3.451424	0.0006888114	0.008802338	−0.7951185	AF119915	0.069096116	0.198191490	3.018605	0.002892228	0.02453079	−2.094146	THOC3	0.323138419	2.696617364	2.692519	0.007732674	0.04907160	−2.968569
1024	KIF2A	0.42266301	3.102090947	4.159999	4.838578e−05	0.001254014	1.654883	SRPK3	−0.52611808	0.98394665	−3.451156	0.0006894539	0.008802338	−0.7959695	PGD	0.383330902	5.820221038	3.018446	0.002893673	0.02453520	−2.094594	ZNF547	0.213348322	1.605348200	2.692372	0.007735944	0.04907744	−2.968941
1025	RNF208	−0.70750070	2.723835257	−4.157657	4.884182e−05	0.001264598	1.646155	OR8D4	0.02304585	0.01022392	3.451047	0.0006897170	0.008802338	−0.7963177	HOXB−AS3	−0.602001940	2.017168174	−3.017806	0.002899500	0.02456732	−2.096398	LRP4	−0.529366672	1.555998906	−2.692312	0.007737293	0.04907744	−2.969095
1026	TNFSF18	0.41571813	0.467888557	4.157085	4.895396e−05	0.001265384	1.644022	AK092451	0.05593662	0.10727543	3.451026	0.0006897664	0.008802338	−0.7963831	SERPINB2	0.693632801	0.823977249	3.017772	0.002899812	0.02456732	−2.096495	TCF20	0.256947780	3.653129797	2.691149	0.007763295	0.04923060	−2.972049
1027	CLEC4A	0.44825199	1.432434145	4.157015	4.896755e−05	0.001265384	1.643763	KRCC1	−0.45702284	3.56372039	−3.450902	0.0006900635	0.008802338	−0.7967762	ARTN	−0.341519340	0.615693522	−3.017725	0.002900238	0.02456732	−2.096626	MIR1470	0.058921420	0.045491888	2.690266	0.007783087	0.04934433	−2.974291
1028	ANP32AP1	0.06141706	0.070038581	4.155856	4.919550e−05	0.001270038	1.639442	PCSK1N	−1.58536299	3.02154629	−3.450839	0.0006902168	0.008802338	−0.7969790	RNF186	−0.916295987	2.018076249	−3.017376	0.002903426	0.02458648	−2.097611	DEFB110	0.013463897	0.002560749	2.690161	0.007785451	0.04934752	−2.974558
1029	TRMT12	−0.35856366	2.407685790	−4.154643	4.943495e−05	0.001274980	1.634925	MEGF6	−0.46583078	1.96271970	−3.450054	0.0006921053	0.008822182	−0.7994730	IGFL3	0.175305370	0.161735754	3.017216	0.002904888	0.02459101	−2.098063	QRFP	0.149923374	0.306071664	2.689986	0.007789373	0.04935225	−2.975002
1030	PRKAG2	−0.37772868	1.934802822	−4.154160	4.953055e−05	0.001276205	1.633128	CGREF1	−0.59303993	2.27103486	−3.449605	0.0006931873	0.008831732	−0.8008989	HMMR	0.519046533	2.797419851	3.015785	0.002917984	0.02469399	−2.102096	ZNF498	0.181224302	1.527998356	2.689962	0.007789915	0.04935225	−2.975063
1031	CDC25A	0.64674166	1.852520709	4.152971	4.976683e−05	0.001279863	1.628701	DKFZP434I0714	0.20128217	0.90367175	3.449337	0.0006938345	0.008835736	−0.8017507	ST8SIA6	0.186552944	0.262912637	3.014824	0.002926817	0.02476086	−2.104806	SCARNA7	−0.624484111	2.695184971	−2.689734	0.007795024	0.04937282	−2.975640
1032	HELLS	0.55445174	2.321437265	4.152961	4.976897e−05	0.001279863	1.628661	EFCAB7	0.22547739	1.49765660	3.448982	0.0006946927	0.008838540	−0.8028789	STON2	0.313952256	1.189796335	3.014339	0.002931275	0.02479067	−2.106171	DQ588228	0.015069144	0.001705384	2.689433	0.007801801	0.04940396	−2.976406
1033	ARL6IP6	0.37564916	2.893907519	4.152548	4.985112e−05	0.001280735	1.627126	CACHD1	0.47189933	1.44530981	3.448970	0.0006947207	0.008838540	−0.8029158	FAM43B	−0.160827583	0.316630240	−3.014206	0.002932505	0.02479317	−2.106547	C17orf75	0.216338437	1.823397073	2.689337	0.007803961	0.04940585	−2.976649
1034	C5orf42	−0.44776488	1.132922634	−4.149631	5.043633e−05	0.001294516	1.616270	CCDC125	0.26995743	1.43022162	3.448093	0.0006968460	0.008861331	−0.8057038	FBXO32	−0.721431441	3.261352361	−3.013848	0.002935802	0.02480845	−2.107555	IQGAP3	0.460746962	3.330475531	2.689174	0.007807626	0.04941726	−2.977063
1035	CD3E	1.02948341	3.379772853	4.149173	5.052897e−05	0.001295641	1.614563	DDX23	0.24920963	5.05871922	3.447557	0.0006981467	0.008873618	−0.8074057	CEP170P1	0.045749219	0.043521643	3.013807	0.002936182	0.02480845	−2.107671	CLEC6A	0.120620694	0.158471857	2.688938	0.007812948	0.04943916	−2.977663
1036	ZAP70	0.69485279	1.375626299	4.148650	5.063472e−05	0.001297099	1.612618	PSMB8	0.52667877	5.53170893	3.446559	0.0007005738	0.008898498	−0.8105732	NUBP2	0.200453111	4.796801503	3.013069	0.002942994	0.02485809	−2.109748	CYP11B1	0.006625814	0.005647737	2.688116	0.007831465	0.04953653	−2.979747
1037	MIR655	0.02204410	0.003350662	4.146959	5.097825e−05	0.001304568	1.606329	PIP4K2C	−0.39145351	4.14026285	−3.446477	0.0007007747	0.008898498	−0.8108348	TRIM64B	0.010531994	0.003123038	3.012447	0.002948753	0.02489881	−2.111501	FBXO22−AS1	0.262907100	3.427332700	2.688089	0.007832069	0.04953653	−2.979815
1038	HOTAIRM1	−0.67067570	2.207173492	−4.146732	5.102457e−05	0.001304568	1.605484	ZNF223	0.28865309	1.24645896	3.445619	0.0007028684	0.008920815	−0.8135574	MYO19	0.338290649	3.220875933	3.012221	0.002950849	0.02490858	−2.112138	TPRG1L	−0.228814468	4.886113728	−2.687951	0.007835185	0.04954443	−2.980165
1039	SIGLEC10	0.77168522	2.096884811	4.145666	5.124236e−05	0.001308875	1.601522	DEDD	−0.19855674	4.11935462	−3.445321	0.0007035974	0.008925798	−0.8145035	OR7D4	0.027619436	0.010788165	3.010663	0.002965317	0.02500020	−2.116522	ORC6	0.352894100	1.640741458	2.687736	0.007840048	0.04956337	−2.980711
1040	FNDC4	−0.65554057	1.988035622	−4.145422	5.129246e−05	0.001308895	1.600614	LOC254559	0.10430772	0.19529623	3.445135	0.0007040533	0.008927315	−0.8150946	FAM126A	−0.412250263	1.953584020	−3.010623	0.002965693	0.02500020	−2.116636	OR4B1	0.009403717	0.003299523	2.687618	0.007842715	0.04956842	−2.981010
1041	CENPM	0.65250018	2.637767661	4.143192	5.175152e−05	0.001319277	1.592326	HAUS5	0.37157654	2.21660510	3.444966	0.0007044672	0.008928298	−0.8156310	KRTAP27−1	0.032139177	0.009683365	3.010481	0.002967011	0.02500020	−2.117034	SPATA20	0.286263409	3.865966438	2.686992	0.007856880	0.04964352	−2.982599
1042	MBD4	0.30963322	3.566212840	4.142964	5.179874e−05	0.001319277	1.591478	DUOXA1	−0.78848990	1.34641521	−3.444690	0.0007051448	0.008929208	−0.8165084	BC038576	0.046308236	0.042055765	3.010468	0.002967136	0.02500020	−2.117072	DPM3	−0.407863944	5.157198125	−2.686927	0.007858337	0.04964352	−2.982762
1043	BC040631	0.16642176	0.313075969	4.140055	5.240395e−05	0.001333412	1.580674	UBE2NL	0.17491017	0.39729955	3.444662	0.0007052120	0.008929208	−0.8165953	LOC100506172	0.008753801	0.005275559	3.010448	0.002967314	0.02500020	−2.117126	SFXN2	0.369737274	2.661324377	2.686710	0.007863268	0.04966284	−2.983314
1044	DZIP3	0.28827975	1.356742263	4.138550	5.271976e−05	0.001340163	1.575087	SLC35B4	0.20500639	1.88418866	3.444433	0.0007057743	0.008931614	−0.8173227	DNMT1	0.374915308	3.975224456	3.010444	0.002967355	0.02500020	−2.117138	SNORD88C	0.307384874	0.990654766	2.686363	0.007871123	0.04970063	−2.984193
1045	ARF3	0.30817910	5.984395008	4.137004	5.304620e−05	0.001346390	1.569346	C16orf58	−0.30810024	4.25420690	−3.444311	0.0007060750	0.008931614	−0.8177115	TRNA_Cys	0.024670448	0.092523836	3.009722	0.002974085	0.02504895	−2.119169	KRT16P2	0.172442964	0.126559801	2.686170	0.007875490	0.04971637	−2.984682
1046	PFN2	−1.08663062	4.059701817	−4.136909	5.306621e−05	0.001346390	1.568995	COQ6	−0.19342551	2.49631475	−3.443929	0.0007070129	0.008939217	−0.8189229	SS18L2	0.282072059	4.153403983	3.009522	0.002975957	0.02504974	−2.119733	GIMAP7	0.559627695	3.323062616	2.685400	0.007892983	0.04981495	−2.986635
1047	TMEM194B	0.33778210	1.323431902	4.136597	5.313228e−05	0.001346779	1.567838	KCNH3	0.33755927	0.40964479	3.442175	0.0007113369	0.008989605	−0.8244870	PCDHB3	−0.289383927	0.619111664	−3.009408	0.002977023	0.02504974	−2.120054	ANP32C	0.075987494	0.257875164	2.685223	0.007897011	0.04982852	−2.987084
1048	FZD1	−0.56153752	3.231887387	−4.136339	5.318703e−05	0.001346880	1.566880	USP43	0.46172931	1.82260458	3.441190	0.0007137755	0.009016130	−0.8276099	PITX2	−0.288227456	0.339161356	−3.009353	0.002977530	0.02504974	−2.120207	HIST1H2BN	0.347762122	1.386943018	2.685133	0.007899046	0.04982952	−2.987310
1049	FAM174B	−0.81690054	2.648857883	−4.134848	5.350433e−05	0.001352368	1.561349	SPIRE1	−0.53865440	2.16826958	−3.439992	0.0007167536	0.009044320	−0.8314090	HIST2H3D	0.149853446	0.259175235	3.009308	0.002977954	0.02504974	−2.120334	BHLHE41	−0.563746032	2.969305425	−2.684883	0.007904731	0.04985353	−2.987944
1050	BCAS4	−0.70043096	2.219097784	−4.134696	5.353697e−05	0.001352368	1.560782	HMOX1	0.62774735	4.49561540	3.439930	0.0007169072	0.009044320	−0.8316046	MAZ	0.271667175	5.724570501	3.009181	0.002979143	0.02505180	−2.120692	TGS1	0.242315578	3.246597123	2.684491	0.007913675	0.04989808	−2.988939
1051	PASK	0.32426326	1.402673801	4.134604	5.355661e−05	0.001352368	1.560441	CCDC48	−0.40044273	1.38443251	−3.439739	0.0007173839	0.009044320	−0.8322111	TMPRSS4	0.742263008	4.877636264	3.008763	0.002983055	0.02507675	−2.121869	KIAA1432	0.245372908	2.141714037	2.684373	0.007916353	0.04990311	−2.989237
1052	PNPLA2	−0.42067153	5.920071065	−4.133637	5.376343e−05	0.001356300	1.556857	NT5DC1	0.27071378	3.13366021	3.439667	0.0007175630	0.009044320	−0.8324388	KIAA0020	0.324175686	3.646077030	3.008176	0.002988553	0.02511501	−2.123519	C1orf56	−0.236588532	2.498593193	−2.684290	0.007918242	0.04990316	−2.989447
1053	CD6	0.79063198	2.180421886	4.132445	5.401962e−05	0.001360670	1.552437	HK1	−0.43359799	4.35156231	−3.439607	0.0007177112	0.009044320	−0.8326273	BC032915	0.042361028	0.041393507	3.007654	0.002993454	0.02514824	−2.124988	FRMD4B	0.305668510	2.140949845	2.683583	0.007934384	0.04999302	−2.991239

Supporting Material 6B: EBV−HEMG signature

	EBV.HEMG
1	MLYCD,C1orf115,ECHDC2,SLC6A8,SCNN1A,HSD17B12,DNAJA4,ACPL2,PPP1R1B,DUSP23,PPIC,MAP1LC3A,PHYH,ZNF480,NMNAT3,KCNK6,CAMK2N1,TP53I13,FAM71E1,WNK2,SARM1,ZNF32,CERK,GOLT1A,DSC2,LZTFL1,ZNF669,PPARA,NMB,TMEM117,
2	HOXA10,CISD1,DAZAP2,FBXO44,PDE4A,SLC46A1,B3GALT4,TCEA3,PPIE,DBN1,BMP8B,ZNF416,NOL3,FUT6,TPD52L1,YBX2,HOXA6,RAB11FIP5,PODXL2,CDH17,FAM114A1,C10orf116,FUT3,A2LD1,NINL,NDRG1,RCOR2,UBXN10,CDX2,RIPK3,
3	TTC22,PCYOX1,MAPRE3,FAM188A,C9orf129,F12,BAIAP2,RAB36,RHOD,KBTBD10,HYLS1,FARP1,C2orf70,ANXA5,LOC148189,MFSD6L,FAH,PKN3,B3GALNT1,LOC440335,IRS2,FN3K,FAM174A,PRKCDBP,TFF3,MAPK14,SLC22A3,MT1G,C1QTNF1,HOXB7,
4	KRT7,AMIGO1,SCRN1,PLOD2,MATN2,LDLRAD3,ARL13B,CDKN1C,TMEM65,LTBP3,CHRM3,ADM2,SNTA1,C7orf41,SLC44A4,C2CD4B,HOXA5,NELF,PRMT6,EPPK1,PPP3CA,OSBP2,LYRM2,CIB2,ULK2,HSPB1,OBSL1,CCNI2,HOXB9,ZXDB,
5	ESPN,DSG2,GRIN2D,SULF2,GGT6,HES4,CHPF,MGA,HOXC11,HEPH,NKD2,MYO7B,C4orf48,IGSF8,KCNK15,CCDC149,FADS3,FBXO2,GPR162,LEPRE1,ProSAPiP1,TRPM2,RHOF,ZNF91,TTC14,SH3PXD2A,NR1I2,KHDRBS3,FAIM,C19orf43,
6	ENTPD8,HES6,SEPN1,LOC388588,NHLRC1,ANKH,LONRF3,PINK1,ZNF718,SHF,PTGS2,SNX33,TMEM220,ESD,NEURL1B,HOXC10,TMEM168,LRRC8E,PLEKHG3,FLYWCH2,CDCA7L,RIMKLA,CXorf26,GLB1L2,ICA1,ABTB1,NUDT19,P4HA2,GPR153,PIGZ,
7	HDAC11,FAM59B,DPP4,SLC25A4,FAM3B,GSTP1,TPBG,BAHCC1,MUC20,KBTBD11,FAM84A,PLA2R1,ARRDC4,CERCAM,PPP2R5B,SLC4A3,RNF144A,AIFM2,ABCD1,SLC36A4,ALG1L,SHISA4,ACAA2,EMP2,VWA2,TRAPPC9,ZNF514,ZNF185,C16orf5,MAPK15,
8	FAM109B,ACHE,ACY1,PAOX,UNC45A,KLF7,MAGED1,LRRC6,FLJ90757,IL28RA,LAPTM4B,PLK1S1,TMEM52,SCMH1,LPCAT2,WFDC2,GUCY2C,LRRN1,PLCD1,PPP1R14D,SKAP2,SLC16A10,KIAA1383,TMEM9,CLRN3,COBL,ST6GALNAC4,HOXA11,LARP6,RIOK3,
9	KLF11,ASAH2B,BMP4,MPZL1,FAM65A,CITED4,THEM4,CNTD2,ZNF37A,KCNH8,ZC4H2,FLOT1,ACADM,ASS1,CDX1,HOXB3,JPH1,RNF208,TRMT12,PRKAG2,C5orf42,FNDC4,PFN2,FZD1,FAM174B,BCAS4,PNPLA2,FAM50B,MID2,MYO1C,
10	SLC16A4,KLK1,TMEM120B,THNSL2,MXRA7,FOXO3,PRRX2,PDLIM4,C1orf88,CA9,PIGM,VANGL2,SERINC2,ZXDA,EPDR1,SLC25A29,TMEM171,ZBTB7B,PLIN2,ESYT3,MAGIX,SIGMAR1,MIR196A1,CRABP2,PTK6,SNX21,GNG12,MIR196B,GPC1,SEMA6A,
11	ERN2,SLC6A20,FOXJ1,TBX3,NPW,WDR72,SLC20A2,OSBPL5,PDE9A,DTD1,C2orf65,ASCL2,LOC389332,SLC25A23,KLHL35,RAB6B,MLXIPL,MFAP3L,PROS1,TMEM192,RAB34,BMP7,SCARA3,MAPK8IP1,FABP1,TM4SF20,TPPP3,ARHGEF10,CACNG4,SELM,
12	TMEM55A,ABHD11,ISYNA1,DUOX1,ADM,NSUN5,ANKRD9,TM6SF2,MEIS3P1,BAI2,QPRT,CTDSP1,ADAMTS17,KCTD11,SHC1,SPIN3,GFPT1,ADRA2C,B3GNT8,SLC7A4,JAG2,CCDC24,APCDD1,EPHX1,ZNF649,HOXA13,CTSF,CXorf61,TUBB6,PURA,
13	TDRD5,GLOD5,SETD7,CUTA,SLC38A1,MFAP2,PRDM5,FAM3A,CD276,DNAJC4,TRIM68,BEGAIN,RANBP17,BCL2L10,HDAC5,ILVBL,SMAD5,ROCK1,BNIP3,TMEM61,KCNH2,FBXL2,VEGFB,EIF3L,SLC16A2,MIB2,DIP2C,ILDR1,UNC93B1,TEAD2,
14	MT1L,PHGDH,ADIPOR1,SLC29A4,PAK6,RBP4,NPNT,SLC39A13,FOXD1,ANPEP,EGFR,RAB15,HSPA1L,LRRC58,SOBP,TIMP2,LTK,SHC2,NT5C3L,GNAI1,ST6GALNAC2,SNX19,TFAP2C,CAPN9,C5orf62,KRBA1,ACSS1,MARVELD1,KISS1,CTF1,
15	KCNIP3,BRD3,EIF2C4,ABHD8,LRRC3,DACT1,DYM,ITM2C,SNED1,PTGR1,LRRN4,GBE1,CKB,MMP17,HLTF,LARP4,MAMSTR,KCNQ4,INF2,ZNF841,CILP2,SLC39A4,PHKA1,MGST3,MT1M,FAM20C,NOV,WNT7B,SYT7,PLCB1,
16	PLEKHG5,NPTXR,OSR1,NEK3,CYP2R1,HPS1,PDZRN3,ZNF844,KIAA1586,MSRA,PPP1R9A,STMN3,MIB1,PITRM1,GPRIN1,MMP2,OGDH,ARMCX3,PF4,TFR2,DST,TP53INP2,RTN4,WTIP,ABLIM2,SMOC1,TLCD1,F10,MSI1,BCAM,
17	COL16A1,PID1,KIF7,HECTD3,ESYT1,BDKRB2,PXMP4,PLD2,WISP3,FAM171A2,C8orf47,C16orf45,LPPR2,ABHD15,GPR158,PGM1,SH3PXD2B,REEP6,CDR2,HMGCS2,FBXO41,SCIN,RUSC2,GPSM1,GXYLT2,FAM168B,PROC,TMEM181,PTP4A2,COL1A2,
18	LOC149837,SRPK3,PCSK1N,MEGF6,CGREF1,C16orf58,SPIRE1,CCDC48,HK1,RGN,PYROXD1,ALS2CL,ARPC1B,AFG3L2,RPL39L,PRPF40B,PPP1R11,HRH1,APLP1,ME1,GATA2,METRN,LPAR1,LOC645166,PPL,ENPP5,ASAP3,ENDOD1,TSPAN18,ABCC6P2,
19	NMU,RALBP1,BCKDHB,PTP4A3,FMO5,WIPI1,PRSS21,FGF18,TNNC1,NFATC4,ZNF530,TNFRSF10D,G0S2,VWA1,SULT1A2,IGFBP4,WWTR1,SLC34A2,C20orf195,RXFP4,OLFM1,BAG3,CCNE1,SLC25A27,COL1A1,MOCS1,COL5A1,MYOM3,LZTS2,TBCEL,
20	CRABP1,C9orf24,FAM132A,SLC9A3R2,PBXIP1,ZNF702P,GNAL,OPN3,DCHS1,SATB1,PARM1,CHST5,SERP2,PLXNB3,WNK4,SPON1,SACS,MT1H,AGPAT1,PCDH20,PPP2R3A,BCORL1,IAH1,ETNK2,ANO7,ARHGEF4,HOMER2,RILPL1,FBLN1,NRSN2,
21	IL17RD,KIF12,KIAA1598,ERO1LB,C4BPB,FADS1,RAB3IL1,WBSCR27,ST3GAL4,ITGA5,KBTBD7,TMEM151A,MYH10,DPYSL3,SNAPC2,ADRA2A,MGLL,SMO,FAM103A1,CYP2W1,CHP2,NR3C2,RASGEF1A,EGR4,C6orf123,NPM2,TNFRSF11B,OXT,ORAI3,CAV2,
22	PLA2G4F,TM4SF4,YPEL3,SYT1,TRAF5,FAM131C,SLC25A34,HSPA12A,ARVCF,TGFBI,PRNP,ULBP3,MPP6,PDGFRL,BEX5,COL9A3,NACAD,SIX2,LIPE,VSTM2L,KLK10,MAPK3,RASA3,VWA5A,NEIL1,KCNK9,ARHGDIG,H2AFY2,TPM2,GRAMD4,
23	MAGED2,HAPLN4,TRIM54,EFNA1,TSPAN7,AATK,MESP2,CHRD,TMEM158,TMEM231,MXRA5,NT5DC3,SEMA4F,NR0B2,TTC28,AHNAK2,IGSF3,GABRB3,TNRC18,PHGR1,PCOLCE,SCARF2,FXYD4,WFS1,PKIG,PAQR5,MX2,XYLT1,ERGIC1,PXDN,
24	ECE1,GALNT3,DNALI1,AKR1C4,EPHX4,SCG5,FKBP9,CAPS,TMEM2,C3orf70,STK32C,FAM171B,CLU,CNPY4,MEF2D,MYRIP,KCNMB4,RMND5A,KLHL24,GRINA,GGT1,NGFRAP1,ULBP2,NEU4,MEIS3,HOTAIR,UCHL1,LEFTY1,RGS11,FAM92A1,
25	TSPAN2,MARK1,KIF26B,MAP4K2,CCDC107,DGKD,ANGPTL4,CHST6,HOXA2,GGT7,ARSI,ATE1,SCUBE2,LOXL2,C21orf88,ULK1,SDC2,PLSCR4,NBEA,SULT4A1,PLEKHB1,CHN1,CCL28,CHST10,SH3RF3,KIF26A,CCNJL,DEPDC7,COL6A1,SLC3A1,
26	RARRES1,HYI,PRLR,CELSR1,POMT1,FEM1B,CLDN23,MUC6,HOXA4,INPP5A,RASSF10,LPCAT1,OSTBETA,ARTN,RNF186,FBXO32,FAM126A,PITX2,NRTN,ADAMTS9,HOXA1,ZHX2,SEMA4C,ERLIN2,MSX1,MESP1,SLC7A9,GAL,PRKAB1,MAFK,
27	AKR7L,NANOS3,COL12A1,TGFB1I1,ARHGAP6,FRG1B,IGFBPL1,EPHB1,YES1,ZNF141,CCDC74A,PAQR7,SCARB1,DIRAS3,RHOBTB3,OLFM2,PLA2G16,ARHGEF17,FAM83F,FSCN1,KLHDC9,PON1,BACE1,CD109,FAM127C,DPEP1,RNASET2,TRAM2,MRI1,EEF1A2,
28	C11orf53,CAP2,PELI3,ALDOC,TPST1,PRKAA2,GJB5,KCNJ3,SRPX,PYCR2,PBX1,ZFP3,LIMCH1,ZIC5,ASB13,P2RY2,MXI1,FOXF2,MICAL2,HOXB13,MYEF2,CADPS,MUC12,ABLIM3,FGD1,SFRP4,SATB2,PTPRN2,TRIM47,RET,
29	TNNC2,MME,SDK2,MYO18A,CENPB,HOXB8,EHD2,MXRA8,BRSK2,PTRF,CCDC136,NUP62CL,H19,FJX1,PPP1R3G,SLC26A11,MCF2L,IER3,RORC,DCBLD2,C10orf125,FIBCD1,SV2A,PCBP3,FAM19A5,PALM,CYP2B6,ZNF467,SRMS,GMPR,
30	ZNF615,GALNTL2,DIRAS1,COL6A2,WNT5B,NAPG,B3GNT9,NQO2,FAM176A,RAB3B,SEMA6C,DACT2,FGFR1,SLC35B2,ACE,SELS,GCNT3,B4GALNT4,ANTXR2,DAPK3,STARD4,ALPI,AS3MT,NR4A1,TMX4,ADRA1D,CLGN,KIFAP3,ITGA7,CTBP2,
31	ZNF577,KLHDC5,CCDC90A,ZNF274,MAPK8IP2,SSBP2,KRT17,SALL2,DSEL,DACH1,APOOL,C14orf132,SHISA2,NOTUM,ARMCX2,DFNB31,MEF2A,SMAD9,XPNPEP2,CACNA2D2,ATP2C2,MUC2,FKBP1B,GLIS2,ROCK2,FSTL3,TIGD5,TNFAIP8L1,L1CAM,TNNI3,
32	KIAA1324L,COL6A3,MMP24,KDELR3,EFCAB4B,KLC4,NT5E,KRT20,GNE,TFCP2L1,UAP1L1,ORM1,PTGER1,ZSWIM4,MRGPRF,NUAK2,COX6B2,RGMB,PFKFB2,GOLGA8B,LRRC10B,FAM110C,PTPN18,FAM3D,HIP1,SH3BP4,APBB1,RARRES2,VIPR1,CARD14,
33	SLC16A9,ELN,CBX5,ZNF827,ABHD14A,SERPINF1,ARHGEF10L,FBLN2,PCLO,GALNS,EGFL7,KCNK3,GDPD5,CIDEC,TUBA4A,C1orf198,BGN,ADAMTSL4,CSDE1,PHF1,PTPRU,TSPAN9,ESPNL,TRIM2,AFAP1L2,DKK3,SEC14L4,LRP4,DPM3,BHLHE41,

Supporting Material 6C: EBV−HOMG signature

	EBV.HOMG
1	ZNF683,FCRL6,IFNG,ATP2C1,FASLG,GZMH,ITGAE,CD244,ATP2A1,DDB2,SIGLEC8,JAKMIP1,CASP4,FAM115C,CD101,RTBDN,CRTAM,GBP5,IL18RAP,XCL2,TBX21,SYCE2,GZMB,DNAJC5B,PRKCQ,KLRK1,APOC1P1,SLC26A9,HAPLN3,BAX,
2	LOC400043,SLA2,TIFAB,LAP3,C5orf20,XCR1,FAM26F,IDO1,CLEC12A,UBE2L6,CD8A,RCL1,KLRD1,ARHGEF3,OR56B1,THEMIS,DPY19L1,GNGT2,HAS3,LAG3,GBP4,CCR5,PRKCG,ACAD8,IRF1,GFI1B,CTSC,FDXR,LTA4H,RASGEF1B,
3	C19orf38,GPR25,ADAMDEC1,CCL5,IL18BP,JAK2,SUCNR1,FCGR1A,B2M,SIDT1,MDM2,NKG7,SESN2,PSMB10,IKZF2,ACER3,CD226,C4orf36,GBP1,IL21,C5orf56,C14orf105,CD40,UBASH3A,HPS5,APC2,SLC9A7,KDELC2,APOL4,CD274,
4	AMH,SCML4,SLFN12L,VRK2,TRIM24,SUSD4,FHDC1,APOL6,UBE2Z,COL22A1,PSPC1,CD200R1,SFMBT2,PAPD4,POLQ,DUT,ANKRD24,ZNF793,ETV7,TRIM26,HNRPLL,GOLPH3,ALDH16A1,GRAP2,GZMA,CD8B,STAT1,ZNF434,SYNE2,TMEM86A,
5	SMTNL1,PRF1,CD38,ADORA3,EI24,ZFYVE28,ACPP,CD3G,BBC3,CD74,APOL3,KLRC1,C1QA,C1QB,ZNF267,SLC35D2,CASS4,CX3CL1,ZNF222,BRIP1,IL12RB1,EED,GABBR1,PRPS2,HOXC4,ITGB7,LGMN,SIRPG,PCNA,PVRL1,
6	RAB33A,ZDHHC18,GFI1,MCM5,GTPBP8,KIAA0101,CACNG8,PLCH1,NRAP,CIITA,CHPT1,BCL2L11,PSMA1,TFEC,MTF2,C4orf21,STAT4,GABPB1,C11orf93,LSM12,TRIM10,KDM2B,SAMHD1,MEI1,GAB3,RASGRP1,NCR1,TMEM101,MLLT3,H2AFX,
7	RQCD1,PTPN22,EIF4E,LACTB,PLCXD2,ADAM28,SCAND3,SPATS2,UBD,NUP35,PDCD1LG2,PRR22,APOBEC3F,BUD13,RRM2B,TAP1,AEN,SYTL3,SLAMF8,CPOX,HNRNPD,PYHIN1,ZNF226,MMP25,ALG9,RPS6KB2,PRAM1,HAT1,WDFY1,PIK3R6,
8	SLC38A9,BTN2A2,PPP2R2B,ZCCHC8,TNFSF14,FAM110A,C12orf75,PSME2,CLEC7A,RPE,CBX2,HLA−DQB2,EIF4A1,AOAH,PSTK,ATR,C1QC,IRAK3,HPRT1,CD47,ACAD9,CCDC78,ALOX15B,IL2RA,VSIG8,UNC5CL,KCTD5,NUDC,IL2RB,SNRPA,
9	PLA2G2D,FCGR3A,IL15RA,MS4A6A,ZNF286A,GAD1,ATIC,TMEM218,C8A,RPS19,TTC12,NECAP2,CTLA4,TAP2,NMI,CD2,HDAC1,HLA−DPA1,RARRES3,NFAM1,HLA−DOA,TMIGD2,ARHGAP19,ARPC3,TNFSF13B,MRPL44,ATP11C,PLA2G7,IL29,EIF3M,
10	PLA1A,GTF2B,IL18,LIG1,CD3D,MPHOSPH6,PANK2,QSER1,CLUAP1,CLP1,GCA,ICOS,RCN1,C6orf211,HLA−DRA,HIST2H2AB,SNRPF,IL21R,FAM122C,MOV10,BIRC3,CD80,ASCC3,AGFG2,DOK2,HAVCR2,MCM3,ANKMY1,ZNRF1,AMICA1,
11	E2F7,HLA−DPB1,FAM117B,EXOC6,AMOT,DEPDC1B,HLA−DMB,RFFL,MTAP,CABIN1,SCO1,ARPC5L,MANEAL,HLA−DQB1,M6PR,FANCM,SH2D1A,ZNHIT3,REC8,MED17,MRPL28,RAB43,KCNJ15,ABI3,NUP50,CD86,PRDM1,CD247,RPSA,RAP2C,
12	UBE2N,RFC2,NLRC3,CHEK1,DNA2,PHACTR4,TBC1D12,AQP11,AK2,RNF19B,ITK,ART3,THYN1,HLA−DMA,CCR8,CHAF1B,AIF1,USP28,NLRC5,TRAT1,TALDO1,LILRB4,NFKB1,STRADB,SOCS1,MS4A7,HLA−DRB5,CMTM1,CLDN18,CCDC97,
13	ZNF8,PSMD9,TMPO,HABP2,GNLY,KIF2A,CLEC4A,CDC25A,HELLS,ARL6IP6,CD3E,ZAP70,SIGLEC10,CENPM,ARF3,TMEM194B,PASK,CD6,SPN,CCDC134,PBX4,ZNF227,CD300A,CMKLR1,CD33,C6orf130,EZH2,RFX7,HOMER1,CASP1,
14	DAZAP1,PCDP1,CHORDC1,BTF3,NPM1,C4orf32,MCM2,ICAM1,PAQR4,GPRC5B,MRPL11,ZNF689,ITGAL,CXCL17,MAP3K5,ALDH3A1,GDPD1,C12orf5,ACOT7,TNFAIP8L2,C6orf141,RANBP6,SSFA2,TFAM,CXorf65,GRWD1,RAPGEF2,TTC26,FOXP3,C10orf81,
15	CEACAM21,PPA1,NPL,IRF2,SLBP,SNX20,CLCN5,CDKN2AIPNL,SYPL1,CNPY2,EBI3,ZNF155,SYCP3,ZNF195,RHEBL1,PTGES3,TIGIT,TMED5,CCR2,PKP4,KIAA0753,ZW10,CCDC153,GPR65,CBFB,CCL17,PRKY,UTP18,FYB,TRAFD1,
16	HMGN2,PRMT1,TP53,NCK1,FUT8,C8orf80,IL12RB2,CASP2,SLC44A1,IFT57,RCC2,ZNF616,PTAFR,LAX1,ZNF655,NEK4,LRIG3,EOMES,LIPA,ALPK1,MTHFD1L,ZNF202,TBC1D10C,GSDMC,CCR6,SIGLEC7,FIGNL1,BST2,GYS1,SLC35B1,
17	POLE,TBK1,RNF166,IL6R,MPND,HEATR3,TPK1,BCL11B,NOL11,ST8SIA4,GTF2H3,SOX21,MSX2,CCDC109B,TBXAS1,NOB1,LMNB1,FAM133B,TMEM106A,LCP2,EPHA4,BCAT2,CALCOCO2,MS4A4A,MAP4K1,MDK,MTG1,RRM1,KHSRP,LGALS9,
18	ARHGAP9,IKZF3,OASL,RRM2,SLC10A5,TRIM21,RPA3,TUBA1B,TEX10,SHPK,RPL24,PSMB9,AKAP5,PSMB6,ZMYND8,RABGAP1L,ZBP1,PCGF6,GEMIN7,PATL2,RB1,RGS10,FAM78A,ANKMY2,IL10,BATF,CD209,CD84,GSTO1,LILRB1,
19	TNIP3,SNRPC,CYTIP,GAR1,PLAC1,PDCD1,WDR76,FAR1,BIN2,BATF2,APOBEC3D,SCO2,CCNG1,EPSTI1,MANEA,SIRPB2,RASAL3,PIK3R2,E2F2,LARP1,THG1L,CAPRIN1,RDM1,TOPBP1,CDK5,TLE6,MINPP1,PIK3C2G,BTN3A3,RGS1,
20	TMEM161B,WDR77,XKRX,ODF3B,STT3A,CYTH1,SLC6A14,ZNF618,TRIM5,CD4,DDX47,ZNF280C,GLS2,PSTPIP1,SLC25A17,RIPK2,KEL,RPS27L,FOXRED2,RPS13,PRIM1,SNX4,CDCA4,XRN1,ZBTB32,TSFM,PPP2R4,FAM135A,CLEC4E,NACAP1,
21	SIGLEC12,HIST1H2AL,SAMD10,DEF6,MATK,LYSMD2,DEAF1,43160,FRAT2,RPL23AP53,SIGLEC1,DOCK10,VPS13C,DMXL2,DDO,TMEM109,LSM6,FBXO5,DENND2D,PI4K2B,PRKD2,MR1,XRCC1,TNFRSF1B,ARNTL2,NR1H3,FBL,ATAD5,PCNXL3,ADAP2,
22	ZNF543,PATL1,HK3,RELL1,ENC1,CLEC2D,SAMSN1,C11orf35,FCER1G,C12orf45,KCNA3,BIN3,POLD1,FIGN,SLC47A1,LST1,IKBKE,NOC3L,NEU3,ZWINT,PHF5A,NEK6,LCK,KCNJ10,ALAS1,FRMD5,NAA25,PARP14,FANCL,ACTR3B,
23	UMPS,MYO1F,TSPO2,SLAMF6,TAF1D,CDV3,RAN,LY9,RPL6,TOP1,PHACTR2,TIFA,SGMS1,PLK4,ACP5,CA13,CDT1,FMNL2,PRODH,RAPH1,LAIR1,TRIM16L,SGK1,FCHSD2,TRIM25,RNF213,MSH6,ASB9,PTPRCAP,APOL2,
24	SPTY2D1,GINS3,LAMP3,C3orf37,SAMD3,GM2A,ZNF831,CACHD1,CCDC125,PSMB8,ZNF223,HAUS5,KCNH3,USP43,NT5DC1,PION,RFC4,TRAF3IP3,CRISP3,STAT2,SLC35F2,DMKN,NCAPD3,ZNF259,NUB1,DTX2,FAM173A,ADCK2,IL7,PHC1,
25	IL4I1,PRPF19,ZNF431,CCNE2,DTX4,NCKAP1L,ATP13A4,ARSG,ALDH3B2,SAMD9L,STMN1,SCLY,LILRB5,SMC4,SELPLG,EIF3F,ACAP1,MBOAT1,OVOL2,MICB,TYROBP,DBR1,MEX3D,PIK3R3,DUS2L,HNRNPH1,OSBPL7,FANCA,HCST,UBA2,
26	PDE6G,TRNP1,IFI30,COMMD8,HMGB2,APOE,FMO3,MAT1A,MYO7A,C7orf31,PSME1,BRI3BP,SLAMF1,MLKL,HIST1H3B,COTL1,ZBTB39,CCDC104,PTMA,XRCC2,CHAF1A,C3AR1,RWDD2B,TGM2,MTHFD1,EIF2S2,ZNF581,MNX1,RGS19,P2RY10,
27	PIK3R5,ZDHHC14,ZNF304,IL10RA,PARVG,FPR3,RFC5,RPS11,CYTH4,RFX3,LPXN,SEMA4D,ABCA12,MPZL2,RIF1,TFDP1,DHX58,CDC14B,TNFRSF17,AGPAT5,ZMYND15,GNPDA1,DIAPH1,RPS7,PDE12,MND1,TRAF3IP2,SIT1,LILRB2,SF3B5,
28	RAD54L,TNFRSF4,NAA16,UBXN11,ZNF92,TOR3A,PSMA5,DDX6,PRKAR1B,CCR1,NAA50,DHX33,TMEM62,IFFO2,PML,FGD2,BTN3A1,SAT1,MPHOSPH9,PRKX,GAL3ST4,PILRA,TRAK1,FAM81A,FBXL19,XCL1,AKR1B1,APOL1,JAK3,DTNB,
29	PAQR6,DKK1,GPC4,CCDC34,CEP78,NCAPH2,RPL35,HLA−E,HEXIM2,SIGLEC9,CTSS,RAVER2,CDC7,GSTK1,MCM4,TNFSF8,DAG1,DDX26B,U2AF1,RBX1,43344,PHF15,CHIT1,RPL28,TAPBP,WDR90,PTK2B,BAAT,GMFG,LETMD1,
30	ARHGAP30,ESCO2,CCNO,IL27RA,PTPN7,MORC4,RALGPS1,ZNF485,MPDU1,LIMK2,C1QBP,GIMAP4,IRF4,CCL20,CISH,ZNF860,RAB11FIP3,CENPV,AVIL,SPI1,ZNF232,CASP7,PGAM5,HLA−G,MYO1G,ZNF239,SENP2,LRRC20,TNFRSF9,PRMT3,
31	NACA,FERMT1,DOCK2,SEC14L1,FAM40B,ZBTB46,KLHL6,RPL41,NCOA3,PCDH7,TOR2A,FAM160A1,NAT10,TYMP,RASGRP3,TPRG1,AHCY,CSTF2,CD52,MAST4,SNRPD2,OSBPL10,ECT2,HCK,PIGL,OAS2,GBP2,DRAM1,C9orf64,TNS4,
32	C5orf58,FXYD5,SLC25A22,C20orf72,TMEM41A,FZD5,NMUR1,FAM57A,MTHFD2,ARHGAP25,PPIF,RPL27A,LMBR1,IL15,MAP3K1,CD300C,ELL2,TMEM30A,GZMK,TMEM33,CYLD,MIR155HG,CLEC2B,TTC19,PVT1,CDK12,ATP11B,TRAP1,RELT,LMNB2,
33	SCGB1A1,ZNF90,HTT,CD53,PLEKHA7,CHD3,PGD,STON2,MYO19,DNMT1,SS18L2,MAZ,TMPRSS4,KIAA0020,ITGA4,MCCC2,LYN,RAB11FIP4,CCNC,ARHGAP15,RPS3A,ABCC4,POU2F3,ACSM3,TRAPPC4,NXT2,PAK1,DNASE1L3,FCRL5,PKMYT1,
34	FAM113B,LYPD6,ANKRD22,CCT2,MEIS2,PIK3CG,ALKBH2,LYZ,SHMT2,MYBBP1A,TSC22D4,ADA,PIM2,RFX5,SH2D4A,TRAM1,APOBEC3C,ASF1B,IGFL2,ECHDC1,NCAPG2,HS3ST1,SDCCAG3,TNFAIP8,HMGN1,CCT8,TM7SF3,HIPK2,STEAP1,NEB,
35	RAB8B,HNRNPF,GINS2,IKZF1,CD72,UCP2,DDAH1,NDOR1,LRRC61,C20orf118,GPR39,LTA,SSX2IP,CALM3,TNFSF10,P2RX3,C17orf58,FAM46C,BZW2,ERAP1,FAM49B,A4GNT,HIP1R,RNASEH2A,GALM,ATP2A2,MRPL23,HAUS6,CD48,PELI2,
36	MCM6,UHRF1,PPP3CC,SAMD1,RAD51,ZNF69,LAPTM5,GLCCI1,COASY,DTL,OSBPL3,CD40LG,HDAC9,ARAP2,HSPH1,FMO2,CERKL,HEXDC,NEURL3,WDR45L,MLF1IP,RARG,CCDC28B,GTF3C6,MPEG1,IGSF9B,CD7,TMEM48,TREM2,SASH3,
37	NPC2,AP1S1,MGST2,ZMAT3,SMAGP,SPINK5,KRT13,B9D2,POLB,HCLS1,TEAD4,HIST1H1E,MARS2,IRF8,PA2G4,NUP205,NDUFB6,GSDMD,BCYRN1,SERPINB1,PSMC4,MSR1,STEAP2,C14orf80,ZNF518A,RBL1,CXorf21,FGFR1OP,RPL26L1,DLGAP5,
38	OTX1,BRCA2,WAS,POLA1,GRHL1,NSA2,PTPRC,PPP1R16B,C17orf53,NUDT12,ITGB2,HLA−F,PLLP,SNORA76,SAAL1,KALRN,HLA−C,CST7,PLD3,PRRG4,MSH2,IGF2BP2,LILRB3,KITLG,UBASH3B,FBXL14,KIF18B,HCP5,TIMELESS,UGCG,
39	FLVCR1,KIF11,UGT8,RTP4,TIPIN,CCDC138,RPS15,GNA15,IQGAP3,SPATA20,GIMAP7,FRMD4B,

Supporting Material 7A:methylation correlated genes (meSCGs)

signature.id	Genes
positive cis−meSCGs	[illegible]
negative cis−meSCGs	[illegible]
positive trans−meSCGs	[illegible]
negative trans−meSCGs	[illegible]

Supporting Material 7B: MSigDB signatures enriched in meSCGs

	set1_Name	set2_Name	Background	set1_size	set2_size	expected.overlap	actual.overlap	enrichment.foldchange	odds.ratio	FET_pvalue	corrected.FET.pvalue
1	cis_NEG	SIGNAL_TRANSDUCTION	26538	4004	1612	243.21531	452	1.858436	2.3445925355341699	2.092710e−43	6.905943e−40
2	cis_NEG	MEMBRANE	26538	4004	1947	293.75944	512	1.742923	2.1557128575142999	9.264462e−41	8.634478e−38
3	cis_NEG	PLASMA_MEMBRANE	26538	4004	1389	209.56952	396	1.889588	2.3808186883784801	1.033535e−39	9.632547e−37
4	trans_NEG	HALLMARK_E2F_TARGETS	26538	8266	198	61.67262	147	2.383554	6.4679994705948802	8.533025e−36	1.706605e−33
5	trans_POS	HALLMARK_E2F_TARGETS	26538	7352	198	54.85327	139	2.534033	6.2480032285349001	1.440063e−35	2.880126e−33
6	cis_NEG	HALLMARK_ALLOGRAFT_REJECTION	26538	4004	196	29.57209	103	3.483014	6.37049006676334	1.475272e−34	2.950544e−32
7	cis_NEG	PLASMA_MEMBRANE_PART	26538	4004	1127	170.03949	325	1.911321	2.3936416370505098	1.394435e−33	1.299613e−30
8	cis_NEG	MEMBRANE_PART	26538	4004	1630	245.93112	426	1.732192	2.1092139904171399	4.496690e−33	4.190915e−30
9	trans_NEG	PLASMA_MEMBRANE	26538	8266	1389	432.64278	631	1.458478	1.9096001439513199	1.497114e−30	1.395310e−27
10	trans_NEG	MEMBRANE	26538	8266	1947	606.44743	837	1.380169	1.7418487092003201	2.005608e−30	1.869227e−27
11	trans_NEG	SIGNAL_TRANSDUCTION	26538	8266	1612	502.10234	706	1.406088	1.7898671449213901	2.214669e−28	7.308408e−25
12	trans_NEG	RB_P107_DN.V1_UP	26538	8266	138	42.98395	106	2.466037	7.4036982171184897	2.447140e−28	1.850038e−25
13	cis_NEG	HALLMARK_INTERFERON_GAMMA_RESPONSE	26538	4004	197	29.72296	95	3.196182	5.3442926977970302	3.510299e−28	7.020599e−26
14	trans_POS	HALLMARK_ALLOGRAFT_REJECTION	26538	7352	196	54.29919	128	2.357309	4.9812340428831101	4.621172e−28	9.242344e−26
15	trans_NEG	HALLMARK_EPITHELIAL_MESENCHYMAL_TRANSITION	26538	8266	199	61.98410	137	2.210244	4.9496478448888599	6.775337e−28	1.355067e−25
16	cis_POS	MEMBRANE	26538	1529	1947	112.17737	231	2.059239	2.4156028668402301	6.036373e−27	5.625900e−24
17	cis_NEG	P53_DN.V1_UP	26538	4004	191	28.81770	91	3.157782	5.21681252950534	1.456378e−26	1.101022e−23
18	cis_POS	PLASMA_MEMBRANE	26538	1529	1389	80.02792	183	2.286702	2.6831504643794801	1.512530e−26	1.409678e−23
19	cis_NEG	INTEGRAL_TO_MEMBRANE	26538	4004	1294	195.23611	338	1.731237	2.0809729484170498	3.475253e−26	3.238936e−23
20	trans_NEG	HALLMARK_ALLOGRAFT_REJECTION	26538	8266	196	61.04966	133	2.178554	4.72628681869607	3.596731e−26	7.193461e−24
21	cis_NEG	IMMUNE_SYSTEM_PROCESS	26538	4004	321	48.43183	125	2.580947	3.67242015602445	1.044284e−25	3.446138e−22
22	trans_NEG	IMMUNE_SYSTEM_PROCESS	26538	8266	321	99.98440	190	1.900296	3.25784944539781	1.923727e−25	6.348301e−22
23	trans_POS	REACTOME_CELL_CYCLE	26538	7352	393	108.87542	206	1.892071	2.92868571730925	2.087282e−25	5.627313e−22
24	cis_NEG	INTRINSIC_TO_MEMBRANE	26538	4004	1312	197.95192	339	1.712537	2.04961730583979	2.266534e−25	2.112409e−22
25	trans_POS	RB_P107_DN.V1_UP	26538	7352	138	38.23106	97	2.537204	6.2415694464597804	2.611354e−25	1.974184e−22
26	trans_POS	IMMUNE_SYSTEM_PROCESS	26538	7352	321	88.92878	176	1.979112	3.2205343707150198	8.660981e−25	2.858124e−21
27	trans_NEG	PLASMA_MEMBRANE_PART	26538	8266	1127	351.03557	510	1.452844	1.88158216696407	2.512184e−24	2.341355e−21
28	cis_POS	ANATOMICAL_STRUCTURE_DEVELOPMENT	26538	1529	993	57.21219	142	2.481989	2.9061336466287702	4.496449e−24	1.483828e−20
29	cis_POS	MULTICELLULAR_ORGANISMAL_DEVELOPMENT	26538	1529	1029	59.28634	145	2.445757	2.8590396022510101	6.218746e−24	2.052186e−20
30	cis_NEG	LEF1_UP.V1_DN	26538	4004	184	27.76155	85	3.061789	4.9148313862871502	9.308673e−24	7.037357e−21
31	cis_NEG	ANATOMICAL_STRUCTURE_DEVELOPMENT	26538	4004	993	149.82184	270	1.802140	2.18128065866965	9.648281e−24	3.183933e−20
32	cis_NEG	INTEGRAL_TO_PLASMA_MEMBRANE	26538	4004	946	142.73058	260	1.821614	2.21161291785464	1.300565e−23	1.212127e−20
33	cis_NEG	HALLMARK_ESTROGEN_RESPONSE_LATE	26538	4004	199	30.02472	89	2.964224	4.6340523863645204	1.395829e−23	2.791658e−21
34	cis_NEG	REACTOME_IMMUNE_SYSTEM	26538	4004	876	132.16912	245	1.853686	2.2623045454572401	2.021012e−23	5.448648e−20
35	trans_POS	HALLMARK_G2M_CHECKPOINT	26538	7352	199	55.13030	122	2.212939	4.1873283213445296	3.280139e−23	6.560278e−21
36	trans_NEG	MEMBRANE_PART	26538	8266	1630	507.70895	691	1.361016	1.6838260398122	3.551244e−23	3.309760e−20
37	cis_POS	MEMBRANE_PART	26538	1529	1630	93.91326	195	2.076384	2.40126621142698	4.110168e−23	3.830676e−20
38	cis_NEG	INTRINSIC_TO_PLASMA_MEMBRANE	26538	4004	960	144.84287	261	1.801953	2.1781191183092199	5.703422e−23	5.315589e−20
39	cis_POS	SYSTEM_DEVELOPMENT	26538	1529	843	48.56986	125	2.573612	3.01185692758306	1.196136e−22	3.947248e−19
40	cis_NEG	HALLMARK_ESTROGEN_RESPONSE_EARLY	26538	4004	198	29.87384	87	2.912247	4.4866656290402602	1.922472e−22	3.844943e−20
41	trans_NEG	INTEGRAL_TO_MEMBRANE	26538	8266	1294	403.05238	565	1.401803	1.76541585047961	2.004582e−22	1.868271e−19
42	cis_NEG	IMMUNE_RESPONSE	26538	4004	227	34.24930	94	2.744581	4.0490045845801301	6.916444e−22	2.282427e−18
43	cis_NEG	MULTICELLULAR_ORGANISMAL_DEVELOPMENT	26538	4004	1029	155.25345	271	1.745533	2.08549552508088	1.163371e−21	3.839126e−18
44	cis_NEG	BIOPOLYMER_METABOLIC_PROCESS	26538	4004	1660	250.45746	393	1.569129	1.8267030628711101	1.245150e−21	4.108996e−18
45	trans_NEG	INTRINSIC_TO_MEMBRANE	26538	8266	1312	408.65898	568	1.389912	1.7382088031310099	1.557127e−21	1.451243e−18
46	cis_NEG	P53_DN.V1_DN	26538	4004	189	28.51594	83	2.910653	4.4787160217004702	1.855134e−21	1.402482e−18
47	trans_NEG	IMMUNE_RESPONSE	26538	8266	227	70.70548	140	1.980045	3.6009870891399398	1.962308e−21	6.475618e−18
48	cis_POS	PLASMA_MEMBRANE_PART	26538	1529	1127	64.93266	147	2.263884	2.6078738907482202	5.756293e−21	5.364865e−18
49	trans_NEG	HALLMARK_G2M_CHECKPOINT	26538	8266	199	61.98410	126	2.032779	3.8585859325326002	7.638373e−21	1.527675e−18
50	trans_POS	REACTOME_DNA_REPLICATION	26538	7352	185	51.25179	112	2.185290	4.0500045489680199	8.326941e−21	2.244943e−17
51	trans_NEG	HALLMARK_INTERFERON_GAMMA_RESPONSE	26538	8266	197	61.36114	125	2.037120	3.88084013170677	8.333130e−21	1.666626e−18
52	trans_POS	REACTOME_CELL_CYCLE_MITOTIC	26538	7352	304	84.21916	161	1.911679	2.9813637808380502	9.528495e−21	2.568882e−17
53	cis_NEG	REACTOME_HEMOSTASIS	26538	4004	446	67.29158	145	2.154801	2.77533447777834	1.219997e−20	3.289111e−17
54	cis_NEG	MEK_UP.V1_UP	26538	4004	194	29.27033	83	2.835636	4.2760454175732399	1.424844e−20	1.077182e−17
55	cis_NEG	SYSTEM_DEVELOPMENT	26538	4004	843	127.19014	230	1.808316	2.1792686600536202	1.466609e−20	4.839810e−17
56	trans_POS	PLASMA_MEMBRANE	26538	7352	1389	384.80398	539	1.400713	1.70655008252525	2.307452e−20	2.150545e−17
57	trans_NEG	INTEGRAL_TO_PLASMA_MEMBRANE	26538	8266	946	294.65808	427	1.449137	1.8632940633220001	2.900490e−20	2.703257e−17
58	trans_NEG	KEGG_CELL_ADHESION_MOLECULES_CAMS	26538	8266	128	39.86917	90	2.257383	5.2813178527909503	8.700721e−20	6.473337e−17
59	cis_NEG	KEGG_CELL_ADHESION_MOLECULES_CAMS	26538	4004	128	19.31238	63	3.262156	5.5254306675491804	9.415016e−20	7.004772e−17
60	trans_NEG	INTRINSIC_TO_PLASMA_MEMBRANE	26538	8266	960	299.01877	430	1.438037	1.83701339444533	1.181203e−19	1.100881e−16
61	trans_NEG	REACTOME_CELL_CYCLE_MITOTIC	26538	8266	304	94.68928	170	1.795346	2.8421314106753801	2.408017e−19	6.492013e−16
62	trans_POS	IMMUNE_RESPONSE	26538	7352	227	62.88733	127	2.019485	3.3545827400929902	2.880662e−19	9.506185e−16
63	trans_NEG	REACTOME_CELL_CYCLE	26538	8266	393	122.41081	207	1.691027	2.49749223374618	4.017154e−19	1.083025e−15
64	trans_POS	SIGNAL_TRANSDUCTION	26538	7352	1612	446.58316	605	1.354731	1.61873776318075	5.893130e−19	1.944733e−15
65	trans_NEG	REACTOME_DNA_REPLICATION	26538	8266	185	57.62341	116	2.013071	3.75459583919845	8.096624e−19	2.182850e−15
66	cis_NEG	POSITIVE_REGULATION_OF_BIOLOGICAL_PROCESS	26538	4004	693	104.55844	194	1.855422	2.24839471760454	8.198120e−19	2.705379e−15
67	cis_POS	INTRINSIC_TO_MEMBRANE	26538	1529	1312	75.59153	157	2.076952	2.3632252608558302	1.115826e−18	1.039950e−15
68	cis_POS	NERVOUS_SYSTEM_DEVELOPMENT	26538	1529	372	21.43296	70	3.265997	3.9251038301655701	1.177733e−18	3.886518e−15
69	cis_POS	LEF1_UP.V1_UP	26538	1529	189	10.88933	48	4.407985	5.7154195496863496	1.213478e−18	9.173896e−16
70	cis_NEG	INTRACELLULAR_SIGNALING_CASCADE	26538	4004	657	99.12684	186	1.876384	2.2819357324991199	1.214953e−18	4.009344e−15
71	cis_POS	INTEGRAL_TO_MEMBRANE	26538	1529	1294	74.55445	155	2.079017	2.3640354691724101	1.728313e−18	1.610788e−15
72	cis_NEG	ERB2_UP.V1_UP	26538	4004	188	28.36506	78	2.749862	4.0499118767744999	1.821540e−18	1.377085e−15
73	trans_NEG	CELL_PROLIFERATION_GO_0008283	26538	8266	504	156.98485	250	1.592510	2.2123186927633398	2.379812e−18	7.853378e−15
74	cis_NEG	REGULATION_OF_DEVELOPMENTAL_PROCESS	26538	4004	431	65.02841	136	2.091394	2.6503740749553399	3.440860e−18	1.135484e−14
75	trans_NEG	REACTOME_IMMUNOREGULATORY_INTERACTIONS_BETWEEN_A_LYMPHOID_AND_A_NON_LYMPHOID_CELL	26538	8266	58	18.06572	50	2.767673	13.894532498007999	4.473293e−18	1.206000e−14
76	cis_NEG	NEGATIVE_REGULATION_OF_BIOLOGICAL_PROCESS	26538	4004	665	100.33386	186	1.853811	2.2430138991184401	4.816713e−18	1.589515e−14
77	cis_NEG	RAF_UP.V1_DN	26538	4004	191	28.81770	78	2.706670	3.94183679475969	5.529071e−18	4.179978e−15
78	cis_NEG	ORGAN_DEVELOPMENT	26538	4004	567	85.54782	165	1.928746	2.3659852088603799	6.493073e−18	2.142714e−14
79	cis_NEG	POSITIVE_REGULATION_OF_CELLULAR_PROCESS	26538	4004	654	98.67420	183	1.854588	2.24337098777705	8.586986e−18	2.833705e−14
80	trans_POS	REACTOME_MITOTIC_M_M_G1_PHASES	26538	7352	165	45.71106	98	2.143901	3.8548835302790598	1.395761e−17	3.762970e−14
81	trans_POS	HALLMARK_EPITHELIAL_MESENCHYMAL_TRANSITION	26538	7352	199	55.13030	112	2.031551	3.3956258441544298	2.002771e−17	4.005541e−15
82	trans_NEG	REACTOME_IMMUNE_SYSTEM	26538	8266	876	272.85462	390	1.429333	1.8120341715586401	2.095790e−17	5.650250e−14
83	cis_NEG	STK33_NOMO_DN	26538	4004	275	41.49145	98	2.361933	3.1688946757259302	2.386886e−17	1.804486e−14
84	cis_NEG	E2F1_UP.V1_DN	26538	4004	184	27.76155	75	2.701578	3.9270149198147402	2.709008e−17	2.048010e−14
85	cis_NEG	HALLMARK_INTERFERON_ALPHA_RESPONSE	26538	4004	97	14.63516	50	3.416429	6.0495381153576799	4.769176e−17	9.538352e−15
86	cis_POS	RAF_UP.V1_DN	26538	1529	191	11.00456	46	4.180086	5.3177475010383404	5.961129e−17	4.506614e−14
87	trans_POS	MEMBRANE	26538	7352	1947	539.39046	701	1.299615	1.5174976772953901	6.440440e−17	6.002490e−14
88	cis_NEG	RAF_UP.V1_UP	26538	4004	191	28.81770	76	2.637268	3.77168132125467	8.377302e−17	6.333240e−14
89	cis_NEG	CELL_DEVELOPMENT	26538	4004	567	85.54782	162	1.893678	2.3038108686281098	8.469337e−17	2.794881e−13
90	trans_NEG	HALLMARK_INFLAMMATORY_RESPONSE	26538	8266	200	62.29558	119	1.910248	3.2802212278085299	1.023402e−16	2.046804e−14
91	cis_NEG	RPS14_DN.V1_UP	26538	4004	188	28.36506	75	2.644098	3.7873065735168399	1.125453e−16	8.508427e−14
92	cis_NEG	HALLMARK_COMPLEMENT	26538	4004	196	29.57209	77	2.603807	3.6931348239736002	1.235352e−16	2.470703e−14
93	cis_NEG	LEF1_UP.V1_UP	26538	4004	189	28.51594	75	2.630108	3.7539131196470299	1.592570e−16	1.203983e−13
94	trans_POS	LEF1_UP.V1_UP	26538	7352	189	52.35994	106	2.024449	3.3665731550546298	2.000255e−16	1.512193e−13
95	cis_POS	P53_DN.V1_DN	26538	1529	189	10.88933	45	4.132486	5.2351627004715899	2.068368e−16	1.563686e−13
96	cis_NEG	NEGATIVE_REGULATION_OF_CELLULAR_PROCESS	26538	4004	636	95.95840	175	1.823707	2.18825478046036	2.650125e−16	8.745414e−13
97	cis_NEG	REACTOME_ADAPTIVE_IMMUNE_SYSTEM	26538	4004	503	75.89163	147	1.936973	2.3740998137750702	2.818465e−16	7.598581e−13
98	cis_NEG	CSR_LATE_UP.V1_DN	26538	4004	164	24.74399	68	2.748142	4.0378192926526602	2.862835e−16	2.164304e−13
99	cis_NEG	HALLMARK_APICAL_JUNCTION	26538	4004	199	30.02472	77	2.564554	3.6018321198560299	3.365681e−16	6.731362e−14
100	cis_NEG	HALLMARK_KRAS_SIGNALING_UP	26538	4004	199	30.02472	77	2.564554	3.6018321198560299	3.365681e−16	6.731362e−14

	set1_Name	set2_Name	Background	set1_size	set2_size	expected.overlap	actual.overlap	enrichment.foldchange	odds.ratio	FET_pvalue	corrected.FET.pvalue
1	trans_NEG	REACTOME_ADAPTIVE_IMMUNE_SYSTEM	26538	8266	503	156.67337	243	1.550997	2.0982400351426902	3.960473e−16	1.067744e−12
2	trans_NEG	REACTOME_MITOTIC_M_M_G1_PHASES	26538	8266	165	51.39385	102	1.984673	3.6107769856860101	4.110326e−16	1.108144e−12
3	cis_NEG	TRANSCRIPTION	26538	4004	743	112.10234	196	1.748402	2.0688480405277301	4.883948e−16	1.611703e−12
4	trans_POS	PLASMA_MEMBRANE_PART	26538	7352	1127	312.22036	434	1.390044	1.6740712803968201	6.349125e−16	5.917384e−13
5	trans_POS	KEGG_CELL_ADHESION_MOLECULES_CAMS	26538	7352	128	35.46070	79	2.227819	4.2418889256296701	8.816687e−16	6.559615e−13
6	trans_NEG	CELL_CYCLE_PROCESS	26538	8266	193	60.11523	114	1.896358	3.22030053614175	9.409322e−16	3.105076e−12
7	cis_NEG	HALLMARK_EPITHELIAL_MESENCHYMAL_TRANSITION	26538	4004	199	30.02472	76	2.531248	3.52509474569385	1.211830e−15	2.423660e−13
8	cis_NEG	REACTOME_GENERIC_TRANSCRIPTION_PATHWAY	26538	4004	338	50.99676	109	2.137391	2.7258327378095699	1.393169e−15	3.755984e−12
9	trans_POS	CELL_CYCLE_PROCESS	26538	7352	193	53.46808	106	1.982491	3.2112668564770801	1.419074e−15	4.682943e−12
10	cis_NEG	LTE2_UP.V1_UP	26538	4004	184	27.76155	72	2.593515	3.6656294715276698	1.525199e−15	1.153050e−12
11	cis_POS	INTRINSIC_TO_PLASMA_MEMBRANE	26538	1529	960	55.31087	119	2.151476	2.4251446123306901	1.814467e−15	1.691083e−12
12	cis_NEG	STK33_DN	26538	4004	271	40.88793	93	2.274510	2.9863754556869	2.339654e−15	1.768778e−12
13	cis_NEG	APOPTOSIS_GO	26538	4004	422	63.67051	127	1.994644	2.4694883663282998	2.662489e−15	8.786213e−12
14	trans_POS	AKT_UP.V1_DN	26538	7352	187	51.80586	103	1.988192	3.2309893973385901	2.748921e−15	2.078184e−12
15	cis_NEG	EGFR_UP.V1_UP	26538	4004	190	28.66682	73	2.546498	3.55782085750567	3.036436e−15	2.295546e−12
16	cis_NEG	PROGRAMMED_CELL_DEATH	26538	4004	423	63.82139	127	1.989929	2.4607273052180898	3.247326e−15	1.071618e−11
17	trans_NEG	CELL_CYCLE_GO_0007049	26538	8266	313	97.49258	164	1.682179	2.4619630199870701	3.523365e−15	1.162710e−11
18	cis_POS	INTEGRAL_TO_PLASMA_MEMBRANE	26538	1529	946	54.50426	117	2.146621	2.4166445412262698	3.734880e−15	3.480908e−12
19	cis_NEG	KEGG_PATHWAYS_IN_CANCER	26538	4004	325	49.03535	105	2.141313	2.7315086996120699	4.014414e−15	2.986724e−12
20	trans_NEG	DEFENSE_RESPONSE	26538	8266	256	79.73834	140	1.755743	2.6964726338817702	4.092977e−15	1.350682e−11
21	cis_NEG	HALLMARK_MYOGENESIS	26538	4004	199	30.02472	75	2.497942	3.4496250385449501	4.271743e−15	8.543486e−13
22	trans_NEG	RECEPTOR_ACTIVITY	26538	8266	569	177.23091	265	1.495224	1.95763681911309	5.324251e−15	8.433614e−12
23	cis_NEG	HALLMARK_INFLAMMATORY_RESPONSE	26538	4004	200	30.17560	75	2.485452	3.4218755357289798	5.833822e−15	1.166764e−12
24	trans_NEG	HALLMARK_KRAS_SIGNALING_UP	26538	8266	199	61.98410	115	1.855315	3.0547251813377101	6.326978e−15	1.265396e−12
25	cis_NEG	PROTEIN_KINASE_CASCADE	26538	4004	289	43.60374	96	2.201646	2.84340360565148	8.628660e−15	2.847458e−11
26	cis_NEG	RNA_BIOSYNTHETIC_PROCESS	26538	4004	629	94.90225	169	1.780780	2.11462077520261	9.101695e−15	3.003560e−11
27	trans_POS	HALLMARK_INTERFERON_GAMMA_RESPONSE	26538	7352	197	54.57623	106	1.942237	3.0692393437330998	9.108956e−15	1.821791e−12
28	trans_POS	LYMPHOCYTE_ACTIVATION	26538	7352	59	16.34517	45	2.753108	8.4324654612013603	1.218575e−14	4.021297e−11
29	cis_NEG	TRANSCRIPTION_DNA_DEPENDENT	26538	4004	627	94.60050	168	1.775889	2.1062366312715999	1.424950e−14	4.702335e−11
30	trans_NEG	INTRACELLULAR_SIGNALING_CASCADE	26538	8266	657	204.64097	297	1.451322	1.8544071150016199	1.445045e−14	4.768648e−11
31	trans_NEG	RPS14_DN.V1_UP	26538	8266	188	58.55784	109	1.861407	3.0771782546463999	2.342630e−14	1.771028e−11
32	cis_NEG	PROTEIN_METABOLIC_PROCESS	26538	4004	1206	181.95885	279	1.533314	1.7457468576609501	2.945641e−14	9.720617e−11
33	trans_POS	REACTOME_IMMUNOREGULATORY_INTERACTIONS_BETWEEN_A_LYMPHOID_AND_A_NON_LYMPHOID_CELL	26538	7352	58	16.06813	44	2.738340	8.2439152335050103	3.377316e−14	9.105245e−11
34	cis_NEG	KEGG_CHEMOKINE_SIGNALING_PATHWAY	26538	4004	186	28.06331	70	2.494360	3.4385475057298698	3.759938e−14	2.797394e−11
35	cis_NEG	NUCLEOBASENUCLEOSIDENUCLEOTIDE_AND_NUCLEIC_ACID_METABOLIC_PROCESS	26538	4004	1226	184.97641	282	1.524519	1.7327747327145	4.427661e−14	1.461128e−10
36	cis_NEG	CYTOPLASM	26538	4004	2087	314.88236	437	1.387820	1.5505980758715101	5.250732e−14	4.893682e−11
37	trans_NEG	SNF5_DN.V1_UP	26538	8266	174	54.19715	102	1.882018	3.15802049817913	5.932733e−14	4.485146e−11
38	cis_NEG	ESC_V6.5_UP_EARLY.V1_DN	26538	4004	169	25.49838	65	2.549182	3.5587369322920002	9.258786e−14	6.999642e−11
39	cis_POS	SIGNAL_TRANSDUCTION	26538	1529	1612	92.87618	166	1.787326	1.9845465866059699	1.201603e−13	3.965289e−10
40	trans_NEG	POSITIVE_REGULATION_OF_BIOLOGICAL_PROCESS	26538	8266	693	215.85417	307	1.422257	1.78720364398675	1.224335e−13	4.040307e−10
41	cis_NEG	BMI1_DN.V1_UP	26538	4004	143	21.57555	58	2.688228	3.8817270056501001	1.350767e−13	1.021180e−10
42	trans_POS	E2F3_UP.V1_UP	26538	7352	183	50.69772	98	1.933026	3.03591794788397	1.355150e−13	1.024493e−10
43	cis_NEG	REGULATION_OF_APOPTOSIS	26538	4004	332	50.09149	103	2.056237	2.57159593406094	1.358118e−13	4.481790e−10
44	cis_NEG	REGULATION_OF_CELLULAR_METABOLIC_PROCESS	26538	4004	779	117.53395	195	1.659095	1.92421721363075	1.446750e−13	4.774275e−10
45	cis_NEG	REGULATION_OF_PROGRAMMED_CELL_DEATH	26538	4004	333	50.24237	103	2.050063	2.5603065238843099	1.678138e−13	5.537854e−10
46	cis_NEG	ESC_J1_UP_LATE.V1_UP	26538	4004	187	28.21418	69	2.445578	3.3308356020930399	1.749725e−13	1.322792e−10
47	cis_NEG	REACTOME_AXON_GUIDANCE	26538	4004	242	36.51247	82	2.245808	2.9235172092222599	2.262543e−13	6.099816e−10
48	trans_POS	CELL_CYCLE_GO_0007049	26538	7352	313	86.71249	147	1.695258	2.3376181270088301	2.416431e−13	7.974223e−10
49	trans_POS	CELL_PROLIFERATION_GO_0008283	26538	7352	504	139.62650	215	1.539822	1.9698081192299699	2.553611e−13	8.426917e−10
50	cis_NEG	CELL_PROLIFERATION_GO_0008283	26538	4004	504	76.04251	139	1.827925	2.1842558548645501	2.626997e−13	8.669089e−10
51	trans_NEG	LEUKOCYTE_ACTIVATION	26538	8266	67	20.86902	50	2.395896	6.5342898699676599	3.032804e−13	1.000825e−09
52	cis_NEG	REGULATION_OF_METABOLIC_PROCESS	26538	4004	790	119.19361	196	1.644383	1.90095568385769	3.071836e−13	1.013706e−09
53	cis_NEG	PROTEIN_MODIFICATION_PROCESS	26538	4004	623	93.99699	163	1.734098	2.03638798206712	3.137103e−13	1.035244e−09
54	trans_NEG	AKT_UP.V1_DN	26538	8266	187	58.24636	106	1.819856	2.9172107579415001	3.989728e−13	3.016235e−10
55	cis_NEG	BIOPOLYMER_MODIFICATION	26538	4004	641	96.71279	166	1.716422	2.0085947251683098	4.819522e−13	1.590442e−09
56	cis_POS	REACTOME_DEVELOPMENTAL_BIOLOGY	26538	1529	384	22.12435	61	2.757143	3.1755510058548002	5.679527e−13	1.531200e−09
57	trans_POS	LEUKOCYTE_ACTIVATION	26538	7352	67	18.56146	47	2.532129	6.1641144756373896	6.119552e−13	2.019452e−09
58	cis_NEG	TRANSCRIPTION_FROM_RNA_POLYMERASE_II_PROMOTER	26538	4004	452	68.19685	127	1.862256	2.2383967676260901	6.771950e−13	2.234744e−09
59	cis_POS	REACTOME_AXON_GUIDANCE	26538	1529	242	13.94295	46	3.299158	3.9267914050264099	6.832196e−13	1.841960e−09
60	cis_NEG	BMI1_DN_MEL18_DN.V1_DN	26538	4004	140	21.12292	56	2.651149	3.7907258885637698	7.024223e−13	5.310313e−10
61	trans_NEG	CELL_CYCLE_PHASE	26538	8266	170	52.95124	98	1.850759	3.0326963899097699	7.562511e−13	2.495629e−09
62	cis_NEG	DEFENSE_RESPONSE	26538	4004	256	38.62476	84	2.174771	2.7859104433281598	8.513678e−13	2.809514e−09
63	cis_NEG	STK33_SKM_DN	26538	4004	265	39.98267	86	2.150932	2.74135559527878	9.069867e−13	6.856819e−10
64	trans_NEG	LEF1_UP.V1_UP	26538	8266	189	58.86932	106	1.800598	2.8466073840375299	1.005104e−12	7.598584e−10
65	trans_POS	CELL_CYCLE_PHASE	26538	7352	170	47.09624	91	1.932214	3.03116771015255	1.026468e−12	3.387345e−09
66	cis_POS	TRANSMEMBRANE_TRANSPORTER_ACTIVITY	26538	1529	370	21.31773	59	2.767649	3.1871535485654001	1.156725e−12	1.832252e−09
67	cis_NEG	RNA_METABOLIC_PROCESS	26538	4004	827	124.77609	201	1.610885	1.8495805322613299	1.214983e−12	4.009443e−09
68	trans_NEG	LYMPHOCYTE_ACTIVATION	26538	8266	59	18.37719	45	2.448687	7.1379155232224099	1.246438e−12	4.113245e−09
69	trans_POS	REACTOME_MITOTIC_PROMETAPHASE	26538	7352	83	22.99405	54	2.348434	4.88754912988475	1.430403e−12	3.856365e−09
70	trans_POS	MEMBRANE_PART	26538	7352	1630	451.56982	577	1.277765	1.46656396021918	1.506620e−12	1.404170e−09
71	cis_NEG	TBK1.DF_UP	26538	4004	285	43.00023	90	2.093012	2.6340083921961099	1.537517e−12	1.162363e−09
72	cis_NEG	KEGG_LEUKOCYTE_TRANSENDOTHELIAL_MIGRATION	26538	4004	116	17.50185	49	2.799705	4.15435107823573	1.707094e−12	1.270078e−09
73	cis_NEG	REACTOME_CELL_SURFACE_INTERACTIONS_AT_THE_VASCULAR_WALL	26538	4004	84	12.67375	40	3.156130	5.1574106995434299	1.769471e−12	4.770493e−09
74	cis_NEG	REGULATION_OF_GENE_EXPRESSION	26538	4004	666	100.48474	169	1.681847	1.9539877953745199	1.874096e−12	6.184517e−09
75	trans_NEG	ORGANELLE_ORGANIZATION_AND_BIOGENESIS	26538	8266	467	145.46017	217	1.491817	1.9435071866557001	2.037476e−12	6.723669e−09
76	trans_NEG	P53_DN.V1_UP	26538	8266	191	59.49228	106	1.781744	2.7793267593176201	2.465334e−12	1.863793e−09
77	trans_NEG	CSR_LATE_UP.V1_UP	26538	8266	168	52.32828	96	1.834572	2.97008044914352	2.632635e−12	1.990272e−09
78	cis_NEG	REACTOME_DEVELOPMENTAL_BIOLOGY	26538	4004	384	57.93715	111	1.915869	2.3248914521972699	2.685372e−12	7.239764e−09
79	cis_NEG	POST_TRANSLATIONAL_PROTEIN_MODIFICATION	26538	4004	470	70.91265	129	1.819139	2.16653285375937	2.730451e−12	9.010487e−09
80	cis_NEG	REACTOME_CYTOKINE_SIGNALING_IN_IMMUNE_SYSTEM	26538	4004	261	39.37915	84	2.133108	2.7063414118995901	2.738834e−12	7.383897e−09
81	trans_NEG	HALLMARK_COMPLEMENT	26538	8266	196	61.04966	108	1.769051	2.7354514769931599	2.834147e−12	5.668294e−10
82	trans_NEG	POSITIVE_REGULATION_OF_CELLULAR_PROCESS	26538	8266	654	203.70653	287	1.408890	1.75474208677953	2.914750e−12	9.618676e−09
83	cis_POS	SUBSTRATE_SPECIFIC_TRANSMEMBRANE_TRANSPORTER_ACTIVITY	26538	1529	339	19.53165	55	2.815942	3.2482479162735198	3.335102e−12	5.282802e−09
84	trans_NEG	HALLMARK_MYOGENESIS	26538	8266	199	61.98410	109	1.758516	2.6994655398804599	3.746636e−12	7.493273e−10
85	trans_NEG	RPS14_DN.V1_DN	26538	8266	183	57.00045	102	1.789459	2.80575819426815	4.452152e−12	3.365827e−09
86	trans_NEG	KEGG_HEMATOPOIETIC_CELL_LINEAGE	26538	8266	84	26.16414	57	2.178554	4.6917898019776896	4.897915e−12	3.644049e−09
87	trans_NEG	CELL_ACTIVATION	26538	8266	74	23.04936	52	2.256028	5.2508844751735202	5.151134e−12	1.699874e−08
88	trans_NEG	BIOPOLYMER_METABOLIC_PROCESS	26538	8266	1660	517.05328	644	1.245520	1.4350385324639501	5.207498e−12	1.718474e−08
89	cis_NEG	HALLMARK_P53_PATHWAY	26538	4004	199	30.02472	69	2.298106	3.0217698981495298	5.222133e−12	1.044427e−09
90	cis_POS	KEGG_CALCIUM_SIGNALING_PATHWAY	26538	1529	176	10.14033	37	3.648797	4.4365939240607197	5.418908e−12	4.031668e−09
91	cis_NEG	CELLULAR_MACROMOLECULE_METABOLIC_PROCESS	26538	4004	1112	167.77632	252	1.502000	1.69265055533028	5.614057e−12	1.852639e−08
92	trans_POS	CELL_ACTIVATION	26538	7352	74	20.50072	49	2.390160	5.1421338950995903	5.825262e−12	1.922337e−08
93	trans_POS	INTEGRAL_TO_PLASMA_MEMBRANE	26538	7352	946	262.07672	357	1.362197	1.61138552587003	5.832777e−12	5.436149e−09
94	cis_NEG	KEGG_CYTOKINE_CYTOKINE_RECEPTOR_INTERACTION	26538	4004	260	39.22828	83	2.115821	2.6734971956191398	5.947560e−12	4.424985e−09
95	cis_NEG	AKT_UP.V1_DN	26538	4004	187	28.21418	66	2.339249	3.10425202663914	6.053558e−12	4.576490e−09
96	cis_NEG	KEGG_AXON_GUIDANCE	26538	4004	127	19.16150	51	2.661586	3.8121986062831899	6.156834e−12	4.580685e−09
97	trans_POS	DNA_METABOLIC_PROCESS	26538	7352	253	70.09029	121	1.726345	2.4153778638010199	6.757560e−12	2.229995e−08
98	cis_NEG	REGULATION_OF_TRANSCRIPTION	26538	4004	560	84.49167	146	1.727981	2.0219535790332102	7.211666e−12	2.379850e−08
99	trans_NEG	REACTOME_HEMOSTASIS	26538	8266	446	138.91913	207	1.490076	1.93805012083308	7.474672e−12	2.015171e−08
100	cis_NEG	DNA_BINDING	26538	4004	597	90.07416	153	1.698600	1.9766602272307101	9.406053e−12	1.489919e−08

	set1_Name	set2_Name	Background	set1_size	set2_size	expected.overlap	actual.overlap	enrichment.foldchange	odds.ratio	FET_pvalue	corrected.FET.pvalue
1	trans_POS	RPS14_DN.V1_UP	26538	7352	188	52.082900	96	1.843215	2.7457690872961602	9.463075e−12	7.154085e−09
2	trans_NEG	HALLMARK_APICAL_JUNCTION	26538	8266	199	61.984098	108	1.742382	2.6448418101059201	1.011858e−11	2.023716e−09
3	cis_NEG	KEGG_B_CELL_RECEPTOR_SIGNALING_PATHWAY	26538	4004	74	11.164971	36	3.224370	5.3705274692750002	1.022742e−11	7.609204e−09
4	trans_NEG	CSR_LATE_UP.V1_DN	26538	8266	164	51.082372	93	1.820589	2.9168823014321301	1.038649e−11	7.852189e−09
5	cis_NEG	PROTEIN_AMINO_ACID_PHOSPHORYLATION	26538	4004	276	41.642324	86	2.065207	2.5811622934113001	1.059588e−11	3.496640e−08
6	trans_NEG	REACTOME_MITOTIC_PROMETAPHASE	26538	8266	83	25.852664	56	2.166121	4.6089207217461903	1.075801e−11	2.900359e−08
7	trans_NEG	REACTOME_INTEGRIN_CELL_SURFACE_INTERACTIONS	26538	8266	79	24.606753	54	2.194520	4.7991624528505099	1.138929e−11	3.070553e−08
8	cis_NEG	CELLULAR_PROTEIN_METABOLIC_PROCESS	26538	4004	1098	165.664029	248	1.497006	1.6843626481666401	1.193238e−11	3.937687e−08
9	trans_POS	INTRINSIC_TO_PLASMA_MEMBRANE	26538	7352	960	265.955234	360	1.353611	1.5948774871572	1.211776e−11	1.129376e−08
10	cis_NEG	HALLMARK_IL2_STAT5_SIGNALING	26538	4004	198	29.873841	68	2.276239	2.9772222531699999	1.220126e−11	2.440252e−09
11	cis_POS	HALLMARK_ESTROGEN_RESPONSE_EARLY	26538	1529	198	11.407868	39	3.418693	4.0905826960832101	1.223087e−11	2.446174e−09
12	trans_NEG	ESC_V6.5_UP_EARLY.V1_DN	26538	8266	169	52.639762	95	1.804719	2.8590474862041999	1.238650e−11	9.364193e−09
13	trans_NEG	NUCLEOBASENUCLEOSIDENUCLEOTIDE_AND_NUCLEIC_ACID_METABOLIC_PROCESS	26538	8266	1226	381.871882	490	1.283153	1.5013728726006701	1.368926e−11	4.517456e−08
14	trans_POS	P53_DN.V1_DN	26538	7352	189	52.359937	96	1.833463	2.7161042650912299	1.401944e−11	1.059869e−08
15	trans_POS	KEGG_SYSTEMIC_LUPUS_ERYTHEMATOSUS	26538	7352	124	34.352551	70	2.037694	3.4053787588959099	1.591068e−11	1.183755e−08
16	cis_NEG	KEGG_T_CELL_RECEPTOR_SIGNALING_PATHWAY	26538	4004	107	16.143945	45	2.787423	4.1196373118463896	1.629930e−11	1.212668e−08
17	trans_POS	HALLMARK_INFLAMMATORY_RESPONSE	26538	7352	200	55.407340	100	1.804815	2.6317178304468598	1.756709e−11	3.513419e−09
18	trans_POS	CSR_LATE_UP.V1_DN	26538	7352	164	45.434019	86	1.892855	2.89936807296698	1.821942e−11	1.377388e−08
19	cis_POS	RECEPTOR_ACTIVITY	26538	1529	569	32.783217	75	2.287756	2.55960812985668	1.876869e−11	2.972961e−08
20	cis_NEG	MTOR_UP.V1_DN	26538	4004	183	27.610671	64	2.317944	3.05949696175235	1.982593e−11	1.498840e−08
21	cis_NEG	REACTOME_COSTIMULATION_BY_THE_CD28_FAMILY	26538	4004	59	8.901801	31	3.482441	6.2709989577734104	2.155503e−11	5.811235e−08
22	cis_NEG	LYMPHOCYTE_ACTIVATION	26538	4004	59	8.901801	31	3.482441	6.2709989577734104	2.155503e−11	7.113158e−08
23	trans_POS	INTEGRAL_TO_MEMBRANE	26538	7352	1294	358.485493	465	1.297124	1.4950746227445699	2.202590e−11	2.052814e−08
24	cis_POS	KRAS.KIDNEY_UP.V1_UP	26538	1529	143	8.239016	32	3.883959	4.7937864284517504	2.580002e−11	1.950481e−08
25	cis_NEG	REGULATION_OF_NUCLEOBASENUCLEOSIDENUCLEOTIDE_AND_NUCLEIC_ACID_METABOLIC_PROCESS	26538	4004	610	92.035572	154	1.673266	1.9367008601761699	2.742077e−11	9.048855e−08
26	cis_NEG	REACTOME_PLATELET_ACTIVATION_SIGNALING_AND_AGGREGATION	26538	4004	197	29.722963	67	2.254149	2.9326972528006401	2.823724e−11	7.612759e−08
27	trans_POS	REACTOME_IMMUNE_SYSTEM	26538	7352	876	242.684151	331	1.363913	1.6124753073145499	2.937375e−11	7.919163e−08
28	trans_NEG	TRANSMEMBRANE_RECEPTOR_ACTIVITY	26538	8266	410	127.705931	191	1.495624	1.9498498509823301	3.206542e−11	5.079162e−08
29	trans_POS	T_CELL_ACTIVATION	26538	7352	43	11.912578	33	2.770181	8.6449845717813201	3.243348e−11	1.070305e−07
30	trans_NEG	CAMP_UP.V1_DN	26538	8266	195	60.738187	105	1.728731	2.5991357266022002	3.649163e−11	2.758768e−08
31	trans_POS	M_PHASE	26538	7352	114	31.582184	65	2.058122	3.4838859074986299	4.616829e−11	1.523554e−07
32	trans_NEG	HALLMARK_IL2_STAT5_SIGNALING	26538	8266	198	61.672620	106	1.718753	2.56688330740295	4.675426e−11	9.350852e−09
33	trans_POS	RECEPTOR_ACTIVITY	26538	7352	569	157.633883	229	1.452733	1.7819225454891101	4.679827e−11	7.412846e−08
34	trans_POS	SNF5_DN.V1_UP	26538	7352	174	48.204386	89	1.846305	2.7535440724459801	4.731774e−11	3.577221e−08
35	cis_POS	ION_TRANSMEMBRANE_TRANSPORTER_ACTIVITY	26538	1529	273	15.729030	46	2.924529	3.38602913460854	5.289014e−11	8.377798e−08
36	cis_NEG	CELL_ACTIVATION	26538	4004	74	11.164971	35	3.134804	5.0860258380261101	5.415153e−11	1.787000e−07
37	cis_POS	SUBSTRATE_SPECIFIC_TRANSPORTER_ACTIVITY	26538	1529	385	22.181965	57	2.569655	2.91358074859646	5.797295e−11	9.182915e−08
38	trans_NEG	PRC2_EZH2_UP.V1_UP	26538	8266	189	58.869319	102	1.732651	2.6114093424411702	5.797394e−11	4.382830e−08
39	cis_POS	REACTOME_NEURONAL_SYSTEM	26538	1529	274	15.786646	46	2.913855	3.371042775042	6.007787e−11	1.619699e−07
40	cis_NEG	KEGG_NATURAL_KILLER_CELL_MEDIATED_CYTOTOXICITY	26538	4004	126	19.010626	49	2.577506	3.6131274840708998	6.202418e−11	4.614599e−08
41	trans_POS	INTRINSIC_TO_MEMBRANE	26538	7352	1312	363.472153	468	1.287581	1.4774142529999099	6.434986e−11	5.997407e−08
42	cis_NEG	MEL18_DN.V1_DN	26538	4004	142	21.424674	53	2.473783	3.38275811304355	6.496922e−11	4.911673e−08
43	cis_NEG	TRANSCRIPTION_FACTOR_ACTIVITY	26538	4004	350	52.807295	100	1.893678	2.2831056164858698	6.628409e−11	1.049940e−07
44	cis_NEG	GCNP_SHH_UP_LATE.V1_DN	26538	4004	175	26.403648	61	2.310287	3.0423449103062001	6.790864e−11	5.133893e−08
45	trans_POS	RPS14_DN.V1_DN	26538	7352	183	50.697716	92	1.814677	2.6589572531397101	7.609555e−11	5.752824e−08
46	trans_POS	HALLMARK_KRAS_SIGNALING_UP	26538	7352	199	55.130304	98	1.777607	2.5527166205065002	8.375638e−11	1.675128e−08
47	cis_NEG	KEGG_TYPE_I_DIABETES_MELLITUS	26538	4004	37	5.582485	23	4.120029	9.2921043731868895	8.474758e−11	6.305220e−08
48	cis_NEG	CELL_JUNCTION	26538	4004	82	12.371995	37	2.990625	4.6609986717199599	8.640177e−11	8.052645e−08
49	cis_NEG	REGULATION_OF_SIGNAL_TRANSDUCTION	26538	4004	219	33.042279	71	2.148762	2.7301797398400001	8.939757e−11	2.950120e−07
50	trans_POS	CSR_LATE_UP.V1_UP	26538	7352	168	46.542166	86	1.847787	2.7573657122812598	9.432482e−11	7.130957e−08
51	cis_NEG	KEGG_VIRAL_MYOCARDITIS	26538	4004	65	9.807069	32	3.262952	5.4927642364012099	9.608907e−11	7.149027e−08
52	trans_NEG	KEGG_ECM_RECEPTOR_INTERACTION	26538	8266	84	26.164142	55	2.102114	4.2139256023528704	9.790743e−11	7.284313e−08
53	trans_NEG	REACTOME_G2_M_CHECKPOINTS	26538	8266	41	12.770593	33	2.584062	9.1500211034146304	9.987576e−11	2.692650e−07
54	cis_NEG	KEGG_INTESTINAL_IMMUNE_NETWORK_FOR_IGA_PRODUCTION	26538	4004	43	6.487753	25	3.853414	7.8582512687105401	1.014923e−10	7.551028e−08
55	cis_POS	ORGAN_DEVELOPMENT	26538	1529	567	32.667986	73	2.234604	2.4879563346846001	1.044056e−10	3.445386e−07
56	trans_NEG	MITOTIC_CELL_CYCLE	26538	8266	153	47.656116	86	1.804595	2.8565516982378001	1.143250e−10	3.772726e−07
57	cis_NEG	NEGATIVE_REGULATION_OF_DEVELOPMENTAL_PROCESS	26538	4004	194	29.270329	65	2.220679	2.86588582213597	1.146703e−10	3.784121e−07
58	cis_NEG	EGFR_UP.V1_DN	26538	4004	194	29.270329	65	2.220679	2.86588582213597	1.146703e−10	8.669077e−08
59	trans_NEG	CORDENONSI_YAP_CONSERVED_SIGNATURE	26538	8266	56	17.442761	41	2.350545	6.0665144520977101	1.160450e−10	8.773003e−08
60	cis_NEG	KEGG_ALLOGRAFT_REJECTION	26538	4004	32	4.828096	21	4.349541	10.793951701707501	1.271797e−10	9.462168e−08
61	trans_POS	REACTOME_CHROMOSOME_MAINTENANCE	26538	7352	116	32.136257	65	2.022638	3.3464765070517601	1.286940e−10	3.469592e−07
62	cis_NEG	ANATOMICAL_STRUCTURE_MORPHOGENESIS	26538	4004	373	56.277489	104	1.847986	2.2071074111722302	1.311615e−10	4.328329e−07
63	cis_NEG	KEGG_FOCAL_ADHESION	26538	4004	199	30.024719	66	2.198189	2.8226737567693299	1.359645e−10	1.011576e−07
64	trans_NEG	P53_DN.V1_DN	26538	8266	189	58.869319	101	1.715664	2.5559742719316598	1.515843e−10	1.145977e−07
65	trans_NEG	KEGG_CYTOKINE_CYTOKINE_RECEPTOR_INTERACTION	26538	8266	260	80.984249	130	1.605250	2.22980261294629	1.579567e−10	1.175198e−07
66	cis_POS	KEGG_CELL_ADHESION_MOLECULES_CAMS	26538	1529	128	7.374783	29	3.932319	4.8639932347419501	1.591442e−10	1.184033e−07
67	cis_NEG	IDENTICAL_PROTEIN_BINDING	26538	4004	299	45.112518	88	1.950678	2.3771755958336702	1.732448e−10	2.744198e−07
68	cis_NEG	CELL_SURFACE_RECEPTOR_LINKED_SIGNAL_TRANSDUCTION_GO_0007166	26538	4004	635	95.807521	156	1.628265	1.8665237012689699	1.803612e−10	5.951921e−07
69	trans_NEG	GCNP_SHH_UP_LATE.V1_UP	26538	8266	180	56.066019	97	1.730103	2.6020607403453799	1.885467e−10	1.425413e−07
70	trans_POS	RESPONSE_TO_STRESS	26538	7352	496	137.410204	202	1.470051	1.8152977425952299	1.946507e−10	6.423472e−07
71	cis_POS	KRAS.600_UP.V1_UP	26538	1529	274	15.786646	45	2.850511	3.2810271380049101	2.036902e−10	1.539898e−07
72	cis_NEG	REGULATION_OF_RNA_METABOLIC_PROCESS	26538	4004	464	70.007386	122	1.742673	2.03924260053138	2.139653e−10	7.060856e−07
73	trans_NEG	REGULATION_OF_DEVELOPMENTAL_PROCESS	26538	8266	431	134.246967	196	1.459996	1.86418729065927	2.332638e−10	7.697705e−07
74	cis_NEG	PHOSPHORYLATION	26538	4004	310	46.772176	90	1.924221	2.3321603529094102	2.367999e−10	7.814396e−07
75	trans_NEG	MTOR_UP.V1_DN	26538	8266	183	57.000452	98	1.719285	2.5670649819962401	2.417450e−10	1.827592e−07
76	trans_NEG	E2F3_UP.V1_UP	26538	8266	183	57.000452	98	1.719285	2.5670649819962401	2.417450e−10	1.827592e−07
77	trans_POS	REACTOME_ADAPTIVE_IMMUNE_SYSTEM	26538	7352	503	139.349461	204	1.463945	1.80264808787135	2.427304e−10	6.544012e−07
78	cis_NEG	BIOCARTA_CTL_PATHWAY	26538	4004	14	2.112292	13	6.154453	73.384249265328506	2.487222e−10	2.158909e−07
79	trans_POS	REACTOME_G2_M_CHECKPOINTS	26538	7352	41	11.358505	31	2.729232	8.1187388039225308	2.503601e−10	6.749708e−07
80	trans_POS	KEGG_HEMATOPOIETIC_CELL_LINEAGE	26538	7352	84	23.271083	51	2.191561	4.05397596038849	2.507128e−10	1.865304e−07
81	cis_NEG	LEUKOCYTE_ACTIVATION	26538	4004	67	10.108825	32	3.165551	5.17850148692375	2.612721e−10	8.621981e−07
82	trans_POS	PRC2_EZH2_UP.V1_UP	26538	7352	189	52.359937	93	1.776167	2.5475588462715399	2.647966e−10	2.001862e−07
83	cis_NEG	CAHOY_OLIGODENDROCUTIC	26538	4004	96	14.484287	40	2.761613	4.0501916137945102	3.008093e−10	2.274118e−07
84	trans_NEG	NON_MEMBRANE_BOUND_ORGANELLE	26538	8266	623	194.050720	267	1.375929	1.67980677956334	3.212145e−10	2.993719e−07
85	trans_NEG	INTRACELLULAR_NON_MEMBRANE_BOUND_ORGANELLE	26538	8266	623	194.050720	267	1.375929	1.67980677956334	3.212145e−10	2.993719e−07
86	cis_NEG	LTE2_UP.V1_DN	26538	4004	194	29.270329	64	2.186515	2.7993259261986401	3.299559e−10	2.494467e−07
87	trans_POS	REACTOME_DNA_STRAND_ELONGATION	26538	7352	30	8.311101	25	3.008025	13.0843629227557	3.424001e−10	9.231106e−07
88	trans_POS	REACTOME_HEMOSTASIS	26538	7352	446	123.558369	184	1.489175	1.8539530020809101	3.651741e−10	9.845094e−07
89	trans_NEG	REACTOME_ACTIVATION_OF_ATR_IN_RESPONSE_TO_REPLICATION_STRESS	26538	8266	35	10.901726	29	2.660129	10.7146715117436	3.763085e−10	1.014528e−06
90	cis_NEG	RECEPTOR_ACTIVITY	26538	4004	569	85.849574	142	1.654056	1.9034515579875499	3.796939e−10	6.014351e−07
91	trans_NEG	AKT_UP_MTOR_DN.V1_DN	26538	8266	182	56.688974	97	1.711091	2.5405618545295301	4.202769e−10	3.177293e−07
92	trans_NEG	REACTOME_DNA_STRAND_ELONGATION	26538	8266	30	9.344336	26	2.782434	14.4109993444001	4.344498e−10	1.171277e−06
93	trans_NEG	HALLMARK_ESTROGEN_RESPONSE_LATE	26538	8266	199	61.984098	104	1.677850	2.4379344204617901	4.410252e−10	8.820504e−08
94	cis_NEG	SNF5_DN.V1_UP	26538	4004	174	26.252770	59	2.247382	2.9154069565631802	4.786273e−10	3.618422e−07
95	trans_POS	INTRACELLULAR_SIGNALING_CASCADE	26538	7352	657	182.013113	254	1.395504	1.66781669709939	4.850571e−10	1.600688e−06
96	cis_NEG	REGULATION_OF_TRANSCRIPTIONDNA_DEPENDENT	26538	4004	455	68.649484	119	1.733443	2.0236084485128898	5.046670e−10	1.665401e−06
97	trans_POS	MTOR_UP.V1_DN	26538	7352	183	50.697716	90	1.775228	2.5442566688553101	5.283420e−10	3.994265e−07
98	cis_NEG	REACTOME_GPVI_MEDIATED_ACTIVATION_CASCADE	26538	4004	31	4.677218	20	4.276047	10.279846976016801	5.578117e−10	1.503860e−06
99	trans_NEG	RESPONSE_TO_STRESS	26538	8266	496	154.493029	219	1.417540	1.7678797399524999	5.655483e−10	1.866309e−06
100	cis_POS	HALLMARK_UV_RESPONSE_DN	26538	1529	143	8.239016	30	3.641212	4.4088720786042002	5.702073e−10	1.140415e−07

	set1_Name	set2_Name	Background	set1_size	set2_size	expected.overlap	actual.overlap	enrichment.foldchange	odds.ratio	FET_pvalue	corrected.FET.pvalue
1	cis_NEG	CAHOY_ASTROGLIAL	26538	4004	98	14.786043	40	2.705254	3.91015120974772	6.320253e−10	4.778111e−07
2	cis_POS	KEGG_AXON_GUIDANCE	26538	1529	127	7.317168	28	3.826617	4.6931724337303402	6.398267e−10	4.760310e−07
3	cis_NEG	ATF2_S_UP.V1_DN	26538	4004	184	27.761549	61	2.197284	2.818624972177	6.806421e−10	5.145654e−07
4	cis_POS	STK33_NOMO_DN	26538	1529	275	15.844261	44	2.777031	3.1779516281293101	7.568177e−10	5.721541e−07
5	cis_NEG	HOXA9_DN.V1_UP	26538	4004	189	28.515939	62	2.174223	2.7749408954695398	7.985996e−10	6.037413e−07
6	cis_NEG	ESC_V6.5_UP_LATE.V1_UP	26538	4004	185	27.912427	61	2.185406	2.7958425189645499	8.672191e−10	6.556176e−07
7	trans_NEG	HALLMARK_MYC_TARGETS_V1	26538	8266	196	61.049665	102	1.670771	2.41603130951875	8.773064e−10	1.754613e−07
8	trans_NEG	REGULATION_OF_CELL_PROLIFERATION	26538	8266	301	93.754842	144	1.535921	2.04568181479689	8.846832e−10	2.919454e−06
9	cis_POS	HALLMARK_EPITHELIAL_MESENCHYMAL_TRANSITION	26538	1529	199	11.465483	36	3.139859	3.67504735740413	8.847237e−10	1.769447e−07
10	cis_NEG	NOTCH_DN.V1_UP	26538	4004	181	27.308916	60	2.197085	2.8177836247752799	9.394161e−10	7.101986e−07
11	trans_NEG	NUCLEUS	26538	8266	1404	437.314945	541	1.237095	1.41274375148512	9.499176e−10	8.853232e−07
12	trans_NEG	REACTOME_G1_S_TRANSITION	26538	8266	105	32.705177	63	1.926301	3.3335404392978099	9.612405e−10	2.591504e−06
13	trans_NEG	T_CELL_ACTIVATION	26538	8266	43	13.393549	33	2.463873	7.3191925058081901	9.805584e−10	3.235843e−06
14	trans_NEG	M_PHASE	26538	8266	114	35.508478	67	1.886873	3.16856354623989	9.879071e−10	3.260093e−06
15	cis_NEG	MEL18_DN.V1_UP	26538	4004	139	20.972040	50	2.384127	3.1888670354108899	9.934230e−10	7.510278e−07
16	cis_NEG	MEK_UP.V1_DN	26538	4004	190	28.666817	62	2.162779	2.7531931947867299	1.012333e−09	7.653238e−07
17	cis_NEG	I_KAPPAB_KINASE_NF_KAPPAB_CASCADE	26538	4004	111	16.747456	43	2.567554	3.5863524283938499	1.035952e−09	3.418640e−06
18	cis_NEG	KRAS.600_UP.V1_DN	26538	4004	276	41.642324	81	1.945136	2.3653078658800899	1.050170e−09	7.939282e−07
19	cis_POS	REACTOME_TRANSMEMBRANE_TRANSPORT_OF_SMALL_MOLECULES	26538	1529	404	23.276660	56	2.405843	2.6937693579372199	1.064412e−09	2.869655e−06
20	trans_NEG	CELL_SURFACE_RECEPTOR_LINKED_SIGNAL_TRANSDUCTION_GO_0007166	26538	8266	635	197.788454	269	1.360039	1.64564113833528	1.107941e−09	3.656204e−06
21	cis_NEG	AKT_UP_MTOR_DN.V1_DN	26538	4004	182	27.459794	60	2.185013	2.7946361473040602	1.196874e−09	9.048367e−07
22	cis_POS	ANATOMICAL_STRUCTURE_MORPHOGENESIS	26538	1529	373	21.490580	53	2.466197	2.7702526474522502	1.223404e−09	4.037233e−06
23	cis_NEG	RESPONSE_TO_EXTERNAL_STIMULUS	26538	4004	305	46.017786	87	1.890573	2.27357018980004	1.226756e−09	4.048294e−06
24	cis_NEG	KRAS.DF.V1_UP	26538	4004	191	28.817695	62	2.151456	2.7313199051732302	1.280036e−09	9.677071e−07
25	cis_POS	CAHOY_NEURONAL	26538	1529	99	5.703934	24	4.207622	5.3005534151321303	1.383199e−09	1.045698e−06
26	cis_NEG	KEGG_PRIMARY_IMMUNODEFICIENCY	26538	4004	35	5.280730	21	3.976723	8.4798703853423198	1.440383e−09	1.071645e−06
27	trans_NEG	HALLMARK_IL6_JAK_STAT3_SIGNALING	26538	8266	86	26.787098	54	2.015896	3.7479317730207899	1.450028e−09	2.900057e−07
28	trans_POS	STK33_SKM_UP	26538	7352	268	74.245836	120	1.616252	2.1344128493989301	1.466930e−09	1.108999e−06
29	trans_POS	NUCLEOBASENUCLEOSIDENUCLEOTIDE_AND_NUCLEIC_ACID_METABOLIC_PROCESS	26538	7352	1226	339.646997	433	1.274853	1.4515009819048501	1.502143e−09	4.957073e−06
30	cis_NEG	IL2_UP.V1_UP	26538	4004	183	27.610671	60	2.173073	2.7718556271940602	1.520821e−09	1.149740e−06
31	trans_NEG	REACTOME_CELL_SURFACE_INTERACTIONS_AT_THE_VASCULAR_WALL	26538	8266	84	26.164142	53	2.025673	3.7969029218412	1.593700e−09	4.296615e−06
32	trans_NEG	HALLMARK_ESTROGEN_RESPONSE_EARLY	26538	8266	198	61.672620	102	1.653894	2.3654434343756301	1.812721e−09	3.625443e−07
33	trans_POS	POSITIVE_REGULATION_OF_BIOLOGICAL_PROCESS	26538	7352	693	191.986435	263	1.369888	1.61820601062008	1.966904e−09	6.490782e−06
34	trans_NEG	RESPONSE_TO_WOUNDING	26538	8266	184	57.311930	96	1.675044	2.4279672086746702	2.274181e−09	7.504796e−06
35	cis_NEG	REACTOME_INTEGRIN_CELL_SURFACE_INTERACTIONS	26538	4004	79	11.919361	34	2.852502	4.2799000243031697	2.296070e−09	6.190204e−06
36	cis_POS	REACTOME_HEMOSTASIS	26538	1529	446	25.696511	59	2.296032	2.55340108010168	2.332307e−09	6.287899e−06
37	cis_NEG	REACTOME_IMMUNOREGULATORY_INTERACTIONS_BETWEEN_A_LYMPHOID_AND_A_NON_LYMPHOID_CELL	26538	4004	58	8.750923	28	3.199662	5.2822168584958096	2.499641e−09	6.739033e−06
38	trans_POS	DEFENSE_RESPONSE	26538	7352	256	70.921396	115	1.621513	2.1463301534074799	2.530238e−09	8.349786e−06
39	cis_NEG	KRAS.DF.V1_DN	26538	4004	190	28.666817	61	2.127896	2.6866691253391499	2.801183e−09	2.117694e−06
40	cis_NEG	WNT_UP.V1_DN	26538	4004	164	24.743990	55	2.222762	2.8652186722706801	2.876220e−09	2.174423e−06
41	cis_NEG	SNF5_DN.V1_DN	26538	4004	160	24.140478	54	2.236907	2.8923932859181498	3.089117e−09	2.335373e−06
42	trans_POS	HALLMARK_MYOGENESIS	26538	7352	199	55.130304	94	1.705051	2.3534782938610701	3.190676e−09	6.381351e−07
43	cis_NEG	REACTOME_GENERATION_OF_SECOND_MESSENGER_MOLECULES	26538	4004	25	3.771950	17	4.506953	12.0005478684712	3.366011e−09	9.074766e−06
44	trans_NEG	REACTOME_MITOTIC_G1_G1_S_PHASES	26538	8266	128	39.869169	72	1.805907	2.85813397724763	3.464295e−09	9.339739e−06
45	trans_NEG	APOPTOSIS_GO	26538	8266	422	131.443666	188	1.430271	1.7938847700610501	3.958483e−09	1.306300e−05
46	trans_NEG	HALLMARK_APOPTOSIS	26538	8266	159	49.524983	85	1.716305	2.5549891036378201	4.064863e−09	8.129726e−07
47	cis_POS	TRANSMEMBRANE_RECEPTOR_ACTIVITY	26538	1529	410	23.622353	55	2.328303	2.5911467756227502	4.920847e−09	7.794621e−06
48	trans_NEG	PROGRAMMED_CELL_DEATH	26538	8266	423	131.755144	188	1.426889	1.78615537649526	4.946819e−09	1.632450e−05
49	trans_NEG	REACTOME_ACTIVATION_OF_THE_PRE_REPLICATIVE_COMPLEX	26538	8266	30	9.344336	25	2.675417	11.0785676346882	5.114053e−09	1.378749e−05
50	trans_NEG	HALLMARK_INTERFERON_ALPHA_RESPONSE	26538	8266	97	30.213354	58	1.919681	3.3034475729772899	5.270903e−09	1.054181e−06
51	cis_NEG	CYCLIN_D1_UP.V1_UP	26538	4004	184	27.761549	59	2.125242	2.6808648842003899	5.326771e−09	4.027039e−06
52	trans_NEG	REACTOME_S_PHASE	26538	8266	106	33.016655	62	1.877840	3.13062786081856	5.337890e−09	1.439095e−05
53	cis_NEG	NFE2L2.V2	26538	4004	442	66.688070	113	1.694456	1.96009373949746	5.523984e−09	4.176132e−06
54	trans_NEG	REACTOME_GENERATION_OF_SECOND_MESSENGER_MOLECULES	26538	8266	25	7.786947	22	2.825241	16.250675489808799	5.614778e−09	1.513744e−05
55	cis_NEG	KRAS.600.LUNG.BREAST_UP.V1_DN	26538	4004	281	42.396714	80	1.886939	2.26513960819051	6.145075e−09	4.645677e−06
56	cis_POS	ESTABLISHMENT_OF_LOCALIZATION	26538	1529	857	49.376479	92	1.863235	2.0288911952775299	6.393924e−09	2.109995e−05
57	trans_POS	REGULATION_OF_IMMUNE_SYSTEM_PROCESS	26538	7352	64	17.730349	40	2.256019	4.3673734177284702	6.420169e−09	2.118656e−05
58	trans_NEG	TBK1.DF_UP	26538	8266	285	88.771196	135	1.520764	2.0058880363365801	6.437775e−09	4.866958e−06
59	trans_POS	MITOTIC_CELL_CYCLE	26538	7352	153	42.386615	76	1.793019	2.59209478508008	6.447816e−09	2.127779e−05
60	trans_NEG	KEGG_INTESTINAL_IMMUNE_NETWORK_FOR_IGA_PRODUCTION	26538	8266	43	13.393549	32	2.389210	6.4509971476272101	6.655637e−09	4.951794e−06
61	trans_POS	REACTOME_GENERATION_OF_SECOND_MESSENGER_MOLECULES	26538	7352	25	6.925918	21	3.032089	13.7300220922316	7.158485e−09	1.929927e−05
62	cis_POS	STK33_SKM_DN	26538	1529	265	15.268106	41	2.685336	3.0485304277816301	7.747555e−09	5.857152e−06
63	trans_NEG	EXTRACELLULAR_MATRIX_PART	26538	8266	57	17.754239	39	2.196658	4.8070412527719002	7.904163e−09	7.366680e−06
64	trans_NEG	EGFR_UP.V1_UP	26538	8266	190	59.180797	97	1.639045	2.32102252303071	8.167998e−09	6.175007e−06
65	trans_NEG	KEGG_LEUKOCYTE_TRANSENDOTHELIAL_MIGRATION	26538	8266	116	36.131434	66	1.826664	2.9331654617770302	8.222871e−09	6.117816e−06
66	cis_POS	AKT_UP.V1_DN	26538	1529	187	10.774098	33	3.062902	3.5598981165669001	8.360613e−09	6.320623e−06
67	trans_POS	REACTOME_G1_S_TRANSITION	26538	7352	105	29.088854	57	1.959513	3.11503510522639	8.419203e−09	2.269817e−05
68	trans_NEG	CAHOY_ASTROGLIAL	26538	8266	98	30.524832	58	1.900092	3.2206496353634502	8.999307e−09	6.803476e−06
69	cis_NEG	REACTOME_CELL_CELL_COMMUNICATION	26538	4004	118	17.803602	43	2.415242	3.2506171520274201	9.172212e−09	2.472828e−05
70	trans_NEG	CELL_DEVELOPMENT	26538	8266	567	176.607958	240	1.358942	1.64096837088508	9.389723e−09	3.098609e−05
71	trans_POS	STK33_NOMO_DN	26538	7352	275	76.185093	120	1.575111	2.03729794287	9.424630e−09	7.125020e−06
72	cis_NEG	REACTOME_SIGNALING_BY_ILS	26538	4004	106	15.993067	40	2.501084	3.4349400351716599	9.469838e−09	2.553068e−05
73	cis_NEG	HALLMARK_GLYCOLYSIS	26538	4004	200	30.175597	62	2.054640	2.5523678896667001	9.490146e−09	1.898029e−06
74	cis_NEG	KRAS.300_UP.V1_DN	26538	4004	139	20.972040	48	2.288762	2.9922613802798401	9.839831e−09	7.438912e−06
75	trans_NEG	M_PHASE_OF_MITOTIC_CELL_CYCLE	26538	8266	85	26.475620	52	1.964071	3.4986534590983802	1.071519e−08	3.536013e−05
76	trans_NEG	KEGG_CHEMOKINE_SIGNALING_PATHWAY	26538	8266	186	57.934886	95	1.639772	2.3228127417725002	1.125295e−08	8.372194e−06
77	cis_NEG	REGULATION_OF_BIOLOGICAL_QUALITY	26538	4004	412	62.161730	106	1.705229	1.9753566506109099	1.131247e−08	3.733115e−05
78	cis_NEG	ATF2_UP.V1_DN	26538	4004	183	27.610671	58	2.100637	2.6348173590746802	1.151695e−08	8.706817e−06
79	cis_NEG	KEGG_REGULATION_OF_ACTIN_CYTOSKELETON	26538	4004	210	31.684377	64	2.019923	2.4907760915872399	1.156679e−08	8.605692e−06
80	cis_NEG	ERB2_UP.V1_DN	26538	4004	192	28.968573	60	2.071210	2.5816746908286401	1.171146e−08	8.853866e−06
81	cis_POS	VOLTAGE_GATED_CHANNEL_ACTIVITY	26538	1529	71	4.090700	19	4.644682	6.0381335556956	1.231085e−08	1.950038e−05
82	trans_POS	CORDENONSI_YAP_CONSERVED_SIGNATURE	26538	7352	56	15.514055	36	2.320476	4.7152313337620102	1.232528e−08	9.317915e−06
83	cis_POS	METAL_ION_TRANSMEMBRANE_TRANSPORTER_ACTIVITY	26538	1529	144	8.296631	28	3.374864	4.0029996976352296	1.253196e−08	1.985063e−05
84	cis_NEG	EIF4E_DN	26538	4004	99	14.936921	38	2.544032	3.5296726201645598	1.277762e−08	9.659881e−06
85	trans_POS	HALLMARK_IL2_STAT5_SIGNALING	26538	7352	198	54.853267	92	1.677202	2.2809379048697398	1.289143e−08	2.578286e−06
86	trans_NEG	CYTOPLASM	26538	8266	2087	650.054337	765	1.176825	1.3076501234754201	1.317737e−08	1.228131e−05
87	trans_POS	REACTOME_CELL_CYCLE_CHECKPOINTS	26538	7352	111	30.751074	59	1.918632	2.9766469511963698	1.320078e−08	3.558931e−05
88	trans_POS	REACTOME_S_PHASE	26538	7352	106	29.365890	57	1.941027	3.0516452645869498	1.330982e−08	3.588326e−05
89	trans_NEG	INFLAMMATORY_RESPONSE	26538	8266	124	38.623257	69	1.786488	2.7879824042190799	1.331817e−08	4.394997e−05
90	trans_POS	REACTOME_ACTIVATION_OF_ATR_IN_RESPONSE_TO_REPLICATION_STRESS	26538	7352	35	9.696285	26	2.681439	7.5609548802538402	1.362925e−08	3.674446e−05
91	cis_POS	CATION_TRANSMEMBRANE_TRANSPORTER_ACTIVITY	26538	1529	210	12.099254	35	2.892740	3.3242195632745499	1.383231e−08	2.191037e−05
92	trans_NEG	HALLMARK_MITOTIC_SPINDLE	26538	8266	199	61.984098	100	1.613317	2.2478830784857302	1.400110e−08	2.800220e−06
93	cis_POS	TRANSPORT	26538	1529	783	45.112932	85	1.884160	2.050139587251	1.463550e−08	4.829716e−05
94	cis_POS	STK33_DN	26538	1529	271	15.613799	41	2.625882	2.9682936479133302	1.494155e−08	1.129581e−05
95	cis_NEG	KEGG_HEMATOPOIETIC_CELL_LINEAGE	26538	4004	84	12.673751	34	2.682710	3.85094187983547	1.532536e−08	1.140207e−05
96	cis_POS	REACTOME_TRANSMISSION_ACROSS_CHEMICAL_SYNAPSES	26538	1529	182	10.486020	32	3.051682	3.5422796829808298	1.533744e−08	4.134974e−05
97	cis_NEG	REGULATION_OF_CELL_PROLIFERATION	26538	4004	301	45.414274	83	1.827619	2.16684022026014	1.607795e−08	5.305725e−05
98	trans_POS	POSITIVE_REGULATION_OF_CELLULAR_PROCESS	26538	7352	654	181.182003	246	1.357751	1.5932746377806899	1.616907e−08	5.335792e−05
99	cis_NEG	BIOCARTA_TCR_PATHWAY	26538	4004	45	6.789509	23	3.387579	5.9113112754731896	1.659820e−08	1.440724e−05
100	trans_NEG	STK33_SKM_UP	26538	8266	268	83.476072	127	1.521394	2.0064914870684101	1.726308e−08	1.305089e−05

	set1_Name	set2_Name	Background	set1_size	set2_size	expected.overlap	actual.overlap	enrichment.foldchange	odds.ratio	FET_pvalue	corrected.FET.pvalue
1	cis_POS	MTOR_UP.V1_DN	26538	1529	183	10.543636	32	3.035006	3.5186805674107098	1.757497e−08	1.328667e−05
2	cis_NEG	PHOSPHOTRANSFERASE_ACTIVITY_ALCOHOL_GROUP_AS_ACCEPTOR	26538	4004	331	49.940613	89	1.782117	2.0940244583795802	1.838229e−08	2.911755e−05
3	trans_NEG	DNA_METABOLIC_PROCESS	26538	8266	253	78.803904	121	1.535457	2.04153955969816	1.923615e−08	6.347929e−05
4	cis_NEG	KEGG_TIGHT_JUNCTION	26538	4004	129	19.463260	45	2.312048	3.0376569066633601	1.977933e−08	1.471582e−05
5	cis_NEG	HALLMARK_MTORC1_SIGNALING	26538	4004	199	30.024719	61	2.031659	2.5105769944459402	1.984557e−08	3.969113e−06
6	cis_NEG	REGULATION_OF_MULTICELLULAR_ORGANISMAL_PROCESS	26538	4004	146	22.028186	49	2.224423	2.8656219630516402	1.993461e−08	6.578422e−05
7	trans_NEG	EXTRACELLULAR_REGION	26538	8266	435	135.492878	190	1.402288	1.7310447458148699	2.061718e−08	1.921521e−05
8	trans_NEG	POSITIVE_REGULATION_OF_CELL_PROLIFERATION	26538	8266	144	44.852815	77	1.716726	2.5548201081924802	2.110995e−08	6.966285e−05
9	cis_NEG	KEGG_GRAFT_VERSUS_HOST_DISEASE	26538	4004	33	4.978974	19	3.816048	7.66844595822492	2.277391e−08	1.694379e−05
10	cis_NEG	RELA_DN.V1_DN	26538	4004	138	20.821162	47	2.257319	2.9291860862472898	2.289329e−08	1.730732e−05
11	trans_POS	ANATOMICAL_STRUCTURE_DEVELOPMENT	26538	7352	993	275.097445	353	1.283182	1.4615115023046801	2.313892e−08	7.635843e−05
12	trans_POS	M_PHASE_OF_MITOTIC_CELL_CYCLE	26538	7352	85	23.548120	48	2.038379	3.4007904283027899	2.329652e−08	7.687853e−05
13	trans_NEG	KEGG_GRAFT_VERSUS_HOST_DISEASE	26538	8266	33	10.278770	26	2.529486	8.2324299587048806	2.338777e−08	1.740050e−05
14	cis_NEG	KEGG_GLUTATHIONE_METABOLISM	26538	4004	49	7.393021	24	3.246305	5.4288496537491602	2.390774e−08	1.778736e−05
15	trans_POS	MULTICELLULAR_ORGANISMAL_DEVELOPMENT	26538	7352	1029	285.070766	364	1.276876	1.45072354974471	2.571122e−08	8.484704e−05
16	trans_POS	CHROMOSOMAL_PART	26538	7352	95	26.318487	52	1.975797	3.17099166419638	2.581109e−08	2.405594e−05
17	trans_POS	REACTOME_MITOTIC_G1_G1_S_PHASES	26538	7352	128	35.460698	65	1.833015	2.7074561085009998	2.629466e−08	7.089041e−05
18	trans_NEG	ORGANELLE_PART	26538	8266	1177	366.609466	453	1.235647	1.4053076746999	2.723758e−08	2.538542e−05
19	cis_NEG	ESC_J1_UP_EARLY.V1_UP	26538	4004	178	26.856282	56	2.085173	2.60557314873614	2.726556e−08	2.061276e−05
20	cis_POS	REACTOME_INTEGRATION_OF_ENERGY_METABOLISM	26538	1529	114	6.568166	24	3.653988	4.4148851055314502	2.730786e−08	7.362199e−05
21	trans_NEG	KEGG_PRIMARY_IMMUNODEFICIENCY	26538	8266	35	10.901726	27	2.476672	7.4809127008335299	2.797381e−08	2.081251e−05
22	trans_NEG	STK33_NOMO_DN	26538	8266	275	85.656417	129	1.506017	1.9682428260528	2.848943e−08	2.153801e−05
23	cis_NEG	TRANSFERASE_ACTIVITY_TRANSFERRING_PHOSPHORUS_CONTAINING_GROUPS	26538	4004	419	63.217876	106	1.676741	1.93060472815539	2.925751e−08	4.634390e−05
24	cis_NEG	KINASE_ACTIVITY	26538	4004	364	54.919587	95	1.729802	2.0115282456526198	3.038760e−08	4.813396e−05
25	cis_NEG	KRAS.600.LUNG.BREAST_UP.V1_UP	26538	4004	276	41.642324	77	1.849080	2.2006311936733098	3.044157e−08	2.301383e−05
26	cis_NEG	CELL_CELL_ADHESION	26538	4004	86	12.975507	34	2.620322	3.7024837020919601	3.091919e−08	1.020333e−04
27	cis_NEG	HALLMARK_IL6_JAK_STAT3_SIGNALING	26538	4004	86	12.975507	34	2.620322	3.7024837020919601	3.091919e−08	6.183838e−06
28	cis_NEG	T_CELL_ACTIVATION	26538	4004	43	6.487753	22	3.391004	5.9223239063375797	3.318687e−08	1.095167e−04
29	cis_POS	ESC_V6.5_UP_EARLY.V1_DN	26538	1529	169	9.737019	30	3.081025	3.5804910083037198	3.418316e−08	2.584247e−05
30	trans_POS	REACTOME_ACTIVATION_OF_THE_PRE_REPLICATIVE_COMPLEX	26538	7352	30	8.311101	23	2.767383	8.5971606742282205	3.489516e−08	9.407736e−05
31	cis_POS	GENERATION_OF_NEURONS	26538	1529	83	4.782086	20	4.182275	5.2471983490715601	3.574592e−08	1.179615e−04
32	trans_POS	REACTOME_E2F_MEDIATED_REGULATION_OF_DNA_REPLICATION	26538	7352	32	8.865174	24	2.707223	7.8501054683096401	3.650138e−08	9.840771e−05
33	trans_NEG	REACTOME_INTERFERON_GAMMA_SIGNALING	26538	8266	57	17.754239	38	2.140334	4.4364929499614396	3.673667e−08	9.904206e−05
34	trans_POS	REGULATION_OF_LYMPHOCYTE_ACTIVATION	26538	7352	34	9.419248	25	2.654140	7.2690705080509304	3.682095e−08	1.215091e−04
35	trans_POS	ORGAN_DEVELOPMENT	26538	7352	567	157.079810	216	1.375097	1.6242340416671199	3.778184e−08	1.246801e−04
36	cis_NEG	REGULATION_OF_IMMUNE_SYSTEM_PROCESS	26538	4004	64	9.656191	28	2.899694	4.4009232610001199	3.789368e−08	1.250491e−04
37	trans_NEG	RESPONSE_TO_EXTERNAL_STIMULUS	26538	8266	305	95.000754	140	1.473673	1.8907005035516	4.052158e−08	1.337212e−04
38	cis_NEG	REACTOME_INTERFERON_GAMMA_SIGNALING	26538	4004	57	8.600045	26	3.023240	4.7442398352217801	4.055340e−08	1.093320e−04
39	trans_POS	ORGANELLE_ORGANIZATION_AND_BIOGENESIS	26538	7352	467	129.376140	183	1.414480	1.69891731613351	4.082704e−08	1.347292e−04
40	cis_POS	NEURON_DIFFERENTIATION	26538	1529	76	4.378778	19	4.339111	5.5080932233093103	4.121582e−08	1.360122e−04
41	trans_POS	KEGG_CELL_CYCLE	26538	7352	124	34.352551	63	1.833925	2.7097270560524098	4.175905e−08	3.106873e−05
42	trans_NEG	CELL_CYCLE_CHECKPOINT_GO_0000075	26538	8266	47	14.639460	33	2.254181	5.2266704628048197	4.191803e−08	1.383295e−04
43	cis_POS	KRAS.600.LUNG.BREAST_UP.V1_DN	26538	1529	281	16.189954	41	2.532435	2.8434827343201499	4.239453e−08	3.205026e−05
44	cis_NEG	REACTOME_SIGNALING_BY_RHO_GTPASES	26538	4004	111	16.747456	40	2.388422	3.1923402791333002	4.260514e−08	1.148635e−04
45	cis_NEG	POSITIVE_REGULATION_OF_DEVELOPMENTAL_PROCESS	26538	4004	212	31.986133	63	1.969604	2.4014762484254102	4.294559e−08	1.417205e−04
46	cis_POS	KRAS.DF.V1_DN	26538	1529	190	10.946944	32	2.923190	3.3618498402260002	4.418303e−08	3.340237e−05
47	trans_NEG	REGULATION_OF_APOPTOSIS	26538	8266	332	103.410656	150	1.450527	1.8370839358695701	4.785814e−08	1.579319e−04
48	cis_NEG	PROTEIN_DOMAIN_SPECIFIC_BINDING	26538	4004	72	10.863215	30	2.761613	4.04251113247643	4.850192e−08	7.682704e−05
49	trans_NEG	HOXA9_DN.V1_DN	26538	8266	188	58.557842	94	1.605250	2.22438626405678	5.107513e−08	3.861280e−05
50	trans_POS	CELL_CYCLE_CHECKPOINT_GO_0000075	26538	7352	47	13.020725	31	2.380820	5.0729924027970501	5.235656e−08	1.727766e−04
51	trans_POS	CHROMOSOME	26538	7352	122	33.798478	62	1.834402	2.7109305388852598	5.263015e−08	4.905130e−05
52	cis_NEG	BIOCARTA_TCYTOTOXIC_PATHWAY	26538	4004	13	1.961414	11	5.608199	31.028218199725501	5.284656e−08	4.587081e−05
53	cis_NEG	BIOCARTA_THELPER_PATHWAY	26538	4004	13	1.961414	11	5.608199	31.028218199725501	5.284656e−08	4.587081e−05
54	cis_NEG	HALLMARK_APOPTOSIS	26538	4004	159	23.989600	51	2.125921	2.6787350309022302	5.558961e−08	1.111792e−05
55	trans_POS	BIOCARTA_TCYTOTOXIC_PATHWAY	26538	7352	13	3.601477	13	3.609630	Inf	5.618902e−08	4.877207e−05
56	trans_POS	BIOCARTA_THELPER_PATHWAY	26538	7352	13	3.601477	13	3.609630	Inf	5.618902e−08	4.877207e−05
57	cis_NEG	INTERCELLULAR_JUNCTION	26538	4004	65	9.807069	28	2.855083	4.28175548824108	5.700302e−08	5.312682e−05
58	cis_NEG	BIOCARTA_TCRA_PATHWAY	26538	4004	11	1.659658	10	6.025338	56.413480675575798	5.748824e−08	4.989979e−05
59	trans_NEG	KEGG_ALLOGRAFT_REJECTION	26538	8266	32	9.967292	25	2.508204	7.9148331992689904	5.955541e−08	4.430922e−05
60	trans_NEG	REACTOME_E2F_MEDIATED_REGULATION_OF_DNA_REPLICATION	26538	8266	32	9.967292	25	2.508204	7.9148331992689904	5.955541e−08	1.605614e−04
61	trans_NEG	INTRACELLULAR_ORGANELLE_PART	26538	8266	1172	365.052076	449	1.229962	1.39419382209593	5.975326e−08	5.569004e−05
62	trans_POS	MITOSIS	26538	7352	82	22.717010	46	2.024914	3.3489628435036001	5.976665e−08	1.972299e−04
63	trans_NEG	REGULATION_OF_PROGRAMMED_CELL_DEATH	26538	8266	333	103.722134	150	1.446172	1.82694725585937	6.049123e−08	1.996211e−04
64	cis_NEG	HALLMARK_TNFA_SIGNALING_VIA_NFKB	26538	4004	200	30.175597	60	1.988362	2.4333100253283901	6.125372e−08	1.225074e−05
65	cis_NEG	NUCLEUS	26538	4004	1404	211.832693	284	1.340681	1.4596458029911099	6.140436e−08	5.722886e−05
66	cis_POS	ION_TRANSPORT	26538	1529	183	10.543636	31	2.940162	3.3839053867347801	6.227725e−08	2.055149e−04
67	cis_NEG	ESTABLISHMENT_OF_LOCALIZATION	26538	4004	857	129.302434	187	1.446222	1.59868794534171	6.244191e−08	2.060583e−04
68	cis_NEG	REACTOME_TCR_SIGNALING	26538	4004	51	7.694777	24	3.118999	5.0263616519903698	6.377114e−08	1.719270e−04
69	trans_POS	KEGG_CYTOKINE_CYTOKINE_RECEPTOR_INTERACTION	26538	7352	260	72.029543	112	1.554918	1.9899597406267999	6.661485e−08	4.956145e−05
70	trans_NEG	KEGG_FOCAL_ADHESION	26538	8266	199	61.984098	98	1.581051	2.1585529461725601	7.009534e−08	5.215093e−05
71	cis_NEG	KEGG_ADHERENS_JUNCTION	26538	4004	73	11.014093	30	2.723783	3.94830271860075	7.048733e−08	5.244257e−05
72	cis_NEG	KEGG_ENDOCYTOSIS	26538	4004	178	26.856282	55	2.047938	2.53752032326685	7.154149e−08	5.322687e−05
73	cis_NEG	NEGATIVE_REGULATION_OF_APOPTOSIS	26538	4004	147	22.179064	48	2.164203	2.7497007700132499	7.286789e−08	2.404640e−04
74	trans_NEG	NEGATIVE_REGULATION_OF_BIOLOGICAL_PROCESS	26538	8266	665	207.132791	271	1.308339	1.5380401558082699	7.305701e−08	2.410881e−04
75	cis_POS	LTE2_UP.V1_DN	26538	1529	194	11.177406	32	2.862918	3.2783273330761298	7.308303e−08	5.525077e−05
76	trans_POS	POSITIVE_REGULATION_OF_CELL_PROLIFERATION	26538	7352	144	39.893285	70	1.754681	2.4825974938216899	7.551126e−08	2.491872e−04
77	trans_POS	REGULATION_OF_CELL_PROLIFERATION	26538	7352	301	83.388047	126	1.511008	1.8943166233676201	7.708773e−08	2.543895e−04
78	trans_POS	AKT_UP_MTOR_DN.V1_DN	26538	7352	182	50.420680	84	1.665983	2.25107302480406	7.830846e−08	5.920120e−05
79	cis_NEG	E2F3_UP.V1_UP	26538	4004	183	27.610671	56	2.028201	2.5024781770493698	7.901123e−08	5.973249e−05
80	cis_NEG	BMI1_DN_MEL18_DN.V1_UP	26538	4004	143	21.575552	47	2.178392	2.77615845765731	7.931839e−08	5.996470e−05
81	trans_NEG	MITOSIS	26538	8266	82	25.541186	49	1.918470	3.2957021839988401	8.185899e−08	2.701347e−04
82	trans_POS	REACTOME_TELOMERE_MAINTENANCE	26538	7352	73	20.223679	42	2.076773	3.5502295566628499	8.412059e−08	2.267891e−04
83	cis_POS	CELL_CELL_SIGNALING	26538	1529	399	22.988582	51	2.218493	2.44514640159275	8.559634e−08	2.824679e−04
84	trans_NEG	PROTEINACEOUS_EXTRACELLULAR_MATRIX	26538	8266	98	30.524832	56	1.834572	2.9604824118276301	8.844107e−08	8.242708e−05
85	cis_NEG	REACTOME_SIGNALING_BY_THE_B_CELL_RECEPTOR_BCR	26538	4004	122	18.407114	42	2.281726	2.9751834737088698	8.865646e−08	2.390178e−04
86	cis_POS	ACTIVE_TRANSMEMBRANE_TRANSPORTER_ACTIVITY	26538	1529	121	6.971475	24	3.442600	4.09533774307031	9.093831e−08	1.440463e−04
87	cis_NEG	NEGATIVE_REGULATION_OF_PROGRAMMED_CELL_DEATH	26538	4004	148	22.329942	48	2.149580	2.7216863351043701	9.217457e−08	3.041761e−04
88	cis_NEG	HALLMARK_COAGULATION	26538	4004	135	20.368528	45	2.209291	2.8344011488524101	9.309810e−08	1.861962e−05
89	trans_NEG	BIOCARTA_NO2IL12_PATHWAY	26538	8266	17	5.295124	16	3.021648	35.430842831952901	9.343373e−08	8.110048e−05
90	trans_NEG	REACTOME_G1_S_SPECIFIC_TRANSCRIPTION	26538	8266	17	5.295124	16	3.021648	35.430842831952901	9.343373e−08	2.518973e−04
91	trans_POS	KEGG_PRIMARY_IMMUNODEFICIENCY	26538	7352	35	9.696285	25	2.578307	6.5415425132472498	9.480019e−08	7.053134e−05
92	cis_NEG	RESPONSE_TO_WOUNDING	26538	4004	184	27.761549	56	2.017178	2.4829504730883301	9.703944e−08	3.202301e−04
93	cis_NEG	AKT_UP.V1_UP	26538	4004	166	25.045746	52	2.076201	2.5875632874137202	9.820600e−08	7.424374e−05
94	cis_POS	CAHOY_OLIGODENDROCUTIC	26538	1529	96	5.531087	21	3.796722	4.6291376229833396	9.933631e−08	7.509825e−05
95	trans_NEG	KEGG_CELL_CYCLE	26538	8266	124	38.623257	67	1.734706	2.61127134019257	1.001509e−07	7.451225e−05
96	trans_POS	CAHOY_ASTROGLIAL	26538	7352	98	27.149597	52	1.915314	2.9637651158912801	1.011543e−07	7.647264e−05
97	trans_NEG	MULTICELLULAR_ORGANISMAL_DEVELOPMENT	26538	8266	1029	320.510739	398	1.241768	1.4142095441129101	1.029340e−07	3.396821e−04
98	cis_POS	VOLTAGE_GATED_CATION_CHANNEL_ACTIVITY	26538	1529	65	3.745007	17	4.539377	5.8459668408272902	1.034092e−07	1.638001e−04
99	trans_NEG	ESC_J1_UP_LATE.V1_DN	26538	8266	183	57.000452	91	1.596479	2.1996472045698199	1.145148e−07	8.657319e−05
100	cis_NEG	MTOR_UP.N4.V1_UP	26538	4004	194	29.270329	58	1.981529	2.4205944610792298	1.149950e−07	8.693621e−05

	set1_Name	set2_Name	Background	set1_size	set2_size	expected.overlap	actual.overlap	enrichment.foldchange	odds.ratio	FET_pvalue	corrected.FET.pvalue
1	cis_NEG	REGULATION_OF_I_KAPPAB_KINASE_NF_KAPPAB_CASCADE	26538	4004	90	13.579019	34	2.503863	3.4374022285321502	1.152824e−07	3.804319e−04
2	cis_POS	ERB2_UP.V1_UP	26538	1529	188	10.831713	31	2.861967	3.27549381525023	1.171583e−07	8.857166e−05
3	cis_NEG	PTEN_DN.V1_DN	26538	4004	176	26.554526	54	2.033552	2.5112882114858301	1.213071e−07	9.170820e−05
4	trans_NEG	STK33_SKM_DN	26538	8266	265	82.541638	123	1.490157	1.9285758256204899	1.231030e−07	9.306585e−05
5	trans_POS	HALLMARK_IL6_JAK_STAT3_SIGNALING	26538	7352	86	23.825156	47	1.972705	3.1585223672731799	1.268677e−07	2.537354e−05
6	cis_NEG	KRAS.PROSTATE_UP.V1_UP	26538	4004	132	19.915894	44	2.209291	2.8339379239291498	1.286632e−07	9.726940e−05
7	cis_NEG	JNK_DN.V1_DN	26538	4004	181	27.308916	55	2.013994	2.4769622399312099	1.332537e−07	1.007398e−04
8	cis_NEG	REACTOME_PD1_SIGNALING	26538	4004	16	2.414048	12	4.970904	16.929360365289501	1.374264e−07	3.705017e−04
9	trans_NEG	EXTRACELLULAR_MATRIX	26538	8266	99	30.836310	56	1.816041	2.8914789936368699	1.420019e−07	1.323457e−04
10	cis_POS	METAL_ION_TRANSPORT	26538	1529	115	6.625782	23	3.471288	4.1360409709108499	1.429747e−07	4.718164e−04
11	cis_POS	KEGG_ARRHYTHMOGENIC_RIGHT_VENTRICULAR_CARDIOMYOPATHY_ARVC	26538	1529	74	4.263547	18	4.221837	5.3071098095398996	1.464671e−07	1.089715e−04
12	cis_NEG	HALLMARK_HYPOXIA	26538	4004	200	30.175597	59	1.955222	2.37520987614245	1.503000e−07	3.006000e−05
13	trans_NEG	STK33_DN	26538	8266	271	84.410506	125	1.480858	1.9062917621734099	1.505127e−07	1.137876e−04
14	cis_POS	KEGG_DILATED_CARDIOMYOPATHY	26538	1529	90	5.185395	20	3.856987	4.7214123338957004	1.513080e−07	1.125732e−04
15	trans_NEG	NEGATIVE_REGULATION_OF_CELLULAR_PROCESS	26538	8266	636	198.099932	259	1.307421	1.53537514004047	1.513782e−07	4.995482e−04
16	trans_NEG	HALLMARK_MTORC1_SIGNALING	26538	8266	199	61.984098	97	1.564917	2.1152140457766202	1.522872e−07	3.045744e−05
17	trans_NEG	MEK_UP.V1_UP	26538	8266	194	60.426709	95	1.572152	2.1342030717335301	1.546975e−07	1.169513e−04
18	cis_NEG	NOTCH_DN.V1_DN	26538	4004	182	27.459794	55	2.002928	2.4570968754937499	1.631677e−07	1.233548e−04
19	trans_POS	REACTOME_DEPOSITION_OF_NEW_CENPA_CONTAINING_NUCLEOSOMES_AT_THE_CENTROMERE	26538	7352	60	16.622202	36	2.165778	3.92853216283233	1.633676e−07	4.404389e−04
20	cis_POS	PTEN_DN.V1_UP	26538	1529	181	10.428405	30	2.876758	3.2943808303697302	1.659010e−07	1.254212e−04
21	trans_POS	NON_MEMBRANE_BOUND_ORGANELLE	26538	7352	623	172.593865	231	1.338402	1.5552355765214601	1.663776e−07	1.550639e−04
22	trans_POS	INTRACELLULAR_NON_MEMBRANE_BOUND_ORGANELLE	26538	7352	623	172.593865	231	1.338402	1.5552355765214601	1.663776e−07	1.550639e−04
23	trans_NEG	E2F1_UP.V1_UP	26538	8266	187	58.246364	92	1.579498	2.1535101259551199	1.837883e−07	1.389440e−04
24	trans_NEG	ESC_J1_UP_LATE.V1_UP	26538	8266	187	58.246364	92	1.579498	2.1535101259551199	1.837883e−07	1.389440e−04
25	cis_NEG	RESPONSE_TO_STRESS	26538	4004	496	74.835481	118	1.576792	1.7797820689023001	1.854154e−07	6.118709e−04
26	cis_POS	KEGG_HYPERTROPHIC_CARDIOMYOPATHY_HCM	26538	1529	83	4.782086	19	3.973161	4.90372849304695	1.872353e−07	1.393031e−04
27	trans_NEG	REACTOME_TCR_SIGNALING	26538	8266	51	15.885372	34	2.140334	4.4348232512881696	1.920104e−07	5.176600e−04
28	cis_NEG	STK33_SKM_UP	26538	4004	268	40.435300	73	1.805353	2.1273390566012398	1.938673e−07	1.465637e−04
29	cis_NEG	HALLMARK_CHOLESTEROL_HOMEOSTASIS	26538	4004	72	10.863215	29	2.669560	3.81571153558528	1.948833e−07	3.897665e−05
30	cis_POS	CATION_CHANNEL_ACTIVITY	26538	1529	117	6.741013	23	3.411950	4.0478048663715498	1.986495e−07	3.146608e−04
31	trans_NEG	CYTOSKELETON_ORGANIZATION_AND_BIOGENESIS	26538	8266	205	63.852966	99	1.550437	2.0774210983880899	2.008857e−07	6.629229e−04
32	cis_NEG	HEMOPOIETIC_OR_LYMPHOID_ORGAN_DEVELOPMENT	26538	4004	76	11.466727	30	2.616265	3.6902806221951598	2.048311e−07	6.759427e−04
33	cis_POS	KRAS.600_UP.V1_DN	26538	1529	276	15.901877	39	2.452541	2.73581642623828	2.119116e−07	1.602052e−04
34	trans_NEG	REACTOME_G_ALPHA_I_SIGNALLING_EVENTS	26538	8266	190	59.180797	93	1.571456	2.1320635447199501	2.132645e−07	5.749611e−04
35	trans_NEG	REGULATION_OF_CELL_CYCLE	26538	8266	180	56.066019	89	1.587414	2.1745363936947202	2.174586e−07	7.176132e−04
36	trans_POS	STK33_SKM_DN	26538	7352	265	73.414726	112	1.525579	1.92444621893663	2.201833e−07	1.664586e−04
37	cis_NEG	KRAS.KIDNEY_UP.V1_UP	26538	4004	143	21.575552	46	2.132043	2.68801284808448	2.215617e−07	1.675006e−04
38	trans_POS	TRANSMEMBRANE_RECEPTOR_ACTIVITY	26538	7352	410	113.585048	161	1.417440	1.70270216281622	2.218384e−07	3.513920e−04
39	cis_POS	CSR_LATE_UP.V1_DN	26538	1529	164	9.448941	28	2.963295	3.4113760021277999	2.253135e−07	1.703370e−04
40	trans_NEG	IDENTICAL_PROTEIN_BINDING	26538	8266	299	93.131886	135	1.449557	1.83328237849871	2.289239e−07	3.626155e−04
41	trans_NEG	REGULATION_OF_IMMUNE_SYSTEM_PROCESS	26538	8266	64	19.934584	40	2.006563	3.6970067082743698	2.297673e−07	7.582322e−04
42	trans_POS	STK33_DN	26538	7352	271	75.076946	114	1.518442	1.9090373318642599	2.310782e−07	1.746951e−04
43	cis_NEG	KRAS.600_UP.V1_UP	26538	4004	274	41.340568	74	1.790009	2.1026405322396799	2.317584e−07	1.752093e−04
44	trans_POS	SYSTEM_DEVELOPMENT	26538	7352	843	233.541940	300	1.284566	1.46055586158844	2.337754e−07	7.714588e−04
45	cis_POS	LEF1_UP.V1_DN	26538	1529	184	10.601251	30	2.829855	3.22982121262929	2.400634e−07	1.814879e−04
46	cis_POS	MEK_UP.V1_UP	26538	1529	194	11.177406	31	2.773452	3.1541742152449102	2.416832e−07	1.827125e−04
47	trans_POS	REACTOME_SYNTHESIS_OF_DNA	26538	7352	90	24.933303	48	1.925136	2.9953173505639299	2.524766e−07	6.806769e−04
48	trans_NEG	BIOCARTA_TCYTOTOXIC_PATHWAY	26538	8266	13	4.049212	13	3.210501	Inf	2.580657e−07	2.240010e−04
49	trans_NEG	BIOCARTA_THELPER_PATHWAY	26538	8266	13	4.049212	13	3.210501	Inf	2.580657e−07	2.240010e−04
50	cis_POS	NEUROGENESIS	26538	1529	93	5.358241	20	3.732568	4.5268701514395104	2.674880e−07	8.827103e−04
51	trans_POS	REGULATION_OF_T_CELL_ACTIVATION	26538	7352	28	7.757028	21	2.707223	7.8473044313891602	2.683282e−07	8.854829e−04
52	trans_NEG	REACTOME_PD1_SIGNALING	26538	8266	16	4.983646	15	3.009845	33.212444025407102	2.831246e−07	7.633038e−04
53	cis_NEG	REACTOME_CELL_JUNCTION_ORGANIZATION	26538	4004	77	11.617605	30	2.582288	3.6115993497796999	2.873158e−07	7.746033e−04
54	cis_POS	PTEN_DN.V1_DN	26538	1529	176	10.140327	29	2.859868	3.2695706020772599	3.012514e−07	2.277461e−04
55	cis_NEG	APICAL_JUNCTION_COMPLEX	26538	4004	34	5.129852	18	3.508873	6.3547393348463297	3.026662e−07	2.820849e−04
56	cis_NEG	APICOLATERAL_PLASMA_MEMBRANE	26538	4004	34	5.129852	18	3.508873	6.3547393348463297	3.026662e−07	2.820849e−04
57	cis_POS	KEGG_CHEMOKINE_SIGNALING_PATHWAY	26538	1529	186	10.716482	30	2.799426	3.1881610252252899	3.055098e−07	2.272993e−04
58	cis_NEG	AKT_UP_MTOR_DN.V1_UP	26538	4004	176	26.554526	53	1.995893	2.4439971841186101	3.071591e−07	2.322123e−04
59	trans_POS	REACTOME_G1_S_SPECIFIC_TRANSCRIPTION	26538	7352	17	4.709624	15	3.184968	19.610095949806801	3.210358e−07	8.655126e−04
60	cis_NEG	PROTEIN_TYROSINE_KINASE_ACTIVITY	26538	4004	62	9.354435	26	2.779430	4.0844171589580798	3.211965e−07	5.087753e−04
61	cis_POS	CELL_SURFACE_RECEPTOR_LINKED_SIGNAL_TRANSDUCTION_GO_0007166	26538	1529	635	36.585839	69	1.885976	2.04088617959663	3.235920e−07	1.067854e−03
62	trans_NEG	CYTOSKELETON	26538	8266	362	112.754993	158	1.401268	1.72590436758708	3.244550e−07	3.023920e−04
63	trans_NEG	REACTOME_CYTOKINE_SIGNALING_IN_IMMUNE_SYSTEM	26538	8266	261	81.295727	120	1.476092	1.8942978937189601	3.260770e−07	8.791036e−04
64	cis_NEG	ALK_DN.V1_DN	26538	4004	136	20.519406	44	2.144312	2.7100759280337101	3.354694e−07	2.536148e−04
65	cis_NEG	BIOCARTA_TCAPOPTOSIS_PATHWAY	26538	4004	10	1.508780	9	5.965085	50.764156761173098	3.475874e−07	3.017059e−04
66	trans_POS	KEGG_INTESTINAL_IMMUNE_NETWORK_FOR_IGA_PRODUCTION	26538	7352	43	11.912578	28	2.350457	4.8857919402851202	3.479758e−07	2.588940e−04
67	cis_NEG	IL2_UP.V1_DN	26538	4004	186	28.063305	55	1.959855	2.38155579803631	3.584227e−07	2.709675e−04
68	trans_NEG	REACTOME_CLASS_A1_RHODOPSIN_LIKE_RECEPTORS	26538	8266	293	91.263019	132	1.446369	1.8253720953840999	3.603809e−07	9.715869e−04
69	trans_POS	REGULATION_OF_MULTICELLULAR_ORGANISMAL_PROCESS	26538	7352	146	40.447359	69	1.705921	2.35111363443764	3.634149e−07	1.199269e−03
70	trans_NEG	PTEN_DN.V2_UP	26538	8266	137	42.672470	71	1.663836	2.3898342123114502	3.686320e−07	2.786858e−04
71	cis_NEG	HALLMARK_ANDROGEN_RESPONSE	26538	4004	98	14.786043	35	2.367097	3.14515860267417	3.737046e−07	7.474093e−05
72	cis_NEG	REGULATION_OF_TRANSCRIPTION_FROM_RNA_POLYMERASE_II_PROMOTER	26538	4004	287	43.301982	76	1.755116	2.0469455155502101	3.767188e−07	1.243172e−03
73	cis_NEG	HEMOPOIESIS	26538	4004	74	11.164971	29	2.597409	3.64578603860652	3.898978e−07	1.286663e−03
74	trans_POS	BIOPOLYMER_METABOLIC_PROCESS	26538	7352	1660	459.880925	549	1.193787	1.3129170011020099	3.913881e−07	1.291581e−03
75	trans_POS	HALLMARK_COMPLEMENT	26538	7352	196	54.299194	87	1.602234	2.0958755989437301	3.941878e−07	7.883755e−05
76	trans_NEG	JNK_DN.V1_UP	26538	8266	187	58.246364	91	1.562329	2.1075495574126499	3.985332e−07	3.012911e−04
77	cis_NEG	BIOCARTA_NO2IL12_PATHWAY	26538	4004	17	2.564926	12	4.678498	13.541932217516299	4.025886e−07	3.494469e−04
78	trans_POS	HALLMARK_MTORC1_SIGNALING	26538	7352	199	55.130304	88	1.596218	2.08184083552359	4.144036e−07	8.288072e−05
79	trans_NEG	KEGG_VIRAL_MYOCARDITIS	26538	8266	65	20.246062	40	1.975693	3.5489070319753799	4.177460e−07	3.108030e−04
80	cis_POS	HALLMARK_ESTROGEN_RESPONSE_LATE	26538	1529	199	11.465483	31	2.703767	3.0596943758442201	4.301933e−07	8.603866e−05
81	cis_NEG	MTOR_UP.V1_UP	26538	4004	164	24.743990	50	2.020693	2.4869235336417401	4.330314e−07	3.273717e−04
82	trans_NEG	REACTOME_CELL_CYCLE_CHECKPOINTS	26538	8266	111	34.574045	60	1.735406	2.6121946207375499	4.480757e−07	1.208012e−03
83	trans_NEG	REACTOME_SYNTHESIS_OF_DNA	26538	8266	90	28.033009	51	1.819284	2.90226343192036	4.684160e−07	1.262850e−03
84	cis_POS	IL21_UP.V1_DN	26538	1529	180	10.370789	29	2.796316	3.1824562772724598	4.897465e−07	3.702483e−04
85	trans_POS	GCNP_SHH_UP_LATE.V1_UP	26538	7352	180	49.866606	81	1.624334	2.1477751803583698	4.909569e−07	3.711634e−04
86	cis_NEG	HALLMARK_XENOBIOTIC_METABOLISM	26538	4004	197	29.722963	57	1.917709	2.3099022914525	4.911277e−07	9.822553e−05
87	trans_NEG	SYSTEM_DEVELOPMENT	26538	8266	843	262.575853	329	1.252971	1.4320897601756699	4.984372e−07	1.644843e−03
88	trans_POS	IMMUNE_SYSTEM_DEVELOPMENT	26538	7352	79	21.885899	43	1.964735	3.1292354778560099	4.994407e−07	1.648154e−03
89	trans_NEG	ANATOMICAL_STRUCTURE_DEVELOPMENT	26538	8266	993	309.297536	381	1.231824	1.3943290354266	5.047081e−07	1.665537e−03
90	trans_POS	ESC_V6.5_UP_EARLY.V1_DN	26538	7352	169	46.819203	77	1.644624	2.19664542571812	5.070755e−07	3.833491e−04
91	trans_NEG	RECEPTOR_SIGNALING_PROTEIN_ACTIVITY	26538	8266	81	25.229708	47	1.862883	3.0673028286391801	5.083171e−07	8.051743e−04
92	trans_NEG	REACTOME_AXON_GUIDANCE	26538	8266	242	75.377647	112	1.485852	1.91688685526226	5.267643e−07	1.420157e−03
93	trans_NEG	PROTEIN_METABOLIC_PROCESS	26538	8266	1206	375.642324	454	1.208597	1.35399211828122	5.303467e−07	1.750144e−03
94	cis_NEG	IMMUNE_SYSTEM_DEVELOPMENT	26538	4004	79	11.919361	30	2.516913	3.4638769700766501	5.518767e−07	1.821193e−03
95	trans_NEG	CHROMOSOMAL_PART	26538	8266	95	29.590399	53	1.791122	2.8008704141315501	5.524754e−07	5.149071e−04
96	cis_NEG	PROTEIN_DIMERIZATION_ACTIVITY	26538	4004	179	27.007160	53	1.962443	2.3853746580847401	5.536100e−07	8.769182e−04
97	trans_NEG	REACTOME_COSTIMULATION_BY_THE_CD28_FAMILY	26538	8266	59	18.377195	37	2.013365	3.7296528801754398	5.739506e−07	1.547371e−03
98	trans_POS	BIOCARTA_CTL_PATHWAY	26538	7352	14	3.878514	13	3.351799	33.979694407282302	5.845417e−07	5.073822e−04
99	trans_POS	POSITIVE_REGULATION_OF_T_CELL_ACTIVATION	26538	7352	21	5.817771	17	2.922081	11.1130595640845	5.881272e−07	1.940820e−03
100	cis_POS	AKT_UP_MTOR_DN.V1_DN	26538	1529	182	10.486020	29	2.765587	3.1406073163889299	6.204666e−07	4.690727e−04

	set1_Name	set2_Name	Background	set1_size	set2_size	expected.overlap	actual.overlap	enrichment.foldchange	odds.ratio	FET_pvalue	corrected.FET.pvalue
1	cis_NEG	TGFB_UP.V1_DN	26538	4004	189	28.515939	55	1.928746	2.3280953794711801	6.317161e−07	0.0004775774
2	trans_POS	KEGG_GRAFT_VERSUS_HOST_DISEASE	26538	7352	33	9.142211	23	2.515803	6.0163005955098496	6.338180e−07	0.0004715606
3	cis_NEG	ANTI_APOPTOSIS	26538	4004	117	17.652724	39	2.209291	2.83162530420651	6.502590e−07	0.0021458548
4	trans_POS	CELL_DEVELOPMENT	26538	7352	567	157.079810	210	1.336900	1.55078136478997	6.531278e−07	0.0021553219
5	trans_NEG	KEGG_LEISHMANIA_INFECTION	26538	8266	68	21.180496	41	1.935743	3.3685378548935399	6.573543e−07	0.0004890716
6	cis_NEG	REACTOME_ANTIGEN_ACTIVATES_B_CELL_RECEPTOR_LEADING_TO_GENERATION_OF_SECOND_MESSENGERS	26538	4004	29	4.375462	16	3.656757	6.9495723182187801	6.611076e−07	0.0017823460
7	trans_POS	POSITIVE_REGULATION_OF_LYMPHOCYTE_ACTIVATION	26538	7352	23	6.371844	18	2.824928	9.4140819798165207	6.728614e−07	0.0022204425
8	trans_NEG	REACTOME_GPCR_LIGAND_BINDING	26538	8266	393	122.410807	168	1.372428	1.6639736662853699	7.062099e−07	0.0019039419
9	cis_NEG	MUSCLE_DEVELOPMENT	26538	4004	92	13.880775	33	2.377389	3.1654491540904202	7.104719e−07	0.0023445571
10	trans_POS	REACTOME_EXTENSION_OF_TELOMERES	26538	7352	27	7.479991	20	2.673800	7.4725087271875701	7.322283e−07	0.0019740874
11	trans_POS	BIOCARTA_TCRA_PATHWAY	26538	7352	11	3.047404	11	3.609630	Inf	7.337703e−07	0.0006369126
12	trans_POS	REACTOME_UNWINDING_OF_DNA	26538	7352	11	3.047404	11	3.609630	Inf	7.337703e−07	0.0019782446
13	cis_NEG	KEGG_SMALL_CELL_LUNG_CANCER	26538	4004	84	12.673751	31	2.446000	3.3094482550576698	7.485910e−07	0.0005569517
14	cis_NEG	REGULATION_OF_MOLECULAR_FUNCTION	26538	4004	317	47.828322	81	1.693557	1.9508671552734	7.756131e−07	0.0025595231
15	cis_NEG	PROTEIN_KINASE_ACTIVITY	26538	4004	282	42.547592	74	1.739229	2.02108096278433	7.760946e−07	0.0012293339
16	cis_NEG	CELL_FRACTION	26538	4004	488	73.628457	114	1.548314	1.7363680880930601	7.845678e−07	0.0007312172
17	cis_NEG	LEADING_EDGE	26538	4004	46	6.940387	21	3.025768	4.7468387465420303	7.956222e−07	0.0007415199
18	cis_NEG	RNA_POLYMERASE_II_TRANSCRIPTION_FACTOR_ACTIVITY	26538	4004	181	27.308916	53	1.940758	2.3480370361641598	8.104261e−07	0.0012837149
19	cis_POS	CATION_TRANSPORT	26538	1529	145	8.354247	25	2.992490	3.4473160153455198	8.147790e−07	0.0026887709
20	cis_POS	ION_CHANNEL_ACTIVITY	26538	1529	145	8.354247	25	2.992490	3.4473160153455198	8.147790e−07	0.0012906100
21	trans_NEG	REACTOME_TRANSLOCATION_OF_ZAP_70_TO_IMMUNOLOGICAL_SYNAPSE	26538	8266	12	3.737735	12	3.210501	Inf	8.293494e−07	0.0022359260
22	trans_NEG	CAHOY_OLIGODENDROCUTIC	26538	8266	96	29.901877	53	1.772464	2.7355880087033699	8.608631e−07	0.0006508125
23	cis_NEG	TRANSCRIPTION_FACTOR_BINDING	26538	4004	303	45.716030	78	1.706185	1.9699076847771699	9.003448e−07	0.0014261462
24	trans_NEG	CYTOKINE_BINDING	26538	8266	47	14.639460	31	2.117564	4.2949073798694499	9.511334e−07	0.0015065953
25	cis_NEG	REACTOME_METABOLISM_OF_VITAMINS_AND_COFACTORS	26538	4004	50	7.543899	22	2.916264	4.4406960267353899	9.545302e−07	0.0025734135
26	cis_NEG	REACTOME_SEMAPHORIN_INTERACTIONS	26538	4004	65	9.807069	26	2.651149	3.7696730387403101	9.658156e−07	0.0026038387
27	cis_NEG	POSITIVE_REGULATION_OF_SIGNAL_TRANSDUCTION	26538	4004	123	18.557992	40	2.155406	2.7291562352150698	9.689749e−07	0.0031976171
28	trans_POS	REACTOME_PD1_SIGNALING	26538	7352	16	4.432587	14	3.158426	18.300926164763901	1.027080e−06	0.0027690069
29	trans_POS	REGULATION_OF_DEVELOPMENTAL_PROCESS	26538	7352	431	119.402819	165	1.381877	1.63292739348218	1.029322e−06	0.0033967640
30	cis_NEG	JNK_DN.V1_UP	26538	4004	187	28.214183	54	1.913931	2.3024742280019099	1.035740e−06	0.0007830196
31	trans_NEG	ORGAN_DEVELOPMENT	26538	8266	567	176.607958	230	1.302320	1.5231897904690701	1.038840e−06	0.0034281707
32	cis_POS	GATED_CHANNEL_ACTIVITY	26538	1529	119	6.856244	22	3.208754	3.7487112401672902	1.091232e−06	0.0017285120
33	cis_POS	JNK_DN.V1_UP	26538	1529	187	10.774098	29	2.691641	3.0406201580990202	1.101151e−06	0.0008324700
34	trans_POS	ESC_J1_UP_LATE.V1_DN	26538	7352	183	50.697716	81	1.597705	2.0842946533542799	1.118317e−06	0.0008454475
35	trans_NEG	KEGG_TYPE_I_DIABETES_MELLITUS	26538	8266	37	11.524682	26	2.256028	5.2374827730446496	1.138593e−06	0.0008471135
36	trans_NEG	BASEMENT_MEMBRANE	26538	8266	37	11.524682	26	2.256028	5.2374827730446496	1.138593e−06	0.0010611690
37	cis_NEG	BMI1_DN.V1_DN	26538	4004	137	20.670284	43	2.080281	2.5913862929638198	1.141558e−06	0.0008630175
38	trans_NEG	IL2_UP.V1_UP	26538	8266	183	57.000452	88	1.543847	2.0588919314483101	1.165279e−06	0.0008809507
39	trans_NEG	REACTOME_SIGNALING_BY_RHO_GTPASES	26538	8266	111	34.574045	59	1.706482	2.5188239549236902	1.172340e−06	0.0031606286
40	cis_NEG	KEGG_AUTOIMMUNE_THYROID_DISEASE	26538	4004	47	7.091265	21	2.961390	4.5641260747185903	1.233961e−06	0.0009180673
41	cis_NEG	HALLMARK_BILE_ACID_METABOLISM	26538	4004	111	16.747456	37	2.209291	2.8307018886361099	1.244912e−06	0.0002489824
42	cis_NEG	CRX_DN.V1_UP	26538	4004	133	20.066772	42	2.093012	2.6142376732592001	1.264165e−06	0.0009557084
43	trans_POS	MTOR_UP.V1_UP	26538	7352	164	45.434019	74	1.628736	2.1573200428966199	1.319779e−06	0.0009977533
44	cis_POS	HALLMARK_KRAS_SIGNALING_DN	26538	1529	199	11.465483	30	2.616549	2.94140483839635	1.330474e−06	0.0002660947
45	trans_POS	MEL18_DN.V1_DN	26538	7352	142	39.339212	66	1.677715	2.2776752822872299	1.354111e−06	0.0010237081
46	cis_NEG	ACTIN_BINDING	26538	4004	74	11.164971	28	2.507844	3.4425235974078401	1.418333e−06	0.0022466398
47	trans_POS	RB_DN.V1_UP	26538	7352	134	37.122918	63	1.697065	2.3268955322194	1.433263e−06	0.0010835468
48	trans_NEG	KEGG_ANTIGEN_PROCESSING_AND_PRESENTATION	26538	8266	74	23.049363	43	1.865561	3.0768373315001498	1.456051e−06	0.0010833019
49	trans_POS	RESPONSE_TO_WOUNDING	26538	7352	184	50.974753	81	1.589022	2.0639566201352499	1.458821e−06	0.0048141105
50	trans_POS	E2F1_UP.V1_DN	26538	7352	184	50.974753	81	1.589022	2.0639566201352499	1.458821e−06	0.0011028689
51	trans_POS	DNA_REPLICATION	26538	7352	102	28.257744	51	1.804815	2.6207675318246602	1.459233e−06	0.0048154681
52	cis_POS	CENTRAL_NERVOUS_SYSTEM_DEVELOPMENT	26538	1529	121	6.971475	22	3.155717	3.6728511449251302	1.462078e−06	0.0048248569
53	trans_NEG	DNA_REPLICATION	26538	8266	102	31.770744	55	1.731152	2.5972891067723398	1.473455e−06	0.0048624012
54	cis_POS	KRAS.BREAST_UP.V1_DN	26538	1529	140	8.066169	24	2.975390	3.421817464628	1.492774e−06	0.0011285370
55	cis_POS	SKELETAL_DEVELOPMENT	26538	1529	103	5.934396	20	3.370183	3.9802308446049701	1.494275e−06	0.0049311067
56	trans_NEG	HOXA9_DN.V1_UP	26538	8266	189	58.869319	90	1.528810	2.0206653856160401	1.501657e−06	0.0011352528
57	trans_NEG	TRANSFERASE_ACTIVITY_TRANSFERRING_PHOSPHORUS_CONTAINING_GROUPS	26538	8266	419	130.509232	176	1.348564	1.6140684627662001	1.506751e−06	0.0023866936
58	trans_NEG	REGULATION_OF_MULTICELLULAR_ORGANISMAL_PROCESS	26538	8266	146	45.475771	73	1.605250	2.2212487134956	1.507592e−06	0.0049750552
59	trans_POS	JNK_DN.V1_UP	26538	7352	187	51.805863	82	1.582832	2.04971017006301	1.526232e−06	0.0011538311
60	cis_NEG	CAHOY_ASTROCYTIC	26538	4004	99	14.936921	34	2.276239	2.9602891011189398	1.537429e−06	0.0011622961
61	trans_POS	HALLMARK_APOPTOSIS	26538	7352	159	44.048836	72	1.634549	2.17113504723687	1.549373e−06	0.0003098746
62	trans_POS	REACTOME_INTEGRIN_CELL_SURFACE_INTERACTIONS	26538	7352	79	21.885899	42	1.919044	2.9734142485639699	1.555089e−06	0.0041925212
63	trans_NEG	PROTEIN_DIMERIZATION_ACTIVITY	26538	8266	179	55.754541	86	1.542475	2.0550943561384001	1.604372e−06	0.0025413253
64	trans_POS	KEGG_ALLOGRAFT_REJECTION	26538	7352	32	8.865174	22	2.481621	5.7549897149627602	1.609890e−06	0.0011977581
65	cis_NEG	REACTOME_CD28_DEPENDENT_VAV1_PATHWAY	26538	4004	11	1.659658	9	5.422804	25.372569846831102	1.653034e−06	0.0044565791
66	trans_NEG	ERB2_UP.V1_DN	26538	8266	192	59.803753	91	1.521644	2.0026884864608099	1.694640e−06	0.0012811481
67	trans_NEG	INTERLEUKIN_BINDING	26538	8266	24	7.475469	19	2.541647	8.4161844794729905	1.733314e−06	0.0027455686
68	cis_NEG	KRAS.LUNG.BREAST_UP.V1_DN	26538	4004	139	20.972040	43	2.050349	2.5371986410174299	1.756156e−06	0.0013276542
69	cis_POS	BIOPOLYMER_MODIFICATION	26538	1529	641	36.931532	67	1.814168	1.95082210260795	1.885191e−06	0.0062211292
70	trans_NEG	STK33_UP	26538	8266	279	86.902329	124	1.426889	1.7799787152012601	1.888350e−06	0.0014275924
71	trans_NEG	CHROMOSOME	26538	8266	122	38.000301	63	1.657882	2.37074893522691	1.915601e−06	0.0017853398
72	trans_POS	KEGG_LEUKOCYTE_TRANSENDOTHELIAL_MIGRATION	26538	7352	116	32.136257	56	1.742580	2.4465910456272901	1.916735e−06	0.0014260507
73	cis_NEG	KEGG_NEUROTROPHIN_SIGNALING_PATHWAY	26538	4004	126	19.010626	40	2.104086	2.6337428584686	1.929188e−06	0.0014353155
74	cis_POS	SUBSTRATE_SPECIFIC_CHANNEL_ACTIVITY	26538	1529	152	8.757555	25	2.854678	3.25641012451627	1.998283e−06	0.0031652804
75	cis_NEG	ACTIN_FILAMENT_BASED_PROCESS	26538	4004	113	17.049212	37	2.170188	2.75616453076035	2.023906e−06	0.0066788884
76	cis_NEG	STK33_NOMO_UP	26538	4004	284	42.849348	73	1.703643	1.96469372359848	2.100151e−06	0.0015877142
77	cis_NEG	REACTOME_CD28_CO_STIMULATION	26538	4004	31	4.677218	16	3.420837	6.0225207356274097	2.163493e−06	0.0058327779
78	cis_NEG	TIGHT_JUNCTION	26538	4004	31	4.677218	16	3.420837	6.0225207356274097	2.163493e−06	0.0020163758
79	cis_NEG	MEMBRANE_FRACTION	26538	4004	335	50.544125	83	1.642130	1.87158185264348	2.171490e−06	0.0020238285
80	cis_NEG	TRANSPORT	26538	4004	783	118.137463	166	1.405143	1.53634926728929	2.185929e−06	0.0072135658
81	cis_POS	E2F3_UP.V1_UP	26538	1529	183	10.543636	28	2.655630	2.99096532563577	2.199356e−06	0.0016627132
82	cis_POS	REACTOME_REGULATION_OF_INSULIN_SECRETION	26538	1529	88	5.070164	18	3.550181	4.2437955058091497	2.258887e−06	0.0060899588
83	trans_NEG	REGULATION_OF_T_CELL_ACTIVATION	26538	8266	28	8.721381	21	2.407876	6.6451885864618703	2.294595e−06	0.0075721634
84	cis_POS	EGFR_UP.V1_DN	26538	1529	194	11.177406	29	2.594520	2.9108183827174199	2.360722e−06	0.0017847062
85	cis_NEG	BIOCARTA_CTLA4_PATHWAY	26538	4004	19	2.866682	12	4.186025	9.6735501357358196	2.434866e−06	0.0021134633
86	cis_NEG	B_CELL_ACTIVATION	26538	4004	19	2.866682	12	4.186025	9.6735501357358196	2.434866e−06	0.0080350562
87	trans_NEG	KINASE_ACTIVITY	26538	8266	364	113.377949	155	1.367109	1.65155577949782	2.437628e−06	0.0038612035
88	trans_POS	KEGG_CHEMOKINE_SIGNALING_PATHWAY	26538	7352	186	51.528827	81	1.571936	2.0244432568417898	2.451567e−06	0.0018239659
89	cis_NEG	POSITIVE_REGULATION_OF_I_KAPPAB_KINASE_NF_KAPPAB_CASCADE	26538	4004	84	12.673751	30	2.367097	3.1424596159467901	2.481501e−06	0.0081889537
90	cis_NEG	BIOCARTA_IL2RB_PATHWAY	26538	4004	38	5.733363	18	3.139518	5.0830957749662398	2.508653e−06	0.0021775105
91	cis_NEG	VEGF_A_UP.V1_UP	26538	4004	192	28.968573	54	1.864089	2.2185647060364602	2.514271e−06	0.0019007891
92	trans_NEG	BIOCARTA_CTL_PATHWAY	26538	8266	14	4.360690	13	2.981179	28.777288760079301	2.569089e−06	0.0022299691
93	trans_POS	RESPONSE_TO_DNA_DAMAGE_STIMULUS	26538	7352	158	43.771799	71	1.622049	2.14069388802859	2.569745e−06	0.0084801574
94	trans_NEG	KEGG_DNA_REPLICATION	26538	8266	36	11.213204	25	2.229514	5.0353936083214297	2.594007e−06	0.0019299409
95	trans_NEG	REGULATION_OF_LYMPHOCYTE_ACTIVATION	26538	8266	34	10.590248	24	2.266236	5.3170750158681104	2.609581e−06	0.0086116187
96	cis_POS	CAMP_UP.V1_DN	26538	1529	195	11.235021	29	2.581214	2.8931688560926601	2.622809e−06	0.0019828439
97	trans_NEG	EXTRACELLULAR_REGION_PART	26538	8266	329	102.476223	142	1.385687	1.6903944754634099	2.630418e−06	0.0024515499
98	trans_POS	BIOCARTA_BLYMPHOCYTE_PATHWAY	26538	7352	10	2.770367	10	3.609630	Inf	2.651247e−06	0.0023012826
99	trans_POS	BIOCARTA_TCAPOPTOSIS_PATHWAY	26538	7352	10	2.770367	10	3.609630	Inf	2.651247e−06	0.0023012826
100	cis_NEG	MESODERM_DEVELOPMENT	26538	4004	22	3.319316	13	3.916470	8.1512595258078502	2.664229e−06	0.0087919552

	set1_Name	set2_Name	Background	set1_size	set2_size	expected.overlap	actual.overlap	enrichment.foldchange	odds.ratio	FET_pvalue	corrected.FET.pvalue
1	trans_NEG	BIOCARTA_TCRA_PATHWAY	26538	8266	11	3.426257	11	3.210501	Inf	2.665070e−06	0.0023132807
2	trans_NEG	REACTOME_UNWINDING_OF_DNA	26538	8266	11	3.426257	11	3.210501	Inf	2.665070e−06	0.0071850284
3	trans_NEG	INTERLEUKIN_RECEPTOR_ACTIVITY	26538	8266	19	5.918080	16	2.703580	11.8125672626044	2.668534e−06	0.0042269584
4	cis_POS	PROTEIN_MODIFICATION_PROCESS	26538	1529	623	35.894453	65	1.810865	1.94547260819449	2.843482e−06	0.0093834920
5	trans_NEG	HALLMARK_UV_RESPONSE_DN	26538	8266	143	44.541337	71	1.594025	2.1899895080531602	2.883804e−06	0.0005767608
6	trans_POS	REACTOME_M_G1_TRANSITION	26538	7352	78	21.608863	41	1.897370	2.9022251498530802	3.025068e−06	0.0081555832
7	cis_NEG	REACTOME_METABOLISM_OF_LIPIDS_AND_LIPOPROTEINS	26538	4004	469	70.761776	108	1.526248	1.70259234519109	3.057512e−06	0.0082430527
8	trans_POS	KEGG_P53_SIGNALING_PATHWAY	26538	7352	68	18.838496	37	1.964063	3.1251233469860602	3.104702e−06	0.0023098986
9	trans_POS	REACTOME_TCR_SIGNALING	26538	7352	51	14.128872	30	2.123312	3.7389944791917999	3.110953e−06	0.0083871295
10	cis_POS	REACTOME_G_ALPHA_S_SIGNALLING_EVENTS	26538	1529	117	6.741013	21	3.115259	3.6135522368389501	3.116724e−06	0.0084026877
11	trans_NEG	KEGG_NATURAL_KILLER_CELL_MEDIATED_CYTOTOXICITY	26538	8266	126	39.246213	64	1.630731	2.2917584413409502	3.302442e−06	0.0024570166
12	cis_NEG	REACTOME_PHOSPHORYLATION_OF_CD3_AND_TCR_ZETA_CHAINS	26538	4004	14	2.112292	10	4.734194	14.099808748468201	3.368120e−06	0.0090804525
13	cis_POS	HALLMARK_HEDGEHOG_SIGNALING	26538	1529	36	2.074158	11	5.303357	7.2396719377292502	3.528582e−06	0.0007057164
14	trans_NEG	MTOR_UP.V1_UP	26538	8266	164	51.082372	79	1.546522	2.0646363399458298	3.677644e−06	0.0027802988
15	cis_NEG	KEGG_MAPK_SIGNALING_PATHWAY	26538	4004	263	39.680910	68	1.713670	1.9791818437311099	3.707005e−06	0.0027580120
16	trans_POS	RESPONSE_TO_EXTERNAL_STIMULUS	26538	7352	305	84.496194	121	1.432017	1.7280354912990199	3.715777e−06	0.0122620651
17	trans_POS	HEMOPOIETIC_OR_LYMPHOID_ORGAN_DEVELOPMENT	26538	7352	76	21.054789	40	1.899805	2.90984383451138	3.814010e−06	0.0125862314
18	cis_POS	REACTOME_PLATELET_HOMEOSTASIS	26538	1529	74	4.263547	16	3.752744	4.5487688415669796	3.815893e−06	0.0102876486
19	trans_POS	POSITIVE_REGULATION_OF_IMMUNE_SYSTEM_PROCESS	26538	7352	49	13.574798	29	2.136312	3.7947547353546001	3.821289e−06	0.0126102546
20	cis_POS	SYSTEM_PROCESS	26538	1529	552	31.803753	59	1.855127	1.9958303496082199	3.834480e−06	0.0126537824
21	cis_NEG	REACTOME_APOPTOSIS	26538	4004	143	21.575552	43	1.992997	2.4352856529298101	4.002963e−06	0.0107919890
22	cis_NEG	ORGAN_MORPHOGENESIS	26538	4004	143	21.575552	43	1.992997	2.4352856529298101	4.002963e−06	0.0132097788
23	cis_NEG	CAMP_UP.V1_DN	26538	4004	195	29.421207	54	1.835411	2.1710785331615199	4.177988e−06	0.0031585591
24	trans_POS	BIOCARTA_IL7_PATHWAY	26538	7352	17	4.709624	14	2.972636	12.1974787800778	4.321951e−06	0.0037514536
25	trans_POS	BIOCARTA_NO2IL12_PATHWAY	26538	7352	17	4.709624	14	2.972636	12.1974787800778	4.321951e−06	0.0037514536
26	trans_POS	REACTOME_CELL_SURFACE_INTERACTIONS_AT_THE_VASCULAR_WALL	26538	7352	84	23.271083	43	1.847787	2.7470273511738399	4.346465e−06	0.0117180702
27	cis_POS	REACTOME_TRANSPORT_OF_INORGANIC_CATIONS_ANIONS_AND_AMINO_ACIDS_OLIGOPEPTIDES	26538	1529	92	5.300626	18	3.395826	4.0139705325762396	4.404905e−06	0.0118756233
28	trans_POS	STK33_UP	26538	7352	279	77.293240	112	1.449027	1.76169351712348	4.405997e−06	0.0033309339
29	trans_NEG	ADHERENS_JUNCTION	26538	8266	23	7.163991	18	2.512566	7.9722560349125802	4.439057e−06	0.0041372009
30	cis_POS	KRAS.300_UP.V1_DN	26538	1529	139	8.008554	23	2.871929	3.2770808682467298	4.556529e−06	0.0034447363
31	cis_POS	KRAS.300_UP.V1_UP	26538	1529	139	8.008554	23	2.871929	3.2770808682467298	4.556529e−06	0.0034447363
32	trans_POS	HALLMARK_TNFA_SIGNALING_VIA_NFKB	26538	7352	200	55.407340	85	1.534093	1.93976345519566	4.591084e−06	0.0009182168
33	trans_POS	DNA_BINDING	26538	7352	597	165.390911	215	1.299951	1.4828683399803499	4.650460e−06	0.0073663282
34	trans_POS	REGULATION_OF_CELL_CYCLE	26538	7352	180	49.866606	78	1.564173	2.0062948392734201	4.669008e−06	0.0154077249
35	trans_NEG	REACTOME_IL_3_5_AND_GM_CSF_SIGNALING	26538	8266	43	13.393549	28	2.090559	4.1362040892731402	4.671532e−06	0.0125944505
36	trans_POS	APOPTOSIS_GO	26538	7352	422	116.909488	159	1.360026	1.5904239055789999	4.680814e−06	0.0154466860
37	trans_NEG	HALLMARK_MYC_TARGETS_V2	26538	8266	56	17.442761	34	1.949233	3.4257716530381499	4.701042e−06	0.0009402085
38	trans_POS	REACTOME_COSTIMULATION_BY_THE_CD28_FAMILY	26538	7352	59	16.345165	33	2.018946	3.3224248406956902	4.714865e−06	0.0127112771
39	cis_NEG	KEGG_FC_EPSILON_RI_SIGNALING_PATHWAY	26538	4004	78	11.768483	28	2.379236	3.1665679394280599	4.733532e−06	0.0035217479
40	trans_POS	HEMOPOIESIS	26538	7352	74	20.500716	39	1.902373	2.9179105939408299	4.809088e−06	0.0158699900
41	trans_NEG	CELL_SUBSTRATE_ADHERENS_JUNCTION	26538	8266	16	4.983646	14	2.809188	15.4980904292853	4.855635e−06	0.0045254516
42	cis_NEG	KEGG_ANTIGEN_PROCESSING_AND_PRESENTATION	26538	4004	74	11.164971	27	2.418278	3.2479913921453698	4.882769e−06	0.0036327799
43	cis_NEG	KEGG_ARRHYTHMOGENIC_RIGHT_VENTRICULAR_CARDIOMYOPATHY_ARVC	26538	4004	74	11.164971	27	2.418278	3.2479913921453698	4.882769e−06	0.0036327799
44	trans_NEG	ATF2_UP.V1_DN	26538	8266	183	57.000452	86	1.508760	1.9699355753924199	4.921085e−06	0.0037203406
45	cis_NEG	LYMPHOCYTE_DIFFERENTIATION	26538	4004	26	3.922828	14	3.568854	6.5846383627095202	4.923296e−06	0.0162468760
46	cis_NEG	KEGG_PANCREATIC_CANCER	26538	4004	70	10.561459	26	2.461781	3.34054943777774	4.977505e−06	0.0037032635
47	cis_POS	REACTOME_L1CAM_INTERACTIONS	26538	1529	84	4.839702	17	3.512613	4.1852705482423804	4.994295e−06	0.0134646198
48	trans_POS	B_CELL_ACTIVATION	26538	7352	19	5.263697	15	2.849708	9.8028844664642296	5.031354e−06	0.0166034668
49	cis_NEG	ESC_J1_UP_EARLY.V1_DN	26538	4004	177	26.705404	50	1.872280	2.23098714889626	5.072565e−06	0.0038348592
50	cis_POS	INORGANIC_ANION_TRANSMEMBRANE_TRANSPORTER_ACTIVITY	26538	1529	19	1.094694	8	7.307976	11.949904569911601	5.083807e−06	0.0080527506
51	cis_NEG	SINGH_KRAS_DEPENDENCY_SIGNATURE_	26538	4004	20	3.017560	12	3.976723	8.4630116030445102	5.247379e−06	0.0039670184
52	trans_NEG	REACTOME_CHEMOKINE_RECEPTORS_BIND_CHEMOKINES	26538	8266	54	16.819806	33	1.961973	3.4832402170885399	5.293354e−06	0.0142708825
53	trans_NEG	POSITIVE_REGULATION_OF_DEVELOPMENTAL_PROCESS	26538	8266	212	66.033311	97	1.468956	1.8748186002498499	5.382447e−06	0.0177620767
54	trans_POS	REACTOME_FANCONI_ANEMIA_PATHWAY	26538	7352	21	5.817771	16	2.750194	8.3657381806797293	5.383682e−06	0.0145144062
55	trans_POS	PROGRAMMED_CELL_DEATH	26538	7352	423	117.186525	159	1.356811	1.5843161157163499	5.466903e−06	0.0180407797
56	cis_NEG	KRAS.PROSTATE_UP.V1_DN	26538	4004	140	21.122918	42	1.988362	2.4268056291027502	5.498099e−06	0.0041565629
57	trans_POS	RESPONSE_TO_ENDOGENOUS_STIMULUS	26538	7352	195	54.022157	83	1.536407	1.9446263592520801	5.504111e−06	0.0181635669
58	trans_POS	CAMP_UP.V1_DN	26538	7352	195	54.022157	83	1.536407	1.9446263592520801	5.504111e−06	0.0041611081
59	trans_NEG	REACTOME_EXTENSION_OF_TELOMERES	26538	8266	27	8.409903	20	2.378149	6.3279602915659598	5.574393e−06	0.0150285637
60	cis_NEG	REGULATION_OF_CATALYTIC_ACTIVITY	26538	4004	271	40.887934	69	1.687539	1.93860895150971	5.594545e−06	0.0184619996
61	trans_POS	DNA_REPAIR	26538	7352	122	33.798478	57	1.686466	2.29845530240339	5.651612e−06	0.0186503192
62	cis_NEG	REACTOME_TRANSLOCATION_OF_ZAP_70_TO_IMMUNOLOGICAL_SYNAPSE	26538	4004	12	1.810536	9	4.970904	16.917476199800401	5.718797e−06	0.0154178765
63	cis_NEG	KRAS.LUNG_UP.V1_DN	26538	4004	136	20.519406	41	1.998109	2.44352334757617	6.184435e−06	0.0046754325
64	cis_NEG	RPS14_DN.V1_DN	26538	4004	183	27.610671	51	1.847112	2.1894811940702898	6.257570e−06	0.0047307231
65	trans_POS	KEGG_DNA_REPLICATION	26538	7352	36	9.973321	23	2.306153	4.62810361322079	6.336873e−06	0.0047146334
66	trans_POS	E2F1_UP.V1_UP	26538	7352	187	51.805863	80	1.544227	1.9616231052534601	6.401019e−06	0.0048391704
67	cis_POS	KRAS.PROSTATE_UP.V1_UP	26538	1529	132	7.605245	22	2.892740	3.3041771107288	6.462261e−06	0.0048854690
68	cis_NEG	ATF2_S_UP.V1_UP	26538	4004	188	28.365061	52	1.833241	2.1669187400414498	6.465213e−06	0.0048877012
69	cis_NEG	PRC2_EZH2_UP.V1_DN	26538	4004	188	28.365061	52	1.833241	2.1669187400414498	6.465213e−06	0.0048877012
70	cis_NEG	REACTOME_SIGNALING_BY_SCF_KIT	26538	4004	75	11.315849	27	2.386034	3.18018675373908	6.538657e−06	0.0176282189
71	trans_POS	REACTOME_TRANSLOCATION_OF_ZAP_70_TO_IMMUNOLOGICAL_SYNAPSE	26538	7352	12	3.324440	11	3.308828	28.744603988008802	6.571569e−06	0.0177169490
72	trans_NEG	KEGG_P53_SIGNALING_PATHWAY	26538	8266	68	21.180496	39	1.841317	2.9819575440370398	6.867860e−06	0.0051096879
73	trans_NEG	CELL_MATRIX_JUNCTION	26538	8266	18	5.606602	15	2.675417	11.0663278118487	7.259525e−06	0.0067658777
74	cis_POS	REACTOME_NEUROTRANSMITTER_RECEPTOR_BINDING_AND_DOWNSTREAM_TRANSMISSION_IN_THE_POSTSYNAPTIC_CELL	26538	1529	133	7.662861	22	2.870990	3.27428121171249	7.327104e−06	0.0197538732
75	cis_POS	KRAS.KIDNEY_UP.V1_DN	26538	1529	133	7.662861	22	2.870990	3.27428121171249	7.327104e−06	0.0055392908
76	trans_POS	PROTEIN_DIMERIZATION_ACTIVITY	26538	7352	179	49.589570	77	1.552746	1.9803097816794499	7.416114e−06	0.0117471240
77	cis_POS	ORGAN_MORPHOGENESIS	26538	1529	143	8.239016	23	2.791596	3.1673470757019002	7.425436e−06	0.0245039386
78	trans_NEG	REACTOME_CHROMOSOME_MAINTENANCE	26538	8266	116	36.131434	59	1.632927	2.2972704211580801	7.426831e−06	0.0200227351
79	cis_NEG	REGULATION_OF_PROTEIN_AMINO_ACID_PHOSPHORYLATION	26538	4004	30	4.526340	15	3.313936	5.6447565045616903	7.502585e−06	0.0247585313
80	cis_NEG	EXTRACELLULAR_REGION	26538	4004	435	65.631924	100	1.523649	1.69733445049758	7.518308e−06	0.0070070634
81	trans_NEG	FOCAL_ADHESION	26538	8266	13	4.049212	12	2.963539	26.560541526698401	7.684754e−06	0.0071621910
82	trans_POS	KEGG_VIRAL_MYOCARDITIS	26538	7352	65	18.007386	35	1.943647	3.0543775678898699	7.780858e−06	0.0057889584
83	trans_POS	NUCLEUS	26538	7352	1404	388.959530	461	1.185213	1.2942155488520799	7.914963e−06	0.0073767457
84	cis_POS	REACTOME_SLC_MEDIATED_TRANSMEMBRANE_TRANSPORT	26538	1529	239	13.770103	32	2.323875	2.5610343778134399	7.990008e−06	0.0215410611
85	cis_NEG	NERVOUS_SYSTEM_DEVELOPMENT	26538	4004	372	56.126611	88	1.567884	1.76051539684392	8.048127e−06	0.0265588187
86	trans_POS	HOXA9_DN.V1_DN	26538	7352	188	52.082900	80	1.536013	1.9433647401838601	8.139962e−06	0.0061538115
87	cis_POS	REACTOME_POTASSIUM_CHANNELS	26538	1529	96	5.531087	18	3.254333	3.8069368394883298	8.239259e−06	0.0222130425
88	trans_POS	EXTRACELLULAR_REGION	26538	7352	435	120.510965	162	1.344276	1.56090277585031	8.250727e−06	0.0076896775
89	cis_NEG	PHOSPHOPROTEIN_PHOSPHATASE_ACTIVITY	26538	4004	80	12.070239	28	2.319755	3.0445128991372599	8.283141e−06	0.0131204953
90	trans_POS	KEGG_NATURAL_KILLER_CELL_MEDIATED_CYTOTOXICITY	26538	7352	126	34.906624	58	1.661576	2.2355651970762902	8.321312e−06	0.0061910558
91	trans_POS	VEGF_A_UP.V1_DN	26538	7352	191	52.914010	81	1.530786	1.9319486097497101	8.367861e−06	0.0063261028
92	cis_POS	INTRACELLULAR_SIGNALING_CASCADE	26538	1529	657	37.853380	66	1.743570	1.8639231345224601	8.407231e−06	0.0277438627
93	trans_POS	REGULATION_OF_MITOSIS	26538	7352	41	11.358505	25	2.200994	4.0877679384679499	8.476655e−06	0.0279729624
94	cis_NEG	CRX_DN.V1_DN	26538	4004	133	20.066772	40	1.993345	2.4348118078298602	8.500685e−06	0.0064265178
95	trans_NEG	BIOCARTA_BLYMPHOCYTE_PATHWAY	26538	8266	10	3.114779	10	3.210501	Inf	8.563345e−06	0.0074329832
96	trans_NEG	BIOCARTA_TCAPOPTOSIS_PATHWAY	26538	8266	10	3.114779	10	3.210501	Inf	8.563345e−06	0.0074329832
97	trans_NEG	REACTOME_PECAM1_INTERACTIONS	26538	8266	10	3.114779	10	3.210501	Inf	8.563345e−06	0.0230867773
98	trans_POS	CHROMATIN_BINDING	26538	7352	32	8.865174	21	2.368820	4.9930966142832398	8.668071e−06	0.0137302250
99	trans_POS	BMI1_DN_MEL18_DN.V1_DN	26538	7352	140	38.785138	63	1.624334	2.1449418527002302	8.670153e−06	0.0065546353
100	cis_NEG	INFLAMMATORY_RESPONSE	26538	4004	124	18.708870	38	2.031122	2.5008612968807702	8.724398e−06	0.0287905118

	set1_Name	set2_Name	Background	set1_size	set2_size	expected.overlap	actual.overlap	enrichment.foldchange	odds.ratio	FET_pvalue	corrected.FET.pvalue
1	cis_NEG	REACTOME_THE_ROLE_OF_NEF_IN_HIV1_REPLICATION_AND_DISEASE_PATHOGENESIS	26538	4004	27	4.073706	14	3.436674	6.0779140049189602	8.802950e−06	0.023732753
2	cis_POS	IL2_UP.V1_DN	26538	1529	186	10.716482	27	2.519484	2.8092870368843101	9.050248e−06	0.006841987
3	trans_NEG	RECEPTOR_BINDING	26538	8266	374	116.492727	156	1.339139	1.5929911037043101	9.139823e−06	0.014477479
4	cis_NEG	CORDENONSI_YAP_CONSERVED_SIGNATURE	26538	4004	56	8.449167	22	2.603807	3.6559285852303698	9.187632e−06	0.006945850
5	cis_NEG	PHOSPHORIC_ESTER_HYDROLASE_ACTIVITY	26538	4004	152	22.933454	44	1.918595	2.3071074800034799	9.242630e−06	0.014640326
6	cis_NEG	POSITIVE_REGULATION_OF_MULTICELLULAR_ORGANISMAL_PROCESS	26538	4004	64	9.656191	24	2.485452	3.3908395296889502	9.408543e−06	0.031048191
7	trans_NEG	REACTOME_M_G1_TRANSITION	26538	8266	78	24.295275	43	1.769891	2.7246197652639199	9.492686e−06	0.025592281
8	cis_NEG	HOMEOSTATIC_PROCESS	26538	4004	205	30.929987	55	1.778210	2.0783246058657499	9.533808e−06	0.031461565
9	cis_POS	KEGG_PROSTATE_CANCER	26538	1529	88	5.070164	17	3.352949	3.9491030541048402	9.632638e−06	0.007166682
10	trans_NEG	PHOSPHORIC_ESTER_HYDROLASE_ACTIVITY	26538	8266	152	47.344638	73	1.541885	2.0519033766022998	9.632946e−06	0.015258587
11	trans_POS	KEGG_LEISHMANIA_INFECTION	26538	7352	68	18.838496	36	1.910981	2.9452193337608499	9.645286e−06	0.007176093
12	cis_NEG	PHOSPHORIC_MONOESTER_HYDROLASE_ACTIVITY	26538	4004	111	16.747456	35	2.089870	2.6056521661037499	9.722009e−06	0.015399662
13	cis_NEG	BIOCARTA_BCR_PATHWAY	26538	4004	34	5.129852	16	3.118999	5.0182874999715903	1.008689e−05	0.008755423
14	cis_NEG	REGULATION_OF_LYMPHOCYTE_ACTIVATION	26538	4004	34	5.129852	16	3.118999	5.0182874999715903	1.008689e−05	0.033286748
15	trans_NEG	MEL18_DN.V1_DN	26538	8266	142	44.229859	69	1.560032	2.0985357310506401	1.014971e−05	0.007673180
16	trans_NEG	VEGF_A_UP.V1_DN	26538	8266	191	59.492275	88	1.479184	1.89818265735501	1.024965e−05	0.007748738
17	cis_POS	RAPA_EARLY_UP.V1_UP	26538	1529	177	10.197943	26	2.549534	2.8475790801252199	1.061205e−05	0.008022710
18	cis_POS	CALCIUM_CHANNEL_ACTIVITY	26538	1529	33	1.901311	10	5.259528	7.14985833691712	1.067761e−05	0.016913332
19	cis_NEG	KEGG_FC_GAMMA_R_MEDIATED_PHAGOCYTOSIS	26538	4004	94	14.182531	31	2.185788	2.7830634167536301	1.123710e−05	0.008360405
20	trans_NEG	NUCLEAR_PART	26538	8266	572	178.165348	226	1.268485	1.45632302712503	1.124906e−05	0.010484120
21	cis_POS	HALLMARK_APICAL_JUNCTION	26538	1529	199	11.465483	28	2.442112	2.7096004703857002	1.134497e−05	0.002268994
22	cis_POS	HALLMARK_KRAS_SIGNALING_UP	26538	1529	199	11.465483	28	2.442112	2.7096004703857002	1.134497e−05	0.002268994
23	trans_NEG	JNK_DN.V1_DN	26538	8266	181	56.377496	84	1.489956	1.9236652734391699	1.146022e−05	0.008663929
24	trans_NEG	KRAS.600_UP.V1_UP	26538	8266	274	85.344939	119	1.394342	1.7072489101013599	1.146383e−05	0.008666658
25	cis_NEG	PROTEIN_COMPLEX	26538	4004	805	121.456779	166	1.366741	1.4819684513448099	1.160903e−05	0.010819620
26	cis_NEG	POSITIVE_REGULATION_OF_IMMUNE_SYSTEM_PROCESS	26538	4004	49	7.393021	20	2.705254	3.8955354436881402	1.183975e−05	0.039071181
27	cis_NEG	MOLECULAR_ADAPTOR_ACTIVITY	26538	4004	49	7.393021	20	2.705254	3.8955354436881402	1.183975e−05	0.018754167
28	cis_NEG	ACTIN_CYTOSKELETON_ORGANIZATION_AND_BIOGENESIS	26538	4004	103	15.540433	33	2.123493	2.66664327431076	1.186563e−05	0.039156571
29	cis_NEG	KEGG_CHRONIC_MYELOID_LEUKEMIA	26538	4004	73	11.014093	26	2.360612	3.1269152467862802	1.202825e−05	0.008949015
30	trans_POS	JNK_DN.V1_DN	26538	7352	181	50.143643	77	1.535588	1.94203710091375	1.204315e−05	0.009104618
31	trans_NEG	KEGG_CALCIUM_SIGNALING_PATHWAY	26538	8266	176	54.820107	82	1.495802	1.9376378982921401	1.207860e−05	0.008986478
32	trans_POS	DNA_DEPENDENT_DNA_REPLICATION	26538	7352	56	15.514055	31	1.998188	3.24523864019935	1.209816e−05	0.039923939
33	trans_POS	ORGANELLE_PART	26538	7352	1177	326.072198	391	1.199121	1.3149232753021101	1.210387e−05	0.011280808
34	cis_POS	PRC2_EDD_UP.V1_DN	26538	1529	189	10.889329	27	2.479492	2.7569885464442501	1.219805e−05	0.009221726
35	trans_POS	KEGG_TYPE_I_DIABETES_MELLITUS	26538	7352	37	10.250358	23	2.243824	4.2972481405751601	1.235178e−05	0.009189727
36	cis_POS	KEGG_PATHWAYS_IN_CANCER	26538	1529	325	18.725036	39	2.082773	2.2626290256321502	1.239443e−05	0.009221455
37	cis_NEG	REACTOME_NETRIN1_SIGNALING	26538	4004	38	5.733363	17	2.965101	4.5708992384838103	1.243547e−05	0.033526026
38	trans_NEG	REGULATION_OF_BIOLOGICAL_QUALITY	26538	8266	412	128.328887	169	1.316929	1.5485370780412	1.260019e−05	0.041580638
39	trans_NEG	E2F1_UP.V1_DN	26538	8266	184	57.311930	85	1.483112	1.9072692985551201	1.268357e−05	0.009588779
40	trans_NEG	ATF2_S_UP.V1_DN	26538	8266	184	57.311930	85	1.483112	1.9072692985551201	1.268357e−05	0.009588779
41	trans_NEG	KEGG_ARRHYTHMOGENIC_RIGHT_VENTRICULAR_CARDIOMYOPATHY_ARVC	26538	8266	74	23.049363	41	1.778791	2.7549713891925198	1.289775e−05	0.009595927
42	trans_POS	MITOTIC_SISTER_CHROMATID_SEGREGATION	26538	7352	16	4.432587	13	2.932824	11.3253869435121	1.293423e−05	0.042682962
43	cis_NEG	REACTOME_INNATE_IMMUNE_SYSTEM	26538	4004	257	38.775642	65	1.676310	1.92004146360949	1.294715e−05	0.034905509
44	cis_POS	PRC1_BMI_UP.V1_DN	26538	1529	179	10.313174	26	2.521047	2.81013488346498	1.301216e−05	0.009837191
45	trans_POS	REACTOME_G_ALPHA_I_SIGNALLING_EVENTS	26538	7352	190	52.636973	80	1.519844	1.9078442451875	1.301288e−05	0.035082720
46	cis_POS	KEGG_GAP_JUNCTION	26538	1529	90	5.185395	17	3.278439	3.8398683508662801	1.316101e−05	0.009791793
47	cis_NEG	POSITIVE_REGULATION_OF_CELLULAR_METABOLIC_PROCESS	26538	4004	227	34.249303	59	1.722663	1.99106401827268	1.326039e−05	0.043759297
48	cis_NEG	KEGG_JAK_STAT_SIGNALING_PATHWAY	26538	4004	154	23.235210	44	1.893678	2.2649617543687901	1.329082e−05	0.009888371
49	cis_NEG	ENZYME_LINKED_RECEPTOR_PROTEIN_SIGNALING_PATHWAY	26538	4004	140	21.122918	41	1.941020	2.3444039131657601	1.349677e−05	0.044539330
50	cis_NEG	IL21_UP.V1_UP	26538	4004	178	26.856282	49	1.824527	2.1517481083094299	1.352464e−05	0.010224630
51	trans_NEG	HYDROLASE_ACTIVITY_ACTING_ON_ESTER_BONDS	26538	8266	264	82.230161	115	1.398514	1.71602640730334	1.352570e−05	0.021424708
52	trans_POS	HALLMARK_MYC_TARGETS_V1	26538	7352	196	54.299194	82	1.510151	1.88683902696268	1.359871e−05	0.002719741
53	trans_NEG	CHROMOSOMEPERICENTRIC_REGION	26538	8266	30	9.344336	21	2.247351	5.1677562923533404	1.379222e−05	0.012854349
54	trans_POS	INTERLEUKIN_BINDING	26538	7352	24	6.648881	17	2.556821	6.3486203097313698	1.379738e−05	0.021855050
55	trans_POS	HALLMARK_ESTROGEN_RESPONSE_LATE	26538	7352	199	55.130304	83	1.505524	1.8769872364970701	1.387258e−05	0.002774516
56	trans_POS	HALLMARK_APICAL_JUNCTION	26538	7352	199	55.130304	83	1.505524	1.8769872364970701	1.387258e−05	0.002774516
57	trans_POS	INTRACELLULAR_ORGANELLE_PART	26538	7352	1172	324.687015	389	1.198077	1.3130472196557901	1.390787e−05	0.012962132
58	trans_POS	KEGG_ANTIGEN_PROCESSING_AND_PRESENTATION	26538	7352	74	20.500716	38	1.853594	2.7636035266375099	1.400241e−05	0.010417797
59	cis_NEG	HOXA9_DN.V1_DN	26538	4004	188	28.365061	51	1.797987	2.1091220818168099	1.419773e−05	0.010733487
60	cis_NEG	TRANSMEMBRANE_RECEPTOR_PROTEIN_TYROSINE_KINASE_ACTIVITY	26538	4004	42	6.336875	18	2.840517	4.2353019355191597	1.439844e−05	0.022807133
61	trans_NEG	IMMUNE_SYSTEM_DEVELOPMENT	26538	8266	79	24.606753	43	1.747488	2.6487961535293598	1.457631e−05	0.048101816
62	trans_NEG	REACTOME_SIGNALING_BY_PDGF	26538	8266	118	36.754390	59	1.605250	2.21916593540639	1.459958e−05	0.039360468
63	trans_POS	CELL_SURFACE_RECEPTOR_LINKED_SIGNAL_TRANSDUCTION_GO_0007166	26538	7352	635	175.918306	224	1.273318	1.4355291800724299	1.482139e−05	0.048910591
64	cis_POS	KEGG_MELANOGENESIS	26538	1529	100	5.761549	18	3.124160	3.6209763280130098	1.483864e−05	0.011039948
65	trans_NEG	KEGG_AUTOIMMUNE_THYROID_DISEASE	26538	8266	47	14.639460	29	1.980947	3.5701622456336302	1.502651e−05	0.011179727
66	cis_POS	KRAS.LUNG.BREAST_UP.V1_DN	26538	1529	139	8.008554	22	2.747063	3.1056382688122302	1.510696e−05	0.011420864
67	cis_NEG	CRX_NRL_DN.V1_UP	26538	4004	136	20.519406	40	1.949374	2.35841842834503	1.528593e−05	0.011556161
68	cis_NEG	TRANSMEMBRANE_RECEPTOR_ACTIVITY	26538	4004	410	61.859974	94	1.519561	1.69027605094293	1.554037e−05	0.024615944
69	cis_POS	JNK_DN.V1_DN	26538	1529	181	10.428405	26	2.493191	2.7736560995589699	1.589542e−05	0.012016937
70	cis_NEG	BCAT_GDS748_UP	26538	4004	46	6.940387	19	2.737599	3.97425894900362	1.590537e−05	0.012024457
71	cis_NEG	PRC1_BMI_UP.V1_DN	26538	4004	179	27.007160	49	1.814334	2.1351052052489798	1.592533e−05	0.012039551
72	cis_NEG	HYDROLASE_ACTIVITY_ACTING_ON_ESTER_BONDS	26538	4004	264	39.831788	66	1.656968	1.8905058265027299	1.666676e−05	0.026400155
73	trans_NEG	REACTOME_SIGNALING_BY_ILS	26538	8266	106	33.016655	54	1.635538	2.3039862685958998	1.673789e−05	0.045125343
74	cis_NEG	TRANSMEMBRANE_RECEPTOR_PROTEIN_KINASE_ACTIVITY	26538	4004	50	7.543899	20	2.651149	3.7655006256480301	1.694481e−05	0.026840587
75	cis_POS	BMI1_DN_MEL18_DN.V1_DN	26538	1529	140	8.066169	22	2.727441	3.07919853574808	1.696170e−05	0.012823049
76	cis_NEG	GCNP_SHH_UP_EARLY.V1_DN	26538	4004	165	24.894868	46	1.847770	2.18906595872923	1.718946e−05	0.012995234
77	cis_NEG	REACTOME_CHEMOKINE_RECEPTORS_BIND_CHEMOKINES	26538	4004	54	8.147411	21	2.577506	3.5947545366692002	1.754421e−05	0.047299193
78	trans_POS	PTEN_DN.V2_UP	26538	7352	137	37.954028	61	1.607208	2.1037192638616902	1.784992e−05	0.013494537
79	trans_NEG	PRC2_EZH2_UP.V1_DN	26538	8266	188	58.557842	86	1.468633	1.8728812022085	1.796525e−05	0.013581727
80	cis_NEG	REACTOME_INTERFERON_SIGNALING	26538	4004	151	22.782576	43	1.887407	2.25410882419704	1.810454e−05	0.048809832
81	trans_NEG	PHOSPHOTRANSFERASE_ACTIVITY_ALCOHOL_GROUP_AS_ACCEPTOR	26538	8266	331	103.099179	139	1.348216	1.61054986256535	1.825293e−05	0.028912639
82	cis_NEG	KEGG_HYPERTROPHIC_CARDIOMYOPATHY_HCM	26538	4004	83	12.522873	28	2.235909	2.8780746571578	1.826088e−05	0.013586097
83	cis_NEG	IL21_UP.V1_DN	26538	4004	180	27.158038	49	1.804254	2.1187169029301902	1.871347e−05	0.014147383
84	trans_NEG	CYTOSKELETAL_PROTEIN_BINDING	26538	8266	157	48.902027	74	1.513230	1.97959419465382	1.913908e−05	0.030316300
85	trans_POS	IL2_UP.V1_UP	26538	7352	183	50.697716	77	1.518806	1.9052091512178599	1.924688e−05	0.014550641
86	trans_NEG	RB_DN.V1_UP	26538	8266	134	41.738036	65	1.557333	2.09092440447438	1.937196e−05	0.014645203
87	trans_NEG	RAF_UP.V1_UP	26538	8266	191	59.492275	87	1.462375	1.8582500447695101	1.968606e−05	0.014882661
88	trans_POS	KRAS.KIDNEY_UP.V1_UP	26538	7352	143	39.616248	63	1.590257	2.0642045290037099	1.971041e−05	0.014901069
89	trans_NEG	KEGG_SYSTEMIC_LUPUS_ERYTHEMATOSUS	26538	8266	124	38.623257	61	1.579359	2.1487811557143401	2.005856e−05	0.014923566
90	trans_NEG	ENZYME_REGULATOR_ACTIVITY	26538	8266	318	99.049966	134	1.352853	1.6198414893227	2.092839e−05	0.033150567
91	cis_NEG	KRAS.KIDNEY_UP.V1_DN	26538	4004	133	20.066772	39	1.943511	2.34800026854482	2.094381e−05	0.015833523
92	cis_POS	KEGG_PROGESTERONE_MEDIATED_OOCYTE_MATURATION	26538	1529	84	4.839702	16	3.305989	3.8787885286713801	2.101797e−05	0.015637371
93	cis_NEG	SH3_SH2_ADAPTOR_ACTIVITY	26538	4004	43	6.487753	18	2.774458	4.0656567077516303	2.128282e−05	0.033711982
94	cis_POS	MEL18_DN.V1_DN	26538	1529	142	8.181400	22	2.689026	3.0276410491921801	2.129871e−05	0.016101823
95	cis_NEG	ENZYME_REGULATOR_ACTIVITY	26538	4004	318	47.979200	76	1.584020	1.7822300126745301	2.216014e−05	0.035101663
96	cis_NEG	ALK_DN.V1_UP	26538	4004	138	20.821162	40	1.921122	2.3100848388734501	2.226516e−05	0.016832461
97	trans_POS	PHOSPHORIC_ESTER_HYDROLASE_ACTIVITY	26538	7352	152	42.109579	66	1.567339	2.0118405820802399	2.238573e−05	0.035459000
98	trans_NEG	MYC_UP.V1_DN	26538	8266	168	52.328284	78	1.490590	1.92452202429096	2.265939e−05	0.017130501
99	trans_NEG	SPINDLE	26538	8266	39	12.147637	25	2.058013	3.9558438736241599	2.269296e−05	0.021149839
100	cis_NEG	HALLMARK_UV_RESPONSE_DN	26538	4004	143	21.575552	41	1.900299	2.2751519160989502	2.350309e−05	0.004700617

	set1_Name	set2_Name	Background	set1_size	set2_size	expected.overlap	actual.overlap	enrichment.foldchange	odds.ratio	FET_pvalue	corrected.FET.pvalue
1	trans_POS	BIOCARTA_NKT_PATHWAY	26538	7352	29	8.034064	19	2.364930	4.9682252059190803	2.429786e−05	0.021090547
2	trans_NEG	BIOCARTA_CTLA4_PATHWAY	26538	8266	19	5.918080	15	2.534606	8.3018730436671593	2.447574e−05	0.021244944
3	trans_POS	KRAS.DF.V1_DN	26538	7352	190	52.636973	79	1.500846	1.86647236737511	2.514086e−05	0.019006488
4	cis_NEG	E2F1_UP.V1_UP	26538	4004	187	28.214183	50	1.772158	2.06725968480273	2.607965e−05	0.019716218
5	trans_NEG	PRC2_EDD_UP.V1_UP	26538	8266	187	58.246364	85	1.459319	1.8508837026015099	2.678974e−05	0.020253047
6	cis_NEG	ACTIN_CYTOSKELETON	26538	4004	125	18.859748	37	1.961850	2.3790335944156999	2.683305e−05	0.025008406
7	cis_NEG	RB_DN.V1_DN	26538	4004	125	18.859748	37	1.961850	2.3790335944156999	2.683305e−05	0.020285789
8	cis_NEG	KEGG_PHOSPHATIDYLINOSITOL_SIGNALING_SYSTEM	26538	4004	76	11.466727	26	2.267430	2.9389192822094001	2.719523e−05	0.020233252
9	trans_NEG	BASAL_LAMINA	26538	8266	21	6.541035	16	2.446096	7.0847076023810098	2.815000e−05	0.026235803
10	cis_NEG	BIOCARTA_ASBCELL_PATHWAY	26538	4004	11	1.659658	8	4.820271	15.032992481510499	2.864315e−05	0.024862253
11	cis_NEG	IMMUNOLOGICAL_SYNAPSE	26538	4004	11	1.659658	8	4.820271	15.032992481510499	2.864315e−05	0.026695414
12	trans_POS	KRAS.600_UP.V1_UP	26538	7352	274	75.908056	107	1.409600	1.6819268109133301	2.885246e−05	0.021812458
13	trans_NEG	PROTEIN_COMPLEX	26538	8266	805	250.739694	304	1.212413	1.3543519320617099	2.967106e−05	0.027653431
14	cis_NEG	CYCLIN_D1_KE_.V1_UP	26538	4004	188	28.365061	50	1.762732	2.0521947703272199	3.038470e−05	0.022970831
15	cis_NEG	KEGG_LEISHMANIA_INFECTION	26538	4004	68	10.259703	24	2.339249	3.0820464978899502	3.055891e−05	0.022735829
16	cis_POS	PRC2_EZH2_UP.V1_DN	26538	1529	188	10.831713	26	2.400359	2.6529457955267302	3.113424e−05	0.023537484
17	cis_POS	HALLMARK_MYOGENESIS	26538	1529	199	11.465483	27	2.354894	2.5955600719760001	3.122156e−05	0.006244313
18	trans_POS	P53_DN.V1_UP	26538	7352	191	52.914010	79	1.492988	1.84971923895437	3.133405e−05	0.023688539
19	trans_NEG	BIOCARTA_NKT_PATHWAY	26538	8266	29	9.032859	20	2.214139	4.9213621010904296	3.134862e−05	0.027210606
20	trans_POS	MEK_UP.V1_UP	26538	7352	194	53.745120	80	1.488507	1.8403364254399499	3.181053e−05	0.024048764
21	cis_NEG	RECEPTOR_COMPLEX	26538	4004	56	8.449167	21	2.485452	3.38903865358431	3.347156e−05	0.031195496
22	trans_POS	ADHERENS_JUNCTION	26538	7352	23	6.371844	16	2.511047	5.9741489144226803	3.566300e−05	0.033237918
23	cis_NEG	KEGG_INSULIN_SIGNALING_PATHWAY	26538	4004	136	20.519406	39	1.900640	2.2750830866840799	3.652531e−05	0.027174828
24	trans_NEG	KRAS.DF.V1_UP	26538	8266	191	59.492275	86	1.445566	1.81888374864367	3.705168e−05	0.028011074
25	trans_POS	ATF2_UP.V1_DN	26538	7352	183	50.697716	76	1.499081	1.86234334501836	3.715127e−05	0.028086363
26	trans_POS	PROTEINACEOUS_EXTRACELLULAR_MATRIX	26538	7352	98	27.149597	46	1.694316	2.3166972686299201	3.755545e−05	0.035001681
27	cis_NEG	KEGG_APOPTOSIS	26538	4004	86	12.975507	28	2.157912	2.72865152387753	3.814499e−05	0.028379875
28	cis_POS	BMI1_DN.V1_DN	26538	1529	137	7.893323	21	2.660477	2.98817888868947	3.822203e−05	0.028895857
29	trans_POS	BIOCARTA_ATRBRCA_PATHWAY	26538	7352	21	5.817771	15	2.578307	6.5340017240883004	3.881552e−05	0.033691874
30	trans_NEG	IL21_UP.V1_UP	26538	8266	178	55.443063	81	1.460958	1.85430273159498	3.891639e−05	0.029420789
31	cis_NEG	WNT_UP.V1_UP	26538	4004	175	26.403648	47	1.780057	2.0791104868741801	3.968199e−05	0.029999588
32	cis_NEG	PRC1_BMI_UP.V1_UP	26538	4004	180	27.158038	48	1.767433	2.0591643718676602	4.024440e−05	0.030424765
33	cis_NEG	ESC_V6.5_UP_LATE.V1_DN	26538	4004	180	27.158038	48	1.767433	2.0591643718676602	4.024440e−05	0.030424765
34	cis_NEG	IL15_UP.V1_UP	26538	4004	180	27.158038	48	1.767433	2.0591643718676602	4.024440e−05	0.030424765
35	trans_NEG	NFE2L2.V2	26538	8266	442	137.673223	177	1.285653	1.4868602382026099	4.046432e−05	0.030591024
36	cis_NEG	BIOCARTA_TOB1_PATHWAY	26538	4004	20	3.017560	11	3.645330	6.8938988076174397	4.050612e−05	0.035159314
37	cis_NEG	RUFFLE	26538	4004	30	4.526340	14	3.093007	4.9378624023065099	4.075650e−05	0.037985055
38	trans_POS	BIOCARTA_CTLA4_PATHWAY	26538	7352	19	5.263697	14	2.659727	7.31779418961949	4.082471e−05	0.035435852
39	cis_POS	RAF_UP.V1_UP	26538	1529	191	11.004559	26	2.362657	2.6044565552619199	4.100121e−05	0.030996915
40	trans_POS	SPINDLE	26538	7352	39	10.804431	23	2.128756	3.7597489377887201	4.152734e−05	0.038703478
41	cis_NEG	EXTRACELLULAR_REGION_PART	26538	4004	329	49.638857	77	1.551204	1.7336913942769101	4.212241e−05	0.039258090
42	trans_NEG	SNF5_DN.V1_DN	26538	8266	160	49.836461	74	1.484857	1.9102420724065701	4.247973e−05	0.032114675
43	trans_NEG	CYCLIN_D1_KE_.V1_DN	26538	8266	189	58.869319	85	1.443876	1.8148995359162901	4.314919e−05	0.032620784
44	trans_POS	KRAS.PROSTATE_UP.V1_UP	26538	7352	132	36.568845	58	1.586050	2.05370327829859	4.522043e−05	0.034186649
45	cis_NEG	KEGG_ACUTE_MYELOID_LEUKEMIA	26538	4004	57	8.600045	21	2.441848	3.2947552277494001	4.550662e−05	0.033856926
46	trans_NEG	CYCLIN_D1_UP.V1_UP	26538	8266	184	57.311930	83	1.448215	1.8246867244026701	4.627054e−05	0.034980525
47	trans_POS	PRC2_EDD_UP.V1_UP	26538	7352	187	51.805863	77	1.486318	1.8353661725031101	4.694729e−05	0.035492148
48	trans_POS	ESC_J1_UP_LATE.V1_UP	26538	7352	187	51.805863	77	1.486318	1.8353661725031101	4.694729e−05	0.035492148
49	trans_POS	CAHOY_OLIGODENDROCUTIC	26538	7352	96	26.595523	45	1.692014	2.3105777775231999	4.730707e−05	0.035764149
50	trans_NEG	CELL_JUNCTION	26538	8266	82	25.541186	43	1.683555	2.44465915449427	4.844889e−05	0.045154364
51	trans_POS	HALLMARK_MITOTIC_SPINDLE	26538	7352	199	55.130304	81	1.469246	1.8000678703213699	4.916895e−05	0.009833789
52	trans_POS	EXTRACELLULAR_MATRIX	26538	7352	99	27.426634	46	1.677202	2.2728749652652298	5.143925e−05	0.047941381
53	cis_NEG	RB_P107_DN.V1_UP	26538	4004	138	20.821162	39	1.873094	2.2289282854388102	5.215566e−05	0.039429681
54	cis_NEG	KRAS.LUNG.BREAST_UP.V1_UP	26538	4004	138	20.821162	39	1.873094	2.2289282854388102	5.215566e−05	0.039429681
55	cis_POS	KRAS.PROSTATE_UP.V1_DN	26538	1529	140	8.066169	21	2.603466	2.9125041392447502	5.297853e−05	0.040051766
56	trans_NEG	KRAS.KIDNEY_UP.V1_UP	26538	8266	143	44.541337	67	1.504221	1.9565064875117699	5.826720e−05	0.044050007
57	cis_NEG	KEGG_PROSTATE_CANCER	26538	4004	88	13.277263	28	2.108868	2.6375961169508302	6.063282e−05	0.045110821
58	trans_NEG	RELA_DN.V1_DN	26538	8266	138	42.983948	65	1.512192	1.97594617759636	6.107170e−05	0.046170206
59	cis_NEG	PKCA_DN.V1_UP	26538	4004	168	25.347502	45	1.775323	2.0709769576492199	6.132978e−05	0.046365314
60	trans_NEG	KEGG_DILATED_CARDIOMYOPATHY	26538	8266	90	28.033009	46	1.640923	2.3182592879980701	6.177265e−05	0.045958851
61	cis_NEG	PTEN_DN.V2_DN	26538	4004	139	20.972040	39	1.859619	2.20654392531481	6.206216e−05	0.046918990
62	cis_NEG	KEGG_DORSO_VENTRAL_AXIS_FORMATION	26538	4004	24	3.621072	12	3.313936	5.64126674824261	6.231310e−05	0.046360945
63	trans_NEG	ESC_V6.5_UP_LATE.V1_DN	26538	8266	180	56.066019	81	1.444725	1.8164518960573801	6.280445e−05	0.047480167
64	cis_NEG	PRC2_SUZ12_UP.V1_DN	26538	4004	183	27.610671	48	1.738458	2.0131595042178398	6.311765e−05	0.047716945
65	cis_POS	ESC_V6.5_UP_LATE.V1_UP	26538	1529	185	10.658867	25	2.345465	2.5813658653087002	6.450207e−05	0.048763564
66	trans_NEG	HALLMARK_GLYCOLYSIS	26538	8266	200	62.295576	88	1.412620	1.74472633867586	8.478575e−05	0.016957150
67	trans_NEG	HALLMARK_TNFA_SIGNALING_VIA_NFKB	26538	8266	200	62.295576	87	1.396568	1.7093286210145799	1.502414e−04	0.030048278
68	cis_NEG	HALLMARK_PEROXISOME	26538	4004	102	15.389555	30	1.949374	2.3550002240229402	1.683351e−04	0.033667018
69	trans_NEG	HALLMARK_P53_PATHWAY	26538	8266	199	61.984098	86	1.387453	1.6894732074443899	2.134498e−04	0.042689963
70	trans_POS	HALLMARK_ESTROGEN_RESPONSE_EARLY	26538	7352	198	54.853267	78	1.421975	1.70368732922322	2.349156e−04	0.046983117

Supporting Material 7C: Modules enriched with meSCGs

	module	methylation_signature	Background	set1_size	set2_size	expected.overlap	actual.overlap	enrichment.foldchange	odds.ratio	FET_pvalue	corrected.FET.pvalue
1	M6	trans_NEG	26538	2273	8266	707.98922	1375	1.942120	3.860248	5.835937e−201	1.433306e−197
2	M6	trans_POS	26538	2273	7352	629.70442	1214	1.927889	3.385273	7.160354e−162	1.758583e−158
3	M6	cis_NEG	26538	2273	4004	342.94566	695	2.026560	2.789121	2.565080e−86	6.299837e−83
4	M106	trans_NEG	26538	547	8266	170.37840	387	2.271415	5.559260	2.685184e−82	6.594813e−79
5	M378	trans_NEG	26538	462	8266	143.90278	342	2.376605	6.527884	2.525700e−81	6.203119e−78
6	M106	trans_POS	26538	547	7352	151.53908	362	2.388823	5.318698	8.532897e−80	2.095679e−76
7	M378	trans_POS	26538	462	7352	127.99096	320	2.500177	6.102490	3.513064e−78	8.628085e−75
8	M102	trans_POS	26538	322	7352	89.20582	249	2.791298	9.176935	3.018654e−77	7.413813e−74
9	M102	trans_NEG	26538	322	8266	100.29588	257	2.562418	8.987490	2.882464e−73	7.079331e−70
10	M111	trans_NEG	26538	787	8266	245.13309	479	1.954041	3.587430	4.586695e−68	1.126492e−64
11	M109	trans_NEG	26538	865	8266	269.42837	509	1.889185	3.302026	1.850560e−65	4.544976e−62
12	M111	trans_POS	26538	787	7352	218.02788	430	1.972225	3.275970	1.424693e−58	3.499045e−55
13	M109	trans_POS	26538	865	7352	239.63675	460	1.919572	3.094955	4.902986e−58	1.204173e−54
14	M378	cis_NEG	26538	462	4004	69.70563	212	3.041361	4.983012	7.177665e−57	1.762835e−53
15	M399	trans_NEG	26538	644	8266	200.59176	390	1.944247	3.512429	1.254216e−54	3.080354e−51
16	M633	trans_NEG	26538	387	8266	120.54194	268	2.223293	5.111245	3.645395e−54	8.953091e−51
17	M357	trans_POS	26538	138	7352	38.23106	123	3.217279	21.745322	4.841170e−52	1.188991e−48
18	M384	trans_NEG	26538	442	8266	137.67322	291	2.113701	4.378678	1.497771e−51	3.678525e−48
19	M635	trans_NEG	26538	499	8266	155.42746	317	2.039537	3.964012	6.068380e−51	1.490394e−47
20	M26	trans_NEG	26538	820	8266	255.41186	460	1.801013	2.931913	6.298655e−51	1.546950e−47
21	M106	cis_NEG	26538	547	4004	82.53026	226	2.738390	4.139428	8.810243e−51	2.163796e−47
22	M105	trans_NEG	26538	328	8266	102.16474	230	2.251266	5.306983	4.135910e−48	1.015779e−44
23	M357	trans_NEG	26538	138	8266	42.98395	124	2.884798	19.860441	2.569197e−47	6.309948e−44
24	M399	trans_POS	26538	644	7352	178.41164	350	1.961755	3.211779	5.038952e−47	1.237567e−43
25	M633	trans_POS	26538	387	7352	107.21320	239	2.229203	4.321848	5.498868e−45	1.350522e−41
26	M635	trans_POS	26538	499	7352	138.24131	286	2.068846	3.605148	1.719481e−44	4.223046e−41
27	M112	trans_POS	26538	603	7352	167.05313	323	1.933517	3.102613	1.032415e−41	2.535611e−38
28	M384	trans_POS	26538	442	7352	122.45022	257	2.098812	3.720134	1.368996e−41	3.362254e−38
29	M116	trans_NEG	26538	454	8266	141.41096	278	1.965901	3.578094	1.994543e−40	4.898598e−37
30	M112	trans_NEG	26538	603	8266	187.82116	339	1.804908	2.916976	6.026384e−38	1.480080e−34
31	M403	trans_POS	26538	285	7352	78.95546	181	2.292432	4.630743	1.083560e−36	2.661224e−33
32	M403	trans_NEG	26538	285	8266	88.77120	192	2.162864	4.648080	1.699827e−36	4.174776e−33
33	M26	trans_POS	26538	820	7352	227.17010	391	1.721177	2.455816	3.969468e−35	9.749014e−32
34	M105	trans_POS	26538	328	7352	90.86804	197	2.167979	4.004646	7.442228e−35	1.827811e−31
35	M729	trans_NEG	26538	339	8266	105.59100	215	2.036158	3.907842	9.731584e−35	2.390077e−31
36	M112	cis_POS	26538	603	1529	34.74214	121	3.482802	4.372580	2.860373e−34	7.025076e−31
37	M209	trans_NEG	26538	243	8266	75.68913	167	2.206394	4.936510	1.482835e−33	3.641842e−30
38	M367	trans_POS	26538	134	7352	37.12292	104	2.801504	9.160920	3.374124e−33	8.286848e−30
39	M116	trans_POS	26538	454	7352	125.77466	246	1.955879	3.158359	4.764666e−33	1.170202e−29
40	M367	trans_NEG	26538	134	8266	41.73804	106	2.539650	8.463255	2.619496e−30	6.433482e−27
41	M113	trans_NEG	26538	318	8266	99.04997	196	1.978799	3.613055	2.153893e−29	5.289961e−26
42	M729	trans_POS	26538	339	7352	93.91544	192	2.044392	3.473096	2.494311e−29	6.126028e−26
43	M111	cis_POS	26538	787	1529	45.34339	132	2.911119	3.512941	4.397994e−29	1.080147e−25
44	M627	trans_NEG	26538	134	8266	41.73804	104	2.491732	7.747237	1.694951e−28	4.162801e−25
45	M104	trans_POS	26538	244	7352	67.59696	150	2.219035	4.229890	2.142344e−28	5.261597e−25
46	M534	trans_NEG	26538	186	8266	57.93489	131	2.261159	5.332969	2.907022e−28	7.139646e−25
47	M399	cis_POS	26538	644	1529	37.10438	114	3.072414	3.720405	2.803031e−27	6.884245e−24
48	M355	cis_NEG	26538	183	4004	27.61067	87	3.150956	5.190941	2.250174e−25	5.526427e−22
49	M121	cis_NEG	26538	194	4004	29.27033	90	3.074786	4.958959	3.091542e−25	7.592826e−22
50	M101	cis_NEG	26538	236	4004	35.60720	100	2.808420	4.218413	4.173652e−24	1.025049e−20
51	M209	trans_POS	26538	243	7352	67.31992	142	2.109331	3.721323	7.476159e−24	1.836145e−20
52	M104	trans_NEG	26538	244	8266	76.00060	152	1.999984	3.701571	8.712165e−24	2.139708e−20
53	M355	trans_NEG	26538	183	8266	57.00045	123	2.157878	4.584600	9.663060e−24	2.373248e−20
54	M627	trans_POS	26538	134	7352	37.12292	93	2.505191	5.981974	1.104592e−23	2.712878e−20
55	M137	cis_NEG	26538	243	4004	36.66335	101	2.754795	4.080470	1.471329e−23	3.613584e−20
56	M403	cis_POS	26538	285	1529	16.42042	67	4.080286	5.210655	1.947934e−23	4.784126e−20
57	M534	trans_POS	26538	186	7352	51.52883	116	2.251167	4.377481	4.900300e−23	1.203514e−19
58	M635	cis_POS	26538	499	1529	28.75013	90	3.130420	3.761086	2.573637e−22	6.320852e−19
59	M113	trans_POS	26538	318	7352	88.09767	169	1.918325	3.006013	6.434410e−22	1.580291e−18
60	M139	cis_NEG	26538	86	4004	12.97551	52	4.007551	8.706200	6.874186e−22	1.688300e−18
61	M475	cis_NEG	26538	154	4004	23.23521	73	3.141784	5.147279	1.987480e−21	4.881251e−18
62	M126	cis_NEG	26538	246	4004	37.11598	98	2.640372	3.794756	2.718895e−21	6.677606e−18
63	M211	trans_NEG	26538	172	8266	53.57420	113	2.109224	4.278254	1.030670e−20	2.531326e−17
64	M367	cis_NEG	26538	134	4004	20.21765	66	3.264474	5.536643	1.205350e−20	2.960339e−17
65	M627	cis_NEG	26538	134	4004	20.21765	66	3.264474	5.536643	1.205350e−20	2.960339e−17
66	M400	trans_POS	26538	112	7352	31.02811	78	2.513849	6.039809	3.069886e−20	7.539640e−17
67	M109	cis_NEG	26538	865	4004	130.50946	233	1.785311	2.141176	4.667011e−20	1.146218e−16
68	M386	trans_NEG	26538	130	8266	40.49212	91	2.247351	5.203411	8.818287e−20	2.165771e−16
69	M102	cis_NEG	26538	322	4004	48.58271	114	2.346514	3.145406	1.077022e−19	2.645165e−16
70	M409	trans_NEG	26538	117	8266	36.44291	84	2.304975	5.673549	1.722225e−19	4.229784e−16
71	M373	trans_NEG	26538	71	8266	22.11493	59	2.667881	10.935336	1.836729e−19	4.511007e−16
72	M109	cis_POS	26538	865	1529	49.83740	120	2.407830	2.773700	2.229772e−19	5.476321e−16
73	M423	trans_POS	26538	153	7352	42.38662	95	2.241274	4.316929	6.127212e−19	1.504843e−15
74	M423	trans_NEG	26538	153	8266	47.65612	101	2.119350	4.333971	6.816761e−19	1.674196e−15
75	M185	trans_POS	26538	225	7352	62.33326	125	2.005350	3.300923	1.199186e−18	2.945200e−15
76	M386	trans_POS	26538	130	7352	36.01477	84	2.332376	4.808622	1.772026e−18	4.352095e−15
77	M478	cis_NEG	26538	68	4004	10.25970	42	4.093686	9.175639	1.916497e−18	4.706917e−15
78	M101	trans_NEG	26538	236	8266	73.50878	137	1.863723	3.093529	9.922599e−18	2.436990e−14
79	M400	trans_NEG	26538	112	8266	34.88552	79	2.264550	5.332526	1.127441e−17	2.768995e−14
80	M28	cis_NEG	26538	244	4004	36.81423	91	2.471870	3.401670	1.160482e−17	2.850143e−14
81	M700	trans_POS	26538	180	7352	49.86661	104	2.085564	3.607754	2.245046e−17	5.513833e−14
82	M226	cis_NEG	26538	223	4004	33.64579	85	2.526319	3.519716	2.914954e−17	7.159128e−14
83	M373	trans_POS	26538	71	7352	19.66961	54	2.745352	8.342207	3.341593e−17	8.206952e−14
84	M640	trans_POS	26538	85	7352	23.54812	61	2.590440	6.678535	3.779805e−17	9.283201e−14
85	M409	trans_POS	26538	117	7352	32.41329	76	2.344717	4.877128	4.747028e−17	1.165870e−13
86	M727	trans_NEG	26538	110	8266	34.26257	77	2.247351	5.196232	5.854297e−17	1.437815e−13
87	M9	cis_NEG	26538	91	4004	13.72990	48	3.496020	6.345677	5.952784e−17	1.462004e−13
88	M104	cis_NEG	26538	244	4004	36.81423	89	2.417544	3.282012	1.320718e−16	3.243684e−13
89	M727	trans_POS	26538	110	7352	30.47404	72	2.362667	4.983244	1.698638e−16	4.171856e−13
90	M620	cis_NEG	26538	117	4004	17.65272	55	3.115666	5.047735	2.653620e−16	6.517290e−13
91	M513	trans_POS	26538	188	7352	52.08290	105	2.016017	3.334407	4.094872e−16	1.005701e−12
92	M358	trans_NEG	26538	65	8266	20.24606	52	2.568401	8.890835	6.013297e−16	1.476866e−12
93	M620	trans_NEG	26538	117	8266	36.44291	79	2.167774	4.629934	6.100156e−16	1.498198e−12
94	M111	cis_NEG	26538	787	4004	118.74098	204	1.718025	2.021284	8.788594e−16	2.158479e−12
95	M185	trans_NEG	26538	225	8266	70.08252	128	1.826418	2.946967	1.040714e−15	2.555993e−12
96	M358	trans_POS	26538	65	7352	18.00739	49	2.721106	8.037747	1.816856e−15	4.462197e−12
97	M211	trans_POS	26538	172	7352	47.65031	97	2.035663	3.406468	2.295273e−15	5.637190e−12
98	M640	trans_NEG	26538	85	8266	26.47562	62	2.341777	5.995552	2.715093e−15	6.668269e−12
99	M122	cis_NEG	26538	133	4004	20.06677	58	2.890350	4.401398	2.874605e−15	7.060030e−12
100	M112	cis_NEG	26538	603	4004	90.97943	165	1.813597	2.168158	3.285251e−15	8.068576e−12

	module	methylation_signature	Background	set1_size	set2_size	expected.overlap	actual.overlap	enrichment.foldchange	odds.ratio	FET_pvalue	corrected.FET.pvalue
1	M427	trans_NEG	26538	499	8266	155.427463	238	1.531261	2.045816	4.958807e−15	1.217883e−11
2	M410	trans_NEG	26538	172	8266	53.574195	103	1.922567	3.328793	6.300024e−15	1.547286e−11
3	M729	cis_POS	26538	339	1529	19.531653	60	3.071937	3.620006	6.505241e−15	1.597687e−11
4	M469	trans_POS	26538	316	7352	87.543598	151	1.724855	2.417237	1.925238e−14	4.728384e−11
5	M437	cis_NEG	26538	76	4004	11.466727	40	3.488354	6.305518	2.530019e−14	6.213727e−11
6	M700	trans_NEG	26538	180	8266	56.066019	105	1.872792	3.121502	3.998555e−14	9.820450e−11
7	M458	cis_NEG	26538	101	4004	15.238677	47	3.084257	4.944336	6.171925e−14	1.515825e−10
8	M438	cis_NEG	26538	112	4004	16.898334	50	2.958871	4.583188	7.539053e−14	1.851591e−10
9	M633	cis_POS	26538	387	1529	22.297196	63	2.825467	3.273863	7.573337e−14	1.860012e−10
10	M357	cis_NEG	26538	138	4004	20.821162	57	2.737599	4.002911	8.645940e−14	2.123443e−10
11	M384	cis_NEG	26538	442	4004	66.688070	127	1.904389	2.310490	1.172811e−13	2.880423e−10
12	M301	trans_POS	26538	113	7352	31.305147	69	2.204110	4.121383	1.222538e−13	3.002554e−10
13	M212	trans_NEG	26538	127	8266	39.557691	80	2.022363	3.789271	1.368165e−13	3.360213e−10
14	M119	cis_NEG	26538	181	4004	27.308916	67	2.453411	3.346669	3.274929e−13	8.043226e−10
15	M469	trans_NEG	26538	316	8266	98.427010	160	1.625570	2.292141	3.391989e−13	8.330724e−10
16	M212	trans_POS	26538	127	7352	35.183661	74	2.103249	3.670296	4.823872e−13	1.184743e−09
17	M249	trans_POS	26538	113	7352	31.305147	68	2.172167	3.970657	4.983805e−13	1.224022e−09
18	M659	trans_NEG	26538	478	8266	148.886427	223	1.497786	1.958996	6.148699e−13	1.510121e−09
19	M513	trans_NEG	26538	188	8266	58.557842	106	1.810176	2.881479	6.354082e−13	1.560562e−09
20	M170	trans_NEG	26538	72	8266	22.426407	52	2.318695	5.776693	9.077649e−13	2.229471e−09
21	M384	cis_POS	26538	442	1529	25.466049	66	2.591686	2.955287	1.192988e−12	2.929978e−09
22	M400	cis_NEG	26538	112	4004	16.898334	48	2.840517	4.259862	1.508528e−12	3.704944e−09
23	M170	trans_POS	26538	72	7352	19.946643	48	2.406420	5.246625	6.579693e−12	1.615973e−08
24	M210	trans_NEG	26538	59	8266	18.377195	44	2.394272	6.512823	8.428101e−12	2.069942e−08
25	M116	cis_POS	26538	454	1529	26.157435	65	2.484953	2.809856	1.180039e−11	2.898175e−08
26	M633	cis_NEG	26538	387	4004	58.389781	109	1.866765	2.240293	2.437298e−11	5.986005e−08
27	M634	trans_NEG	26538	100	8266	31.147788	63	2.022616	3.784782	5.175504e−11	1.271104e−07
28	M139	cis_POS	26538	86	1529	4.954933	24	4.843658	6.415606	5.843481e−11	1.435159e−07
29	M764	trans_NEG	26538	67	8266	20.869018	47	2.252142	5.217983	5.971586e−11	1.466621e−07
30	M10	cis_NEG	26538	142	4004	21.424674	53	2.473783	3.382758	6.496922e−11	1.595644e−07
31	M387	trans_POS	26538	50	7352	13.851835	36	2.598934	6.737230	9.515023e−11	2.336890e−07
32	M20	trans_NEG	26538	496	8266	154.493029	222	1.436958	1.812678	9.553522e−11	2.346345e−07
33	M600	trans_POS	26538	48	7352	13.297762	35	2.632022	7.053476	9.716857e−11	2.386460e−07
34	M121	cis_POS	26538	194	1529	11.177406	37	3.310249	3.925490	1.083890e−10	2.662033e−07
35	M372	trans_NEG	26538	28	8266	8.721381	25	2.866519	18.472725	2.387155e−10	5.862853e−07
36	M535	trans_NEG	26538	25	8266	7.786947	23	2.953661	25.486493	3.233535e−10	7.941563e−07
37	M764	trans_POS	26538	67	7352	18.561459	43	2.316628	4.696917	5.152408e−10	1.265432e−06
38	M355	trans_POS	26538	183	7352	50.697716	90	1.775228	2.544257	5.283420e−10	1.297608e−06
39	M103	trans_NEG	26538	312	8266	97.181099	149	1.533220	2.039378	5.319064e−10	1.306362e−06
40	M387	trans_NEG	26538	50	8266	15.573894	37	2.375771	6.314562	5.679299e−10	1.394836e−06
41	M435	cis_NEG	26538	76	4004	11.466727	34	2.965101	4.586184	6.584150e−10	1.617067e−06
42	M424	trans_NEG	26538	111	8266	34.574045	66	1.908946	3.259951	6.636855e−10	1.630012e−06
43	M114	trans_POS	26538	112	7352	31.028111	62	1.998188	3.254780	6.657706e−10	1.635133e−06
44	M622	trans_NEG	26538	56	8266	17.442761	40	2.293215	5.547599	6.715642e−10	1.649362e−06
45	M14	trans_NEG	26538	280	8266	87.213807	136	1.559386	2.105876	7.353974e−10	1.806136e−06
46	M131	trans_POS	26538	384	7352	106.382094	161	1.513413	1.903929	1.133771e−09	2.784541e−06
47	M656	trans_NEG	26538	55	8266	17.131283	39	2.276537	5.408230	1.552076e−09	3.811898e−06
48	M131	trans_NEG	26538	384	8266	119.607506	175	1.463119	1.869346	1.721446e−09	4.227872e−06
49	M651	trans_NEG	26538	78	8266	24.295275	50	2.058013	3.964807	2.072536e−09	5.090148e−06
50	M403	cis_NEG	26538	285	4004	43.000226	82	1.906967	2.299857	2.329493e−09	5.721235e−06
51	M622	trans_POS	26538	56	7352	15.514055	37	2.384934	5.102213	2.496058e−09	6.130318e−06
52	M642	trans_POS	26538	52	7352	14.405909	35	2.429559	5.393303	3.185640e−09	7.823932e−06
53	M378	cis_POS	26538	462	1529	26.618359	60	2.254083	2.499987	3.469086e−09	8.520076e−06
54	M155	cis_NEG	26538	99	4004	14.936921	39	2.610980	3.684034	3.474429e−09	8.533198e−06
55	M422	trans_POS	26538	30	7352	8.311101	24	2.887704	10.468213	3.807179e−09	9.350431e−06
56	M424	trans_POS	26538	111	7352	30.751074	60	1.951151	3.086805	4.288699e−09	1.053304e−05
57	M210	trans_POS	26538	59	7352	16.345165	38	2.324846	4.741227	4.493842e−09	1.103688e−05
58	M440	cis_NEG	26538	46	4004	6.940387	24	3.458020	6.169838	4.745018e−09	1.165376e−05
59	M388	trans_POS	26538	34	7352	9.419248	26	2.760305	8.506736	4.768346e−09	1.171106e−05
60	M6	cis_POS	26538	2273	1529	130.960020	196	1.496640	1.623380	4.916790e−09	1.207564e−05
61	M478	cis_POS	26538	68	1529	3.917854	19	4.849594	6.408572	5.618313e−09	1.379858e−05
62	M249	trans_NEG	26538	113	8266	35.197001	65	1.846748	3.009046	5.849913e−09	1.436739e−05
63	M399	cis_NEG	26538	644	4004	97.165423	152	1.564342	1.767793	5.900985e−09	1.449282e−05
64	M121	trans_NEG	26538	194	8266	60.426709	99	1.638348	2.319320	5.929029e−09	1.456169e−05
65	M20	trans_POS	26538	496	7352	137.410204	196	1.426386	1.724203	6.389962e−09	1.569375e−05
66	M667	cis_NEG	26538	60	4004	9.052679	28	3.093007	4.951723	6.537347e−09	1.605572e−05
67	M410	trans_POS	26538	172	7352	47.650313	83	1.741856	2.449987	7.551370e−09	1.854617e−05
68	M373	cis_NEG	26538	71	4004	10.712337	31	2.893860	4.387741	7.600178e−09	1.866604e−05
69	M371	trans_NEG	26538	59	8266	18.377195	40	2.176611	4.671124	7.679017e−09	1.885966e−05
70	M634	trans_POS	26538	100	7352	27.703670	55	1.985297	3.205882	8.217436e−09	2.018202e−05
71	M421	trans_NEG	26538	96	8266	29.901877	57	1.906235	3.246060	1.022311e−08	2.510796e−05
72	M114	trans_NEG	26538	112	8266	34.885523	64	1.834572	2.962395	1.091967e−08	2.681870e−05
73	M50	cis_NEG	26538	38	4004	5.733363	21	3.662771	6.982635	1.126172e−08	2.765878e−05
74	M727	cis_POS	26538	110	1529	6.337704	24	3.786860	4.620918	1.306372e−08	3.208449e−05
75	M168	trans_NEG	26538	90	8266	28.033009	54	1.926301	3.330960	1.485638e−08	3.648727e−05
76	M404	trans_POS	26538	61	7352	16.899239	38	2.248622	4.328443	1.744373e−08	4.284180e−05
77	M642	trans_NEG	26538	52	8266	16.196850	36	2.222654	4.990320	1.833796e−08	4.503804e−05
78	M417	trans_NEG	26538	86	8266	26.787098	52	1.941233	3.395344	1.890248e−08	4.642448e−05
79	M137	trans_NEG	26538	243	8266	75.689125	117	1.545797	2.067715	2.043079e−08	5.017801e−05
80	M104	cis_POS	26538	244	1529	14.058181	38	2.703052	3.068395	2.258567e−08	5.547041e−05
81	M301	cis_POS	26538	113	1529	6.510551	24	3.686324	4.464653	2.279233e−08	5.597797e−05
82	M372	trans_POS	26538	28	7352	7.757028	22	2.836138	9.593169	3.186704e−08	7.826545e−05
83	M14	trans_POS	26538	280	7352	77.570277	120	1.546984	1.973135	3.271768e−08	8.035461e−05
84	M437	trans_NEG	26538	76	8266	23.672319	47	1.985441	3.597104	3.326099e−08	8.168900e−05
85	M60	trans_POS	26538	147	7352	40.724395	72	1.767982	2.520038	3.348565e−08	8.224076e−05
86	M388	cis_NEG	26538	34	4004	5.129852	19	3.703811	7.156999	4.429541e−08	1.087895e−04
87	M684	cis_NEG	26538	47	4004	7.091265	23	3.243427	5.418306	4.775602e−08	1.172888e−04
88	M103	trans_POS	26538	312	7352	86.435451	130	1.504013	1.879421	6.674623e−08	1.639287e−04
89	M414	trans_POS	26538	43	7352	11.912578	29	2.434402	5.422724	6.720896e−08	1.650652e−04
90	M388	trans_NEG	26538	34	8266	10.590248	26	2.455089	7.202958	6.977476e−08	1.713668e−04
91	M168	trans_POS	26538	90	7352	24.933303	49	1.965243	3.132778	8.093979e−08	1.987881e−04
92	M371	trans_POS	26538	59	7352	16.345165	36	2.202486	4.099534	8.912022e−08	2.188793e−04
93	M404	trans_NEG	26538	61	8266	19.000151	39	2.052615	3.932049	1.346498e−07	3.307000e−04
94	M650	trans_NEG	26538	61	8266	19.000151	39	2.052615	3.932049	1.346498e−07	3.307000e−04
95	M106	cis_POS	26538	547	1529	31.515676	63	1.999005	2.177461	1.363581e−07	3.348955e−04
96	M553	trans_POS	26538	44	7352	12.189615	29	2.379074	5.060965	1.448212e−07	3.556809e−04
97	M475	trans_NEG	26538	154	8266	47.967594	79	1.646945	2.341153	1.463594e−07	3.594586e−04
98	M367	cis_POS	26538	134	1529	7.720476	25	3.238142	3.796546	1.723199e−07	4.232178e−04
99	M724	trans_NEG	26538	41	8266	12.770593	29	2.270842	5.356666	2.170959e−07	5.331876e−04
100	M414	trans_NEG	26538	43	8266	13.393549	30	2.239884	5.115418	2.176348e−07	5.345110e−04

	module	methylation_signature	Background	set1_size	set2_size	expected.overlap	actual.overlap	enrichment.foldchange	odds.ratio	FET_pvalue	corrected.FET.pvalue
1	M685	cis_NEG	26538	54	4004	8.147411	24	2.945721	4.523276	2.452621e−07	0.0006023637
2	M153	cis_NEG	26538	37	4004	5.582485	19	3.403502	5.963486	2.666241e−07	0.0006548289
3	M715	trans_POS	26538	30	7352	8.311101	22	2.647062	7.193788	2.687533e−07	0.0006600580
4	M640	cis_NEG	26538	85	4004	12.824629	32	2.495199	3.417062	2.951251e−07	0.0007248272
5	M116	cis_NEG	26538	454	4004	68.498606	109	1.591273	1.799805	3.321740e−07	0.0008158193
6	M456	cis_NEG	26538	106	4004	15.993067	37	2.313503	3.036488	3.412832e−07	0.0008381915
7	M438	cis_POS	26538	112	1529	6.452935	22	3.409301	4.041867	3.686885e−07	0.0009054990
8	M386	cis_POS	26538	130	1529	7.490014	24	3.204266	3.745903	3.697673e−07	0.0009081486
9	M422	trans_NEG	26538	30	8266	9.344336	23	2.461384	7.279863	3.766537e−07	0.0009250616
10	M715	trans_NEG	26538	30	8266	9.344336	23	2.461384	7.279863	3.766537e−07	0.0009250616
11	M156	cis_NEG	26538	17	4004	2.564926	12	4.678498	13.541932	4.025886e−07	0.0009887575
12	M226	trans_NEG	26538	223	8266	69.459567	105	1.511671	1.979429	4.218778e−07	0.0010361319
13	M414	cis_POS	26538	43	1529	2.477466	13	5.247296	7.138140	5.268278e−07	0.0012938891
14	M624	trans_POS	26538	14	7352	3.878514	13	3.351799	33.979694	5.845417e−07	0.0014356343
15	M442	cis_NEG	26538	60	4004	9.052679	25	2.761613	4.038685	6.241999e−07	0.0015330351
16	M139	trans_NEG	26538	86	8266	26.787098	49	1.829239	2.938781	6.252048e−07	0.0015355031
17	M117	trans_NEG	26538	637	8266	198.411410	256	1.290248	1.500765	6.405917e−07	0.0015732933
18	M535	trans_POS	26538	25	7352	6.925918	19	2.743319	8.281533	7.195871e−07	0.0017673060
19	M36	trans_POS	26538	225	7352	62.333258	96	1.540109	1.954550	9.362255e−07	0.0022993698
20	M427	trans_POS	26538	499	7352	138.241314	187	1.352707	1.578798	1.083446e−06	0.0026609438
21	M640	cis_POS	26538	85	1529	4.897317	18	3.675482	4.434253	1.328487e−06	0.0032627638
22	M740	trans_NEG	26538	24	8266	7.475469	19	2.541647	8.416184	1.733314e−06	0.0042570180
23	M600	trans_NEG	26538	48	8266	14.950938	31	2.073448	4.041780	1.883449e−06	0.0046257505
24	M724	trans_POS	26538	41	7352	11.358505	26	2.289034	4.535499	1.938966e−06	0.0047621012
25	M600	cis_POS	26538	48	1529	2.765544	13	4.700703	6.117876	2.121060e−06	0.0052093226
26	M666	cis_NEG	26538	52	4004	7.845655	22	2.804100	4.144210	2.140903e−06	0.0052580589
27	M565	trans_NEG	26538	44	8266	13.705027	29	2.116012	4.284865	2.183195e−06	0.0053619281
28	M423	cis_POS	26538	153	1529	8.815171	25	2.836020	3.230842	2.259579e−06	0.0055495252
29	M626	trans_NEG	26538	14	8266	4.360690	13	2.981179	28.777289	2.569089e−06	0.0063096820
30	M728	trans_NEG	26538	14	8266	4.360690	13	2.981179	28.777289	2.569089e−06	0.0063096820
31	M60	cis_POS	26538	147	1529	8.469478	24	2.833705	3.226194	3.629444e−06	0.0089139143
32	M41	cis_NEG	26538	120	4004	18.105358	38	2.098826	2.623262	3.682203e−06	0.0090434912
33	M634	cis_POS	26538	100	1529	5.761549	19	3.297724	3.872486	3.802924e−06	0.0093399812
34	M243	trans_NEG	26538	76	8266	23.672319	43	1.816468	2.890055	3.843427e−06	0.0094394556
35	M764	cis_POS	26538	67	1529	3.860238	15	3.885771	4.754667	4.824757e−06	0.0118496029
36	M361	trans_NEG	26538	27	8266	8.409903	20	2.378149	6.327960	5.574393e−06	0.0136907094
37	M642	cis_POS	26538	52	1529	2.996006	13	4.339111	5.490376	5.626503e−06	0.0138186924
38	M364	trans_NEG	26538	29	8266	9.032859	21	2.324845	5.814149	5.865257e−06	0.0144050714
39	M30	cis_NEG	26538	59	4004	8.901801	23	2.583747	3.610352	6.747877e−06	0.0165727856
40	M478	trans_NEG	26538	68	8266	21.180496	39	1.841317	2.981958	6.867860e−06	0.0168674644
41	M656	trans_POS	26538	55	7352	15.237019	31	2.034519	3.380508	7.346371e−06	0.0180426880
42	M77	trans_POS	26538	60	7352	16.622202	33	1.985297	3.199243	7.697101e−06	0.0189040803
43	M421	trans_POS	26538	96	7352	26.595523	47	1.767215	2.512693	7.719226e−06	0.0189584191
44	M361	cis_NEG	26538	27	4004	4.073706	14	3.436674	6.077914	8.802950e−06	0.0216200454
45	M673	cis_NEG	26538	10	4004	1.508780	8	5.302298	22.545580	9.013974e−06	0.0221383205
46	M759	trans_NEG	26538	20	8266	6.229558	16	2.568401	8.856407	9.453529e−06	0.0232178669
47	M489	cis_NEG	26538	41	4004	6.185997	18	2.909798	4.419695	9.574967e−06	0.0235161199
48	M668	cis_NEG	26538	34	4004	5.129852	16	3.118999	5.018287	1.008689e−05	0.0247734098
49	M539	trans_POS	26538	14	7352	3.878514	12	3.093969	15.684163	1.025145e−05	0.0251775649
50	M28	trans_NEG	26538	244	8266	76.000603	108	1.421041	1.765281	1.047084e−05	0.0257163732
51	M101	trans_POS	26538	236	7352	65.380662	96	1.468324	1.799801	1.081845e−05	0.0265701086
52	M740	trans_POS	26538	24	7352	6.648881	17	2.556821	6.348620	1.379738e−05	0.0338863660
53	M388	cis_POS	26538	34	1529	1.958927	10	5.104836	6.852053	1.434762e−05	0.0352377498
54	M703	trans_POS	26538	20	7352	5.540734	15	2.707223	7.841709	1.493564e−05	0.0366819233
55	M716	trans_POS	26538	20	7352	5.540734	15	2.707223	7.841709	1.493564e−05	0.0366819233
56	M759	trans_POS	26538	20	7352	5.540734	15	2.707223	7.841709	1.493564e−05	0.0366819233
57	M620	trans_POS	26538	117	7352	32.413294	54	1.665983	2.245932	1.525816e−05	0.0374740370
58	M127	cis_NEG	26538	185	4004	27.912427	50	1.791317	2.098061	1.910263e−05	0.0469160512
59	M150	cis_NEG	26538	16	4004	2.414048	10	4.142420	9.399957	2.011218e−05	0.0493955123
